# Circulating miR-330-3p in Late Pregnancy is Associated with Pregnancy Outcomes Among Lean Women with GDM

**DOI:** 10.1038/s41598-020-57838-6

**Published:** 2020-01-22

**Authors:** Shona Pfeiffer, Begoña Sánchez-Lechuga, Paul Donovan, Luise Halang, Jochen H. M. Prehn, Antonio Campos-Caro, Maria M. Byrne, Cristina López-Tinoco

**Affiliations:** 1 Centre for Systems Medicine, Department of Physiology and Medical Physics, Royal College of Surgeons in Ireland, 123 St. Stephen’s Green, Dublin, 2 Ireland; 2 Servicio de Endocrinología y Nutrición, Hospital Universitario Puerta del Mar, Cádiz, Spain; 3 Department of Endocrinology, Mater Misericordiae University Hospital, Eccles Street, Dublin, 7 Ireland

**Keywords:** RNA sequencing, Gestational diabetes

## Abstract

Gestational Diabetes Mellitus (GDM) is characterised by insulin resistance accompanied by reduced beta-cell compensation to increased insulin demand, typically observed in the second and third trimester and associated with adverse pregnancy outcomes. There is a need for a biomarker that can accurately monitor status and predict outcome in GDM, reducing foetal-maternal morbidity and mortality risks. To this end, circulating microRNAs (miRNAs) present themselves as promising candidates, stably expressed in serum and known to play crucial roles in regulation of glucose metabolism. We analysed circulating miRNA profiles in a cohort of GDM patients (*n* = 31) and nondiabetic controls (*n* = 29) during the third trimester for miRNA associated with insulin-secretory defects and glucose homeostasis. We identified miR-330-3p as being significantly upregulated in lean women with GDM compared to nondiabetic controls. Furthermore, increased levels of miR-330-3p were associated with better response to treatment (diet vs. insulin), with lower levels associated with exogenous insulin requirement. We observed miR-330-3p to be significantly related to the percentage of caesarean deliveries, with miR-330-3p expression significantly higher in spontaneously delivered GDM patients. We report this strong novel association of circulating miR-330-3p with risk of primary caesarean delivery as a pregnancy outcome linked with poor maternal glycaemic control, strengthening the growing body of evidence for roles of diabetes-associated miRNAs in glucose homeostasis and adaptation to the complex changes related to pregnancy.

## Introduction

Gestational Diabetes Mellitus (GDM) is diagnosed as the development of hyperglycaemia during the second or third trimester in pregnant women not previously diagnosed with diabetes^1^. The global epidemic of obesity and diabetes has resulted in an increase in the prevalence of diabetes mellitus in women of childbearing age and estimates currently place 5% to 20% of pregnancies as affected by GDM worldwide, due to differences in population demographics, diagnostic criteria, screening methods and maternal lifestyle^2^. The significant risk-associations between hyperglycaemia not considered to be within the diagnostic range for overt diabetes and adverse perinatal and maternal outcomes require more accurate measures for monitoring status and predicting outcome in GDM^3^.

The pathophysiology of GDM manifests as a result of the progressive insulin resistance (IR) and subsequent increased insulin secretion requirement that occurs during normal pregnancy^4^. As a result, most women with GDM develop pancreatic beta-cell dysfunction secondary to IR, resulting in impaired glucose tolerance in a setting of IR similar to that of T2DM^5^. However, the possible causes of beta-cell dysfunction and IR are not well described, including the contributions of autoimmune and monogenic factors. Meta-analysis of gene expression profiles has indicated that the transcriptome profile in GDM is more similar to that of autoimmune type 1 diabetes mellitus (T1DM) than to T2DM^6^. Cross-sectional studies on small cohorts of women with GDM have shown that the prevalence of monogenic GCK mutations associated with impaired glucose regulation is between 0% and 5%^7–9^ and *HNF1A* or *HNF4A* loss-of-function mutations associated with beta-cell dysfunction contribute to <1% of cases^10^. Taken together, the ability to diagnose GDM distinct from undiagnosed or incidental development of other forms of diabetes prior to, or during, pregnancy represents a significant challenge^11^.

Systemic regulation of glucose homeostasis requires concerted actions of tightly regulated signalling pathways. MicroRNAs (miRNAs) are well known to play key roles in the modulation of such pathways through base pairing of target mRNAs, leading to repression of protein production or mRNA degradation^12^. Evidence has implicated miRNAs as playing crucial roles in glucose metabolism^13^, pancreatic development^14,15^, insulin secretion^15–18^ and insulin deficiency^19^. Furthermore, the secretion of miRNAs and easy detection in biofluids has resulted in examination of unique miRNA differential expression profiling of urine, serum and whole blood as diagnostic biomarkers for disease progression^20–22^. Previously we have demonstrated significant increases in extracellular novel diabetes-linked miRNAs miR-224 and miR-103-3p in biofluids of patients with HNF1A-MODY primary beta-cell dysfunction, capable of differentiating these patients from T2DM^23,24^. Such ease of access and a minimally invasive approach places circulating miRNA profiling as a promising novel clinical approach in the progression and management of GDM and associated outcomes.

MiR-103-3p dysregulation has been widely reported by us and others in HNF1A-MODY, T1DM and T2DM^23–25^. Elevated levels detected in a leptin-deficient *ob/ob* mouse model of obesity associated with T2DM were shown to negatively regulate insulin sensitivity while inhibition of miR-103-3p resulted in increased glucose tolerance and a reduction in hyperglycaemia^19^. Furthermore, target analysis and miRNA-modulation has highlighted roles for miR-103-3p in peripheral insulin sensitivity^19^. Elevated expression of miR-224 in HNF1A-MODY and patients with T1DM, first reported by us as a novel miRNA in the field of diabetes, presents a potential role for miR-224 in beta-cell failure, distinct from relative insulin deficiency. Akehurst *et al*. previously reported miR-206 as a novel prognostic pregnancy-associated circulating miRNA associated with the development of preeclampsia as a complication of pregnancy^26^. MiR-206 has also been reported to play a central role in glucose homeostasis regulation through modulation of glucokinase activity, negatively regulating glucose-stimulated insulin secretion^27^. Furthermore, the therapeutic potential of miR-206 in settings of hyperglycaemia has been reported, elucidating mechanistic roles in the inhibition of glucose production and lipogenesis and promotion of insulin signalling^28^. Of particular interest, dysregulation of miR-330-3p has been observed in a small cohort of pregnant women diagnosed with GDM^29^. Despite the emergence of compelling evidence for miRNAs such as these having relevant, functional roles in the pathogenesis of GDM, circulating miRNAs remain poorly considered in this setting.

The mounting evidence for central roles of miRNAs in important diabetes-associated pathways with implications for glucose homeostasis further supports profiling of miRNA dysregulation in GDM as a predictor of pregnancy outcome linked to disease severity and progression. Altered circulating miRNAs associated with the development and pathogenesis of insulin secretory defects and impaired glucose processing in such a clinically heterogeneous and often asymptomatic cohort would be invaluable in monitoring glycaemic management, treatment response and progression, allowing for tighter control impacting on associated complications. Therefore, we aimed to establish a signature of these potentially significant diabetes-associated miRNAs in lean women with GDM without risk factors for IR for improved management and outcome prediction in GDM.

## Results

### GDM and nondiabetic cohort profiling

Serum from GDM patients (*n* = 31) and age- and body mass index (BMI)-matched nondiabetic (*n* = 29) cohorts was collected for profiling. The clinical characteristics of both cohorts are presented in Table 1. Weight gain was higher in nondiabetic controls (11.8 ± 4.1) compared to the GDM cohort (9.3 ± 4.1) with no significant differences observed in other variables. Within the GDM cohort, twelve (38%) women were treated with diet and nineteen (61%) received insulin. Insulin-treated GDM patients were observed to have significantly increased mean fasting glucose levels (102 ± 17.7 vs 79.9 ± 6.7, *p* < 0.001) and HbA1c levels (5.13 ± 0.48 vs 4.96 ± 0.30, *p* = 0.01) compared with the diet-treated group. There were no significant differences in the rest of the analytical and clinical variables and the obstetrical or perinatal complications between groups treated with insulin versus diet.

**Table 1:** Demographic, clinical, laboratory variables and obstetric and perinatal outcomes in women with GDM and controls. Data are presented as means ± SD or n (%). BMI, body mass index; BP, blood pressure; DM, diabetes mellitus; LGA, large for gestational age; SGA, small for gestational age.

Characteristic	GDM (*n* = 31)	Controls (*n* = 29)	*p* value
Age (y)	31.9 ± 1.8	31.0 ± 3.6	NS
Pre-gestational BMI (kg/m^2^)	22.5 ± 1.8	22.3 ± 1.8	NS
DM family history n (%)	14 (51.3)	14 (48.3)	NS
GDM personal history n (%)	9 (29)	3 (10)	NS
Systolic BP (mmHg)	106.9 ± 12.3	106.8 ± 18	NS
Diastolic BP (mmHg)	64 ± 9	61 ± 6	NS
Basal glucose (mg/dl)	92.5 ± 13	83 ± 4	0.01
Total- cholesterol (mmol/l)	244.4 ± 46.1	240.2 ± 30.1	NS
LDL- cholesterol;(mmol/l)	142.4 ± 44.6	139.3 ± 10.7	NS
HDL- cholesterol (mmol/l)	71.1 ± 16.4	77.6 ± 26.3	NS
TG (mmol/l)	193.8 ± 63.1	217.2 ± 68.0	NS
Weight gain (kg)	9.3 ± 4.1	11.8 ± 4.1	0.03
Gestational age at delivery (wk)	39.1 ± 1.3	39.2 ± 1.2	NS
Caesarean section n (%)	18 (39.0)	5 (18)	0.06
Birth weight (g)	3202 ± 589	3307 ± 545	NS
Macrosomia n (%)	3 (14.1)	3 (9)	NS
HbA1c	5.4 ± 0.36	4.8 ± 0.33	0.02
LGA n (%)	3 (10)	3 (9)	NS
SGA n (%)	2 (7)	5 (16)	NS

### miRNA profiling in the serum of GDM patients: miR-330-3p levels are elevated in the serum of patients with GDM

**Figure 1:**
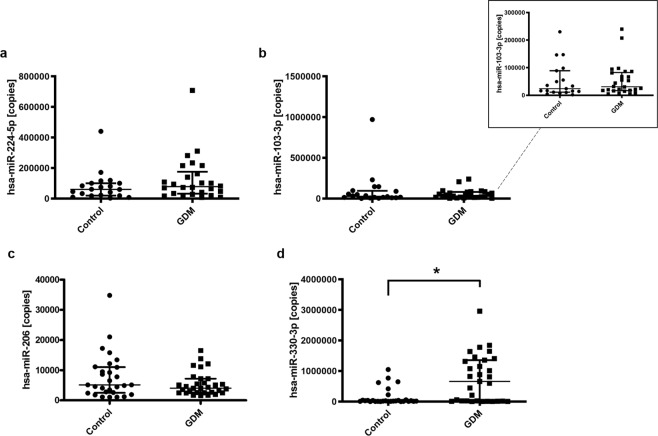
Detection of diabetes-associated circulating serum miRNAs associated with glucose tolerance and homeostasis in the serum of patients with GDM and non-diabetic controls. **(a)**, miR-224-5p levels were detectable in serum of control (median [IQR], 60298 [19595–100208] copies, *n* = 20) and gestational diabetes mellitus (GDM) patients (median [IQR], 78205 [32239–175925] copies, *n* = 26). Significance determined by Mann–Whitney *U* test, *p* = 0.215. (**b**), miR-103-3p levels detected in serum of control (median [IQR], 28588 [11769–95796] copies, *n* = 20) and gestational diabetes mellitus (GDM) patients (median [IQR], 30804 [16991–82426] copies, *n* = 26). Significance determined by Mann–Whitney *U* test, *p* = 0.763. (**c**), miR-206 levels were detected in serum of control (median [IQR], 5109 [2480–11028] copies, *n* = 29) and gestational diabetes mellitus (GDM) patients (median [IQR], 4048 [2541–7195] copies, *n* = 31). Significance determined by Mann–Whitney *U* test, *p* = 0.420. (**d**), miR-330-3p levels detected in the serum of gestational diabetes mellitus (GDM) patients (median [IQR], 656943 [16149–1355000] copies, *n* = 31) were found to be significantly higher than those of nondiabetic controls (median [IQR], 20098 [8734–50016] copies, *n* = 29). Significance determined by Mann–Whitney *U* test, *p* = 0.003.

We performed miRNA absolute quantitative PCR analysis of the expression of four miRNAs of interest in the serum from women with GDM (*n* =31) compared with healthy controls (*n* = 29). Here we found miR-224 to be detectable in the serum of both GDM (median [IQR], 78205 [32239–175925] copies, *n* = 26) and control (median [IQR], 60298 [19595–100208] copies, *n* = 20) cohorts, although no significant difference was determined between groups (Fig. 1(a)). Levels of miR-103-3p were also detectable in both GDM (median [IQR], 30804 [16991–82426] copies, *n* = 26) and control (median [IQR], 28588 [11769–95796] copies, *n* = 20) cohorts, but similar to miR-224, no significant difference was observed in expression levels between groups (Fig. 1(b)). Next we examined expression of miR-206, shown to play a role in glucose tolerance and homeostasis^27^. Similar to miR-103-3p and miR-224, while levels of miR-206 were detectable in both GDM (median [IQR], 4048 [2541–7195] copies, *n* = 31) and control (median [IQR], 5109 [2480–11028] copies, *n* = 29) groups, they were not found to differ significantly (Fig. 1(c)). Finally, we examined expression levels of miR-330-3p as a potential GDM-associated miRNA. Interestingly, we found significantly increased serum expression of miR-330-3p in our GDM cohort, demonstrating a 5.2-fold difference in mean miRNA expression (*p* = 0.003, Mann–Whitney *U* test) in GDM patients (median [IQR], 656943 [16149–1355000] copies, *n* = 31) compared to control (median [IQR], 20098 [8734–50016] copies, *n* = 29) (Fig. 1(d)).

Multivariate analysis was performed based on GDM diagnosis as the dependent variable and independent variables considered to be of clinical significance or statistically significant based on univariate analysis. The results of this final model are presented in Table 2, and indicate that increased circulating serum miR-330-3p (OR: 1.99) is associated with onset or development GDM and suggests miR-330-3p is associated with phenotypic onset.

**Table 2:** Multivariate logistic regression for GDM (final model). OR, odd ratio; 95% CI, 95% confidence interval.

Variables	OR	Z-score	*p*-value	95% CI
Age	0.9	−0.6	0.8	0.5-1.6
Pre-gestational BMI	1.2	0.2	0.6	0.5-2.7
miR-330-3p	1.99	0.6	0.03	1.06-3.7
miR-224-5p	4.66	1.5	0.9	0.00-5.7E + 15
miR-206	1.09	0.09	0.9	0.02-57
miR-103-3p	1.051	0.05	0.9	0.2-5.3
TG	1.01	0.1	0.2	0.9-1.03
Weight gain	0.8	−0.1	0.4	0.6-1.2
Constant	0.00	−15.9	0.6	

### Levels of miR-330-3p are associated with type of treatment and delivery in women with GDM

**Figure 2:**
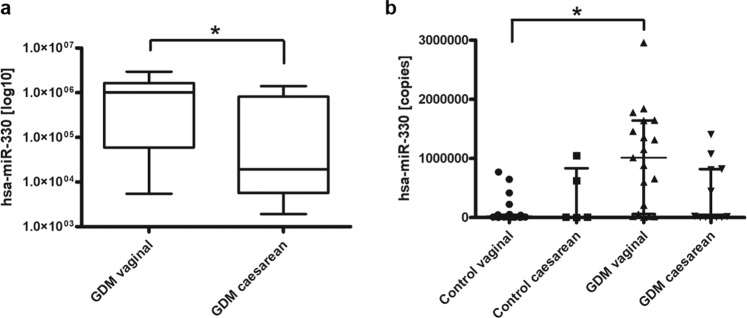
Circulating miR-330-3p levels are associated with risk of primary caesarean section in women with GDM. (**a**), analysis of miR-330-3p expression in GDM cohort by incidence of vaginal (spontaneous) delivery (median [IQR], 1012000 [59518–1640000] copies, *n* = 19) and those delivered by caesarean section (median [IQR], 19278 [5677–817926] copies, *n* = 12). Significance determined by Mann-Whitney *U* Test, *p* = 0.012. (**b**), analysis of miR-330-3p expression (copies) by incidence of vaginal (spontaneous) delivery: control, (median [IQR], 23160 [8580–43841] copies, *n* = 24), GDM (median [IQR], 1012000 [59518–1640000] copies, *n* = 19) and caesarean delivery: control (median [IQR], 9957 [5216–834332] copies, *n* = 5), GDM (median [IQR], 19278 [5677–817926] copies, *n* = 12). Significance determined by Kruskal-Wallis Test adjusted by Bonferroni correction for multiple tests, *p* = 0.001.

**Figure 3:**
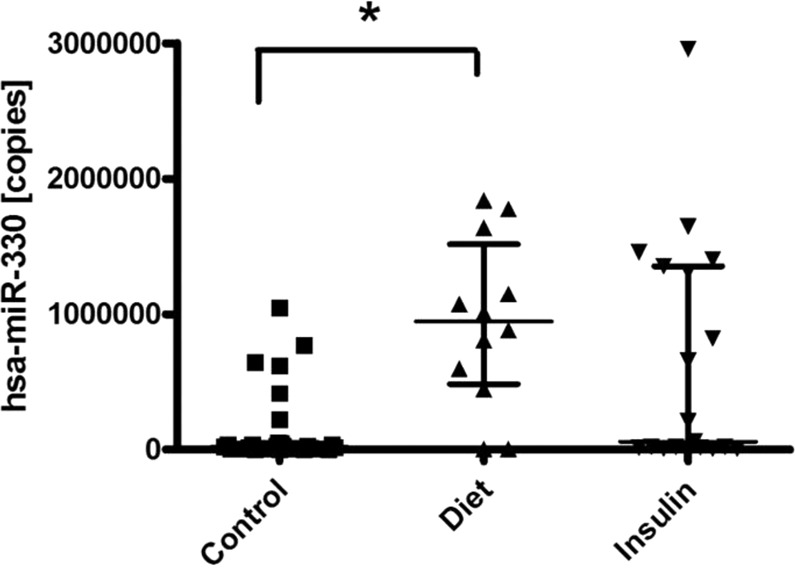
Levels of miR-330-3p are associated with type of treatment. Analysis of miR-330-3p expression (copies) in GDM patients treated by diet (median [IQR], 946252 [484236–1517000] copies, *n* = 12) or insulin (median [IQR], 59518 [12967–1355000] copies, *n* = 19) compared to non-diabetic controls (median [IQR], 20098 [8734–50016] copies, *n* = 29). Significance determined by Kruskal-Wallis Test, *p* = 0.004.

In assessing the relationships between the different miRNA and obstetric and perinatal variables in GDM patients, we observed that the levels of miR-330-3p were significantly related to the percentage of caesarean deliveries (10.6 ± 2.7; *p* = 0.012) (Table 3, Fig. 2(a)). Spontaneously delivered GDM patients displayed significantly higher expression of miR330-3p (median [IQR], 1012000 [59518–1640000] copies, *n* = 19) compared to GDM patients delivered by primary caesarean section, observed to have significantly lower copy number (median [IQR], 19278 [5677–817926] copies, *n* = 12). Furthermore, we observed significantly increased circulating miR-330-3p levels in spontaneously delivered GDM patients (median [IQR], 1012000 [59518–1640000] copies, *n* = 19) compared to spontaneously delivered control (median [IQR], 23160 [8580–43841] copies, *n* = 24; *p* = 0.001, Kruskal-Wallis test) (Fig. 2(b)). Significantly increased levels of circulating miR-330-3p were also observed in GDM patients treated by diet (median [IQR], 946252 [484236–1517000] copies, *n* = 12) but not those treated by insulin (median [IQR], 59518 [12967–1355000] copies, *n* = 19) when compared to nondiabetic controls (median [IQR], 20098 [8734–50016] copies, *n* = 29; *p* = 0.004, Kruskal-Wallis test)(Fig. 3).

**Table 3:** Relationships between levels of miRNA and the percentage of macrosomia and Caesarean delivery. Data expressed as mean ± SD; *p <0.05.

Variables	Caesarean (*n* = 12)	Vaginal (*n* = 19)	Macrosomia Yes (*n* = 3)	Macrosomia No (*n* = 28)
miRNA-330-3p	10.6 ± 2.7*	12.8 ± 1.9	10.5 ± 2.5	12.1 ± 2.4
miRNA-224-5p	2.3 ± 0.1	2.4 ± 0.1	2.4 ± 0.7	2.1 ± 0.1
miRNA-206	10.8 ± 0.9	11.4 ± 1.2	11.7 ± 0.2	11.1 ± 1.1
miRNA-103-3p	9.9 ± 0.9	10.7 ± 1.1	10.8 ± 0.8	10.4 ± 1.1

In order to fully demonstrate the significant association of miR-330-3p and risk of primary caesarean delivery in GDM patients, multivariate analysis was performed based on primary caesarean delivery as the dependent variable and the potential of confounding by factors that might be associated with both miR-330-3p and primary caesarean delivery as independent variables. The results of this final model are presented in Table 4, and indicate a protective effect for circulating serum miR-330-3p (OR: 0.4) for the presence of primary caesarean delivery. Patients with previous caesarean section were excluded from primary caesarean delivery analysis.

**Table 4:** Multivariate logistic regression for Cesarean Section (final model). OR, odd ratio; 95% CI, 95% confidence interval.

Variables	OR	Z-score	*p*-value	95% CI
**Age**	0.7	−0.3	0.3	0.3-1.4
**Pre-gestational BMI**	1.4	0.3	0.2	0.7-2.6
**Pregnancies**	0.7	−0.2	0.6	0.1-2.9
**miR-330-3p**	0.4	−0.8	0.02	0.2-0.8
**Glucose**	0.9	−0.2	0.6	0.8-1.06
**Gestational delivery weeks**	0.8	−0.1	0.4	0.6-1.2
**Constant**		−15.9	0.1	

### Identification of miR-330-3p targets and pathway enrichment

**Figure 4:**
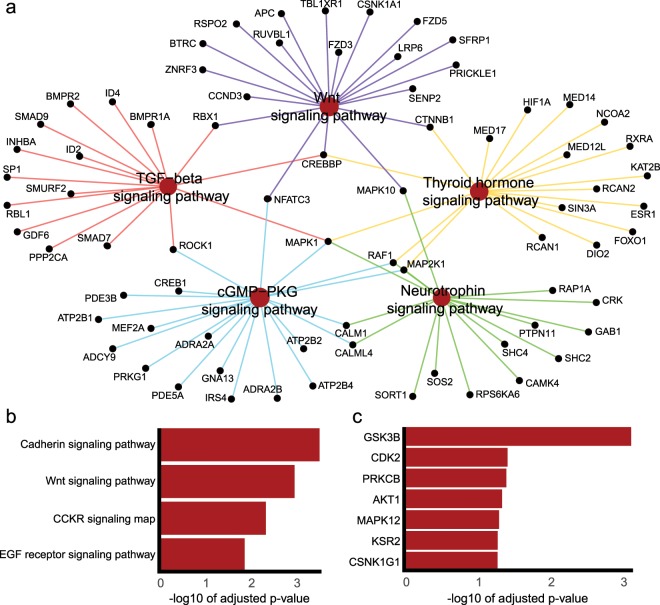
MiR-330-3p targets multiple overlapping KEGG pathways. (**a**), multiple pathways were identified using KEGG pathway analysis. Edges are coloured based on their pathway of origin. (**b,c**), bar plots showing the −log10 of the adjusted *p*-values of significantly enriched (*p*-adj < 0.05) Panther pathways and kinase enrichment analysis terms, respectively, for predicted targets of miR-330-3p.

1,031 gene targets of miR-330-3p were predicted using TargetScan. The predicted miR-330-3p target genes were analysed using the gene ontology enrichment analysis tool. In total, 24 databases were analysed, the full results of which are available in supplementary file EnrichR_Analysis.xlsx. A Kyoto Encyclopedia of Genes and Genomes (KEGG)^30^ pathway analysis revealed 12 pathways overrepresented in the predicted gene targets of miR-330-3p, including the insulin signalling pathway (*p-*adj <0.05). 16 proteins associated with this pathway are present in the TargetScan predictions. The analysis of the databases ‘InterPro Domains 2019’, ‘Pfam domains’, and ‘Panther 2016’ displayed enrichment of Cadherin domains/pathways (Fig. 4). Panther 2016 also revealed a significant number of miR-330-3p target genes are associated with cholecystokinin receptor (CCKR) signalling. Furthermore, several databases analysed by EnrichR (KEGG, Panther, Reactome) revealed a relationship between the Wnt signalling pathway and the predicted miR-330-3p targets. A kinase enrichment analysis showed that Glycogen synthase kinase 3 (GSK3), a protein involved in Wnt and insulin signalling, shares a significant number of phosphorylation targets with the TargetScan gene predictions. In addition, GSKIP (GSK3B Interacting Protein), a negative regulator of GSK3-beta, is a predicted target of miR-330-3p.

## Discussion

Given the challenges of GDM management and outcome prediction in the setting of increased global incidence, undiagnosed and untreated forms of diabetes, there is a need for a biomarker that can accurately monitor GDM pathogenesis and reduce foetal-maternal morbidity and mortality risks. To this end, circulating microRNAs present themselves as promising candidates, stably expressed in serum and known to play crucial roles in pancreatic development^14,15^, insulin secretion^15–17^ and insulin deficiency^19,31^. Furthermore, evidence from maternal blood profiling in the first weeks of pregnancy demonstrating increasing concentrations of placental and trophoblast-derived extracellular vesicles (EVs) implicates circulating microRNAs in maternal environmental homeostasis in response to stimuli such as high glucose concentration^32–34^.

Previously we demonstrated upregulation of miR-103-3p and miR-224 in individuals with an insulin-secretory defect as seen in T1DM and HNF1A-MODY, which were not found here to be significant in our GDM cohort^23,24^. Pregnancy itself is considered to be a diabetogenic endocrine–metabolic adaptation process, with altered glucose metabolism resulting in the progressive development of IR and consequent increased postprandial glucose, circulating lipids and beta-cell demand^35,36^. Beta-cell adaptation to pregnancy, increasing capacity through expansion of cell mass and secretion, is associated with alterations to the endogenous miRNA profile in response to the physiological changes of pregnancy^37^. Several islet microRNAs have been reported to contribute to this adaptive response, with effects on secretory activity and survival which are reversed after pregnancy^38^. Therefore, where we would observe differences in miR-103-3p and miR-224 between nondiabetic controls and diabetics with an insulin-secretory defect, it is likely that these physiological adaptive responses to pregnancy are why we do not observe differences in these miRNAs in our GDM cohort compared to nondiabetic pregnant controls.

Previous work by Sebastiani *et al*. identified increased levels of miR-330-3p in women diagnosed with GDM (*n* = 21) compared to nondiabetic subjects (*n* = 10) at 28th–33th week of gestation^29^. Here, we confirmed upregulation of circulating miR-330-3p in a larger GDM cohort (*n* = 31) compared to a matched nondiabetic cohort (*n* = 29). Interestingly, further analysis found significantly elevated miR-330-3p levels in GDM patients treated by diet compared to those treated by insulin, a finding found to be independent of incidence of T2DM in a first-degree relative. The increase in circulating miR-330-3p observed in the diet-treated GDM patient group is further indicative that miR-330-3p dysregulation signals an increased beta-cell adaptive response to developing IR as seen in GDM. This is supported by Sebastiani *et al*., who reported an inverse correlation between insulinaemia and circulating miR-330-3p levels in GDM patients, but not in nondiabetic controls^29^. Insulin levels within the range of normal nondiabetic controls were observed to correlate with increased circulating miR-330-3p compared to hyperinsulinemia, observed to correlate with low levels of miR-330-3p in women with GDM. Current treatment strategies in the initial management of women with GDM centre on medical nutrition therapy, however this approach remains a challenging treatment with regard to adherence in GDM^39,40^. Furthermore, emphasis on restriction of dietary carbohydrate and a corresponding increased reliance on fat can result in exacerbation of maternal IR^41–44^. Diet-treated GDM patients with increased levels of circulating miR-330-3p may achieve better glycaemic control, with levels of miR-330-3p decreasing with increasing disease severity and exogenous insulin requirement. GDM patients receiving nutrition therapy with significantly or progressively lower levels of miR-330-3p may be candidates for progression to insulin therapy or future T2DM development due to progressive decline in compensatory insulin secretion in a setting of uncontrolled hyperglycaemia and increased IR, resulting in a persistent and progressive metabolic load^45^.

Importantly, further linking miR-330-3p with diabetic phenotype severity, on measurement of pregnancy outcomes we observed a significant correlation between miR-330-3p levels and percentage of primary caesarean deliveries, with increased miR-330-3p expression significantly higher in spontaneous delivery in GDM patients but not those delivered by caesarean section when compared to spontaneously-delivered nondiabetic controls. Significant associations between poor maternal glycaemic control during pregnancy and adverse outcomes are well established, and glucose levels are a significant predictor of primary caesarean delivery^3^. As a known GDM complication related to hyperglycaemia during pregnancy, this novel finding that lower miR-330-3p is associated with higher risk of primary caesarean delivery suggests a better outcome associated with increased miR-330-3p, and further strengthens the role for miR-330-associated with disease severity and control, impacting on pregnancy outcomes.

The heterogeneous aetiology of GDM and associated risk factors, in concert with genetic predisposition and the degree to which individual patients can achieve glycaemic control and insulin secretory requirements, place miRNAs in an attractive position as clinically relevant targets, regulating multiple genes in diverse biological pathways^46,47^. Roles in beta-cell function and preservation in GDM, including proliferation, hyperplasia and hypertrophy, are balanced against progressive dysfunction and beta-cell exhaustion in the challenge to compensate for increased insulin demand, resulting in beta-cell demise and progression of the diabetic phenotype^48–50^. As such, these findings highlight miR-330-3p as a potential biomarker and regulatory target in the pathogenesis of GDM. To examine potential mechanisms though which miR-330-3p might mediate such processes in the control of hyperglycaemia, Sebastiani *et al*. highlighted two experimentally validated targets of miR-330-3p: E2F transcription factor 1 (E2F1), facilitating glucose homeostasis through upregulation of beta-cell proliferation, insulin secretion and glucose tolerance^51^, and cell division cycle 42 (CDC42), a potent modulator of insulin secretion and importantly, second-phase insulin release^52,53^.

We carried out pathway enrichment analysis to further elucidate potential pathways regulated by miR-330-3p, revealing significant overrepresentation of cadherin signalling. Calcium-dependent aggregation of islet cells is known to be mediated by E-cadherin (uvomorulin)^54^. A study from 2015 found that specific cadherins adhere to beta-cells, and that this process promotes insulin secretion^55^. Our analysis also revealed a significant number of miR-330-3p target genes associated with CCKR signalling. Gastric hormones cholecystokinin (CCK) and gastrin are key regulators of the digestion of fats and proteins, and activation of CCKR signalling has been shown to improve glucose tolerance and insulin resistance^56–58^. Of particular interest, our analyses revealed a relationship between Wnt signalling and predicted miR-330-3p targets. Wnt signalling is associated with T2DM through Wnt signalling pathway effector TCF7L2, implicated as one of the strongest candidate genes in the pathogenesis of impaired beta-cell function and insulin secretion in T2DM^59,60^. A kinase enrichment analysis showed that Glycogen synthase kinase 3 (GSK3), involved in the regulation of Wnt and insulin signalling, shares a significant number of phosphorylation targets with the TargetScan gene predictions. GSK3 is a negative regulator of insulin signalling, strongly linked with insulin resistance and numerous studies targeting GSK3 provide compelling evidence for GSK3-inhibition improving insulin sensitivity as a therapeutic target in T2DM^61–65^. Furthermore, GSKIP (GSK3B Interacting Protein), a negative regulator of GSK3-beta, is a predicted target of miR-330-3p and of significant interest, increased levels of adipose and skeletal muscle GSK3-beta have been observed in GDM patients^66^. Given our findings in our GDM cohort, miR-330-3p is uniquely poised in the coordination of an adaptive response to progressive IR and subsequent increased insulin demand. Modulation of these target genes in response to onset of hyperglycaemia and diabetic phenotype may play key roles in maternal glycaemic homeostasis, disease progression and pregnancy outcomes, with individuals expressing higher levels of miR-330-3p achieving better outcomes, glycaemic management and lower risk for future progression. Lower serum miR-330-3p is potentially indicative of inadequate beta-cell adaptation to peripheral IR, resulting in pathological glucose intolerance and hyperglycaemia observed in GDM patients requiring treatment by insulin^4^.

We report upregulation of circulating miR-330-3p associated with maternal glycaemic management and pregnancy outcomes in patients with GDM. This further strengthens the growing body of evidence for miRNAs such as miR-330-3p playing pivotal roles mediating both disease pathology and adaptation to the complex changes related to normal pregnancy, highlighting their potential as novel targets in conditions such as GDM. We report the significant association of miR-330-3p with risk of primary caesarean delivery as a pregnancy outcome well established to be linked with poor maternal glycaemic control, and propose that miR-330-3p levels associated with pregnancy outcome merit further research, with implications for within-cohort discrimination linked to disease severity, functional beta-cell adaptation to peripheral IR and exogenous insulin requirement. We propose that circulating levels of miR-330-3p may ultimately help guide the choice of a personalized therapy in GDM, associated with pregnancy outcomes and/or severity and type of treatment in lean women with GDM.

## Methods

### Patient cohorts

Pregnant women (*n* = 31 GDM patients, *n* = 29 matched nondiabetic controls) attending the Endocrinology and Pregnancy Clinic, Puerta del Mar University Hospital, Cádiz were recruited during the third trimester (26–30 weeks) and followed throughout the pregnancy. Consecutive participants with GDM who met the eligibility criteria were enrolled and nondiabetic controls were age- and BMI-matched to each case. The study and protocol were approved by the Puerta del Mar Hospital Research Ethics Board and carried out in accordance with the principles of the Declaration of Helsinki. Written informed consent was obtained from all participants.

### Subjects of study

#### Inclusion criteria

Universal screening with 50 g glucose load was employed for all participants. Women with a pre-gestational BMI < 25(kg/m^2^) in the second or third trimester were diagnosed with GDM according to the criteria of the National Diabetes Group (NDDG), presenting two abnormally high values on a 3-hour oral glucose tolerance test (OGTT). Diagnostic thresholds were <105, 190, 165 and 145 mg/dl at fasting, 60, 120 and 180 min, respectively. Nondiabetic control participants had blood glucose <140 mg at 60 min post-glucose load. *Exclusion criteria:* Women with a pre-gestational BMI>25 (kg/m^2^); chronic hypertension, or who are treated with antihypertensive drugs; diagnosis of placental insufficiency, pre-gestational diabetes, chronic underlying systemic disease or acute infectious process; smoking; lack of informed consent; positive glutamate dehydrogenase (GAD), and islet antigen 2 (IL-2) antibodies.

### Study variables

#### Clinical and demographic variables

At the time of inclusion in the study, the clinical and demographic characteristics of the participating women were collected. The data are taken from the clinical history; family history of diabetes, age, obstetric history, parity, height, previous and current weight, body mass index, and gestational age. Throughout gestation, data was collected regarding gestational complications, values of blood pressure, levels of HbA1c, type of treatment (diet or insulin), type of delivery (eutocic, dystocic, caesarean), week of end of gestation, weight of the newborn, Apgar test and complications of newborn (hypoglycaemia, hyperbilirubinemia, infections, admission to ICU).

#### Analytical variables

A venous blood sample for biochemical analyses was taken at the time of inclusion into the study, in all cases following a minimum of 8 hours fasting. The blood was maintained at room temperature (RT). For serum collection, blood samples are kept at RT for a minimum of 30 to a maximum of 60 min to allow a clot to form. The samples were then centrifuged at 4 °C, serum was distributed in aliquots and stored at −80 °C.

Glucose was determined in venous blood using the Modular DPD biochemical system (Roche Diagnostics). HbA1c was measured in the Cobas Integra 700 auto-analyzer (Roche Diagnostics) using an immunoturbidimetric method for completely haemolyzed, anti-coagulated blood. The laboratory reference range for healthy individuals was 4.5–5.7%. Lipid profiles, including total cholesterol (Total-chol), triglycerides (TG), LDL-cholesterol (LDL-chol) and HDL-cholesterol (HDL-chol) are quantified in the Modular DPD biochemical auto-analyzer using enzymatic colorimetry.

### RNA isolation from serum

Total RNA enriched with miRNAs was isolated from serum samples (200 μl), taken from 31 patients with GDM and 29 nondiabetic control pregnant women, using the miRNeasy Serum/Plasma kit (Qiagen) according to manufacturer's instructions. Synthetic *C. elegans* miRNA (cel-miR-39) spike-in control was added (50 pmol) to each sample for input normalisation prior to RNA isolation.

### Reverse transcription and quantitative real-time-PCR measurement of circulating miRNAs

Measurement of circulating miRNAs was carried out as previously described^23,24^. Briefly, reverse transcription reactions were performed using a fixed input volume of 3 µl serum RNA using the Taqman miRNA Reverse Transcription kit (Applied Biosystems) according to the manufacturer's instructions with miRNA-specific TaqMan microRNA Assays for miRNAs of interest: hsa-miR-224-5p (assay ID_002099), hsa-miR-103-3p (assay ID_000439), hsa-miR-330-3p (assay ID_000544), hsa-miR-206, (assay ID_000510) and cel-miR-39 (assay ID_000200) (Applied Biosystems). Quantitative real-time PCR (qPCR) was performed using TaqMan Universal PCR Master Mix according to the manufacturer's instructions with an input volume of 1.33 µl RT product in a total reaction volume of 20 µl and cycled using the StepOnePlus™ Real-Time PCR System (Applied Biosystems). Cycling parameters were as follows: 95 °C for 10 min, followed by 40 cycles of 95 °C 15 s and 60 °C 1 min. All PCR amplification reactions were carried out using miRNA-specific Taqman assays. Specific amplification was carried out for internal control cel-miR-39 (serum spike-in) for sample input normalisation by a median normalization procedure as previously described^67^. For generation of standard curves, synthetic single-stranded RNA oligonucleotides corresponding to the mature miRNA sequences of each miRNA (Sigma Aldrich) in serial 10-fold dilutions were reverse transcribed and run in parallel with the serum samples. Data were normalised to synthetic cel-miR-39 spike-in control and target copy number calculated from standard curves. Serum samples were measured in triplicate using synthetic single-stranded RNA Oligonucleotides as standards to obtain absolute miRNA copy numbers which were then normalized against spiked-in synthetic *C. elegans* miRNA control.

### Bioinformatic target prediction and analysis

TargetScan (Release 7.2: March 2018) was used to predict gene targets for miR-330-3p^68^. Gene ontology enrichment analysis and alternative analyses were carried out using EnrichR (version: January 23^rd^, 2019)^69^. An adjusted p-value cut-off of below 0.05 was used to filter results. The code used in the bioinformatic analyses can be found here: https://github.com/GiantSpaceRobot/TargetScan-to-GO.

### Statistical analysis

Descriptive statistics of the variables measured are presented as mean, median, standard deviation (SD) and percentages for the qualitative variables. The Kolmogorov-Smirnov and Shapiro-Wilk statistical tests were used to test the normality of the distributions. Comparisons between quantitative variables and groups were with the Student’s t test for parametric variables. Non-parametric variables were evaluated with the Mann–Whitney *U* test or Kruskal-Wallis test. For each categorical variable of interest, associations between the dependent and independent variables were determined using the Mantel–Henszel v2 test with Yates’ correction. The Fisher test was applied in the case that the variable contained less than 5 measurements. The magnitude of association was calculated using the odds ratio [OR] with the precision described by the 95% confidence interval (95% CI) using the Cornfield approximation. Multivariate analysis was performed using non-conditional logistic regression. The stepwise technique was used to select the independent variables introduced into the model. The initial criterion for acceptance was a level of significance of *p* = 0.05. Statistical analyses were performed with the Statistical Package for Social Sciences (SPSS version 25.0 for Windows).

## Supplementary information

Supplementary data.

## Data Availability

All data generated or analysed during this study are included in this published article and its Supplementary Information files.
