# Supplementary material for: Circulating miR-330-3p in Late Pregnancy is Associated with Pregnancy Outcomes Among Lean Women with GDM
Source: Sci Rep. 2020 Jan 22;10:908. doi: 10.1038/s41598-020-57838-6 (PMC6976655; doi:10.1038/s41598-020-57838-6)
Supplement: Supplementary file 1 — Supplementary data. [file 41598_2020_57838_MOESM1_ESM.zip › Supplimentary File_EnrichR_Analysis Chromosome_Location_hg19.pdf]

Term	Overlap	P.value	Adjusted.P.va	Old.P.value	Old.Adjustec
chr5	74/1265	0,140522513	1	0	0
chr4_ctg9_hap1	1/6	0,272334438	1	0	0
chr10	55/1074	0,544395219	1	0	0
chr18	20/411	0,639322352	1	0	0
chr17_ctg5_hap1	1/22	0,688439703	1	0	0
chr4	47/1031	0,833411266	1	0	0
chr14	40/898	0,855090794	1	0	0
chrX	51/1137	0,871504196	1	0	0
chr3	69/1513	0,877536318	1	0	0
chr2	77/1751	0,943935309	1	0	0
chr1	119/2689	0,972972297	1	0	0
chrY	3/141	0,978722323	1	0	0
chr21	11/365	0,983088236	1	0	0
chr6_apd_hap1	2/115	0,983807014	1	0	0
chr8	37/996	0,988574062	1	0	0
chr12	52/1351	0,991846245	1	0	0
chr6_mann_hap4	3/181	0,996065394	1	0	0
chr22	19/623	0,996356476	1	0	0
chr6_ssto_hap7	3/190	0,997342065	1	0	0
chr13	18/613	0,997563372	1	0	0
chr6_mcf_hap5	3/207	0,99874526	1	0	0
chr6_dbb_hap3	3/210	0,998902193	1	0	0
chr6_qbl_hap6	3/218	0,999232352	1	0	0
chr9	35/1092	0,999452682	1	0	0
chr15	30/972	0,999477429	1	0	0
chr20	21/757	0,9996181	1	0	0
chr6_cox_hap2	3/235	0,999643007	1	0	0
chr11	54/1651	0,999945938	1	0	0
chr7	39/1295	0,999954004	1	0	0
chr16	30/1113	0,999982932	1	0	0
chr17	43/1529	0,999992693	1	0	0
chr6	38/1416	0,999993203	1	0	0
chr19	16/1756	0,999993585	1	0	0

Odds.Ratio	Combined.Score
1,13368263	2,224724703
3,22997416	4,201306255
0,992450161	0,603488886
0,943058149	0,421873751
0,880902044	0,328864994
0,883465289	0,16099215
0,863244764	0,135138917
0,869280645	0,119556165
0,883813154	0,115458643
0,852226192	0,049171443
0,857642823	0,023499129
0,412337127	0,008868262
0,584050122	0,009961793
0,337040782	0,005502368
0,719934	0,008273277
0,745930376	0,006107065
0,3212129	0,001266339
0,59103861	0,002157396
0,305997552	0,000814404
0,569065594	0,001388293
0,280867318	0,000352637
0,276854928	0,0003041
0,266695114	0,000204807
0,621148877	0,000340059
0,598143363	0,000312654
0,537617892	0,000205356
0,247402276	8,83367E-05
0,633865311	3,4269E-05
0,583640119	2,68457E-05
0,522367789	8,9157E-06
0,545018531	3,98238E-06
0,520080585	3,53494E-06
0,176581731	1,1328E-06

Genes

RAB3C;TCERG1;TNFAIP8;ANKRD33B;MAML1;SETD9;PRDM6;KIAA0141;PPWD1;PHAX;AFF4;CDH6;ADAMT
YTHDC1
NPFFR1;HPSE2;FRMPD2;ADK;LOXL4;NUDT5;BMI1;MYPN;FAM204A;FAM107B;TIAL1;ARL5B;SH3PXD2A;EI
ZBTB14;ONECUT2;ROCK1;ESCO1;TXNL1;ST8SIA3;GATA6;PTPRM;TRAPPC8;LDLRAD4;APCDD1;SMAD7;C
KANSL1
OTUD4;PRDM8;GPM6A;SCOC;TBC1D19;GABRB1;TENM3;FAM114A1;HPGD;UBA6;YTHDC1;ADH1B;HSPA4
ABHD4;CHURC1-FNTB;NRXN3;DLST;GSKIP;SIX1;HIF1A;FBLN5;NOP9;EFS;AP5M1;SIPA1L1;PABPN1;SLC2
ALAS2;COX7B;FRMPD4;SH3KBP1;IRS4;PHF8;AMOT;RBM3;MED14;NHSL2;RPS6KA6;NKRF;GPRASP2;IL13
GPR27;SLC7A14;ZBTB20;CLDN1;LMOD3;SERP1;MECOM;RUVBL1;DAG1;TMEM108;UBXN7;SCN5A;SEC62
FAM49A;NCKAP1;NRP2;USP37;BMPR2;DUSP19;RND3;HK2;GLS;EFEMP1;DPYSL5;KIF5C;PAPOLG;PSD4;A
TDRKH;CHRM3;GABPB2;TMEM167B;POGK;HNRNPU;HNRNPR;ELK4;RIMS3;RAVER2;SNIP1;GJA9;PTGFR
PCDH11Y;SPRY3;ZFY
RCAN1;ADAMTS5;CXADR;C21ORF59;NRIP1;LTN1;DYRK1A;S100B;BACH1;JAM2;RUNX1
TAP2;GNL1
DOCK5;TNKS;PLAG1;MTMR9;AARD;JPH1;MTDH;AP3M2;SDR16C5;EFR3A;TMEM65;ZNF706;ERI1;TRPS1;T
HRK;UHRF1BP1L;BTG1;KCNC2;DENND5B;TM7SF3;NUDT4;EPS8;ING4;MRPL42;SART3;ALDH2;MFSD5;DI
PRR3;TAP2;GNL1
CBX6;MTMR3;ST13;KIAA1671;MIEF1;UQCR10;GRAMD4;RBX1;BCL2L13;ZNF70;ZNRF3;MB;MAPK1;ASPHD
PRR3;TAP2;GNL1
MBNL2;ATP8A2;ZDHHC20;SPRYD7;GPALPP1;SMAD9;FNDC3A;ATP11A;KCNRG;FOXO1;RAP2A;HSPH1;UB
PRR3;TAP2;GNL1
PRR3;TAP2;GNL1
PRR3;TAP2;GNL1
SLC24A2;SET;PALM2;DIRAS2;PRUNE2;TYRP1;GLIS3;ZDHHC21;RORB;ELAVL2;ALAD;TMEM245;RXRA;TM
SHC4;ABHD2;SNAP23;ETFA;FMN1;ABHD17C;CALML4;CTDSPL2;DTWD1;SNX1;SPRED1;APH1B;SIN3A;CH
NAPB;MOCS3;NECAB3;STAU1;PHF20;SPATA2;CBFA2T2;GPCPD1;SULF2;ITCH;RBL1;SNPH;VAPB;SALL4;E
PRR3;TAP2;GNL1
CREBZF;CELF1;PDE3B;RSF1;MED17;IL18BP;GRM5;SESN3;TRIM3;FBXO3;SLC25A45;SOX6;RBM7;TEAD1;
CCDC71L;RBM28;ADCYAP1R1;RALA;DLX6;SEMA3A;PPP1R9A;CYTH3;KIAA1549;ZNRF2;TMEM248;KIAA08
PHLPP2;SF3B3;LITAF;JPH3;EEF2K;ZNF629;ATXN1L;GSE1;ZNF423;UNKL;VKORC1;CREBBP;SEPT12;HSDL
ANKRD13B;HDAC5;PSMD11;NUFIP2;CALCOCO2;SRSF1;TUBD1;PPM1E;IKZF3;ZBTB4;PFAS;PITPNC1;DCA
ZNF292;PRR3;KLHL32;TNFAIP3;COL19A1;CTGF;UBE2J1;PCMT1;OGFRL1;CYB5R4;CCND3;BCLAF1;RNF2
KANK2;NAPA;SYT5;SHC2;RANBP3;TSHZ3;KLK13;ACTN4;EPOR;RNF126;CDC34;KIAA0355;IGLON5;CDC37

TS2;DPYSL3;EMB;PELO;ZNF366;PCDHAC2;PCDHAC1;CCDC112;UTP15;CSNK1G3;C5ORF15;TTC33;E[illegible]

PC1;BTRC;PRKG1;FBXW4;ZCCHC24;ENTPD1;WDR37;GRID1;SFMBT2;KIAA1462;ARID5B;SORCS1;GT
[illegible]TIF;CDH2;VAPA;PQLC1;ASXL3;ZNF236;MAPRE2;RNF165

[illegible]L;MRFAP1;NDNF;PPM1K;ANTXR2;AFF1;GNRHR;APBB2;SLIT2;EIF4E;N4BP2;ATOH1;GALNT7;SEPT11;
2A17;FNTB;FAM177A1;MAP3K9;BCL2L2-PABPN1;ZC3H14;SNX6;GPR135;MAPK1IP1L;RAB2B;NAA30;E
[illegible]RA1;PCYT1B;CHST7;GAB3;SMC1A;ZFX;SYTL4;RAP2C;IL1RAPL1;ARMCX6;DCX;TBC1D25;AGTR2;AM
[illegible];BSN;AP2M1;CCDC50;C3ORF14;APPL1;KPNA1;EOMES;MME;NSUN3;C3ORF62;CMC1;MSL2;FNDC3B;
[illegible]RL5A;CNPPD1;SCN1A;TNS1;KCMF1;ST6GAL2;DNMT3A;AHSA2;GPR75;ADRA2B;CREB1;HECW2;TET[illegible]
N;ZNF687;IL6R;WLS;KCNH1;PRKAB2;ALG6;TPM3;BROX;ANGEL2;VASH2;ALG14;RC3H1;YOD1;POU3F

TMEM68;PSD3;RSPO2;HAS2;PTK2B;LYN;KLF10;NCOA2;ARHGEF10;FZD3;ZBTB10;TMEM64;HOOK3;G[illegible]
[illegible]2B;TSFM;ANO6;HCFC2;PGAM5;ERGIC2;SRSF9;USP15;DCTN2;PDE1B;AGAP2;PPM1H;TMTC3;DTX1;[illegible]

[illegible]2;PIM3;SNRPD3;NPTXR;DGCR2;TNRC6B

[illegible]L3;GJA3;XPO4;GPC5;WDFY2;LNX2

EM203;FRRS1L;S1PR3;ZNF367;SH3GL2;ABCA1;AAED1;ABCA2;ZNF462;FAM78A;OMD;SUSD1;ZBTB34
[illegible]P1;EMC7;RNF111;UBL7;MEF2A;CA12;MAP2K1;VPS13C;TMOD3;SLC30A4;TMOD2;SORD;RAB11A;PIA[illegible]
[illegible]2F1;MACROD2;SERINC3;RBM12;LSM14B;CDS2;TGM2

;FNBP4;USP47;SWAP70;MYEOV;USP2;ZFP91;SLC39A13;DKK3;FCHSD2;MYOD1;SIK3;SIK2;PAFAH1B2
[illegible]395;UBN2;TRIM24;HOXA3;SOSTDC1;ZNF800;SNX8;AGFG2;PSPH;SRPK2;MGAM;GPR37;COA1;CCZ1B;
L1;MMP2;NFATC3;MTSS1L;BFAR;SHISA9;CDYL2;DDX19B;MAF;ADCY9;PDP2;CRISPLD2;RFWD3;CDIP[illegible]
[illegible]F7;TXNDC17;GJC1;GNA13;EFNB3;FADS6;C1QBP;CHAD;IGF2BP1;METTL16;MYH10;HS3ST3B1;SMUR
17;PGM3;SH3BGRL2;HIVEP2;TFAP2B;TPBG;B3GAT2;TAP2;TFEB;C6ORF62;DEK;SOD2;BTBD9;GNL1;E
[illegible];CARM1;DAND5

BF1;ACSL6;PCDHA13;PCDHA12;PCDHA11;PCDHA10;ADAM19;SUB1;RAPGEF6;DCP2;KCTD16;PPIC;R

PBP4;PAX2;ADRA2A;NRG3;ADAM12;LCOR;COMMD3-BMI1;TFAM;WDFY4;SLC29A3;PRTFDC1;CHST3;

PLA2G12A;PCGF3;LGI2;GAB1;HAUS3;NSG1;PHOX2B;EREG;LETM1;GPRIN3;MAPK10;FRAS1;TMEM33
IF2B2;TMED8;BCL11B;ITPK1;RNASE6;YLPM1;DIO2;SYNJ2BP;TSHR;TTC9;ALDH6A1;HNRNPC;CALM1;
ER1;ZNF275;BHLHB9;XIAP;DRP2;PGRMC1;LONRF3;MBNL3;RAI2;PLXNA3;MCTS1;IGBP1;MAGEE1;US
;IL17RD;SENP2;NAALADL2;SUMF1;KAT2B;ACAP2;RAP2B;TBL1XR1;CLDN18;CD47;DENND6A;COL6A5
3;FAM84A;RBMS1;MAPRE3;SLC25A12;TRIB2;DLX1;INSIG2;CUL3;ATL2;SLC1A4;HOXD12;TTL;GIGYF2;
1;HPCAL4;RUNX3;MOB3C;C1ORF115;KIAA1614;CMPK1;TCEANC2;KANK4;PTGFR;IGSF3;C1ORF52;ZN

DF6;SORBS3;AZIN1;SFRP1;FAM167A;RRM2B;CRISPLD1;MICU3;LRP12;FGFR1
CREBL2;PRICKLE1;TRHDE;LRP6;RIC8B;TDG;NT5DC3;MLEC;TAC3;HOXC8;CBX5;FOXJ2;GATC;RASSF

;PAX5;GAPVD1;C9ORF64;HNRNPK;ABHD17B;SLC25A51;NAA35;LHX6;SLC28A3
1;EIF3J;ASB7;SCG3

;TBCEL;MYRF;ATL3;DCUN1D5;ZBTB44;DTX4;NPAT;NXF1;RNF214;ARHGAP20;CARNS1;SLC17A6;SCN
;CNPY1;PDAP1;INHBA;LSM5;FOXP2;HIPK2;FAM133B;RNF148;SP4;KLHL7;FAM185A;ELMO1;ARHGEF5
1;TERF2IP;ZNF597;GLYR1
RF2;MRPL27;UBE2G1;HELZ;DYNLL2;ABHD15;DHX40;MYO1C;KANSL1;PPP1R1B;NAA38;ANKRD40;SPE
SR1;FBXO30;GTF2H5;ADAT2;PTP4A1;MARCKS;RCAN2;CNKSR3;ZNF318;ID4;PDE7B;FRK

RNF180;NPR3;RHOBTB3;MAT2B;JAKMIP2;PPP2CA;NEURL1B;PURA;PCDHA1;MIER3;NSD1;G3BP1;PCD

PFKFB3;ARL3;FUT11;ADD3;ZNF25;PAPSS2;TNKS2;VTI1A;EIF4EBP2;MCMBP;SMNDC1;ACBD5;KCNIP2

3;STIM2;OSTC;OCIAD1;WDFY3;PDE5A;SPRY1;NAT8L;UBE2K
;SOS2;TECPR2;DNAL1
SP9X;GABRA3;L1CAM;SMARCA1;CLCN5;GLUD2;DIAPH2;XK;RPA4;RLIM;SPRY3;OTC
;RAF1;CGGBP1;FYTTD1;USP13;PPM1L;SHOX2;DCUN1D1;PDHB;MED12L;NCKIPSD;FBXO40;ATXN7;Z
FAM117B;ADD2;TRAK2;DFNB59;GBX2;MAT2A;SERTAD2;ERBB4;MGAT5;GPC1;VPS54;RAB11FIP5;MAF
MPSTE24;NKAIN1;RAP1A;PRDM16;PLXNA2;CLSPN;RIMKLA;SRGAP2;MARK1;EDEM3;B3GALNT2;CAD

F8;PTPN11;ATP2B1;TBX5;GRIN2B;C12ORF74;TPCN1;PARP11;DPY19L2;SP1;ETNK1;METAP2

I3B;MPZL3;RELT;TRIM44;TMEM86A;RRM1;CPSF7;ZBTB16;GTF2H1;PARVA;SORL1;SYT7;CAPRIN1;C11
;FKBP6

EM1;AIPL1;SMTNL2;CRK;TMEM106A;NFE2L1;YPEL2

DHA5;PCDHA4;PCDHA3;PCBD2;PCDHA2;PCDHA9;PCDHA8;PPARGC1B;PCDHA7;PCDHA6;GABRA1;T

2;GPAM;CCDC6;PDCD4;CDK1;BMPR1A

ZMAT3;TRA2B;IP6K1;RPP14;ZNF148;TIMMDC1;CRBN;TIPARP;EDEM1;CNBP;SS18L2;ATP2B2;U2SURP;
P3K2;SEPT10;EGR4;FZD5;XRCC5;GFPT1;SLC4A10;NBEAL1;PLEKHA3;KLHL23;MARCH7;ASPRV1;PUM
M3;PHC2;RABGAP1L;NEK7;SELE;EFNA3;ZZZ3;AGO1;FUBP1;REN;LHX4;CC2D1B;DYRK3;TTC22;CEL

ORF57;RAD9A

RIM41;SRD5A1;CSNK1A1;PCDHB15;FBXL17;SREK1IP1;LNPEP;CNOT6;OCLN;CDKN2AIPNL;APC;ZFP6

;STXBP5L;BFSP2;ARHGAP31;NCEH1;CTNNB1;OSTN
12;REEP1;SESTD1;ID2;CCNYL1;NABP1;STRN;FAM171B;TRIP12;PTPN4;MXD1;PLEKHM3;ITM2C
F3;RCSD1;SMG7;CDC73;CKS1B;CAPZB;MAN1A2;DDI2;KIF1B;CEP170;CTBS;PHACTR4;NDC1;DFFA;SG

[illegible]2;CAMK4;JMY;SSBP2

[illegible]IP1;LRRC40;PROX1;DIEXF;ENAH;TGFBR3;NOS1AP;ST6GALNAC3;DTL;CRP;LUZP1;FAM129A;LRP8;Z

DHHC18;RBBP5;USP1;S1PR1;LRIG2;SRSF10;S100PBP;SLC25A24;PRRX1;SORT1;FAM46C;ATP2B4;LA

APTM5;C1ORF21;HIPK1;CDC42BPA;GATAD2B;HEYL;NECAP2;NFIA;PITHD1;TMEM56;KIF26B;CAPZA1;[illegible]
