# Supplementary material for: Circulating miR-330-3p in Late Pregnancy is Associated with Pregnancy Outcomes Among Lean Women with GDM
Source: Sci Rep. 2020 Jan 22;10:908. doi: 10.1038/s41598-020-57838-6 (PMC6976655; doi:10.1038/s41598-020-57838-6)
Supplement: Supplementary file 1 — Supplementary data. [file 41598_2020_57838_MOESM1_ESM.zip › Supplimentary File_EnrichR_Analysis CORUM.pdf]

Term
RICH1/AMOT polarity complex, Flag-Rich1 precipitated (human)
SMAD7-SMURF2 complex (human)
SP1-E2F1 complex (mouse)
ITCH-FAM/USP9x complex (human)
SNF2L-RSF1 complex (human)
Grin2b-Lrp8 complex (mouse)
Car-Lnx2 complex (mouse)
SNX complex (SNX1, SNX6) (human)
MMP-2-claudin-1 complex (human)
Emerin complex 52 (human)
BP-SMAD complex (human)
APC-Axin-1-beta-catenin complex (human)
Mad-Max-mSin3a complex (mouse)
MAD1-mSin3A-HDAC2 complex (human)
CAND1-CUL3-RBX1 complex (human)
GABP(gamma)1-E2F1-DP1 complex (human)
Trip(Br2)-Dp1-E2F1 complex (mouse)
E2F1-p107-cyclinA complex (human)
CBP-RARA-RXRA-DNA complex, ligand stimulated (human)
TCF4-CTNNB1-CREBBP complex (human)
ELMO1-DOCK1-CRKII complex (human)
Scrib-APC-beta-catenin complex (mouse)
Axin2-Ctnnb1-Apc complex (mouse)
BMI1-HPH1-HPH2 complex (human)
RAF1-MAP2K1-YWHAE complex (human)
Raf1-PP2A core enzyme complex (Ppp2r1a, Ppp2ca), untreated (mouse)
Tankyrin 1-tankyrin 2-TRF1 complex (human)
Ubiquitin E3 ligase (BMI1, SPOP, CUL3) (human)
Ubiquitin E3 ligase (CDC34, CUL1, RBX1) (human)
Emerin complex 25 (human)
Multisubunit ACTR coactivator complex (human)
AXIN-APC-betaCatenin-GSK3B complex (human)
Raf1-PP2A holoenzyme complex (Ppp2r1a, Ppp2r2b, Ppp2ca), PDGF stimulated (mouse)
KSR1-RAF1-MEK complex (human)
SRC-1 complex (human)
TFTC-type histone acetyl transferase complex (human)
Ubiquitin E3 ligase (SKP1A, SKP2, CUL1, CKS1B, RBX1) (human)
MAP2K1-BRAF-RAF1-YWHAE-KSR1 complex (human)
Ubiquitin E3 ligase (CDC34, NEDD8, BTRC, CUL1, SKP1A, RBX1) (human)
Ubiquitin E3 ligase (SMAD3, BTRC, CUL1, SKP1A, RBX1) (human)
TRF-Rap1 complex I, 2MD (human)
Ksr1-Mek-Braf-Erk complex, EGF induced (mouse)
LSm2-8 complex (human)
SRC-3 complex (human)
TERF2-RAP1 complex (human)
MLL1-WDR5 complex (human)
CIN85 homotetramer complex (human)
Myod1 homodimer complex (mouse)
EPOR receptor complex (human)
p27-cyclinE-Cdk2 - Ubiquitin E3 ligase (SKP1A, SKP2, CUL1, CKS1B, RBX1) complex (human)
NuA4/Tip60-HAT complex B (human)
BRD4 complex (human)

Ubiquitin E3 ligase (CSN1, CSN8, HRT1, SKP1, SKP2, CUL1, CUL2, CUL3) (human)
DMAP1-associated complex (human)
CRSP-Mediator 2 complex (human)
TFIIH transcription factor complex (human)
B-Ksr1-MEK-MAPK-14-3-3 complex (mouse)
BCL6-HDAC5 complex (human)
HDAC4-ERK2 complex (human)
SMAD1-CBP complex (human)
MAD-MAX complex (human)
CCND3-CDK4 complex (human)
CCND3-CDK6 complex (human)
Bipartite complex (TFC4, CTNNB1) (human)
Ku antigen complex (human)
CIN85-BLNK complex (human)
USP1-UAF1 complex (human)
Frs2-Shp2 complex, FGF stimulated (mouse)
Grb2-Shp2 complex, FGF stimulated (mouse)
Shp2-Sos complex, FGF stimulated (mouse)
LMO4-CREB complex (human)
PDGFRA-SHP-2 complex, PDGF stimulated (human)
GRB2-SHP-2 complex, PDGF stimulated (human)
FHL2-CREB complex (human)
FHL3-CREB complex (human)
ACT-CREB complex (human)
Farnesyltransferase (human)
Beta-dystroglycan-caveolin-3 complex (human)
Klf5-Pias1 complex (mouse)
Angiomotin isoform p80-Angiostatin complex (mouse)
CREBBP-SMAD2 hexameric complex (human)
CREBBP-SMAD3 hexameric complex (human)
RCP-Rab11 complex (human)
Mlx-Mad1 complex (mouse)
SMAD6-HOXC8 complex (human)
Catulin (alpha) - catenin (beta) complex (human)
Catenin (alpha) - catenin (beta) complex (human)
GR-hnRNP U complex (human)
RANBMP-CD39 complex (human)
SMC1-SMC3 complex (human)
TCF4-CTNNB1 complex (human)
Cell-cell junction complex (CDH1-CTNNB1) (human)
SNARE complex (STX2, SNAP23) (human)
SNARE complex (STX4, SNAP23) (human)
CDH1-CKS1B complex (human)
SNARE complex (STX6, SNAP23) (human)
ELMO1-DOCK1 complex (human)
ELMO1-DOCK2 complex (human)
ARNT-HIF1A complex (human)
Myod1-Tcf3 complex (mouse)
SHC3-GAB1 complex (human)
EDG1-HTR1D complex (human)
SNARE complex (SNAP23, STX1A) (human)
CERF complex (CECR2-containing remodeling factor complex) (human)
Remodeling and spacing factor (RSF) complex (human)

HTR1A-EDG3 complex (human)
HTR1A-EDG1 complex (human)
SCRIB-APC complex (human)
CRKII-C3G complex (human)
EPO-EPOR complex (human)
Grin2a-Lrp8 complex (mouse)
CCNB2-CDC2 complex (human)
CCNB1-CDC2 complex (human)
HNF4A-SUB1 complex (human)
CDC2-CCNA2 complex (human)
PCNA-PAF complex (human)
p33ING1b-PCNA complex (human)
Stat1-alpha-dimer-CBP DNA-protein complex (human)
Car-Jam3 complex (mouse)
GRASP-GRP1 complex (human)
Cdh23-Myo1c complex (mouse)
ESPL1-CDC2 complex (human)
CyclinB1-Cdc2 complex (mouse)
Psd3-Actn1 complex (mouse)
NRP2-VEGFC complex (human)
NRP2-VEGFD complex, heparin dependent (human)
NRP2-VEGFR3 complex (human)
2AR-mGluR2 complex (human)
PLXNA2-RANBPM complex (human)
PLXNA3-RANBPM complex (human)
RUNX1-CBF-beta-DNA complex (human)
NCBP-NIP1 complex (human)
RAF1-BRAF complex, RAS stimulated (human)
RAF1-CNK1 complex, RAS stimulated (human)
TRF2-Rap1 complex III (human)
pRB-E2F-1 complex (human)
FOXO3-PCAF complex, oxidative stress stimulated (human)
SP1-MCAF2 complex (human)
RIAM-Rap1-GTP complex (human)
FN1-TGM2 complex (human)
p53-SP1 complex (human)
MT1-MMP-claudin-1 complex (human)
ER-alpha-c-Jun complex (human)
TEAD1-YAP DNA-protein complex (mouse)
DGCR8 multiprotein complex (human)
CRSP complex (human)
NK-3-Groucho-HIPK2-SIN3A-RbpA48-HDAC1 complex (human)
Ksr1 complex (Ksr1, Mek, 14-3-3, Mapk), EGF stimulated (mouse)
Rap1 complex (human)
C complex spliceosome (human)
UTX-MLL2/3 complex (human)
PC2 complex (human)
RNA polymerase II complex, chromatin structure modifying (human)
Polycomb repressive complex 1 (PRC1, hPRC-H) (human)
STAGA complex (SPT3-TAF9-GCN5 acetyltransferase complex) (human)
ARC92-Mediator complex (human)
H2AX complex, isolated from cells without IR exposure (human)
PLC-gamma-2-Lyn-FcR-gamma complex (human)

Ku antigen-YY1-alphaMyHC promoter complex (human)
CRK-BCAR1-DOCK1 complex (human)
nNos-Capon-Dexras1 complex (mouse)
Ku antigen-NARG1 complex (human)
EB1-APC-mDia1 complex (mouse)
Sos1-Abi1-Eps8 complex (mouse)
Src1-Ep300-Crebbp complex (mouse)
BMP2-BRIA complex (human)
OGT-TRAK1-TRAK2 complex (human)
Mad-Max-mSin3B complex (mouse)
CAND1-CUL1-RBX1 complex (human)
CAND1-CUL2-RBX1 complex (human)
CAND1-CUL4A-RBX1 complex (human)
CAND1-CUL4B-RBX1 complex (human)
Ubiquitin E3 ligase (SKP1A, BTRC, CUL1) (human)
RalBP1-CDC2-CCNB1 complex (human)
DNA-PK-Ku antigen complex (human)
G protein complex (Hdac5, Gnb1, Gng2) (mouse)
Ubiquitin E3 ligase (DDB1, CUL4A, RBX1) (human)
CREBBP-SMAD2-SMAD4 pentameric complex (human)
CREBBP-SMAD3-SMAD4 pentameric complex (human)
Polycystin-1-E-cadherin-beta-catenin complex (human)
SMURF2-SMAD3-SnoN complex, TGF(beta)-dependent (human)
Ku70/Ku86/Werner complex (human)
p300-CBP-p270 complex (human)
FHL2-p53-HIPK2 complex (human)
Epo-R-PLC-gamma-TRPC2 complex (mouse)
Epo-R-IP(3)R type II-TRPC2 complex (mouse)
EIF3 complex (EIF3B, EIF3J, EIF3I) (human)
CyclinD3-CDK4-CDK6 complex (human)
Trip(Br1)-Dp1-E2F1 complex (mouse)
E2F4-p107-cyclinE complex (human)
SH3KBP1-CBLB-EGFR complex (human)
Sh3kbp1-Cblb-Egfr complex (mouse)
Sh3kbp1-Cblb-Pdgfrb complex (mouse)
YWHAQ-CALM1-CABIN1 complex (human)
p14-Mp1-MEK1 complex (human)
PU.1-SIN3A-HDAC complex (human)
MAF1 complex (human)
p14-Mp1-ERK2 complex (human)
SNARE complex (STX4, SNAP23, VAMP1) (human)
SNARE complex (STX4, SNAP23, VAMP2) (human)
NCOR-SIN3-HDAC1 complex (human)
TCF4-CTNNB1-EP300 complex (human)
VHL-TBP1-HIF1A complex (human)
HIF1A-OS9-EGLN1 complex (human)
HIF1A-OS9-EGLN3 complex (human)
SNARE complex (STX11, VAMP2, SNAP23) (human)
Rb-NeuroD1-Ngfi-B complex (mouse)
LATS1-HTRA2-BIRC4 complex (human)
Lef1-Tle1-Ctnnb1 complex (mouse)
ELMO1-DOCK1-RAC1 complex (human)
6S methyltransferase complex (human)

Actin-ribonucleoprotein complex (POLR2A, GTF2F1, HNRNPU) (human)
Chromatin remodeling complex (TACC2, TACC3, PCAF) (human)
SNARE complex (VAMP3, STX6, VTI1A) (human)
CALM1-FKBP38-BCL2 complex (human)
Fgr-Pyk2-p190RhoGap complex (mouse)
CDC2-CCNB1-CCNF complex (human)
CDC2-CCNB1-PTCH1 complex (human)
CDC2-CCNA2-CDK2 complex (human)
BAR-BCL2-CASP8 complex (human)
Hsp110-Hsc70-Hsp25 complex (mouse)
SNX complex (SNX1a, SNX2, SNX4) (human)
FARP2-NRP1-PlexinA2 complex (human)
FARP2-NRP1-PlexinA3 complex (human)
Ternary complex (LRRC7, CAMK2a, ACTN4) (human)
CEBPE-E2F1-RB1 complex (human)
PCNA-KU antigen complex (human)
CIN85-SH3GL3-CBL complex (human)
Smcb-Smcd-PW29 complex (mouse)
PlexinA1-NRP1-SEMA3A complex (human)
PGAM5-KEAP1-NRF2 complex (human)
Keap1-Nrf2-Cul3 complex (mouse)
GABAA receptor (human)
Smrt-Sin3A-Hdac7 complex (mouse)
TIP49-TIP48-BAF53 complex (human)
HSP90-CDC37-LRRK2 complex (human)
PHAX-CBC complex (cap binding complex) (human)
CAV1-VDAC1-ESR1 complex (human)
FE65-TSHZ3-HDAC1 complex (human)
Ksr1-PP2A core enzyme complex (Ppp2r1a, Ppp2ca), untreated (mouse)
TRF1 telomere length regulation complex (human)
CNK1-SRC-RAF1 complex (human)
SNARE complex (Stx4, Vamp8, Snap23) (mouse)
TRF2-Ku complex (human)
Ubiquitin E3 ligase (H2AFY, SPOP, CUL3) (human)
REST-CoREST-mSIN3A complex (human)
Ubiquitin E3 ligase (SPOP, DAXX, CUL3) (human)
MKK4-ARRB2-JNK3 complex (human)
EB1-APC-mDia2 complex (mouse)
IKK-alpha--ER-alpha-AIB1 complex (human)
AR-AKT-APPL complex (human)
CTGF/Hcs24-actin complex (human)
ITGB3-ITGAV-CD47 complex (human)
ITGA4-ITGB1-JAM2 complex (human)
ITGA4-ITGB1-CD47 complex (human)
ITGA2-ITGB1-CD47 complex (human)
ITGA2-ITGB1-CHAD complex (human)
ITGA9-ITGB1-ADAM12 complex (human)
FOXO1-FHL2-SIRT1 complex (human)
Er-alpha-p53-hdm2 complex (human)
ER-alpha-GRIP1-c-Jun complex (human)
SMAD3-SMAD4-SP1 complex (human)
PIP complex (mouse)
SMAD3-SMAD4-FOXO1 complex (human)

MBD1-Suv39h1-HP1 complex (human)
MDC1-p53BP1-SMC1 complex (human)
BRCA1 B complex (human)
9-1-1 complex (human)
SCF subcomplex (WEE1, SKP2, BTRC) (human)
Ubiquitin E3 ligase (CUL1, RBX1, SKP1A) (human)
RBPJ-NotchIC-Mastermind complex (human)
ARC-L complex (human)
DRIP complex (human)
NuA4/Tip60-HAT complex A (human)
Emerin complex 24 (human)
Smad1-Notch1-p300-Pcaf complex (mouse)
Ubiquitin E3 ligase (SKP1A, FBXW8, CUL7, RBX1) (human)
hNURF complex (human)
Axin-PP2A A-PP2A C-GSK3-beta-beta-catenin complex (human)
SWAP complex (mouse)
Cohesin-SA1 complex (human)
Cohesin-SA2 complex (human)
SKI-NCOR1-SIN3A-HDAC1 complex (human)
Wave-2 complex (mouse)
Gamma-secretase complex (APH1B, PSEN1, PSENEN, NCSTN) (human)
HERP1/HEY2-NCOR-SIN3A complex (human)
Gamma-secretase complex (APH1B, PSEN2, PSENEN, NCSTN) (human)
Xin-Cdh2-Ctnnb1-Ctnnd1 complex (mouse)
Polycystin-1-E-cadherin-beta-catenin-Flotillin-2 complex (human)
SMAD3-cSKI-SIN3A-HDAC1 complex (human)
HSP90-FKBP38-CAM-Ca(2+) complex (human)
Epo-R-PLC-gamma 1-IP(3)R type II-TRPC2 signaling complex (mouse)
p32-CBF-DNA complex (human)
MSL complex (human)
ZO1-(beta)cadherin-(VE)cadherin-VEGFR2 complex (human)
CyclinD3-CDK4-CDK6-p21 complex (human)
MGC1-DNA-PKcs-Ku complex (human)
PU.1-Sin3A-Hdac-MeCP2 complex (mouse)
p14-Mp1-Erk1/2 complex (mouse)
Ubiquitin E3 ligase (FBXO31, SKP1A, CUL1, RBX1) (human)
WRN-Ku70-Ku80-PARP1 complex (human)
NCOR-SIN3-RPD3 complex (human)
MeCP2-SIN3A-HDAC complex (human)
20S methylosome-SmD complex (human)
Polycomb repressive complex (human)
Bmi1-Mel18-Mph1-M33 polycomb repressor complex (mouse)
CDC2-PCNA-CCNB1-GADD45A complex (human)
CDC2-PCNA-CCNB1-GADD45B complex (human)
CDC2-PCNA-CCNB1-GADD45G complex (human)
Ubiquitin E3 ligase (SKP1A, SKP2, CUL1, RBX1) (human)
Ubiquitin E3 ligase (FBXW11, SKP1A, CUL1, RBX1) (human)
ESR1-RELA-BCL3-NCOA3 complex (human)
SNX complex (SNX1a, SNX2, SNX4, LEPR) (human)
SNX complex (SNX1a, SNX2, SNX4, INSR) (human)
SNX complex (SNX1a, SNX2, SNX4, EGFR) (human)
SNX complex (SNX1,1a,2,4, PDGF receptor) (human)
SNX complex (SNX1a, SNX2, SNX4, TFRC) (human)

PS1-E-cadherin-catenin complex, brain (mouse)
SNARE complex (STX4, VAMP8, VAMP3, SNAP23) (human)
BRAF-MAP2K1-MAP2K2-YWHAE complex (human)
Ksr1-PP2A holoenzyme complex (Ppp2r1a, Ppp2r2b, Ppp2ca), PDGF stimulated (mouse)
KSR1-BRAF-MEK complex (human)
RAF1-RAS complex, EGF induced (human)
Ksr1-Mek-Braf complex, EGF induced (mouse)
Ubiquitin E3 ligase (FBXW7, CUL1, SKP1A, RBX1) (human)
Ubiquitin E3 ligase (GLMN, FBXW8, SKP1A, RBX1) (human)
Neddylin ligase (FBXO11, SKP1, CUL1, RBX1) (human)
ING4 complex (ING4, MYST2, C1orf149, PHF17) (human)
Vigilin-DNA-PK-Ku antigen complex (human)
Ubiquitin E3 ligase (CDT1, DDB1, CUL4A, RBX1) (human)
Ubiquitin E3 ligase (FBXO18, SKP1A, CUL1, RBX1) (human)
Ubiquitin E3 ligase (NIPA, SKP1A, CUL1, RBX1) (human)
Ubiquitin E3 ligase (Fbxo32, Skp1a, Cul1, Rbx1) (mouse)
Ubiquitin E3 ligase (Fbxo2, Skp1a, Cul1, Rbx1) (mouse)
LIG1-9-1-1 complex (human)
FEN1-9-1-1 complex (human)
RAD9-RAD1-HUS1-POLB complex (human)
ITGAV-ITGB3-CD47-FCER2 complex (human)
ITGAV-ITGB3-PXN-PTK2b complex (human)
ITGA2B-ITGB3-FN1-TGM2 complex (human)
ITGA2b-ITGB3-CD47-SRC complex (human)
ITGA5-ITGB1-FN1-TGM2 complex (human)
p130Cas-ER-alpha-cSrc-kinase- PI3-kinase p85-subunit complex (human)
PLC-gamma-2-SLP-76-Lyn-Grb2 complex (human)
FGFR2-c-Cbl-Lyn-Fyn complex (human)
p21(ras)GAP-Fyn-Lyn-Yes complex, thrombin stimulated (human)
CD20-LCK-LYN-FYN-p75/80 complex, (Raji human B cell line) (human)
RAD9-RAD1-HUS1-APE1 complex (human)
DAXX-Axin-p53-HIPK2 complex (human)
ING4 complex (ING4, MYST2, C1orf149, PHF15) (human)
ING4 complex (ING4, MYST2, C1orf149, PHF16) (human)
ITGA2b-ITGB3-CD47-FAK complex (human)
NuA4/Tip60 HAT complex (human)
TRAP complex (human)
Mediator complex (mouse)
CF IIAm complex (Cleavage factor IIAm complex) (human)
SMCC complex (human)
TFTC complex (TATA-binding protein-free TAF-II-containing complex) (human)
SMRT complex (human)
SMAD3-E2F4/5-p107-DP1 complex (human)
Wave-2 complex (human)
Wave-2 complex (Rac-activated) (mouse)
Ubiquitin E3 ligase (ASB7, TCEB1, TCEB2, CUL5, RNF7) (human)
Ubiquitin E3 ligase (WSB1, TCEB1, TCEB2, CUL5, RBX1) (human)
Cell cycle kinase complex CDK5 (human)
Rag1-Rag2-Ku70-Ku80 protein-DNA complex (mouse)
TIP60 histone acetylase complex (human)
Decapping complex (human)
EIF3 complex (EIF3A, EIF3B, EIF3G, EIF3I, EIF3J) (human)
Retromer complex (SNX1, SNX2, VPS35, VPS29, VPS26A) (human)

NCOA6-DNA-PK-Ku-PARP1 complex (human)
SWI/SNF chromatin-remodeling complex (human)
RAF1-PPP2-PIN1 complex (human)
53BP1-containing complex (human)
IKBKB-CDC37-KIAA1967-HSP90AB1-HSP90AA1 complex (human)
NCOR-HDAC3 complex (human)
TCF4-CTNNB1-SUMO1-EP300-HADAC6 complex (human)
UTM-SGCE-DAG1-CAV1-NOS3 complex (human)
Kif3-cadherin-catenin complex (mouse)
TNF-alpha/NF-kappa B signaling complex 9 (human)
SNARE complex (VAMP4, STX6, STX16, VTI1a, VTI1b) (human)
Eya1/3-Dach1/2-Six1 complex (mouse)
Splicing-associated factors complex (human)
Agap11-AP3 complex (mouse)
TFIIH transcription factor core complex (human)
LMO4-gp130 complex (human)
Retromer complex (SNX1, SNX2, VPS35, VPS29, VPS26B) (human)
Ubiquitin E3 ligase (DDB1, DDB2, CUL4A, CUL4B, RBX1) (human)
Paf complex (human)
cMYC-ATPase-helicase complex (human)
CRM1-RAN-PHAX-CBC complex (cap binding complex) (human)
PPP2R1A-PPP2R1B-PPP2CA-PPME1-EIF4A1 complex (human)
Ubiquitin E3 ligase (DET1, DDB1, CUL4A, RBX1, COP1) (human)
SRm160/300 complex (human)
Septin complex (human)
SNARE complex (SNAP25, VAMP3, VAMP2, NAPB, STX13) (human)
Ubiquitin E3 ligase (Neurl2, Tceb1, Tceb2, Cul5, Rbx1) (mouse)
Ubiquitin E3 ligase (NFKBIA, FBXW11, BTRC, CUL1, SKP1A) (human)
ARC complex (human)
17S U2 snRNP (human)
LARC complex (LCR-associated remodeling complex) (human)
HCF-1 complex (human)
NCOR complex (human)
Telosome complex (human)
DHX9-ADAR-vigilin-DNA-PK-Ku antigen complex (human)
Kif17-Lin10-Lin2-Lin7-NR2B complex (mouse)
VEGF transcriptional complex (human)
Cell cycle kinase complex CDC2 (human)
NCOR-SIN3-HDAC-HESX1 complex (human)
Sarcoglycan-sarcospan-dystroglycan complex (mouse)
cRET-Shc-Grb2-Gab2-P85PI3K-Shp2 complex (mouse)
Ikaros-NuRD complex (mouse)
SWI/SNF chromatin-remodeling complex (Mecp2, Smarc) (mouse)
Ku-ORC complex (human)
TNF-alpha/NF-kappa B signaling complex 8 (human)
20S methylosome and RG-containing Sm protein complex (human)
TFIIH transcription factor complex (ERCC2, ERCC3, GTF2H1, CDK7, CCNH, GTF2H2) (human)
Ubiquitin E3 ligase (AHR, ARNT, DDB1, TBL3, CUL4B, RBX1) (human)
FIB-associated protein complex (human)
SMAD3/4-E2F4/5-p107-DP1 complex (human)
HES1 promoter corepressor complex (human)
ESR1-CDK7-CCNH-MNAT1-MTA1-HDAC2 complex (human)
Set1B complex (human)

Set1A complex (human)
BRCA1-BARD1-BACH1-DNA damage complex I (human)
ITGA2b-ITGB3-CD9-GP1b-CD47 complex (human)
Large Drosha complex (human)
DNMT3B complex (human)
AP3 adapter complex (human)
60S APC containing complex (human)
NAT complex (human)
SMN1-SIP1-SNRP complex (human)
LSm1-7 complex (human)
p300-CBP-p270-SWI/SNF complex (human)
SWI/SNF-related complex (mouse)
SIN3 complex (human)
Postsplicing complex (human)
Toposome (human)
LINC complex, S-phase (human)
TFIIIC containing-TOP1-SUB1 complex (human)
p400-associated complex (human)
DNA double-strand break end-joining complex (human)
Menin-associated histone methyltransferase complex (human)
MLL-HCF complex (human)
ASCOM complex (human)
NCOR2 complex (human)
SMN complex, U7 snRNA specific (human)
HBO1 complex (human)
Emerin complex 32 (human)
RAD17-RFC-9-1-1 checkpoint supercomplex (human)
HDAC2-asscociated core complex (human)
DNA-PK-Ku-eIF2-NF90-NF45 complex (human)
BRMS1-SIN3-HDAC complex (human)
Kinase maturation complex 2 (human)
TNF-alpha/NF-kappa B signaling complex 7 (human)
6S methyltransferase and RG-containing Sm proteins complex (human)
RICH1/AMOT polarity complex, Flag-Amot precipitated (human)
SF3b complex (human)
SMN containing complex (human)
BRCA1-BARD1-BACH1-DNA damage complex II (human)
18S U11/U12 snRNP (human)
MeCP1 complex (human)
SIN3-ING1b complex I (human)
URI complex (Unconventional prefoldin RPB5 Interactor) (human)
MTA2 complex (human)
Emerin regulatory complex (human)
BRAF-RAF1-14-3-3 complex (human)
Profilin 2 complex (mouse)
NUMAC complex (nucleosomal methylation activator complex) (human)
Rnase/Mrp complex (human)
SRCAP-associated chromatin remodeling complex (human)
PCAF complex (human)
Cytochrome bc1-complex (EC 1.10.2.2), mitochondrial (mouse)
HDAC1-associated core complex cII (human)
TNF-alpha/NF-kappa B signaling complex (SEC16A, CHUK, IKBKB, NFKB2, REL, IKBKG, MAP3K14, RELA, F
PTIP-HMT complex (human)

TNF-alpha/NF-kappa B signaling complex 10 (human)
mRNA decay complex (UPF1, UPF2, UPF3B, DCP2, XRN1, XRN2, EXOSC2, EXOSC4, EXOSC10, PARN) (hu[illegible]
TRAP-SMCC mediator complex (human)
Ksr1 complex (Ksr1, Mek, 14-3-3), unstimulated (mouse)
NCOR1 complex (human)
Kaiso-NCOR complex (human)
RSmad complex (human)
ALL-1 supercomplex (human)
Polycystin-1 multiprotein complex (ACTN1, CDH1, SRC, JUP, VCL, CTNNB1, PXN, BCAR1, PKD1, PTK2, TLN[illegible]
SIN3-SAP25 complex (human)
Ikaros complex (mouse)
TNF-alpha/NF-kappa B signaling complex (CHUK, BTRC, NFKB2, PPP6C, REL, CUL1, IKBKE, SAPS2, SAPS[illegible]
P2X7 receptor signalling complex (human)
BRM-SIN3A-HDAC complex (human)
Ksr1-CK2-MEK-14-3-3 complex, PDGF treated (mouse)
ING2 complex (human)
INO80 chromatin remodeling complex (human)
Cytochrome c oxidase (EC 1.9.3.1), mitochondrial (mouse)
DDB2 complex (human)
CSA complex (human)
RC complex during G2/M-phase of cell cycle (human)
eIF3 complex (EIF3S6, EIF3S5, EIF3S4, EIF3S3, EIF3S6IP, EIF3S2, EIF3S9, EIF3S12, EIF3S10, EIF3S8, EI[illegible]
HES1 promoter-Notch enhancer complex (human)
Gata1-Fog1-MeCP1 complex (mouse)
Mediator complex (human)
BRG1-SIN3A complex (human)
CSA-POLIIa complex (human)
TNF-alpha/NF-kappa B signaling complex 6 (human)
AP3-BLOC1 complex (human)
BRM-SIN3A complex (human)
12S U11 snRNP (human)
SNF2h-cohesin-NuRD complex (human)
Kinase maturation complex 1 (human)
SIN3-ING1b complex II (human)
SMN complex (human)
CEN complex (human)
CtBP complex (human)
Anti-HDAC2 complex (human)
PA700 complex (human)
39S ribosomal subunit, mitochondrial (human)
RNA polymerase II holoenzyme complex (human)
Spliceosome (human)
BRCA1-RNA polymerase II complex (human)
CDC5L complex (human)
Parvulin-associated pre-rRNP complex (mouse)
PA700-20S-PA28 complex (human)
Nop56p-associated pre-rRNA complex (human)
55S ribosome, mitochondrial (human)

Overlap	P.value	Adjusted.P.value	Old.P.value	Old.Adjusted
4/9	0,000720595	1	0	0
2/2	0,002660068	1	0	0
2/2	0,002660068	1	0	0
2/2	0,002660068	1	0	0
2/2	0,002660068	0,882078685	0	0
2/2	0,002660068	0,735065571	0	0
2/2	0,002660068	0,630056204	0	0
2/2	0,002660068	0,551299178	0	0
2/2	0,002660068	0,490043714	0	0
5/23	0,005598899	0,928297415	0	0
3/8	0,00630859	0,950876566	0	0
2/3	0,007706229	1	0	0
2/3	0,007706229	0,982840556	0	0
2/3	0,007706229	0,912637659	0	0
2/3	0,007706229	0,851795148	0	0
2/3	0,007706229	0,798557951	0	0
2/3	0,007706229	0,751583954	0	0
2/3	0,007706229	0,70982929	0	0
2/3	0,007706229	0,672469854	0	0
2/3	0,007706229	0,638846361	0	0
2/3	0,007706229	0,608425106	0	0
2/3	0,007706229	0,580769419	0	0
2/3	0,007706229	0,555518575	0	0
2/3	0,007706229	0,532371968	0	0
2/3	0,007706229	0,511077089	0	0
2/3	0,007706229	0,491420278	0	0
2/3	0,007706229	0,473219527	0	0
2/3	0,007706229	0,456318829	0	0
2/3	0,007706229	0,440583697	0	0
4/16	0,007791259	0,430596907	0	0
2/4	0,014885615	0,796140287	0	0
2/4	0,014885615	0,771260903	0	0
2/4	0,014885615	0,74788936	0	0
2/4	0,014885615	0,725892614	0	0
2/4	0,014885615	0,705152825	0	0
3/11	0,016549986	0,76221881	0	0
2/5	0,02396506	1	0	0
2/5	0,02396506	1	0	0
2/5	0,02396506	1	0	0
2/5	0,02396506	0,99335172	0	0
2/6	0,034729741	1	0	0
2/6	0,034729741	1	0	0
2/7	0,04698191	1	0	0
2/7	0,04698191	1	0	0
2/7	0,04698191	1	0	0
4/27	0,048056222	1	0	0
1/1	0,051599784	1	0	0
1/1	0,051599784	1	0	0
1/1	0,051599784	1	0	0
2/8	0,060539704	1	0	0
2/8	0,060539704	1	0	0
2/8	0,060539704	1	0	0

2/8	0,060539704	1	0	0
2/10	0,090917569	1	0	0
2/10	0,090917569	1	0	0
2/10	0,090917569	1	0	0
2/10	0,090917569	1	0	0
1/2	0,10053959	1	0	0
1/2	0,10053959	1	0	0
1/2	0,10053959	1	0	0
1/2	0,10053959	1	0	0
1/2	0,10053959	1	0	0
1/2	0,10053959	1	0	0
1/2	0,10053959	1	0	0
1/2	0,10053959	1	0	0
1/2	0,10053959	1	0	0
1/2	0,10053959	1	0	0
1/2	0,10053959	1	0	0
1/2	0,10053959	1	0	0
1/2	0,10053959	1	0	0
1/2	0,10053959	1	0	0
1/2	0,10053959	1	0	0
1/2	0,10053959	1	0	0
1/2	0,10053959	1	0	0
1/2	0,10053959	1	0	0
1/2	0,10053959	1	0	0
1/2	0,10053959	1	0	0
1/2	0,10053959	1	0	0
1/2	0,10053959	1	0	0
1/2	0,10053959	1	0	0
1/2	0,10053959	1	0	0
1/2	0,10053959	1	0	0
1/2	0,10053959	1	0	0
1/2	0,10053959	1	0	0
1/2	0,10053959	1	0	0
1/2	0,10053959	1	0	0
1/2	0,10053959	1	0	0
1/2	0,10053959	1	0	0
1/2	0,10053959	1	0	0
1/2	0,10053959	1	0	0
1/2	0,10053959	1	0	0
1/2	0,10053959	1	0	0
1/2	0,10053959	1	0	0
1/2	0,10053959	1	0	0
1/2	0,10053959	1	0	0
1/2	0,10053959	1	0	0
1/2	0,10053959	1	0	0
1/2	0,10053959	1	0	0
1/2	0,10053959	1	0	0
1/2	0,10053959	1	0	0
1/2	0,10053959	1	0	0
1/2	0,10053959	1	0	0
1/2	0,10053959	1	0	0
1/2	0,10053959	1	0	0
1/2	0,10053959	1	0	0

1/2	0,10053959	1	0	0
1/2	0,10053959	1	0	0
1/2	0,10053959	1	0	0
1/2	0,10053959	1	0	0
1/2	0,10053959	1	0	0
1/2	0,10053959	1	0	0
1/2	0,10053959	1	0	0
1/2	0,10053959	1	0	0
1/2	0,10053959	1	0	0
1/2	0,10053959	1	0	0
1/2	0,10053959	1	0	0
1/2	0,10053959	1	0	0
1/2	0,10053959	1	0	0
1/2	0,10053959	1	0	0
1/2	0,10053959	1	0	0
1/2	0,10053959	1	0	0
1/2	0,10053959	1	0	0
1/2	0,10053959	1	0	0
1/2	0,10053959	1	0	0
1/2	0,10053959	1	0	0
1/2	0,10053959	1	0	0
1/2	0,10053959	1	0	0
1/2	0,10053959	1	0	0
1/2	0,10053959	1	0	0
1/2	0,10053959	1	0	0
1/2	0,10053959	1	0	0
1/2	0,10053959	1	0	0
1/2	0,10053959	1	0	0
1/2	0,10053959	1	0	0
1/2	0,10053959	1	0	0
1/2	0,10053959	1	0	0
1/2	0,10053959	1	0	0
1/2	0,10053959	1	0	0
1/2	0,10053959	1	0	0
1/2	0,10053959	1	0	0
1/2	0,10053959	1	0	0
1/2	0,10053959	1	0	0
1/2	0,10053959	1	0	0
1/2	0,10053959	1	0	0
2/11	0,107443713	1	0	0
2/11	0,107443713	1	0	0
2/11	0,107443713	1	0	0
2/11	0,107443713	1	0	0
2/11	0,107443713	1	0	0
7/80	0,118458914	1	0	0
2/12	0,124685754	1	0	0
2/12	0,124685754	1	0	0
3/25	0,136044545	1	0	0
2/13	0,142525984	1	0	0
2/13	0,142525984	1	0	0
2/13	0,142525984	1	0	0
2/13	0,142525984	1	0	0
1/3	0,146956321	1	0	0

1/3	0,146956321	1	0	0
1/3	0,146956321	1	0	0
1/3	0,146956321	1	0	0
1/3	0,146956321	1	0	0
1/3	0,146956321	1	0	0
1/3	0,146956321	1	0	0
1/3	0,146956321	1	0	0
1/3	0,146956321	1	0	0
1/3	0,146956321	1	0	0
1/3	0,146956321	1	0	0
1/3	0,146956321	1	0	0
1/3	0,146956321	1	0	0
1/3	0,146956321	1	0	0
1/3	0,146956321	1	0	0
1/3	0,146956321	1	0	0
1/3	0,146956321	1	0	0
1/3	0,146956321	1	0	0
1/3	0,146956321	1	0	0
1/3	0,146956321	1	0	0
1/3	0,146956321	1	0	0
1/3	0,146956321	1	0	0
1/3	0,146956321	1	0	0
1/3	0,146956321	1	0	0
1/3	0,146956321	1	0	0
1/3	0,146956321	1	0	0
1/3	0,146956321	1	0	0
1/3	0,146956321	1	0	0
1/3	0,146956321	1	0	0
1/3	0,146956321	1	0	0
1/3	0,146956321	1	0	0
1/3	0,146956321	1	0	0
1/3	0,146956321	1	0	0
1/3	0,146956321	1	0	0
1/3	0,146956321	1	0	0
1/3	0,146956321	1	0	0
1/3	0,146956321	1	0	0
1/3	0,146956321	1	0	0
1/3	0,146956321	1	0	0
1/3	0,146956321	1	0	0
1/3	0,146956321	1	0	0
1/3	0,146956321	1	0	0
1/3	0,146956321	1	0	0
1/3	0,146956321	1	0	0
1/3	0,146956321	1	0	0
1/3	0,146956321	1	0	0
1/3	0,146956321	1	0	0
1/3	0,146956321	1	0	0
1/3	0,146956321	1	0	0
1/3	0,146956321	1	0	0
1/3	0,146956321	1	0	0
1/3	0,146956321	1	0	0
1/3	0,146956321	1	0	0
1/3	0,146956321	1	0	0

1/3	0,146956321	1	0	0
1/3	0,146956321	1	0	0
1/3	0,146956321	1	0	0
1/3	0,146956321	1	0	0
1/3	0,146956321	1	0	0
1/3	0,146956321	1	0	0
1/3	0,146956321	1	0	0
1/3	0,146956321	1	0	0
1/3	0,146956321	1	0	0
1/3	0,146956321	1	0	0
1/3	0,146956321	1	0	0
1/3	0,146956321	1	0	0
1/3	0,146956321	1	0	0
1/3	0,146956321	1	0	0
1/3	0,146956321	1	0	0
1/3	0,146956321	1	0	0
1/3	0,146956321	1	0	0
1/3	0,146956321	1	0	0
1/3	0,146956321	1	0	0
1/3	0,146956321	1	0	0
1/3	0,146956321	1	0	0
1/3	0,146956321	1	0	0
1/3	0,146956321	1	0	0
1/3	0,146956321	1	0	0
1/3	0,146956321	1	0	0
1/3	0,146956321	1	0	0
1/3	0,146956321	1	0	0
1/3	0,146956321	1	0	0
1/3	0,146956321	1	0	0
1/3	0,146956321	1	0	0
1/3	0,146956321	1	0	0
1/3	0,146956321	1	0	0
1/3	0,146956321	0,998580248	0	0
1/3	0,146956321	0,99450441	0	0
1/3	0,146956321	0,990461709	0	0
1/3	0,146956321	0,986451743	0	0
1/3	0,146956321	0,982474115	0	0
1/3	0,146956321	0,978528436	0	0
1/3	0,146956321	0,974614322	0	0
1/3	0,146956321	0,970731396	0	0
1/3	0,146956321	0,966879288	0	0
1/3	0,146956321	0,96305763	0	0
1/3	0,146956321	0,959266065	0	0
1/3	0,146956321	0,955504237	0	0
1/3	0,146956321	0,951771799	0	0
1/3	0,146956321	0,948068407	0	0
1/3	0,146956321	0,944393723	0	0
1/3	0,146956321	0,940747415	0	0
1/3	0,146956321	0,937129156	0	0
1/3	0,146956321	0,933538623	0	0
1/3	0,146956321	0,929975498	0	0
1/3	0,146956321	0,92643947	0	0
1/3	0,146956321	0,922930229	0	0

1/3	0,146956321	0,919447474	0	0
1/3	0,146956321	0,915990904	0	0
1/3	0,146956321	0,912560227	0	0
1/3	0,146956321	0,909155151	0	0
1/3	0,146956321	0,905775392	0	0
1/3	0,146956321	0,902420668	0	0
1/3	0,146956321	0,899090703	0	0
2/14	0,160856918	0,98051754	0	0
2/14	0,160856918	0,9769259	0	0
2/15	0,179580553	1	0	0
2/15	0,179580553	1	0	0
1/4	0,190979919	1	0	0
1/4	0,190979919	1	0	0
1/4	0,190979919	1	0	0
1/4	0,190979919	1	0	0
1/4	0,190979919	1	0	0
1/4	0,190979919	1	0	0
1/4	0,190979919	1	0	0
1/4	0,190979919	1	0	0
1/4	0,190979919	1	0	0
1/4	0,190979919	1	0	0
1/4	0,190979919	1	0	0
1/4	0,190979919	1	0	0
1/4	0,190979919	1	0	0
1/4	0,190979919	1	0	0
1/4	0,190979919	1	0	0
1/4	0,190979919	1	0	0
1/4	0,190979919	1	0	0
1/4	0,190979919	1	0	0
1/4	0,190979919	1	0	0
1/4	0,190979919	1	0	0
1/4	0,190979919	1	0	0
1/4	0,190979919	1	0	0
1/4	0,190979919	1	0	0
1/4	0,190979919	1	0	0
1/4	0,190979919	1	0	0
1/4	0,190979919	1	0	0
1/4	0,190979919	1	0	0
1/4	0,190979919	1	0	0
1/4	0,190979919	1	0	0
1/4	0,190979919	1	0	0
1/4	0,190979919	1	0	0
1/4	0,190979919	1	0	0
1/4	0,190979919	1	0	0
1/4	0,190979919	1	0	0
1/4	0,190979919	1	0	0
1/4	0,190979919	1	0	0
1/4	0,190979919	1	0	0
1/4	0,190979919	1	0	0
1/4	0,190979919	1	0	0
1/4	0,190979919	1	0	0
1/4	0,190979919	1	0	0
1/4	0,190979919	0,998879196	0	0

1/4	0,190979919	0,995738066	0	0
1/4	0,190979919	0,99261663	0	0
1/4	0,190979919	0,989514703	0	0
1/4	0,190979919	0,986432103	0	0
1/4	0,190979919	0,983368649	0	0
1/4	0,190979919	0,980324164	0	0
1/4	0,190979919	0,977298473	0	0
1/4	0,190979919	0,9742914	0	0
1/4	0,190979919	0,971302776	0	0
1/4	0,190979919	0,968332432	0	0
1/4	0,190979919	0,965380198	0	0
1/4	0,190979919	0,962445912	0	0
1/4	0,190979919	0,959529409	0	0
1/4	0,190979919	0,956630529	0	0
1/4	0,190979919	0,953749112	0	0
1/4	0,190979919	0,950885	0	0
1/4	0,190979919	0,948038039	0	0
1/4	0,190979919	0,945208075	0	0
1/4	0,190979919	0,942394956	0	0
1/4	0,190979919	0,939598531	0	0
1/4	0,190979919	0,936818654	0	0
1/4	0,190979919	0,934055177	0	0
1/4	0,190979919	0,931307956	0	0
1/4	0,190979919	0,928576848	0	0
1/4	0,190979919	0,925861711	0	0
1/4	0,190979919	0,923162406	0	0
1/4	0,190979919	0,920478794	0	0
1/4	0,190979919	0,917810739	0	0
1/4	0,190979919	0,915158107	0	0
1/4	0,190979919	0,912520764	0	0
1/4	0,190979919	0,909898578	0	0
1/4	0,190979919	0,907291419	0	0
1/4	0,190979919	0,904699157	0	0
1/4	0,190979919	0,902121667	0	0
1/4	0,190979919	0,899558821	0	0
2/16	0,198607672	0,932837167	0	0
2/16	0,198607672	0,930202034	0	0
2/16	0,198607672	0,927581746	0	0
2/16	0,198607672	0,92497618	0	0
2/17	0,217857207	1	0	0
2/17	0,217857207	1	0	0
1/5	0,232733649	1	0	0
1/5	0,232733649	1	0	0
1/5	0,232733649	1	0	0
1/5	0,232733649	1	0	0
1/5	0,232733649	1	0	0
1/5	0,232733649	1	0	0
1/5	0,232733649	1	0	0
1/5	0,232733649	1	0	0
1/5	0,232733649	1	0	0
1/5	0,232733649	1	0	0
1/5	0,232733649	1	0	0
1/5	0,232733649	1	0	0

1/5	0,232733649	1	0	0
1/5	0,232733649	1	0	0
1/5	0,232733649	1	0	0
1/5	0,232733649	1	0	0
1/5	0,232733649	1	0	0
1/5	0,232733649	1	0	0
1/5	0,232733649	1	0	0
1/5	0,232733649	1	0	0
1/5	0,232733649	1	0	0
1/5	0,232733649	1	0	0
1/5	0,232733649	1	0	0
1/5	0,232733649	1	0	0
1/5	0,232733649	1	0	0
1/5	0,232733649	1	0	0
1/5	0,232733649	1	0	0
1/5	0,232733649	0,999669405	0	0
1/5	0,232733649	0,99708628	0	0
1/5	0,232733649	0,99451647	0	0
1/5	0,232733649	0,991959872	0	0
1/5	0,232733649	0,989416385	0	0
1/5	0,232733649	0,986885909	0	0
1/5	0,232733649	0,984368342	0	0
1/5	0,232733649	0,981863588	0	0
1/5	0,232733649	0,979371549	0	0
1/5	0,232733649	0,976892127	0	0
1/5	0,232733649	0,974425228	0	0
1/5	0,232733649	0,971970756	0	0
1/5	0,232733649	0,969528619	0	0
2/18	0,237255632	0,985889319	0	0
3/33	0,241257655	1	0	0
2/19	0,256736405	1	0	0
2/19	0,256736405	1	0	0
1/6	0,272334438	1	0	0
1/6	0,272334438	1	0	0
1/6	0,272334438	1	0	0
1/6	0,272334438	1	0	0
1/6	0,272334438	1	0	0
1/6	0,272334438	1	0	0
1/6	0,272334438	1	0	0
1/6	0,272334438	1	0	0
1/6	0,272334438	1	0	0
1/6	0,272334438	1	0	0
1/6	0,272334438	1	0	0
1/6	0,272334438	1	0	0
1/6	0,272334438	1	0	0
1/6	0,272334438	1	0	0
1/6	0,272334438	1	0	0
1/6	0,272334438	1	0	0
1/6	0,272334438	1	0	0
1/6	0,272334438	1	0	0
1/6	0,272334438	1	0	0
1/6	0,272334438	1	0	0
1/6	0,272334438	1	0	0

1/6	0,272334438	1	0	0
1/6	0,272334438	1	0	0
1/6	0,272334438	1	0	0
2/20	0,276239439	1	0	0
1/7	0,309893194	1	0	0
1/7	0,309893194	1	0	0
1/7	0,309893194	1	0	0
1/7	0,309893194	1	0	0
1/7	0,309893194	1	0	0
1/7	0,309893194	1	0	0
1/7	0,309893194	1	0	0
1/7	0,309893194	1	0	0
1/7	0,309893194	1	0	0
1/7	0,309893194	1	0	0
1/7	0,309893194	1	0	0
1/7	0,309893194	1	0	0
1/7	0,309893194	1	0	0
1/7	0,309893194	1	0	0
1/7	0,309893194	1	0	0
1/7	0,309893194	1	0	0
1/7	0,309893194	1	0	0
1/7	0,309893194	1	0	0
1/7	0,309893194	1	0	0
1/7	0,309893194	1	0	0
1/7	0,309893194	1	0	0
2/22	0,315101353	1	0	0
1/8	0,345515118	1	0	0
1/8	0,345515118	1	0	0
1/8	0,345515118	1	0	0
1/8	0,345515118	1	0	0
1/8	0,345515118	1	0	0
1/8	0,345515118	1	0	0
1/8	0,345515118	1	0	0
1/8	0,345515118	1	0	0
1/8	0,345515118	1	0	0
1/8	0,345515118	1	0	0
1/8	0,345515118	1	0	0
2/24	0,353472123	1	0	0
1/9	0,379299997	1	0	0
1/9	0,379299997	1	0	0
1/9	0,379299997	1	0	0
1/9	0,379299997	1	0	0
1/9	0,379299997	1	0	0
1/9	0,379299997	1	0	0
1/9	0,379299997	1	0	0
1/10	0,411342481	1	0	0
1/10	0,411342481	1	0	0
1/10	0,411342481	1	0	0
1/10	0,411342481	1	0	0
1/10	0,411342481	1	0	0
1/10	0,411342481	1	0	0
1/10	0,411342481	1	0	0
1/10	0,411342481	1	0	0

1/10	0,411342481	1	0	0
1/10	0,411342481	1	0	0
1/10	0,411342481	1	0	0
1/10	0,411342481	1	0	0
1/10	0,411342481	1	0	0
1/10	0,411342481	1	0	0
1/10	0,411342481	1	0	0
2/28	0,42762859	1	0	0
1/11	0,441732347	1	0	0
1/11	0,441732347	1	0	0
1/12	0,470554753	1	0	0
1/12	0,470554753	1	0	0
1/12	0,470554753	1	0	0
1/12	0,470554753	1	0	0
1/12	0,470554753	1	0	0
1/12	0,470554753	1	0	0
1/13	0,497890471	1	0	0
1/13	0,497890471	1	0	0
1/13	0,497890471	1	0	0
1/13	0,497890471	1	0	0
1/13	0,497890471	1	0	0
1/13	0,497890471	1	0	0
1/13	0,497890471	1	0	0
1/13	0,497890471	1	0	0
2/33	0,513571053	1	0	0
1/14	0,523816115	1	0	0
1/14	0,523816115	1	0	0
1/14	0,523816115	1	0	0
1/15	0,548404355	1	0	0
1/15	0,548404355	1	0	0
1/15	0,548404355	1	0	0
1/16	0,571724118	1	0	0
1/16	0,571724118	1	0	0
1/16	0,571724118	1	0	0
1/16	0,571724118	1	0	0
2/37	0,57590642	1	0	0
1/17	0,593840785	1	0	0
1/18	0,614816365	1	0	0
1/20	0,653576518	1	0	0
2/48	0,716381176	1	0	0
1/24	0,719797384	1	0	0
6/142	0,747195833	1	0	0
1/26	0,74800173	1	0	0
1/30	0,796185909	1	0	0
2/62	0,83664746	1	0	0
1/36	0,851763893	1	0	0
3/104	0,909462536	1	0	0
2/78	0,916272592	1	0	0

Odds.Ratio	Combined.Score
8,613264427	62,32070152
19,37984496	114,9109193
19,37984496	114,9109193
19,37984496	114,9109193
19,37984496	114,9109193
19,37984496	114,9109193
19,37984496	114,9109193
19,37984496	114,9109193
19,37984496	114,9109193
4,213009774	21,84523656
7,26744186	36,81572007
12,91989664	62,86468155
12,91989664	62,86468155
12,91989664	62,86468155
12,91989664	62,86468155
12,91989664	62,86468155
12,91989664	62,86468155
12,91989664	62,86468155
12,91989664	62,86468155
12,91989664	62,86468155
12,91989664	62,86468155
12,91989664	62,86468155
12,91989664	62,86468155
12,91989664	62,86468155
12,91989664	62,86468155
12,91989664	62,86468155
12,91989664	62,86468155
12,91989664	62,86468155
12,91989664	62,86468155
4,84496124	23,5210893
9,689922481	40,76899225
9,689922481	40,76899225
9,689922481	40,76899225
9,689922481	40,76899225
9,689922481	40,76899225
5,285412262	21,67743134
7,751937984	28,92370822
7,751937984	28,92370822
7,751937984	28,92370822
7,751937984	28,92370822
6,45994832	21,70645267
6,45994832	21,70645267
5,53709856	16,93240671
5,53709856	16,93240671
5,53709856	16,93240671
2,871088142	8,714854065
19,37984496	57,44646892
19,37984496	57,44646892
19,37984496	57,44646892
4,84496124	13,58747994
4,84496124	13,58747994
4,84496124	13,58747994

4,84496124	13,58747994
3,875968992	9,293806274
3,875968992	9,293806274
3,875968992	9,293806274
3,875968992	9,293806274
9,689922481	22,25972573
9,689922481	22,25972573
9,689922481	22,25972573
9,689922481	22,25972573
9,689922481	22,25972573
9,689922481	22,25972573
9,689922481	22,25972573
9,689922481	22,25972573
9,689922481	22,25972573
9,689922481	22,25972573
9,689922481	22,25972573
9,689922481	22,25972573
9,689922481	22,25972573
9,689922481	22,25972573
9,689922481	22,25972573
9,689922481	22,25972573
9,689922481	22,25972573
9,689922481	22,25972573
9,689922481	22,25972573
9,689922481	22,25972573
9,689922481	22,25972573
9,689922481	22,25972573
9,689922481	22,25972573
9,689922481	22,25972573
9,689922481	22,25972573
9,689922481	22,25972573
9,689922481	22,25972573
9,689922481	22,25972573
9,689922481	22,25972573
9,689922481	22,25972573
9,689922481	22,25972573
9,689922481	22,25972573
9,689922481	22,25972573
9,689922481	22,25972573
9,689922481	22,25972573
9,689922481	22,25972573
9,689922481	22,25972573
9,689922481	22,25972573
9,689922481	22,25972573
9,689922481	22,25972573
9,689922481	22,25972573
9,689922481	22,25972573
9,689922481	22,25972573
9,689922481	22,25972573
9,689922481	22,25972573
9,689922481	22,25972573
9,689922481	22,25972573
9,689922481	22,25972573

9,689922481	22,25972573
9,689922481	22,25972573
9,689922481	22,25972573
9,689922481	22,25972573
9,689922481	22,25972573
9,689922481	22,25972573
9,689922481	22,25972573
9,689922481	22,25972573
9,689922481	22,25972573
9,689922481	22,25972573
9,689922481	22,25972573
9,689922481	22,25972573
9,689922481	22,25972573
9,689922481	22,25972573
9,689922481	22,25972573
9,689922481	22,25972573
9,689922481	22,25972573
9,689922481	22,25972573
9,689922481	22,25972573
9,689922481	22,25972573
9,689922481	22,25972573
9,689922481	22,25972573
9,689922481	22,25972573
9,689922481	22,25972573
9,689922481	22,25972573
9,689922481	22,25972573
9,689922481	22,25972573
9,689922481	22,25972573
9,689922481	22,25972573
9,689922481	22,25972573
9,689922481	22,25972573
9,689922481	22,25972573
9,689922481	22,25972573
9,689922481	22,25972573
9,689922481	22,25972573
9,689922481	22,25972573
9,689922481	22,25972573
9,689922481	22,25972573
9,689922481	22,25972573
3,523608175	7,860423421
3,523608175	7,860423421
3,523608175	7,860423421
3,523608175	7,860423421
3,523608175	7,860423421
1,695736434	3,617326474
3,22997416	6,724672735
3,22997416	6,724672735
2,325581395	4,639006775
2,981514609	5,80867905
2,981514609	5,80867905
2,981514609	5,80867905
2,981514609	5,80867905
6,45994832	12,38772527

6,45994832	12,38772527
6,45994832	12,38772527
6,45994832	12,38772527
6,45994832	12,38772527
6,45994832	12,38772527
6,45994832	12,38772527
6,45994832	12,38772527
6,45994832	12,38772527
6,45994832	12,38772527
6,45994832	12,38772527
6,45994832	12,38772527
6,45994832	12,38772527
6,45994832	12,38772527
6,45994832	12,38772527
6,45994832	12,38772527
6,45994832	12,38772527
6,45994832	12,38772527
6,45994832	12,38772527
6,45994832	12,38772527
6,45994832	12,38772527
6,45994832	12,38772527
6,45994832	12,38772527
6,45994832	12,38772527
6,45994832	12,38772527
6,45994832	12,38772527
6,45994832	12,38772527
6,45994832	12,38772527
6,45994832	12,38772527
6,45994832	12,38772527
6,45994832	12,38772527
6,45994832	12,38772527
6,45994832	12,38772527
6,45994832	12,38772527
6,45994832	12,38772527
6,45994832	12,38772527
6,45994832	12,38772527
6,45994832	12,38772527
6,45994832	12,38772527
6,45994832	12,38772527
6,45994832	12,38772527
6,45994832	12,38772527
6,45994832	12,38772527
6,45994832	12,38772527
6,45994832	12,38772527
6,45994832	12,38772527
6,45994832	12,38772527
6,45994832	12,38772527
6,45994832	12,38772527
6,45994832	12,38772527
6,45994832	12,38772527
6,45994832	12,38772527
6,45994832	12,38772527
6,45994832	12,38772527

6,45994832	12,38772527
6,45994832	12,38772527
6,45994832	12,38772527
6,45994832	12,38772527
6,45994832	12,38772527
6,45994832	12,38772527
6,45994832	12,38772527
6,45994832	12,38772527
6,45994832	12,38772527
6,45994832	12,38772527
6,45994832	12,38772527
6,45994832	12,38772527
6,45994832	12,38772527
6,45994832	12,38772527
6,45994832	12,38772527
6,45994832	12,38772527
6,45994832	12,38772527
6,45994832	12,38772527
6,45994832	12,38772527
6,45994832	12,38772527
6,45994832	12,38772527
6,45994832	12,38772527
6,45994832	12,38772527
6,45994832	12,38772527
6,45994832	12,38772527
6,45994832	12,38772527
6,45994832	12,38772527
6,45994832	12,38772527
6,45994832	12,38772527
6,45994832	12,38772527
6,45994832	12,38772527
6,45994832	12,38772527
6,45994832	12,38772527
6,45994832	12,38772527
6,45994832	12,38772527
6,45994832	12,38772527
6,45994832	12,38772527
6,45994832	12,38772527
6,45994832	12,38772527
6,45994832	12,38772527
6,45994832	12,38772527
6,45994832	12,38772527
6,45994832	12,38772527
6,45994832	12,38772527
6,45994832	12,38772527
6,45994832	12,38772527
6,45994832	12,38772527
6,45994832	12,38772527
6,45994832	12,38772527
6,45994832	12,38772527
6,45994832	12,38772527
6,45994832	12,38772527
6,45994832	12,38772527

6,45994832	12,38772527
6,45994832	12,38772527
6,45994832	12,38772527
6,45994832	12,38772527
6,45994832	12,38772527
6,45994832	12,38772527
6,45994832	12,38772527
2,76854928	5,058804027
2,76854928	5,058804027
2,583979328	4,437032068
2,583979328	4,437032068
4,84496124	8,021254818
4,84496124	8,021254818
4,84496124	8,021254818
4,84496124	8,021254818
4,84496124	8,021254818
4,84496124	8,021254818
4,84496124	8,021254818
4,84496124	8,021254818
4,84496124	8,021254818
4,84496124	8,021254818
4,84496124	8,021254818
4,84496124	8,021254818
4,84496124	8,021254818
4,84496124	8,021254818
4,84496124	8,021254818
4,84496124	8,021254818
4,84496124	8,021254818
4,84496124	8,021254818
4,84496124	8,021254818
4,84496124	8,021254818
4,84496124	8,021254818
4,84496124	8,021254818
4,84496124	8,021254818
4,84496124	8,021254818
4,84496124	8,021254818
4,84496124	8,021254818
4,84496124	8,021254818
4,84496124	8,021254818
4,84496124	8,021254818
4,84496124	8,021254818
4,84496124	8,021254818
4,84496124	8,021254818
4,84496124	8,021254818
4,84496124	8,021254818
4,84496124	8,021254818
4,84496124	8,021254818
4,84496124	8,021254818
4,84496124	8,021254818
4,84496124	8,021254818
4,84496124	8,021254818
4,84496124	8,021254818
4,84496124	8,021254818

4,84496124	8,021254818
4,84496124	8,021254818
4,84496124	8,021254818
4,84496124	8,021254818
4,84496124	8,021254818
4,84496124	8,021254818
4,84496124	8,021254818
4,84496124	8,021254818
4,84496124	8,021254818
4,84496124	8,021254818
4,84496124	8,021254818
4,84496124	8,021254818
4,84496124	8,021254818
4,84496124	8,021254818
4,84496124	8,021254818
4,84496124	8,021254818
4,84496124	8,021254818
4,84496124	8,021254818
4,84496124	8,021254818
4,84496124	8,021254818
4,84496124	8,021254818
4,84496124	8,021254818
4,84496124	8,021254818
4,84496124	8,021254818
4,84496124	8,021254818
4,84496124	8,021254818
4,84496124	8,021254818
4,84496124	8,021254818
4,84496124	8,021254818
4,84496124	8,021254818
4,84496124	8,021254818
4,84496124	8,021254818
4,84496124	8,021254818
4,84496124	8,021254818
4,84496124	8,021254818
2,42248062	3,915755568
2,42248062	3,915755568
2,42248062	3,915755568
2,42248062	3,915755568
2,27998176	3,474499418
2,27998176	3,474499418
3,875968992	5,650622543
3,875968992	5,650622543
3,875968992	5,650622543
3,875968992	5,650622543
3,875968992	5,650622543
3,875968992	5,650622543
3,875968992	5,650622543
3,875968992	5,650622543
3,875968992	5,650622543
3,875968992	5,650622543
3,875968992	5,650622543
3,875968992	5,650622543

3,875968992	5,650622543
3,875968992	5,650622543
3,875968992	5,650622543
3,875968992	5,650622543
3,875968992	5,650622543
3,875968992	5,650622543
3,875968992	5,650622543
3,875968992	5,650622543
3,875968992	5,650622543
3,875968992	5,650622543
3,875968992	5,650622543
3,875968992	5,650622543
3,875968992	5,650622543
3,875968992	5,650622543
3,875968992	5,650622543
3,875968992	5,650622543
3,875968992	5,650622543
3,875968992	5,650622543
3,875968992	5,650622543
3,875968992	5,650622543
3,875968992	5,650622543
3,875968992	5,650622543
3,875968992	5,650622543
3,875968992	5,650622543
3,875968992	5,650622543
3,875968992	5,650622543
3,875968992	5,650622543
3,875968992	5,650622543
2,153316107	3,097797377
1,761804087	2,505091275
2,03998368	2,773776792
2,03998368	2,773776792
3,22997416	4,201306255
3,22997416	4,201306255
3,22997416	4,201306255
3,22997416	4,201306255
3,22997416	4,201306255
3,22997416	4,201306255
3,22997416	4,201306255
3,22997416	4,201306255
3,22997416	4,201306255
3,22997416	4,201306255
3,22997416	4,201306255
3,22997416	4,201306255
3,22997416	4,201306255
3,22997416	4,201306255
3,22997416	4,201306255
3,22997416	4,201306255
3,22997416	4,201306255
3,22997416	4,201306255
3,22997416	4,201306255
3,22997416	4,201306255
3,22997416	4,201306255

3,22997416	4,201306255
3,22997416	4,201306255
3,22997416	4,201306255
1,937984496	2,493192356
2,76854928	3,243431827
2,76854928	3,243431827
2,76854928	3,243431827
2,76854928	3,243431827
2,76854928	3,243431827
2,76854928	3,243431827
2,76854928	3,243431827
2,76854928	3,243431827
2,76854928	3,243431827
2,76854928	3,243431827
2,76854928	3,243431827
2,76854928	3,243431827
2,76854928	3,243431827
2,76854928	3,243431827
2,76854928	3,243431827
2,76854928	3,243431827
2,76854928	3,243431827
2,76854928	3,243431827
2,76854928	3,243431827
2,76854928	3,243431827
2,76854928	3,243431827
1,761804087	2,034638719
2,42248062	2,574415889
2,42248062	2,574415889
2,42248062	2,574415889
2,42248062	2,574415889
2,42248062	2,574415889
2,42248062	2,574415889
2,42248062	2,574415889
2,42248062	2,574415889
2,42248062	2,574415889
2,42248062	2,574415889
2,42248062	2,574415889
1,61498708	1,679506874
2,153316107	2,087484578
2,153316107	2,087484578
2,153316107	2,087484578
2,153316107	2,087484578
2,153316107	2,087484578
2,153316107	2,087484578
2,153316107	2,087484578
1,937984496	1,721568072
1,937984496	1,721568072
1,937984496	1,721568072
1,937984496	1,721568072
1,937984496	1,721568072
1,937984496	1,721568072
1,937984496	1,721568072
1,937984496	1,721568072

1,937984496	1,721568072
1,937984496	1,721568072
1,937984496	1,721568072
1,937984496	1,721568072
1,937984496	1,721568072
1,937984496	1,721568072
1,937984496	1,721568072
1,38427464	1,17594164
1,761804087	1,43948402
1,761804087	1,43948402
1,61498708	1,217446632
1,61498708	1,217446632
1,61498708	1,217446632
1,61498708	1,217446632
1,61498708	1,217446632
1,61498708	1,217446632
1,490757305	1,03961712
1,490757305	1,03961712
1,490757305	1,03961712
1,490757305	1,03961712
1,490757305	1,03961712
1,490757305	1,03961712
1,490757305	1,03961712
1,490757305	1,03961712
1,174536058	0,782671939
1,38427464	0,895092168
1,38427464	0,895092168
1,38427464	0,895092168
1,291989664	0,776152959
1,291989664	0,776152959
1,291989664	0,776152959
1,21124031	0,6772029
1,21124031	0,6772029
1,21124031	0,6772029
1,21124031	0,6772029
1,047559187	0,578053737
1,13999088	0,594099447
1,076658053	0,523720552
0,968992248	0,412108201
0,80749354	0,269333725
0,80749354	0,265492182
0,818866689	0,238640656
0,745378652	0,216420683
0,645994832	0,1472368
0,625156289	0,111498182
0,538329027	0,086372691
0,559033989	0,053053149
0,496919102	0,043451287

Genes
CAPZB;SH3KBP1;CAPZA1;AMOT
SMURF2;SMAD7
SP1;E2F1
ITCH;USP9X
RSF1;SMARCA1
GRIN2B;LRP8
CXADR;LNX2
SNX1;SNX6
MMP2;CLDN1
HNRNPK;C1QBP;PDCD4;HNRNPU;NAA38
KLF10;SMAD9;BACH1
APC;CTNNB1
SIN3A;MXD1
SIN3A;MXD1
CUL3;RBX1
GABPB2;E2F1
SERTAD2;E2F1
RBL1;E2F1
CREBBP;RXRA
CREBBP;CTNNB1
ELMO1;CRK
APC;CTNNB1
APC;CTNNB1
PHC2;BMI1
MAP2K1;RAF1
PPP2CA;RAF1
TNKS2;TNKS
CUL3;BMI1
CDC34;RBX1
HNRNPK;CDC37;G3BP1;NAA38
KAT2B;CREBBP
APC;CTNNB1
PPP2CA;RAF1
MAP2K1;RAF1
NCOA2;CREBBP
MED14;ESR1;MED17
CKS1B;RBX1
MAP2K1;RAF1
BTRC;RBX1
BTRC;RBX1
TNKS;TERF2IP
MAP2K1;MAPK1
NAA38;LSM5
NCOA2;CREBBP
XRCC5;TERF2IP
MGAM;PHF20;RBBP5;RUVBL1
SH3KBP1
MYOD1
EPOR
CKS1B;RBX1
RUVBL1;EPC1
MED14;MED17

CUL3;RBX1
RUVBL1;EPC1
MED14;MED17
GTF2H1;GTF2H5
MAP2K1;MAPK1
HDAC5
MAPK1
CREBBP
MXD1
CCND3
CCND3
CTNNB1
XRCC5
SH3KBP1
USP1
PTPN11
PTPN11
PTPN11
CREB1
PTPN11
PTPN11
CREB1
CREB1
CREB1
FNTB
DAG1
PIAS1
AMOT
CREBBP
CREBBP
RAB11A
MXD1
HOXC8
CTNNB1
CTNNB1
HNRNPU
ENTPD1
SMC1A
CTNNB1
CTNNB1
SNAP23
SNAP23
CKS1B
SNAP23
ELMO1
ELMO1
HIF1A
MYOD1
GAB1
S1PR1
SNAP23
SMARCA1
RSF1

S1PR3
S1PR1
APC
CRK
EPOR
LRP8
CDK1
CDK1
SUB1
CDK1
KIAA0101
KIAA0101
CREBBP
CXADR
CYTH3
MYO1C
CDK1
CDK1
PSD3
NRP2
NRP2
NRP2
ADRA2A
PLXNA2
PLXNA3
RUNX1
NECAB3
RAF1
RAF1
TERF2IP
E2F1
KAT2B
SP1
RAP1A
TGM2
SP1
CLDN1
ESR1
TEAD1
HNRNPU;HNRNPR
MED14;MED17
SIN3A;HIPK2
MAP2K1;MAPK1
XRCC5;TERF2IP
SF3B3;HNRNPK;HNRNPU;PPWD1;HNRNPR;SNRPD3;HNRNPC
RBBP5;N4BP2
MED14;MED17
KAT2B;CREBBP;GTF2H1
PHC2;BMI1
SF3B3;ATXN7
MED14;MED17
HNRNPR;CALM1
LYN

XRCC5
CRK
NOS1AP
XRCC5
APC
EPS8
CREBBP
BMPR1A
TRAK2
MXD1
RBX1
RBX1
RBX1
RBX1
BTRC
CDK1
XRCC5
HDAC5
RBX1
CREBBP
CREBBP
CTNNB1
SMURF2
XRCC5
CREBBP
HIPK2
EPOR
EPOR
EIF3J
CCND3
E2F1
RBL1
SH3KBP1
SH3KBP1
SH3KBP1
CALM1
MAP2K1
SIN3A
MRFAP1
MAPK1
SNAP23
SNAP23
SIN3A
CTNNB1
HIF1A
HIF1A
HIF1A
SNAP23
RBL1
XIAP
CTNNB1
ELMO1
SNRPD3

HNRNPU
KAT2B
VTI1A
CALM1
PTK2B
CDK1
CDK1
CDK1
BFAR
HSPH1
SNX1
PLXNA2
PLXNA3
ACTN4
E2F1
XRCC5
SH3KBP1
SMC1A
SEMA3A
PGAM5
CUL3
GABRA1
SIN3A
RUVBL1
CDC37
PHAX
ESR1
TSHZ3
PPP2CA
TNKS
RAF1
SNAP23
XRCC5
CUL3
SIN3A
CUL3
MAPK10
APC
ESR1
APPL1
CTGF
CD47
JAM2
CD47
CD47
CHAD
ADAM12
FOXO1
ESR1
ESR1
SP1
PARVA
FOXO1

CBX5
SMC1A
BACH1
RAD9A
BTRC
RBX1
MAML1
MED14;MED17
MED14;MED17
RUVBL1;EPC1
C1QBP;HNRNPU
KAT2B
RBX1
SMARCA1
CTNNB1
SWAP70
SMC1A
SMC1A
SIN3A
NCKAP1
APH1B
SIN3A
APH1B
CTNNB1
CTNNB1
SIN3A
CALM1
EPOR
C1QBP
MSL2
CTNNB1
CCND3
XRCC5
SIN3A
MAPK1
RBX1
XRCC5
SIN3A
SIN3A
SNRPD3
BMI1
BMI1
CDK1
CDK1
CDK1
RBX1
RBX1
ESR1
SNX1
SNX1
SNX1
SNX1
SNX1

CTNNB1
SNAP23
MAP2K1
PPP2CA
MAP2K1
RAF1
MAP2K1
RBX1
RBX1
RBX1
ING4
XRCC5
RBX1
RBX1
RBX1
RBX1
RBX1
RAD9A
RAD9A
RAD9A
CD47
PTK2B
TGM2
CD47
TGM2
ESR1
LYN
LYN
LYN
LYN
RAD9A
HIPK2
ING4
ING4
CD47
RUVBL1;EPC1
MED14;MED17
MED14;MED17
CPSF7;CAPRIN1
MED14;MED17
SF3B3;ATXN7
TBL1XR1
RBL1
NCKAP1
NCKAP1
ASB7
RBX1
CCND3
XRCC5
RUVBL1
DCP2
EIF3J
SNX1

XRCC5
SIN3A
RAF1
XRCC5
CDC37
TBL1XR1
CTNNB1
DAG1
CTNNB1
CDC37
VTI1A
SIX1
DEK
AP3M2
GTF2H1
PTPN11
SNX1
RBX1
CDC73
RUVBL1
PHAX
PPP2CA
RBX1
TRA2B
SEPT11
NAPB
RBX1
BTRC
MED14;MED17
SF3B3;SNRPD3;SMNDC1
HNRNPC;GATAD2B
SP1;SIN3A
TBL1XR1
TERF2IP
XRCC5
GRIN2B
HIF1A
CDK1
SIN3A
DAG1
PTPN11
IKZF3
SIN3A
XRCC5
CDC37
SNRPD3
GTF2H1
RBX1
C1QBP
RBL1
CREBBP
ESR1
RBBP5

RBBP5
BACH1
CD47
HNRNPU;TARDBP
SIN3A
AP3M2
APC
MED14
SNRPD3
LSM5
CREBBP
SIN3A
SIN3A
NXF1
HNRNPC
RBL1
SUB1
RUVBL1
XRCC5
RBBP5
RBBP5
RBBP5
SIN3A
SNRPD3
ING4
RBL1;TBL1XR1
RAD9A
GATAD2B
XRCC5
SIN3A
CDC37
CDC37
SNRPD3
AMOT
SF3B3
SNRPD3
BACH1
SF3B3;SNRPD3
GATAD2B
SIN3A
RUVBL1
SIN3A
RBL1
RAF1
NCKAP1
CARM1
RPP14
RUVBL1
KAT2B
UQCR10
GATAD2B
USP2
RBBP5

CDC37
DCP2
MED17
MAP2K1
SF3B3
TBL1XR1
CREBBP
RBBP5;SIN3A
CTNNB1
SIN3A
IKZF3
BTRC
ACTN4
SIN3A
MAP2K1
SIN3A
RUVBL1
COX7B
RBX1
RBX1
CDK1
EIF3J
MAML1
GATAD2B
MED14;MED17
SIN3A
RBX1
CDC37
AP3M2
SIN3A
SNRPD3
SMC1A
CDC37
SIN3A
SNRPD3
RSF1;BMI1
LCOR
SIN3A
PSMD11
MRPL42;MRPL27
GTF2H1
TCERG1;SF3B3;TRA2B;PPWD1;SNRPD3;SMNDC1
GTF2H1
SNRPD3
RBM28;GTPBP4
PSMD11
RBM28;IGF2BP1;HNRNPU
MRPL42;MRPL27
