# Supplementary material for: Circulating miR-330-3p in Late Pregnancy is Associated with Pregnancy Outcomes Among Lean Women with GDM
Source: Sci Rep. 2020 Jan 22;10:908. doi: 10.1038/s41598-020-57838-6 (PMC6976655; doi:10.1038/s41598-020-57838-6)
Supplement: Supplementary file 1 — Supplementary data. [file 41598_2020_57838_MOESM1_ESM.zip › Supplimentary File_EnrichR_Analysis ENCODE_Histone_Modifications.pdf]

Term	Overlap	P.value
H3K79me2_heart_mm9	168/2000	8,45551E-11
H3K79me2_liver_mm9	161/2000	4,99403E-09
H3K27me3_ES-Bruce4_mm9	145/2000	1,32818E-05
H3K27me3_small intestine_mm9	141/2000	6,90562E-05
H3ac_myocyte_mm9	138/2000	0,00021794
H3K27me3_MCF-7_hg19	75/989	0,000522637
H3K27me3_kidney_mm9	116/1675	0,000627151
H3K27me3_thymus_mm9	133/2000	0,001251038
H3K27me3_bronchial epithelial cell_hg19	136/2082	0,002162405
H3K4me3_H7_hg19	98/1452	0,003595088
H3K4me3_B cell_hg19	176/2843	0,004896319
H3K27me3_spleen_mm9	128/2000	0,005807156
H3K4me3_epithelial cell of proximal tubule_hg19	108/1655	0,006346519
H3K27me3_cerebellum_mm9	98/1486	0,006890928
H3K27me3_erythroblast_mm9	167/2712	0,007511074
H3K27ac_limb_mm9	127/2000	0,007693969
H3K4me3_GM12866_hg19	127/2000	0,007693969
H3K27me3_brain_mm9	127/2000	0,007693969
H3K4me3_fibroblast of pedal digit skin_hg19	129/2043	0,008655591
H3K9me3_G1E-ER4_mm9	170/2782	0,009300997
H3K27ac_brain_mm9	126/2000	0,010106674
H3K4me3_HEK293_hg19	132/2116	0,011599381
H2AFZ_A549_hg19	186/3109	0,014653488
H3K27me3_testis_mm9	124/2000	0,016995215
H3K4me3_fibroblast of foreskin _hg19	96/1505	0,017382136
H3K4me3_fibroblast of pulmonary artery_hg19	129/2093	0,017847551
H4K20me1_A549_hg19	122/2000	0,027612894
H3K4me3_BJ_hg19	115/1883	0,031021693
H3K4me3_cardiac fibroblast_hg19	171/2902	0,031376252
H3K4me3_brain microvascular endothelial cell_hg19	127/2101	0,031674218
H3K4me3_SK-N-SH_hg19	121/2000	0,034746365
H3K27ac_cortical plate_mm9	121/2000	0,034746365
H3K27me3_Caco-2_hg19	28/380	0,037497008
H3K27ac_thymus_mm9	120/2000	0,043349379
H3K4me3_skeletal muscle cell_hg19	106/1754	0,047412546
H3K27me3_BJ_hg19	124/2090	0,052964224
H3K27ac_CH12.LX_mm9	119/2000	0,053621588
H3K4me3_astrocyte of the spinal cord_hg19	117/1965	0,054487723
H3K4me3_retinal pigment epithelial cell_hg19	120/2021	0,055350639
H3K4me3_fibroblast of mammary gland_hg19	119/2005	0,056758737
H3K79me2_CH12.LX_mm9	118/2000	0,065764172
H3K4me3_fibroblast of the aortic adventitia_hg19	98/1648	0,075755267
H3K4me3_MCF-7_hg19	111/1890	0,079921027
H3K27ac_MCF-7_hg19	117/2000	0,079973136
H3K9ac_NT2-D1_hg19	117/2000	0,079973136
H3K9ac_H1-hESC_hg19	117/2000	0,079973136
H3K4me3_foreskin fibroblast_hg19	187/3309	0,088739057
H3K4me3_Jurkat_hg19	120/2071	0,093909574
H3K27me3_liver_mm9	116/2000	0,096431518
H3K4me3_kidney epithelial cell_hg19	112/1936	0,106116012
H3K27me3_GM12878_hg19	181/3221	0,107722882
H3K27ac_cerebellum_mm9	115/2000	0,11530075

H3K9ac_ES-E14_mm9	115/2000	0,11530075
H3K4me3_thymus_mm9	115/2000	0,11530075
H3K4me3_cardiac mesoderm_hg19	207/3727	0,122611649
H3K9ac_K562_hg19	190/3409	0,124432915
H3K4me3_HL-60_hg19	108/1881	0,127210347
H3K4me3_fibroblast of upper leg skin_hg19	113/1975	0,128939902
H3K4me3_fibroblast of skin of abdomen_hg19	118/2074	0,136380018
H3K9me3_CD14-positive monocyte_hg19	114/2000	0,136711508
H3K27ac_heart_mm9	164/2941	0,14479946
H3K27ac_A549_hg19	166/2991	0,158467021
H3K27me3_heart_mm9	113/2000	0,160754492
H2AFZ_fibroblast of lung_hg19	113/2000	0,160754492
H3K27ac_ES-E14_mm9	113/2000	0,160754492
H3K27ac_spleen_mm9	113/2000	0,160754492
H3K27me3_H7_hg19	113/2015	0,181979678
H3K27me3_SK-N-SH_hg19	120/2149	0,186326435
H2AFZ_T-cell acute lymphoblastic leukemia_hg19	112/2000	0,187471663
H3K4me3_cerebellum_mm9	112/2000	0,187471663
H3K4me3_fibroblast of villous mesenchyme_hg19	111/1982	0,188535695
H3K4me3_HepG2_hg19	196/3586	0,191126328
H3K4me3_HCT116_hg19	114/2040	0,191326169
H3K4me3_keratinocyte_hg19	183/3344	0,196371859
H3K4me3_WERI-Rb-1_hg19	90/1606	0,216012789
H3K4me3_kidney_mm9	111/2000	0,216848491
H2AFZ_astrocyte_hg19	111/2000	0,216848491
H3K9ac_liver_mm9	111/2000	0,216848491
H2AFZ_fibroblast of dermis_hg19	111/2000	0,216848491
H3K4me3_LNCaP clone FGC_hg19	89/1591	0,222751806
H3K9me3_mononuclear cell_hg19	13/198	0,223771993
H3K4me3_fibroblast of gingiva_hg19	114/2061	0,224393692
H3K27ac_H1-hESC_hg19	110/2000	0,248807824
H2AFZ_endothelial cell of umbilical vein_hg19	110/2000	0,248807824
H3K27ac_HeLa-S3_hg19	110/2000	0,248807824
H3K4me3_Caco-2_hg19	111/2029	0,266889358
H3K4me3_choroid plexus epithelial cell_hg19	113/2069	0,270827324
H3K4me3_GM12864_hg19	71/1279	0,274427923
H3K4me3_epithelial cell of esophagus_hg19	114/2093	0,280050682
H3K27ac_kidney_mm9	109/2000	0,28320591
H3K4me1_HCT116_hg19	109/2000	0,28320591
H3K4me3_GM12875_hg19	109/2000	0,28320591
H3K9ac_fibroblast of lung_hg19	109/2000	0,28320591
H3K27me3_cardiac mesoderm_hg19	187/3485	0,28502537
H3K4me3_fibroblast of lung_hg19	275/5182	0,300683665
H3K27me3_CD14-positive monocyte_hg19	184/3445	0,311346593
H4K20me1_K562_hg19	108/2000	0,319831055
H3K27me3_T-cell acute lymphoblastic leukemia_hg19	108/2000	0,319831055
H4K20me1_fibroblast of lung_hg19	108/2000	0,319831055
H3K4me3_H1-hESC_hg19	108/2000	0,319831055
H3K27ac_skeletal muscle myoblast_hg19	108/2000	0,319831055
H3K4me3_GM12865_hg19	79/1451	0,322885385
H3K4me3_erythroblast_mm9	143/2674	0,332483503
H3K4me3_mammary epithelial cell_hg19	189/3567	0,35276452
H3K79me2_MEL cell line_mm9	107/2000	0,358405258

H3K27ac_small intestine_mm9	107/2000	0,358405258
H3K4me3_lung_mm9	107/2000	0,358405258
H3K27ac_olfactory bulb_mm9	107/2000	0,358405258
H3K4me3_endothelial cell of umbilical vein_hg19	185/3497	0,363762371
H3K27ac_liver_mm9	163/3076	0,365840536
H3K4me3_CD14-positive monocyte_hg19	192/3639	0,376024787
H3K4me3_bronchial epithelial cell_hg19	99/1859	0,38368825
H3K27me3_C2C12_mm9	68/1269	0,389479617
H3K4me3_placenta_mm9	106/2000	0,398589016
H3K27me3_skeletal muscle myoblast_hg19	106/2000	0,398589016
H4K20me1_endothelial cell of umbilical vein_hg19	106/2000	0,398589016
H3K27ac_HCT116_hg19	106/2000	0,398589016
H3K9ac_CD14-positive monocyte_hg19	106/2000	0,398589016
H3K4me3_cardiac muscle cell_hg19	110/2087	0,420258864
H3K9me3_GM12878_hg19	105/2000	0,439989249
H3K9ac_ES-Bruce4_mm9	105/2000	0,439989249
H3K9me3_brain_mm9	28/522	0,443818292
H3K9me3_G1E_mm9	160/3067	0,452508038
H3K4me2_A549_hg19	142/2720	0,453399689
H3K4me3_GM06990_hg19	97/1852	0,453594484
H3K27me3_splenic B cell_mm9	21/391	0,457008175
H3K4me3_olfactory bulb_mm9	104/2000	0,482170129
H3K27ac_embryonic fibroblast_mm9	104/2000	0,482170129
H3K9ac_heart_mm9	104/2000	0,482170129
H3K4me3_Panc1_hg19	170/3295	0,51449332
H3K27me3_myocyte_mm9	61/1183	0,522213087
H3K9ac_myotube_hg19	103/2000	0,524666354
H2AFZ_HeLa-S3_hg19	103/2000	0,524666354
H4K20me1_mammary epithelial cell_hg19	103/2000	0,524666354
H3K27ac_K562_hg19	103/2000	0,524666354
H3K4me3_K562_hg19	237/4606	0,532954495
H3K4me3_A549_hg19	200/3895	0,544718944
H3K27me3_endothelial cell of umbilical vein_hg19	174/3393	0,550197929
H3K79me3_C2C12_mm9	102/2000	0,566998216
H3K4me3_small intestine_mm9	102/2000	0,566998216
H2AFZ_keratinocyte_hg19	102/2000	0,566998216
H3K27me3_A549_hg19	147/2891	0,592531377
H3K27me3_megakaryocyte_mm9	134/2638	0,593959768
H2AFZ_CD14-positive monocyte_hg19	101/2000	0,608687688
H3K9ac_endothelial cell of umbilical vein_hg19	101/2000	0,608687688
H3K4me3_brown adipose tissue_mm9	101/2000	0,608687688
H3K4me3_bone marrow macrophage_mm9	101/2000	0,608687688
H3K4me3_HeLa-S3_hg19	182/3589	0,61802194
H3K4me3_astrocyte of the cerebellum_hg19	80/1594	0,622185598
H3K4me2_GM12878_hg19	100/2000	0,649274627
H3K27ac_ES-Bruce4_mm9	100/2000	0,649274627
H3K9ac_mammary epithelial cell_hg19	100/2000	0,649274627
H3K4me1_mammary epithelial cell_hg19	100/2000	0,649274627
H2AFZ_H1-hESC_hg19	100/2000	0,649274627
H4K20me1_GM12878_hg19	100/2000	0,649274627
H3K27me3_H1-hESC_hg19	100/2000	0,649274627
H3K27me3_kidney epithelial cell_hg19	111/2217	0,650517571
H2AFZ_myotube_hg19	99/2000	0,688332188

H3K27ac_MEL cell line_mm9	99/2000	0,688332188
H3K9me3_T-cell acute lymphoblastic leukemia_hg19	99/2000	0,688332188
H3K27ac_placenta_mm9	99/2000	0,688332188
H3K27ac_bone marrow macrophage_mm9	99/2000	0,688332188
H3K27me3_GM06990_hg19	8/179	0,7107409
H3K79me2_C2C12_mm9	98/2000	0,725480583
H3K4me3_spleen_mm9	98/2000	0,725480583
H3ac_C2C12_mm9	98/2000	0,725480583
H4K20me1_keratinocyte_hg19	98/2000	0,725480583
H3K9ac_GM12878_hg19	98/2000	0,725480583
H2AFZ_osteoblast_hg19	98/2000	0,725480583
H3K9ac_HeLa-S3_hg19	98/2000	0,725480583
H3K4me3_WI38_hg19	124/2521	0,734845704
H3K27me3_mammary epithelial cell_hg19	127/2586	0,743736078
H3K4me3_G1E_mm9	124/2531	0,750730668
H3K4me1_MEL cell line_mm9	207/4170	0,75131584
H3K27me3_fibroblast of lung_hg19	150/3052	0,759590931
H3K4me3_NT2-D1_hg19	97/2000	0,760398424
H3K4me3_myotube_hg19	97/2000	0,760398424
H3K27ac_brown adipose tissue_mm9	97/2000	0,760398424
H3K4me1_megakaryocyte_mm9	122/2505	0,771684513
H3K4me3_BE2C_hg19	76/1594	0,785623584
H3K4me3_SK-N-MC_hg19	67/1414	0,788239131
H3K27me3_HepG2_hg19	96/2000	0,79283108
H3K27ac_GM12878_hg19	96/2000	0,79283108
H3K4me3_myocyte_mm9	96/2000	0,79283108
H3K4me2_osteoblast_hg19	96/2000	0,79283108
H4K20me1_skeletal muscle myoblast_hg19	96/2000	0,79283108
H3K4me3_mononuclear cell_hg19	96/2000	0,79283108
H3K27me3_astrocyte_hg19	96/2000	0,79283108
H2AFZ_HepG2_hg19	96/2000	0,79283108
H3K27ac_fibroblast of lung_hg19	177/3617	0,799419818
H3K4me3_GM12878_hg19	178/3640	0,803211616
H3K4me3_limb_mm9	95/2000	0,822595669
H2AFZ_mammary epithelial cell_hg19	95/2000	0,822595669
H2AFZ_B cell_hg19	95/2000	0,822595669
H3K36me3_T-cell acute lymphoblastic leukemia_hg19	95/2000	0,822595669
H3K4me1_thymus_mm9	95/2000	0,822595669
H2AFZ_GM12878_hg19	95/2000	0,822595669
H3K9me3_CH12.LX_mm9	129/2689	0,831461201
H3K9me3_skeletal muscle myoblast_hg19	94/2000	0,84958257
H3K9ac_keratinocyte_hg19	94/2000	0,84958257
H3K36me3_heart_mm9	94/2000	0,84958257
H3K9ac_A549_hg19	94/2000	0,84958257
H3K4me3_testis_mm9	94/2000	0,84958257
H3K4me3_liver_mm9	137/2880	0,865342556
H3K4me3_embryonic fibroblast_mm9	93/2000	0,873753555
H3K4me2_keratinocyte_hg19	93/2000	0,873753555
H3K27ac_endothelial cell of umbilical vein_hg19	93/2000	0,873753555
H3K4me3_bone marrow_mm9	93/2000	0,873753555
H3K27ac_mammary epithelial cell_hg19	93/2000	0,873753555
H4K20me1_fibroblast of dermis_hg19	93/2000	0,873753555
H3K27ac_bone marrow_mm9	93/2000	0,873753555

H3K9ac_skeletal muscle myoblast_hg19	93/2000	0,873753555
H3K4me1_erythroblast_mm9	124/2632	0,878613092
H2AFZ_K562_hg19	92/2000	0,895136886
H3K27ac_astrocyte_hg19	92/2000	0,895136886
H3K4me3_CH12.LX_mm9	198/4143	0,901241748
H3K9me3_ES-E14_mm9	5/155	0,90708417
H3K4me3_G1E-ER4_mm9	121/2605	0,908271036
H3K9me3_K562_hg19	91/2000	0,913819879
H3K4me2_HepG2_hg19	91/2000	0,913819879
H3K4me1_T-cell acute lymphoblastic leukemia_hg19	91/2000	0,913819879
H3K4me3_cortical plate_mm9	91/2000	0,913819879
H3K4me3_brain_mm9	91/2000	0,913819879
H3K4me3_ES-E14_mm9	149/3190	0,921480448
H3K4me1_A549_hg19	137/2951	0,923898677
H3K27ac_testis_mm9	90/2000	0,929939577
H3K4me1_spleen_mm9	90/2000	0,929939577
H3K4me1_testis_mm9	90/2000	0,929939577
H4K20me1_astrocyte_hg19	90/2000	0,929939577
H4K20me1_H1-hESC_hg19	90/2000	0,929939577
H3K79me2_myocyte_mm9	90/2000	0,929939577
H3K9me3_MCF-7_hg19	6/190	0,931235676
H3K9ac_fibroblast of dermis_hg19	89/2000	0,943672242
H4K20me1_HepG2_hg19	89/2000	0,943672242
H3K9ac_astrocyte_hg19	89/2000	0,943672242
H3K27me3_HeLa-S3_hg19	89/2000	0,943672242
H3K4me2_mammary epithelial cell_hg19	89/2000	0,943672242
H3K4me3_fibroblast of dermis_hg19	89/2000	0,943672242
H3K4me3_astrocyte_hg19	89/2000	0,943672242
H3K27me3_keratinocyte_hg19	99/2221	0,951527675
H3K9ac_HepG2_hg19	88/2000	0,955222384
H3K4me3_C2C12_mm9	88/2000	0,955222384
H3K4me1_endothelial cell of umbilical vein_hg19	88/2000	0,955222384
H3K4me2_K562_hg19	88/2000	0,955222384
H3K9ac_CH12.LX_mm9	88/2000	0,955222384
H3K36me3_myocyte_mm9	7/236	0,963003056
H3K27ac_HepG2_hg19	87/2000	0,964811974
H3K4me1_keratinocyte_hg19	87/2000	0,964811974
H3K36me3_testis_mm9	87/2000	0,964811974
H3K4me2_skeletal muscle myoblast_hg19	87/2000	0,964811974
H3K4me2_B cell_hg19	87/2000	0,964811974
H3K4me1_HepG2_hg19	87/2000	0,964811974
H3K4me1_HeLa-S3_hg19	87/2000	0,964811974
H3K4me3_NB4_hg19	87/2000	0,964811974
H3K4me2_fibroblast of dermis_hg19	87/2000	0,964811974
H3K4me3_megakaryocyte_mm9	108/2441	0,966297791
H3K4me3_MEL cell line_mm9	188/4088	0,969687513
H3K27ac_Panc1_hg19	86/2000	0,972670436
H3K4me1_fibroblast of dermis_hg19	86/2000	0,972670436
H3K4me3_osteoblast_hg19	85/2000	0,979025839
H3K27ac_B cell_hg19	85/2000	0,979025839
H3K4me2_H1-hESC_hg19	85/2000	0,979025839
H3K9ac_T-cell acute lymphoblastic leukemia_hg19	85/2000	0,979025839
H3K4me3_skeletal muscle myoblast_hg19	85/2000	0,979025839

H3K27ac_fibroblast of dermis_hg19	85/2000	0,979025839
H3K9me3_fibroblast of dermis_hg19	85/2000	0,979025839
H3K27me3_fibroblast of dermis_hg19	85/2000	0,979025839
H3K27me3_NT2-D1_hg19	85/2000	0,979025839
H3K27me3_mononuclear cell_hg19	85/2000	0,979025839
H3K27ac_myotube_hg19	85/2000	0,979025839
H3K4me1_small intestine_mm9	84/2000	0,984097596
H3K4me2_endothelial cell of umbilical vein_hg19	84/2000	0,984097596
H4K20me1_CD14-positive monocyte_hg19	84/2000	0,984097596
H3K4me1_Panc1_hg19	84/2000	0,984097596
H3K27ac_keratinocyte_hg19	84/2000	0,984097596
H3K4me1_CD14-positive monocyte_hg19	83/2000	0,988090825
H3K4me1_olfactory bulb_mm9	83/2000	0,988090825
H3K4me3_ES-Bruce4_mm9	83/2000	0,988090825
H3K4me2_fibroblast of lung_hg19	83/2000	0,988090825
H3K4me3_heart_mm9	138/3158	0,988386076
H3K36me3_C2C12_mm9	12/407	0,98937942
H3K9ac_MEL cell line_mm9	82/2000	0,991192379
H3K36me3_fibroblast of dermis_hg19	82/2000	0,991192379
H3K9me1_K562_hg19	82/2000	0,991192379
H3K9me3_myotube_hg19	82/2000	0,991192379
H2AFZ_skeletal muscle myoblast_hg19	81/2000	0,993568462
H3K4me2_CH12.LX_mm9	81/2000	0,993568462
H4K20me1_T-cell acute lymphoblastic leukemia_hg19	81/2000	0,993568462
H3K27ac_T-cell acute lymphoblastic leukemia_hg19	81/2000	0,993568462
H3K4me1_astrocyte_hg19	81/2000	0,993568462
H3K4me1_brown adipose tissue_mm9	80/2000	0,995363635
H3K36me3_astrocyte_hg19	80/2000	0,995363635
H3K4me1_mononuclear cell_hg19	80/2000	0,995363635
H3K27me3_osteoblast_hg19	80/2000	0,995363635
H3K27ac_CD14-positive monocyte_hg19	80/2000	0,995363635
H3K36me3_osteoblast_hg19	79/2000	0,996700997
H3K4me1_placenta_mm9	79/2000	0,996700997
H3K36me3_erythroblast_mm9	128/3059	0,997089257
H3K9me3_A549_hg19	78/2000	0,997683245
H3K4me2_myotube_hg19	78/2000	0,997683245
H3K27ac_osteoblast_hg19	78/2000	0,997683245
H3K4me1_lung_mm9	78/2000	0,997683245
H3K9me1_keratinocyte_hg19	78/2000	0,997683245
H3K4me1_brain_mm9	78/2000	0,997683245
H3K4me1_G1E_mm9	101/2521	0,998326287
H3K4me2_T-cell acute lymphoblastic leukemia_hg19	77/2000	0,998394385
H3K4me1_H1-hESC_hg19	77/2000	0,998394385
H3K4me1_osteoblast_hg19	77/2000	0,998394385
H3K4me2_CD14-positive monocyte_hg19	77/2000	0,998394385
H3K4me1_GM12878_hg19	77/2000	0,998394385
H3K9me3_NT2-D1_hg19	58/1583	0,998590366
H3K4me2_HeLa-S3_hg19	76/2000	0,998901818
H3K4me2_astrocyte_hg19	76/2000	0,998901818
H3K9me3_astrocyte_hg19	76/2000	0,998901818
H3K36me3_K562_hg19	104/2634	0,999211051
H3K36me3_ES-Bruce4_mm9	75/2000	0,999258613
H3K79me2_K562_hg19	75/2000	0,999258613

H4K20me1_B cell_hg19	75/2000	0,999258613
H3K4me3_T-cell acute lymphoblastic leukemia_hg19	75/2000	0,999258613
H3K4me1_embryonic fibroblast_mm9	75/2000	0,999258613
H4K20me1_osteoblast_hg19	75/2000	0,999258613
H3K36me3_G1E-ER4_mm9	116/2903	0,999320025
H3K4me1_K562_hg19	152/3684	0,9994383
H3K27me3_G1E_mm9	100/2569	0,999448741
H4K20me1_myotube_hg19	74/2000	0,999505788
H3K4me2_myocyte_mm9	74/2000	0,999505788
H3K36me3_myotube_hg19	74/2000	0,999505788
H3K36me3_MEL cell line_mm9	135/3352	0,999637378
H3K9me1_endothelial cell of umbilical vein_hg19	73/2000	0,999674465
H3K36me3_CD14-positive monocyte_hg19	73/2000	0,999674465
H3K4me1_G1E-ER4_mm9	91/2415	0,999739657
H3K36me3_Caco-2_hg19	7/382	0,9997775
H3K4me1_bone marrow_mm9	72/2000	0,999787833
H3K4me1_CH12.LX_mm9	158/3885	0,999809005
H3K36me3_G1E_mm9	117/3013	0,999834011
H3K36me3_kidney epithelial cell_hg19	2/215	0,999860424
H3K4me1_myotube_hg19	71/2000	0,999862864
H3K36me3_HepG2_hg19	88/2389	0,99987393
H3K36me3_A549_hg19	120/3112	0,999903645
H3K4me1_skeletal muscle myoblast_hg19	70/2000	0,999911752
H3K4me1_kidney_mm9	70/2000	0,999911752
H3K36me3_U2OS_hg19	70/2000	0,999911752
H3K9me3_U2OS_hg19	31/1078	0,99993199
H3K9me3_ES-Bruce4_mm9	12/570	0,999938601
H3K79me2_HeLa-S3_hg19	69/2000	0,999943108
H3K27me3_CH12.LX_mm9	96/2618	0,999946968
H3K36me3_keratinocyte_hg19	97/2651	0,999955468
H3K27me3_K562_hg19	127/3318	0,999957726
H3K79me2_CD14-positive monocyte_hg19	68/2000	0,999962899
H3K36me3_HeLa-S3_hg19	78/2234	0,999964628
H3K4me1_cerebellum_mm9	67/2000	0,99997519
H3K36me3_liver_mm9	67/2000	0,99997519
H3K36me3_megakaryocyte_mm9	112/3028	0,999976205
H3K36me3_splenic B cell_mm9	66/2000	0,999982698
H3K9me3_osteoblast_hg19	66/2000	0,999982698
H3K27me3_MEL cell line_mm9	94/2672	0,999986677
H3K9me3_erythroblast_mm9	84/2445	0,999987067
H3K36me3_mammary epithelial cell_hg19	65/2000	0,99998721
H3K36me3_fibroblast of lung_hg19	65/2000	0,99998721
H3K9me3_HepG2_hg19	57/1809	0,999987389
H3K36me3_thymus_mm9	33/1235	0,999990958
H3K36me3_kidney_mm9	31/1187	0,999991407
H3K36me3_spleen_mm9	63/2000	0,999991423
H3K9me3_keratinocyte_hg19	63/2000	0,999991423
H3K4me1_cortical plate_mm9	63/2000	0,999991423
H3K9me3_fibroblast of lung_hg19	76/2323	0,999991747
H3K9me3_megakaryocyte_mm9	102/2939	0,999991768
H3K4me1_heart_mm9	131/3628	0,999991999
H3K4me1_ES-E14_mm9	79/2411	0,999992161
H3K36me3_cardiac mesoderm_hg19	83/2514	0,999992277

H3K9me3_mammary epithelial cell_hg19	62/2000	0,999992306
H3K36me3_ES-E14_mm9	62/2000	0,999992306
H3K4me1_NT2-D1_hg19	62/2000	0,999992306
H3K36me3_NT2-D1_hg19	62/2000	0,999992306
H3K36me3_skeletal muscle myoblast_hg19	62/2000	0,999992306
H3K79me2_endothelial cell of umbilical vein_hg19	62/2000	0,999992306
H3K36me3_CH12.LX_mm9	142/4031	0,999992311
H3K4me1_liver_mm9	117/3706	0,999992447
H3K36me3_endothelial cell of umbilical vein_hg19	96/2929	0,999992742
H3K79me2_keratinocyte_hg19	61/2000	0,999992802
H3K4me1_fibroblast of lung_hg19	61/2000	0,999992802
H3K79me2_A549_hg19	86/2787	0,999992866
H3K27me3_G1E-ER4_mm9	78/2653	0,999992945
H3K36me3_GM12878_hg19	77/2531	0,999993004
H3K9me3_endothelial cell of umbilical vein_hg19	60/2000	0,999993075
H3K36me3_H7_hg19	54/1851	0,999993188
H3K4me1_limb_mm9	59/2000	0,999993222
H3K4me1_bone marrow macrophage_mm9	59/2000	0,999993222
H3K9me3_H1-hESC_hg19	59/2000	0,999993222
H3K79me2_skeletal muscle myoblast_hg19	59/2000	0,999993222
H3K79me2_GM12878_hg19	58/2000	0,999993301
H3K79me2_fibroblast of dermis_hg19	58/2000	0,999993301
H4K20me1_HeLa-S3_hg19	58/2000	0,999993301
H3K79me2_T-cell acute lymphoblastic leukemia_hg19	58/2000	0,999993301
H3K27me3_myotube_hg19	58/2000	0,999993301
H3K36me3_MCF-7_hg19	37/1395	0,999993324
H3K79me2_myotube_hg19	56/2000	0,999993362
H3K36me3_small intestine_mm9	56/2000	0,999993362
H3K79me2_mammary epithelial cell_hg19	54/2000	0,999993378
H3K9me3_HeLa-S3_hg19	54/2000	0,999993378
H3K79me2_osteoblast_hg19	54/2000	0,999993378
H3K36me3_H1-hESC_hg19	54/2000	0,999993378
H3K79me2_H1-hESC_hg19	54/2000	0,999993378
H3K79me2_astrocyte_hg19	51/2000	0,999993382
H3K36me3_brain_mm9	49/2000	0,999993383
H3K4me1_ES-Bruce4_mm9	49/2000	0,999993383
H3K79me2_HepG2_hg19	48/2000	0,999993383
H3K79me2_fibroblast of lung_hg19	45/2000	0,999993383
H3K36me3_bronchial epithelial cell_hg19	37/1681	0,999993653
H3K36me3_BJ_hg19	26/1302	0,999994048
H3K36me3_GM06990_hg19	22/1255	0,999994105
H3K36me3_SK-N-SH_hg19	9/707	0,999994963

Adjusted.P.value	Old.P.value	Old.Adjustec	Odds.Ratio
3,48367E-08	0	0	1,627906977
1,02877E-06	0	0	1,560077519
0,001824028	0	0	1,40503876
0,007112788	0	0	1,36627907
0,017958248	0	0	1,337209302
0,035887747	0	0	1,469654572
0,036912338	0	0	1,342126576
0,064428434	0	0	1,28875969
0,098990083	0	0	1,265926472
0,148117635	0	0	1,308006065
0,183389404	0	0	1,199737148
0,199379036	0	0	1,240310078
0,201135822	0	0	1,26466662
0,202790181	0	0	1,278078604
0,20630416	0	0	1,193375409
0,198119693	0	0	1,230620155
0,186465594	0	0	1,230620155
0,176106394	0	0	1,230620155
0,187689659	0	0	1,223690651
0,191600533	0	0	1,184246457
0,198283328	0	0	1,220930233
0,21722477	0	0	1,208950631
0,26248857	0	0	1,159424626
0,291751186	0	0	1,201550388
0,286457599	0	0	1,236189446
0,282815034	0	0	1,194457716
0,421352315	0	0	1,182170543
0,456462057	0	0	1,183580547
0,445759166	0	0	1,141955027
0,434992598	0	0	1,171461357
0,461790403	0	0	1,17248062
0,447359453	0	0	1,17248062
0,46814446	0	0	1,427988576
0,525292471	0	0	1,162790698
0,5581134	0	0	1,171187894
0,606146116	0	0	1,149808983
0,597083626	0	0	1,153100775
0,590761631	0	0	1,153914433
0,584729828	0	0	1,150708261
0,584614991	0	0	1,150225212
0,66084973	0	0	1,143410853
0,743123093	0	0	1,152442237
0,765754952	0	0	1,138181371
0,748839364	0	0	1,13372093
0,732198489	0	0	1,13372093
0,716281131	0	0	1,13372093
0,777882794	0	0	1,095204294
0,806057181	0	0	1,122926796
0,810811945	0	0	1,124031008
0,874395938	0	0	1,121148056
0,870231913	0	0	1,089025749
0,91353671	0	0	1,114341085

0,896300168	0	0	1,114341085
0,879702017	0	0	1,114341085
0,918472718	0	0	1,076369173
0,915470732	0	0	1,080132163
0,919485314	0	0	1,112718371
0,915917922	0	0	1,108821509
0,9523486	0	0	1,10261413
0,938752352	0	0	1,104651163
0,977989794	0	0	1,080684996
1	0	0	1,075578156
1	0	0	1,09496124
1	0	0	1,09496124
1	0	0	1,09496124
1	0	0	1,09496124
1	0	0	1,086810164
1	0	0	1,0821691
1	0	0	1,085271318
1	0	0	1,085271318
1	0	0	1,085349541
1	0	0	1,059244175
1	0	0	1,082991336
1	0	0	1,060559697
1	0	0	1,086043616
1	0	0	1,075581395
1	0	0	1,075581395
1	0	0	1,075581395
1	0	0	1,075581395
1	0	0	1,084101949
1	0	0	1,272414063
1	0	0	1,07195649
1	0	0	1,065891473
1	0	0	1,065891473
1	0	0	1,065891473
1	0	0	1,060208374
1	0	0	1,058444892
1	0	0	1,075816257
1	0	0	1,055567284
1	0	0	1,05620155
1	0	0	1,05620155
1	0	0	1,05620155
1	0	0	1,05620155
1	0	0	1,03989412
1	0	0	1,028455686
1	0	0	1,035091864
1	0	0	1,046511628
1	0	0	1,046511628
1	0	0	1,046511628
1	0	0	1,046511628
1	0	0	1,046511628
1	0	0	1,055139733
1	0	0	1,036394102
1	0	0	1,026854695
1	0	0	1,036821705

1	0	0	1,036821705
1	0	0	1,036821705
1	0	0	1,036821705
1	0	0	1,025242013
1	0	0	1,026955373
1	0	0	1,022514491
1	0	0	1,032062749
1	0	0	1,03847869
1	0	0	1,027131783
1	0	0	1,027131783
1	0	0	1,027131783
1	0	0	1,027131783
1	0	0	1,027131783
1	0	0	1,021458048
1	0	0	1,01744186
1	0	0	1,01744186
1	0	0	1,039531914
1	0	0	1,011012453
1	0	0	1,011741906
1	0	0	1,015035076
1	0	0	1,040861238
1	0	0	1,007751938
1	0	0	1,007751938
1	0	0	1,007751938
1	0	0	0,999870605
1	0	0	0,999298853
1	0	0	0,998062016
1	0	0	0,998062016
1	0	0	0,998062016
1	0	0	0,998062016
1	0	0	0,997182643
1	0	0	0,99511399
1	0	0	0,993838203
1	0	0	0,988372093
1	0	0	0,988372093
1	0	0	0,988372093
1	0	0	0,985415845
1	0	0	0,984419721
1	0	0	0,978682171
1	0	0	0,978682171
1	0	0	0,978682171
1	0	0	0,978682171
1	0	0	0,982761712
1	0	0	0,972639647
1	0	0	0,968992248
1	0	0	0,968992248
1	0	0	0,968992248
1	0	0	0,968992248
1	0	0	0,968992248
1	0	0	0,968992248
1	0	0	0,968992248
1	0	0	0,970303469
1	0	0	0,959302326

1	0	0	0,959302326
1	0	0	0,959302326
1	0	0	0,959302326
1	0	0	0,959302326
1	0	0	0,866138322
1	0	0	0,949612403
1	0	0	0,949612403
1	0	0	0,949612403
1	0	0	0,949612403
1	0	0	0,949612403
1	0	0	0,949612403
1	0	0	0,949612403
1	0	0	0,953233152
1	0	0	0,951755727
1	0	0	0,94946692
1	0	0	0,962021081
1	0	0	0,952482551
1	0	0	0,939922481
1	0	0	0,939922481
1	0	0	0,939922481
1	0	0	0,943848737
1	0	0	0,924007664
1	0	0	0,918281197
1	0	0	0,930232558
1	0	0	0,930232558
1	0	0	0,930232558
1	0	0	0,930232558
1	0	0	0,930232558
1	0	0	0,930232558
1	0	0	0,930232558
1	0	0	0,930232558
1	0	0	0,948363992
1	0	0	0,947695715
1	0	0	0,920542636
1	0	0	0,920542636
1	0	0	0,920542636
1	0	0	0,920542636
1	0	0	0,920542636
1	0	0	0,920542636
1	0	0	0,929713648
1	0	0	0,910852713
1	0	0	0,910852713
1	0	0	0,910852713
1	0	0	0,910852713
1	0	0	0,910852713
1	0	0	0,921888458
1	0	0	0,901162791
1	0	0	0,901162791
1	0	0	0,901162791
1	0	0	0,901162791
1	0	0	0,901162791
1	0	0	0,901162791
1	0	0	0,901162791

1	0	0	0,901162791
1	0	0	0,913032209
1	0	0	0,891472868
1	0	0	0,891472868
1	0	0	0,926190997
1	0	0	0,625156289
1	0	0	0,90017706
1	0	0	0,881782946
1	0	0	0,881782946
1	0	0	0,881782946
1	0	0	0,881782946
1	0	0	0,881782946
1	0	0	0,90520279
1	0	0	0,899708153
1	0	0	0,872093023
1	0	0	0,872093023
1	0	0	0,872093023
1	0	0	0,872093023
1	0	0	0,872093023
1	0	0	0,872093023
1	0	0	0,611995104
1	0	0	0,862403101
1	0	0	0,862403101
1	0	0	0,862403101
1	0	0	0,862403101
1	0	0	0,862403101
1	0	0	0,862403101
1	0	0	0,862403101
1	0	0	0,863847209
1	0	0	0,852713178
1	0	0	0,852713178
1	0	0	0,852713178
1	0	0	0,852713178
1	0	0	0,852713178
1	0	0	0,57482591
1	0	0	0,843023256
1	0	0	0,843023256
1	0	0	0,843023256
1	0	0	0,843023256
1	0	0	0,843023256
1	0	0	0,843023256
1	0	0	0,843023256
1	0	0	0,843023256
1	0	0	0,843023256
1	0	0	0,857445004
1	0	0	0,891245316
1	0	0	0,833333333
1	0	0	0,833333333
1	0	0	0,823643411
1	0	0	0,823643411
1	0	0	0,823643411
1	0	0	0,823643411
1	0	0	0,823643411

1	0	0	0,823643411
1	0	0	0,823643411
1	0	0	0,823643411
1	0	0	0,823643411
1	0	0	0,823643411
1	0	0	0,823643411
1	0	0	0,813953488
1	0	0	0,813953488
1	0	0	0,813953488
1	0	0	0,813953488
1	0	0	0,813953488
1	0	0	0,804263566
1	0	0	0,804263566
1	0	0	0,804263566
1	0	0	0,804263566
1	0	0	0,846870996
1	0	0	0,57139592
1	0	0	0,794573643
1	0	0	0,794573643
1	0	0	0,794573643
1	0	0	0,794573643
1	0	0	0,784883721
1	0	0	0,784883721
1	0	0	0,784883721
1	0	0	0,784883721
1	0	0	0,784883721
1	0	0	0,775193798
1	0	0	0,775193798
1	0	0	0,775193798
1	0	0	0,775193798
1	0	0	0,775193798
1	0	0	0,765503876
1	0	0	0,765503876
1	0	0	0,81092519
1	0	0	0,755813953
1	0	0	0,755813953
1	0	0	0,755813953
1	0	0	0,755813953
1	0	0	0,755813953
1	0	0	0,755813953
1	0	0	0,776423777
1	0	0	0,746124031
1	0	0	0,746124031
1	0	0	0,746124031
1	0	0	0,746124031
1	0	0	0,746124031
1	0	0	0,710063808
1	0	0	0,736434109
1	0	0	0,736434109
1	0	0	0,736434109
1	0	0	0,7651875
1	0	0	0,726744186
1	0	0	0,726744186

1	0	0	0,726744186
1	0	0	0,726744186
1	0	0	0,726744186
1	0	0	0,726744186
1	0	0	0,774392703
1	0	0	0,799602724
1	0	0	0,754373101
1	0	0	0,717054264
1	0	0	0,717054264
1	0	0	0,717054264
1	0	0	0,780512849
1	0	0	0,707364341
1	0	0	0,707364341
1	0	0	0,730255028
1	0	0	0,355128049
1	0	0	0,697674419
1	0	0	0,788163579
1	0	0	0,752552891
1	0	0	0,180277628
1	0	0	0,687984496
1	0	0	0,713866202
1	0	0	0,747294793
1	0	0	0,678294574
1	0	0	0,678294574
1	0	0	0,678294574
1	0	0	0,557305375
1	0	0	0,407996736
1	0	0	0,668604651
1	0	0	0,710643666
1	0	0	0,709107869
1	0	0	0,741784301
1	0	0	0,658914729
1	0	0	0,676646333
1	0	0	0,649224806
1	0	0	0,649224806
1	0	0	0,716823856
1	0	0	0,639534884
1	0	0	0,639534884
1	0	0	0,681775983
1	0	0	0,665810624
1	0	0	0,629844961
1	0	0	0,629844961
1	0	0	0,610641881
1	0	0	0,517842011
1	0	0	0,50612906
1	0	0	0,610465116
1	0	0	0,610465116
1	0	0	0,610465116
1	0	0	0,634037115
1	0	0	0,67259074
1	0	0	0,699768382
1	0	0	0,635009437
1	0	0	0,639827817

1	0	0	0,600775194
1	0	0	0,600775194
1	0	0	0,600775194
1	0	0	0,600775194
1	0	0	0,600775194
1	0	0	0,600775194
1	0	0	0,682693621
1	0	0	0,611829968
1	0	0	0,635187817
1	0	0	0,591085271
1	0	0	0,591085271
1	0	0	0,598014592
1	0	0	0,569780591
1	0	0	0,58958833
1	0	0	0,581395349
1	0	0	0,565376352
1	0	0	0,571705426
1	0	0	0,571705426
1	0	0	0,571705426
1	0	0	0,571705426
1	0	0	0,562015504
1	0	0	0,562015504
1	0	0	0,562015504
1	0	0	0,562015504
1	0	0	0,562015504
1	0	0	0,514017393
1	0	0	0,542635659
1	0	0	0,542635659
1	0	0	0,523255814
1	0	0	0,523255814
1	0	0	0,523255814
1	0	0	0,523255814
1	0	0	0,523255814
1	0	0	0,494186047
1	0	0	0,474806202
1	0	0	0,474806202
1	0	0	0,465116279
1	0	0	0,436046512
1	0	0	0,426564107
1	0	0	0,387001512
1	0	0	0,339726366
0,999994963	0	0	0,246702411

Combined.Score
37,75705233
29,82091838
15,77734745
13,08975963
11,274401
11,10562587
9,897274357
8,613788862
7,768401321
7,361702401
6,38172771
6,385940172
6,399021955
6,361699451
5,837248836
5,989820304
5,989820304
5,989820304
5,811979687
5,539471146
5,609636276
5,388055466
4,896339327
4,896105711
5,009425666
4,808754175
4,243368567
4,110656361
3,953110261
4,044180083
3,939160052
3,939160052
4,688792127
3,649375475
3,570797759
3,378298177
3,373746323
3,357636981
3,33022689
3,299933652
3,111998547
2,97358598
2,875861422
2,863852197
2,863852197
2,863852197
2,652645215
2,656196796
2,629021058
2,514984355
2,426559827
2,407212259

2,407212259
2,407212259
2,259011764
2,250983052
2,294328795
2,271319806
2,196749201
2,19812586
2,088321662
1,981439522
2,001454435
2,001454435
2,001454435
2,001454435
1,851772645
1,818320169
1,816882642
1,816882642
1,810870901
1,752859154
1,791024666
1,726320935
1,66427242
1,644086793
1,644086793
1,644086793
1,644086793
1,627992757
1,904966254
1,60188163
1,482734417
1,482734417
1,482734417
1,400451607
1,382618877
1,3911021
1,343509871
1,332483858
1,332483858
1,332483858
1,332483858
1,305251272
1,235891612
1,207795432
1,192983883
1,192983883
1,192983883
1,192983883
1,192983883
1,192791009
1,141240947
1,069935899
1,063873345

1,063873345
1,063873345
1,063873345
1,036780549
1,032662919
1,000121643
0,988638612
0,979226986
0,944780904
0,944780904
0,944780904
0,944780904
0,944780904
0,885486061
0,835324841
0,835324841
0,84445341
0,801682074
0,800268854
0,802437693
0,815050557
0,735112977
0,735112977
0,735112977
0,664486714
0,649224041
0,643742749
0,643742749
0,643742749
0,643742749
0,627546217
0,604517137
0,593795662
0,560801458
0,560801458
0,560801458
0,515718812
0,512827245
0,485866735
0,485866735
0,485866735
0,485866735
0,472935717
0,461533893
0,418507265
0,418507265
0,418507265
0,418507265
0,418507265
0,418507265
0,418507265
0,417217849
0,358283807

0,358283807
0,358283807
0,358283807
0,358283807
0,29574062
0,304750534
0,304750534
0,304750534
0,304750534
0,304750534
0,304750534
0,304750534
0,293686109
0,281785405
0,272220068
0,275069876
0,261909117
0,257456743
0,257456743
0,257456743
0,24462622
0,222942261
0,218508472
0,215948925
0,215948925
0,215948925
0,215948925
0,215948925
0,215948925
0,215948925
0,215948925
0,212309338
0,20767526
0,179773221
0,179773221
0,179773221
0,179773221
0,179773221
0,179773221
0,171597846
0,148478233
0,148478233
0,148478233
0,148478233
0,148478233
0,133332573
0,121618152
0,121618152
0,121618152
0,121618152
0,121618152
0,121618152
0,121618152

0,121618152
0,118156089
0,09875614
0,09875614
0,096306958
0,060965261
0,086608238
0,079467863
0,079467863
0,079467863
0,079467863
0,079467863
0,074021799
0,071214482
0,063345058
0,063345058
0,063345058
0,063345058
0,063345058
0,063345058
0,0436003
0,049999005
0,049999005
0,049999005
0,049999005
0,049999005
0,049999005
0,049999005
0,04292155
0,039063731
0,039063731
0,039063731
0,039063731
0,039063731
0,021670186
0,030198815
0,030198815
0,030198815
0,030198815
0,030198815
0,030198815
0,030198815
0,030198815
0,030198815
0,029395976
0,027433788
0,023091636
0,023091636
0,01745897
0,01745897
0,01745897
0,01745897
0,01745897

0,01745897
0,01745897
0,01745897
0,01745897
0,01745897
0,01745897
0,01304784
0,01304784
0,01304784
0,01304784
0,01304784
0,009635606
0,009635606
0,009635606
0,009635606
0,009893056
0,006101012
0,007029305
0,007029305
0,007029305
0,007029305
0,005064313
0,005064313
0,005064313
0,005064313
0,005064313
0,003602439
0,003602439
0,003602439
0,003602439
0,003602439
0,002529574
0,002529574
0,002363837
0,001753067
0,001753067
0,001753067
0,001753067
0,001753067
0,001753067
0,0013006
0,001198951
0,001198951
0,001198951
0,001198951
0,001198951
0,001001636
0,000809183
0,000809183
0,000809183
0,000603932
0,000538998
0,000538998

0,000538998
0,000538998
0,000538998
0,000538998
0,000526747
0,000449263
0,000415969
0,000354464
0,000354464
0,000354464
0,000283082
0,00023031
0,00023031
0,000190141
7,90249E-05
0,000148039
0,00015055
0,000124926
2,51643E-05
9,4354E-05
9,0003E-05
7,20089E-05
5,98606E-05
5,98606E-05
5,98606E-05
3,79039E-05
2,50513E-05
3,80396E-05
3,76877E-05
3,1579E-05
3,13592E-05
2,44471E-05
2,3935E-05
1,61077E-05
1,61077E-05
1,70572E-05
1,10652E-05
1,10652E-05
9,08325E-06
8,61099E-06
8,05595E-06
8,05595E-06
7,70106E-06
4,68256E-06
4,34894E-06
5,23611E-06
5,23611E-06
5,23611E-06
5,23255E-06
5,53709E-06
5,599E-06
4,97772E-06
4,94111E-06

4,62224E-06
4,62224E-06
4,62224E-06
4,62224E-06
4,62224E-06
4,62224E-06
5,249E-06
4,62089E-06
4,61045E-06
4,25485E-06
4,25485E-06
4,26609E-06
4,01957E-06
4,12461E-06
4,02642E-06
3,85161E-06
3,87491E-06
3,87491E-06
3,87491E-06
3,87491E-06
3,76516E-06
3,76516E-06
3,76516E-06
3,76516E-06
3,76516E-06
3,43155E-06
3,60181E-06
3,60181E-06
3,46498E-06
3,46498E-06
3,46498E-06
3,46498E-06
3,46498E-06
3,2704E-06
3,14198E-06
3,14198E-06
3,07784E-06
2,88546E-06
2,7076E-06
2,3035E-06
2,00272E-06
1,24269E-06

Genes

NCKAP1;TCERG1;UHRF1BP1L;DENND5B;HNRNPU;HNRNPR;GLS;TIAL1;EFR3A;DAG1;FBXO3;SNIP1;SNR
OTUD4;NCKAP1;TCERG1;UHRF1BP1L;DENND5B;MAML1;HNRNPR;LDLRAD4;BACH1;DCAF7;FAM107B;P
TDRKH;PRDM8;ANKRD13B;CHRM3;HPSE2;PRDM6;POGK;ANTXR2;IKZF3;CDH6;GJA3;CDH2;FRRS1L;HO
NCKAP1;HPSE2;PRDM6;LDLRAD4;IKZF3;CTGF;CDH6;CDH2;DPYSL5;KIF5C;FRRS1L;IGLON5;SALL4;RAV
OTUD4;NCKAP1;HNRNPR;NUDT5;DAG1;SNIP1;PELO;RNF111;PSPH;FBXW4;CMC1;ATP11A;MOB3C;MAF;
HRK;CCDC71L;RAB3C;NRP2;DYRK3;ONECUT2;IRS4;TNFAIP3;RCSD1;BMI1;PPP1R9A;CLDN1;CTGF;GJA3
PRDM8;RAB3C;NPFFR1;PRDM6;IRS4;IKZF3;CDH6;EFEMP1;GJA3;CDH2;DPYSL5;KIF5C;FRRS1L;SALL4;R
PRDM8;CHRM3;NPFFR1;DENND5B;PRDM6;CTGF;EPS8;CDH6;DPYSL5;KIF5C;FRRS1L;HOXA3;MAP3K9;P
NPFFR1;ATP8A2;HPSE2;PRDM6;KLHL32;IRS4;RORB;IKZF3;GJA3;CDH2;DPYSL5;FADS6;KIF5C;SALL4;BS
TMEM167B;POGK;ZBTB20;NUDT5;TXNDC17;CDH2;FADS6;RAVER2;TRIM24;PIM3;PTGFRN;SNRPD3;C21
TMEM167B;KIAA0141;PHAX;NUDT5;TXNDC17;PCMT1;RPS6KA6;GJA3;DPYSL5;TRIM24;MAP3K9;SNRPD3
CHRM3;RAB3C;NPFFR1;ATP8A2;PRDM6;CDH6;DPYSL5;FRRS1L;IGLON5;RAVER2;MACROD2;BSN;SOX6;
GABPB2;ZNF292;NUDT5;TXNDC17;TIAL1;RIMS3;GJA3;CDH2;FADS6;KIAA0895;PTGFRN;SNRPD3;PELO;IL
NPFFR1;HPSE2;PRDM6;CLDN1;IKZF3;CTGF;CDH6;GJA3;SALL4;HOXA3;SOX6;TNS1;SFMBT2;FNDC3B;VA
TDRKH;FAM49A;CHRM3;DENND5B;LOXL4;CTGF;CDH6;EFEMP1;CDH2;FADS6;KIF5C;FRRS1L;RAVER2;D
NCKAP1;GABPB2;ANTXR2;CDH6;TIAL1;PCMT1;CDH2;PAPOLG;HOXA3;PIM3;RNF111;PSPH;KPNA1;FBXW
ATP8A2;KIAA0141;RORB;CLDN1;NUDT4;AMOT;RPS6KA6;GJA3;KIAA0895;RAVER2;PIM3;PTGFRN;SNRPD
NPFFR1;ATP8A2;LOXL4;LDLRAD4;ANTXR2;CDH6;GJA3;FADS6;FRRS1L;PTGFRN;BSN;SH3GL2;PCDHAC2
RAB3C;MAML1;FRMPD4;POGK;KLHL32;ZBTB20;RORB;AMOT;EFR3A;CDH2;KIAA0895;RAVER2;PIM3;MAP
TDRKH;PRDM8;ANKRD13B;DENND5B;PRDM6;POGK;ANTXR2;IKZF3;EPS8;CDH2;DPYSL5;FRRS1L;DPYS
TDRKH;NCKAP1;GABPB2;MRFAP1;RORB;ANTXR2;TIAL1;PCMT1;CDH2;KIF5C;FNTB;DAG1;PIM3;PTGFRN
RAB3C;GABPB2;TMEM167B;HPSE2;FRMPD4;POGK;ZBTB20;NUDT4;EFR3A;SESN3;GJA3;CDH2;KIAA0895
NCKAP1;TCERG1;TMEM167B;PRDM6;IRS4;MRFAP1;CLDN1;IKZF3;NUDT4;CDH6;TIAL1;EFR3A;RPS6KA6;
PRDM8;ANKRD13B;UHRF1BP1L;NPFFR1;ATP8A2;PRDM6;LOXL4;PHAX;LDLRAD4;ANTXR2;IKZF3;CDH6;D
NRP2;TMEM167B;ZNF292;KLHL32;TNFAIP3;PHAX;RCSD1;PPP1R9A;SMG7;MYPN;PITPNC1;TXNDC17;GL
RAB3C;MAML1;POGK;KIAA0141;ZBTB20;RORB;NUDT5;AMOT;EFEMP1;GJA3;CDH2;KIAA0895;RAVER2;M
CCDC71L;ANKRD13B;NPFFR1;ATP8A2;HPSE2;IRS4;PHAX;BACH1;DCAF7;GJA3;FADS6;KIF5C;IGLON5;KI
OTUD4;RAB3C;ATP8A2;MAML1;FRMPD4;POGK;KLHL32;KIAA0141;ZBTB20;RORB;AMOT;RIMS3;SESN3;G
GABPB2;TMEM167B;MAML1;FRMPD4;POGK;KLHL32;ZBTB20;RORB;CTGF;NUDT4;AMOT;RPS6KA6;EFEM
GABPB2;MAML1;FRMPD4;POGK;KLHL32;IRS4;ZBTB20;RORB;NUDT5;AMOT;RIMS3;SESN3;GJA3;CDH2;K
GABPB2;POGK;KIAA0141;ZBTB20;LOXL4;EPS8;EFEMP1;CDH2;FADS6;KIAA0895;RAVER2;PIM3;PTGFRN;
NCKAP1;GABPB2;KLHL32;NUDT5;ANTXR2;CDH6;PCMT1;CDH2;DAG1;CHP1;PIM3;RNF111;PSPH;IL13RA1
FAM49A;RNF180;SHOX2;SIX1;PRICKLE1;APCDD1;BMI1;COL19A1;ADD2;ARHGAP20;KIF5C;ERBB4;SCN5
CCDC71L;GABPB2;HNRNPU;NUDT5;SESN3;PAPOLG;TRIM24;EPC1;SEC62;RNF111;PSPH;MEF2A;TMEM1
RAB3C;FRMPD4;RORB;CLDN1;AMOT;CDH6;RIMS3;CDH2;KIAA0895;SNRPD3;IL6R;CSNK1G3;CXADR;ST6
RAB3C;NPFFR1;ATP8A2;HPSE2;PRDM6;KLHL32;IRS4;RORB;CDH6;CCND3;SESN3;DPYSL5;FADS6;KIF5C
GABPB2;HNRNPR;NUDT5;ANTXR2;SNRPD3;PSPH;KPNA1;MEF2A;FBXW4;RAB2B;MBNL2;DYRK1A;TAP2;
RAB3C;MAML1;KLHL32;KIAA0141;ZBTB20;RORB;NUDT5;AMOT;SESN3;RAVER2;MAP3K9;PTGFRN;SNRP
RAB3C;POGK;IRS4;ZBTB20;NUDT5;TXNDC17;NUDT4;AMOT;GJA3;CDH2;FADS6;KIAA0895;RAVER2;SNRP
RAB3C;MRFAP1;ZBTB20;NUDT5;AMOT;EPS8;GJA3;CDH2;RAVER2;PTGFRN;SNRPD3;PELO;IL6R;SH3GL2
UHRF1BP1L;TMEM167B;MRFAP1;ANTXR2;FAM107B;SESN3;PAPOLG;TRIM24;DIP2B;SLC25A45;MEF2A;F
RAB3C;GABPB2;TMEM167B;FRMPD4;POGK;ZBTB20;RORB;NUDT5;TXNDC17;AMOT;CCND3;RIMS3;GJA3
GABPB2;ZBTB20;RORB;CLDN1;TXNDC17;NUDT4;AMOT;CCND3;EFEMP1;GJA3;CDH2;KIAA0895;RAVER2
GABPB2;KIAA0141;PHAX;NUDT5;TXNDC17;TIAL1;EFR3A;KIAA0895;EPC1;IL6R;RNF111;MEF2A;FBXW4;C
KIAA0141;CLDN1;GJA3;CDH2;KIAA0895;PCDHAC1;TMEM194B;ALG6;CMC1;ALG14;FNDC3A;PCDHA13;H
OTUD4;LTN1;PHAX;TM7SF3;TXNDC17;RIMS3;TRIM24;FBXO3;PCDHAC1;FNBP4;BROX;TMOD2;CMC1;GP
PRDM8;RAB3C;GABPB2;FRMPD4;KLHL32;ZBTB20;PHAX;RORB;NUDT5;CLDN1;TXNDC17;CCND3;EFEMP
GABPB2;KIAA0141;MRFAP1;ZBTB20;NUDT5;CLDN1;IKZF3;TXNDC17;AMOT;TIAL1;EFEMP1;CDH2;KIAA08
RAB3C;NPFFR1;ATP8A2;HPSE2;KLHL32;IRS4;IKZF3;EPS8;GJA3;FRRS1L;IGLON5;RAVER2;MAP3K9;PTG
RAB3C;GABPB2;ATP8A2;FRMPD4;POGK;KIAA0141;RORB;NUDT5;NUDT4;RIMS3;GJA3;CDH2;KIAA0895;R
PRDM8;CCDC71L;NCKAP1;ANKRD13B;RAB3C;NPFFR1;ATP8A2;HPSE2;PRDM6;KLHL32;LOXL4;CTGF;FA
TDRKH;GABPB2;ATP8A2;MAML1;KLHL32;NUDT5;TIAL1;PCMT1;CDH2;FADS6;KIF5C;FNTB;DAG1;PIM3;PC

OTUD4;NCKAP1;ANKRD13B;PRDM6;KLHL32;MRFAP1;LOXL4;DPYSL3;CHP1;MAP3K9;TGM2;FBXW4;ST6G
NCKAP1;NUDT5;ANTXR2;TIAL1;GJA3;FADS6;FRRS1L;FNTB;DAG1;SNIP1;PIM3;BSN;PELO;SH3GL2;AGFG
NPFFR1;GABPB2;HPSE2;ZNF292;PHAX;NUDT5;TXNDC17;EPS8;TIAL1;GJA3;RAVER2;TRIM24;SNRPD3;TM
OTUD4;GABPB2;ATP8A2;TMEM167B;LTN1;KLHL32;KIAA0141;ZBTB20;CALML4;NUDT5;TM7SF3;IKZF3;TXN
POGK;KIAA0141;ZBTB20;LOXL4;RORB;NUDT4;EPS8;RIMS3;CDH2;KIAA0895;RAVER2;DPYSL3;MAP3K9;P
RAB3C;GABPB2;TMEM167B;POGK;ZBTB20;RORB;NUDT5;TXNDC17;AMOT;RIMS3;GJA3;CDH2;KIAA0895;
RAB3C;GABPB2;TMEM167B;POGK;ZBTB20;RORB;NUDT5;TXNDC17;AMOT;SESN3;CDH2;RAVER2;SNRPD
NCKAP1;RAB3C;ATP8A2;HPSE2;FRMPD4;LOXL4;CLDN1;IKZF3;AMOT;CDH6;FADS6;SALL4;DPYSL3;FBXO
OTUD4;NCKAP1;GABPB2;HNRNPR;RORB;NUDT5;ANTXR2;NUDT4;TIAL1;PCMT1;EFR3A;CCND3;CDH2;FN
MAML1;LTN1;KIAA0141;IRS4;MRFAP1;ZBTB20;RIMS3;RAVER2;FBXO3;SNRPD3;ZNF687;KCNH1;PCDHAC
PRDM8;CHRM3;RAB3C;NPFFR1;ATP8A2;HPSE2;PRDM6;KLHL32;IKZF3;CDH6;EFEMP1;GJA3;FADS6;FRR
TCERG1;KLHL32;KIAA0141;IRS4;MRFAP1;DCAF7;NUDT4;GJA3;TRIM24;EPC1;ZNF687;SEC62;SH3GL2;AG
NCKAP1;GABPB2;HNRNPU;TIAL1;CHP1;MAP3K9;SNRPD3;PELO;PSPH;KPNA1;RAB2B;MAP2K1;CXADR;S
NCKAP1;GABPB2;MRFAP1;NUDT5;TXNDC17;NUDT4;TIAL1;PCMT1;EFR3A;FNTB;SNIP1;PIM3;AGFG2;TGM
PRDM8;NPFFR1;FRMPD4;PRDM6;LOXL4;CLDN1;IKZF3;EPS8;EFEMP1;GJA3;FADS6;DPYSL3;IL6R;WLS;ST
NPFFR1;ATP8A2;HPSE2;FRMPD4;PRDM6;IRS4;CLDN1;IKZF3;CTGF;EPS8;CDH6;EFEMP1;DPYSL5;FADS6
ZNF292;KIAA0141;MRFAP1;IKZF3;TIAL1;CDH2;RAVER2;SEC62;RNF111;AGFG2;FBXW4;RAB2B;TMEM194
TDRKH;CHRM3;GABPB2;ATP8A2;PHAX;NUDT5;ANTXR2;CLDN1;IKZF3;TXNDC17;TIAL1;PCMT1;SEC62;AG
RAB3C;HPSE2;POGK;ZBTB20;NUDT5;AMOT;RIMS3;SESN3;GJA3;CDH2;KIAA0895;RAVER2;PIM3;MAP3K9;
KIAA0141;NUDT5;EPS8;RAVER2;PTGFRN;SNRPD3;PELO;TMEM194B;CXADR;BROX;CMC1;HPCAL4;C9OF
MAML1;KIAA0141;MRFAP1;ZBTB20;NUDT5;CTGF;NUDT4;AMOT;SESN3;KIAA0895;RAVER2;TRIM24;PIM3;
TMEM167B;FRMPD4;KLHL32;KIAA0141;IRS4;ZBTB20;RORB;IKZF3;NUDT4;AMOT;EPS8;GJA3;CDH2;FADS
GPR27;ATP8A2;KIAA0141;DUSP19;TNFAIP3;SIX1;PPP1R9A;SMG7;CLDN1;PITPNC1;ELAVL2;TXNDC17;PC
SCOC;GABPB2;PHAX;RORB;NUDT5;ANTXR2;CLDN1;CDH6;TIAL1;PCMT1;FNTB;DAG1;BSN;RNF111;PCDH
FRMPD4;POGK;MRFAP1;HNRNPU;PHAX;TM7SF3;IKZF3;CDH2;FADS6;RUVBL1;TRIM24;FBXO3;EPC1;ZNF
OTUD4;PRDM6;KLHL32;MRFAP1;HNRNPR;CALML4;IKZF3;MAP3K9;PTGFRN;BSN;RNF111;KPNA1;FBXW4;
POGK;LTN1;KIAA0141;IRS4;PHAX;IKZF3;SESN3;CDH2;RUVBL1;FBXO3;EPC1;SEC62;AGFG2;TGM2;IL13R
GPR27;TMEM167B;DIRAS2;IRS4;DUSP19;TNFAIP3;PHAX;HNRNPR;CTDSPL2;NUDT5;PPP1R9A;CLDN1;PI
EGR4;SWAP70;PCDHB15;FNDC3A;PCDHA12;MAPK10;DDX19B;PCDHA1;PCDHA3;PCDHA2;MACROD2;TR
RAB3C;GABPB2;TMEM167B;FRMPD4;POGK;ZBTB20;RORB;NUDT5;AMOT;SESN3;GJA3;KIAA0895;RAVER
TDRKH;GABPB2;KIAA0141;HNRNPR;CTGF;EPS8;TIAL1;EFR3A;KIAA0895;RAVER2;PIM3;PTGFRN;ZNF687
TCERG1;DENND5B;IRS4;IKZF3;DCAF7;RPS6KA6;FADS6;RUVBL1;FBXO3;SEC62;PELO;TNS1;PRKAB2;PC
GABPB2;MAML1;LTN1;ANTXR2;TXNDC17;PCMT1;CCND3;WLS;FNBP4;KCMF1;RAB2B;PRKAB2;ALG6;BRO
RAB3C;GABPB2;KIAA0141;ZBTB20;RORB;NUDT5;AMOT;EPS8;GJA3;FADS6;KIAA0895;RAVER2;PIM3;PTG
RAB3C;GABPB2;TMEM167B;POGK;ZBTB20;RORB;EPS8;RIMS3;SESN3;GJA3;CDH2;KIAA0895;RAVER2;PI
GABPB2;TTC22;POGK;PDE3B;ZBTB20;RORB;CTDSPL2;JPH1;PITPNC1;CCND3;SPRED1;RIMS3;ALDH2;KI
RAB3C;MAML1;KIAA0141;ZBTB20;RORB;NUDT5;TXNDC17;AMOT;EPS8;EFEMP1;GJA3;CDH2;FADS6;RAV
GABPB2;MAML1;NUDT5;ANTXR2;EPS8;TIAL1;PCMT1;EFR3A;FNTB;SNIP1;TGM2;IL13RA1;TMEM194B;MA
ANKRD13B;DENND5B;KIAA0141;MRFAP1;LOXL4;HNRNPU;TM7SF3;ANTXR2;CTGF;EPS8;TIAL1;CCND3;R
GABPB2;TMEM167B;MRFAP1;CLDN1;IKZF3;EPS8;PCMT1;CDH2;DPYSL3;SNRPD3;PELO;TGM2;TMEM194
SCOC;KIAA0141;ZBTB20;LOXL4;CLDN1;AMOT;RIMS3;CDH2;FBXO3;MACROD2;SLC25A45;KCMF1;BROX;
PRDM8;FAM49A;ANKRD13B;NPFFR1;ATP8A2;HPSE2;DENND5B;FRMPD4;PRDM6;IRS4;RORB;ANTXR2;CL
GABPB2;HPSE2;KIAA0141;PHAX;NUDT5;CLDN1;TXNDC17;GLS;TIAL1;EFR3A;GJA3;RAVER2;FBXO3;SNR
PRDM8;ANKRD13B;NPFFR1;HPSE2;DENND5B;FRMPD4;KLHL32;IRS4;RORB;CLDN1;IKZF3;CTGF;AMOT;C
FAM49A;ATP8A2;TMEM167B;LTN1;HNRNPU;PHAX;PCMT1;KIAA0895;BSN;IL6R;AGFG2;KCNH1;IL13RA1;T
MRFAP1;PHAX;SESN3;TRIM24;HOXA3;BTRC;AGFG2;C3ORF14;TNS1;EOMES;PRKAB2;C21ORF59;TMOD
CCDC71L;FAM49A;MAML1;KLHL32;MRFAP1;ANTXR2;EPS8;FBXO3;AP2M1;RNF111;MEF2A;CSNK1G3;GA
OTUD4;FRMPD4;KLHL32;KIAA0141;ZBTB20;SESN3;SNRPD3;BSN;TGM2;EOMES;KCMF1;TMEM194B;BRO
GABPB2;KIAA0141;ZBTB20;TM7SF3;DCAF7;FBXO3;PIM3;SLC25A45;PELO;PSPH;MEF2A;FNBP4;TPM3;SW
SNAP23;DUSP19;SIX1;CTDSPL2;NUDT5;CLDN1;LITAF;PITPNC1;PHF8;AMOT;CCND3;SPRED1;GJA3;KIAA
TDRKH;UHRF1BP1L;PRDM6;KLHL32;NUDT5;ANTXR2;EFR3A;EFEMP1;SESN3;DPYSL5;KIF5C;DPYSL3;MA
FAM49A;RAB3C;GABPB2;TMEM167B;MAML1;FRMPD4;POGK;KLHL32;KIAA0141;IRS4;ZBTB20;RORB;NUD
TMEM167B;POGK;DCAF7;CCND3;EPC1;CHP1;SLC25A45;MEF2A;FBXW4;TMEM194B;MBNL2;ALG6;YOD1;

OTUD4;GABPB2;NUDT5;TIAL1;PCMT1;EFR3A;PAPOLG;FNTB;HOXA3;DAG1;SNIP1;PIM3;RNF111;AGFG2;F
TDRKH;GABPB2;LOXL4;PHAX;NUDT5;ANTXR2;IKZF3;TIAL1;PCMT1;FADS6;HOXA3;SNIP1;BSN;PELO;RNF
NCKAP1;GABPB2;MAML1;NUDT5;NUDT4;TIAL1;PCMT1;CDH2;FNTB;TRIM24;PIM3;RNF111;PCDHAC2;TME
RAB3C;GABPB2;KLHL32;KIAA0141;IRS4;ZBTB20;RORB;AMOT;RPS6KA6;RIMS3;SESN3;GJA3;CDH2;DPYS
NCKAP1;TCERG1;GABPB2;MRFAP1;NUDT5;ANTXR2;TXNDC17;TIAL1;PCMT1;EFR3A;CCND3;CDH2;FNTB
GABPB2;TMEM167B;KLHL32;KIAA0141;ZBTB20;CLDN1;IKZF3;TXNDC17;RPS6KA6;RIMS3;GJA3;CDH2;FAD
RAB3C;GABPB2;TMEM167B;FRMPD4;KIAA0141;RORB;RIMS3;GJA3;FADS6;KIAA0895;RAVER2;PIM3;PTGF
HRK;GPR27;NPFFR1;TTC22;PRDM6;IKZF3;CDH6;JPH3;DPYSL5;FRRS1L;SLC22A17;SALL4;RSPO2;TMEM
TDRKH;NCKAP1;KLHL32;LDLRAD4;DPYSL5;BSN;PELO;SH3GL2;PCDHAC2;KCNH1;TGM2;ALG14;GPR75;H
FAM49A;RAB3C;NPFFR1;LTN1;IRS4;CDH6;GJA3;IGLON5;SALL4;DPYSL3;MAP3K9;MACROD2;IL6R;AP2M1
MAML1;MRFAP1;HNRNPU;IKZF3;GJA3;HOXA3;FBXO3;EPC1;MAP3K9;SEC62;AP2M1;TGM2;MEF2A;FNBP4
ZNF292;POGK;KIAA0141;PHAX;HNRNPR;NUDT5;TXNDC17;PCMT1;RUVBL1;MAP3K9;PSPH;IL13RA1;PRKA
ZNF292;KIAA0141;ZBTB20;RORB;TM7SF3;CLDN1;IKZF3;RPS6KA6;RIMS3;SESN3;KIAA0895;RUVBL1;SLC2
GABPB2;TMEM167B;MAML1;FRMPD4;POGK;KLHL32;RORB;NUDT5;NUDT4;AMOT;RIMS3;GJA3;CDH2;KIA
CCDC71L;FAM49A;RAB3C;TCERG1;NPFFR1;HPSE2;FRMPD2;IRS4;PHAX;RORB;RPS6KA6;CDH2;KIF5C;R
NCKAP1;PRDM6;KLHL32;CCND3;EFEMP1;FADS6;IGLON5;BSN;ST6GAL2;FNDC3B;ARID5B;FNDC3A;SHISA
RBM28;CUL3;BMI1;ADAMTS5;PCDHA1;PCDHA5;TRIM24;PCDHA4;SH3BGRL2;PCDHA3;PCDHA2;PCDHA9;
PRDM8;FAM49A;DENND5B;PRDM6;POGK;ANTXR2;CLDN1;EPS8;CDH2;DPYSL5;RAVER2;DPYSL3;PIM3;M
FAM49A;GABPB2;MAML1;ZNF292;PRDM6;KLHL32;KIAA0141;ZBTB20;IKZF3;TXNDC17;RPS6KA6;RIMS3;SN
RAB3C;GABPB2;ZBTB20;NUDT5;CLDN1;EPS8;RIMS3;CDH2;KIAA0895;RAVER2;PTGFRN;SNRPD3;PELO;K
DLX1;KCNIP2;SORT1;PRDM6;RHOBTB3;GAB1;JPH1;LRP8;DKK3;STXBP5L;CTIF;EFNA3;TMEM56;IGF2BP1
TDRKH;NCKAP1;ATP8A2;LOXL4;ANTXR2;TIAL1;PCMT1;EFEMP1;FADS6;FNTB;RNF111;PCDHAC2;AGFG2;
OTUD4;NCKAP1;ANTXR2;TIAL1;PCMT1;PAPOLG;FNTB;TRIM24;HOXA3;SNIP1;PIM3;RNF111;PSPH;KPNA1
OTUD4;NCKAP1;PRDM6;KLHL32;HNRNPR;LDLRAD4;NUDT5;SNIP1;CHP1;MAP3K9;BSN;FBXW4;KCMF1;R
PRDM8;ATP8A2;TMEM167B;KLHL32;KIAA0141;IRS4;ZBTB20;NUDT5;IKZF3;TXNDC17;AMOT;CDH6;CCND3
HRK;GPR27;NPFFR1;HPSE2;TTC22;PRDM6;RCSD1;IKZF3;JPH3;GJA3;DPYSL5;FRRS1L;SLC22A17;SALL4
GABPB2;KIAA0141;ZBTB20;TM7SF3;BACH1;FBXO3;SLC25A45;PELO;FNBP4;KCMF1;TMEM194B;BROX;CM
LTN1;ANTXR2;FAM204A;DCAF7;EFR3A;RAVER2;EPC1;SNIP1;SEC62;SH3GL2;RNF111;IL13RA1;PRKAB2;A
MAML1;MRFAP1;ANTXR2;PCMT1;PIM3;AP2M1;KCNH1;CSNK1G3;GPR75;HPCAL4;PIAS1;ACAP2;CDC34;A
ZNF292;LTN1;KIAA0141;TM7SF3;SESN3;SNRPD3;ZNF687;AP2M1;KCMF1;RAB2B;TMEM194B;C5ORF15;SV
MAML1;KIAA0141;NUDT5;IKZF3;TXNDC17;GJA3;RAVER2;PTGFRN;SNRPD3;BSN;TMEM194B;CXADR;C5O
GABPB2;ZNF292;NUDT5;IKZF3;TXNDC17;GLS;DPYSL5;FBXO3;PTGFRN;SNRPD3;BSN;EBF1;C9ORF64;DK
PRDM8;RAB3C;NPFFR1;ATP8A2;HPSE2;FRMPD4;PRDM6;IRS4;RORB;NUDT5;CLDN1;IKZF3;CDH6;RPS6K
TMEM167B;HNRNPU;NUDT5;ANTXR2;TXNDC17;PCMT1;EFR3A;RUVBL1;DAG1;SNIP1;CHP1;KPNA1;RAB2
SCOC;ATP8A2;KLHL32;LOXL4;PHAX;RORB;ANTXR2;CLDN1;TIAL1;EFEMP1;FADS6;FNTB;DAG1;PTGFRN;
OTUD4;FAM49A;NCKAP1;KIAA0141;MRFAP1;FADS6;TRIM24;ZNF687;SEC62;SH3GL2;IL13RA1;PCDHAC1;
NPFFR1;FRMPD4;EPS8;ELK4;RPS6KA6;SESN3;DPYSL5;FADS6;DPYSL3;FNTB;MACROD2;BSN;PELO;IL6F
TDRKH;FAM49A;CHRM3;NPFFR1;ATP8A2;DENND5B;POGK;KLHL32;CTGF;AMOT;CDH6;EFEMP1;SESN3;C
UHRF1BP1L;MRFAP1;ZBTB20;DCAF7;NUDT4;AMOT;EFR3A;FADS6;PELO;TNS1;RAB2B;C5ORF15;KIAA146
FAM49A;KLHL32;KIAA0141;ZBTB20;CALML4;SLC25A45;ZNF687;KCMF1;PRKAB2;TMEM194B;BROX;USP2;
PRDM6;KLHL32;ANTXR2;CLDN1;TIAL1;PCMT1;DPYSL5;FADS6;FNTB;HOXA3;DAG1;SEC62;PELO;AGFG2;
TDRKH;NCKAP1;KLHL32;RORB;NUDT5;EFR3A;DPYSL5;KIF5C;FNTB;HOXA3;DAG1;MACROD2;SLC25A45;
GABPB2;TMEM167B;POGK;KLHL32;KIAA0141;ZBTB20;CTGF;TXNDC17;NUDT4;RIMS3;KIAA0895;RAVER2;
TNFAIP8;FRMPD4;TNFAIP3;PHAX;PITPNC1;TXNDC17;NUDT4;CDH6;NHSL2;GJA3;CDH2;TRIM24;KIF1B;MA
GABPB2;KLHL32;KIAA0141;CLDN1;GJA3;DPYSL5;RAVER2;SLC25A45;PELO;KCNH1;TGM2;IL13RA1;EOME
NCKAP1;GABPB2;NUDT5;TXNDC17;ELK4;TIAL1;PAPOLG;SEC62;RNF111;PSPH;KPNA1;TMEM194B;ESCO
FRMPD4;KIAA0141;ZBTB20;RORB;BACH1;TXNDC17;FBXO3;SNRPD3;SLC25A45;AGFG2;KCMF1;RAB2B;U
FAM49A;ATP8A2;FRMPD4;PRDM6;IRS4;CLDN1;IKZF3;AMOT;ELK4;FADS6;PCDHAC2;C3ORF14;KCNH1;TN
FRMPD4;FRMPD2;MRFAP1;AMOT;EFR3A;RPS6KA6;PAPOLG;RAVER2;DPYSL3;SNRPD3;SH3GL2;PCDHAC
PRDM8;SCOC;ATP8A2;ZNF292;PRDM6;LTN1;MRFAP1;LOXL4;HNRNPU;CALML4;PCMT1;SESN3;GJA3;DPY
NPFFR1;ATP8A2;DENND5B;FRMPD4;LOXL4;ANTXR2;CLDN1;IKZF3;EPS8;GJA3;IL6R;SH3GL2;WLS;TGM2;
PRDM8;RAB3C;NPFFR1;ATP8A2;HPSE2;FRMPD4;PRDM6;IRS4;RORB;IKZF3;GJA3;DPYSL5;FADS6;SALL4;
ATP8A2;TMEM167B;KLHL32;KIAA0141;CLDN1;IKZF3;CTGF;SESN3;FADS6;FBXO3;BSN;PELO;AGFG2;TGM

OTUD4;TCERG1;POGK;NUDT5;ANTXR2;TIAL1;FNTB;DAG1;SNIP1;PIM3;RNF111;KPNA1;RAB2B;TMEM194
RAB3C;TCERG1;IRS4;HNRNPU;PHAX;DCAF7;NUDT4;SESN3;CDH2;TRIM24;SNIP1;RNF111;C3ORF14;PSP
MAML1;NUDT5;ANTXR2;TIAL1;PCMT1;RPS6KA6;PAPOLG;FNTB;SNIP1;PIM3;RNF111;KPNA1;TGM2;FBXW
OTUD4;TCERG1;GABPB2;HNRNPR;NUDT5;TIAL1;PCMT1;CCND3;FNTB;TRIM24;PIM3;RNF111;PSPH;KPN
GPRIN3;RAB3C;PALM2;ASXL3;RASSF8;FMN1;SPRY1;PPM1E
MRFAP1;HNRNPU;NUDT5;ANTXR2;TXNDC17;SNIP1;BTRC;AGFG2;KPNA1;FNBP4;RAB2B;YOD1;EREG;AC
NCKAP1;GABPB2;PHAX;NUDT5;TIAL1;PCMT1;FADS6;FNTB;HOXA3;PTGFRN;BSN;AGFG2;TGM2;ALG14;A
NCKAP1;TMEM167B;KLHL32;LOXL4;HNRNPR;ANTXR2;BACH1;DCAF7;CCND3;CHP1;BTRC;KPNA1;MEF2
NPFFR1;ATP8A2;HPSE2;PRDM6;IRS4;MRFAP1;CTGF;TXNDC17;CDH2;DPYSL5;FBXO3;SNIP1;SNRPD3;S
NCKAP1;ANKRD13B;GABPB2;KIAA0141;ZBTB20;RORB;TM7SF3;AMOT;FBXO3;PELO;IL13RA1;RAB2B;BRO
LTN1;KLHL32;EPS8;CDH2;FBXO3;SEC62;SH3GL2;AGFG2;PSPH;RAB2B;C21ORF59;USP2;GAPVD1;BCMO
LTN1;KIAA0141;ANTXR2;RAVER2;MAP3K9;IL13RA1;EOMES;KCMF1;RAB2B;PRKAB2;BROX;USP2;ALG14;
PRDM8;GABPB2;ATP8A2;TMEM167B;FRMPD4;PHAX;CLDN1;NUDT4;AMOT;RIMS3;CDH2;KIAA0895;MAP3
FAM49A;NPFFR1;ATP8A2;FRMPD4;PRDM6;RORB;IKZF3;CDH6;DPYSL5;FADS6;KIF5C;BSN;SH3GL2;KCNH
NCKAP1;ANKRD13B;PHAX;LDLRAD4;IKZF3;SESN3;FADS6;DAG1;EPC1;PELO;SH3GL2;KPNA1;MEF2A;RA
DCAF7;TXNDC17;DAG1;DIP2B;SEC62;PRKAB2;TMEM194B;ENTPD1;ALG6;ESCO1;OMD;RNASE6;RC3H1;
FAM49A;RAB3C;NPFFR1;ATP8A2;HPSE2;FRMPD2;KLHL32;IRS4;RORB;IKZF3;CDH6;CCND3;RIMS3;SESN
ATP8A2;FRMPD4;KIAA0141;IRS4;CDH2;SLC25A45;ZNF687;TGM2;PCDHAC1;PRKAB2;TMEM194B;USP2;P
KIAA0141;ZBTB20;RIMS3;GJA3;BSN;KCMF1;BROX;ALG14;FTSJ2;HPCAL4;FOXP2;C9ORF64;GTPBP4;BCM
OTUD4;MAML1;NUDT5;ANTXR2;TIAL1;PCMT1;EFR3A;CDH2;PAPOLG;HOXA3;RNF111;RAB2B;MAP2K1;ES
PRDM8;CCDC71L;ATP8A2;IRS4;PHAX;CLDN1;IKZF3;SESN3;GJA3;CDH2;DPYSL5;IGLON5;DPYSL3;MAP3K
NRP2;GABRB1;TMEM167B;DUSP19;TNFAIP3;SIX1;RORB;JPH1;LITAF;PITPNC1;ELAVL2;ADAMTS5;MED14
KIAA0141;MRFAP1;TNFAIP3;SIX1;NUDT5;PPP1R9A;SMG7;PITPNC1;PHF8;PCMT1;SH3BGRL2;MAP3K9;SN
OTUD4;RAB3C;TNFAIP8;PPWD1;HNRNPU;NUDT5;PITPNC1;TIAL1;EFR3A;TRPS1;SOSTDC1;SLC25A45;ME
FAM49A;ANKRD13B;ONECUT2;TTC22;MAML1;CELF3;LTN1;KIAA0141;BMI1;TXNDC17;ING4;TIAL1;DTWD1;
NCKAP1;UHRF1BP1L;BTG1;TMEM167B;ETFA;FMN1;BMI1;MED17;ING4;ARL5B;UBL3;KIF5C;SIN3A;PAPOL
GPR27;TNFAIP8;TTC22;DIRAS2;KLHL32;KIAA0141;IRS4;ZBTB20;PPWD1;ELAVL2;NHSL2;NKRF;SIN3A;SH
HRK;USP37;BTG1;CELF3;MRFAP1;RSF1;BACH1;MED17;IL18BP;MED14;SH3PXD2A;DPYSL5;NKRF;MAN1
NRP2;TTC22;MAML1;KLHL32;KIAA0141;ADK;PPP1R9A;IKZF3;ALAD;DTWD1;ARL5B;MAN1A2;RUVBL1;SH3
HRK;RAB3C;NPFFR1;ATP8A2;ANKRD33B;ONECUT2;KCNC2;FRMPD4;DIRAS2;KLHL32;CLDN1;LITAF;CDH
OTUD4;C9ORF91;RBM28;TCERG1;CHURC1-FNTB;LTN1;ADK;PHAX;RSF1;ANTXR2;LITAF;PFAS;CKS1B;EF
GABPB2;ZNF292;LTN1;KIAA0141;MRFAP1;ZBTB20;NUDT5;TM7SF3;FAM204A;AMOT;CDH6;PCMT1;CDH2;
RAB3C;GABPB2;KLHL32;KIAA0141;ZBTB20;LOXL4;RORB;CLDN1;TXNDC17;EPS8;RIMS3;GJA3;IGLON5;K
TDRKH;DYRK3;ATP8A2;CALML4;ANTXR2;MED17;ING4;CDH6;TIAL1;PCMT1;ADAMTS5;CDH2;SH3PXD2A;
HRK;CREBZF;DYRK3;BMPR2;ANKRD33B;TMEM167B;DIRAS2;KIAA0141;IRS4;ZBTB20;PPWD1;HNRNPU;J
COX7B;TTC22;MAML1;PHF20;HNRNPR;TIAL1;ARL5B;CAPZB;CDH2;FADS6;KIAA0895;C1QBP;FBXO3;KIF1
TDRKH;GABPB2;CALCOCO2;PDE3B;MRFAP1;TNFAIP3;PHAX;BACH1;NUDT4;EFR3A;UBL3;SIPA1L1;FAM7
NCKAP1;NRP2;TNFAIP8;ATP8A2;LTN1;RCSD1;BMI1;CLDN1;BACH1;CTGF;NUDT4;TIAL1;MRPL42;SPRED1
NCKAP1;MOCS3;DIRAS2;KIAA0141;ETFA;RCSD1;IKZF3;MED17;TXNDC17;RBM3;XPO4;ALDH2;MB;PSD4;
RAB3C;SCOC;MAML1;PRDM6;AMOT;CDH2;SALL4;PCDHAC2;KPNA1;PCDHAC1;EOMES;CXADR;ST6GAL2
RAB3C;UHRF1BP1L;NPFFR1;TMEM167B;ONECUT2;PCDH11Y;HPSE2;TTC22;PHF20;SLC7A14;KLHL32;FM
DYRK3;TNFAIP8;SH3KBP1;KIAA0141;ZBTB20;PPWD1;RSF1;TXNDC17;AMOT;ALAD;DTWD1;ARL5B;NKRF;
FAM49A;RBM28;PHF20;ZBTB20;RCSD1;NUDT5;TXNDC17;ING4;PCMT1;DTWD1;ARL5B;SERP1;CAPZB;PA
GPR27;DIRAS2;TNFAIP3;BMI1;DTWD1;ARL5B;RPS6KA6;RIMS3;NKRF;SH3BGRL2;FBXO3;EPC1;PTGFRN;
TDRKH;NCKAP1;DYRK3;ATP8A2;TTC22;KLHL32;RORB;CALML4;NUDT5;ANTXR2;CLDN1;ELAVL2;ING4;TIA
GABPB2;PRDM6;KLHL32;RORB;NUDT5;ANTXR2;CLDN1;TXNDC17;TIAL1;PCMT1;EFEMP1;CDH2;KIF5C;F
GPR27;DYRK3;ANKRD33B;KLHL32;LOXL4;RCSD1;CLDN1;MED17;ING4;MRPL42;EFEMP1;SART3;DPYSL5
GPR27;KCNC2;FRMPD4;DIRAS2;KLHL32;RSF1;RCSD1;IKZF3;ELK4;GJA3;DPYSL5;FADS6;MAN1A2;PGM3;
TNFAIP8;GABPB2;SH3KBP1;ZNF292;LTN1;KIAA0141;PTPRM;PHAX;CALML4;TM7SF3;MYPN;CTGF;TXNDC
NRP2;DYRK3;GABPB2;NUDT5;BMI1;IKZF3;PFAS;MED17;ING4;TIAL1;MRPL42;PCMT1;ALDH2;TRPS1;FNTE
TNFAIP8;GABPB2;TTC22;KIAA0141;DUSP19;ZBTB20;PPWD1;PHAX;ELAVL2;ING4;SDR16C5;CAPZB;SNRP
OTUD4;TCERG1;COX7B;MOCS3;SLC7A14;MRFAP1;SIX1;AFF4;CDC73;PITPNC1;DCAF7;CTGF;PCMT1;JPH
GPR27;NCKAP1;GABPB2;HNRNPR;NUDT5;PITPNC1;MED17;ING4;TIAL1;PCMT1;SH3PXD2A;ALDH2;TRIM

TNFAIP8;KIAA0141;PPWD1;TM7SF3;CLDN1;AFF1;TXNDC17;AMOT;ARL5B;RPS6KA6;NKRF;DPYSL3;SH3B
TDRKH;ATP8A2;DENND5B;KLHL32;PHAX;RORB;LDLRAD4;ANTXR2;IKZF3;SESN3;DPYSL5;KIF5C;RAVER2
RBM28;COX7B;UHRF1BP1L;TMEM167B;ZNF292;KIAA0141;MRFAP1;LOXL4;DCAF7;MED17;MRPL42;KIAA1
TNFAIP8;GABPB2;LTN1;KIAA0141;ZBTB20;PPWD1;FMN1;LITAF;ING4;ARL5B;SH3PXD2A;FAM73A;PIM3;AP
NCKAP1;HNRNPU;LDLRAD4;TXNDC17;DPYSL5;DAG1;BSN;MEF2A;PRKAB2;ALG6;RNASE6;HPCAL4;RUN
TRIM24;EPT1;HCFC2;HIPK1;PCDHA8
NCKAP1;TMEM167B;PHAX;CCND3;DAG1;EPC1;PELO;TGM2;MEF2A;CMC1;FNDC3A;FOXP2;MOB3C;CDIP
C9ORF91;CCDC71L;RBM28;TCERG1;ALAS2;TNFAIP8;NPFFR1;MOCS3;CHURC1-FNTB;POGK;ADK;SIX1;P
GPR27;NRP2;TNFAIP8;ATP8A2;SH3KBP1;KLHL32;KIAA0141;ZBTB20;PPWD1;FMN1;RORB;RCSD1;JPH1;N
TDRKH;DENND5B;PHF20;CHURC1-FNTB;SNAP23;FRMPD2;PDE3B;KLHL32;LOXL4;PHAX;C1ORF213;TM7
TDRKH;USP37;GABPB2;SLC7A14;TNFAIP3;NUDT5;ANTXR2;CLDN1;ELAVL2;MED17;TXNDC17;ING4;CDH6
TDRKH;ATP8A2;NUDT5;ANTXR2;CLDN1;MED17;CDH6;TIAL1;PCMT1;ADAMTS5;CCND3;EFEMP1;FADS6;T
ANKRD13B;GABPB2;ATP8A2;TMEM167B;IRS4;MRFAP1;LOXL4;CCND3;SESN3;EPC1;SNIP1;PTGFRN;SNR
CCDC71L;FAM49A;ANKRD13B;UHRF1BP1L;IRS4;LOXL4;PHAX;ANTXR2;IKZF3;AMOT;ELK4;EFEMP1;DPYS
NCKAP1;PDE3B;MRFAP1;CALML4;MED17;ING4;TIAL1;EFR3A;CCND3;UBL3;FAM154B;FAM73A;SH3BGRL2
HRK;DENND5B;KLHL32;LOXL4;PHAX;PPP1R9A;AFF1;MED17;JPH3;DPYSL5;DPYSL3;PGM3;SH3BGRL2;KI
CREBZF;DOCK5;SCOC;ALAS2;COX7B;HPSE2;TTC22;CALCOCO2;DIRAS2;LDLRAD4;RCSD1;ANTXR2;LITA
HRK;C9ORF91;ATP8A2;HPSE2;CALCOCO2;BMI1;HK2;ARL5B;CCND3;JPH3;GJA3;DPYSL5;SIN3A;RSPO2;
HRK;ATP8A2;ONECUT2;SH3KBP1;LTN1;PHAX;BMI1;C1ORF213;CDH6;EFR3A;SART3;SERP1;GJA3;SH3PX
DOCK5;BTG1;PHF20;PPWD1;ETFA;RND3;PFAS;MED17;TXNDC17;FAM107B;ING4;ARL5B;SERP1;DDI2;RU
DDX19B;PCDHA1;SUB1;PCDHB15;PCDHA3;PCDHA2
SCOC;DYRK3;GABPB2;TM7SF3;JPH1;ING4;ARL5B;RPS6KA6;MECOM;NKRF;TMEM194B;USP2;C3ORF62;
HRK;NRP2;CELF1;PRDM6;LTN1;KIAA0141;IKZF3;RND3;DCAF7;ARL5B;SERP1;CDH2;NKRF;PAPOLG;SNIP
TDRKH;DYRK3;MAML1;KIAA0141;ZBTB20;ETFA;TM7SF3;CLDN1;JPH1;ALAD;NHSL2;SERP1;SNRPD3;UBL
FAM49A;RAB3C;DYRK3;ANKRD33B;HPSE2;CELF3;KLHL32;IRS4;PTPRM;SIX1;RCSD1;BMI1;IKZF3;CCND3;
HRK;GPR27;FAM49A;COX7B;ANKRD33B;FRMPD4;DIRAS2;KLHL32;KIAA0141;IRS4;ZBTB20;BMI1;C1ORF2
GPR27;DIRAS2;KIAA0141;ZBTB20;PPWD1;C1ORF213;CLDN1;JPH1;MYPN;PITPNC1;ELAVL2;ARL5B;CDH2
FAM49A;DYRK3;KCNC2;FRMPD4;DIRAS2;KLHL32;KIAA0141;ADK;IRS4;ZBTB20;JPH1;ALAD;RIMS3;RAVER
PRDM8;FAM49A;RAB3C;NPFFR1;FRMPD4;LOXL4;RORB;FAM107B;GJA3;CDH2;IGLON5;DPYSL3;SH3GL2;
DYRK3;MAML1;KLHL32;KIAA0141;ZBTB20;LOXL4;NUDT5;TM7SF3;DTWD1;MED14;SPRED1;XPO4;TRIM3;
DOCK5;DYRK3;BMPR2;TMEM167B;PRR3;POGK;PPWD1;LOXL4;HNRNPR;EPS8;SPRED1;SART3;XPO4;NK
PRDM8;DYRK3;ATP8A2;SNAP23;ETFA;PPP1R9A;LITAF;MYPN;IL18BP;PHF8;AMOT;ADAMTS5;NHSL2;DPY
UHRF1BP1L;ANKRD33B;MAML1;PPWD1;RORB;CALML4;NUDT5;JPH1;IKZF3;ALAD;KIAA1549;SERP1;FADS
NCKAP1;UHRF1BP1L;PRR3;SNAP23;KLHL32;PPWD1;SMG7;ARL5B;SIPA1L1;NKRF;FADS6;SIN3A;SLC22A
CBX5;SP1;SUB1;HNRNPU;SMNDC1;CGGBP1;PSPH
C9ORF91;TNFAIP8;TMEM167B;KIAA0141;PHAX;FMN1;RSF1;C1ORF213;TXNDC17;ALAD;ING4;DTWD1;PS
RAB3C;DYRK3;GABRB1;UHRF1BP1L;FRMPD4;SNAP23;DIRAS2;FMN1;LITAF;IKZF3;PFAS;MYPN;HK2;PHF
TCERG1;USP37;BTG1;MOCS3;PDE3B;HNRNPU;CTDSPL2;CDC73;IL18BP;NUDT4;FAM107B;TIAL1;FBXO3;
DYRK3;TNFAIP8;ATP8A2;DIRAS2;KLHL32;KIAA0141;PPWD1;C1ORF213;TM7SF3;CLDN1;IKZF3;PITPNC1;A
KLHL32;KIAA0141;IRS4;CALML4;TM7SF3;CLDN1;DTWD1;NHSL2;KIAA1549;ARL5B;RPS6KA6;RIMS3;FADS
RAB3C;NPFFR1;SNAP23;DUSP19;FMN1;RORB;ANTXR2;IKZF3;ELK4;MRPL42;NHSL2;MECOM;XPO4;MB;S
CCDC71L;SCOC;DYRK3;UHRF1BP1L;GABPB2;TMEM167B;CELF1;CHURC1-FNTB;KLHL32;PHAX;FMN1;LI
PRDM8;DYRK3;GABPB2;BMPR2;SH3KBP1;DUSP19;MRFAP1;ZBTB20;TNFAIP3;RSF1;CTDSPL2;NUDT5;A
GPR27;FAM49A;DYRK3;ATP8A2;TTC22;DIRAS2;KLHL32;KIAA0141;ADK;IRS4;ZBTB20;JPH1;AMOT;NHSL2;
TDRKH;TMEM167B;IRS4;MRFAP1;LOXL4;PHAX;LDLRAD4;ANTXR2;CDH2;FADS6;SNIP1;PTGFRN;AGFG2;
NCKAP1;POGK;PHAX;DCAF7;FAM107B;TIAL1;PCMT1;CCND3;FADS6;DAG1;CHP1;PELO;RNF111;PSPH;TG
TNFAIP8;GABPB2;LTN1;KIAA0141;ADK;CTDSPL2;LITAF;PFAS;PITPNC1;DCAF7;TXNDC17;DTWD1;ARL5B;
CCDC71L;RAB3C;CELF1;TTC22;PPWD1;LOXL4;PHAX;RCSD1;ELAVL2;HK2;CTGF;PHF8;ELK4;CDH6;EFEM
GPR27;TNFAIP8;ATP8A2;KLHL32;KIAA0141;ZBTB20;PPWD1;RCSD1;JPH1;ELAVL2;RIMS3;SH3BGRL2;SCN
NRP2;UHRF1BP1L;TTC22;KIAA0141;ZBTB20;PPP1R9A;PITPNC1;ALAD;ING4;ARL5B;RPS6KA6;ALDH2;RU
FAM49A;DYRK3;FRMPD4;SH3KBP1;CELF3;DIRAS2;KLHL32;KIAA0141;PPWD1;TNFAIP3;RCSD1;IKZF3;SES
OTUD4;DENND5B;FRMPD2;KLHL32;KIAA0141;ZBTB20;NUDT5;C1ORF213;JPH1;IKZF3;CKS1B;DTWD1;KIA
GPR27;DYRK3;TNFAIP8;SH3KBP1;KLHL32;KIAA0141;ZBTB20;PPWD1;FMN1;C1ORF213;CLDN1;BACH1;N

TNFAIP8;KIAA0141;ADK;KIAA1671;ZBTB20;RSF1;TM7SF3;FAM204A;AFF1;AMOT;ALAD;ING4;ARL5B;RUVB
CHRM3;NPFFR1;MOCS3;TMEM167B;PPWD1;SIX1;CALML4;C1ORF213;BACH1;CKS1B;TIAL1;RBM3;ADAM
FAM49A;RAB3C;GABRB1;ATP8A2;ONECUT2;KCNC2;DIRAS2;KLHL32;RORB;IKZF3;ELAVL2;LMOD3;SDR16
ANKRD13B;NPFFR1;ATP8A2;DENND5B;FRMPD4;PHF20;DIRAS2;SLC7A14;RCSD1;BMI1;TM7SF3;JPH1;IKZ
PRDM8;GABRB1;NPFFR1;FRMPD4;PRDM6;DIRAS2;IRS4;PTPRM;LOXL4;RORB;CDH6;ALDH2;DPYSL5;DP
C9ORF91;COX7B;ZNF292;LTN1;KIAA0141;TNFAIP3;PPP1R9A;ELAVL2;ING4;ARL5B;RUVBL1;ZNF800;PTGF
HRK;PRDM8;ATP8A2;PRDM6;KLHL32;IRS4;LOXL4;CLDN1;IKZF3;PITPNC1;FAM107B;CDH6;SDR16C5;UBL
GPR27;DOCK5;MOCS3;SNAP23;KLHL32;KIAA0141;ADK;IRS4;FMN1;NUDT5;CLDN1;JPH1;ELAVL2;TXNDC1
C9ORF91;NCKAP1;DOCK5;DYRK3;USP37;MOCS3;LTN1;PHAX;FMN1;TXNDC17;PHF8;TIAL1;MED14;ALDH
PRDM8;RBM28;HPSE2;CHURC1-FNTB;SNAP23;LOXL4;FMN1;LITAF;IKZF3;MYPN;FAM204A;PHF8;ELK4;RE
TNFAIP8;TTC22;MAML1;LTN1;KIAA0141;ZBTB20;PPWD1;RSF1;ELAVL2;ING4;DTWD1;MED14;ARL5B;TRPS
NCKAP1;DYRK3;DIRAS2;KLHL32;PHAX;CALML4;C1ORF213;PHF8;SPRED1;GJA3;CDH2;DPYSL5;FADS6;T
HRK;PRDM8;DOCK5;TNFAIP8;ATP8A2;CELF3;TNFAIP3;PHAX;RCSD1;BMI1;LMOD3;FAM107B;AMOT;DPYS
ATP8A2;SLC7A14;NUDT5;IKZF3;ELAVL2;HK2;MED17;ING4;MRPL42;PCMT1;ADAMTS5;SH3PXD2A;DPYSL
HRK;ATP8A2;DIRAS2;KIAA0141;PPWD1;LOXL4;JPH1;ELAVL2;TXNDC17;NHSL2;KIAA1549;NKRF;SH3BGRL
NCKAP1;GABPB2;ATP8A2;RORB;NUDT5;ANTXR2;TIAL1;PCMT1;EFEMP1;FADS6;KIF5C;FNTB;HOXA3;SEC
KLF10;SF3B3;CBX5;MAT2A;MAML1;CSNK1A1;SP1;CNBP;SUB1;SRSF1;HNRNPU;AZIN1
NCKAP1;UHRF1BP1L;PRR3;POGK;ADK;MRFAP1;TNFAIP3;ANTXR2;FAM107B;CKS1B;PCMT1;CCND3;SES
CCDC71L;USP37;ANKRD33B;TMEM167B;CELF1;MAML1;CALCOCO2;CTDSPL2;CKS1B;SART3;SLC22A17;
CCDC71L;RBM28;ANKRD13B;DOCK5;SCOC;PHF20;POGK;CELF3;KLHL32;RCSD1;AMOT;ELK4;ARL5B;SAF
GPR27;CCDC71L;FAM49A;NPFFR1;ONECUT2;SLC7A14;IRS4;C1ORF213;ANTXR2;LITAF;IKZF3;HK2;FAM1
DOCK5;TNFAIP8;KLHL32;KIAA0141;ADK;CTDSPL2;CDC73;TIAL1;SERP1;CAPZB;GJA3;FADS6;DDI2;TMEM
NCKAP1;HPSE2;TTC22;KLHL32;ADK;LOXL4;TNFAIP3;LDLRAD4;ARL5B;CCND3;SPRED1;DPYSL5;MAN1A2
BMPR2;DENND5B;CALCOCO2;KIAA0141;ADK;JPH1;DCAF7;MED17;PCMT1;CCND3;ADAMTS2;KIAA0895;F
DYRK3;GABPB2;MAML1;FRMPD2;ZBTB20;LITAF;DTWD1;SERP1;KIF1B;MAP3K9;UBL7;VKORC1;MAPK1IP1
RAB3C;DYRK3;MOCS3;TTC22;SNAP23;IRS4;LOXL4;PHAX;PHF8;ELK4;MECOM;GJA3;ALDH2;MFSD5;TRIM
TDRKH;ATP8A2;KLHL32;FMN1;CALML4;LMOD3;IL18BP;EFEMP1;GJA3;FADS6;MB;KIF5C;PSD3;HOXA3;TM
CCDC71L;GABPB2;CELF1;MAML1;PPWD1;SMG7;ANTXR2;GLS;SART3;SLC22A17;PAPOLG;SLC25A45;AP2
RAB3C;CALCOCO2;DIRAS2;DUSP19;PPP1R9A;LITAF;IKZF3;HK2;IL18BP;PHF8;ELK4;MRPL42;ADAMTS5;M
FAM49A;RAB3C;ANKRD33B;DIRAS2;KLHL32;LITAF;IKZF3;CDC73;LMOD3;SDR16C5;KIAA1549;CCND3;JPH
MOCS3;LTN1;KIAA0141;ZBTB20;BMI1;TM7SF3;PITPNC1;NUDT4;EPS8;ING4;DTWD1;ARL5B;NKRF;C1QBP;
CCDC71L;GABPB2;MAML1;KIAA1671;PPWD1;ANTXR2;ADAMTS5;ADAMTS2;SART3;SIPA1L1;SH3PXD2A;S
PRDM8;CHRM3;NRP2;ATP8A2;TTC22;CALCOCO2;CELF3;TNFAIP3;RCSD1;RND3;HK2;ADAMTS5;CCND3;J
CCDC71L;TMEM167B;MAML1;AMOT;ELK4;FNTB;FBXO3;CHP1;SNRPD3;SLC25A45;SEC62;BTRC;AP2M1;A
GPR27;CCDC71L;CREBZF;RAB3C;NPFFR1;MOCS3;PHF20;C1ORF213;PPP1R9A;SMG7;IKZF3;PITPNC1;A
GPR27;OTUD4;DYRK3;TNFAIP8;ATP8A2;FRMPD4;KIAA0141;TM7SF3;CLDN1;NHSL2;ALDH2;MAP3K9;UBL
TNFAIP8;GABPB2;SNAP23;KIAA0141;ZBTB20;PPWD1;PHAX;LITAF;ING4;MB;CNPPD1;TSFM;DFFA;TMEM1
TDRKH;TNFAIP8;GABPB2;POGK;DIRAS2;LOXL4;PHAX;CALML4;NUDT5;IL18BP;DTWD1;NHSL2;DPYSL5;N
C9ORF91;CALCOCO2;SIX1;FMN1;CLDN1;JPH1;BACH1;IL18BP;CTGF;ALAD;DTWD1;MED14;CCND3;SART
HRK;CCDC71L;HPSE2;KCNC2;KLHL32;SIX1;PHAX;CALML4;LITAF;AFF4;ELAVL2;CCND3;EFEMP1;ALDH2;F
ANKRD13B;TMEM167B;MAML1;MRFAP1;RORB;LDLRAD4;EFR3A;SESN3;DPYSL5;FADS6;PAPOLG;FNTB;S
TDRKH;GABPB2;DENND5B;PDE3B;KLHL32;KIAA0141;ZBTB20;NUDT5;BMI1;C1ORF213;IKZF3;TXNDC17;C
PRDM8;ANKRD13B;GABRB1;TTC22;FRMPD4;SNAP23;SLC7A14;CALML4;ANTXR2;JPH1;PFAS;PHF8;ELK4
RAB3C;TTC22;PRDM6;CHURC1-FNTB;SNAP23;DUSP19;PPP1R9A;LITAF;IKZF3;ELAVL2;HK2;PHF8;SLC22
RAB3C;TTC22;KLHL32;KIAA0141;PPWD1;LOXL4;C1ORF213;CLDN1;RND3;RPS6KA6;RIMS3;NKRF;FADS6;
RAB3C;DIRAS2;DUSP19;PPWD1;ETFA;PHAX;FMN1;CALML4;ANTXR2;CLDN1;EPS8;ELK4;MECOM;TRIM3;
PRDM8;CHRM3;TCERG1;TNFAIP8;PHF20;SLC7A14;PTPRM;C1ORF213;LITAF;IKZF3;PITPNC1;CTGF;SIPA1
CCDC71L;MAML1;SNAP23;KLHL32;KIAA0141;PPWD1;BMI1;C1ORF213;ELK4;CCND3;TRPS1;EPC1;ZNF367
GPR27;TTC22;FRMPD4;PRDM6;KLHL32;KIAA0141;IRS4;ZBTB20;RCSD1;IKZF3;RIMS3;GJA3;DPYSL5;FADS
SCOC;NPFFR1;ATP8A2;ANKRD33B;KCNC2;PHF20;PRDM6;CELF3;SLC7A14;FMN1;CALML4;C1ORF213;IK
TMEM167B;MAML1;ZNF292;MRFAP1;NUDT4;CCND3;FNTB;PSPH;FBXW4;MAP2K1;MBNL2;C5ORF15;TMO
CREBZF;COX7B;GABPB2;MOCS3;TMEM167B;MAML1;AFF4;HK2;IL18BP;CTGF;ING4;RBM3;SART3;SLC22
KLHL32;KIAA0141;PPWD1;PHAX;CALML4;BMI1;C1ORF213;TXNDC17;ALAD;ING4;BSN;UNKL;CNPPD1;APF

HRK;C9ORF91;NCKAP1;NRP2;TMEM167B;CALCOCO2;ADK;MRFAP1;PHAX;C1ORF213;PITPNC1;ALAD;RB
HRK;DYRK3;DENND5B;KLHL32;KIAA0141;ADK;ZBTB20;NUDT5;C1ORF213;IKZF3;TXNDC17;CKS1B;RAVEF
PRDM8;GABRB1;ANKRD33B;KCNC2;KLHL32;LOXL4;CALML4;LMOD3;CCND3;JPH3;ALDH2;SALL4;PSD3;H
SH3KBP1;PRDM6;CHURC1-FNTB;SNAP23;KIAA0141;LOXL4;HNRNPU;SIX1;ETFA;PHAX;TIAL1;MRPL42;PC
TMEM167B;MAML1;GLS;TIAL1;EFR3A;CCND3;PAPOLG;CHP1;SLC25A45;BTRC;AP2M1;AGFG2;PSPH;FNB
OTUD4;RAB3C;UHRF1BP1L;KLHL32;CALML4;IKZF3;TXNDC17;ELK4;EFR3A;RUVBL1;FNTB;DIP2B;SLC25A
DENND5B;KLHL32;RORB;LDLRAD4;CALML4;CTGF;CDH6;EFEMP1;SESN3;GJA3;CDH2;DPYSL5;KIF5C;IGL
CREBZF;RBM28;COX7B;TNFAIP8;MAML1;PPWD1;CTDSPL2;C1ORF213;TM7SF3;ELAVL2;MB;PGM3;UBXN
TDRKH;CCDC71L;TNFAIP8;POGK;DIRAS2;KLHL32;LOXL4;CALML4;RCSD1;CKS1B;NHSL2;GJA3;KIF5C;SIN
GABPB2;TMEM167B;MAML1;CHURC1-FNTB;KIAA1671;PPWD1;PTPRM;C1ORF213;ANTXR2;TIAL1;JPH3;S
GABPB2;MAML1;POGK;MRFAP1;HNRNPU;NUDT4;PAPOLG;FNTB;FBXO3;SNIP1;SLC25A45;RNF111;PSPH
MOCS3;MAML1;KIAA0141;PPWD1;PTPRM;CALML4;RCSD1;ANTXR2;BACH1;IL18BP;GLS;ALAD;KIAA1549;
FAM49A;TMEM167B;MAML1;PPWD1;RCSD1;NUDT5;ANTXR2;HK2;GLS;SART3;PAPOLG;C1QBP;PIM3;SNR
CCDC71L;SCOC;ALAS2;RORB;LMOD3;EFR3A;SESN3;NKRF;FADS6;PAPOLG;PSD4;PSD3;PGM3;FBXO3;SI
CREBZF;RBM3;KIAA0141;PIM3;TARDBP;DYNLL2;CTGF
PRDM8;TNFAIP8;ATP8A2;LDLRAD4;PFAS;IL18BP;ING4;C1QBP;KIF1B;SLC25A45;PELO;HSDL1;TMED8;SG
ANKRD13B;HPSE2;POGK;MRFAP1;LDLRAD4;NUDT4;EFR3A;DPYSL5;FADS6;PAPOLG;HOXA3;FBXO3;MA
TMEM167B;MAML1;GLS;FAM107B;ELK4;EFR3A;SLC25A45;BTRC;AP2M1;AGFG2;PSPH;KPNA1;FNBP4;FB
YOD1;CTGF
PRDM8;PHF20;SNAP23;ETFA;PPP1R9A;FAM204A;PHF8;ELK4;RBM3;EFR3A;UBL3;MECOM;MFSD5;DPYSL
MAML1;KIAA0141;MRFAP1;PPWD1;SMG7;ANTXR2;BACH1;HK2;CTGF;GLS;RBM3;ARL5B;SART3;SIPA1L1;
SCOC;MAML1;MRFAP1;BACH1;DCAF7;GLS;TIAL1;EPC1;SLC25A45;AP2M1;PSPH;FBXW4;CSNK1G3;TMEN
TDRKH;NRP2;PRDM6;ETFA;LMOD3;PHF8;ELK4;UBL3;SIPA1L1;MFSD5;DPYSL3;FNTB;TRIM3;RSPO2;EMB;
PRDM8;TNFAIP8;TMEM167B;ONECUT2;POGK;KLHL32;ZBTB20;IKZF3;ING4;SALL4;RAVER2;DPYSL3;TRIM
USP37;MOCS3;KIAA0141;KIAA1671;HNRNPU;PHAX;HNRNPR;CALML4;MRPL42;RUVBL1;HOXA3;PIM3;SN
TNFAIP8;C1ORF213;C1ORF52;PCDHA1;SNIP1;PCDHA4;PCDHA3;PCDHA2;MACROD2;SEC62;EIF4E;SNX8
TDRKH;POGK;ATXN1L;CMC1;EPT1;TRIM24;ALG14;PARVA;RBM12;PCDHA8;DYNLL2;FKBP6
TMEM167B;SNAP23;DUSP19;NUDT5;C1ORF213;IL18BP;PHF8;ALAD;ING4;RBM3;ADAMTS5;SDR16C5;SEF
HRK;NCKAP1;ANKRD13B;USP37;TMEM167B;DENND5B;TTC22;BMI1;RND3;PITPNC1;GLS;CCND3;ADAMT
TMEM167B;MAML1;KIAA0141;MRFAP1;BACH1;FAM204A;TIAL1;AP2M1;TMEM194B;RC3H1;YOD1;GAPVD1
NPFFR1;ATP8A2;HPSE2;DENND5B;PRDM6;FRMPD2;LTN1;IKZF3;FAM107B;AMOT;EFEMP1;MAP3K9;PELO
GABPB2;BMPR2;KIAA0141;ZBTB20;C1ORF213;CCND3;SIN3A;RUVBL1;PIM3;SNRPD3;SLC25A45;UNKL;AG
ANKRD33B;TMEM167B;MAML1;KIAA1671;MRFAP1;PPWD1;PTPRM;SMG7;ANTXR2;PFAS;HK2;CKS1B;RBN
DOCK5;TNFAIP8;NPFFR1;ANKRD33B;TTC22;SH3KBP1;POGK;CALML4;IL18BP;CCND3;GRM5;EFEMP1;ALI
OTUD4;COX7B;MOCS3;TMEM167B;MAML1;PRR3;MRFAP1;ZBTB20;PHAX;IL18BP;ING4;RBM3;ALDH2;MFS
UHRF1BP1L;TMEM167B;MAML1;CCND3;PAPOLG;CHP1;SNRPD3;SLC25A45;AP2M1;AGFG2;PSPH;FNBP4;
BTG1;MOCS3;TMEM167B;LTN1;MRFAP1;AFF1;IL18BP;FAM107B;ING4;RBM3;SART3;C1QBP;PGM3;SNIP1;
FAM49A;CHRM3;FRMPD4;DIRAS2;RORB;C1ORF213;CDH6;DTWD1;PIM3;MACROD2;SEC62;PCDHAC1;ME
TDRKH;FAM49A;NCKAP1;SCOC;USP37;HPSE2;CALCOCO2;PDE3B;MRFAP1;LDLRAD4;BMI1;IKZF3;MYPN;
TENM3;ONECUT2;KCNC2;SH3KBP1;POGK;IRS4;SIX1;LDLRAD4;IKZF3;PHF8;ING4;RBM3;NHSL2;SH3PXD2
CCDC71L;TMEM167B;MAML1;PPWD1;SMG7;ANTXR2;PFAS;HK2;PAPOLG;FAM154B;SLC25A45;CNPPD1;A
CREBZF;SCOC;GABPB2;ANKRD33B;TMEM167B;PRDM6;FMN1;C1ORF213;PFAS;CTGF;SART3;NKRF;HOX
CHRM3;POGK;RSF1;LITAF;ELAVL2;TIAL1;ADAMTS5;EEF2K;SLC22A17;SOSTDC1;EMB;MACROD2;SH3GL2
BHLHB9;GABPB2;NECAB3;TMEM167B;SRSF1;FUT11;AGAP2;HNRNPU;IL18BP;NCKIPSD;RBM3;NXF1;EFN
COX7B;DCUN1D5;FUT11;IL18BP;CTGF;NUDT4;AMOT;NCKIPSD;RBM3;EFNB3;MAT2A;PABPN1;MFSD5;PIM
CREBZF;BTG1;TMEM167B;ZBTB20;RCSD1;IL18BP;RBM3;SERP1;MFSD5;C1QBP;FAM154B;SNIP1;CEP170
CHRM3;RAB3C;COX7B;NPFFR1;TMEM167B;ONECUT2;HPSE2;SH3KBP1;PRDM6;IRS4;PHAX;IKZF3;RND3
TNFAIP8;ATP8A2;DIRAS2;RCSD1;LMOD3;NUDT4;ALDH2;FADS6;SLC22A17;FBXO3;SOSTDC1;CEP170;UN
CHRM3;TMEM167B;PHF20;ETFA;C1ORF213;SMG7;BACH1;DTWD1;PIM3;MACROD2;SEC62;WLS;PCDHAC
PRDM8;NCKAP1;SCOC;IRS4;CTGF;CDH6;KIF5C;FRRS1L;IGLON5;TRIM24;SH3GL2;PCDHAC2;KPNA1;SCN
TDRKH;PRDM8;CHRM3;ATP8A2;HPSE2;ZBTB20;PHAX;CALML4;EFEMP1;DPYSL5;FADS6;KIF5C;SALL4;RA
TDRKH;PRDM8;CHRM3;UHRF1BP1L;NPFFR1;KCNC2;TTC22;PHF20;TM7SF3;ANTXR2;EFEMP1;FADS6;MB
CREBZF;BTG1;MOCS3;MAML1;POGK;KIAA0141;MRFAP1;HNRNPU;C1ORF213;CTGF;TXNDC17;RBM3;SL

C9ORF91;IRS4;PHAX;IKZF3;RND3;PITPNC1;AMOT;CDH6;NHSL2;ADAMTS2;MECOM;SNIP1;PIM3;MACROD
TDRKH;CREBZF;COX7B;MAML1;PFAS;CTGF;CKS1B;ING4;RBM3;SART3;CAPZB;C1QBP;FAM73A;PTGFRN
C9ORF91;ANKRD13B;BACH1;CTGF;CCND3;EEF2K;RIMS3;TNS1;MMP2;USP2;KIAA1462;CNPY1;SLC39A1
GABPB2;MOCS3;KIAA0141;KIAA1671;HNRNPU;CALML4;BACH1;HK2;IL18BP;PGM3;CNPPD1;PSPH;DFFA;
USP37;GABPB2;MAML1;PPWD1;BMI1;ANTXR2;PFAS;TIAL1;EPC1;SCN5A;UNKL;PSPH;KPNA1;DGCR2;VK
DOCK5;CALCOCO2;KIAA0141;C1ORF213;PFAS;CTGF;ING4;CCND3;SIN3A;FAM154B;CTBS;UNKL;CNPPD1
FAM49A;GABPB2;TMEM167B;MAML1;LTN1;PAPOLG;PIM3;SNRPD3;SLC25A45;BTRC;AP2M1;AGFG2;PSP
TDRKH;ATP8A2;POGK;IRS4;RORB;CALML4;IKZF3;DPYSL5;KIF5C;PAPOLG;FBXO3;MAP3K9;PTGFRN;SLC
CCDC71L;FAM49A;BTG1;MAML1;POGK;KIAA0141;KIAA1671;MRFAP1;PPWD1;SMG7;ANTXR2;BACH1;PFA
TDRKH;KIAA0141;PFAS;ING4;SIN3A;PSD4;FAM154B;CEP170;CTBS;AGFG2;C3ORF14;APPL1;UBL7;TSFM;
SNAP23;LTN1;PHAX;BMI1;LITAF;HK2;LMOD3;PHF8;ELK4;RBM3;NHSL2;ALDH2;SLC22A17;MFSD5;TMEM1
C9ORF91;TMEM167B;CALCOCO2;SNAP23;KIAA0141;KIAA1671;PPWD1;C1ORF213;PFAS;CTGF;PHF8;ALA
SCOC;NRP2;GABRB1;HPSE2;CALML4;IKZF3;CDH6;EFEMP1;SESN3;ALDH2;DPYSL5;SLC22A17;GNRHR;H
BTG1;ANKRD33B;TMEM167B;MAML1;POGK;PPWD1;SMG7;BACH1;HK2;RBM3;ARL5B;SART3;SIPA1L1;PA
CHRM3;DIRAS2;RORB;PITPNC1;CTGF;AMOT;CDH6;SDR16C5;ARL5B;ADAMTS2;MACROD2;SEC62;C3OR
CREBZF;BTG1;POGK;KIAA0141;MRFAP1;LITAF;RND3;CTGF;RBM3;CAPZB;PGM3;SNRPD3;GPRASP2;PSP
TDRKH;PRDM8;HPSE2;KCNC2;SIX1;FMN1;CALML4;IKZF3;ELAVL2;EFEMP1;RIMS3;ALDH2;FADS6;SALL4;
CCDC71L;ANKRD33B;SIX1;RORB;IKZF3;LMOD3;IL18BP;GJA3;KIF5C;FAM73A;HOXA3;CEP170;EMB;MACR
C9ORF91;PHF20;FMN1;RCSD1;C1ORF213;LITAF;PITPNC1;TIAL1;ADAMTS5;SART3;MB;KIF5C;SNIP1;KIF1
ANKRD13B;KIAA0141;PFAS;TXNDC17;ALAD;ING4;CCND3;SIN3A;FAM154B;CNPPD1;AGFG2;TGM2;ARHG
GABPB2;TTC22;POGK;KIAA0141;BMI1;C1ORF213;TXNDC17;CKS1B;ALAD;ALDH2;UNKL;CCDC112;UTP15;
TNFAIP8;GABPB2;KIAA0141;TM7SF3;ALAD;ING4;C1QBP;FAM154B;PIM3;UNKL;AGFG2;PHACTR4;UTP15;
OTUD4;CCDC71L;ALAS2;COX7B;ONECUT2;LTN1;PHAX;BACH1;HK2;MRPL42;NKRF;MB;MAN1A2;MFSD5;
PRDM8;ANKRD13B;KIAA0141;MRFAP1;C1ORF213;ALAD;ING4;DDI2;KIF1B;BSN;UBL7;CHST7;RNASE6;AL
RAB3C;NPFFR1;ANKRD33B;FRMPD4;CLDN1;LITAF;SDR16C5;GRM5;RIMS3;ALDH2;MAP3K9;PCDHAC2;T
PHLPP2;BTG1;NECAB3;IGSF3;MAML1;POGK;SRSF1;FUT11;KIAA0141;MRFAP1;CALML4;NCKIPSD;RBM3;
DYRK3;KIAA0141;PFAS;TXNDC17;ALAD;ELK4;ING4;CCND3;UNKL;APPL1;TGM2;UBL7;TSFM;C21ORF59;S
CREBZF;TMEM167B;MAML1;LTN1;IL18BP;TXNDC17;AMOT;RBM3;SERP1;MFSD5;PSD3;SNIP1;SLC25A45;
TDRKH;CALCOCO2;KIAA0141;PFAS;ALAD;ING4;SIN3A;PSD4;CEP170;UNKL;CNPPD1;C3ORF14;TGM2;UB
ANKRD33B;TMEM167B;FRMPD4;POGK;DIRAS2;SLC7A14;ETFA;ANTXR2;PITPNC1;ING4;EFEMP1;SNIP1;S
TDRKH;ANKRD33B;KIAA0141;PPWD1;PFAS;ALAD;ING4;UNKL;GPRASP2;UBL7;KLF10;ARHGEF10;TSFM;A
MAML1;PHF20;ZNF292;MRFAP1;PPWD1;HK2;IL18BP;SIPA1L1;MB;PAPOLG;SCN5A;AP2M1;KPNA1;KCMF1
CALCOCO2;KIAA0141;PPWD1;PFAS;ING4;RIMS3;SERP1;PGM3;CNPPD1;AGFG2;UBL7;FNBP4;TSFM;DFF
KIAA0141;LOXL4;C1ORF213;PFAS;ALAD;ING4;CTBS;AGFG2;ARHGEF10;TSFM;C21ORF59;SLC30A4;SOR
GABPB2;MOCS3;HNRNPU;IL18BP;ING4;RBM3;SLC22A17;MFSD5;C1QBP;SNIP1;RBM7;PELO;DGCR2;NAA
PRDM8;ANKRD13B;KCNC2;TTC22;TNFAIP3;SLC22A17;IGLON5;PSD4;HOXA3;FBXO3;SOSTDC1;SLC25A4
PHLPP2;TMEM167B;PHF20;KIAA0141;DUSP19;LOXL4;CALML4;UQCR10;C1ORF213;C1ORF52;ZNF25;TXN
BHLHB9;NECAB3;KIAA0141;UQCR10;C1ORF213;DTX4;ZBTB4;PFAS;PITPNC1;ING4;RNF214;APH1B;EFS;S
SET;BTG1;POGK;KIAA0141;MRFAP1;C1ORF52;RND3;BACH1;MED17;RBM3;EFS;SIPA1L1;TMEM203;MAT2
POGK;SRSF1;KIAA0141;MRFAP1;BACH1;CTGF;NCKIPSD;RBM3;PURA;ADAMTS2;SIPA1L1;CAPZB;PIM3;S
CREBZF;BTG1;CBX5;CSNK1A1;POGK;SRSF1;LAPTM5;RC3H1;YOD1;DYNLL2;BACH1;RBM3;HNRNPK;SIP
CREBZF;RBM3;MAT2A;SP1;MGAT5;SERBP1;SRSF1;TARDBP;CNPPD1

RPD3;MACROD2;SEC62;AGFG2;MEF2A;KCMF1;CSNK1G3;PRKAB2;FNDC3B;DYRK1A;ZBTB34;YOD1;FI
CMT1;EFR3A;PAPOLG;CHP1;PELO;RNF111;MEF2A;CXADR;RC3H1;FNDC3A;HCFC2;GTPBP4;FAM133E
XA3;SOX6;PCDHAC2;KCNH1;PCDHAC1;TNS1;EOMES;ST6GAL2;SLC30A4;EBF1;ATP11A;POU3F1;SHIS
ER2;HOXA3;BSN;SOX6;PCDHAC2;KCNH1;TNS1;EOMES;ST6GAL2;SFMBT2;TMOD2;VASH2;EBF1;ACSI
TBL1XR1;SUB1;ADAM12;CDIP1;TFAM;WDFY3;SRSF9;GLYR1;TBCEL;FAM114A1;IGSF3;DCUN1D5;YTHI
;PSD3;RSPO2;TMEM108;EPC1;SOSTDC1;PELO;PRKG1;CXADR;SFMBT2;TMOD2;GAB3;PROX1;FOXP
AVER2;SOX6;PCDHAC2;EOMES;ENTPD1;SFMBT2;TMOD2;VASH2;ACSL6;POU3F1;SHISA9;HPCAL4;F
TGFRN;SOX6;SH3GL2;PCDHAC2;KCNH1;TGM2;TNS1;EOMES;ST6GAL2;TMOD2;FNDC3B;VASH2;EBF
N;SH3GL2;KCNH1;PCDHAC1;EOMES;ST6GAL2;SFMBT2;TMOD2;VASH2;EBF1;CNPY1;POU3F1;SHISA
RF59;BROX;DYRK1A;PCDHA13;EREG;GPRIN3;MAF;CRISPLD2;HECW2;FAM185A;ADAM12;ELMO1;C
;MACROD2;PELO;PSPH;EOMES;KCMF1;TMEM194B;MAP2K1;CXADR;C21ORF59;TMOD2;DYRK1A;AR
PCDHAC2;KCNH1;IL13RA1;TNS1;CXADR;ST6GAL2;SFMBT2;TMOD2;FNDC3B;GPR75;SHISA9;EREG;S
6R;CSNK1G3;CXADR;C21ORF59;GPR75;ARID5B;PCDHA13;HCFC2;C9ORF64;PCDHA10;KIAA1614;HE
SH2;POU3F1;SHISA9;FOXP2;SFRP1;CRISPLD2;ADAM12;TRIB2;KANK4;KCTD16;DLX1;FAM114A1;DLX
PYSL3;MAP3K9;SH3GL2;PCDHAC2;WLS;KCNH1;SCN1A;IL13RA1;PCDHAC1;ENTPD1;CXADR;ST6GAL
4;TMEM194B;MAP2K1;ESCO1;ALG14;RUNX3;GTPBP4;FAM133B;CDC37;TCEANC2;WDFY3;AGTR2;CG
3;PELO;TGM2;IL13RA1;EOMES;KCMF1;CXADR;C21ORF59;BROX;DYRK1A;ATP11A;FNDC3A;POU3F1;
;KCNH1;TGM2;IL13RA1;PCDHAC1;TNS1;FBXW4;CXADR;ST6GAL2;SWAP70;SLC30A4;FNDC3B;YOD1;
3K9;PTGFRN;SNRPD3;PELO;IL6R;SH3GL2;TGM2;EOMES;KCMF1;TMEM194B;ST6GAL2;DYRK1A;GPR
L3;HOXA3;PIM3;MAP3K9;PTGFRN;MACROD2;SH3GL2;PCDHAC2;KPNA1;SCN1A;PCDHAC1;EOMES;S
;SH3GL2;RNF111;FBXW4;ST6GAL2;TMOD3;ALG14;PCDHA11;FAM133B;CDC37;TCEANC2;TBC1D25;W
;RAVER2;PIM3;PTGFRN;SNRPD3;PELO;SH3GL2;KCMF1;TMEM194B;BROX;DYRK1A;EBF1;PCDHA13;
CDH2;FADS6;TRIM24;BTRC;AGFG2;KCNH1;TNS1;MEF2A;RAB2B;PRKAB2;CXADR;ALG6;C21ORF59;U
PYSL5;HOXA3;PTGFRN;PCDHAC2;WLS;TNS1;EOMES;SWAP70;VASH2;POU3F1;PCDHA11;FOXP2;DK
S;CDH6;ADAMTS5;MED14;SPRED1;CDH2;SLC22A17;TRIM3;FAM73A;MAP3K9;SEC62;IL6R;RNF111;HS
AP3K9;PTGFRN;SNRPD3;PELO;IL6R;SH3GL2;KCMF1;RAB2B;TMEM194B;CXADR;ALG14;GPR75;FTSJ
A0895;TRIM24;BSN;IL6R;PCDHAC2;TGM2;PCDHAC1;EOMES;TMOD2;KIAA1462;HPCAL4;FOXP2;ADA
JA3;CDH2;KIAA0895;SNRPD3;PELO;IL6R;TMEM194B;CXADR;BROX;EREG;BCMO1;ADCY9;HECW2;FA
IP1;RIMS3;SESN3;GJA3;CDH2;KIAA0895;RAVER2;PIM3;MAP3K9;PTGFRN;SNRPD3;MACROD2;IL6R;S
IAA0895;RAVER2;PIM3;SNRPD3;ZNF687;IL6R;SH3GL2;KCMF1;TMEM194B;ST6GAL2;BROX;DYRK1A;G
SNRPD3;TMEM194B;CXADR;ST6GAL2;BROX;DYRK1A;YOD1;HECW2;FAM185A;ADAM12;ZNF318;WDF
;FBXW4;TMEM194B;SLC30A4;ACSL6;ALG14;FNDC3A;FAM133B;SUB1;TCEANC2;WDFY3;CGGBP1;YT
;PRKG1;ATOH1;MARK1;PAX5;PROX1;CDYL2;FOXP2;EFNA3;MAF;CRISPLD2;HECW2;KIF26B;FAM84A
94B;DYRK1A;FNDC3A;FTSJ2;RUNX1;ACAP2;ADAM12;CMPK1;TFAM;DENND6A;RAF1;BHLHB9;DCUN1
GAL2;ARID5B;EREG;BCMO1;FAM133B;KIAA1614;HECW2;FAM185A;WDFY3;CGGBP1;PPIC;PTGFR;PD
;BSN;SH3GL2;KCNH1;PCDHAC1;EOMES;CXADR;ST6GAL2;TPM3;SFMBT2;TMOD2;VASH2;ACSL6;CN
LG14;FNDC3A;FTSJ2;TBL1XR1;CDC37;CDIP1;TFAM;WDFY2;WDFY4;DCUN1D5;YTHDC1;UBA6;CUL3;
3;PELO;IL6R;SH3GL2;TMEM194B;ST6GAL2;DYRK1A;ARID5B;EREG;BCMO1;KIAA1614;HECW2;FAM1
D3;PELO;IL6R;SH3GL2;RAB2B;TMEM194B;ARID5B;C9ORF64;EREG;RUNX1;GPRIN3;KIAA1614;HECW
;TMEM194B;ST6GAL2;SWAP70;VASH2;EREG;BCMO1;KIAA1614;HECW2;FAM185A;ADAM12;ZNF318;S
BXW4;CSNK1G3;ENTPD1;TMOD3;SWAP70;CMC1;RNASE6;EBF1;YOD1;FNDC3A;CMPK1;DENND6A;SI
;CDH2;KIAA0895;RAVER2;SNRPD3;ZNF687;PELO;IL6R;TMEM194B;C9ORF64;EREG;BCMO1;TBL1XR1
PTGFRN;SNRPD3;SLC25A45;PELO;IL6R;EOMES;RAB2B;TMEM194B;SWAP70;PCDHA13;SHISA9;PCD
21ORF59;CMC1;ALG14;ARID5B;ZBTB34;FNDC3A;GAPVD1;FTSJ2;SYTL4;C9ORF64;GTPBP4;DHX40;AF
CFC2;C9ORF64;BCMO1;CDC37;ADAM12;CMPK1;COMMD3-BMI1;TFAM;WDFY3;ANKRD40;CGGBP1;GL
R75;ATP11A;PCDHA13;HCFC2;GTPBP4;FAM185A;COMMD3-BMI1;TFAM;ASPHD2;WDFY3;CGGBP1;TB
1;GJA3;CDH2;KIAA0895;RAVER2;MAP3K9;SNRPD3;PELO;IL6R;SH3GL2;KPNA1;EOMES;TMEM194B;M
5;RAVER2;SNRPD3;SLC25A45;PELO;SH3GL2;EOMES;TMEM194B;CXADR;ARID5B;FTSJ2;RBX1;MAF;
RN;SOX6;SH3GL2;KCNH1;PCDHAC1;ST6GAL2;TMOD2;DIO2;EBF1;ACSL6;ATP11A;POU3F1;SHISA9;H
AVER2;PIM3;PTGFRN;SNRPD3;SH3GL2;TMEM194B;BROX;DYRK1A;ARID5B;SHISA9;MAF;ADCY9;FAM
M107B;EPS8;CDH6;EFEMP1;SESN3;CDH2;DPYSL5;FADS6;IGLON5;RAVER2;DPYSL3;MACROD2;BSN;
DHAC2;KPNA1;SCN1A;TMEM194B;ALG14;FAM133B;CDC37;SUB1;TCEANC2;TFAM;WDFY3;SHC2;YTH

;AL2;ALG6;SHISA9;PCDHA11;GPRIN3;ADAM12;CDIP1;SCG3;WDFY2;DENND6A;FKBP6;PPIC;GLYR1;D
2;IL13RA1;FBXW4;ESCO1;ALG14;GTPBP4;FAM133B;GPRIN3;MOB3C;CDC37;WDFY3;KANK4;PFKFB3;
MEM194B;ST6GAL2;SFMBT2;BROX;EREG;ADCY9;CRISPLD2;HECW2;CGGBP1;PTGFR;GPM6A;PDE1B
NDC17;NUDT4;RIMS3;FADS6;SNRPD3;SLC25A45;ZNF687;SEC62;IL13RA1;TNS1;KCMF1;RAB2B;TMEM
TGFRN;SNRPD3;SH3GL2;TGM2;KCMF1;TMEM194B;CXADR;VASH2;HECW2;FAM185A;KCTD16;SHC4;
RAVER2;PIM3;SNRPD3;PELO;IL6R;EOMES;KCMF1;TMEM194B;BROX;DYRK1A;EBF1;C9ORF64;EREG;
)3;PELO;IL6R;SH3GL2;RAB2B;TMEM194B;BROX;GPR75;EREG;BCMO1;GPRIN3;HECW2;FAM185A;ZNI
)3;MACROD2;SH3GL2;C3ORF14;KCNH1;EOMES;SFMBT2;SLC30A4;ACSL6;CNPY1;FOXP2;DKK3;SFRP
NTB;PIM3;PTGFRN;RNF111;PSPH;KPNA1;TGM2;TMEM194B;MAP2K1;ESCO1;TMOD3;ALG14;YOD1;GA
1;KCMF1;RAB2B;TMEM194B;CXADR;SLC30A4;USP2;CMC1;VASH2;EBF1;CNPY1;HCFC2;PCDHA12;C9
S1L;RAVER2;MAP3K9;BSN;SOX6;SH3GL2;PCDHAC2;KCNH1;SCN1A;PCDHAC1;EOMES;CXADR;ST6G
FG2;PSPH;PCDHAC1;RAB2B;PRKAB2;C21ORF59;USP2;GPR75;YOD1;PCDHA13;HCFC2;PCDHA10;FA
WAP70;RBX1;SIK3;CMPK1;CDIP1;SLC25A51;TBCEL;ABHD4;DCUN1D5;YTHDC1;MTMR9;SPATA2;SLC1
M2;TMEM194B;ALG14;YOD1;ACTN4;HCFC2;RUNX1;FAM133B;CDC37;SUB1;WDFY3;CGGBP1;YTHDC1;
T6GAL2;KIAA1462;EBF1;ACSL6;HPCAL4;SYTL4;FOXP2;GPRIN3;MOB3C;SFRP1;C1ORF115;CRISPLD2
;IL6R;SH3GL2;KCNH1;EOMES;SFMBT2;CNPY1;POU3F1;SHISA9;SYTL4;FOXP2;EREG;GPRIN3;SFRP1
3;C21ORF59;ALG14;ACAP2;TBL1XR1;FAM185A;CMPK1;COMMD3-BMI1;SRSF9;ZNF275;PFKFB3;PSMD
FG2;TGM2;PCDHAC1;TMEM194B;MAP2K1;ALG14;SHISA9;PCDHA12;FOXP2;GTPBP4;PCDHA10;ADC
;SNRPD3;PELO;IL6R;TMEM194B;C21ORF59;BROX;C9ORF64;RBX1;EREG;BCMO1;ADCY9;HECW2;FAI
RF64;ADCY9;CRISPLD2;CDC37;HECW2;FAM64A;CGGBP1;KCTD16;GPM6A;SHOX2;AGAP2;GATA6;TM
PTGFRN;SNRPD3;ZNF687;IL6R;PCDHAC2;AGFG2;PCDHAC1;EOMES;KCMF1;TMEM194B;CXADR;DYR
6;KIAA0895;PIM3;PTGFRN;SNRPD3;PELO;SH3GL2;AGFG2;PCDHAC1;EOMES;RAB2B;TMEM194B;CXA
MT1;MED14;NHSL2;GRM5;SPRED1;GJA3;CDH2;FADS6;SIN3A;KIAA0895;MAP3K9;SNRPD3;SH3GL2;C
HAC2;AGFG2;KCNH1;TGM2;ESCO1;ALG14;SHISA9;SYTL4;FOXP2;GPRIN3;MOB3C;CDC37;NPTXR;CG
687;SEC62;RAB2B;TMOD3;BROX;USP2;RBX1;MOB3C;ADCY9;SUB1;FAM185A;CMPK1;SCG3;TBC1D1
;RAB2B;GPR75;ATP11A;POU3F1;RBX1;EREG;TBL1XR1;WDFY3;ANKRD40;KANK4;GLYR1;TBC1D19;AE
A1;RAB2B;C21ORF59;SWAP70;BROX;OMD;USP2;GPR75;HCFC2;ZNF318;CMPK1;TBC1D25;SRSF9;BH
TPNC1;ELAVL2;NUDT4;PHF8;TIAL1;KIAA0895;TRIM3;SH3BGRL2;MAP3K9;PTGFRN;SNRPD3;PELO;IL6
IM44;PCDHA6
2;PIM3;MAP3K9;SNRPD3;ZNF687;IL6R;SH3GL2;TMEM194B;CXADR;DYRK1A;ARID5B;BCMO1;ADCY9;
;MEF2A;FNBP4;KCMF1;RAB2B;TMEM194B;MBNL2;C21ORF59;BROX;ALG14;FNDC3A;GTPBP4;DKK3;C
DHA10;EREG;RNF126;SUB1;CMPK1;COMMD3-BMI1;SCG3;CGGBP1;BHLHB9;PSMD11;UBA6;ARL3;AG
X;USP2;FTSJ2;HCFC2;ZNF318;TBC1D25;TFAM;CGGBP1;FAM114A1;UBA6;TNKS;ARL3;ABHD2;CUL3;
FRN;SNRPD3;SLC25A45;TMEM194B;CXADR;SWAP70;BROX;TMOD2;DYRK1A;ARID5B;BCMO1;MAF;A
M3;MAP3K9;SNRPD3;MACROD2;ZNF687;PELO;KCMF1;TMEM194B;ST6GAL2;DYRK1A;EBF1;C9ORF6
AA0895;SH3BGRL2;KIF1B;CEP170;PTGFRN;SNRPD3;PELO;EOMES;CXADR;C21ORF59;ARID5B;FNDC
ER2;SNRPD3;PELO;IL6R;SH3GL2;RAB2B;TMEM194B;C9ORF64;EREG;TBL1XR1;HECW2;FAM185A;ZN
P2K1;ESCO1;TMOD3;ALG14;FAM133B;CDC37;SUB1;WDFY3;CGGBP1;PTGFR;YTHDC1;UBA6;ABHD2;
IMS3;FNTB;DAG1;PIM3;SLC25A45;PCDHAC2;WLS;IL13RA1;TNS1;FBXW4;TMOD3;SWAP70;USP2;YOD
B;C21ORF59;EBF1;ALG14;ARID5B;HPCAL4;C9ORF64;FAM185A;ELMO1;WDFY4;CGGBP1;SHC4;DLX1;
FTSJ2;FOXP2;C9ORF64;ADCY9;CDC37;FAM185A;COMMD3-BMI1;WDFY3;ANKRD40;PPIC;SHC4;PTGF
LDN1;IKZF3;EPS8;CDH6;CCND3;EFEMP1;GJA3;DPYSL5;FADS6;DPYSL3;FNTB;IL6R;SH3GL2;PCDHAC
PD3;PELO;AGFG2;TNS1;MEF2A;TMEM194B;CXADR;SFMBT2;EBF1;FNDC3A;HPCAL4;C9ORF64;GTPB
DH6;EFEMP1;CDH2;FADS6;DPYSL3;MAP3K9;MACROD2;BSN;PELO;SH3GL2;PCDHAC2;KCNH1;PCDH
NS1;FNBP4;RAB2B;CXADR;ESCO1;SLC30A4;ACSL6;HCFC2;GTPBP4;FAM133B;CRISPLD2;ZNF318;TB
2;SLC30A4;CMC1;RNASE6;ARID5B;GAPVD1;SYTL4;FAM133B;ADAM19;MOB3C;CDC37;TBC1D25;DLX1
PVD1;PIAS1;FAM133B;ACAP2;CRISPLD2;SUB1;ARMCX6;GLYR1;PSMD11;YTHDC1;CUL3;C1ORF52;GI
X;USP2;CMC1;ALG14;GPR75;FTSJ2;HCFC2;COMMD3-BMI1;ASPHD2;WDFY3;FAM64A;SHC4;NECAB3;
VAP70;BROX;CMC1;ALG14;ATP11A;MOB3C;FAM185A;SIK3;TBC1D25;TFAM;WDFY3;PFKFB3;BHLHB9;
0895;TRPS1;FNTB;MAP3K9;SNRPD3;PELO;EOMES;GPR37;WDR37;GAB1;ARID5B;PAX5;PROX1;CDYL
P3K9;PTGFRN;MACROD2;BSN;GPR75;YOD1;FNDC3A;POU3F1;GTPBP4;RBX1;GPRIN3;ADAM12;WDF
T5;RIMS3;GJA3;CDH2;FADS6;KIF5C;KIAA0895;RAVER2;PIM3;PTGFRN;SNRPD3;BSN;IL6R;KCNH1;TG
FTSJ2;CMPK1;CDIP1;WDFY3;FAM64A;WDFY4;CGGBP1;MTMR9;UBE2J1;CYB5R4;ZMAT3;VTI1A;MYH1

PSPH;RAB2B;TMEM194B;TMOD3;ALG14;EREG;TCEANC2;CGGBP1;PFKFB3;YTHDC1;UBA6;HSPA4L;FU
F111;AGFG2;TGM2;TMEM194B;ESCO1;ALG14;SHISA9;SYTL4;GTPBP4;ADCY9;CDC37;ADAM12;ARMCX
EM194B;ST6GAL2;ALG14;ACTN4;FNDC3A;RBX1;FAM133B;SUB1;ELMO1;TCEANC2;CGGBP1;SHC2;YTI
SL5;KIAA0895;RAVER2;TRIM24;PIM3;PTGFRN;SNRPD3;MACROD2;ZNF687;PELO;IL6R;KCNH1;EOMES
;TRIM24;SNIP1;PIM3;RNF111;KPNA1;TGM2;IL13RA1;RAB2B;TMEM194B;MAP2K1;ESCO1;TMOD3;ALG
DS6;RUVBL1;MAP3K9;PTGFRN;SNRPD3;MACROD2;SLC25A45;BSN;PELO;IL6R;SH3GL2;KCNH1;TGM2
FRN;SNRPD3;IL6R;EOMES;KCMF1;TMEM194B;CXADR;SWAP70;BROX;C9ORF64;EREG;FAM185A;SCC
108;MAP3K9;BSN;SH3GL2;KCNH1;GRID1;TMOD2;PAX5;POU3F1;SHISA9;HPCAL4;ADRA2B;PAX2;ADR
HPCAL4;CDC37;KANK4;PPIC;SET;PFKFB3;NECAB3;HSPA4L;GLIS3;RHOBTB3;MTMR9;FBLN5;NPAT;AP
;SH3GL2;PCDHAC2;SCN1A;PCDHAC1;CXADR;RNASE6;ACSL6;GAPVD1;SHISA9;EREG;DKK3;C1ORF1
4;EOMES;KCMF1;CSNK1G3;YOD1;FOXP2;GTPBP4;PIAS1;MOB3C;ACAP2;TFAM;SIK2;GLYR1;UBA6;SP
AB2;CXADR;C5ORF15;TMOD3;ANGEL2;SLC30A4;ALG14;FNDC3A;GAPVD1;GTPBP4;ADAM19;TBL1XR
25A45;TGM2;KCMF1;TPM3;BROX;CMC1;ALG14;HPCAL4;RUNX3;GTPBP4;RBX1;BCMO1;CDC37;COMM
A0895;RAVER2;DPYSL3;PIM3;SNRPD3;ZNF687;PELO;IL6R;KCMF1;TMEM194B;DYRK1A;EBF1;RUNX1;
UVBL1;SNIP1;SNRPD3;MACROD2;PCDHAC2;C3ORF14;PCDHAC1;FBXW4;CSNK1G3;RNASE6;YOD1;F
A9;PCDHA11;PCDHA10;GPRIN3;MOB3C;ADCY9;ADAM12;TBC1D25;WDFY3;KANK4;SHC2;ABHD4;PDE
;PCDHA8;PELO;PCDHA7;JAM2;PCDHA6;SFMBT2;GABRA3;EPT1;PAX5;MARCH7;IL17RD;PCDHA12;PC
IAP3K9;PTGFRN;MACROD2;PCDHAC2;KPNA1;SCN1A;PCDHAC1;EOMES;CXADR;ST6GAL2;FNDC3B;F
NIP1;BSN;PCDHAC1;RAB2B;TMEM194B;CXADR;USP2;VASH2;EBF1;CNPY1;FTSJ2;PCDHA12;DKK3;MA
KCNH1;EOMES;RAB2B;TMEM194B;C21ORF59;ALG14;ARID5B;HECW2;FAM185A;COMMD3-BMI1;PPIC;
I;EMB;SLIT2;HOXC8;TEAD1;SLC28A3;FGFR1;BMPR1A
KCNH1;TGM2;PCDHAC1;ST6GAL2;ALG14;PCDHA12;SYTL4;PCDHA11;MOB3C;CDC37;ADAM12;KANK
;ESCO1;ALG14;GTPBP4;FAM133B;CDC37;SLC25A51;TBC1D25;TFAM;WDFY3;CGGBP1;YTHDC1;UBA6
AB2B;SLC30A4;DIO2;ALG14;ATP11A;FNDC3A;GAPVD1;POU3F1;SHISA9;GTPBP4;RBX1;GPRIN3;SLC2
;SESN3;CDH2;FADS6;DPYSL3;SNRPD3;SLC25A45;PELO;PCDHAC2;CSNK1G3;RAB2B;TMEM194B;CX
;MAP3K9;BSN;SH3GL2;HSDL1;GRID1;TMOD2;POU3F1;HPCAL4;ADRA2B;PAX2;ADRA2A;TGFBR3;SFR
MC1;KIAA1462;FTSJ2;SHISA9;C9ORF64;BCMO1;FAM185A;TBC1D25;TFAM;WDFY3;DLX1;PFKFB3;BHL
LG6;BROX;USP2;VASH2;ALG14;ARID5B;FNDC3A;HCFC2;RBX1;BCMO1;NPTXR;TBCEL;NPR3;DCUN1D
RMCX6;TFAM;AGTR2;SIK2;PPIC;GLYR1;PDE1B;C1ORF52;FBLN5;ATXN7;PRDM16;MCMBP;SCN3B;SY
WAP70;ALG14;ATP11A;HCFC2;RUNX1;DHX40;SUB1;ZNF318;TBC1D25;TFAM;SCG3;GLYR1;ZNF275;PF
ORF15;ALG6;BROX;EBF1;HPCAL4;C9ORF64;ADCY9;HECW2;ASPHD2;CGGBP1;KCTD16;SHOX2;TMTC
KK3;CRISPLD2;HECW2;FAM64A;CGGBP1;ZNF275;PTGFR;SHOX2;TMTC3;SNX1;APH1B;PCDHA1;RNF2
A6;SESN3;GJA3;DPYSL5;FADS6;KIF5C;IGLON5;MAP3K9;BSN;SH3GL2;PCDHAC2;KCNH1;PCDHAC1;E
2B;MAP2K1;MBNL2;SUB1;TFAM;FAM64A;DENND6A;PPIC;GLYR1;TBCEL;PSMD11;ABHD4;TSHZ3;DCUN
BSN;PCDHAC2;AGFG2;FBXW4;ESCO1;ALG14;GTPBP4;EREG;BCMO1;MOB3C;CDC37;ADAM12;CDIP1
C21ORF59;OMD;USP2;KIAA1462;YOD1;PCDHA13;PCDHA10;ADAM12;SCG3;GPM6A;BHLHB9;DLX6;TR
R;PCDHAC2;SCN1A;PCDHAC1;EOMES;TPM3;TMOD2;USP2;KIAA1462;VASH2;EBF1;ARID5B;ZBTB34;A
CDH2;KIF5C;RAVER2;DIP2B;SH3GL2;PCDHAC2;SCN1A;IL13RA1;PCDHAC1;ST6GAL2;TPM3;SFMBT2;T
S2;FNDC3A;FTSJ2;HCFC2;ACAP2;MAF;ELMO1;WDFY3;KCTD16;PTGFR;TBC1D19;TBCEL;PFKFB3;ARL
CMC1;EBF1;ARID5B;ATP11A;FTSJ2;GTPBP4;EREG;BCMO1;FAM185A;SIK3;COMMD3-BMI1;TFAM;WDF
PCDHAC1;ESCO1;ALG14;GTPBP4;MOB3C;CDC37;HECW2;CDIP1;WDFY3;NPTXR;CGGBP1;KANK4;PF
BSN;RNF111;KCNH1;IL13RA1;PCDHAC1;EOMES;TMEM194B;ESCO1;ALG14;FOXP2;GTPBP4;SFRP1;C
;PIM3;MAP3K9;PTGFRN;SNRPD3;ZNF687;BSN;IL6R;SH3GL2;EOMES;RAB2B;TMEM194B;CXADR;ALG6
AP3K9;SNRPD3;MACROD2;IL6R;RNF111;PRKG1;DGCR2;MAP2K1;CXADR;ST6GAL2;WDR37;TMOD3;BI
ES;RAB2B;TMOD3;TMOD2;USP2;KIAA1462;HPCAL4;EREG;BCMO1;DHX40;GPRIN3;C1ORF115;CRISPL
1;ALG14;ACTN4;FTSJ2;GTPBP4;TCEANC2;SLC25A51;TFAM;DENND6A;CGGBP1;YTHDC1;FOXO1;AP5
JSP2;CMC1;DYRK1A;ATP11A;FTSJ2;GTPBP4;FAM185A;WDFY3;ANKRD40;FAM64A;PPIC;GLYR1;PFKFE
IS1;ST6GAL2;ACSL6;GPR75;POU3F1;C9ORF64;KIAA1614;CRISPLD1;HECW2;PTGFR;GPM6A;ADCYAP
C1;PRKAB2;MBNL2;TPM3;C21ORF59;ALG14;YOD1;FNDC3A;PCDHA13;EREG;BCMO1;C1ORF115;CRIS
YSL5;PAPOLG;SNIP1;SNRPD3;PELO;AP2M1;IL13RA1;FNBP4;EOMES;ESCO1;BROX;YOD1;ATP11A;FO
EOMES;TMOD2;KIAA1462;ACSL6;POU3F1;EREG;DHX40;GPRIN3;C1ORF115;CRISPLD2;ADAM12;GPM
;SH3GL2;PCDHAC2;PCDHAC1;EOMES;ST6GAL2;SFMBT2;TMOD2;ACSL6;CNPY1;HPCAL4;FOXP2;SFF
12;IL13RA1;RAB2B;KIAA1462;FTSJ2;FAM133B;C1ORF115;FAM185A;TBC1D25;COMMD3-BMI1;CGGBP1

B;ESCO1;VASH2;ALG14;FNDC3A;RUNX3;GTPBP4;FAM133B;ADCY9;CDC37;SUB1;TCEANC2;TBC1D25
'H;KCMF1;CXADR;SWAP70;YOD1;FNDC3A;FOXP2;PIAS1;DHX40;ADAM12;ELMO1;ANKRD40;SET;MTM
'4;SWAP70;ALG14;HCFC2;FAM133B;GPRIN3;CDC37;TCEANC2;TBC1D25;WDFY3;CGGBP1;YTHDC1;UE
A1;TMEM194B;ESCO1;TMOD3;RNASE6;ALG14;FNDC3A;GTPBP4;FAM133B;CDC37;SUB1;TCEANC2;TE

;AP2;SLC25A51;ANKRD40;MTMR3;PSMD11;ABHD4;DCUN1D5;TMTC3;SPATA2;APCDD1;ZMPSTE24;BC
.CTN4;RUNX1;CDC37;WDFY3;CGGBP1;YTHDC1;UBA6;HSPA4L;FUT11;MTMR9;SPATA2;VTI1A;MCMBP;
A;DIO2;ALG14;ATP11A;GAPVD1;RBX1;EREG;ADAM12;TFAM;WDFY3;NPTXR;GLYR1;FAM114A1;TMTC3
H3GL2;MEF2A;EBF1;YOD1;FNDC3A;GTPBP4;CDC37;ARMCX6;KANK4;SRSF9;GATA6;DCUN1D1;NKAIN
)X;CMC1;ALG14;CDC37;FAM185A;WDFY4;DLX1;FAM114A1;IGSF3;UBA6;ARL3;TMTC3;SPATA2;DTX1;C
1;MOB3C;ACAP2;MAF;C1ORF115;PFKFB3;BHLHB9;FAM114A1;ABHD2;MTMR9;CREBL2;BCL2L13;RNF
HCFC2;HPCAL4;FOXP2;SIK3;CGGBP1;PPIC;TNKS;ARL3;TMTC3;FBLN5;ZMPSTE24;NPAT;BCL2L13;IP6
K9;SNRPD3;PELO;IL6R;RNF111;MEF2A;CXADR;CMC1;ALG14;GPR75;ARID5B;HCFC2;C9ORF64;EREG;
H1;ST6GAL2;SFMBT2;TMOD2;KIAA1462;VASH2;EBF1;ACSL6;CNPY1;POU3F1;HPCAL4;FOXP2;C9ORF6
B2B;DYRK1A;YOD1;FNDC3A;FOXP2;MOB3C;ADAM12;CMPK1;CDIP1;ASPHD2;ANKRD40;WDFY2;GLYF
ACTN4;GAPVD1;HCFC2;RUNX3;RUNX1;FAM133B;DHX40;CDC37;TFAM;TBCEL;MTMR3;NECAB3;DCUN
3;GJA3;FADS6;KIF5C;SALL4;HOXA3;PTGFRN;BSN;IL6R;SH3GL2;KCNH1;PCDHAC1;EOMES;ST6GAL2;
CDHA13;FTSJ2;EREG;MOB3C;CDC37;COMMD3-BMI1;ASPHD2;WDFY3;SCG3;SHC4;BHLHB9;TSHZ3;A
MO1;FAM185A;TFAM;ASPHD2;ANKRD40;IGSF3;PDE1B;RHOBTB3;TMTC3;NKAIN1;ARHGAP20;APH1B;V
CO1;TMOD3;ALG14;ACTN4;FAM133B;CDC37;TCEANC2;TBC1D25;CGGBP1;YTHDC1;SPATA2;GIGYF2;
K9;PTGFRN;BSN;SH3GL2;PCDHAC2;WLS;KCNH1;IL13RA1;PCDHAC1;EOMES;ST6GAL2;SLC30A4;FND
;SPRED1;CDH2;SIN3A;TRPS1;SH3BGRL2;SNRPD3;MACROD2;SLC25A45;GPR135;NCOA2;GPR37;HSI
IRPD3;PELO;RNF111;CHST7;TMED8;C21ORF59;BROX;ANGEL2;NSUN3;KAT2B;HECW2;FAM185A;LCO
ETTL16;PCDHAC2;CCDC50;AGFG2;PSPH;KPNA1;TMED8;ESCO1;C21ORF59;BROX;NSUN3;ALG14;LRI
;CCND3;JPH3;IGLON5;KIF1B;MAP3K9;EMB;RBM7;IL13RA1;RAB2B;TSFM;PLA2G12A;C3ORF62;CMC1;I
G;FAM154B;SNIP1;SEC62;AGFG2;UBL7;VKORC1;UTP15;ESCO1;TTC33;SWAP70;SORD;ALG14;LRRC4
BGRL2;SCN5A;SLC25A45;CNPPD1;SH3GL2;PSPH;TMEM194B;GRID1;OMD;USP2;C3ORF62;DNMT3A;
A2;C1QBP;FAM154B;FBXO3;PIM3;SNRPD3;IL6R;AP2M1;SH3GL2;APPL1;MEF2A;EOMES;ARHGEF10;R
BGRL2;KIF1B;MAP3K9;UBL7;PRKAB2;PLA2G12A;C5ORF15;USP2;C3ORF62;AHSA2;GAB1;ALG14;LRR
6;SDR16C5;NHSL2;CCND3;ADAMTS2;MECOM;SLC22A17;IGLON5;SALL4;RAVER2;DPYSL3;FNTB;SOS
R3A;MB;MFSD5;PAPOLG;FBXO3;CEP170;PTGFRN;SNRPD3;METTL16;RNF111;APPL1;FBXW4;USP47;
KIAA0895;PAPOLG;DPYSL3;FBXO3;SNRPD3;ZNF687;SEC62;PELO;IL6R;AP2M1;PSPH;MEF2A;PRKAB2
AA0895;PTGFRN;SNRPD3;BSN;PELO;KCNH1;TGM2;IL13RA1;EOMES;KCMF1;RAB2B;TMEM194B;C5O
DPYSL5;FADS6;FRRS1L;FNTB;HOXA3;RSPO2;SH3BGRL2;SCN5A;BSN;AGFG2;TGM2;PCDHAC1;PHAC
PH1;ING4;DTWD1;NHSL2;SERP1;CAPZB;CDH2;FADS6;FBXO3;ZNF687;SEC62;APPL1;KCNH1;IL13RA1
B;SCN5A;METTL16;PSPH;WLS;FNBP4;ZCCHC24;ARHGEF10;TSFM;USP2;AHSA2;GAB3;PROX1;RUNX
3A;UBXN7;BTRC;RBM7;UNKL;KPNA1;UBL7;PHACTR4;VKORC1;KCMF1;PRKAB2;WDR37;ALG6;ZFP91;
;GJA3;SH3PXD2A;TRIM3;PGM3;SH3BGRL2;PIM3;RBM7;CTBS;TGM2;FBXW4;EOMES;CXADR;ALG6;DN
AM73A;RBM7;PELO;DGCR2;IL13RA1;RAB2B;PLA2G12A;C21ORF59;VPS13C;USP2;C3ORF62;DNMT3A
;FNDC3B;VASH2;POU3F1;SHISA9;PCDHA12;PCDHA11;FOXP2;PCDHA10;DHX40;SFRP1;MAF;ADCY9;I
IN1;CTDSPL2;LITAF;BACH1;SESN3;ALDH2;TMEM108;SNIP1;MACROD2;SH3GL2;PCDHAC2;C3ORF14;
TRPS1;FBXO3;SOSTDC1;CEP170;SNRPD3;RBM7;ARHGEF10;RAB2B;GPR37;BROX;NSUN3;USP2;CM
POLG;SALL4;RUVBL1;FNTB;PSD3;DAG1;EPC1;CEP170;BTRC;ARL5A;RNF111;CCDC50;UTP15;KCMF1
SNRPD3;RBM7;UNKL;PCDHAC1;KCMF1;TMEM194B;GPR37;USP2;C3ORF62;DNMT3A;GAB1;VASH2;EE
L1;CCND3;UBL3;GJA3;CDH2;KIF5C;TRPS1;FNTB;FAM73A;SH3BGRL2;SOX6;UNKL;AGFG2;KCNH1;PC
NTB;DAG1;BSN;RNF111;TGM2;TMEM194B;ESCO1;TMOD3;ALG14;FNDC3A;SHISA9;SYTL4;GTPBP4;EF
;FADS6;SIN3A;SLC22A17;FNTB;FAM73A;HOXA3;RSPO2;SH3BGRL2;BSN;GPRASP2;AGFG2;KCNH1;TC
SNRPD3;PELO;KCNH1;IL13RA1;PCDHAC1;KCMF1;USP2;C3ORF62;DNMT3A;EBF1;ACSL6;LRRC40;GF
17;ING4;DTWD1;CCND3;RUVBL1;SH3BGRL2;PTGFRN;PRKAB2;TMED8;BROX;NSUN3;FTSJ2;HCFC2;
3;FAM73A;HOXA3;DAG1;SH3BGRL2;PTGFRN;SLC25A45;METTL16;TGM2;CCDC112;VKORC1;UTP15;Z
D3;ZNF687;RBM7;PELO;UBL7;IL13RA1;UTP15;RAB2B;CXADR;TMED8;NSUN3;USP2;CMC1;AHSA2;GA
H3;EEF2K;SART3;MECOM;DPYSL5;NKRF;C1QBP;SNIP1;SEC62;CNPPD1;PRKG1;APPL1;TNS1;FNBP4;
3;HOXA3;PIM3;KIF1B;CTBS;UNKL;RNF111;CCDC50;TGM2;VKORC1;MAPK1IP1L;TMEM194B;ESCO1;VF

GRL2;FBXO3;PIM3;SLC25A45;RBM7;AGFG2;NCOA2;TMEM194B;NSUN3;USP2;C3ORF62;AHSA2;GAB1
2;MAP3K9;MACROD2;BSN;SOX6;ENTPD1;ALG6;TMOD3;CMC1;FNDC3B;VASH2;GPR75;ATP11A;POU3F
549;EFR3A;FADS6;MB;TMEM108;FBXO3;RNF111;KCMF1;MAPK1IP1L;TSFM;PCYT1B;ESCO1;TPM3;C2
PL1;UTP15;TMED8;MME;SGIP1;BROX;NSUN3;ITPK1;GAB1;FTSJ2;HCFC2;CBFA2T2;SENP2;DIEXF;C9
X3;FAM64A;CGGBP1;IGSF3;HSPA4L;MTMR9;RNF214;APH1B;RIC8B;MLEC;HIVEP2;RIMKLA;RAB11FIP

1;ANKRD40;WDFY2;CGGBP1;TBC1D19;ABHD4;UBA6;SHOX2;HSPA4L;TMTC3;MTMR9;CREBL2;SLC1A
HAX;NUDT4;TIAL1;CCND3;EEF2K;SESN3;ALDH2;SNIP1;ZNF367;RNF111;GPR135;VKORC1;CSNK1G3;
HSL2;RIMS3;SLC22A17;TRPS1;SCN5A;BSN;KCNH1;TMEM194B;C5ORF15;ALG6;GRID1;SGIP1;VASH2;
SF3;IKZF3;AFF4;IL18BP;AMOT;ELK4;ING4;RBM3;UBL3;RIMS3;ZNF687;METTL16;IL6R;TSFM;SFMBT2;E
;TIAL1;MRPL42;PCMT1;ADAMTS5;ALDH2;TRPS1;FNTB;RSPO2;SH3BGRL2;SOX6;RNF111;PRKG1;TGM
RPS1;FNTB;FAM73A;RSPO2;SH3BGRL2;AGFG2;TGM2;PCDHAC1;CCDC112;UTP15;ZCCHC24;MAPK1I
PD3;PELO;PCDHAC2;ST6GAL2;DYRK1A;FNDC3A;FTSJ2;SHISA9;EREG;GPRIN3;SFRP1;CRISPLD2;CD
L5;FADS6;DIP2B;AP2M1;SH3GL2;KPNA1;IL13RA1;TNS1;KIAA1462;CNPY1;GAPVD1;FOXP2;RUNX1;DH
2;SNIP1;PIM3;UNKL;AGFG2;PHACTR4;VKORC1;MAPK1IP1L;DFFA;GPR37;MME;TMOD3;ACSL6;ALG14;
F1B;PELO;SH3GL2;KCNH1;TGM2;IL13RA1;VKORC1;EOMES;HS3ST3B1;ARHGEF10;TSFM;WDR37;AL
F;MYPN;AFF1;ELAVL2;IL18BP;AMOT;JPH3;DPYSL5;FADS6;SIN3A;IGLON5;DPYSL3;EMB;PELO;CNPPD
JA9;METTL16;RBM7;IL6R;RNF111;AGFG2;TGM2;EOMES;UTP15;RAB2B;TSFM;CXADR;ESCO1;SGIP1;
D2A;FAM154B;MAP3K9;CNPPD1;MEF2A;FNBP4;VKORC1;EOMES;HS3ST3B1;KLF10;DFFA;GPR37;GR
VBL1;FNTB;SNIP1;CEP170;AGFG2;FBXW4;ZCCHC24;KLF10;MBNL2;EBF1;LRRC40;HAUS3;ARID5B;YC

CMC1;PROX1;FTSJ2;SYTL4;DIEXF;CDYL2;NAALADL2;EREG;BCMO1;RAP2C;FAM185A;FAM84A;LCOR
1;SNRPD3;RNF111;AGFG2;PSPH;KCNH1;UTP15;SLC30A4;HAUS3;SMC1A;CBFA2T2;HPCAL4;PAX2;RB
7;KCMF1;TMEM194B;PLA2G12A;GPR37;SGIP1;NSUN3;USP2;C3ORF62;CMC1;AHSA2;LRRC40;ARID5E
;ADAMTS2;JPH3;EFEMP1;SART3;MECOM;SH3PXD2A;DPYSL5;SLC22A17;IGLON5;PAPOLG;PSD4;SH3
13;IKZF3;NHSL2;RIMS3;CDH2;ALDH2;NKRF;FADS6;SH3BGRL2;SNRPD3;SCN5A;SLC25A45;BSN;PCDH
;NKRF;RAVER2;SH3BGRL2;SOSTDC1;SLC25A45;RBM7;SH3GL2;TGM2;TMEM194B;USP2;C3ORF62;DI
R2;SNRPD3;SCN5A;SLC25A45;ZNF687;RBM7;AGFG2;TMEM194B;TMED8;NSUN3;USP2;C3ORF62;CMC
PCDHAC2;TGM2;PCDHAC1;TPM3;SFMBT2;TMOD2;VASH2;EBF1;SHISA9;HPCAL4;SYTL4;ADAM19;GP
PTGFRN;SLC25A45;RBM7;UNKL;TGM2;EOMES;KCMF1;RAB2B;TMEM194B;C21ORF59;BROX;DNMT3A
RF;MAN1A2;FAM154B;SH3BGRL2;EPC1;PTGFRN;PELO;CNPPD1;KPNA1;GPR135;MEF2A;KLF10;TTC
SL5;TRIM3;PELO;SH3GL2;PCDHAC2;PHACTR4;TNS1;HS3ST3B1;UTP15;ZCCHC24;TTC33;SGIP1;USP
S6;TRIM3;TMEM108;EPC1;SNRPD3;AGFG2;KCNH1;IL13RA1;UTP15;KCMF1;RAB2B;PCYT1B;GPR37;MI
17;FAM154B;DAG1;CNPPD1;APPL1;UTP15;RAB2B;TSFM;PLA2G12A;ALG6;VPS13C;DYRK1A;SORD;AL

D4;FAM154B;RBM7;PSPH;TNS1;MEF2A;RAB2B;PCYT1B;TMED8;BROX;NSUN3;CMC1;FTSJ2;SENP2;D
8;EFEMP1;MECOM;CDH2;MB;SLC22A17;MFSD5;TMEM108;SEC62;IL6R;CCDC50;C3ORF14;IL13RA1;P
MACROD2;SOX6;FNBP4;RAB2B;USP47;HSDL1;TMED8;OMD;MSL2;SORD;ACSL6;LRRC40;YOD1;PTP4
LDH2;DPYSL5;NKRF;RAVER2;SLC25A45;BSN;AGFG2;UTP15;KCMF1;TMEM194B;GPR37;TPM3;OMD;I
6;RAVER2;MAP3K9;MACROD2;BSN;RBM7;ZNF367;TGM2;UBL7;PCDHAC1;PRKAB2;C5ORF15;ESCO1;
LC22A17;MFSD5;MACROD2;KCNH1;DNMT3A;GAPVD1;HPCAL4;SUMF1;EREG;DHX40;RAP2C;CRISPL
TAF;HK2;EPS8;ELK4;EFEMP1;SART3;SERP1;MECOM;PAPOLG;TRIM3;DIP2B;IL6R;ZNF366;IL13RA1;PH
TXR2;LITAF;PITPNC1;TXNDC17;PHF8;EPS8;CCND3;SPRED1;C1QBP;FAM73A;PTGFRN;SNRPD3;PEL
DPYSL5;NKRF;FADS6;KIF5C;RAVER2;TMEM108;SH3BGRL2;SOSTDC1;MAP3K9;SCN5A;SH3GL2;TGM
PSPH;KPNA1;KCNH1;FBXW4;EOMES;ALG6;SLC30A4;FNDC3B;DYRK1A;ALG14;GPR75;YOD1;HCFC2;
M2;TMEM194B;ENTPD1;ESCO1;RNASE6;DYRK1A;TAP2;ALG14;FNDC3A;FTSJ2;HCFC2;RUNX3;GTPB
CDH2;MFSD5;SH3BGRL2;MAP3K9;SLC25A45;RBM7;CNPPD1;KPNA1;MEF2A;UTP15;C5ORF15;C21OR
IP1;MECOM;SLC22A17;MFSD5;FNTB;RSPO2;SOSTDC1;MAP3K9;DIP2B;IL6R;KPNA1;PHACTR4;USP47
5A;AGFG2;ARHGEF10;TMEM194B;OMD;C3ORF62;GPR75;C6ORF62;PROX1;ZFX;GTPBP4;CDYL2;ADI
VBL1;MAP3K9;EMB;AP2M1;UBL7;PRKAB2;DFFA;PLA2G12A;ALG6;GAB1;ALG14;HAUS3;SLC39A13;GAE
SN3;GJA3;SH3PXD2A;FADS6;FBXO3;SOSTDC1;SNRPD3;BSN;RBM7;TGM2;TNS1;HS3ST3B1;RAB2B;N
A1549;SART3;SESN3;XPO4;KIF1B;MAP3K9;EMB;RBM7;ZNF367;KCMF1;C5ORF15;ALG6;SLC30A4;CM
HSL2;RPS6KA6;RIMS3;SESN3;GJA3;SLC25A45;BSN;KCMF1;ARHGEF10;TMED8;NSUN3;C3ORF62;GAE

L1;TRIM3;RBM7;CNPPD1;UTP15;TMEM194B;USP2;CMC1;GAB1;YOD1;RUNX3;C9ORF64;BCMO1;RUN
TS5;ADAMTS2;SPRED1;SART3;MECOM;MB;PIM3;SNRPD3;MACROD2;TEAD1;RNF111;HS3ST3B1;VPS
C5;CDH2;ALDH2;DPYSL3;SCN5A;IL6R;PCDHAC2;KCNH1;PCDHAC1;CCDC112;PCYT1B;CXADR;GPR3
ZF3;ADAMTS5;EFEMP1;RIMS3;GJA3;FADS6;DPYSL3;FAM154B;RSPO2;CTBS;SH3GL2;PCDHAC2;GPR
YSL3;SH3BGRL2;SOSTDC1;MACROD2;PCDHAC2;CCDC50;PRKG1;C3ORF14;SCN1A;TGM2;ST6GAL2;
RN;RBM7;IL6R;PSPH;UBL7;FNBP4;KCMF1;C5ORF15;ALG6;TPM3;VPS13C;NSUN3;USP2;ALG14;FTSJ
3;MECOM;CDH2;DPYSL5;SCN5A;EMB;SEC62;PCDHAC2;KCNH1;CCDC112;PLA2G12A;GPR37;CHST7;
7;ALAD;ADAMTS5;DTWD1;NHSL2;SESN3;TMEM108;SOSTDC1;SNRPD3;BSN;PELO;SH3GL2;KCNH1;
2;SIN3A;C1QBP;SNIP1;SNRPD3;CTBS;PSPH;KPNA1;GPR135;TTC33;SFMBT2;C3ORF62;HAUS3;SLC3
M3;ADAMTS5;SH3PXD2A;MB;MFSD5;PAPOLG;FNTB;TRIM3;HOXA3;RSPO2;SOSTDC1;SLC25A45;IL6
1;TRIM3;HOXA3;SH3BGRL2;CEP170;SNRPD3;UBL7;UTP15;NCOA2;RAB2B;TMEM194B;TMED8;BROX
RIM3;TMEM108;ZNF366;CCDC50;KCNH1;VKORC1;EOMES;ARHGEF10;DFFA;GPR37;ESCO1;TMOD3;
L5;DDI2;KIF1B;EMB;AGFG2;SCN1A;IL13RA1;FBXW4;CCDC112;ENTPD1;GPR37;MME;TMOD2;ITPK1;V
;TRPS1;FNTB;RSPO2;BSN;RNF111;PCDHAC2;PRKG1;TGM2;PCDHAC1;ZCCHC24;MAPK1IP1L;TMEM
2;SNRPD3;SCN5A;SLC25A45;RBM7;TMEM194B;USP2;C3ORF62;DNMT3A;LRRC40;GPR75;PROX1;SE
62;BSN;RNF111;PCDHAC2;AGFG2;KCNH1;ESCO1;ALG14;FOXP2;GTPBP4;EREG;GPRIN3;MOB3C;CD

N3;ALDH2;FADS6;TRIM24;DAG1;CHP1;UBXN7;SLC25A45;PELO;CCDC50;APPL1;CCDC112;UTP15;TSF
PSD3;PIM3;GPRASP2;AP2M1;MEF2A;FBXW4;UTP15;ARHGEF10;CSNK1G3;WDR37;TMED8;C5ORF15;
T3;NKRF;SIN3A;RAVER2;ZNF800;SCN5A;KCNH1;SCN1A;ARHGEF10;NAA30;CXADR;HSDL1;TMED8;
7B;ADAMTS5;ADAMTS2;EFEMP1;MB;SLC22A17;TMEM108;SOSTDC1;SCN5A;MACROD2;SH3GL2;PC
108;SH3BGRL2;KIF1B;ZNF687;PELO;AGFG2;ZCCHC24;MAPK1IP1L;ESCO1;VPS13C;C3ORF62;GAB1;
;IGLON5;PAPOLG;PGM3;DAG1;SEC62;BSN;CNPPD1;APPL1;TGM2;UBL7;CCDC112;ALG6;ESCO1;TPM
BXO3;CEP170;SCN5A;IL6R;AP2M1;AGFG2;PSPH;APPL1;KLF10;RAB2B;TSFM;CHST7;C5ORF15;TMOD
1L;RAB2B;CHST7;C5ORF15;ALG6;LRRC40;SLC39A13;ZFY;SENP2;ZFX;RUNX3;DIEXF;GTPBP4;FAM18
3;SOSTDC1;MACROD2;SLC25A45;PELO;SH3GL2;GPR135;PHACTR4;EOMES;TSFM;USP47;TMEM194
EM108;CEP170;SCN5A;EMB;SLC25A45;BSN;SH3GL2;PRKG1;GPR135;EOMES;GPR37;ST6GAL2;GRID
2M1;PSPH;WLS;KPNA1;SCN1A;GPR135;HS3ST3B1;KCMF1;MAPK1IP1L;TTC33;NSUN3;RC3H1;FTSJ2;
ECOM;MFSD5;TRIM3;PGM3;SH3BGRL2;SNIP1;PELO;GPR135;HS3ST3B1;USP47;MBNL2;TPM3;USP2;
3;MECOM;ALDH2;IGLON5;DPYSL3;ZNF800;PTGFRN;SCN5A;MACROD2;PCDHAC2;PCDHAC1;CXADR
;TRIM3;RBM7;PELO;UBL7;MEF2A;KCMF1;NCOA2;DFFA;PLA2G12A;USP2;CMC1;GAB1;ALG14;ARID5B;
LC22A17;FNTB;HOXA3;RBM7;AP2M1;C3ORF14;TSFM;PRKAB2;WDR37;TTC33;MSL2;ZFP91;DIEXF;FA
PH3;MECOM;GJA3;RAVER2;SCN5A;EMB;SH3GL2;PCDHAC2;WLS;GPR135;EOMES;ST6GAL2;DIO2;G
GFG2;PSPH;KPNA1;FNBP4;FBXW4;KCMF1;RNASE6;YOD1;FTSJ2;GTPBP4;PIAS1;CDC34;ADCY9;CD
OT;TIAL1;ADAMTS2;EEF2K;KIF5C;SNIP1;SCN5A;C3ORF14;EOMES;PRKAB2;GPR37;SFMBT2;SWAP
7;TMEM194B;TMED8;GRID1;NSUN3;C3ORF62;DNMT3A;ARID5B;FTSJ2;HPCAL4;ZFX;DIEXF;PAX2;ADR
94B;WDR37;TMED8;NSUN3;USP2;CMC1;LRRC40;PROX1;CBFA2T2;SENP2;RUNX3;SUMF1;BCMO1;FA
KRF;KIF5C;FRRS1L;TMEM108;SH3BGRL2;SCN5A;EMB;BSN;SH3GL2;PCDHAC2;EOMES;HS3ST3B1;U
3;SERP1;ALDH2;SLC22A17;TRIM3;PGM3;FBXO3;SNIP1;SNRPD3;TMEM194B;CHST7;ESCO1;VPS13C;
ADS6;TRIM3;SH3BGRL2;SOSTDC1;CEP170;SEC62;SOX6;CTBS;WLS;KCNH1;SCN1A;PCYT1B;SGIP1;
EC62;SOX6;TPM3;RC3H1;GPR75;FNDC3A;FOXP2;DHX40;MOB3C;ACAP2;ADCY9;DLX1;BHLHB9;ARL
KS1B;ADAMTS2;SH3PXD2A;SCN5A;BSN;IL6R;CHST7;USP2;GAB1;ALG14;FTSJ2;SENP2;RUNX3;C9OF
;CDH6;MFSD5;PSD4;SOSTDC1;SLC25A45;RNF111;CCDC50;SCN1A;TNS1;EOMES;NAA30;ALG6;C3OR
17;MFSD5;SALL4;TMEM108;EMB;CCDC50;WLS;TGM2;UBL7;GPR37;SGIP1;OMD;KIAA1462;VASH2;T
BSN;CNPPD1;SH3GL2;KCNH1;ARHGEF10;GPR37;ST6GAL2;C5ORF15;ESCO1;USP2;CMC1;GAB1;ALG
PSD3;FBXO3;PELO;TGM2;GPR135;FBXW4;HS3ST3B1;USP47;TTC33;USP2;DNMT3A;CNPY1;HPCAL4;
L1;ALDH2;SLC22A17;TMEM108;SNIP1;MACROD2;C3ORF14;PCDHAC1;HSDL1;TMED8;SWAP70;VASH
;UTP15;RAB2B;TMEM194B;GPR37;CHST7;ALG6;SFMBT2;DNMT3A;VASH2;LRRC40;GAPVD1;HPCAL4
6;SNRPD3;SLC25A45;BSN;PCDHAC1;PCYT1B;USP2;C3ORF62;LRRC40;GPR75;ARID5B;SORCS1;FT
ZF3;PITPNC1;TIAL1;ADAMTS5;ADAMTS2;SART3;TMEM108;SCN5A;MACROD2;SEC62;EOMES;GPR37;
D3;YOD1;RUNX1;CDC37;CMPK1;GLYR1;DLX1;IGSF3;ARL3;SPATA2;ZNF25;BCL2L13;ATXN7;CLSPN;R
17;MFSD5;C1QBP;TRIM24;DAG1;SNIP1;PIM3;UNKL;PELO;CNPPD1;CCDC112;NAA30;MSL2;HAUS3;C
L1;FBXW4;RAB2B;TMEM194B;WDR37;TTC33;SWAP70;NSUN3;SORD;ALG14;SLC39A13;FTSJ2;C9OR

3M3;RIMS3;SERP1;MECOM;MFSD5;C1QBP;UBXN7;SNRPD3;METTL16;AGFG2;APPL1;ZCCHC24;KLF1(
R2;KIF1B;MAP3K9;BSN;RBM7;RAB2B;TMEM194B;C5ORF15;BROX;USP2;LRRC40;ZFP91;FTSJ2;SENP2
IOXA3;SOSTDC1;CEP170;SCN5A;SH3GL2;PCDHAC1;ENTPD1;TMOD2;ACSL6;GPR75;GAB3;HPCAL4;A
CMT1;GJA3;ALDH2;SIN3A;C1QBP;TRIM3;TRIM24;SNIP1;SCN5A;APPL1;GPR135;FNBP4;KLF10;RAB2B;
P4;FBXW4;KCMF1;ANGEL2;GPR75;FTSJ2;GTPBP4;CDC34;ADCY9;CDC37;TCEANC2;SLC25A51;TFAM
45;PELO;KCNH1;TGM2;IL13RA1;PCDHAC1;TNS1;ALG6;SFMBT2;RNASE6;KIAA1462;EBF1;GPR75;GAI
LON5;HOXA3;MAP3K9;WLS;ENTPD1;TPM3;TMOD2;DIO2;EBF1;GPR75;SHISA9;FOXP2;EREG;BCMO1;
7;GJA9;SLC25A45;AP2M1;ZNF366;WLS;APPL1;TNS1;RAB2B;HSDL1;MBNL2;RC3H1;GPR75;ANO6;GAI
N3A;PGM3;MAP3K9;SEC62;CTBS;KCNH1;UBL7;GPR37;WDR37;NSUN3;DIO2;SORD;EBF1;GPR75;GAP
ART3;C1QBP;GNRHR;SCN5A;SLC25A45;GPRASP2;AP2M1;MAPK1IP1L;ARHGEF10;RAB2B;TSFM;TTC
;CSNK1G3;RAB2B;MBNL2;ESCO1;SWAP70;VASH2;YOD1;HCFC2;RUNX3;GTPBP4;RBX1;CDC37;SUB1
ARL5B;SERP1;CAPZB;SNRPD3;SCN5A;ZNF687;ARL5A;UTP15;CXADR;SLC30A4;TFEB;FTSJ2;SMC1A;I
RPD3;ARL5A;AP2M1;WLS;KPNA1;MAPK1IP1L;ENTPD1;WDR37;ZFP91;HAUS3;FTSJ2;DIEXF;GTPBP4;PI
EC62;SOX6;BTRC;CTBS;GPRASP2;CSNK1G3;PCYT1B;NAA30;TPM3;ITPK1;DIO2;EBF1;SLC39A13;GAE

IP1;VPS13C;NSUN3;TAP2;SLC39A13;FTSJ2;CBFA2T2;RUNX3;FOXP2;GTPBP4;DHX40;CDIP1;FAM64A;
P3K9;DIP2B;SLC25A45;BTRC;TGM2;TNS1;MBNL2;TPM3;SWAP70;USP2;RNASE6;DIO2;ACSL6;GAPVD
XW4;ANGEL2;ZBTB34;YOD1;FTSJ2;CDC34;ADCY9;CDC37;CMPK1;TCEANC2;SLC25A51;TFAM;IGSF3;

L3;RSPO2;PIM3;EMB;PELO;CCDC50;EOMES;TPM3;C3ORF62;ARID5B;ANO6;RUNX3;ADRA2A;SUMF1;E
NKRF;PAPOLG;PGM3;PIM3;SLC25A45;CNPPD1;AP2M1;PSPH;HS3ST3B1;CSNK1G3;TSFM;RC3H1;YOI
M194B;DYRK1A;PCDHA11;PIAS1;DHX40;ADCY9;ARMCX6;TFAM;FAM64A;RAF1;PPIC;GLYR1;NECAB3;Z
;SEC62;ARL5A;IL6R;SH3GL2;KPNA1;GPR135;MBNL2;TPM3;TMOD2;C3ORF62;CNPY1;C6ORF62;GAB3
I3;FAM73A;TMEM108;CEP170;SEC62;DGCR2;ENTPD1;USP2;EBF1;ACSL6;GPR75;PROX1;GAPVD1;FT
RPD3;ZNF367;GPR135;TSFM;DFFA;WDR37;C3ORF62;MSL2;RC3H1;GPR75;SMC1A;C9ORF64;GTPBP4
;PCDHA7;C3ORF14;PCDHAC1;TMEM194B;EIF2B2;SWAP70;RNASE6;LAPTM5;PARVA;FNDC3A;PCDH

RP1;SIN3A;FAM154B;PGM3;FAM73A;TMEM108;SLC25A45;ZNF687;WLS;UBL7;KLF10;TSFM;TMEM194B
S2;MB;SLC22A17;IGLON5;DAG1;TMEM108;KIF1B;MAP3K9;SCN5A;DIP2B;SOX6;ARL5A;APPL1;KCNH1
;PIAS1;RUNX1;ARMCX6;TFAM;SET;NECAB3;IGSF3;ARL3;C1ORF52;GIGYF2;RNF214;HOXC8;SYT5;FZI
;IL6R;WLS;CXADR;TMOD2;KIAA1462;VASH2;HPCAL4;SYTL4;DKK3;BCMO1;GPRIN3;SFRP1;MAF;HEC
GFG2;UBL7;UTP15;TSFM;C21ORF59;BROX;SORD;GAB3;C9ORF64;ADCY9;FAM185A;ZNF318;TCEANC
M3;ADAMTS5;SART3;SIPA1L1;SIN3A;SLC25A45;ZNF367;AP2M1;VKORC1;CSNK1G3;TSFM;NSUN3;CBF
DH2;SOX6;TGM2;FBXW4;HS3ST3B1;ZCCHC24;ARHGEF10;GPR37;ST6GAL2;MME;TAP2;SHISA9;ADR
SD5;PSD3;CHP1;PIM3;SLC25A45;PELO;CNPPD1;AP2M1;RNF111;AGFG2;UBL7;NAA30;ANGEL2;SORD;
;ANGEL2;TAP2;FTSJ2;GTPBP4;MOB3C;CDC34;ADCY9;CDC37;ARMCX6;TCEANC2;TFAM;NECAB3;DC
PIM3;SNRPD3;RBM7;UNKL;AP2M1;PSPH;DGCR2;UBL7;FNBP4;MBNL2;RNASE6;TAP2;YOD1;PAX5;GN
EF2A;PAX5;PCDHA13;SHISA9;PCDHA12;PCDHA11;PIAS1;DDX19B;NRG3;IL1RAPL1;ELMO1;DCX;ASPH
;SESN3;DPYSL5;FADS6;MB;SLC22A17;PAPOLG;GNRHR;SOSTDC1;BSN;SOX6;ARL5A;GPRASP2;TMO
2A;DPYSL5;PCDHAC2;PCDHAC1;FNBP4;CCDC112;HS3ST3B1;CHST7;ST6GAL2;VASH2;POU3F1;SMC
AP2M1;ARHGEF10;CSNK1G3;ZFP91;HAUS3;YOD1;DIEXF;PIAS1;ADCY9;FCHSD2;TET3;TFAM;PPIC;PH
XA3;CNPPD1;AP2M1;GPR135;ARHGEF10;NAA30;WDR37;YOD1;GAPVD1;DIEXF;RUNX1;ADCY9;CRISP
2;GPR37;TTC33;DIO2;SORCS1;FNDC3A;PCDHA13;PCDHA12;GTPBP4;NAALADL2;DDX19B;NRG3;MY
IB3;PABPN1;CARNS1;RBM7;SMNDC1;PSPH;BCL11B;GATC;PDAP1;HELZ;DIEXF;GATAD2B;SP1;LCOR;
M3;SLC25A45;RBM7;PELO;SMNDC1;CPSF7;CBX5;GATC;SORD;KCNRG;GATAD2B;MAF;TMEM33;SP1;L
;SLC25A45;RBM7;PELO;RNF111;PSPH;UBL7;SWAP70;RNASE6;PAX5;GNL1;DIEXF;PIAS1;MAF;TMEM3
;PHF8;AMOT;RBM3;ADAMTS2;TMEM108;SNIP1;SCN5A;MACROD2;GPRASP2;C3ORF14;EOMES;CHST
KL;ARHGEF10;NAA30;PLA2G12A;ALG6;MMP2;GAB1;GAPVD1;RBMS1;FAM64A;NPTXR;PTGFR;GPM6
C1;GPR37;MMP2;PAX5;PCDHA13;PCDHA12;PCDHA11;DIEXF;PCDHA10;PIAS1;DHX40;DDX19B;ADCY9
N1A;IL13RA1;PCDHAC1;CXADR;ST6GAL2;SFMBT2;ANGEL2;PCDHA12;HPCAL4;PCDHA11;FOXP2;PCD
AVER2;HOXA3;SLC25A45;SCN1A;PCDHAC1;TMEM194B;ST6GAL2;ALG6;SFMBT2;OMD;RNASE6;DIO2;
3;IGLON5;FBXO3;SNIP1;PTGFRN;CNPPD1;SCN1A;PCDHAC1;MAPK1IP1L;MME;MMP2;CNPY1;GAPVD
C22A17;PGM3;PSPH;TSFM;HSDL1;RC3H1;C6ORF62;YOD1;PCDHA11;PCDHA10;DHX40;CRISPLD1;TE

D2;SH3GL2;C3ORF14;HS3ST3B1;CHST7;ST6GAL2;SWAP70;MYEOV;GAB3;FNDC3A;PAX5;PROX1;PCD
I;METTL16;UNKL;PELO;AP2M1;DGCR2;MAPK1IP1L;NAA30;FTSJ2;DIEXF;RBX1;TMEM33;ARMCX6;TBC
3;HPCAL4;BCMO1;MOB3C;CDC34;CRISPLD2;NOS1AP;CMPK1;FAM84A;ST6GALNAC3;PRTFDC1;RNF1
NAA30;WDR37;C3ORF62;MSL2;RC3H1;C6ORF62;C9ORF64;RNF126;TMEM33;TET3;ARMCX6;CRK;PAF
ORC1;TMEM194B;TMED8;C3ORF62;MSL2;LRRC40;ZFP91;ZFY;DIEXF;FAM101B;ACAP2;CDC34;TET3;T
1;UBL7;RAB2B;TSFM;PLA2G12A;ALG6;SORD;CBFA2T2;C9ORF64;SUMF1;RBX1;ADAM19;FAM101B;NR
H;KPNA1;IL13RA1;FNBP4;ANGEL2;RNASE6;ACSL6;GPR75;YOD1;FTSJ2;GTPBP4;MOB3C;ACAP2;CDC
25A45;SOX6;EOMES;ESCO1;TMOD3;RNASE6;GPR75;FTSJ2;HPCAL4;BCMO1;FAM133B;DHX40;SFRP
S;CTGF;RBM3;SART3;CAPZB;PGM3;HOXA3;SNIP1;PIM3;SCN5A;SLC25A45;AP2M1;CCDC50;ARHGEF
TMEM194B;ALG6;MMP2;SORD;ALG14;CBFA2T2;C9ORF64;CDYL2;SUMF1;FAM185A;CNKSR3;ARMCX
08;ARL5A;IL6R;ZNF366;PHACTR4;HSDL1;MMP2;USP2;C3ORF62;KIAA1462;GPR75;HPCAL4;EREG;PTF
AD;ING4;FAM154B;BSN;CTBS;CNPPD1;UBL7;GPR135;TSFM;DFFA;TMEM194B;HSDL1;WDR37;SORD;A
IOXA3;RSPO2;KIF1B;PRKG1;WLS;GPR135;CSNK1G3;GPR37;TPM3;ITPK1;DIO2;EBF1;ACSL6;TFEB;CN
POLG;FAM154B;SLC25A45;AP2M1;CSNK1G3;WDR37;TTC33;TFEB;RC3H1;PAX5;DIEXF;PIAS1;ACAP2;I
F14;KCNH1;ST6GAL2;DIO2;SORCS1;FNDC3A;PAX5;PROX1;PCDHA13;SHISA9;PCDHA12;ADRA2A;PCI
PH;C6ORF62;YOD1;DYNLL2;FAM101B;ARMCX6;TFAM;FAM64A;SLC24A2;SET;IGSF3;SRSF1;FUT11;C1
PSD4;SH3BGRL2;SOX6;PRKG1;SCN1A;TGM2;PCDHAC1;SGIP1;SFMBT2;TMOD2;EREG;DKK3;BCMO1;
OD2;TEAD1;WLS;PCDHAC1;EOMES;GPR37;TPM3;MMP2;USP2;TAP2;GPR75;GAPVD1;FAM101B;CDC
B;MACROD2;C3ORF14;TMED8;SWAP70;VASH2;DIO2;SORD;HAUS3;PAX5;DHX40;ENAH;RNF126;TMEI
EF10;TSFM;C21ORF59;SLC30A4;C3ORF62;SORD;FTSJ2;DIEXF;SUMF1;FAM101B;ADCY9;KIAA1614;AF
;ALG6;TTC33;BROX;RNASE6;SORD;SLC39A13;HCFC2;DIEXF;C9ORF64;SUMF1;SUB1;FAM185A;ARMC
TSFM;TMEM194B;TTC33;C21ORF59;ALG14;GPR75;SENP2;C9ORF64;SUMF1;RUNX1;FAM101B;DDX19
SNRPD3;TGM2;KLF10;PRKAB2;MBNL2;TMOD3;SWAP70;C3ORF62;RC3H1;ANO6;FNDC3A;GAPVD1;FT
G14;SLC39A13;SUMF1;DHX40;ADAM19;RRM2B;FAM185A;ARMCX6;ASPHD2;FAM64A;TMEM106A;SLC
GM2;PCDHAC1;PCYT1B;HSDL1;RNASE6;GAB3;SORCS1;IL17RD;ADRA2A;PCDHA10;NRG3;IL1RAPL1;
ATXN7;C10ORF2;SMNDC1;AP2M1;C3ORF14;PSPH;CPSF7;CBX5;FZD5;CSNK1A1;RC3H1;PDAP1;C6OF
ORD;ALG14;SENP2;C9ORF64;SUMF1;FAM101B;CREB1;RBL1;FAM185A;ARMCX6;DCX;TERF2IP;SLC2
RBM7;PELO;CNPPD1;TSFM;TAP2;YOD1;GNL1;DIEXF;FAM101B;MAF;FCHSD2;TMEM33;TET3;LCOR;LF
L7;IL13RA1;TSFM;CXADR;CHST7;HSDL1;ALG6;SORD;ALG14;PROX1;DHX40;ARMCX6;FAM84A;COMM
NRPD3;SCN5A;TGM2;MAPK1IP1L;MME;VPS13C;SWAP70;SLC39A13;ZBTB34;PAX5;PCDHA12;DHX40;
LG6;SORD;ALG14;C9ORF64;SUMF1;DHX40;FAM101B;KIAA1614;FAM185A;ARMCX6;TERF2IP;FAM64A
;ARHGEF10;HAUS3;DIEXF;GTPBP4;PIAS1;ACAP2;TET3;TFAM;PGAM5;ST6GALNAC3;SET;NECAB3;GF
A;WDR37;ALG6;NSUN3;SORD;SLC39A13;SUMF1;RBL1;TCEANC2;TBC1D25;TERF2IP;ANKRD40;SCG3;
D;ALG14;FTSJ2;C9ORF64;SUMF1;DHX40;FAM101B;RBL1;FAM185A;TET3;ARMCX6;ZNF318;ASPHD2;F
30;AHSA2;PCDHA12;DYNLL2;PCDHA11;TMEM33;LCOR;ASPHD2;LPHN2;TMEM106A;BHLHB9;DLX6;SF
5;SOX6;ARHGEF10;PCYT1B;NSUN3;ACSL6;DHX40;CRISPLD1;ADAM12;ST6GALNAC3;TMEM106A;SLC
IDC17;ZMPSTE24;RNF214;DFNB59;MIER3;SIN3A;FAM73A;PCBD2;LRIG2;SNX8;CNPPD1;ZC3H14;UBL7
SESN3;PABPN1;C10ORF2;UNKL;GPRASP2;AGFG2;UBL7;TMEM194B;HSDL1;TIMMDC1;TMED8;ALG6;S
A;MFSD5;VTI1A;C10ORF2;SNIP1;ZNF800;PIM3;BCL2L2-PABPN1;CNPPD1;CBX5;FZD5;CSNK1A1;GATC
LIT2;BCL2L2-PABPN1;CSNK1A1;RC3H1;YOD1;GAPVD1;FAM101B;MARCKS;ADCY9;SNPH;TET3;TARDI
A1L1;EFNB3;PABPN1;MGAT5;RPA4;TET3;BCL2L2-PABPN1;TARDBP;RAD9A

NDC3A;HCFC2;RBX1;PIAS1;FAM133B;CDC37;SUB1;FAM185A;SIK3;CDIP1;WDFY3;RAF1;CGGBP1;MTI
3;MAF;ADCY9;TBL1XR1;SUB1;FAM185A;SIK3;CDIP1;SLC25A51;TFAM;WDFY3;DENND6A;RAF1;CGGBI
SA9;PCDHA11;FOXP2;SFRP1;MAF;CRISPLD2;HECW2;ADAM12;SIK2;KANK4;SHC4;DLX1;SHC2;PFKFB
L6;ATP11A;POU3F1;SHISA9;HPCAL4;DKK3;ADAM19;HECW2;ADAM12;NPTXR;KANK4;PPIC;SHC4;DLX
DC1;UBA6;HSPA4L;DCUN1D1;GSKIP;UBE2J1;NCKIPSD;BCL2L13;ZMAT3;MLEC;HIVEP2;SNX8;CREBBI
2;MAF;CRISPLD1;CRISPLD2;FAM84A;SCG3;LPHN2;TRIB2;MAPRE2;PTGFR;HPGD;PLAG1;PDE1B;SH
OXP2;SFRP1;HECW2;ELMO1;ASPHD2;NPTXR;KANK4;KCTD16;SHC4;DLX1;TSHZ3;IGSF3;DLX6;RNF1
1;ACSL6;POU3F1;SHISA9;HPCAL4;FOXP2;DKK3;ADAM19;SFRP1;MAF;HECW2;WDFY3;NPTXR;KANK
9;ADAM19;GPRIN3;SFRP1;C1ORF115;CRISPLD2;HECW2;ELMO1;NPTXR;KANK4;SHC4;DLX1;GPM6A;
GGBP1;SET;DLX6;SPATA2;DTX4;MED12L;NKAIN1;ARHGAP20;LONRF3;RAI2;BCL11B;SMURF2;FAM78
ID5B;FTSJ2;HPCAL4;FOXP2;MAF;CRISPLD1;CRISPLD2;TBL1XR1;FAM185A;CGGBP1;KCTD16;PPIC;S
FRP1;MAF;CRISPLD2;HECW2;NPTXR;KANK4;PPIC;DLX1;SHC2;IGSF3;DLX6;SHOX2;NPR3;GLIS3;AA
CW2;FAM185A;ADAM12;SCG3;PPIC;PTGFR;SHOX2;RHOBTB3;DTX4;ZNF25;C17ORF96;NKAIN1;ZMAT
6;SHOX2;NPR3;GATA6;GLIS3;FBLN5;GJC1;PRDM16;HOXC8;TFAP2B;EGR4;FZD5;BCL11B;NEK7;GDF
2;TPM3;SFMBT2;TMOD2;SLC30A4;FNDC3B;GPR75;ARID5B;SYTL4;EREG;DKK3;SFRP1;ADCY9;CRISP
GBP1;PTGFR;MTMR3;YTHDC1;UBA6;CUL3;SPATA2;MED12L;ZMPSTE24;NPAT;BCL2L13;MCMBP;CLS
HCFC2;HPCAL4;RUNX3;FOXP2;BCMO1;RUNX1;ADCY9;TBL1XR1;FAM185A;WDFY3;KCTD16;PPIC;DL
;ATP11A;FNDC3A;SHISA9;SYTL4;PCDHA10;RBX1;EREG;SFRP1;MAF;CRISPLD2;HECW2;ADAM12;EL
75;PCDHA13;RUNX3;PCDHA10;GPRIN3;ADCY9;KIAA1614;HECW2;FAM185A;ZNF318;SIK3;CGGBP1;K
T6GAL2;SLC30A4;FNDC3B;VASH2;POU3F1;PCDHA12;PCDHA10;RBX1;BCMO1;ADCY9;CRISPLD1;HE
DFY3;CGGBP1;SHC4;YTHDC1;UBA6;PDE1B;CUL3;HSPA4L;MTMR9;SPATA2;CREBL2;DTX4;NPAT;VTI1
PCDHA10;BCMO1;ADCY9;CRISPLD2;HECW2;FAM185A;ZNF318;SIK3;SCG3;CGGBP1;KCTD16;PTGFR
SP2;VASH2;ACSL6;ALG14;HCFC2;BCMO1;FAM133B;ADAM19;GPRIN3;MOB3C;SUB1;FAM185A;TBC1D
K3;SFRP1;MAF;HECW2;KANK4;SET;PFKFB3;DLX6;SHOX2;NPR3;GATA6;DTX1;SLC1A4;ZBTB4;TRHDE
3ST3B1;CSNK1G3;CXADR;WDR37;TMED8;TMOD3;BROX;DNMT3A;CMC1;FNDC3B;SORD;ARID5B;ZF
2;C9ORF64;EREG;BCMO1;MAF;HECW2;FAM185A;ADAM12;ZNF318;CGGBP1;KCTD16;SHC4;IGSF3;CU
M19;ADCY9;TBC1D25;WDFY2;NPTXR;TRIB2;GLYR1;IGSF3;YTHDC1;PDE1B;GLIS3;SPATA2;C1ORF52;
M185A;ZNF318;WDFY3;CGGBP1;KCTD16;IGSF3;PDE1B;DLX6;RNF180;SPATA2;DTX4;ARHGAP20;PRD
H3GL2;KCMF1;RAB2B;TMEM194B;ST6GAL2;SWAP70;DYRK1A;ARID5B;PCDHA10;EREG;BCMO1;ADA
APVD1;EREG;BCMO1;GPRIN3;KIAA1614;HECW2;FAM185A;ZNF318;SIK3;CGGBP1;PTGFR;TBC1D19;
Y3;SCG3;CGGBP1;KCTD16;PTGFR;GPM6A;TBC1D19;DLX6;NPR3;SPATA2;NKAIN1;ARHGAP20;MLEC;
HDC1;UBA6;PDE1B;CUL3;HSPA4L;FUT11;DCUN1D1;SPATA2;DTX4;GIGYF2;ATXN7;RNF217;VTI1A;MC
;LHX6;TRIB2
D5;YTHDC1;HSPA4L;DCUN1D1;SPATA2;ZBTB4;BCL2L13;RNF214;RIC8B;AP5M1;GSE1;RBM12;SRPK2
E1B;DLX6;CUL3;SHOX2;RHOBTB3;ARHGAP20;EFNB3;MLEC;C9ORF171;PCDHA3;PCDHA6;TMEM86A
Y1;ZBTB34;POU3F1;SHISA9;HPCAL4;FOXP2;GPRIN3;C1ORF115;NPTXR;KANK4;DLX1;GPM6A;IGSF3
;HSPA4L;MTMR9;HSPH1;RAP1A;RIC8B;ERI1;ZMAT3;MLEC;HIVEP2;SNX6;EDEM3;B3GALNT2;MCTS1;
85A;ZNF318;SIK3;KCTD16;IGSF3;DLX6;CUL3;SHOX2;MTMR9;SPATA2;ZNF25;ZBTB4;C17ORF96;TRHD
2;FAM185A;SIK3;CGGBP1;KCTD16;DLX1;BHLHB9;IGSF3;DLX6;CUL3;SPATA2;ZNF25;MED12L;ARHGA
IK3;KCTD16;SHC4;DLX6;ABHD2;CUL3;SHOX2;GATA6;SPATA2;ZBTB4;TRHDE;MED12L;HSPH1;MIER3;
K2;GLYR1;TBCEL;DCUN1D5;YTHDC1;UBA6;TNKS;AGAP2;DCUN1D1;SPATA2;GSKIP;GIGYF2;BCL2L13
;HECW2;FAM185A;SIK3;CGGBP1;KCTD16;IGSF3;PDE1B;CUL3;NPR3;SPATA2;ZNF25;ARHGAP20;PRD
HA12;DHX40;MAF;HECW2;FAM185A;ZNF318;SIK3;KANK4;GPM6A;IGSF3;PDE1B;CUL3;AGAP2;ATXN7;
RMCX6;CGGBP1;SRSF9;AGAP2;RHOBTB3;TMTC3;C1ORF52;NCKIPSD;RIC8B;ATXN7;ZMAT3;VTI1A;RE
YR1;SET;BHLHB9;HSPA4L;TMTC3;CREBL2;ZBTB4;TRHDE;FBLN5;BCL2L13;VTI1A;PCDHA4;LONRF3;R
C1D19;HSPA4L;C1ORF52;ZBTB4;FBXO40;RNF214;ARHGAP20;ZNF706;VTI1A;IP6K1;N4BP2;RAB11FIP
AP2K1;TMOD3;ANGEL2;DYRK1A;ALG14;ARID5B;HPCAL4;C9ORF64;EREG;BCMO1;FAM133B;C1ORF1
ADCY9;CRISPLD2;HECW2;FAM185A;ZNF318;SIK3;CGGBP1;KCTD16;TBC1D19;IGSF3;CUL3;SHOX2;M
IPCAL4;PCDHA11;FOXP2;EREG;ADAM19;SFRP1;CRISPLD2;HECW2;ADAM12;ASPHD2;FAM64A;SCG3
1185A;CGGBP1;KCTD16;PDE1B;NPR3;SPATA2;DTX4;FBLN5;ARHGAP20;MLEC;C9ORF171;SCN3B;ZNF
PELO;SH3GL2;C3ORF14;WLS;KCNH1;EOMES;CXADR;ST6GAL2;TPM3;SFMBT2;TMOD2;KIAA1462;AC
IDC1;UBA6;HSPA4L;DCUN1D1;MTMR9;SPATA2;CREBL2;DTX4;ZMPSTE24;NPAT;CYB5R4;MIER3;VTI1A

LX1;FAM114A1;ABHD4;UBA6;PDE1B;GATA6;DCUN1D1;UBE2J1;RNF214;ARHGAP20;RIC8B;G3BP1;SC
;TSHZ3;DCUN1D5;HSPA4L;FUT11;MTMR9;APCDD1;NPAT;RNF214;ATXN7;VTI1A;MCMBP;GSE1;SRGAF
;MTMR9;MED12L;UBE2J1;HSPH1;PRDM16;VTI1A;C9ORF171;PCDHA2;PCDHA7;TMEM86A;BCL11B;FA
194B;SWAP70;BROX;ANGEL2;CMC1;ALG14;ARID5B;ATP11A;FTSJ2;HPCAL4;PIAS1;CRISPLD2;CDC37
GPM6A;PDE1B;CUL3;NPR3;RHOBTB3;APCDD1;ARHGAP20;MIER3;LONRF3;HIVEP2;MYH10;TMEM86A
;BCMO1;HECW2;FAM185A;SIK3;CGGBP1;KCTD16;PDE1B;DLX6;CUL3;SHOX2;GATA6;SPATA2;ZBTB4;F
F318;SIK3;FAM64A;CGGBP1;KCTD16;TBC1D19;DLX6;CUL3;SHOX2;SPATA2;ARHGAP20;ATXN7;PRDM1
P1;HECW2;ADAM12;NPTXR;SHC4;PTGFR;IGSF3;PDE1B;RNF180;SHOX2;GLIS3;C17ORF96;TRHDE;ME
PVD1;FAM133B;MAF;CDC37;SUB1;TCEANC2;TBC1D25;CGGBP1;PPIC;TBC1D19;YTHDC1;UBA6;ABHD
ORF64;GTPBP4;DKK3;RNF126;TBL1XR1;TBC1D25;COMMD3-BMI1;WDFY3;TRIB2;KANK4;GLYR1;TBCI
AL2;TMOD2;VASH2;EBF1;ACSL6;POU3F1;HPCAL4;FOXP2;EREG;SFRP1;MAF;CRISPLD2;HECW2;ADA
M133B;RNF126;C1ORF115;CMPK1;SCG3;PPIC;IGSF3;DLX6;GLIS3;FBXO40;APH1B;VTI1A;MCMBP;SC
1A4;GJC1;BCL2L13;HSPH1;RIC8B;ERI1;B3GALNT2;MCTS1;EIF2B2;XRCC5;NEK7;GFPT1;MRPL27;EPT
;UBA6;ABHD2;HSPA4L;MTMR9;CREBL2;ZMPSTE24;NPAT;ZMAT3;VTI1A;MCMBP;RBM12;SRGAP2;RAB
;ADAM12;KANK4;DLX1;PTGFR;GPM6A;SHC2;PDE1B;RNF180;SHOX2;NPR3;HSPA4L;GLIS3;SLC1A4;TF
;MAF;CRISPLD2;KANK4;DLX1;GPM6A;SHC2;RNF180;NPR3;GATA6;GLIS3;APCDD1;DTX4;TRHDE;FOX
11;DCUN1D5;UBA6;ZBTB4;ZMPSTE24;MTDH;BCL2L13;ERI1;VTI1A;HIVEP2;RIMKLA;ZNF148;EDEM3;E
Y9;CDC37;SUB1;ARMCX6;ELMO1;CGGBP1;KANK4;PPIC;YTHDC1;UBA6;PDE1B;ARL3;HSPA4L;FUT11;
M185A;ZNF318;SIK3;CGGBP1;GPM6A;PDE1B;CUL3;SHOX2;SPATA2;ZNF25;TRHDE;ARHGAP20;ATXN7
TC3;ZNF25;ZBTB4;RIC8B;ZNF706;VTI1A;MCMBP;SCN3B;N4BP2;PCDHA7;EDEM3;CREBBP;TMEM86A;
K1A;ALG14;HECW2;FAM185A;ARMCX6;WDFY3;CGGBP1;IGSF3;CUL3;SHOX2;MTMR9;SPATA2;DTX4;
DR;ST6GAL2;C21ORF59;USP2;KIAA1462;VASH2;ARID5B;FTSJ2;HCFC2;C9ORF64;BCMO1;CRISPLD2
CDC50;EOMES;CXADR;TMED8;SGIP1;C21ORF59;ANGEL2;ALG14;ZBTB34;GAB3;PCDHA13;FTSJ2;ZF
GBP1;KANK4;PPIC;PTGFR;YTHDC1;UBA6;PDE1B;FUT11;MTMR9;DTX1;RNF217;G3BP1;VTI1A;MCMBP
9;TBCEL;BHLHB9;FAM114A1;YTHDC1;UBA6;ARL3;MTMR9;ZNF25;NCKIPSD;NPAT;APH1B;ZNF706;VTI1
HD4;CUL3;TMTC3;SLC1A4;NPAT;ARHGAP20;APH1B;RIC8B;ZMAT3;G3BP1;HIVEP2;RIMKLA;SNX6;PLX
LHB9;YTHDC1;PDE1B;ABHD2;TMTC3;CREBL2;BCL2L13;RAP1A;MCMBP;PCDHA3;ZC3H14;PCDHA7;E
R;DGCR2;EOMES;HS3ST3B1;CXADR;WDR37;TMED8;TMOD3;NSUN3;ZBTB34;HCFC2;CDYL2;ADRA2

HECW2;FAM185A;SIK3;CGGBP1;KCTD16;TBC1D19;IGSF3;UBA6;DLX6;RNF180;CUL3;SHOX2;SPATA2;
DC37;ZNF318;TFAM;WDFY3;SCG3;CGGBP1;GLYR1;TBC1D19;BHLHB9;SPATA2;CREBL2;ZBTB4;ZMP
AP2;APCDD1;ZNF25;NPAT;BCL2L13;RAP1A;APH1B;EFNB3;ZMAT3;VTI1A;SCN3B;RIMKLA;ZNF148;SN
TMTC3;ZMPSTE24;NPAT;BCL2L13;MIER3;G3BP1;VTI1A;IP6K1;N4BP2;FZD3;EIF2B2;ST13;GFPT1;MRPL
DCY9;HECW2;FAM185A;ZNF318;WDFY3;KCTD16;SHC4;DLX6;CUL3;SPATA2;DTX4;NCKIPSD;ARHGAP
;BCMO1;RUNX1;KIAA1614;FAM185A;ZNF318;CGGBP1;KCTD16;PTGFR;GPM6A;IGSF3;CUL3;RNF214;
3A;PAX5;PROX1;ZFX;CDYL2;KAT2B;FAM185A;CNKSR3;LCOR;RBMS1;MAPRE2;CGGBP1;FYTTD1;PPI
F318;SIK3;SCG3;CGGBP1;KCTD16;IGSF3;DLX6;ABHD2;CUL3;ZNF25;MED12L;ATXN7;PRDM16;PCDHA
HSPA4L;SPATA2;GJC1;ATXN7;MCMBP;SRGAP2;LYN;ZBTB14;XRCC5;ZBTB16;NEK7;MRPL27;HELZ;MA
1;FNDC3A;SIK3;WDFY2;SET;TNKS;ABHD2;AGAP2;GIGYF2;GJC1;SNX1;ERI1;MCMBP;C9ORF171;ABC
;SET;FAM114A1;ABHD4;CUL3;SHOX2;DTX4;MLEC;ABCA1;B3GALNT2;MCTS1;ABCA2;TMEM86A;FAM7
R;DCUN1D1;TMTC3;CREBL2;ZBTB4;TRHDE;NPAT;BCL2L13;APH1B;ZNF706;VTI1A;C9ORF171;PCDHA
2;WLS;KCNH1;PCDHAC1;EOMES;ST6GAL2;TPM3;SFMBT2;EBF1;ACSL6;CNPY1;ARID5B;SYTL4;FOXP
P4;EREG;FAM133B;HECW2;SIK3;FAM64A;CGGBP1;KCTD16;PTGFR;PDE1B;SHOX2;ZNF25;ZMPSTE24
HAC1;FNBP4;CXADR;ST6GAL2;C21ORF59;SFMBT2;SLC30A4;KIAA1462;VASH2;EBF1;ACSL6;CNPY1;P
C1D25;COMMD3-BMI1;TFAM;TNKS;NPR3;GATA6;FOXO1;MED12L;ZMPSTE24;NKAIN1;CYB5R4;EFNB3
;TBC1D19;PDE1B;RHOBTB3;C17ORF96;FBXO40;PCDHA1;RBM12;RIMKLA;MYH10;ZC3H14;PPARGC1
YF2;FBLN5;SNX1;ZNF706;ATXN7;MIER3;MCMBP;MLEC;SCN3B;ZC3H14;PLXNA3;CBX5;TIPARP;SRD5
;ARL3;GATA6;TRHDE;FBLN5;APH1B;RIC8B;ZNF706;TAC3;SCN3B;RIMKLA;PCDHA8;RAI2;EDEM3;CBX6
TNKS;GLIS3;TMTC3;SPATA2;CREBL2;C1ORF52;DTX4;VTI1A;PLXNA3;EIF2B2;ST13;GFPT1;MRPL27;NF
2;EREG;PTP4A1;KAT2B;RBL1;FAM185A;WDFY4;PRTFDC1;PPIC;FYTTD1;GPM6A;TBC1D19;RALA;DLX
Y3;ANKRD40;FAM64A;WDFY2;NPTXR;WDFY4;CGGBP1;KANK4;PPIC;DLX1;TBC1D19;NECAB3;FAM11
M2;PCDHAC1;EOMES;RAB2B;TMEM194B;ST6GAL2;USP2;KIAA1462;DYRK1A;GPR75;ARID5B;PCDHA
10;SNX6;EDEM3;CREBBP;RABGAP1L;CBX5;XRCC5;ST13;NEK7;NBEAL1;LNPEP;TRAPPC8;LSM5;ABH

UT11;MTMR9;SPATA2;CREBL2;NCKIPSD;NPAT;G3BP1;VTI1A;RBM12;LYN;EDEM3;EIF2B2;ZBTB14;XRC
X6;TFAM;WDFY3;NPTXR;CGGBP1;KANK4;PPIC;PTGFR;BHLHB9;YTHDC1;UBA6;PDE1B;FUT11;DTX1;M
HDC1;UBA6;PDE1B;HSPA4L;FUT11;MTMR9;SPATA2;RNF217;G3BP1;MCMBP;EDEM3;CBX5;ZBTB14;NE
;KCMF1;PRKAB2;TMEM194B;CXADR;BROX;USP2;DYRK1A;EBF1;ALG14;GPR75;ARID5B;FTSJ2;C9OR
14;FNDC3A;EREG;FAM133B;GPRIN3;CDC37;SUB1;TCEANC2;TBC1D25;WDFY3;CGGBP1;YTHDC1;UB
2;PCDHAC1;TNS1;KCMF1;PRKAB2;CXADR;TPM3;C21ORF59;BROX;USP2;VASH2;EBF1;ALG14;ARID5E
G3;CGGBP1;KCTD16;ABHD2;CREBL2;DTX4;ARHGAP20;C9ORF171;SCN3B;N4BP2;ZC3H14;PCDHA7;T
A2A;TGFBR3;SFRP1;SNPH;CRISPLD2;NOS1AP;SMTNL2;NAT8L;KANK4;KCTD16;SHC2;ADCYAP1R1;P
H1B;RIC8B;VTI1A;RIMKLA;PLXNA3;MARK1;ABCA1;MCTS1;CBX5;ST13;NEK7;NBEAL1;SMAD9;TRAPP
115;CRISPLD1;CDC37;ELMO1;WDFY2;AIPL1;KANK4;SHC2;ADCYAP1R1;DLX6;RNF180;NPR3;TRHDE;F
ATA2;MCMBP;SCN3B;LYN;CBX5;EDEM1;NEK7;GFPT1;PARVA;BFAR;BTBD9;SMAD7;CNOT6;FUBP1;CA
1;TFAM;WDFY3;PPIC;TBC1D19;NECAB3;YTHDC1;UBA6;ZBTB4;BCL2L13;HSPH1;RIC8B;VTI1A;MLEC;N
ID3-BMI1;TFAM;WDFY3;CGGBP1;SHC4;GPM6A;BHLHB9;NPR3;HSPA4L;TMTC3;CREBL2;APCDD1;APH
;ADCY9;KIAA1614;FAM185A;CGGBP1;KCTD16;TBC1D19;IGSF3;PDE1B;NPR3;SPATA2;DTX4;NKAIN1;R
PCDHA13;GAPVD1;PCDHA12;HPCAL4;PCDHA10;RUNX1;DHX40;FAM64A;SCG3;WDFY2;TBC1D19;PSM
1B;HSPA4L;RNF214;PCDHA1;ZMAT3;PCDHA4;GSE1;HIVEP2;PCDHA9;PCDHA7;SNX6;MARK1;CADM3;
DHA11;PCDHA10;LHX4;FKBP6
POU3F1;PCDHA12;PCDHA11;PCDHA10;DKK3;BCMO1;ADCY9;CRISPLD1;HECW2;ADAM12;ELMO1;FAM
AF;CRISPLD2;COMMD3-BMI1;WDFY3;FAM64A;SCG3;CGGBP1;KANK4;ZNF275;DLX1;ADCYAP1R1;BHL
NPR3;MTMR9;DTX4;NCKIPSD;ARHGAP20;G3BP1;MCMBP;C9ORF171;CLSPN;SMURF2;TIPARP;ST13;

4;PPIC;PTGFR;PFKFB3;YTHDC1;UBA6;PDE1B;FUT11;DCUN1D1;MTMR9;CREBL2;NPAT;ATXN7;PCDHA
6;CUL3;MTMR9;SPATA2;DTX4;NPAT;PRDM16;VTI1A;MCMBP;RBM12;PLXNA3;EDEM3;EIF2B2;ZBTB14;X
25A51;WDFY3;CGGBP1;KANK4;GLYR1;TBC1D19;FAM114A1;PDE1B;ARL3;CUL3;SLC1A4;CYB5R4;APH
ADR;C5ORF15;C21ORF59;ANGEL2;USP2;KIAA1462;EBF1;ALG14;ARID5B;YOD1;PCDHA13;FTSJ2;HPC
P1;SNPH;CRISPLD2;MAPRE3;SMTNL2;NAT8L;FKBP6;KCTD16;RNF165;PPM1H;DTX1;NDNF;HOXD12;
HB9;RNF180;TMTC3;CREBL2;FBXO40;APH1B;VTI1A;N4BP2;EDEM3;FZD3;CBX6;CPSF7;EIF2B2;CCZ1
D1;TMTC3;MTMR9;SPATA2;FBLN5;ZMPSTE24;FBXO40;VTI1A;MCMBP;SNX8;GFPT1;MRPL27;FBXO30;
T5;RABGAP1L;TIPARP;GFPT1;ST8SIA3;PARVA;BFAR;L1CAM;BTBD9;AZIN1;PUM2;SMAD7;EFNA3;FUB
KFB3;BHLHB9;FAM114A1;TNKS;TMTC3;CREBL2;NPAT;ZMAT3;IP6K1;SNX8;PLXNA3;B3GALNT2;ST13;
3;MTMR9;NKAIN1;APH1B;RIC8B;VTI1A;SCN3B;N4BP2;ABCA2;CREBBP;TMEM86A;CCZ1B;EGR4;FZD5
217;PRDM16;PCDHA5;C9ORF171;PCDHA3;PCDHA2;SCN3B;RIMKLA;PCDHA8;PCDHA7;EDEM3;B3GAL
EOMES;CXADR;MBNL2;TPM3;SFMBT2;TMOD2;USP2;CNPY1;PCDHA13;SHISA9;FOXP2;GPRIN3;SFRP
N1D5;TNKS;HSPA4L;RHOBTB3;DCUN1D1;ZMPSTE24;BCL2L13;AP5M1;VTI1A;MLEC;EDEM3;CBX6;CB
1;WDFY3;PTGFR;YTHDC1;UBA6;PDE1B;HSPA4L;MTMR9;EFNB3;ATXN7;RNF217;VTI1A;MCMBP;ABCA
RHDE;MED12L;NCKIPSD;BCL2L13;APH1B;ERI1;ZMAT3;VTI1A;MCMBP;IP6K1;SCN3B;ZNF148;GFPT1;S
CTN4;HPCAL4;SYTL4;DKK3;RUNX1;ADAM19;SFRP1;CRISPLD1;CRISPLD2;CDC37;HECW2;ADAM12;A
MOD2;SLC30A4;FNDC3B;GPR75;PCDHA12;SYTL4;FOXP2;PCDHA10;EREG;GPRIN3;SFRP1;ELMO1;S
3;C1ORF52;FBLN5;BCL2L13;RAP1A;PLXNA2;C9ORF171;PCDHA3;ZC3H14;B3GALNT2;SRD5A1;GFPT
FY3;ANKRD40;BHLHB9;UBA6;ARL3;TMTC3;SPATA2;NCKIPSD;NPAT;APH1B;VTI1A;C9ORF171;N4BP2;
IC;PTGFR;SHC2;NECAB3;YTHDC1;UBA6;PDE1B;ARL3;HSPA4L;MTMR9;MED12L;VTI1A;MCMBP;GSE1
CDC37;CDIP1;PPIC;SHC4;YTHDC1;FUT11;MTMR9;APCDD1;PRDM16;MCMBP;SCN3B;MARK1;EIF2B2;Z
6;BROX;USP2;VASH2;ALG14;ARID5B;ATP11A;FTSJ2;HCFC2;HPCAL4;FOXP2;C9ORF64;RBX1;EREG;B
ROX;NSUN3;AHSA2;SORD;PROX1;HCFC2;C9ORF64;EREG;PTP4A1;KAT2B;ADCY9;KIAA1614;SNPH;F
D2;FAM64A;BHLHB9;ARL3;NPR3;ZBTB4;UBE2J1;APH1B;EFNB3;ATXN7;PLXNA2;SCN3B;RIMKLA;MYH
M1;SRGAP2;ZC3H14;MCTS1;ACBD5;MRPL27;HOOK3;BTBD9;AZIN1;PUM2;HNRNPK;CAPRIN1;CDK1;
33;ABHD2;SPATA2;SLC1A4;C17ORF96;NCKIPSD;NPAT;BCL2L13;APH1B;RIC8B;ZNF706;VTI1A;C9ORF1
1R1;TBCEL;BHLHB9;RNF180;ABHD2;NPR3;AGAP2;GLIS3;SLC1A4;FOXO1;EFNB3;SCN3B;RIMKLA;N4
PLD2;FAM185A;COMMD3-BMI1;KCTD16;BHLHB9;ABHD4;TNKS;ABHD2;HSPA4L;TMTC3;UBE2J1;BCL2
XP2;GTPBP4;CDC34;CRISPLD2;NPTXR;TBCEL;PFKFB3;IGSF3;DLX6;DCUN1D1;SPATA2;ZBTB4;ARHG
I6A;FAM114A1;RNF180;HSPA4L;RHOBTB3;APCDD1;TRHDE;FBLN5;ARHGAP20;APH1B;CHAD;PRDM1
RP1;ELMO1;NPTXR;KANK4;DLX1;PTGFR;GPM6A;PDE1B;DLX6;SHOX2;NPR3;AGAP2;DTX1;C17ORF96
I;TBCEL;BHLHB9;MTMR9;CREBL2;HSPH1;RAP1A;VTI1A;C9ORF171;PCDHA3;PCDHA7;TIPARP;SRD5A

;TFAM;WDFY3;YTHDC1;UBA6;HSPA4L;MTMR9;SPATA2;DTX4;MCMBP;CLSPN;PLXNA3;SRPK2;EIF2B2
IR3;RNF180;GATA6;MTMR9;UBE2J1;BCL2L13;CYB5R4;RNF214;RIC8B;PCDHA3;RBM12;ST13;EDEM1;Z
BA6;ABHD2;MTMR9;SPATA2;ZMPSTE24;NPAT;VTI1A;IP6K1;RBM12;ZBTB14;SMURF2;XRCC5;MRPL27;
3C1D25;CGGBP1;YTHDC1;UBA6;TMTC3;MTMR9;SPATA2;DTX4;GIGYF2;ZMPSTE24;VTI1A;PLXNA2;AB

L2L13;RNF214;AP5M1;SNX6;EDEM3;CBX5;EDEM1;MRPL27;SREK1IP1;BFAR;INHBA;LSM5;AZIN1;ZFP
;GSE1;SRGAP2;MARK1;MCTS1;CBX5;ZBTB14;FZD5;ZBTB16;SMAD9;NSG1;SYT7;PARP11;ALDH6A1;N
;FBLN5;UBE2J1;AP5M1;RAB11FIP5;SNX6;EDEM3;MCTS1;CPSF7;ZBTB14;ST13;GFPT1;SREK1IP1;HEL
I1;SCN3B;RAI2;CPSF7;CBX5;EDEM1;MRPL27;PARVA;BFAR;HOOK3;PHOX2B;PUM2;SMAD7;RCAN2;FU
REBL2;UBE2J1;NPAT;BCL2L13;APH1B;RIC8B;VTI1A;C9ORF171;N4BP2;MARK1;B3GALNT2;CPSF7;FZI
214;VTI1A;MCMBP;MLEC;PCDHA3;LONRF3;IP6K1;MARK1;EDEM3;CPSF7;EIF2B2;MAGEE1;MRPL27;B
K1;N4BP2;EDEM3;TMEM86A;FZD5;ST13;GFPT1;NBEAL1;RASSF8;SREK1IP1;LNPEP;TRAPPC8;HOOK
;BCMO1;ADCY9;KIAA1614;TBL1XR1;HECW2;FAM185A;WDFY3;CGGBP1;PPIC;PTGFR;GPM6A;BHLHB
54;GPRIN3;KIAA1614;HECW2;ELMO1;SCG3;KANK4;GPM6A;SHC2;RNF180;NPR3;GLIS3;RHOBTB3;DT
R1;TBC1D19;DCUN1D5;UBA6;HSPA4L;DCUN1D1;TRHDE;NPAT;RNF214;APH1B;RIC8B;AP5M1;ERI1;RA
N1D5;HSPA4L;ZBTB4;GIGYF2;MTDH;CYB5R4;RNF214;SNX1;APH1B;AP5M1;MIER3;N4BP2;LYN;EDEM3
;TPM3;SFMBT2;TMOD2;VASH2;ACSL6;CNPY1;POU3F1;SHISA9;FOXP2;GPRIN3;MAF;C1ORF115;ELMC
RL3;SHOX2;TMTC3;CREBL2;TRHDE;PRDM16;VTI1A;SCN3B;RIMKLA;PCDHA7;EDEM3;B3GALNT2;CB
/TI1A;C9ORF171;PCDHA3;SCN3B;RIMKLA;N4BP2;PCDHA7;PCDHA6;CPSF7;EGR4;FZD5;BCL11B;NFA
;GJC1;MCMBP;RBM12;SRPK2;EIF2B2;ZBTB14;ZBTB16;MRPL27;EPT1;BTBD9;PARP11;HNRNPK;OSTC
C3B;DIO2;EBF1;GPR75;ATP11A;SHISA9;HPCAL4;EREG;GPRIN3;SFRP1;CRISPLD2;KANK4;ADCYAP1
DL1;C21ORF59;MMP2;DNMT3A;SORD;PROX1;PCDHA10;PTP4A1;HECW2;FAM185A;IL1RAPL1;RBMS1
R;ST6GALNAC3;CGGBP1;FYTTD1;ABHD4;PALM2;SPATA2;ZBTB44;ADD3;LRP8;TRHDE;FAM117B;ZNR
RC40;ARID5B;ANO6;SORCS1;ZFY;SENP2;GTPBP4;EREG;PTP4A1;CDC37;CMPK1;CLDN18;ERGIC2;R
LRRC40;GAB3;CBFA2T2;SHISA9;SENP2;GTPBP4;ENAH;ARMCX6;TFAM;PGAM5;FAM64A;DTL;TRIB2;S
40;HAUS3;ARID5B;RBX1;FAM133B;ADCY9;RBL1;SUB1;NAA35;SLC25A12;CGGBP1;TBC1D19;SF3B3;S
;LRRC40;GPR75;ARID5B;PAX5;PROX1;CDYL2;ADRA2A;BCMO1;ENAH;RNF126;C1ORF115;SNPH;SUB
AB2B;USP47;NAA30;TMOD2;CNPY1;C6ORF62;GAB3;YOD1;PAX5;ZFY;SMC1A;ZFX;FAM133B;CDC34;C
RC40;PROX1;ADCY9;CDC37;NOS1AP;COMMD3-BMI1;TFAM;ASPHD2;WDFY3;NAA38;RAPGEF6;WDFY
TDC1;MAP3K9;SCN5A;MACROD2;BSN;SH3GL2;PCDHAC2;KCNH1;PCDHAC1;PCYT1B;CXADR;ST6GA
;DFFA;CHST7;TMED8;C21ORF59;TMOD3;SWAP70;CNPY1;ANO6;GAPVD1;DIEXF;PTP4A1;KAT2B;DDX
;ESCO1;ANGEL2;USP2;ALG14;GPR75;ARID5B;ATP11A;FNDC3A;HCFC2;C9ORF64;RBX1;EREG;BCMC
RF15;C21ORF59;TMOD2;USP2;KIAA1462;DYRK1A;ALG14;YOD1;HPCAL4;EREG;BCMO1;MAF;C1ORF
TR4;VKORC1;UTP15;MAPK1IP1L;TMEM194B;GPR37;SGIP1;ITPK1;ALG14;PROX1;CDYL2;EREG;RAP2
;FNBP4;KCMF1;RAB2B;WDR37;SWAP70;BROX;C3ORF62;ALG14;GPR75;ANO6;PROX1;PCDHA13;SM
3;EREG;ENAH;RBL1;RRM2B;TBL1XR1;FAM185A;SIK3;NAA38;SLC25A12;ZNF597;DCP2;PRTFDC1;KA
;HAUS3;GAPVD1;CBFA2T2;DHX40;ACAP2;CDC37;TET3;ARMCX6;CMPK1;LCOR;FAM64A;SLC25A12;R
NMT3A;DIO2;TAP2;SORD;GAB3;GPCPD1;GTPBP4;SUMF1;PIAS1;RUNX1;DHX40;ENAH;TGFBR3;RNF1
;AHSA2;ANO6;HCFC2;C1ORF115;RBL1;CMPK1;LCOR;ST6GALNAC3;PRTFDC1;KCTD16;HDAC5;RAL
HECW2;ADAM12;WDFY3;NPTXR;DLX1;TSHZ3;DLX6;NPR3;GATA6;DTX4;TRHDE;MED12L;NOP9;GJC1;
GPR135;PCDHAC1;GPR37;EBF1;CNPY1;PCDHA13;ZFY;POU3F1;SHISA9;ADRA2B;FOXP2;ADRA2A;D
C1;LRRC40;ARID5B;PROX1;SENP2;ZFX;SYTL4;GTPBP4;BCMO1;ENAH;KAT2B;RAP2A;SNPH;ADAM12;
;TMEM194B;TTC33;NSUN3;OMD;DYRK1A;GPR75;CBFA2T2;GTPBP4;SUMF1;RAP2C;PTP4A1;FAM185
BF1;ACSL6;LRRC40;CNPY1;SENP2;HPCAL4;GTPBP4;PAX2;DKK3;SNPH;NOS1AP;COMMD3-BMI1;WD
DHAC1;UTP15;MAPK1IP1L;TMEM194B;MAP2K1;ST6GAL2;MME;GRID1;NSUN3;ITPK1;ALG14;RC3H1;
REG;RUNX1;GPRIN3;ADCY9;CDC37;SUB1;ADAM12;ARMCX6;CDIP1;TFAM;WDFY3;CGGBP1;KANK4;P
GM2;FBXW4;HS3ST3B1;DFFA;CXADR;GPR37;CHST7;ST6GAL2;GRID1;VPS13C;NSUN3;ITPK1;SORD;
PR75;C6ORF62;PAX5;FTSJ2;SENP2;SYTL4;GTPBP4;ADRA2A;BCMO1;CRISPLD2;STIM2;NOS1AP;ANK
CBFA2T2;SENP2;GTPBP4;SUMF1;ADAM19;MOB3C;RAP2A;TBL1XR1;FAM185A;IL1RAPL1;NOS1AP;TB
CCHC24;MAPK1IP1L;TMEM194B;NSUN3;ITPK1;CMC1;ALG14;RC3H1;HAUS3;DIEXF;GTPBP4;CDYL2;S
B1;SLC39A13;HCFC2;CBFA2T2;SENP2;FAM84A;TFAM;WDFY3;SLC25A12;ZNF597;GLYR1;RALA;USP1
RAB2B;NAA30;WDR37;TTC33;YOD1;GAPVD1;HPCAL4;GTPBP4;ADAM19;ACAP2;ADCY9;CRISPLD2;C
PS13C;NSUN3;ALG14;HAUS3;CDYL2;KAT2B;FCHSD2;CDC37;ERGIC2;CRK;CGGBP1;SLC29A3;CHST3;

1;LRRC40;ATP11A;FTSJ2;SYTL4;DIEXF;C9ORF64;KAT2B;RAP2A;FAM185A;TFAM;PGAM5;WDFY3;NAA3
F1;SHISA9;HPCAL4;FOXP2;SUB1;TCEANC2;FAM64A;NPTXR;GLYR1;TBC1D19;BHLHB9;FAM114A1;SH
21ORF59;BROX;AHSA2;GAB1;KIAA1462;ALG14;SMC1A;CBFA2T2;HPCAL4;PGAM5;SCG3;SLC25A12;TF
ORF64;RAP2C;KAT2B;CRISPLD1;TBL1XR1;FAM185A;CNKSR3;NAA35;PGAM5;WDFY3;SLC25A12;DTL;
5;EDEM3;B3GALNT2;SRPK2;ST13;MRPL27;EPT1;NBEAL1;L1CAM;OCLN;STRN;DAND5;CC2D1B;MOCS

A4;APH1B;RIC8B;AP5M1;VTI1A;RAB11FIP5;PLXNA3;SRPK2;CREBBP;XRCC5;GFPT1;MRPL27;NFATC3;
;PRKAB2;CHST7;TMED8;TTC33;SWAP70;USP2;ARID5B;FNDC3A;GAPVD1;PIAS1;DHX40;PTP4A1;RAP
;LRRC40;CNPY1;FTSJ2;DIEXF;PAX2;DKK3;RAP2A;CRISPLD1;CRISPLD2;FAM84A;ANKRD40;FAM64A;S
BROX;SLC30A4;USP2;DNMT3A;GAB1;TFEB;GAPVD1;GPCPD1;RUNX3;PIAS1;BCMO1;PTP4A1;RAP2A;
M2;PCDHAC1;VKORC1;HS3ST3B1;UTP15;ZCCHC24;MAPK1IP1L;TMEM194B;SGIP1;TMOD3;ITPK1;ALG
IP1L;TMEM194B;GPR37;SGIP1;NSUN3;ITPK1;ALG14;PROX1;SHISA9;PCDHA12;PCDHA11;CDYL2;ERE
DIP1;ANKRD40;FAM64A;SCG3;FKBP6;GLYR1;FAM114A1;DCUN1D5;TNKS;PDE1B;ARL3;ABHD2;SHOX2
HX40;C1ORF115;CRISPLD2;ARMCX6;SCG3;KANK4;TBC1D19;TBCEL;SET;BHLHB9;NECAB3;YTHDC1;F
;ACTN4;IL17RD;ZFX;CDYL2;SUMF1;RAP2C;KAT2B;DDX19B;FCHSD2;CDC37;FAM84A;ERGIC2;CGGBP
G6;MME;GRID1;TPM3;USP2;GPR75;HAUS3;FTSJ2;SHISA9;ADRA2B;SUMF1;CRISPLD2;FAM84A;CDIP1
D1;PCDHAC1;ZCCHC24;TMEM194B;ST6GAL2;MBNL2;ALG6;GRID1;EBF1;GPR75;ANO6;GAPVD1;PCDH
;KIAA1462;SORD;EBF1;RC3H1;PROX1;CBFA2T2;HPCAL4;PAX2;DHX40;RAP2C;FAM101B;NOS1AP;CM
ID1;SLC30A4;DNMT3A;KIAA1462;EBF1;CNPY1;GAB3;PAX5;ZFY;IL17RD;SENP2;HPCAL4;FOXP2;CRISF
D1;CBFA2T2;RBL1;SLC25A51;TBC1D25;TFAM;TERF2IP;PGAM5;ANKRD40;FAM64A;ERGIC2;KANK2;D

;PGAM5;ANKRD40;MAPRE3;RAPGEF6;TMEM106A;SHC4;RALA;USP15;BHLHB9;HPGD;UBA6;DLX6;GA
X1;RAP2A;CRISPLD2;MYOD1;TET3;TERF2IP;CRK;SOS2;ZNF597;GLYR1;MTMR3;PFKFB3;ZDHHC20;P
3;PROX1;DIEXF;CDYL2;SNPH;FAM185A;COMMD3-BMI1;ASPHD2;PGAM5;WDFY3;ANKRD40;ST6GALN
BGRL2;SCN5A;BSN;PRKG1;KCNH1;MEF2A;EOMES;HS3ST3B1;PCYT1B;TMOD2;KIAA1462;ACSL6;TF
HAC1;UTP15;RAB2B;GRID1;GPR75;ARID5B;SORCS1;PAX5;PCDHA13;FTSJ2;C9ORF64;CDYL2;BCMO1
NMT3A;LRRC40;ARID5B;C6ORF62;PROX1;FTSJ2;DIEXF;GTPBP4;CDYL2;BCMO1;C1ORF115;NOS1AP
C1;GAB1;LRRC40;GPR75;ATP11A;PROX1;FTSJ2;DIEXF;NOS1AP;COMMD3-BMI1;ASPHD2;WDFY3;ANK
RIN3;CRISPLD1;CRISPLD2;ADAM12;ELMO1;PTGFR;GPM6A;ADCYAP1R1;NPR3;GATA6;TRHDE;FBLN5
;CMC1;ALG14;LRRC40;C6ORF62;YOD1;SENP2;DIEXF;MOB3C;SNPH;TERF2IP;WDFY3;ANKRD40;MAF
33;SORD;ALG14;ZBTB34;FTSJ2;GNL1;EREG;MOB3C;RBL1;NOS1AP;FAM64A;MAPRE3;SLC25A12;RAF
2;C3ORF62;VASH2;TFEB;ARID5B;CDYL2;SUMF1;EREG;BCMO1;DHX40;PTP4A1;SFRP1;DDX19B;HEC
ME;TPM3;ANGEL2;TMOD2;KIAA1462;EBF1;ALG14;C6ORF62;SLC39A13;PROX1;SENP2;HPCAL4;DYNL
LG14;LRRC40;HAUS3;SLC39A13;YOD1;CBFA2T2;RUNX3;RAP2C;TGFBR3;FAM101B;RRM2B;NOS1AP;T

IEXF;RAP2C;C1ORF115;ARMCX6;ZNF318;TFAM;PGAM5;ANKRD40;CGGBP1;KANK2;TBC1D19;USP15;
CDHAC1;MYEOV;DNMT3A;EBF1;GAPVD1;HPCAL4;C9ORF64;NAALADL2;SUMF1;BCMO1;DDX19B;C1C
4A1;DDX19B;CREB1;RBL1;TMEM33;SUB1;WDFY4;CRK;CGGBP1;TMEM106A;PAFAH1B2;FYTTD1;ROC
DNMT3A;LRRC40;ARID5B;C6ORF62;GAB3;ATP11A;PROX1;FTSJ2;HPCAL4;DIEXF;ADRA2A;EREG;C1O
USP2;CMC1;EBF1;LRRC40;CDYL2;PAX2;ADRA2A;EREG;ENAH;SFRP1;RAP2A;ADCY9;SUB1;TFAM;DT
LD1;CRISPLD2;HECW2;MAPRE3;SCG3;ZNF236;SMTNL2;ST6GALNAC3;KANK4;SHC4;DLX1;TBCEL;PFK
HACTR4;DFFA;GPR37;TTC33;TPM3;TMOD2;DNMT3A;VASH2;SLC39A13;HPCAL4;DIEXF;NAALADL2;SU
LO;CCDC50;EOMES;HS3ST3B1;RAB2B;TMEM194B;C21ORF59;EBF1;CDYL2;PTP4A1;KAT2B;RBL1;FAM
2;UTP15;TMEM194B;USP2;C3ORF62;DNMT3A;GPR75;PAX5;PROX1;SENP2;GTPBP4;CDYL2;BCMO1;R
FOXP2;RBX1;GPRIN3;ANKRD40;SCG3;SHC4;SHC2;NECAB3;ARL3;SHOX2;DTX1;FBLN5;NOP9;ARHGA
BP4;FAM133B;RNF126;CDC37;CDIP1;TFAM;WDFY3;WDFY2;ABHD4;YTHDC1;UBA6;ARL3;ABHD2;SHOX
F59;ANGEL2;C3ORF62;CMC1;GAPVD1;FTSJ2;PCDHA12;SUMF1;RBL1;TMEM33;CNKSR3;RBMS1;FAM
7;TPM3;TMOD3;OMD;KIAA1462;VASH2;GPR75;GAPVD1;DIEXF;BCMO1;DHX40;GPRIN3;PTP4A1;C1OR
RA2A;BCMO1;SFRP1;C1ORF115;CDC37;NOS1AP;FAM84A;COMMD3-BMI1;TFAM;PGAM5;WDFY3;ANK
B3;GAPVD1;CBFA2T2;SENP2;DIEXF;C9ORF64;GTPBP4;FAM185A;CMPK1;MAPRE3;TMEM106A;GLYR1
ISUN3;C3ORF62;CMC1;ALG14;LRRC40;HCFC2;PCDHA12;DIEXF;NAALADL2;PCDHA10;BCMO1;CMPK1
C1;GAB1;ALG14;ATP11A;SENP2;ZFX;RUNX3;RAP2B;NOS1AP;TCEANC2;ASPHD2;FAM64A;PRTFDC1;
B1;ATP11A;PROX1;FTSJ2;CDYL2;EREG;BCMO1;SFRP1;RAP2A;CRISPLD1;NOS1AP;WDFY3;ANKRD40

X1;DDX19B;STIM2;FAM185A;ZNF318;CMPK1;LCOR;TBC1D25;WDFY3;FAM64A;SLC25A12;SOS2;PPIC;
13C;MMP2;SWAP70;OMD;DIO2;SORCS1;PAX5;PROX1;DIEXF;PIAS1;DHX40;PTP4A1;DDX19B;TCEAN
7;ST6GAL2;TPM3;RNASE6;KIAA1462;VASH2;CNPY1;GAB3;SORCS1;PROX1;IL17RD;SHISA9;SYTL4;E
135;ST6GAL2;SGIP1;CNPY1;PAX5;SYTL4;ADRA2B;EREG;DHX40;PTP4A1;KIAA1614;ADAM12;FAM84A
;SGIP1;MMP2;VASH2;EBF1;ACSL6;CNPY1;PCDHA13;PCDHA12;ADRA2B;PCDHA10;EREG;SFRP1;C1O
2;SMC1A;RAP2C;MOB3C;KIAA1614;CNKSR3;TBC1D25;SOS2;USP15;SF3B3;DCUN1D5;FUT11;CREBL
ST6GAL2;WDR37;EBF1;GPR75;PROX1;GAPVD1;ADRA2B;BCMO1;DHX40;FAM64A;SCG3;ST6GALNAC
HS3ST3B1;UTP15;KCMF1;RAB2B;PRKAB2;TMEM194B;ALG6;USP2;C3ORF62;CMC1;LRRC40;PROX1;F
9A13;HCFC2;GTPBP4;DHX40;ADAM19;GPRIN3;FAM101B;ADCY9;FCHSD2;CDC37;CMPK1;TMEM106A;
R;CCDC50;TGM2;PHACTR4;USP47;DFFA;KIAA1462;EBF1;TFEB;C6ORF62;IL17RD;PTP4A1;TMEM33;A
;MYEOV;NSUN3;USP2;ARID5B;FTSJ2;HCFC2;SENP2;C9ORF64;RAP2C;CREB1;FAM185A;CNKSR3;PG
JSP2;C3ORF62;DNMT3A;GAB1;VASH2;CNPY1;ARID5B;DIEXF;ADRA2A;FAM133B;DHX40;CRISPLD1;A
ASH2;TAP2;GPR75;GAB3;POU3F1;IL17RD;PAX2;DKK3;MAF;RRM2B;TET3;SLC25A51;NAPA;FAM114A1;
194B;GPR37;ESCO1;ITPK1;ALG14;HAUS3;IL17RD;SHISA9;PCDHA12;PCDHA11;FOXP2;CDYL2;PCDHA
NP2;C9ORF64;CDYL2;BCMO1;RUNX1;C1ORF115;FAM185A;NOS1AP;FAM64A;SCG3;ST6GALNAC3;SH
C37;ADAM12;TFAM;SCG3;CGGBP1;KANK4;PPIC;SHC4;TBCEL;NECAB3;YTHDC1;UBA6;PDE1B;HSPA

M;TMEM194B;ALG6;CMC1;ALG14;SLC39A13;DIEXF;GTPBP4;FCHSD2;TERF2IP;WDFY3;SLC25A12;E
;TTC33;TMOD3;C3ORF62;MSL2;DIEXF;PIAS1;RUNX1;DHX40;ENAH;FAM101B;RAP2A;RAP2B;RBL1;AR
LG6;ESCO1;TTC33;ANGEL2;TMOD2;KIAA1462;CNPY1;SENP2;PTP4A1;ACAP2;CDC37;FAM185A;ZNF
DHAC2;PCDHAC1;EOMES;CXADR;GPR37;YOD1;PAX5;PCDHA13;PCDHA12;ADRA2B;PCDHA11;PAX2;
IAS1;EREG;FAM133B;NOS1AP;CMPK1;COMMD3-BMI1;NAA38;SCG3;KANK2;RALA;BHLHB9;ZDHHC2
13;RNASE6;VASH2;SORD;SLC39A13;GAB3;IL17RD;HPCAL4;ENAH;FAM101B;CNKSR3;ARMCX6;FAM64
2;SLC30A4;CNPY1;GAB3;FNDC3A;GAPVD1;HCFC2;SENP2;ENAH;ADAM19;SFRP1;MAF;TET3;TERF2
5A;ZNF318;CMPK1;TFAM;FAM64A;WDFY2;CGGBP1;SLC29A3;PRTFDC1;UBA6;PPM1H;TMTC3;CREBL
B;ALG6;MME;SFMBT2;NSUN3;USP2;C3ORF62;TFEB;GPR75;HPCAL4;RUNX3;SUMF1;EREG;DDX19B;
1;SWAP70;ACSL6;GPR75;GAPVD1;HCFC2;HPCAL4;ADRA2B;EREG;DKK3;SNPH;FAM64A;KANK4;ARH
ZFY;DIEXF;ENAH;FAM101B;ADCY9;FCHSD2;ARMCX6;TFAM;PGAM5;FAM64A;SLC25A12;TMEM106A;G
;C3ORF62;DNMT3A;VASH2;EBF1;TFEB;ADRA2B;SUMF1;DHX40;MAF;KIAA1614;CRISPLD1;ARMCX6;E
;ST6GAL2;SFMBT2;RNASE6;VASH2;TFEB;GAB3;SORCS1;SHISA9;FOXP2;DKK3;NRG3;CRISPLD1;ELM
;CBFA2T2;SENP2;DIEXF;GTPBP4;CREB1;FAM185A;COMMD3-BMI1;MAPRE3;SLC25A12;SLC29A3;HDA
M133B;ENAH;FCHSD2;CNKSR3;TET3;ARMCX6;TFAM;ANKRD40;FAM64A;SLC25A12;ZNF597;PPIC;GL
PR75;SORCS1;GAPVD1;POU3F1;IL17RD;EREG;GPRIN3;DDX19B;CRISPLD2;ADAM12;SCG3;COL6A5;
37;CMPK1;TCEANC2;SLC25A51;TFAM;NECAB3;IGSF3;DCUN1D5;YTHDC1;INSIG2;ABHD2;DCUN1D1;
0;TMOD2;ACTN4;PIAS1;DHX40;ENAH;DDX19B;IL1RAPL1;NAA35;FAM64A;SCG3;CD47;MAPRE2;PSM
A2A;BCMO1;C1ORF115;IL1RAPL1;ARMCX6;TFAM;ANKRD40;SCG3;TMEM106A;BHLHB9;SEMA3A;PAL
M185A;TBC1D25;TFAM;WDFY3;MAPRE3;SLC25A12;SOS2;PRTFDC1;GLYR1;USP15;BHLHB9;UBA6;T
TP15;GRID1;TMOD2;USP2;SORD;ALG14;SORCS1;GAPVD1;SHISA9;HPCAL4;DIEXF;EREG;DKK3;FAM
C3ORF62;CNPY1;SMC1A;IL17RD;HPCAL4;GTPBP4;RUNX1;FCHSD2;CDC37;SUB1;TET3;ZNF318;TCE
SWAP70;TMOD2;DYRK1A;ZBTB34;ATP11A;GAPVD1;GPCPD1;ADRA2B;EREG;BCMO1;DHX40;CRISPL
3;SHOX2;AGAP2;RHOBTB3;NCKIPSD;APH1B;ATXN7;TMEM248;PLXNA2;HOXC8;RAB11FIP5;LYN;EIF2
RF64;BCMO1;MAF;SNPH;CRISPLD2;ARMCX6;NOS1AP;ASPHD2;FAM64A;MAPRE3;NAT8L;PRTFDC1;H
F62;GAB3;GAPVD1;POU3F1;SYTL4;ADRA2B;NAALADL2;DDX19B;RBL1;CRISPLD2;SUB1;ELMO1;TER
EB;GAPVD1;HPCAL4;RUNX3;ADRA2A;SUMF1;EREG;BCMO1;DHX40;C1ORF115;SCG3;ZNF236;SET;H
14;LRRC40;HAUS3;ARID5B;DIEXF;GTPBP4;PGAM5;COL6A5;PRTFDC1;KANK2;FYTTD1;USP13;TBC1
SUMF1;EREG;DHX40;DDX19B;C1ORF115;CRISPLD2;FAM185A;ELMO1;FAM64A;SLC29A3;KCTD16;DL
2;HAUS3;FNDC3A;PCDHA13;PCDHA11;DHX40;RAP2A;RNF126;DDX19B;MYOD1;FAM64A;CD47;ATL3;
;ZFX;DIEXF;EREG;BCMO1;RUNX1;RAP2A;STIM2;HECW2;ADAM12;FAM64A;CGGBP1;ARL3;SHOX2;TU
J2;CDYL2;ADRA2A;BCMO1;ENAH;MAF;HECW2;COMMD3-BMI1;WDFY3;FAM64A;PRTFDC1;PLAG1;DL
;ALG6;MYEOV;OMD;PAX5;PCDHA12;DIEXF;PAX2;ADRA2A;PIAS1;DHX40;DDX19B;ADAM12;NAA35;SM
M12;SNX6;PLXNA3;SRPK2;ACBD5;FZD5;SRD5A1;FAM78A;LNPEP;HELZ;PUM2;SP1;SP4;OSTC;FUBP
BFA2T2;GNL1;DIEXF;RBX1;DHX40;TCEANC2;NAA35;TFAM;NAA38;FAM64A;LPHN2;CRK;TBCEL;BHLH
F64;SUMF1;RBX1;TGFBR3;DDX19B;FAM185A;ARMCX6;TERF2IP;ANKRD40;SLC25A12;PRTFDC1;SRS

);TSFM;HSDL1;ESCO1;SWAP70;ITPK1;GPR75;HCFC2;RBX1;TMEM33;TBL1XR1;CDC37;TET3;TERF2IP
2;RUNX3;SUMF1;MAF;SNPH;NOS1AP;ASPHD2;ANKRD40;FAM64A;PRTFDC1;KANK2;HDAC5;ABHD4;A
ADRA2B;RUNX3;DKK3;RUNX1;DHX40;GPRIN3;CRISPLD1;COL6A5;ST6GALNAC3;NAT8L;TMEM106A;AT
TSFM;CXADR;C21ORF59;ACSL6;RC3H1;GAPVD1;SMC1A;CBFA2T2;GTPBP4;DHX40;MOB3C;CRISPLD
M;NECAB3;YTHDC1;INSIG2;GSKIP;AARD;ZBTB4;ZMPSTE24;MTDH;CYB5R4;RNF214;ATXN7;MIER3;SN
PVD1;HPCAL4;C9ORF64;FAM133B;DHX40;C1ORF115;CRISPLD2;SUB1;SCG3;SHC4;DLX1;TBCEL;BHL
ADAM19;SFRP1;HECW2;FKBP6;KCTD16;SHC4;ADCYAP1R1;TBC1D19;PFKFB3;FAM114A1;RNF180;RH
PVD1;RUNX3;FAM101B;CRISPLD2;STIM2;CDC37;TFAM;ZNF236;SLC25A12;DTL;SHC4;PHLPP2;NAPB;S
VD1;FTSJ2;RUNX3;DHX40;GPRIN3;ADCY9;RBL1;ADAM12;NOS1AP;TBC1D25;TFAM;TMEM106A;SHC4
33;ZFY;ZFX;FAM101B;DDX19B;TET3;ARMCX6;TFAM;MAPRE3;KANK2;SLC24A2;BHLHB9;TTL;FBLN5;N
;FAM185A;TBC1D25;DENND6A;CGGBP1;GLYR1;SHC4;PFKFB3;NECAB3;FAM114A1;TNKS;RNF180;FU
DIEXF;GTPBP4;RUNX1;ADCY9;TET3;TFAM;MAPRE3;SRSF9;PPIC;HDAC5;SF3B3;ATL3;ZDHHC20;ARL
IAS1;ACAP2;ADCY9;FCHSD2;TMEM33;TET3;ARMCX6;TFAM;PGAM5;HDAC5;YTHDC1;TMTC3;GRAMD
33;FNDC3A;GAPVD1;FTSJ2;DIEXF;FOXP2;SUMF1;DHX40;FAM101B;ACAP2;RAP2B;SUB1;CDIP1;SMTN

;SLC29A3;PAFAH1B2;SHC4;NAPB;STAU1;ATL3;LUZP1;PDE1B;ARL3;PRUNE2;FAM129A;DTX1;ZDHHC2
1;FTSJ2;HCFC2;HPCAL4;FAM133B;DHX40;MOB3C;FAM185A;ARMCX6;CDIP1;DENND6A;NPTXR;SHC4
YTHDC1;INSIG2;PDE1B;GSKIP;ZBTB4;ZMPSTE24;RNF214;ATXN7;MCMBP;SNX8;SNX6;PLXNA3;CPSF

ENAH;PTP4A1;DDX19B;ARMCX6;ZNF236;TMEM106A;SLC24A2;TBC1D19;TBCEL;ROCK1;HPGD;NRXN
D1;FTSJ2;ZFY;CBFA2T2;DYNLL2;DIEXF;PIAS1;CDC34;FCHSD2;CNKSR3;TET3;TFAM;GLYR1;PHLPP2;
ZNF706;ATXN7;MIER3;VTI1A;MCMBP;LONRF3;IP6K1;SRPK2;CREBBP;ACBD5;MAGEE1;TIPARP;NEK7;
;HPCAL4;SUMF1;EREG;BCMO1;PTP4A1;C1ORF115;MAPRE3;TMEM106A;KANK4;FYTTD1;TBCEL;ATL
SJ2;POU3F1;SHISA9;FOXP2;EREG;BCMO1;CRISPLD2;TFAM;COL6A5;LPHN2;SHC4;USP13;FAM129A;
4;FAM101B;TET3;ARMCX6;TMEM106A;PHLPP2;PSMD11;SF3B3;SRSF1;UQCR10;GRAMD4;GIGYF2;C2
A13;DEK;SMARCA1;PCDHA12;PCDHA11;DHX40;TMEM56;RFWD3;TBC1D25

;ALG6;TTC33;BROX;KIAA1462;SLC39A13;SUMF1;ARMCX6;TERF2IP;NAA38;MAPRE3;SLC25A12;PHL
;GPR135;HS3ST3B1;NCOA2;ARHGEF10;PRKAB2;SLC30A4;TFEB;GAPVD1;IL17RD;HPCAL4;ADRA2B;
D5;SMURF2;SRD5A1;NEK7;GFPT1;SS18L2;MRPL27;PARVA;BFAR;INHBA;ZFP62;OSTC;RAD9A;PHF20;
W2;ELMO1;KANK4;FKBP6;PPIC;SHC4;ADCYAP1R1;BHLHB9;PDE1B;RNF180;NPR3;FUT11;RHOBTB3;
2;TERF2IP;FAM64A;SLC25A12;SET;DCTN2;FAM114A1;DCUN1D5;ARL3;TMTC3;UQCR10;ZMPSTE24;N
FA2T2;DIEXF;PIAS1;ACAP2;RNF126;FCHSD2;TET3;TBC1D25;TFAM;SLC25A12;CRK;TMEM106A;ATL3;
A2B;SUMF1;EREG;GPRIN3;CRISPLD1;CRISPLD2;ADAM12;ASPHD2;SMTNL2;TMEM106A;SLC29A3;KA
YOD1;GNL1;DIEXF;FCHSD2;LCOR;NAA35;TFAM;PGAM5;LPHN2;TMEM106A;TBCEL;DCTN2;TNKS;FUT
UN1D5;YTHDC1;UBA6;INSIG2;DCUN1D1;TMTC3;GSKIP;MTDH;BCL2L13;RNF214;SNX1;ATXN7;CHAD;
L1;DIEXF;FAM101B;TET3;LCOR;TFAM;WDFY4;TBCEL;SF3B3;DCTN2;MTMR9;DTX1;PDHB;NOP9;GNA
D2;SCG3;SPEM1;SMTNL2;NPTXR;SHC4;CRP;PTGFR;USP15;IGSF3;HPGD;PRUNE2;TYRP1;GLIS3;FA
D2;DNMT3A;ACSL6;CNPY1;GAPVD1;HPCAL4;DYNLL2;SYTL4;ADRA2B;EREG;FAM101B;SNPH;ARMC
1A;PCDHA12;ZFX;ADRA2B;ADRA2A;PCDHA10;DHX40;RAP2C;ADAM19;DDX19B;CLDN18;NAA38;SCG
LPP2;ATL3;YTHDC1;ATL2;PPM1K;C1ORF52;AP3M2;NXF1;ATXN7;MIER3;MGAT5;MCMBP;SLC25A24;P
LD2;TET3;ARMCX6;TFAM;NAA38;CRK;TMEM106A;GLYR1;KANK2;HDAC5;ATL3;DCTN2;ADH1B;GNA13
D1;FAM84A;ST6GALNAC3;LPHN2;TRIB2;CRP;PTGFR;TSHZ3;SRSF1;MTMR9;UQCR10;C1ORF52;ZNR
TFAM;TARDBP;MXD1;TMEM106A;DAND5;GLYR1;RAD9A
LCOR;TARDBP;SERINC3;TMEM106A
33;LCOR;TFAM;DENND6A;TMEM106A;DCP2;CHST3;SF3B3;NECAB3;SRSF1;FUT11;AGAP2;MTMR9;DT
7;SWAP70;VASH2;GAB3;PAX5;PROX1;GPCPD1;ADRA2A;DHX40;DDX19B;RAP2B;TBC1D25;CD47;SHC
A;SHC2;RNF180;PRUNE2;DTX1;PAPSS2;NOP9;NCKIPSD;FBXO40;ZDHHC18;RAP1A;EFS;TMEM248;CA
;C1ORF115;ST6GALNAC3;LPHN2;ZNF597;SLC24A2;PTGFR;MTMR3;ABHD4;ABHD2;PALM2;NPR3;PRU
HA10;RBX1;EREG;ELMO1;ASPHD2;SCG3;AARD;DTX4;TRHDE;FBXO40;PCDHA1;PCDHA5;PCDHA4;P
ALG14;CNPY1;GPR75;GAPVD1;FTSJ2;HCFC2;SHISA9;DKK3;ADAM19;CRISPLD1;FAM64A;SCG3;NPT
1;FOXP2;SUMF1;KAT2B;NRG3;IL1RAPL1;ASPHD2;MAPRE3;NPTXR;CHST3;KANK2;SHC4;GPM6A;TBC
T3;ARMCX6;NAA35;TFAM;NAA38;FAM64A;TMEM106A;PHLPP2;NECAB3;SRSF1;FUT11;GATA6;PDHB;C

)HA13;PCDHA12;PCDHA11;DIEXF;ADRA2A;PCDHA10;DHX40;DDX19B;SHC2;DLX6;HOXD12;C1ORF52;
:1D25;TMEM106A;KANK2;SET;NECAB3;SRSF1;FUT11;PDHB;NCKIPSD;GNA13;NXF1;TMEM203;MAT2A
I65;DLX1;SHC2;PFKFB3;DCTN2;PDE1B;FUT11;GRAMD4;TRHDE;PAPSS2;NKAIN1;GPC1;CARNS1;C10(
=AH1B2;ZNF597;GLYR1;PHLPP2;PSMD11;DCTN2;NUFIP2;CUL3;LRP8;C21ORF119;PPP2CA;BCLAF1;TM
TFAM;FAM64A;CRK;CGGBP1;TMEM106A;ZNF597;PPIC;GLYR1;KANK2;BHLHB9;ATL3;ATL2;PURA;RNF2
RG3;CNKSR3;ARMCX6;ZNF318;TERF2IP;WDFY2;SLC25A12;TMEM106A;PRTFDC1;PHLPP2;TBC1D19;E
34;ADCY9;CDC37;ELMO1;TCEANC2;SLC25A51;TFAM;FAM64A;WDFY4;SET;BHLHB9;NECAB3;YTHDC1
1;CDC37;TRIB2;SHC4;TBC1D19;NECAB3;INSIG2;PDE1B;DLX6;TMTC3;ZMPSTE24;ARHGAP20;ERI1;ZM
10;WDR37;TTC33;FNDC3B;RC3H1;YOD1;SMC1A;SYTL4;DIEXF;CDYL2;ENAH;FAM101B;ACAP2;FCHSD
6;FAM84A;ASPHD2;BHLHB9;ARL3;PDHB;UQCR10;ZNF25;ZBTB4;EFS;TMEM203;PABPN1;KIAA0355;FA
P4A1;C1ORF115;MAPRE3;ZNF236;KCTD16;TBC1D19;SET;DCTN2;FAM114A1;YTHDC1;ADH1B;PALM2;N
LG14;SLC39A13;CBFA2T2;SENP2;SUMF1;RBX1;FAM101B;ARMCX6;ZNF318;COMMD3-BMI1;FAM64A;S
NPY1;PAX5;GAPVD1;HPCAL4;ADRA2B;FOXP2;ADRA2A;EREG;BCMO1;ACAP2;RAP2A;ADCY9;MYOD1;
FCHSD2;STIM2;TET3;ARMCX6;TFAM;SLC25A12;DTL;TMEM106A;GLYR1;HDAC5;ATL3;YTHDC1;ATL2;S
DHA10;DHX40;DDX19B;MAF;ADAM12;FAM84A;DCX;AGTR2;CD47;TSHZ3;HPGD;PLAG1;SRSF1;C1ORF
)RF52;NXF1;ZNF629;TMEM203;MAT2A;C10ORF2;IP6K1;BCL2L2-PABPN1;RPP14;SMNDC1;IGBP1;CPS
;CRISPLD1;SCG3;COL6A5;NAT8L;NECAB3;PRUNE2;GRAMD4;COL19A1;NSD1;CHAD;PTK2B;GSE1;EIF
37;ARMCX6;PDE5A;AIPL1;DLX1;PTGFR;TBCEL;MYRF;PRUNE2;FAM129A;DTX1;ZBTB4;GRAMD4;TTL;
M33;DTL;SLC24A2;SET;PSMD11;C1ORF52;GRAMD4;MED12L;C21ORF119;BCL2L13;LRIG2;EIF4E;SNX8
RMCX6;TFAM;TERF2IP;PGAM5;FAM64A;SLC25A12;DTL;BHLHB9;UBA6;ARL3;UQCR10;ZNF25;GRAMD4
CX6;COMMD3-BMI1;TERF2IP;TMEM106A;KANK2;HDAC5;XIAP;PDHB;UQCR10;BCL2L13;APH1B;MIER3
B;KIAA1614;HECW2;ARMCX6;FAM64A;SLC25A12;PRTFDC1;BHLHB9;HSPA4L;CREBL2;UQCR10;ADD3
TSJ2;CRISPLD2;SUB1;TFAM;NAA38;SLC24A2;FAM114A1;C1ORF52;HIF1A;RIC8B;RBBP5;MGAT5;ZNF42
29A3;PRTFDC1;DLX1;SET;NECAB3;RHOBTB3;ZDHHC21;PDHB;UQCR10;C17ORF96;CYTH3;AP3M2;RN
CLDN18;NAT8L;PRTFDC1;CRP;DLX1;SHC2;SET;IGSF3;PPM1E;TRHDE;C21ORF119;EFNB3;GBX2;ERI1
RF62;KCNRG;PCDHA12;DYNLL2;PUM2;SP1;VAPB;RFWD3;TET3;TARDBP;LSM14B;MXD1
5A12;BHLHB9;PDHB;UQCR10;ZNF25;TTL;RNF214;ZDHHC18;MAT2A;PABPN1;MIER3;IGF2BP1;C10ORF
PHN2;TMEM106A;TBCEL;FUT11;PDHB;UBE2J1;NCKIPSD;NXF1;TMEM203;PCBD2;GLTSCR1L;SMNDC1
ID3-BMI1;TERF2IP;ASPHD2;SLC25A12;DTL;RNF165;PDHB;UQCR10;ZBTB4;TRHDE;ZDHHC18;C10ORF
DDX19B;IL1RAPL1;ADAM12;TET3;MAPRE3;SCG3;SLC24A2;SPATA2;TRHDE;FBLN5;BCL2L13;PABPN1;
A;PRTFDC1;BHLHB9;NECAB3;SEMA3A;UQCR10;ZBTB4;TRHDE;AP3M2;DRP2;EFS;MAT2A;PABPN1;US
RAMD4;AP3M2;NXF1;MAT2A;PABPN1;ATXN7;MIER3;C10ORF2;SLC25A24;CREBBP;TIMMDC1;SORT1;C
;PAFAH1B2;TUBD1;PDHB;UQCR10;ZMPSTE24;TRAK2;NKAIN1;RNF214;SNX1;TMEM203;MAT2A;MIER3
AM64A;TMEM106A;KANK2;HDAC5;BHLHB9;NECAB3;ARL3;UQCR10;GRAMD4;ZDHHC18;APH1B;GBX2
RSF1;FUT11;NRXN3;MTMR9;PDHB;NCKIPSD;NXF1;PABPN1;TDG;PRDM16;PCBD2;GLTSCR1L;SMNDC
C29A3;SHC4;PDE1B;FAM129A;DTX1;HOXD12;TRHDE;COL19A1;JAKMIP2;DRP2;ERBB4;CHAD;PLXNA2
7;TSFM;TMEM194B;ACBD5;TIMMDC1;CBX5;SS18L2;GATC;ATP2B2;GTF2H1;SENP2;DIEXF;GTF2H5;SU
SORD;GPR75;FTSJ2;C9ORF64;SUMF1;TPCN1;RUNX1;FAM101B;ZNF70;MAF;KIAA1614;GPAM;FAM185A
C;RC3H1;C6ORF62;SLC39A13;YOD1;GAPVD1;ADCY9;VAPB;SP4;SERBP1;TET3;TARDBP
BP;RAD9A

MR3;PSMD11;YTHDC1;CUL3;SPATA2;HIF1A;NOP9;MTDH;NPAT;RNF214;RAP1A;ATXN7;RNF217;VTI1A;
P1;SRSF9;MTMR3;DCUN1D5;YTHDC1;FUT11;DCUN1D1;TMTC3;MTMR9;HIF1A;GIGYF2;FOXO1;NOP9;
3;FAM114A1;PDE1B;SHOX2;NPR3;GATA6;GLIS3;DTX1;APCDD1;TRHDE;MED12L;FBLN5;UBE2J1;CTIF;
1;SHC2;ADCYAP1R1;PFKFB3;TSHZ3;DLX6;SHOX2;AGAP2;GLIS3;DTX1;AARD;TRHDE;MED12L;FBLN5
P;TMEM86A;CPSF7;ST13;NEK7;SREK1IP1;LNPEP;HOOK3;MARCH7;ZZZ3;SESTD1;FUBP1;STRN;MXD
OX2;PRUNE2;FAM129A;NRXN3;PRICKLE1;PPM1E;TRHDE;COL19A1;CYTH3;SERTAD2;ERBB4;TRA2B;
80;SHOX2;AGAP2;GATA6;DTX1;APCDD1;TRHDE;MED12L;FBLN5;GJC1;ARHGAP20;EFNB3;PRDM16;S
4;DLX1;FAM114A1;DLX6;SHOX2;NPR3;GATA6;GLIS3;RHOBTB3;MED12L;FBLN5;GJC1;NKAIN1;ARHGA
SHC2;ADCYAP1R1;SHOX2;AGAP2;GATA6;DTX1;APCDD1;TRHDE;FBLN5;GJC1;NKAIN1;ARHGAP20;EF
A;ST13;ZBTB16;ST8SIA3;LNPEP;RCAN1;GLUD2;SP1;STRN;LRP12;FGFR1;NRP2;BMPR2;DUSP19;CTD
HC4;SET;BHLHB9;ABHD4;TNKS;CUL3;NPR3;HSPA4L;RHOBTB3;SPATA2;CREBL2;APCDD1;DTX4;C17C
RD;APCDD1;TRHDE;FBLN5;UBE2J1;ARHGAP20;EFNB3;RNF217;PLXNA2;RIMKLA;N4BP2;MARK1;CAD
T3;PRDM16;C9ORF171;PCDHA3;LONRF3;PCDHA2;ZNF148;PCDHA6;TMEM86A;CCZ1B;ST13;RASSF8;
;PHOX2B;ARHGAP31;EFNA3;LHX6;LHX4;HRK;DOCK5;ANKRD33B;ONECUT2;KCNC2;TTC22;PTPRM;S
LD2;ADAM12;ELMO1;WDFY3;SCG3;KANK4;PPIC;PTGFR;GPM6A;TBC1D19;SET;PFKFB3;PDE1B;NPR
PN;SRGAP2;ABCA1;SRPK2;CBX5;ZBTB14;MAGEE1;SMURF2;NEK7;MRPL27;BFAR;LSM5;BTBD9;ESR
K1;AGAP2;GATA6;RHOBTB3;SLC1A4;FOXO1;MED12L;ARHGAP20;APH1B;C9ORF171;RIMKLA;ZNF148;
MO1;SCG3;KCTD16;PPIC;PTGFR;NECAB3;FAM114A1;TSHZ3;PDE1B;CUL3;AARD;TRHDE;FOXO1;FBLN
CTD16;IGSF3;PDE1B;DLX6;CUL3;SHOX2;NPR3;MED12L;ARHGAP20;ATXN7;PRDM16;MCMBP;MLEC;C
CW2;ADAM12;ELMO1;NPTXR;TRIB2;WDFY4;PTGFR;SHC2;NPR3;AGAP2;GATA6;AARD;DTX4;TRHDE;F
A;MCMBP;RIMKLA;RAI2;EDEM3;ABCA2;CBX5;ZBTB14;SMURF2;XRCC5;NEK7;MRPL27;HELZ;BFAR;M
;IGSF3;DLX6;RNF180;CUL3;NPR3;SPATA2;DTX4;ZBTB4;UBE2J1;RNF214;ARHGAP20;ATXN7;MIER3;LC
025;SIK2;ZNF275;PSMD11;NECAB3;FAM114A1;ABHD4;IGSF3;DCUN1D5;ABHD2;NPR3;DCUN1D1;MTM
E;MED12L;FBLN5;CTIF;RNF214;ZMAT3;RNF217;PRDM16;PLXNA2;PCDHA4;HOXC8;PCDHA6;TFAP2B;C
Y;HCFC2;ZFX;C9ORF64;CDYL2;EREG;BCMO1;PTP4A1;HECW2;FAM185A;RBMS1;WDFY3;CGGBP1;FY
JL3;SHOX2;MTMR9;ARHGAP20;EFNB3;ATXN7;MIER3;MCMBP;MLEC;PCDHA3;CLSPN;SCN3B;ZC3H14;
APCDD1;GIGYF2;BCL2L13;NKAIN1;CYB5R4;EIF4EBP2;SCN3B;MARK1;SRPK2;CBX5;FZD5;ZBTB16;M
M16;MLEC;C9ORF171;ZNF148;N4BP2;HOXC8;PCDHA7;LYN;TMEM86A;CCZ1B;EGR4;FZD5;SMURF2;
M19;ADCY9;KIAA1614;TBL1XR1;HECW2;FAM185A;ZNF318;SIK3;CGGBP1;KCTD16;BHLHB9;IGSF3;PDE
GSF3;PDE1B;DLX6;CUL3;SPATA2;CREBL2;ZNF25;ARHGAP20;APH1B;ATXN7;PRDM16;LONRF3;CLSP
;C9ORF171;CLSPN;PCDHA8;RAI2;TMEM86A;CCZ1B;EGR4;FZD5;SMURF2;LNPEP;LSM5;RCAN1;ARHG
MBP;RBM12;RAI2;PLXNA3;EDEM3;SRPK2;TMEM86A;CBX5;ZBTB14;MAGEE1;ZBTB16;NEK7;MRPL27;

;TMEM86A;ACBD5;XRCC5;FAM78A;ST13;MRPL27;BFAR;FBXO30;AZIN1;PUM2;SMAD7;PARP11;CNOT
;EGR4;FZD5;SMURF2;ST13;LNPEP;KLHL23;BTBD9;RCAN1;GLUD2;DIAPH2;ARHGAP31;SP1;CAPRIN1
;PDE1B;DLX6;RNF180;AGAP2;GATA6;DTX1;NKAIN1;EFNB3;CHAD;PRDM16;C9ORF171;LONRF3;RIMK
XRCC5;MRPL27;TRAPPC8;HELZ;HOOK3;MARCH7;SMAD7;PARP11;ZFP62;SP4;STRN;UBE2K;NFE2L1;
E;MED12L;ARHGAP20;EFNB3;ATXN7;PRDM16;MLEC;C9ORF171;PCDHA3;CLSPN;IP6K1;LYN;CBX5;C
P20;ATXN7;PRDM16;MCMBP;CLSPN;SCN3B;TMEM86A;CBX6;EGR4;SMURF2;LNPEP;BTBD9;FBXO30;
PCDHA4;MLEC;CLSPN;IP6K1;PCDHA6;LYN;TMEM86A;CBX6;CCZ1B;SMURF2;FAM78A;ST13;ZBTB16;S
;RIC8B;AP5M1;ZMAT3;EDEM3;B3GALNT2;MCTS1;CPSF7;EDEM1;MRPL27;NFATC3;LNPEP;LSM5;FBX
M16;PCDHA3;LONRF3;PCDHA7;LYN;TMEM86A;SMURF2;GLUD2;DIAPH2;SP1;SESTD1;PPP1R1B;FGF
G3BP1;PRDM16;C9ORF171;CLSPN;PCDHA8;PCDHA7;PCDHA6;CBX5;SMURF2;LNPEP;PARVA;PARP1
BM12;ZNF148;SNX6;PCDHA6;ABCA2;ACBD5;CCZ1B;NEK7;MRPL27;ZBTB10;EPT1;NBEAL1;SREK1IP1;
RIMKLA;FZD3;XRCC5;ST13;GFPT1;ST8SIA3;MRPL27;MARCH7;SMARCA1;GLUD2;ARHGAP31;STRN;DN
5;PLXNA3;MCTS1;CPSF7;MRPL27;EPT1;SREK1IP1;TRAPPC8;HOOK3;SMARCA1;FBXO30;C7ORF60;S
15;KIAA1614;HECW2;FAM185A;ADAM12;SIK3;WDFY3;CGGBP1;KCTD16;PPIC;GPM6A;TBC1D19;RNF1
TMR9;DTX1;UBE2J1;ARHGAP20;RIC8B;G3BP1;MLEC;C9ORF171;CLSPN;PCDHA8;B3GALNT2;TMEM8
;NPTXR;KANK4;PPIC;SHC2;TSHZ3;PDE1B;GLIS3;AARD;TRHDE;FBLN5;ARHGAP20;EFNB3;PCDHA1;P
148;LYN;TMEM86A;FZD5;BCL11B;SMURF2;TIPARP;ZBTB16;LNPEP;BTBD9;FBXO30;RCAN1;GLUD2;A
SL6;POU3F1;SHISA9;FOXP2;EREG;GPRIN3;MAF;ADCY9;C1ORF115;KIAA1614;CRISPLD1;HECW2;WD
;MCMBP;LONRF3;MCTS1;CBX5;ZBTB14;MAGEE1;NEK7;MRPL27;NBEAL1;NSG1;MARCH7;OCLN;ZFP

N3B;SNX6;B3GALNT2;SYT5;XRCC5;NEK7;GFPT1;MRPL27;SMAD9;HOOK3;ARHGAP31;ZZZ3;SESTD1;
P2;ABCA1;CBX5;EGR4;ZBTB16;MRPL27;SREK1IP1;HELZ;ESR1;SYT7;PARP11;ALDH6A1;ZFP62;PPP1R
M78A;ST13;ST8SIA3;LNPEP;GLUD2;PPP1R1B;STRN;GPR27;NRP2;GABRB1;MOCS3;PHF20;SH3KBP1
7;FAM185A;TBC1D25;WDFY3;WDFY2;SIK2;CGGBP1;PPIC;BHLHB9;FAM114A1;SHOX2;AGAP2;DCUN1D
A;CCZ1B;FZD5;SMURF2;SS18L2;LNPEP;KLHL23;BTBD9;FBXO30;ARHGAP31;SP1;DNAL1;LRP12;BMPR
RNF214;ARHGAP20;ATXN7;PRDM16;MCMBP;PCDHA3;CLSPN;ZC3H14;TMEM86A;CBX6;LSM5;RCAN1;
16;MCMBP;PCDHA3;CLSPN;IP6K1;LYN;TMEM86A;CBX6;CBX5;SMURF2;LNPEP;RCAN1;GLUD2;DIAPH
ED12L;GJC1;NKAIN1;ARHGAP20;EFNB3;CHAD;C9ORF171;SCN3B;MYH10;MARK1;SYT5;CADM3;EGR4
D2;CUL3;HSPA4L;MTMR9;SPATA2;DTX4;GIGYF2;ZMPSTE24;NPAT;ATXN7;G3BP1;VTI1A;MCMBP;CLSPN
EL;PFKFB3;BHLHB9;IGSF3;UBA6;SHOX2;TMTC3;CREBL2;DTX4;ZBTB4;FBLN5;ZMPSTE24;NCKIPSD;N
M12;ASPHD2;SCG3;NPTXR;KANK4;DLX1;GPM6A;DTX1;AARD;APCDD1;TRHDE;MED12L;PRDM16;SCN
N3B;RIMKLA;PCDHA8;PCDHA7;PCDHA6;EIF2B2;XRCC5;SMAD9;SMARCA1;GDF6;FUBP1;LSM14B;UBE
1;LNPEP;TRAPPC8;HOOK3;MARCH7;BTBD9;FBXO30;PARP11;ZFP62;FUBP1;NABP1;SERINC3;UBE2K;
11FIP5;EDEM3;MCTS1;ZBTB14;FZD5;SMURF2;XRCC5;ZBTB16;NEK7;KLHL23;HELZ;MARCH7;BTBD9;F
RHDE;NKAIN1;ARHGAP20;PRDM16;C9ORF171;LONRF3;SYT5;ABCA2;TFAP2B;FAM78A;ZBTB16;SLC4A
O1;MED12L;FBLN5;ARHGAP20;CHAD;LONRF3;RAI2;EGR4;ZBTB16;SLC4A10;GDF6;PHOX2B;REEP1;C
3GALNT2;TMEM86A;RABGAP1L;CPSF7;GFPT1;MRPL27;SREK1IP1;KLHL23;ARHGAP31;ZFP62;MXD1;
DTX4;MED12L;PRDM16;PCDHA5;MCMBP;PCDHA4;PCDHA3;CLSPN;SCN3B;PCDHA9;SNX8;RAI2;PCD
7;MIER3;PRDM16;MLEC;PCDHA3;PCDHA2;CLSPN;PCDHA7;TMEM86A;CCZ1B;EGR4;SMURF2;ZBTB16
EGR4;FZD5;ST13;LNPEP;ALDH6A1;STRN;NRP2;FMN1;C1ORF213;JPH1;NHSL2;XPO4;NKRF;KIF1B;ZN
ATXN7;MIER3;G3BP1;MLEC;C9ORF171;CLSPN;B3GALNT2;CBX6;CBX5;CCZ1B;SMURF2;TIPARP;ST13
;HECW2;FAM185A;ADAM12;ZNF318;WDFY3;ANKRD40;FAM64A;SCG3;SHC4;ZNF275;PFKFB3;BHLHB9
X;CDYL2;PTP4A1;RBL1;HECW2;FAM185A;RBMS1;WDFY3;LPHN2;CHST3;PPIC;FYTTD1;SHOX2;RHOB
P;SRGAP2;MARK1;ABCA1;MCTS1;TMEM86A;CBX5;ZBTB14;MAGEE1;SRD5A1;NEK7;MRPL27;SREK1IP
1A;PLXNA2;LONRF3;IP6K1;SNX8;SRD5A1;MRPL27;SREK1IP1;TRAPPC8;HOOK3;AZIN1;ARHGAP31;UE
XNA3;XRCC5;EPT1;PARVA;NSG1;BTBD9;ABHD15;ALDH6A1;EFNA3;UBE2K;NFE2L1;PLEKHM3;LRP12;F
EDEM3;TMEM86A;CBX5;MRPL27;EPT1;SREK1IP1;GLUD2;SP4;OSTC;SERINC3;DAND5;DOCK5;COX7B;
A;MOB3C;FAM185A;IL1RAPL1;LCOR;RBMS1;WDFY3;CHST3;PTGFR;PPM1L;TSHZ3;PDE1B;ATL2;RHO

;ZBTB4;ARHGAP20;ATXN7;PRDM16;MCMBP;C9ORF171;PCDHA3;LONRF3;CLSPN;ZNF148;TMEM86A;I
STE24;BCL2L13;FBXO40;RAP1A;RIC8B;MCMBP;IP6K1;N4BP2;SNX6;B3GALNT2;SRPK2;CPSF7;SMURF
X8;SNX6;SRD5A1;RASSF8;RCAN1;UBE2K;LRP12;RAD9A;COX7B;DYRK3;MOCS3;CELF1;ADK;JPH1;CD
27;EPT1;NBEAL1;LNPEP;TRAPPC8;HOOK3;RCAN1;STRN;TECPR2;DNAL1;PLEKHM3;LRP12;DYRK3;T
20;PRDM16;C9ORF171;MCTS1;TMEM86A;CBX5;EGR4;FZD5;SMURF2;FAM78A;ZBTB16;LNPEP;KLHL2
;ARHGAP20;MIER3;MCMBP;C9ORF171;CLSPN;ZC3H14;TMEM86A;SMURF2;PARVA;BTBD9;FBXO30;RC
M1H;RHOBTB3;NRXN3;CREBL2;ZDHHC21;ZBTB44;FAM117B;COL19A1;FBXO40;RIC8B;ZNF629;UBN2;
A4;MLEC;C9ORF171;CLSPN;ZNF148;LYN;TMEM86A;CBX6;CCZ1B;SMURF2;SS18L2;LNPEP;TRAPPC8;I
RCH7;ESR1;PARP11;ALDH6A1;ID4;LSM14B;DAND5;MOCS3;PRR3;DUSP19;PPWD1;BMI1;MED17;ING4
A2;FZD3;SS18L2;SMAD7;EFNA3;NCEH1;NFE2L1;DNAL1;RAD9A;ITM2C;FGFR1;C9ORF91;MOCS3;BMF
8A;ST13;GFPT1;LNPEP;BTBD9;LHX4;ITM2C;BMPR1A;C9ORF91;NRP2;DYRK3;BMPR2;TTC22;DIRAS2;
7;FZD5;RASSF8;SREK1IP1;LNPEP;HELZ;ZFP62;SP4;FUBP1;STRN;MXD1;NFE2L1;RAD9A;DYRK3;PPW
P2;EREG;GPRIN3;MOB3C;SFRP1;C1ORF115;KIAA1614;CRISPLD2;ADAM12;ELMO1;NPTXR;KANK4;PPI
4;HSPH1;APH1B;RIC8B;PRDM16;VTI1A;PCDHA4;MLEC;C9ORF171;PCDHA3;SCN3B;PCDHA7;PCDHA6
CDHA12;SYTL4;PCDHA11;PCDHA10;BCMO1;GPRIN3;KIAA1614;CRISPLD1;CRISPLD2;ADAM12;AIPL1;
3;CHAD;VTI1A;EDEM3;SYT5;ABCA2;RABGAP1L;CBX5;MRPL27;TRAPPC8;PHOX2B;SESTD1;ID2;OSTC
B;SNX6;SRPK2;FAM78A;HELZ;BTBD9;ESR1;AZIN1;ABHD15;BFSP2;OSTC;MXD1;DNAL1;PLEKHM3;FG
5A1;ST13;NEK7;GFPT1;ST8SIA3;PARVA;BFAR;L1CAM;BTBD9;ID2;LSM14B;BMPR1A;MOCS3;ANKRD33
6;CPSF7;FAM78A;ST13;NFATC3;EPT1;RASSF8;SREK1IP1;LNPEP;HOOK3;SMAD7;ZZZ3;MXD1;LRP12;F
ATC3;RASSF8;SREK1IP1;TRAPPC8;PARVA;HELZ;SMARCA1;RCAN1;ID4;STRN;CALM1;TECPR2;DNAL
6;SEMA3A;SHOX2;MTMR9;SLC1A4;ZDHHC21;ZBTB44;PPM1E;FAM117B;FBXO40;DFNB59;ZNF629;ZNF
4A1;ABHD4;UBA6;FBLN5;ARHGAP20;APH1B;RIC8B;MIER3;ZMAT3;HIVEP2;RIMKLA;N4BP2;RAB11FIP5
13;FTSJ2;HPCAL4;C9ORF64;BCMO1;ADCY9;CRISPLD2;FAM185A;ZNF318;ASPHD2;ANKRD40;FAM64A
D15;RCAN1;PARP11;HNRNPK;ZFP62;CAPRIN1;PPP1R1B;JMY;NABP1;MXD1;UBE2K;RAD9A;DYRK3;PD

:C5;ZBTB16;NEK7;MRPL27;HELZ;BTBD9;RCAN1;PARP11;ALDH6A1;HNRNPK;CAPRIN1;ID4;LSM14B;M
/IED12L;RNF217;PRDM16;MCMBP;MARK1;EDEM3;MCTS1;CBX5;SRD5A1;ZBTB16;SREK1IP1;ESR1;PA
EK7;MRPL27;PARVA;MARCH7;BTBD9;SMAD7;PARP11;ALDH6A1;HNRNPK;ZFP62;STRN;LSM14B;PLEK
RF64;EREG;BCMO1;MAF;CRISPLD2;FAM185A;WDFY3;ANKRD40;CGGBP1;PTGFR;BHLHB9;DLX6;CUL3
A6;ABHD2;CUL3;HSPA4L;MTMR9;SPATA2;GIGYF2;ZMPSTE24;NPAT;CYB5R4;PRDM16;VTI1A;MCMBP;
3;HPCAL4;GTPBP4;EREG;KIAA1614;FAM185A;COMMD3-BMI1;WDFY3;WDFY4;DLX1;PTGFR;TBC1D19
MEM86A;CCZ1B;FZD5;SMURF2;ST13;LNPEP;KLHL23;BTBD9;RCAN1;GLUD2;DIAPH2;ARHGAP31;SP1
PDE1B;DLX6;PPM1H;DTX1;NDNF;HOXD12;COL19A1;ARHGAP20;EFS;GBX2;IGF2BP1;IP6K1;EGR4;BCL
C8;MARCH7;L1CAM;GDF6;ALDH6A1;OCLN;EFNA3;ID4;DYRK3;PTPRM;FMN1;RCSD1;PFAS;MED17;CK
FBLN5;FBXO40;ARHGAP20;EFNB3;ERI1;CHAD;MARK1;EDEM3;FZD3;ST8SIA3;KLHL23;GRIN2B;C12OR
ALM1;SERINC3;TECPR2;BMPR1A;MOCS3;ANKRD33B;CELF1;SLC7A14;SIX1;ETFA;C1ORF213;PITPNC
N4BP2;ZC3H14;PLXNA3;CBX5;MRPL27;SREK1IP1;MARCH7;NCEH1;FUBP1;STRN;UBE2K;LRP12;DOCI
H1B;RIC8B;G3BP1;CREBBP;EDEM1;SMAD7;C7ORF60;OCLN;FUBP1;LSM14B;TECPR2;PLEKHM3;RAD9
RNF214;ARHGAP20;PRDM16;MCMBP;PCDHA3;LONRF3;ZC3H14;PCDHA6;TMEM86A;CCZ1B;SMURF2;L
MD11;DLX6;GLIS3;ARHGAP20;PCDHA5;MLEC;LONRF3;SNX8;PCDHA8;PCDHA7;PCDHA6;MARK1;CBX5
;ZBTB14;XRCC5;FAM78A;NEK7;NFATC3;NBEAL1;SMAD9;NSG1;L1CAM;BTBD9;FBXO30;SESTD1;RCAI

M64A;AIPL1;NPTXR;TRIB2;PPIC;PTGFR;TSHZ3;AGAP2;GATA6;DTX4;TRHDE;FOXO1;MED12L;GJC1;AF
LHB9;SPATA2;TRHDE;FBLN5;NPAT;APH1B;ZNF706;PCDHA1;VTI1A;PCDHA5;PCDHA4;PCDHA3;PCDHA
MRPL27;LNPEP;LSM5;BTBD9;RCAN1;ARHGAP31;SP1;SESTD1;LHX4;FGFR1;NRP2;BMPR2;CHURC1-I

A5;MCMBP;RAB11FIP5;ABCA1;MCTS1;CBX5;ZBTB14;FZD5;ZBTB16;NEK7;MRPL27;SREK1IP1;SMAD9;
XRCC5;MRPL27;BTBD9;ESR1;PARP11;ARHGAP31;HNRNPK;SP4;CAPRIN1;ID4;STRN;LRP12;RAD9A;M
1B;RIC8B;RIMKLA;SNX6;PLXNA3;EDEM3;SRPK2;XRCC5;ST13;GFPT1;L1CAM;BTBD9;PARP11;SESTD1
CAL4;BCMO1;MOB3C;MAF;ADCY9;CDC37;SUB1;HECW2;FAM185A;ASPHD2;WDFY3;CGGBP1;SHC4;TE
NEURL1B;ARHGAP20;EFS;GBX2;IGF2BP1;GPC5;MARK1;EGR4;FAM78A;ZBTB16;B3GAT2;SUSD1;SYT
B;SS18L2;RASSF8;TRAPPC8;PARVA;ID2;MXD1;TECPR2;DNAL1;TNFAIP8;SH3KBP1;ADK;PPP1R9A;AR
UBE2K;DNAL1;PLEKHM3;LRP12;BMPR1A;CREBZF;RBM28;NRP2;COX7B;CELF1;ADK;PPWD1;PFAS;C
P1;LSM14B;BMPR1A;HRK;GPR27;BTG1;CELF1;DIRAS2;PDE3B;PPWD1;PTPRM;HK2;ADAMTS5;ADAM
SREK1IP1;HOOK3;RCAN1;PARP11;C7ORF60;CNOT6;HNRNPK;NCEH1;SP4;OSTC;RAD9A;ITM2C;DYRK
;FAM78A;SS18L2;EPT1;LNPEP;SMARCA1;C7ORF60;HNRNPK;ZZZ3;SESTD1;STRN;TECPR2;ITM2C;CC
LNT2;TMEM86A;ST13;EPT1;RASSF8;LNPEP;GDF6;GLUD2;PPP1R1B;STRN;TECPR2;ITM2C;GPR27;KC
1;CRISPLD2;ADAM12;ASPHD2;NPTXR;KANK4;DLX1;PTGFR;GPM6A;SHC2;ADCYAP1R1;TSHZ3;PDE1E
K5;GFPT1;HELZ;INHBA;LSM5;ZFP62;SESTD1;OSTC;HNRNPC;CALM1;SERINC3;RAD9A;CDS2;DOCK5;
1;EDEM3;B3GALNT2;MCTS1;CBX5;ZBTB14;EGR4;MAGEE1;MRPL27;SREK1IP1;ESR1;PARP11;ALDH6/
S18L2;SMAD9;PARVA;AZIN1;REEP1;C7ORF60;OSTC;LHX4;UBE2K;DAND5;RAD9A;ITM2C;CREBZF;CO
ARMCX6;AGTR2;NPTXR;KANK4;SHC4;PTGFR;GPM6A;PSMD11;IGSF3;SHOX2;RHOBTB3;SLC1A4;TRH
CG3;KANK4;KCTD16;SHC4;PTGFR;GPM6A;BHLHB9;PDE1B;GLIS3;RHOBTB3;AARD;TRHDE;RNF217;F
1;INHBA;HOOK3;BTBD9;ARHGAP31;SESTD1;UBE2K;NFE2L1;ITM2C;BMPR1A;NRP2;BMPR2;DIRAS2;B
CREBBP;FZD3;CPSF7;LNPEP;HELZ;C7ORF60;ZZZ3;STRN;TECPR2;RAD9A;ITM2C;DYRK3;SH3KBP1;TI
1;MARK1;CBX5;ZBTB14;SRD5A1;SREK1IP1;HELZ;ESR1;PARP11;ALDH6A1;RCAN2;PPP1R1B;ID4;RAD9
ZBTB14;MAGEE1;ZBTB16;MRPL27;SREK1IP1;SYT7;PARP11;ALDH6A1;PPP1R1B;ID4;LHX6;STRN;RAD9
CMO1;HECW2;FAM185A;COMMD3-BMI1;WDFY3;ANKRD40;FAM64A;CGGBP1;KCTD16;PPIC;SET;FAM1
AM185A;IL1RAPL1;LCOR;WDFY3;PPIC;FYTTD1;PTGFR;TNKS;RNF180;CUL3;RHOBTB3;SPATA2;SLC1/
10;RAB11FIP5;MARK1;TMEM86A;EGR4;RASSF8;HELZ;REEP1;TECPR2;PLEKHM3;GPR27;DOCK5;NRF
SERINC3;UBE2K;RAD9A;COX7B;PHF20;ADK;SMG7;PFAS;CDC73;ELAVL2;ALAD;MRPL42;UBL3;SART3
171;HIVEP2;EDEM3;CREBBP;FZD3;MRPL27;RASSF8;ESR1;RCAN1;ZZZ3;LHX6;STRN;DNAL1;RAD9A;T
BP2;CADM3;TMEM86A;CBX6;EGR4;FZD5;MRPL27;KLHL23;PARVA;GDF6;DIAPH2;ARHGAP31;RCAN2;
2L13;APH1B;PCDHA1;PCDHA2;IP6K1;ZC3H14;LYN;ST8SIA3;MRPL27;EPT1;SREK1IP1;PARVA;RCAN2;F
AP20;EIF4EBP2;SCN3B;RAB11FIP5;ABCA1;SYT5;TFAP2B;RABGAP1L;CPSF7;EIF2B2;CBX5;EGR4;FZD
6;C9ORF171;ABCA2;TIPARP;ST8SIA3;SLC4A10;SMAD9;GRIN2B;ABHD15;REEP1;ARHGAP31;GPR27;D
;NKAIN1;ARHGAP20;EFNB3;CHAD;PRDM16;C9ORF171;TAC3;RIMKLA;SYT5;CADM3;EGR4;FAM78A;ZI
A1;SS18L2;INHBA;REEP1;HNRNPK;ZFP62;FUBP1;LRP12;GPR27;RBM28;BMPR2;DIRAS2;CTDSPL2;MY

2;CBX5;TIPARP;XRCC5;MRPL27;HELZ;BFAR;MARCH7;LSM5;BTBD9;PARP11;HNRNPK;CAPRIN1;PPP1
ZBTB16;C12ORF74;ID2;MOCS3;ONECUT2;PDE3B;PTPRM;TNFAIP3;RCSD1;BMI1;CDC73;ING4;ADAMT
RASSF8;BTBD9;PARP11;HNRNPK;CAPRIN1;STRN;CALM1;MOCS3;PPWD1;BMI1;PFAS;CDC73;MED17
CA1;ZBTB14;SMURF2;XRCC5;MRPL27;TRAPPC8;MARCH7;BTBD9;PARP11;HNRNPK;CALM1;LSM14B

62;ID2;OSTC;HNRNPC;RAD9A;CDS2;DOCK5;BTG1;PHF20;SNAP23;PPWD1;CTDSPL2;MED14;XPO4;M
CEH1;PPP1R1B;CDK1;LHX4;MXD1;LRP12;NRP2;DYRK3;SIX1;MED17;EEF2K;SH3PXD2A;TRPS1;FAM7
Z;BTBD9;SESTD1;RCAN2;UBE2K;TECPR2;PLEKHM3;LRP12;PRR3;ADK;FMN1;CTDSPL2;CDC73;DTW
JBP1;SERINC3;FGFR1;HRK;BTG1;CELF3;PDE3B;ADK;TNFAIP3;SMG7;LITAF;ELAVL2;MED17;CKS1B;S
D5;SMURF2;SRD5A1;NBEAL1;TRAPPC8;HELZ;BTBD9;EFNA3;ZZZ3;STRN;MXD1;TECPR2;DNAL1;PLEK
TBD9;AZIN1;ALDH6A1;OSTC;UBE2K;DAND5;RAD9A;ITM2C;CREBZF;RBM28;ADK;KIAA1671;RSF1;CTD
3;FBXO30;RCAN1;EFNA3;STRN;TECPR2;DNAL1;PLEKHM3;RAD9A;DYRK3;SH3KBP1;ALAD;DTWD1;S
9;TNKS;CUL3;SHOX2;HSPA4L;RHOBTB3;SPATA2;ZNF25;C17ORF96;ARHGAP20;EFNB3;ATXN7;ZMAT3
X1;APCDD1;C17ORF96;FOXO1;FBLN5;NKAIN1;ARHGAP20;EFNB3;CHAD;TAC3;LONRF3;SYT5;TFAP2E
B11FIP5;EDEM3;MCTS1;CREBBP;TMEM86A;XRCC5;NBEAL1;SREK1IP1;LNPEP;HELZ;HOOK3;MARCH
;B3GALNT2;FAM78A;ST13;LNPEP;L1CAM;PARP11;PPP1R1B;STRN;ITM2C;SH3KBP1;BMI1;MED17;PH
1;NPTXR;KANK4;DLX1;GPM6A;SHC2;ADCYAP1R1;IGSF3;PDE1B;RNF180;SHOX2;HSPA4L;AGAP2;GA
X6;CPSF7;ST13;MRPL27;RASSF8;SREK1IP1;LNPEP;HOOK3;GLUD2;CNOT6;SP1;NFE2L1;DYRK3;DIRA
TC3;RASSF8;SREK1IP1;LNPEP;TRAPPC8;ZZZ3;MXD1;RAD9A;TNFAIP8;DIRAS2;FMN1;C1ORF213;JPH
;MOCS3;PRR3;PPWD1;SMG7;MED17;ALAD;MRPL42;ARL5B;UBL3;SART3;SH3BGRL2;UBXN7;CTBS;U
R1;BHLHB9;FAM114A1;AARD;APCDD1;FBLN5;NCKIPSD;SCN3B;RIMKLA;MYH10;MARK1;ABCA1;MAGE
;CD47;COL6A5;DTL;CGGBP1;CHST3;PPIC;FYTTD1;SHOX2;SPATA2;SLC1A4;ZDHHC21;ZBTB44;ZNF2
F2;PGRMC1;RIC8B;GBX2;ZMAT3;ERBB4;PCDHA5;SLIT2;MCTS1;IGBP1;TFAP2B;ACBD5;CCZ1B;FAM7
APGEF6;CGGBP1;KCTD16;ZNF275;USP15;SF3B3;DCUN1D5;DLST;DTX1;SLC1A4;NDNF;UQCR10;TRH
LC29A3;PRTFDC1;KANK2;NAPA;SHC2;SF3B3;NECAB3;FAM114A1;TSHZ3;UBA6;PLAG1;AGAP2;UQCR
TAU1;YTHDC1;FUT11;AGAP2;TMTC3;GSKIP;PPM1K;UQCR10;DTX4;GJC1;NXF1;CYB5R4;BCLAF1;HSP
1;HECW2;NOS1AP;FAM84A;TBC1D25;WDFY3;ANKRD40;FAM64A;PRTFDC1;SHC4;BHLHB9;UBA6;PLA
REB1;CRISPLD2;SUB1;TERF2IP;SMTNL2;DTL;CRK;PAFAH1B2;KANK4;SRSF9;GLYR1;FYTTD1;PSMD1
4;PRTFDC1;PPIC;KANK2;BHLHB9;NECAB3;LUZP1;SEMA3A;TMTC3;SPATA2;PRICKLE1;UQCR10;LRP6
L2;HSDL1;MME;TPM3;TMOD2;USP2;KIAA1462;VASH2;GAB3;SORCS1;ATP11A;IL17RD;SHISA9;EREG;
19B;RBL1;RRM2B;STIM2;FAM185A;TCEANC2;COMMD3-BMI1;NAA38;SLC25A12;ZNF597;KCTD16;PPIC
1;RUNX1;DHX40;KIAA1614;TBL1XR1;FAM185A;ADAM12;TFAM;WDFY3;CGGBP1;PPIC;SHC4;NECAB3
115;FAM185A;COMMD3-BMI1;ANKRD40;SHC2;MTMR3;BHLHB9;FAM114A1;PDE1B;ARL3;RHOBTB3;MT
2C;GPRIN3;MOB3C;CDC37;ADAM12;FAM84A;SCG3;SMTNL2;ERGIC2;SLC29A3;KANK4;CHST3;PPIC;N
C1A;C9ORF64;CDYL2;PCDHA10;RRM2B;COMMD3-BMI1;NAA38;SLC25A12;CGGBP1;TMEM106A;DCP2
NK2;DLX1;HDAC5;BHLHB9;NECAB3;DCUN1D5;PLAG1;FAM129A;TMTC3;MTMR9;PRICKLE1;PDHB;C1C
AF1;CRK;SOS2;TMEM106A;ZNF597;PHLPP2;MTMR3;INSIG2;PLAG1;ARL3;ABHD2;ATL2;CREBL2;UQC
26;CRISPLD2;SUB1;SIK3;FAM64A;MAPRE3;PDE5A;NPTXR;ST6GALNAC3;MAPRE2;SHC4;USP15;ARL
;NECAB3;ROCK1;LUZP1;UBA6;SEMA3A;NRXN3;MTMR9;DLST;TUBD1;PDHB;PURA;ZNF706;TMEM203
;FBXO40;CTIF;PCDHA1;RNF217;PCDHA5;PCDHA4;PCDHA3;PCDHA2;PCDHA9;PCDHA8;MYH10;PCDH
K3;DHX40;SUB1;NOS1AP;FAM84A;TCEANC2;NAA35;SCG3;CD47;SPEM1;ST6GALNAC3;MAPRE2;KAN
;WDFY3;SLC25A12;TMEM106A;SHC4;DLX1;HDAC5;BHLHB9;NECAB3;ARL3;SPATA2;CREBL2;ZDHHC2
A;SIK3;CMPK1;LCOR;ANKRD40;CD47;SLC25A12;ARHGEF5;TBC1D19;RALA;USP15;ABHD4;DCUN1D5;
FY3;ST6GALNAC3;TRIB2;TMEM106A;KANK4;PRTFDC1;HDAC5;USP15;PFKFB3;BHLHB9;UBA6;PDE1B;
ACTN4;PROX1;PCDHA12;GTPBP4;CDYL2;EREG;RAP2C;MOB3C;CDC37;FAM84A;SMTNL2;ST6GALNA
PIC;PTGFR;YTHDC1;UBA6;PDE1B;HSPA4L;FUT11;APCDD1;MED12L;NPAT;EFNB3;RNF217;PRDM16;M
LG14;HAUS3;FTSJ2;SYTL4;CDYL2;ADRA2A;EREG;GPRIN3;FAM84A;CDIP1;SMTNL2;MAPRE2;SLC29A
RD40;SCG3;ZNF236;ST6GALNAC3;SHC4;HDAC5;BHLHB9;NECAB3;PLAG1;ARL3;AGAP2;UQCR10;GR
C1D25;WDFY3;WDFY2;SLC25A12;TRIB2;SOS2;TMEM106A;PRTFDC1;KANK2;HDAC5;TBC1D19;PFKF
UMF1;RUNX1;RAP2C;ADCY9;FCHSD2;CDC37;SUB1;ARMCX6;CDIP1;TFAM;SMTNL2;ST6GALNAC3;T
5;PFKFB3;BHLHB9;NECAB3;UBA6;UQCR10;TRAK2;C21ORF119;GNA13;NPAT;BCL2L13;NXF1;RIC8B;Z
DC37;SIK3;SIK2;RAF1;CRK;ZNF597;GLYR1;PHLPP2;DLX1;PFKFB3;STAU1;ABHD2;PALM2;SHOX2;SPA
;NAPA;PHLPP2;NAPB;PFKFB3;HPGD;YTHDC1;UBA6;ABHD2;MTMR9;CREBL2;ZDHHC21;ZBTB44;PPP2

38;ANKRD40;SLC25A12;RAPGEF6;TMEM106A;SHC4;HDAC5;USP15;PFKFB3;GLIS3;TMTC3;CREBL2;Z
OX2;APCDD1;DTX4;FOXO1;ZMAT3;HIVEP2;SCN3B;RIMKLA;SNX6;MCTS1;SRPK2;FZD3;RABGAP1L;ZE
RIB2;PRTFDC1;KCTD16;TBCEL;PFKFB3;BHLHB9;ROCK1;FAM114A1;DCUN1D5;SRSF1;GATA6;DCUN1D
;SOS2;TMEM106A;USP15;BHLHB9;UBA6;TMTC3;SPATA2;CREBL2;UQCR10;C1ORF52;ZBTB4;FAM117B
S3;ADK;RCSD1;SMG7;MED17;XPO4;NKRF;SLC22A17;UBL7;CCDC112;NSUN3;SLC39A13;GAB3;PROX1

TRAPPC8;HOOK3;MARCH7;LSM5;FBXO30;ZFP62;ID2;STRN;LSM14B;TECPR2;NFE2L1;RAD9A;CC2D1
2A;RNF126;CDC34;SUB1;CMPK1;ANKRD40;CD47;SHC4;TBC1D19;PSMD11;SF3B3;STAU1;IGSF3;YTHD
ST6GALNAC3;SLC25A12;DTL;SOS2;KANK2;SHC4;SHC2;BHLHB9;IGSF3;PDE1B;RNF180;SEMA3A;ATL2
MAF;SNPH;CRISPLD2;HECW2;ARMCX6;NOS1AP;MAPRE3;PRTFDC1;FYTTD1;DLX1;TBCEL;ABHD4;LU
G14;RC3H1;HAUS3;PCDHA12;SYTL4;DIEXF;FOXP2;GTPBP4;DKK3;RAP2C;FAM101B;ADCY9;CDC37;SU
EG;CDC37;ADAM12;FAM84A;ST6GALNAC3;ERGIC2;SLC29A3;KANK4;CHST3;PPIC;NAPA;PTGFR;YTHD
2;FBLN5;NPAT;RIC8B;EFNB3;PCDHA1;GSE1;HIVEP2;RIMKLA;PCDHA7;MARK1;B3GALNT2;XRCC5;FAM
PDE1B;RNF180;AGAP2;RHOBTB3;TRHDE;FBLN5;GJC1;CTIF;SNX1;ATXN7;EIF4EBP2;PCDHA4;RBM12;
P1;SLC29A3;CHST3;PHLPP2;HPGD;UBA6;ZDHHC20;HSPA4L;MTMR9;SPATA2;ZBTB44;UQCR10;UBE2J1
1;WDFY3;CHST3;PPIC;USP13;SHC2;MTMR3;NECAB3;FAM114A1;DLX6;ARL3;NPR3;TMTC3;SLC1A4;ZE
HA11;CDYL2;EREG;FAM101B;ADCY9;RRM2B;CLDN18;RBMS1;DENND6A;KCTD16;AMER1;BHLHB9;FAM
PK1;FAM84A;TBC1D25;COMMD3-BMI1;NAA38;ZNF236;ST6GALNAC3;MAPRE2;ZNF597;GLYR1;SHC4;F
PLD2;CDC37;MYOD1;FAM84A;TBC1D25;TERF2IP;ASPHD2;FAM64A;CD47;RAPGEF6;CRK;MAPRE2;KA
CTN2;NUFIP2;DCUN1D5;TNKS;ZDHHC20;HSPA4L;GSKIP;ZDHHC21;PDHB;UQCR10;MTDH;TRAK2;BC

ATA6;TMTC3;CREBL2;UQCR10;C1ORF52;FAM117B;FOXO1;TRAK2;NCKIPSD;DRP2;NPAT;BCL2L13;FBX
PRUNE2;DLST;SPATA2;UQCR10;C1ORF52;DTX4;ZBTB4;LRP6;C21ORF119;NKAIN1;PURA;RIC8B;TMEM
IAC3;PPIC;SHC4;DLX1;HDAC5;USP15;BHLHB9;UBA6;TMTC3;UQCR10;TRAK2;C21ORF119;NEURL1B;N
EB;CNPY1;GAB3;PAX5;IL17RD;HPCAL4;ADRA2B;DIEXF;PAX2;ADRA2A;BCMO1;CRISPLD2;MYOD1;CL
I;NRG3;CRISPLD2;ASPHD2;ANKRD40;SCG3;ST6GALNAC3;NAT8L;TMEM106A;DLX1;HDAC5;ADCYAP1
';CMPK1;ELMO1;WDFY3;ANKRD40;FAM64A;SHC4;GPM6A;BHLHB9;PDE1B;DLX6;PALM2;GLIS3;SPATA
RD40;SCG3;ST6GALNAC3;RAPGEF6;KANK4;PRTFDC1;SHC4;DLX1;HDAC5;BHLHB9;TMTC3;UQCR10;
5;CHAD;PRDM16;LONRF3;RIMKLA;SYT5;EGR4;SMAD9;SMARCA1;PHOX2B;GLUD2;OCLN;ITM2C;HRK;
PRE3;SLC25A12;TRIB2;CRK;PPIC;KANK2;HDAC5;INSIG2;PLAG1;ARL3;RNF180;TMTC3;TUBD1;CREBL
PGEF6;SOS2;MAPRE2;CHST3;SRSF9;DLX1;SF3B3;FAM114A1;DCUN1D5;NPR3;HSPA4L;CREBL2;UBE2
W2;ARMCX6;ELMO1;SCG3;ZNF236;SMTNL2;PRTFDC1;SHC4;FYTTD1;TBCEL;BHLHB9;DLX6;SEMA3A;
L2;RUNX1;MAF;CRISPLD2;NOS1AP;COL6A5;ST6GALNAC3;PRTFDC1;PLAG1;AGAP2;ZDHHC21;PRIC
TERF2IP;FAM64A;MAPRE3;NPTXR;GLYR1;KANK2;PHLPP2;RALA;ROCK1;FAM114A1;YTHDC1;ARL3;HS

;UBA6;PLAG1;TUBD1;CREBL2;PDHB;ZMPSTE24;TRAK2;C21ORF119;BCL2L13;RIC8B;TMEM65;ZNF629
ORF115;TMEM33;CRISPLD2;SCG3;ZNF236;ST6GALNAC3;TMEM106A;SLC29A3;FYTTD1;TBCEL;MTMR
K1;YTHDC1;SRSF1;HSPA4L;ZBTB44;PDHB;NOP9;MTDH;NXF1;ZNRF2;SNX1;BCLAF1;RIC8B;MAT2A;TF
ORF115;SNPH;ARMCX6;NOS1AP;ANKRD40;SCG3;RAPGEF6;TMEM106A;BHLHB9;PLAG1;PDE1B;C17O
L;PRTFDC1;KANK2;SHC2;TBC1D19;BHLHB9;SEMA3A;TMTC3;SPATA2;UQCR10;TTL;TRHDE;C21ORF1
KFB3;PPM1L;HPGD;ADH1B;RNF180;SHOX2;AGAP2;GLIS3;MAT2B;LRP8;COL19A1;PAPSS2;GJC1;FBX
IMF1;BCMO1;RUNX1;DHX40;DDX19B;TMEM33;HECW2;ARMCX6;ZNF236;KCTD16;FYTTD1;ZNF275;DL
I185A;COMMD3-BMI1;RBMS1;SCG3;ZNF236;CGGBP1;KCTD16;DLX1;SET;SHOX2;MTMR9;FAM117B;DF
RUNX1;GPRIN3;C1ORF115;HECW2;NOS1AP;CMPK1;TFAM;ANKRD40;FAM64A;SCG3;ST6GALNAC3;SH
AP20;G3BP1;SCN3B;RIMKLA;PLXNA3;MARK1;EDEM3;ABCA2;CREBBP;ZBTB14;EGR4;BCL11B;XRCC5;
K2;HSPA4L;TMTC3;SPATA2;DTX4;FOXO1;UBE2J1;NCKIPSD;NPAT;BCL2L13;RIC8B;MIER3;ZMAT3;CLSF
I64A;ZNF236;SLC25A12;LPHN2;TMEM106A;PPIC;DLX1;TBC1D19;USP15;BHLHB9;ATL2;TMTC3;CREBL
F115;SUB1;ARMCX6;SCG3;ZNF236;ST6GALNAC3;SLC29A3;KCTD16;DLX1;NAPB;TBCEL;SET;ADH1B;F
RD40;ST6GALNAC3;RAPGEF6;PRTFDC1;SHC4;BHLHB9;PLAG1;TMTC3;UQCR10;ZNRF3;RIC8B;TMEM
I;USP13;TBC1D19;PFKFB3;DCUN1D5;PDE1B;CUL3;HSPA4L;RHOBTB3;TMTC3;ZBTB44;UQCR10;TTL;L
1;COMMD3-BMI1;ASPHD2;WDFY3;ANKRD40;SOS2;SHC4;BHLHB9;NRXN3;PRICKLE1;UQCR10;FBLN5;
HDAC5;NECAB3;PLAG1;ARL3;PPM1H;TMTC3;PDHB;DTX4;ZNF25;C21ORF119;CYTH3;BCLAF1;ZNRF3;
;FAM64A;ST6GALNAC3;RAPGEF6;PRTFDC1;SHC4;HDAC5;USP15;BHLHB9;DLX6;TMTC3;UQCR10;ZM

;SHC4;HDAC5;USP15;PFKFB3;BHLHB9;LUZP1;DCUN1D5;UBA6;CREBL2;PDHB;UQCR10;PAPSS2;NXF
C2;FAM64A;CD47;WDFY2;PAFAH1B2;SHC4;DLX1;PTGFR;RALA;MTMR3;ABHD2;SRSF1;TYRP1;FAM12
REG;GPRIN3;NRG3;CRISPLD2;IL1RAPL1;ELMO1;DCX;AGTR2;SMTNL2;PSMD11;IGSF3;DLX6;RNF180;
;COMMD3-BMI1;SCG3;CD47;COL6A5;MAPRE2;RNF165;GPM6A;SHC2;HPGD;RNF180;PRUNE2;GLIS3;
RF115;CRISPLD1;CRISPLD2;IL1RAPL1;CNKSR3;ADAM12;CLDN18;COL6A5;SMTNL2;KANK4;PRTFDC
2;SLC1A4;UQCR10;C1ORF52;ZNF25;FAM117B;LRP6;DRP2;FBXO40;PURA;BCLAF1;TMEM65;TMEM203
3;KANK4;PPIC;SHC4;CRP;USP13;SHC2;TBC1D19;DLX6;PRUNE2;ADD3;JAKMIP2;ARHGAP20;GBX2;R
TSJ2;HPCAL4;BCMO1;CRISPLD2;ANKRD40;FAM64A;RAPGEF6;DLX1;BHLHB9;PLAG1;DLX6;SHOX2;P
DLX1;SET;PLAG1;FUT11;AGAP2;MTMR9;TUBD1;LRP8;ZMPSTE24;ERI1;TMEM68;ATXN1L;NT5DC3;VT
RMCX6;ZNF236;ST6GALNAC3;MAPRE2;SLC29A3;KANK4;TBCEL;SET;DCTN2;ABHD4;DLX6;SLC1A4;M
AM5;WDFY3;SLC25A12;DLX1;USP15;BHLHB9;ARL3;CREBL2;UQCR10;GRAMD4;NPAT;BCL2L13;RIC8B
RMCX6;KANK4;PRTFDC1;KCTD16;PPIC;GPM6A;TBC1D19;BHLHB9;PPM1L;LUZP1;PDE1B;RNF180;FU
;RNF180;SHOX2;NPR3;PRUNE2;NDNF;LRP8;PAPSS2;FBLN5;NCKIPSD;AP3M2;DRP2;RXRA;TMEM248
10;EREG;RAP2C;GPRIN3;ADCY9;CDC37;ADAM12;ARMCX6;ELMO1;FAM84A;SMTNL2;CGGBP1;SLC2
HC4;BHLHB9;PDE1B;PALM2;C17ORF96;FAM117B;PAPSS2;ZMPSTE24;NPAT;NXF1;RIC8B;TMEM203;PR
4L;MTMR9;DTX4;ZMPSTE24;NPAT;RNF217;VTI1A;MCMBP;GSE1;MARK1;ABCA1;B3GALNT2;MCTS1;ZE

RGIC2;TMEM106A;SLC29A3;RALA;YTHDC1;UBA6;DCUN1D1;TMTC3;FOXO1;PAPSS2;NEURL1B;NKAIN
MCX6;TBC1D25;TFAM;FAM64A;ZNF236;TMEM106A;PRTFDC1;PPIC;HDAC5;ATL3;DCTN2;ATL2;FBLN5
318;CMPK1;SCG3;DCP2;GLYR1;TBC1D19;PPM1L;ZDHHC20;ABHD2;RHOBTB3;TUBD1;MAT2B;DTX4;C
ADRA2A;PCDHA10;DKK3;DHX40;DDX19B;NRG3;NAA35;FAM64A;MAPRE3;SCG3;SPEM1;DLX6;ABHD2
0;RHOBTB3;MTMR9;CREBL2;ZBTB44;ZNF25;TRHDE;C21ORF119;DRP2;BCL2L13;PURA;OGFRL1;APH
A;MAPRE3;SMTNL2;NPTXR;TMEM106A;KCTD16;KANK2;SHC4;TBC1D19;MYRF;PPM1L;ARL3;RHOBT
IP;ASPHD2;CRK;ZNF597;KCTD16;KANK2;NECAB3;ARL3;PPM1H;C1ORF52;APCDD1;DTX4;FAM117B;C
2;PDHB;UQCR10;TRAK2;C21ORF119;NPAT;BCL2L13;BCLAF1;TMEM65;TMEM203;TNKS2;TDG;UBN2;V
C1ORF115;ARMCX6;ZNF236;ST6GALNAC3;KCTD16;TBCEL;BHLHB9;PALM2;PRUNE2;FAM129A;COL1
IGEF5;DLX1;PTGFR;NECAB3;MYRF;DLX6;PRUNE2;DTX1;ZBTB4;TRHDE;ADD2;ZDHHC18;GBX2;NSD1
LYR1;NECAB3;ATL3;DCTN2;YTHDC1;FUT11;PPM1K;FBLN5;NCKIPSD;AP3M2;PPP2CA;NXF1;PABPN1;
LMO1;MAPRE2;PPIC;RNF165;DLX1;TBCEL;BHLHB9;HPGD;PDE1B;DLX6;RNF180;NPR3;GATA6;SLC1A
MO1;COL6A5;SMTNL2;SHC2;ADCYAP1R1;PFKFB3;PSMD11;HPGD;RNF180;HSPA4L;FAM129A;TRHDE;
AC5;TBC1D19;PFKFB3;NECAB3;DCUN1D5;SEMA3A;FUT11;TMTC3;ZBTB44;UQCR10;ZBTB4;ZMPSTE2
YR1;ATL3;UQCR10;PURA;NXF1;TMEM203;ATXN7;MIER3;MGAT5;MCMBP;TRIM44;S100PBP;SLC25A24
SLC29A3;RNF165;SHC4;GPM6A;TBCEL;BHLHB9;PALM2;SHOX2;PRUNE2;FAM129A;GRAMD4;FBLN5;J
TMTC3;GSKIP;MTDH;RNF214;SNX1;ATXN7;ERI1;MIER3;TMEM248;VTI1A;MCMBP;CLSPN;CPSF7;SRD
D11;ATL3;DCTN2;ABHD2;PALM2;HSPA4L;PRUNE2;MTMR9;UQCR10;ZNRF3;APH1B;RIC8B;MAT2A;PAB
M2;TMTC3;UQCR10;C17ORF96;FBXO40;SNX1;APH1B;EFS;RIC8B;VTI1A;C10ORF2;C9ORF171;GPC5;
MTC3;CREBL2;PDHB;UQCR10;TRAK2;GJC1;DRP2;NPAT;SNX1;APH1B;RIC8B;TMEM203;RBBP5;VTI1A;
101B;CRISPLD1;CRISPLD2;SCG3;SLC25A12;TRIB2;SHC4;USP13;ADCYAP1R1;FAM114A1;FAM129A;R
ANC2;TFAM;TERF2IP;NPTXR;TMEM106A;NECAB3;ZDHHC20;PDHB;GRAMD4;NCKIPSD;DRP2;ZNRF3;
D1;ELMO1;SCG3;DENND6A;TMEM106A;FAM114A1;LUZP1;PRUNE2;PAPSS2;FBLN5;SLC17A6;SCN3B;
B2;MAGEE1;NEK7;NBEAL1;KLHL23;NSG1;L1CAM;LSM5;BTBD9;REEP1;PARP11;OCLN;EFNA3;LHX4;R
DAC5;NECAB3;ABHD4;SEMA3A;PPM1H;TUBD1;APCDD1;DTX4;FOXO1;MED12L;JAKMIP2;TRAK2;NEU
F2IP;COL6A5;ZNF236;SMTNL2;FYTTD1;TBC1D19;TBCEL;NECAB3;PPM1L;ABHD4;HPGD;PDE1B;PALM
PGD;YTHDC1;UBA6;RNF180;PALM2;NPR3;PPM1E;CYTH3;GJC1;NXF1;ZNRF2;TRA2B;TMEM68;CHAD;
D19;BHLHB9;RNF180;SEMA3A;TMTC3;LRP6;AP3M2;NKAIN1;PURA;NXF1;ZNF629;TDG;UBN2;NT5DC3;
X1;GPM6A;TBC1D19;BHLHB9;PDE1B;NPR3;TTL;ADD2;GJC1;FBXO40;ZNRF2;ARHGAP20;PABPN1;TR
HOXD12;C1ORF52;GNA13;RIC8B;PCDHA1;TRA2B;PCDHA3;PCDHA2;SRSF10;EIF4E;TRIM44;PCDHA7;
JBD1;PDHB;UQCR10;C17ORF96;PAPSS2;FBLN5;GJC1;BCL2L13;ZDHHC18;GPC1;TMEM68;NT5DC3;S
X6;PALM2;PDHB;ZMPSTE24;TRAK2;TMEM203;VTI1A;PCDHA3;PCBD2;RIMKLA;MPZL3;N4BP2;SMND
TNL2;NPTXR;PPIC;RNF165;DLX1;DLX6;PRUNE2;FAM129A;HIF1A;GNA13;NEURL1B;GBX2;PABPN1;PC
1;RAD9A;CALCOCO2;CHURC1-FNTB;DUSP19;TNFAIP3;BMI1;PFAS;RBM3;EEF2K;CAPZB;XPO4;MFSD
B9;DCTN2;IGSF3;DCUN1D5;MTMR9;NOP9;GNA13;NXF1;BCLAF1;EFNB3;TMEM203;G3BP1;IGF2BP1;E
F9;KANK2;DLX1;ARL3;CREBL2;SLC1A4;PDHB;UQCR10;ZNF25;C17ORF96;TRAK2;C21ORF119;NXF1;S

P;GLYR1;PHLPP2;SET;ATL3;UBA6;MTMR9;DLST;PDHB;TTL;NXF1;ZNRF3;ERI1;NT5DC3;IGF2BP1;C10O
ARL3;SEMA3A;PPM1H;TUBD1;UQCR10;DTX4;FOXO1;ZMPSTE24;TRAK2;NPAT;ZNRF3;APH1B;TMEM20
TL3;SEMA3A;AGAP2;FAM129A;NDNF;ADD3;PPM1E;GRAMD4;COL19A1;ADD2;GBX2;CARNS1;SLC17A
D2;CDC37;TET3;ARMCX6;NOS1AP;FAM84A;TBC1D25;PGAM5;CRK;RNF165;PPM1L;DCTN2;ABHD2;PA
X6;CPSF7;SRD5A1;XRCC5;EDEM1;NEK7;NBEAL1;KLHL23;BFAR;MARCH7;BTBD9;JMY;LSM14B;PLEK
HB9;TSHZ3;INSIG2;ARL3;RNF180;SHOX2;FUT11;AGAP2;GATA6;UBE2J1;FBXO40;RIC8B;EFNB3;PCDH
IOBTB3;SLC1A4;APCDD1;GJC1;PLXNA2;HIVEP2;MYH10;LYN;MGAM;EGR4;ZBTB16;ST8SIA3;ZBTB10;S
SF3B3;UBA6;ZDHHC20;GLIS3;MAT2B;COL19A1;LRP6;BCL2L13;CTIF;HSPH1;MGAT5;TMEM68;FAM63B;
;PFKFB3;PDE1B;NPR3;NDNF;PAPSS2;NEURL1B;RNF214;APH1B;MGAT5;VTI1A;GSE1;GPC5;LONRF3;
NXF1;APH1B;PABPN1;ATXN7;MGAT5;VTI1A;APBB2;SRGAP2;HOXC8;TRIM44;SLC25A24;TRIM41;TIMMD
T11;MTMR9;GIGYF2;APH1B;ATXN7;TMEM248;G3BP1;EIF4EBP2;MCTS1;TMEM86A;CPSF7;ZBTB14;SM
3;ATL2;BCL2L13;NXF1;ZNF706;TMEM203;ATXN7;FAM63B;C10ORF2;EIF4EBP2;MCMBP;LONRF3;SCN3
4;NXF1;EFNB3;TNKS2;ATXN7;MIER3;MCMBP;LONRF3;IP6K1;SRGAP2;TRIM44;S100PBP;SLC25A24;A
NL2;TMEM106A;SLC29A3;SHC4;BHLHB9;NECAB3;INSIG2;ARL3;SHOX2;PRUNE2;FUT11;ZBTB44;PPM1

21;PPM1E;PAPSS2;FBLN5;NCKIPSD;CYTH3;DRP2;NEURL1B;NXF1;ARHGAP20;ZNRF3;ZMAT3;NSD1;N
;TBCEL;SET;PFKFB3;PSMD11;IGSF3;RNF180;HSPA4L;FUT11;DTX1;DTX4;ZBTB4;ZMPSTE24;CYB5R4;
F7;MAGEE1;SRD5A1;XRCC5;GFPT1;KLHL23;BFAR;MARCH7;BTBD9;HNRNPK;JMY;STRN;LSM14B;RBM

3;ADD3;COL19A1;NCKIPSD;FBXO40;CYB5R4;ZNRF2;TMEM203;C9ORF171;LONRF3;SCN3B;MBNL3;M
HDAC5;YTHDC1;INSIG2;DLX6;ATL2;SRSF1;PPM1H;PPM1K;C1ORF52;AP3M2;NXF1;ZNRF3;ZNF629;MA
GFPT1;MRPL27;SREK1IP1;PARVA;BFAR;CNOT6;PQLC1;ZFP62;SP4;OSTC;FUBP1;LSM14B;NFE2L1;C
3;PALM2;NPR3;FUT11;GATA6;GLIS3;DRP2;NSD1;GPC5;MPZL3;ATOH1;MARK1;CA12;ABCA2;TMEM86A
MTMR9;FBLN5;DFNB59;ARHGAP20;RBBP5;ZMAT3;NT5DC3;HAS2;GPC5;SCN3B;EIF4E;ATOH1;GABRA
1ORF119;PURA;DFNB59;BCLAF1;TMEM203;PABPN1;MGAT5;TDG;E2F1;SLIT2;IP6K1;MAP3K2;EIF2B2;

PP2;HDAC5;FAM114A1;ABHD4;UQCR10;ZBTB4;PAPSS2;TMEM203;MAT2A;PABPN1;MGAT5;SLIT2;MPZ
PAX2;DKK3;BCMO1;ENAH;RAP2A;SNPH;NOS1AP;RBMS1;ASPHD2;MAPRE3;ST6GALNAC3;RNF165;N
;SNAP23;SMG7;RND3;PITPNC1;RBM3;ARL5B;SART3;SIPA1L1;PGM3;CNPPD1;GPR135;ARHGEF10;W
DTX4;MED12L;FBLN5;GJC1;FBXO40;MIER3;CHAD;PLXNA2;IP6K1;RIMKLA;MARK1;CADM3;FZD3;EGR4
CKIPSD;CYTH3;AP3M2;CTIF;RNF214;ZDHHC18;APH1B;PABPN1;MIER3;USP1;C10ORF2;MPZL3;N4BP
PPM1L;DCTN2;ATL2;FUT11;AP3M2;PURA;NXF1;TMEM203;PABPN1;ATXN7;MIER3;MCMBP;BCL2L2-PA
NK4;PPIC;DLX1;PTGFR;SHC2;FAM114A1;HPGD;DLX6;NPR3;PRUNE2;PAPSS2;NOP9;NCKIPSD;NSD1;
T11;MTMR9;PDHB;NOP9;UBE2J1;GNA13;NXF1;HSPH1;EFNB3;TMEM203;MAT2A;PABPN1;EIF4EBP2;P
VTI1A;MCMBP;SRGAP2;SNX6;ABCA2;CPSF7;MAGEE1;SRD5A1;EDEM1;KLHL23;BFAR;MARCH7;BTBD
13;NXF1;RNF214;EFNB3;TMEM203;PABPN1;CARNS1;CHAD;LYN;CPSF7;TIPARP;SRD5A1;CSNK1A1;K
M129A;TRHDE;ARHGAP20;PCDHA1;NRIP1;PCDHA4;C9ORF171;PCDHA3;S1PR3;PCDHA2;PCDHA9;S
X6;MAPRE3;NAT8L;MAPRE2;FKBP6;CHST3;SHC4;ROCK1;FAM114A1;NUFIP2;TUBD1;HOXD12;ADD3;F
3;COL6A5;NPTXR;NAT8L;SHC4;DLX1;SHOX2;NRXN3;DTX4;GRAMD4;TRHDE;COL19A1;CTIF;PGRMC1;
HC2;ACBD5;TIMMDC1;TIPARP;CSNK1A1;NEK7;GFPT1;GATC;PDAP1;PLEKHA3;GTF2H1;PARVA;BFAR;
;PURA;NXF1;MAT2A;PABPN1;VTI1A;TRIM44;SEPT10;CBX5;FZD5;SRD5A1;NEK7;SUSD1;MRPL27;MTS
RF3;PABPN1;PCDHA1;PCDHA5;PCDHA4;MAPK1;PCDHA3;SLIT2;PCDHA2;TRIM44;PCDHA7;SLC25A24;

X1;NCKIPSD;AP3M2;NXF1;TMEM203;PABPN1;CARNS1;GLTSCR1L;SMNDC1;EIF2B2;CBX5;KCNIP2;G
C2;HOXD12;C1ORF52;ARHGAP20;GBX2;LONRF3;SNX8;RAI2;ATOH1;PCDHA6;CA12;IGBP1;EGR4;GAB
ARNS1;PLXNA2;APBB2;S1PR3;EIF4E;SNX8;JAM2;SLC25A24;PHC2;SUSD1;KLHL23;PARVA;S100B;ASP
UNE2;TYRP1;FAM129A;C1ORF52;HIF1A;GRAMD4;PABPN1;PCDHA1;TRA2B;PCDHA5;PLXNA2;FAM63B;
CDHA3;LONRF3;PCDHA2;RIMKLA;PCDHA9;PCDHA8;PCDHA7;PCDHA6;ZBTB10;PHOX2B;REEP1;ARH
XR;SHC4;SHC2;TBC1D19;NECAB3;ARL3;SHOX2;NOP9;NCKIPSD;GJC1;SNX1;EFNB3;LONRF3;RIMKLA
EL;CUL3;ATL2;PRUNE2;FAM129A;DTX1;ADD3;LRP6;NCKIPSD;GNA13;GBX2;NSD1;CARNS1;HAS2;VP
C1ORF52;NCKIPSD;AP3M2;ZNF629;TMEM203;MAT2A;PABPN1;MGAT5;C10ORF2;PCDHA3;IP6K1;BCL2

;RXRA;GBX2;PCDHA1;PRDM16;PCDHA5;PCDHA4;PCDHA3;LONRF3;PCDHA2;ZNF423;PCDHA9;PCDH
\;TRA2B;E2F1;EIF4EBP2;GLTSCR1L;IGBP1;CBX6;TIMMDC1;EIF2B2;FZD5;USP9X;CSNK1A1;EPT1;KCN
ORF2;TAC3;LONRF3;SLIT2;SEPT10;SEPT12;RANBP3;EGR4;FAM78A;LGI2;ATP2B4;LAPTM5;MTSS1L;A
MEM203;PABPN1;MGAT5;TDG;C10ORF2;ZNF423;SEPT10;CREBBP;EIF2B2;CNBP;SS18L2;GATC;SREK
214;ATXN7;MCMBP;IP6K1;TIPARP;SRD5A1;NEK7;SUSD1;GATC;PLEKHA3;GTF2H1;PARVA;SOD2;BTBD
3HLHB9;NECAB3;ARL3;CREBL2;UQCR10;ZNF25;PURA;RNF214;ZNF629;RNF217;N4BP2;SNX8;ACBD5;
1;INSIG2;AGAP2;DCUN1D1;MTMR9;GSKIP;NOP9;MTDH;NCKIPSD;FBXO40;RNF214;EFNB3;ATXN7;CH
MAT3;SCN3B;RIMKLA;PLXNA3;MCTS1;FZD3;EGR4;MAGEE1;XRCC5;HELZ;L1CAM;ESR1;PARP11;ARH
D2;TET3;TBC1D25;TFAM;NAA38;SLC25A12;GLYR1;KANK2;ATL3;DCTN2;YTHDC1;ATL2;SRSF1;FUT11;F
M177A1;USP1;S1PR1;C10ORF2;S1PR3;N4BP2;SNX8;S100PBP;TIMMDC1;SPRYD7;GTF2H5;ADAT2;TP
NPR3;PRUNE2;SLC1A4;ZDHHC21;COL19A1;DRP2;PGRMC1;BCLAF1;RBM12;MPZL3;ZC3H14;JAM2;AB
SLC25A12;DTL;ZNF597;PRTFDC1;GLYR1;TBC1D19;BHLHB9;NECAB3;FAM114A1;ARL3;FUT11;PDHB;U
RBMS1;MAPRE3;ARHGEF5;KCTD16;SHC4;HDAC5;TBC1D19;RNF180;FAM129A;PPM1K;PAPSS2;FBLN
SRSF1;FUT11;FAM129A;PPM1K;AP3M2;PURA;NXF1;PABPN1;ATXN7;MIER3;MGAT5;MCMBP;RPP14;M
F52;TRHDE;PCDHA1;PCDHA5;PCDHA4;PCDHA3;PCDHA2;SNX8;PCDHA8;TRIM44;PCDHA7;SLC25A24
F7;TRIM41;EIF2B2;RANBP3;CBX5;FZD5;SRD5A1;CSNK1A1;GATC;SMAD7;SP1;VAPB;RFWD3;SERBP1
F4E;RAI2;ATOH1;GABRA1;SEPT12;MAGEE1;FAM78A;KCNIP2;SUSD1;LAPTM5;S100B;ASPRV1;TSHR;A
FBLN5;NEURL1B;TMEM248;S1PR3;SLIT2;ABCA2;GABRA1;FZD3;FBXL17;KLHL23;KCNRG;ASPRV1;TS
3;TRIM44;RAB11FIP5;ABCA1;BCL11B;FAM46C;LAPTM5;RASSF8;PARVA;KCNRG;HIPK1;SYNJ2BP;STX
4;RNF214;ZDHHC18;APH1B;GBX2;MIER3;E2F1;C10ORF2;SNX8;HOXC8;RABGAP1L;SPRYD7;GTF2H1;
;NT5DC3;GPC5;PCBD2;IP6K1;BCL2L2-PABPN1;MPZL3;S100PBP;B3GALNT2;ST13;GATC;PDAP1;BTBD
3;ZDHHC18;ZNRF3;APH1B;PABPN1;MIER3;C10ORF2;S100PBP;CA12;TMEM86A;GATC;PDAP1;TPCN1;
23;RELT;CBX5;TXNL1;MRPL27;NFATC3;EPT1;PARVA;KCNRG;BFSP2;ALDH6A1;RNF148;MARCKS;ETN
NF214;ZDHHC18;APH1B;C10ORF2;PCBD2;RIMKLA;BCL2L2-PABPN1;MPZL3;SNX8;MAGEE1;SORT1;NE
;CHAD;IGF2BP1;GPC5;PCDHA2;RIMKLA;PCDHA9;MARK1;ABCA1;GABRA1;SEPT12;B3GAT2;GABRA3;

F2;ZC3H14;TIMMDC1;FAM78A;SS18L2;HIPK1;GTF2H5;ADAT2;TPCN1;HEYL;ALDH6A1;ZNF70;FRAS1;P
I;SNX6;SYT5;IGBP1;MGAM;CPSF7;EIF2B2;CBX5;CSNK1A1;EPT1;KCNRG;MARCH7;GATAD2B;VAPB;O
F2;SLIT2;SNX8;HOXC8;ZC3H14;SNX6;TIMMDC1;GTF2H1;GTF2H5;TPCN1;ZNF70;GPAM;ID2;NUPL1;DN
;PCDHA1;PCDHA3;SNX8;TRIM44;SLC25A24;TIPARP;NEK7;KLK13;PARVA;BFSP2;VAPB;SESTD1;TME
P1;C10ORF2;CLSPN;N4BP2;S100PBP;ABCA1;MAGEE1;ESR1;ADAT2;ABHD15;TPCN1;ALDH6A1;ZNF7
CSNK1A1;GATC;PLEKHA3;GTF2H1;BFAR;TTC9;VAPB;PITHD1;ID2;KLHL7;CAPZA1;OCIAD1;RLIM;LSM1
3;TIMMDC1;EIF2B2;CBX5;SORT1;SUSD1;MRPL27;FOXJ2;GATC;NECAP2;GPAM;PITHD1;CALM1;DNAL
2;PABPN1;MIER3;E2F1;C10ORF2;SLIT2;CPSF7;PDAP1;ADAT2;TPCN1;ZNF70;GPAM;KIAA0101;DNAL1;
C1;PCDHA7;TRIM41;TIMMDC1;EIF2B2;CBX5;RASSF8;KCNRG;GATAD2B;ID4;HNRNPC;RAD9A
2;SLC17A6;LONRF3;S1PR3;PCDHA2;SCN3B;SYT5;B3GAT2;SUSD1;ASPRV1;FRAS1;PQLC1;CAMK4;SL
JMF1;NECAP2;PITHD1;ID2;FUBP1;ARMCX6;ID4;TERF2IP;TECPR2;DNAL1;PLEKHM3;RAD9A
A;ARMCX6;SLC25A12;DTL;CRK;PLEKHM3;RAD9A;ITM2C

;EIF4EBP2;MLEC;RBM12;PPARGC1B;SNX6;ABCA1;EDEM3;CREBBP;EDEM1;NBEAL1;TRAPPC8;HELZ;
UBE2J1;BCL2L13;AP5M1;VTI1A;IP6K1;ZC3H14;SNX6;CREBBP;CPSF7;NBEAL1;LNPEP;TRAPPC8;HEL
;ARHGAP20;RNF217;PRDM16;PCDHA4;PCDHA3;HIVEP2;HOXC8;RAI2;MARK1;LYN;ABCA1;TFAP2B;C
;GJC1;NKAIN1;EFNB3;PRDM16;SCN3B;RIMKLA;MARK1;TFAP2B;CADM3;FZD3;EGR4;BCL11B;ZBTB16
1;SERINC3;UBE2K;TECPR2;NFE2L1;LRP12;CC2D1B;DOCK5;NRP2;DYRK3;MOCS3;BMPR2;PRR3;PP
PLXNA2;HAS2;GPC5;LONRF3;RAI2;MARK1;LYN;ZNF462;SEPT10;ZBTB16;SMAD9;DEK;SMARCA1;SO
CN3B;MARK1;TFAP2B;FZD3;EGR4;BCL11B;NSG1;GDF6;SYT7;PHOX2B;REEP1;EFNA3;PPP1R1B;LHX
P20;EFNB3;PRDM16;PLXNA2;PCDHA2;MARK1;LYN;FZD3;EGR4;ZBTB16;PARVA;NSG1;SMARCA1;GD
NB3;CHAD;TAC3;LONRF3;RAI2;SYT5;TFAP2B;CADM3;EGR4;ZBTB16;ST8SIA3;SLC4A10;SMAD9;GDF
SPL2;PPP1R9A;PITPNC1;ELAVL2;ADAMTS2;SPRED1;KIF1B;CCDC50;GPR37;MME;ANO6;CDYL2;ADR
RF96;GIGYF2;MED12L;FBLN5;NCKIPSD;ARHGAP20;RIC8B;ATXN7;N4BP2;MYH10;MCTS1;CCZ1B;MA
M3;FZD3;EGR4;BCL11B;SMAD9;GDF6;REEP1;EFNA3;RCAN2;ID4;LHX6;LHX4;LRP12;FGFR1;GPR27;D
LNPEP;BTBD9;RCAN1;ARHGAP31;SP1;SESTD1;CAPRIN1;NRP2;TTC22;DIRAS2;DUSP19;SIX1;CTDSP
SIX1;ELAVL2;HK2;GRM5;MECOM;SH3PXD2A;SCN5A;PRKG1;HS3ST3B1;ZCCHC24;GPR37;MME;GRID1
;GATA6;GLIS3;RHOBTB3;AARD;APCDD1;TRHDE;FOXO1;FBLN5;CTIF;ARHGAP20;ZMAT3;CHAD;PRD
1;PARP11;HNRNPK;OSTC;ID4;LSM14B;LRP12;COX7B;MOCS3;PPWD1;SIX1;BMI1;AFF1;MED17;MRPL4
;B3GALNT2;TMEM86A;FAM78A;NEK7;RASSF8;LNPEP;KLHL23;LSM5;BTBD9;GLUD2;DIAPH2;ARHGAP
5;UBE2J1;NKAIN1;ARHGAP20;RAP1A;CHAD;HIVEP2;SCN3B;RIMKLA;MARK1;RABGAP1L;FAM78A;SM
C9ORF171;PCDHA3;HOXC8;ZC3H14;LYN;CCZ1B;EGR4;FZD5;SMURF2;ST13;SS18L2;PARVA;LSM5;BT
FOXO1;MED12L;GJC1;CTIF;NKAIN1;ARHGAP20;PCDHA1;RNF217;PCDHA5;PCDHA4;PCDHA3;HIVEP2;
ARCH7;BTBD9;PARP11;ALDH6A1;OSTC;RCAN2;STRN;GPR27;PPWD1;PFAS;PITPNC1;ELAVL2;MED17
ONRF3;CLSPN;HOXC8;TMEM86A;CBX6;CBX5;FZD5;SMURF2;ZBTB16;LNPEP;PARVA;BTBD9;RCAN1;G
R9;ZNF25;FBLN5;NCKIPSD;BCL2L13;CYB5R4;RNF214;RAP1A;EFNB3;PCDHA1;ATXN7;MIER3;VTI1A;P
CADM3;BCL11B;GDF6;PHOX2B;EFNA3;PQLC1;RCAN2;PPP1R1B;LHX6;LHX4;HRK;NRP2;ANKRD33B;O
TTD1;PTGFR;GPM6A;NUFIP2;SHOX2;ATL2;SPATA2;ZDHHC21;ZBTB44;ZNF25;LRP8;PPM1E;FAM117B;
;PCDHA7;TMEM86A;CCZ1B;EGR4;SMURF2;ZBTB16;SS18L2;LNPEP;LSM5;FBXO30;RCAN1;GLUD2;AR
RPL27;BFAR;ABHD15;SMAD7;REEP1;ALDH6A1;ZZZ3;ID2;ID4;SERINC3;HRK;DOCK5;MOCS3;PHF20;C
ST13;LNPEP;LSM5;BTBD9;RCAN1;GLUD2;ARHGAP31;SP1;SESTD1;NRP2;BMPR2;SNAP23;DUSP19;T
E1B;DLX6;RNF180;CUL3;SHOX2;MTMR9;SPATA2;ZNF25;ARHGAP20;EFNB3;ATXN7;PRDM16;MCMBP;
N;ZC3H14;TMEM86A;CBX6;SMURF2;ZBTB16;LNPEP;BTBD9;FBXO30;GLUD2;ARHGAP31;SP1;SESTD1
GAP31;SP1;PPP1R1B;LRP12;BMPR1A;NRP2;BMPR2;SNAP23;DIRAS2;DUSP19;TNFAIP3;CTDSPL2;PP
MARCH7;BTBD9;AZIN1;RCAN1;PARP11;STRN;LSM14B;TECPR2;GPR27;COX7B;MOCS3;PTPRM;LITAF;

6;HNRNPK;FUBP1;CAPRIN1;HNRNPC;CALM1;SERINC3;UBE2K;COX7B;CELF1;ADK;TNFAIP3;RSF1;CT
;DNAL1;RAD9A;SNAP23;DUSP19;TNFAIP3;PPP1R9A;SMG7;MYPN;PITPNC1;ELAVL2;ADAMTS2;SPRE
LA;MARK1;TFAP2B;FZD3;EGR4;BCL11B;ZBTB16;GRIN2B;SYT7;PHOX2B;REEP1;OCLN;EFNA3;RCAN
CC2D1B;BMPR1A;NRP2;COX7B;MOCS3;PHF20;PRR3;ADK;PPWD1;CTDSPL2;RCSD1;SMG7;CDC73;D
CZ1B;EGR4;SMURF2;ZBTB16;EPT1;LNPEP;PARVA;FBXO30;GLUD2;ARHGAP31;SP1;SESTD1;PPP1R1
GLUD2;ARHGAP31;SP1;SESTD1;JMY;BMPR1A;FGFR1;NRP2;BMPR2;KCNC2;TTC22;SH3KBP1;SNAP2
SS18L2;ZBTB10;LNPEP;FBXO30;GLUD2;ARHGAP31;SP1;PPP1R1B;DNAL1;FGFR1;NRP2;CALCOCO2;
O30;PARP11;HNRNPK;ZFP62;ID2;FUBP1;NABP1;STRN;UBE2K;TECPR2;RAD9A;COX7B;TNFAIP8;BTG
R1;NRP2;BMPR2;TTC22;SNAP23;DUSP19;TNFAIP3;CTDSPL2;PITPNC1;ADAMTS5;ADAMTS2;C1QBP;
1;OCLN;DIAPH2;SP1;GPR27;NRP2;BMPR2;SH3KBP1;SNAP23;DIRAS2;DUSP19;TNFAIP3;CTDSPL2;PP
HELZ;ESR1;AZIN1;HNRNPK;NCEH1;ZZZ3;OSTC;FUBP1;SERINC3;UBE2K;COX7B;MOCS3;SNAP23;C1
NAL1;PLEKHM3;RAD9A;DOCK5;DYRK3;TNFAIP8;GABRB1;DIRAS2;ADK;PHF8;DTWD1;NHSL2;SPRED1
TRN;LSM14B;DNAL1;PLEKHM3;RAD9A;GPR27;C9ORF91;BMPR2;ADK;PPWD1;PTPRM;TNFAIP3;JPH1;
180;CUL3;SHOX2;RHOBTB3;MTMR9;SPATA2;SLC1A4;ZNF25;C17ORF96;HSPH1;ATXN7;ZMAT3;PRDM1
6A;FZD5;BCL11B;SMURF2;ZBTB16;ZBTB10;LNPEP;TRAPPC8;MARCH7;FBXO30;DIAPH2;ARHGAP31;
CDHA2;SCN3B;RIMKLA;PCDHA6;MARK1;TFAP2B;CADM3;FAM78A;L1CAM;SMARCA1;GDF6;GRIN2B;
RHGAP31;SP1;SESTD1;LRP12;FGFR1;GPR27;NRP2;DYRK3;BMPR2;DUSP19;TNFAIP3;CTDSPL2;PPP
FY3;SCG3;NPTXR;DLX1;PTGFR;GPM6A;SHC2;PSMD11;PDE1B;RNF180;SHOX2;NPR3;GATA6;GLIS3;
62;NABP1;STRN;CALM1;MXD1;BMPR1A;GPR27;MOCS3;SLC7A14;DUSP19;TNFAIP3;MED17;MRPL42;

;UBE2K;NFE2L1;PLEKHM3;RAD9A;BMPR1A;HRK;NRP2;KCNC2;TTC22;CALCOCO2;PRR3;ADK;PPWD1
R1B;ID4;RAD9A;GPR27;NRP2;DYRK3;PDE3B;BMI1;RND3;AFF1;MED17;ING4;MRPL42;ARL5B;SIPA1L1;S
I;CHURC1-FNTB;BMI1;LITAF;PFAS;MED17;PHF8;NHSL2;MECOM;KIF1B;GPRASP2;GPR135;GPR37;MM
D1;TMTC3;CREBL2;DTX4;C17ORF96;ZMPSTE24;NPAT;BCL2L13;RNF214;RIC8B;ZNF706;ZMAT3;VTI1A;
R1A;GPR27;NRP2;DYRK3;BMPR2;TTC22;TNFAIP3;SIX1;CTDSPL2;JPH1;PITPNC1;PHF8;ADAMTS5;NHS
;SESTD1;PPP1R1B;DNAL1;FGFR1;BMPR2;SNAP23;DUSP19;FMN1;CTDSPL2;PPP1R9A;JPH1;MYPN;P
I2;ARHGAP31;SP1;SESTD1;JMY;FGFR1;BMPR2;SH3KBP1;SNAP23;PDE3B;DUSP19;SIX1;FMN1;CTDS
;SLC4A10;RASSF8;PARVA;SMARCA1;GRIN2B;SYT7;PHOX2B;EFNA3;RCAN2;PPP1R1B;ID4;BMPR1A;H
N;IP6K1;RBM12;EIF2B2;CBX5;ZBTB14;XRCC5;ZBTB16;MRPL27;RASSF8;TRAPPC8;HELZ;BFAR;MARC
NPAT;BCL2L13;RNF214;ZNF706;VTI1A;PCDHA5;PCDHA4;C9ORF171;PCDHA3;IP6K1;RIMKLA;ZNF148;M
N3B;RIMKLA;MARK1;TFAP2B;SLC4A10;NSG1;L1CAM;GRIN2B;PHOX2B;REEP1;OCLN;PPP1R1B;LHX4
E2K;NFE2L1;DAND5;RAD9A;ITM2C;CREBZF;DOCK5;COX7B;TNFAIP8;MOCS3;BMPR2;LITAF;CDC73;M
;TECPR2;PLEKHM3;LRP12;RAD9A;BMPR1A;DOCK5;MOCS3;SNAP23;ADK;CDC73;ALAD;XPO4;NKRF;K
PARP11;ALDH6A1;NCEH1;ID4;LSM14B;MXD1;RBM28;COX7B;PTPRM;BMI1;SMG7;LITAF;PITPNC1;MED
A10;SMAD9;GRIN2B;PHOX2B;ABHD15;REEP1;RCAN1;RCAN2;PPP1R1B;LHX6;LHX4;HRK;GPR27;KCN
OCLN;RCAN2;PPP1R1B;ID4;LHX6;LHX4;HRK;NRP2;GABRB1;ANKRD33B;ONECUT2;KCNC2;SLC7A14;T
;UBE2K;TECPR2;ITM2C;C9ORF91;CREBZF;COX7B;CTDSPL2;MED17;CKS1B;KIAA1549;ARL5B;XPO4;C
HA6;ABCA1;MCTS1;CBX5;ST13;SREK1IP1;AZIN1;PARP11;ALDH6A1;ARHGAP31;CDK1;USP37;SLC7A1
5;LNPEP;KLHL23;PARVA;LSM5;FBXO30;GLUD2;ARHGAP31;SP1;BMPR2;SNAP23;TNFAIP3;SIX1;CTDS
NF367;GPR135;NSUN3;ANO6;SORCS1;PAX5;PROX1;ZFY;ZFX;CDYL2;KAT2B;RAP2A;RAP2B;MAPRE2;K
;ZBTB10;LNPEP;PARVA;MARCH7;FBXO30;REEP1;OCLN;ARHGAP31;SP1;SESTD1;JMY;LRP12;FGFR1;
9;NECAB3;PDE1B;ARL3;RNF180;NPR3;SPATA2;ZNF25;MED12L;NPAT;APH1B;RIC8B;ATXN7;PRDM16;M
BTB3;SPATA2;CREBL2;SLC1A4;ZDHHC21;ZBTB44;FAM117B;ARHGAP20;EFS;ZNF629;ZMAT3;UBN2;C9
P1;ESR1;PARP11;ALDH6A1;PPP1R1B;LHX6;DYRK3;TTC22;PTPRM;MED17;ING4;ADAMTS5;ADAMTS2;S
BE2K;DNAL1;LRP12;CREBZF;DIRAS2;CDC73;ELAVL2;ING4;NHSL2;MB;DDI2;FAM73A;SH3BGRL2;KIF1E
RAD9A;BMPR1A;HRK;PRR3;ADK;PPWD1;RSF1;CTDSPL2;ING4;ARL5B;XPO4;PGM3;SH3BGRL2;TEAD
;TNFAIP8;MOCS3;DIRAS2;ADK;FMN1;CDC73;MED17;ING4;SPRED1;SH3PXD2A;MB;DDI2;TMEM108;SC
BTB3;MTMR9;SPATA2;ZDHHC21;ZBTB44;MAT2B;PDHB;PPM1E;FAM117B;PAPSS2;DFNB59;EFNB3;ZN

EGR4;SMURF2;LSM5;FBXO30;RCAN1;GLUD2;DIAPH2;ARHGAP31;SP1;SESTD1;LHX4;FGFR1;GPR27;
F2;SS18L2;MRPL27;SREK1IP1;TRAPPC8;FBXO30;CALM1;DNAL1;BMPR1A;NRP2;SNAP23;DIRAS2;PP
DC73;IL18BP;ING4;RBM3;SPRED1;CAPZB;MB;MAN1A2;C1QBP;TMEM108;METTL16;ARL5A;HS3ST3B1
TTC22;ADK;PPWD1;SIX1;ING4;SDR16C5;SPRED1;CAPZB;RBM7;UTP15;PLA2G12A;GPR37;TMED8;NS
23;PARVA;LSM5;FBXO30;PARP11;OCLN;SP1;LRP12;HRK;NRP2;DYRK3;BMPR2;SNAP23;DUSP19;TNFA
CAN1;GLUD2;DIAPH2;ARHGAP31;SP1;SESTD1;PPP1R1B;DNAL1;TNFAIP8;BMPR2;TTC22;SNAP23;TN
;C9ORF171;GPC5;JAM2;SLC25A24;TRIM41;USP9X;LNPEP;PLEKHA3;TMEM64;HIPK1;LSM5;BTBD9;HE
BTBD9;FBXO30;GLUD2;ARHGAP31;SP1;PPP1R1B;DNAL1;NRP2;BMPR2;TTC22;SH3KBP1;SNAP23;DU
4;MRPL42;UBL3;SART3;SH3PXD2A;TRIM3;SH3BGRL2;METTL16;UNKL;PHACTR4;VKORC1;MAPK1IP1L
PR2;FMN1;MYPN;PHF8;NKRF;MFSD5;DDI2;SH3BGRL2;SCN5A;CCDC50;NAA30;MYEOV;TFEB;C6ORF6
TNFAIP3;CTDSPL2;PPP1R9A;JPH1;RND3;PITPNC1;SPRED1;ALDH2;SH3BGRL2;KIF1B;HS3ST3B1;NC
WD1;TNFAIP3;PPP1R9A;ARL5B;TMEM108;RBM7;UBL7;HS3ST3B1;PLA2G12A;NSUN3;C3ORF62;AHSA2
IC;SHC4;DLX1;PTGFR;GPM6A;ADCYAP1R1;PDE1B;RNF180;SHOX2;NPR3;HSPA4L;AGAP2;GATA6;GLI
;LYN;TMEM86A;CCZ1B;EGR4;BCL11B;MAGEE1;ST13;MRPL27;LNPEP;SMARCA1;GLUD2;OCLN;NCEH
;KANK4;PPIC;SHC4;DLX1;GPM6A;SHC2;ADCYAP1R1;BHLHB9;PDE1B;NPR3;HSPA4L;GATA6;GLIS3;RH
;RCAN2;LHX6;LHX4;MXD1;FGFR1;COX7B;TTC22;PPWD1;SMG7;PITPNC1;ARL5B;NKRF;DDI2;CEP170
GFR1;TNFAIP3;ETFA;MYPN;ING4;DTWD1;MED14;ADAMTS2;EEF2K;MB;NCOA2;CHST7;VPS13C;LRRC4
B;SMG7;CDC73;ADAMTS5;SART3;SERP1;KIF1B;SCN5A;HS3ST3B1;ARHGEF10;WDR37;MMP2;GAB1;F
RAD9A;GPR27;DYRK3;SH3KBP1;RCSD1;SH3PXD2A;NKRF;RBM7;ZNF367;HS3ST3B1;ZCCHC24;TMED
1;PLEKHM3;LRP12;NRP2;ADK;TNFAIP3;RCSD1;PITPNC1;ING4;MED14;ARL5B;EEF2K;TRIM3;SH3BGR
F706;UBN2;NSD1;C9ORF171;PCBD2;MBNL3;MPZL3;N4BP2;IGBP1;TMEM86A;FZD5;SMURF2;ST13;KC
5;PLXNA3;MARK1;EDEM3;MCTS1;SRPK2;CREBBP;CPSF7;ST13;NBEAL1;NSG1;MARCH7;LSM5;BTBD
A;CGGBP1;PTGFR;TBC1D19;BHLHB9;PDE1B;DLX6;CUL3;NPR3;SPATA2;NPAT;RNF214;APH1B;RIC8B;E
DE3B;DUSP19;SMG7;AFF1;DTWD1;SERP1;NKRF;MAN1A2;TRIM3;CEP170;ARL5A;UBL7;UTP15;PLA2G

OCS3;BMI1;PFAS;CDC73;MED17;MRPL42;UBL3;SART3;ALDH2;METTL16;CTBS;PHACTR4;ARHGEF10
RP11;ALDH6A1;HNRNPK;PPP1R1B;CDK1;STRN;TTC22;SIX1;SMG7;HK2;MED17;MRPL42;ADAMTS5;TI
HM3;GPR27;MOCS3;PTPRM;SMG7;MED17;ING4;MRPL42;UBL3;SIPA1L1;ALDH2;C1QBP;TRPS1;SH3B
;TMTC3;SPATA2;DTX4;ZNF25;NPAT;ARHGAP20;APH1B;RIC8B;VTI1A;PCDHA4;C9ORF171;PCDHA3;SC
IP6K1;RBM12;SRGAP2;B3GALNT2;MCTS1;SRPK2;EIF2B2;ZBTB14;TIPARP;XRCC5;ZBTB16;NEK7;MRF
;BHLHB9;RHOBTB3;TMTC3;MTMR9;TRHDE;GIGYF2;RIC8B;ATXN7;ZMAT3;MLEC;C9ORF171;PCDHA3;
;DNAL1;LRP12;NRP2;BMPR2;TTC22;DUSP19;TNFAIP3;CTDSPL2;PPP1R9A;PITPNC1;ADAMTS5;KIAA1
11B;FAM78A;FAM46C;ZBTB16;B3GAT2;SUSD1;ATP2B2;TBX5;GDF6;SORL1;SYT7;STXBP5L;REEP1;CA
S1B;ARL5B;SIPA1L1;SH3PXD2A;NKRF;TRIM3;FAM73A;TMEM108;SCN5A;CTBS;CCDC112;ARHGEF10;
F74;OCLN;PPP1R1B;ID4;GABRB1;BTG1;ONECUT2;DIRAS2;PDE3B;JPH1;ADAMTS5;SDR16C5;NHSL2
1;MED17;CKS1B;SART3;SERP1;NKRF;KIF1B;SCN5A;CNPPD1;APPL1;DGCR2;ARHGEF10;NAA30;WDR
K5;TNFAIP8;ADK;PPWD1;ETFA;CTDSPL2;PPP1R9A;DTWD1;SERP1;TRIM3;EMB;APPL1;UBL7;UTP15;N
9A;BMPR1A;NRP2;DYRK3;DIRAS2;PITPNC1;ALAD;DTWD1;MED14;ARL5B;KIF1B;ZNF367;UBL7;GPR13
LSM5;BTBD9;RCAN1;GLUD2;DIAPH2;SP1;SESTD1;DNAL1;GPR27;NRP2;BMPR2;SNAP23;TNFAIP3;CTI
5;EGR4;ST13;ST8SIA3;BFAR;SMARCA1;GLUD2;HNRNPK;ID4;HNRNPC;GABRB1;PHF20;BMI1;RBM3;AI
N2;PPP1R1B;PLEKHM3;LRP12;RAD9A;BMPR1A;HRK;RCSD1;ADAMTS5;SART3;SH3PXD2A;NKRF;RBM

RHGAP20;PCDHA1;RNF217;PCDHA5;PCDHA4;PCDHA3;HIVEP2;SCN3B;RIMKLA;PCDHA9;PCDHA8;PC
2;SCN3B;RIMKLA;PCDHA8;MARK1;TMEM86A;CPSF7;SRD5A1;RASSF8;SREK1IP1;HELZ;BFAR;GDF6;
FNTB;DIRAS2;DUSP19;CTDSPL2;PITPNC1;PHF8;SPRED1;KIF1B;GPR37;PAX5;IL17RD;ZFX;CDYL2;PTF

MARCH7;GDF6;ESR1;ABHD15;PARP11;ALDH6A1;PPP1R1B;ID4;CDK1;NABP1;LSM14B;HRK;DYRK3;TN
IOCS3;SIX1;BMI1;MED17;MRPL42;SH3PXD2A;ALDH2;RSPO2;METTL16;PHACTR4;MAPK1IP1L;TSFM;T
1;PPP1R1B;UBE2K;LRP12;CC2D1B;HRK;KCNC2;SNAP23;ADK;PPWD1;CTDSPL2;PFAS;CDC73;SIPA1L
3CEL;BHLHB9;IGSF3;PDE1B;DLX6;SHOX2;SLC1A4;TRHDE;ZMPSTE24;ARHGAP20;APH1B;EFNB3;PCI
7;TSHR;STXBP5L;REEP1;OCLN;EFNA3;CAMK4;PPP1R1B;LHX4;LSM14B
L5B;RBM7;UBL7;TMED8;NSUN3;C3ORF62;GAB1;PROX1;DIEXF;RAP2C;SNPH;CD47;SLC25A12;RAPG
DC73;RBM3;SDR16C5;SART3;SERP1;C1QBP;FAM154B;FAM73A;KIF1B;ARL5A;DGCR2;TMED8;DNMT3
ITS2;JPH3;SART3;SERP1;MECOM;NKRF;SLC22A17;KIF1B;SCN5A;ARHGEF10;CHST7;GRID1;VPS13C;
3;TNFAIP8;BTG1;ADK;ING4;CAPZB;NKRF;MAN1A2;FAM154B;FAM73A;SOSTDC1;METTL16;TSFM;SGI
2D1B;NRP2;SH3KBP1;SMG7;JPH1;LITAF;PHF8;MED14;NKRF;CTBS;ZCCHC24;NCOA2;PCYT1B;GPR3
NC2;RSF1;RCSD1;C1ORF213;SMG7;JPH1;MYPN;NHSL2;SLC22A17;SH3BGRL2;CHST7;WDR37;TMED
3;RNF180;SHOX2;NPR3;HSPA4L;AGAP2;DTX1;APCDD1;DTX4;TRHDE;MED12L;FBLN5;FBXO40;NKAIN
DYRK3;BTG1;PHF20;CTDSPL2;RND3;MED17;ALAD;MRPL42;MED14;SART3;SERP1;NKRF;C1QBP;CEF
1;ID4;RAD9A;DYRK3;MED17;MRPL42;ADAMTS5;ARL5B;SH3PXD2A;TRPS1;FAM73A;TMEM108;SH3B
X7B;MOCS3;BMPR2;DIRAS2;ADK;RSF1;C1ORF213;JPH1;CDC73;ING4;XPO4;MB;TMEM108;METTL16;
DE;FBLN5;FBXO40;NKAIN1;ARHGAP20;EFNB3;TAC3;LONRF3;SCN3B;RIMKLA;PCDHA9;HOXC8;LYN;S
CDHA2;PPARGC1B;ABCA1;CADM3;ZBTB10;SLC4A10;PARVA;INHBA;SMARCA1;GRIN2B;PHOX2B;ABH
MI1;RND3;PITPNC1;MED17;IL18BP;RBM3;MED14;ALDH2;C1QBP;TMEM108;SH3BGRL2;WDR37;C3OR
NFAIP3;MYPN;PITPNC1;ALAD;DTWD1;KIAA1549;SH3PXD2A;RBM7;ARHGEF10;PLA2G12A;NSUN3;AH
9A;GPR27;DYRK3;MED17;ING4;MRPL42;ADAMTS5;ARL5B;TRPS1;FAM73A;TMEM108;SH3BGRL2;CCD
A;HRK;DYRK3;PFAS;MED17;ING4;MRPL42;ADAMTS5;SH3PXD2A;SIN3A;FAM73A;RSPO2;SH3BGRL2;
114A1;ARL3;CUL3;SHOX2;SPATA2;DTX4;FBLN5;ZMPSTE24;NPAT;BCL2L13;NKAIN1;RIC8B;LONRF3;SC
A4;ZDHHC21;ZBTB44;ZNF25;LRP8;C17ORF96;FAM117B;DRP2;ARHGAP20;APH1B;ZMAT3;MAPK1;BCL
2;DIRAS2;PTPRM;C1ORF213;PPP1R9A;JPH1;PHF8;KIAA1549;SERP1;MECOM;SH3BGRL2;SCN5A;C3
;MFSD5;EMB;METTL16;RBM7;UBL7;PHACTR4;USP47;LRRC40;HAUS3;ANO6;SMC1A;IL17RD;RAP2C;
NFAIP8;TTC22;MYPN;DTWD1;SDR16C5;MED14;ARL5B;MFSD5;TRIM3;RBM7;UTP15;GPR37;CHST7;TM
TECPR2;DNAL1;ITM2C;GABRB1;SNAP23;DIRAS2;SDR16C5;NHSL2;ALDH2;MFSD5;TMEM108;SH3BGF
UBP1;DAND5;ADK;LITAF;CDC73;SERP1;FAM154B;TMEM108;SOSTDC1;UBXN7;METTL16;PRKG1;APF
5;SMAD7;HNRNPK;SERINC3;TECPR2;CREBZF;DOCK5;BTG1;ADK;FMN1;MED17;SART3;SERP1;MEC
OCK5;ONECUT2;TTC22;PHF20;DIRAS2;SLC7A14;PTPRM;TNFAIP3;FMN1;ADAMTS5;SDR16C5;GRM5;
BTB16;ST8SIA3;SLC4A10;GDF6;SYT7;PHOX2B;REEP1;PPP1R1B;HRK;GPR27;GABRB1;KCNC2;TTC22
PN;CDC73;ING4;RBM3;SPRED1;SERP1;CAPZB;DDI2;FAM73A;ZNF800;CEP170;GAB1;SENP2;CDYL2;F

R1B;CDK1;CALM1;LSM14B;RAD9A;MOCS3;BMI1;PFAS;CDC73;MED17;PHF8;MRPL42;SIPA1L1;METTL
S5;SERP1;PSD4;SOSTDC1;ARL5A;GPRASP2;GPR135;HS3ST3B1;MAPK1IP1L;GPR37;WDR37;GRID1;\
;PHF8;MRPL42;MED14;SART3;SIPA1L1;XPO4;SH3BGRL2;KIF1B;METTL16;CTBS;PHACTR4;MAPK1IP1
;DAND5;MOCS3;BMI1;MED17;PHF8;ALAD;MRPL42;ALDH2;SH3BGRL2;METTL16;CTBS;PHACTR4;USP

IAN1A2;C1QBP;UBXN7;DGCR2;UTP15;KLF10;TTC33;LRRC40;HAUS3;SLC39A13;TERF2IP;ERGIC2;PAI
'3A;SH3BGRL2;KIF1B;CTBS;CCDC112;VKORC1;UTP15;ZCCHC24;KLF10;MAPK1IP1L;ARHGEF10;NSUI
'D1;ARL5B;SART3;DDI2;FAM154B;SH3BGRL2;RBM7;CTBS;UBL7;UTP15;USP47;GPR37;TTC33;VPS13C
ART3;MECOM;SLC22A17;SCN5A;ARL5A;APPL1;DGCR2;ARHGEF10;GRID1;C6ORF62;SLC39A13;PAX5
KHM3;RAD9A;ADK;C1ORF213;KIAA1549;ALDH2;NKRF;KIF1B;RBM7;PLA2G12A;WDR37;C3ORF62;DNM
DSPL2;JPH1;MYPN;CDC73;ING4;RBM3;FAM73A;SH3BGRL2;ZCCHC24;PLA2G12A;CHST7;TMED8;C3O
PRED1;SERP1;NKRF;MAN1A2;RBM7;UNKL;KLF10;GPR37;CHST7;TMED8;NSUN3;GAB1;SENP2;FCHSI
;MLEC;C9ORF171;PPARGC1B;B3GALNT2;TMEM86A;CCZ1B;EGR4;MAGEE1;ST13;LNPEP;KLHL23;SM
;CADM3;EGR4;FAM78A;ZBTB10;KLHL23;GDF6;GRIN2B;PHOX2B;OCLN;RCAN2;PPP1R1B;LHX6;LHX4
H7;CNOT6;HNRNPK;PPP1R1B;STRN;PLEKHM3;MOCS3;PRR3;ADK;TNFAIP3;ETFA;MED17;ARL5B;SAR
F8;MED14;XPO4;NKRF;CCDC112;VKORC1;TMED8;ITPK1;TFEB;FAM101B;KAT2B;RAP2A;MAPRE3;SMT
TA6;APCDD1;TRHDE;FBXO40;NKAIN1;EFNB3;CHAD;TAC3;LONRF3;SCN3B;RIMKLA;MARK1;LYN;TFAI
AS2;ADK;TNFAIP3;SIX1;RCSD1;PITPNC1;ELAVL2;CKS1B;SH3PXD2A;NKRF;FAM154B;NSUN3;DNMT3A
I3;ALDH2;SLC22A17;RBM7;UBL7;ARHGEF10;NSUN3;GAB1;PAX5;PROX1;ZFX;DIEXF;RAP2A;SNPH;NA
NKL;PHACTR4;MAPK1IP1L;ARHGEF10;TTC33;NSUN3;ITPK1;ZFP91;IL17RD;ZFX;RAP2C;FCHSD2;ERG
E1;ZBTB10;PARVA;NSG1;L1CAM;SYT7;REEP1;OCLN;SESTD1;RCAN2;LHX4;PLEKHM3;CC2D1B;BMPI
;FAM117B;ZNF629;TMEM203;MAT2A;ZMAT3;UBN2;PCDHA2;MPZL3;PCDHA8;JAM2;ZNF462;CCZ1B;SC
8A;SORT1;ZBTB10;FBXL17;LNPEP;PLEKHA3;KLHL23;TMEM64;RCAN1;ITCH;ARHGAP31;FRAS1;KLHL
DE;COL19A1;ZMPSTE24;APH1B;ZNF629;TMEM203;MAT2A;ERI1;RBBP5;TDG;UBN2;TMEM68;VTI1A;SL
10;LRP6;C21ORF119;BCL2L13;NXF1;RXRA;KIAA0355;RBBP5;UBN2;TMEM68;VTI1A;FAM63B;C10ORF
H1;APH1B;RIC8B;TMEM203;RNF217;CARNS1;LONRF3;HIVEP2;CLSPN;RBM12;MPZL3;SRSF10;N4BP2
G1;PALM2;TMTC3;UQCR10;ZMPSTE24;NKAIN1;ZNRF3;APH1B;RIC8B;TMEM203;RNF217;VTI1A;C10OI
I1;STAU1;PALM2;TYRP1;DCUN1D1;DLST;SPATA2;PPM1K;C1ORF52;LRP6;NCKIPSD;NXF1;CYB5R4;DFI
;ZMPSTE24;TRAK2;NCKIPSD;C21ORF119;APH1B;RIC8B;ZNF629;TMEM203;RBBP5;TDG;UBN2;TMEM6
;GPRIN3;NRG3;IL1RAPL1;NOS1AP;ELMO1;FAM84A;SCG3;COL6A5;MAPRE2;SLC24A2;SHC2;PPM1L;R
;DCUN1D5;YTHDC1;SEMA3A;AGAP2;TMTC3;TUBD1;ZNF25;MED12L;ZMPSTE24;PPP2CA;BCL2L13;N
;NPR3;RHOBTB3;SPATA2;CREBL2;TRHDE;MTDH;BCL2L13;ZNF706;ZMAT3;VTI1A;MCMBP;C9ORF171;
MR9;SPATA2;DTX4;UBE2J1;NPAT;APH1B;G3BP1;VTI1A;PLXNA2;C9ORF171;CLSPN;SCN3B;MYH10;RA
APA;DLX1;NAPB;PTGFR;HPGD;YTHDC1;PDE1B;HSPA4L;FUT11;MTMR9;TUBD1;CREBL2;PPM1E;PUR
;SHC4;ZNF275;HDAC5;PSMD11;UBA6;ATL2;ZDHHC21;GRAMD4;LRP6;NCKIPSD;NEURL1B;NPAT;BCL2
RF52;TRHDE;PPP2CA;BCL2L13;NXF1;CYB5R4;RIC8B;MAT2A;TDG;UBN2;VTI1A;PLXNA2;EIF4EBP2;LI
R10;TRAK2;BCL2L13;PURA;NXF1;RNF214;ZNRF2;PGRMC1;CARNS1;NRIP1;FAM63B;PTK2B;B3GALNT
;ABHD2;SHOX2;CREBL2;NDNF;NEURL1B;APH1B;EFNB3;SERTAD2;ZMAT3;E2F1;PLXNA2;SLIT2;RIMK
;KIAA0355;TDG;UBN2;VTI1A;IGF2BP1;EIF4EBP2;MAPK1;PCBD2;LRIG2;RIMKLA;BCL2L2-PABPN1;MP
IA7;PCDHA6;EGR4;FZD5;BCL11B;FAM78A;ZBTB16;NSG1;GRIN2B;ABHD15;ARHGAP31;RCAN2;ID4;LH
NK4;ZNF597;SHC2;PPM1L;IGSF3;PDE1B;DLX6;GATA6;NDNF;PDHB;UQCR10;TTL;TRHDE;C21ORF119;
1;UQCR10;PAPSS2;TRAK2;C21ORF119;GNA13;NPAT;NXF1;APH1B;RIC8B;EFNB3;TMEM203;TDG;TME
;UBA6;PLAG1;RHOBTB3;XIAP;GSKIP;CREBL2;ZDHHC21;HIF1A;NOP9;ZMPSTE24;TRAK2;PURA;OGFR
;TMTC3;PRICKLE1;UQCR10;DTX4;FBLN5;NEURL1B;BCL2L13;RNF214;ZNF706;TMEM203;RBBP5;TDG;
C3;CGGBP1;SLC29A3;KANK4;AMER1;USP15;UBA6;PDE1B;MTMR9;CREBL2;NPAT;GBX2;TMEM68;VTI
CMBP;GSE1;RAI2;MARK1;MCTS1;SRPK2;TMEM86A;ZBTB14;MAGEE1;SMURF2;MRPL27;SREK1IP1;HI
A3;KANK4;PPIC;SHC4;AMER1;USP15;HPGD;YTHDC1;PDE1B;HSPA4L;DTX1;DTX4;PPM1E;MED12L;GB
AMD4;FAM117B;NEURL1B;NPAT;NXF1;APH1B;RIC8B;EFNB3;CHAD;PRDM16;PCDHA4;PCBD2;SCN3B;
33;BHLHB9;NECAB3;PLAG1;SEMA3A;TMTC3;CREBL2;UQCR10;C21ORF119;DRP2;NPAT;BCL2L13;OGI
IEM106A;SLC29A3;PPIC;NAPA;AMER1;NAPB;TSHZ3;YTHDC1;UBA6;ZDHHC20;ARL3;FUT11;DTX1;DT
NF629;TMEM203;RBBP5;TMEM68;C10ORF2;APBB2;PCBD2;MPZL3;SMNDC1;MYH10;S100PBP;EDEM3
TA2;UQCR10;ADD3;C1ORF52;NXF1;CYB5R4;SNX1;GBX2;IGF2BP1;C10ORF2;LRIG2;ZNF148;ZC3H14;
CA;NPAT;PURA;ZNRF2;TMEM65;SERTAD2;RBBP5;MGAT5;ATXN1L;NRIP1;VTI1A;USP1;VPS54;LRIG2;

:BTB44;UQCR10;FAM117B;NPAT;PURA;BCLAF1;APH1B;TMEM68;VTI1A;C10ORF2;VPS54;APBB2;PCBD
3TB16;MRPL27;L1CAM;SYT7;REEP1;OCLN;ARHGAP31;SESTD1;PPP1R1B;ID4;LHX4;TECPR2;BMPR1A
D1;MTMR9;TUBD1;CREBL2;HIF1A;FOXO1;ADD2;NCKIPSD;PPP2CA;BCL2L13;RNF214;RAP1A;ZNF629;
3;PAPSS2;UBE2J1;TRAK2;DRP2;NPAT;OGFRL1;TMEM203;SERTAD2;RBBP5;NT5DC3;VTI1A;PLXNA2;S
1;CBFA2T2;ZFX;GNL1;CDYL2;FAM101B;TMEM33;MAPRE3;ERGIC2;MAPRE2;TMEM106A;CHST3;KANK

B;MOCS3;PRR3;CELF3;PPWD1;TNFAIP3;CTDSPL2;LITAF;MED14;ARL5B;XPO4;ALDH2;TRIM3;PGM3;A
DC1;FUT11;MTMR9;ZBTB44;C1ORF52;ZNF25;ZMPSTE24;C21ORF119;AP3M2;BCL2L13;SNX1;DFNB59;
2;AGAP2;TUBD1;NDNF;PRICKLE1;ZNF25;NPAT;SNX1;ARHGAP20;EFNB3;ZNF629;ZNF706;S1PR3;SCN
JZP1;SEMA3A;ATL2;PPM1H;FAM129A;DTX4;C17ORF96;FOXO1;MED12L;NEURL1B;ZNRF2;SNX1;MGA
UB1;ADAM12;ARMCX6;ELMO1;ST6GALNAC3;ERGIC2;SLC29A3;KANK4;PPIC;NAPA;NAPB;PTGFR;HPG
DC1;UBA6;PDE1B;PRUNE2;FUT11;MTMR9;CREBL2;ZDHHC21;PURA;EFS;GBX2;RNF217;CHAD;PCDHA
178A;ST13;ZBTB16;GFPT1;MRPL27;SREK1IP1;LNPEP;HOOK3;NSG1;L1CAM;BTBD9;FBXO30;CNOT6;A
SNX8;SNX6;ABCA1;MCTS1;SYT5;TMEM86A;MGAM;EGR4;BCL11B;KLHL23;C12ORF74;REEP1;DIAPH2
1;PURA;OGFRL1;NXF1;ZNRF2;RBBP5;TMEM68;NRIP1;MAPK1;LRIG2;RPP14;MBNL3;MPZL3;EDEM3;M
3TB44;APCDD1;PAPSS2;FBLN5;NXF1;RNF214;ARHGAP20;TMEM65;TMEM203;PABPN1;ZMAT3;IGF2BF
M114A1;PDE1B;PALM2;PRUNE2;GATA6;PRICKLE1;TRHDE;PAPSS2;DRP2;NEURL1B;OGFRL1;APH1B;
PHLPP2;FYTTD1;SHOX2;PPM1H;SLC1A4;UQCR10;NKAIN1;PGRMC1;GBX2;CARNS1;CHAD;N4BP2;PL
NK2;SET;TSHZ3;PDE1B;NPR3;PRUNE2;GLIS3;PPM1H;DLST;SPATA2;PPM1K;UQCR10;PURA;EFS;RIC8
L2L13;PURA;RNF214;TMEM203;TMEM68;NT5DC3;VTI1A;PLXNA2;HAS2;MLEC;RPP14;XRCC5;PTPN11

XO40;APH1B;RIC8B;TDG;UBN2;NSD1;TMEM68;PRDM16;VTI1A;PLXNA2;PTK2B;VPS54;HIVEP2;N4BP2;
203;MAT2A;ERI1;MIER3;RBBP5;ATXN1L;NT5DC3;HAS2;C10ORF2;MCMBP;LRIG2;RPP14;SMNDC1;SY
NPAT;BCL2L13;APH1B;EFS;RIC8B;TMEM68;VTI1A;C9ORF171;PCBD2;SLIT2;MPZL3;N4BP2;TRIM44;TM
DN18;MAPRE3;SMTNL2;ST6GALNAC3;ADCYAP1R1;IGSF3;ATL2;GATA6;FAM129A;NRXN3;PPM1E;TRH
1R1;BHLHB9;UBA6;DLX6;SHOX2;AGAP2;SPATA2;TRAK2;NPAT;APH1B;TMEM203;GPC5;PCDHA3;APBB
.2;UQCR10;C17ORF96;NPAT;EFS;RIC8B;TNKS2;TDG;PRDM16;IGF2BP1;PCDHA4;PCDHA3;PCBD2;SCN
;TRAK2;NEURL1B;APH1B;RIC8B;ZNF706;TMEM203;VTI1A;C10ORF2;C9ORF171;PCBD2;SCN3B;RIMKL
;GABRB1;CELF3;DIRAS2;SLC7A14;RCSD1;PPP1R9A;ADAMTS2;JPH3;SLC22A17;TMEM108;SCN5A;GF
.2;UQCR10;PPM1E;C21ORF119;NPAT;BCL2L13;ZDHHC18;RIC8B;ZNF629;TDG;VTI1A;C9ORF171;LRIG2
2J1;TRAK2;PURA;CYB5R4;RNF214;TMEM203;RBBP5;S1PR1;CLSPN;EMC7;TRIM44;PLXNA3;ABCA2;SE
SHOX2;PRUNE2;SPATA2;SLC1A4;ZDHHC21;PAPSS2;ZMPSTE24;GJC1;DRP2;DFNB59;TMEM203;NSD
KLE1;PDHB;UQCR10;C17ORF96;NPAT;NT5DC3;VTI1A;TAC3;APBB2;LRIG2;SCN3B;MPZL3;SNX8;SMND
SPA4L;DCUN1D1;MTMR9;DTX1;CREBL2;ZBTB44;LRP8;TRAK2;AP5M1;TMEM203;GPC1;G3BP1;MPZL3;

9;RBBP5;TDG;TMEM68;VTI1A;PTK2B;PCBD2;LRIG2;IP6K1;BCL2L2-PABPN1;N4BP2;TRIM44;S100PBP;
:3;BHLHB9;NECAB3;HPGD;YTHDC1;PALM2;PPM1E;GRAMD4;COL19A1;GJC1;CTIF;ZNRF2;ZDHHC18;A
RA2B;UBN2;MLEC;GLTSCR1L;IP6K1;SRSF10;ZC3H14;GALNT7;AAED1;CPSF7;SEPT12;RANBP3;CBX5;
RF96;EFS;RIC8B;VTI1A;GPC5;PCDHA3;LONRF3;PCBD2;SCN3B;RIMKLA;MPZL3;PCDHA7;MARK1;CPS
119;NEURL1B;NPAT;ZNRF3;APH1B;RXRA;RIC8B;IGF2BP1;PLXNA2;HAS2;VPS54;MLEC;RIMKLA;MPZL3
O40;NKAIN1;ARHGAP20;MGAT5;TRA2B;S1PR1;TAC3;LONRF3;SCN3B;RPP14;SNX8;RELT;JAM2;RAI2;
X1;NAPB;TBC1D19;YTHDC1;ZDHHC20;MTMR9;MAT2B;GJC1;CTIF;CYB5R4;ZNRF2;ZDHHC18;PABPN1
FNB59;HSPH1;ZNF629;MAT2A;RBBP5;ZMAT3;TDG;UBN2;G3BP1;VTI1A;MCMBP;MLEC;S1PR3;CLSPN;
IC4;PALM2;TUBD1;PAPSS2;ZMPSTE24;GJC1;DRP2;CHAD;VTI1A;IGF2BP1;C10ORF2;VPS54;GPC5;PC
;GFPT1;MRPL27;NBEAL1;SREK1IP1;NSG1;L1CAM;FBXO30;SYT7;OCLN;ZFP62;SESTD1;RCAN2;LSM1
N;RBM12;SNX8;PCDHA8;RAB11FIP5;PLXNA3;SRPK2;CPSF7;ZBTB14;XRCC5;ST13;MRPL27;NBEAL1;
.2;SLC1A4;ZBTB44;UQCR10;ZBTB4;LRP6;ZMPSTE24;C21ORF119;GJC1;DFNB59;RIC8B;RBBP5;TDG;N
PALM2;FAM129A;XIAP;ZDHHC21;ADD3;COL19A1;GJC1;DRP2;TRA2B;NSD1;CHAD;GPC5;RBM12;SCN
1203;TNKS2;TDG;VTI1A;C10ORF2;PCDHA4;GPC5;SCN3B;MPZL3;PCDHA7;PCDHA6;SEPT10;TMEM86
LRP6;C21ORF119;NXF1;HSPH1;RIC8B;TMEM65;UBN2;FAM63B;C10ORF2;MCMBP;LRIG2;BCL2L2-PAB
;BCL2L13;FBXO40;APH1B;EFS;TMEM203;CARNS1;SLIT2;RIMKLA;EDEM3;TRIM41;ST13;SUSD1;LGI2;K
;TMEM203;SERTAD2;VPS54;MPZL3;N4BP2;TRIM44;B3GALNT2;FZD3;RABGAP1L;CPSF7;CCZ1B;FZD5
IPSTE24;NPAT;PURA;EFNB3;TMEM203;TNKS2;VTI1A;C10ORF2;C9ORF171;APBB2;PCBD2;SCN3B;RIM

'1;ZNRF2;RIC8B;ZNF706;TNKS2;TDG;TMEM68;VTI1A;PLXNA2;C10ORF2;MCMBP;VPS54;PCBD2;IP6K1
9A;UQCR10;C1ORF52;HIF1A;GIGYF2;GNA13;PABPN1;TRA2B;VTI1A;IGF2BP1;C9ORF171;ATOH1;CA12
;GATA6;DTX1;PPM1E;BCL2L13;FBXO40;ARHGAP20;EFNB3;GBX2;CARNS1;CHAD;S1PR1;GPC5;JAM2;
FAM129A;DTX1;NDNF;PPM1E;C17ORF96;TRHDE;COL19A1;PURA;APH1B;CARNS1;PRDM16;SLC17A6
1;PRUNE2;GATA6;RHOBTB3;C17ORF96;LRP6;ADD2;GJC1;EFS;PCDHA1;ERBB4;CHAD;SLC17A6;PCDI
3;TMEM68;VTI1A;PLXNA2;FAM63B;C10ORF2;MCMBP;VPS54;PCBD2;N4BP2;SMNDC1;TRIM44;CPSF7;E
NF217;S1PR1;SCN3B;RIMKLA;EIF4E;RAI2;MARK1;FZD3;SEPT12;EGR4;ST8SIA3;KLHL23;PARVA;NSG1
'RUNE2;TMTC3;SPATA2;TUBD1;UQCR10;DTX4;PAPSS2;NEURL1B;APH1B;RIC8B;TMEM203;RBBP5;C1
I1A;C10ORF2;C9ORF171;MBNL3;SRSF10;SNX8;ZC3H14;S100PBP;ABCA2;PHC2;CCZ1B;CRBN;FAM78/
AT2B;COL19A1;PAPSS2;GJC1;CYB5R4;ARHGAP20;PABPN1;ZMAT3;NSD1;RNF217;LONRF3;SCN3B;MI
3;TMEM65;ZNF629;TMEM203;RBBP5;PLXNA2;S1PR1;C10ORF2;HIVEP2;IP6K1;MPZL3;TRIM44;S100PB
T11;NDNF;LRP8;JAKMIP2;TRAK2;C21ORF119;GJC1;NPAT;RIC8B;TNKS2;PABPN1;CHAD;PRDM16;IGF2
;ZMAT3;GPC1;RNF217;CARNS1;CHAD;SLC17A6;MLEC;RIMKLA;HOXC8;SLC25A24;LYN;SYT5;FZD3;BC
9A3;KANK4;PPIC;PTGFR;HPGD;YTHDC1;PDE1B;HSPA4L;MED12L;SNX1;GBX2;RNF217;PRDM16;PCDI
RDM16;IGF2BP1;C10ORF2;PCDHA4;VPS54;GPC5;PCDHA3;APBB2;LONRF3;PCBD2;HIVEP2;RIMKLA;M
3TB14;SRD5A1;NEK7;MRPL27;SREK1IP1;HELZ;NSG1;ESR1;PARP11;ALDH6A1;PPP1R1B;ID4;CDK1;RA

I1;PURA;RNF214;APH1B;RIC8B;TMEM65;TMEM203;ZMAT3;VTI1A;FAM63B;N4BP2;PCDHA8;TRIM44;RA
;PURA;NXF1;EFS;TMEM203;PABPN1;MGAT5;MCMBP;IP6K1;SLC25A24;SRPK2;PHC2;TRIM41;SRD5A1
21ORF119;SNX1;PABPN1;RNF217;N4BP2;SNX8;RELT;SYT5;IGBP1;TMEM86A;RABGAP1L;FAM46C;TXN
2;PRUNE2;ZBTB44;TRHDE;C21ORF119;ARHGAP20;GBX2;PCDHA1;PCDHA5;HAS2;PCDHA4;C9ORF17'
1B;EFS;ZNF629;ZNF706;VTI1A;MCMBP;PCDHA3;RIMKLA;BCL2L2-PABPN1;SRSF10;SMNDC1;SEPT10;
B3;CREBL2;UQCR10;PAPSS2;JAKMIP2;NEURL1B;SNX1;APH1B;RXRA;TMEM203;GPC1;SCN3B;EIF4E;I
21ORF119;CYB5R4;CARNS1;SLC17A6;GPC5;LRIG2;PLXNA3;EDEM3;SYT5;GABRA1;FZD3;CRBN;MRP
TI1A;E2F1;VPS54;LRIG2;IP6K1;MPZL3;N4BP2;B3GALNT2;ABCA2;RABGAP1L;CPSF7;CCZ1B;USP9X;G
9A1;CYTH3;GJC1;NEURL1B;CYB5R4;ZNRF2;DFNB59;ARHGAP20;NSD1;CHAD;SLIT2;BCL2L2-PABPN1
;CARNS1;HAS2;GPC5;LONRF3;S1PR3;FZD3;SEPT12;EGR4;TPBG;SUSD1;FBXL17;KLHL23;NSG1;S10
ATXN7;MIER3;MCMBP;RPP14;TRIM44;S100PBP;SLC25A24;PLXNA3;ACBD5;TIMMDC1;TIPARP;CSNK1
.4;PPM1E;TTL;COL19A1;LRP6;GJC1;ZNRF2;PABPN1;NSD1;LONRF3;EIF4E;MYH10;JAM2;ATOH1;CA12;
FBLN5;FBXO40;ARHGAP20;EFNB3;GBX2;CHAD;S1PR1;LONRF3;SCN3B;JAM2;LYN;SEPT12;BCL11B;F
4;RIC8B;ZNF629;ZMAT3;TDG;C10ORF2;SNX8;B3GALNT2;SEPT10;FZD3;ACBD5;CCZ1B;USP9X;FBXL1
I;PHC2;ACBD5;TIMMDC1;CBX5;TIPARP;SRD5A1;CSNK1A1;CNBP;NEK7;GFPT1;MRPL27;GATC;PLEKH
AKMIP2;DRP2;PCDHA1;RNF217;CHAD;S1PR1;SLC17A6;S1PR3;PPARGC1B;ATOH1;ABCA2;PHC2;SEP
)5A1;XRCC5;RASSF8;LNPEP;KLHL23;BFAR;MARCH7;PQLC1;JMY;LSM14B;UBE2K;RBM28;BTG1;SNAP
PN1;HAS2;MPZL3;SNX8;ATOH1;SLC25A24;EIF2B2;CBX5;TIPARP;NEK7;ST8SIA3;PARVA;ATP2B1;CDC
PCDHA3;SCN3B;MPZL3;PCDHA7;MARK1;ZNF462;CPSF7;EGR4;FZD5;BCL11B;FAM46C;GABRA3;LGI2;
FAM63B;PCBD2;N4BP2;ZNF462;TRIM41;TIMMDC1;CBX5;CCZ1B;FZD5;NFATC3;GATC;LNPEP;PARVA;A
HOBTB3;PAPSS2;GJC1;DRP2;BCL2L13;TMEM68;CARNS1;IGF2BP1;GPC5;MYH10;RAI2;MARK1;SEPT1
RBBP5;MGAT5;NT5DC3;C10ORF2;C9ORF171;BCL2L2-PABPN1;S100PBP;RRM1;SRD5A1;SS18L2;GTF
RAI2;ATOH1;SLC25A24;SNX6;ABCA1;SYT5;FAM78A;KCNIP2;LGI2;SMAD9;KLHL23;S100B;SYNJ2BP;GI
AD9A;CDS2;NRP2;CELF3;DUSP19;ETFA;IL18BP;ADAMTS5;DTWD1;SART3;NKRF;C1QBP;TRIM3;PSD3
IRL1B;NPAT;APH1B;SERTAD2;TMEM68;PLXNA2;PCBD2;RIMKLA;MPZL3;N4BP2;FZD3;FZD5;SPRYD7;G
I2;NPR3;NRXN3;SLC1A4;COL19A1;DRP2;EFNB3;ZMAT3;CHAD;SCN3B;TRIM44;EDEM3;ABCA2;MRPL2
C9ORF171;GPC5;SCN3B;MPZL3;MARK1;ABCA2;TMEM86A;FZD5;MAGEE1;FAM46C;TPBG;TRAPPC8;/
;VTI1A;IGF2BP1;RIMKLA;SMNDC1;MYH10;TRIM44;S100PBP;PLXNA3;CPSF7;TIMMDC1;CNBP;KLK13;S
\2B;NSD1;IGF2BP1;EIF4EBP2;APBB2;LONRF3;SCN3B;RPP14;MPZL3;EIF4E;SNX8;RAB11FIP5;CRBN;M
;TXNL1;DEK;PARP11;GLUD2;VAPA;TMEM56;ID2;RFWD3;REN;LRP12
CN3B;MYH10;ZC3H14;SYT5;ZNF462;TMEM86A;CCZ1B;FZD5;FAM46C;LNPEP;KLHL23;HELZ;HOOK3;S
C1;ZNF462;TMEM86A;CPSF7;EGR4;SRD5A1;KCNIP2;GABRA3;GATC;ATP2B2;GTF2H1;HELZ;SYT7;U2S
CDHA5;LONRF3;PCDHA2;PCDHA9;MPZL3;EIF4E;PCDHA8;TRIM44;ATOH1;PCDHA7;SLC25A24;TFAP2B
)5;C1QBP;KIF1B;ZNF367;CCDC50;UBL7;UTP15;TSFM;DFFA;NAA30;WDR37;TTC33;SMC1A;DYNLL2;PT
EIF4EBP2;MAPK1;GLTSCR1L;SRSF10;RRM1;TRIM41;TIMMDC1;CSNK1A1;CNBP;HELZ;KCNRG;MARCH
SNX1;APH1B;KIAA0355;LRIG2;N4BP2;SLC25A24;ACBD5;CPSF7;SPRYD7;GATC;PDAP1;MTSS1L;EPOR

RF2;MLEC;ZC3H14;SRPK2;ABCA2;CPSF7;FZD5;FAM78A;SUSD1;MRPL27;FOXJ2;BFAR;SYT7;ALDH6A
3;TDG;VTI1A;MPZL3;SNX8;B3GALNT2;FZD3;RABGAP1L;CCZ1B;FZD5;KCNIP2;SPRYD7;GATC;PDAP1;
6;SCN3B;EIF4E;SYT5;GABRA1;SUSD1;RASSF8;KLHL23;KCNRG;INHBA;L1CAM;S100B;ASPRV1;SORB
LM2;SHOX2;PRUNE2;TUBD1;NEURL1B;NXF1;ZNRF3;ERI1;CARNS1;FAM63B;MCMBP;LRIG2;CPSF7;CF
HM3;RBM28;BTG1;PRR3;PDE3B;PPWD1;BMI1;SMG7;AFF4;CDC73;AFF1;HK2;ARL5B;SART3;RBM7;UN
IA1;CHAD;TAC3;RBM12;RIMKLA;LYN;SYT5;FZD5;GFPT1;LNPEP;PARVA;BTBD9;RCAN1;NCEH1;CAPRI
SMAD9;L1CAM;OCLN;ARHGAP31;NCEH1;NRP2;GABRB1;ONECUT2;JPH1;LITAF;NHSL2;SH3PXD2A;AL
;PCDHA3;IP6K1;HOXC8;S100PBP;LYN;EDEM3;GABRA1;CPSF7;NEK7;LAPTM5;PDAP1;KLHL23;INHBA;
RBM12;EIF4E;PPARGC1B;PRRX1;FBXL17;LSM5;SYT7;ADAT2;ALDH6A1;FAM167A;PQLC1;SESTD1;RC
DC1;TIPARP;SRD5A1;CSNK1A1;GFPT1;MRPL27;PCDHB15;SREK1IP1;PLEKHA3;GTF2H1;BFAR;ATP2B
MURF2;MRPL27;HELZ;L1CAM;LSM5;FBXO30;AZIN1;PUM2;PARP11;CNOT6;HNRNPK;PQLC1;SP1;CDK1
3B;TIPARP;SORT1;NEK7;FOXJ2;NFATC3;PLEKHA3;PARVA;S100B;BTBD9;PUM2;ZNF70;NCEH1;NFIA;PI
CBD5;SEPT12;TIMMDC1;TIPARP;SRD5A1;SORT1;CSNK1A1;PLEKHA3;BFAR;S100B;SOD2;SULF2;TPC
IK;PAPSS2;RXRA;EFS;ATXN7;TMEM248;IGF2BP1;S1PR1;GLTSCR1L;MPZL3;EIF4E;PLXNA3;ABCA1;SE

T5DC3;GPC5;MPZL3;EIF4E;PLXNA3;MAP3K2;FZD3;SMURF2;MRPL27;TRAPPC8;SOD2;ASPRV1;SORB
ARHGAP20;APH1B;ATXN7;MCMBP;SCN3B;RIMKLA;MYH10;RAB11FIP5;PLXNA3;ABCA1;ABCA2;CBX6;
128;PRR3;PPWD1;RCSD1;BMI1;SMG7;AFF4;AFF1;HK2;ARL5B;SART3;SIPA1L1;FAM154B;CEP170;RBM

PZL3;EIF4E;SNX8;ABCA2;FZD3;FAM46C;SYNJ2BP;EPOR;MAPK10;REEP1;PARP11;DIAPH2;FAM167A;
AT2A;MGAT5;MCMBP;LONRF3;BCL2L2-PABPN1;SYT5;RRM1;ACBD5;TRIM41;TIMMDC1;FZD5;TIPARP;S
DS2;BMPR1A;COX7B;USP37;ANKRD33B;CELF1;PHF20;CALCOCO2;KIAA1671;PPWD1;PTPRM;SMG7;F
A;FZD5;SREK1IP1;ESR1;SULF2;PARP11;MYO1C;PDP2;KIAA0101;APC;RCAN2;FUBP1;LHX6;RAB3GAP2
A1;FZD3;SEPT12;XRCC5;EPT1;PLEKHA3;KLHL23;KCNRG;S100B;ESR1;TSHR;ABHD15;HEYL;KIF26B;R
CBX5;CNBP;SS18L2;MRPL27;GATC;SREK1IP1;KCNRG;ATP2B1;MARCH7;SOD2;PUM2;SMAD7;HNRNP

L3;SNX8;EDEM3;TIMMDC1;ST13;TPBG;GATC;PDAP1;SREK1IP1;GTF2H1;KCNRG;SOD2;BTBD9;STXBI
APB;HDAC5;ADCYAP1R1;IGSF3;SHOX2;ATL2;HSPA4L;ADD3;PPM1E;ZBTB4;TTL;FOXO1;PAPSS2;FBLN
DR37;TTC33;NSUN3;MSL2;HAUS3;C6ORF62;SMC1A;DIEXF;ENAH;DDX19B;CREB1;SNPH;TET3;NAA38
4;EDEM1;ZBTB16;RASSF8;SMAD9;TRAPPC8;PARVA;GDF6;SELE;SYT7;PHOX2B;REEP1;RCAN1;RCAN
2;SNX8;S100PBP;ACBD5;EIF2B2;FZD5;SPRYD7;NBEAL1;GTF2H1;GTF2H5;ADAT2;ABHD15;ALDH6A1;C
BPN1;SLC25A24;ACBD5;TIMMDC1;TIPARP;CSNK1A1;NEK7;GFPT1;ST8SIA3;PLEKHA3;GTF2H1;PARVA
CARNS1;SLC17A6;SCN3B;TRIM44;ATOH1;PRRX1;KCNIP2;TPBG;B3GAT2;NEK7;SUSD1;S100B;DPY19I
CBD2;GLTSCR1L;SRD5A1;KCNIP2;CNBP;HELZ;KCNRG;GATAD2B;SMAD7;CDKN2AIPNL;OCIAD1;RLIM
9;LSM14B;LRP12;RBM28;BTG1;ANKRD33B;PRR3;SNAP23;DIRAS2;BMI1;SMG7;AFF4;AFF1;CKS1B;SA
CNIP2;CNBP;LAPTM5;GATC;PDAP1;SORL1;GATAD2B;CDKN2AIPNL;OCIAD1;LSM14B;SERINC3
NX8;PCDHA8;TRIM44;PCDHA7;CA12;ZNF462;GABRA1;CRBN;FAM46C;GABRA3;GRIN2B;RAB11A;RNF1
OXO1;ADD2;GJC1;NEURL1B;FBXO40;APH1B;RXRA;EFS;RIC8B;MBNL3;MPZL3;SNX8;MARK1;TMEM86
;EFS;GBX2;PCDHA1;NSD1;PCDHA5;LONRF3;PCDHA9;MBNL3;PCDHA8;TRIM44;PCDHA7;PCDHA6;SE
INHBA;SULF2;VAPA;VAPB;SP4;KLHL7;OSTC;CAPZA1;OCIAD1;RLIM;RAB3GAP2;TARDBP;LSM14B;LN
S1L;GTF2H1;PARVA;BFAR;KCNRG;SMAD7;VAPB;OSTC;SERBP1;RPA4;SPRY3;RAB3GAP2;SPRY1;RA
GABRA1;CADM3;CBX6;EIF2B2;ST13;PCDHB15;SPRY3;REN;TNRC6B

ATC;MARCH7;GATAD2B;SMAD7;CDKN2AIPNL;PITHD1;ETNK1;OCIAD1;TRIP12;SERINC3;NFE2L1;RAD
RA3;EPT1;PARVA;DEK;GRIN2B;HEYL;DIAPH2;HNRNPK;SERBP1;RPA4;RLIM;LHX4;LSM14B
RV1;ESR1;TSHR;ADAT2;ABHD15;BFSP2;ARHGAP31;DPY19L2;PQLC1;CDK1;CTNNB1;YPEL2;ITM2C
;PCDHA4;PCDHA3;PCDHA2;SNX8;PCDHA8;TRIM44;ATOH1;PCDHA7;SLC25A24;PCDHA6;EDEM3;CA1
GAP31;ITM2C;TENM3;ONECUT2;KCNC2;DIRAS2;SLC7A14;PTPRM;JPH1;PHF8;RBM3;ADAMTS5;MED
;CADM3;FZD3;XRCC5;ZBTB16;SLC4A10;RASSF8;SMAD9;L1CAM;GRIN2B;ESR1;REEP1;PARP11;BFSF
S54;GLTSCR1L;SCN3B;EIF4E;TRIM44;PCDHA7;MARK1;LYN;SEPT12;FAM78A;B3GAT2;KLHL23;ATP2B2
L2-PABPN1;RPP14;SRSF10;SMNDC1;TRIM44;IGBP1;CPSF7;TRIM41;CBX5;FZD5;FAM78A;CSNK1A1;CI

IA8;TRIM44;ATOH1;PCDHA7;PCDHA6;PLXNA3;CRBN;MAGEE1;GABRA3;PARVA;CDC42BPA;RNF148;SI
IRG;MARCH7;GATAD2B;HNRNPK;VAPB;FUBP1;CAPRIN1;SERBP1;TCEA3;TARDBP;LSM14B
ATP2B2;SORBS3;LETM1;EFNA3;PDP2;SP4;PPP1R1B;PDCD4;CD244;DAND5;SLC28A3;ITM2C;CC2D1B
1IP1;ATP2B1;L1CAM;SOD2;PUM2;CDKN2AIPNL;ID2;CAPRIN1;EIF3J;RPA4;CTNNB1;HNRNPC;TARDBP
09;ID2;OSTC;RPA4;JMY;RAB3GAP2;TECPR2;RAD9A;BMPR1A
;NBEAL1;KLHL23;SOD2;TPCN1;PARP11;C7ORF60;ZNF70;GPAM;ZFP62;MXD1;DNAL1;PLEKHM3;RAD9
AD;SNX6;ABCA2;CPSF7;MAGEE1;SRD5A1;XRCC5;GFPT1;HELZ;BFAR;MARCH7;BTBD9;ABHD15;ZZZ3
GAP31;PLEKHM3;RAD9A;ITM2C;CC2D1B;HRK;ALAS2;GABRB1;BMPR2;KCNC2;SH3KBP1;DIRAS2;DUS
FOXO1;ZMPSTE24;NCKIPSD;AP3M2;NXF1;GBX2;TMEM203;PABPN1;ATXN7;MIER3;MGAT5;TRA2B;MC
PCN1;ALDH6A1;ZNF70;HNRNPK;FRAS1;GPAM;KIAA0101;FUBP1;DNAL1;PLEKHM3;RAD9A
CA2;TMEM86A;TSHR;DIAPH2;PDP2;KIAA0101;APC;RCAN2;OCIAD1;NUPL1
IQCR10;C1ORF52;ZNF25;FAM117B;NCKIPSD;RNF214;APH1B;TMEM203;HAS2;C10ORF2;VPS54;LRIG2
I5;AP3M2;FBXO40;APH1B;ZMAT3;NSD1;HAS2;MYH10;ATOH1;SYT5;TIPARP;SORT1;KCNIP2;B3GAT2;S
AP3K2;RRM1;ACBD5;TIMMDC1;SRD5A1;CSNK1A1;GFPT1;MRPL27;GATC;PLEKHA3;GTF2H1;BFAR;AZ
;PCDHA6;TFAP2B;GABRA1;MGAM;PHC2;CRBN;BCL11B;GABRA3;PCDHB15;ID4;SPRY3
;HNRNPC;TARDBP;RAD9A
ABHD15;HEYL;OCLN;DPY19L2;TMEM56;RCAN2;LHX4
HR;STXBP5L;SULF2;CDKN2AIPNL;PQLC1;CTNNB1;CD244
BP5L;MAPK10;PARP11;GLUD2;RFWD3;SPRY3;CTNNB1;REN;LSM14B
;HELZ;ADAT2;TPCN1;PDP2;CDKN2AIPNL;GPAM;MXD1;NUPL1;PLEKHM3;RAD9A
09;ABHD15;C7ORF60;ZNF70;GPAM;PITHD1;MXD1;TECPR2;DNAL1;PLEKHM3
ZNF70;HNRNPK;FRAS1;PDP2;GPAM;PITHD1;MXD1;DNAL1;PLEKHM3;RAD9A
K1;OSTC;RCAN2;RPA4;REN;UBE2K;RAD9A
BEAL1;ABHD15;ZNF70;GPAM;PITHD1;FUBP1;PDCD4;CCNYL1;MXD1;NUPL1;DNAL1
S100B;C12ORF74;EPOR;TSHR;GLUD2;OCLN;SPRY3;CD244

DP2;GPAM;NUPL1;LNX2;RAD9A
CIAD1;CALM1;SERINC3;RAD9A
IAL1;PLEKHM3;RAD9A
M56;ASXL3;CAPRIN1;REN;FRK;FGFR1
0;GPAM;MXD1;SSBP2;RAD9A
4B;LNX2;RAD9A
1;PLEKHM3;C11ORF57;RAD9A
RAD9A

C28A3;METAP2

;BFAR;MARCH7;LSM5;ALDH6A1;CNOT6;HNRNPK;ZFP62;SESTD1;OSTC;FUBP1;JMY;STRN;MXD1;PLE
Z;HOOK3;LSM5;AZIN1;PUM2;CNOT6;OCLN;HNRNPK;ZZZ3;FUBP1;UBE2K;LRP12;BMPR1A;RBM28;CO
ADM3;FZD3;EGR4;BCL11B;FAM78A;ZBTB16;SMAD9;PARVA;NSG1;GRIN2B;SYT7;PHOX2B;ABHD15;RE
;SLC4A10;SMAD9;NSG1;GDF6;SYT7;PHOX2B;ABHD15;RCAN2;PPP1R1B;ID4;LHX6;LHX4;HRK;GPR27
VD1;RSF1;CTDSPL2;SMG7;AFF4;CDC73;MED17;ADAMTS5;DTWD1;MED14;SART3;SIPA1L1;XPO4;DDI
RL1;TSHR;STXBP5L;MARCKS;KIF26B;RCAN2;CAMK4;LHX6;LHX4;LRP12;ITM2C
6;LHX4;HRK;GPR27;NRP2;ONECUT2;DIRAS2;SIX1;BMI1;JPH1;HK2;ADAMTS5;JPH3;MECOM;SCN5A;E
F6;PHOX2B;OCLN;EFNA3;PPP1R1B;LHX6;LHX4;BMPR1A;FGFR1;HRK;GPR27;BMPR2;ANKRD33B;KCN
6;GRIN2B;PHOX2B;REEP1;EFNA3;RCAN2;ID4;LHX6;LHX4;HRK;GPR27;ONECUT2;KCNC2;CELF3;DIRA
A2A;PTP4A1;KAT2B;RAP2A;IL1RAPL1;NOS1AP;NAT8L;MAPRE2;FYTTD1;PPM1L;PPM1H;ZDHHC21;ZB
GEE1;ST13;SS18L2;SREK1IP1;LNPEP;KLHL23;SMARCA1;LSM5;BTBD9;RCAN1;SP1;SESTD1;CAPRIN
OCK5;BMPR2;ANKRD33B;CELF3;PPP1R9A;JPH1;ADAMTS5;NHSL2;SPRED1;EEF2K;SIPA1L1;TMEM10
L2;PPP1R9A;PITPNC1;ELAVL2;PHF8;ADAMTS5;MED14;SPRED1;TRPS1;KIF1B;CCDC50;GPR135;HS3S
1;SORCS1;PAX5;IL17RD;ADRA2B;PAX2;ADRA2A;FAM101B;FCHSD2;PDE5A;ARHGEF5;MYRF;PLAG1;F
M16;LONRF3;SCN3B;ABCA1;CADM3;FZD3;PHC2;MAGEE1;FAM78A;ZBTB16;ZBTB10;SMAD9;PARVA;N
2;ARL5B;SART3;SIPA1L1;METTL16;CTBS;PHACTR4;KLF10;MAPK1IP1L;TTC33;SGIP1;VPS13C;NSUN3
31;LRP12;BMPR1A;NRP2;BMPR2;TTC22;DUSP19;TNFAIP3;SIX1;CTDSPL2;JPH1;PITPNC1;PHF8;SPRE
IAD9;PARVA;NSG1;MARCH7;L1CAM;SMARCA1;PHOX2B;CNOT6;ARHGAP31;SP1;SESTD1;RCAN2;PPF
3D9;FBXO30;GLUD2;ARHGAP31;SP1;PPP1R1B;JMY;LHX4;LRP12;FGFR1;TNFAIP8;BMPR2;SNAP23;DU
;PCDHA2;SCN3B;RIMKLA;PCDHA9;PCDHA8;PCDHA7;PCDHA6;MARK1;FZD3;EGR4;BCL11B;FAM78A;Z
;ING4;MRPL42;ADAMTS5;UBL3;SIPA1L1;SH3PXD2A;CTBS;PHACTR4;VKORC1;MAPK1IP1L;PCYT1B;G
LUD2;ARHGAP31;SP1;SESTD1;DNAL1;LRP12;FGFR1;DYRK3;BMPR2;ONECUT2;DUSP19;TNFAIP3;CT
CDHA5;PLXNA2;EIF4EBP2;PCDHA4;CLSPN;SCN3B;RIMKLA;ZNF148;SNX8;PCDHA8;ZC3H14;PCDHA7
NECUT2;KCNC2;DIRAS2;SIX1;RCSD1;ELAVL2;ADAMTS5;MECOM;TRPS1;SCN5A;CTBS;TEAD1;HS3ST
ARHGAP20;ZNF629;ATXN7;TDG;UBN2;HAS2;C10ORF2;C9ORF171;MPZL3;N4BP2;TMEM86A;ACBD5;C
RHGAP31;SP1;SESTD1;PPP1R1B;NRP2;BMPR2;SNAP23;DUSP19;TNFAIP3;SIX1;CTDSPL2;BMI1;PPP1
ELF3;SLC7A14;CDC73;CKS1B;NHSL2;ADAMTS2;MECOM;MAN1A2;RSPO2;KIF1B;APPL1;GPR135;HS3S
NFAIP3;CTDSPL2;PPP1R9A;JPH1;MYPN;PITPNC1;PHF8;ADAMTS5;SPRED1;FAM154B;TMEM108;KIF1
MLEC;CLSPN;ZC3H14;PCDHA7;PCDHA6;LYN;TMEM86A;CBX6;CCZ1B;EGR4;FZD5;SMURF2;ST13;ZBT
;JMY;LHX4;FGFR1;NRP2;DYRK3;BMPR2;SH3KBP1;DUSP19;TNFAIP3;CTDSPL2;PPP1R9A;MYPN;ADA
P1R9A;PITPNC1;ELAVL2;PHF8;ADAMTS5;ADAMTS2;SPRED1;TRPS1;FAM154B;SH3BGRL2;KIF1B;PRK
;CDC73;MED17;MRPL42;UBL3;SART3;SIPA1L1;ALDH2;SLC22A17;TRIM3;PSD3;FAM73A;SH3BGRL2;UN

TDSPL2;AFF4;CDC73;PITPNC1;MED17;PHF8;DTWD1;MED14;EEF2K;SART3;CAPZB;MAN1A2;PSD4;EM
D1;PRKG1;GPR135;NCOA2;WDR37;GAB1;GAB3;PAX5;CDYL2;ADRA2A;PTP4A1;KAT2B;SNPH;IL1RAPL
2;PPP1R1B;LHX6;LHX4;ITM2C;HRK;GPR27;GABRB1;ONECUT2;KCNC2;TTC22;CELF3;DIRAS2;SLC7A1
TWD1;MED14;CAPZB;XPO4;ALDH2;NKRF;PGM3;CNPPD1;NSUN3;LRRC40;ZFP91;SLC39A13;SMC1A;G
B;JMY;BMPR2;CALCOCO2;SH3KBP1;DUSP19;CTDSPL2;PPP1R9A;PITPNC1;ELAVL2;SPRED1;MECOM
23;DIRAS2;DUSP19;TNFAIP3;SIX1;CTDSPL2;BMI1;PPP1R9A;PITPNC1;ADAMTS5;SPRED1;C1QBP;ZNF
SH3KBP1;SNAP23;PDE3B;DUSP19;TNFAIP3;FMN1;CTDSPL2;PPP1R9A;MYPN;PITPNC1;ELAVL2;PHF8;
1;BMPR2;DIRAS2;MED17;ING4;MRPL42;ARL5B;SERP1;SIPA1L1;XPO4;MAN1A2;UBXN7;CEP170;APPL
KIF1B;CEP170;PRKG1;HS3ST3B1;CDYL2;ADRA2A;PTP4A1;KAT2B;RAP2A;IL1RAPL1;NOS1AP;PAFAH1
P1R9A;LITAF;PITPNC1;PHF8;NHSL2;SPRED1;MECOM;C1QBP;TRPS1;FAM154B;SH3BGRL2;PRKG1;H
ORF213;PITPNC1;PHF8;DTWD1;ARL5B;XPO4;MB;MAN1A2;SIN3A;MFSD5;TRPS1;TRIM3;EMB;RBM7;U
;NKRF;FAM154B;TRIM3;EMB;RBM7;WDR37;TMED8;MME;NSUN3;GAB1;SORCS1;PAX5;ZFY;SENP2;DI
;DTWD1;CAPZB;SCN5A;GPRASP2;APPL1;HS3ST3B1;GPR37;AHSA2;GAB1;SORD;LRRC40;SENP2;ADI
6;C9ORF171;PCDHA3;CLSPN;IP6K1;PCDHA8;PCDHA7;MCTS1;TMEM86A;ACBD5;CBX6;CBX5;CCZ1B;
SP1;JMY;LHX4;BMPR1A;FGFR1;BMPR2;ONECUT2;SH3KBP1;SNAP23;TNFAIP3;SIX1;CTDSPL2;PITPN
PHOX2B;ARHGAP31;RCAN2;PPP1R1B;LHX6;LHX4;GPR27;GABRB1;KCNC2;SH3KBP1;DIRAS2;PTPRM
1R9A;PITPNC1;ELAVL2;PHF8;RBM3;SPRED1;KIF1B;PRKG1;GPR135;HS3ST3B1;GPR37;C3ORF62;ZFX
SLC1A4;APCDD1;TRHDE;MED12L;GJC1;NKAIN1;ARHGAP20;EFNB3;C9ORF171;SCN3B;MARK1;ABCA1
JBL3;SART3;NKRF;FAM73A;SH3BGRL2;PHACTR4;VKORC1;MAPK1IP1L;SGIP1;NSUN3;ITPK1;PROX1;

I;ETFA;CTDSPL2;RCSD1;CDC73;MED14;NKRF;PGM3;SH3BGRL2;RBM7;UBL7;KLF10;ARHGEF10;GPR3
SH3PXD2A;TRPS1;FAM73A;KIF1B;PHACTR4;CCDC112;VKORC1;UTP15;MAPK1IP1L;MME;ITPK1;ZFX;A
ME;NSUN3;C6ORF62;PROX1;DIEXF;CDYL2;KAT2B;PAFAH1B2;FYTTD1;SF3B3;DLST;FAM117B;COL19A1
MCMBP;TAC3;HIVEP2;IP6K1;SCN3B;EDEM3;ABCA2;TMEM86A;FZD3;CCZ1B;FZD5;XRCC5;EDEM1;EPT
SL2;SPRED1;FAM154B;SH3BGRL2;KIF1B;CEP170;HS3ST3B1;GPR37;GAB1;PAX5;ZFX;CDYL2;PTP4A1;
HF8;ADAMTS5;ADAMTS2;SPRED1;C1QBP;SH3BGRL2;KIF1B;CEP170;PRKG1;HS3ST3B1;GPR37;GRID
PL2;LITAF;MYPN;PITPNC1;ELAVL2;ADAMTS5;SPRED1;C1QBP;RSPO2;SH3BGRL2;KIF1B;CEP170;CCD
HRK;NRP2;GABRB1;SH3KBP1;SLC7A14;JPH1;PHF8;NHSL2;ADAMTS2;JPH3;MECOM;SH3PXD2A;SLC2
CH7;BTBD9;PARP11;ALDH6A1;HNRNPK;CAPRIN1;STRN;LSM14B;DAND5;PLEKHM3;LRP12;PDE3B;PPW
MYH10;PLXNA3;EDEM3;B3GALNT2;CREBBP;TMEM86A;MGAM;EIF2B2;CCZ1B;EGR4;SRD5A1;SS18L2;
;BMPR1A;HRK;GPR27;ONECUT2;KCNC2;TTC22;CELF3;SLC7A14;JPH1;GRM5;MECOM;RSPO2;TMEM1
IED17;EEF2K;MB;C1QBP;SH3BGRL2;UBXN7;KIF1B;SCN5A;METTL16;APPL1;NCOA2;C3ORF62;GAB1;A
KIF1B;METTL16;UTP15;TTC33;MSL2;SMC1A;CDYL2;CREB1;RBL1;RAPGEF6;AMER1;USP13;RALA;SF3
D17;IL18BP;ING4;MRPL42;UBL3;KIF1B;CTBS;UNKL;PHACTR4;VKORC1;UTP15;MAPK1IP1L;TSFM;TMEI
IC2;TTC22;DIRAS2;PDE3B;SLC7A14;TNFAIP3;SIX1;RCSD1;ELAVL2;ADAMTS5;SDR16C5;GRM5;ZNF80
TNFAIP3;SIX1;RCSD1;BMI1;PPP1R9A;JPH1;SDR16C5;NHSL2;GRM5;JPH3;MECOM;RSPO2;HS3ST3B1;
C1QBP;UBXN7;KIF1B;CEP170;MAPK1IP1L;CHST7;WDR37;DNMT3A;AHSA2;SLC39A13;ANO6;SMC1A;N
14;TNFAIP3;RCSD1;LITAF;HK2;MED17;ING4;MRPL42;MECOM;SH3PXD2A;ALDH2;TRPS1;RSPO2;PRKG
PL2;PPP1R9A;PITPNC1;ELAVL2;ADAMTS5;ADAMTS2;FAM154B;SH3BGRL2;PRKG1;GPR135;HS3ST3B
KANK2;FYTTD1;ATL2;PRUNE2;ZDHHC21;PRICKLE1;FAM117B;PAPSS2;NEURL1B;UBN2;TMEM68;MBNI
;NRP2;BMPR2;SNAP23;DUSP19;TNFAIP3;SIX1;CTDSPL2;PITPNC1;PHF8;SPRED1;SH3BGRL2;KIF1B;C
ICMBP;MLEC;C9ORF171;LONRF3;SCN3B;RIMKLA;N4BP2;MYH10;TMEM86A;CPSF7;CCZ1B;EGR4;FZD
ORF171;MPZL3;N4BP2;JAM2;TRIM44;PCDHA7;MCTS1;ZNF462;FAM78A;ST13;B3GAT2;ST8SIA3;SS18L
SH3PXD2A;SH3BGRL2;CCDC112;VKORC1;UTP15;ZCCHC24;MAPK1IP1L;GPR37;MME;GRID1;NSUN3;I
3;CEP170;TMED8;GAB1;SMC1A;SENP2;DIEXF;CDYL2;RRM2B;MAPRE3;ST6GALNAC3;ERGIC2;CRK;R
1;UBL7;USP47;GRID1;SMC1A;SENP2;GNL1;DIEXF;ADRA2A;PTP4A1;RAP2B;RBL1;RRM2B;TMEM33;NA
OSTDC1;UBXN7;METTL16;DGCR2;WDR37;GAB1;ANO6;SMC1A;RAP2C;DDX19B;RBL1;TMEM33;PGAM
F706;ATXN7;ZMAT3;C10ORF2;MLEC;C9ORF171;PCDHA2;N4BP2;IGBP1;SEPT10;TMEM86A;TRIM41;ST

NRP2;BMPR2;SNAP23;DUSP19;CTDSPL2;PPP1R9A;JPH1;PITPNC1;ADAMTS5;MED14;ADAMTS2;SPR
WD1;ING4;DTWD1;NHSL2;CAPZB;SIN3A;TRPS1;LRRC40;DDX19B;PGAM5;NAA38;SPEM1;DCP2;ZDHH
;ZCCHC24;PLA2G12A;TMED8;C3ORF62;ANO6;SMC1A;CDYL2;RBL1;RRM2B;NAA35;PGAM5;NAA38;SL
UN3;CBFA2T2;SENP2;RRM2B;PGAM5;SLC25A12;ERGIC2;SOS2;PRTFDC1;KANK2;PPM1L;SEMA3A;LR
AIP3;CTDSPL2;PPP1R9A;PITPNC1;MAN1A2;TRPS1;FAM154B;KIF1B;CCDC50;HS3ST3B1;GPR37;CDYL
FAIP3;FMN1;CTDSPL2;PPP1R9A;MYPN;PITPNC1;ADAMTS5;MED14;KIAA1549;ADAMTS2;SPRED1;C1
YL;ARHGAP31;FAM167A;XK;NFIA;CAPRIN1;CCDC6;YPEL2;C11ORF57;TNRC6B
JSP19;TNFAIP3;SIX1;CTDSPL2;BMI1;PPP1R9A;PITPNC1;ELAVL2;SPRED1;FAM154B;SH3BGRL2;KIF1B
L;ARHGEF10;TMED8;MME;TTC33;ITPK1;PROX1;ZFX;CDYL2;SUMF1;RAP2C;RBL1;ERGIC2;CRK;SLC2
52;DYNLL2;PAX2;NAALADL2;PTP4A1;LCOR;KANK2;PPM1L;SEMA3A;SRSF1;XIAP;PDHB;CYTH3;PPP2C
OA2;C3ORF62;AHSA2;SORD;C6ORF62;GAB3;PAX5;KAT2B;NOS1AP;LCOR;MAPRE3;PDE5A;ST6GALNA
2;GAB1;LRRC40;CDYL2;RAP2C;RAP2B;IL1RAPL1;FAM84A;SLC25A12;RAPGEF6;SOS2;TMEM106A;HDA
S3;RHOBTB3;DTX1;SLC1A4;C17ORF96;TRHDE;FBLN5;NKAIN1;ARHGAP20;CHAD;PRDM16;C9ORF17
1;ZZZ3;SESTD1;PPP1R1B;ID4;NRP2;ADK;RSF1;RCSD1;BMI1;JPH1;LITAF;MYPN;NHSL2;SLC22A17;C1
HOBTB3;DTX1;C17ORF96;TRHDE;FBLN5;GJC1;NKAIN1;ARHGAP20;CHAD;PCDHA5;PCDHA3;PCDHA2;
;ARL5A;UBL7;UTP15;KLF10;TSFM;PCYT1B;PLA2G12A;HSDL1;SGIP1;TFEB;LRRC40;GAB3;CBFA2T2;R
40;ANO6;PROX1;ZFY;NAA38;ERGIC2;RAPGEF6;SEMA3A;FAM129A;TUBD1;MAT2B;UQCR10;ADD3;FAM
PAX5;CBFA2T2;DYNLL2;ADRA2B;ENAH;FCHSD2;TET3;MAPRE3;LPHN2;CRK;SOS2;MAPRE2;STAU1;AT
8;C3ORF62;DNMT3A;AHSA2;GAB1;LRRC40;ZFP91;DIEXF;CDYL2;KAT2B;RAP2A;RAPGEF6;SOS2;RAL
L2;EMB;RBM7;APPL1;WDR37;TMED8;NSUN3;CBFA2T2;SENP2;RAP2C;KAT2B;SOS2;USP15;FAM117B;
NIP2;ATP2B4;LNPEP;PLEKHA3;KLHL23;SYNJ2BP;AZIN1;ARHGAP31;XK;DPY19L2;SP1;LHX4;NUPL1;TI
9;ESR1;SYT7;OCLN;ARHGAP31;SESTD1;RCAN2;FUBP1;NABP1;LRP12;BMPR1A;NRP2;DUSP19;FMN1
EFNB3;VTI1A;MCMBP;C9ORF171;PCDHA3;SCN3B;RIMKLA;HOXC8;ZC3H14;EDEM3;CREBBP;TMEM86
12A;TTC33;ZFP91;HAUS3;SLC39A13;SUMF1;PTP4A1;FAM101B;CREB1;RBL1;TERF2IP;NAA38;CD47;E

;TTC33;NSUN3;SORD;IL17RD;CDYL2;KAT2B;FAM84A;RBMS1;ERGIC2;MAPRE2;SLC29A3;KANK2;AME
RPS1;FAM73A;PHACTR4;CCDC112;HS3ST3B1;UTP15;ZCCHC24;MAPK1IP1L;GPR37;NSUN3;ITPK1;HA
GRL2;CTBS;UNKL;PHACTR4;MAPK1IP1L;ARHGEF10;TTC33;ZFX;CDYL2;RAP2C;KAT2B;ST6GALNAC3
CN3B;N4BP2;LYN;TMEM86A;CPSF7;CCZ1B;EGR4;FZD5;SMURF2;FAM78A;ST13;MRPL27;KLHL23;PAR
PL27;EPT1;RASSF8;TRAPPC8;HELZ;MARCH7;LSM5;BTBD9;ESR1;PARP11;ALDH6A1;OCLN;HNRNPK;C
RBM12;SCN3B;RIMKLA;ZNF148;N4BP2;MYH10;PLXNA3;CPSF7;CCZ1B;EGR4;TIPARP;SRD5A1;ST13;E
1549;SPRED1;MAN1A2;TMEM108;SH3BGRL2;KIF1B;CEP170;HS3ST3B1;GPR37;GRID1;ZFY;CDYL2;KA
MK4;PPP1R1B;LHX4;SLC28A3
;TSFM;GPR37;MME;NSUN3;SORD;HAUS3;SLC39A13;PROX1;ADRA2B;FAM84A;RBMS1;NAA38;MAPRE
;RSPO2;TMEM108;PRKG1;PCYT1B;GPR37;GRID1;GAB3;SORCS1;ZFY;ADRA2B;NRG3;NOS1AP;CLDN
37;HAUS3;PAX5;ZFY;GPCPD1;PAX2;ENAH;RAP2B;MAPRE3;LPHN2;PAFAH1B2;SLC24A2;FYTTD1;HDA
IAA30;TMED8;NSUN3;AHSA2;ANO6;ZFX;RAP2C;RBL1;FCHSD2;CNKSR3;NAA38;SLC25A12;DTL;KANK
5;GAB1;IL17RD;DIEXF;RAP2C;TMEM33;PGAM5;NAA38;ST6GALNAC3;SLC25A12;SOS2;PRTFDC1;USF
DSPL2;PPP1R9A;JPH1;PITPNC1;ADAMTS5;NHSL2;ADAMTS2;SPRED1;TMEM108;KIF1B;CEP170;PRK
LDH2;PSD3;SH3BGRL2;TEAD1;ZFP91;ADRA2A;NRG3;TMEM33;FAM84A;SMTNL2;SLC25A12;ZNF597;N
/I7;CNPPD1;MME;GRID1;TTC33;SGIP1;NSUN3;GNL1;ADRA2B;ADRA2A;ENAH;KAT2B;CREB1;RBL1;FC

DHA7;PCDHA6;MARK1;CADM3;PHC2;EGR4;BCL11B;FAM78A;ZBTB10;RASSF8;SMAD9;PARVA;NSG1;
GLUD2;SP4;STRN;MXD1;DAND5;RAD9A;GPR27;DIRAS2;ETFA;JPH1;CDC73;ELAVL2;DTWD1;NHSL2;JF
P4A1;KAT2B;CNKSR3;NOS1AP;RBMS1;MAPRE2;PRTFDC1;FYTTD1;ZDHHC21;ZBTB44;FAM117B;DFNE

NFAIP8;PDE3B;PTPRM;ELAVL2;ING4;ADAMTS2;SH3PXD2A;FAM73A;SH3BGRL2;SCN5A;PHACTR4;CC
TMED8;TTC33;NSUN3;MSL2;RAP2C;FAM101B;ERGIC2;CRK;MAPRE2;TMEM106A;SLC29A3;DCP2;NAP
1;TRIM3;SH3BGRL2;APPL1;KLF10;NSUN3;SENP2;GNL1;DIEXF;ENAH;FCHSD2;RRM2B;TMEM33;NAA
DHA4;C9ORF171;LONRF3;RIMKLA;N4BP2;PCDHA8;MYH10;MCTS1;TMEM86A;ST13;ZBTB10;LNPEP;P

EF6;FYTTD1;HDAC5;RALA;USP15;HPGD;SEMA3A;ZDHHC21;PPM1K;UQCR10;FAM117B;NXF1;BCLAF
A;ZFX;DIEXF;NAALADL2;PTP4A1;KAT2B;RBL1;RRM2B;NAA35;NAA38;SLC25A12;PAFAH1B2;DCP2;CH
;TFEB;PAX5;ADRA2B;PAX2;ADRA2A;ENAH;TET3;TERF2IP;SMTNL2;CRK;SOS2;MAPRE2;FYTTD1;ATL3
IP1;GAB1;CBFA2T2;SENP2;TGFBR3;KAT2B;CD47;SLC25A12;ZNF597;PRTFDC1;FYTTD1;LUZP1;HPGD
37;WDR37;NSUN3;GAB1;LRRC40;C6ORF62;SLC39A13;ANO6;DYNLL2;ZFX;DIEXF;CDYL2;PAX2;RAP2C
8;NSUN3;GAB1;LRRC40;C6ORF62;SLC39A13;CDYL2;KAT2B;RAP2A;TMEM106A;RNF165;FYTTD1;PRU
1;HSPH1;EFNB3;PCDHA1;CHAD;PRDM16;TAC3;RIMKLA;RAI2;MARK1;SYT5;TFAP2B;CADM3;FZD3;EG
170;ZCCHC24;KLF10;HAUS3;DIEXF;CREB1;TERF2IP;LPHN2;KANK2;SF3B3;DCTN2;NUFIP2;ZBTB44;T
GRL2;CCDC112;MAPK1IP1L;ARHGEF10;GPR37;TMED8;MME;NSUN3;HAUS3;ZFX;ADRA2B;CDYL2;RA
APPL1;MAPK1IP1L;PLA2G12A;C3ORF62;ANO6;PTP4A1;NAA38;ST6GALNAC3;SLC25A12;TMEM106A;
SYT5;ABCA2;TFAP2B;FZD3;RABGAP1L;CBX6;BCL11B;MAGEE1;FAM78A;ST8SIA3;RASSF8;KLHL23;SM
HD15;BFSP2;OCLN;SESTD1;ID4;COX7B;GABRB1;ANKRD33B;KCNC2;PDE3B;SLC7A14;PTPRM;FMN1;
F62;DNMT3A;PAX5;PROX1;CBFA2T2;SENP2;DDX19B;RBL1;FCHSD2;SLC25A12;CHST3;KANK2;RALA;
SA2;GAB1;LRRC40;PROX1;DIEXF;RAP2A;SNPH;SLC25A12;RAPGEF6;SOS2;TMEM106A;PRTFDC1;HD
C112;HS3ST3B1;UTP15;MAPK1IP1L;VPS13C;NSUN3;ITPK1;HAUS3;PROX1;ZFX;CDYL2;RAP2C;KAT2E
CTBS;UNKL;CCDC112;VKORC1;UTP15;ZCCHC24;MAPK1IP1L;ARHGEF10;DFFA;NSUN3;ITPK1;HAUS3
CN3B;RIMKLA;N4BP2;EDEM3;MCTS1;TMEM86A;CPSF7;CCZ1B;FZD5;SMURF2;SRD5A1;SS18L2;EPT1;
2L2-PABPN1;MPZL3;PPARGC1B;JAM2;RAI2;TMEM86A;CCZ1B;EGR4;ST13;KLHL23;RAB11A;GLUD2;N
ORF62;GAB1;SLC39A13;PROX1;DIEXF;CDYL2;ENAH;CREB1;SNPH;NOS1AP;PRTFDC1;CHST3;RNF16
CREB1;RBL1;FCHSD2;TMEM33;CD47;ERGIC2;RAPGEF6;TMEM106A;PAFAH1B2;MYRF;ROCK1;NUFIP2
MED8;NSUN3;AHSA2;GAB1;LRRC40;SLC25A12;RAPGEF6;ZNF597;HDAC5;USP15;SF3B3;HPGD;UQCR
L2;SOSTDC1;GPRASP2;CCDC50;TTC33;C3ORF62;DNMT3A;TFEB;PAX5;ADRA2A;SUMF1;DDX19B;ZN
PL1;UBL7;PCYT1B;NAA30;PLA2G12A;SORD;ZFY;DIEXF;CDYL2;ADRA2A;DDX19B;IL1RAPL1;NAA38;CC
OM;SH3PXD2A;CNPPD1;HS3ST3B1;ZCCHC24;NAA30;HAUS3;C6ORF62;IL17RD;ADRA2B;RAP2B;TET
;MECOM;TMEM108;SH3BGRL2;ZNF800;CCDC50;GPR135;GRID1;TTC33;SGIP1;GAB3;SORCS1;NRG3;
;CELF3;SLC7A14;RCSD1;JPH1;SDR16C5;GRM5;ADAMTS2;JPH3;SLC22A17;RSPO2;TMEM108;SCN5A
FCHSD2;RRM2B;SNPH;NOS1AP;PGAM5;NAA38;NAT8L;CRK;DCP2;PALM2;ATL2;SRSF1;PRUNE2;DLST

.16;CTBS;ARL5A;PHACTR4;VKORC1;TTC33;VPS13C;AHSA2;HAUS3;ERGIC2;NAPA;AMER1;PLAG1;SR
VPS13C;MSL2;SORCS1;ZFY;DYNLL2;ADRA2A;TMEM33;SNPH;FAM84A;TMEM106A;SF3B3;ZDHHC20;T
L;TTC33;NSUN3;RBL1;PAFAH1B2;DCP2;NAPA;PHLPP2;AMER1;USP15;STAU1;ZDHHC20;PALM2;TUBD
47;TTC33;NSUN3;ITPK1;ZFP91;CDYL2;SUMF1;KAT2B;RBL1;ERGIC2;SLC29A3;DCP2;NAPA;PHLPP2;A

FAH1B2;ARHGEF5;DCTN2;SRSF1;ZDHHC21;UQCR10;NXF1;TMEM65;TMEM203;TNKS2;TRA2B;TMEM
N3;ITPK1;HAUS3;FAM84A;SMTNL2;ST6GALNAC3;ERGIC2;SLC29A3;CHST3;RNF165;AMER1;NAPB;PP
;GNL1;ADRA2A;ENAH;RBL1;FCHSD2;RRM2B;TERF2IP;RBMS1;SLC29A3;ARHGEF5;KANK2;ROCK1;T
;SMC1A;PAX2;KAT2B;TET3;LPHN2;SLC24A2;PALM2;PRUNE2;TUBD1;NDNF;LRP6;PURA;NXF1;MAT2A
T3A;GAB1;CBFA2T2;SENP2;RAP2B;PDE5A;RAPGEF6;PRTFDC1;KANK2;HDAC5;USP15;PLAG1;ZBTB
RF62;GAB1;SMC1A;SENP2;CDYL2;RRM2B;NAA38;ST6GALNAC3;SLC25A12;ERGIC2;RAPGEF6;TMEM
D2;RRM2B;MAPRE3;SLC25A12;NAT8L;RAPGEF6;SOS2;PRTFDC1;HDAC5;USP15;PPM1L;PLAG1;SEM
ARCA1;BTBD9;GDF6;RCAN1;ARHGAP31;CAPRIN1;JMY;STRN;BMPR1A;NRP2;TNFAIP3;FMN1;JPH1;M
;GPR27;ANKRD33B;KCNC2;DIRAS2;SLC7A14;SIX1;BMI1;PPP1R9A;AFF1;ADAMTS5;ADAMTS2;SERP1
T3;NKRF;TRIM3;PGM3;RBM7;APPL1;UBL7;CCDC112;UTP15;KLF10;PLA2G12A;LRRC40;SLC39A13;GA
TNL2;PAFAH1B2;KANK2;MYRF;ZDHHC20;ATL2;FAM129A;TUBD1;GRAMD4;TRAK2;PPP2CA;NEURL1B;
2B;CADM3;EGR4;BCL11B;FAM78A;ZBTB16;ST8SIA3;SLC4A10;SMARCA1;GRIN2B;C12ORF74;SYT7;P
;GAB1;LRRC40;SLC39A13;PAX5;SENP2;KAT2B;RAP2B;IL1RAPL1;SOS2;PRTFDC1;PRUNE2;TRAK2;C2
A38;RAPGEF6;TMEM106A;PRTFDC1;HPGD;SEMA3A;HOXD12;UQCR10;NEURL1B;ERBB4;TDG;S1PR1
IC2;MAPRE2;PAFAH1B2;ARHGEF5;NAPA;PHLPP2;ZDHHC20;TUBD1;PURA;RBBP5;MGAT5;TMEM68;U
1A;GPR27;ALAS2;NRP2;KCNC2;TTC22;DUSP19;PTPRM;FMN1;PPP1R9A;IL18BP;GRM5;JPH3;UBL3;S
RT1;ST13;B3GAT2;SS18L2;LNPEP;PLEKHA3;KLHL23;LSM5;BTBD9;TTC9;ARHGAP31;EFNA3;SP1;ST
7;CAMK4;CAPRIN1;ID4;CTNNB1;PDE7B
C17A6;PCDHA7;S100PBP;MARK1;IGBP1;RRM1;RABGAP1L;CBX5;FZD5;TPBG;B3GAT2;MRPL27;PCD
2;PCBD2;N4BP2;TRIM44;RAB11FIP5;PLXNA3;SRPK2;CPSF7;EIF2B2;CCZ1B;FZD5;SMURF2;SPRYD7;S
;SMNDC1;GALNT7;SRPK2;IGBP1;RABGAP1L;TIMMDC1;CBX5;TIPARP;EDEM1;CNBP;EPT1;GATC;SRE
RF2;PCDHA4;VPS54;GPC5;PCDHA3;APBB2;SLIT2;SCN3B;RIMKLA;MPZL3;MARK1;TMEM86A;EGR4;S
NB59;ZNRF3;TMEM203;TNKS2;UBN2;MCMBP;LRIG2;SMNDC1;PLXNA3;LYN;ABCA2;FZD3;CPSF7;CRB
8;G3BP1;NT5DC3;VTI1A;PLXNA2;VPS54;MPZL3;TRIM44;RAB11FIP5;B3GALNT2;TIMMDC1;EGR4;SRD
NF180;HSPA4L;PPM1H;FAM129A;DTX4;COL19A1;NEURL1B;NKAIN1;ARHGAP20;CARNS1;CHAD;GPC
XF1;HSPH1;ERI1;VTI1A;FAM63B;PTK2B;RPP14;MPZL3;ZC3H14;TRIM44;PCDHA7;SRPK2;RABGAP1L;
N4BP2;CBX5;TIPARP;GFPT1;MRPL27;LNPEP;TRAPPC8;PARVA;HELZ;BTBD9;AZIN1;ARHGAP31;NCE
B11FIP5;MARK1;ABCA2;TMEM86A;CBX5;CCZ1B;EGR4;FZD5;SMURF2;TIPARP;ST13;MRPL27;RASSF
A;GBX2;TMEM68;PTK2B;LRIG2;MPZL3;ATOH1;B3GALNT2;MAP3K2;TMEM86A;ZBTB14;FBXL17;SREK1
L13;ZNF629;ERI1;TDG;HAS2;VPS54;PCBD2;LRIG2;RIMKLA;BCL2L2-PABPN1;SMNDC1;PLXNA3;CPSF
RIG2;RIMKLA;BCL2L2-PABPN1;MPZL3;SMNDC1;MYH10;B3GALNT2;CBX6;TIMMDC1;CBX5;SRD5A1;X
2;SRPK2;MAGEE1;SRD5A1;SPRYD7;MRPL27;FBXL17;SREK1IP1;UBE2G1;GTF2H1;SYNJ2BP;LSM5;B
LA;SRSF10;MYH10;JAM2;B3GALNT2;ABCA2;FAM46C;PCGF3;GTF2H1;HELZ;NSG1;L1CAM;BTBD9;SY
L3;SNX8;MYH10;RAB11FIP5;PLXNA3;EDEM3;B3GALNT2;IGBP1;CREBBP;TMEM86A;CRBN;ST13;FAM
X4;ITM2C;FGFR1;HRK;GPR27;DOCK5;ONECUT2;DIRAS2;PTPRM;PPP1R9A;ELAVL2;ADAMTS5;ADAM
AP3M2;ARHGAP20;PABPN1;PCDHA1;TRA2B;PTK2B;C9ORF171;LONRF3;SLIT2;MPZL3;SNX8;ZC3H14;
M68;S1PR1;C9ORF171;PCBD2;SLIT2;IP6K1;MPZL3;MYH10;S100PBP;EDEM3;CREBBP;CPSF7;TRIM41
L1;CYB5R4;AP5M1;TMEM203;MAT2A;TMEM68;VTI1A;GPC5;TRIM44;EDEM3;SRPK2;CRBN;NEK7;MRP
;TMEM68;PLXNA2;C10ORF2;PCDHA4;C9ORF171;PCBD2;SCN3B;RIMKLA;TRIM44;S100PBP;EDEM3;B
1A;MCMBP;MLEC;MAPK1;LRIG2;MBNL3;MPZL3;PCDHA6;MCTS1;MAP3K2;GABRA1;CBX5;ZBTB14;CS
ELZ;ESR1;SYT7;PARP11;HNRNPK;SP1;PPP1R1B;LRP12;RAD9A;BMI1;PFAS;HK2;MED17;ING4;MRPL4
X2;TMEM68;VTI1A;PTK2B;GSE1;JAM2;RAI2;ATOH1;ABCA1;TRIM41;ZBTB16;ZBTB10;SREK1IP1;HELZ;
N4BP2;SNX8;SEPT11;TMEM86A;CPSF7;EGR4;XRCC5;SORT1;GABRA3;KLK13;GATC;PDAP1;LNPEP;H
RL1;MAT2A;MIER3;RBBP5;TMEM68;VTI1A;FAM63B;C10ORF2;VPS54;N4BP2;SMNDC1;PLXNA3;TMEM
K4;PPM1E;MED12L;NPAT;UBN2;RNF217;VTI1A;MCMBP;APBB2;SRGAP2;MAP3K2;SEPT11;SRD5A1;SU
;SRPK2;CPSF7;TRIM41;TIMMDC1;FZD5;SS18L2;RASSF8;PDAP1;LNPEP;GTF2H1;U2SURP;ADAT2;PU
S100PBP;LYN;EDEM3;SYT5;EGR4;TIPARP;SRD5A1;FAM78A;YLPM1;LAPTM5;PDAP1;PTPN11;GATAD2
RIMKLA;MBNL3;SRGAP2;MPZL3;TRIM44;EDEM3;MCTS1;MAP3K2;TRIM41;CBX5;ZBTB14;ZBTB16;NEK

)2;IP6K1;N4BP2;S100PBP;FZD3;CPSF7;CCZ1B;FZD5;SS18L2;FOXJ2;GATC;RASSF8;PDAP1;SREK1IP1
\;NRP2;BMPR2;TTC22;DIRAS2;DUSP19;TNFAIP3;FMN1;RCSD1;BMI1;PPP1R9A;LITAF;SIPA1L1;SLC22/
;TMEM203;ATXN1L;VTI1A;C10ORF2;PTK2B;TAC3;ZNF148;MPZL3;SRSF10;LYN;FZD3;EDEM1;MRPL27;
1PR1;C10ORF2;VPS54;MLEC;PCBD2;MPZL3;SMNDC1;EDEM3;CPSF7;TIMMDC1;EIF2B2;NEK7;MRPL2
2;PHLPP2;FYTTD1;DLST;PRICKLE1;UQCR10;PAPSS2;TRAK2;TMEM65;SERTAD2;GPC1;TMEM68;GPC

\PPL1;UBL7;VKORC1;KLF10;AHSA2;GNL1;RAP2B;CREB1;FCHSD2;RRM2B;NAA35;TERF2IP;SOS2;SL(
RIC8B;TMEM203;MAT2A;PABPN1;TRA2B;UBN2;FAM63B;MPZL3;ZC3H14;TRIM44;SNX6;CPSF7;EIF2B2
3B;N4BP2;PCDHA8;RAI2;PCDHA7;SEPT11;TMEM86A;SEPT12;FAM78A;LGI2;RASSF8;SREK1IP1;ATP2I
T5;TRA2B;E2F1;PLXNA2;RBM12;RIMKLA;RPP14;MPZL3;PPARGC1B;FZD3;CCZ1B;MAGEE1;SPRYD7;F
3D;UBA6;HSPA4L;FUT11;CREBL2;NPAT;CARNS1;PCDHA5;MCMBP;PCDHA3;CLSPN;MPZL3;JAM2;RAI2
\5;MCMBP;PCDHA4;PCDHA3;PCDHA9;MBNL3;MPZL3;ABCA1;EDEM3;ZBTB14;KCNIP2;FBXL17;SMAD9
\RHGAP31;ZFP62;SESTD1;SP4;PPP1R1B;LHX4;TECPR2;NFE2L1;DAND5;LRP12;RAD9A;BMPR1A;HRK
2;RCAN2;DAND5;HRK;PHF20;CALCOCO2;SNAP23;DIRAS2;DUSP19;JPH1;LITAF;IL18BP;PHF8;RBM3;S/
ICTS1;CBX5;CRBN;ZBTB14;SMURF2;ZBTB16;NEK7;MRPL27;ATP2B4;GATC;TMEM64;MARCH7;SYNJ2I
P1;RIMKLA;EIF4E;MYH10;SLC25A24;MARK1;B3GALNT2;SORT1;KCNIP2;ZBTB16;GFPT1;SPRYD7;LGI2
EFNB3;GPC1;NRIP1;GSE1;S1PR3;PCBD2;SRGAP2;EIF4E;JAM2;ABCA1;AAED1;SEPT11;ZBTB14;SLC4/
XNA3;CPSF7;CBX5;FAM78A;B3GAT2;MRPL27;GATC;TRAPPC8;ATP2B1;LSM5;SORBS3;PHOX2B;ABHD
3B;RBBP5;ERBB4;PRDM16;MCMBP;ZNF423;RPP14;RELT;ABCA1;EDEM3;SYT5;SRPK2;CBX5;PRRX1;F
;SYNJ2BP;LSM5;GATAD2B;GTF2H5;ADAT2;TPCN1;PUM2;SMAD7;NECAP2;MARCKS;PDP2;CDKN2AIP

TRIM44;LYN;CBX6;TIMMDC1;SRD5A1;FAM46C;ZBTB16;MRPL27;SREK1IP1;LNPEP;MARCH7;SMARCA
T5;CA12;IGBP1;SEPT10;TRIM41;EIF2B2;CBX5;CRBN;ST13;ZBTB16;CNBP;TRAPPC8;BFAR;SOD2;ESR
EM86A;CCZ1B;EGR4;FZD5;FAM46C;GABRA3;LGI2;GATC;PDAP1;LNPEP;TRAPPC8;HELZ;U2SURP;AD/
IDE;ADD2;GJC1;NKAIN1;PLXNA2;TAC3;SCN3B;SLC25A24;SYT5;ZNF462;SEPT11;SRD5A1;FAM46C;ST
2;SCN3B;RIMKLA;EDEM3;TMEM86A;RANBP3;CBX5;KLK13;PCDHB15;PDAP1;HELZ;S100B;GDF6;SOR
N3B;RIMKLA;MPZL3;B3GALNT2;TMEM86A;SRD5A1;FAM78A;ZBTB16;GATC;BTBD9;ADAT2;LETM1;TTC
LA;MPZL3;N4BP2;CPSF7;CCZ1B;EGR4;XRCC5;GABRA3;LGI2;GATC;PDAP1;SREK1IP1;LNPEP;TRAPP(
PRASP2;HS3ST3B1;GPR37;MME;GRID1;GAB3;SORCS1;IL17RD;ADRA2B;NRG3;IL1RAPL1;COL6A5;MA
2;IP6K1;N4BP2;PCDHA7;EDEM3;CA12;CREBBP;SEPT12;CCZ1B;FAM46C;SUSD1;FOXJ2;PCDHB15;TR/
EPT10;CPSF7;TIMMDC1;ZBTB14;SORT1;ST13;FOXJ2;GATC;SREK1IP1;GTF2H1;TRAPPC8;MARCH7;U2
1;VPS54;C9ORF171;SCN3B;MPZL3;JAM2;CA12;ABCA2;CBX5;FAM46C;PDAP1;SOD2;SYNJ2BP;SORL1
DC1;SYT5;CREBBP;CBX6;TRIM41;RANBP3;XRCC5;PCGF3;GABRA3;PCDHB15;SREK1IP1;GTF2H5;SM/
MCTS1;CREBBP;XRCC5;SORT1;NBEAL1;PDAP1;MTSS1L;SREK1IP1;GTF2H1;SYNJ2BP;BTBD9;ESR1;

CPSF7;TRIM41;TIMMDC1;SMURF2;YLPM1;GATC;PDAP1;LNPEP;GTF2H1;TRAPPC8;HOOK3;ADAT2;AL
\RHGAP20;EFS;PABPN1;MGAT5;TRA2B;C10ORF2;C9ORF171;MPZL3;EIF4E;JAM2;TRIM44;ABCA2;TME
CNBP;KCNRG;DEK;BTBD9;GATAD2B;FBXO30;U2SURP;AZIN1;PUM2;DPY19L2;KANSL1;KLHL7;CARM
SF7;EGR4;BCL11B;FAM46C;GABRA3;GATC;PDAP1;GTF2H1;TRAPPC8;LETM1;HEYL;GLUD2;XK;DPY19
3;N4BP2;MYH10;RAI2;MARK1;B3GALNT2;CA12;SEPT10;SUSD1;PDAP1;LNPEP;ADAT2;PUM2;LETM1;H
MCTS1;ABCA2;TPBG;ATP2B4;BFAR;ATP2B1;S100B;C12ORF74;EPOR;STXBP5L;SULF2;PUM2;REEP1;N
I;TRA2B;FAM63B;MCMBP;LONRF3;SNX8;CRBN;SORT1;TPBG;UBE2G1;PARVA;SYNJ2BP;LSM5;REEP1
IP6K1;RPP14;ZNF148;MPZL3;SEPT10;CBX6;BCL11B;USP9X;ST13;ZBTB16;ZBTB10;LNPEP;KLHL23;HE
DHA3;SCN3B;MPZL3;TMEM86A;EGR4;FZD5;SRD5A1;GATC;BTBD9;TSHR;TTC9;GLUD2;XK;GPAM;RFW
4B;UBE2K;PLEKHM3;BMPR1A;HRK;BMPR2;PRR3;FMN1;SMG7;IL18BP;SIPA1L1;SH3PXD2A;SLC22A17
SREK1IP1;TRAPPC8;HELZ;PARP11;EFNA3;HNRNPK;NCEH1;ZFP62;ID2;OSTC;PPP1R1B;NABP1;STRN
ISD1;TMEM68;G3BP1;VTI1A;C10ORF2;BCL2L2-PABPN1;MPZL3;SMNDC1;ZNF462;CPSF7;EIF2B2;SRD
3B;RPP14;MPZL3;EIF4E;SNX8;TRIM44;ABCA2;RANBP3;TSHR;NECAP2;OCLN;MYO1C;KIAA0101;KIF26
\;CPSF7;RANBP3;EGR4;SRD5A1;FAM46C;GATC;PDAP1;TRAPPC8;HELZ;SORL1;SORBS3;SYT7;EPOR
PN1;MPZL3;RAB11FIP5;PLXNA3;B3GALNT2;FZD3;RABGAP1L;TIMMDC1;CCZ1B;MAGEE1;TIPARP;GFP
KLK13;PCDHB15;NFATC3;EPT1;GATC;NBEAL1;SREK1IP1;LNPEP;ATP2B2;HOOK3;TSHR;SMAD7;LETM
;SPRYD7;MRPL27;GATC;GTF2H1;HOOK3;SYNJ2BP;MAPK10;RCAN1;FAM167A;ZNF70;ZZZ3;PDP2;GP/
IKLA;N4BP2;ATOH1;PCDHA7;CREBBP;EGR4;FZD5;ST13;FAM46C;EPT1;GATC;NBEAL1;RASSF8;SREK

;TRIM44;CBX6;EIF2B2;CBX5;EDEM1;GATC;BTBD9;ADAT2;FAM167A;ZZZ3;CDKN2AIPNL;GPAM;SP1;EI
2;SEPT11;CBX5;TIPARP;ST13;FAM46C;NEK7;GABRA3;PCDHB15;ATP2B4;PARVA;DEK;HIPK1;CDC42BF
LYN;SYT5;FZD3;SEPT12;FAM78A;SORT1;B3GAT2;KLHL23;ATP2B2;L1CAM;SMARCA1;SELE;SYT7;PH
;C9ORF171;RIMKLA;ABCA1;SYT5;CA12;ABCA2;CADM3;FAM78A;B3GAT2;PCGF3;SLC4A10;ATP2B2;IN
HA3;S1PR3;BCL2L2-PABPN1;HOXC8;MYH10;PCDHA6;SYT5;CA12;GABRA1;BCL11B;KLK13;PCDHB15;
EIF2B2;CRBN;ST13;GFPT1;SS18L2;ZBTB10;EPT1;GATC;RASSF8;SREK1IP1;GTF2H1;TRAPPC8;U2SU
1;L1CAM;SMARCA1;S100B;LSM5;GDF6;EPOR;TSHR;STXBP5L;ABHD15;TTC9;OCLN;FAM167A;DPY19L
0ORF2;PCDHA4;GPC5;PCDHA3;PCBD2;MPZL3;N4BP2;PCDHA7;TMEM86A;EGR4;GATC;HELZ;ADAT2;
A;SORT1;ST13;GFPT1;MRPL27;FOXJ2;TPCN1;C20ORF112;MYO1C;CDKN2AIPNL;ZFP62;PITHD1;ID2;C
BNL3;EIF4E;RELT;MAP3K2;ABCA2;SEPT12;BCL11B;TPBG;PARVA;KCNRG;EPOR;PUM2;REEP1;ITCH;D
P;LYN;EDEM3;GATC;RASSF8;PDAP1;LNPEP;GTF2H1;ADAT2;C7ORF60;ZNF70;ZZZ3;GPAM;ZFP62;KIF
BP1;C10ORF2;RPP14;EIF4E;MYH10;SLC25A24;ABCA1;MCTS1;TMEM86A;TIPARP;EPT1;GATC;PDAP1
CL11B;FAM46C;ZBTB16;SUSD1;FBXL17;BFAR;L1CAM;GRIN2B;TSHR;HEYL;TMEM56;CARM1;RCAN2;C
HA5;PCDHA3;MBNL3;MPZL3;RAI2;PCDHA6;MARK1;MCTS1;MAP3K2;LAPTM5;FBXL17;SREK1IP1;CDC
IPZL3;ATOH1;TMEM86A;FZD3;CPSF7;EGR4;FZD5;FAM78A;GATC;AZIN1;ADAT2;C20ORF112;ITCH;FAM
AD9A;HRK;DOCK5;DYRK3;BMPR2;SLC7A14;PTPRM;PFAS;MED17;ING4;MRPL42;ADAMTS5;NHSL2;SA

AB11FIP5;SRPK2;IGBP1;CREBBP;FZD5;FAM78A;MRPL27;FOXJ2;NBEAL1;HELZ;MARCH7;HIPK1;RAB1
;CSNK1A1;NEK7;GFPT1;MRPL27;GATC;PLEKHA3;PARVA;BFAR;SOD2;BTBD9;CNOT6;PITHD1;OSTC;F
NL1;PCGF3;MRPL27;GTF2H1;TRAPPC8;KCNRG;S100B;TSHR;STXBP5L;SULF2;NECAP2;CLCN5;EFNA
1;PCDHA3;PCDHA2;MPZL3;PCDHA8;TRIM44;ATOH1;PCDHA7;SLC25A24;PCDHA6;EGR4;BCL11B;TXN
;CPSF7;EIF2B2;CNBP;PCGF3;PCDHB15;GATC;PDAP1;TMEM64;SOD2;CLCN5;FAM167A;HNRNPK;VAP
PLXNA3;ACBD5;FZD5;XRCC5;SORT1;MRPL27;PCDHB15;MTSS1L;L1CAM;LETM1;MAPK10;HEYL;OCL
L27;GATC;TRAPPC8;S100B;SOD2;LSM5;TTC9;BFSP2;ITCH;ARHGAP31;FAM167A;ID2;KLHL7;CAMK4;
ATC;NBEAL1;TRAPPC8;SYNJ2BP;FBXO30;GTF2H5;ADAT2;MAPK10;ZNF70;GPAM;ID2;CCDC6;CCNYL
;N4BP2;ATOH1;MARK1;MCTS1;ABCA2;TMEM86A;CBX5;EGR4;TPBG;NEK7;GABRA3;NFATC3;GTF2H5;
0B;ASPRV1;SORBS3;ESR1;TSHR;STXBP5L;OCLN;FAM167A;RCAN2;PPP1R1B;TCEA3;LHX6;PDE7B;O
A1;NEK7;GFPT1;GATC;PDAP1;PLEKHA3;GTF2H1;PARVA;BFAR;VAPA;VAPB;PITHD1;KLHL7;CAPZA1;C
;EGR4;LSM5;ESR1;EPOR;TSHR;STXBP5L;SULF2;NECAP2;OCLN;RCAN2;PPP1R1B;TCEA3;SLC28A3;F
AM78A;ST13;KCNIP2;B3GAT2;SLC4A10;KLHL23;TBX5;GRIN2B;C12ORF74;SELE;EPOR;STXBP5L;OCL
7;GTF2H1;HOOK3;GTF2H5;ADAT2;SMAD7;C7ORF60;CDKN2AIPNL;GPAM;SESTD1;ID2;CCNYL1;PDE7
A3;GTF2H1;BFAR;SOD2;LSM5;BTBD9;CNOT6;MARCKS;PITHD1;ID2;CAPZA1;SERBP1;RPA4;OCIAD1;
T12;EGR4;TPBG;L1CAM;S100B;TSHR;STXBP5L;SULF2;MAPK10;TTC9;DPY19L2;PDP2;KIF26B;RCAN2
23;PPWD1;SMG7;CDC73;AFF1;CKS1B;DTWD1;ARL5B;SART3;PSD4;FAM154B;PSD3;ARL5A;UNKL;GP
42BPA;SELE;FBXO30;STXBP5L;ARHGAP31;HNRNPK;GPAM;ZFP62;RAB3GAP2;HNRNPC;LSM14B;SSB
;GATC;PDAP1;TRAPPC8;ADAT2;LETM1;REEP1;HEYL;DPY19L2;GPAM;KIAA0101;RFWD3;ASXL3;CAMK
DAT2;KIAA0101;RFWD3;EIF3J;JMY;CCDC6;CCNYL1;FRK;PLEKHM3;LRP12;YPEL2;C11ORF57;RAD9A
2;TIMMDC1;XRCC5;MTSS1L;GTF2H1;HELZ;KCNRG;L1CAM;SYT7;GTF2H5;TSHR;TTC9;XK;PDP2;KIF2
2H1;HOOK3;S100B;SOD2;BTBD9;PHOX2B;TPCN1;LETM1;PARP11;MYO1C;PITHD1;CAMK4;LHX6;TARD
DF6;ASPRV1;TSHR;HIPK2;ADAT2;ARHGAP31;NFIA;TMEM56;SP4;RCAN2;CAPZA1;TCEA3;SPRY1;LHX
;PGM3;RSPO2;DGCR2;GPR135;PCYT1B;TMED8;GAB3;FAM101B;FCHSD2;NOS1AP;MAPRE3;TMEM1
ATC;SREK1IP1;PLEKHA3;GTF2H1;SYNJ2BP;MAPK10;C20ORF112;RCAN1;FAM167A;ZNF70;PDP2;GP
7;SLC4A10;KCNRG;S100B;PHOX2B;TSHR;HIPK2;SULF2;REEP1;MYO1C;PDP2;RCAN2;FUBP1;DAND5
ATP2B1;S100B;SYNJ2BP;EPOR;MAPK10;PARP11;NECAP2;EFNA3;RCAN2;CAPZA1;PTPN4;FGFR1
REK1IP1;PARVA;SMARCA1;ADAT2;LETM1;HEYL;XK;DPY19L2;GPAM;KIAA0101;DNAL1;LRP12;SLC28
MAGEE1;KCNIP2;C12ORF74;STXBP5L;OCLN;DPY19L2;FRAS1;NFIA;APC;CAPZA1;PDE7B;CD244;FGF

YT7;ADAT2;ABHD15;PUM2;MARCKS;ZNF70;GPAM;TCEA3;TRIP12;LSM14B;DNAL1;RAD9A;ITM2C
URP;ADAT2;LETM1;HEYL;GLUD2;FAM167A;XK;RFWD3;SPRY3;LHX6;STRN;PLEKHM3;RAD9A;TNRC6
;BCL11B;NEK7;ST8SIA3;LGI2;KLK13;PARVA;TTC9;ARHGAP31;SP4;SPRY3;CTNNB1;LSM14B;NUPL1;L
P4A1;DDX19B;RBL1;TET3;TERF2IP;SLC25A12;CRK;TMEM106A;PRTFDC1;NAPA;ATL2;PDHB;UQCR10
17;GATAD2B;GTF2H5;SMAD7;CNOT6;FUBP1;SERBP1;OCIAD1;HNRNPC;LSM14B;LNX2
;GTF2H5;SMAD7;GLUD2;ZNF70;GPAM;ZFP62;PITHD1;MXD1;TECPR2;DNAL1;RAD9A;ITM2C;CDS2

1;MARCKS;HNRNPK;CDKN2AIPNL;CCNYL1;MXD1;SERINC3;RAD9A;ITM2C;FGFR1
;SREK1IP1;GTF2H1;ADAT2;FAM167A;PDP2;GPAM;ID2;FAM171B;TRIP12;NFE2L1;TNRC6B
S3;ESR1;SELE;TSHR;HEYL;OCLN;DPY19L2;FRAS1;PQLC1;TMEM56;RCAN2;ASXL3;CAMK4;PPP1R1B
RBN;FAM78A;SPRYD7;MRPL27;TRAPPC8;SOD2;ALDH6A1;ZNF70;PITHD1;ID2;CARM1;MXD1
IKL;CNPPD1;TTC33;MSL2;SORD;HAUS3;SLC39A13;DIEXF;ADRA2A;SUMF1;PTP4A1;DDX19B;CREB1;T
N1;LHX6;LHX4;CALM1;DAND5;PLEKHM3;CC2D1B;HRK;DOCK5;ALAS2;CHURC1-FNTB;PDE3B;DUSP1
LDH2;RSPO2;HS3ST3B1;GPR37;TMED8;IL17RD;DYNLL2;TGFBR3;SNPH;RBMS1;MAPRE3;NAT8L;ARH
GRIN2B;ASPRV1;ESR1;EFNA3;NCEH1;PITHD1;RCAN2;SPRY1;TECPR2
AN2;PPP1R1B;ID4;SPRY1;LHX4;SERINC3;PLEKHM3;TNRC6B;CC2D1B
1;BTBD9;PUM2;BFSP2;CNOT6;PITHD1;TMEM56;SP4;RPA4;OCIAD1;TARDBP;MXD1;TECPR2;RAD9A;C
;HNRNPC;PLEKHM3;CREBZF;RBM28;BMPR2;CELF1;PPWD1;IL18BP;RBM3;DTWD1;SERP1;MFSD5;C1
ITHD1;KLHL7;CAPZA1;RAB3GAP2;PTPN4;LSM14B;NFE2L1;BMPR1A
N1;VAPB;SP4;KLHL7;CAPZA1;OCIAD1;RLIM;RAB3GAP2;LSM14B;LNX2
EPT10;FZD5;MAGEE1;SORT1;KCNIP2;GFPT1;SUSD1;GTF2H1;KCNRG;L1CAM;SOD2;REEP1;RCAN1;P

3S3;SYT7;ALDH6A1;CDKN2AIPNL;GPAM;PQLC1;TMEM56;TECPR2;ITM2C
;FZD5;SRD5A1;FAM78A;ST8SIA3;L1CAM;BTBD9;ABHD15;PARP11;BFSP2;OCLN;PQLC1;JMY;LHX4;ITM
I7;UNKL;VKORC1;NAA30;TTC33;MSL2;SORD;SLC39A13;CBFA2T2;DIEXF;ADRA2A;SUMF1;DDX19B;CR

PDP2;APC;CAPZA1;TCEA3;PDE7B;FRK;PLEKHM3;FGFR1
SRD5A1;CSNK1A1;GFPT1;MRPL27;PLEKHA3;KLHL23;BFAR;SOD2;RNF148;VAPA;SP1;VAPB;SP4;ID2;C
PFAS;MYPN;CDC73;SART3;SIN3A;METTL16;RBM7;UNKL;GPRASP2;ARHGEF10;WDR37;TTC33;NSUN
2;PDE7B;FRK;ITM2C
AB3GAP2;LHX4;FRK;DAND5;RAD9A;CC2D1B
K;PITHD1;ID2;FUBP1;CAPRIN1;EIF3J;RPA4;CTNNB1;HNRNPC

P5L;HNRNPK;GPAM;PITHD1;ID2;KLHL7;DNAL1;PLEKHM3
N5;ADD2;CYTH3;NKAIN1;ARHGAP20;ZNRF3;APH1B;GPC5;SNX8;ABCA1;SYT5;SEPT12;SMURF2;SRD5
;TMEM106A;PHLPP2;ATL3;ATL2;UQCR10;PPP2CA;PURA;NXF1;TMEM203;MAT2A;PABPN1;MGAT5;C1
2;LHX6;REN;SERINC3;FGFR1;HRK;NRP2;ANKRD33B;TTC22;DIRAS2;PPWD1;FMN1;RCSD1;LITAF;NH
CLCN5;ZNF70;SP4;DNAL1;PLEKHM3;C11ORF57;RAD9A
;BFAR;SOD2;VAPA;ZFP62;VAPB;ID2;FUBP1;CAPZA1;SERBP1;OCIAD1;RLIM;RAB3GAP2;LSM14B;FRK
L2;FRAS1;PDP2;PQLC1;RCAN2;RAD9A
;LSM14B;LNX2
RT3;PSD4;FAM154B;SH3BGRL2;ARL5A;UNKL;UBL7;VKORC1;TTC33;SORD;HAUS3;DIEXF;CREB1;FC

148;SP4;ASXL3;SPRY3;NUPL1
A;MGAM;FZD3;PHC2;SEPT12;CRBN;TIPARP;ZBTB16;SLC4A10;HELZ;CDC42BPA;SELE;HIPK2;ABHD1
PT10;CADM3;FZD3;USP9X;TPBG;LGI2;YLPM1;ZBTB10;L1CAM;SOD2;SYT7;ABHD15;RNF148;OCLN;A
X2
D9A

9A

2;PHC2;BCL11B;ST13;FAM46C;NEK7;GABRA3;SUSD1;C1ORF21;PARVA;SELE;NFIA;VAPB;SPRY3;LHX
14;NHSL2;JPH3;SH3PXD2A;RSPO2;TMEM108;SCN5A;GPR135;ARHGEF10;GPR37;CHST7;SMC1A;DY
P2;PQLC1;SESTD1;LHX6;TECPR2;PLEKHM3;CC2D1B;GABRB1;KCNC2;DIRAS2;ETFA;PFAS;ELAVL2;M
2;KCNRG;NSG1;L1CAM;BTBD9;ABHD15;HEYL;FRAS1;PDP2;VAPA;TMEM56;KIF26B;PPP1R1B;LHX4;U
NBP;GATC;PDAP1;KCNRG;SOD2;SMAD7;CNOT6;HNRNPK;SP1;ZFP62;VAPB;SP4;ID2;RFWD3;SERBP1

PRY3;LSM14B

›

)A
3;PQLC1;LSM14B;MXD1;SERINC3;RBM28;BTG1;ANKRD33B;PRR3;SNAP23;DIRAS2;PPWD1;RCSD1;BI
SP19;PTPRM;FMN1;ADAMTS5;JPH3;UBL3;PSD4;C1QBP;TRPS1;TRIM3;PSD3;TMEM108;SCN5A;DGCR
MBP;LONRF3;IP6K1;BCL2L2-PABPN1;S100PBP;SLC25A24;ACBD5;TIMMDC1;CBX5;TIPARP;CSNK1A1;

2;N4BP2;HOXC8;S100PBP;PLXNA3;ABCA1;MAGEE1;TXNL1;MRPL27;FOXJ2;ADAT2;ABHD15;TPCN1;C
T8SIA3;KLK13;L1CAM;SORBS3;STXBP5L;REEP1;OCLN;CAMK4;FAM171B;LHX4;PDE7B;LSM14B;SLC2
IN1;VAPA;VAPB;OSTC;CAPZA1;RPA4;OCIAD1;RLIM;RAB3GAP2;LSM14B;FRK;LNX2;BMPR1A

:KHM3;LRP12;ITM2C;CDS2;CREBZF;BTG1;MOCS3;BMPR2;CELF1;PTPRM;SMG7;CDC73;MED17;SPRE
X7B;MOCS3;CELF1;PDE3B;CDC73;MED17;ADAMTS2;SPRED1;SERP1;NKRF;MAN1A2;SH3BGRL2;UB
EP1;EFNA3;ID2;PPP1R1B;LHX4;HRK;GPR27;GABRB1;ANKRD33B;ONECUT2;KCNC2;PDE3B;SLC7A14
;NRP2;ANKRD33B;KCNC2;CELF3;PDE3B;SIX1;RCSD1;JPH1;NHSL2;ADAMTS2;JPH3;MECOM;SH3PXD
2;TRIM3;SH3BGRL2;KIF1B;METTL16;ARL5A;TEAD1;APPL1;UTP15;USP47;PLA2G12A;TTC33;ITPK1;HA

MB;PRKG1;HS3ST3B1;GRID1;SORCS1;PAX5;PROX1;ADRA2A;NRG3;SNPH;NAT8L;MYRF;PLAG1;NDN
NC2;TTC22;CELF3;PTPRM;SIX1;BMI1;PPP1R9A;JPH1;NHSL2;ADAMTS2;MECOM;TRIM3;TMEM108;SH
S2;PDE3B;SLC7A14;PPP1R9A;ELAVL2;ADAMTS5;NHSL2;KIAA1549;ADAMTS2;JPH3;SLC22A17;RSPO
TB44;FAM117B;PAPSS2;C21ORF119;EFS;MAT2A;UBN2;NSD1;HAS2;MBNL3;MPZL3;ATOH1;CA12;PLEK
1;PPP1R1B;LHX4;UBE2K;RAD9A;BMPR1A;NRP2;DYRK3;BMPR2;TTC22;SNAP23;SIX1;CTDSPL2;PPP1
8;SH3BGRL2;SCN5A;EMB;PRKG1;GPR135;ARHGEF10;CHST7;MME;GRID1;GAB1;PROX1;PAX2;ADRA
ST3B1;CDYL2;ADRA2A;IL1RAPL1;LCOR;PDE5A;LPHN2;PRTFDC1;FYTTD1;PPM1L;NUFIP2;HPGD;PAL
AM129A;NDNF;HOXD12;PPM1E;GBX2;IGF2BP1;S1PR1;HAS2;PTK2B;S1PR3;SLIT2;RELT;JAM2;ATOH1
SG1;SMARCA1;BTBD9;SESTD1;RCAN2;FGFR1;HRK;GPR27;GABRB1;KCNC2;SH3KBP1;DIRAS2;SLC7
;SENP2;ZFX;CDYL2;RAP2C;KAT2B;ST6GALNAC3;ERGIC2;CHST3;NAPA;ZDHHC20;DLST;ZBTB44;FAM
D1;SART3;KIF1B;HS3ST3B1;NSUN3;PAX5;ZFY;CDYL2;PTP4A1;KAT2B;RAP2A;RRM2B;LCOR;MAPRE3
1R1B;BMPR1A;GPR27;DOCK5;CELF3;TNFAIP3;BMI1;AFF1;GRM5;JPH3;SIPA1L1;SH3PXD2A;ALDH2;S
JSP19;SIX1;CTDSPL2;MYPN;PITPNC1;ADAMTS5;ADAMTS2;SPRED1;C1QBP;SH3BGRL2;KIF1B;CEP17
BTB10;SMAD9;NSG1;SMAD7;REEP1;OCLN;ARHGAP31;EFNA3;SESTD1;ID4;ITM2C;BMPR1A;FGFR1;H
PR37;TMED8;TTC33;SGIP1;VPS13C;MSL2;PROX1;ZFX;CDYL2;TGFBR3;KAT2B;FAM84A;ST6GALNAC
DSPL2;PPP1R9A;PITPNC1;ELAVL2;PHF8;MED14;ADAMTS2;SPRED1;KIF1B;PRKG1;HS3ST3B1;GPR3
;B3GALNT2;SRPK2;TMEM86A;CPSF7;CBX5;MAGEE1;MRPL27;EPT1;SMARCA1;RCAN1;GLUD2;CNOT
T3B1;ZCCHC24;GRID1;DNMT3A;ANO6;SORCS1;PROX1;ADRA2B;PAX2;ADRA2A;NRG3;RBMS1;ST6GA
CZ1B;SORT1;ST13;B3GAT2;PLEKHA3;KLHL23;SMARCA1;LSM5;GDF6;RAB11A;ABHD15;RCAN1;ARH
R9A;PITPNC1;ELAVL2;ADAMTS5;ADAMTS2;SPRED1;C1QBP;FAM154B;SH3BGRL2;KIF1B;PRKG1;HS3
ST3B1;KLF10;MAPK1IP1L;TSFM;GRID1;VPS13C;GAB1;GAB3;PDE5A;SOS2;PAFAH1B2;KANK2;PPM1L;
B;CCDC50;PRKG1;HS3ST3B1;GPR37;WDR37;ZFX;CDYL2;PTP4A1;KAT2B;IL1RAPL1;NOS1AP;FAM84A
B16;MRPL27;RASSF8;PARVA;BTBD9;FBXO30;SYT7;RCAN1;GLUD2;DIAPH2;ARHGAP31;SP1;SESTD1;
MTS5;MED14;SPRED1;C1QBP;TRPS1;KIF1B;PRKG1;HS3ST3B1;GPR37;CDYL2;ADRA2A;PTP4A1;KAT
G1;GPR135;HS3ST3B1;GPR37;GRID1;C3ORF62;GAB3;ZFX;CDYL2;PTP4A1;KAT2B;RAP2B;RRM2B;NO
NKL;PHACTR4;MAPK1IP1L;TMED8;VPS13C;ZFP91;PROX1;ZFX;CDYL2;SNPH;ST6GALNAC3;SLC29A3;

IB;RBM7;CNPPD1;KLF10;USP47;NSUN3;MSL2;ZFP91;HAUS3;SMC1A;CDYL2;PTP4A1;TMEM33;USP15
1;PRTFDC1;FYTTD1;SEMA3A;PALM2;ZDHHC21;ZBTB44;FAM117B;COL19A1;NEURL1B;ZNRF3;MAT2A
4;RCSD1;BMI1;PPP1R9A;JPH1;JPH3;MECOM;RSPO2;TMEM108;SH3BGRL2;GPR37;GRID1;GAB3;PAX
NL1;DIEXF;SUMF1;RBL1;RRM2B;TERF2IP;NAA38;CD47;ERGIC2;SOS2;SLC29A3;RALA;USP15;ROCK
;FAM154B;EMB;PRKG1;GPR135;HS3ST3B1;GPR37;PROX1;ZFX;CDYL2;PTP4A1;KAT2B;RAP2A;SNPH
800;KIF1B;EMB;CCDC50;PRKG1;PHACTR4;HS3ST3B1;GPR37;NSUN3;GAB1;CDYL2;PTP4A1;KAT2B;S
;SPRED1;FAM154B;KIF1B;CEP170;CCDC50;PRKG1;GPR135;HS3ST3B1;PLA2G12A;GPR37;CHST7;PR
1;UTP15;NSUN3;HAUS3;SUMF1;RAP2C;DTL;PHLPP2;RALA;ROCK1;DCTN2;XIAP;ZBTB44;PPM1K;UQC
B2;PRTFDC1;FYTTD1;ATL3;ZBTB44;FAM117B;ADD2;DFNB59;ZNF629;MAT2A;UBN2;HAS2;MAPK1;S1P
IS3ST3B1;GPR37;ZFX;CDYL2;ADRA2A;PTP4A1;KAT2B;RBL1;RBMS1;NAT8L;LPHN2;TMEM106A;CHST
SP47;WDR37;NSUN3;ANO6;SUMF1;PTP4A1;DDX19B;FCHSD2;RALA;USP15;SF3B3;SRSF1;PPM1H;DL
EXF;CDYL2;RAP2C;KAT2B;RBL1;FCHSD2;NAA38;SOS2;ZNF597;RALA;USP15;PPM1L;SEMA3A;PALM2
RA2B;ADRA2A;CREB1;PGAM5;NAA38;COL6A5;SLC25A12;RAPGEF6;PRTFDC1;CHST3;USP15;HPGD;
;FAM78A;ST13;ZBTB16;LNPEP;KLHL23;ESR1;GLUD2;DIAPH2;ARHGAP31;SP1;SESTD1;CAPRIN1;LHX
C1;PHF8;NHSL2;SPRED1;KIF1B;HS3ST3B1;NCOA2;GAB1;PROX1;ZFX;CDYL2;KAT2B;CNKSR3;NOS1A
;JPH1;ELAVL2;GRM5;MECOM;TRPS1;TMEM108;SCN5A;EMB;ARHGEF10;MMP2;SORCS1;ENAH;SNPH
;CDYL2;ADRA2A;KAT2B;RAP2B;NOS1AP;RBMS1;NAT8L;MAPRE2;PRTFDC1;CHST3;FYTTD1;NRXN3;
1;ABCA2;EGR4;SLC4A10;SMAD9;KLHL23;PARVA;REEP1;OCLN;EFNA3;RCAN2;LHX6;LHX4;ITM2C;HRK
ZFX;CDYL2;ENAH;SNPH;SLC25A12;ERGIC2;CRK;MAPRE2;SLC29A3;KANK2;AMER1;USP15;SRSF1;N

37;TTC33;NSUN3;DIEXF;RAP2C;FAM101B;RBL1;FCHSD2;CLDN18;CD47;RAPGEF6;DTL;SLC29A3;ARH
ADRA2B;ADRA2A;FCHSD2;SMTNL2;ST6GALNAC3;NAT8L;ERGIC2;SLC29A3;NAPA;NAPB;MYRF;HPGD;
1;PAPSS2;DFNB59;UBN2;NSD1;HAS2;MBNL3;MPZL3;JAM2;CA12;GABRA1;USP9X;GABRA3;YLPM1;TM
T1;SREK1IP1;TRAPPC8;HOOK3;RCAN1;C7ORF60;GLUD2;EFNA3;PQLC1;SESTD1;SP4;OSTC;LHX6;LS
;NOS1AP;RBMS1;PDE5A;NAT8L;MAPRE2;FYTTD1;SEMA3A;NDNF;ZDHHC21;ZBTB44;FAM117B;LRP6;D
)1;ZFY;CDYL2;ADRA2A;KAT2B;RAP2A;NOS1AP;CHST3;FYTTD1;PPM1L;ZDHHC21;ZBTB44;FAM117B;D
)C50;PRKG1;HS3ST3B1;GPR37;ZFY;CDYL2;ADRA2A;PTP4A1;KAT2B;RAP2A;NOS1AP;CHST3;FYTTD1
2A17;RSPO2;TMEM108;SH3BGRL2;SCN5A;GPR135;GPR37;MMP2;SORCS1;ADRA2B;SNPH;MYOD1;C
VD1;TNFAIP3;BMI1;SMG7;AFF1;MED17;ALAD;ING4;MRPL42;ADAMTS5;SIPA1L1;SH3PXD2A;ALDH2;TR
MRPL27;RASSF8;SREK1IP1;LNPEP;TRAPPC8;HOOK3;RCAN1;C7ORF60;STRN;LSM14B;TECPR2;DNA
108;SCN5A;EMB;GPR37;MME;MMP2;SORCS1;PAX5;ADRA2B;NRG3;SNPH;MYOD1;HPGD;PPM1E;PAP
ANO6;PROX1;RBL1;CNKSR3;NOS1AP;PGAM5;SLC25A12;ERGIC2;TMEM106A;USP13;HDAC5;TUBD1;F
3B3;STAU1;PPM1L;FAM129A;ZDHHC21;LRP6;BCLAF1;EFS;TNKS2;RBBP5;USP1;LRIG2;MPZL3;EMC7;S
D8;VPS13C;NSUN3;SORD;ZFP91;ZFX;CDYL2;RBMS1;ST6GALNAC3;ERGIC2;MAPRE2;SLC29A3;ARHG
0;CCDC50;PRKG1;GPR135;SGIP1;MMP2;ADRA2B;NRG3;IL1RAPL1;COL6A5;SMTNL2;MAPRE2;FYTTD
;GPR37;HSDL1;MME;GRID1;GAB3;PAX5;CDYL2;PAX2;ADRA2A;NRG3;MYOD1;NOS1AP;FAM84A;COL6
NOS1AP;PGAM5;SLC25A12;CRK;TMEM106A;NAPA;NAPB;HDAC5;RALA;PPM1L;PLAG1;ATL2;ZDHHC21
G1;CCDC112;UTP15;ZCCHC24;MAPK1IP1L;DFFA;SGIP1;VPS13C;NSUN3;ITPK1;HAUS3;CDYL2;SUMF1
1;PLA2G12A;GPR37;SORD;ZFY;ZFX;CDYL2;PTP4A1;KAT2B;SNPH;NOS1AP;ST6GALNAC3;CHST3;FYT
L3;MPZL3;CA12;SORT1;PCGF3;GATC;GTF2H1;C1ORF21;TMEM64;HIPK1;ADAT2;ITCH;DPY19L2;NFIA;
CCDC50;PRKG1;HS3ST3B1;GPR37;GAB1;ZFX;PTP4A1;KAT2B;IL1RAPL1;NOS1AP;RBMS1;NAT8L;PAFA
)5;SMURF2;XRCC5;FAM78A;LNPEP;KLHL23;TRAPPC8;HELZ;BTBD9;ARHGAP31;SP1;RCAN2;PPP1R1
L2;YLPM1;LNPEP;KLHL23;TMEM64;HIPK1;LSM5;BTBD9;REEP1;RCAN1;ZZZ3;CAPRIN1;LHX6;STRN;C1
TPK1;HAUS3;PROX1;CDYL2;RAP2C;FAM84A;ST6GALNAC3;ERGIC2;MAPRE2;SLC29A3;CHST3;NAPA;
OCK1;DLST;ZBTB44;DRP2;NEURL1B;EFS;ZNF629;TMEM203;TDG;UBN2;NSD1;S1PR1;S1PR3;PCBD2;
AA38;ERGIC2;DCP2;FYTTD1;HPGD;PALM2;PDHB;PPP2CA;OGFRL1;TMEM65;MAT2A;TNKS2;PABPN1;
5;ST6GALNAC3;SLC25A12;TMEM106A;KANK2;ATL2;PPM1K;NXF1;DFNB59;EFS;ZNF629;TMEM203;TD
T13;MRPL27;ZBTB10;PLEKHA3;KLHL23;RCAN1;MARCKS;OCLN;NFIA;VAPA;CAPZA1;CAPRIN1;CTNNB

ED1;C1QBP;TRPS1;KIF1B;CEP170;CCDC50;PRKG1;HS3ST3B1;GPR37;ZFX;CDYL2;KAT2B;NOS1AP;C
C20;ATL2;DLST;TUBD1;ZDHHC21;ZBTB44;UQCR10;FAM117B;ZNF629;TNKS2;SERTAD2;KIAA0355;RB
LC25A12;ERGIC2;TMEM106A;USP13;HDAC5;ROCK1;PLAG1;TUBD1;C21ORF119;PPP2CA;NEURL1B;O
RP6;TRAK2;C21ORF119;PURA;NXF1;GBX2;RBBP5;NRIP1;FAM63B;C10ORF2;VPS54;SRSF10;SMNDC1;
2;PTP4A1;KAT2B;NOS1AP;RBMS1;NAT8L;MAPRE2;CHST3;FYTTD1;SRSF1;PRUNE2;ZDHHC21;ZBTB4
QBP;TMEM108;KIF1B;CEP170;PRKG1;HS3ST3B1;ZFY;CDYL2;KAT2B;RAP2A;SNPH;NOS1AP;CHST3;F

3;CEP170;PRKG1;HS3ST3B1;GPR37;GAB1;C6ORF62;PROX1;ZFX;CDYL2;KAT2B;NOS1AP;ST6GALNAC
9A3;CHST3;NAPA;PHLPP2;HPGD;ZDHHC20;TUBD1;PURA;OGFRL1;TMEM203;RBBP5;MGAT5;TMEM68
CA;NEURL1B;ZDHHC18;TMEM203;PABPN1;FAM63B;HAS2;C10ORF2;SRSF10;EIF4E;SMNDC1;SORT1;
AC3;FYTTD1;NRXN3;DLST;ZDHHC21;ZBTB44;PRICKLE1;PPM1E;FAM117B;COL19A1;DFNB59;MAT2A;
AC5;USP15;SF3B3;PPM1H;UQCR10;FAM117B;LRP6;TRAK2;DRP2;PURA;TDG;TMEM68;VPS54;PCBD2;
1;LONRF3;RIMKLA;RAI2;SYT5;ABCA2;TFAP2B;EGR4;FZD5;BCL11B;FAM78A;ZBTB16;ST8SIA3;SLC4A1
QBP;TRIM3;RSPO2;SH3BGRL2;SOSTDC1;KIF1B;ZNF367;TMED8;MME;GAB1;LRRC40;PROX1;ZFY;DIE
;ZNF423;PCDHA9;PCDHA7;RAB11FIP5;PCDHA6;PLXNA3;SYT5;TFAP2B;FZD3;EGR4;BCL11B;ZBTB16;
RAP2B;RBL1;PDE5A;ERGIC2;PAFAH1B2;NAPA;FYTTD1;USP15;PPM1L;DLST;PRICKLE1;UQCR10;ADD2
M117B;LRP6;CYTH3;AP3M2;ZNF629;KIAA0355;ATXN1L;USP1;SLC17A6;LRIG2;MPZL3;SLC25A24;GALN
TL3;PPM1L;LUZP1;SRSF1;PPM1H;PPM1K;UQCR10;ADD3;ADD2;CYTH3;NXF1;BCLAF1;TNKS2;UBN2;S
A;PRUNE2;PRICKLE1;UQCR10;AP3M2;NXF1;EFS;TMEM203;RBBP5;TMEM68;NT5DC3;NRIP1;C10ORF
;LRP6;AP3M2;DRP2;OGFRL1;TMEM203;TNKS2;RBBP5;FAM63B;C10ORF2;PTK2B;VPS54;PCBD2;SMN
NRC6B;BMPR1A
;RSF1;PPP1R9A;MED17;PHF8;ADAMTS5;SPRED1;NKRF;TRIM3;PGM3;FAM73A;TMEM108;SCN5A;EM
A;CBX6;CPSF7;CCZ1B;EGR4;SMURF2;ST13;LNPEP;PARVA;HELZ;BTBD9;SYT7;RCAN1;GLUD2;DIAPH
ERGIC2;SLC29A3;RALA;ROCK1;FAM129A;ZDHHC21;ZBTB44;MAT2B;FAM117B;PAPSS2;TMEM203;TNK

:R1;USP15;HPGD;ZDHHC20;TUBD1;PURA;MGAT5;TMEM68;NRIP1;USP1;VPS54;RPP14;MPZL3;MAP3K
AUS3;CDYL2;SUMF1;RAP2C;SNPH;FAM84A;SMTNL2;ST6GALNAC3;ERGIC2;SLC29A3;NAPA;AMER1;N/
3;SLC25A12;ERGIC2;CRK;MAPRE2;AMER1;USP13;USP15;DCTN2;ZDHHC21;CYTH3;ZNRF2;TMEM203;
VA;HELZ;BTBD9;RCAN1;GLUD2;DIAPH2;ARHGAP31;EFNA3;SESTD1;LHX4;LRP12;RAD9A;ITM2C;GPR
CAPRIN1;JMY;STRN;CALM1;LSM14B;RAD9A;BTG1;MOCS3;PPWD1;BMI1;SMG7;MED17;IL18BP;ING4;M
EDEM1;SREK1IP1;LNPEP;KLHL23;HELZ;LSM5;SYT7;PARP11;SP1;CAPRIN1;DNAL1;PLEKHM3;LRP12;F
T2B;NOS1AP;MAPRE3;CHST3;FYTTD1;PPM1H;ZDHHC21;ZBTB44;FAM117B;DFNB59;ZNF629;TDG;UBI

E3;ST6GALNAC3;SLC29A3;AMER1;USP15;PRUNE2;LRP6;AP3M2;NEURL1B;ZNRF2;ZNRF3;GBX2;TMEI
I18;COL6A5;SMTNL2;NAT8L;RNF165;NAPA;HDAC5;PPM1L;HPGD;PLAG1;SEMA3A;FAM129A;NDNF;UQ
AC5;STAU1;ATL3;ZDHHC20;PALM2;ATL2;PRUNE2;PRICKLE1;UQCR10;NXF1;TMEM203;SLC17A6;LRIG
K2;USP15;SF3B3;SEMA3A;ZDHHC21;FAM117B;BCLAF1;ZDHHC18;ZNRF3;GBX2;TMEM203;ATXN1L;FAN
P15;SEMA3A;TRAK2;C21ORF119;NXF1;ZNF629;TMEM203;TNKS2;TDG;TMEM68;VPS54;S1PR3;PCBD2
G1;PHACTR4;HS3ST3B1;ZFY;CDYL2;PTP4A1;KAT2B;RAP2A;NRG3;IL1RAPL1;NOS1AP;NAT8L;FYTTD1
NAPB;STAU1;PPM1L;PPM1E;C21ORF119;AP3M2;ZNRF3;GBX2;PABPN1;TRA2B;USP1;SLIT2;MBNL3;AT
HSD2;CD47;COL6A5;RAPGEF6;SOS2;USP13;RALA;FAM129A;DLST;PAPSS2;NEURL1B;RXRA;TMEM20

SYT7;SMAD7;REEP1;BFSP2;ID2;ID4;ITM2C;BMPR1A;FGFR1;HRK;GPR27;DOCK5;NRP2;ANKRD33B;TE
PH3;SART3;SERP1;TMEM108;SH3BGRL2;SOSTDC1;SCN5A;UNKL;PCYT1B;C3ORF62;DNMT3A;GAB1;I
359;ZNF629;MAT2A;UBN2;PCBD2;MBNL3;IGBP1;SORT1;ATP2B4;PDAP1;GTF2H1;PTPN11;TMEM64;HII

DC112;UTP15;MAPK1IP1L;GPR37;TMED8;NSUN3;HAUS3;SLC39A13;PROX1;ERGIC2;SLC29A3;MYRF;
A;PHLPP2;PALM2;TUBD1;PURA;OGFRL1;MGAT5;GPC1;TMEM68;CARNS1;USP1;MAPK1;LRIG2;RPP14
35;FYTTD1;USP15;SF3B3;PRUNE2;PDHB;UQCR10;PPP2CA;TMEM65;MAT2A;TNKS2;RBBP5;TMEM68;N
ARVA;HELZ;REEP1;PARP11;GLUD2;ARHGAP31;ZZZ3;TECPR2;LRP12;RAD9A;HRK;GPR27;C9ORF91;N

1;TMEM203;SERTAD2;RBBP5;TMEM68;C10ORF2;VPS54;APBB2;S1PR3;PCBD2;MPZL3;S100PBP;TIMM
IST3;ROCK1;ATL2;PPM1H;XIAP;PDHB;FAM117B;C21ORF119;OGFRL1;UBN2;NRIP1;C10ORF2;MAPK1;I
3;PALM2;ATL2;ADD2;DRP2;GNA13;ERBB4;NT5DC3;RPP14;SLC25A24;TRIM41;CSNK1A1;KCNIP2;B3GA
;PPM1H;TUBD1;PDHB;UQCR10;TRAK2;NXF1;TMEM65;RBBP5;TDG;UBN2;TMEM68;ATXN1L;FAM63B;V
;KAT2B;RAP2A;MAPRE2;KANK2;FYTTD1;TUBD1;ZDHHC21;PRICKLE1;UQCR10;FAM117B;TRAK2;UBN
JNE2;TUBD1;ZDHHC21;UQCR10;FAM117B;PAPSS2;UBN2;HAS2;C10ORF2;GPC5;MPZL3;FAM46C;PCD
R4;MAGEE1;ST8SIA3;SLC4A10;KLHL23;SMARCA1;GRIN2B;C12ORF74;ESR1;SYT7;PHOX2B;REEP1;C
TRAK2;NXF1;TMEM65;TMEM203;MAT2A;HAS2;RPP14;EMC7;TRIM44;SEPT11;TIMMDC1;PTPN11;SYNJ
P2C;SNPH;MAPRE3;COL6A5;ST6GALNAC3;SLC29A3;AMER1;USP15;PPM1E;GBX2;TMEM68;USP1;HA
JSP13;HDAC5;ROCK1;PLAG1;TUBD1;ZDHHC21;GRAMD4;LRP6;PURA;ZNF629;TMEM203;UBN2;YLPM
IARCA1;BTBD9;SYT7;REEP1;BFSP2;GLUD2;OCLN;ARHGAP31;SESTD1;REN;LHX4;GABRB1;CHURC1-
JPH1;ELAVL2;ADAMTS5;SDR16C5;MED14;GRM5;ADAMTS2;TRPS1;RSPO2;TMEM108;EMB;GPRASP2;
;LUZP1;ATL2;SRSF1;PPM1K;MAT2B;PDHB;OGFRL1;PGRMC1;TMEM203;ATXN1L;PCBD2;MPZL3;KCNIF
DAC5;SEMA3A;UQCR10;FAM117B;PAPSS2;TRAK2;TMEM203;RBBP5;TDG;TMEM68;NT5DC3;HAS2;C10
3;FAM84A;ST6GALNAC3;ERGIC2;SLC29A3;RNF165;AMER1;PURA;GBX2;TMEM203;TMEM68;HAS2;LRI
3;SORCS1;IL17RD;CDYL2;RAP2C;FAM84A;SMTNL2;ST6GALNAC3;ERGIC2;DCP2;AMER1;MYRF;ZDHH
SREK1IP1;LNPEP;TRAPPC8;PARVA;BTBD9;SYT7;RCAN1;ZZZ3;LSM14B;DNAL1;PLEKHM3;LRP12;RAD
FIA;SP1;CAPRIN1;PPP1R1B;CTNNB1;BMPR1A
65;NAPA;NUFIP2;HPGD;SEMA3A;PRICKLE1;TTL;JAKMIP2;TRAK2;NEURL1B;ZNRF2;ZNF629;IGF2BP1;I
2;PLAG1;FAM129A;XIAP;PURA;NXF1;BCLAF1;SERTAD2;RBBP5;MGAT5;NSD1;TMEM68;USP1;PCBD2;F
R10;TRAK2;C21ORF119;AP3M2;GNA13;ZNRF3;TMEM203;RBBP5;TMEM68;C10ORF2;PCBD2;MPZL3;TR
IF236;ATL3;PALM2;ZDHHC21;MAT2B;PPM1E;GRAMD4;COL19A1;NXF1;ZNF629;TRA2B;IGF2BP1;SLIT2
OL6A5;SLC25A12;PRTFDC1;SF3B3;ATL3;PPM1L;HPGD;TUBD1;DRP2;TDG;TMEM68;S1PR1;C10ORF2;M
3;FAM84A;CRK;MAPRE2;TMEM106A;PAFAH1B2;CHST3;FYTTD1;LUZP1;ATL2;PRICKLE1;CYTH3;PPP2C
MYOD1;NOS1AP;COL6A5;NAT8L;CHST3;HPGD;PALM2;PRUNE2;FAM129A;NDNF;PRICKLE1;PPM1E;C
;EMB;PRKG1;GPR37;GAB3;SORCS1;ADRA2B;ADRA2A;MYOD1;COL6A5;SMTNL2;MAPRE2;RNF165;PA
;LRP6;OGFRL1;ZDHHC18;EFS;ZNF629;TMEM203;NT5DC3;SRSF10;SMNDC1;SEPT10;FAM46C;CNBP;T

SF1;TUBD1;ZNRF3;MGAT5;TMEM68;USP1;FAM63B;VPS54;RPP14;MBNL3;S100PBP;MAP3K2;TRIM41;
TYRP1;LRP6;C21ORF119;CYTH3;OGFRL1;BCLAF1;TNKS2;RBBP5;TRA2B;SLIT2;EIF4E;TRIM44;MAP3K
)1;PURA;PGRMC1;TMEM203;MAT2A;MGAT5;UBN2;TMEM68;USP1;VPS54;LRIG2;MBNL3;MPZL3;S100F
MER1;NAPB;USP15;ROCK1;TUBD1;ZDHHC21;PURA;MGAT5;ATXN1L;USP1;VPS54;TRIM44;MAP3K2;T

68;HAS2;VPS54;LRIG2;RPP14;EMC7;TRIM44;RRM1;GATC;PTPN11;SYNJ2BP;GTF2H5;NECAP2;MARC
M1E;NEURL1B;TMEM68;ATXN1L;VPS54;APBB2;LRIG2;MBNL3;MPZL3;MAP3K2;CSNK1A1;TXNL1;SUS
UBD1;UQCR10;TRAK2;PURA;OGFRL1;TMEM203;TNKS2;FAM63B;TRIM44;SLC25A24;SEPT11;SORT1;S
;RBBP5;UBN2;IGF2BP1;C10ORF2;LRIG2;RPP14;ATOH1;S100PBP;TRIM41;B3GAT2;YLPM1;LAPTM5;TM
44;UQCR10;TRAK2;ZNF629;TMEM203;TDG;UBN2;C10ORF2;VPS54;MPZL3;TRIM44;GATC;PDAP1;GTF
1106A;PRTFDC1;HDAC5;TUBD1;COL19A1;LRP6;C21ORF119;DRP2;PPP2CA;TMEM203;LRIG2;MPZL3;C
A3A;UQCR10;TRAK2;GBX2;SEPT10;TIMMDC1;RANBP3;GATC;SOD2;GTF2H5;TTC9;FAM167A;ZNF70;G
IYPN;PITPNC1;ADAMTS2;SPRED1;SH3BGRL2;KIF1B;CTBS;GPR135;TMED8;NSUN3;SORD;PROX1;CD
;ALDH2;SLC22A17;RSPO2;TMEM108;SCN5A;PRKG1;MME;GRID1;GAB3;SORCS1;IL17RD;CDYL2;ADR
B3;GNL1;FAM101B;RAP2A;CREB1;FCHSD2;RRM2B;NAA35;SLC25A12;ERGIC2;RAPGEF6;DTL;TMEM1
PURA;GLTSCR1L;RANBP3;CRBN;USP9X;SPRYD7;SUSD1;YLPM1;UBE2G1;KCNRG;ASPRV1;SORBS3;
HOX2B;OCLN;EFNA3;RCAN2;PPP1R1B;LHX6;HRK;GABRB1;ONECUT2;KCNC2;TTC22;CELF3;DIRAS2;
21ORF119;TMEM203;RBBP5;TDG;UBN2;S1PR1;C10ORF2;PCBD2;MPZL3;PCDHB15;GATC;GTF2H1;TS
1;APBB2;PCBD2;CA12;SEPT10;TRIM41;TIMMDC1;FAM46C;GATC;PDAP1;GTF2H1;ADAT2;LETM1;C20O
SP1;FAM63B;VPS54;LRIG2;RPP14;MPZL3;SLC25A24;MAP3K2;TRIM41;GATC;PDAP1;TMEM64;SYNJ2
H3PXD2A;TRPS1;PSD3;PGM3;FAM73A;TMEM108;EMB;CTBS;TEAD1;GPR135;ARHGEF10;GPR37;MM
RN;C11ORF57

HB15;PDAP1;LNPEP;SMARCA1;SYNJ2BP;LSM5;C12ORF74;FBXO30;TSHR;STXBP5L;ITCH;DIAPH2;AR
USD1;MRPL27;ZBTB10;GATC;NBEAL1;TRAPPC8;HELZ;ADAT2;LETM1;CLCN5;MARCKS;HNRNPK;ZZZ
EK1IP1;LNPEP;BTBD9;GTF2H5;AZIN1;SULF2;PARP11;ITCH;PDP2;KLHL7;FUBP1;JMY;ID4;LHX6;TRIP12
RD5A1;PCDHB15;GATC;PDAP1;LNPEP;TRAPPC8;SORBS3;U2SURP;ADAT2;LETM1;TTC9;ALDH6A1;EF
N;SMURF2;GATC;PDAP1;KLHL23;TRAPPC8;PTPN11;L1CAM;SOD2;SYT7;PHOX2B;RAB11A;SULF2;PIT
)5A1;ST13;SPRYD7;PCGF3;GATC;PDAP1;KLHL23;FBXO30;ADAT2;LETM1;RCAN1;C7ORF60;XK;FRAS1
5;TAC3;RIMKLA;MBNL3;LYN;GABRA1;CBX6;PRRX1;FAM78A;KCNIP2;ST8SIA3;SLC4A10;KLHL23;GRIN
CBX6;TRIM41;RANBP3;CBX5;MRPL27;GATC;PDAP1;LNPEP;PARVA;TMEM64;SYNJ2BP;NECAP2;ARHG
11;ZZZ3;SP1;SESTD1;STRN;CALM1;MXD1;UBE2K;TECPR2;DNAL1;PLEKHM3;RAD9A;TNFAIP8;ANKRD
8;SREK1IP1;LNPEP;HELZ;BTBD9;FBXO30;REEP1;RCAN1;ARHGAP31;SP1;ID2;LHX6;TECPR2;PLEKH
IP1;ESR1;ADAT2;TPCN1;LETM1;MAPK10;PARP11;ALDH6A1;XK;DPY19L2;NFIA;TMEM56;KIF26B;RCA
7;EIF2B2;SRD5A1;PCGF3;YLPM1;KLK13;PCDHB15;NFATC3;GATC;BTBD9;HEYL;XK;HNRNPK;VAPA;C
RCC5;SORT1;KCNIP2;GATC;NBEAL1;RASSF8;PDAP1;SREK1IP1;SOD2;SYNJ2BP;SORL1;HEYL;XK;NF
TBD9;GATAD2B;RCAN1;PARP11;ZNF70;PDP2;VAPA;APC;ID2;KLHL7;OSTC;CAMK4;RPA4;JMY;ASB7;CT
T7;TSHR;STXBP5L;TPCN1;SMAD7;TTC9;FAM167A;SESTD1;CARM1;RCAN2;CTNNB1;FAM171B;LHX4;L
46C;TXNL1;PCGF3;GATC;HIPK1;LETM1;HEYL;RCAN1;ITCH;CLCN5;ARHGAP31;XK;DPY19L2;VAPA;VA
TS2;JPH3;MECOM;TRPS1;TEAD1;GPR135;ARHGEF10;GPR37;MME;GRID1;MMP2;GAB1;PAX5;DYNLL
TRIM44;ATOH1;PCDHA7;MARK1;SEPT10;SEPT11;EGR4;CSNK1A1;SUSD1;LAPTM5;SLC4A10;TRAPPC
;CCZ1B;FZD5;XRCC5;GATC;RASSF8;LNPEP;HELZ;SMARCA1;ADAT2;ZNF70;XK;ZZZ3;RFWD3;PPP1R
L27;ATP2B4;GATC;SREK1IP1;GTF2H1;PARVA;KCNRG;SYNJ2BP;GATAD2B;GTF2H5;PARP11;XK;GPAM
3GALNT2;CREBBP;RABGAP1L;CBX6;SEPT12;RASSF8;PDAP1;SREK1IP1;TRAPPC8;REEP1;HEYL;ARH
NK1A1;MRPL27;FOXJ2;SREK1IP1;TMEM64;LSM5;ADAT2;TPCN1;LETM1;PARP11;APC;PPP1R1B;RAB3
2;ADAMTS5;SH3PXD2A;ALDH2;SLC22A17;PSD4;TRPS1;FAM73A;RSPO2;SH3BGRL2;METTL16;PHACT
L1CAM;SYT7;ADAT2;OCLN;FAM167A;PDP2;TMEM56;PPP1R1B;TCEA3;CDK1;LHX6;FAM171B;MXD1;N
ELZ;GDF6;STXBP5L;REEP1;XK;DPY19L2;RFWD3;ASXL3;CAMK4;PPP1R1B;SPRY3;LHX6;PDE7B;PLEK
186A;CPSF7;TIMMDC1;ST13;NBEAL1;RASSF8;PDAP1;HELZ;TMEM64;GTF2H5;LETM1;RCAN1;ZNF70;F
SD1;FBXL17;SREK1IP1;CDC42BPA;ESR1;SYT7;RAB11A;AZIN1;LETM1;PARP11;HNRNPK;KLHL7;FAM1
M2;RCAN1;FAM167A;ZNF70;ZZZ3;GPAM;PITHD1;RFWD3;CAMK4;EIF3J;CCNYL1;ASB7;STRN;CALM1;
3;SULF2;RCAN1;MARCKS;PITHD1;ID2;SERBP1;CCDC6;CALM1;TARDBP;SERINC3;TECPR2;LNX2;RAD
7;MRPL27;PLEKHA3;PARVA;HELZ;TMEM64;MARCH7;LETM1;RCAN1;PARP11;ARHGAP31;PDP2;APC;K

;LNPEP;GTF2H1;TRAPPC8;BTBD9;SULF2;ADAT2;LETM1;HEYL;RCAN1;FAM167A;CDKN2AIPNL;GPAM
A17;PGM3;TMEM108;KIF1B;SCN5A;EMB;CTBS;TEAD1;CCDC50;APPL1;CCDC112;ARHGEF10;SORD;LI
ZBTB10;NFATC3;GATC;NBEAL1;SOD2;HIPK1;SYNJ2BP;SYT7;STXBP5L;CLCN5;EFNA3;ZNF70;HNRNPI
27;EPT1;GATC;PDAP1;HELZ;ADAT2;ZNF70;GPAM;PQLC1;NFIA;SP4;CAPZA1;CCNYL1;STRN;TECPR2;D
5;MPZL3;TIMMDC1;SORT1;SPRYD7;PCDHB15;GATC;MTSS1L;GTF2H5;STXBP5L;LETM1;XK;GPAM;ET

C29A3;NAPA;ROCK1;PLAG1;FAM129A;TUBD1;ZBTB44;PPM1K;UQCR10;PAPSS2;LRP6;PURA;OGFRL1
2;TIPARP;TXNL1;GTF2H1;DEK;MARCKS;NFIA;VAPA;VAPB;PITHD1;ETNK1;ASXL3;CAPRIN1;OCIAD1;SP
B2;C1ORF21;PARVA;SYT7;TSHR;OCLN;ARHGAP31;XK;PDP2;ASXL3;EIF3J;LHX6;STRN;SERINC3;DAN
PLEKHA3;GTF2H1;S100B;BTBD9;SORL1;MAPK10;NECAP2;FAM167A;GPAM;KIAA0101;ID2;KIF26B;SPR
2;MCTS1;EIF2B2;CBX5;CRBN;LAPTM5;SREK1IP1;S100B;ESR1;SYT7;AZIN1;TPCN1;PARP11;ALDH6A1;
9;STXBP5L;ABHD15;TPCN1;LETM1;PARP11;ALDH6A1;NCEH1;APC;TMEM56;RCAN2;PPP1R1B;TCEA3;
K;NRP2;DYRK3;MOCS3;ONECUT2;KCNC2;PRR3;SNAP23;ADK;DUSP19;PPWD1;RCSD1;SMG7;MED17;
ART3;SERP1;MECOM;SLC22A17;MFSD5;RSPO2;TMEM108;SOSTDC1;PHACTR4;CCDC112;TSFM;PCY
3P;ADAT2;TPCN1;LETM1;PARP11;HNRNPK;APC;ID4;NABP1;LSM14B;MXD1;NUPL1;PLEKHM3;RAD9A
;GATC;SREK1IP1;UBE2G1;L1CAM;SYT7;PDP2;PQLC1;SESTD1;KIF26B;TCEA3;FAM171B;LRP12;CC2D
A10;SMAD9;KLHL23;NSG1;L1CAM;MAPK10;OCLN;DPY19L2;PDP2;KIF26B;RCAN2;ASXL3;CAMK4;PDE
015;TTC9;CLCN5;ZNF70;HNRNPK;CDKN2AIPNL;ID2;RCAN2;LHX6;SPRY1;MXD1;NFE2L1;RAD9A;TNRC
ZD5;FAM78A;SORT1;B3GAT2;PLEKHA3;TRAPPC8;L1CAM;SOD2;ITCH;ID2;TCEA3;TARDBP
NL;ZFP62;PITHD1;OSTC;CAPZA1;OCIAD1;NABP1;HNRNPC;PTPN4;TARDBP;TECPR2;NUPL1;LRP12;R

A1;BTBD9;SULF2;FAM167A;ZNF70;GPAM;KIF26B;RFWD3;CAPZA1;FRK;DNAL1;LNX2;RAD9A;TNRC6B
1;REEP1;ZZZ3;SP1;CAMK4;EIF3J;CCNYL1;SPRY1;TARDBP;MXD1
AT2;PUM2;LETM1;FAM167A;XK;RFWD3;ASXL3;CAPZA1;TCEA3;SPRY3;ASB7;MXD1;RAD9A;TNRC6B
8SIA3;S100B;BTBD9;SYT7;TSHR;HIPK2;SULF2;REEP1;TTC9;OCLN;ID2;ID4;LHX4;CD244;LNX2
BS3;SYT7;HEYL;GLUD2;FAM167A;XK;DPY19L2;RFWD3;ASXL3;EIF3J;TCEA3;SPRY3;ASB7;YPEL2;RAD
9;GLUD2;FAM167A;DPY19L2;GPAM;RFWD3;ASXL3;CAPZA1;CAMK4;CCDC6;SPRY3;FRK;YPEL2;RAD9
C8;HELZ;U2SURP;ADAT2;LETM1;ZNF70;XK;DPY19L2;ZZZ3;RFWD3;CAMK4;TCEA3;CCDC6;PLEKHM3;
PRE2;SLC24A2;HPGD;NRXN3;NDNF;PPM1E;TTL;PAPSS2;JAKMIP2;ERBB4;HAS2;SLC17A6;ATOH1;GA
APPC8;SYT7;ADAT2;ITCH;SESTD1;ID2;CAPZA1;ASB7;STRN;TECPR2;DNAL1;LRP12;RAD9A
2SURP;AZIN1;MARCKS;GPAM;SP1;ZFP62;KIF26B;CAPZA1;FAM171B;DAND5;PLEKHM3;LRP12;TNRC6
;SELE;SULF2;ADAT2;REEP1;GLUD2;DPY19L2;CAMK4;EIF3J;TCEA3;CCDC6;LNX2;FGFR1
AD7;HEYL;TTC9;HNRNPK;GPAM;EIF3J;CCDC6;LHX6;LSM14B;TECPR2;FRK;DNAL1;PLEKHM3;RAD9A;
;GTF2H5;OCLN;CAPZA1;CCDC6;NABP1;MXD1;SSBP2;TECPR2;NUPL1;PLEKHM3;LNX2;TNRC6B;CC2D

DH6A1;KIAA0101;SP1;PITHD1;RFWD3;ASB7;STRN;CALM1;LSM14B;TECPR2;DNAL1;PLEKHM3;RAD9A
EM86A;EGR4;PLEKHA3;C12ORF74;SULF2;REEP1;OCLN;FAM167A;MYO1C;GPAM;KIAA0101;ID2;RCAN
1;FUBP1;CAPZA1;CAPRIN1;CCNYL1;TRIP12;HNRNPC;CALM1;TARDBP;MXD1
9L2;GPAM;KIAA0101;KIF26B;ASXL3;CAMK4;PPP1R1B;CCDC6;SPRY3;LHX6;FRK;RAD9A;TNRC6B
EYL;GLUD2;XK;RFWD3;SPRY3;ASB7;TECPR2;LRP12;YPEL2
NECAP2;FAM167A;MYO1C;PDP2;KIF26B;RCAN2;FAM171B;CD244;DAND5;FGFR1
1;NECAP2;CLCN5;DIAPH2;FAM167A;NCEH1;PDP2;GPAM;APC;RLIM;ASB7;NUPL1;DAND5
LZ;TMEM64;FBXO30;TTC9;DIAPH2;ARHGAP31;SP1;KIF26B;TRIP12;TARDBP;C11ORF57;TNRC6B
VD3;ASXL3;EIF3J;CCDC6;SPRY3;ASB7;LHX6;DAND5;YPEL2;RAD9A;TNRC6B
7;SCN5A;TEAD1;MME;CBFA2T2;GNL1;ADRA2B;SUMF1;MAPRE3;DTL;SOS2;PHLPP2;USP13;PDHB;UQ
N;MXD1;TECPR2;NFE2L1;DAND5;LRP12;RAD9A;CC2D1B;BMPR1A;ALAS2;DYRK3;BMPR2;ANKRD33B;
5A1;MRPL27;GATC;SREK1IP1;PARVA;ADAT2;MARCKS;ARHGAP31;MYO1C;GPAM;PPP1R1B;DNAL1;PL
6B;RCAN2;LHX6;FAM171B;SERINC3;NUPL1;FRK;DAND5
2;ADAT2;LETM1;EFNA3;XK;DPY19L2;ZZZ3;KIAA0101;CAMK4;CCDC6;SPRY3;LHX6;PLEKHM3;RAD9A
PT1;SPRYD7;PCGF3;GATC;SREK1IP1;TRAPPC8;SOD2;GTF2H5;SMAD7;GPAM;RFWD3;RAB3GAP2;LSM
1;MARCKS;ZNF70;DPY19L2;ZZZ3;ASXL3;CAPZA1;CAMK4;EIF3J;RAB3GAP2;DNAL1;RAD9A
AM;ID2;CCDC6;ASB7;FAM171B;TRIP12;TECPR2;PLEKHM3;RAD9A;TNRC6B
1IP1;LNPEP;ADAT2;GLUD2;XK;DPY19L2;CAMK4;CCDC6;MXD1;TECPR2;RAD9A;TNRC6B

F3J;CCDC6;CCNYL1;FRK;DNAL1;PLEKHM3;RAD9A
?A;RAB11A;PUM2;HNRNPK;VAPB;RFWD3;CAPZA1;SERBP1;RAB3GAP2;TNRC6B
)X2B;STXBP5L;SULF2;OCLN;EFNA3;XK;FRAS1;SPRY1;ITM2C
HBA;GRIN2B;SORBS3;TSHR;SULF2;ARHGAP31;MYO1C;DPY19L2;KIF26B;PPP1R1B;JMY;RAD9A
SLC4A10;ATP2B2;GRIN2B;SORBS3;PHOX2B;TSHR;OCLN;MYO1C;XK;CAMK4;TCEA3;ID4;SPRY1;FGFI
RP;ADAT2;ALDH6A1;SERBP1;EIF3J;HNRNPC;PDE7B;TECPR2;PLEKHM3;C11ORF57
.2;KIF26B;PDCD4;LHX6;FAM171B;SPRY1;LHX4
TTC9;EFNA3;XK;DPY19L2;GPAM;CCDC6;RAD9A;TNRC6B;ITM2C
)STC;CARM1;RPA4;CCNYL1;ASB7;RAD9A;CDS2;CC2D1B
)IAPH2;MYO1C;PDP2;RCAN2;OCIAD1;SPRY3;FRK;DAND5
26B;RFWD3;CAPZA1;CCNYL1;TECPR2;DNAL1;PLEKHM3;RAD9A
;PLEKHA3;GTF2H1;LSM5;SORBS3;TSHR;LETM1;REEP1;ASB7
CDC6;LHX4;SLC28A3
42BPA;RAB11A;TPCN1;PARP11;DPY19L2;CAPZA1;PPP1R1B;LHX6;FAM171B;MXD1
I167A;XK;DPY19L2;GPAM;ASXL3;EIF3J;SPRY3;ASB7;LHX6;TRIP12;RAD9A
RT3;SIPA1L1;SH3PXD2A;ALDH2;TRPS1;TRIM3;FAM73A;RSPO2;SH3BGRL2;METTL16;CCDC112;UTP1!

1A;LETM1;PDP2;ETNK1;TRIP12;SPRY1;SERINC3;TECPR2;CC2D1B
RPA4;OCIAD1;RAB3GAP2;TARDBP;UBE2K;TECPR2;NFE2L1
3;PDP2;CDKN2AIPNL;SPRY3;TECPR2;DNAL1;RAD9A
L1;SUSD1;LGI2;LAPTM5;PARVA;DEK;SULF2;ETNK1;RAB3GAP2;LHX4;LSM14B;NUPL1
A;PITHD1;FUBP1;CAMK4;SERINC3;UBE2K;FRK;LRP12;C11ORF57
N;GPAM;LHX4;MXD1;UBE2K;PLEKHM3
SPRY3;RAB3GAP2;MXD1;TECPR2
1;STRN;FAM171B;MXD1;TECPR2;PLEKHM3
;TSHR;RCAN2;CAPZA1;LHX6;PTPN4;NUPL1;PLEKHM3
TC
)CIAD1;RLIM;RAB3GAP2;LSM14B;LNX2
GFR1
N;XK;FRAS1;ASXL3;CAMK4;ID4;FRK
B;PTPN4;DNAL1;PLEKHM3;LRP12;RAD9A
RLIM;RAB3GAP2;LSM14B;LNX2
;CDK1;FAM171B;LHX4
R135;VKORC1;TTC33;MSL2;SORD;LRRC40;HAUS3;ADRA2B;DIEXF;PTP4A1;CREB1;TET3;PGAM5;NA
P2;NUPL1;NFE2L1;TNRC6B
(4;PPP1R1B;TECPR2;RAD9A

6B;LHX4;CC2D1B
)BP;LSM14B;NUPL1;TNRC6B;CDS2
4;SLC28A3
)6A;SLC29A3;MYRF;ATL3;ZDHHC21;PPM1K;ADD3;PPM1E;PAPSS2;SLIT2;MPZL3;EIF4E;CRBN;SORT1;
AM;ID2;SPRY3;TECPR2
;RAD9A

A3;RAD9A
R1

B
NX2
);PURA;TMEM203;MAT2A;PABPN1;E2F1;C10ORF2;GPC5;BCL2L2-PABPN1;RPP14;CNBP;TXNL1;GATC;

TET3;PGAM5;ST6GALNAC3;SLC25A12;HDAC5;ATL3;PPM1L;ATL2;UQCR10;PPM1E;GRAMD4;FAM117B
9;C1ORF213;PPP1R9A;JPH1;CDC73;PHF8;UBL3;NKRF;MAN1A2;MFSD5;TRIM3;TMEM108;SH3BGRL2;
GEF5;LUZP1;PRUNE2;NDNF;PPM1K;HOXD12;PPM1E;TTL;COL19A1;PAPSS2;EFS;TMEM65;NSD1;ATO

DS2
1QBP;FAM154B;FAM73A;CEP170;RBM7;CTBS;UNKL;CNPPD1;KLF10;MAPK1IP1L;TSFM;USP47;PCYT1

ARP11;NECAP2;OCLN;EFNA3;NCEH1;PDP2;TMEM56;KLHL7;TCEA3;FAM171B;TARDBP;PLEKHM3;CDS

I2C;CELF1;PHF20;SH3KBP1;SNAP23;JPH1;PITPNC1;IL18BP;ALAD;ING4;SLC22A17;PSD3;SOSTDC1;S
REB1;FCHSD2;TET3;LCOR;PGAM5;ST6GALNAC3;SLC25A12;SOS2;ATL3;ATL2;UQCR10;FAM117B;CYTI

APZA1;RPA4;OCIAD1;RLIM;RAB3GAP2;SPRY1;TARDBP;LSM14B;LNX2
3;C3ORF62;MSL2;TFEB;DIEXF;ENAH;PTP4A1;FAM101B;RBL1;FCHSD2;TET3;MAPRE3;SLC25A12;TME

A1;SPRYD7;TMEM64;DEK;SORL1;TSHR;STXBP5L;ABHD15;TPCN1;REEP1;BFSP2;OCLN;EFNA3;FAM
0ORF2;BCL2L2-PABPN1;TRIM44;S100PBP;SLC25A24;TRIM41;CSNK1A1;KCNIP2;CNBP;GATC;GTF2H1
SL2;ADAMTS2;MECOM;PRKG1;PCYT1B;GPR37;TMED8;GRID1;SGIP1;DNMT3A;SORCS1;PAX5;PROX1

;LNX2;RAD9A;BMPR1A

HSD2;CNKSR3;TET3;LCOR;PGAM5;PDE5A;SLC25A12;HDAC5;DLST;UQCR10;AP3M2;PPP2CA;NXF1;M

5;HEYL;EFNA3;CDKN2AIPNL;GPAM;NFIA;TMEM56;KLHL7;CTNNB1;FAM171B;SPRY1;LHX4;PDE7B;FR
RHGAP31;XK;FRAS1;KIF26B;RLIM;LHX6;LHX4;ITM2C

6;NUPL1;TNRC6B
NLL2;ZFX;RAP2C;SNPH;FAM84A;CLDN18;NAA38;NAT8L;SLC24A2;USP13;HPGD;NRXN3;GRAMD4;COL
ED17;IL18BP;ING4;JPH3;SLC22A17;MFSD5;SOSTDC1;EMB;VKORC1;ARHGEF10;GPR37;TMED8;NSUN
BE2K;RAD9A
1;RPA4;TCEA3;ID4;HNRNPC;TARDBP;LSM14B;C11ORF57;RAD9A

MI1;SMG7;AFF4;PFAS;AFF1;IL18BP;ING4;RBM3;SART3;SIPA1L1;SH3PXD2A;PSD4;FAM154B;PSD3;UB
2;UBL7;CCDC112;UTP15;ARHGEF10;WDR37;TTC33;SGIP1;ANO6;ADRA2B;SUMF1;FAM101B;TMEM10
;NEK7;GFPT1;MRPL27;GATC;PLEKHA3;PARVA;BFAR;KCNRG;VAPA;VAPB;SP4;OSTC;FUBP1;OCIAD1;

7ORF60;ZNF70;DPY19L2;FRAS1;PDP2;GPAM;PITHD1;ID2;FUBP1;TECPR2;NUPL1;DNAL1;PLEKHM3;L
28A3

:D1;C1QBP;UBXN7;CEP170;ARL5A;UNKL;APPL1;DGCR2;MAPK1IP1L;ARHGEF10;USP47;DFFA;NAA30
XN7;CEP170;ARL5A;CCDC50;UBL7;UTP15;USP47;TMED8;VPS13C;SORD;LRRC40;PROX1;CBFA2T2;S
;PTPRM;SIX1;RCSD1;BMI1;JPH1;ADAMTS5;NHSL2;ADAMTS2;JPH3;MECOM;SLC22A17;RSPO2;TMEM
)2A;SLC22A17;TRPS1;TMEM108;EMB;PRKG1;GPR135;CCDC112;HS3ST3B1;ZCCHC24;GPR37;GRID1;
AUS3;SENP2;GNL1;RBL1;RRM2B;TERF2IP;CD47;FYTTD1;USP13;RALA;PPM1L;ROCK1;ZDHHC20;PALM

IF;HOXD12;PPM1E;ADD2;JAKMIP2;EFS;GBX2;IGF2BP1;S1PR1;HAS2;GPC5;S1PR3;ATOH1;PRRX1;TPE
3BGRL2;SCN5A;PRKG1;GPR135;HS3ST3B1;GRID1;GAB1;PAX5;PROX1;IL17RD;ADRA2B;PAX2;ADRA2
2;TMEM108;SCN5A;PRKG1;MME;GRID1;GAB3;SORCS1;PAX5;PROX1;ADRA2A;NRG3;MYOD1;NOS1A
KHA3;TMEM64;HIPK1;TSHR;TTC9;FRAS1;NFIA;KIF26B;ASXL3;YPEL2;TNRC6B
R9A;JPH1;PITPNC1;ELAVL2;PHF8;NHSL2;EEF2K;SLC22A17;TMEM108;SH3BGRL2;KIF1B;CEP170;SCN
A2A;ENAH;TGFBR3;MYOD1;NOS1AP;RBMS1;PDE5A;COL6A5;ARHGEF5;CHST3;RNF165;SLC24A2;USF
M2;NRXN3;ZDHHC21;ZBTB44;LRP8;FAM117B;COL19A1;ZNRF3;ZNF629;UBN2;C10ORF2;VPS54;SLIT2;
;PRRX1;TPBG;TBX5;SULF2;FRAS1;TCEA3
A14;PTPRM;PPP1R9A;JPH1;ELAVL2;ADAMTS5;MED14;GRM5;ADAMTS2;MB;TRPS1;RSPO2;TMEM108
117B;LRP6;PURA;ZNRF2;RBBP5;MGAT5;TDG;TMEM68;NRIP1;USP1;VPS54;MAPK1;PCBD2;LRIG2;RP
;PDE5A;MAPRE2;PRTFDC1;FYTTD1;PPM1H;NRXN3;DLST;ZDHHC21;ZBTB44;PPM1E;FAM117B;PAPSS
SH3BGRL2;ARL5A;TEAD1;CCDC50;CCDC112;ZCCHC24;NAA30;TMED8;GRID1;MMP2;ITPK1;ZFX;CDYL
70;EMB;CCDC50;PRKG1;GPR135;HS3ST3B1;CHST7;ZFX;CDYL2;KAT2B;SNPH;NOS1AP;ST6GALNAC3
IRK;GPR27;NRP2;ANKRD33B;TENM3;ONECUT2;KCNC2;PDE3B;SLC7A14;PTPRM;SIX1;FMN1;JPH1;RN
3;SLC25A12;ERGIC2;CRK;SLC29A3;NAPA;PHLPP2;AMER1;NAPB;ZDHHC20;PALM2;ZDHHC21;ZBTB44
7;GAB3;CDYL2;PTP4A1;KAT2B;RAP2A;RRM2B;IL1RAPL1;NOS1AP;NAT8L;MAPRE2;CHST3;FYTTD1;PF
6;EFNA3;ZFP62;SESTD1;OSTC;FUBP1;MXD1;DNAL1;LRP12;RAD9A;ITM2C;CC2D1B;BMPR1A;RBM28;I
LNAC3;ARHGEF5;CHST3;LUZP1;PLAG1;PPM1H;NDNF;HOXD12;LRP8;COL19A1;PAPSS2;JAKMIP2;ZD
GAP31;NFIA;CAPRIN1;CCDC6;ID4;CTNNB1;LHX4;TARDBP;BMPR1A
ST3B1;GPR37;CHST7;ZFX;CDYL2;PTP4A1;KAT2B;RBL1;IL1RAPL1;NOS1AP;PAFAH1B2;CHST3;FYTTD
LUZP1;ZDHHC20;PALM2;PRUNE2;FAM129A;DLST;TUBD1;PPM1K;PURA;OGFRL1;DFNB59;ZNRF3;RBE
;RBMS1;MAPRE3;LPHN2;CHST3;FYTTD1;ZDHHC21;ZBTB44;FAM117B;ZNRF2;DFNB59;ZNRF3;ZNF62
;PPP1R1B;JMY;LHX6;LHX4;FGFR1;NRP2;BMPR2;DIRAS2;DUSP19;TNFAIP3;SIX1;CTDSPL2;BMI1;PPP1
2B;RAP2A;IL1RAPL1;NOS1AP;LPHN2;MAPRE2;PRTFDC1;CHST3;FYTTD1;PPM1L;SEMA3A;ATL2;DLST
OS1AP;RBMS1;NAT8L;LPHN2;MAPRE2;CHST3;FYTTD1;ZDHHC21;ZBTB44;LRP8;FAM117B;DFNB59;EFS
NAPA;AMER1;ZDHHC20;ZDHHC21;NXF1;ZNRF2;MGAT5;TDG;TMEM68;CARNS1;USP1;FAM63B;VPS54

;STAU1;ROCK1;NUFIP2;PPP2CA;ZNRF2;PGRMC1;TMEM203;MAT2A;TNKS2;MGAT5;TMEM68;USP1;PT
;UBN2;HAS2;VPS54;MBNL3;MPZL3;JAM2;ZNF462;B3GAT2;PCDHB15;PTPN11;TMEM64;HIPK1;RAB11A
5;PROX1;ADRA2A;NRG3;MYOD1;NOS1AP;FAM84A;MAPRE2;RNF165;PPM1L;DCTN2;HPGD;PALM2;PF
1;ZDHHC21;PDHB;NXF1;BCLAF1;TMEM65;TMEM203;TNKS2;RBBP5;TMEM68;SRSF10;TRIM44;GALNT
;IL1RAPL1;NOS1AP;FYTTD1;PPM1L;DLST;ZDHHC21;ZBTB44;FAM117B;EFS;GBX2;ZNF629;MAT2A;UBN
NPH;IL1RAPL1;NOS1AP;NAT8L;PRTFDC1;FYTTD1;PALM2;ZBTB44;FAM117B;DFNB59;EFS;ZNF629;MA
OX1;ZFX;CDYL2;KAT2B;RAP2A;NOS1AP;LPHN2;FYTTD1;SEMA3A;PDHB;FAM117B;DFNB59;ZNF629;M
R10;LRP6;GNA13;PGRMC1;TMEM65;TMEM203;TMEM68;LRIG2;EMC7;SMNDC1;GALNT7;CRBN;CNBP
R3;MBNL3;MPZL3;CA12;TTC9;C20ORF112;DPY19L2;NFIA;ASXL3;CAMK4;PTPN4;C11ORF57;TNRC6B
3;FYTTD1;HPGD;ATL2;ZBTB44;MAT2B;PPM1E;FAM117B;DFNB59;TMEM65;MAT2A;UBN2;MAPK1;PCBD
ST;TUBD1;ZNF629;MAT2A;TNKS2;RBBP5;TDG;UBN2;USP1;MPZL3;SRSF10;TRIM44;FOXJ2;GATC;PDA
;PDHB;ADD3;C21ORF119;OGFRL1;BCLAF1;RBBP5;TDG;UBN2;TMEM68;S1PR1;C10ORF2;PCBD2;SLIT
SEMA3A;ZDHHC21;UQCR10;NEURL1B;PURA;ZNRF3;EFS;TMEM203;SERTAD2;TDG;S1PR1;VPS54;MP
4;BMPR1A;FGFR1;GPR27;NRP2;BMPR2;SNAP23;DUSP19;TNFAIP3;FMN1;CTDSPL2;PPP1R9A;SMG7;
P;LCOR;RBMS1;NAT8L;PAFAH1B2;PRTFDC1;FYTTD1;PPM1L;HPGD;SRSF1;FAM129A;ZBTB44;FAM117
I;COL6A5;SLC24A2;USP13;PRUNE2;FAM129A;HOXD12;COL19A1;ADD2;NEURL1B;EFS;GBX2;IGF2BP1
ZDHHC21;ZBTB44;FAM117B;COL19A1;PAPSS2;GBX2;ZNF629;MAT2A;UBN2;HAS2;VPS54;MAPK1;MBN
;GPR27;DOCK5;GABRB1;KCNC2;DIRAS2;PDE3B;PTPRM;SIX1;BMI1;PPP1R9A;JPH1;MYPN;ELAVL2;SI
KF1;ZNRF2;TMEM203;MGAT5;TMEM68;USP1;VPS54;LRIG2;MPZL3;TRIM41;CSNK1A1;TMEM64;ADAT2

IGEF5;KANK2;RALA;FAM129A;TUBD1;TRAK2;PPP2CA;NEURL1B;NXF1;TMEM65;TMEM203;MAT2A;TNI
;ZDHHC20;ZBTB44;PPM1E;PAPSS2;TMEM68;USP1;APBB2;MBNL3;MPZL3;JAM2;AAED1;SEPT11;CSNK
IEM64;HIPK1;HEYL;TTC9;XK;NFIA;KIF26B;CAPZA1;TARDBP;YPEL2;C11ORF57;PRDM8;OTUD4;RAB3C
SM14B;UBE2K;TECPR2;DNAL1;PLEKHM3;LRP12;RAD9A;DYRK3;TNFAIP8;MOCS3;TTC22;SH3KBP1;AD
DFNB59;ZNF629;UBN2;MAPK1;APBB2;PCBD2;SORT1;KCNIP2;MTSS1L;C1ORF21;HIPK1;HEYL;MARCK
FNB59;EFS;ZNF629;MAT2A;UBN2;HAS2;VPS54;MAPK1;PCBD2;SLIT2;LRIG2;MBNL3;MPZL3;JAM2;PCI
;PALM2;ZDHHC21;ZBTB44;FAM117B;DFNB59;EFS;ZNF629;MAT2A;UBN2;HAS2;VPS54;MAPK1;S1PR3;
OL6A5;SMTNL2;RNF165;HPGD;PALM2;PRUNE2;NDNF;PPM1E;COL19A1;ZNRF3;EFS;GBX2;SLC17A6;
RIM3;PGM3;CTBS;UNKL;PHACTR4;UTP15;ZCCHC24;MAPK1IP1L;TMED8;MME;TTC33;SGIP1;VPS13C;N
L1;PLEKHM3;LRP12;RAD9A;ITM2C;DIRAS2;PPWD1;TNFAIP3;BMI1;ELAVL2;ALAD;ING4;DTWD1;MED1
SS2;ADD2;JAKMIP2;GBX2;ERBB4;HAS2;SLC17A6;GPC5;MBNL3;B3GAT2;SUSD1;KLK13;SORL1;TSHR;
PDHB;LRP6;C21ORF119;EFS;ZNF629;TMEM203;UBN2;USP1;LRIG2;MPZL3;TRIM41;YLPM1;GATC;PDAI
EPT10;RRM1;TIMMDC1;TXNL1;PDAP1;PLEKHA3;SOD2;SORL1;U2SURP;GTF2H5;HIPK2;LETM1;NECA
GEF5;ZDHHC21;ZBTB44;TMEM68;ATXN1L;NRIP1;USP1;VPS54;MAPK1;LRIG2;RPP14;MPZL3;JAM2;TRI
1;PPM1L;HPGD;PRUNE2;FAM129A;NDNF;HOXD12;PPM1E;COL19A1;ERBB4;SLC17A6;SLIT2;MBNL3;J
A5;SMTNL2;MAPRE2;HPGD;PALM2;PPM1H;FAM129A;NDNF;PRICKLE1;HOXD12;GBX2;ERBB4;NRIP1;I
I;CYTH3;PPP2CA;TMEM203;TDG;MBNL3;MPZL3;SRSF10;RELT;TRIM44;RANBP3;CNBP;TXNL1;FOXJ2;
;RAP2C;FCHSD2;FAM84A;SMTNL2;ERGIC2;SLC29A3;CHST3;NAPA;AMER1;PLAG1;ZDHHC20;PALM2;I
TD1;ZDHHC21;ZBTB44;FAM117B;EFS;ZNF629;MAT2A;UBN2;HAS2;MAPK1;SLIT2;MPZL3;CA12;B3GAT
KIF26B;CAMK4;OTUD4;RAB3C;ATP8A2;TMEM167B;KLHL32;ZBTB20;LOXL4;RIMS3;CDH2;KIAA0895;SL
H1B2;CHST3;FYTTD1;SEMA3A;ATL2;ZDHHC21;ZBTB44;FAM117B;ZNF629;MAT2A;UBN2;PCBD2;RPP1
B;LHX6;PLEKHM3;RAD9A;ITM2C;BMPR1A;C9ORF91;NRP2;DYRK3;TNFAIP8;DIRAS2;DUSP19;PPWD1;
1ORF57;TNRC6B
;AMER1;MYRF;HPGD;PPM1E;EFS;VPS54;LRIG2;MBNL3;MPZL3;MAP3K2;FOXJ2;PLEKHA3;TPCN1;LET
LRIG2;BCL2L2-PABPN1;MPZL3;SRSF10;CNBP;TXNL1;FOXJ2;GATC;PTPN11;S100B;SYNJ2BP;NECAP2
RBBP5;TMEM68;NT5DC3;USP1;MPZL3;TRIM44;SEPT11;GATC;PDAP1;ATP2B1;U2SURP;LETM1;XK;GP
G;ATXN1L;HAS2;MBNL3;MPZL3;SRSF10;SMNDC1;SEPT10;FAM46C;GATC;SOD2;SORBS3;RAB11A;LE
1;YPEL2;C11ORF57;BMPR1A

HST3;FYTTD1;ATL3;ZDHHC21;ZBTB44;FAM117B;DFNB59;ZNRF3;EFS;ZNF629;MAT2A;UBN2;HAS2;MA
BP5;TDG;PCBD2;BCL2L2-PABPN1;MPZL3;SRSF10;SEPT12;TRIM41;TIMMDC1;YLPM1;PCDHB15;GATC
GFRL1;ZNF629;TMEM203;KIAA0355;TDG;UBN2;MAPK1;MPZL3;SRSF10;CNBP;TXNL1;PCGF3;YLPM1;
TRIM44;CA12;TRIM41;FAM46C;GATC;PDAP1;GTF2H1;GTF2H5;LETM1;CDKN2AIPNL;GPAM;RFWD3;C
4;FAM117B;COL19A1;JAKMIP2;ZNF629;MAT2A;UBN2;TMEM68;MAPK1;PCBD2;LRIG2;MBNL3;ATOH1;C
YTTD1;SEMA3A;PALM2;PRUNE2;NRXN3;ZDHHC21;FAM117B;COL19A1;ZDHHC18;EFS;ZNF629;MAT2A

C3;TMEM106A;PRTFDC1;FYTTD1;SEMA3A;ZBTB44;PPM1E;FAM117B;ZNRF2;DFNB59;EFS;ZNF629;MA
;USP1;VPS54;S1PR3;MPZL3;SRSF10;JAM2;MAP3K2;SUSD1;TMEM64;DEK;SULF2;LETM1;PDP2;APC;
CSNK1A1;FAM46C;TPBG;KLK13;ATP2B4;FBXL17;PLEKHA3;SOD2;ITCH;MARCKS;XK;PDP2;KIAA0101;
TDG;UBN2;NRIP1;MPZL3;SEPT10;SORT1;FAM46C;YLPM1;GATC;C1ORF21;PTPN11;TMEM64;HIPK1;HE
MBNL3;TRIM44;S100PBP;CA12;TIMMDC1;GATC;PDAP1;GTF2H1;SORL1;GTF2H5;ADAT2;HEYL;FAM16
0;GRIN2B;SYT7;PHOX2B;ABHD15;REEP1;OCLN;EFNA3;RCAN2;PPP1R1B;LHX6;LHX4;HRK;GPR27;G
EXF;CDYL2;SUMF1;KAT2B;RAP2A;PGAM5;MAPRE3;RAPGEF6;FYTTD1;SF3B3;ATL2;XIAP;ZDHHC21;U
ST8SIA3;SLC4A10;RASSF8;PARVA;GDF6;GRIN2B;PHOX2B;REEP1;GLUD2;OCLN;DIAPH2;EFNA3;RCA
;PURA;PGRMC1;TDG;ATXN1L;S1PR1;C10ORF2;VPS54;SLC25A24;USP9X;B3GAT2;C1ORF21;KCNRG;
T7;ZNF462;SEPT12;SPRYD7;GATC;UBE2G1;C1ORF21;PTPN11;SOD2;SORL1;U2SURP;SULF2;KIAA01
LC25A24;TIMMDC1;CSNK1A1;KCNIP2;FAM46C;LAPTM5;UBE2G1;PLEKHA3;KCNRG;GATAD2B;VAPA;V
2;SLC17A6;PCBD2;MPZL3;TIMMDC1;SUSD1;LGI2;GATC;PDAP1;TPCN1;LETM1;HEYL;ZNF70;DPY19L2
DC1;TRIM44;S100PBP;TRIM41;TIMMDC1;GATC;GTF2H1;U2SURP;ADAT2;HEYL;CDKN2AIPNL;GPAM;P

B;CCDC50;APPL1;CCDC112;VKORC1;ARHGEF10;GPR37;TMED8;MME;TTC33;SORD;PROX1;GNL1;CD
2;ARHGAP31;EFNA3;SP1;SESTD1;ID4;DNAL1;RAD9A;ITM2C;FGFR1;HRK;GPR27;TNFAIP8;BMPR2;AN
S2;TDG;PCBD2;EIF4E;S100PBP;SLC25A24;SEPT11;TRIM41;SORT1;CNBP;ATP2B4;GTF2H5;HIPK2;MY

K2;TRIM41;CSNK1A1;SUSD1;GATC;PDAP1;TMEM64;RAB11A;ADAT2;TPCN1;PDP2;APC;TMEM56;OCIAI
APB;PPM1E;DRP2;NEURL1B;ERBB4;MBNL3;MPZL3;SUSD1;LAPTM5;RAB11A;TPCN1;LETM1;TMEM56;
;TMEM68;NRIP1;USP1;VPS54;LRIG2;RPP14;MPZL3;MAP3K2;SEPT10;TRIM41;CSNK1A1;GATC;PDAP1;
27;DYRK3;BMPR2;DUSP19;PPWD1;TNFAIP3;SIX1;RSF1;CTDSPL2;JPH1;PITPNC1;ELAVL2;IL18BP;ALA
MRPL42;UBL3;SART3;SIPA1L1;SH3PXD2A;ALDH2;MAN1A2;PSD4;FAM73A;SH3BGRL2;UBXN7;METTL1(
RAD9A;BMPR1A;GPR27;NRP2;BMPR2;ANKRD33B;TTC22;PPWD1;TNFAIP3;PPP1R9A;JPH1;PITPNC1;A
N2;LRIG2;MBNL3;SUSD1;PLEKHA3;PTPN11;HIPK1;C20ORF112;DPY19L2;PDP2;NFIA;ASXL3;CAMK4;Y

M68;GPC5;MBNL3;MPZL3;TRIM44;CSNK1A1;KCNIP2;MTSS1L;TMEM64;ADAT2;TPCN1;APC;CCNYL1;R
CR10;JAKMIP2;DFNB59;ZNF629;PTK2B;SLC17A6;GPC5;SLIT2;TPBG;B3GAT2;ATP2B2;STXBP5L;XK;FI
2;RPP14;SLC25A24;CRBN;CSNK1A1;KCNIP2;YLPM1;PLEKHA3;PTPN11;PITHD1;ETNK1;RPA4;OCIAD1;
M63B;C10ORF2;VPS54;MAPK1;LRIG2;BCL2L2-PABPN1;TRIM44;IGBP1;SEPT11;TIMMDC1;GATC;GTF2H
;MPZL3;TRIM44;SLC25A24;CA12;SEPT10;TRIM41;TIMMDC1;USP9X;SPRYD7;GATC;ADAT2;HEYL;FAM
;NDNF;ZDHHC21;ZBTB44;FAM117B;EFS;ZNF629;MAT2A;UBN2;HAS2;MAPK1;MBNL3;MPZL3;JAM2;CA
OH1;SEPT10;PRRX1;CSNK1A1;FAM46C;TXNL1;LAPTM5;DEK;GATAD2B;STXBP5L;VAPA;APC;VAPB;ET
)3;TMEM68;PCBD2;MPZL3;SLC25A24;TIMMDC1;FOXJ2;GATC;PDAP1;MTSS1L;TSHR;TTC9;FAM167A;N

ENM3;ONECUT2;KCNC2;PDE3B;PTPRM;SIX1;JPH1;RND3;ELAVL2;ADAMTS5;GRM5;ADAMTS2;MECON
LRRC40;SLC39A13;SENP2;DIEXF;CDYL2;ENAH;SNPH;NOS1AP;PGAM5;MAPRE3;ST6GALNAC3;TMEN
PK1;HIPK2;HEYL;TTC9;C20ORF112;XK;FRAS1;NFIA;ASXL3;CCDC6;YPEL2;TNRC6B

PALM2;PRUNE2;ZBTB44;TMEM68;SLIT2;MBNL3;MPZL3;CSNK1A1;FOXJ2;ADAT2;LETM1;TTC9;APC;RA
4;MPZL3;TRIM44;S100PBP;MAP3K2;GATC;PTPN11;TMEM64;DEK;GTF2H5;TPCN1;LETM1;PDP2;APC;S
NT5DC3;SRSF10;SMNDC1;TRIM44;IGBP1;SEPT11;SORT1;SPRYD7;PDAP1;GTF2H1;TTC9;FAM167A;XK
NRP2;GABRB1;TTC22;SNAP23;DIRAS2;ADK;DUSP19;PPP1R9A;JPH1;LITAF;MYPN;PITPNC1;ELAVL2;CI

MDC1;GATC;PDAP1;GTF2H1;GATAD2B;ADAT2;LETM1;FAM167A;ZNF70;GPAM;RFWD3;CAPZA1;CCNYL
PCBD2;BCL2L2-PABPN1;GATC;PDAP1;SYNJ2BP;ADAT2;LETM1;XK;PDP2;GPAM;KIAA0101;PTPN4;C11
T2;PLEKHA3;GATAD2B;TSHR;SULF2;PITHD1;ASXL3;EIF3J;OCIAD1;RLIM;RAB3GAP2;TARDBP
VPS54;BCL2L2-PABPN1;MPZL3;TRIM41;KLK13;FBXL17;PDAP1;GTF2H1;ZNF70;PDP2;GPAM;KLHL7;CC
2;C10ORF2;GPC5;MPZL3;SORT1;SPRYD7;PCGF3;KLK13;PCDHB15;GATC;GTF2H1;KCNRG;HIPK1;GT
HB15;GATC;PLEKHA3;SORBS3;ADAT2;LETM1;HEYL;ITCH;ZNF70;XK;CAMK4;CCDC6;CTNNB1;TRIP12
CLN;EFNA3;RCAN2;FUBP1;PPP1R1B;ID4;LHX6;HNRNPC;ITM2C;BMPR1A;HRK;DOCK5;GABRB1;ONE
2BP;GATAD2B;U2SURP;GTF2H5;NECAP2;MARCKS;MYO1C;PITHD1;CAPZA1;OCIAD1;CCNYL1;TARDB
S2;VPS54;LRIG2;MBNL3;MPZL3;CSNK1A1;FOXJ2;TMEM64;APC;RAB3GAP2;FAM171B;PDE7B;NUPL1;
1;GATC;SORL1;ADAT2;CLCN5;PDP2;KIAA0101;VAPB;ASXL3;EIF3J;TCEA3;SPRY3;TRIP12;FRK;C11OR
FNTB;PTPRM;TNFAIP3;SIX1;BMI1;PITPNC1;HK2;SDR16C5;NHSL2;KIAA1549;GRM5;ADAMTS2;JPH3;S
GPR37;CHST7;MME;GRID1;SGIP1;MMP2;GAB1;GAB3;SORCS1;SMC1A;FAM101B;NRG3;IL1RAPL1;RB
P2;CNBP;PCGF3;ATP2B4;PDAP1;UBE2G1;HIPK1;SORBS3;U2SURP;LETM1;ZNF70;XK;SERBP1;FRK;MI
ORF2;PCBD2;SLIT2;MPZL3;TRIM44;S100PBP;CA12;TRIM41;GATC;PDAP1;LETM1;GPAM;RFWD3;ASXL
G2;MBNL3;MPZL3;JAM2;PCDHB15;LETM1;TTC9;XK;TMEM56;KIF26B;RAB3GAP2;FAM171B;NUPL1
C21;GBX2;VPS54;MBNL3;MAP3K2;SUSD1;CDC42BPA;LETM1;FAM167A;APC;TMEM56;KIF26B;ASB7;F
)9A;ITM2C;HRK;NRP2;BMPR2;ANKRD33B;ONECUT2;SNAP23;ADK;DUSP19;PPWD1;TNFAIP3;CTDSPL

MPZL3;CA12;PDAP1;GTF2H1;C1ORF21;CDC42BPA;SORBS3;HEYL;XK;GPAM;ASXL3;TCEA3;PDE7B;TI
RPP14;MPZL3;TRIM41;PDAP1;KCNRG;SYNJ2BP;RAB11A;MYO1C;PDP2;RFWD3;OCIAD1
RIM41;TIMMDC1;GATC;GTF2H1;ADAT2;LETM1;FAM167A;GPAM;RFWD3;CAPZA1;ASB7;FAM171B;YPEL
;EIF4E;ATOH1;SUSD1;PLEKHA3;S100B;SORBS3;STXBP5L;SULF2;FAM167A;ZNF70;DPY19L2;FRAS1;F
MBNL3;MPZL3;TRIM44;SEPT11;SEPT12;TRIM41;RANBP3;PCDHB15;GATC;PDAP1;TSHR;ADAT2;NECA
CA;GNA13;BCLAF1;IGF2BP1;C10ORF2;LRIG2;S100PBP;CSNK1A1;B3GAT2;LGI2;KLK13;KCNRG;ASPR
OL19A1;NEURL1B;GBX2;CARNS1;GPC5;SLIT2;RELT;JAM2;SPRYD7;SUSD1;MTSS1L;ATP2B2;TSHR;SU
ALM2;PRUNE2;NDNF;HOXD12;PPM1E;JAKMIP2;GBX2;ERBB4;CARNS1;IGF2BP1;GPC5;SLIT2;GABRA1
TXNL1;PCDHB15;GATC;SOD2;TPCN1;ZNF70;GPAM;TCEA3;PDE7B;C11ORF57;TNRC6B

SUSD1;PDAP1;ADAT2;LETM1;PDP2;APC;KLHL7;ASB7;YPEL2
2;SEPT11;TPBG;TMEM64;ATP2B1;RNF148;ZNF70;VAPB;CARM1;RFWD3;NUPL1;YPEL2
PBP;MAP3K2;TRIM41;SUSD1;TMEM64;ADAT2;LETM1;PDP2;APC;SERBP1;TNRC6B
RIM41;CSNK1A1;GATC;GTF2H1;TMEM64;TPCN1;PDP2;APC;ASB7;PDE7B;NUPL1

KS;MYO1C;PDP2;CDKN2AIPNL;PITHD1;CAPZA1;OCIAD1;RLIM;CCNYL1;TARDBP;FRK
D1;FOXJ2;GATC;LETM1;XK;APC;TMEM56;FAM171B;PDE7B;TNRC6B
SPRYD7;FOXJ2;GATC;GPAM;VAPA;ETNK1;KLHL7;FAM171B;TRIP12
MEM64;DEK;SORBS3;TTC9;CAPZA1;TARDBP;NUPL1
2H1;SORBS3;GTF2H5;ADAT2;FAM167A;ZNF70;XK;NFIA;PITHD1;TNRC6B
CNBP;GATC;PDAP1;HIPK1;XK;DPY19L2;PDP2;CDKN2AIPNL;CAMK4;EIF3J;SPRY3;NUPL1
PAM;PITHD1;EIF3J;TCEA3;CCDC6;CCNYL1;TRIP12;SSBP2;TNRC6B
YL2;PTP4A1;KAT2B;SNPH;LCOR;CD47;ST6GALNAC3;LPHN2;PRTFDC1;FYTTD1;RALA;PALM2;PRUNE
A2A;NRG3;MYOD1;IL1RAPL1;NOS1AP;COL6A5;NAT8L;MAPRE2;RNF165;SLC24A2;HDAC5;PPM1L;HP
06A;SLC29A3;KANK2;RALA;PLAG1;ZBTB44;PPM1K;PRICKLE1;PDHB;UQCR10;PURA;ZDHHC18;GBX2
GATAD2B;GTF2H5;LETM1;XK;PDP2;TMEM56;RFWD3;CAPZA1;SERBP1;RLIM;NUPL1;YPEL2;CCDC71L
;SLC7A14;RCSD1;PPP1R9A;JPH1;ELAVL2;LMOD3;SDR16C5;NHSL2;KIAA1549;JPH3;MECOM;RSPO2;T
HR;ADAT2;LETM1;HEYL;FAM167A;DPY19L2;GPAM;NFIA;RFWD3;EIF3J;TNRC6B
RF112;XK;DPY19L2;KIAA0101;RFWD3;CAPZA1;CAMK4;TCEA3;CCDC6;TNRC6B
BP;ADAT2;TPCN1;LETM1;NECAP2;PDP2;APC;SERBP1;TARDBP;OTC
E;SORCS1;SUMF1;NRG3;MAPRE3;LUZP1;PPM1E;TTL;PAPSS2;NEURL1B;RXRA;TMEM65;GPC1;IGF2E

HGAP31;ZNF70;ASXL3;FUBP1;CAPZA1;SERBP1;PDE7B;NUPL1;YPEL2;C11ORF57
3;GPAM;RFWD3;CCDC6;CCNYL1;LSM14B;RAD9A;TNRC6B
;SPRY1;SSBP2;TECPR2;DAND5
NA3;DPY19L2;RFWD3;ASXL3;CAMK4;SERBP1;SPRY3;ASB7;YPEL2;RAD9A
HD1;ID2;RCAN2;PPP1R1B;TARDBP
;RFWD3;EIF3J;STRN;LSM14B;NFE2L1;DNAL1;LRP12;RAD9A;ITM2C;BMPR1A
2B;SORBS3;ESR1;SELE;PHOX2B;EPOR;STXBP5L;REEP1;OCLN;ASXL3;OTC
AP31;PDP2;CDKN2AIPNL;ETNK1;FUBP1;TECPR2;DNAL1;DAND5;PLEKHM3;ITM2C
33B;SNAP23;ADK;DUSP19;PPWD1;CTDSPL2;MYPN;PITPNC1;ING4;ARL5B;SART3;TRPS1;TRIM3;RBM
M3;DOCK5;NRP2;DYRK3;BMPR2;ONECUT2;TTC22;SNAP23;DIRAS2;CTDSPL2;JPH1;PITPNC1;PHF8;M
2;PPP1R1B;TCEA3;FAM171B;MXD1;NUPL1;RAD9A
AMK4;EIF3J;TRIP12;UBE2K;LRP12;LNX2;RAD9A;BMPR1A
A;OSTC;CCNYL1;LHX4;LRP12;TNRC6B;BMPR1A
NNB1;STRN;PTPN4;DNAL1;TNRC6B
RP12;RAD9A;CC2D1B;BMPR1A
PB;PITHD1;FUBP1;CAMK4;UBE2K;LRP12;TNRC6B
;PAX2;ADRA2A;TGFBR3;RAP2B;NRG3;FAM84A;NAA38;PDE5A;NAT8L;SLC24A2;HDAC5;HPGD;SEMA3
8;DEK;S100B;XK;HNRNPK;VAPB;RFWD3;FUBP1;ASB7;NUPL1
1B;SPRY3;RAD9A;TNRC6B;ITM2C
;PDCD4;ASB7;STRN;LHX4;PTPN4
GAP31;FAM167A;ZNF70;XK;GPAM;RFWD3;CCDC6;FAM171B;SERINC3;TECPR2;ITM2C
GAP2;STRN;FAM171B;PDE7B;NUPL1;RAD9A
R4;CCDC112;UTP15;ZCCHC24;KLF10;MAPK1IP1L;DFFA;MME;NSUN3;ITPK1;HAUS3;CDYL2;ADRA2A;
UPL1;TNRC6B
HM3;YPEL2;RAD9A;ITM2C
RAS1;GPAM;ZFP62;EIF3J;CCNYL1;STRN;TECPR2;DNAL1;PLEKHM3
71B;PDE7B;LRP12;RAD9A;TNRC6B
TECPR2;DNAL1;PLEKHM3;YPEL2
9A;FGFR1
ANSL1;OSTC;CAPZA1;LSM14B

;CAPZA1;STRN;TECPR2;DNAL1;RAD9A;TNRC6B
RRC40;FAM101B;RAP2A;RAP2B;RRM2B;FYTTD1;RALA;LUZP1;PRUNE2;PPM1K;PAPSS2;PURA;GBX2;
K;KIF26B;EIF3J;CCNYL1;DAND5;ITM2C;CC2D1B
DNAL1;PLEKHM3
NK1;CAMK4;CCDC6;FAM171B;TRIP12;NUPL1;UHRF1BP1L;TMEM167B;KLHL32;CCND3;SESN3;FADS6

;NXF1;BCLAF1;ZDHHC18;TMEM65;TMEM203;PABPN1;NSD1;CARNS1;NT5DC3;S1PR1;PCBD2;MPZL3;
PRY1;NUPL1;NFE2L1
D5;LRP12
Y3;TECPR2;LRP12
HNRNPK;TMEM56;CDK1
RAB3GAP2;PDE7B;NUPL1;RAD9A;TNRC6B
ING4;ARL5B;SH3BGRL2;RBM7;CNPPD1;UBL7;GRID1;AHSA2;SORD;HAUS3;ANO6;GAB3;GNL1;ADRA2
T1B;GPR37;CHST7;DNMT3A;TFEB;NAALADL2;SUMF1;DDX19B;FCHSD2;SNPH;MAPRE3;SLC29A3;PR

1B;BMPR1A
7B;RAD9A
C6B;CC2D1B

RAD9A;BMPR1A

D9A
A;TNRC6B;ITM2C
RAD9A;TNRC6B
ABRA1;PRRX1;GABRA3;LGI2;KLK13;SORBS3;TSHR;TTC9;FAM167A;DPY19L2;KIF26B;ASXL3;FAM171B

B

;ITM2C
D1B;BMPR1A

A
I2;PDE7B;CALM1

CR10;PAPSS2;TRAK2;TMEM203;GPC1;USP1;GPC5;MPZL3;ATOH1;IGBP1;SEPT11;TIMMDC1;GTF2H1;
PRR3;SNAP23;PPWD1;PFAS;PITPNC1;MED17;CKS1B;ING4;MRPL42;ARL5B;SART3;SIPA1L1;CAPZB;S
LEKHM3;C11ORF57

M14B;PLEKHM3

R1

5;ZCCHC24;MAPK1IP1L;DFFA;GPR37;TMED8;MME;TTC33;ITPK1;SORD;HAUS3;SLC39A13;SORCS1;A

A38;ST6GALNAC3;SLC25A12;PHLPP2;USP15;ATL3;ATL2;DLST;UQCR10;GRAMD4;PPP2CA;NXF1;TNK

;MTSS1L;SYNJ2BP;TSHR;STXBP5L;MARCKS;KANSL1;TCEA3;CTNNB1;FAM171B;PTPN4;YPEL2

;FBXL17;GTF2H1;PTPN11;KCNRG;ATP2B1;ITCH;KIAA0101;VAPA;PITHD1;RFWD3;CAPZA1;ASB7;TARD

;CYTH3;PPP2CA;GNA13;NXF1;PABPN1;NSD1;SEPT11;TIMMDC1;SORT1;CSNK1A1;FAM46C;PCGF3;YL
SOSTDC1;UBXN7;KIF1B;SCN5A;CCDC50;GPR37;MME;SGIP1;DNMT3A;TFEB;PAX5;PAX2;PTP4A1;DDX
)H1;PRRX1;KCNIP2;STXBP5L;FAM167A;DPY19L2;KIF26B;CAMK4;TCEA3;FAM171B;PDE7B;SLC28A3

B;NSUN3;HAUS3;GAB3;SUMF1;LCOR;NAA38;DTL;CRK;TMEM106A;PAFAH1B2;USP15;ATL3;DCTN2;SF

S2

CN5A;UNKL;GPR135;ZCCHC24;KLF10;USP47;NAA30;HSDL1;WDR37;NSUN3;AHSA2;SORD;GPCPD1;I
H3;PPP2CA;GNA13;NXF1;TNKS2;PABPN1;EMC7;TRIM44;SEPT10;SEPT11;TIMMDC1;SORT1;CSNK1A1

EM106A;PAFAH1B2;PHLPP2;ATL3;ATL2;TUBD1;PDHB;UQCR10;NXF1;ZNRF3;GBX2;MGAT5;SLC25A24;T

167A;ID2;CAMK4;JMY;CCNYL1;FAM171B;LSM14B;MXD1
;PTPN11;SOD2;ITCH;SERBP1;EIF3J;RPA4;OCIAD1;RAB3GAP2;TARDBP
I;ENAH;NOS1AP;CLDN18;RBMS1;NAA38;MAPRE2;PPM1L;PRUNE2;FAM129A;NDNF;ZDHHC21;PRICKL

IAT2A;PABPN1;NSD1;SLC25A24;SEPT11;TIMMDC1;SORT1;CSNK1A1;FAM46C;FOXJ2;GATC;PDAP1;KC

K;CD244;PLEKHM3;SLC28A3;FGFR1

L19A1;NSD1;APBB2;MBNL3;MPZL3;ATOH1;SEPT10;USP9X;TXNL1;LGI2;PCDHB15;MAPK10;RNF148;XK
N3;SORD;SENP2;ADRA2B;SUMF1;RRM2B;NAT8L;SLC29A3;CHST3;ATL3;HPGD;FAM129A;NDNF;ADD3;

XN7;CEP170;RBM7;UNKL;CNPPD1;VKORC1;TTC33;PAX5;SENP2;GNL1;DIEXF;ADRA2A;SUMF1;DDX1[illegible]
[illegible]6A;FAM129A;XIAP;ZDHHC21;MAT2B;GRAMD4;TTL;NEURL1B;GBX2;ERBB4;NSD1;CARNS1;ATXN1L;SL[illegible]
RLIM;RAB3GAP2;TARDBP;LSM14B;C11ORF57;LNX2;RAD9A

NX2

;HSDL1;LRRC40;IL17RD;CBFA2T2;SENP2;CNKSR3;TET3;LCOR;PGAM5;NAA38;LPHN2;ERGIC2;SOS2;
ENP2;SUMF1;PTP4A1;FCHSD2;TERF2IP;CD47;ERGIC2;CRK;PHLPP2;FYTTD1;NAPB;RALA;STAU1;LU
/108;PRKG1;HS3ST3B1;GPR37;GRID1;DNMT3A;SORCS1;PAX5;ADRA2B;PAX2;FAM101B;NRG3;SNPH;
SGIP1;SORCS1;PROX1;ADRA2B;ADRA2A;NRG3;RBMS1;SMTNL2;ST6GALNAC3;CHST3;RNF165;PLA
/2;TUBD1;PRICKLE1;OGFRL1;BCLAF1;TMEM65;TMEM203;TNKS2;PABPN1;TMEM68;NRIP1;S1PR1;VF

3G;LGI2;TBX5;TTC9;MARCKS;NFIA;TMEM56;KIF26B;FAM171B
A;ENAH;FAM101B;NRG3;SNPH;NOS1AP;NAT8L;CHST3;MYRF;PLAG1;PALM2;NDNF;HOXD12;PAPSS2;
P;COL6A5;SMTNL2;NAT8L;MAPRE2;SLC24A2;HDAC5;PPM1L;PALM2;PRUNE2;PPM1H;NDNF;PPM1E;C

N5A;GPR135;PHACTR4;VKORC1;HS3ST3B1;ZCCHC24;ARHGEF10;GPR37;WDR37;GRID1;NSUN3;AHS
P13;MYRF;PLAG1;PPM1H;PPM1E;PAPSS2;NEURL1B;ZNRF3;EFS;GBX2;ATOH1;SORT1;KCNIP2;TPBG;
;JAM2;ATOH1;IGBP1;TTC9;XK;FRAS1;NFIA;CAPZA1;CCDC6;TARDBP;C11ORF57

3;KIF1B;EMB;APPL1;CCDC112;ZCCHC24;GPR37;CHST7;MME;SGIP1;MMP2;GAB1;ANO6;SORCS1;FAM
P14;MBNL3;MPZL3;S100PBP;MAP3K2;SEPT10;SEPT11;TRIM41;CSNK1A1;PDAP1;TMEM64;SYNJ2BP;
S2;TRAK2;ZNF629;ERBB4;MGAT5;UBN2;NRIP1;VPS54;MBNL3;JAM2;ZNF462;USP9X;SORT1;SUSD1;YL
.2;ENAH;FCHSD2;ARHGEF5;PALM2;ATL2;PRUNE2;FAM129A;PPM1E;COL19A1;PAPSS2;CYTH3;NEURL
;FYTTD1;ZDHHC21;ZBTB44;FAM117B;DFNB59;ZDHHC18;EFS;MAT2A;UBN2;HAS2;MAPK1;S1PR3;MBN
ND3;ELAVL2;ADAMTS5;GRM5;ADAMTS2;MECOM;TRPS1;TMEM108;SH3BGRL2;SCN5A;EMB;TEAD1;G
;MGAT5;TDG;USP1;VPS54;MAPK1;LRIG2;RPP14;MPZL3;JAM2;MAP3K2;TMEM64;SYNJ2BP;RAB11A;A
PM1L;NDNF;ZDHHC21;ZBTB44;FAM117B;COL19A1;PURA;DFNB59;ZNRF3;ZNF629;MAT2A;UBN2;NRIP
NRP2;COX7B;BMPR2;DIRAS2;ADK;PPWD1;AFF1;ELAVL2;MED17;CKS1B;RBM3;NHSL2;SERP1;XPO4;
HHC18;GBX2;ERBB4;NRIP1;IGF2BP1;S1PR1;PTK2B;GPC5;S1PR3;ATOH1;SLC25A24;PRRX1;TPBG;B3

1;PALM2;ZDHHC21;ZBTB44;FAM117B;DFNB59;ZNF629;MAT2A;ERBB4;UBN2;HAS2;MAPK1;PCBD2;SL
BP5;S1PR1;LRIG2;MBNL3;JAM2;LGI2;PTPN11;KCNRG;TBX5;SOD2;ASPRV1;RAB11A;STXBP5L;SULF2;
9;MAT2A;UBN2;HAS2;PCBD2;MBNL3;MPZL3;SORT1;PCDHB15;PTPN11;HIPK1;TTC9;FRAS1;NFIA;YPE
1R9A;JPH1;PITPNC1;ELAVL2;ADAMTS5;MED14;KIAA1549;ADAMTS2;SPRED1;MECOM;C1QBP;TMEM1
;ZDHHC21;FAM117B;DFNB59;EFS;ZNF629;MAT2A;UBN2;HAS2;MAPK1;S1PR3;LRIG2;MBNL3;MPZL3;C
S;ZNF629;MAT2A;UBN2;HAS2;PCBD2;SLIT2;MBNL3;MPZL3;CA12;YLPM1;PCDHB15;PDAP1;PLEKHA3;
4;RPP14;MPZL3;SRSF10;AAED1;TRIM41;CSNK1A1;PDAP1;TMEM64;DEK;RAB11A;TPCN1;LETM1;XK;P

TK2B;MAPK1;PCBD2;RPP14;MPZL3;GALNT7;TRIM41;TIMMDC1;CNBP;PDAP1;UBE2G1;DEK;SYNJ2BP;
A;TTC9;XK;FRAS1;NFIA;CTNNB1;TRIP12;TNRC6B
RUNE2;NDNF;PRICKLE1;PPM1E;COL19A1;ADD2;GBX2;ERBB4;IGF2BP1;GPC5;JAM2;ATOH1;B3GAT2;
7;TIMMDC1;TXNL1;GATC;PDAP1;GTF2H1;SYNJ2BP;U2SURP;GTF2H5;ADAT2;LETM1;VAPA;KANSL1;E
N2;S1PR1;HAS2;MAPK1;S1PR3;MPZL3;JAM2;CA12;B3GAT2;PTPN11;TMEM64;HIPK1;TTC9;FRAS1;NF
AT2A;UBN2;VPS54;MAPK1;S1PR3;MPZL3;KCNIP2;B3GAT2;PLEKHA3;PTPN11;HIPK1;TSHR;TTC9;FAM1
AT2A;UBN2;HAS2;MAPK1;SLIT2;MBNL3;MPZL3;PTPN11;TMEM64;DEK;HIPK1;HEYL;TTC9;C20ORF112
;FOXJ2;PTPN11;DEK;SYNJ2BP;CDC42BPA;GATAD2B;ADAT2;NECAP2;ITCH;MARCKS;MYO1C;SERBP

)2;SEPT10;TRIM41;DEK;TTC9;C20ORF112;FRAS1;NFIA;KIF26B;CAPZA1;YPEL2;TNRC6B
AP1;GTF2H1;CDC42BPA;U2SURP;GTF2H5;SULF2;NECAP2;NFIA;CAPZA1;TARDBP;FRK;C11ORF57;ME
T2;MPZL3;SLC25A24;LGI2;PDAP1;PLEKHA3;TMEM64;ADAT2;HEYL;C20ORF112;XK;DPY19L2;GPAM;PI
ZL3;TRIM44;SEPT10;TIMMDC1;SPRYD7;LGI2;PCDHB15;ATP2B4;GATC;PDAP1;SOD2;HIPK1;ADAT2;M
JPH1;LITAF;PFAS;MYPN;PITPNC1;ELAVL2;CKS1B;NHSL2;ADAMTS2;SPRED1;C1QBP;TRPS1;FAM154
7B;DFNB59;ZNF629;MAT2A;UBN2;MAPK1;S1PR3;PCBD2;RPP14;MPZL3;ATOH1;TRIM41;KCNIP2;KLK1
I;HAS2;SLC17A6;S1PR3;MBNL3;JAM2;SLC25A24;KCNIP2;ATP2B2;TSHR;STXBP5L;HEYL;DPY19L2;KIF
L3;MPZL3;CA12;PDAP1;PTPN11;TMEM64;HIPK1;HIPK2;TTC9;NFIA;ASXL3;YPEL2;TNRC6B
DR16C5;NHSL2;KIAA1549;ADAMTS2;JPH3;MECOM;SH3PXD2A;NKRF;TRPS1;PSD3;SH3BGRL2;SOST
;TPCN1;LETM1;TTC9;NECAP2;XK;PDP2;APC;TMEM56;RFWD3;SERBP1;RAB3GAP2;SPRY1;TARDBP

KS2;NT5DC3;NRIP1;IGF2BP1;MPZL3;EMC7;SRSF10;SEPT11;FOXJ2;GATC;LETM1;GPAM;ASB7;SSBP2
(1A1;KCNIP2;TXNL1;MTSS1L;PLEKHA3;TMEM64;LETM1;TCEA3;RAB3GAP2;FAM171B;PDE7B;NUPL1
;TMEM167B;IRS4;ZBTB20;LOXL4;FAM204A;AMOT;EFEMP1;CDH2;FADS6;PIM3;IL6R;SH3GL2;PCDHA(
)K;PPWD1;ETFA;SMG7;ALAD;ING4;DTWD1;ARL5B;XPO4;ALDH2;NKRF;MAN1A2;FAM154B;TRIM3;SH3I
(S;FAM167A;DPY19L2;FRAS1;CAMK4;PDCD4;FAM171B;YPEL2;TNRC6B
DHB15;PDAP1;PTPN11;HIPK1;TTC9;C20ORF112;DPY19L2;NFIA;ASXL3;CAPZA1;YPEL2;TNRC6B
SLIT2;MBNL3;MPZL3;CA12;B3GAT2;PCDHB15;PTPN11;TMEM64;HIPK1;TTC9;C20ORF112;DPY19L2;NI
SLIT2;MBNL3;CA12;GABRA1;TPBG;B3GAT2;LGI2;STXBP5L;TTC9;FAM167A;FRAS1;ASXL3;CAMK4;TCI
NSUN3;MSL2;HAUS3;PROX1;IL17RD;ZFX;CDYL2;SUMF1;RAP2C;KAT2B;ST6GALNAC3;ERGIC2;MAPRI
4;NHSL2;KIAA1549;ARL5B;CAPZB;NKRF;SH3BGRL2;UNKL;UBL7;PLA2G12A;TMED8;C3ORF62;DNMT3
;STXBP5L;TTC9;FAM167A;DPY19L2;FRAS1;TMEM56;CAMK4;FAM171B;SLC28A3
P1;PTPN11;HIPK1;SYNJ2BP;CDC42BPA;HIPK2;CLCN5;PITHD1;ASXL3;EIF3J;SPRY3;FRK
AP2;PDP2;VAPA;PITHD1;RFWD3;RLIM;RAB3GAP2;PTPN4;TARDBP;SSBP2
M44;MAP3K2;AAED1;SUSD1;GATC;ADAT2;TPCN1;LETM1;PDP2;APC;YPEL2
AM2;ATOH1;CA12;PRRX1;B3GAT2;TBX5;TSHR;SULF2;XK;DPY19L2;KIF26B;ASXL3;TCEA3
IGF2BP1;GPC5;SLIT2;ATOH1;CA12;GABRA1;PRRX1;B3GAT2;LGI2;TBX5;SORBS3;TSHR;ASXL3;RPA4;(
GATC;PDAP1;S100B;SYNJ2BP;U2SURP;NECAP2;ITCH;FAM167A;ZNF70;PDP2;VAPA;PITHD1;EIF3J;CT
PPM1E;HAS2;SLC17A6;MBNL3;MPZL3;JAM2;PCDHB15;FBXL17;TPCN1;TMEM56;PDE7B;NUPL1
'2;PDAP1;PTPN11;HIPK1;CDC42BPA;SORL1;TTC9;C20ORF112;NFIA;ASXL3;YPEL2;TNRC6B
LC25A45;TGM2;EOMES;KCMF1;DYRK1A;ALG14;CNPY1;ARID5B;FTSJ2;BCMO1;MOB3C;MAF;FAM185A
I4;MBNL3;CA12;TRIM41;PLEKHA3;TMEM64;HIPK1;HIPK2;TTC9;C20ORF112;MARCKS;NFIA;KIF26B;YPI
TNFAIP3;SIX1;CTDSPL2;MYPN;PITPNC1;ELAVL2;ALAD;ADAMTS5;NHSL2;KIAA1549;ARL5B;NKRF;MAI

M1;MAPK10;XK;TCEA3;CCNYL1;RAB3GAP2;FAM171B;PDE7B;PTPN4;TNRC6B
2;ZNF70;DPY19L2;GPAM;KIAA0101;APC;CAMK4;TCEA3;C11ORF57
AM;CARM1;ASB7;FAM171B;TRIP12;PDE7B;SSBP2
TM1;ITCH;ZNF70;XK;CDKN2AIPNL;PITHD1;EIF3J;FRK;C11ORF57

PK1;MBNL3;MPZL3;CA12;SORT1;PTPN11;TMEM64;HIPK1;TTC9;C20ORF112;NFIA;ASXL3;SPRY1;YPEI
;FBXL17;PDAP1;C1ORF21;SOD2;HIPK1;GTF2H5;NECAP2;NFIA;CAPZA1;PDCD4;TRIP12;TNRC6B
GATC;TMEM64;U2SURP;NECAP2;ASXL3;CCNYL1;TRIP12;FRK;TNRC6B
APZA1;EIF3J;TCEA3;CCNYL1;TRIP12;PDE7B;TNRC6B
CA12;ZNF462;MTSS1L;GTF2H1;PTPN11;HIPK1;TTC9;C20ORF112;NFIA;ASXL3;CAMK4;TNRC6B
;TMEM68;HAS2;VPS54;MBNL3;MPZL3;JAM2;CA12;PDAP1;PTPN11;HIPK1;TTC9;C20ORF112;FAM167A

T2A;UBN2;MAPK1;JAM2;CA12;ZNF462;SEPT10;TMEM64;HIPK1;XK;FRAS1;NFIA;ASXL3;CAMK4;YPEL2
KLHL7;ASXL3;OCIAD1;ASB7;PDE7B
CARM1;PDCD4;PTPN4;SSBP2;NUPL1;YPEL2
EYL;TTC9;XK;DPY19L2;NFIA;KIF26B;CAMK4;CCDC6;YPEL2;TNRC6B
57A;ZNF70;NFIA;KIAA0101;PITHD1;RFWD3;CAPZA1;EIF3J;FRK;TNRC6B
ABRB1;KCNC2;TTC22;DIRAS2;PDE3B;SLC7A14;SIX1;RCSD1;C1ORF213;LITAF;ELAVL2;ADAMTS5;SDF
QCR10;FAM117B;TRAK2;DFNB59;UBN2;HAS2;C10ORF2;MBNL3;MPZL3;JAM2;CA12;ZNF462;KLK13;PC
N2;PPP1R1B;ID4;LHX4;FGFR1;NRP2;ANKRD33B;ONECUT2;TTC22;SLC7A14;KIAA1671;SIX1;PPP1R9A
TPCN1;NECAP2;KIAA0101;KIF26B;ASB7;C11ORF57
01;KLHL7;CAMK4;CCDC6;OCIAD1;PDCD4;FAM171B;YPEL2;C11ORF57
APB;CAPZA1;OCIAD1;RLIM;RAB3GAP2;TARDBP;LNX2
2;RFWD3;ASXL3;CAPZA1;CAMK4;EIF3J;TCEA3
ITHD1;CAPZA1;EIF3J;TRIP12;PDE7B;FRK

YL2;RAP2C;FAM101B;RAP2B;FCHSD2;RRM2B;MAPRE3;ERGIC2;DTL;SOS2;TMEM106A;CHST3;USP1
IKRD33B;ONECUT2;TTC22;DIRAS2;DUSP19;PPWD1;SIX1;CTDSPL2;PPP1R9A;PITPNC1;ELAVL2;PHF8
O1C;CDKN2AIPNL;CARM1;CCNYL1;TRIP12;TNRC6B

D1;PTPN4;OTC;TNRC6B
;TCEA3;CCNYL1;FAM171B;PDE7B;NUPL1;TNRC6B
;TMEM64;ADAT2;TPCN1;NECAP2;RAB3GAP2;FAM171B;TNRC6B
AD;ADAMTS5;DTWD1;NHSL2;JPH3;MECOM;FAM154B;KIF1B;PRKG1;HS3ST3B1;ZCCHC24;NCOA2;GPI
6;CTBS;UNKL;PHACTR4;MAPK1IP1L;DFFA;CHST7;MME;TTC33;NSUN3;ITPK1;SORD;HAUS3;PROX1;Z
ADAMTS5;DTWD1;NHSL2;TMEM108;CEP170;RBM7;UBL7;GPR135;HS3ST3B1;MAPK1IP1L;NCOA2;GPR
PEL2;TNRC6B

RAB3GAP2;FAM171B;PDE7B;PTPN4
RAS1;ETNK1;KIF26B;ASXL3;CAMK4
;RAB3GAP2;TARDBP;LNX2
H1;LETM1;KLHL7;SERBP1;EIF3J;FAM171B;NUPL1
I167A;ZNF70;CDKN2AIPNL;PITHD1;RFWD3;CAMK4;TNRC6B
12;B3GAT2;PDAP1;PTPN11;HIPK1;SORL1;TTC9;C20ORF112;NFIA;ASXL3;TNRC6B
TNK1;RPA4;OCIAD1;NUPL1;C11ORF57;TNRC6B
MYO1C;XK;CCNYL1;FAM171B;TRIP12

M;TRPS1;SCN5A;EMB;TEAD1;GPR135;HS3ST3B1;ARHGEF10;CHST7;MME;GRID1;SGIP1;MMP2;DNMT
M106A;PRTFDC1;RNF165;USP13;HDAC5;PLAG1;PRUNE2;PDHB;UQCR10;NEURL1B;EFS;TMEM203;TD

AB3GAP2;FAM171B;PDE7B;PTPN4;NUPL1
ERBP1
K;CCNYL1;TRIP12
KS1B;DTWD1;KIAA1549;SPRED1;NKRF;SLC22A17;MFSD5;FAM154B;TRIM3;SCN5A;RBM7;CCDC50;HS

L1;PDE7B;FRK;TNRC6B
ORF57

DC6;CCNYL1
F2H5;RAB11A;LETM1;HEYL;TTC9;ZNF70;GPAM;NFIA;KIF26B;KLHL7;CAMK4;FAM171B;TRIP12;FRK;YF
;TARDBP;FAM49A;PRDM6;KLHL32;ZBTB20;RIMS3;CDH2;FADS6;KIF5C;MACROD2;SLC25A45;IL6R;PSI
CUT2;KCNC2;TTC22;CELF3;DIRAS2;SLC7A14;DUSP19;RCSD1;JPH1;LITAF;ELAVL2;ADAMTS5;GRM5;
P;NUPL1
OTC;TNRC6B
F57;LNX2
ART3;SH3PXD2A;RSPO2;SOSTDC1;SCN5A;EMB;HS3ST3B1;ZCCHC24;HSDL1;MMP2;DNMT3A;GAB1;
MS1;ARHGEF5;SLC24A2;AMER1;NAPB;HPGD;PALM2;PPM1H;FAM129A;TTL;COL19A1;DRP2;NEURL1
ETAP2
L3;CAMK4;TCEA3

AM171B;SSBP2
2;C1ORF213;PPP1R9A;MYPN;PITPNC1;ALAD;MRPL42;ADAMTS5;SPRED1;SERP1;TRPS1;FAM154B;S

NRC6B

2
PDP2;TCEA3
P2;PDP2;C11ORF57
V1;FRAS1
JLF2;FAM167A;DPY19L2
I;PRRX1;KCNIP2;B3GAT2;LGI2;S100B;SORBS3;EPOR;TSHR;STXBP5L;TCEA3

E2;ZDHHC21;ZBTB44;LRP8;FAM117B;ZNF629;UBN2;C10ORF2;MPZL3;ATOH1;GALNT7;IGBP1;TRIM41;F
GD;PLAG1;PPM1H;FAM129A;NRXN3;NDNF;PPM1E;TTL;C21ORF119;GBX2;ERBB4;IGF2BP1;GPC5;SLIT
2;TMEM203;SERTAD2;PCBD2;SLIT2;LRIG2;MPZL3;GALNT7;SEPT10;SEPT11;TIMMDC1;SORT1;SPRYD
L;FAM49A;TM7SF3;CCND3;SESN3;PAPOLG;CHP1;AP2M1;KPNA1;TGM2;MAP2K1;TPM3;TMOD3;SWAP
TMEM108;SH3BGRL2;SCN5A;GPR37;TMED8;GRID1;GAB3;PAX5;PROX1;ADRA2A;NRG3;FAM84A;COL6

BP1;HAS2;GPC5;APBB2;MPZL3;EIF4E;ATOH1;SEPT12;KCNIP2;KCNRG;TSHR;STXBP5L;HEYL;MARCKS

M7;PRKG1;HS3ST3B1;MAPK1IP1L;PLA2G12A;CHST7;TMED8;SGIP1;NSUN3;C3ORF62;AHSA2;GAB1;LF
ED14;SPRED1;MECOM;ALDH2;FAM154B;SH3BGRL2;KIF1B;APPL1;C3ORF62;DNMT3A;GAB1;SLC39A1

3A;TYRP1;NDNF;PRICKLE1;GRAMD4;TRAK2;OGFRL1;ZNRF3;ERBB4;GPC1;NSD1;NRIP1;IGF2BP1;SLC

RAP2C;FCHSD2;FAM84A;SMTNL2;ST6GALNAC3;ERGIC2;SLC29A3;RNF165;AMER1;NAPB;USP15;HPC

MGAT5;GPC1;TMEM68;GPC5;SLIT2;MPZL3;ATOH1;SLC25A24;IGBP1;SEPT10;TRIM41;PLEKHA3;SOD2

;;CHP1;MAP3K9;SLC25A45;PSPH;KPNA1;FBXW4;RAB2B;VASH2;DIO2;DYRK1A;ALG14;FTSJ2;MOB3C;

EMC7;RELT;AAED1;IGBP1;SEPT10;SEPT11;TIMMDC1;SORT1;SPRYD7;MTSS1L;U2SURP;GTF2H5;LET

2B;RAP2A;RAP2B;RBL1;NAA35;MAPRE3;CD47;SLC25A12;DTL;TMEM106A;SLC29A3;RALA;ROCK1;UQ
RTFDC1;ATL3;PPM1L;PALM2;PRUNE2;FAM129A;ZDHHC21;MAT2B;COL19A1;ZNRF2;PGRMC1;PABPN1

3

MARCKS;FAM167A;KIF26B;CCDC6;ASB7
SIN3A;SLC22A17;MFSD5;UBXN7;KIF1B;CTBS;APPL1;UBL7;PHACTR4;CCDC112;VKORC1;MAPK1IP1L;N

[illegible]DRA2B;DIEXF;CDYL2;RAP2C;FAM84A;ST6GALNAC3;ERGIC2;SLC29A3;NAPA;AMER1;USP15;HPGD;Z[illegible]

S2;PABPN1;TRIM44;TIMMDC1;SORT1;CSNK1A1;FAM46C;PCGF3;YLPM1;FOXJ2;GATC;PDAP1;PLEKH[illegible]

[illegible]BP

LPM1;GATC;PDAP1;PLEKHA3;KCNRG;DEK;TPCN1;VAPB;KLHL7;CAPZA1;OCIAD1;RAB3GAP2;LNX2
X19B;SPEM1;SLC25A12;CRK;SLC29A3;PRTFDC1;CHST3;ATL3;PPM1L;FAM129A;XIAP;ZDHHC21;PRIC

RSF1;XIAP;ZDHHC21;TTL;AP3M2;BCLAF1;MAT2A;PABPN1;S1PR1;SRSF10;EIF4E;SMNDC1;CRBN;USP

L17RD;DIEXF;SUMF1;ENAH;TGFBR3;FAM101B;DDX19B;CNKSR3;NOS1AP;RBMS1;MAPRE3;SMTNL2;
;FAM46C;PCGF3;GATC;FBXL17;PDAP1;PLEKHA3;KCNRG;TPCN1;VAPB;KLHL7;CAPZA1;OCIAD1;RAB3

TRIM41;TIMMDC1;CSNK1A1;FOXJ2;GATC;PLEKHA3;GTF2H1;SOD2;PITHD1;KLHL7;CAPZA1;RPA4;OCI

LE1;TTL;COL19A1;DRP2;EFS;S1PR1;MAPK1;GPC5;ATOH1;SORT1;B3GAT2;LGI2;GATC;PDAP1;SORL1;

CNRG;ASPRV1;TPCN1;ITCH;VAPB;PITHD1;ETNK1;CAPZA1;OCIAD1;RAB3GAP2;CD244

K;DPY19L2;TMEM56;RLIM
;GRAMD4;COL19A1;JAKMIP2;DRP2;GBX2;TMEM203;NSD1;TMEM68;CARNS1;S1PR3;EIF4E;GABRA1;S

9B;FCHSD2;TET3;LCOR;NAA35;PGAM5;SLC25A12;HDAC5;DCTN2;ATL2;UQCR10;AP3M2;PPP2CA;NXI
LIT2;MPZL3;JAM2;AAED1;SEPT12;ATP2B4;PLEKHA3;KCNRG;GTF2H5;STXBP5L;ADAT2;FRAS1;PITHD

;PAFAH1B2;ARHGEF5;PHLPP2;FYTTD1;SF3B3;ROCK1;DCTN2;NUFIP2;PLAG1;TUBD1;ZDHHC21;ZBTE
ZP1;HPGD;ATL2;ZDHHC21;PDHB;PAPSS2;LRP6;PPP2CA;NEURL1B;OGFRL1;NXF1;PGRMC1;MAT2A;T
;MYOD1;NOS1AP;ARHGEF5;CHST3;PALM2;NDNF;PRICKLE1;HOXD12;PPM1E;COL19A1;PAPSS2;ADD
G1;HOXD12;COL19A1;ADD2;EFS;ERBB4;IGF2BP1;S1PR1;HAS2;S1PR3;JAM2;ATOH1;PRRX1;KCNIP2;E
PS54;SMNDC1;TRIM44;TIMMDC1;FOXJ2;PDAP1;PLEKHA3;GTF2H1;ATP2B1;GTF2H5;TTC9;GPAM;VAP

;EFS;IGF2BP1;S1PR1;S1PR3;JAM2;TPBG;B3GAT2;CDC42BPA;TSHR;STXBP5L;SULF2;TTC9;FRAS1;TM
COL19A1;ADD2;GBX2;ERBB4;S1PR1;HAS2;SLC17A6;GPC5;SLIT2;JAM2;ATOH1;PRRX1;LGI2;ATP2B2;T

A2;SORD;GAB3;PAX5;PROX1;ZFX;CDYL2;ADRA2A;PTP4A1;KAT2B;RBL1;RRM2B;SNPH;CNKSR3;NOS
;B3GAT2;LGI2;MTSS1L;ATP2B2;CDC42BPA;STXBP5L;TTC9;KIF26B;CAMK4;FAM171B;SPRY1;PDE7B

I101B;RAP2A;DDX19B;NRG3;MYOD1;IL1RAPL1;RBMS1;MAPRE3;MAPRE2;ARHGEF5;RNF165;KANK2;
RAB11A;ADAT2;FRAS1;PDP2;APC;KIF26B;KLHL7;RFWD3;SERBP1;OCIAD1;PDE7B;NUPL1
LPM1;PDAP1;PLEKHA3;C1ORF21;TMEM64;HIPK1;U2SURP;HEYL;TTC9;FAM167A;DPY19L2;NFIA;PDE7
L1B;GPC1;ATXN1L;S1PR3;MBNL3;SLC25A24;GALNT7;KCNIP2;B3GAT2;FOXJ2;TSHR;HEYL;TTC9;DPY
NL3;MPZL3;GALNT7;CA12;B3GAT2;PCDHB15;PDAP1;PTPN11;HIPK1;TTC9;C20ORF112;DPY19L2;NFIA;
PR135;HS3ST3B1;ZCCHC24;ARHGEF10;MME;GRID1;SGIP1;MMP2;DNMT3A;GAB1;SORCS1;PROX1;A
DAT2;LETM1;XK;FRAS1;PDP2;APC;RFWD3;SERBP1;RLIM;RAB3GAP2;FAM171B;NUPL1
1;VPS54;MAPK1;MBNL3;MPZL3;CA12;B3GAT2;KLK13;PCDHB15;PDAP1;MTSS1L;GTF2H1;PTPN11;HIP
TRPS1;FAM73A;TMEM108;SH3BGRL2;UBXN7;APPL1;DGCR2;UBL7;MAPK1IP1L;PLA2G12A;CHST7;WD
GAT2;LGI2;ATP2B2;SORL1;SORBS3;SULF2;TTC9;FRAS1;CAMK4;TCEA3;FAM171B

IT2;MBNL3;MPZL3;JAM2;CA12;TRIM41;SORT1;B3GAT2;PCDHB15;PTPN11;HIPK1;CDC42BPA;TTC9;C2
TTC9;FRAS1;KIF26B;CAMK4;CCNYL1;SPRY1;TARDBP
L2;TNRC6B
108;KIF1B;CEP170;CCDC50;PRKG1;GPR135;HS3ST3B1;GPR37;CHST7;MME;GRID1;GAB3;ZFY;ZFX;CD
CA12;USP9X;PDAP1;ATP2B2;PTPN11;HIPK1;TTC9;C20ORF112;FRAS1;NFIA;ASXL3;PTPN4;YPEL2;TNR
PTPN11;TMEM64;HIPK1;TTC9;XK;FRAS1;NFIA;ASXL3;YPEL2;TNRC6B
DP2;NFIA;APC;KIF26B;FAM171B;SPRY1;TARDBP

ADAT2;MYO1C;CDKN2AIPNL;APC;CAPZA1;TRIP12

LGI2;ATP2B2;SORL1;SORBS3;TSHR;STXBP5L;TTC9;ASXL3
TNK1;FAM171B;TRIP12
A;ASXL3;YPEL2;TNRC6B
67A;FRAS1;NFIA;ASXL3;CAPZA1;YPEL2;TNRC6B
;FRAS1;NFIA;ASXL3;YPEL2;TNRC6B
1;RLIM;ASB7;RAB3GAP2;PTPN4;LNX2

TAP2
THD1;CAPZA1;CAMK4
ARCKS;GPAM;NFIA;PITHD1;CAPZA1;CAMK4;EIF3J;FAM171B
3;SH3BGRL2;KIF1B;UNKL;CCDC50;PRKG1;GPR135;HS3ST3B1;PLA2G12A;GPR37;TMED8;GRID1;DNM
3;PDAP1;TMEM64;HIPK1;TSHR;HEYL;TTC9;C20ORF112;YPEL2;TNRC6B
26B;CAMK4;FAM171B;SLC28A3

DC1;SCN5A;TEAD1;PRKG1;CCDC112;HS3ST3B1;GPR37;MME;GRID1;GAB3;SORCS1;IL17RD;ADRA2A

2;NUPL1

C2;PCDHAC1;EOMES;RAB2B;C21ORF59;ANGEL2;DYRK1A;CNPY1;ARID5B;PCDHA13;SHISA9;PCDHA
BGRL2;SCN5A;METTL16;RBM7;CTBS;TEAD1;APPL1;UTP15;KLF10;GPR37;WDR37;NSUN3;ITPK1;GAB

FIA;ASXL3;SPRY1;YPEL2;TNRC6B
EA3
E2;SLC29A3;NAPA;PHLPP2;AMER1;USP15;MYRF;ZDHHC20;TUBD1;ZDHHC21;PAPSS2;PURA;ZNRF2;I
3A;GAB1;LRRC40;SLC39A13;PROX1;SENP2;DIEXF;TMEM33;SNPH;CNKSR3;NOS1AP;PGAM5;ST6GAL

CD244
TNNB1;FAM171B

A;ZNF318;ANKRD40;SHC4;SHC2;BHLHB9;INSIG2;DLX6;RNF180;SPATA2;NCKIPSD;BCL2L13;EFNB3;G3
EL2;TNRC6B
N1A2;TRPS1;TRIM3;TMEM108;SH3BGRL2;SOSTDC1;ZNF367;UTP15;ARHGEF10;GPR37;WDR37;NSUN

L2;TNRC6B

A;NFIA;ASXL3;YPEL2;TNRC6B

2;TNRC6B

R16C5;GRM5;ADAMTS2;JPH3;MECOM;SH3PXD2A;MAN1A2;RSPO2;TMEM108;SH3BGRL2;SCN5A;EMB
CDHB15;GATC;PLEKHA3;HIPK1;SORBS3;ADAT2;LETM1;TTC9;DPY19L2;PDP2;GPAM;NFIA;RFWD3;CAF
A;JPH1;LITAF;RND3;ELAVL2;SDR16C5;NHSL2;KIAA1549;GRM5;JPH3;SH3PXD2A;NKRF;SLC22A17;PGM

3;LUZP1;PAPSS2;NEURL1B;PURA;NXF1;SERTAD2;ERBB4;CARNS1;NT5DC3;S1PR1;GPC5;SLIT2;LRIG
3;ALAD;ADAMTS5;MED14;NHSL2;SPRED1;NKRF;SH3BGRL2;KIF1B;CEP170;SCN5A;RBM7;ZNF367;HS3

R37;WDR37;NSUN3;C3ORF62;AHSA2;ZFP91;PROX1;ZFY;ZFX;CDYL2;ADRA2A;KAT2B;RAP2A;RAP2B;I
:FX;ADRA2B;CDYL2;RAP2C;KAT2B;ERGIC2;CRK;MAPRE2;SLC29A3;DCP2;NAPA;PHLPP2;CRP;AMER1
?37;WDR37;TMED8;GRID1;VPS13C;C3ORF62;DNMT3A;AHSA2;GAB1;SORD;LRRC40;HAUS3;C6ORF62

'3A;GAB1;SORCS1;PAX2;ADRA2A;ENAH;TGFBR3;NRG3;FAM84A;CLDN18;NAA38;PDE5A;SMTNL2;NAT
)G;S1PR1;VPS54;GPC5;MPZL3;ATOH1;SEPT12;RANBP3;KCNIP2;FAM46C;GABRA3;SUSD1;KLK13;PCD

S3ST3B1;GRID1;C3ORF62;GAB1;ZFP91;PAX5;PROX1;SENP2;PAX2;PTP4A1;ZNF236;LPHN2;TMEM106

'EL2;C11ORF57;ATP8A2;TMEM167B;KLHL32;ZBTB20;LOXL4;RORB;RIMS3;FADS6;KIAA0895;PIM3;ZNF
PH;IL13RA1;PCDHAC1;RAB2B;C21ORF59;USP2;VASH2;CNPY1;FTSJ2;PCDHA12;FOXP2;MAF;FAM185
ADAMTS2;JPH3;SLC22A17;RSPO2;TMEM108;SOSTDC1;PRKG1;HS3ST3B1;GAB3;ADRA2B;ADRA2A;P

GAB3;SORCS1;PAX5;IL17RD;ADRA2B;DIEXF;PAX2;ADRA2A;KAT2B;NRG3;IL1RAPL1;CLDN18;MAPRE
B;CARNS1;HAS2;GPC5;MBNL3;JAM2;IGBP1;GABRA1;SEPT12;USP9X;GABRA3;LGI2;KLK13;ATP2B2;S

H3BGRL2;KIF1B;CEP170;RBM7;UNKL;ZNF367;PHACTR4;UTP15;GPR37;CHST7;GRID1;GAB1;ANO6;ZI

PRRX1;B3GAT2;SUSD1;YLPM1;PLEKHA3;HIPK1;TTC9;C20ORF112;NFIA;CAMK4;CTNNB1;PTPN4;TNR
T2;JAM2;GABRA1;B3GAT2;GABRA3;LGI2;ATP2B2;TBX5;SORBS3;STXBP5L;DPY19L2;ASXL3;TCEA3;CD
7;MTSS1L;GTF2H5;STXBP5L;LETM1;MARCKS;CARM1;CCDC6;PDCD4;ASB7;TRIP12;SSBP2;TNRC6B
70;VASH2;DIO2;DYRK1A;TAP2;MOB3C;TBC1D25;ANKRD40;DENND6A;AIPL1;WDFY4;SHC4;PSMD11;F
6A5;SMTNL2;NAT8L;MAPRE2;SLC29A3;RNF165;HDAC5;PPM1L;HPGD;PALM2;PRUNE2;NDNF;ZDHHC2

S;FAM167A;MYO1C;DPY19L2;CDKN2AIPNL;GPAM;KIF26B;YPEL2

RRC40;ZFY;GPCPD1;CBFA2T2;DIEXF;PTP4A1;DDX19B;SLC25A12;LPHN2;DTL;SOS2;TMEM106A;FYTT
13;ANO6;PAX5;PROX1;IL17RD;ZFX;CDYL2;PTP4A1;KAT2B;RRM2B;SNPH;CNKSR3;NOS1AP;LCOR;RB

C17A6;APBB2;SLIT2;ATOH1;TPBG;SUSD1;LGI2;SULF2;XK;NFIA;TMEM56;KIF26B;ASXL3;TCEA3;SPRY

GD;ZDHHC20;PPM1E;DRP2;PURA;ERBB4;MBNL3;MPZL3;MAP3K2;SUSD1;LAPTM5;FBXL17;RAB11A;S

2;SORBS3;GTF2H5;TSHR;STXBP5L;HEYL;CLCN5;MARCKS;FRAS1;KIF26B;YPEL2

;TBL1XR1;CMPK1;ANKRD40;NPTXR;GLYR1;SHC4;TBC1D19;ABHD4;UBA6;ARL3;CUL3;RHOBTB3;DTX1

TM1;MARCKS;GPAM;CARM1;CAMK4;ASB7;NUPL1

CR10;TRAK2;NEURL1B;TMEM65;TMEM203;SERTAD2;IGF2BP1;S1PR1;S1PR3;MPZL3;SMNDC1;ATOH1
;TRA2B;NSD1;C10ORF2;GPC5;APBB2;MBNL3;MPZL3;EIF4E;JAM2;TRIM44;SEPT12;CRBN;PRRX1;TPB

NSUN3;ITPK1;AHSA2;ZFP91;HAUS3;SLC39A13;SENP2;DIEXF;ADRA2A;SUMF1;RAP2C;FAM101B;RAP2

DHHC20;SRSF1;TUBD1;ZBTB44;PPM1E;PURA;GBX2;CARNS1;E2F1;HAS2;PTK2B;MBNL3;MPZL3;ATO[illegible]

[illegible]A3;KCNRG;SOD2;TPCN1;ITCH;XK;VAPB;PITHD1;KLHL7;CAPZA1;OCIAD1;RAB3GAP2;LNX2

KLE1;ADD3;LRP8;GRAMD4;NEURL1B;ZNRF2;PABPN1;ERBB4;TRA2B;NSD1;S1PR3;BCL2L2-PABPN1;M

P9X;CSNK1A1;CNBP;YLPM1;GTF2H1;KCNRG;DEK;SOD2;GATAD2B;EPOR;ADAT2;LETM1;CLCN5;APC;F

ST6GALNAC3;TMEM106A;DCP2;KANK2;LUZP1;PRUNE2;ADD3;PPM1E;TTL;JAKMIP2;CYTH3;NEURL1E
3GAP2;LNX2

IAD1;RLIM;RAB3GAP2;PTPN4;FRK;LNX2

;TSHR;SULF2;HEYL;TTC9;FAM167A;FRAS1;FAM171B;CD244;C11ORF57

SEPT12;SUSD1;LAPTM5;PLEKHA3;KCNRG;S100B;SYNJ2BP;SORBS3;EPOR;TSHR;STXBP5L;DPY19L2

F1;TMEM203;PABPN1;PCBD2;JAM2;IGBP1;SEPT10;RRM1;TIMMDC1;SORT1;CSNK1A1;KCNIP2;CNBP;
1;KIF26B;CAMK4;FRK

344;FAM117B;LRP6;TRAK2;CYTH3;GNA13;NXF1;MAT2A;TNKS2;PABPN1;RBBP5;TRA2B;ATXN1L;NRIP1
TNKS2;TRA2B;VPS54;MAPK1;GLTSCR1L;LRIG2;EMC7;SRSF10;SMNDC1;MAP3K2;SEPT10;CSNK1A1;C
2;NEURL1B;RXRA;ERBB4;GPC5;TRIM44;PRRX1;SORT1;FAM46C;B3GAT2;SUSD1;LGI2;FOXJ2;ATP2B2
33GAT2;LGI2;ATP2B2;SORBS3;EPOR;STXBP5L;HEYL;TTC9;FAM167A;ASXL3;CAMK4;FAM171B
A;APC;KANSL1;ETNK1;CAPZA1;OCIAD1;CCNYL1;RAB3GAP2;FAM171B;TRIP12;NUPL1

MEM56;KIF26B;TCEA3;FAM171B
TBX5;SORBS3;TSHR;STXBP5L;TTC9;ASXL3;YPEL2

S1AP;LCOR;RBMS1;MAPRE3;SMTNL2;ZNF597;PRTFDC1;KANK2;FYTTD1;RALA;HPGD;SEMA3A;ATL2;Z

SLC24A2;AMER1;USP13;RALA;MYRF;HPGD;PALM2;PPM1H;FAM129A;ZDHHC21;ADD3;PPM1E;TTL;DR

7B;C11ORF57;TNRC6B
19L2;KIF26B;LNX2
;ASXL3;SPRY1;YPEL2;TNRC6B
ADRA2B;PAX2;ADRA2A;ENAH;TGFBR3;NRG3;CNKSR3;FAM84A;NAA38;PDE5A;SMTNL2;NAT8L;LPHN2

K1;TTC9;C20ORF112;CLCN5;FAM167A;NFIA;ASXL3;TNRC6B
OR37;TMED8;NSUN3;C3ORF62;DNMT3A;SORD;LRRC40;HAUS3;ANO6;SMC1A;CDYL2;RBL1;IL1RAPL1;

0ORF112;NFIA;ASXL3;YPEL2;TNRC6B

DYL2;PTP4A1;KAT2B;RAP2B;SNPH;IL1RAPL1;NOS1AP;RBMS1;PRTFDC1;CHST3;FYTTD1;SEMA3A;XIA
RC6B

MT3A;SORD;PROX1;ZFY;CDYL2;SUMF1;PTP4A1;KAT2B;DDX19B;RBL1;SNPH;IL1RAPL1;LCOR;RBMS1;

A;ENAH;NRG3;IL1RAPL1;CNKSR3;FAM84A;RBMS1;PDE5A;COL6A5;LPHN2;MAPRE2;RNF165;PPM1L;HI

.12;PCDHA10;MAF;FAM185A;ADAM12;ELMO1;WDFY3;SCG3;SHC4;DLX1;SHC2;TBC1D19;DLX6;RNF18
31;C6ORF62;GAB3;SENP2;ZFX;DIEXF;SUMF1;RAP2C;TGFBR3;KAT2B;CREB1;FCHSD2;RRM2B;PDE5A

RBBP5;MGAT5;TMEM68;CARNS1;ATXN1L;USP1;FAM63B;HAS2;VPS54;MAPK1;LRIG2;RPP14;MPZL3;J
.NAC3;SLC25A12;NAT8L;SOS2;TMEM106A;PRTFDC1;KANK2;NAPA;USP13;RALA;USP15;PRICKLE1;PD

3BP1;CLSPN;SMURF2;SRD5A1;SREK1IP1;KLHL23;TRAPPC8;BTBD9;FBXO30;SYT7;RCAN1;ARHGAP3

N3;C3ORF62;DNMT3A;LRRC40;ANO6;PAX5;SENP2;CDYL2;ENAH;PTP4A1;KAT2B;SNPH;NOS1AP;LCO

3;CCDC50;PRKG1;GPR135;HS3ST3B1;GPR37;MME;SGIP1;MMP2;PAX5;IL17RD;ADRA2B;ADRA2A;PTP
PZA1;CAMK4;TCEA3;CTNNB1;TARDBP;YPEL2;PRDM8;ANKRD13B;RAB3C;TMEM167B;POGK;KLHL32;
M3;RSPO2;SH3BGRL2;SOSTDC1;SCN5A;GPRASP2;CCDC50;GPR135;HS3ST3B1;GPR37;SGIP1;DNMT

32;MPZL3;SEPT11;TIMMDC1;PDAP1;GTF2H1;ATP2B1;SORL1;GTF2H5;STXBP5L;HEYL;FAM167A;CCD
3ST3B1;UTP15;GPR37;SGIP1;C3ORF62;ANO6;SORCS1;PROX1;CDYL2;KAT2B;RAP2A;RRM2B;NOS1A

IL1RAPL1;NOS1AP;RBMS1;PDE5A;RAPGEF6;MAPRE2;PRTFDC1;CHST3;FYTTD1;RALA;PLAG1;SEMA
I;USP15;HPGD;PLAG1;ZDHHC20;TUBD1;PURA;NXF1;PGRMC1;MGAT5;UBN2;TMEM68;USP1;VPS54;M
2;PAX5;PROX1;ZFX;DIEXF;CDYL2;ENAH;KAT2B;RBMS1;PGAM5;PDE5A;RAPGEF6;PRTFDC1;KANK2;F

T8L;LPHN2;SLC24A2;NAPB;PPM1L;LUZP1;HPGD;PALM2;TYRP1;PPM1H;PPM1E;GRAMD4;TTL;JAKMIP
DHB15;ATP2B4;PDAP1;ATP2B2;GTF2H1;ADAT2;LETM1;HEYL;ITCH;ZNF70;XK;GPAM;ASXL3;CAMK4;EI

A;PRTFDC1;FYTTD1;HDAC5;PPM1L;HPGD;PALM2;PRUNE2;ZDHHC21;ZBTB44;UQCR10;PPM1E;FAM1

F687;IL6R;KCNH1;IL13RA1;EOMES;RAB2B;C21ORF59;ANGEL2;VASH2;DYRK1A;ALG14;ARID5B;FTSJ2
A;ELMO1;WDFY3;SCG3;KANK4;DLX1;BHLHB9;RHOBTB3;SPATA2;SLC1A4;DTX4;TRHDE;FBLN5;ARHG
PTP4A1;NRG3;MYOD1;NOS1AP;FAM84A;COL6A5;SMTNL2;MAPRE2;RNF165;HDAC5;HPGD;PALM2;PRU

3;LPHN2;MAPRE2;RNF165;HDAC5;DCTN2;ADH1B;PPM1K;ADD3;NEURL1B;PGRMC1;ERBB4;GPC5;MP
100B;SORL1;STXBP5L;MARCKS;DPY19L2;FRAS1;ASXL3;CCNYL1;FAM171B

FX;CDYL2;KAT2B;RAP2A;RAP2B;RRM2B;LCOR;RBMS1;MAPRE3;NAT8L;DTL;MAPRE2;PRTFDC1;CHST

C6B
D244

AM114A1;RNF180;NCKIPSD;BCL2L13;RAP1A;TMEM248;IP6K1;PLXNA3;CPSF7;ZBTB14;SMURF2;ZBTE
21;PPM1E;COL19A1;ADD2;JAKMIP2;GBX2;ERBB4;CARNS1;SLC17A6;GPC5;MBNL3;ATOH1;B3GAT2;LC

D1;RALA;USP15;STAU1;LUZP1;PLAG1;SEMA3A;ATL2;ZBTB44;MAT2B;UQCR10;ADD3;PAPSS2;TRAK2
MS1;MAPRE3;ZNF236;ST6GALNAC3;PAFAH1B2;PRTFDC1;CHST3;FYTTD1;HPGD;SEMA3A;ATL2;NRXI

1

ULF2;ADAT2;LETM1;APC;CAPZA1;TCEA3;RAB3GAP2;FAM171B;PDE7B;OTC;TNRC6B

1;NPAT;ARHGAP20;IP6K1;PLXNA3;CPSF7;EIF2B2;ZBTB14;TRAPPC8;HELZ;HOOK3;ESR1;FBXO30;RC/

1;SLC25A24;IGBP1;SEPT10;SORT1;KCNIP2;FAM46C;SPRYD7;FOXJ2;LAPTM5;PDAP1;ATP2B1;U2SURI
3G;ATP2B2;GTF2H1;SYNJ2BP;EPOR;TSHR;STXBP5L;SULF2;MAPK10;KIF26B;SPRY3;SPRY1;NUPL1

2A;FCHSD2;TMEM33;NOS1AP;NAA35;MAPRE3;SMTNL2;SLC25A12;ERGIC2;MAPRE2;TMEM106A;SLC2

H1;MAP3K2;FOXJ2;PLEKHA3;TPCN1;LETM1;DPY19L2;APC;FAM171B;PDE7B;NUPL1

MPZL3;EIF4E;ATOH1;CA12;IGBP1;RRM1;SEPT12;CRBN;GABRA3;SUSD1;FOXJ2;KLK13;C1ORF21;ASP

PITHD1;KLHL7;SERBP1;OCIAD1;RLIM;CTNNB1;TARDBP

B;RXRA;PTK2B;GPC5;GLTSCR1L;EIF4E;AAED1;RRM1;SEPT12;PCGF3;SUSD1;ATP2B4;SOD2;CDC42B

2;NFIA;PITHD1;TMEM56;ASXL3;CAMK4

YLPM1;GATC;PDAP1;KCNRG;SORL1;GATAD2B;VAPB;PITHD1;KLHL7;CAPZA1;OCIAD1;RAB3GAP2;LN[illegible]

1;FAM63B;APBB2;SMNDC1;TRIM44;MAP3K2;CRBN;CSNK1A1;TXNL1;PCGF3;FOXJ2;GATC;PDAP1;PLE
CNBP;YLPM1;PDAP1;GTF2H1;PTPN11;TMEM64;HIPK1;CDC42BPA;GATAD2B;HIPK2;XK;GPAM;VAPA;PI
2;HEYL;TMEM56;KIF26B;ASXL3;CAMK4

ZDHHC21;ZBTB44;PPM1E;FAM117B;COL19A1;PAPSS2;DFNB59;ZNF629;UBN2;PTK2B;VPS54;BCL2L2-

RP2;GPC1;HAS2;GPC5;S1PR3;JAM2;SLC25A24;GALNT7;IGBP1;SEPT10;GABRA1;USP9X;GABRA3;LGI

;RNF165;SLC24A2;NAPB;PPM1L;LUZP1;HPGD;PPM1H;PRICKLE1;PPM1E;GRAMD4;TTL;COL19A1;ADI

;CNKSR3;NOS1AP;NAA35;PGAM5;ST6GALNAC3;SLC25A12;PAFAH1B2;PRTFDC1;NAPB;RALA;DCTN2

AP;ZDHHC21;ZBTB44;FAM117B;ZNRF2;DFNB59;ZDHHC18;EFS;ZNF629;MAT2A;UBN2;NRIP1;HAS2;VP

;ST6GALNAC3;PAFAH1B2;PRTFDC1;FYTTD1;PALM2;ATL2;ZDHHC21;ZBTB44;PPM1E;FAM117B;DFNB5

PGD;SEMA3A;PRUNE2;PPM1H;NDNF;PRICKLE1;PPM1E;PAPSS2;LRP6;EFS;GBX2;TMEM65;ZNF629;E

0;CUL3;NPR3;RHOBTB3;SPATA2;ARHGAP20;ATXN7;ZMAT3;G3BP1;CLSPN;CBX6;EIF2B2;SMURF2;ZB
;SLC25A12;NAT8L;SOS2;PRTFDC1;HDAC5;USP15;LUZP1;TUBD1;PDHB;UQCR10;PPM1E;LRP6;PPP2

AM2;S100PBP;SLC25A24;MAP3K2;TRIM41;CSNK1A1;KCNIP2;GATC;TMEM64;DEK;SULF2;TPCN1;LET
HB;UQCR10;TRAK2;NEURL1B;BCLAF1;ZNRF3;TMEM203;TNKS2;RBBP5;TMEM68;HAS2;C10ORF2;VP

1;SP1;LHX6;LRP12;RAD9A;FGFR1;DYRK3;TNFAIP8;BMPR2;SNAP23;PPWD1;TNFAIP3;CTDSPL2;PITP

R;RBMS1;ST6GALNAC3;RAPGEF6;TMEM106A;PRTFDC1;CHST3;FYTTD1;HDAC5;PLAG1;PALM2;DLS

4A1;NRG3;MYOD1;FAM84A;PDE5A;CD47;COL6A5;SMTNL2;MAPRE2;CHST3;RNF165;SLC24A2;FYTTD
MRFAP1;ZBTB20;RORB;CTGF;AMOT;CDH6;EFEMP1;RIMS3;SESN3;CDH2;KIF5C;KIAA0895;SNIP1;IL6
3A;SORCS1;PROX1;ADRA2A;NRG3;SNPH;COL6A5;SMTNL2;LPHN2;PRTFDC1;CHST3;SLC24A2;FYTT

6;ASB7;FAM171B;TRIP12;PDE7B;LNX2
P;ST6GALNAC3;TMEM106A;PRTFDC1;CHST3;FYTTD1;HDAC5;PPM1L;PLAG1;PPM1H;ZBTB44;MAT2B

.3A;ZBTB44;UQCR10;FAM117B;PAPSS2;TRAK2;C21ORF119;DRP2;NEURL1B;ZNRF3;ZNF629;TMEM203
IAPK1;RPP14;MPZL3;TRIM44;MAP3K2;TRIM41;CRBN;SUSD1;TMEM64;DEK;RAB11A;ADAT2;LETM1;PD
YTTD1;RALA;PPM1L;SEMA3A;ZDHHC21;ZBTB44;MAT2B;PDHB;LRP8;PPM1E;LRP6;TRAK2;C21ORF11

?2;NEURL1B;RXRA;GPC1;NSD1;SLC17A6;GPC5;APBB2;JAM2;TRIM44;ATOH1;SLC25A24;TPBG;LGI2;S
F3J;CCDC6;SPRY3;ASB7;CTNNB1

I17B;TRAK2;ZNF629;RBBP5;GPC1;UBN2;TMEM68;S1PR1;HAS2;GPC5;MBNL3;MPZL3;SMNDC1;ATOH1

;RBX1;MAF;FAM185A;ZNF318;WDFY3;SCG3;SRSF9;BHLHB9;UBA6;TNKS;SPATA2;CREBL2;C17ORF96
GAP20;ATXN7;ZMAT3;RAI2;PLXNA3;MARK1;MGAM;CPSF7;SRD5A1;KLHL23;TRAPPC8;BFAR;SYT7;REI
UNE2;FAM129A;NDNF;HOXD12;ADD3;COL19A1;ADD2;CYTH3;ZNRF3;EFS;ERBB4;CARNS1;HAS2;SLC

?ZL3;SORT1;B3GAT2;GABRA3;LGI2;HIPK2;SULF2;TTC9;RAB3GAP2;SSBP2

T3;PPM1L;PLAG1;PPM1H;TUBD1;ZBTB44;UQCR10;FAM117B;TRAK2;NEURL1B;DFNB59;ZNRF3;ZNF62

316;HELZ;BTBD9;FBXO30;AZIN1;ABHD15;CNOT6;EFNA3;PQLC1;ZFP62;UBE2K;NFE2L1;PLEKHM3;LRI
GI2;ATP2B2;SORL1;SORBS3;TSHR;STXBP5L;SULF2;TTC9;FAM167A;FRAS1;ASXL3

;DRP2;OGFRL1;DFNB59;ZNF629;MAT2A;TNKS2;RBBP5;TDG;UBN2;C10ORF2;PTK2B;PCBD2;LRIG2;RI
N3;TUBD1;ZBTB44;PRICKLE1;TTL;FAM117B;DFNB59;RXRA;TMEM65;ZNF629;MAT2A;RBBP5;GPC1;TD

AN1;ARHGAP31;SP4;LHX4;MXD1;UBE2K;NFE2L1;PLEKHM3;DYRK3;PRR3;SNAP23;PPWD1;RND3;CKS

P;GTF2H5;STXBP5L;LETM1;TTC9;FAM167A;KIF26B;CARM1;TCEA3;FAM171B;NUPL1;TNRC6B

29A3;DCP2;KANK2;PHLPP2;AMER1;RALA;ZDHHC20;TUBD1;ZBTB44;PDHB;UQCR10;PAPSS2;TRAK2;N

'RV1;EPOR;TSHR;STXBP5L;HEYL;XK;DPY19L2;FRAS1;PDP2;ETNK1;SPRY1;TARDBP;NUPL1

;PA;ASPRV1;SORL1;SORBS3;EPOR;TSHR;STXBP5L;ADAT2;TPCN1;LETM1;MAPK10;HEYL;ITCH;FRAS

X2

:KHA3;GTF2H1;PTPN11;DEK;SYNJ2BP;CDC42BPA;GATAD2B;LETM1;NECAP2;ITCH;VAPA;ETNK1;CARI
THD1;TMEM56;ETNK1;CAPZA1;SERBP1;OCIAD1;CCNYL1;RAB3GAP2;NUPL1;LNX2

-PABPN1;MPZL3;SMNDC1;JAM2;IGBP1;SEPT10;USP9X;SORT1;SUSD1;YLPM1;PCDHB15;PLEKHA3;C1

2;MTSS1L;SORL1;SORBS3;STXBP5L;MARCKS;XK;DPY19L2;KIF26B;ASXL3;CAMK4;TCEA3;FAM171B;S

D2;JAKMIP2;NEURL1B;ERBB4;GPC1;NSD1;SLC17A6;GPC5;S1PR3;SLIT2;JAM2;ATOH1;SEPT10;TPBG;

;NUFIP2;TUBD1;ZDHHC21;PURA;OGFRL1;NXF1;TMEM203;TDG;ATXN1L;HAS2;MAPK1;S1PR3;PCBD2

S54;MAPK1;S1PR3;SLIT2;MBNL3;MPZL3;CA12;ZNF462;PCDHB15;PDAP1;PLEKHA3;GTF2H1;PTPN11;I

59;EFS;ZNF629;MAT2A;TDG;UBN2;NSD1;HAS2;C10ORF2;VPS54;MAPK1;PCBD2;SLIT2;MPZL3;JAM2;CA

:RBB4;GPC1;CARNS1;IGF2BP1;S1PR1;HAS2;GPC5;SLIT2;ATOH1;SLC25A24;SEPT10;GABRA1;PRRX1

TB16;SREK1IP1;HELZ;BTBD9;DIAPH2;ARHGAP31;SP1;JMY;CDK1;FGFR1;DYRK3;BMPR2;TTC22;SNAF
CA;GNA13;PURA;NXF1;GBX2;TMEM203;UBN2;TMEM68;NT5DC3;NRIP1;PTK2B;VPS54;MAPK1;LRIG2;C

M1;XK;FRAS1;PDP2;APC;KIF26B;KLHL7;CAPZA1;TARDBP;NUPL1;OTC;TNRC6B
'S54;MPZL3;SMNDC1;TRIM44;S100PBP;SEPT12;SORT1;GTF2H5;ADAT2;HEYL;ITCH;FAM167A;XK;DPY

NC1;DTWD1;PSD4;TRPS1;FAM154B;SCN5A;RBM7;CCDC50;PHACTR4;UTP15;SENP2;PTP4A1;RRM2B;

T;ZDHHC21;ZBTB44;UQCR10;GRAMD4;FAM117B;NEURL1B;NXF1;EFS;ZNF629;TMEM203;ERBB4;TDG;

)1;PPM1L;HPGD;PALM2;PRUNE2;FAM129A;NDNF;ZDHHC21;HOXD12;PPM1E;COL19A1;ERBB4;CARNS
R;SH3GL2;PCDHAC2;C21ORF59;USP2;DYRK1A;GPR75;ZBTB34;FTSJ2;PCDHA10;BCMO1;GPRIN3;MAF
D1;USP13;HPGD;PALM2;NDNF;PPM1E;COL19A1;JAKMIP2;EFS;GPC1;S1PR1;HAS2;SLC17A6;GPC5;SI

;UQCR10;FAM117B;COL19A1;TRAK2;NEURL1B;DFNB59;TMEM203;UBN2;TMEM68;HAS2;PTK2B;MAPK

3;MAT2A;TNKS2;RBBP5;TDG;UBN2;C10ORF2;MAPK1;PCBD2;MBNL3;MPZL3;CA12;ZNF462;SEPT11;TR
)P2;APC;TMEM56;OCIAD1;ASB7;SPRY1;SSBP2;OTC;TNRC6B
9;ZNRF2;ZNF629;TMEM203;TNKS2;RBBP5;ERBB4;TDG;UBN2;VPS54;MPZL3;JAM2;ATOH1;GALNT7;CA

ORL1;SULF2;MAPK10;NECAP2;MARCKS;NFIA;KIF26B;ASXL3

1;CA12;ZNF462;GATC;PTPN11;DEK;RAB11A;SULF2;ADAT2;HEYL;TTC9;FAM167A;ZNF70;XK;DPY19L2;I

;NPAT;ATXN7;G3BP1;CLSPN;IP6K1;PLXNA3;SMURF2;ZBTB16;GFPT1;SREK1IP1;TRAPPC8;HOOK3;LS
EP1;RCAN1;ARHGAP31;SP1;MXD1;RAD9A;BMPR1A;SNAP23;DUSP19;CTDSPL2;PPP1R9A;PITPNC1;E
:17A6;GPC5;ATOH1;CA12;PRRX1;KCNIP2;TPBG;B3GAT2;LGI2;PCDHB15;ATP2B2;TBX5;ASPRV1;SORL

29;MAT2A;UBN2;NT5DC3;MAPK1;PCBD2;SLIT2;LRIG2;RPP14;MBNL3;GALNT7;RANBP3;USP9X;SORT1

P12;RBM28;ALAS2;BMPR2;SNAP23;PPWD1;AFF4;AFF1;PITPNC1;LMOD3;ALAD;CAPZB;ALDH2;SIN3A;

PP14;MPZL3;JAM2;TRIM44;GALNT7;ZNF462;TRIM41;TIMMDC1;RANBP3;USP9X;YLPM1;GATC;PDAP1;
G;UBN2;CARNS1;MAPK1;PCBD2;MBNL3;MPZL3;SLC25A24;CA12;IGBP1;SEPT10;SPRYD7;SUSD1;PD

[illegible]1B;ARL5B;CAPZB;PGM3;RBM7;CNPPD1;PHACTR4;UTP15;KLF10;VPS13C;SORD;HAUS3;TGFBR3;CR

[illegible]NEURL1B;PURA;PGRMC1;SERTAD2;RBBP5;MGAT5;UBN2;NT5DC3;LRIG2;RPP14;SMNDC1;MAP3K2;I[illegible]

1;PDP2;PITHD1;KLHL7;CAMK4;CTNNB1;YPEL2

M1;CAPZA1;SERBP1;TRIP12

ORF21;HIPK1;CDC42BPA;U2SURP;GTF2H5;RAB11A;TSHR;SULF2;HEYL;TTC9;DPY19L2;FRAS1;TCEA

SPRY1;PDE7B

;B3GAT2;SULF2;NECAP2;MARCKS;FRAS1;KIF26B;ASXL3;TCEA3

;LRIG2;RPP14;MPZL3;SRSF10;JAM2;CSNK1A1;FAM46C;FOXJ2;PCDHB15;GATC;GTF2H1;TMEM64;SO

HIPK1;TTC9;C20ORF112;FRAS1;NFIA;ASXL3;LNX2;TNRC6B

A12;SEPT10;TRIM41;USP9X;SORT1;B3GAT2;YLPM1;PCDHB15;PLEKHA3;PTPN11;TMEM64;TBX5;HIPK

;SORT1;TPBG;B3GAT2;LGI2;MTSS1L;C1ORF21;SOD2;SORBS3;EPOR;STXBP5L;HIPK2;FAM167A;DPY

P23;DUSP19;TNFAIP3;CTDSPL2;PPP1R9A;RND3;PITPNC1;ELAVL2;ADAMTS5;DTWD1;SPRED1;SART3
GALNT7;SEPT12;SPRYD7;KLK13;ATP2B4;GATC;PDAP1;PLEKHA3;GTF2H5;HEYL;CLCN5;ZNF70;XK;CD

Y19L2;GPAM;RFWD3;CAPZA1;CCDC6;CCNYL1;ASB7;FAM171B

;SNPH;NOS1AP;LCOR;RBMS1;SLC25A12;CRK;SOS2;PPM1L;PLAG1;NDNF;ZBTB44;C21ORF119;ZNF6

;UBN2;NSD1;C10ORF2;MAPK1;PCBD2;SLIT2;MBNL3;ZNF462;LGI2;GATC;PDAP1;PLEKHA3;HIPK1;LET

S1;SLC17A6;GPC5;SLIT2;MBNL3;JAM2;ATOH1;SLC25A24;CA12;GABRA1;PRRX1;KCNIP2;TPBG;B3GA
F;C1ORF115;KIAA1614;STIM2;FAM185A;ADAM12;ZNF318;WDFY3;ANKRD40;PPIC;SHC4;DLX1;TBC1D1
LIT2;JAM2;ATOH1;CA12;GABRA1;PRRX1;USP9X;TPBG;B3GAT2;KLK13;TBX5;EPOR;TSHR;STXBP5L;T

K1;APBB2;PCBD2;MBNL3;MPZL3;CA12;TRIM41;RANBP3;SORT1;LGI2;PCDHB15;PDAP1;PLEKHA3;GTF

RIM41;SORT1;B3GAT2;SUSD1;GATC;PDAP1;PTPN11;TMEM64;HIPK1;ADAT2;LETM1;TTC9;FAM167A;XK

A12;IGBP1;SEPT10;TIMMDC1;RANBP3;KCNIP2;SUSD1;YLPM1;KLK13;GATC;PDAP1;TMEM64;HIPK1;AI

PITHD1;CAMK4;TCEA3;CCDC6;TRIP12;TARDBP;C11ORF57

SM5;BTBD9;SYT7;SMAD7;CNOT6;SP1;JMY;LSM14B;SERINC3;DNAL1;PLEKHM3;LRP12;RAD9A;TNFAIP
ELAVL2;DTWD1;KIAA1549;SIN3A;TMEM108;SCN5A;RBM7;UNKL;UTP15;C3ORF62;DNMT3A;SORD;PTP
L1;SORBS3;TSHR;HEYL;TTC9;FAM167A;DPY19L2;FRAS1;ASXL3;TCEA3

;PCGF3;GATC;PDAP1;PTPN11;TMEM64;HIPK1;GTF2H5;RAB11A;TTC9;ITCH;FAM167A;ZNF70;GPAM;N

UNKL;MAPK1IP1L;NAA30;HAUS3;GPCPD1;RRM2B;NOS1AP;ST6GALNAC3;SLC25A12;CRK;HDAC5;MA

PTPN11;TBX5;GTF2H5;ITCH;MARCKS;CDKN2AIPNL;GPAM;APC;RFWD3;CAPZA1;PTPN4;C11ORF57
AP1;GTF2H1;PTPN11;CDC42BPA;SORBS3;HIPK2;LETM1;TTC9;XK;DPY19L2;FRAS1;NFIA;CCDC6;SPR

EB1;RBL1;RRM2B;NAA35;TERF2IP;NAA38;ST6GALNAC3;SLC25A12;SOS2;HDAC5;RALA;PPM1L;ROC

BP1;SEPT11;TRIM41;SUSD1;FOXJ2;ATP2B4;GATC;PDAP1;GTF2H1;TMEM64;HIPK1;CDC42BPA;U2SU

\3;CCDC6;FAM171B;TARDBP;C11ORF57;TNRC6B

)D2;HIPK1;U2SURP;LETM1;HEYL;NECAP2;ZNF70;DPY19L2;PDP2;VAPB;CCNYL1;PTPN4

<1;CDC42BPA;SULF2;TTC9;C20ORF112;FAM167A;XK;FRAS1;NFIA;ASXL3;CAPZA1;TARDBP;YPEL2;TN

'19L2;KIF26B;ASXL3;CTNNB1;SPRY1

3;FAM154B;CCDC50;PRKG1;HS3ST3B1;ADRA2A;PTP4A1;RBL1;SNPH;IL1RAPL1;LCOR;NAT8L;HPGD;P
)KN2AIPNL;GPAM;NFIA;KLHL7;CAMK4;CCDC6;PDCD4;CCNYL1;FAM171B;TRIP12;TARDBP

29;MAT2A;RBBP5;TDG;IGF2BP1;VPS54;PCBD2;SEPT12;LGI2;ATP2B4;PDAP1;ATP2B2;PTPN11;TSHR;C

M1;TTC9;XK;DPY19L2;FRAS1;RFWD3;ASXL3;CAMK4;SPRY3;YPEL2;TNRC6B

T2;GABRA3;SUSD1;ATP2B2;TBX5;SORBS3;TSHR;SULF2;MYO1C;XK;DPY19L2;KIF26B;TCEA3
19;UBA6;DLX6;NPR3;SPATA2;SLC1A4;DTX4;C17ORF96;ARHGAP20;ATXN7;LONRF3;CLSPN;CBX6;CPS
TC9;MYO1C;XK;DPY19L2;FRAS1;KIF26B;CAMK4;TCEA3;FAM171B;NUPL1;OTC

F2H1;PTPN11;HIPK1;LETM1;TTC9;C20ORF112;FAM167A;XK;DPY19L2;GPAM;RFWD3;ASXL3;CAPZA1;S

<;DPY19L2;FRAS1;NFIA;RFWD3;ASXL3;PTPN4;TNRC6B

DAT2;LETM1;HEYL;C20ORF112;ZNF70;XK;DPY19L2;PDP2;CDKN2AIPNL;GPAM;KIAA0101;KIF26B;CAM

?8;BMPR2;ANKRD33B;TTC22;SNAP23;DUSP19;PPWD1;TNFAIP3;CTDSPL2;PITPNC1;KIAA1549;ARL5B
'4A1;RBL1;NOS1AP;TERF2IP;ST6GALNAC3;DTL;PRTFDC1;HDAC5;USP15;HPGD;PLAG1;PALM2;ZBTB

FIA;EIF3J;CCDC6;TRIP12;YPEL2;TNRC6B

AT2B;LRP6;CYTH3;AP3M2;GNA13;ZNRF3;TDG;S1PR1;FAM63B;VPS54;LRIG2;EIF4E;SMNDC1;TRIM44;S

RY3;PDE7B;YPEL2;TNRC6B

K1;ZBTB44;PPM1E;TTL;TMEM203;MAT2A;TNKS2;RBBP5;NT5DC3;PCBD2;GALNT7;IGBP1;SEPT11;PDA

IRP;GTF2H5;RAB11A;ADAT2;TPCN1;LETM1;PDP2;ETNK1;KLHL7;CAPZA1;ASB7;TRIP12;TNRC6B

IRC6B

ꞋALM2;NDNF;ZBTB44;ZNRF3;EFS;MAT2A;NRIP1;VPS54;MAPK1;S1PR3;SLIT2;ATOH1;GALNT7;B3GAT2

C20ORF112;FAM167A;ASXL3;ASB7;SSBP2;TNRC6B

ƽF7;EIF2B2;ZBTB16;KLHL23;SYT7;DIAPH2;ARHGAP31;SP1;OSTC;CAPRIN1;RAD9A;BMPR1A;FGFR1;T

ƽPRY3;YPEL2;TNRC6B

1K4;FAM171B;YPEL2;C11ORF57;TNRC6B

;SPRED1;MAN1A2;TMEM108;SCN5A;METTL16;RBM7;UTP15;ARHGEF10;VPS13C;DNMT3A;HAUS3;SE
44;PDHB;PPM1E;ZNF629;TMEM203;S1PR1;VPS54;ATOH1;SEPT11;TRIM41;PDAP1;ASXL3;SPRY3;SPR

SLC25A24;SEPT12;ATP2B4;FBXL17;SOD2;CDC42BPA;U2SURP;EPOR;NECAP2;MYO1C;VAPA;KANSL1

\P1;U2SURP;TPCN1;FRAS1;CARM1;ASB7;PDE7B;SSBP2;LNX2;TNRC6B

;ATP2B4;TSHR;C20ORF112;FRAS1;ASXL3;[illegible]

NFAIP8;BMPR2;SNAP23;PDE3B;DUSP19;T[illegible]

:NP2;TGFBR3;FCHSD2;RRM2B;SNPH;NOS[illegible]
:Y1;TNRC6B

[illegible];CARM1;OCIAD1;PDCD4;SPRY1;SSBP2;M[illegible]
