# Supplementary material for: Circulating miR-330-3p in Late Pregnancy is Associated with Pregnancy Outcomes Among Lean Women with GDM
Source: Sci Rep. 2020 Jan 22;10:908. doi: 10.1038/s41598-020-57838-6 (PMC6976655; doi:10.1038/s41598-020-57838-6)
Supplement: Supplementary file 1 — Supplementary data. [file 41598_2020_57838_MOESM1_ESM.zip › Supplimentary File_EnrichR_Analysis EnrichR_Summary.pdf]

EnrichR_Summary

Term	Overlap	P.value
**Enrichr_Libraries_Most_Popular_Genes**		
TargetScan_microRNA	35/127	1,21082E-16
TargetScan_microRNA_2017	27/101	8,33131E-13
miRTarBase_2017	24/102	2,81454E-10
Transcription_Factor_PPIs	17/105	2,5557E-05
Virus_Perturbations_from_GEO_down	18/119	3,83562E-05
KEA_2015	17/114	7,49444E-05
ChEA_2016	16/104	8,26667E-05
GO_Biological_Process_2017b	15/100	0,000182415
VirusMINT	21/186	0,000633831
ChEA_2015	14/104	0,000904356
**GO_Molecular_Function_2018**		
ubiquitin-like protein ligase activity (GO:0061659)	25/187	1,22731E-05
transcription coactivator activity (GO:0003713)	33/292	2,06855E-05
ubiquitin protein ligase activity (GO:0061630)	25/193	2,12508E-05
transcription regulatory region DNA binding (GO:0044212)	39/375	2,64135E-05
transcriptional activator activity, RNA polymerase II core promoter proximal reg	22/176	0,00011098
core promoter proximal region sequence-specific DNA binding (GO:0000987)	30/279	0,000120193
ubiquitin-protein transferase activity (GO:0004842)	40/418	0,00013251
nuclear hormone receptor binding (GO:0035257)	11/57	0,000135674
transcription factor activity, RNA polymerase II core promoter proximal region s	30/281	0,000136555
RNA polymerase II transcription factor binding (GO:0001085)	17/122	0,000175778
**GO_Cellular_Component_2018**		
cytoplasmic stress granule (GO:0010494)	10/42	4,15801E-05
bicellular tight junction (GO:0005923)	13/73	8,21235E-05
nuclear body (GO:0016604)	54/619	0,000116896
caveola (GO:0005901)	9/57	0,002412653
early endosome (GO:0005769)	22/223	0,002772087
nucleoplasm part (GO:0044451)	34/408	0,004191642
cytoplasmic vesicle (GO:0031410)	20/216	0,008403976
RISC complex (GO:0016442)	3/9	0,009102597
RNAi effector complex (GO:0031332)	3/9	0,009102597
chromatin (GO:0000785)	25/297	0,01137395
**GO_Biological_Process_2018**		
negative regulation of transcription, DNA-templated (GO:0045892)	84/814	9,3087E-10
positive regulation of transcription, DNA-templated (GO:0045893)	103/1121	6,78218E-09
positive regulation of nucleic acid-templated transcription (GO:1903508)	58/503	8,34905E-09
regulation of transcription from RNA polymerase II promoter (GO:0006357)	125/1479	2,24254E-08
nervous system development (GO:0007399)	51/456	1,75869E-07
generation of neurons (GO:0048699)	23/131	2,30365E-07
positive regulation of transcription from RNA polymerase II promoter (GO:0045	78/849	5,13194E-07
regulation of transcription, DNA-templated (GO:0006355)	127/1599	5,52827E-07
positive regulation of gene expression (GO:0010628)	71/772	1,62614E-06
negative regulation of gene expression (GO:0010629)	60/619	2,02412E-06

Chromosome_Location_hg19

chr5	74/1265	0,1405225
chr4_ctg9_hap1	1/6	0,2723344
chr10	55/1074	0,5443952
chr18	20/411	0,6393224
chr17_ctg5_hap1	1/22	0,6884397
chr4	47/1031	0,8334113
chr14	40/898	0,8550908
chrX	51/1137	0,8715042
chr3	69/1513	0,8775363
chr2	77/1751	0,9439353

OMIM_Disease

hypogonadism	4/15	0,006088819
adenoma	3/14	0,03254043
alopecia	2/11	0,107443713
ehlers-danlos	2/11	0,107443713
hypothyroidism	2/11	0,107443713
rheumatoid_arthritis	2/13	0,142525984
migraine	2/16	0,198607672
myocardial_infarction	2/16	0,198607672
leukemia	6/78	0,214282986
thyroid_carcinoma	2/17	0,217857207

OMIM_Expanded

hypogonadism	11/90	0,006531759
coloboma	10/86	0,012982942
coronary_artery_disease	9/87	0,035222731
alopecia	9/88	0,03751493
migraine	9/93	0,050500138
myocardial_infarction	9/93	0,050500138
cataract	11/124	0,055761461
lateral_sclerosis	9/96	0,05955625
bare_lymphocyte_syndrome	8/83	0,064178868
kallmann_syndrome	8/83	0,064178868

ENCODE_Histone_Modifications_2015

H3K79me2_heart_mm9	168/2000	8,45551E-11
H3K79me2_liver_mm9	161/2000	4,99403E-09
H3K27me3_ES-Bruce4_mm9	145/2000	1,32818E-05
H3K27me3_small intestine_mm9	141/2000	6,90562E-05
H3ac_myocyte_mm9	138/2000	0,00021794
H3K27me3_MCF-7_hg19	75/989	0,000522637
H3K27me3_kidney_mm9	116/1675	0,000627151
H3K27me3_thymus_mm9	133/2000	0,001251038
H3K27me3_bronchial epithelial cell_hg19	136/2082	0,002162405
H3K4me3_H7_hg19	98/1452	0,003595088

CORUM

RICH1/AMOT polarity complex, Flag-Rich1 precipitated (human)	4/9	0,000720595
SMAD7-SMURF2 complex (human)	2/2	0,002660068
SP1-E2F1 complex (mouse)	2/2	0,002660068
ITCH-FAM/USP9x complex (human)	2/2	0,002660068
SNF2L-RSF1 complex (human)	2/2	0,002660068
Grin2b-Lrp8 complex (mouse)	2/2	0,002660068
Car-Lnx2 complex (mouse)	2/2	0,002660068
SNX complex (SNX1, SNX6) (human)	2/2	0,002660068
MMP-2-claudin-1 complex (human)	2/2	0,002660068
Emerin complex 52 (human)	5/23	0,005598899

InterPro_Domains_2019

Cadherin, C-terminal catenin-binding domain	15/37	1,42144E-10
Cadherin, N-terminal	17/65	2,04148E-08
Cadherin-like	19/115	6,43229E-06
WWE domain	5/12	0,000211584
PPM-type phosphatase domain	6/20	0,000385926
RNA recognition motif domain	24/223	0,000545548
PTB/PI domain	8/41	0,001020845
Sortilin, C-terminal	3/5	0,001266489
Sortilin, N-terminal	3/5	0,001266489
VPS10	3/5	0,001266489

KEA_2015

GSK3B	54/527	1,25017E-06
CSNK1G1	5/12	0,000211584
PRKCB	27/250	0,00023972
CDK2	48/553	0,000308248
KSR2	3/4	0,000526873
AKT1	23/210	0,000551991
MAPK12	5/15	0,000704559
CSNK1A1	12/84	0,00121882
CDK4	5/17	0,001331814
CSNK2A1	27/286	0,001876122

miRTarBase_2017

mmu-miR-340-5p	89/576	8,01129E-21
hsa-miR-330-3p	40/146	9,98109E-19
hsa-miR-190a-3p	91/648	1,90724E-18
hsa-miR-19b-3p	96/713	3,75729E-18
mmu-miR-30e-5p	63/366	2,25953E-17
hsa-miR-5011-5p	89/653	3,15672E-17
hsa-miR-106b-5p	124/1091	4,11418E-17
hsa-miR-19a-3p	81/570	7,9292E-17
hsa-miR-93-5p	131/1220	4,79946E-16
hsa-miR-27a-3p	66/429	1,29224E-15

NCI-Nature_2016

Regulation of nuclear SMAD2/3 signaling_Homo sapiens_246aac04-6195-11e	15/81	1,46368E-05

TGF-beta receptor signaling_Homo sapiens_1f188fcc-6196-11e5-8ac5-06603e	10/54	0,000385529
Canonical Wnt signaling pathway_Homo sapiens_9dbe253d-618f-11e5-8ac5-0	6/20	0,000385926
Retinoic acid receptors-mediated signaling_Homo sapiens_5797691b-6195-11	7/28	0,0004346
Signaling events mediated by Hepatocyte Growth Factor Receptor (c-Met)_Ho	12/77	0,000551486
Regulation of Telomerase_Homo sapiens_4dfe97ca-6195-11e5-8ac5-06603eb	11/67	0,000590265
FoxO family signaling_Homo sapiens_d06dbbda-6192-11e5-8ac5-06603eb7f3	9/48	0,000680763
Coregulation of Androgen receptor activity_Homo sapiens_27e0e369-6191-11	10/59	0,000801712
IFN-gamma pathway_Homo sapiens_51b1ed75-6193-11e5-8ac5-06603eb7f30	8/40	0,000860022
FOXM1 transcription factor network_Homo sapiens_c51cda49-6192-11e5-8ac	8/40	0,000860022

NURSA_Human_Endogenous_Complexome

BL2746 (C1D)	14/97	0,00044404
BL4923 (TRIM29)	32/354	0,001540601
a-CLSPN (CLSPN)	24/259	0,004122345
ab_N_YW (RFWD3)	33/394	0,004392394
ab_M_YW (RFWD3)	37/460	0,005168788
BL2378 (RFWD3)	24/268	0,006282132
BL360 (HLTF)	13/115	0,006363472
BL2805 (UBR5)	15/142	0,006643322
BL5230 (TAF2)	7/44	0,006864818
BL963 (ROCK2)	12/104	0,007301214

Panther_2016

Cadherin signaling pathway_Homo sapiens_P00012	23/150	2,68135E-06
Wnt signaling pathway_Homo sapiens_P00057	32/278	1,90014E-05
CCKR signaling map ST_Homo sapiens_P06959	21/165	0,000121807
EGF receptor signaling pathway_Homo sapiens_P00018	15/109	0,000474856
Insulin/IGF pathway-mitogen activated protein kinase kinase/MAP kinase casc	6/29	0,003178531
TGF-beta signaling pathway_Homo sapiens_P00052	11/88	0,005513595
B cell activation_Homo sapiens_P00010	8/57	0,008547798
Pyrimidine Metabolism_Homo sapiens_P02771	3/10	0,01250999
Integrin signalling pathway_Homo sapiens_P00034	14/156	0,03120266
FGF signaling pathway_Homo sapiens_P00021	10/99	0,03158489

Pfam_Domains_2019

Cadherin_2	17/65	2,04148E-08
Cadherin	19/114	5,6382E-06
RRM_1	24/206	0,000167329
zf-RING_2	9/52	0,001241762
Sortilin-Vps10	3/5	0,001266489
Sortilin_C	3/5	0,001266489
WW	7/38	0,002954873
Glyco_hydro_47	3/7	0,004099378
PID	5/26	0,009632009
ZZ	4/17	0,009778786

Pfam_InterPro_Domains

Cadherin_2	17/63	1,22075E-08
Cadherin	19/109	2,84083E-06

PP2C_N	5/19	0,002296337
Znf_RING	21/230	0,008215434
WW_Rsp5_WWP	7/46	0,008772983
Znf_ZZ	4/17	0,009778786
BTB_POZ	12/116	0,01662752
OTU	3/12	0,02123357
PTB_PID	4/22	0,02450931
PDEase	4/22	0,02450931

Phosphatase_Substrates_from_DEPOD

CDC25A	3/8	0,00630859
PTPN1	6/38	0,01246591
PPP2CA	10/88	0,01510233
PTPN11	4/21	0,02088453
PTPRJ	3/17	0,05423619
PPP5C	2/8	0,0605397
DUPD1	1/3	0,14695632
DUSP4	1/3	0,14695632
PTPN7	1/3	0,14695632
PTPRZ1	1/3	0,14695632

PPI_Hub_Proteins

SMAD3	40/328	4,09214E-07
CREBBP	40/334	6,56799E-07
GRIN1	23/149	2,38463E-06
UBC	54/540	2,6161E-06
GSK3B	65/696	2,70501E-06
CRK	28/215	6,27929E-06
AR	30/243	8,7767E-06
SMAD4	28/221	1,06321E-05
CDK2	61/675	1,51392E-05
EP300	38/357	1,99393E-05

Rare_Diseases_GeneRIF_Gene_Lists

Calabro_syndrome	5/12	0,000211584
Parathyroid_carcinoma	23/199	0,000254983
Mental_retardation_X-linked_dysmorphism	21/177	0,000325662
Fragile_X_syndrome	15/107	0,000388092
Kabuki_syndrome	5/15	0,000704559
Corpus_callosum_agenesis	17/138	0,000760493
Chorea_minor	34/369	0,000804123
Sydenham's_chorea	34/369	0,000804123
Rhabdomyosarcoma_embryonal	18/156	0,001168658
Acute_myeloblastic_leukemia_type_2	31/335	0,001249083

Reactome_2016

YAP1- and WWTR1 (TAZ)-stimulated gene expression_Homo sapiens_R-HSA	11/29	9,59953E-08
Developmental Biology_Homo sapiens_R-HSA-1266738	68/786	2,05984E-05
Signaling by WNT in cancer_Homo sapiens_R-HSA-4791275	9/34	4,07577E-05

Signaling by NOTCH1_Homo sapiens_R-HSA-1980143	13/72	7,07991E-05
Axon guidance_Homo sapiens_R-HSA-422475	47/515	0,000112274
Nuclear signaling by ERBB4_Homo sapiens_R-HSA-1251985	9/39	0,000130506
Signaling by Wnt_Homo sapiens_R-HSA-195721	31/295	0,000142116
Signaling by NOTCH_Homo sapiens_R-HSA-157118	17/122	0,000175778
TCF dependent signaling in response to WNT_Homo sapiens_R-HSA-201681	23/199	0,000254983
Diseases of signal transduction_Homo sapiens_R-HSA-5663202	29/288	0,000468209

TargetScan_microRNA_2017

mmu-miR-3061-3p	238/1740	4,37282E-47
mmu-miR-344b	242/1909	4,50804E-42
mmu-miR-1196	209/1517	2,20956E-41
hsa-miR-28-3p	219/1654	1,02113E-40
mmu-miR-221	241/1946	3,8758E-40
mmu-miR-1928	241/1946	3,8758E-40
mmu-miR-222	241/1946	3,8758E-40
mmu-miR-208b	197/1419	2,437E-39
mmu-miR-208a-3p	197/1419	2,437E-39
mmu-miR-669a-3-3p	213/1617	3,62375E-39

WikiPathways_2019_Human

Brain-Derived Neurotrophic Factor (BDNF) signaling pathway WP2380	22/144	4,6682E-06
EGF/EGFR Signaling Pathway WP437	23/162	1,00262E-05
TGF-beta Signaling Pathway WP366	20/132	1,41576E-05
VEGFA-VEGFR2 Signaling Pathway WP3888	28/236	3,59086E-05
ID signaling pathway WP53	6/16	9,5257E-05
Rett syndrome causing genes WP4312	10/48	0,000139497
Signaling of Hepatocyte Growth Factor Receptor WP313	8/34	0,000267181
Angiopoietin Like Protein 8 Regulatory Pathway WP3915	17/132	0,000454164
Androgen receptor signaling pathway WP138	13/90	0,000696619
ErbB Signaling Pathway WP673	13/91	0,000775073

KEGG_2019_Human

TGF-beta signaling pathway	16/90	1,31565E-05
Thyroid hormone signaling pathway	18/116	2,70083E-05
cGMP-PKG signaling pathway	20/166	0,000365494
Neurotrophin signaling pathway	16/119	0,000406905
Wnt signaling pathway	19/158	0,00051929
Long-term potentiation	11/67	0,000590265
Renal cell carcinoma	11/69	0,000762684
Signaling pathways regulating pluripotency of stem cells	17/139	0,000825755
ErbB signaling pathway	12/85	0,001353898
Breast cancer	17/147	0,001541982

Adjusted.P.valu	Ol	Ol	Odds.Ratio	Combined.Sc
1,45298E-14	0	0	5,340902	195,74442
4,99879E-11	0	0	5,180751	144,09525
1,12582E-08	0	0	4,559964	100,27839
0,000766709	0	0	3,137689	33,17981
0,000920549	0	0	2,931405	29,80827
0,001498888	0	0	2,889977	27,45121
0,001417144	0	0	2,981515	28,0283
0,002736219	0	0	2,906977	25,02683
0,008451081	0	0	2,188047	16,11218
0,01085227	0	0	2,608825	18,2834
0,014126389	0	0	2,590888	29,29802
0,011904522	0	0	2,190188	23,62353
0,008153216	0	0	2,510343	27,00907
0,007600497	0	0	2,015504	21,2467
0,025547517	0	0	2,422481	22,0595
0,0230571	0	0	2,083854	18,80972
0,021788393	0	0	1,854531	16,55883
0,019520149	0	0	3,73997	33,30538
0,017463899	0	0	2,069023	18,41178
0,020231989	0	0	2,70047	23,34905
0,01854471	0	0	4,614249	46,548034
0,01831354	0	0	3,451205	32,466476
0,01737846	0	0	1,690649	15,307524
0,26901084	0	0	3,059976	18,442559
0,24727016	0	0	1,911913	11,257639
0,31157874	0	0	1,614987	8,841509
0,53545333	0	0	1,79443	8,575672
0,50746981	0	0	6,459948	30,35656
0,45108427	0	0	6,459948	30,35656
0,50727834	0	0	1,6313	7,302399
4,75023E-06	0	0	1,999886	41,58743
1,73047E-05	0	0	1,780664	33,49245
1,42017E-05	0	0	2,234654	41,56706
2,86092E-05	0	0	1,637918	28,84876
0,000179492	0	0	2,167483	33,71199
0,000195926	0	0	3,402568	52,00349
0,000374118	0	0	1,78048	25,78601
0,000352634	0	0	1,539237	22,17767
0,00092202	0	0	1,782343	23,75739
0,00103291	0	0	1,878499	24,62782

1	0	0	1,1336826	2,2247247
1	0	0	3,2299742	4,20130625
1	0	0	0,9924502	0,60348889
1	0	0	0,9430581	0,42187375
1	0	0	0,880902	0,32886499
1	0	0	0,8834653	0,16099215
1	0	0	0,8632448	0,13513892
1	0	0	0,8692806	0,11955617
1	0	0	0,8838132	0,11545864
1	0	0	0,8522262	0,04917144

0,5479937	0	0	5,167959	26,363313
1	0	0	4,152824	14,224551
1	0	0	3,523608	7,860423
1	0	0	3,523608	7,860423
1	0	0	3,523608	7,860423
1	0	0	2,981515	5,808679
1	0	0	2,422481	3,915756
1	0	0	2,422481	3,915756
1	0	0	1,490757	2,296449
1	0	0	2,279982	3,474499

1	0	0	2,368648	11,916854
1	0	0	2,25347	9,789343
1	0	0	2,004812	6,708227
1	0	0	1,98203	6,507035
1	0	0	1,875469	5,599736
1	0	0	1,875469	5,599736
1	0	0	1,71918	4,962709
1	0	0	1,81686	5,125062
1	0	0	1,867937	5,129506
1	0	0	1,867937	5,129506

3,48367E-08	0	0	1,627907	37,757052
1,02877E-06	0	0	1,560078	29,820918
0,001824028	0	0	1,405039	15,777347
0,007112788	0	0	1,366279	13,08976
0,01795825	0	0	1,337209	11,274401
0,03588775	0	0	1,469655	11,105626
0,03691234	0	0	1,342127	9,897274
0,06442843	0	0	1,28876	8,613789
0,09899008	0	0	1,265926	7,768401
0,1481176	0	0	1,308006	7,361702

1	0	0	8,613264	62,3207
1	0	0	19,379845	114,91092
1	0	0	19,379845	114,91092
1	0	0	19,379845	114,91092
0,8820787	0	0	19,379845	114,91092
0,7350656	0	0	19,379845	114,91092
0,6300562	0	0	19,379845	114,91092
0,5512992	0	0	19,379845	114,91092
0,4900437	0	0	19,379845	114,91092
0,9282974	0	0	4,21301	21,84524
1,52236E-07	0	0	7,856694	178,14412
1,09321E-05	0	0	5,068575	89,74929
0,002296328	0	0	3,201887	38,27594
0,05665171	0	0	8,074935	68,32111
0,08266525	0	0	5,813953	45,6969
0,09738036	0	0	2,085723	15,67154
0,1561893	0	0	3,781433	26,0432
0,1695512	0	0	11,627907	77,57566
0,1507122	0	0	11,627907	77,57566
0,135641	0	0	11,627907	77,57566
0,000535072	0	0	1,985791	26,99133
0,045279049	0	0	8,074935	68,32111
0,034200085	0	0	2,093023	17,44752
0,032982508	0	0	1,682157	13,59957
0,045100315	0	0	14,534884	109,71732
0,03937537	0	0	2,122554	15,92336
0,043078736	0	0	6,459948	46,88591
0,065206886	0	0	2,768549	18,57661
0,063335171	0	0	5,699954	37,74061
0,080298009	0	0	1,829566	11,48702
2,59566E-17	0	0	2,994455	138,56373
1,61694E-15	0	0	5,309547	220,07234
2,05982E-15	0	0	2,721552	111,04172
3,04341E-15	0	0	2,609348	104,69443
1,46417E-14	0	0	3,335875	127,86005
1,70463E-14	0	0	2,641357	100,35682
1,90428E-14	0	0	2,202659	83,10523
3,21132E-14	0	0	2,753978	102,09931
1,72781E-13	0	0	2,080951	73,40107
4,18685E-13	0	0	2,981515	102,21348
0,00305909	0	0	3,58886	39,95109

0,04028775	0	0	3,58886	28,21165
0,02688615	0	0	5,813953	45,6969
0,02270783	0	0	4,844961	37,50526
0,0230521	0	0	3,020236	22,66051
0,02056091	0	0	3,181766	23,65623
0,02032564	0	0	3,633721	26,49817
0,02094471	0	0	3,284719	23,41598
0,01997162	0	0	3,875969	27,35873
0,01797446	0	0	3,875969	27,35873
0,7974952	0	0	2,797091	21,592415
1	0	0	1,75185	11,344252
1	0	0	1,795816	9,861423
1	0	0	1,623185	8,810455
1	0	0	1,558814	8,207336
1	0	0	1,735509	8,799108
1	0	0	2,190765	11,079096
1	0	0	2,047167	10,264787
1	0	0	3,083157	15,358272
1	0	0	2,236136	11,001151
0,000300311	0	0	2,971576	38,12292
0,001064076	0	0	2,230774	24,25074
0,004547465	0	0	2,466526	22,230974
0,013295965	0	0	2,666951	20,408841
0,071199092	0	0	4,009623	23,06069
0,102920435	0	0	2,422481	12,598204
0,136764775	0	0	2,719978	12,952758
0,175139856	0	0	5,813953	25,472255
0,388299725	0	0	1,739217	6,030303
0,353750769	0	0	1,95756	6,76352
1,24122E-05	0	0	5,068575	89,74929
0,001714014	0	0	3,229974	39,03729
0,03391195	0	0	2,257846	19,63321
0,1887478	0	0	3,354204	22,44373
0,154005	0	0	11,627907	77,57566
0,1283375	0	0	11,627907	77,57566
0,2566518	0	0	3,569971	20,79258
0,3115527	0	0	8,305648	45,65548
0,6506957	0	0	3,726893	17,30271
0,5945502	0	0	4,559964	21,10141
3,79653E-06	0	0	5,229482	95,287528
0,000441749	0	0	3,378138	43,143601

0,2380536	0	0	5,099959	30,989596
0,63875	0	0	1,769464	8,496508
0,5456796	0	0	2,949107	13,967201
0,5068671	0	0	4,559964	21,101413
0,738737	0	0	2,004812	8,213104
0,8254552	0	0	4,844961	18,663622
0,8469328	0	0	3,523608	13,068014
0,7622395	0	0	3,523608	13,068014
0,3722068	0	0	7,267442	36,81572
0,3677444	0	0	3,059976	13,41725
0,2970126	0	0	2,202255	9,233849
0,3080468	0	0	3,691399	14,281087
0,6399871	0	0	3,419973	9,967192
0,5953071	0	0	4,844961	13,58748
1	0	0	6,459948	12,387725
1	0	0	6,459948	12,387725
0,9633803	0	0	6,459948	12,387725
0,8670423	0	0	6,459948	12,387725
0,000157547	0	0	2,363396	34,76325
0,000126434	0	0	2,32094	33,04064
0,000306028	0	0	2,99152	38,72961
0,0002518	0	0	1,937984	24,91052
0,000208285	0	0	1,809899	23,20365
0,000402921	0	0	2,523887	30,23176
0,000482719	0	0	2,392573	27,85771
0,00051167	0	0	2,455365	28,11794
0,000647622	0	0	1,751364	19,43703
0,000767661	0	0	2,062841	22,32575
0,4747953	0	0	8,074935	68,32111
0,2860907	0	0	2,239882	18,53348
0,2435949	0	0	2,299304	18,46261
0,2177194	0	0	2,716801	21,33848
0,316206	0	0	6,459948	46,88591
0,2844243	0	0	2,387372	17,14502
0,257779	0	0	1,785677	12,7243
0,2255566	0	0	1,785677	12,7243
0,2913855	0	0	2,236136	15,09816
0,2802941	0	0	1,793359	11,98922
0,000146873	0	0	7,350976	118,78417
0,015757742	0	0	1,676628	18,09132
0,020786399	0	0	5,129959	51,85294

0,027080665	0	0	3,499139	33,43659
0,034355824	0	0	1,768646	16,08507
0,033278897	0	0	4,472272	40,00043
0,031062406	0	0	2,036526	18,04132
0,033617444	0	0	2,70047	23,34905
0,043347071	0	0	2,239882	18,53348
0,07163602	0	0	1,951443	14,96092
2,98664E-44	0	0	2,650806	282,9632
1,5395E-39	0	0	2,456743	233,8886
5,03042E-39	0	0	2,669998	249,9471
1,74358E-38	0	0	2,566013	236,2849
5,29434E-38	0	0	2,400073	217,8034
4,41195E-38	0	0	2,400073	217,8034
3,78167E-38	0	0	2,400073	217,8034
2,08059E-37	0	0	2,690507	239,2131
1,84941E-37	0	0	2,690507	239,2131
2,47502E-37	0	0	2,552818	225,9584
0,00220339	0	0	2,96081	36,34316
0,002366175	0	0	2,751459	31,67016
0,002227457	0	0	2,93634	32,78501
0,004237214	0	0	2,299304	23,5323
0,008992264	0	0	7,267442	67,28875
0,010973734	0	0	4,037468	35,8425
0,018015611	0	0	4,559964	37,51749
0,026795695	0	0	2,495889	19,21099
0,036533815	0	0	2,799311	20,34895
0,036583433	0	0	2,768549	19,82988
0,004052187	0	0	3,445306	38,72041
0,004159278	0	0	3,007217	31,63402
0,037524086	0	0	2,334921	18,47917
0,031331647	0	0	2,605693	20,34247
0,031988253	0	0	2,330488	17,62559
0,030300288	0	0	3,181766	23,65623
0,033558075	0	0	3,089541	22,17878
0,031791566	0	0	2,370197	16,82653
0,046333407	0	0	2,735978	18,0705
0,047493057	0	0	2,241207	14,51111

Genes

OTUD4;GPM6A;PLAG1;AFF4;PCDHA1;TRPS1;DAG1;PCDHA5;PCDHA4;PCDHA3;PCDHA2;PCDHA9;PCDH/
SLC24A2;ANKRD33B;BMPR2;DCUN1D5;ONECUT2;NUFIP2;KIAA1549;SH3PXD2A;UBN2;PSD3;KIF1B;SOX6
CBX6;SF3B3;NUFIP2;TPM3;CELF1;CNBP;GATA6;XIAP;YOD1;HOOK3;SYNJ2BP;SOD2;DCAF7;TMEM245;CF
NCOA2;HDAC5;CREBBP;XRCC5;CUL3;ESR1;RUNX1;PIAS1;KAT2B;CREB1;RXRA;SP1;SIN3A;MYOD1;CDK
KCMF1;SRPK2;IGBP1;MBNL2;SORT1;ATP2B4;CREBL2;PARVA;PTPN11;TMEM64;GAPVD1;C1ORF213;AMC
HDAC5;MAP2K1;NFATC3;ESR1;FOXO1;ADD2;EEF2K;CREB1;RXRA;APC;PPP1R1B;CDK1;DCX;E2F1;MAPK
NRP2;BTG1;ARID5B;PROX1;SMG7;RUNX1;SMAD7;SULF2;ENAH;NFIA;TBL1XR1;ID2;KIF26B;PLXNA2;HIVE
LYN;TFAP2B;CREBBP;BMPR2;PROX1;RUNX3;HIF1A;ESR1;PAX2;EREG;SMAD7;SFRP1;PTK2B;CTNNB1;R/
LYN;CREBBP;SET;XRCC5;SRSF1;PIAS1;PPP2CA;KAT2B;PURA;ITCH;HNRNPK;RBL1;SP1;C1QBP;RUVBL1;
NRP2;BTG1;ARID5B;RUNX1;SMAD7;SULF2;SPRED1;NFIA;TBL1XR1;SERTAD2;ID2;ELMO1;PLXNA2;CALM1

CUL3;RNF180;LTN1;XIAP;UBE2J1;ZNRF2;ZNRF3;RNF217;LONRF3;BTRC;RNF111;SMURF2;MSL2;RC3H1;E
TCERG1;MAML1;DTX1;RORB;MED12L;MTDH;MED17;ING4;NPAT;MED14;RXRA;PRDM16;RUVBL1;NRIP1;T
CUL3;RNF180;LTN1;XIAP;UBE2J1;ZNRF2;ZNRF3;RNF217;LONRF3;BTRC;RNF111;SMURF2;MSL2;RC3H1;U
HDAC5;GATA6;HNRNPU;SIX1;RORB;IKZF3;ELK4;PURA;RXRA;SIN3A;RBBP5;ERBB4;SALL4;TRIM24;E2F1;I
MEF2A;KLF10;BCL11B;ONECUT2;PLAG1;EBF1;NFATC3;TFEB;FOXJ2;SIX1;ESR1;LITAF;PHOX2B;ELK4;HNI
ONECUT2;PLAG1;SIX1;LITAF;ELK4;NKRF;NSD1;ZNF148;TEAD1;MEF2A;KLF10;CREBBP;NFATC3;TFEB;FO
CUL3;RNF180;LTN1;KLHL32;XIAP;TNFAIP3;DTX4;BACH1;UBE2J1;FBXO40;ZNRF2;ZNRF3;RNF217;TRIM24
NCOA2;MED14;RXRA;MYOD1;NSD1;NRIP1;CTNNB1;TRIP12;ACTN4;HIF1A;MED17
TSHZ3;ONECUT2;PLAG1;SIX1;BACH1;LITAF;ELK4;NKRF;ZNF148;TEAD1;MEF2A;KLF10;CREBBP;BCL11B;I
EOMES;MEF2A;TCERG1;HDAC5;CREBBP;GATA6;MTDH;HIPK2;CREB1;RBL1;SP1;SIN3A;ID2;TRPS1;ID4;C

TIAL1;DYRK3;STAU1;NUFIP2;CAPRIN1;IGF2BP1;RC3H1;EIF4E;GIGYF2;PUM2
CXADR;FRMPD2;CLDN1;AMOT;MTDH;RAP2C;CYTH3;OCLN;RAP2B;APC;VAPA;CLDN18;STRN
HNRNPU;ZBTB20;PPWD1;BMI1;DCAF7;SART3;MECOM;PAPOLG;UBXN7;SNRPD3;SCN1A;FNBP4;NCOA2;S
BMPR2;NOS1AP;ATP2B4;MAPK1;SCN5A;LRP8;SELE;LRP6;BMPR1A
RABGAP1L;MAP2K1;FZD5;SORT1;LNPEP;NSG1;SORL1;LRP6;NEURL1B;SNX1;ITCH;RAP1A;TMEM108;MA
AMER1;NUFIP2;PHF20;TNKS;ZBTB20;PPWD1;DTX1;BMI1;HIF1A;ZBTB4;DCAF7;MTDH;MED17;ING4;MED1
ABCA1;ABCA2;RAB3C;RALA;SH3KBP1;SORT1;RNASE6;RHOBTB3;TRAPPC8;RND3;RAB11A;TRAK2;AMOT
AGO1;EIF4E;DCP2
AGO1;EIF4E;DCP2
AFF4;SDR16C5;RXRA;SIN3A;TRPS1;NRIP1;E2F1;MCMBP;MEF2A;CREBBP;DFFA;ESCO1;DNMT3A;RUNX3

CREBZF;TCERG1;HNRNPU;RSF1;RORB;BMI1;BACH1;CDC73;ELK4;ING4;SDR16C5;MECOM;NKRF;SIN3A;
MAML1;HNRNPU;RORB;IKZF3;ELK4;EPC1;BTRC;RNF111;MEF2A;EOMES;MAP2K1;EBF1;POU3F1;RUNX3;I
SIX1;RSF1;RORB;MED17;PHF8;CKS1B;MED14;MECOM;EPC1;BTRC;TEAD1;RNF111;EOMES;MAP2K1;PRC
TCERG1;MAML1;HNRNPU;RORB;IKZF3;BACH1;ELK4;TIAL1;RUVBL1;EPC1;RNF111;MEF2A;EOMES;USP2;
NCKAP1;CHRM3;COX7B;RPS6KA6;CDH2;DPYSL5;SALL4;TRIM3;PCDHAC2;SH3GL2;PCDHAC1;TMOD2;DY
GPM6A;MAP2K1;FZD3;FZD5;USP9X;SEMA3A;SIX1;NDNF;ATP2B2;PROX1;SMARCA1;RUNX3;PHOX2B;RUI
BMPR2;ONECUT2;MAML1;HNRNPU;SIX1;RORB;LITAF;IKZF3;CDC73;MED17;ELK4;MED14;MECOM;NKRF;S
RORB;IKZF3;BACH1;ELK4;TIAL1;EFEMP1;SALL4;RUVBL1;EPC1;SOX6;BTRC;RNF111;EOMES;MAP2K1;AR
SIX1;RSF1;RORB;MED17;PHF8;CKS1B;RBM3;MED14;MECOM;EPC1;BTRC;TEAD1;RNF111;EOMES;UTP15
CREBZF;RSF1;RORB;BMI1;ING4;MECOM;NKRF;SIN3A;EPC1;METTL16;BTRC;KLF10;USP47;MAP2K1;SFM

RAB3C;TCERG1;TNFAIP8;ANKRD33B;MAML1;SETD9;PRDM6;KIAA0141;PPWD1;PHAX;AFF4;CDH6;ADAM
YTHDC1
NPFFR1;HPSE2;FRMPD2;ADK;LOXL4;NUDT5;BMI1;MYPN;FAM204A;FAM107B;TIAL1;ARL5B;SH3PXD2A;E
ZBTB14;ONECUT2;ROCK1;ESCO1;TXNL1;ST8SIA3;GATA6;PTPRM;TRAPPC8;LDLRAD4;APCDD1;SMAD7;
KANSL1
OTUD4;PRDM8;GPM6A;SCOC;TBC1D19;GABRB1;TENM3;FAM114A1;HPGD;UBA6;YTHDC1;ADH1B;HSPA4
ABHD4;CHURC1-FNTB;NRXN3;DLST;GSKIP;SIX1;HIF1A;FBLN5;NOP9;EFS;AP5M1;SIPA1L1;PABPN1;SLC2
ALAS2;COX7B;FRMPD4;SH3KBP1;IRS4;PHF8;AMOT;RBM3;MED14;NHSL2;RPS6KA6;NKRF;GPRASP2;IL13
GPR27;SLC7A14;ZBTB20;CLDN1;LMOD3;SERP1;MECOM;RUVBL1;DAG1;TMEM108;UBXN7;SCN5A;SEC62
FAM49A;NCKAP1;NRP2;USP37;BMPR2;DUSP19;RND3;HK2;GLS;EFEMP1;DPYSL5;KIF5C;PAPOLG;PSD4;

GNRHR;TAC3;SLC29A3;FGFR1
APC;PLAG1;TSHR
RBM28;CLDN1
ADAMTS2;SLC39A13
GLIS3;TSHR
CD244;RUNX1
ESR1;SCN1A
ESR1;LRP8
NSD1;ZBTB16;ACSL6;PTPN11;DEK;RUNX1
TRIM24;TSHR

PPP2CA;CREBBP;CREB1;APC;PLAG1;GNRHR;CTNNB1;TAC3;SLC29A3;TSHR;FGFR1
PPP2CA;IGBP1;CREBBP;CREB1;APC;PLAG1;CTNNB1;GDF6;PAX2;TSHR
MEF2A;PPP2CA;CREBBP;CREB1;APC;PLAG1;CTNNB1;TSHR;LRP6
PPP2CA;RBM28;CREBBP;CREB1;APC;PLAG1;CTNNB1;CLDN1;TSHR
PPP2CA;CREBBP;CREB1;APC;PLAG1;CTNNB1;ESR1;TSHR;SCN1A
PPP2CA;CREBBP;CREB1;APC;PLAG1;CTNNB1;LRP8;ESR1;TSHR
PPP2CA;CREBBP;BFSP2;MAF;CREB1;GJA3;APC;PLAG1;SORD;CTNNB1;TSHR
PPP2CA;CREBBP;CREB1;APC;PLAG1;VAPB;CTNNB1;TARDBP;TSHR
PPP2CA;CREBBP;CREB1;APC;PLAG1;TAP2;CTNNB1;TSHR
PPP2CA;CREBBP;CREB1;APC;PLAG1;CTNNB1;TSHR;FGFR1

NCKAP1;TCERG1;UHRF1BP1L;DENND5B;HNRNPU;HNRNPR;GLS;TIAL1;EFR3A;DAG1;FBXO3;SNIP1;SNR
OTUD4;NCKAP1;TCERG1;UHRF1BP1L;DENND5B;MAML1;HNRNPR;LDLRAD4;BACH1;DCAF7;FAM107B;P
TDRKH;PRDM8;ANKRD13B;CHRM3;HPSE2;PRDM6;POGK;ANTXR2;IKZF3;CDH6;GJA3;CDH2;FRRS1L;HO
NCKAP1;HPSE2;PRDM6;LDLRAD4;IKZF3;CTGF;CDH6;CDH2;DPYSL5;KIF5C;FRRS1L;IGLON5;SALL4;RAV
OTUD4;NCKAP1;HNRNPR;NUDT5;DAG1;SNIP1;PELO;RNF111;PSPH;FBXW4;CMC1;ATP11A;MOB3C;MAF;
HRK;CCDC71L;RAB3C;NRP2;DYRK3;ONECUT2;IRS4;TNFAIP3;RCSD1;BMI1;PPP1R9A;CLDN1;CTGF;GJA3
PRDM8;RAB3C;NPFFR1;PRDM6;IRS4;IKZF3;CDH6;EFEMP1;GJA3;CDH2;DPYSL5;KIF5C;FRRS1L;SALL4;R
PRDM8;CHRM3;NPFFR1;DENND5B;PRDM6;CTGF;EPS8;CDH6;DPYSL5;KIF5C;FRRS1L;HOXA3;MAP3K9;P
NPFFR1;ATP8A2;HPSE2;PRDM6;KLHL32;IRS4;RORB;IKZF3;GJA3;CDH2;DPYSL5;FADS6;KIF5C;SALL4;BS
TMEM167B;POGK;ZBTB20;NUDT5;TXNDC17;CDH2;FADS6;RAVER2;TRIM24;PIM3;PTGFRN;SNRPD3;C21

CAPZB;SH3KBP1;CAPZA1;AMOT
SMURF2;SMAD7
SP1;E2F1
ITCH;USP9X
RSF1;SMARCA1
GRIN2B;LRP8
CXADR;LNX2
SNX1;SNX6
MMP2;CLDN1
HNRNPK;C1QBP;PDCD4;HNRNPU;NAA38

PCDHA13;PCDHA12;PCDHA11;PCDHA10;PCDHA1;PCDHA5;PCDHA4;PCDHA3;PCDHA2;PCDHA9;PCDHA
PCDH11Y;PCDHB15;PCDHA13;PCDHA12;PCDHA11;PCDHA10;PCDHA1;PCDHA5;PCDHA4;PCDHA3;PCDH
PCDH11Y;PCDHB15;PCDHA13;PCDHA12;PCDHA11;PCDHA10;CDH6;CDH2;PCDHA1;PCDHA5;PCDHA4;PC
PARP11;TIPARP;DTX1;TRIP12;DTX4
PHLPP2;PDP2;PPM1L;PPM1H;PPM1K;PPM1E
RBM28;CPSF7;CELF1;CELF3;SRSF1;HNRNPR;ELAVL2;U2SURP;TIAL1;RBM3;SART3;PABPN1;TRA2B;RAV
SHC4;EPS8;SHC2;RABGAP1L;NOS1AP;APBB2;TNS1;APPL1
SORT1;SORCS1;SORL1
SORT1;SORCS1;SORL1
SORT1;SORCS1;SORL1

JPH1;AFF1;MED14;CCND3;GRM5;SIN3A;PAPOLG;DPYSL3;KIF1B;BSN;FNBP4;MEF2A;MAP2K1;PCYT1B;C
APC;NFATC3;CTNNB1;FOXO1;LRP6
PDE3B;ADD3;ADD2;BCLAF1;GRM5;EEF2K;ATXN7;EMB;CLSPN;BSN;EIF4E;ABCA1;PCYT1B;GFPT1;NFATC
BTG1;NUFIP2;SH3KBP1;ADD3;JPH1;GIGYF2;FOXO1;AFF1;AMOT;ADD2;NPAT;BCLAF1;RXRA;ATXN7;SIN3
MAP2K1;MAPK1;RAF1
SRPK2;CREBBP;RANBP3;PFKFB3;PHF20;CELF1;PDE3B;AGAP2;FAM129A;XIAP;ESR1;FOXO1;HK2;EEF2K
MEF2A;C20ORF112;CCND3;EEF2K;RXRA
SLC24A2;GJC1;CREB1;APC;PPP1R1B;NFATC3;CTNNB1;HNRNPC;BSN;TARDBP;FOXO1;LRP6
RBL1;CELF1;SOD2;RUNX3;FOXO1
HIF1A;HSPH1;MYH10;MEF2A;ABCA1;NCOA2;CBX5;MME;DEK;L1CAM;ESR1;PIAS1;OCLN;ITCH;CDC34;XK

OTUD4;MAML1;LTN1;PPP1R9A;AFF4;AFF1;NUDT4;MED14;UBL3;KIF5C;MAN1A2;DPYSL3;PSD3;FBXO3;Z
HRK;PFKFB3;ONECUT2;ATL3;CELF1;SPATA2;JPH1;COL19A1;PCMT1;SDR16C5;TRIM24;E2F1;ZNF800;SN
CREBZF;CHRM3;ANKRD33B;KIAA1671;HNRNPU;ZBTB20;CALML4;BACH1;PITPNC1;PHF8;CTGF;ELK4;AR
OTUD4;USP37;BTG1;BMPR2;SH3KBP1;LTN1;HNRNPU;TNFAIP3;DCAF7;AFF1;ALAD;EFR3A;UBL3;SESN3;
FAM49A;TENM3;KCNC2;RSF1;BACH1;AFF4;PITPNC1;MED17;NHSL2;GRM5;KIF5C;PAPOLG;DAG1;SNIP1;
CREBZF;CHRM3;TCERG1;ANKRD33B;CELF1;KIAA1671;HNRNPU;CALML4;BACH1;PITPNC1;PHF8;CTGF;
OTUD4;CCDC71L;DENND5B;HNRNPR;ELK4;SESN3;KIF5C;PTGFRN;AGFG2;EOMES;TMOD3;YOD1;RUNX
OTUD4;USP37;BTG1;BMPR2;SH3KBP1;HNRNPU;TNFAIP3;DCAF7;AFF1;EFR3A;UBL3;SESN3;ZNF800;CEP
OTUD4;CCDC71L;DENND5B;POGK;HNRNPR;ELK4;SESN3;PTGFRN;AGFG2;TMOD3;YOD1;RUNX3;EREG;
HRK;OTUD4;DYRK3;BMPR2;CELF1;ZBTB20;BMI1;LITAF;PFAS;AFF4;DCAF7;CDH6;TIAL1;ADAMTS5;MED1

NCOA2;DLX1;CREBBP;RUNX3;ESR1;FOXO1;RUNX1;SMAD7;KAT2B;RBL1;CREB1;SIN3A;SP1;MYOD1;SNI

TGFBR3;PPP2CA;ITCH;OCLN;SMURF2;XIAP;CTNNB1;RNF111;CTGF;SMAD7
RANBP3;FZD5;APC;CUL3;CTNNB1;LRP6
NCOA2;KAT2B;CREBBP;RXRA;NRIP1;CDK1;MAPK1
MAP2K1;RAP1A;APC;SH3KBP1;GAB1;MAPK1;CTNNB1;PTPN11;RAF1;CRK;EIF4E;SH3GL2
SIN3A;TNKS;XRCC5;SP1;E2F1;TERF2IP;MAPK1;HNRNPC;MXD1;ESR1;RAD9A
MAPK10;CSNK1G3;KAT2B;RALA;CREBBP;CSNK1A1;CTNNB1;SOD2;FOXO1
NCOA2;CCND3;XRCC5;CARM1;ZNF318;NRIP1;PTK2B;CTNNB1;APPL1;PIAS1
CREBBP;MAP2K1;RAP1A;MAPK1;PTPN11;CALM1;PIAS1;SMAD7
CREBBP;MAP2K1;SP1;MMP2;NFATC3;CDK1;ESR1;CKS1B

GALNT7;SEPT10;SF3B3;HNRNPU;DLST;CTDSPL2;ESR1;PCMT1;DDX19B;HNRNPK;TBL1XR1;SIN3A;RBBP
SET;SF3B3;HNRNPU;HNRNPR;GIGYF2;PHF8;HSPH1;RBBP5;RUVBL1;CEP170;SNRPD3;PTGFRN;RBM7;K
MTMR3;SF3B3;HNRNPU;HNRNPR;LNPEP;SMARCA1;SMC1A;ADD3;GTPBP4;CDC73;SMAD7;C20ORF112;
RBM28;SET;SF3B3;HNRNPU;HNRNPR;MED17;PHF8;CAPZB;RUVBL1;G3BP1;AP2M1;FNBP4;SEPT10;CPS
SET;SF3B3;NUFIP2;DCTN2;HNRNPU;DLST;HNRNPR;GIGYF2;CDC73;PCMT1;NPAT;CAPZB;G3BP1;ATXN1L
FYTTD1;MTMR3;SF3B3;ROCK1;LUZP1;HNRNPU;LRRC40;HNRNPR;ACTN4;HCFC2;SMC1A;GIGYF2;GATA
CPSF7;SF3B3;CUL3;DEK;GAPVD1;SMARCA1;SMC1A;CDC42BPA;HNRNPK;RUVBL1;SNRPD3;TARDBP;RA
SET;SF3B3;TPM3;HNRNPU;DLST;HNRNPR;PDHB;SMC1A;HNRNPK;SIN3A;RUVBL1;CCDC6;TERF2IP;HNR
HNRNPK;TRIM3;PDCD4;ASB7;CEP170;GAPVD1;HCFC2
ABCA1;DOCK5;HNRNPK;XPO4;ROCK1;GPAM;RUVBL1;HSPA4L;FAM129A;HNRNPU;TARDBP;DYNLL2

FZD3;PCDH11Y;FZD5;PCDHB15;PCDHA13;PCDHA12;PCDHA11;PCDHA10;CDH6;CDH2;PCDHA1;ERBB4;P
PCDH11Y;LRP6;PPP2CA;CDH6;PCDHA1;CDH2;PCDHA5;PCDHA4;PCDHA3;PCDHA2;BTRC;PCDHA9;PCD
LYN;MAP2K1;ROCK1;CSNK1A1;PTPN11;FOXO1;TPCN1;ELK4;MAPK10;CREB1;SP1;CAMK4;PTK2B;MAPK1
MAP3K2;MAP2K1;SHC2;GAB1;GAB3;EREG;PPP2CA;MAPK10;NRG3;ERBB4;SPRY3;MAPK1;SPRY1;RAF1;
RPS6KA6;MAP2K1;IRS4;MAPK1;RAF1;SOS2
MAPK10;CREBBP;BMPR2;SMURF2;SNIP1;MAPK1;SMAD9;INHBA;GDF6;SMAD7;BMPR1A
LYN;MAP3K2;MAPK10;MAP2K1;NFATC3;MAPK1;RAF1;SOS2
ALDH6A1;DPYSL5;DPYSL3
MAP2K1;PARVA;ACTN4;RND3;RAP2C;MAPK10;RAP2A;RAP1A;RAP2B;ELMO1;PTK2B;RAF1;SOS2;CRK
PPP2CA;MAP3K2;MAPK10;MAP2K1;SPRY3;MAPK1;SPRY1;RAF1;SOS2;FGFR1

PCDH11Y;PCDHB15;PCDHA13;PCDHA12;PCDHA11;PCDHA10;PCDHA1;PCDHA5;PCDHA4;PCDHA3;PCDH
PCDH11Y;PCDHB15;PCDHA13;PCDHA12;PCDHA11;PCDHA10;CDH6;CDH2;PCDHA1;PCDHA5;PCDHA4;PC
RBM28;CPSF7;CELF1;CELF3;SRSF1;HNRNPR;ELAVL2;U2SURP;TIAL1;RBM3;SART3;PABPN1;TRA2B;RAV
RNF126;ZNRF2;RNF148;ZNRF3;RFWD3;LTN1;RLIM;RNF111;RNF165
SORT1;SORCS1;SORL1
SORT1;SORCS1;SORL1
DRP2;FNBP4;TCERG1;ITCH;SMURF2;HECW2;APBB2
EDEM3;MAN1A2;EDEM1
SHC4;SHC2;NOS1AP;APBB2;APPL1
KCMF1;DRP2;CREBBP;ZZZ3

PCDH11Y;PCDHB15;PCDHA13;PCDHA12;PCDHA11;PCDHA10;PCDHA1;PCDHA5;PCDHA4;PCDHA3;PCDH
PCDH11Y;PCDHB15;PCDHA13;PCDHA12;PCDHA11;PCDHA10;CDH6;CDH2;PCDHA1;PCDHA5;PCDHA4;PC

PDP2;PPM1L;PPM1H;PPM1K;PPM1E
TRIM41;RNF180;PCGF3;RC3H1;DTX1;BFAR;MARCH7;BMI1;DTX4;RBX1;RNF126;RNF148;ZNRF2;ZNRF3;R
DRP2;FNBP4;TCERG1;ITCH;SMURF2;HECW2;APBB2
KCMF1;DRP2;CREBBP;ZZZ3
ZBTB16;KLHL7;KLHL32;RHOBTB3;ZBTB10;ZBTB20;ZBTB34;KLHL23;ZBTB44;BTBD9;BACH1;ZBTB4
OTUD4;TNFAIP3;YOD1
SHC4;NOS1AP;APBB2;APPL1
PDE1B;PDE3B;PDE5A;PDE7B

CDK1;MAPK1;RAF1
GAB1;CTNNB1;PTPN11;RAF1;CRK;EPOR
MAP2K1;BMPR2;RBL1;CREB1;CAMK4;PPP1R1B;CDK1;MAPK1;RAF1;PRKG1
GAB1;PTK2B;SPRY1;SELE
LYN;OCLN;MAPK1
CDC37;RAF1
MAPK1
MAPK1
MAPK1
ADD2

HDAC5;PSMD11;PLAG1;HIF1A;JPH1;DCAF7;FOXO1;JPH3;MECOM;SIN3A;PRDM16;FBXO3;MAPK1;BTRC;F
SET;MAML1;HIF1A;FOXO1;MTDH;PCMT1;NPAT;MECOM;CDH2;SERTAD2;TDG;RUVBL1;E2F1;SNIP1;MAPK
MAP2K1;RALA;ATP2B4;HNRNPU;PTPN11;L1CAM;GRIN2B;LRP8;RAB11A;PPP2CA;MAPK10;GRM5;RAP2A;
CALCOCO2;SH3KBP1;TNFAIP3;BACH1;SPRED1;EEF2K;SCN5A;CCDC50;SH3GL2;UBL7;GPR37;USP2;DIO
JPH1;AFF4;AFF1;MED14;CCND3;GRM5;SIN3A;PAPOLG;DPYSL3;KIF1B;BSN;APPL1;FNBP4;MEF2A;MAP2K
SH3KBP1;IRS4;DTX1;HNRNPR;EFS;PCDHA1;ERBB4;PCDHA5;PTK2B;PCDHA3;PCDHA2;PCDHA9;PCDHA
HIF1A;MED12L;FOXO1;MED14;SART3;NSD1;TDG;TRIM3;RUVBL1;NRIP1;TRIM24;MAPK1;NCOA2;CREBBP
DLX1;RALA;PSMD11;FOXO1;ARL5B;EFEMP1;MECOM;TDG;SNIP1;MAPK1;APBB2;BTRC;ZNF423;HOXC8;M
BTG1;SH3KBP1;JPH1;AFF4;AFF1;AMOT;CKS1B;SIN3A;DPYSL3;BSN;FNBP4;PCYT1B;RUNX1;ADCY9;RBL1
SET;MAML1;GATA6;HNRNPU;DTX1;HIF1A;ING4;ATXN7;TDG;SNIP1;MAPK1;EMB;BTRC;ZNF148;N4BP2;ME

CRP;PTPN11;SCN5A;SMC1A;RAF1
CREBBP;EGR4;ROCK1;MMP2;PRUNE2;SNAP23;DIO2;RUNX3;ZFX;ESR1;PAX2;PHOX2B;CDC73;EPOR;SM
AMER1;GABRA1;BMPR2;ZBTB14;AGAP2;PDHB;L1CAM;SMC1A;AFF4;PHF8;RCAN1;GRM5;IL1RAPL1;DCX;
GABRA1;BMPR2;ZBTB14;NUFIP2;AGAP2;AFF4;RCAN1;GRM5;BCLAF1;CAPRIN1;CAMK4;TARDBP;EIF4E;S
RAP1A;HNRNPK;RBBP5;MACROD2;SCN1A
EOMES;ZNF462;PRRX1;HNRNPU;SLC1A4;PDHB;L1CAM;CREB1;NFIA;KANSL1;SIN3A;DCX;RAB3GAP2;GP
TCERG1;ATP8A2;ROCK1;HSPA4L;FOXO1;ADD2;GRM5;JPH3;DPYSL5;ATXN7;ZMAT3;IGLON5;GPRASP2;S
TCERG1;ATP8A2;ROCK1;HSPA4L;FOXO1;ADD2;GRM5;JPH3;DPYSL5;ATXN7;ZMAT3;IGLON5;GPRASP2;S
AMER1;NCOA2;MAP2K1;PTPN11;PAX5;FOXO1;PAX2;EPOR;STIM2;SALL4;MYOD1;DAG1;ELMO1;CDK1;CT
DLX1;NRP2;SET;XIAP;BMI1;HIF1A;GLS;SPRED1;RXRA;MECOM;NSD1;PTK2B;MYH10;TGM2;NCOA2;CREB

NCOA2;KAT2B;CREBBP;RXRA;TBL1XR1;CARM1;HIPK1;TBX5;TEAD1;CTGF;HIPK2
NRP2;MED17;MED14;CCND3;RPS6KA6;SPRED1;CDH2;DPYSL5;SALL4;DPYSL3;HOXA3;SCN5A;AP2M1;SH
AMER1;PPP2CA;TNKS2;FZD5;APC;TNKS;CSNK1A1;CTNNB1;LRP6

HDAC5;CREBBP;MAML1;DTX1;DTX4;HIF1A;RBX1;KAT2B;NEURL1B;HEYL;ITCH;APH1B;TBL1XR1
SHC2;NRP2;PSMD11;ROCK1;CUL3;SEMA3A;AGAP2;PPP2CA;RPS6KA6;SPRED1;RAP1A;APH1B;EFNB3;DI
APH1B;NRG3;ERBB4;PTPN11;BTRC;S100B;ESR1;EREG;RBX1
AMER1;PSMD11;TNKS;CUL3;XIAP;PRICKLE1;CDC73;LRP6;PPP2CA;ZNRF3;TNKS2;RBBP5;RUVBL1;RSPC
HDAC5;CREBBP;MAML1;DTX1;DTX4;HIF1A;RBX1;KAT2B;NEURL1B;HEYL;ITCH;APH1B;CREB1;TBL1XR1;A
AMER1;CREBBP;PSMD11;FZD5;SMURF2;CSNK1A1;TNKS;CUL3;XIAP;CDC73;RBX1;LRP6;PPP2CA;SFRP1
AMER1;HDAC5;PSMD11;MAML1;TNKS;FOXO1;LRP6;PPP2CA;NEURL1B;APH1B;TNKS2;ERBB4;TRIM24;CF

HPSE2;FRMPD4;KIAA0141;HNRNPU;LDLRAD4;CALML4;ANTXR2;BACH1;DCAF7;GLS;ELK4;RPS6KA6;SEC
NCKAP1;TCERG1;MAML1;FRMPD4;NUDT4;TIAL1;RPS6KA6;DPYSL3;DAG1;SNRPD3;SEC62;SOX6;BTRC;F
SCOC;ZNF292;HNRNPR;DCAF7;EPS8;ELK4;RPS6KA6;DPYSL3;SEC62;SOX6;CXADR;C5ORF15;ALG6;FND
TCERG1;FRMPD4;FRMPD2;CALML4;ANTXR2;BACH1;TXNDC17;RPS6KA6;GJA3;DIP2B;SOX6;TNS1;PRKAI
TDRKH;FRMPD4;CALML4;ANTXR2;IKZF3;BACH1;RPS6KA6;DPYSL5;DPYSL3;SEC62;SOX6;WLS;PRKAB2;
TDRKH;FRMPD4;CALML4;ANTXR2;IKZF3;BACH1;RPS6KA6;DPYSL5;DPYSL3;SEC62;SOX6;WLS;PRKAB2;
TDRKH;FRMPD4;CALML4;ANTXR2;IKZF3;BACH1;RPS6KA6;DPYSL5;DPYSL3;SEC62;SOX6;WLS;PRKAB2;
KIAA0141;LDLRAD4;CALML4;ANTXR2;GLS;RPS6KA6;RAVER2;HOXA3;SOX6;FNBP4;CMC1;EBF1;RC3H1;F
KIAA0141;LDLRAD4;CALML4;ANTXR2;GLS;RPS6KA6;RAVER2;HOXA3;SOX6;FNBP4;CMC1;EBF1;RC3H1;F
FRMPD4;LDLRAD4;ANTXR2;BACH1;DCAF7;GLS;EPS8;EFR3A;GJA3;DPYSL3;PTGFRN;SOX6;BTRC;FNBP4

MEF2A;SHC4;MAP3K2;MAP2K1;SHC2;SORT1;PTPN11;GRIN2B;PPP2CA;MAPK10;MARCKS;RAP1A;CREB1
MEF2A;MAP3K2;MAP2K1;RALA;ROCK1;SH3KBP1;GAB1;PTPN11;FOXO1;EPS8;ELK4;ITCH;RAP1A;CREB1;
MEF2A;KLF10;MAP2K1;CREBBP;SMURF2;ROCK1;RBX1;PIAS1;SMAD7;TGFBR3;ITCH;RBL1;SP1;SIN3A;CD
HDAC5;NRP2;SHC2;ROCK1;FOXO1;AMOT;CTGF;RAP1A;GPC1;PTK2B;MAPK1;EIF4E;PRKG1;MAP2K1;MM
ELK4;RBL1;MYOD1;ID2;PAX5;PAX2
HDAC5;EIF2B2;TBL1XR1;GABRA3;HIVEP2;SMARCA1;SMC1A;GRIN2B;CRK;SCN1A
MAP2K1;RAP1A;GAB1;PTK2B;MAPK1;PTPN11;RAF1;CRK
MAP3K2;PRKAB2;MAP2K1;SHC2;IRS4;DIO2;FOXO1;MAPK10;RPS6KA6;RXRA;SESN3;MAPK1;MAP3K9;RA
NCOA2;CREBBP;ROCK1;FOXO1;PIAS1;KAT2B;CREB1;SIN3A;SP1;CARM1;ZNF318;CTNNB1;RAD9A
SHC4;MAP2K1;SHC2;GAB1;FOXO1;EREG;MAPK10;NRG3;ERBB4;MAPK1;RAF1;SOS2;CRK

CREBBP;BMPR2;ROCK1;SMURF2;SMAD9;INHBA;GDF6;RBX1;SMAD7;PPP2CA;RBL1;SP1;ID2;ID4;MAPK1;
NCOA2;MAP2K1;CREBBP;DIO2;HIF1A;ESR1;FOXO1;MED12L;MED17;RCAN1;KAT2B;MED14;RXRA;SIN3A;
MEF2A;MAP2K1;ROCK1;PDE3B;IRS4;NFATC3;ATP2B4;ATP2B2;CALML4;ATP2B1;ADRA2B;ADRA2A;GNA13
SHC4;MAP2K1;SHC2;SORT1;GAB1;PTPN11;CALML4;MAPK10;RPS6KA6;RAP1A;CAMK4;MAPK1;RAF1;CAL
CREBBP;FZD3;FZD5;CSNK1A1;NFATC3;PRICKLE1;SENP2;RBX1;LRP6;MAPK10;CCND3;SFRP1;ZNRF3;AP
GRM5;RPS6KA6;MAP2K1;CREBBP;RAP1A;CAMK4;MAPK1;CALML4;RAF1;CALM1;GRIN2B
MAP2K1;CREBBP;RAP1A;GAB1;MAPK1;PTPN11;RAF1;SOS2;CRK;HIF1A;RBX1
MAP2K1;FZD3;BMPR2;FZD5;PCGF3;SMAD9;INHBA;BMI1;APC;ID2;ID4;COMMD3-BMI1;MAPK1;CTNNB1;RA
SHC4;MAPK10;MAP2K1;SHC2;NRG3;ERBB4;GAB1;MAPK1;RAF1;SOS2;CRK;EREG
SHC4;MAP2K1;SHC2;FZD3;FZD5;CSNK1A1;ESR1;LRP6;HEYL;APC;SP1;E2F1;MAPK1;CTNNB1;RAF1;SOS2

\8;PCDHAC2;PCDHA7;PCDHAC1;PCDHA6;MBNL2;BCL11B;DYRK1A;FNDC3B;ZFP91;ATP2B2;PCDHA1
;;RIMKLA;MBNL3;PPARGC1B;FZD3;CBX5;HELZ;PARVA;C1ORF21;HIPK2;ENAH;NFIA;SESTD1;LCOR;S
RISPLD2;ZMAT3;UBN2;G3BP1;LCOR;MAPK1;CRK;DNAL1;NFE2L1;TNRC6B
.1;MAPK1;CTNNB1
)T;LRP6;UBL3;TBL1XR1;TRIP12;SH3GL2
(1;CTNNB1;RAF1
:P2;CALM1
AF1
;CDK1;E2F1;MAPK1;BTRC;RAF1;KPNA1
1

3FAR;UBE2G1;RBX1;PIAS1;RNF126;ITCH;CDC34;HECW2;RLIM;TRIP12;RNF165
RIM24;PPARGC1B;MEF2A;NCOA2;TFAP2B;CREBBP;PRRX1;ARID5B;ACTN4;HCFC2;ZFX;HIPK2;PIAS1
JBE2G1;BFAR;RBX1;RNF126;ITCH;CDC34;HECW2;RLIM;TRIP12;UBE2K;RNF165
HIVEP2;TEAD1;MEF2A;BCL11B;ZBTB14;XRCC5;TIPARP;TFEB;ARID5B;PROX1;ESR1;PAX2;RUNX1;SM
RNPK;CREB1;NFIA;SP1;NKRF;TFAM;SSBP2;NFE2L1
)XJ2;TBX5;ESR1;PAX2;GATAD2B;PHOX2B;HNRNPK;CREB1;NFIA;SP1;MYOD1;TFAM;HNRNPC;MXD1;S
;FBXO3;LONRF3;BTRC;UNKL;RNF111;FBXW4;SMURF2;MSL2;RC3H1;ZFP91;UBE2G1;KLHL23;BFAR;F

EBF1;NFATC3;TFEB;FOXJ2;PROX1;ESR1;PHOX2B;HNRNPK;CREB1;RBL1;NFIA;SP1;TFAM;MXD1;SSB
TNNB1;TEAD1

SFMBT2;ANGEL2;DYRK1A;ACTN4;SENP2;PIAS1;CDC34;MYOD1;COMMD3-BMI1;TERF2IP;WDFY3;RAI

\PK1;WDFY2;SNX8;SH3GL2;WLS;RAB11FIP5;SNX6;APPL1;KCNH1
4;RBBP5;PAPOLG;UBXN7;SNRPD3;SCN1A;NCOA2;CREBBP;TRIM41;CBX5;ZBTB16;GTF2H1;ACTN4;G
Γ;LRP6;ADD2;OCLN;SPRED1;KIF1B;RAPGEF6;WLS

;ESR1;GATAD2B;PHOX2B;HNRNPK;MAF;RBL1;SP1;MYOD1;ASXL3;HNRNPC;MXD1

C1QBP;TRPS1;EPC1;BTRC;ZNF366;MEF2A;EOMES;KLF10;USP47;USP2;ARID5B;PROX1;HCFC2;CBF
PIAS1;RUNX1;SFRP1;TBL1XR1;ZNF318;TFAM;DLX1;TNKS;GATA6;GLIS3;CREBL2;HIF1A;FOXO1;NPAT
)X1;POU3F1;RUNX3;PAX2;PIAS1;RUNX1;SFRP1;CREB1;TBL1XR1;ZNF318;TFAM;MAPRE3;MYRF;GAT
;EBF1;ARID5B;HCFC2;RUNX3;FOXP2;RUNX1;TBL1XR1;SUB1;TFAM;CGGBP1;DLX1;TSHZ3;TNKS;GAT
'RK1A;PROX1;PCDHA13;PCDHA12;HPCAL4;PCDHA11;PCDHA10;DCX;GPM6A;DLX6;SHOX2;TRAK2;E
NX1;ID2;IL1RAPL1;ID4;DCX;CTNNB1;LRP12;MARK1;FGFR1;ITM2C
SIN3A;EPC1;TEAD1;RNF111;MEF2A;EOMES;KLF10;NCOA2;EBF1;TFEB;PROX1;PAX2;RUNX1;KAT2B;C
ID5B;POU3F1;HCFC2;RUNX3;FOXP2;PIAS1;EREG;DKK3;RUNX1;SFRP1;TBL1XR1;SUB1;ZNF318;TFAI
;;MAP2K1;DYRK1A;PROX1;POU3F1;RUNX3;PAX2;PIAS1;RUNX1;KAT2B;SFRP1;CREB1;TBL1XR1;ZNF3
IBT2;DNMT3A;ARID5B;PROX1;CBFA2T2;PAX2;FOXP2;DKK3;EREG;SFRP1;RBL1;ZNF318;DCP2;HDAC5

TS2;DPYSL3;EMB;PELO;ZNF366;PCDHAC2;PCDHAC1;CCDC112;UTP15;CSNK1G3;C5ORF15;TTC33;EI

PC1;BTRC;PRKG1;FBXW4;ZCCHC24;ENTPD1;WDR37;GRID1;SFMBT2;KIAA1462;ARID5B;SORCS1;GT
CTIF;CDH2;VAPA;PQLC1;ASXL3;ZNF236;MAPRE2;RNF165

L;MRFAP1;NDNF;PPM1K;ANTXR2;AFF1;GNRHR;APBB2;SLIT2;EIF4E;N4BP2;ATOH1;GALNT7;SEPT11;
2A17;FNTB;FAM177A1;MAP3K9;BCL2L2-PABPN1;ZC3H14;SNX6;GPR135;MAPK1IP1L;RAB2B;NAA30;E
RA1;PCYT1B;CHST7;GAB3;SMC1A;ZFX;SYTL4;RAP2C;IL1RAPL1;ARMCX6;DCX;TBC1D25;AGTR2;AM
;BSN;AP2M1;CCDC50;C3ORF14;APPL1;KPNA1;EOMES;MME;NSUN3;C3ORF62;CMC1;MSL2;FNDC3B;
ARL5A;CNPPD1;SCN1A;TNS1;KCMF1;ST6GAL2;DNMT3A;AHSA2;GPR75;ADRA2B;CREB1;HECW2;TET

RPD3;MACROD2;SEC62;AGFG2;MEF2A;KCMF1;CSNK1G3;PRKAB2;FNDC3B;DYRK1A;ZBTB34;YOD1;FI
CMT1;EFR3A;PAPOLG;CHP1;PELO;RNF111;MEF2A;CXADR;RC3H1;FNDC3A;HCFC2;GTPBP4;FAM133E
XA3;SOX6;PCDHAC2;KCNH1;PCDHAC1;TNS1;EOMES;ST6GAL2;SLC30A4;EBF1;ATP11A;POU3F1;SHIS
ER2;HOXA3;BSN;SOX6;PCDHAC2;KCNH1;TNS1;EOMES;ST6GAL2;SFMBT2;TMOD2;VASH2;EBF1;ACSI
TBL1XR1;SUB1;ADAM12;CDIP1;TFAM;WDFY3;SRSF9;GLYR1;TBCEL;FAM114A1;IGSF3;DCUN1D5;YTHI
;PSD3;RSPO2;TMEM108;EPC1;SOSTDC1;PELO;PRKG1;CXADR;SFMBT2;TMOD2;GAB3;PROX1;FOXP
AVER2;SOX6;PCDHAC2;EOMES;ENTPD1;SFMBT2;TMOD2;VASH2;ACSL6;POU3F1;SHISA9;HPCAL4;F
TGFRN;SOX6;SH3GL2;PCDHAC2;KCNH1;TGM2;TNS1;EOMES;ST6GAL2;TMOD2;FNDC3B;VASH2;EBF
N;SH3GL2;KCNH1;PCDHAC1;EOMES;ST6GAL2;SFMBT2;TMOD2;VASH2;EBF1;CNPY1;POU3F1;SHISA
ORF59;BROX;DYRK1A;PCDHA13;EREG;GPRIN3;MAF;CRISPLD2;HECW2;FAM185A;ADAM12;ELMO1;C

3;PCDHA7;PCDHAC2;PCDHAC1;PCDHA6
A2;PCDHA9;PCDHA8;PCDHAC2;PCDHA7;PCDHAC1;PCDHA6
CDHA3;PCDHA2;PCDHA9;PCDHA8;PCDHAC2;PCDHA7;PCDHAC1;PCDHA6

ER2;G3BP1;IGF2BP1;RBMS1;HNRNPC;RBM12;TARDBP;RBM7;SRSF10;PPARGC1B;SRSF9

XADR;CBFA2T2;ADRA2A;RUNX1;CREB1;RBL1;SNPH;DCX;TERF2IP;TNKS;CUL3;FUT11;ADD3;GIGYF2

C3;TFEB;PTPN11;GRIN2B;RAB11A;OCLN;MARCKS;CREB1;APC;SP4;PPP1R1B;DCX;CTNNB1
A;TRA2B;DPYSL3;E2F1;BSN;MARK1;FNBP4;CREBBP;PCYT1B;ZBTB16;GFPT1;NFATC3;SORBS3;ESR1

C;CREB1;TRA2B;S1PR1;PDCD4;CTNNB1;SIK2;RAF1;MXD1;PPARGC1B

;CREB1;SP1;SUB1;CDC37;CAPZA1;PPP1R1B;CDK1;CTNNB1;HNRNPC;TARDBP;RAD9A

NF800;CEP170;RNF111;AGFG2;APPL1;KPNA1;DGCR2;SCN1A;MEF2A;ZCCHC24;CSNK1G3;CXADR;WD
RPD3;PTGFRN;SLIT2;SH3GL2;KPNA1;MAP3K2;BCL11B;USP9X;ZBTB16;CNBP;GFPT1;FBXL17;HCFC2;
L5B;SPRED1;MAN1A2;DDI2;SNRPD3;RBM7;MBNL2;GRID1;ANGEL2;NSUN3;SLC30A4;RC3H1;LRRC40;
EPC1;ZNF800;CEP170;MAP3K9;SOX6;ZNF367;RNF111;MEF2A;KLF10;RAB2B;MBNL2;TMOD3;ANGEL2;
PTGFRN;SCN1A;USP2;SLC30A4;FNDC3B;DIO2;ARID5B;ZBTB34;GPCPD1;ACAP2;PGAM5;PAFAH1B2;S
ELK4;SPRED1;MAN1A2;DDI2;SNRPD3;MBNL2;GRID1;ANGEL2;NSUN3;SLC30A4;RC3H1;LRRC40;ARID5
3;EREG;GPRIN3;ACAP2;CRISPLD2;TBL1XR1;CMPK1;TFAM;TRIB2;GPM6A;MTMR3;YTHDC1;ABHD2;HS
P170;MAP3K9;SOX6;ZNF367;RNF111;MEF2A;RAB2B;MBNL2;TMOD3;ANGEL2;FNDC3A;EREG;DHX40;R
GPRIN3;ACAP2;CRISPLD2;TBL1XR1;CMPK1;TFAM;SET;MTMR3;PSMD11;YTHDC1;ABHD2;HSPA4L;GA
4;ARL5B;SERP1;DDI2;ZNF800;FNDC3A;DYNLL2;ZFX;RUNX1;TGFBR3;SFRP1;FCHSD2;ELMO1;TERF2I

P1

5;RUVBL1
PNA1;FNBP4;MAP2K1;XRCC5;SFMBT2;GAPVD1;HCFC2;SMC1A;DYNLL2;GATAD2B;PIAS1;DIAPH2;HN
3CLAF1;HNRNPK;SNPH;CAPRIN1;G3BP1;IGF2BP1;HNRNPC;CLSPN;ZNF148;TARDBP;LPHN2;RAPGEF
F7;CRBN;CSNK1A1;TPM3;XRCC5;VPS13C;TMOD3;YLPM1;MSL2;GAPVD1;GATAD2B;HNRNPK;MYO1C;
.;CEP170;SNRPD3;MYH10;KPNA1;TSFM;SEPT11;PCYT1B;CRBN;TPM3;XRCC5;TMOD3;ZFP91;ACTN4;
D2B;HK2;PCMT1;DIAPH2;HNRNPK;ZZZ3;FUBP1;RFWD3;CAPRIN1;G3BP1;CEP170;TARDBP
PGEF6
NPC;TARDBP

CDHA5;PCDHA4;CTNNB1;PCDHA3;PCDHA2;PCDHA9;PCDHA8;PCDHAC2;PCDHA7;PCDHAC1;PCDHA
HA8;PCDHA7;PCDHAC2;PCDHA6;PCDHAC1;CSNK1G3;CREBBP;FZD3;FZD5;CSNK1A1;NFATC3;PCDHE
;CTNNB1;RAF1;CALM1;CRK;EIF4E;AP2M1;PRKG1
SOS2

A2;PCDHA9;PCDHA8;PCDHAC2;PCDHA7;PCDHAC1;PCDHA6
CDHA3;PCDHA2;PCDHA9;PCDHA8;PCDHAC2;PCDHA7;PCDHAC1;PCDHA6
ER2;G3BP1;IGF2BP1;RBMS1;HNRNPC;RBM12;TARDBP;RBM7;SRSF10;PPARGC1B;SRSF9

A2;PCDHA9;PCDHA8;PCDHAC2;PCDHA7;PCDHAC1;PCDHA6
CDHA3;PCDHA2;PCDHA9;PCDHA8;PCDHAC2;PCDHA7;PCDHAC1;PCDHA6

FWD3;TRIM3;TRIM24;LONRF3;RNF111;RNF165;LNX2

RNF111;RAB11FIP5;TGM2;CREBBP;SMURF2;USP9X;TPM3;ZBTB16;DYRK1A;SMAD9;RUNX3;ESR1;RU
1;N4BP2;ABCA1;LYN;NCOA2;CREBBP;EBF1;PAX5;PROX1;DEK;ESR1;RUNX1;HIPK2;PIAS1;MAPK10;K
CDH2;ERBB4;PTK2B;MAPK1;CTNNB1;BSN;RAF1;CALM1;MYH10;APPL1
2;RUNX3;RUNX1;KAT2B;MYOD1;PGAM5;RAF1;HDAC5;USP15;INSIG2;UBA6;RNF180;DLST;XIAP;HIF1
K1;PCYT1B;CXADR;CBFA2T2;ADRA2A;RUNX1;CREB1;RBL1;SNPH;DCX;TERF2IP;DCTN2;TNKS;YTHD
3;PCDHA7;AP2M1;TNS1;PCDHA6;GAB1;PTPN11;PCDHA12;PCDHA11;PCDHA10;MAPK10;SP1;ID4;ELM
;XRCC5;ZBTB16;GTF2H1;PAX5;ESR1;RUNX1;PIAS1;KAT2B;SP1;CDC37;ZNF318;CDK1;CTNNB1;CALM
EF2A;RAB2B;CREBBP;USP9X;SMAD9;ESR1;FBXO30;PIAS1;SMAD7;OCLN;RBL1;SP1;MYOD1;CTNNB
;SNPH;CDC37;MYOD1;DCX;TFAM;TERF2IP;RAPGEF6;DTL;NUFIP2;YTHDC1;ADD3;GIGYF2;FOXO1;A
F2A;NCOA2;CREBBP;MAP2K1;ZBTB16;PAX5;PROX1;DEK;RUNX3;ESR1;RUNX1;HIPK2;SMAD7;KAT2B

AD7;SFRP1;CREB1;APC;TRPS1;CTNNB1;MAPK1;RAF1;FGFR1
RLIM;TARDBP;CRK;EIF4E;SMNDC1;CGGBP1;APPL1
MNDC1;CGGBP1

C5;ZNF148;TEAD1;FGFR1
CN1A;KPNA1;TGM2;LYN;ABCA2;CREBBP;BCL11B;TFEB;GRIN2B;SORL1;PIAS1;MAPK10;RCAN1;XK;C
CN1A;KPNA1;TGM2;LYN;ABCA2;CREBBP;BCL11B;TFEB;GRIN2B;SORL1;PIAS1;MAPK10;RCAN1;XK;C
NNB1;SPRY1;RAF1;FGFR1
BP;MME;ZBTB16;MMP2;DNMT3A;GAB1;PTPN11;DEK;RUNX1;HIPK2;ID2;CAMK4;CARM1;CTNNB1;CD2

H3GL2;SCN1A;MEF2A;EOMES;NCOA2;MAP2K1;MMP2;EBF1;IL17RD;RBX1;EREG;ENAH;CREB1;NRG3;

PYSL5;ERBB4;GPC1;DPYSL3;PLXNA2;MAPK1;SCN5A;SLIT2;SRGAP2;SCN3B;MYH10;AP2M1;SH3GL2;

)2;BTRC;SOX6;AP2M1;PRKG1;WLS;CREBBP;FZD3;FZD5;SMURF2;CSNK1A1;RBX1;SFRP1;APC;AGO1
AGO1;E2F1;TNRC6B
;ZNRF3;TNKS2;APC;RBBP5;RUVBL1;RSPO2;CTNNB1;BTRC;SOX6
REBBP;FZD5;CSNK1A1;GAB1;PTPN11;EREG;RBX1;KAT2B;HEYL;CREB1;NRG3;APC;TBL1XR1;CDC37;

;62;AGFG2;SCN1A;ST6GAL2;MBNL2;C5ORF15;FNDC3B;ACSL6;RC3H1;RUNX3;EREG;SFRP1;ADCY9;
RNF111;SCN1A;PRKAB2;ENTPD1;EBF1;ACTN4;FNDC3A;GAPVD1;HPCAL4;RUNX1;ADCY9;SUB1;TFAM
)C3B;EBF1;ACSL6;YOD1;HPCAL4;EREG;DHX40;SFRP1;CRISPLD2;SUB1;AGTR2;CGGBP1;KCTD16;ZN
B2;CXADR;ST6GAL2;FNDC3B;EBF1;RC3H1;FNDC3A;HPCAL4;SYTL4;RUNX1;CRISPLD1;TRIB2;CGGBI
CXADR;ST6GAL2;SFMBT2;FNDC3B;ACSL6;YOD1;FNDC3A;GAPVD1;EREG;RUNX1;TCEANC2;TRIB2;C
CXADR;ST6GAL2;SFMBT2;FNDC3B;ACSL6;YOD1;FNDC3A;GAPVD1;EREG;RUNX1;TCEANC2;TRIB2;C
CXADR;ST6GAL2;SFMBT2;FNDC3B;ACSL6;YOD1;FNDC3A;GAPVD1;EREG;RUNX1;TCEANC2;TRIB2;C
NDC3A;HCFC2;EREG;RUNX1;FAM133B;RNF126;CDIP1;CGGBP1;SET;MTMR3;IGSF3;DCUN1D5;MTMF
NDC3A;HCFC2;EREG;RUNX1;FAM133B;RNF126;CDIP1;CGGBP1;SET;MTMR3;IGSF3;DCUN1D5;MTMF
4;PRKAB2;ENTPD1;ST6GAL2;ALG6;RNASE6;FNDC3B;RC3H1;GAPVD1;HCFC2;FAM133B;SFRP1;COMI

;CDH2;APC;CAMK4;IGF2BP1;PTK2B;MAPK1;CTNNB1;RAF1;EIF4E
;SP1;E2F1;PTK2B;MAPK1;RAF1;SOS2;CRK;SH3GL2;AP2M1
)K1;SNIP1;MAPK1;BTRC;RAF1;RNF111
P2;GAB1;PTPN11;SOD2;SELE;RAB11A;RCAN1;OCLN;ITCH;CREB1;RCAN2;CTNNB1;RAF1;CRK

F1;SOS2;CRK;EIF4E

BMPR1A
RCAN2;MAPK1;CTNNB1;RAF1
;ADCY9;CREB1;MAPK1;PDE5A;RAF1;CALM1;PRKG1
M1;SOS2;CRK
C;TBL1XR1;RUVBL1;RSPO2;CTNNB1;BTRC

F1;FGFR1;BMPR1A

2;FGFR1

3;PCDHA12;HIPK1;PCDHA11;PCDHA10;RAP2C;CNOT6;TBL1XR1;SP1;SP4;RNF165;TNRC6B
TRN;TNRC6B

;RAP2C;KAT2B;SUB1;CARM1;JMY;CTNNB1

AD7;CREB1;RBL1;KANSL1;TBL1XR1;SP1;AGO1;MYOD1;CARM1;TFAM;NFE2L1

SSBP2;NFE2L1
BXO30;RBX1;RNF126;ITCH;CDC34;HECW2;RFWD3;KLHL7;RLIM;TRIP12;DTL;UBE2K;RNF165

BP2;NFE2L1

F1;GLYR1;AMER1;FYTTD1;HDAC5;NUFIP2;TNKS;PLAG1;YTHDC1;SRSF1;DTX1;HIF1A;ZBTB4;MTDH;N

GTF2H5;HIPK2;MYO1C;KANSL1;MYOD1;COMMD3-BMI1;TERF2IP

A2T2;RUNX3;FOXP2;PAX2;EREG;DKK3;SFRP1;CREB1;TBL1XR1;ZNF318;CGGBP1;KANK2;DLX1;HDAC
;PRDM16;ZNF423;PPARGC1B;TFAP2B;CREBBP;EGR4;FZD5;BCL11B;ZBTB16;SS18L2;NFATC3;INHBA;
A6;CREBL2;HIF1A;FOXO1;LRP6;NPAT;ERBB4;NSD1;PRDM16;NRIP1;E2F1;PCBD2;ZNF423;TRIM44;MA
A6;GLIS3;ZBTB4;HIF1A;FOXO1;MED12L;MTDH;PRDM16;ZNF148;PPARGC1B;ABCA2;TFAP2B;CREBBP
FNB3;PCDHA1;GBX2;ERBB4;PCDHA5;PCDHA4;PCDHA3;PCDHA2;PCDHA9;PCDHA8;PCDHA7;ATOH1;

CREB1;RBL1;TBL1XR1;SUB1;MYOD1;TET3;TFAM;DLX1;HDAC5;INSIG2;TNKS;PLAG1;GLIS3;HIF1A;FOX
M;ZNF275;SET;TSHZ3;GATA6;CREBL2;ZBTB4;HIF1A;FOXO1;MED12L;NPAT;ZNF706;PRDM16;HIVEP2;Z
318;NOS1AP;TFAM;MAPRE3;CRP;MYRF;ROCK1;GATA6;FAM129A;CREBL2;HIF1A;FOXO1;LRP6;NPAT;E
5;SET;TSHZ3;GATA6;PRICKLE1;HIF1A;ZBTB4;GIGYF2;FOXO1;BCLAF1;ZNF706;PRDM16;IGF2BP1;E2F

BF1;ACSL6;PCDHA13;PCDHA12;PCDHA11;PCDHA10;ADAM19;SUB1;RAPGEF6;DCP2;KCTD16;PPIC;R

PBP4;PAX2;ADRA2A;NRG3;ADAM12;LCOR;COMMD3-BMI1;TFAM;WDFY4;SLC29A3;PRTFDC1;CHST3;

PLA2G12A;PCGF3;LGI2;GAB1;HAUS3;NSG1;PHOX2B;EREG;LETM1;GPRIN3;MAPK10;FRAS1;TMEM3
IF2B2;TMED8;BCL11B;ITPK1;RNASE6;YLPM1;DIO2;SYNJ2BP;TSHR;TTC9;ALDH6A1;HNRNPC;CALM1;
IER1;ZNF275;BHLHB9;XIAP;DRP2;PGRMC1;LONRF3;MBNL3;RAI2;PLXNA3;MCTS1;IGBP1;MAGEE1;US
;IL17RD;SENP2;NAALADL2;SUMF1;KAT2B;ACAP2;RAP2B;TBL1XR1;CLDN18;CD47;DENND6A;COL6A5
3;FAM84A;RBMS1;MAPRE3;SLC25A12;TRIB2;DLX1;INSIG2;CUL3;ATL2;SLC1A4;HOXD12;TTL;GIGYF2;

NDC3A;HCFC2;RBX1;PIAS1;FAM133B;CDC37;SUB1;FAM185A;SIK3;CDIP1;WDFY3;RAF1;CGGBP1;MT
3;MAF;ADCY9;TBL1XR1;SUB1;FAM185A;SIK3;CDIP1;SLC25A51;TFAM;WDFY3;DENND6A;RAF1;CGGB
SA9;PCDHA11;FOXP2;SFRP1;MAF;CRISPLD2;HECW2;ADAM12;SIK2;KANK4;SHC4;DLX1;SHC2;PFKFB
L6;ATP11A;POU3F1;SHISA9;HPCAL4;DKK3;ADAM19;HECW2;ADAM12;NPTXR;KANK4;PPIC;SHC4;DLX
DC1;UBA6;HSPA4L;DCUN1D1;GSKIP;UBE2J1;NCKIPSD;BCL2L13;ZMAT3;MLEC;HIVEP2;SNX8;CREBB
2;MAF;CRISPLD1;CRISPLD2;FAM84A;SCG3;LPHN2;TRIB2;MAPRE2;PTGFR;HPGD;PLAG1;PDE1B;SH
OXP2;SFRP1;HECW2;ELMO1;ASPHD2;NPTXR;KANK4;KCTD16;SHC4;DLX1;TSHZ3;IGSF3;DLX6;RNF1
1;ACSL6;POU3F1;SHISA9;HPCAL4;FOXP2;DKK3;ADAM19;SFRP1;MAF;HECW2;WDFY3;NPTXR;KANK
9;ADAM19;GPRIN3;SFRP1;C1ORF115;CRISPLD2;HECW2;ELMO1;NPTXR;KANK4;SHC4;DLX1;GPM6A;
GGBP1;SET;DLX6;SPATA2;DTX4;MED12L;NKAIN1;ARHGAP20;LONRF3;RAI2;BCL11B;SMURF2;FAM78

;FOXO1;LRP6;ADD2;BCLAF1;RXRA;ATXN7;TRA2B;E2F1;GFPT1;NFATC3;YLPM1;SLC4A10;HELZ;ESR1

;RUNX1;PUM2;RCAN1;MARCKS;ADCY9;HNRNPK;RBL1;APC;SNPH;ID2;MYOD1;PPP1R1B;CCDC6;DC[illegible]

[illegible]R37;TMOD2;PIAS1;TGFBR3;ACAP2;CREB1;NRG3;TMEM33;IL1RAPL1;DCX;DENND6A;SMTNL2;CRK;[illegible]
TGFBR3;EFNA3;NCEH1;SP1;CDC37;SP4;AGO1;CAPZA1;PDCD4;DENND6A;PAFAH1B2;DCP2;PPIC;TN[illegible]
;ARID5B;PROX1;PIAS1;ADCY9;CREB1;ZNF318;SIK3;SLC25A12;CRK;SLC24A2;AMER1;STAU1;NUFIP2;
;FNDC3A;DYNLL2;EREG;PTP4A1;DHX40;RAP2C;KAT2B;CREB1;HECW2;TBC1D25;MAPRE3;WDFY2;D[illegible]
[illegible]LC29A3;AMER1;GPM6A;PPM1L;ZDHHC20;YTHDC1;INSIG2;PALM2;GSKIP;ZDHHC21;ZNF25;LRP8;GIG
5B;PROX1;PIAS1;ADCY9;CREB1;ZNF318;SIK3;SLC25A12;SLC24A2;AMER1;ZNF275;STAU1;NUFIP2;IN[illegible]
[illegible]SPA4L;GATA6;DCUN1D1;MTMR9;ZBTB4;HIF1A;NPAT;TMEM245;ZNF706;ZMAT3;VTI1A;ABCA1;RABGAP
[illegible]AP2C;KAT2B;HECW2;WDFY3;DENND6A;SLC25A12;RAF1;RAPGEF6;PAFAH1B2;DCP2;USP13;NAPB;S[illegible]
[illegible]TA6;MTMR9;SPATA2;ZBTB4;HIF1A;MED12L;UBE2J1;NPAT;TMEM245;ZNF706;ZMAT3;VTI1A;IP6K1;ZNF
P;ANKRD40;PPIC;PHLPP2;STAU1;NUFIP2;PLAG1;SRSF1;TMTC3;ABHD17C;HIF1A;FOXO1;NEURL1B;[illegible]

RNPK;NFIA;TBL1XR1;SP1;FUBP1;ZNF318;TARDBP
F6
;SP1;RFWD3;CAPRIN1;SERBP1;HNRNPC;TARDBP;WDFY4
GAPVD1;SMC1A;SMARCA1;GATAD2B;MYO1C;SP1;RFWD3;CAPRIN1;SERBP1;TARDBP

A6
B15;PCDHA13;PCDHA12;PCDHA11;PCDHA10;SFRP1;APC;TBL1XR1;CTNNB1

NX1;HIPK2;RBX1;SMAD7;KAT2B;HEYL;ITCH;RBL1;SP1;MYOD1;SIK3;CTNNB1;SLC25A12;CRK
AT2B;MAF;CREB1;NFIA;APC;SP1;CAMK4;MYOD1;CARM1;CTNNB1;FGFR1

;FOXO1;LRP6;ERBB4;USP1;E2F1;MAPK1;EIF4E;MARK1;CREBBP;SMURF2;ZBTB16;LAPTM5;GRIN2B;
C1;CUL3;FUT11;GSKIP;ADD3;GIGYF2;FOXO1;LRP6;ADD2;BCLAF1;RXRA;ATXN7;TRA2B;IGF2BP1;E2F
IO1;SOS2;FGFR1
I1;RAF1;RAD9A
1
DD2;PPP2CA;NPAT;BCLAF1;RXRA;ATXN7;TRA2B;IGF2BP1;E2F1;GSE1;MAPK1;MARK1;LYN;CREBBP;S
;MAF;CREB1;SP1;SUB1;CAMK4;MYOD1;CARM1;JMY;CTNNB1

REB1;APC;SP1;PPP1R1B;CTNNB1;TARDBP;UBE2K
REB1;APC;SP1;PPP1R1B;CTNNB1;TARDBP;UBE2K

244;FGFR1

;TBL1XR1;MYOD1;DCX;COL6A5;RAF1;SOS2;SHC2;PSMD11;ROCK1;CUL3;SEMA3A;GATA6;AGAP2;FO

;PLXNA3;SCN1A;LYN;MAP2K1;MMP2;PTPN11;L1CAM;IL17RD;GRIN2B;RBX1;EREG;ENAH;EFNA3;CRE

;CTNNB1;CALM1;TNRC6B

CTNNB1;FGFR1

CRISPLD1;SIK2;ZNF275;GPM6A;ADCYAP1R1;MTMR3;TSHZ3;IGSF3;DCUN1D5;TMTC3;ZNF25;GIGYF2;
I;AGTR2;TRIB2;PTGFR;DCUN1D5;DCUN1D1;TMTC3;MTMR9;GSKIP;ZNF25;UBE2J1;PCDHA1;PCDHA5;
IF275;GPM6A;DCUN1D5;ZBTB4;GIGYF2;UBE2J1;MTDH;APH1B;RIC8B;ZNF148;HOXC8;ABCA1;EDEM3;
P1;KCTD16;MTMR3;IGSF3;DCUN1D5;AARD;ZMPSTE24;UBE2J1;MTDH;APH1B;AP5M1;PCDHA1;PCDH
GGBP1;KCTD16;GPM6A;SET;IGSF3;SHOX2;DCUN1D1;AARD;ZNF25;MED12L;UBE2J1;MTDH;APH1B;Z
GGBP1;KCTD16;GPM6A;SET;IGSF3;SHOX2;DCUN1D1;AARD;ZNF25;MED12L;UBE2J1;MTDH;APH1B;Z
GGBP1;KCTD16;GPM6A;SET;IGSF3;SHOX2;DCUN1D1;AARD;ZNF25;MED12L;UBE2J1;MTDH;APH1B;Z
R9;MED12L;MTDH;CYB5R4;PCDHA1;MIER3;PRDM16;PCDHA5;PCDHA4;PCDHA3;PCDHA2;RBM12;RIM
R9;MED12L;MTDH;CYB5R4;PCDHA1;MIER3;PRDM16;PCDHA5;PCDHA4;PCDHA3;PCDHA2;RBM12;RIM
MD3-BMI1;AGTR2;KCTD16;ZNF275;PTGFR;MTMR3;IGSF3;DCUN1D5;SHOX2;CYB5R4;MCMBP;RBM12;

IPAT;NXF1;BCLAF1;SRSF10;ZC3H14;S100PBP;CREBBP;TRIM41;CBX5;SMURF2;CSNK1A1;ZBTB16;YLI

C5;SET;TSHZ3;GATA6;GLIS3;PRICKLE1;LRP8;ZBTB4;FOXO1;MTDH;BCLAF1;RXRA;ZNF706;NSD1;PRD
SMARCA1;GDF6;ESR1;PHOX2B;SMAD7;HNRNPK;SP1;AGO1;ID2;LHX4;NFE2L1;BMPR1A;BMPR2;ONE
AP3K2;TFAP2B;CREBBP;ZBTB16;CNBP;SS18L2;INHBA;TBX5;SMARCA1;GDF6;ESR1;HIPK2;SP1;ID2;CA
P;CBX6;CBX5;EGR4;FZD5;BCL11B;SMURF2;ZBTB16;SS18L2;NFATC3;SMAD9;INHBA;GDF6;ESR1;PHO
;PCDHA6;LYN;EIF2B2;FZD5;BCL11B;ZBTB16;SLC4A10;PCDHB15;INHBA;L1CAM;S100B;PHOX2B;TSHI

XO1;LRP6;RXRA;PRDM16;NRIP1;E2F1;PPARGC1B;ZNF462;TFAP2B;CREBBP;EGR4;FZD5;BCL11B;CNE
ZNF423;ZNF148;PPARGC1B;SNX6;ABCA2;TFAP2B;CREBBP;CBX5;ZBTB14;BCL11B;SMURF2;XRCC5;Z
ERBB4;NSD1;PRDM16;NRIP1;E2F1;MAPK1;PCBD2;ZNF423;TRIM44;MAP3K2;SRPK2;TFAP2B;CREBBP
1;ZNF148;ZNF423;SNX6;TFAP2B;CBX5;ZBTB14;SMURF2;TIPARP;XRCC5;ZBTB16;ESR1;HEYL;ID2;CAI

RNF180;NPR3;RHOBTB3;MAT2B;JAKMIP2;PPP2CA;NEURL1B;PURA;PCDHA1;MIER3;NSD1;G3BP1;PCD

PFKFB3;ARL3;FUT11;ADD3;ZNF25;PAPSS2;TNKS2;VTI1A;EIF4EBP2;MCMBP;SMNDC1;ACBD5;KCNIP2

3;STIM2;OSTC;OCIAD1;WDFY3;PDE5A;SPRY1;NAT8L;UBE2K
;SOS2;TECPR2;DNAL1
SP9X;GABRA3;L1CAM;SMARCA1;CLCN5;GLUD2;DIAPH2;XK;RPA4;RLIM;SPRY3;OTC
;RAF1;CGGBP1;FYTTD1;USP13;PPM1L;SHOX2;DCUN1D1;PDHB;MED12L;NCKIPSD;FBXO40;ATXN7;Z
FAM117B;ADD2;TRAK2;DFNB59;GBX2;MAT2A;SERTAD2;ERBB4;MGAT5;GPC1;VPS54;RAB11FIP5;MAF

MR3;PSMD11;YTHDC1;CUL3;SPATA2;HIF1A;NOP9;MTDH;NPAT;RNF214;RAP1A;ATXN7;RNF217;VTI1A;
P1;SRSF9;MTMR3;DCUN1D5;YTHDC1;FUT11;DCUN1D1;TMTC3;MTMR9;HIF1A;GIGYF2;FOXO1;NOP9;
3;FAM114A1;PDE1B;SHOX2;NPR3;GATA6;GLIS3;DTX1;APCDD1;TRHDE;MED12L;FBLN5;UBE2J1;CTIF;
1;SHC2;ADCYAP1R1;PFKFB3;TSHZ3;DLX6;SHOX2;AGAP2;GLIS3;DTX1;AARD;TRHDE;MED12L;FBLN5
P;TMEM86A;CPSF7;ST13;NEK7;SREK1IP1;LNPEP;HOOK3;MARCH7;ZZZ3;SESTD1;FUBP1;STRN;MXD
OX2;PRUNE2;FAM129A;NRXN3;PRICKLE1;PPM1E;TRHDE;COL19A1;CYTH3;SERTAD2;ERBB4;TRA2B;
80;SHOX2;AGAP2;GATA6;DTX1;APCDD1;TRHDE;MED12L;FBLN5;GJC1;ARHGAP20;EFNB3;PRDM16;S
4;DLX1;FAM114A1;DLX6;SHOX2;NPR3;GATA6;GLIS3;RHOBTB3;MED12L;FBLN5;GJC1;NKAIN1;ARHGA
SHC2;ADCYAP1R1;SHOX2;AGAP2;GATA6;DTX1;APCDD1;TRHDE;FBLN5;GJC1;NKAIN1;ARHGAP20;EF
A;ST13;ZBTB16;ST8SIA3;LNPEP;RCAN1;GLUD2;SP1;STRN;LRP12;FGFR1;NRP2;BMPR2;DUSP19;CTD

;RCAN1;C20ORF112;MARCKS;ARHGAP31;NFIA;APC;FUBP1;ID2;PPP1R1B;CCDC6;CTNNB1;NFE2L1

X;TFAM;TERF2IP;RAPGEF6;DTL;SRSF9;RAD9A

PAFAH1B2;SLC24A2;NAPB;PPM1L;IGSF3;NPR3;NDNF;PPM1K;FAM117B;JAKMIP2;CYTH3;RNF214;ZNR
RC6B
;INSIG2;PLAG1;ADH1B;SHOX2;TMTC3;SPATA2;TUBD1;DTX4;LRP8;PAPSS2;LRP6;ADD2;PURA;RIC8B;
ENND6A;SLC25A12;RAF1;RAPGEF6;PAFAH1B2;DCP2;USP13;NAPB;SF3B3;NUFIP2;TNKS;YTHDC1;PR
YF2;FAM117B;MTDH;LRP6;JAKMIP2;TMEM245;MIER3;NSD1;PLXNA2;MYH10;PPARGC1B;TRIM44;GAL
SIG2;PLAG1;ADH1B;SHOX2;TMTC3;SPATA2;TUBD1;DTX4;LRP8;FAM117B;PAPSS2;LRP6;ADD2;PURA;
1L;ACBD5;EIF2B2;CBX5;SREK1IP1;SMAD9;HOOK3;ABHD15;SMAD7;CNOT6;AGO1;SP4;NABP1;DNAL
F3B3;NUFIP2;TNKS;PRUNE2;SPATA2;GSKIP;XIAP;CREBL2;ABHD17C;ZBTB4;MED12L;TRAK2;ZDHHC1
148;ABCA1;RABGAP1L;ACBD5;EIF2B2;CBX5;SREK1IP1;HOOK3;ABHD15;SMAD7;AGO1;SP4;NABP1;M
RXRA;RBBP5;MIER3;NSD1;TMEM68;GSE1;APBB2;ZNF148;ABCA1;PHC2;ZBTB10;UBE2G1;HIPK2;ITCH

;ESR1;EPOR;SMAD7;RCAN1;ITCH;HNRNPK;APC;SP1;SP4;CDK1;PDCD4;CTNNB1;TARDBP;FGFR1
1;GSE1;MAPK1;GFPT1;NFATC3;YLPM1;SLC4A10;HELZ;GRIN2B;SORBS3;ESR1;U2SURP;RCAN1;C20C

ORT1;ZBTB16;GFPT1;NFATC3;SORBS3;ESR1;GATAD2B;U2SURP;PUM2;RCAN1;MARCKS;HNRNPK;A

XO1;PPP2CA;RAP1A;APH1B;RXRA;EFNB3;RBBP5;ERBB4;GPC1;PLXNA2;MAPK1;SLIT2;SCN3B;SRGA

:B1;NRG3;DCX;COL6A5;CALM1;RAF1;SOS2;FGFR1

;MED12L;ZMPSTE24;MTDH;SNX1;APH1B;ZNF706;PCDHA1;ERI1;PCDHA5;PCDHA4;MLEC;PCDHA3;PC
;PCDHA4;GSE1;PCDHA3;PCDHA2;RBM12;SCN3B;ZNF148;PCDHA9;PCDHA8;PPARGC1B;PCDHA7;PCI
;MCTS1;SRPK2;CADM3;FZD3;FZD5;BCL11B;ST13;EDEM1;PUM2;OCLN;SESTD1;FUBP1;ID4;CDS2;DIR
A5;PCDHA4;PCDHA3;PCDHA2;RIMKLA;PCDHA9;PCDHA8;PPARGC1B;PCDHA7;RAB11FIP5;PCDHA6;B
:NF706;PCDHA1;ERI1;MIER3;PCDHA5;PCDHA4;PCDHA3;PCDHA2;RBM12;RIMKLA;ZNF148;PCDHA9;P
:NF706;PCDHA1;ERI1;MIER3;PCDHA5;PCDHA4;PCDHA3;PCDHA2;RBM12;RIMKLA;ZNF148;PCDHA9;P
:NF706;PCDHA1;ERI1;MIER3;PCDHA5;PCDHA4;PCDHA3;PCDHA2;RBM12;RIMKLA;ZNF148;PCDHA9;P
KLA;ZNF148;PCDHA9;PCDHA8;PPARGC1B;PCDHA7;SNX6;PCDHA6;SRPK2;BCL11B;FAM78A;EDEM1;
KLA;ZNF148;PCDHA9;PCDHA8;PPARGC1B;PCDHA7;SNX6;PCDHA6;SRPK2;BCL11B;FAM78A;EDEM1;
;ZNF148;N4BP2;PPARGC1B;ABCA1;B3GALNT2;CREBBP;FZD3;BCL11B;ST8SIA3;PARVA;PUM2;HNRNF

PM1;HIPK2;MYO1C;TRIP12

M16;NRIP1;E2F1;ZNF423;ZNF148;SNX6;IGBP1;TFAP2B;CREBBP;CBX6;CBX5;ZBTB14;SMURF2;USP9
CUT2;SIX1;RSF1;LITAF;CDC73;MED17;PHF8;CKS1B;MED14;MECOM;NKRF;SIN3A;TEAD1;UTP15;KLF
MK4;CTNNB1;NFE2L1
X2B;SMAD7;HNRNPK;SP1;AGO1;SP4;JMY;ID4;LHX4;MXD1;NFE2L1;CC2D1B;BMPR1A;BMPR2;ONECU
;RCAN1;FGFR1

P;SS18L2;FOXJ2;NFATC3;SMAD9;GTF2H1;INHBA;TBX5;ESR1;PHOX2B;HIPK2;SMAD7;HEYL;HNRNP
BTB16;SS18L2;NFATC3;INHBA;SMARCA1;GDF6;ESR1;SP1;SP4;ID2;JMY;ID4;NFE2L1;CREBZF;BTG1;S
;ZBTB16;CNBP;SS18L2;INHBA;DEK;TBX5;SMARCA1;GDF6;ESR1;HIPK2;MYO1C;SP1;ID2;FUBP1;CAM
RIN1;RLIM;PDCD4;ID4;CTNNB1;TARDBP

DHA5;PCDHA4;PCDHA3;PCBD2;PCDHA2;PCDHA9;PCDHA8;PPARGC1B;PCDHA7;PCDHA6;GABRA1;TF

2;GPAM;CCDC6;PDCD4;CDK1;BMPR1A

ZMAT3;TRA2B;IP6K1;RPP14;ZNF148;TIMMDC1;CRBN;TIPARP;EDEM1;CNBP;SS18L2;ATP2B2;U2SURP;
P3K2;SEPT10;EGR4;FZD5;XRCC5;GFPT1;SLC4A10;NBEAL1;PLEKHA3;KLHL23;MARCH7;ASPRV1;PUM

;EIF4EBP2;MLEC;RBM12;PPARGC1B;SNX6;ABCA1;EDEM3;CREBBP;EDEM1;NBEAL1;TRAPPC8;HELZ;
UBE2J1;BCL2L13;AP5M1;VTI1A;IP6K1;ZC3H14;SNX6;CREBBP;CPSF7;NBEAL1;LNPEP;TRAPPC8;HEL
;ARHGAP20;RNF217;PRDM16;PCDHA4;PCDHA3;HIVEP2;HOXC8;RAI2;MARK1;LYN;ABCA1;TFAP2B;CA
5;GJC1;NKAIN1;EFNB3;PRDM16;SCN3B;RIMKLA;MARK1;TFAP2B;CADM3;FZD3;EGR4;BCL11B;ZBTB16
1;SERINC3;UBE2K;TECPR2;NFE2L1;LRP12;CC2D1B;DOCK5;NRP2;DYRK3;MOCS3;BMPR2;PRR3;PPW
PLXNA2;HAS2;GPC5;LONRF3;RAI2;MARK1;LYN;ZNF462;SEPT10;ZBTB16;SMAD9;DEK;SMARCA1;SOF
CN3B;MARK1;TFAP2B;FZD3;EGR4;BCL11B;NSG1;GDF6;SYT7;PHOX2B;REEP1;EFNA3;PPP1R1B;LHX
AP20;EFNB3;PRDM16;PLXNA2;PCDHA2;MARK1;LYN;FZD3;EGR4;ZBTB16;PARVA;NSG1;SMARCA1;GDF
FNB3;CHAD;TAC3;LONRF3;RAI2;SYT5;TFAP2B;CADM3;EGR4;ZBTB16;ST8SIA3;SLC4A10;SMAD9;GDF6
SPL2;PPP1R9A;PITPNC1;ELAVL2;ADAMTS2;SPRED1;KIF1B;CCDC50;GPR37;MME;ANO6;CDYL2;ADR

:F2;TNKS2;ATXN7;TMEM248;MAPK1;MYH10;SLC25A24;ABCA1;MAP3K2;TFAP2B;CADM3;RABGAP1L;S

SERTAD2;ZMAT3;UBN2;FAM177A1;NRIP1;IGF2BP1;LONRF3;MBNL3;SRGAP2;EMC7;RPP14;CA12;ACB
:UNE2;SPATA2;GSKIP;XIAP;CREBL2;ABHD17C;LRP8;ZBTB4;MED12L;TRAK2;ZDHHC18;RAP1A;ATXN7;
_NT7;RABGAP1L;NFATC3;PCDHB15;HELZ;NSG1;HIPK1;AZIN1;APC;CAMK4;NABP1;TRIP12;TNRC6B
RIC8B;SERTAD2;ZMAT3;NRIP1;IGF2BP1;LONRF3;MBNL3;SRGAP2;EMC7;RPP14;CA12;ACBD5;CBX6;F
1;LRP12;BMPR2;ANKRD33B;SMG7;MYPN;AFF1;ELAVL2;MED17;SPRED1;DDI2;ZNF800;CEP170;KLF10
18;RAP1A;ATXN7;ZMAT3;ERBB4;UBN2;MLEC;MAPK1;MBNL3;ZNF423;B3GALNT2;ABCA1;ACBD5;CBX5
IXD1;DNAL1;LRP12;BMPR2;ANKRD33B;CALCOCO2;PDE3B;SMG7;RND3;PFAS;MYPN;AFF1;ELAVL2;M
l;MYO1C;APC;GPAM;SP1;PITHD1;SP4;CARM1;SERBP1;JMY;ID4;CCNYL1;TARDBP

ORF112;MARCKS;ARHGAP31;NFIA;APC;KANSL1;FUBP1;ID2;PPP1R1B;CCDC6;CTNNB1

APC;KANSL1;SP1;ID2;PPP1R1B;CCDC6;CTNNB1

AP2;MYH10;PLXNA3;LYN;CREBBP;LGI2;PTPN11;L1CAM;GRIN2B;CNOT6;EFNA3;CARM1;CTNNB1;CALM

:DHA2;PCDHA9;N4BP2;PCDHA8;PPARGC1B;PCDHA7;RAB11FIP5;PCDHA6;MCTS1;SRPK2;FZD3;FZD5
DHA6;MCTS1;CREBBP;FZD3;EDEM1;ST8SIA3;RASSF8;SMARCA1;HNRNPK;SESTD1;STRN;TECPR2;D
AS2;FMN1;PFAS;IL18BP;MRPL42;UBL3;SERP1;SH3PXD2A;C1QBP;GNRHR;RSPO2;KIF1B;CTBS;ZNF3
33GALNT2;MCTS1;CREBBP;FZD3;EDEM1;ST8SIA3;PARVA;SMARCA1;STRN;SLC7A14;FMN1;PHF8;MR
'CDHA8;PCDHA7;PCDHA6;MCTS1;PHC2;FZD5;BCL11B;TIPARP;ST13;NFATC3;RASSF8;SESTD1;STRN
'CDHA8;PCDHA7;PCDHA6;MCTS1;PHC2;FZD5;BCL11B;TIPARP;ST13;NFATC3;RASSF8;SESTD1;STRN
'CDHA8;PCDHA7;PCDHA6;MCTS1;PHC2;FZD5;BCL11B;TIPARP;ST13;NFATC3;RASSF8;SESTD1;STRN
ST8SIA3;SESTD1;STRN;HNRNPC;CDS2;BTG1;MOCS3;SH3KBP1;DIRAS2;FMN1;JPH3;SH3PXD2A;KIF1
ST8SIA3;SESTD1;STRN;HNRNPC;CDS2;BTG1;MOCS3;SH3KBP1;DIRAS2;FMN1;JPH3;SH3PXD2A;KIF1
'K;NCEH1;SESTD1;STRN;HNRNPC;CALM1;TECPR2;DAND5;BTG1;PHF20;FMN1;BMI1;MRPL42;UBL3;M

X;XRCC5;ZBTB16;CNBP;ESR1;GATAD2B;SMAD7;HEYL;NFIA;ID2;PDCD4;ID4;RLIM;CTNNB1;MXD1;FRK
F10;NCOA2;TFEB;PROX1;PAX2;KAT2B;CREB1;RBL1;MYOD1;TET3;MAPRE3;HDAC5;MYRF;PLAG1;LRP

T2;SIX1;BMI1;LITAF;CDC73;MED17;SDR16C5;MED14;MECOM;NKRF;SIN3A;C1QBP;TRPS1;ZNF367;TE

K;NFIA;SP1;AGO1;ASXL3;CTNNB1;LHX4;SSBP2;NFE2L1;BMPR1A
SIX1;RSF1;LITAF;ELAVL2;MED17;PHF8;CKS1B;ING4;MED14;MECOM;NKRF;SIN3A;ZNF367;TEAD1;KLF
K4;CTNNB1;NFE2L1

RIM41;SRD5A1;CSNK1A1;PCDHB15;FBXL17;SREK1IP1;LNPEP;CNOT6;OCLN;CDKN2AIPNL;APC;ZFP6

;STXBP5L;BFSP2;ARHGAP31;NCEH1;CTNNB1;OSTN
12;REEP1;SESTD1;ID2;CCNYL1;NABP1;STRN;FAM171B;TRIP12;PTPN4;MXD1;PLEKHM3;ITM2C

;BFAR;MARCH7;LSM5;ALDH6A1;CNOT6;HNRNPK;ZFP62;SESTD1;OSTC;FUBP1;JMY;STRN;MXD1;PLE
Z;HOOK3;LSM5;AZIN1;PUM2;CNOT6;OCLN;HNRNPK;ZZZ3;FUBP1;UBE2K;LRP12;BMPR1A;RBM28;CO
ADM3;FZD3;EGR4;BCL11B;FAM78A;ZBTB16;SMAD9;PARVA;NSG1;GRIN2B;SYT7;PHOX2B;ABHD15;RE
;SLC4A10;SMAD9;NSG1;GDF6;SYT7;PHOX2B;ABHD15;RCAN2;PPP1R1B;ID4;LHX6;LHX4;HRK;GPR27
VD1;RSF1;CTDSPL2;SMG7;AFF4;CDC73;MED17;ADAMTS5;DTWD1;MED14;SART3;SIPA1L1;XPO4;DDI
RL1;TSHR;STXBP5L;MARCKS;KIF26B;RCAN2;CAMK4;LHX6;LHX4;LRP12;ITM2C
6;LHX4;HRK;GPR27;NRP2;ONECUT2;DIRAS2;SIX1;BMI1;JPH1;HK2;ADAMTS5;JPH3;MECOM;SCN5A;E
F6;PHOX2B;OCLN;EFNA3;PPP1R1B;LHX6;LHX4;BMPR1A;FGFR1;HRK;GPR27;BMPR2;ANKRD33B;KCN
;GRIN2B;PHOX2B;REEP1;EFNA3;RCAN2;ID4;LHX6;LHX4;HRK;GPR27;ONECUT2;KCNC2;CELF3;DIRA
A2A;PTP4A1;KAT2B;RAP2A;IL1RAPL1;NOS1AP;NAT8L;MAPRE2;FYTTD1;PPM1L;PPM1H;ZDHHC21;ZB

ORT1;NFATC3;SMAD9;LNPEP;TRAPPC8;PLEKHA3;HIPK1;AZIN1;PUM2;REEP1;PARP11;FRAS1;NFIA;A

D5;CBX6;FZD5;PRRX1;ZBTB16;ATP2B4;SMAD9;GTF2H1;PLEKHA3;KLHL23;L1CAM;GRIN2B;EPOR;AD
;ZMAT3;UBN2;MLEC;MAPK1;MBNL3;ZNF423;HOXC8;B3GALNT2;ABCA2;ACBD5;CBX5;ST13;FAM46C;G

FZD5;PRRX1;ZBTB16;ATP2B4;SMAD9;GTF2H1;PLEKHA3;KLHL23;L1CAM;GRIN2B;EPOR;ADAT2;MARC
;WDR37;VPS13C;MMP2;GAB1;CBFA2T2;SUMF1;RAP2C;PTP4A1;KAT2B;CREB1;RBL1;CNKSR3;TET3;
;FAM46C;GFPT1;TMEM64;ESR1;GATAD2B;AZIN1;CNOT6;NFIA;GPAM;AGO1;SERBP1;RLIM;SERINC3;
ED17;SPRED1;SH3PXD2A;DDI2;ZNF800;CEP170;UNKL;KLF10;NAA30;WDR37;VPS13C;GAB1;ZFP91;C

M1;FGFR1

5;FAM78A;NBEAL1;RASSF8;GDF6;ALDH6A1;OCLN;NCEH1;STRN;TECPR2;CDS2;GABRB1;PHF20;SETD
DIRAS2;SLC7A14;LITAF;PFAS;PHF8;MED14;UBL3;MECOM;XPO4;SH3PXD2A;SH3BGRL2;SOSTDC1;ZN
67;TEAD1;APPL1;ZCCHC24;MME;NAALADL2;ENAH;RAP2A;TMEM33;CNKSR3;MICU3;CD47;RAPGEF6
PL42;SERP1;GNRHR;CTBS;ZCCHC24;PCYT1B;ANO6;SORCS1;ZFY;IL17RD;CBFA2T2;DYNLL2;RAP2C;
;CC2D1B;SH3KBP1;CHURC1-FNTB;DIRAS2;UBL3;SERP1;XPO4;UBXN7;KIF1B;EMB;ZCCHC24;WDR37;
;CC2D1B;SH3KBP1;CHURC1-FNTB;DIRAS2;UBL3;SERP1;XPO4;UBXN7;KIF1B;EMB;ZCCHC24;WDR37;
;CC2D1B;SH3KBP1;CHURC1-FNTB;DIRAS2;UBL3;SERP1;XPO4;UBXN7;KIF1B;EMB;ZCCHC24;WDR37;
1B;EMB;TEAD1;NDC1;PCYT1B;ITPK1;ANO6;CBFA2T2;GNL1;CDYL2;RAP2A;RAP2B;TMEM33;TET3;MIC
1B;EMB;TEAD1;NDC1;PCYT1B;ITPK1;ANO6;CBFA2T2;GNL1;CDYL2;RAP2A;RAP2B;TMEM33;TET3;MIC
MECOM;SH3PXD2A;RSPO2;UBXN7;KIF1B;EMB;CTBS;ZNF367;WDR37;ITPK1;GAB1;SORCS1;IL17RD;G

K;CC2D1B
'6;BCLAF1;RXRA;ERBB4;NSD1;NRIP1;E2F1;PCBD2;TRIM44;MAP3K2;ZNF462;CNBP;FOXJ2;GTF2H1;TE

EAD1;ZNF366;KLF10;NCOA2;TFEB;PROX1;PAX2;TGFBR3;KAT2B;CREB1;RBL1;MYOD1;TET3;CRK;KAN

10;NCOA2;TSFM;USP47;TFEB;PROX1;CBFA2T2;PAX2;CREB1;MYOD1;TERF2IP;MAPRE3;CRK;USP13;

;2;CAMK4;JMY;SSBP2

:KHM3;LRP12;ITM2C;CDS2;CREBZF;BTG1;MOCS3;BMPR2;CELF1;PTPRM;SMG7;CDC73;MED17;SPRE
)X7B;MOCS3;CELF1;PDE3B;CDC73;MED17;ADAMTS2;SPRED1;SERP1;NKRF;MAN1A2;SH3BGRL2;UB)
:EP1;EFNA3;ID2;PPP1R1B;LHX4;HRK;GPR27;GABRB1;ANKRD33B;ONECUT2;KCNC2;PDE3B;SLC7A14
';NRP2;ANKRD33B;KCNC2;CELF3;PDE3B;SIX1;RCSD1;JPH1;NHSL2;ADAMTS2;JPH3;MECOM;SH3PXD
2;TRIM3;SH3BGRL2;KIF1B;METTL16;ARL5A;TEAD1;APPL1;UTP15;USP47;PLA2G12A;TTC33;ITPK1;HA

:MB;PRKG1;HS3ST3B1;GRID1;SORCS1;PAX5;PROX1;ADRA2A;NRG3;SNPH;NAT8L;MYRF;PLAG1;NDN
NC2;TTC22;CELF3;PTPRM;SIX1;BMI1;PPP1R9A;JPH1;NHSL2;ADAMTS2;MECOM;TRIM3;TMEM108;SH3
AS2;PDE3B;SLC7A14;PPP1R9A;ELAVL2;ADAMTS5;NHSL2;KIAA1549;ADAMTS2;JPH3;SLC22A17;RSPO
TB44;FAM117B;PAPSS2;C21ORF119;EFS;MAT2A;UBN2;NSD1;HAS2;MBNL3;MPZL3;ATOH1;CA12;PLEK

ABHD17B;SESTD1;AGO1;FUBP1;ID2;KLHL7;ASB7;STRN;PDE7B;SERINC3;TARDBP;CDS2;LNX2

AT2;MARCKS;NFIA;SP1;RCAN2;FUBP1;ID4;NABP1;STRN;MXD1;UBE2K;PLEKHM3;NFE2L1;BMPR1A
GFPT1;TMEM64;HIPK1;ESR1;GATAD2B;AZIN1;CNOT6;NFIA;GPAM;SESTD1;AGO1;SERBP1;RLIM;SERIN

CKS;NFIA;SP1;RCAN2;FUBP1;ID4;NABP1;STRN;MXD1;PLEKHM3;NFE2L1;LRP12;BMPR1A
MAPRE3;CD47;ERGIC2;CRK;ZNF597;KANK2;PHLPP2;SF3B3;ATL3;NUFIP2;ZDHHC20;FAM129A;XIAP;A
CALM1;MXD1;C11ORF57;TNRC6B
6ORF62;RAP2C;PTP4A1;KAT2B;CREB1;CNKSR3;TET3;MAPRE3;CD47;ERGIC2;CRK;ZNF597;PHLPP2;

9;SLC7A14;JPH1;LITAF;MED14;SH3PXD2A;GNRHR;SH3BGRL2;ZNF800;KIF1B;CTBS;ZNF367;ZCCHC
F800;ZNF367;LRRC40;C6ORF62;GNL1;RAP2C;RAP2B;CLDN18;CHST3;KANK2;FYTTD1;ZDHHC20;FAM
;PAFAH1B2;DCP2;FYTTD1;NAPB;PRUNE2;GPALPP1;FAM129A;XIAP;ZDHHC21;TRAK2;USP1;GPC5;ME
;RAP2B;TMEM33;RAPGEF6;PAFAH1B2;RNF165;STAU1;ATL2;XIAP;ZDHHC21;PAPSS2;BCLAF1;UBN2;
;GAB1;C6ORF62;IL17RD;GNL1;ENAH;CNKSR3;TET3;CLDN18;ZNF236;RAPGEF6;PAFAH1B2;DCP2;CH
;GAB1;C6ORF62;IL17RD;GNL1;ENAH;CNKSR3;TET3;CLDN18;ZNF236;RAPGEF6;PAFAH1B2;DCP2;CH
;GAB1;C6ORF62;IL17RD;GNL1;ENAH;CNKSR3;TET3;CLDN18;ZNF236;RAPGEF6;PAFAH1B2;DCP2;CH
U3;CD47;ATL2;ZDHHC21;FAM117B;COL19A1;PAPSS2;PURA;PGRMC1;BCLAF1;SERTAD2;UBN2;NSD1
U3;CD47;ATL2;ZDHHC21;FAM117B;COL19A1;PAPSS2;PURA;PGRMC1;BCLAF1;SERTAD2;UBN2;NSD1
NL1;NAALADL2;RAP2C;TMEM33;CNKSR3;PGAM5;CD47;ZNF236;SMTNL2;PAFAH1B2;ZDHHC20;FAM1

3X5;HIPK2;HEYL;NFIA;ASXL3;CAMK4;CTNNB1;SSBP2

NK2;HDAC5;USP15;PLAG1;XIAP;LRP8;LRP6;RXRA;NSD1;NRIP1;E2F1;IGBP1;ZNF462;USP9X;CNBP;FO

HDAC5;MYRF;PRICKLE1;LRP6;PPP2CA;BCLAF1;ZNF629;ERBB4;NSD1;NRIP1;E2F1;APBB2;PCBD2;SF

:D1;C1QBP;UBXN7;CEP170;ARL5A;UNKL;APPL1;DGCR2;MAPK1IP1L;ARHGEF10;USP47;DFFA;NAA30
XN7;CEP170;ARL5A;CCDC50;UBL7;UTP15;USP47;TMED8;VPS13C;SORD;LRRC40;PROX1;CBFA2T2;S
;PTPRM;SIX1;RCSD1;BMI1;JPH1;ADAMTS5;NHSL2;ADAMTS2;JPH3;MECOM;SLC22A17;RSPO2;TMEM
)2A;SLC22A17;TRPS1;TMEM108;EMB;PRKG1;GPR135;CCDC112;HS3ST3B1;ZCCHC24;GPR37;GRID1;
AUS3;SENP2;GNL1;RBL1;RRM2B;TERF2IP;CD47;FYTTD1;USP13;RALA;PPM1L;ROCK1;ZDHHC20;PALM

IF;HOXD12;PPM1E;ADD2;JAKMIP2;EFS;GBX2;IGF2BP1;S1PR1;HAS2;GPC5;S1PR3;ATOH1;PRRX1;TPE
3BGRL2;SCN5A;PRKG1;GPR135;HS3ST3B1;GRID1;GAB1;PAX5;PROX1;IL17RD;ADRA2B;PAX2;ADRA2
2;TMEM108;SCN5A;PRKG1;MME;GRID1;GAB3;SORCS1;PAX5;PROX1;ADRA2A;NRG3;MYOD1;NOS1A
KHA3;TMEM64;HIPK1;TSHR;TTC9;FRAS1;NFIA;KIF26B;ASXL3;YPEL2;TNRC6B

NC3;PTPN4;CALM1;MXD1;C11ORF57;TNRC6B

BHD17C;PDHB;FAM117B;PURA;MAT2A;TNKS2;E2F1;HAS2;MAPK1;MAP3K2;TRIM41;PRRX1;CSNK1A1

;SF3B3;ATL3;LUZP1;NUFIP2;ZDHHC20;PRUNE2;PPM1H;FAM129A;XIAP;PDHB;FAM117B;PURA;ZNRF3

24;PCYT1B;GAB1;ANO6;SORCS1;PAX5;ZFY;IL17RD;ZFX;GNL1;ENAH;RAP2B;TMEM33;MICU3;CD47;P
129A;FAM117B;TRAK2;NEURL1B;GBX2;UBN2;CARNS1;HAS2;MBNL3;SRSF10;MAP3K2;CA12;TIMMDC
NL3;SRSF10;MAP3K2;USP9X;FAM46C;PCGF3;UBE2G1;SYNJ2BP;HIPK2;ITCH;DPY19L2;TMEM56;ET
BNL3;SRSF10;MAP3K2;GABRA1;CSNK1A1;GTF2H1;TMEM64;GTF2H5;STXBP5L;TTC9;XK;DPY19L2;N
ST3;RNF165;NAPB;ATL2;TUBD1;ZDHHC21;GRAMD4;PAPSS2;NEURL1B;PURA;PGRMC1;BCLAF1;ZDH
ST3;RNF165;NAPB;ATL2;TUBD1;ZDHHC21;GRAMD4;PAPSS2;NEURL1B;PURA;PGRMC1;BCLAF1;ZDH
ST3;RNF165;NAPB;ATL2;TUBD1;ZDHHC21;GRAMD4;PAPSS2;NEURL1B;PURA;PGRMC1;BCLAF1;ZDH
;APBB2;MBNL3;MAP3K2;CA12;USP9X;SORT1;CSNK1A1;YLPM1;TMEM64;HIPK1;SYNJ2BP;RAB11A;IT
;APBB2;MBNL3;MAP3K2;CA12;USP9X;SORT1;CSNK1A1;YLPM1;TMEM64;HIPK1;SYNJ2BP;RAB11A;IT
29A;XIAP;ZDHHC21;DRP2;PURA;UBN2;SRSF10;MAP3K2;SPRYD7;C1ORF21;TMEM64;SYNJ2BP;GATA

)XJ2;ATP2B4;GTF2H1;DEK;TBX5;SOD2;GATAD2B;HIPK2;HEYL;NFIA;ASXL3;PDCD4;CTNNB1;SSBP2;FI

₹SF10;TRIM44;MAP3K2;CNBP;FOXJ2;ATP2B4;DEK;TBX5;SOD2;HIPK2;HEYL;NFIA;CARM1;CAMK4;PD(

;HSDL1;LRRC40;IL17RD;CBFA2T2;SENP2;CNKSR3;TET3;LCOR;PGAM5;NAA38;LPHN2;ERGIC2;SOS2;
ENP2;SUMF1;PTP4A1;FCHSD2;TERF2IP;CD47;ERGIC2;CRK;PHLPP2;FYTTD1;NAPB;RALA;STAU1;LU
108;PRKG1;HS3ST3B1;GPR37;GRID1;DNMT3A;SORCS1;PAX5;ADRA2B;PAX2;FAM101B;NRG3;SNPH;
SGIP1;SORCS1;PROX1;ADRA2B;ADRA2A;NRG3;RBMS1;SMTNL2;ST6GALNAC3;CHST3;RNF165;PLA
M2;TUBD1;PRICKLE1;OGFRL1;BCLAF1;TMEM65;TMEM203;TNKS2;PABPN1;TMEM68;NRIP1;S1PR1;V

3G;LGI2;TBX5;TTC9;MARCKS;NFIA;TMEM56;KIF26B;FAM171B
A;ENAH;FAM101B;NRG3;SNPH;NOS1AP;NAT8L;CHST3;MYRF;PLAG1;PALM2;NDNF;HOXD12;PAPSS2;
P;COL6A5;SMTNL2;NAT8L;MAPRE2;SLC24A2;HDAC5;PPM1L;PALM2;PRUNE2;PPM1H;NDNF;PPM1E;

;FAM46C;FOXJ2;TMEM64;ATP2B1;SOD2;SYNJ2BP;U2SURP;ADAT2;TTC9;ITCH;ZNF70;CDKN2AIPNL;[illegible]

;TNKS2;SERTAD2;E2F1;HAS2;MAPK1;APBB2;MAP3K2;CSNK1A1;FAM46C;FOXJ2;MTSS1L;TMEM64;A[illegible]

AFAH1B2;DCP2;NAPB;ATL2;XIAP;ZDHHC21;MIEF1;PURA;PGRMC1;SERTAD2;FAM177A1;APBB2;MBNL
C1;SORT1;FAM46C;PCGF3;GABRA3;YLPM1;UBE2G1;C1ORF21;HIPK1;SYNJ2BP;GATAD2B;STXBP5L;T
NK1;KLHL7;CAPZA1;CCDC6;RAB3GAP2;TARDBP;RORB;TM7SF3;CDH6;KIF5C;PAPOLG;MACROD2;IL6
IFIA;SERBP1;CCDC6;TARDBP;SLC28A3;CCDC71L;TMEM167B;POGK;FAM107B;CDH6;EFEMP1;KIF5C;
HC18;TMEM65;UBN2;TMEM68;APBB2;MBNL3;SRSF10;MAP3K2;GABRA1;USP9X;UBE2G1;C1ORF21;T
HC18;TMEM65;UBN2;TMEM68;APBB2;MBNL3;SRSF10;MAP3K2;GABRA1;USP9X;UBE2G1;C1ORF21;T
HC18;TMEM65;UBN2;TMEM68;APBB2;MBNL3;SRSF10;MAP3K2;GABRA1;USP9X;UBE2G1;C1ORF21;T
CH;TMEM56;RLIM;TARDBP;YPEL2;OTUD4;CCDC71L;FAM49A;PCDH11Y;LTN1;KLHL32;MRFAP1;AMOT;
CH;TMEM56;RLIM;TARDBP;YPEL2;OTUD4;CCDC71L;FAM49A;PCDH11Y;LTN1;KLHL32;MRFAP1;AMOT;
D2B;GTF2H5;RAB11A;HIPK2;PDP2;GPAM;TMEM56;KLHL7;SERBP1;RLIM;CTNNB1;TARDBP;FRK;CCD

RK

CD4;RLIM;CTNNB1;SSBP2

;PAFAH1B2;ARHGEF5;PHLPP2;FYTTD1;SF3B3;ROCK1;DCTN2;NUFIP2;PLAG1;TUBD1;ZDHHC21;ZBTE
ZP1;HPGD;ATL2;ZDHHC21;PDHB;PAPSS2;LRP6;PPP2CA;NEURL1B;OGFRL1;NXF1;PGRMC1;MAT2A;T
;MYOD1;NOS1AP;ARHGEF5;CHST3;PALM2;NDNF;PRICKLE1;HOXD12;PPM1E;COL19A1;PAPSS2;ADD
G1;HOXD12;COL19A1;ADD2;EFS;ERBB4;IGF2BP1;S1PR1;HAS2;S1PR3;JAM2;ATOH1;PRRX1;KCNIP2;E
PS54;SMNDC1;TRIM44;TIMMDC1;FOXJ2;PDAP1;PLEKHA3;GTF2H1;ATP2B1;GTF2H5;TTC9;GPAM;VAP

;EFS;IGF2BP1;S1PR1;S1PR3;JAM2;TPBG;B3GAT2;CDC42BPA;TSHR;STXBP5L;SULF2;TTC9;FRAS1;TM
COL19A1;ADD2;GBX2;ERBB4;S1PR1;HAS2;SLC17A6;GPC5;SLIT2;JAM2;ATOH1;PRRX1;LGI2;ATP2B2;T

GPAM;APC;KANSL1;CCDC6;OCIAD1;RLIM;TRIP12;PTPN4;TNRC6B

TP2B1;SOD2;SYNJ2BP;U2SURP;ADAT2;TTC9;ITCH;ZNF70;CDKN2AIPNL;GPAM;CCDC6;OCIAD1;PDCD

_3;SRSF10;MAP3K2;CSNK1A1;FAM46C;PCGF3;UBE2G1;C1ORF21;SYNJ2BP;GATAD2B;STXBP5L;ADA
TC9;XK;PDP2;NFIA;ETNK1;RFWD3;CCDC6;RAB3GAP2;TARDBP;C11ORF57;OTUD4;FAM49A;TMEM16
R;RAB2B;TMOD2;SLC30A4;ARID5B;ADAM19;GPRIN3;MAF;TBL1XR1;PFKFB3;BHLHB9;TNKS;ARL3;RN
PCDHAC2;KPNA1;KCNH1;PCDHAC1;RAB2B;TPM3;TMOD3;ANGEL2;TMOD2;DYRK1A;CNPY1;ARID5B;
MEM64;HIPK1;GATAD2B;HIPK2;PDP2;GPAM;SERBP1;RAB3GAP2;TARDBP;CCDC71L;ANKRD13B;DEN
MEM64;HIPK1;GATAD2B;HIPK2;PDP2;GPAM;SERBP1;RAB3GAP2;TARDBP;CCDC71L;ANKRD13B;DEN
MEM64;HIPK1;GATAD2B;HIPK2;PDP2;GPAM;SERBP1;RAB3GAP2;TARDBP;CCDC71L;ANKRD13B;DEN
CCND3;CDH2;IL6R;PCDHAC2;PCDHAC1;CSNK1G3;CNPY1;ARID5B;PCDHA13;SHISA9;PCDHA12;PCD
CCND3;CDH2;IL6R;PCDHAC2;PCDHAC1;CSNK1G3;CNPY1;ARID5B;PCDHA13;SHISA9;PCDHA12;PCD
C71L;FAM49A;PCDH11Y;TM7SF3;AMOT;PAPOLG;CSNK1G3;TMOD2;SLC30A4;SHISA9;FOXP2;ACAP2;

344;FAM117B;LRP6;TRAK2;CYTH3;GNA13;NXF1;MAT2A;TNKS2;PABPN1;RBBP5;TRA2B;ATXN1L;NRIP1
TNKS2;TRA2B;VPS54;MAPK1;GLTSCR1L;LRIG2;EMC7;SRSF10;SMNDC1;MAP3K2;SEPT10;CSNK1A1;C
2;NEURL1B;RXRA;ERBB4;GPC5;TRIM44;PRRX1;SORT1;FAM46C;B3GAT2;SUSD1;LGI2;FOXJ2;ATP2B2
33GAT2;LGI2;ATP2B2;SORBS3;EPOR;STXBP5L;HEYL;TTC9;FAM167A;ASXL3;CAMK4;FAM171B
A;APC;KANSL1;ETNK1;CAPZA1;OCIAD1;CCNYL1;RAB3GAP2;FAM171B;TRIP12;NUPL1

MEM56;KIF26B;TCEA3;FAM171B
TBX5;SORBS3;TSHR;STXBP5L;TTC9;ASXL3;YPEL2

)4;RLIM;PTPN4;TNRC6B

T2;ITCH;TMEM56;KIF26B;CAPZA1;SERBP1;RLIM;CCDC71L;FAM49A;PCDH11Y;AMOT;CDH6;CCND3;EF
7B;PCDH11Y;KLHL32;ZBTB20;FAM107B;AMOT;CDH6;PAPOLG;MACROD2;SH3GL2;PCDHAC2;KPNA1;II
F180;NPR3;GLIS3;FOXO1;FBLN5;TMEM245;ARHGAP20;RAP1A;ATXN7;CBX5;SREK1IP1;MARCH7;ESR
ZBTB34;PCDHA13;SHISA9;PCDHA12;PCDHA11;FOXP2;PCDHA10;ACAP2;MAF;TBL1XR1;ADAM12;CMI
ND5B;POGK;FAM107B;AMOT;RIMS3;CDH2;FNTB;IL6R;PCDHAC2;KPNA1;KCNH1;PCDHAC1;TMOD3;TI
ND5B;POGK;FAM107B;AMOT;RIMS3;CDH2;FNTB;IL6R;PCDHAC2;KPNA1;KCNH1;PCDHAC1;TMOD3;TI
ND5B;POGK;FAM107B;AMOT;RIMS3;CDH2;FNTB;IL6R;PCDHAC2;KPNA1;KCNH1;PCDHAC1;TMOD3;TI
HA11;FOXP2;PCDHA10;ACAP2;TBC1D25;ANKRD40;GLYR1;SHC4;BHLHB9;INSIG2;RHOBTB3;CTIF;ZM
HA11;FOXP2;PCDHA10;ACAP2;TBC1D25;ANKRD40;GLYR1;SHC4;BHLHB9;INSIG2;RHOBTB3;CTIF;ZM
MAF;TBL1XR1;FAM185A;ADAM12;WDFY3;KANK4;BHLHB9;ABHD4;UBA6;RHOBTB3;FBLN5;TMEM245;A

1;FAM63B;APBB2;SMNDC1;TRIM44;MAP3K2;CRBN;CSNK1A1;TXNL1;PCGF3;FOXJ2;GATC;PDAP1;PLE
CNBP;YLPM1;PDAP1;GTF2H1;PTPN11;TMEM64;HIPK1;CDC42BPA;GATAD2B;HIPK2;XK;GPAM;VAPA;PI
2;HEYL;TMEM56;KIF26B;ASXL3;CAMK4

FEMP1;MACROD2;PCDHAC2;KPNA1;PCDHAC1;CSNK1G3;TMOD2;SLC30A4;PCDHA13;POU3F1;PCDH
L13RA1;PCDHAC1;TPM3;TMOD2;SLC30A4;DIO2;DYRK1A;ALG14;CNPY1;ARID5B;PCDHA13;PCDHA12
R1;FBXO30;AZIN1;ABHD15;CNOT6;SP1;SP4;CAPRIN1;JMY;SERINC3;UBE2K;NFE2L1;DNAL1;LRP12;BM
PK1;WDFY3;DENND6A;KANK4;TBC1D19;BHLHB9;FAM114A1;RNF180;ABHD2;GLIS3;FBXO40;ATXN7;ZI
MOD2;DIO2;ARID5B;ZBTB34;PCDHA13;SHISA9;PCDHA12;PCDHA11;FOXP2;PCDHA10;GPRIN3;ACAP2
MOD2;DIO2;ARID5B;ZBTB34;PCDHA13;SHISA9;PCDHA12;PCDHA11;FOXP2;PCDHA10;GPRIN3;ACAP2
MOD2;DIO2;ARID5B;ZBTB34;PCDHA13;SHISA9;PCDHA12;PCDHA11;FOXP2;PCDHA10;GPRIN3;ACAP2
AT3;SRGAP2;MYH10;SMAD9;KLHL23;TRAPPC8;REEP1;CAPRIN1;NABP1;DNAL1;CREBZF;TNFAIP8;BM
AT3;SRGAP2;MYH10;SMAD9;KLHL23;TRAPPC8;REEP1;CAPRIN1;NABP1;DNAL1;CREBZF;TNFAIP8;BM
ARHGAP20;PLXNA2;MARK1;TFAP2B;CBX5;NEK7;ZBTB10;SLC4A10;SREK1IP1;HELZ;BFAR;SELE;AZIN1

:KHA3;GTF2H1;PTPN11;DEK;SYNJ2BP;CDC42BPA;GATAD2B;LETM1;NECAP2;ITCH;VAPA;ETNK1;CARI
THD1;TMEM56;ETNK1;CAPZA1;SERBP1;OCIAD1;CCNYL1;RAB3GAP2;NUPL1;LNX2

A12;PCDHA11;PCDHA10;ADAM19;ADAM12;WDFY3;SCG3;BHLHB9;UBA6;TNKS;CUL3;NPR3;TMEM245
;PCDHA11;PCDHA10;ACAP2;MAF;STIM2;TBL1XR1;CMPK1;WDFY3;PSMD11;UBA6;RNF180;ABHD2;GL
MPR1A;ONECUT2;SIX1;CTDSPL2;PPP1R9A;AFF4;GRM5;ADAMTS2;SPRED1;TRPS1;PSD3;CEP170;CN
MAT3;ACBD5;CBX5;ZBTB14;SRD5A1;ZBTB10;SREK1IP1;INHBA;HOOK3;CNOT6;OSTC;CAPRIN1;JMY;
2;MAF;ELMO1;WDFY3;NPTXR;RAF1;KANK4;DLX1;YTHDC1;TNKS;ARL3;NPR3;GLIS3;TRHDE;ARHGAP
2;MAF;ELMO1;WDFY3;NPTXR;RAF1;KANK4;DLX1;YTHDC1;TNKS;ARL3;NPR3;GLIS3;TRHDE;ARHGAP
2;MAF;ELMO1;WDFY3;NPTXR;RAF1;KANK4;DLX1;YTHDC1;TNKS;ARL3;NPR3;GLIS3;TRHDE;ARHGAP
MPR2;ANKRD33B;ONECUT2;CELF1;SIX1;ELAVL2;LMOD3;RBM3;KIAA1549;SPRED1;TRPS1;PSD3;CCD
MPR2;ANKRD33B;ONECUT2;CELF1;SIX1;ELAVL2;LMOD3;RBM3;KIAA1549;SPRED1;TRPS1;PSD3;CCD
1;CNOT6;ZFP62;RCAN2;JMY;DNAL1;LRP12;CREBZF;RBM28;USP37;BMPR2;ONECUT2;CELF1;CTDSP

M1;CAPZA1;SERBP1;TRIP12

5;IP6K1;SRGAP2;ZC3H14;ACBD5;NEK7;GFPT1;ZBTB10;SLC4A10;SREK1IP1;KLHL23;HELZ;HOOK3;FB
IS3;CREBL2;TRHDE;FBXO40;ATXN7;ZMAT3;CLSPN;ZC3H14;TFAP2B;NEK7;ZBTB10;SLC4A10;SREK1I
PPD1;CCDC50;KLF10;USP47;NAA30;GRID1;SGIP1;ZFP91;TGFBR3;CREB1;RBL1;LCOR;RBMS1;PDE5
CDK1;MXD1;UBE2K;NFE2L1;PLEKHM3;BMPR1A;CREBZF;BMPR2;ONECUT2;CELF1;SNAP23;PPP1R9A
20;ATXN7;G3BP1;PLXNA2;MARK1;CPSF7;GFPT1;ZBTB10;BTBD9;ESR1;FBXO30;SP4;CAPRIN1;JMY;M
20;ATXN7;G3BP1;PLXNA2;MARK1;CPSF7;GFPT1;ZBTB10;BTBD9;ESR1;FBXO30;SP4;CAPRIN1;JMY;M
20;ATXN7;G3BP1;PLXNA2;MARK1;CPSF7;GFPT1;ZBTB10;BTBD9;ESR1;FBXO30;SP4;CAPRIN1;JMY;M
C50;USP47;PLA2G12A;HAUS3;GPCPD1;CREB1;RRM2B;DCX;LCOR;TERF2IP;PDE5A;SOS2;SLC24A2;
C50;USP47;PLA2G12A;HAUS3;GPCPD1;CREB1;RRM2B;DCX;LCOR;TERF2IP;PDE5A;SOS2;SLC24A2;
L2;AFF4;ELAVL2;DTWD1;SPRED1;TRPS1;PGM3;CEP170;UNKL;CCDC50;DGCR2;HSDL1;SGIP1;VPS13

XO30;AZIN1;SMAD7;REEP1;SP1;CAPRIN1;JMY;MXD1;UBE2K;CREBZF;BMPR2;ONECUT2;CTDSPL2;PI
P1;KLHL23;HELZ;HOOK3;MARCH7;BTBD9;ESR1;SELE;SP1;ZFP62;SP4;RCAN2;CAPRIN1;JMY;LSM14E
A;NAT8L;SLC24A2;RALA;USP15;PPM1L;ROCK1;LUZP1;NUFIP2;SRSF1;NDNF;MAT2B;LRP8;GNA13;ERI
A;RND3;ADAMTS5;ARL5B;SPRED1;EEF2K;MAN1A2;TRPS1;PSD3;SCN5A;UNKL;PHACTR4;HS3ST3B1;U
XD1;UBE2K;DNAL1;PLEKHM3;CREBZF;ONECUT2;CELF1;PPP1R9A;ELAVL2;MB;MAN1A2;TRPS1;PSD3
XD1;UBE2K;DNAL1;PLEKHM3;CREBZF;ONECUT2;CELF1;PPP1R9A;ELAVL2;MB;MAN1A2;TRPS1;PSD3
XD1;UBE2K;DNAL1;PLEKHM3;CREBZF;ONECUT2;CELF1;PPP1R9A;ELAVL2;MB;MAN1A2;TRPS1;PSD3
NUFIP2;PLAG1;SEMA3A;SRSF1;ZBTB44;LRP8;LRP6;CYTH3;AP3M2;GNA13;ATXN1L;NRIP1;MAPK1;S1
NUFIP2;PLAG1;SEMA3A;SRSF1;ZBTB44;LRP8;LRP6;CYTH3;AP3M2;GNA13;ATXN1L;NRIP1;MAPK1;S1
3C;ZFP91;SMC1A;CREB1;NRG3;RRM2B;DCX;LCOR;SOS2;PRTFDC1;SLC24A2;CRP;RALA;NUFIP2;ZBT

PP1R9A;RND3;AFF1;RBM3;SDR16C5;SPRED1;TRPS1;PSD3;TMEM108;CEP170;METTL16;UNKL;CNPP
3;SERINC3;NFE2L1;CREBZF;BMPR2;ONECUT2;CELF3;PDE3B;CDC73;RBM3;ADAMTS5;GRM5;SPRED
BB4;ATXN1L;NRIP1;MAPK1;S100PBP;LGI2;PDAP1;ATP2B2;ATP2B1;SORL1;MARCKS;FAM167A;APC;KA
UTP15;TSFM;USP47;NAA30;TTC33;MSL2;ZFP91;SENP2;FAM84A;DCX;LCOR;RBMS1;NAA38;PDE5A;SL
3;PGM3;TMEM108;METTL16;CCDC50;KLF10;MAPK1IP1L;ARHGEF10;TSFM;GRID1;SGIP1;MSL2;ZFP91
3;PGM3;TMEM108;METTL16;CCDC50;KLF10;MAPK1IP1L;ARHGEF10;TSFM;GRID1;SGIP1;MSL2;ZFP91
3;PGM3;TMEM108;METTL16;CCDC50;KLF10;MAPK1IP1L;ARHGEF10;TSFM;GRID1;SGIP1;MSL2;ZFP91
00PBP;IGBP1;ATP2B1;CLCN5;MYO1C;ABHD17B;APC;VAPB;EIF3J;PDCD4;ASB7
00PBP;IGBP1;ATP2B1;CLCN5;MYO1C;ABHD17B;APC;VAPB;EIF3J;PDCD4;ASB7
B44;MAT2B;ADD3;LRP8;PPM1E;TNKS2;ERBB4;NRIP1;VPS54;EIF4E;GALNT7;SEPT10;PRRX1;CNBP;TF

'D1;CCDC50;KLF10;VPS13C;DNMT3A;SMC
1;TRPS1;PGM3;CEP170;ARL5A;MAPK1IP1
ANSL1;ASB7;LNX2
LC24A2;USP15;PPM1L;LUZP1;PLAG1;SRS
I;SMC1A;TGFBR3;RRM2B;SNPH;DCX;LCO
I;SMC1A;TGFBR3;RRM2B;SNPH;DCX;LCO
I;SMC1A;TGFBR3;RRM2B;SNPH;DCX;LCO

PBG;ATP2B4;PTPN11;CDC42BPA;MARCKS
