# Supplementary material for: Circulating miR-330-3p in Late Pregnancy is Associated with Pregnancy Outcomes Among Lean Women with GDM
Source: Sci Rep. 2020 Jan 22;10:908. doi: 10.1038/s41598-020-57838-6 (PMC6976655; doi:10.1038/s41598-020-57838-6)
Supplement: Supplementary file 1 — Supplementary data. [file 41598_2020_57838_MOESM1_ESM.zip › Supplimentary File_EnrichR_Analysis GO_Biological_Process_2018.pdf]

| Term |
| --- |
| negative regulation of transcription, DNA-templated (GO:0045892) |
| positive regulation of transcription, DNA-templated (GO:0045893) |
| positive regulation of nucleic acid-templated transcription (GO:1903508) |
| regulation of transcription from RNA polymerase II promoter (GO:0006357) |
| nervous system development (GO:0007399) |
| generation of neurons (GO:0048699) |
| positive regulation of transcription from RNA polymerase II promoter (GO:0045944) |
| regulation of transcription, DNA-templated (GO:0006355) |
| positive regulation of gene expression (GO:0010628) |
| negative regulation of gene expression (GO:0010629) |
| negative regulation of cellular macromolecule biosynthetic process (GO:2000113) |
| negative regulation of nucleic acid-templated transcription (GO:1903507) |
| regulation of angiogenesis (GO:0045765) |
| negative regulation of transcription from RNA polymerase II promoter (GO:0000122) |
| negative regulation of cellular response to growth factor stimulus (GO:0090288) |
| regulation of BMP signaling pathway (GO:0030510) |
| neuron differentiation (GO:0030182) |
| regulation of mRNA splicing, via spliceosome (GO:0048024) |
| neuron development (GO:0048666) |
| protein acylation (GO:0043543) |
| transcription from RNA polymerase II promoter (GO:0006366) |
| protein polyubiquitination (GO:0000209) |
| neuron migration (GO:0001764) |
| modification by symbiont of host morphology or physiology (GO:0044003) |
| modification-dependent protein catabolic process (GO:0019941) |
| ubiquitin-dependent protein catabolic process (GO:0006511) |
| transcription, DNA-templated (GO:0006351) |
| positive regulation of cell differentiation (GO:0045597) |
| regulation of myeloid cell differentiation (GO:0045637) |
| regulation of RNA splicing (GO:0043484) |
| Wnt signaling pathway (GO:0016055) |
| cellular response to organic substance (GO:0071310) |
| ventricular septum development (GO:0003281) |
| cellular response to BMP stimulus (GO:0071773) |
| beta-catenin destruction complex disassembly (GO:1904886) |
| negative regulation of cell differentiation (GO:0045596) |
| regulation of cell cycle (GO:0051726) |
| positive regulation of fat cell differentiation (GO:0045600) |
| central nervous system development (GO:0007417) |
| cardiac ventricle development (GO:0003231) |
| sympathetic nervous system development (GO:0048485) |
| cardiac epithelial to mesenchymal transition (GO:0060317) |
| protein ubiquitination (GO:0016567) |
| ganglion development (GO:0061548) |
| Rap protein signal transduction (GO:0032486) |
| motor neuron axon guidance (GO:0008045) |
| sympathetic ganglion development (GO:0061549) |
| spliceosomal complex assembly (GO:0000245) |
| regulation of fat cell differentiation (GO:0045598) |
| proteasome-mediated ubiquitin-dependent protein catabolic process (GO:0043161) |
| negative regulation of B cell activation (GO:0050869) |
| proteasomal protein catabolic process (GO:0010498) |

positive regulation of proteasomal protein catabolic process (GO:1901800)
protein phosphorylation (GO:0006468)
axon guidance (GO:0007411)
protein acetylation (GO:0006473)
regulation of Wnt signaling pathway (GO:0030111)
regulation of canonical Wnt signaling pathway (GO:0060828)
positive regulation of neuron differentiation (GO:0045666)
modulation by virus of host morphology or physiology (GO:0019048)
negative regulation of blood vessel morphogenesis (GO:2000181)
oxygen homeostasis (GO:0032364)
dosage compensation by inactivation of X chromosome (GO:0009048)
monoubiquitinated protein deubiquitination (GO:0035520)
regulation of antigen receptor-mediated signaling pathway (GO:0050854)
negative regulation of fibroblast growth factor receptor signaling pathway (GO:0040037)
regulation of nitric-oxide synthase activity (GO:0050999)
positive regulation of Wnt signaling pathway (GO:0030177)
regulation of cellular component movement (GO:0051270)
regulation of protein catabolic process (GO:0042176)
negative regulation of MAPK cascade (GO:0043409)
regulation of macromolecule metabolic process (GO:0060255)
positive regulation of vasculature development (GO:1904018)
negative regulation of angiogenesis (GO:0016525)
positive regulation of protein catabolic process (GO:0045732)
pathway-restricted SMAD protein phosphorylation (GO:0060389)
ephrin receptor signaling pathway (GO:0048013)
ventricular septum morphogenesis (GO:0060412)
synaptic transmission, glutamatergic (GO:0035249)
gas homeostasis (GO:0033483)
positive regulation of urine volume (GO:0035810)
positive regulation of dendritic spine morphogenesis (GO:0061003)
regulation of cell differentiation (GO:0045595)
positive regulation of protein metabolic process (GO:0051247)
actin cytoskeleton reorganization (GO:0031532)
regulation of intracellular estrogen receptor signaling pathway (GO:0033146)
positive regulation of catabolic process (GO:0009896)
regulation of telomere maintenance via telomerase (GO:0032210)
kidney development (GO:0001822)
regulation of chondrocyte differentiation (GO:0032330)
RNA processing (GO:0006396)
positive regulation of canonical Wnt signaling pathway (GO:0090263)
myotube differentiation (GO:0014902)
autonomic nervous system development (GO:0048483)
N-terminal protein amino acid acetylation (GO:0006474)
regulation of cell junction assembly (GO:1901888)
regulation of cartilage development (GO:0061035)
protein localization to chromosome, telomeric region (GO:0070198)
negative regulation of BMP signaling pathway (GO:0030514)
atrial cardiac muscle cell to AV node cell signaling (GO:0086026)
regulation of aspartic-type endopeptidase activity involved in amyloid precursor protein catabolic process (GO:1
peptidyl-lysine acetylation (GO:0018394)
regulation of stress-activated protein kinase signaling cascade (GO:0070302)
barbed-end actin filament capping (GO:0051016)
negative regulation of neurogenesis (GO:0050768)

mRNA stabilization (GO:0048255)
positive regulation of angiogenesis (GO:0045766)
Notch signaling pathway (GO:0007219)
protein deubiquitination (GO:0016579)
posttranscriptional regulation of gene expression (GO:0010608)
positive regulation of dendrite morphogenesis (GO:0050775)
regulation of bicellular tight junction assembly (GO:2000810)
positive regulation of nitric-oxide synthase activity (GO:0051000)
positive regulation of G2/M transition of mitotic cell cycle (GO:0010971)
negative regulation of cell proliferation (GO:0008285)
regulation of cyclin-dependent protein serine/threonine kinase activity (GO:0000079)
transmembrane receptor protein serine/threonine kinase signaling pathway (GO:0007178)
mRNA splice site selection (GO:0006376)
negative regulation of DNA binding (GO:0043392)
transmembrane receptor protein tyrosine kinase signaling pathway (GO:0007169)
cyclic nucleotide catabolic process (GO:0009214)
protein modification by small protein conjugation (GO:0032446)
protein modification by small protein removal (GO:0070646)
BMP signaling pathway (GO:0030509)
erythrocyte differentiation (GO:0030218)
glial cell differentiation (GO:0010001)
negative regulation of epithelial to mesenchymal transition (GO:0010719)
regulation of vascular smooth muscle cell proliferation (GO:1904705)
negative regulation of telomere maintenance (GO:0032205)
N-glycan processing (GO:0006491)
positive regulation of telomere capping (GO:1904355)
positive regulation of cartilage development (GO:0061036)
positive regulation of transcription of Notch receptor target (GO:0007221)
positive regulation of cell cycle G2/M phase transition (GO:1902751)
regulation of DNA metabolic process (GO:0051052)
positive regulation of cell cycle (GO:0045787)
positive regulation of cell morphogenesis involved in differentiation (GO:0010770)
positive regulation of proteasomal ubiquitin-dependent protein catabolic process (GO:0032436)
Wnt signaling pathway, calcium modulating pathway (GO:0007223)
histone H4 acetylation (GO:0043967)
positive regulation of cyclin-dependent protein kinase activity (GO:1904031)
negative regulation of telomere maintenance via telomere lengthening (GO:1904357)
RNA splicing, via transesterification reactions with bulged adenosine as nucleophile (GO:0000377)
negative regulation of protein phosphorylation (GO:0001933)
renal system development (GO:0072001)
regulation of neuron differentiation (GO:0045664)
regulation of dephosphorylation (GO:0035303)
regulation of cardiac muscle cell proliferation (GO:0060043)
regulation of cellular response to transforming growth factor beta stimulus (GO:1903844)
positive regulation of neuron projection development (GO:0010976)
negative regulation of cell motility (GO:2000146)
negative regulation of cellular process (GO:0048523)
Ras protein signal transduction (GO:0007265)
protein K48-linked ubiquitination (GO:0070936)
mRNA processing (GO:0006397)
positive regulation of smooth muscle cell proliferation (GO:0048661)
positive regulation of stress fiber assembly (GO:0051496)
dorsal/ventral axis specification (GO:0009950)

protein K11-linked deubiquitination (GO:0035871)
regulation of urine volume (GO:0035809)
positive regulation of reproductive process (GO:2000243)
actin filament capping (GO:0051693)
negative regulation of embryonic development (GO:0045992)
protein prenylation (GO:0018342)
intracellular cholesterol transport (GO:0032367)
positive regulation of epidermal growth factor-activated receptor activity (GO:0045741)
regulation of gene expression (GO:0010468)
protein localization to chromosome (GO:0034502)
cellular response to growth factor stimulus (GO:0071363)
regulation of cell migration (GO:0030334)
canonical Wnt signaling pathway (GO:0060070)
protein heterooligomerization (GO:0051291)
negative regulation of MAP kinase activity (GO:0043407)
posttranscriptional gene silencing by RNA (GO:0035194)
purine ribonucleotide catabolic process (GO:0009154)
calcium ion transport into cytosol (GO:0060402)
regulation of gene expression, epigenetic (GO:0040029)
regulation of lipid metabolic process (GO:0019216)
regulation of mRNA processing (GO:0050684)
regulation of transmembrane receptor protein serine/threonine kinase signaling pathway (GO:0090092)
bicellular tight junction assembly (GO:0070830)
neuron projection extension (GO:1990138)
regulation of alternative mRNA splicing, via spliceosome (GO:0000381)
negative regulation of histone acetylation (GO:0035067)
photoreceptor cell development (GO:0042461)
negative regulation of neuron apoptotic process (GO:0043524)
mRNA splicing, via spliceosome (GO:0000398)
hemopoiesis (GO:0030097)
regulation of neuron apoptotic process (GO:0043523)
striated muscle cell development (GO:0055002)
organelle disassembly (GO:1903008)
regulation of fibroblast growth factor receptor signaling pathway (GO:0040036)
cellular response to estradiol stimulus (GO:0071392)
phosphorylation (GO:0016310)
negative regulation of neuron differentiation (GO:0045665)
negative regulation of transmembrane receptor protein serine/threonine kinase signaling pathway (GO:0090101
plasma membrane bounded cell projection organization (GO:0120036)
positive regulation of protein serine/threonine kinase activity (GO:0071902)
beta-catenin-TCF complex assembly (GO:1904837)
protein palmitoylation (GO:0018345)
artery morphogenesis (GO:0048844)
response to estradiol (GO:0032355)
negative regulation of stress-activated MAPK cascade (GO:0032873)
positive regulation of cyclin-dependent protein serine/threonine kinase activity (GO:0045737)
negative regulation of cellular response to transforming growth factor beta stimulus (GO:1903845)
G1/S transition of mitotic cell cycle (GO:0000082)
positive regulation of BMP signaling pathway (GO:0030513)
steroid hormone mediated signaling pathway (GO:0043401)
limb morphogenesis (GO:0035108)
long-term synaptic potentiation (GO:0060291)
regulation of neuron projection development (GO:0010975)

oligodendrocyte development (GO:0014003)
regulation of bone remodeling (GO:0046850)
positive regulation of chondrocyte differentiation (GO:0032332)
regulation of transforming growth factor beta receptor signaling pathway (GO:0017015)
positive regulation of cellular catabolic process (GO:0031331)
RNA splicing (GO:0008380)
actin filament organization (GO:0007015)
positive regulation of viral genome replication (GO:0045070)
protein destabilization (GO:0031648)
positive regulation of autophagy (GO:0010508)
ERBB signaling pathway (GO:0038127)
regulation of hydrolase activity (GO:0051336)
cellular response to nerve growth factor stimulus (GO:1990090)
NLS-bearing protein import into nucleus (GO:0006607)
negative regulation of protein polymerization (GO:0032272)
outflow tract septum morphogenesis (GO:0003148)
negative regulation of ERBB signaling pathway (GO:1901185)
positive regulation of telomere maintenance via telomerase (GO:0032212)
atrial cardiac muscle cell action potential (GO:0086014)
embryonic appendage morphogenesis (GO:0035113)
protein neddylation (GO:0045116)
negative regulation of hormone secretion (GO:0046888)
semaphorin-plexin signaling pathway involved in neuron projection guidance (GO:1902285)
ribonucleoprotein complex disassembly (GO:0032988)
positive regulation of sodium ion transport (GO:0010765)
negative regulation of mRNA splicing, via spliceosome (GO:0048025)
positive regulation of skeletal muscle tissue development (GO:0048643)
regulation of axon guidance (GO:1902667)
positive regulation of transmembrane receptor protein serine/threonine kinase signaling pathway (GO:0090100)
regulation of smooth muscle cell proliferation (GO:0048660)
positive regulation of actin filament bundle assembly (GO:0032233)
myeloid cell differentiation (GO:0030099)
positive regulation of neurogenesis (GO:0050769)
regulation of telomere capping (GO:1904353)
protein K48-linked deubiquitination (GO:0071108)
regulation of dendritic spine morphogenesis (GO:0061001)
protein localization to Golgi apparatus (GO:0034067)
positive regulation of monooxygenase activity (GO:0032770)
positive regulation of protein ubiquitination (GO:0031398)
cellular glucose homeostasis (GO:0001678)
positive regulation of organelle organization (GO:0010638)
stem cell differentiation (GO:0048863)
protein ubiquitination involved in ubiquitin-dependent protein catabolic process (GO:0042787)
mRNA metabolic process (GO:0016071)
post-Golgi vesicle-mediated transport (GO:0006892)
positive regulation of muscle cell differentiation (GO:0051149)
positive regulation of telomere maintenance via telomere lengthening (GO:1904358)
endoplasmic reticulum organization (GO:0007029)
embryonic limb morphogenesis (GO:0030326)
positive regulation of cardiac muscle cell proliferation (GO:0060045)
positive regulation of neural precursor cell proliferation (GO:2000179)
regulation of calcineurin-NFAT signaling cascade (GO:0070884)
negative regulation of hemopoiesis (GO:1903707)

oligodendrocyte differentiation (GO:0048709)
regulation of defense response to virus (GO:0050688)
positive regulation of vascular smooth muscle cell proliferation (GO:1904707)
negative regulation of smooth muscle cell migration (GO:0014912)
histone H4-K16 acetylation (GO:0043984)
gliogenesis (GO:0042063)
pore complex assembly (GO:0046931)
endoplasmic reticulum tubular network organization (GO:0071786)
DNA damage response, signal transduction by p53 class mediator resulting in transcription of p21 class mediatc
protein complex disassembly (GO:0043241)
positive regulation of ion transport (GO:0043270)
regulation of fibroblast proliferation (GO:0048145)
DNA-templated transcription, initiation (GO:0006352)
positive regulation of protein kinase activity (GO:0045860)
regulation of mRNA stability (GO:0043488)
positive regulation of skeletal muscle fiber development (GO:0048743)
positive regulation of aspartic-type peptidase activity (GO:1905247)
peripheral nervous system neuron development (GO:0048935)
ventricular cardiac muscle cell development (GO:0055015)
negative regulation of lymphocyte differentiation (GO:0045620)
dendrite extension (GO:0097484)
regulation of atrial cardiac muscle cell membrane depolarization (GO:0060371)
negative regulation of cAMP-mediated signaling (GO:0043951)
photoreceptor cell differentiation (GO:0046530)
regulation of chemokine-mediated signaling pathway (GO:0070099)
definitive hemopoiesis (GO:0060216)
response to heparin (GO:0071503)
regulation of transforming growth factor beta2 production (GO:0032909)
ventricular compact myocardium morphogenesis (GO:0003223)
negative regulation of systemic arterial blood pressure (GO:0003085)
regulation of respiratory system process (GO:0044065)
regulation of cardiac muscle hypertrophy in response to stress (GO:1903242)
regulation of inositol phosphate biosynthetic process (GO:0010919)
enteric nervous system development (GO:0048484)
positive regulation of trophoblast cell migration (GO:1901165)
collecting duct development (GO:0072044)
negative regulation of smooth muscle cell differentiation (GO:0051151)
chondrocyte development (GO:0002063)
positive regulation of fibroblast apoptotic process (GO:2000271)
positive regulation of aspartic-type endopeptidase activity involved in amyloid precursor protein catabolic proces
negative regulation of catecholamine secretion (GO:0033604)
retinal ganglion cell axon guidance (GO:0031290)
cellular response to transforming growth factor beta stimulus (GO:0071560)
regulation of sodium ion transport (GO:0002028)
positive regulation of NIK/NF-kappaB signaling (GO:1901224)
apical junction assembly (GO:0043297)
protein N-linked glycosylation (GO:0006487)
regulation of stress fiber assembly (GO:0051492)
protein complex assembly (GO:0006461)
regulation of cell cycle G1/S phase transition (GO:1902806)
positive regulation of protein phosphorylation (GO:0001934)
protein glycosylation (GO:0006486)
histone H4-K8 acetylation (GO:0043982)

negative regulation of insulin secretion (GO:0046676)
nitric oxide mediated signal transduction (GO:0007263)
DNA damage response, signal transduction resulting in transcription (GO:0042772)
regulation of mitochondrial translation (GO:0070129)
negative regulation of mRNA processing (GO:0050686)
histone H4-K5 acetylation (GO:0043981)
atrioventricular valve morphogenesis (GO:0003181)
negative regulation of peptidyl-threonine phosphorylation (GO:0010801)
septin ring assembly (GO:0000921)
regulation of B cell differentiation (GO:0045577)
transcription initiation from RNA polymerase II promoter (GO:0006367)
glycoprotein metabolic process (GO:0009100)
chromatin remodeling (GO:0006338)
regulation of proteasomal ubiquitin-dependent protein catabolic process (GO:0032434)
mRNA catabolic process (GO:0006402)
axonal transport (GO:0098930)
negative regulation of phosphorylation (GO:0042326)
axonogenesis (GO:0007409)
negative regulation of gene expression, epigenetic (GO:0045814)
RNA splicing, via transesterification reactions (GO:0000375)
regulation of protein kinase activity (GO:0045859)
regulation of megakaryocyte differentiation (GO:0045652)
epithelial to mesenchymal transition (GO:0001837)
protein monoubiquitination (GO:0006513)
protein K63-linked ubiquitination (GO:0070534)
regulation of peptidyl-threonine phosphorylation (GO:0010799)
mRNA transport (GO:0051028)
nucleic acid-templated transcription (GO:0097659)
RNA export from nucleus (GO:0006405)
positive regulation of cell projection organization (GO:0031346)
negative regulation of protein modification process (GO:0031400)
leukocyte adhesion to vascular endothelial cell (GO:0061756)
heme biosynthetic process (GO:0006783)
positive regulation of myotube differentiation (GO:0010831)
aorta morphogenesis (GO:0035909)
positive regulation of DNA-templated transcription, initiation (GO:2000144)
positive regulation of cardiac muscle tissue growth (GO:0055023)
leukocyte tethering or rolling (GO:0050901)
positive regulation of mRNA processing (GO:0050685)
adherens junction assembly (GO:0034333)
positive regulation of transmembrane transport (GO:0034764)
mitral valve development (GO:0003174)
negative regulation of stress-activated protein kinase signaling cascade (GO:0070303)
lymphatic endothelial cell differentiation (GO:0060836)
central nervous system neuron axonogenesis (GO:0021955)
retinoic acid receptor signaling pathway (GO:0048384)
vascular endothelial growth factor signaling pathway (GO:0038084)
peroxisome proliferator activated receptor signaling pathway (GO:0035357)
positive regulation of neuroblast proliferation (GO:0002052)
positive regulation of cell migration by vascular endothelial growth factor signaling pathway (GO:0038089)
regulation of norepinephrine secretion (GO:0014061)
pharyngeal arch artery morphogenesis (GO:0061626)
forebrain neuron differentiation (GO:0021879)

SA node cell action potential (GO:0086015)
regulation of protein deubiquitination (GO:0090085)
cellular response to platelet-derived growth factor stimulus (GO:0036120)
regulation of skeletal muscle fiber development (GO:0048742)
myoblast fusion (GO:0007520)
cAMP catabolic process (GO:0006198)
venous blood vessel development (GO:0060841)
nuclear body organization (GO:0030575)
mRNA export from nucleus (GO:0006406)
regulation of transcription involved in G1/S transition of mitotic cell cycle (GO:0000083)
cytoskeleton-dependent intracellular transport (GO:0030705)
regulation of ossification (GO:0030278)
negative regulation of neuron projection development (GO:0010977)
peptidyl-tyrosine modification (GO:0018212)
regulation of exocytosis (GO:0017157)
negative regulation of cell migration (GO:0030336)
neuron projection development (GO:0031175)
positive regulation of protein modification by small protein conjugation or removal (GO:1903322)
regulation of RNA metabolic process (GO:0051252)
cell-cell junction assembly (GO:0007043)
cellular response to glucose stimulus (GO:0071333)
regulation of calcium ion transmembrane transporter activity (GO:1901019)
regulation of intracellular steroid hormone receptor signaling pathway (GO:0033143)
negative regulation of immune effector process (GO:0002698)
regulation of membrane depolarization (GO:0003254)
sialylation (GO:0097503)
positive regulation of stem cell proliferation (GO:2000648)
3'-UTR-mediated mRNA stabilization (GO:0070935)
embryo development ending in birth or egg hatching (GO:0009792)
regulation of synaptic vesicle exocytosis (GO:2000300)
regulation of B cell receptor signaling pathway (GO:0050855)
septin ring organization (GO:0031106)
N-terminal protein amino acid modification (GO:0031365)
positive regulation of cell-substrate adhesion (GO:0010811)
gene expression (GO:0010467)
intracellular steroid hormone receptor signaling pathway (GO:0030518)
negative regulation of epithelial cell proliferation (GO:0050680)
regulation of protein serine/threonine kinase activity (GO:0071900)
regulation of cell motility (GO:2000145)
regulation of primary metabolic process (GO:0080090)
negative regulation of epidermal growth factor receptor signaling pathway (GO:0042059)
ameboidal-type cell migration (GO:0001667)
positive regulation of mitotic cell cycle phase transition (GO:1901992)
androgen receptor signaling pathway (GO:0030521)
negative regulation of ERK1 and ERK2 cascade (GO:0070373)
negative regulation of catabolic process (GO:0009895)
cellular protein modification process (GO:0006464)
negative regulation of intracellular steroid hormone receptor signaling pathway (GO:0033144)
negative regulation of homeostatic process (GO:0032845)
chondrocyte differentiation (GO:0002062)
positive regulation of protein tyrosine kinase activity (GO:0061098)
regulation of NIK/NF-kappaB signaling (GO:1901222)
negative regulation of JNK cascade (GO:0046329)

cardiac muscle cell action potential involved in contraction (GO:0086002)
neural crest cell migration (GO:0001755)
protein K63-linked deubiquitination (GO:0070536)
primary neural tube formation (GO:0014020)
cellular response to nitric oxide (GO:0071732)
regulation of fibroblast apoptotic process (GO:2000269)
microvillus assembly (GO:0030033)
regulation of cytoplasmic transport (GO:1903649)
plasma membrane to endosome transport (GO:0048227)
negative regulation of calcium ion transmembrane transport (GO:1903170)
regulation of toll-like receptor 2 signaling pathway (GO:0034135)
negative regulation of bone remodeling (GO:0046851)
mammary gland epithelial cell differentiation (GO:0060644)
ribosome disassembly (GO:0032790)
kidney mesenchyme development (GO:0072074)
regulation of vascular associated smooth muscle cell migration (GO:1904752)
neuron projection extension involved in neuron projection guidance (GO:1902284)
histone H2A-K119 monoubiquitination (GO:0036353)
regulation of calcium ion transmembrane transport via high voltage-gated calcium channel (GO:1902514)
microvillus organization (GO:0032528)
coronary vasculature morphogenesis (GO:0060977)
regulation of protein deacetylation (GO:0090311)
negative regulation of T-helper cell differentiation (GO:0045623)
negative regulation of execution phase of apoptosis (GO:1900118)
axon extension involved in axon guidance (GO:0048846)
positive regulation of multicellular organismal process (GO:0051240)
negative regulation of RNA splicing (GO:0033119)
heart trabecula morphogenesis (GO:0061384)
regulation of cellular response to growth factor stimulus (GO:0090287)
regulation of smooth muscle cell migration (GO:0014910)
mRNA destabilization (GO:0061157)
porphyrin-containing compound biosynthetic process (GO:0006779)
cardiac muscle cell contraction (GO:0086003)
mesenchymal cell differentiation (GO:0048762)
regulation of calcium-mediated signaling (GO:0050848)
negative regulation of cell projection organization (GO:0031345)
regulation of stress-activated MAPK cascade (GO:0032872)
modulation of chemical synaptic transmission (GO:0050804)
regulation of epithelial to mesenchymal transition (GO:0010717)
regulation of actin filament polymerization (GO:0030833)
regulation of embryonic development (GO:0045995)
tube closure (GO:0060606)
cardiac muscle cell action potential (GO:0086001)
non-canonical Wnt signaling pathway (GO:0035567)
negative regulation of transforming growth factor beta receptor signaling pathway (GO:0030512)
regulation of axonogenesis (GO:0050770)
negative regulation of neuron death (GO:1901215)
negative regulation of Wnt signaling pathway (GO:0030178)
positive regulation of developmental process (GO:0051094)
Rac protein signal transduction (GO:0016601)
endocardial cushion development (GO:0003197)
gamma-aminobutyric acid signaling pathway (GO:0007214)
positive regulation of phospholipase activity (GO:0010518)

positive regulation of neuron apoptotic process (GO:0043525)
production of small RNA involved in gene silencing by RNA (GO:0070918)
vascular endothelial growth factor receptor signaling pathway (GO:0048010)
connective tissue development (GO:0061448)
regulation of leukocyte chemotaxis (GO:0002688)
negative regulation of DNA biosynthetic process (GO:2000279)
regulation of ARF protein signal transduction (GO:0032012)
chondroitin sulfate metabolic process (GO:0030204)
chemical synaptic transmission (GO:0007268)
cardiac ventricle morphogenesis (GO:0003208)
branching morphogenesis of an epithelial tube (GO:0048754)
regulation of amyloid precursor protein biosynthetic process (GO:0042984)
negative regulation of toll-like receptor 4 signaling pathway (GO:0034144)
cellular response to reactive nitrogen species (GO:1902170)
vasodilation (GO:0042311)
regulation of microtubule-based movement (GO:0060632)
semaphorin-plexin signaling pathway involved in axon guidance (GO:1902287)
early endosome to Golgi transport (GO:0034498)
Golgi vesicle budding (GO:0048194)
protein hexamerization (GO:0034214)
B cell homeostasis (GO:0001782)
mitral valve morphogenesis (GO:0003183)
negative regulation of amyloid-beta formation (GO:1902430)
negative regulation of transcription by competitive promoter binding (GO:0010944)
positive regulation of vascular endothelial cell proliferation (GO:1905564)
axonal transport of mitochondrion (GO:0019896)
regulation of nephron tubule epithelial cell differentiation (GO:0072182)
negative regulation of peptidyl-lysine acetylation (GO:2000757)
RNA destabilization (GO:0050779)
ornithine metabolic process (GO:0006591)
actin crosslink formation (GO:0051764)
pericardium development (GO:0060039)
mRNA-containing ribonucleoprotein complex export from nucleus (GO:0071427)
positive regulation of cell proliferation (GO:0008284)
negative regulation of cell growth (GO:0030308)
actomyosin structure organization (GO:0031032)
regulation of cell communication (GO:0010646)
regulation of neurogenesis (GO:0050767)
epidermal growth factor receptor signaling pathway (GO:0007173)
positive regulation of apoptotic process (GO:0043065)
regulation of protein ubiquitination (GO:0031396)
regulation of ubiquitin-protein transferase activity (GO:0051438)
gene silencing by miRNA (GO:0035195)
regulation of protein tyrosine kinase activity (GO:0061097)
regulation of dendrite morphogenesis (GO:0048814)
positive regulation of macroautophagy (GO:0016239)
carboxylic acid biosynthetic process (GO:0046394)
adherens junction organization (GO:0034332)
positive regulation of dendritic spine development (GO:0060999)
glial cell development (GO:0021782)
negative regulation of telomere maintenance via telomerase (GO:0032211)
adrenergic receptor signaling pathway (GO:0071875)
heparan sulfate proteoglycan metabolic process (GO:0030201)

dopaminergic neuron differentiation (GO:0071542)
cell projection assembly (GO:0030031)
axon extension (GO:0048675)
adenylate cyclase-activating adrenergic receptor signaling pathway (GO:0071880)
Rho protein signal transduction (GO:0007266)
regulation of epithelial cell proliferation (GO:0050678)
negative regulation of cell cycle (GO:0045786)
cell-cell junction organization (GO:0045216)
regulation of cell proliferation (GO:0042127)
calcium ion homeostasis (GO:0055074)
negative regulation of canonical Wnt signaling pathway (GO:0090090)
cellular protein localization (GO:0034613)
cytosolic transport (GO:0016482)
mitotic cytokinesis (GO:0000281)
cell junction assembly (GO:0034329)
regulation of pathway-restricted SMAD protein phosphorylation (GO:0060393)
cell morphogenesis (GO:0000902)
central nervous system neuron differentiation (GO:0021953)
positive regulation of telomere maintenance (GO:0032206)
aminoglycan catabolic process (GO:0006026)
global genome nucleotide-excision repair (GO:0070911)
cellular response to DNA damage stimulus (GO:0006974)
regulation of phospholipase C activity (GO:1900274)
epithelial cell apoptotic process (GO:1904019)
mesoderm morphogenesis (GO:0048332)
response to laminar fluid shear stress (GO:0034616)
nuclear pore complex assembly (GO:0051292)
negative regulation of B cell proliferation (GO:0030889)
positive regulation of protein exit from endoplasmic reticulum (GO:0070863)
regulation of gene expression by genetic imprinting (GO:0006349)
negative regulation by host of viral process (GO:0044793)
negative regulation of intracellular estrogen receptor signaling pathway (GO:0033147)
ganglioside biosynthetic process (GO:0001574)
negative regulation of pathway-restricted SMAD protein phosphorylation (GO:0060394)
dicarboxylic acid biosynthetic process (GO:0043650)
Wnt signaling pathway involved in midbrain dopaminergic neuron differentiation (GO:1904953)
regulation of neuroblast proliferation (GO:1902692)
positive regulation of mRNA splicing, via spliceosome (GO:0048026)
regulation of phospholipid metabolic process (GO:1903725)
forelimb morphogenesis (GO:0035136)
skeletal muscle thin filament assembly (GO:0030240)
regulation of DNA-templated transcription, initiation (GO:2000142)
ventricular cardiac muscle tissue development (GO:0003229)
phospholipid translocation (GO:0045332)
nucleotide-excision repair, preincision complex stabilization (GO:0006293)
hepaticobiliary system development (GO:0061008)
brain development (GO:0007420)
apoptotic process (GO:0006915)
regulation of G1/S transition of mitotic cell cycle (GO:2000045)
protein methylation (GO:0006479)
negative regulation of protein modification by small protein conjugation or removal (GO:1903321)
macromolecular complex assembly (GO:0065003)
negative regulation of growth (GO:0045926)

enzyme linked receptor protein signaling pathway (GO:0007167)
neural tube closure (GO:0001843)
regulation of actin cytoskeleton organization (GO:0032956)
regulation of Ras protein signal transduction (GO:0046578)
regulation of JNK cascade (GO:0046328)
heterophilic cell-cell adhesion via plasma membrane cell adhesion molecules (GO:0007157)
positive regulation of transferase activity (GO:0051347)
positive regulation of protein autophosphorylation (GO:0031954)
peptidyl-S-diacylglycerol-L-cysteine biosynthetic process from peptidyl-cysteine (GO:0018231)
peptidyl-L-cysteine S-palmitoylation (GO:0018230)
cellular response to hexose stimulus (GO:0071331)
nucleotide-excision repair, DNA incision, 3'-to lesion (GO:0006295)
negative regulation of cell cycle G1/S phase transition (GO:1902807)
negative regulation of myeloid cell differentiation (GO:0045638)
negative regulation of actin filament polymerization (GO:0030837)
nucleotide-excision repair, DNA duplex unwinding (GO:0000717)
cellular response to unfolded protein (GO:0034620)
positive regulation of RNA splicing (GO:0033120)
regulation of monocyte chemotaxis (GO:0090025)
positive regulation of phosphatase activity (GO:0010922)
lipid translocation (GO:0034204)
ventricular cardiac muscle tissue morphogenesis (GO:0055010)
regulation of axon extension (GO:0030516)
negative regulation of endothelial cell migration (GO:0010596)
positive regulation of cell development (GO:0010720)
second-messenger-mediated signaling (GO:0019932)
regulation of protein secretion (GO:0050708)
cell cycle G1/S phase transition (GO:0044843)
negative regulation of defense response to virus (GO:0050687)
negative regulation of JUN kinase activity (GO:0043508)
positive regulation of protein deacetylation (GO:0090312)
calcium ion regulated exocytosis (GO:0017156)
regulation of hemopoiesis (GO:1903706)
mitochondrion distribution (GO:0048311)
negative regulation of amyloid precursor protein catabolic process (GO:1902992)
cellular response to interferon-beta (GO:0035458)
phosphate ion homeostasis (GO:0055062)
camera-type eye morphogenesis (GO:0048593)
metanephric mesenchyme development (GO:0072075)
response to nitric oxide (GO:0071731)
negative regulation of cardiac muscle hypertrophy (GO:0010614)
synaptic transmission, GABAergic (GO:0051932)
carbohydrate phosphorylation (GO:0046835)
regulation of autophagosome maturation (GO:1901096)
midbrain dopaminergic neuron differentiation (GO:1904948)
UDP-N-acetylglucosamine biosynthetic process (GO:0006048)
positive regulation of endothelial cell apoptotic process (GO:2000353)
cellular response to prostaglandin stimulus (GO:0071379)
morphogenesis of an epithelial sheet (GO:0002011)
regulation of trophoblast cell migration (GO:1901163)
embryonic eye morphogenesis (GO:0048048)
mesonephros development (GO:0001823)
positive regulation of receptor biosynthetic process (GO:0010870)

muscle cell migration (GO:0014812)
regulation of non-canonical Wnt signaling pathway (GO:2000050)
positive regulation of cell migration (GO:0030335)
negative regulation of macromolecule metabolic process (GO:0010605)
regulation of cysteine-type endopeptidase activity involved in apoptotic process (GO:0043281)
regulation of monooxygenase activity (GO:0032768)
regulation of axon extension involved in axon guidance (GO:0048841)
adenylate cyclase-activating G-protein coupled receptor signaling pathway (GO:0007189)
positive regulation of telomerase activity (GO:0051973)
actin filament bundle organization (GO:0061572)
regulation of endothelial cell apoptotic process (GO:2000351)
homophilic cell adhesion via plasma membrane adhesion molecules (GO:0007156)
peptidyl-tyrosine phosphorylation (GO:0018108)
response to calcium ion (GO:0051592)
vesicle-mediated transport (GO:0016192)
positive regulation of programmed cell death (GO:0043068)
cell-cell adhesion via plasma-membrane adhesion molecules (GO:0098742)
negative regulation of protein ubiquitination (GO:0031397)
regulation of bone mineralization (GO:0030500)
regulation of smoothened signaling pathway (GO:0008589)
sodium ion transmembrane transport (GO:0035725)
histone deacetylation (GO:0016575)
regulation of cell morphogenesis involved in differentiation (GO:0010769)
cardiac muscle tissue morphogenesis (GO:0055008)
regulation of muscle cell differentiation (GO:0051147)
regulation of filopodium assembly (GO:0051489)
regulation of cytoskeleton organization (GO:0051493)
regulation of cytokine biosynthetic process (GO:0042035)
UDP-N-acetylglucosamine metabolic process (GO:0006047)
positive regulation of amyloid precursor protein catabolic process (GO:1902993)
positive regulation of branching involved in ureteric bud morphogenesis (GO:0090190)
eye photoreceptor cell development (GO:0042462)
response to prostaglandin E (GO:0034695)
spliceosomal tri-snRNP complex assembly (GO:0000244)
regulation of establishment of endothelial barrier (GO:1903140)
regulation of mononuclear cell migration (GO:0071675)
negative regulation of histone modification (GO:0031057)
negative regulation of vascular smooth muscle cell proliferation (GO:1904706)
cardiac muscle fiber development (GO:0048739)
peptidyl-methionine modification (GO:0018206)
L-serine metabolic process (GO:0006563)
regulation of vascular endothelial cell proliferation (GO:1905562)
positive regulation of stem cell differentiation (GO:2000738)
negative regulation of myoblast differentiation (GO:0045662)
negative regulation of androgen receptor signaling pathway (GO:0060766)
regulation of retrograde protein transport, ER to cytosol (GO:1904152)
skeletal myofibril assembly (GO:0014866)
regulation of IRE1-mediated unfolded protein response (GO:1903894)
amino sugar biosynthetic process (GO:0046349)
cell differentiation in hindbrain (GO:0021533)
vesicle organization (GO:0016050)
negative regulation of response to stimulus (GO:0048585)
cellular metal ion homeostasis (GO:0006875)

negative regulation of cell death (GO:0060548)
positive regulation of axonogenesis (GO:0050772)
negative regulation of G0 to G1 transition (GO:0070317)
actin filament bundle assembly (GO:0051017)
positive regulation of multi-organism process (GO:0043902)
myelination (GO:0042552)
negative regulation of cytoskeleton organization (GO:0051494)
regulation of chromosome organization (GO:0033044)
negative regulation of G1/S transition of mitotic cell cycle (GO:2000134)
regulation of epidermal growth factor-activated receptor activity (GO:0007176)
positive regulation of endopeptidase activity (GO:0010950)
DNA replication initiation (GO:0006270)
protein alpha-1,2-demannosylation (GO:0036508)
proteoglycan biosynthetic process (GO:0030166)
cAMP metabolic process (GO:0046058)
camera-type eye development (GO:0043010)
regulation of anatomical structure morphogenesis (GO:0022603)
DNA damage response, signal transduction by p53 class mediator (GO:0030330)
retrograde transport, endosome to Golgi (GO:0042147)
positive regulation of ossification (GO:0045778)
negative regulation of protein serine/threonine kinase activity (GO:0071901)
positive regulation of endothelial cell proliferation (GO:0001938)
extrinsic apoptotic signaling pathway (GO:0097191)
regulation of cardiac conduction (GO:1903779)
skeletal system development (GO:0001501)
regulation of insulin secretion (GO:0050796)
histone modification (GO:0016570)
regulation of cellular amide metabolic process (GO:0034248)
membrane depolarization during action potential (GO:0086010)
DNA damage checkpoint (GO:0000077)
positive regulation of neuron death (GO:1901216)
regulation of translation (GO:0006417)
establishment of protein localization to membrane (GO:0090150)
regulation of acyl-CoA biosynthetic process (GO:0050812)
multivesicular body sorting pathway (GO:0071985)
serine family amino acid biosynthetic process (GO:0009070)
atrial septum development (GO:0003283)
regulation of production of miRNAs involved in gene silencing by miRNA (GO:1903798)
glutamine family amino acid biosynthetic process (GO:0009084)
cardiocyte differentiation (GO:0035051)
poly(A)+ mRNA export from nucleus (GO:0016973)
regulation of execution phase of apoptosis (GO:1900117)
positive regulation of ERAD pathway (GO:1904294)
nucleotide metabolic process (GO:0009117)
regulation of T cell cytokine production (GO:0002724)
endothelial cell differentiation (GO:0045446)
positive regulation of smoothened signaling pathway (GO:0045880)
detection of calcium ion (GO:0005513)
modulation by virus of host process (GO:0019054)
negative regulation of macrophage derived foam cell differentiation (GO:0010745)
glycerolipid catabolic process (GO:0046503)
synaptic vesicle cycle (GO:0099504)
glycosaminoglycan biosynthetic process (GO:0006024)

negative regulation of cysteine-type endopeptidase activity involved in apoptotic process (GO:0043154)
peptidyl-threonine phosphorylation (GO:0018107)
regulation of calcium ion-dependent exocytosis (GO:0017158)
chondroitin sulfate proteoglycan biosynthetic process (GO:0050650)
positive regulation of filopodium assembly (GO:0051491)
response to alcohol (GO:0097305)
regulation of lamellipodium assembly (GO:0010591)
positive regulation of peptidyl-threonine phosphorylation (GO:0010800)
regulation of ryanodine-sensitive calcium-release channel activity (GO:0060314)
negative regulation of signal transduction (GO:0009968)
negative regulation of protein catabolic process (GO:0042177)
ERBB2 signaling pathway (GO:0038128)
regulation of G0 to G1 transition (GO:0070316)
regulation of mitotic cell cycle (GO:0007346)
regulation of endothelial cell migration (GO:0010594)
response to organic cyclic compound (GO:0014070)
positive regulation of plasma membrane bounded cell projection assembly (GO:0120034)
heart development (GO:0007507)
membrane fusion (GO:0061025)
negative regulation of supramolecular fiber organization (GO:1902904)
regulation of signal transduction (GO:0009966)
protein targeting (GO:0006605)
cellular divalent inorganic cation homeostasis (GO:0072503)
regulation of programmed cell death (GO:0043067)
positive regulation of endothelial cell migration (GO:0010595)
cytoskeleton-dependent cytokinesis (GO:0061640)
response to peptide hormone (GO:0043434)
response to growth factor (GO:0070848)
nucleotide biosynthetic process (GO:0009165)
regulation of pri-miRNA transcription from RNA polymerase II promoter (GO:1902893)
response to ketone (GO:1901654)
internal peptidyl-lysine acetylation (GO:0018393)
regulation of rhodopsin mediated signaling pathway (GO:0022400)
regulation of Golgi organization (GO:1903358)
regulation of platelet aggregation (GO:0090330)
regulation of acetyl-CoA biosynthetic process from pyruvate (GO:0010510)
regulation of neural precursor cell proliferation (GO:2000177)
cellular protein complex disassembly (GO:0043624)
purine-containing compound salvage (GO:0043101)
amyloid-beta metabolic process (GO:0050435)
regulation of ventricular cardiac muscle cell membrane repolarization (GO:0060307)
Golgi to vacuole transport (GO:0006896)
oligosaccharide-lipid intermediate biosynthetic process (GO:0006490)
adaptive immune response based on somatic recombination of immune receptors built from immunoglobulin su[illegible]
interleukin-6-mediated signaling pathway (GO:0070102)
regulation of response to interferon-gamma (GO:0060330)
positive regulation of blood vessel diameter (GO:0097755)
ribonucleoside monophosphate biosynthetic process (GO:0009156)
glucosamine-containing compound metabolic process (GO:1901071)
positive regulation of myoblast differentiation (GO:0045663)
myotube cell development (GO:0014904)
regulation of phosphate metabolic process (GO:0019220)
positive regulation of DNA biosynthetic process (GO:2000573)

fat cell differentiation (GO:0045444)
regulation of peptidyl-serine phosphorylation (GO:0033135)
histone acetylation (GO:0016573)
regulation of cation channel activity (GO:2001257)
regulation of MAP kinase activity (GO:0043405)
regulation of substrate adhesion-dependent cell spreading (GO:1900024)
DNA duplex unwinding (GO:0032508)
regulation of MAPK cascade (GO:0043408)
negative regulation of small GTPase mediated signal transduction (GO:0051058)
positive regulation of substrate adhesion-dependent cell spreading (GO:1900026)
protein targeting to vacuole (GO:0006623)
muscle fiber development (GO:0048747)
cellular response to topologically incorrect protein (GO:0035967)
negative regulation of smooth muscle cell proliferation (GO:0048662)
negative regulation of cysteine-type endopeptidase activity (GO:2000117)
muscle contraction (GO:0006936)
negative regulation of apoptotic process (GO:0043066)
response to retinoic acid (GO:0032526)
chloride transmembrane transport (GO:1902476)
apoptotic DNA fragmentation (GO:0006309)
nuclear pore organization (GO:0006999)
endocardial cushion morphogenesis (GO:0003203)
positive regulation of glycolytic process (GO:0045821)
positive regulation of mesonephros development (GO:0061213)
positive regulation of coenzyme metabolic process (GO:0051197)
regulation of mitotic metaphase/anaphase transition (GO:0030071)
positive regulation of potassium ion transmembrane transport (GO:1901381)
regulation of establishment of cell polarity (GO:2000114)
mitochondrion localization (GO:0051646)
mesenchyme morphogenesis (GO:0072132)
negative regulation of lipid catabolic process (GO:0050995)
positive regulation of phosphoprotein phosphatase activity (GO:0032516)
negative regulation of cellular component movement (GO:0051271)
response to cholesterol (GO:0070723)
ganglioside metabolic process (GO:0001573)
regulation of delayed rectifier potassium channel activity (GO:1902259)
negative regulation of muscle cell differentiation (GO:0051148)
monovalent inorganic anion homeostasis (GO:0055083)
JAK-STAT cascade involved in growth hormone signaling pathway (GO:0060397)
positive regulation of potassium ion transport (GO:0043268)
nucleobase-containing small molecule catabolic process (GO:0034656)
regulation of branching involved in ureteric bud morphogenesis (GO:0090189)
selective autophagy (GO:0061912)
regulation of telomerase activity (GO:0051972)
cellular response to retinoic acid (GO:0071300)
cellular response to calcium ion (GO:0071277)
regulation of protein modification process (GO:0031399)
response to cytokine (GO:0034097)
chloride transport (GO:0006821)
regulation of peptide hormone secretion (GO:0090276)
peptidyl-threonine modification (GO:0018210)
positive regulation of signal transduction (GO:0009967)
cellular response to alcohol (GO:0097306)

acute inflammatory response (GO:0002526)
cellular amino acid biosynthetic process (GO:0008652)
positive regulation of extrinsic apoptotic signaling pathway (GO:2001238)
lytic vacuole organization (GO:0080171)
production of miRNAs involved in gene silencing by miRNA (GO:0035196)
regulation of lipid biosynthetic process (GO:0046890)
cGMP metabolic process (GO:0046068)
positive regulation of fibroblast proliferation (GO:0048146)
regulation of vasculature development (GO:1901342)
ribonucleoprotein complex assembly (GO:0022618)
regulation of cytokine-mediated signaling pathway (GO:0001959)
positive regulation of epithelial cell migration (GO:0010634)
mRNA 3'-end processing (GO:0031124)
regulation of apoptotic process (GO:0042981)
membrane depolarization (GO:0051899)
eye development (GO:0001654)
intrinsic apoptotic signaling pathway by p53 class mediator (GO:0072332)
negative regulation of DNA metabolic process (GO:0051053)
regulation of multicellular organismal development (GO:2000026)
negative regulation of protein metabolic process (GO:0051248)
cellular calcium ion homeostasis (GO:0006874)
negative regulation of kinase activity (GO:0033673)
gland development (GO:0048732)
protein localization to microtubule organizing center (GO:1905508)
peptidyl-threonine dephosphorylation (GO:0035970)
regulation of RIG-I signaling pathway (GO:0039535)
protein deacylation (GO:0035601)
negative regulation of striated muscle cell differentiation (GO:0051154)
ventricular cardiac muscle cell action potential (GO:0086005)
extracellular matrix assembly (GO:0085029)
regulation of energy homeostasis (GO:2000505)
glutamine family amino acid catabolic process (GO:0009065)
regulation of osteoblast proliferation (GO:0033688)
genitalia development (GO:0048806)
negative regulation of peptide hormone secretion (GO:0090278)
odontogenesis of dentin-containing tooth (GO:0042475)
artery development (GO:0060840)
negative regulation of lipid storage (GO:0010888)
positive regulation of epithelial cell apoptotic process (GO:1904037)
lipopolysaccharide-mediated signaling pathway (GO:0031663)
histone H2A acetylation (GO:0043968)
cellular macromolecule biosynthetic process (GO:0034645)
nucleotide-excision repair, preincision complex assembly (GO:0006294)
superoxide metabolic process (GO:0006801)
vacuolar transport (GO:0007034)
cortical cytoskeleton organization (GO:0030865)
protein alkylation (GO:0008213)
coenzyme biosynthetic process (GO:0009108)
semaphorin-plexin signaling pathway (GO:0071526)
regulation of cellular biosynthetic process (GO:0031326)
carbohydrate derivative biosynthetic process (GO:1901137)
regulation of myoblast differentiation (GO:0045661)
protein N-linked glycosylation via asparagine (GO:0018279)

sprouting angiogenesis (GO:0002040)
positive regulation of viral life cycle (GO:1903902)
negative regulation of multicellular organismal process (GO:0051241)
nuclear export (GO:0051168)
transforming growth factor beta receptor signaling pathway (GO:0007179)
response to interleukin-1 (GO:0070555)
positive regulation of JNK cascade (GO:0046330)
circulatory system development (GO:0072359)
peptidyl-asparagine modification (GO:0018196)
regulation of synaptic transmission, glutamatergic (GO:0051966)
peptidyl-tyrosine autophosphorylation (GO:0038083)
negative regulation of ossification (GO:0030279)
regulation of defense response to virus by host (GO:0050691)
regulation of cell adhesion mediated by integrin (GO:0033628)
regulation of cation transmembrane transport (GO:1904062)
positive regulation of phospholipase C activity (GO:0010863)
aminoglycan metabolic process (GO:0006022)
ATP-dependent chromatin remodeling (GO:0043044)
regulation of sodium ion transmembrane transport (GO:1902305)
cellular response to insulin stimulus (GO:0032869)
cellular response to lipid (GO:0071396)
glycosaminoglycan metabolic process (GO:0030203)
protein targeting to membrane (GO:0006612)
regulation of DNA replication (GO:0006275)
regulation of protein sumoylation (GO:0033233)
cardiac myofibril assembly (GO:0055003)
positive regulation of transcription initiation from RNA polymerase II promoter (GO:0060261)
regulation of toll-like receptor 4 signaling pathway (GO:0034143)
ureteric bud development (GO:0001657)
RNA stabilization (GO:0043489)
positive regulation of striated muscle cell differentiation (GO:0051155)
gastrulation (GO:0007369)
histone H2A monoubiquitination (GO:0035518)
negative regulation of cell development (GO:0010721)
cardiac atrium morphogenesis (GO:0003209)
negative regulation of phosphoprotein phosphatase activity (GO:0032515)
DNA replication checkpoint (GO:0000076)
pharyngeal system development (GO:0060037)
regulation of transcription from RNA polymerase I promoter (GO:0006356)
neurotrophin signaling pathway (GO:0038179)
regulation of establishment or maintenance of cell polarity (GO:0032878)
synaptic vesicle transport (GO:0048489)
mesonephric tubule development (GO:0072164)
regulation of early endosome to late endosome transport (GO:2000641)
cellular response to inorganic substance (GO:0071241)
membrane depolarization during cardiac muscle cell action potential (GO:0086012)
positive regulation of focal adhesion assembly (GO:0051894)
negative regulation of axon guidance (GO:1902668)
regulation of telomere maintenance via telomere lengthening (GO:1904356)
cartilage development (GO:0051216)
cellular response to interleukin-12 (GO:0071349)
interleukin-12-mediated signaling pathway (GO:0035722)
regulation of actin filament-based process (GO:0032970)

regulation of heart contraction (GO:0008016)
positive regulation of cellular process (GO:0048522)
positive regulation of protein modification process (GO:0031401)
positive regulation of ubiquitin-protein transferase activity (GO:0051443)
renal water homeostasis (GO:0003091)
anterograde axonal transport (GO:0008089)
regulation of protein autophosphorylation (GO:0031952)
lysosome organization (GO:0007040)
nucleobase-containing small molecule interconversion (GO:0015949)
liver development (GO:0001889)
positive regulation of bone mineralization (GO:0030501)
negative regulation of toll-like receptor signaling pathway (GO:0034122)
negative regulation of mitotic cell cycle phase transition (GO:1901991)
regulation of endothelial cell proliferation (GO:0001936)
adenylate cyclase-inhibiting G-protein coupled receptor signaling pathway (GO:0007193)
regulation of viral genome replication (GO:0045069)
nuclear-transcribed mRNA catabolic process, deadenylation-dependent decay (GO:0000288)
polyol metabolic process (GO:0019751)
inositol phosphate metabolic process (GO:0043647)
positive regulation of pathway-restricted SMAD protein phosphorylation (GO:0010862)
response to glucose (GO:0009749)
myofibril assembly (GO:0030239)
regulation of cellular macromolecule biosynthetic process (GO:2000112)
positive regulation of kinase activity (GO:0033674)
protein oligomerization (GO:0051259)
glucan catabolic process (GO:0009251)
regulation of telomerase RNA localization to Cajal body (GO:1904872)
regulation of amyloid-beta formation (GO:1902003)
protein localization to centrosome (GO:0071539)
regulation of lipid catabolic process (GO:0050994)
anterior/posterior axis specification (GO:0009948)
positive regulation of cyclic nucleotide biosynthetic process (GO:0030804)
negative regulation of fibroblast proliferation (GO:0048147)
regulation of chemokine production (GO:0032642)
regulation of cardiac muscle cell membrane repolarization (GO:0099623)
negative regulation of peptidyl-serine phosphorylation (GO:0033137)
dolichol-linked oligosaccharide biosynthetic process (GO:0006488)
acute-phase response (GO:0006953)
regulation of cAMP-mediated signaling (GO:0043949)
negative regulation of oxidative stress-induced intrinsic apoptotic signaling pathway (GO:1902176)
positive regulation of carbohydrate metabolic process (GO:0045913)
piRNA metabolic process (GO:0034587)
positive regulation of morphogenesis of an epithelium (GO:1905332)
negative regulation of calcium-mediated signaling (GO:0050849)
positive regulation of synaptic transmission, glutamatergic (GO:0051968)
glycosphingolipid biosynthetic process (GO:0006688)
calcium-mediated signaling using intracellular calcium source (GO:0035584)
maintenance of protein localization in organelle (GO:0072595)
positive regulation of stress-activated MAPK cascade (GO:0032874)
cellular response to hormone stimulus (GO:0032870)
RNA transport (GO:0050658)
spindle assembly (GO:0051225)
regulation of glutamate receptor signaling pathway (GO:1900449)

negative regulation of receptor activity (GO:2000272)
activation of adenylate cyclase activity (GO:0007190)
endodermal cell differentiation (GO:0035987)
regulation of cell-cell adhesion (GO:0022407)
negative regulation of fat cell differentiation (GO:0045599)
regulation of erythrocyte differentiation (GO:0045646)
regulation of nitric oxide biosynthetic process (GO:0045428)
vasculogenesis (GO:0001570)
protein deacetylation (GO:0006476)
positive regulation of dendrite development (GO:1900006)
Golgi vesicle transport (GO:0048193)
cellular response to organonitrogen compound (GO:0071417)
inorganic anion transmembrane transport (GO:0098661)
cellular response to decreased oxygen levels (GO:0036294)
positive regulation of cell adhesion (GO:0045785)
negative regulation of mitotic cell cycle (GO:0045930)
regulation of epithelial cell migration (GO:0010632)
negative regulation of cell cycle process (GO:0010948)
cellular response to hypoxia (GO:0071456)
cellular response to reactive oxygen species (GO:0034614)
cellular response to oxygen-containing compound (GO:1901701)
positive regulation of cell-cell adhesion (GO:0022409)
chemical synaptic transmission, postsynaptic (GO:0099565)
positive regulation of cell-matrix adhesion (GO:0001954)
embryonic organ morphogenesis (GO:0048562)
regulation of postsynaptic membrane potential (GO:0060078)
exonucleolytic nuclear-transcribed mRNA catabolic process involved in deadenylation-dependent decay (GO:00
positive regulation of biomineral tissue development (GO:0070169)
positive regulation of Notch signaling pathway (GO:0045747)
positive regulation of adenylate cyclase activity (GO:0045762)
activation of MAPKK activity (GO:0000186)
positive regulation of cytokine-mediated signaling pathway (GO:0001961)
regulation of mitotic spindle assembly (GO:1901673)
positive regulation of transforming growth factor beta receptor signaling pathway (GO:0030511)
protein ADP-ribosylation (GO:0006471)
negative regulation of proteolysis involved in cellular protein catabolic process (GO:1903051)
muscle tissue morphogenesis (GO:0060415)
negative regulation of lipid metabolic process (GO:0045833)
intracellular estrogen receptor signaling pathway (GO:0030520)
striated muscle cell differentiation (GO:0051146)
histone deubiquitination (GO:0016578)
insulin secretion (GO:0030073)
regulation of neuron migration (GO:2001222)
nucleotide-sugar biosynthetic process (GO:0009226)
positive regulation of pri-miRNA transcription from RNA polymerase II promoter (GO:1902895)
positive regulation of adherens junction organization (GO:1903393)
protein heterotetramerization (GO:0051290)
mitochondrial calcium ion homeostasis (GO:0051560)
regulation of cAMP biosynthetic process (GO:0030817)
positive regulation of cellular response to transforming growth factor beta stimulus (GO:1903846)
response to interferon-beta (GO:0035456)
activin receptor signaling pathway (GO:0032924)
histone methylation (GO:0016571)

regulation of myotube differentiation (GO:0010830)
negative regulation of binding (GO:0051100)
positive regulation of cell cycle arrest (GO:0071158)
regulation of release of sequestered calcium ion into cytosol (GO:0051279)
small GTPase mediated signal transduction (GO:0007264)
protein maturation (GO:0051604)
regulation of blood vessel endothelial cell migration (GO:0043535)
neurogenesis (GO:0022008)
regulation of cellular protein metabolic process (GO:0032268)
positive regulation of JUN kinase activity (GO:0043507)
peptidyl-serine modification (GO:0018209)
apoptotic mitochondrial changes (GO:0008637)
regulation of wound healing (GO:0061041)
regulation of JUN kinase activity (GO:0043506)
anterior/posterior pattern specification (GO:0009952)
regulation of double-strand break repair (GO:2000779)
regulation of protein localization to early endosome (GO:1902965)
negative regulation of cholesterol storage (GO:0010887)
protein repair (GO:0030091)
hindlimb morphogenesis (GO:0035137)
smooth muscle tissue development (GO:0048745)
regulation of oxidative stress-induced neuron intrinsic apoptotic signaling pathway (GO:1903376)
embryonic camera-type eye development (GO:0031076)
epithelial to mesenchymal transition involved in endocardial cushion formation (GO:0003198)
arginine biosynthetic process (GO:0006526)
negative regulation of chromosome organization (GO:2001251)
response to granulocyte macrophage colony-stimulating factor (GO:0097012)
negative regulation of transforming growth factor beta production (GO:0071635)
regulation of lymphocyte apoptotic process (GO:0070228)
vitamin K metabolic process (GO:0042373)
lipoxin biosynthetic process (GO:2001301)
vascular smooth muscle cell differentiation (GO:0035886)
positive regulation of myelination (GO:0031643)
cellular hyperosmotic response (GO:0071474)
regulation of phagocytosis, engulfment (GO:0060099)
astral microtubule organization (GO:0030953)
adenylate cyclase-inhibiting G-protein coupled acetylcholine receptor signaling pathway (GO:0007197)
establishment of endothelial intestinal barrier (GO:0090557)
cardiac ventricle formation (GO:0003211)
response to DNA damage checkpoint signaling (GO:0072423)
negative regulation of endoplasmic reticulum calcium ion concentration (GO:0032471)
protein deubiquitination involved in ubiquitin-dependent protein catabolic process (GO:0071947)
negative regulation of muscle cell apoptotic process (GO:0010656)
histone H4-K12 acetylation (GO:0043983)
male genitalia development (GO:0030539)
negative regulation of neural precursor cell proliferation (GO:2000178)
regulation of mesenchymal stem cell differentiation (GO:2000739)
3'-phosphoadenosine 5'-phosphosulfate biosynthetic process (GO:0050428)
phosphorylation of RNA polymerase II C-terminal domain (GO:0070816)
hematopoietic stem cell differentiation (GO:0060218)
platelet dense granule organization (GO:0060155)
positive regulation of granulocyte differentiation (GO:0030854)
gap junction assembly (GO:0016264)

rescue of stalled ribosome (GO:0072344)
immune response-inhibiting cell surface receptor signaling pathway (GO:0002767)
citrulline metabolic process (GO:0000052)
regulation of cellular response to oxidative stress (GO:1900407)
regulation of NK T cell activation (GO:0051133)
embryonic skeletal joint morphogenesis (GO:0060272)
negative regulation of vasculature development (GO:1901343)
lens fiber cell development (GO:0070307)
pronephros development (GO:0048793)
positive regulation of blood pressure (GO:0045777)
Golgi disassembly (GO:0090166)
response to UV-A (GO:0070141)
acetyl-CoA biosynthetic process (GO:0006085)
gastrulation with mouth forming second (GO:0001702)
positive regulation of action potential (GO:0045760)
response to lead ion (GO:0010288)
adenylate cyclase-inhibiting G-protein coupled glutamate receptor signaling pathway (GO:0007196)
positive regulation of microtubule motor activity (GO:2000576)
regulation of glial cell differentiation (GO:0045685)
mRNA 5'-splice site recognition (GO:0000395)
cerebral cortex cell migration (GO:0021795)
purine ribonucleoside monophosphate catabolic process (GO:0009169)
neurotransmitter loading into synaptic vesicle (GO:0098700)
positive regulation of protein localization to endosome (GO:1905668)
vesicle fusion with Golgi apparatus (GO:0048280)
negative regulation of calcium ion-dependent exocytosis (GO:0045955)
regulation of aldosterone biosynthetic process (GO:0032347)
heparan sulfate proteoglycan biosynthetic process, enzymatic modification (GO:0015015)
regulation of cell cycle checkpoint (GO:1901976)
regulation of cortisol biosynthetic process (GO:2000064)
SREBP signaling pathway (GO:0032933)
aggrephagy (GO:0035973)
plasma membrane fusion (GO:0045026)
polyphosphate metabolic process (GO:0006797)
coronary vasculature development (GO:0060976)
mesenchymal to epithelial transition involved in metanephros morphogenesis (GO:0003337)
heart trabecula formation (GO:0060347)
regulation of anion channel activity (GO:0010359)
regulation of dendritic cell chemotaxis (GO:2000508)
negative regulation of delayed rectifier potassium channel activity (GO:1902260)
positive regulation of autophagosome maturation (GO:1901098)
negative regulation of DNA repair (GO:0045738)
epithelial tube formation (GO:0072175)
mesenchymal cell differentiation involved in kidney development (GO:0072161)
morphogenesis of an endothelium (GO:0003159)
positive regulation of centrosome cycle (GO:0046607)
maintenance of protein location in extracellular region (GO:0071694)
venous blood vessel morphogenesis (GO:0048845)
cellular response to cold (GO:0070417)
insulin secretion involved in cellular response to glucose stimulus (GO:0035773)
negative regulation of mononuclear cell migration (GO:0071676)
cellular response to granulocyte macrophage colony-stimulating factor stimulus (GO:0097011)
peptidyl-serine autophosphorylation (GO:0036289)

regulation of sarcomere organization (GO:0060297)
glutamate catabolic process (GO:0006538)
regulation of t-circle formation (GO:1904429)
endoplasmic reticulum membrane organization (GO:0090158)
phospholipase C-activating G-protein coupled acetylcholine receptor signaling pathway (GO:0007207)
positive regulation of protein localization to centrosome (GO:1904781)
fructose 6-phosphate metabolic process (GO:0006002)
sphingosine-1-phosphate signaling pathway (GO:0003376)
positive regulation of interferon-gamma secretion (GO:1902715)
regulation of fat cell proliferation (GO:0070344)
embryonic camera-type eye morphogenesis (GO:0048596)
succinyl-CoA metabolic process (GO:0006104)
regulation of endothelial cell development (GO:1901550)
mesodermal cell fate commitment (GO:0001710)
positive regulation of hormone metabolic process (GO:0032352)
SCF complex assembly (GO:0010265)
regulation of superoxide metabolic process (GO:0090322)
N-terminal peptidyl-methionine acetylation (GO:0017196)
positive regulation of protein localization to early endosome (GO:1902966)
positive regulation of amyloid-beta clearance (GO:1900223)
response to gonadotropin (GO:0034698)
snRNA modification (GO:0040031)
negative regulation of bone resorption (GO:0045779)
regulation of adiponectin secretion (GO:0070163)
positive regulation of extrinsic apoptotic signaling pathway via death domain receptors (GO:1902043)
regulation of MHC class I biosynthetic process (GO:0045343)
phagosome-lysosome fusion (GO:0090385)
positive regulation of protein acetylation (GO:1901985)
positive regulation of synaptic transmission (GO:0050806)
insulin receptor signaling pathway (GO:0008286)
MAPK cascade (GO:0000165)
Golgi to endosome transport (GO:0006895)
regulation of epidermal cell differentiation (GO:0045604)
negative regulation of ion transport (GO:0043271)
cellular polysaccharide catabolic process (GO:0044247)
calcium-independent cell-cell adhesion via plasma membrane cell-adhesion molecules (GO:0016338)
negative regulation of response to biotic stimulus (GO:0002832)
positive regulation of gene silencing by miRNA (GO:2000637)
branching involved in ureteric bud morphogenesis (GO:0001658)
growth hormone receptor signaling pathway (GO:0060396)
inactivation of MAPK activity (GO:0000188)
telomere maintenance via telomerase (GO:0007004)
vesicle cytoskeletal trafficking (GO:0099518)
eye morphogenesis (GO:0048592)
negative regulation of plasma membrane bounded cell projection assembly (GO:0120033)
negative regulation of interferon-gamma production (GO:0032689)
chondroitin sulfate biosynthetic process (GO:0030206)
entrainment of circadian clock by photoperiod (GO:0043153)
positive regulation of cAMP biosynthetic process (GO:0030819)
mesoderm formation (GO:0001707)
proteoglycan metabolic process (GO:0006029)
ureteric bud morphogenesis (GO:0060675)
regulation of calcium ion transmembrane transport (GO:1903169)

regulation of protein phosphorylation (GO:0001932)
termination of RNA polymerase II transcription (GO:0006369)
regulation of type I interferon production (GO:0032479)
regulation of peptidyl-tyrosine phosphorylation (GO:0050730)
positive regulation of RNA metabolic process (GO:0051254)
regulation of cyclin-dependent protein kinase activity (GO:1904029)
nuclear-transcribed mRNA catabolic process, exonucleolytic (GO:0000291)
negative regulation of protein complex assembly (GO:0031333)
negative regulation of epithelial cell migration (GO:0010633)
regulation of phosphoprotein phosphatase activity (GO:0043666)
regulation of cardiac muscle contraction (GO:0055117)
neural crest cell development (GO:0014032)
positive regulation of osteoblast differentiation (GO:0045669)
protein kinase B signaling (GO:0043491)
RNA metabolic process (GO:0016070)
endocytosis (GO:0006897)
protein import into nucleus (GO:0006606)
plasma membrane bounded cell projection morphogenesis (GO:0120039)
positive regulation of nervous system development (GO:0051962)
regulation of DNA binding (GO:0051101)
calcium ion transmembrane transport (GO:0070588)
regulation of innate immune response (GO:0045088)
regulation of osteoblast differentiation (GO:0045667)
positive regulation of transcription from RNA polymerase I promoter (GO:0045943)
positive regulation of cellular component movement (GO:0051272)
mRNA transcription (GO:0009299)
dendritic cell differentiation (GO:0097028)
cortical actin cytoskeleton organization (GO:0030866)
intrinsic apoptotic signaling pathway in response to DNA damage by p53 class mediator (GO:0042771)
zinc II ion transport (GO:0006829)
negative regulation of cytokine biosynthetic process (GO:0042036)
establishment of endothelial barrier (GO:0061028)
branched-chain amino acid catabolic process (GO:0009083)
regulation of interleukin-2 production (GO:0032663)
positive regulation of defense response to virus by host (GO:0002230)
cellular response to misfolded protein (GO:0071218)
positive regulation of lipid metabolic process (GO:0045834)
mitochondrial calcium ion transmembrane transport (GO:0006851)
embryonic skeletal system development (GO:0048706)
photoperiodism (GO:0009648)
ERK1 and ERK2 cascade (GO:0070371)
glutamate metabolic process (GO:0006536)
positive regulation of transcription from RNA polymerase II promoter involved in cellular response to chemical st
RNA-dependent DNA biosynthetic process (GO:0006278)
positive regulation of leukocyte cell-cell adhesion (GO:1903039)
negative regulation of ion transmembrane transporter activity (GO:0032413)
regulation of potassium ion transport (GO:0043266)
muscle cell differentiation (GO:0042692)
endoderm formation (GO:0001706)
regulation of response to DNA damage stimulus (GO:2001020)
plasma membrane organization (GO:0007009)
glutamate receptor signaling pathway (GO:0007215)
cardiac muscle tissue development (GO:0048738)

protein autophosphorylation (GO:0046777)
sulfur compound biosynthetic process (GO:0044272)
regulation of Notch signaling pathway (GO:0008593)
negative regulation of developmental process (GO:0051093)
calcium-mediated signaling (GO:0019722)
regulation of cyclic-nucleotide phosphodiesterase activity (GO:0051342)
intracellular sterol transport (GO:0032366)
modulation by symbiont of host cellular process (GO:0044068)
smooth muscle cell migration (GO:0014909)
cellular response to interleukin-18 (GO:0071351)
bleb assembly (GO:0032060)
AV node cell action potential (GO:0086016)
regulation of myosin-light-chain-phosphatase activity (GO:0035507)
protein C-linked glycosylation via tryptophan (GO:0018317)
purine ribonucleoside bisphosphate biosynthetic process (GO:0034036)
positive regulation of dendritic cell chemotaxis (GO:2000510)
negative regulation of transcription elongation from RNA polymerase II promoter (GO:0034244)
regulation of DNA endoreduplication (GO:0032875)
regulation of ER to Golgi vesicle-mediated transport (GO:0060628)
elastic fiber assembly (GO:0048251)
reproductive system development (GO:0061458)
negative regulation of cardiac muscle cell differentiation (GO:2000726)
miRNA loading onto RISC involved in gene silencing by miRNA (GO:0035280)
translational readthrough (GO:0006451)
cellular response to sterol depletion (GO:0071501)
valine metabolic process (GO:0006573)
L-alanine transport (GO:0015808)
molybdopterin cofactor metabolic process (GO:0043545)
regulation of T-helper 17 type immune response (GO:2000316)
chitin metabolic process (GO:0006030)
formation of translation preinitiation complex (GO:0001731)
box C/D snoRNP assembly (GO:0000492)
positive regulation of protein deubiquitination (GO:1903003)
negative regulation of cell adhesion mediated by integrin (GO:0033629)
regulation of smooth muscle cell differentiation (GO:0051150)
fructose metabolic process (GO:0006000)
adenine nucleotide transport (GO:0051503)
protein C-linked glycosylation via 2'-alpha-mannosyl-L-tryptophan (GO:0018406)
glycosylceramide metabolic process (GO:0006677)
regulation of low-density lipoprotein particle receptor biosynthetic process (GO:0045714)
regulation of atrial cardiac muscle cell membrane repolarization (GO:0060372)
positive regulation of NK T cell activation (GO:0051135)
regulation of high voltage-gated calcium channel activity (GO:1901841)
positive regulation of inositol phosphate biosynthetic process (GO:0060732)
negative regulation of membrane protein ectodomain proteolysis (GO:0051045)
regulation of transcription from RNA polymerase II promoter involved in heart development (GO:1901213)
negative regulation of nitric oxide metabolic process (GO:1904406)
regulation by virus of viral protein levels in host cell (GO:0046719)
negative regulation of cardiocyte differentiation (GO:1905208)
regulation of RNA biosynthetic process (GO:2001141)
negative regulation of T cell mediated immunity (GO:0002710)
positive regulation of RIG-I signaling pathway (GO:1900246)
protection from non-homologous end joining at telomere (GO:0031848)

pericardium morphogenesis (GO:0003344)
purine ribonucleotide transport (GO:0015868)
female genitalia development (GO:0030540)
regulation of toll-like receptor 3 signaling pathway (GO:0034139)
activation of signaling protein activity involved in unfolded protein response (GO:0006987)
regulation of establishment of protein localization (GO:0070201)
cellular response to histamine (GO:0071420)
dorsal aorta morphogenesis (GO:0035912)
hyaluronan biosynthetic process (GO:0030213)
epithelial cell differentiation involved in kidney development (GO:0035850)
negative regulation of nitric oxide biosynthetic process (GO:0045019)
molybdopterin cofactor biosynthetic process (GO:0032324)
noradrenergic neuron differentiation (GO:0003357)
positive regulation of isomerase activity (GO:0010912)
negative regulation of T cell cytokine production (GO:0002725)
lens fiber cell differentiation (GO:0070306)
regulation of microtubule binding (GO:1904526)
negative regulation of glycoprotein biosynthetic process (GO:0010561)
regulation of RNA polymerase II regulatory region sequence-specific DNA binding (GO:1903025)
positive regulation of type I interferon-mediated signaling pathway (GO:0060340)
negative regulation of RIG-I signaling pathway (GO:0039536)
kidney epithelium development (GO:0072073)
regulation of protein localization to centrosome (GO:1904779)
negative regulation of cardiac muscle tissue development (GO:0055026)
excitatory chemical synaptic transmission (GO:0098976)
chitin catabolic process (GO:0006032)
store-operated calcium entry (GO:0002115)
T-helper 1 type immune response (GO:0042088)
long term synaptic depression (GO:0060292)
regulation of transcription involved in cell fate commitment (GO:0060850)
metanephric nephron tubule development (GO:0072234)
regulation of systemic arterial blood pressure by renin-angiotensin (GO:0003081)
cellular response to cholesterol (GO:0071397)
negative regulation of heart rate (GO:0010459)
marginal zone B cell differentiation (GO:0002315)
selenocysteine incorporation (GO:0001514)
cellular phosphate ion homeostasis (GO:0030643)
hyperosmotic response (GO:0006972)
regulation of cellular response to insulin stimulus (GO:1900076)
positive regulation of neurological system process (GO:0031646)
chondroitin sulfate proteoglycan metabolic process (GO:0050654)
regulation of Cdc42 protein signal transduction (GO:0032489)
regulation of cyclic nucleotide metabolic process (GO:0030799)
cardiac muscle cell-cardiac muscle cell adhesion (GO:0086042)
cardiac atrium development (GO:0003230)
cellular divalent inorganic anion homeostasis (GO:0072501)
negative regulation of production of miRNAs involved in gene silencing by miRNA (GO:1903799)
regulation of hormone biosynthetic process (GO:0046885)
exonucleolytic trimming to generate mature 3'-end of 5.8S rRNA from tricistronic rRNA transcript (SSU-rRNA, 5.
negative regulation of leukocyte migration (GO:0002686)
positive regulation of metaphase/anaphase transition of cell cycle (GO:1902101)
cellular trivalent inorganic anion homeostasis (GO:0072502)
exonucleolytic trimming involved in rRNA processing (GO:0000459)

brown fat cell differentiation (GO:0050873)
regulation of endocytic recycling (GO:2001135)
regulation of dendritic spine maintenance (GO:1902950)
regulation of MDA-5 signaling pathway (GO:0039533)
oxygen transport (GO:0015671)
dorsal aorta development (GO:0035907)
positive regulation of epithelial cell differentiation involved in kidney development (GO:2000698)
regulation of endothelial cell chemotaxis to fibroblast growth factor (GO:2000544)
melanocyte differentiation (GO:0030318)
vesicle transport along actin filament (GO:0030050)
regulation of low-density lipoprotein particle clearance (GO:0010988)
nucleoside diphosphate metabolic process (GO:0009132)
posttranslational protein targeting to endoplasmic reticulum membrane (GO:0006620)
retrograde axonal transport (GO:0008090)
negative regulation of osteoblast proliferation (GO:0033689)
positive regulation of autophagy of mitochondrion (GO:1903599)
negative regulation of translation (GO:0017148)
Wnt signaling pathway, planar cell polarity pathway (GO:0060071)
spliceosomal snRNP assembly (GO:0000387)
regulation of DNA repair (GO:0006282)
positive regulation of receptor activity (GO:2000273)
regulation of dendrite development (GO:0050773)
negative regulation of Ras protein signal transduction (GO:0046580)
nucleotide-excision repair, DNA incision, 5'-to lesion (GO:0006296)
protein peptidyl-prolyl isomerization (GO:0000413)
regulation of regulated secretory pathway (GO:1903305)
positive regulation of MAP kinase activity (GO:0043406)
response to insulin (GO:0032868)
DNA-templated transcription, termination (GO:0006353)
establishment of protein localization to vacuole (GO:0072666)
positive regulation of protein targeting to membrane (GO:0090314)
regulation of DNA biosynthetic process (GO:2000278)
DNA catabolic process, endonucleolytic (GO:0000737)
cytosolic calcium ion transport (GO:0060401)
positive regulation of erythrocyte differentiation (GO:0045648)
negative regulation of sprouting angiogenesis (GO:1903671)
protein localization to cell surface (GO:0034394)
phosphatidylglycerol metabolic process (GO:0046471)
branched-chain amino acid metabolic process (GO:0009081)
regulation of membrane protein ectodomain proteolysis (GO:0051043)
positive regulation of biosynthetic process (GO:0009891)
regulation of ERBB signaling pathway (GO:1901184)
viral genome replication (GO:0019079)
inner ear morphogenesis (GO:0042472)
negative regulation of nervous system development (GO:0051961)
regulation of bone resorption (GO:0045124)
regulation of insulin secretion involved in cellular response to glucose stimulus (GO:0061178)
regulation of interferon-gamma-mediated signaling pathway (GO:0060334)
hematopoietic progenitor cell differentiation (GO:0002244)
cellular response to nutrient levels (GO:0031669)
action potential (GO:0001508)
regulation of establishment of planar polarity (GO:0090175)
cellular response to starvation (GO:0009267)

positive regulation of epithelial cell proliferation (GO:0050679)
response to metal ion (GO:0010038)
protein dephosphorylation (GO:0006470)
negative regulation of intracellular signal transduction (GO:1902532)
positive regulation of innate immune response (GO:0045089)
cellular amino acid catabolic process (GO:0009063)
lamellipodium assembly (GO:0030032)
zinc II ion transmembrane transport (GO:0071577)
metanephros development (GO:0001656)
positive regulation of ATP metabolic process (GO:1903580)
cellular response to ammonium ion (GO:0071242)
apoptotic nuclear changes (GO:0030262)
regulation of interleukin-8 secretion (GO:2000482)
cellular response to interleukin-6 (GO:0071354)
rRNA methylation (GO:0031167)
histone H3-K4 methylation (GO:0051568)
positive regulation of cell junction assembly (GO:1901890)
cellular response to catecholamine stimulus (GO:0071870)
neuronal action potential (GO:0019228)
cell-cell adhesion mediated by cadherin (GO:0044331)
regulation of transcription elongation from RNA polymerase II promoter (GO:0034243)
positive regulation of mRNA catabolic process (GO:0061014)
nucleic acid phosphodiester bond hydrolysis (GO:0090305)
cellular response to lipopolysaccharide (GO:0071222)
organonitrogen compound biosynthetic process (GO:1901566)
DNA-dependent DNA replication (GO:0006261)
glycosaminoglycan catabolic process (GO:0006027)
regulation of protein kinase B signaling (GO:0051896)
neuron projection morphogenesis (GO:0048812)
negative regulation of protein kinase activity (GO:0006469)
peptidyl-serine phosphorylation (GO:0018105)
positive regulation of protein kinase B signaling (GO:0051897)
skeletal muscle tissue development (GO:0007519)
mitotic DNA damage checkpoint (GO:0044773)
alpha-amino acid catabolic process (GO:1901606)
regulation of type I interferon-mediated signaling pathway (GO:0060338)
mitotic spindle organization (GO:0007052)
glucose homeostasis (GO:0042593)
mitotic nuclear division (GO:0140014)
cell migration involved in heart development (GO:0060973)
positive regulation of mitochondrial calcium ion concentration (GO:0051561)
interleukin-18-mediated signaling pathway (GO:0035655)
phenol-containing compound biosynthetic process (GO:0046189)
endodermal cell fate commitment (GO:0001711)
negative regulation of lipase activity (GO:0060192)
negative regulation of activated T cell proliferation (GO:0046007)
transepithelial transport (GO:0070633)
imidazole-containing compound catabolic process (GO:0052805)
protein localization to microtubule (GO:0035372)
sequestering of extracellular ligand from receptor (GO:0035581)
cellular response to UV-B (GO:0071493)
positive regulation of ryanodine-sensitive calcium-release channel activity (GO:0060316)
regulation of transcription from RNA polymerase II promoter in response to oxidative stress (GO:0043619)

negative regulation of voltage-gated potassium channel activity (GO:1903817)
regulation of striated muscle cell differentiation (GO:0051153)
central nervous system myelination (GO:0022010)
negative regulation of retrograde protein transport, ER to cytosol (GO:1904153)
deadenylation-dependent decapping of nuclear-transcribed mRNA (GO:0000290)
positive regulation of IRE1-mediated unfolded protein response (GO:1903896)
positive regulation of metanephros development (GO:0072216)
genetic imprinting (GO:0071514)
regulation of metaphase/anaphase transition of cell cycle (GO:1902099)
cellular response to epinephrine stimulus (GO:0071872)
negative regulation of centriole replication (GO:0046600)
histidine catabolic process (GO:0006548)
angiogenesis involved in wound healing (GO:0060055)
protein localization to endosome (GO:0036010)
negative regulation of mRNA polyadenylation (GO:1900364)
amyloid precursor protein catabolic process (GO:0042987)
positive regulation of mRNA 3'-end processing (GO:0031442)
exocrine system development (GO:0035272)
positive regulation of vasculogenesis (GO:2001214)
phospholipid homeostasis (GO:0055091)
regulation of nitrogen compound metabolic process (GO:0051171)
histidine metabolic process (GO:0006547)
alpha-amino acid biosynthetic process (GO:1901607)
positive regulation of mitotic sister chromatid separation (GO:1901970)
phospholipid scrambling (GO:0017121)
cGMP-mediated signaling (GO:0019934)
regulation of cytoplasmic mRNA processing body assembly (GO:0010603)
ventricular trabecula myocardium morphogenesis (GO:0003222)
negative regulation of exocytosis (GO:0045920)
peripheral nervous system axon ensheathment (GO:0032292)
pyrimidine nucleobase metabolic process (GO:0006206)
negative regulation of vascular permeability (GO:0043116)
regulation of smooth muscle cell apoptotic process (GO:0034391)
calcium ion import into cytosol (GO:1902656)
norepinephrine metabolic process (GO:0042415)
regulation of ERAD pathway (GO:1904292)
nucleotide phosphorylation (GO:0046939)
positive regulation of production of miRNAs involved in gene silencing by miRNA (GO:1903800)
bone resorption (GO:0045453)
regulation of cilium movement (GO:0003352)
positive regulation of chromatin silencing (GO:0031937)
calcium ion import across plasma membrane (GO:0098703)
glycerol-3-phosphate metabolic process (GO:0006072)
nose development (GO:0043584)
histone H3-K36 demethylation (GO:0070544)
depyrimidination (GO:0045008)
interleukin-1 beta secretion (GO:0050702)
positive regulation of non-canonical Wnt signaling pathway (GO:2000052)
response to vitamin D (GO:0033280)
cellular monovalent inorganic anion homeostasis (GO:0030320)
positive regulation of smooth muscle cell apoptotic process (GO:0034393)
positive regulation of extracellular matrix assembly (GO:1901203)
cellular response to prostaglandin E stimulus (GO:0071380)

regulation of amyloid precursor protein catabolic process (GO:1902991)
nucleoside diphosphate phosphorylation (GO:0006165)
response to epinephrine (GO:0071871)
myelin maintenance (GO:0043217)
aorta development (GO:0035904)
establishment of integrated proviral latency (GO:0075713)
negative regulation of platelet aggregation (GO:0090331)
peptidyl-arginine methylation, to asymmetrical-dimethyl arginine (GO:0019919)
positive regulation of behavior (GO:0048520)
negative regulation of monooxygenase activity (GO:0032769)
cellular cation homeostasis (GO:0030003)
regulation of PERK-mediated unfolded protein response (GO:1903897)
positive regulation of RNA polymerase II transcriptional preinitiation complex assembly (GO:0045899)
protein desumoylation (GO:0016926)
Schwann cell development (GO:0014044)
cellular response to peptide hormone stimulus (GO:0071375)
regulation of cell growth (GO:0001558)
IRE1-mediated unfolded protein response (GO:0036498)
regulation of Rho protein signal transduction (GO:0035023)
regulation of androgen receptor signaling pathway (GO:0060765)
retinoid metabolic process (GO:0001523)
negative regulation of cellular amide metabolic process (GO:0034249)
cAMP-mediated signaling (GO:0019933)
nucleotide-excision repair, DNA incision (GO:0033683)
cognition (GO:0050890)
positive regulation of mitotic nuclear division (GO:0045840)
peptidyl-proline modification (GO:0018208)
JAK-STAT cascade (GO:0007259)
regulation of protein binding (GO:0043393)
mitotic cell cycle phase transition (GO:0044772)
phospholipid transport (GO:0015914)
regulation of epidermal growth factor receptor signaling pathway (GO:0042058)
sodium ion transport (GO:0006814)
negative regulation of programmed cell death (GO:0043069)
cellular response to fibroblast growth factor stimulus (GO:0044344)
mitochondrion organization (GO:0007005)
odontogenesis (GO:0042476)
bicarbonate transport (GO:0015701)
positive regulation of receptor-mediated endocytosis (GO:0048260)
regulation of G2/M transition of mitotic cell cycle (GO:0010389)
positive regulation of purine nucleotide biosynthetic process (GO:1900373)
macromolecule modification (GO:0043412)
receptor catabolic process (GO:0032801)
neuromuscular synaptic transmission (GO:0007274)
sensory perception of pain (GO:0019233)
negative regulation of oxidative stress-induced cell death (GO:1903202)
cellular senescence (GO:0090398)
negative regulation of endothelial cell apoptotic process (GO:2000352)
positive regulation of dephosphorylation (GO:0035306)
positive regulation of vascular endothelial growth factor production (GO:0010575)
chaperone mediated protein folding requiring cofactor (GO:0051085)
actin nucleation (GO:0045010)
regulation of cardiac muscle cell action potential (GO:0098901)

cytoplasmic pattern recognition receptor signaling pathway (GO:0002753)
response to glucocorticoid (GO:0051384)
ear morphogenesis (GO:0042471)
histone mRNA metabolic process (GO:0008334)
negative regulation of circadian rhythm (GO:0042754)
inositol metabolic process (GO:0006020)
secretory granule organization (GO:0033363)
alanine transport (GO:0032328)
negative regulation of cytokine production involved in inflammatory response (GO:1900016)
regulation of protein acetylation (GO:1901983)
positive regulation of production of molecular mediator of immune response (GO:0002702)
positive regulation of voltage-gated potassium channel activity (GO:1903818)
regulation of interleukin-6 biosynthetic process (GO:0045408)
lactation (GO:0007595)
negative regulation of pri-miRNA transcription from RNA polymerase II promoter (GO:1902894)
positive regulation of mitotic metaphase/anaphase transition (GO:0045842)
potassium ion export (GO:0071435)
embryonic forelimb morphogenesis (GO:0035115)
mitochondrial respiratory chain complex III assembly (GO:0034551)
phagolysosome assembly (GO:0001845)
nuclear migration (GO:0007097)
adipose tissue development (GO:0060612)
Cdc42 protein signal transduction (GO:0032488)
negative regulation of tyrosine phosphorylation of STAT protein (GO:0042532)
small nucleolar ribonucleoprotein complex assembly (GO:0000491)
peptidyl-arginine omega-N-methylation (GO:0035247)
positive regulation of leukocyte apoptotic process (GO:2000108)
response to dopamine (GO:1903350)
negative regulation of protein exit from endoplasmic reticulum (GO:0070862)
establishment of viral latency (GO:0019043)
myelin assembly (GO:0032288)
response to leucine (GO:0043201)
regulation of skeletal muscle cell differentiation (GO:2001014)
nucleoside phosphate biosynthetic process (GO:1901293)
respiratory chain complex III assembly (GO:0017062)
columnar/cuboidal epithelial cell differentiation (GO:0002065)
interleukin-1 secretion (GO:0050701)
peptidyl-amino acid modification (GO:0018193)
positive regulation of autophagosome assembly (GO:2000786)
receptor recycling (GO:0001881)
regulation of protein ADP-ribosylation (GO:0010835)
podosome assembly (GO:0071800)
nucleoside triphosphate biosynthetic process (GO:0009142)
negative regulation of activin receptor signaling pathway (GO:0032926)
peptidyl-arginine methylation (GO:0018216)
mitotic DNA replication checkpoint (GO:0033314)
positive regulation of ribosome biogenesis (GO:0090070)
negative regulation of protein sumoylation (GO:0033234)
atrioventricular valve development (GO:0003171)
regulation of sequestering of calcium ion (GO:0051282)
regulation of bile acid biosynthetic process (GO:0070857)
positive regulation of rRNA processing (GO:2000234)
cellular response to superoxide (GO:0071451)

postsynaptic density organization (GO:0097106)
regulation of cell-cell adhesion mediated by cadherin (GO:2000047)
regulation of leukocyte activation (GO:0002694)
regulation of leukocyte degranulation (GO:0043300)
glucose import (GO:0046323)
ether lipid metabolic process (GO:0046485)
negative regulation of chromatin silencing (GO:0031936)
cellular response to leucine (GO:0071233)
negative regulation of lipid localization (GO:1905953)
regulation of cell communication by electrical coupling involved in cardiac conduction (GO:1901844)
regulation of homotypic cell-cell adhesion (GO:0034110)
T cell mediated immunity (GO:0002456)
proline transport (GO:0015824)
muscle cell fate commitment (GO:0042693)
positive regulation of protein homooligomerization (GO:0032464)
cellular response to acidic pH (GO:0071468)
negative regulation of microtubule polymerization (GO:0031115)
antigen processing and presentation of exogenous peptide antigen via MHC class I, TAP-independent (GO:000[illegible]
detection of mechanical stimulus involved in sensory perception (GO:0050974)
mitochondrial fragmentation involved in apoptotic process (GO:0043653)
tRNA wobble uridine modification (GO:0002098)
cytoskeletal anchoring at plasma membrane (GO:0007016)
negative regulation of DNA-templated transcription, elongation (GO:0032785)
Golgi inheritance (GO:0048313)
regulation of glycoprotein biosynthetic process (GO:0010559)
histone H3-K4 trimethylation (GO:0080182)
removal of superoxide radicals (GO:0019430)
small RNA loading onto RISC (GO:0070922)
negative regulation of leukocyte chemotaxis (GO:0002689)
acidic amino acid transport (GO:0015800)
positive regulation of protein sumoylation (GO:0033235)
negative regulation of response to reactive oxygen species (GO:1901032)
actin filament reorganization (GO:0090527)
neuronal ion channel clustering (GO:0045161)
microtubule anchoring at centrosome (GO:0034454)
regulation of interferon-gamma secretion (GO:1902713)
regulation of endothelial cell differentiation (GO:0045601)
positive regulation of mitochondrial translation (GO:0070131)
regulation of mast cell activation involved in immune response (GO:0033006)
mature B cell differentiation involved in immune response (GO:0002313)
ionotropic glutamate receptor signaling pathway (GO:0035235)
neurotrophin TRK receptor signaling pathway (GO:0048011)
negative regulation of mRNA 3'-end processing (GO:0031441)
interleukin-1 beta production (GO:0032611)
positive regulation of ion transmembrane transport (GO:0034767)
negative regulation of protein secretion (GO:0050709)
epithelial cell differentiation (GO:0030855)
regulation of GTPase activity (GO:0043087)
fibroblast growth factor receptor signaling pathway (GO:0008543)
regulation of transcription from RNA polymerase II promoter in response to hypoxia (GO:0061418)
post-translational protein modification (GO:0043687)
heterotypic cell-cell adhesion (GO:0034113)
regulation of spindle assembly (GO:0090169)

regulation of telomere maintenance (GO:0032204)
lamellipodium organization (GO:0097581)
regulation of protein ubiquitination involved in ubiquitin-dependent protein catabolic process (GO:2000058)
regulation of DNA damage response, signal transduction by p53 class mediator (GO:0043516)
modulation of excitatory postsynaptic potential (GO:0098815)
female gamete generation (GO:0007292)
positive regulation of embryonic development (GO:0040019)
regulation of cellular protein localization (GO:1903827)
L-amino acid transport (GO:0015807)
positive regulation of nitric oxide biosynthetic process (GO:0045429)
positive regulation of nitric oxide metabolic process (GO:1904407)
nucleotide-binding domain, leucine rich repeat containing receptor signaling pathway (GO:0035872)
cyclic nucleotide metabolic process (GO:0009187)
misfolded or incompletely synthesized protein catabolic process (GO:0006515)
peptide catabolic process (GO:0043171)
protein stabilization (GO:0050821)
antigen processing and presentation of exogenous peptide antigen (GO:0002478)
antigen processing and presentation of exogenous peptide antigen via MHC class II (GO:0019886)
adenylate cyclase-modulating G-protein coupled receptor signaling pathway (GO:0007188)
regulation of B cell proliferation (GO:0030888)
development of primary male sexual characteristics (GO:0046546)
response to unfolded protein (GO:0006986)
histone H3 acetylation (GO:0043966)
nucleobase-containing compound catabolic process (GO:0034655)
male gonad development (GO:0008584)
RNA catabolic process (GO:0006401)
protein homooligomerization (GO:0051260)
positive regulation of leukocyte chemotaxis (GO:0002690)
RNA 3'-end processing (GO:0031123)
striated muscle contraction (GO:0006941)
regulation of tyrosine phosphorylation of STAT protein (GO:0042509)
regulation of macroautophagy (GO:0016241)
antigen processing and presentation of peptide antigen via MHC class II (GO:0002495)
cellular response to organic cyclic compound (GO:0071407)
nucleic acid metabolic process (GO:0090304)
regulation of nucleic acid-templated transcription (GO:1903506)
protein localization to cell periphery (GO:1990778)
extracellular matrix organization (GO:0030198)
positive regulation of synapse assembly (GO:0051965)
regulation of transcription regulatory region DNA binding (GO:2000677)
negative chemotaxis (GO:0050919)
positive regulation of cAMP metabolic process (GO:0030816)
regulation of microtubule-based process (GO:0032886)
regulation of histone acetylation (GO:0035065)
phosphatidylethanolamine acyl-chain remodeling (GO:0036152)
endocrine system development (GO:0035270)
positive regulation of glucose import (GO:0046326)
diterpenoid metabolic process (GO:0016101)
DNA damage response, signal transduction by p53 class mediator resulting in cell cycle arrest (GO:0006977)
establishment of protein localization to organelle (GO:0072594)
negative regulation of viral-induced cytoplasmic pattern recognition receptor signaling pathway (GO:0039532)
regulation of response to stress (GO:0080134)
glomerular filtration (GO:0003094)

regulation of chromatin organization (GO:1902275)
thyroid gland development (GO:0030878)
3'-UTR-mediated mRNA destabilization (GO:0061158)
regulation of sequestering of zinc ion (GO:0061088)
negative regulation of oxidative stress-induced neuron death (GO:1903204)
positive regulation of cytoplasmic transport (GO:1903651)
chloride ion homeostasis (GO:0055064)
regulation of Arp2/3 complex-mediated actin nucleation (GO:0034315)
mitotic nuclear envelope reassembly (GO:0007084)
nerve growth factor signaling pathway (GO:0038180)
embryonic digestive tract morphogenesis (GO:0048557)
hair cell differentiation (GO:0035315)
positive regulation of glucose import in response to insulin stimulus (GO:2001275)
glutamine metabolic process (GO:0006541)
regulation of protein exit from endoplasmic reticulum (GO:0070861)
regulation of amyloid-beta clearance (GO:1900221)
base-excision repair, AP site formation (GO:0006285)
positive regulation of lipase activity (GO:0060193)
androgen biosynthetic process (GO:0006702)
cell differentiation involved in metanephros development (GO:0072202)
neurotransmitter receptor transport (GO:0099637)
mitotic G1 DNA damage checkpoint (GO:0031571)
negative regulation of gene silencing by miRNA (GO:0060965)
regulation of granulocyte differentiation (GO:0030852)
glycoprotein catabolic process (GO:0006516)
telomere maintenance in response to DNA damage (GO:0043247)
G1 DNA damage checkpoint (GO:0044783)
protein localization to synapse (GO:0035418)
nucleoside metabolic process (GO:0009116)
central nervous system projection neuron axonogenesis (GO:0021952)
positive regulation of natural killer cell mediated immune response to tumor cell (GO:0002857)
mitochondrial respiratory chain complex III biogenesis (GO:0097033)
myeloid dendritic cell differentiation (GO:0043011)
insulin-like growth factor receptor signaling pathway (GO:0048009)
positive regulation of circadian rhythm (GO:0042753)
IRES-dependent viral translational initiation (GO:0075522)
regulation of DNA damage checkpoint (GO:2000001)
positive regulation of smooth muscle cell migration (GO:0014911)
negative regulation of centrosome duplication (GO:0010826)
negative regulation of stem cell differentiation (GO:2000737)
actin filament-based transport (GO:0099515)
regulation of phospholipase activity (GO:0010517)
tRNA wobble base modification (GO:0002097)
negative regulation of sodium ion transmembrane transporter activity (GO:2000650)
stress fiber assembly (GO:0043149)
regulation of mRNA 3'-end processing (GO:0031440)
mitotic G1/S transition checkpoint (GO:0044819)
cellular response to leucine starvation (GO:1990253)
negative regulation of homotypic cell-cell adhesion (GO:0034111)
negative regulation of endoplasmic reticulum unfolded protein response (GO:1900102)
endothelial tube morphogenesis (GO:0061154)
response to histamine (GO:0034776)
medium-chain fatty acid metabolic process (GO:0051791)

positive regulation of vacuole organization (GO:0044090)
regulation of dendritic cell apoptotic process (GO:2000668)
histone H2B ubiquitination (GO:0033523)
positive regulation of nuclease activity (GO:0032075)
pulmonary valve development (GO:0003177)
smooth muscle cell differentiation (GO:0051145)
thyroid hormone generation (GO:0006590)
regulation of B cell apoptotic process (GO:0002902)
low-density lipoprotein particle receptor catabolic process (GO:0032802)
adrenal gland development (GO:0030325)
peptidyl-serine dephosphorylation (GO:0070262)
negative regulation of sodium ion transmembrane transport (GO:1902306)
amino sugar metabolic process (GO:0006040)
regulation of myeloid leukocyte differentiation (GO:0002761)
glucosamine-containing compound catabolic process (GO:1901072)
regulation of cellular respiration (GO:0043457)
regulation of actin filament length (GO:0030832)
regulation of attachment of spindle microtubules to kinetochore (GO:0051988)
phosphatidylglycerol biosynthetic process (GO:0006655)
presynaptic membrane assembly (GO:0097105)
positive regulation of alcohol biosynthetic process (GO:1902932)
response to acidic pH (GO:0010447)
contractile actin filament bundle assembly (GO:0030038)
regulation of mast cell activation (GO:0033003)
positive regulation of natural killer cell mediated cytotoxicity directed against tumor cell target (GO:0002860)
L-glutamate transport (GO:0015813)
cellular response to dopamine (GO:1903351)
regulation of actin filament-based movement (GO:1903115)
mesenchymal to epithelial transition (GO:0060231)
negative regulation of inclusion body assembly (GO:0090084)
regulation of transforming growth factor beta production (GO:0071634)
negative regulation of immune response (GO:0050777)
nuclear-transcribed mRNA catabolic process (GO:0000956)
oligosaccharide metabolic process (GO:0009311)
signal transduction involved in mitotic G1 DNA damage checkpoint (GO:0072431)
synapse assembly (GO:0007416)
positive regulation of blood vessel endothelial cell migration (GO:0043536)
protein complex subunit organization (GO:0071822)
mRNA polyadenylation (GO:0006378)
phosphatidylinositol phosphorylation (GO:0046854)
modulation by host of viral transcription (GO:0043921)
regulation of protein localization to nucleus (GO:1900180)
negative regulation of osteoblast differentiation (GO:0045668)
extrinsic apoptotic signaling pathway via death domain receptors (GO:0008625)
regulation of protein transport (GO:0051223)
regulation of protein targeting to membrane (GO:0090313)
regulation of vascular endothelial growth factor production (GO:0010574)
regulation of autophagy of mitochondrion (GO:1903146)
regulation of protein localization to cell surface (GO:2000008)
telomere maintenance via telomere lengthening (GO:0010833)
negative regulation of peptidyl-tyrosine phosphorylation (GO:0050732)
leukocyte cell-cell adhesion (GO:0007159)
negative regulation of microtubule polymerization or depolymerization (GO:0031111)

cardiac muscle cell development (GO:0055013)
regulation of vesicle-mediated transport (GO:0060627)
protein processing (GO:0016485)
intrinsic apoptotic signaling pathway (GO:0097193)
activation of protein kinase activity (GO:0032147)
intracellular protein transport (GO:0006886)
positive regulation of JAK-STAT cascade (GO:0046427)
DNA replication (GO:0006260)
positive regulation of sequence-specific DNA binding transcription factor activity (GO:0051091)
mitotic spindle assembly (GO:0090307)
aminoglycan biosynthetic process (GO:0006023)
nucleocytoplasmic transport (GO:0006913)
positive regulation of posttranscriptional gene silencing (GO:0060148)
atrial septum morphogenesis (GO:0060413)
replicative senescence (GO:0090399)
regulation of myeloid leukocyte mediated immunity (GO:0002886)
positive regulation of cell cycle phase transition (GO:1901989)
regulation of feeding behavior (GO:0060259)
mononuclear cell differentiation (GO:1903131)
positive regulation by host of viral process (GO:0044794)
gas transport (GO:0015669)
immune response-regulating cell surface receptor signaling pathway (GO:0002768)
positive regulation of nuclear-transcribed mRNA poly(A) tail shortening (GO:0060213)
calcineurin-NFAT signaling cascade (GO:0033173)
modulation by host of viral process (GO:0044788)
urea cycle (GO:0000050)
brain morphogenesis (GO:0048854)
regulation of mitotic centrosome separation (GO:0046602)
positive regulation of histone deacetylation (GO:0031065)
purine nucleobase metabolic process (GO:0006144)
regulation of integrin-mediated signaling pathway (GO:2001044)
phosphatidylinositol dephosphorylation (GO:0046856)
rRNA base methylation (GO:0070475)
lymphocyte mediated immunity (GO:0002449)
negative regulation of myotube differentiation (GO:0010832)
monocyte differentiation (GO:0030224)
renal filtration (GO:0097205)
regulation of short-term neuronal synaptic plasticity (GO:0048172)
negative regulation of cellular senescence (GO:2000773)
regulation of natural killer cell mediated cytotoxicity directed against tumor cell target (GO:0002858)
regulation of nuclear-transcribed mRNA poly(A) tail shortening (GO:0060211)
cellular response to interleukin-2 (GO:0071352)
cochlea morphogenesis (GO:0090103)
response to sterol (GO:0036314)
cytoplasmic mRNA processing body assembly (GO:0033962)
histone mRNA catabolic process (GO:0071044)
regulation of RNA polymerase II transcriptional preinitiation complex assembly (GO:0045898)
icosanoid biosynthetic process (GO:0046456)
progesterone metabolic process (GO:0042448)
cell part morphogenesis (GO:0032990)
negative regulation of potassium ion transport (GO:0043267)
fatty acid transmembrane transport (GO:1902001)
protein localization to mitochondrion (GO:0070585)

negative regulation of heart contraction (GO:0045822)
regulation of synaptic vesicle cycle (GO:0098693)
modulation by host of viral genome replication (GO:0044827)
angiotensin maturation (GO:0002003)
negative regulation of chemotaxis (GO:0050922)
establishment of synaptic vesicle localization (GO:0097480)
cellular response to increased oxygen levels (GO:0036295)
retinal metabolic process (GO:0042574)
positive regulation of fatty acid oxidation (GO:0046321)
inhibition of cysteine-type endopeptidase activity involved in apoptotic process (GO:1990001)
positive regulation of sodium ion transmembrane transporter activity (GO:2000651)
low-density lipoprotein receptor particle metabolic process (GO:0032799)
negative regulation of release of sequestered calcium ion into cytosol (GO:0051280)
negative regulation of translation, ncRNA-mediated (GO:0040033)
regulation of cardiac muscle cell differentiation (GO:2000725)
regulation of receptor biosynthetic process (GO:0010869)
presynaptic membrane organization (GO:0097090)
miRNA mediated inhibition of translation (GO:0035278)
protein carboxylation (GO:0018214)
regulation of chromatin silencing (GO:0031935)
regulation of mRNA polyadenylation (GO:1900363)
peptidyl-glutamic acid carboxylation (GO:0017187)
cardiac muscle cell membrane repolarization (GO:0099622)
regulation of stem cell population maintenance (GO:2000036)
microtubule anchoring at microtubule organizing center (GO:0072393)
negative regulation of blood vessel diameter (GO:0097756)
regulation of histone deacetylation (GO:0031063)
regulation of lipid kinase activity (GO:0043550)
negative regulation of protein localization to nucleus (GO:1900181)
positive regulation of superoxide anion generation (GO:0032930)
regulation of hydrogen peroxide-induced cell death (GO:1903205)
negative regulation of protein ubiquitination involved in ubiquitin-dependent protein catabolic process (GO:2000
protein localization to pre-autophagosomal structure (GO:0034497)
negative regulation of bone mineralization (GO:0030502)
regulation of vascular endothelial growth factor signaling pathway (GO:1900746)
carnitine shuttle (GO:0006853)
Golgi ribbon formation (GO:0090161)
proteasome assembly (GO:0043248)
cellular response to sterol (GO:0036315)
intrinsic apoptotic signaling pathway in response to oxidative stress (GO:0008631)
positive regulation of tumor necrosis factor secretion (GO:1904469)
internal protein amino acid acetylation (GO:0006475)
positive regulation of myoblast fusion (GO:1901741)
inorganic cation transmembrane transport (GO:0098662)
negative regulation of sequence-specific DNA binding transcription factor activity (GO:0043433)
positive regulation of cellular biosynthetic process (GO:0031328)
negative regulation of cell adhesion (GO:0007162)
regulation of TORC1 signaling (GO:1903432)
positive regulation of cell communication (GO:0010647)
regulation of defense response to virus by virus (GO:0050690)
positive regulation of lipid biosynthetic process (GO:0046889)
nucleotide-binding oligomerization domain containing signaling pathway (GO:0070423)
regulation of actin polymerization or depolymerization (GO:0008064)

transcription elongation from RNA polymerase I promoter (GO:0006362)
RNA polyadenylation (GO:0043631)
cellular response to hydrogen peroxide (GO:0070301)
positive regulation of phosphate metabolic process (GO:0045937)
activation of GTPase activity (GO:0090630)
cellular response to molecule of bacterial origin (GO:0071219)
protein localization to membrane (GO:0072657)
positive regulation of organelle assembly (GO:1902117)
positive regulation of gene expression, epigenetic (GO:0045815)
sphingolipid biosynthetic process (GO:0030148)
ER to Golgi vesicle-mediated transport (GO:0006888)
DNA metabolic process (GO:0006259)
positive regulation of macromolecule metabolic process (GO:0010604)
regulation of striated muscle contraction (GO:0006942)
regulation of vasoconstriction (GO:0019229)
positive regulation of myeloid cell differentiation (GO:0045639)
positive regulation of histone modification (GO:0031058)
regulation of neutrophil chemotaxis (GO:0090022)
negative regulation of lymphocyte proliferation (GO:0050672)
regulation of chromosome segregation (GO:0051983)
regulation of keratinocyte differentiation (GO:0045616)
negative regulation of epithelial cell apoptotic process (GO:1904036)
nuclear-transcribed mRNA poly(A) tail shortening (GO:0000289)
synaptic vesicle recycling (GO:0036465)
positive regulation of epidermal growth factor receptor signaling pathway (GO:0045742)
skeletal muscle organ development (GO:0060538)
respiratory system development (GO:0060541)
striated muscle tissue development (GO:0014706)
regulation of macrophage derived foam cell differentiation (GO:0010743)
ion transmembrane transport (GO:0034220)
regulation of mitochondrion organization (GO:0010821)
intrinsic apoptotic signaling pathway in response to DNA damage (GO:0008630)
regulation of cell cycle arrest (GO:0071156)
viral process (GO:0016032)
pulmonary valve morphogenesis (GO:0003184)
regulation of systemic arterial blood pressure (GO:0003073)
regulation of myoblast fusion (GO:1901739)
negative regulation of chondrocyte differentiation (GO:0032331)
negative regulation of ryanodine-sensitive calcium-release channel activity (GO:0060315)
interleukin-2-mediated signaling pathway (GO:0038110)
phospholipid efflux (GO:0033700)
regulation of rRNA processing (GO:2000232)
mitochondrion transport along microtubule (GO:0047497)
response to axon injury (GO:0048678)
alternative mRNA splicing, via spliceosome (GO:0000380)
negative regulation of substrate adhesion-dependent cell spreading (GO:1900025)
exit from mitosis (GO:0010458)
positive regulation of cardiocyte differentiation (GO:1905209)
regulation of cell communication by electrical coupling (GO:0010649)
positive regulation of ruffle assembly (GO:1900029)
histone H3 deacetylation (GO:0070932)
regulation of protein localization to cell periphery (GO:1904375)
tRNA 5'-leader removal (GO:0001682)

negative regulation by host of viral transcription (GO:0043922)
positive regulation of pseudopodium assembly (GO:0031274)
cellular response to fluid shear stress (GO:0071498)
cytoplasmic translational initiation (GO:0002183)
lysine metabolic process (GO:0006553)
regulation of pseudopodium assembly (GO:0031272)
purine nucleoside monophosphate biosynthetic process (GO:0009127)
collagen metabolic process (GO:0032963)
positive regulation of endoplasmic reticulum unfolded protein response (GO:1900103)
regulation of angiotensin levels in blood (GO:0002002)
negative regulation of DNA damage response, signal transduction by p53 class mediator (GO:0043518)
skeletal muscle cell differentiation (GO:0035914)
positive regulation of cyclase activity (GO:0031281)
transcription from mitochondrial promoter (GO:0006390)
prostanoid metabolic process (GO:0006692)
regulation of T cell differentiation in thymus (GO:0033081)
pyrimidine deoxyribonucleotide catabolic process (GO:0009223)
endosome transport via multivesicular body sorting pathway (GO:0032509)
positive regulation of mRNA metabolic process (GO:1903313)
negative regulation of interleukin-12 production (GO:0032695)
keratan sulfate catabolic process (GO:0042340)
regulation of sister chromatid cohesion (GO:0007063)
membrane repolarization during action potential (GO:0086011)
positive regulation of vascular endothelial growth factor receptor signaling pathway (GO:0030949)
positive regulation of neuron migration (GO:2001224)
positive regulation of T cell cytokine production (GO:0002726)
calcineurin-mediated signaling (GO:0097720)
microtubule anchoring (GO:0034453)
positive regulation of SMAD protein import into nucleus (GO:0060391)
eye photoreceptor cell differentiation (GO:0001754)
rRNA 3'-end processing (GO:0031125)
positive regulation of lyase activity (GO:0051349)
cardiac right ventricle morphogenesis (GO:0003215)
regulation of vasculogenesis (GO:2001212)
signal transduction involved in G2 DNA damage checkpoint (GO:0072425)
histone H3-K9 demethylation (GO:0033169)
negative regulation of protein oligomerization (GO:0032460)
protein localization to kinetochore (GO:0034501)
regulation of protein glycosylation (GO:0060049)
retinol metabolic process (GO:0042572)
negative regulation of ERAD pathway (GO:1904293)
protein insertion into membrane (GO:0051205)
entry of bacterium into host cell (GO:0035635)
Golgi localization (GO:0051645)
regulation of cholesterol storage (GO:0010885)
endochondral bone morphogenesis (GO:0060350)
epithelial structure maintenance (GO:0010669)
histone arginine methylation (GO:0034969)
regulation of actomyosin structure organization (GO:0110020)
skeletal muscle fiber development (GO:0048741)
lysine catabolic process (GO:0006554)
regulation of histone methylation (GO:0031060)
pre-mRNA cleavage required for polyadenylation (GO:0098789)

cellular protein complex assembly (GO:0043623)
COPII-coated vesicle budding (GO:0090114)
bile acid and bile salt transport (GO:0015721)
cellular response to drug (GO:0035690)
activation of protein kinase B activity (GO:0032148)
activation of JUN kinase activity (GO:0007257)
'de novo' posttranslational protein folding (GO:0051084)
positive regulation of protein insertion into mitochondrial membrane involved in apoptotic signaling pathway (GC
regulation of protein polymerization (GO:0032271)
CENP-A containing nucleosome assembly (GO:0034080)
CENP-A containing chromatin organization (GO:0061641)
negative regulation of cell morphogenesis involved in differentiation (GO:0010771)
positive regulation of reactive oxygen species biosynthetic process (GO:1903428)
regulation of protein insertion into mitochondrial membrane involved in apoptotic signaling pathway (GO:19007[illegible]
negative regulation of endothelial cell proliferation (GO:0001937)
hippo signaling (GO:0035329)
negative regulation of transferase activity (GO:0051348)
gonad development (GO:0008406)
cytoskeleton organization (GO:0007010)
regulation of autophagy (GO:0010506)
regulation of response to external stimulus (GO:0032101)
positive regulation of protein complex assembly (GO:0031334)
regulation of stem cell differentiation (GO:2000736)
negative regulation of cell cycle G2/M phase transition (GO:1902750)
wound healing (GO:0042060)
negative regulation of viral genome replication (GO:0045071)
heart morphogenesis (GO:0003007)
secondary alcohol metabolic process (GO:1902652)
skeletal system morphogenesis (GO:0048705)
termination of RNA polymerase I transcription (GO:0006363)
negative regulation of blood vessel endothelial cell migration (GO:0043537)
regulation of glucose import (GO:0046324)
positive regulation of protein dephosphorylation (GO:0035307)
positive regulation of histone H3-K4 methylation (GO:0051571)
signal transduction involved in DNA damage checkpoint (GO:0072422)
regulation of plasma membrane bounded cell projection organization (GO:0120035)
mitochondrial genome maintenance (GO:0000002)
regulation of glucose import in response to insulin stimulus (GO:2001273)
negative regulation of epidermal growth factor-activated receptor activity (GO:0007175)
myelination in peripheral nervous system (GO:0022011)
positive regulation of cholesterol efflux (GO:0010875)
positive regulation of endothelial cell chemotaxis (GO:2001028)
positive regulation of protein complex disassembly (GO:0043243)
regulation of macrophage differentiation (GO:0045649)
copper ion homeostasis (GO:0055070)
response to UV-B (GO:0010224)
synaptic vesicle localization (GO:0097479)
leukocyte differentiation (GO:0002521)
neuron fate commitment (GO:0048663)
membrane repolarization (GO:0086009)
negative regulation of reactive oxygen species biosynthetic process (GO:1903427)
protein localization to endoplasmic reticulum (GO:0070972)
cellular response to low-density lipoprotein particle stimulus (GO:0071404)

dicarboxylic acid catabolic process (GO:0043649)
cranial nerve development (GO:0021545)
positive regulation of lamellipodium assembly (GO:0010592)
RNA interference (GO:0016246)
secretion (GO:0046903)
mammary gland epithelium development (GO:0061180)
regulation of cAMP-dependent protein kinase activity (GO:2000479)
negative regulation of blood pressure (GO:0045776)
regulation of cytolysis (GO:0042268)
regulation of transcription initiation from RNA polymerase II promoter (GO:0060260)
regulation of fatty acid oxidation (GO:0046320)
negative regulation of cartilage development (GO:0061037)
negative regulation of ubiquitin-protein transferase activity (GO:0051444)
high-density lipoprotein particle assembly (GO:0034380)
amyloid precursor protein metabolic process (GO:0042982)
negative regulation of interleukin-10 production (GO:0032693)
mitotic DNA integrity checkpoint (GO:0044774)
regulation of synaptic vesicle transport (GO:1902803)
pre-miRNA processing (GO:0031054)
regulation of cellular carbohydrate metabolic process (GO:0010675)
response to iron ion (GO:0010039)
nucleotide-sugar metabolic process (GO:0009225)
regulation of lymphocyte activation (GO:0051249)
intestinal absorption (GO:0050892)
regulation of superoxide anion generation (GO:0032928)
regulation of NMDA receptor activity (GO:2000310)
Notch receptor processing, ligand-dependent (GO:0035333)
positive regulation of cellular carbohydrate metabolic process (GO:0010676)
renal absorption (GO:0070293)
viral translation (GO:0019081)
mRNA cleavage involved in mRNA processing (GO:0098787)
regulation of DNA-templated transcription in response to stress (GO:0043620)
regulation of biomineral tissue development (GO:0070167)
regulation of macromolecule biosynthetic process (GO:0010556)
positive regulation of actin nucleation (GO:0051127)
stress-activated protein kinase signaling cascade (GO:0031098)
positive regulation of actin filament polymerization (GO:0030838)
protein tetramerization (GO:0051262)
organelle membrane fusion (GO:0090174)
negative regulation of calcium ion transmembrane transporter activity (GO:1901020)
7-methylguanosine RNA capping (GO:0009452)
regulation of potassium ion transmembrane transport (GO:1901379)
substrate adhesion-dependent cell spreading (GO:0034446)
G2 DNA damage checkpoint (GO:0031572)
7-methylguanosine mRNA capping (GO:0006370)
oxoacid metabolic process (GO:0043436)
chromatin remodeling at centromere (GO:0031055)
ceramide biosynthetic process (GO:0046513)
regulation of ERK1 and ERK2 cascade (GO:0070372)
protein localization to plasma membrane (GO:0072659)
positive regulation of cellular protein metabolic process (GO:0032270)
cellular response to acid chemical (GO:0071229)
protein-DNA complex assembly (GO:0065004)

lipid transport (GO:0006869)
regulation of calcium ion transport (GO:0051924)
regulation of cell-matrix adhesion (GO:0001952)
positive regulation of mitotic cell cycle (GO:0045931)
positive regulation of secretion by cell (GO:1903532)
branching involved in blood vessel morphogenesis (GO:0001569)
nucleoside salvage (GO:0043174)
myeloid dendritic cell activation (GO:0001773)
establishment or maintenance of actin cytoskeleton polarity (GO:0030950)
positive regulation of response to cytokine stimulus (GO:0060760)
modified amino acid transport (GO:0072337)
cell communication by electrical coupling involved in cardiac conduction (GO:0086064)
cell surface receptor signaling pathway involved in heart development (GO:0061311)
pyrimidine-containing compound catabolic process (GO:0072529)
thymus development (GO:0048538)
pancreas development (GO:0031016)
cardiac left ventricle morphogenesis (GO:0003214)
regulation of lymphocyte differentiation (GO:0045619)
positive regulation of mitochondrial fission (GO:0090141)
negative regulation of biomineral tissue development (GO:0070168)
cell migration involved in sprouting angiogenesis (GO:0002042)
smooth muscle contraction (GO:0006939)
response to ethanol (GO:0045471)
cellular response to osmotic stress (GO:0071470)
regulation of fatty acid metabolic process (GO:0019217)
cAMP biosynthetic process (GO:0006171)
positive regulation of DNA damage response, signal transduction by p53 class mediator (GO:0043517)
negative regulation of cell migration involved in sprouting angiogenesis (GO:0090051)
necroptotic process (GO:0070266)
DNA modification (GO:0006304)
regulation of gastrulation (GO:0010470)
nucleoside triphosphate metabolic process (GO:0009141)
positive regulation of heart rate (GO:0010460)
positive regulation of muscle hypertrophy (GO:0014742)
regulation of protein modification by small protein conjugation or removal (GO:1903320)
regulation of interferon-gamma biosynthetic process (GO:0045072)
regulation of mRNA catabolic process (GO:0061013)
positive regulation of interleukin-8 secretion (GO:2000484)
neuron cell-cell adhesion (GO:0007158)
negative regulation of hydrogen peroxide-induced cell death (GO:1903206)
regulation of nuclear-transcribed mRNA catabolic process, deadenylation-dependent decay (GO:1900151)
clathrin coat assembly (GO:0048268)
regulation of activin receptor signaling pathway (GO:0032925)
regulation of nucleocytoplasmic transport (GO:0046822)
mitochondrial electron transport, ubiquinol to cytochrome c (GO:0006122)
negative regulation of Rho protein signal transduction (GO:0035024)
lysosomal transport (GO:0007041)
SCF-dependent proteasomal ubiquitin-dependent protein catabolic process (GO:0031146)
glycoprotein biosynthetic process (GO:0009101)
endosomal transport (GO:0016197)
regulation of T cell differentiation (GO:0045580)
entry into host cell (GO:0030260)
intra-Golgi vesicle-mediated transport (GO:0006891)

regulation of autophagosome assembly (GO:2000785)
cellular response to glucose starvation (GO:0042149)
transcription initiation from RNA polymerase I promoter (GO:0006361)
positive regulation of catalytic activity (GO:0043085)
regulation of DNA recombination (GO:0000018)
transcription from RNA polymerase I promoter (GO:0006360)
negative regulation of multi-organism process (GO:0043901)
regulation of neurotransmitter receptor activity (GO:0099601)
phosphatidylinositol metabolic process (GO:0046488)
positive regulation of tyrosine phosphorylation of STAT protein (GO:0042531)
regulation of protein complex assembly (GO:0043254)
regulation of transcription from RNA polymerase II promoter in response to stress (GO:0043618)
regulation of organelle organization (GO:0033043)
Fc-gamma receptor signaling pathway involved in phagocytosis (GO:0038096)
cell morphogenesis involved in differentiation (GO:0000904)
RNA modification (GO:0009451)
glycosphingolipid metabolic process (GO:0006687)
epithelial tube morphogenesis (GO:0060562)
response to peptide (GO:1901652)
bone development (GO:0060348)
regulation of alcohol biosynthetic process (GO:1902930)
positive regulation of chemokine production (GO:0032722)
response to glucagon (GO:0033762)
ncRNA metabolic process (GO:0034660)
positive regulation of exocytosis (GO:0045921)
regulation of G-protein coupled receptor protein signaling pathway (GO:0008277)
vesicle fusion (GO:0006906)
axon development (GO:0061564)
regulation of mRNA metabolic process (GO:1903311)
catecholamine metabolic process (GO:0006584)
maintenance of protein location in nucleus (GO:0051457)
phosphatidylethanolamine biosynthetic process (GO:0006646)
regulation of protein homooligomerization (GO:0032462)
mitotic sister chromatid cohesion (GO:0007064)
negative regulation of TORC1 signaling (GO:1904262)
positive regulation of CREB transcription factor activity (GO:0032793)
L-alpha-amino acid transmembrane transport (GO:1902475)
G-protein coupled glutamate receptor signaling pathway (GO:0007216)
lipoxygenase pathway (GO:0019372)
anterograde synaptic vesicle transport (GO:0048490)
positive regulation of transcription elongation from RNA polymerase II promoter (GO:0032968)
unsaturated fatty acid biosynthetic process (GO:0006636)
regulation of tumor necrosis factor secretion (GO:1904467)
intracellular lipid transport (GO:0032365)
cardiac muscle cell differentiation (GO:0055007)
glycerophospholipid catabolic process (GO:0046475)
positive regulation of telomerase RNA localization to Cajal body (GO:1904874)
regulation of centriole replication (GO:0046599)
negative regulation of cell aging (GO:0090344)
presynapse assembly (GO:0099054)
N-acetylglucosamine metabolic process (GO:0006044)
negative regulation of interleukin-2 production (GO:0032703)
regulation of clathrin-dependent endocytosis (GO:2000369)

regulation of cell-substrate junction assembly (GO:0090109)
intra-S DNA damage checkpoint (GO:0031573)
fatty acid beta-oxidation using acyl-CoA dehydrogenase (GO:0033539)
neural crest cell differentiation (GO:0014033)
regulation of mitotic sister chromatid separation (GO:0010965)
mesenchymal cell development (GO:0014031)
positive regulation of viral release from host cell (GO:1902188)
vasculature development (GO:0001944)
positive regulation of membrane protein ectodomain proteolysis (GO:0051044)
regulation of macrophage chemotaxis (GO:0010758)
negative regulation of signal transduction by p53 class mediator (GO:1901797)
negative regulation of axon extension involved in axon guidance (GO:0048843)
positive regulation of cellular response to insulin stimulus (GO:1900078)
body fluid secretion (GO:0007589)
negative regulation of platelet activation (GO:0010544)
negative regulation of cytokine production involved in immune response (GO:0002719)
negative regulation of DNA-dependent DNA replication (GO:2000104)
filopodium assembly (GO:0046847)
negative regulation of RNA metabolic process (GO:0051253)
positive regulation of syncytium formation by plasma membrane fusion (GO:0060143)
protein O-linked fucosylation (GO:0036066)
regulation of protein complex disassembly (GO:0043244)
regulation of RNA export from nucleus (GO:0046831)
regulation of lymphocyte proliferation (GO:0050670)
synaptic vesicle transport along microtubule (GO:0099517)
embryonic cranial skeleton morphogenesis (GO:0048701)
positive regulation of protein export from nucleus (GO:0046827)
regulation of peptide secretion (GO:0002791)
endoderm development (GO:0007492)
inhibition of cysteine-type endopeptidase activity (GO:0097340)
establishment of protein localization to endoplasmic reticulum (GO:0072599)
cellular response to corticosteroid stimulus (GO:0071384)
positive regulation of alpha-beta T cell activation (GO:0046635)
membrane repolarization during cardiac muscle cell action potential (GO:0086013)
regulation of mast cell degranulation (GO:0043304)
heparan sulfate proteoglycan biosynthetic process (GO:0015012)
digestive tract morphogenesis (GO:0048546)
positive regulation of response to wounding (GO:1903036)
embryonic axis specification (GO:0000578)
response to X-ray (GO:0010165)
phototransduction, visible light (GO:0007603)
transport along microtubule (GO:0010970)
positive regulation of protein import (GO:1904591)
positive regulation of stress-activated protein kinase signaling cascade (GO:0070304)
protein localization to chromosome, centromeric region (GO:0071459)
positive regulation of cytokine production involved in immune response (GO:0002720)
Fc-gamma receptor signaling pathway (GO:0038094)
peptidyl-lysine modification (GO:0018205)
protein O-linked glycosylation (GO:0006493)
cellular response to oxidative stress (GO:0034599)
regulation of gene silencing by RNA (GO:0060966)
regulation of posttranscriptional gene silencing (GO:0060147)
Fc receptor mediated stimulatory signaling pathway (GO:0002431)

regulation of heart rate by cardiac conduction (GO:0086091)
positive regulation of peptide secretion (GO:0002793)
negative regulation of protein kinase B signaling (GO:0051898)
anatomical structure homeostasis (GO:0060249)
regulation of mitotic spindle organization (GO:0060236)
regulation of synapse assembly (GO:0051963)
positive regulation of ATPase activity (GO:0032781)
membrane protein proteolysis (GO:0033619)
flagellated sperm motility (GO:0030317)
sulfur compound catabolic process (GO:0044273)
lipid phosphorylation (GO:0046834)
sphingolipid metabolic process (GO:0006665)
positive regulation of peptidyl-tyrosine phosphorylation (GO:0050731)
regulation of cell death (GO:0010941)
chordate embryonic development (GO:0043009)
organonitrogen compound catabolic process (GO:1901565)
positive regulation of protein transport (GO:0051222)
phosphatidylinositol-mediated signaling (GO:0048015)
regulation of secretion by cell (GO:1903530)
positive regulation of nuclear-transcribed mRNA catabolic process, deadenylation-dependent decay (GO:19001
Notch receptor processing (GO:0007220)
embryonic digit morphogenesis (GO:0042733)
negative regulation of response to wounding (GO:1903035)
regulation of cell cycle phase transition (GO:1901987)
regulation of water loss via skin (GO:0033561)
positive regulation of cardiac muscle hypertrophy (GO:0010613)
DNA methylation or demethylation (GO:0044728)
negative regulation of transcription regulatory region DNA binding (GO:2000678)
negative regulation of translational initiation (GO:0045947)
cellular response to pH (GO:0071467)
negative regulation of DNA recombination (GO:0045910)
negative regulation of DNA replication (GO:0008156)
T cell migration (GO:0072678)
bone morphogenesis (GO:0060349)
regulation of SMAD protein import into nucleus (GO:0060390)
positive regulation of cyclic nucleotide metabolic process (GO:0030801)
mRNA transcription from RNA polymerase II promoter (GO:0042789)
G-protein coupled acetylcholine receptor signaling pathway (GO:0007213)
regulation of stem cell proliferation (GO:0072091)
activation of protein kinase A activity (GO:0034199)
protein localization to chromatin (GO:0071168)
detection of visible light (GO:0009584)
neurotransmitter transport (GO:0006836)
regulation of glucose transport (GO:0010827)
mesenchyme development (GO:0060485)
positive regulation of response to endoplasmic reticulum stress (GO:1905898)
programmed necrotic cell death (GO:0097300)
neural tube development (GO:0021915)
DNA alkylation (GO:0006305)
establishment of skin barrier (GO:0061436)
phosphatidylinositol acyl-chain remodeling (GO:0036149)
activation of NF-kappaB-inducing kinase activity (GO:0007250)
regulation of reactive oxygen species biosynthetic process (GO:1903426)

negative regulation of steroid biosynthetic process (GO:0010894)
regulation of histone H3-K4 methylation (GO:0051569)
regulation of cytosolic calcium ion concentration (GO:0051480)
cell morphogenesis involved in neuron differentiation (GO:0048667)
protein localization to organelle (GO:0033365)
regulation of receptor-mediated endocytosis (GO:0048259)
histone exchange (GO:0043486)
organophosphate biosynthetic process (GO:0090407)
calcium ion transmembrane import into cytosol (GO:0097553)
vesicle-mediated transport in synapse (GO:0099003)
centromere complex assembly (GO:0034508)
cardiac muscle contraction (GO:0060048)
regulation of cysteine-type endopeptidase activity (GO:2000116)
spermatid development (GO:0007286)
organic hydroxy compound transport (GO:0015850)
positive regulation of signaling (GO:0023056)
ERAD pathway (GO:0036503)
positive regulation of cytoskeleton organization (GO:0051495)
cellular response to cytokine stimulus (GO:0071345)
regulation of signal transduction by p53 class mediator (GO:1901796)
protein homotetramerization (GO:0051289)
regulation of glycolytic process (GO:0006110)
tRNA modification (GO:0006400)
positive regulation of cell motility (GO:2000147)
regulation of cellular response to heat (GO:1900034)
regulation of phosphorylation (GO:0042325)
regulation of sodium ion transmembrane transporter activity (GO:2000649)
response to steroid hormone (GO:0048545)
organic cyclic compound biosynthetic process (GO:1901362)
base-excision repair (GO:0006284)
positive regulation of mitochondrial outer membrane permeabilization involved in apoptotic signaling pathway (G
anterograde trans-synaptic signaling (GO:0098916)
positive regulation of cytokine production (GO:0001819)
receptor metabolic process (GO:0043112)
ubiquitin-dependent ERAD pathway (GO:0030433)
integrin-mediated signaling pathway (GO:0007229)
response to light stimulus (GO:0009416)
positive regulation of intracellular signal transduction (GO:1902533)
positive regulation of cholesterol transport (GO:0032376)
regulation of tissue remodeling (GO:0034103)
reverse cholesterol transport (GO:0043691)
positive regulation of fatty acid metabolic process (GO:0045923)
positive regulation of monocyte chemotaxis (GO:0090026)
mRNA cis splicing, via spliceosome (GO:0045292)
positive regulation of response to biotic stimulus (GO:0002833)
pigment granule transport (GO:0051904)
regulation of myelination (GO:0031641)
negative regulation of reactive oxygen species metabolic process (GO:2000378)
spindle assembly checkpoint (GO:0071173)
maturation of 5.8S rRNA (GO:0000460)
positive regulation of cellular protein catabolic process (GO:1903364)
peptidyl-lysine trimethylation (GO:0018023)
rRNA catabolic process (GO:0016075)

regulation of protein localization to membrane (GO:1905475)
phospholipid catabolic process (GO:0009395)
positive regulation of calcium ion transmembrane transporter activity (GO:1901021)
positive regulation of signal transduction by p53 class mediator (GO:1901798)
long-term memory (GO:0007616)
heterocycle biosynthetic process (GO:0018130)
regulation of cardiac muscle contraction by regulation of the release of sequestered calcium ion (GO:0010881)
serine family amino acid metabolic process (GO:0009069)
positive regulation by host of viral transcription (GO:0043923)
regulation of lamellipodium organization (GO:1902743)
purine-containing compound metabolic process (GO:0072521)
detection of mechanical stimulus (GO:0050982)
melanosome transport (GO:0032402)
nucleobase-containing compound biosynthetic process (GO:0034654)
positive regulation of cation transmembrane transport (GO:1904064)
cellular response to glucocorticoid stimulus (GO:0071385)
viral entry into host cell (GO:0046718)
mitotic spindle assembly checkpoint (GO:0007094)
mitotic spindle checkpoint (GO:0071174)
mitotic G2 DNA damage checkpoint (GO:0007095)
regulation of cellular protein catabolic process (GO:1903362)
DNA catabolic process (GO:0006308)
positive regulation of lymphocyte migration (GO:2000403)
2-oxoglutarate metabolic process (GO:0006103)
regulation of small GTPase mediated signal transduction (GO:0051056)
plasma membrane bounded cell projection assembly (GO:0120031)
cellular response to metal ion (GO:0071248)
chemical homeostasis (GO:0048878)
Golgi organization (GO:0007030)
metal ion transport (GO:0030001)
regulation of adherens junction organization (GO:1903391)
long-chain fatty-acyl-CoA biosynthetic process (GO:0035338)
establishment of melanosome localization (GO:0032401)
nuclear envelope reassembly (GO:0031468)
cellular response to gamma radiation (GO:0071480)
protein mannosylation (GO:0035268)
ubiquitin-dependent protein catabolic process via the multivesicular body sorting pathway (GO:0043162)
anion homeostasis (GO:0055081)
regulation of oxidative stress-induced intrinsic apoptotic signaling pathway (GO:1902175)
negative regulation of protein dephosphorylation (GO:0035308)
outer dynein arm assembly (GO:0036158)
negative regulation of microtubule depolymerization (GO:0007026)
regulation of cholesterol efflux (GO:0010874)
skeletal muscle contraction (GO:0003009)
positive regulation of natural killer cell mediated cytotoxicity (GO:0045954)
positive regulation of p38MAPK cascade (GO:1900745)
glycogen catabolic process (GO:0005980)
phosphatidylglycerol acyl-chain remodeling (GO:0036148)
blood vessel endothelial cell migration (GO:0043534)
DNA methylation (GO:0006306)
establishment of epithelial cell polarity (GO:0090162)
regulation of dendrite extension (GO:1903859)
membrane protein ectodomain proteolysis (GO:0006509)

receptor localization to synapse (GO:0097120)
release of cytochrome c from mitochondria (GO:0001836)
endoplasmic reticulum calcium ion homeostasis (GO:0032469)
positive regulation of protein binding (GO:0032092)
muscle organ development (GO:0007517)
membrane lipid biosynthetic process (GO:0046467)
signal transduction by p53 class mediator (GO:0072331)
regulation of proteasomal protein catabolic process (GO:0061136)
inositol lipid-mediated signaling (GO:0048017)
DNA replication-independent nucleosome assembly (GO:0006336)
cellular response to amino acid starvation (GO:0034198)
acyl-CoA metabolic process (GO:0006637)
regulation of extrinsic apoptotic signaling pathway via death domain receptors (GO:1902041)
clathrin-dependent endocytosis (GO:0072583)
glycerophospholipid biosynthetic process (GO:0046474)
positive regulation of protein secretion (GO:0050714)
dephosphorylation (GO:0016311)
organic anion transport (GO:0015711)
sensory organ development (GO:0007423)
phosphatidic acid biosynthetic process (GO:0006654)
negative regulation of cytokine-mediated signaling pathway (GO:0001960)
regulation of cholesterol biosynthetic process (GO:0045540)
negative regulation of smoothened signaling pathway (GO:0045879)
positive regulation of chromosome organization (GO:2001252)
primary alcohol metabolic process (GO:0034308)
negative regulation of lymphocyte activation (GO:0051250)
regulation of sprouting angiogenesis (GO:1903670)
protein targeting to lysosome (GO:0006622)
acetylcholine receptor signaling pathway (GO:0095500)
maturation of 5.8S rRNA from tricistronic rRNA transcript (SSU-rRNA, 5.8S rRNA, LSU-rRNA) (GO:0000466)
vesicle transport along microtubule (GO:0047496)
endothelial cell development (GO:0001885)
morphogenesis of an epithelium (GO:0002009)
positive regulation of mononuclear cell migration (GO:0071677)
cyclic nucleotide biosynthetic process (GO:0009190)
regulation of mitochondrial fission (GO:0090140)
nucleosome disassembly (GO:0006337)
chromatin disassembly (GO:0031498)
extrinsic apoptotic signaling pathway in absence of ligand (GO:0097192)
cellular polysaccharide biosynthetic process (GO:0033692)
positive regulation of excitatory postsynaptic potential (GO:2000463)
negative regulation of protein localization to cell periphery (GO:1904376)
positive regulation of blood coagulation (GO:0030194)
regulation of platelet activation (GO:0010543)
aspartate family amino acid catabolic process (GO:0009068)
negative regulation of calcium ion transport (GO:0051926)
telomere capping (GO:0016233)
positive regulation of epithelial cell differentiation (GO:0030858)
positive regulation of transcription regulatory region DNA binding (GO:2000679)
triglyceride biosynthetic process (GO:0019432)
positive regulation of axon extension (GO:0045773)
negative regulation of cell junction assembly (GO:1901889)
negative regulation of protein localization to plasma membrane (GO:1903077)

positive regulation of heart contraction (GO:0045823)
regulation of long-term synaptic potentiation (GO:1900271)
negative regulation of double-strand break repair (GO:2000780)
acylglycerol biosynthetic process (GO:0046463)
positive regulation of peptidyl-serine phosphorylation (GO:0033138)
regulation of cellular response to stress (GO:0080135)
monovalent inorganic cation transport (GO:0015672)
stress-activated MAPK cascade (GO:0051403)
tRNA processing (GO:0008033)
regulation of translational initiation (GO:0006446)
positive regulation of type I interferon production (GO:0032481)
negative regulation of G2/M transition of mitotic cell cycle (GO:0010972)
positive regulation of phosphorylation (GO:0042327)
translational elongation (GO:0006414)
protein modification process (GO:0036211)
regulation of cholesterol metabolic process (GO:0090181)
calcium-dependent cell-cell adhesion via plasma membrane cell adhesion molecules (GO:0016339)
positive regulation of viral transcription (GO:0050434)
phosphatidic acid metabolic process (GO:0046473)
peptide transport (GO:0015833)
telomere organization (GO:0032200)
carbohydrate homeostasis (GO:0033500)
regulation of proteolysis (GO:0030162)
negative regulation of innate immune response (GO:0045824)
regulation of vacuole organization (GO:0044088)
negative regulation of phosphate metabolic process (GO:0045936)
regulation of regulatory T cell differentiation (GO:0045589)
response to progesterone (GO:0032570)
phosphatidylethanolamine metabolic process (GO:0046337)
regulation of DNA-templated transcription, elongation (GO:0032784)
androgen metabolic process (GO:0008209)
excitatory postsynaptic potential (GO:0060079)
regulation of cell development (GO:0060284)
negative regulation of protein depolymerization (GO:1901880)
regulation of response to cytokine stimulus (GO:0060759)
regulation of toll-like receptor signaling pathway (GO:0034121)
dorsal/ventral pattern formation (GO:0009953)
melanosome localization (GO:0032400)
inositol phosphate-mediated signaling (GO:0048016)
cellular response to steroid hormone stimulus (GO:0071383)
immune response-activating cell surface receptor signaling pathway (GO:0002429)
regulation of transport (GO:0051049)
mitochondrial RNA metabolic process (GO:0000959)
positive regulation of interleukin-2 production (GO:0032743)
natural killer cell activation involved in immune response (GO:0002323)
regulation of lipoprotein lipase activity (GO:0051004)
embryonic digestive tract development (GO:0048566)
regulation of cholesterol transport (GO:0032374)
mitochondrial respiratory chain complex IV assembly (GO:0033617)
Arp2/3 complex-mediated actin nucleation (GO:0034314)
autophagy of nucleus (GO:0044804)
regulated exocytosis (GO:0045055)
regulation of ion transmembrane transporter activity (GO:0032412)

ceramide metabolic process (GO:0006672)
regulation of interleukin-6 production (GO:0032675)
glucose metabolic process (GO:0006006)
positive regulation of mitochondrion organization (GO:0010822)
viral life cycle (GO:0019058)
cellular response to interleukin-1 (GO:0071347)
nucleotide-excision repair (GO:0006289)
regulation of cytokine production (GO:0001817)
positive regulation of GTPase activity (GO:0043547)
response to hydrogen peroxide (GO:0042542)
positive regulation of interleukin-6 production (GO:0032755)
substantia nigra development (GO:0021762)
regulation of interferon-gamma production (GO:0032649)
positive regulation of interferon-gamma production (GO:0032729)
mitotic metaphase plate congression (GO:0007080)
hyaluronan metabolic process (GO:0030212)
energy reserve metabolic process (GO:0006112)
histone lysine demethylation (GO:0070076)
mesoderm development (GO:0007498)
positive regulation of T cell mediated immunity (GO:0002711)
mitochondrial fission (GO:0000266)
regulation of cardiac muscle hypertrophy (GO:0010611)
positive regulation of lamellipodium organization (GO:1902745)
protein O-linked glycosylation via threonine (GO:0018243)
negative regulation of G-protein coupled receptor protein signaling pathway (GO:0045744)
negative regulation of protein tyrosine kinase activity (GO:0061099)
response to nicotine (GO:0035094)
negative regulation of viral process (GO:0048525)
protein-DNA complex disassembly (GO:0032986)
mitochondrial respiratory chain complex IV biogenesis (GO:0097034)
negative regulation of cyclin-dependent protein kinase activity (GO:1904030)
positive regulation of receptor internalization (GO:0002092)
phosphatidylserine acyl-chain remodeling (GO:0036150)
negative regulation of cell cycle arrest (GO:0071157)
regulation of ruffle assembly (GO:1900027)
mitochondrial electron transport, cytochrome c to oxygen (GO:0006123)
cellular protein complex localization (GO:0034629)
histone ubiquitination (GO:0016574)
JNK cascade (GO:0007254)
positive regulation of intracellular protein transport (GO:0090316)
hexose biosynthetic process (GO:0019319)
negative regulation of type I interferon production (GO:0032480)
heart contraction (GO:0060047)
microtubule cytoskeleton organization involved in mitosis (GO:1902850)
mitotic nuclear envelope disassembly (GO:0007077)
metaphase plate congression (GO:0051310)
inorganic anion transport (GO:0015698)
regulation of steroid biosynthetic process (GO:0050810)
positive regulation of viral process (GO:0048524)
regulation of focal adhesion assembly (GO:0051893)
negative regulation of immune system process (GO:0002683)
macroautophagy (GO:0016236)
positive regulation of establishment of protein localization to mitochondrion (GO:1903749)

regulation of vascular endothelial growth factor receptor signaling pathway (GO:0030947)
GTP metabolic process (GO:0046039)
regulation of cardiac muscle contraction by calcium ion signaling (GO:0010882)
early endosome to late endosome transport (GO:0045022)
nuclear transport (GO:0051169)
peripheral nervous system development (GO:0007422)
histone lysine methylation (GO:0034968)
embryonic skeletal system morphogenesis (GO:0048704)
rRNA modification (GO:0000154)
regulation of cytokine production involved in inflammatory response (GO:1900015)
purine-containing compound biosynthetic process (GO:0072522)
STAT cascade (GO:0097696)
regulation of microtubule depolymerization (GO:0031114)
protein O-linked glycosylation via serine (GO:0018242)
urogenital system development (GO:0001655)
protein complex localization (GO:0031503)
C21-steroid hormone metabolic process (GO:0008207)
digestive system development (GO:0055123)
glucose transport (GO:0015758)
regulation of calcium ion transport into cytosol (GO:0010522)
regulation of calcium ion import (GO:0090279)
positive regulation of proteolysis involved in cellular protein catabolic process (GO:1903052)
lymphocyte activation involved in immune response (GO:0002285)
activation of phospholipase C activity (GO:0007202)
negative regulation of protein binding (GO:0032091)
cellular response to UV (GO:0034644)
regulation of gene silencing by miRNA (GO:0060964)
nucleus organization (GO:0006997)
mitochondrial gene expression (GO:0140053)
metal ion homeostasis (GO:0055065)
hexose metabolic process (GO:0019318)
nicotinamide nucleotide metabolic process (GO:0046496)
regulation of cell cycle process (GO:0010564)
negative regulation of phosphatase activity (GO:0010923)
cholesterol metabolic process (GO:0008203)
protein sumoylation (GO:0016925)
regulation of protein oligomerization (GO:0032459)
glycolytic process (GO:0006096)
regulation of proteolysis involved in cellular protein catabolic process (GO:1903050)
vesicle-mediated transport between endosomal compartments (GO:0098927)
nucleotide-excision repair, DNA damage recognition (GO:0000715)
membrane assembly (GO:0071709)
regulation of smooth muscle contraction (GO:0006940)
response to alkaloid (GO:0043279)
maturation of LSU-rRNA (GO:0000470)
negative regulation of axonogenesis (GO:0050771)
divalent inorganic cation homeostasis (GO:0072507)
regulation of release of sequestered calcium ion into cytosol by sarcoplasmic reticulum (GO:0010880)
cellular response to amyloid-beta (GO:1904646)
regulation of ion homeostasis (GO:2000021)
negative regulation of axon extension (GO:0030517)
mitotic G2/M transition checkpoint (GO:0044818)
cholesterol efflux (GO:0033344)

negative regulation of viral transcription (GO:0032897)
positive regulation of protein oligomerization (GO:0032461)
TOR signaling (GO:0031929)
glutamine family amino acid metabolic process (GO:0009064)
pyruvate metabolic process (GO:0006090)
RNA secondary structure unwinding (GO:0010501)
regulation of generation of precursor metabolites and energy (GO:0043467)
cellular response to ionizing radiation (GO:0071479)
nuclear envelope disassembly (GO:0051081)
phospholipase C-activating G-protein coupled receptor signaling pathway (GO:0007200)
negative regulation of I-kappaB kinase/NF-kappaB signaling (GO:0043124)
negative regulation of cellular biosynthetic process (GO:0031327)
negative regulation of JAK-STAT cascade (GO:0046426)
cellular response to heat (GO:0034605)
Rab protein signal transduction (GO:0032482)
snRNA transcription (GO:0009301)
snRNA transcription from RNA polymerase II promoter (GO:0042795)
protein transport (GO:0015031)
positive regulation of cell cycle G1/S phase transition (GO:1902808)
phospholipid dephosphorylation (GO:0046839)
epithelium development (GO:0060429)
phosphatidylcholine metabolic process (GO:0046470)
nuclear import (GO:0051170)
cytoplasmic microtubule organization (GO:0031122)
interstrand cross-link repair (GO:0036297)
cellular macromolecule catabolic process (GO:0044265)
endosome to lysosome transport (GO:0008333)
respiratory electron transport chain (GO:0022904)
DNA repair (GO:0006281)
regulation of neuron death (GO:1901214)
regulation of phosphatase activity (GO:0010921)
positive regulation of histone methylation (GO:0031062)
cellular lipid catabolic process (GO:0044242)
negative regulation of protein localization to membrane (GO:1905476)
cellular modified amino acid biosynthetic process (GO:0042398)
positive regulation of DNA-templated transcription, elongation (GO:0032786)
negative regulation of cyclin-dependent protein serine/threonine kinase activity (GO:0045736)
axoneme assembly (GO:0035082)
regulation of AMPA receptor activity (GO:2000311)
limb development (GO:0060173)
regulation of neuronal synaptic plasticity (GO:0048168)
negative regulation of interleukin-6 production (GO:0032715)
mammary gland development (GO:0030879)
transition metal ion homeostasis (GO:0055076)
negative regulation of T cell proliferation (GO:0042130)
cGMP biosynthetic process (GO:0006182)
regulation of cellular senescence (GO:2000772)
positive regulation of neutrophil chemotaxis (GO:0090023)
membrane protein intracellular domain proteolysis (GO:0031293)
positive regulation of immune effector process (GO:0002699)
histone monoubiquitination (GO:0010390)
negative regulation of developmental growth (GO:0048640)
regulation of actin filament bundle assembly (GO:0032231)

ATP generation from ADP (GO:0006757)
activation of MAPK activity (GO:0000187)
positive regulation of protein localization to membrane (GO:1905477)
transcription elongation from RNA polymerase II promoter (GO:0006368)
protein autoubiquitination (GO:0051865)
establishment of protein localization to mitochondrion (GO:0072655)
positive regulation of response to DNA damage stimulus (GO:2001022)
lipid homeostasis (GO:0055088)
response to lipid (GO:0033993)
regulation of cellular component organization (GO:0051128)
calcium ion transport (GO:0006816)
transcription-coupled nucleotide-excision repair (GO:0006283)
protein localization to nucleus (GO:0034504)
ciliary basal body-plasma membrane docking (GO:0097711)
regulation of mitotic cell cycle phase transition (GO:1901990)
myeloid leukocyte differentiation (GO:0002573)
receptor internalization (GO:0031623)
neutrophil degranulation (GO:0043312)
negative regulation of wound healing (GO:0061045)
organophosphate ester transport (GO:0015748)
canonical glycolysis (GO:0061621)
respiratory chain complex IV assembly (GO:0008535)
response to amyloid-beta (GO:1904645)
positive regulation of nuclear division (GO:0051785)
monocarboxylic acid catabolic process (GO:0072329)
positive regulation of glucose transport (GO:0010828)
regulation of glucose metabolic process (GO:0010906)
positive regulation of interleukin-1 beta secretion (GO:0050718)
negative regulation of cellular protein localization (GO:1903828)
cellular response to epidermal growth factor stimulus (GO:0071364)
cellular response to vascular endothelial growth factor stimulus (GO:0035924)
ncRNA 3'-end processing (GO:0043628)
long-chain fatty acid transport (GO:0015909)
long-chain fatty-acyl-CoA metabolic process (GO:0035336)
respiratory tube development (GO:0030323)
positive regulation of neutrophil migration (GO:1902624)
peptide hormone secretion (GO:0030072)
3'-phosphoadenosine 5'-phosphosulfate metabolic process (GO:0050427)
negative regulation of locomotion (GO:0040013)
regulation of nervous system development (GO:0051960)
peptidyl-tyrosine dephosphorylation (GO:0035335)
glycolytic process through glucose-6-phosphate (GO:0061620)
regulation of voltage-gated calcium channel activity (GO:1901385)
glucose catabolic process to pyruvate (GO:0061718)
positive regulation of phosphatidylinositol 3-kinase activity (GO:0043552)
inorganic cation import across plasma membrane (GO:0098659)
potassium ion homeostasis (GO:0055075)
neutral amino acid transport (GO:0015804)
prostaglandin metabolic process (GO:0006693)
organelle assembly (GO:0070925)
response to tumor necrosis factor (GO:0034612)
positive regulation of macromolecule biosynthetic process (GO:0010557)
regulation of cellular amine metabolic process (GO:0033238)

telomere maintenance (GO:0000723)
regulation of cellular amino acid metabolic process (GO:0006521)
transmembrane transport (GO:0055085)
response to UV (GO:0009411)
response to molecule of bacterial origin (GO:0002237)
regulation of defense response (GO:0031347)
stimulatory C-type lectin receptor signaling pathway (GO:0002223)
neutrophil activation involved in immune response (GO:0002283)
response to gamma radiation (GO:0010332)
regulation of p38MAPK cascade (GO:1900744)
positive regulation of granulocyte chemotaxis (GO:0071624)
positive regulation of wound healing (GO:0090303)
regulation of cardiac muscle cell contraction (GO:0086004)
fat-soluble vitamin metabolic process (GO:0006775)
positive regulation of release of cytochrome c from mitochondria (GO:0090200)
regulation of T cell receptor signaling pathway (GO:0050856)
RNA biosynthetic process (GO:0032774)
sodium ion homeostasis (GO:0055078)
apoptotic cell clearance (GO:0043277)
monovalent inorganic cation homeostasis (GO:0055067)
regulation of centrosome cycle (GO:0046605)
regulation of intracellular signal transduction (GO:1902531)
chemokine-mediated signaling pathway (GO:0070098)
positive regulation of proteolysis (GO:0045862)
regulation of hematopoietic stem cell differentiation (GO:1902036)
positive regulation of ubiquitin-protein ligase activity involved in regulation of mitotic cell cycle transition (GO:00[illegible]
positive regulation of apoptotic signaling pathway (GO:2001235)
tumor necrosis factor-mediated signaling pathway (GO:0033209)
positive regulation of lipid kinase activity (GO:0090218)
negative regulation of synaptic transmission (GO:0050805)
cristae formation (GO:0042407)
peptidyl-glutamic acid modification (GO:0018200)
regulation of double-strand break repair via homologous recombination (GO:0010569)
regulation of extrinsic apoptotic signaling pathway in absence of ligand (GO:2001239)
release of sequestered calcium ion into cytosol (GO:0051209)
negative regulation of myeloid leukocyte differentiation (GO:0002762)
phosphatidylserine metabolic process (GO:0006658)
regulation of neurotransmitter secretion (GO:0046928)
regulation of blood vessel diameter (GO:0097746)
negative regulation of organelle assembly (GO:1902116)
phosphatidylcholine acyl-chain remodeling (GO:0036151)
response to hexose (GO:0009746)
neutrophil mediated immunity (GO:0002446)
response to ionizing radiation (GO:0010212)
regulation of hematopoietic progenitor cell differentiation (GO:1901532)
regulation of cytokinesis (GO:0032465)
monocarboxylic acid transport (GO:0015718)
positive regulation of protein import into nucleus (GO:0042307)
regulation of kinase activity (GO:0043549)
innate immune response activating cell surface receptor signaling pathway (GO:0002220)
platelet degranulation (GO:0002576)
positive regulation of translation (GO:0045727)
negative regulation of cell communication (GO:0010648)

antigen processing and presentation of exogenous peptide antigen via MHC class I (GO:0042590)
visual perception (GO:0007601)
intracellular transport of virus (GO:0075733)
negative regulation of cellular component organization (GO:0051129)
positive regulation of response to stimulus (GO:0048584)
transport of virus (GO:0046794)
G-protein coupled receptor signaling pathway, coupled to cyclic nucleotide second messenger (GO:0007187)
cellular response to ketone (GO:1901655)
synaptic transmission, cholinergic (GO:0007271)
negative regulation of response to DNA damage stimulus (GO:2001021)
organelle fission (GO:0048285)
positive regulation of phospholipid metabolic process (GO:1903727)
positive regulation of calcium-mediated signaling (GO:0050850)
regulation of fatty acid biosynthetic process (GO:0042304)
regulation of adenylate cyclase activity (GO:0045761)
regulation of cell morphogenesis (GO:0022604)
protein export from nucleus (GO:0006611)
RNA phosphodiester bond hydrolysis (GO:0090501)
negative regulation of proteasomal protein catabolic process (GO:1901799)
regulation of receptor internalization (GO:0002090)
aerobic respiration (GO:0009060)
anaphase-promoting complex-dependent catabolic process (GO:0031145)
nucleosome organization (GO:0034728)
G2/M transition of mitotic cell cycle (GO:0000086)
regulation of cellular catabolic process (GO:0031329)
positive regulation of cysteine-type endopeptidase activity (GO:2001056)
NIK/NF-kappaB signaling (GO:0038061)
negative regulation of defense response (GO:0031348)
protein lipidation (GO:0006497)
dicarboxylic acid transport (GO:0006835)
collagen fibril organization (GO:0030199)
synaptic vesicle endocytosis (GO:0048488)
keratan sulfate biosynthetic process (GO:0018146)
positive regulation of transcription from RNA polymerase II promoter in response to stress (GO:0036003)
positive regulation of interleukin-1 secretion (GO:0050716)
regulation of activated T cell proliferation (GO:0046006)
cellular response to cadmium ion (GO:0071276)
lung development (GO:0030324)
protein import (GO:0017038)
negative regulation of cellular catabolic process (GO:0031330)
cellular iron ion homeostasis (GO:0006879)
regulation of tumor necrosis factor-mediated signaling pathway (GO:0010803)
anion transport (GO:0006820)
cellular defense response (GO:0006968)
positive regulation of protein targeting to mitochondrion (GO:1903955)
sensory perception of light stimulus (GO:0050953)
regulation of actin cytoskeleton reorganization (GO:2000249)
negative regulation of cation channel activity (GO:2001258)
negative regulation of secretion by cell (GO:1903531)
cyclic purine nucleotide metabolic process (GO:0052652)
multivesicular body assembly (GO:0036258)
fatty-acyl-CoA biosynthetic process (GO:0046949)
positive regulation of cation channel activity (GO:2001259)

protein localization to lysosome (GO:0061462)
regulation of developmental growth (GO:0048638)
phosphatidylinositol 3-kinase signaling (GO:0014065)
regulation of cell migration involved in sprouting angiogenesis (GO:0090049)
replication fork processing (GO:0031297)
acylglycerol catabolic process (GO:0046464)
cellular response to cAMP (GO:0071320)
cellular respiration (GO:0045333)
positive regulation of cell death (GO:0010942)
mitotic sister chromatid segregation (GO:0000070)
positive regulation of DNA metabolic process (GO:0051054)
positive regulation of ubiquitin protein ligase activity (GO:1904668)
mitochondrial translation (GO:0032543)
positive regulation of cell cycle process (GO:0090068)
negative regulation of response to external stimulus (GO:0032102)
negative regulation of signaling (GO:0023057)
positive regulation of homeostatic process (GO:0032846)
cholesterol homeostasis (GO:0042632)
sterol homeostasis (GO:0055092)
regulation of dendritic spine development (GO:0060998)
negative regulation of cell-cell adhesion (GO:0022408)
negative regulation of tumor necrosis factor superfamily cytokine production (GO:1903556)
alpha-amino acid metabolic process (GO:1901605)
organelle transport along microtubule (GO:0072384)
regulation of protein export from nucleus (GO:0046825)
zinc ion homeostasis (GO:0055069)
negative regulation of extrinsic apoptotic signaling pathway via death domain receptors (GO:1902042)
multivesicular body organization (GO:0036257)
cofactor biosynthetic process (GO:0051188)
positive regulation of interleukin-1 beta production (GO:0032731)
cellular zinc ion homeostasis (GO:0006882)
positive regulation of insulin secretion (GO:0032024)
response to cadmium ion (GO:0046686)
regulation of cytokine secretion (GO:0050707)
sarcomere organization (GO:0045214)
positive regulation of release of sequestered calcium ion into cytosol (GO:0051281)
cilium assembly (GO:0060271)
DNA-templated transcription, elongation (GO:0006354)
positive regulation of supramolecular fiber organization (GO:1902905)
regulation of mitotic nuclear division (GO:0007088)
regulation of cell cycle G2/M phase transition (GO:1902749)
response to lipopolysaccharide (GO:0032496)
ion transport (GO:0006811)
positive regulation of MAPK cascade (GO:0043410)
sterol metabolic process (GO:0016125)
dicarboxylic acid metabolic process (GO:0043648)
regulation of protein metabolic process (GO:0051246)
positive regulation of ERK1 and ERK2 cascade (GO:0070374)
dendrite morphogenesis (GO:0048813)
cellular response to amino acid stimulus (GO:0071230)
cellular response to glucagon stimulus (GO:0071377)
microtubule bundle formation (GO:0001578)
cyclic-nucleotide-mediated signaling (GO:0019935)

mitochondrial transmembrane transport (GO:1990542)
mismatch repair (GO:0006298)
positive regulation of myeloid leukocyte differentiation (GO:0002763)
response to zinc ion (GO:0010043)
phosphatidylcholine biosynthetic process (GO:0006656)
sister chromatid segregation (GO:0000819)
positive regulation of cytosolic calcium ion concentration (GO:0007204)
proteolysis (GO:0006508)
response to reactive oxygen species (GO:0000302)
nucleosome assembly (GO:0006334)
negative regulation of cytokine production (GO:0001818)
glycogen metabolic process (GO:0005977)
axonemal dynein complex assembly (GO:0070286)
oligosaccharide biosynthetic process (GO:0009312)
tRNA-containing ribonucleoprotein complex export from nucleus (GO:0071431)
regulation of cell-substrate adhesion (GO:0010810)
tRNA export from nucleus (GO:0006409)
platelet aggregation (GO:0070527)
positive regulation of intrinsic apoptotic signaling pathway (GO:2001244)
regulation of phosphatidylinositol 3-kinase activity (GO:0043551)
positive regulation of nucleocytoplasmic transport (GO:0046824)
regulation of interleukin-12 production (GO:0032655)
positive regulation of calcium ion transport (GO:0051928)
regulation of I-kappaB kinase/NF-kappaB signaling (GO:0043122)
potassium ion transport (GO:0006813)
regulation of vesicle fusion (GO:0031338)
regulation of cellular ketone metabolic process (GO:0010565)
negative regulation of viral life cycle (GO:1903901)
positive regulation of cellular amide metabolic process (GO:0034250)
mitochondrial transport (GO:0006839)
mitochondrial translational elongation (GO:0070125)
organic substance transport (GO:0071702)
positive regulation of protein ubiquitination involved in ubiquitin-dependent protein catabolic process (GO:20000
Fc-epsilon receptor signaling pathway (GO:0038095)
Fc receptor signaling pathway (GO:0038093)
cellular amide metabolic process (GO:0043603)
cellular protein catabolic process (GO:0044257)
positive regulation of leukocyte migration (GO:0002687)
positive regulation of cytokinesis (GO:0032467)
regulation of insulin receptor signaling pathway (GO:0046626)
regulation of interleukin-1 beta secretion (GO:0050706)
macromolecule catabolic process (GO:0009057)
regulation of interleukin-8 production (GO:0032677)
negative regulation of cellular metabolic process (GO:0031324)
keratan sulfate metabolic process (GO:0042339)
response to amino acid (GO:0043200)
regulation of protein targeting to mitochondrion (GO:1903214)
COPII vesicle coating (GO:0048208)
iron ion homeostasis (GO:0055072)
vesicle targeting, rough ER to cis-Golgi (GO:0048207)
negative regulation of dephosphorylation (GO:0035305)
vesicle coating (GO:0006901)
negative regulation of cellular protein metabolic process (GO:0032269)

negative regulation of cell-substrate adhesion (GO:0010812)
regulation of microtubule polymerization (GO:0031113)
maturation of SSU-rRNA from tricistronic rRNA transcript (SSU-rRNA, 5.8S rRNA, LSU-rRNA) (GO:0000462)
tRNA transport (GO:0051031)
cholesterol biosynthetic process (GO:0006695)
regulation of ion transmembrane transport (GO:0034765)
negative regulation of tumor necrosis factor production (GO:0032720)
carbohydrate catabolic process (GO:0016052)
DNA-dependent DNA replication maintenance of fidelity (GO:0045005)
antibacterial humoral response (GO:0019731)
positive regulation of DNA replication (GO:0045740)
positive regulation of protein polymerization (GO:0032273)
NADH dehydrogenase complex assembly (GO:0010257)
mitochondrial respiratory chain complex I biogenesis (GO:0097031)
mitochondrial respiratory chain complex I assembly (GO:0032981)
positive regulation of endocytosis (GO:0045807)
positive regulation of I-kappaB kinase/NF-kappaB signaling (GO:0043123)
carbohydrate biosynthetic process (GO:0016051)
natural killer cell activation (GO:0030101)
regulation of blood circulation (GO:1903522)
endosome organization (GO:0007032)
acylglycerol metabolic process (GO:0006639)
regulation of ubiquitin protein ligase activity (GO:1904666)
inner mitochondrial membrane organization (GO:0007007)
secondary alcohol biosynthetic process (GO:1902653)
glycolipid metabolic process (GO:0006664)
regulation of cell division (GO:0051302)
defense response to Gram-positive bacterium (GO:0050830)
fatty acid catabolic process (GO:0009062)
chromatin assembly (GO:0031497)
positive regulation of cellular metabolic process (GO:0031325)
sodium-independent organic anion transport (GO:0043252)
phospholipid biosynthetic process (GO:0008654)
regulation of osteoclast differentiation (GO:0045670)
regulation of carbohydrate catabolic process (GO:0043470)
regulation of metal ion transport (GO:0010959)
positive regulation of cellular protein localization (GO:1903829)
cellular response to light stimulus (GO:0071482)
cellular transition metal ion homeostasis (GO:0046916)
regulation of protein localization to plasma membrane (GO:1903076)
epidermal cell differentiation (GO:0009913)
muscle filament sliding (GO:0030049)
actin-myosin filament sliding (GO:0033275)
homotypic cell-cell adhesion (GO:0034109)
regulation of cellular pH (GO:0030641)
skin development (GO:0043588)
cilium organization (GO:0044782)
interleukin-1-mediated signaling pathway (GO:0070498)
purine ribonucleoside monophosphate biosynthetic process (GO:0009168)
positive regulation of phagocytosis (GO:0050766)
purine ribonucleoside monophosphate metabolic process (GO:0009167)
positive regulation of peptide hormone secretion (GO:0090277)
positive regulation of reactive oxygen species metabolic process (GO:2000379)

regulation of plasma membrane bounded cell projection assembly (GO:0120032)
regulation of coenzyme metabolic process (GO:0051196)
cellular response to tumor necrosis factor (GO:0071356)
negative regulation of TOR signaling (GO:0032007)
response to cAMP (GO:0051591)
tRNA aminoacylation (GO:0043039)
sterol biosynthetic process (GO:0016126)
regulation of release of cytochrome c from mitochondria (GO:0090199)
regulation of intracellular transport (GO:0032386)
positive regulation of cytokine biosynthetic process (GO:0042108)
regulation of viral transcription (GO:0046782)
determination of heart left/right asymmetry (GO:0061371)
intraciliary transport involved in cilium assembly (GO:0035735)
positive regulation of epithelial to mesenchymal transition (GO:0010718)
regulation of protein dephosphorylation (GO:0035304)
mitochondrial respiratory chain complex assembly (GO:0033108)
positive regulation of hydrolase activity (GO:0051345)
neuropeptide signaling pathway (GO:0007218)
positive regulation of cell growth (GO:0030307)
phosphatidylinositol biosynthetic process (GO:0006661)
recombinational repair (GO:0000725)
negative regulation of ubiquitin-protein ligase activity involved in mitotic cell cycle (GO:0051436)
defense response to Gram-negative bacterium (GO:0050829)
positive regulation of growth (GO:0045927)
regulation of phagocytosis (GO:0050764)
cellular response to peptide (GO:1901653)
negative regulation of STAT cascade (GO:1904893)
monocyte chemotaxis (GO:0002548)
regulation of protein localization (GO:0032880)
negative regulation of proteolysis (GO:0045861)
gluconeogenesis (GO:0006094)
positive regulation of cell division (GO:0051781)
peptide biosynthetic process (GO:0043043)
lipid biosynthetic process (GO:0008610)
regulation of ubiquitin-protein ligase activity involved in mitotic cell cycle (GO:0051439)
determination of left/right symmetry (GO:0007368)
cellular response to radiation (GO:0071478)
positive regulation of protein localization to nucleus (GO:1900182)
glycolipid biosynthetic process (GO:0009247)
translesion synthesis (GO:0019985)
positive regulation of cytokine secretion (GO:0050715)
cell cycle G2/M phase transition (GO:0044839)
regulation of ATP metabolic process (GO:1903578)
digestive tract development (GO:0048565)
spermatogenesis (GO:0007283)
positive regulation of defense response (GO:0031349)
male gamete generation (GO:0048232)
amide biosynthetic process (GO:0043604)
positive regulation of interleukin-8 production (GO:0032757)
regulation of T cell activation (GO:0050863)
positive regulation of intracellular transport (GO:0032388)
regulation of intracellular pH (GO:0051453)
cholesterol transport (GO:0030301)

antigen processing and presentation of exogenous peptide antigen via MHC class I, TAP-dependent (GO:00024
purine ribonucleotide biosynthetic process (GO:0009152)
inflammatory response (GO:0006954)
double-strand break repair via homologous recombination (GO:0000724)
peptide metabolic process (GO:0006518)
protein polymerization (GO:0051258)
negative regulation of ubiquitin protein ligase activity (GO:1904667)
intraciliary transport (GO:0042073)
maturation of SSU-rRNA (GO:0030490)
icosanoid metabolic process (GO:0006690)
negative regulation of autophagy (GO:0010507)
translation (GO:0006412)
response to endoplasmic reticulum stress (GO:0034976)
fatty acid metabolic process (GO:0006631)
positive regulation of cellular component organization (GO:0051130)
extracellular matrix disassembly (GO:0022617)
negative regulation of inflammatory response (GO:0050728)
sensory perception of mechanical stimulus (GO:0050954)
peptide cross-linking (GO:0018149)
regulation of intrinsic apoptotic signaling pathway (GO:2001242)
positive regulation of tumor necrosis factor production (GO:0032760)
regulation of organelle assembly (GO:1902115)
postreplication repair (GO:0006301)
fatty acid biosynthetic process (GO:0006633)
cation transport (GO:0006812)
phagocytosis (GO:0006909)
positive regulation of chemotaxis (GO:0050921)
amino acid transport (GO:0006865)
cellular response to mechanical stimulus (GO:0071260)
regulation of phosphatidylinositol 3-kinase signaling (GO:0014066)
retrograde vesicle-mediated transport, Golgi to ER (GO:0006890)
receptor-mediated endocytosis (GO:0006898)
sensory perception of sound (GO:0007605)
positive regulation of binding (GO:0051099)
protein catabolic process (GO:0030163)
RNA methylation (GO:0001510)
antimicrobial humoral immune response mediated by antimicrobial peptide (GO:0061844)
regulation of reactive oxygen species metabolic process (GO:2000377)
non-recombinational repair (GO:0000726)
negative regulation of NF-kappaB transcription factor activity (GO:0032088)
fatty acid beta-oxidation (GO:0006635)
double-strand break repair via nonhomologous end joining (GO:0006303)
blood circulation (GO:0008015)
nuclear-transcribed mRNA catabolic process, nonsense-mediated decay (GO:0000184)
response to organonitrogen compound (GO:0010243)
carboxylic acid transport (GO:0046942)
coenzyme metabolic process (GO:0006732)
positive regulation of developmental growth (GO:0048639)
B cell differentiation (GO:0030183)
triglyceride metabolic process (GO:0006641)
mitochondrial ATP synthesis coupled electron transport (GO:0042775)
positive regulation of phosphatidylinositol 3-kinase signaling (GO:0014068)
embryonic organ development (GO:0048568)

neutrophil chemotaxis (GO:0030593)
positive regulation of cellular component biogenesis (GO:0044089)
positive regulation of immune response (GO:0050778)
sensory perception (GO:0007600)
protein targeting to mitochondrion (GO:0006626)
cellular protein metabolic process (GO:0044267)
mitochondrial translational termination (GO:0070126)
chromosome organization (GO:0051276)
granulocyte chemotaxis (GO:0071621)
autophagosome organization (GO:1905037)
ATP metabolic process (GO:0046034)
ribosome assembly (GO:0042255)
cell-matrix adhesion (GO:0007160)
positive regulation of response to external stimulus (GO:0032103)
I-kappaB kinase/NF-kappaB signaling (GO:0007249)
autophagosome assembly (GO:0000045)
neutrophil migration (GO:1990266)
regulation of extrinsic apoptotic signaling pathway (GO:2001236)
keratinocyte differentiation (GO:0030216)
cytokine-mediated signaling pathway (GO:0019221)
cell chemotaxis (GO:0060326)
regulation of B cell activation (GO:0050864)
regulation of tumor necrosis factor production (GO:0032680)
translational termination (GO:0006415)
phosphate-containing compound metabolic process (GO:0006796)
negative regulation of intrinsic apoptotic signaling pathway (GO:2001243)
ribosomal large subunit biogenesis (GO:0042273)
glycerophospholipid metabolic process (GO:0006650)
regulation of JAK-STAT cascade (GO:0046425)
DNA synthesis involved in DNA repair (GO:0000731)
O-glycan processing (GO:0016266)
regulation of TOR signaling (GO:0032006)
response to interferon-gamma (GO:0034341)
positive regulation of T cell activation (GO:0050870)
regulation of inflammatory response (GO:0050727)
regulation of endocytosis (GO:0030100)
phospholipid metabolic process (GO:0006644)
negative regulation of hydrolase activity (GO:0051346)
monocarboxylic acid metabolic process (GO:0032787)
double-strand break repair (GO:0006302)
positive regulation of cysteine-type endopeptidase activity involved in apoptotic process (GO:0043280)
B cell activation (GO:0042113)
lymphocyte differentiation (GO:0030098)
positive regulation of inflammatory response (GO:0050729)
rRNA processing (GO:0006364)
nitrogen compound transport (GO:0071705)
negative regulation of extrinsic apoptotic signaling pathway (GO:2001237)
activation of cysteine-type endopeptidase activity involved in apoptotic process (GO:0006919)
steroid biosynthetic process (GO:0006694)
epidermis development (GO:0008544)
negative regulation of endopeptidase activity (GO:0010951)
T cell activation (GO:0042110)
divalent metal ion transport (GO:0070838)

positive regulation of NF-kappaB transcription factor activity (GO:0051092)
T cell receptor signaling pathway (GO:0050852)
ribosome biogenesis (GO:0042254)
ncRNA processing (GO:0034470)
cotranslational protein targeting to membrane (GO:0006613)
proteolysis involved in cellular protein catabolic process (GO:0051603)
rRNA metabolic process (GO:0016072)
protein targeting to ER (GO:0045047)
steroid metabolic process (GO:0008202)
plasma membrane invagination (GO:0099024)
positive regulation of lymphocyte activation (GO:0051251)
phagocytosis, engulfment (GO:0006911)
regulation of protein activation cascade (GO:2000257)
regulation of complement activation (GO:0030449)
viral gene expression (GO:0019080)
regulation of humoral immune response (GO:0002920)
viral transcription (GO:0019083)
regulation of immune effector process (GO:0002697)
regulation of acute inflammatory response (GO:0002673)
complement activation, classical pathway (GO:0006958)
humoral immune response mediated by circulating immunoglobulin (GO:0002455)
regulation of protein processing (GO:0070613)
defense response to bacterium (GO:0042742)
regulation of immune response (GO:0050776)
antigen receptor-mediated signaling pathway (GO:0050851)

| Overlap | P.value | Adjusted.P.value | Old.P.value | Old.Adjusted |
|---|---|---|---|---|
| 84/814 | 9,3087E-10 | 4,75023E-06 | 0 | 0 |
| 103/1121 | 6,78218E-09 | 1,73047E-05 | 0 | 0 |
| 58/503 | 8,34905E-09 | 1,42017E-05 | 0 | 0 |
| 125/1479 | 2,24254E-08 | 2,86092E-05 | 0 | 0 |
| 51/456 | 1,75869E-07 | 0,000179492 | 0 | 0 |
| 23/131 | 2,30365E-07 | 0,000195925 | 0 | 0 |
| 78/849 | 5,13194E-07 | 0,000374118 | 0 | 0 |
| 127/1599 | 5,52827E-07 | 0,000352634 | 0 | 0 |
| 71/772 | 1,62614E-06 | 0,00092202 | 0 | 0 |
| 60/619 | 2,02412E-06 | 0,00103291 | 0 | 0 |
| 51/513 | 5,83665E-06 | 0,002707674 | 0 | 0 |
| 46/445 | 6,04198E-06 | 0,002569351 | 0 | 0 |
| 24/178 | 1,5762E-05 | 0,006187199 | 0 | 0 |
| 53/566 | 2,09263E-05 | 0,007627645 | 0 | 0 |
| 12/56 | 2,26903E-05 | 0,007719226 | 0 | 0 |
| 12/57 | 2,74046E-05 | 0,008740356 | 0 | 0 |
| 20/140 | 3,40393E-05 | 0,010217793 | 0 | 0 |
| 12/61 | 5,58024E-05 | 0,015819977 | 0 | 0 |
| 20/145 | 5,66572E-05 | 0,01521693 | 0 | 0 |
| 9/37 | 8,40737E-05 | 0,021451397 | 0 | 0 |
| 45/486 | 0,000112005 | 0,027217149 | 0 | 0 |
| 30/284 | 0,00016485 | 0,038237594 | 0 | 0 |
| 9/41 | 0,000196629 | 0,043625974 | 0 | 0 |
| 4/7 | 0,000217587 | 0,046264493 | 0 | 0 |
| 18/142 | 0,00038065 | 0,077698292 | 0 | 0 |
| 33/342 | 0,000427725 | 0,08394919 | 0 | 0 |
| 34/357 | 0,000447262 | 0,084532426 | 0 | 0 |
| 22/195 | 0,000481228 | 0,087703731 | 0 | 0 |
| 11/66 | 0,000517076 | 0,090987477 | 0 | 0 |
| 10/56 | 0,000522527 | 0,0888819 | 0 | 0 |
| 15/110 | 0,000524124 | 0,086277628 | 0 | 0 |
| 17/134 | 0,000541587 | 0,086366252 | 0 | 0 |
| 7/29 | 0,000547596 | 0,08467833 | 0 | 0 |
| 13/88 | 0,000559616 | 0,083991845 | 0 | 0 |
| 6/22 | 0,000679876 | 0,099125863 | 0 | 0 |
| 18/151 | 0,000798843 | 0,113236039 | 0 | 0 |
| 23/216 | 0,000816438 | 0,11260223 | 0 | 0 |
| 8/40 | 0,000860022 | 0,115491879 | 0 | 0 |
| 23/218 | 0,000926152 | 0,121183392 | 0 | 0 |
| 5/16 | 0,000981516 | 0,125216844 | 0 | 0 |
| 5/16 | 0,000981516 | 0,122162775 | 0 | 0 |
| 5/16 | 0,000981516 | 0,119254137 | 0 | 0 |
| 43/507 | 0,000988661 | 0,11732878 | 0 | 0 |
| 4/10 | 0,00115204 | 0,133610423 | 0 | 0 |
| 4/10 | 0,00115204 | 0,130641302 | 0 | 0 |
| 4/10 | 0,00115204 | 0,127801274 | 0 | 0 |
| 4/10 | 0,00115204 | 0,125082098 | 0 | 0 |
| 8/42 | 0,001204829 | 0,128088396 | 0 | 0 |
| 11/73 | 0,001233767 | 0,128488035 | 0 | 0 |
| 28/292 | 0,001237167 | 0,126265267 | 0 | 0 |
| 5/17 | 0,001331814 | 0,133259776 | 0 | 0 |
| 24/238 | 0,001360996 | 0,133560861 | 0 | 0 |

| | | | | |
|---|---|---|---|---|
| 11/74 | 0,001382929 | 0,133152575 | 0 | 0 |
| 40/471 | 0,001423623 | 0,13453235 | 0 | 0 |
| 18/159 | 0,001453841 | 0,134889972 | 0 | 0 |
| 7/34 | 0,001506988 | 0,137324257 | 0 | 0 |
| 14/110 | 0,001570736 | 0,140622181 | 0 | 0 |
| 22/214 | 0,001653995 | 0,145523022 | 0 | 0 |
| 13/99 | 0,001712252 | 0,148095256 | 0 | 0 |
| 5/18 | 0,001766355 | 0,150228534 | 0 | 0 |
| 10/66 | 0,001944641 | 0,162680354 | 0 | 0 |
| 3/6 | 0,002435749 | 0,20047783 | 0 | 0 |
| 3/6 | 0,002435749 | 0,197295643 | 0 | 0 |
| 3/6 | 0,002435749 | 0,194212898 | 0 | 0 |
| 4/12 | 0,002499253 | 0,196210599 | 0 | 0 |
| 4/12 | 0,002499253 | 0,193237711 | 0 | 0 |
| 7/37 | 0,002520934 | 0,192004895 | 0 | 0 |
| 16/141 | 0,002539277 | 0,190557766 | 0 | 0 |
| 8/47 | 0,002556811 | 0,189092815 | 0 | 0 |
| 12/92 | 0,002686887 | 0,195874088 | 0 | 0 |
| 11/81 | 0,002892756 | 0,207911715 | 0 | 0 |
| 15/130 | 0,002901519 | 0,20564519 | 0 | 0 |
| 13/105 | 0,002911061 | 0,203495151 | 0 | 0 |
| 10/70 | 0,003038064 | 0,209503284 | 0 | 0 |
| 11/82 | 0,003188793 | 0,216965477 | 0 | 0 |
| 4/13 | 0,003463707 | 0,232569725 | 0 | 0 |
| 11/83 | 0,003508664 | 0,232528755 | 0 | 0 |
| 5/21 | 0,003688236 | 0,241295748 | 0 | 0 |
| 5/21 | 0,003688236 | 0,238241371 | 0 | 0 |
| 3/7 | 0,004099378 | 0,261489073 | 0 | 0 |
| 3/7 | 0,004099378 | 0,258260813 | 0 | 0 |
| 3/7 | 0,004099378 | 0,255111291 | 0 | 0 |
| 14/122 | 0,004139768 | 0,25452091 | 0 | 0 |
| 12/97 | 0,004176125 | 0,253699612 | 0 | 0 |
| 9/62 | 0,004326077 | 0,259717325 | 0 | 0 |
| 6/31 | 0,004512743 | 0,267773559 | 0 | 0 |
| 9/63 | 0,004821424 | 0,282801444 | 0 | 0 |
| 8/52 | 0,004878214 | 0,282880965 | 0 | 0 |
| 9/64 | 0,005359707 | 0,307309933 | 0 | 0 |
| 5/23 | 0,005598899 | 0,31745756 | 0 | 0 |
| 19/194 | 0,005612609 | 0,314737861 | 0 | 0 |
| 13/114 | 0,005915488 | 0,328116664 | 0 | 0 |
| 4/15 | 0,006088819 | 0,334099398 | 0 | 0 |
| 4/15 | 0,006088819 | 0,330545149 | 0 | 0 |
| 4/15 | 0,006088819 | 0,327065727 | 0 | 0 |
| 4/15 | 0,006088819 | 0,323658792 | 0 | 0 |
| 4/15 | 0,006088819 | 0,320322103 | 0 | 0 |
| 4/15 | 0,006088819 | 0,31705351 | 0 | 0 |
| 6/33 | 0,006219281 | 0,32057568 | 0 | 0 |
| 3/8 | 0,00630859 | 0,321927348 | 0 | 0 |
| 3/8 | 0,00630859 | 0,318739948 | 0 | 0 |
| 3/8 | 0,00630859 | 0,315615047 | 0 | 0 |
| 3/8 | 0,00630859 | 0,312550823 | 0 | 0 |
| 3/8 | 0,00630859 | 0,309545527 | 0 | 0 |
| 6/34 | 0,007229061 | 0,351332362 | 0 | 0 |

| | | | | |
|---|---|---|---|---|
| 6/34 | 0,007229061 | 0,348017906 | 0 | 0 |
| 12/104 | 0,007301214 | 0,348206502 | 0 | 0 |
| 8/56 | 0,007686724 | 0,363197707 | 0 | 0 |
| 23/258 | 0,007759174 | 0,363257463 | 0 | 0 |
| 7/45 | 0,00777582 | 0,360727359 | 0 | 0 |
| 4/16 | 0,007791259 | 0,358187334 | 0 | 0 |
| 4/16 | 0,007791259 | 0,354989232 | 0 | 0 |
| 4/16 | 0,007791259 | 0,351847735 | 0 | 0 |
| 4/16 | 0,007791259 | 0,348761351 | 0 | 0 |
| 30/364 | 0,007974428 | 0,353856565 | 0 | 0 |
| 9/68 | 0,007990561 | 0,351515818 | 0 | 0 |
| 15/145 | 0,008017605 | 0,349690943 | 0 | 0 |
| 5/25 | 0,008117773 | 0,351059301 | 0 | 0 |
| 6/35 | 0,008352105 | 0,358157904 | 0 | 0 |
| 32/397 | 0,008610809 | 0,366174643 | 0 | 0 |
| 3/9 | 0,009102597 | 0,383888882 | 0 | 0 |
| 32/399 | 0,009231829 | 0,386147717 | 0 | 0 |
| 23/262 | 0,009256939 | 0,384050068 | 0 | 0 |
| 10/82 | 0,00942286 | 0,387781085 | 0 | 0 |
| 6/36 | 0,009594991 | 0,39170592 | 0 | 0 |
| 5/26 | 0,009632009 | 0,390096361 | 0 | 0 |
| 5/26 | 0,009632009 | 0,387024736 | 0 | 0 |
| 5/26 | 0,009632009 | 0,384001105 | 0 | 0 |
| 4/17 | 0,009778786 | 0,386830574 | 0 | 0 |
| 4/17 | 0,009778786 | 0,383854954 | 0 | 0 |
| 4/17 | 0,009778786 | 0,380924763 | 0 | 0 |
| 4/17 | 0,009778786 | 0,37803897 | 0 | 0 |
| 4/17 | 0,009778786 | 0,375196571 | 0 | 0 |
| 4/17 | 0,009778786 | 0,372396597 | 0 | 0 |
| 7/47 | 0,009860816 | 0,372738832 | 0 | 0 |
| 10/83 | 0,010233025 | 0,38396417 | 0 | 0 |
| 8/59 | 0,010483678 | 0,390497879 | 0 | 0 |
| 9/71 | 0,01053461 | 0,389551544 | 0 | 0 |
| 6/37 | 0,010964166 | 0,402518998 | 0 | 0 |
| 7/48 | 0,011043783 | 0,402545886 | 0 | 0 |
| 5/27 | 0,011329548 | 0,410033213 | 0 | 0 |
| 5/27 | 0,011329548 | 0,407145655 | 0 | 0 |
| 21/237 | 0,011332424 | 0,404401132 | 0 | 0 |
| 17/179 | 0,011358026 | 0,402500031 | 0 | 0 |
| 8/60 | 0,011565043 | 0,407009738 | 0 | 0 |
| 10/85 | 0,01201107 | 0,419811567 | 0 | 0 |
| 4/18 | 0,012068057 | 0,418933994 | 0 | 0 |
| 4/18 | 0,012068057 | 0,416103359 | 0 | 0 |
| 4/18 | 0,012068057 | 0,413310719 | 0 | 0 |
| 11/98 | 0,012147435 | 0,413255729 | 0 | 0 |
| 11/98 | 0,012147435 | 0,410518936 | 0 | 0 |
| 40/535 | 0,012228069 | 0,410525233 | 0 | 0 |
| 20/224 | 0,012252952 | 0,408671978 | 0 | 0 |
| 7/49 | 0,012326291 | 0,408448465 | 0 | 0 |
| 24/284 | 0,012388756 | 0,407869804 | 0 | 0 |
| 6/38 | 0,012465912 | 0,407779153 | 0 | 0 |
| 6/38 | 0,012465912 | 0,405181833 | 0 | 0 |
| 3/10 | 0,01250999 | 0,404040998 | 0 | 0 |

| | | | | |
|---|---|---|---|---|
| 3/10 | 0,01250999 | 0,40149986 | 0 | 0 |
| 3/10 | 0,01250999 | 0,398990485 | 0 | 0 |
| 3/10 | 0,01250999 | 0,396512284 | 0 | 0 |
| 3/10 | 0,01250999 | 0,394064677 | 0 | 0 |
| 3/10 | 0,01250999 | 0,391647102 | 0 | 0 |
| 3/10 | 0,01250999 | 0,38925901 | 0 | 0 |
| 3/10 | 0,01250999 | 0,386899865 | 0 | 0 |
| 3/10 | 0,01250999 | 0,384569143 | 0 | 0 |
| 70/1038 | 0,013059602 | 0,399060758 | 0 | 0 |
| 5/28 | 0,013219845 | 0,401552803 | 0 | 0 |
| 14/140 | 0,013496441 | 0,40752863 | 0 | 0 |
| 26/317 | 0,013618917 | 0,408807847 | 0 | 0 |
| 9/74 | 0,013643204 | 0,407141942 | 0 | 0 |
| 7/50 | 0,013712671 | 0,406835802 | 0 | 0 |
| 8/62 | 0,013970851 | 0,41209972 | 0 | 0 |
| 4/19 | 0,014673856 | 0,430348766 | 0 | 0 |
| 4/19 | 0,014673856 | 0,42788963 | 0 | 0 |
| 4/19 | 0,014673856 | 0,425458439 | 0 | 0 |
| 9/75 | 0,014816518 | 0,427167743 | 0 | 0 |
| 11/101 | 0,015004677 | 0,430162184 | 0 | 0 |
| 5/29 | 0,01531179 | 0,436514328 | 0 | 0 |
| 5/29 | 0,01531179 | 0,434089249 | 0 | 0 |
| 5/29 | 0,01531179 | 0,431690966 | 0 | 0 |
| 5/29 | 0,01531179 | 0,429319037 | 0 | 0 |
| 6/40 | 0,015891236 | 0,443131024 | 0 | 0 |
| 3/11 | 0,016549986 | 0,458992281 | 0 | 0 |
| 3/11 | 0,016549986 | 0,456511242 | 0 | 0 |
| 8/64 | 0,016721962 | 0,45877513 | 0 | 0 |
| 22/262 | 0,017248937 | 0,470702272 | 0 | 0 |
| 9/77 | 0,017383557 | 0,4718526 | 0 | 0 |
| 10/90 | 0,017469059 | 0,471664585 | 0 | 0 |
| 4/20 | 0,01760904 | 0,472941743 | 0 | 0 |
| 4/20 | 0,01760904 | 0,470465608 | 0 | 0 |
| 4/20 | 0,01760904 | 0,468015266 | 0 | 0 |
| 4/20 | 0,01760904 | 0,465590317 | 0 | 0 |
| 30/387 | 0,017615997 | 0,463373373 | 0 | 0 |
| 8/65 | 0,018234756 | 0,477189551 | 0 | 0 |
| 9/78 | 0,018782087 | 0,489005048 | 0 | 0 |
| 12/119 | 0,019963222 | 0,517118394 | 0 | 0 |
| 12/119 | 0,019963222 | 0,514506685 | 0 | 0 |
| 5/31 | 0,020133068 | 0,516276621 | 0 | 0 |
| 5/31 | 0,020133068 | 0,513695238 | 0 | 0 |
| 5/31 | 0,020133068 | 0,51113954 | 0 | 0 |
| 5/31 | 0,020133068 | 0,508609147 | 0 | 0 |
| 5/31 | 0,020133068 | 0,506103683 | 0 | 0 |
| 5/31 | 0,020133068 | 0,503622783 | 0 | 0 |
| 7/54 | 0,020379948 | 0,507311573 | 0 | 0 |
| 11/106 | 0,020850406 | 0,516503013 | 0 | 0 |
| 4/21 | 0,02088453 | 0,514849059 | 0 | 0 |
| 4/21 | 0,02088453 | 0,512373823 | 0 | 0 |
| 4/21 | 0,02088453 | 0,509922274 | 0 | 0 |
| 4/21 | 0,02088453 | 0,507494072 | 0 | 0 |
| 12/120 | 0,021178759 | 0,512204763 | 0 | 0 |

| | | | | |
|---|---|---|---|---|
| 3/12 | 0,021233575 | 0,511108173 | 0 | 0 |
| 3/12 | 0,021233575 | 0,508708604 | 0 | 0 |
| 3/12 | 0,021233575 | 0,506331461 | 0 | 0 |
| 9/80 | 0,021820871 | 0,517915828 | 0 | 0 |
| 11/107 | 0,022196407 | 0,524390126 | 0 | 0 |
| 11/107 | 0,022196407 | 0,521973582 | 0 | 0 |
| 12/121 | 0,022448303 | 0,525475654 | 0 | 0 |
| 5/32 | 0,022876958 | 0,533064459 | 0 | 0 |
| 5/32 | 0,022876958 | 0,530641439 | 0 | 0 |
| 8/68 | 0,023357617 | 0,539338993 | 0 | 0 |
| 9/81 | 0,023465656 | 0,539392976 | 0 | 0 |
| 9/81 | 0,023465656 | 0,536974174 | 0 | 0 |
| 4/22 | 0,024509309 | 0,558352705 | 0 | 0 |
| 4/22 | 0,024509309 | 0,555871137 | 0 | 0 |
| 4/22 | 0,024509309 | 0,55341153 | 0 | 0 |
| 4/22 | 0,024509309 | 0,550973594 | 0 | 0 |
| 6/44 | 0,024584124 | 0,550231504 | 0 | 0 |
| 5/33 | 0,025851548 | 0,576071825 | 0 | 0 |
| 3/13 | 0,026564546 | 0,589386431 | 0 | 0 |
| 3/13 | 0,026564546 | 0,586834975 | 0 | 0 |
| 3/13 | 0,026564546 | 0,584305514 | 0 | 0 |
| 3/13 | 0,026564546 | 0,581797765 | 0 | 0 |
| 3/13 | 0,026564546 | 0,579311449 | 0 | 0 |
| 3/13 | 0,026564546 | 0,576846294 | 0 | 0 |
| 3/13 | 0,026564546 | 0,57440203 | 0 | 0 |
| 3/13 | 0,026564546 | 0,571978393 | 0 | 0 |
| 3/13 | 0,026564546 | 0,569575123 | 0 | 0 |
| 3/13 | 0,026564546 | 0,567191963 | 0 | 0 |
| 9/83 | 0,027016901 | 0,574446867 | 0 | 0 |
| 6/45 | 0,02717002 | 0,575305445 | 0 | 0 |
| 6/45 | 0,02717002 | 0,57292815 | 0 | 0 |
| 6/45 | 0,02717002 | 0,570570421 | 0 | 0 |
| 8/70 | 0,027287786 | 0,570694961 | 0 | 0 |
| 4/23 | 0,028490455 | 0,593415479 | 0 | 0 |
| 4/23 | 0,028490455 | 0,591003221 | 0 | 0 |
| 4/23 | 0,028490455 | 0,588610495 | 0 | 0 |
| 4/23 | 0,028490455 | 0,586237066 | 0 | 0 |
| 4/23 | 0,028490455 | 0,5838827 | 0 | 0 |
| 9/84 | 0,02892752 | 0,590468541 | 0 | 0 |
| 5/34 | 0,029062326 | 0,59085677 | 0 | 0 |
| 5/34 | 0,029062326 | 0,5885121 | 0 | 0 |
| 5/34 | 0,029062326 | 0,586185965 | 0 | 0 |
| 8/71 | 0,029415176 | 0,59096709 | 0 | 0 |
| 10/98 | 0,02971262 | 0,594601963 | 0 | 0 |
| 7/59 | 0,031521278 | 0,628332349 | 0 | 0 |
| 5/35 | 0,032514034 | 0,645599674 | 0 | 0 |
| 5/35 | 0,032514034 | 0,64309735 | 0 | 0 |
| 5/35 | 0,032514034 | 0,640614349 | 0 | 0 |
| 5/35 | 0,032514034 | 0,638150447 | 0 | 0 |
| 3/14 | 0,03254043 | 0,636221509 | 0 | 0 |
| 3/14 | 0,03254043 | 0,633793182 | 0 | 0 |
| 3/14 | 0,03254043 | 0,631383322 | 0 | 0 |
| 3/14 | 0,03254043 | 0,628991719 | 0 | 0 |

| | | | | |
|---|---|---|---|---|
| 3/14 | 0,03254043 | 0,626618165 | 0 | 0 |
| 3/14 | 0,03254043 | 0,624262458 | 0 | 0 |
| 3/14 | 0,03254043 | 0,621924396 | 0 | 0 |
| 3/14 | 0,03254043 | 0,619603783 | 0 | 0 |
| 3/14 | 0,03254043 | 0,617300423 | 0 | 0 |
| 3/14 | 0,03254043 | 0,615014125 | 0 | 0 |
| 3/14 | 0,03254043 | 0,6127447 | 0 | 0 |
| 3/14 | 0,03254043 | 0,610491962 | 0 | 0 |
| 3/14 | 0,03254043 | 0,608255728 | 0 | 0 |
| 6/47 | 0,032866475 | 0,612108108 | 0 | 0 |
| 6/47 | 0,032866475 | 0,609882261 | 0 | 0 |
| 6/47 | 0,032866475 | 0,607672542 | 0 | 0 |
| 16/187 | 0,032921254 | 0,606487947 | 0 | 0 |
| 15/173 | 0,034408026 | 0,631597678 | 0 | 0 |
| 12/129 | 0,034699522 | 0,634665457 | 0 | 0 |
| 2/6 | 0,034729741 | 0,632949528 | 0 | 0 |
| 2/6 | 0,034729741 | 0,630697038 | 0 | 0 |
| 2/6 | 0,034729741 | 0,628460524 | 0 | 0 |
| 2/6 | 0,034729741 | 0,626239816 | 0 | 0 |
| 2/6 | 0,034729741 | 0,624034746 | 0 | 0 |
| 2/6 | 0,034729741 | 0,62184515 | 0 | 0 |
| 2/6 | 0,034729741 | 0,619670866 | 0 | 0 |
| 2/6 | 0,034729741 | 0,617511734 | 0 | 0 |
| 2/6 | 0,034729741 | 0,615367596 | 0 | 0 |
| 2/6 | 0,034729741 | 0,613238297 | 0 | 0 |
| 2/6 | 0,034729741 | 0,611123682 | 0 | 0 |
| 2/6 | 0,034729741 | 0,609023601 | 0 | 0 |
| 2/6 | 0,034729741 | 0,606937903 | 0 | 0 |
| 2/6 | 0,034729741 | 0,604866443 | 0 | 0 |
| 2/6 | 0,034729741 | 0,602809074 | 0 | 0 |
| 2/6 | 0,034729741 | 0,600765654 | 0 | 0 |
| 2/6 | 0,034729741 | 0,59873604 | 0 | 0 |
| 2/6 | 0,034729741 | 0,596720094 | 0 | 0 |
| 2/6 | 0,034729741 | 0,594717677 | 0 | 0 |
| 2/6 | 0,034729741 | 0,592728655 | 0 | 0 |
| 2/6 | 0,034729741 | 0,590752893 | 0 | 0 |
| 2/6 | 0,034729741 | 0,588790258 | 0 | 0 |
| 2/6 | 0,034729741 | 0,586840622 | 0 | 0 |
| 2/6 | 0,034729741 | 0,584903854 | 0 | 0 |
| 2/6 | 0,034729741 | 0,582979828 | 0 | 0 |
| 2/6 | 0,034729741 | 0,581068419 | 0 | 0 |
| 2/6 | 0,034729741 | 0,579169503 | 0 | 0 |
| 10/101 | 0,035575323 | 0,591338346 | 0 | 0 |
| 5/36 | 0,036210659 | 0,599944777 | 0 | 0 |
| 5/36 | 0,036210659 | 0,598003208 | 0 | 0 |
| 5/36 | 0,036210659 | 0,596074166 | 0 | 0 |
| 8/74 | 0,036474739 | 0,598490655 | 0 | 0 |
| 7/61 | 0,03693506 | 0,604101317 | 0 | 0 |
| 16/190 | 0,037291438 | 0,60798149 | 0 | 0 |
| 4/25 | 0,03754088 | 0,610099086 | 0 | 0 |
| 30/413 | 0,037819709 | 0,612679286 | 0 | 0 |
| 12/131 | 0,038387538 | 0,619910147 | 0 | 0 |
| 3/15 | 0,039153343 | 0,63028237 | 0 | 0 |

| | | | | |
|---|---|---|---|---|
| 3/15 | 0,039153343 | 0,62830035 | 0 | 0 |
| 3/15 | 0,039153343 | 0,626330756 | 0 | 0 |
| 3/15 | 0,039153343 | 0,624373473 | 0 | 0 |
| 3/15 | 0,039153343 | 0,622428384 | 0 | 0 |
| 3/15 | 0,039153343 | 0,620495377 | 0 | 0 |
| 3/15 | 0,039153343 | 0,618574338 | 0 | 0 |
| 3/15 | 0,039153343 | 0,616665158 | 0 | 0 |
| 3/15 | 0,039153343 | 0,614767727 | 0 | 0 |
| 3/15 | 0,039153343 | 0,612881937 | 0 | 0 |
| 3/15 | 0,039153343 | 0,61100768 | 0 | 0 |
| 14/161 | 0,039208539 | 0,610003587 | 0 | 0 |
| 6/49 | 0,039285086 | 0,609336762 | 0 | 0 |
| 11/117 | 0,039333505 | 0,608239026 | 0 | 0 |
| 9/89 | 0,039906809 | 0,615240025 | 0 | 0 |
| 5/37 | 0,040155428 | 0,617208278 | 0 | 0 |
| 5/37 | 0,040155428 | 0,6153548 | 0 | 0 |
| 8/76 | 0,041766483 | 0,638126835 | 0 | 0 |
| 18/224 | 0,042182131 | 0,642553479 | 0 | 0 |
| 4/26 | 0,042615227 | 0,647218765 | 0 | 0 |
| 4/26 | 0,042615227 | 0,645298235 | 0 | 0 |
| 13/148 | 0,042752364 | 0,645459504 | 0 | 0 |
| 6/50 | 0,042772237 | 0,643854652 | 0 | 0 |
| 5/38 | 0,044350814 | 0,665653537 | 0 | 0 |
| 5/38 | 0,044350814 | 0,663701474 | 0 | 0 |
| 5/38 | 0,044350814 | 0,661760826 | 0 | 0 |
| 5/38 | 0,044350814 | 0,659831495 | 0 | 0 |
| 10/105 | 0,044579593 | 0,661307162 | 0 | 0 |
| 8/77 | 0,044593491 | 0,659595905 | 0 | 0 |
| 11/120 | 0,045898241 | 0,676932724 | 0 | 0 |
| 7/64 | 0,046145498 | 0,678618086 | 0 | 0 |
| 7/64 | 0,046145498 | 0,676668034 | 0 | 0 |
| 3/16 | 0,046390758 | 0,678315296 | 0 | 0 |
| 3/16 | 0,046390758 | 0,676377252 | 0 | 0 |
| 3/16 | 0,046390758 | 0,674450252 | 0 | 0 |
| 3/16 | 0,046390758 | 0,6725342 | 0 | 0 |
| 3/16 | 0,046390758 | 0,670629004 | 0 | 0 |
| 3/16 | 0,046390758 | 0,668734571 | 0 | 0 |
| 3/16 | 0,046390758 | 0,666850812 | 0 | 0 |
| 3/16 | 0,046390758 | 0,664977636 | 0 | 0 |
| 2/7 | 0,04698191 | 0,671564944 | 0 | 0 |
| 2/7 | 0,04698191 | 0,669689065 | 0 | 0 |
| 2/7 | 0,04698191 | 0,667823636 | 0 | 0 |
| 2/7 | 0,04698191 | 0,66596857 | 0 | 0 |
| 2/7 | 0,04698191 | 0,664123782 | 0 | 0 |
| 2/7 | 0,04698191 | 0,662289186 | 0 | 0 |
| 2/7 | 0,04698191 | 0,660464697 | 0 | 0 |
| 2/7 | 0,04698191 | 0,658650234 | 0 | 0 |
| 2/7 | 0,04698191 | 0,656845713 | 0 | 0 |
| 2/7 | 0,04698191 | 0,655051052 | 0 | 0 |
| 2/7 | 0,04698191 | 0,653266172 | 0 | 0 |
| 2/7 | 0,04698191 | 0,651490992 | 0 | 0 |
| 2/7 | 0,04698191 | 0,649725434 | 0 | 0 |
| 2/7 | 0,04698191 | 0,647969419 | 0 | 0 |

| | | | | |
|---|---|---|---|---|
| 2/7 | 0,04698191 | 0,646222871 | 0 | 0 |
| 2/7 | 0,04698191 | 0,644485713 | 0 | 0 |
| 2/7 | 0,04698191 | 0,642757869 | 0 | 0 |
| 2/7 | 0,04698191 | 0,641039265 | 0 | 0 |
| 2/7 | 0,04698191 | 0,639329827 | 0 | 0 |
| 2/7 | 0,04698191 | 0,637629482 | 0 | 0 |
| 2/7 | 0,04698191 | 0,635938157 | 0 | 0 |
| 2/7 | 0,04698191 | 0,634255781 | 0 | 0 |
| 10/106 | 0,047051385 | 0,63351773 | 0 | 0 |
| 4/27 | 0,048056222 | 0,64534447 | 0 | 0 |
| 4/27 | 0,048056222 | 0,643650653 | 0 | 0 |
| 5/39 | 0,048798539 | 0,651882053 | 0 | 0 |
| 6/52 | 0,050312853 | 0,670356372 | 0 | 0 |
| 6/52 | 0,050312853 | 0,668610652 | 0 | 0 |
| 6/52 | 0,050312853 | 0,666874001 | 0 | 0 |
| 11/122 | 0,050668201 | 0,669844125 | 0 | 0 |
| 14/168 | 0,052731059 | 0,695314195 | 0 | 0 |
| 7/66 | 0,053034329 | 0,697510777 | 0 | 0 |
| 5/40 | 0,053499587 | 0,701821058 | 0 | 0 |
| 8/80 | 0,053817134 | 0,7041765 | 0 | 0 |
| 4/28 | 0,053862291 | 0,702964883 | 0 | 0 |
| 4/28 | 0,053862291 | 0,701171605 | 0 | 0 |
| 4/28 | 0,053862291 | 0,699387454 | 0 | 0 |
| 3/17 | 0,054236191 | 0,70245503 | 0 | 0 |
| 3/17 | 0,054236191 | 0,700676663 | 0 | 0 |
| 3/17 | 0,054236191 | 0,698907278 | 0 | 0 |
| 3/17 | 0,054236191 | 0,697146806 | 0 | 0 |
| 3/17 | 0,054236191 | 0,695395181 | 0 | 0 |
| 3/17 | 0,054236191 | 0,693652336 | 0 | 0 |
| 3/17 | 0,054236191 | 0,691918205 | 0 | 0 |
| 3/17 | 0,054236191 | 0,690192723 | 0 | 0 |
| 3/17 | 0,054236191 | 0,688475826 | 0 | 0 |
| 3/17 | 0,054236191 | 0,686767449 | 0 | 0 |
| 6/53 | 0,054369367 | 0,686749699 | 0 | 0 |
| 29/412 | 0,056507169 | 0,711990326 | 0 | 0 |
| 7/67 | 0,056707536 | 0,712755061 | 0 | 0 |
| 7/67 | 0,056707536 | 0,71100382 | 0 | 0 |
| 8/81 | 0,057143326 | 0,714711745 | 0 | 0 |
| 10/110 | 0,057854045 | 0,721831761 | 0 | 0 |
| 12/140 | 0,058397767 | 0,72683855 | 0 | 0 |
| 5/41 | 0,058454216 | 0,725770958 | 0 | 0 |
| 5/41 | 0,058454216 | 0,724009378 | 0 | 0 |
| 5/41 | 0,058454216 | 0,722256328 | 0 | 0 |
| 5/41 | 0,058454216 | 0,720511748 | 0 | 0 |
| 5/41 | 0,058454216 | 0,718775575 | 0 | 0 |
| 5/41 | 0,058454216 | 0,717047749 | 0 | 0 |
| 63/1002 | 0,059901579 | 0,733040183 | 0 | 0 |
| 4/29 | 0,060030377 | 0,732858885 | 0 | 0 |
| 4/29 | 0,060030377 | 0,731109818 | 0 | 0 |
| 4/29 | 0,060030377 | 0,72936908 | 0 | 0 |
| 4/29 | 0,060030377 | 0,727636612 | 0 | 0 |
| 4/29 | 0,060030377 | 0,725912355 | 0 | 0 |
| 4/29 | 0,060030377 | 0,72419625 | 0 | 0 |

| | | | | |
|---|---|---|---|---|
| 4/29 | 0,060030377 | 0,72248824 | 0 | 0 |
| 4/29 | 0,060030377 | 0,720788268 | 0 | 0 |
| 4/29 | 0,060030377 | 0,719096277 | 0 | 0 |
| 4/29 | 0,060030377 | 0,71741221 | 0 | 0 |
| 2/8 | 0,060539704 | 0,72180867 | 0 | 0 |
| 2/8 | 0,060539704 | 0,720126133 | 0 | 0 |
| 2/8 | 0,060539704 | 0,718451421 | 0 | 0 |
| 2/8 | 0,060539704 | 0,71678448 | 0 | 0 |
| 2/8 | 0,060539704 | 0,715125257 | 0 | 0 |
| 2/8 | 0,060539704 | 0,713473697 | 0 | 0 |
| 2/8 | 0,060539704 | 0,711829749 | 0 | 0 |
| 2/8 | 0,060539704 | 0,710193359 | 0 | 0 |
| 2/8 | 0,060539704 | 0,708564475 | 0 | 0 |
| 2/8 | 0,060539704 | 0,706943046 | 0 | 0 |
| 2/8 | 0,060539704 | 0,70532902 | 0 | 0 |
| 2/8 | 0,060539704 | 0,703722348 | 0 | 0 |
| 2/8 | 0,060539704 | 0,702122979 | 0 | 0 |
| 2/8 | 0,060539704 | 0,700530864 | 0 | 0 |
| 2/8 | 0,060539704 | 0,698945952 | 0 | 0 |
| 2/8 | 0,060539704 | 0,697368196 | 0 | 0 |
| 2/8 | 0,060539704 | 0,695797547 | 0 | 0 |
| 2/8 | 0,060539704 | 0,694233957 | 0 | 0 |
| 2/8 | 0,060539704 | 0,692677379 | 0 | 0 |
| 2/8 | 0,060539704 | 0,691127765 | 0 | 0 |
| 2/8 | 0,060539704 | 0,689585069 | 0 | 0 |
| 16/203 | 0,061119926 | 0,694643614 | 0 | 0 |
| 3/18 | 0,062669827 | 0,710675841 | 0 | 0 |
| 3/18 | 0,062669827 | 0,709100064 | 0 | 0 |
| 3/18 | 0,062669827 | 0,707531258 | 0 | 0 |
| 3/18 | 0,062669827 | 0,705969379 | 0 | 0 |
| 3/18 | 0,062669827 | 0,70441438 | 0 | 0 |
| 3/18 | 0,062669827 | 0,702866217 | 0 | 0 |
| 3/18 | 0,062669827 | 0,701324844 | 0 | 0 |
| 5/42 | 0,063661977 | 0,710868861 | 0 | 0 |
| 5/42 | 0,063661977 | 0,709316746 | 0 | 0 |
| 5/42 | 0,063661977 | 0,707771393 | 0 | 0 |
| 5/42 | 0,063661977 | 0,70623276 | 0 | 0 |
| 8/83 | 0,064178868 | 0,710422478 | 0 | 0 |
| 7/69 | 0,06451613 | 0,712609982 | 0 | 0 |
| 7/69 | 0,06451613 | 0,711070867 | 0 | 0 |
| 4/30 | 0,066556032 | 0,731972915 | 0 | 0 |
| 4/30 | 0,066556032 | 0,73039878 | 0 | 0 |
| 4/30 | 0,066556032 | 0,7288314 | 0 | 0 |
| 11/128 | 0,066941775 | 0,731485818 | 0 | 0 |
| 6/56 | 0,067693612 | 0,738120735 | 0 | 0 |
| 6/56 | 0,067693612 | 0,736546917 | 0 | 0 |
| 8/84 | 0,067889538 | 0,737107051 | 0 | 0 |
| 14/175 | 0,069159965 | 0,749306372 | 0 | 0 |
| 10/114 | 0,07016116 | 0,758543223 | 0 | 0 |
| 3/19 | 0,071669081 | 0,773207868 | 0 | 0 |
| 3/19 | 0,071669081 | 0,771576628 | 0 | 0 |
| 3/19 | 0,071669081 | 0,769952256 | 0 | 0 |
| 3/19 | 0,071669081 | 0,768334709 | 0 | 0 |

| | | | | |
|---|---|---|---|---|
| 3/19 | 0,071669081 | 0,766723945 | 0 | 0 |
| 3/19 | 0,071669081 | 0,76511992 | 0 | 0 |
| 7/71 | 0,072944213 | 0,77710714 | 0 | 0 |
| 4/31 | 0,073433514 | 0,780690048 | 0 | 0 |
| 4/31 | 0,073433514 | 0,779066992 | 0 | 0 |
| 4/31 | 0,073433514 | 0,777450671 | 0 | 0 |
| 4/31 | 0,073433514 | 0,775841042 | 0 | 0 |
| 4/31 | 0,073433514 | 0,774238065 | 0 | 0 |
| 21/290 | 0,074508007 | 0,783947133 | 0 | 0 |
| 5/44 | 0,074831689 | 0,785732737 | 0 | 0 |
| 5/44 | 0,074831689 | 0,784119323 | 0 | 0 |
| 2/9 | 0,07523604 | 0,786740799 | 0 | 0 |
| 2/9 | 0,07523604 | 0,785131923 | 0 | 0 |
| 2/9 | 0,07523604 | 0,783529613 | 0 | 0 |
| 2/9 | 0,07523604 | 0,781933829 | 0 | 0 |
| 2/9 | 0,07523604 | 0,780344533 | 0 | 0 |
| 2/9 | 0,07523604 | 0,778761684 | 0 | 0 |
| 2/9 | 0,07523604 | 0,777185243 | 0 | 0 |
| 2/9 | 0,07523604 | 0,775615172 | 0 | 0 |
| 2/9 | 0,07523604 | 0,774051432 | 0 | 0 |
| 2/9 | 0,07523604 | 0,772493984 | 0 | 0 |
| 2/9 | 0,07523604 | 0,770942791 | 0 | 0 |
| 2/9 | 0,07523604 | 0,769397816 | 0 | 0 |
| 2/9 | 0,07523604 | 0,76785902 | 0 | 0 |
| 2/9 | 0,07523604 | 0,766326368 | 0 | 0 |
| 2/9 | 0,07523604 | 0,764799821 | 0 | 0 |
| 2/9 | 0,07523604 | 0,763279344 | 0 | 0 |
| 2/9 | 0,07523604 | 0,761764901 | 0 | 0 |
| 2/9 | 0,07523604 | 0,760256456 | 0 | 0 |
| 2/9 | 0,07523604 | 0,758753973 | 0 | 0 |
| 2/9 | 0,07523604 | 0,757257416 | 0 | 0 |
| 2/9 | 0,07523604 | 0,755766752 | 0 | 0 |
| 9/101 | 0,076834299 | 0,770305364 | 0 | 0 |
| 29/425 | 0,076862797 | 0,769080101 | 0 | 0 |
| 10/116 | 0,076890741 | 0,767854116 | 0 | 0 |
| 7/72 | 0,077390856 | 0,771338943 | 0 | 0 |
| 7/72 | 0,077390856 | 0,769835358 | 0 | 0 |
| 6/58 | 0,07753903 | 0,769808692 | 0 | 0 |
| 6/58 | 0,07753903 | 0,768313918 | 0 | 0 |
| 22/308 | 0,07759182 | 0,767347007 | 0 | 0 |
| 9/102 | 0,080621837 | 0,795770283 | 0 | 0 |
| 4/32 | 0,080655885 | 0,794569463 | 0 | 0 |
| 4/32 | 0,080655885 | 0,7930385 | 0 | 0 |
| 4/32 | 0,080655885 | 0,791513426 | 0 | 0 |
| 5/45 | 0,080789402 | 0,791301955 | 0 | 0 |
| 5/45 | 0,080789402 | 0,789786051 | 0 | 0 |
| 5/45 | 0,080789402 | 0,788275944 | 0 | 0 |
| 5/45 | 0,080789402 | 0,7867716 | 0 | 0 |
| 3/20 | 0,081209097 | 0,789352426 | 0 | 0 |
| 3/20 | 0,081209097 | 0,787851756 | 0 | 0 |
| 3/20 | 0,081209097 | 0,786356781 | 0 | 0 |
| 3/20 | 0,081209097 | 0,784867469 | 0 | 0 |
| 3/20 | 0,081209097 | 0,783383788 | 0 | 0 |

| | | | | |
|---|---|---|---|---|
| 3/20 | 0,081209097 | 0,781905705 | 0 | 0 |
| 3/20 | 0,081209097 | 0,780433189 | 0 | 0 |
| 3/20 | 0,081209097 | 0,77896621 | 0 | 0 |
| 3/20 | 0,081209097 | 0,777504735 | 0 | 0 |
| 7/73 | 0,081992234 | 0,783532526 | 0 | 0 |
| 7/73 | 0,081992234 | 0,78206798 | 0 | 0 |
| 7/73 | 0,081992234 | 0,780608898 | 0 | 0 |
| 7/73 | 0,081992234 | 0,77915525 | 0 | 0 |
| 47/741 | 0,083934293 | 0,796127688 | 0 | 0 |
| 8/88 | 0,084025832 | 0,79551729 | 0 | 0 |
| 12/149 | 0,084386427 | 0,797451734 | 0 | 0 |
| 23/328 | 0,084638805 | 0,798358268 | 0 | 0 |
| 10/119 | 0,08771202 | 0,825819992 | 0 | 0 |
| 6/60 | 0,088147657 | 0,828393174 | 0 | 0 |
| 6/60 | 0,088147657 | 0,826870392 | 0 | 0 |
| 6/60 | 0,088147657 | 0,825353199 | 0 | 0 |
| 6/60 | 0,088147657 | 0,823841563 | 0 | 0 |
| 4/33 | 0,088215109 | 0,822964723 | 0 | 0 |
| 4/33 | 0,088215109 | 0,821462963 | 0 | 0 |
| 4/33 | 0,088215109 | 0,819966673 | 0 | 0 |
| 4/33 | 0,088215109 | 0,818475824 | 0 | 0 |
| 23/330 | 0,088976948 | 0,824046035 | 0 | 0 |
| 2/10 | 0,090917569 | 0,840493396 | 0 | 0 |
| 2/10 | 0,090917569 | 0,838973516 | 0 | 0 |
| 2/10 | 0,090917569 | 0,837459123 | 0 | 0 |
| 2/10 | 0,090917569 | 0,835950188 | 0 | 0 |
| 2/10 | 0,090917569 | 0,83444668 | 0 | 0 |
| 2/10 | 0,090917569 | 0,832948571 | 0 | 0 |
| 2/10 | 0,090917569 | 0,831455832 | 0 | 0 |
| 2/10 | 0,090917569 | 0,829968433 | 0 | 0 |
| 2/10 | 0,090917569 | 0,828486347 | 0 | 0 |
| 2/10 | 0,090917569 | 0,827009544 | 0 | 0 |
| 2/10 | 0,090917569 | 0,825537997 | 0 | 0 |
| 2/10 | 0,090917569 | 0,824071677 | 0 | 0 |
| 2/10 | 0,090917569 | 0,822610557 | 0 | 0 |
| 2/10 | 0,090917569 | 0,821154609 | 0 | 0 |
| 2/10 | 0,090917569 | 0,819703806 | 0 | 0 |
| 2/10 | 0,090917569 | 0,81825812 | 0 | 0 |
| 2/10 | 0,090917569 | 0,816817525 | 0 | 0 |
| 2/10 | 0,090917569 | 0,815381994 | 0 | 0 |
| 2/10 | 0,090917569 | 0,813951499 | 0 | 0 |
| 2/10 | 0,090917569 | 0,812526015 | 0 | 0 |
| 3/21 | 0,0912632 | 0,814189004 | 0 | 0 |
| 3/21 | 0,0912632 | 0,812768081 | 0 | 0 |
| 3/21 | 0,0912632 | 0,811352109 | 0 | 0 |
| 3/21 | 0,0912632 | 0,809941061 | 0 | 0 |
| 10/120 | 0,091513274 | 0,81075041 | 0 | 0 |
| 17/232 | 0,092745382 | 0,820242086 | 0 | 0 |
| 5/47 | 0,093435321 | 0,824914262 | 0 | 0 |
| 5/47 | 0,093435321 | 0,82348954 | 0 | 0 |
| 5/47 | 0,093435321 | 0,82206973 | 0 | 0 |
| 10/121 | 0,095411477 | 0,838011647 | 0 | 0 |
| 10/121 | 0,095411477 | 0,836571765 | 0 | 0 |

| | | | | |
|---|---|---|---|---|
| 10/121 | 0,095411477 | 0,835136822 | 0 | 0 |
| 4/34 | 0,096102152 | 0,839741924 | 0 | 0 |
| 7/76 | 0,096718088 | 0,843679321 | 0 | 0 |
| 8/91 | 0,097483801 | 0,848907571 | 0 | 0 |
| 8/91 | 0,097483801 | 0,847461391 | 0 | 0 |
| 5/48 | 0,10011571 | 0,868861343 | 0 | 0 |
| 11/138 | 0,100857952 | 0,873816859 | 0 | 0 |
| 3/22 | 0,101803296 | 0,880512235 | 0 | 0 |
| 3/22 | 0,101803296 | 0,879022366 | 0 | 0 |
| 3/22 | 0,101803296 | 0,877537531 | 0 | 0 |
| 3/22 | 0,101803296 | 0,876057704 | 0 | 0 |
| 3/22 | 0,101803296 | 0,874582859 | 0 | 0 |
| 3/22 | 0,101803296 | 0,873112972 | 0 | 0 |
| 3/22 | 0,101803296 | 0,871648017 | 0 | 0 |
| 3/22 | 0,101803296 | 0,87018797 | 0 | 0 |
| 3/22 | 0,101803296 | 0,868732807 | 0 | 0 |
| 3/22 | 0,101803296 | 0,867282501 | 0 | 0 |
| 3/22 | 0,101803296 | 0,865837031 | 0 | 0 |
| 3/22 | 0,101803296 | 0,86439637 | 0 | 0 |
| 3/22 | 0,101803296 | 0,862960496 | 0 | 0 |
| 3/22 | 0,101803296 | 0,861529384 | 0 | 0 |
| 4/35 | 0,104307076 | 0,881256635 | 0 | 0 |
| 4/35 | 0,104307076 | 0,879800012 | 0 | 0 |
| 4/35 | 0,104307076 | 0,878348197 | 0 | 0 |
| 4/35 | 0,104307076 | 0,876901165 | 0 | 0 |
| 6/63 | 0,105462757 | 0,885158631 | 0 | 0 |
| 9/108 | 0,105672448 | 0,885462236 | 0 | 0 |
| 8/93 | 0,107093844 | 0,895901449 | 0 | 0 |
| 2/11 | 0,107443713 | 0,897357231 | 0 | 0 |
| 2/11 | 0,107443713 | 0,895890961 | 0 | 0 |
| 2/11 | 0,107443713 | 0,894429475 | 0 | 0 |
| 2/11 | 0,107443713 | 0,89297275 | 0 | 0 |
| 2/11 | 0,107443713 | 0,891520762 | 0 | 0 |
| 2/11 | 0,107443713 | 0,890073488 | 0 | 0 |
| 2/11 | 0,107443713 | 0,888630905 | 0 | 0 |
| 2/11 | 0,107443713 | 0,887192991 | 0 | 0 |
| 2/11 | 0,107443713 | 0,885759723 | 0 | 0 |
| 2/11 | 0,107443713 | 0,884331078 | 0 | 0 |
| 2/11 | 0,107443713 | 0,882907034 | 0 | 0 |
| 2/11 | 0,107443713 | 0,88148757 | 0 | 0 |
| 2/11 | 0,107443713 | 0,880072662 | 0 | 0 |
| 2/11 | 0,107443713 | 0,878662289 | 0 | 0 |
| 2/11 | 0,107443713 | 0,877256429 | 0 | 0 |
| 2/11 | 0,107443713 | 0,875855061 | 0 | 0 |
| 2/11 | 0,107443713 | 0,874458163 | 0 | 0 |
| 2/11 | 0,107443713 | 0,873065714 | 0 | 0 |
| 2/11 | 0,107443713 | 0,871677692 | 0 | 0 |
| 2/11 | 0,107443713 | 0,870294077 | 0 | 0 |
| 2/11 | 0,107443713 | 0,868914847 | 0 | 0 |
| 2/11 | 0,107443713 | 0,867539982 | 0 | 0 |
| 2/11 | 0,107443713 | 0,86616946 | 0 | 0 |
| 2/11 | 0,107443713 | 0,864803262 | 0 | 0 |
| 2/11 | 0,107443713 | 0,863441368 | 0 | 0 |

| | | | | |
|---|---|---|---|---|
| 2/11 | 0,107443713 | 0,862083755 | 0 | 0 |
| 2/11 | 0,107443713 | 0,860730406 | 0 | 0 |
| 16/222 | 0,111810499 | 0,89430874 | 0 | 0 |
| 10/125 | 0,111968123 | 0,894167965 | 0 | 0 |
| 8/94 | 0,112087573 | 0,893723261 | 0 | 0 |
| 3/23 | 0,112800229 | 0,898002446 | 0 | 0 |
| 3/23 | 0,112800229 | 0,896603689 | 0 | 0 |
| 7/79 | 0,112802756 | 0,895229338 | 0 | 0 |
| 4/36 | 0,112819132 | 0,893968995 | 0 | 0 |
| 4/36 | 0,112819132 | 0,892582997 | 0 | 0 |
| 4/36 | 0,112819132 | 0,891201289 | 0 | 0 |
| 6/65 | 0,117912758 | 0,929998151 | 0 | 0 |
| 7/80 | 0,118458914 | 0,932863946 | 0 | 0 |
| 7/80 | 0,118458914 | 0,931426559 | 0 | 0 |
| 27/411 | 0,118644027 | 0,931446874 | 0 | 0 |
| 18/258 | 0,120199599 | 0,942209761 | 0 | 0 |
| 11/143 | 0,121032554 | 0,947283933 | 0 | 0 |
| 5/51 | 0,12152875 | 0,949710892 | 0 | 0 |
| 5/51 | 0,12152875 | 0,948258735 | 0 | 0 |
| 4/37 | 0,121626858 | 0,947575357 | 0 | 0 |
| 4/37 | 0,121626858 | 0,946130882 | 0 | 0 |
| 4/37 | 0,121626858 | 0,944690805 | 0 | 0 |
| 4/37 | 0,121626858 | 0,943255105 | 0 | 0 |
| 4/37 | 0,121626858 | 0,941823762 | 0 | 0 |
| 4/37 | 0,121626858 | 0,940396756 | 0 | 0 |
| 4/37 | 0,121626858 | 0,938974068 | 0 | 0 |
| 8/96 | 0,122446389 | 0,943872996 | 0 | 0 |
| 2/12 | 0,124685754 | 0,95968537 | 0 | 0 |
| 2/12 | 0,124685754 | 0,958240061 | 0 | 0 |
| 2/12 | 0,124685754 | 0,956799098 | 0 | 0 |
| 2/12 | 0,124685754 | 0,955362463 | 0 | 0 |
| 2/12 | 0,124685754 | 0,953930135 | 0 | 0 |
| 2/12 | 0,124685754 | 0,952502096 | 0 | 0 |
| 2/12 | 0,124685754 | 0,951078326 | 0 | 0 |
| 2/12 | 0,124685754 | 0,949658806 | 0 | 0 |
| 2/12 | 0,124685754 | 0,948243518 | 0 | 0 |
| 2/12 | 0,124685754 | 0,946832441 | 0 | 0 |
| 2/12 | 0,124685754 | 0,945425558 | 0 | 0 |
| 2/12 | 0,124685754 | 0,944022849 | 0 | 0 |
| 2/12 | 0,124685754 | 0,942624297 | 0 | 0 |
| 2/12 | 0,124685754 | 0,941229882 | 0 | 0 |
| 2/12 | 0,124685754 | 0,939839587 | 0 | 0 |
| 2/12 | 0,124685754 | 0,938453393 | 0 | 0 |
| 2/12 | 0,124685754 | 0,937071282 | 0 | 0 |
| 2/12 | 0,124685754 | 0,935693236 | 0 | 0 |
| 2/12 | 0,124685754 | 0,934319237 | 0 | 0 |
| 2/12 | 0,124685754 | 0,932949267 | 0 | 0 |
| 2/12 | 0,124685754 | 0,931583309 | 0 | 0 |
| 2/12 | 0,124685754 | 0,930221345 | 0 | 0 |
| 2/12 | 0,124685754 | 0,928863358 | 0 | 0 |
| 10/128 | 0,125384089 | 0,932704088 | 0 | 0 |
| 10/128 | 0,125384089 | 0,93134644 | 0 | 0 |
| 8/97 | 0,127808605 | 0,947975745 | 0 | 0 |

| | | | | |
|---|---|---|---|---|
| 7/82 | 0,130200832 | 0,964317629 | 0 | 0 |
| 4/38 | 0,130718162 | 0,966746058 | 0 | 0 |
| 4/38 | 0,130718162 | 0,965347004 | 0 | 0 |
| 4/38 | 0,130718162 | 0,963951994 | 0 | 0 |
| 4/38 | 0,130718162 | 0,96256101 | 0 | 0 |
| 4/38 | 0,130718162 | 0,961174035 | 0 | 0 |
| 3/25 | 0,136044545 | 0,998899728 | 0 | 0 |
| 3/25 | 0,136044545 | 0,997464527 | 0 | 0 |
| 3/25 | 0,136044545 | 0,996033445 | 0 | 0 |
| 3/25 | 0,136044545 | 0,994606463 | 0 | 0 |
| 3/25 | 0,136044545 | 0,993183564 | 0 | 0 |
| 3/25 | 0,136044545 | 0,99176473 | 0 | 0 |
| 3/25 | 0,136044545 | 0,990349944 | 0 | 0 |
| 3/25 | 0,136044545 | 0,98893919 | 0 | 0 |
| 3/25 | 0,136044545 | 0,987532448 | 0 | 0 |
| 3/25 | 0,136044545 | 0,986129703 | 0 | 0 |
| 7/83 | 0,136281804 | 0,986448294 | 0 | 0 |
| 7/83 | 0,136281804 | 0,985051059 | 0 | 0 |
| 7/83 | 0,136281804 | 0,983657776 | 0 | 0 |
| 5/53 | 0,136889868 | 0,986651125 | 0 | 0 |
| 6/68 | 0,13788388 | 0,992413881 | 0 | 0 |
| 6/68 | 0,13788388 | 0,991016115 | 0 | 0 |
| 6/68 | 0,13788388 | 0,989622281 | 0 | 0 |
| 6/68 | 0,13788388 | 0,988232362 | 0 | 0 |
| 11/147 | 0,138680087 | 0,992544857 | 0 | 0 |
| 8/99 | 0,138890412 | 0,992657948 | 0 | 0 |
| 8/99 | 0,138890412 | 0,991269615 | 0 | 0 |
| 4/39 | 0,140080408 | 0,998366372 | 0 | 0 |
| 4/39 | 0,140080408 | 0,996973951 | 0 | 0 |
| 4/39 | 0,140080408 | 0,995585408 | 0 | 0 |
| 4/39 | 0,140080408 | 0,994200727 | 0 | 0 |
| 15/214 | 0,142059187 | 1 | 0 | 0 |
| 7/84 | 0,142499285 | 1 | 0 | 0 |
| 2/13 | 0,142525984 | 1 | 0 | 0 |
| 2/13 | 0,142525984 | 1 | 0 | 0 |
| 2/13 | 0,142525984 | 1 | 0 | 0 |
| 2/13 | 0,142525984 | 1 | 0 | 0 |
| 2/13 | 0,142525984 | 1 | 0 | 0 |
| 2/13 | 0,142525984 | 1 | 0 | 0 |
| 2/13 | 0,142525984 | 0,999052329 | 0 | 0 |
| 2/13 | 0,142525984 | 0,997681887 | 0 | 0 |
| 2/13 | 0,142525984 | 0,996315199 | 0 | 0 |
| 2/13 | 0,142525984 | 0,994952251 | 0 | 0 |
| 2/13 | 0,142525984 | 0,993593027 | 0 | 0 |
| 2/13 | 0,142525984 | 0,992237511 | 0 | 0 |
| 2/13 | 0,142525984 | 0,990885689 | 0 | 0 |
| 2/13 | 0,142525984 | 0,989537545 | 0 | 0 |
| 2/13 | 0,142525984 | 0,988193064 | 0 | 0 |
| 2/13 | 0,142525984 | 0,986852233 | 0 | 0 |
| 2/13 | 0,142525984 | 0,985515035 | 0 | 0 |
| 2/13 | 0,142525984 | 0,984181455 | 0 | 0 |
| 2/13 | 0,142525984 | 0,98285148 | 0 | 0 |
| 8/100 | 0,144606297 | 0,995851463 | 0 | 0 |

| | | | | |
|---|---|---|---|---|
| 6/69 | 0,144871287 | 0,996331777 | 0 | 0 |
| 6/69 | 0,144871287 | 0,994990819 | 0 | 0 |
| 5/54 | 0,144876791 | 0,993691215 | 0 | 0 |
| 3/26 | 0,148230982 | 1 | 0 | 0 |
| 3/26 | 0,148230982 | 1 | 0 | 0 |
| 3/26 | 0,148230982 | 1 | 0 | 0 |
| 3/26 | 0,148230982 | 1 | 0 | 0 |
| 3/26 | 0,148230982 | 1 | 0 | 0 |
| 3/26 | 0,148230982 | 1 | 0 | 0 |
| 19/284 | 0,149536979 | 1 | 0 | 0 |
| 4/40 | 0,149700503 | 1 | 0 | 0 |
| 4/40 | 0,149700503 | 1 | 0 | 0 |
| 4/40 | 0,149700503 | 1 | 0 | 0 |
| 12/166 | 0,150423863 | 1 | 0 | 0 |
| 6/70 | 0,15201679 | 1 | 0 | 0 |
| 6/70 | 0,15201679 | 1 | 0 | 0 |
| 6/70 | 0,15201679 | 1 | 0 | 0 |
| 11/150 | 0,152766182 | 1 | 0 | 0 |
| 5/55 | 0,15305853 | 1 | 0 | 0 |
| 5/55 | 0,15305853 | 1 | 0 | 0 |
| 16/234 | 0,15396247 | 1 | 0 | 0 |
| 10/134 | 0,154691609 | 1 | 0 | 0 |
| 8/102 | 0,156377792 | 1 | 0 | 0 |
| 18/269 | 0,157102507 | 1 | 0 | 0 |
| 6/71 | 0,159315798 | 1 | 0 | 0 |
| 6/71 | 0,159315798 | 1 | 0 | 0 |
| 4/41 | 0,159564969 | 1 | 0 | 0 |
| 3/27 | 0,160752832 | 1 | 0 | 0 |
| 3/27 | 0,160752832 | 1 | 0 | 0 |
| 3/27 | 0,160752832 | 1 | 0 | 0 |
| 3/27 | 0,160752832 | 1 | 0 | 0 |
| 3/27 | 0,160752832 | 1 | 0 | 0 |
| 3/27 | 0,160752832 | 1 | 0 | 0 |
| 2/14 | 0,160856918 | 1 | 0 | 0 |
| 2/14 | 0,160856918 | 1 | 0 | 0 |
| 2/14 | 0,160856918 | 1 | 0 | 0 |
| 2/14 | 0,160856918 | 1 | 0 | 0 |
| 2/14 | 0,160856918 | 1 | 0 | 0 |
| 2/14 | 0,160856918 | 1 | 0 | 0 |
| 2/14 | 0,160856918 | 1 | 0 | 0 |
| 2/14 | 0,160856918 | 1 | 0 | 0 |
| 2/14 | 0,160856918 | 1 | 0 | 0 |
| 2/14 | 0,160856918 | 1 | 0 | 0 |
| 2/14 | 0,160856918 | 1 | 0 | 0 |
| 2/14 | 0,160856918 | 1 | 0 | 0 |
| 2/14 | 0,160856918 | 1 | 0 | 0 |
| 2/14 | 0,160856918 | 1 | 0 | 0 |
| 2/14 | 0,160856918 | 1 | 0 | 0 |
| 2/14 | 0,160856918 | 1 | 0 | 0 |
| 2/14 | 0,160856918 | 1 | 0 | 0 |
| 2/14 | 0,160856918 | 1 | 0 | 0 |
| 2/14 | 0,160856918 | 1 | 0 | 0 |
| 5/56 | 0,161427642 | 1 | 0 | 0 |

| | | | | |
|---|---|---|---|---|
| 5/56 | 0,161427642 | 1 | 0 | 0 |
| 7/87 | 0,161941511 | 1 | 0 | 0 |
| 6/72 | 0,166763522 | 1 | 0 | 0 |
| 6/72 | 0,166763522 | 1 | 0 | 0 |
| 7/88 | 0,168674966 | 1 | 0 | 0 |
| 4/42 | 0,169660024 | 1 | 0 | 0 |
| 4/42 | 0,169660024 | 1 | 0 | 0 |
| 14/204 | 0,170207854 | 1 | 0 | 0 |
| 3/28 | 0,173579701 | 1 | 0 | 0 |
| 3/28 | 0,173579701 | 1 | 0 | 0 |
| 3/28 | 0,173579701 | 1 | 0 | 0 |
| 3/28 | 0,173579701 | 1 | 0 | 0 |
| 3/28 | 0,173579701 | 1 | 0 | 0 |
| 3/28 | 0,173579701 | 1 | 0 | 0 |
| 6/73 | 0,174354982 | 1 | 0 | 0 |
| 10/138 | 0,175965695 | 1 | 0 | 0 |
| 30/486 | 0,177857507 | 1 | 0 | 0 |
| 5/58 | 0,178696931 | 1 | 0 | 0 |
| 5/58 | 0,178696931 | 1 | 0 | 0 |
| 2/15 | 0,179580553 | 1 | 0 | 0 |
| 2/15 | 0,179580553 | 1 | 0 | 0 |
| 2/15 | 0,179580553 | 1 | 0 | 0 |
| 2/15 | 0,179580553 | 1 | 0 | 0 |
| 2/15 | 0,179580553 | 1 | 0 | 0 |
| 2/15 | 0,179580553 | 1 | 0 | 0 |
| 2/15 | 0,179580553 | 1 | 0 | 0 |
| 2/15 | 0,179580553 | 1 | 0 | 0 |
| 2/15 | 0,179580553 | 1 | 0 | 0 |
| 2/15 | 0,179580553 | 1 | 0 | 0 |
| 2/15 | 0,179580553 | 1 | 0 | 0 |
| 2/15 | 0,179580553 | 1 | 0 | 0 |
| 2/15 | 0,179580553 | 1 | 0 | 0 |
| 2/15 | 0,179580553 | 1 | 0 | 0 |
| 2/15 | 0,179580553 | 1 | 0 | 0 |
| 2/15 | 0,179580553 | 1 | 0 | 0 |
| 2/15 | 0,179580553 | 1 | 0 | 0 |
| 2/15 | 0,179580553 | 1 | 0 | 0 |
| 2/15 | 0,179580553 | 1 | 0 | 0 |
| 2/15 | 0,179580553 | 1 | 0 | 0 |
| 2/15 | 0,179580553 | 1 | 0 | 0 |
| 2/15 | 0,179580553 | 1 | 0 | 0 |
| 2/15 | 0,179580553 | 1 | 0 | 0 |
| 2/15 | 0,179580553 | 1 | 0 | 0 |
| 4/43 | 0,179971645 | 1 | 0 | 0 |
| 4/43 | 0,179971645 | 1 | 0 | 0 |
| 4/43 | 0,179971645 | 1 | 0 | 0 |
| 4/43 | 0,179971645 | 1 | 0 | 0 |
| 10/139 | 0,181488415 | 1 | 0 | 0 |
| 6/74 | 0,182085029 | 1 | 0 | 0 |
| 6/74 | 0,182085029 | 1 | 0 | 0 |
| 7/90 | 0,182500436 | 1 | 0 | 0 |
| 14/207 | 0,183597092 | 1 | 0 | 0 |
| 3/29 | 0,18668155 | 1 | 0 | 0 |

| | | | | |
|---|---|---|---|---|
| 3/29 | 0,18668155 | 1 | 0 | 0 |
| 3/29 | 0,18668155 | 1 | 0 | 0 |
| 3/29 | 0,18668155 | 1 | 0 | 0 |
| 3/29 | 0,18668155 | 1 | 0 | 0 |
| 3/29 | 0,18668155 | 1 | 0 | 0 |
| 3/29 | 0,18668155 | 1 | 0 | 0 |
| 3/29 | 0,18668155 | 1 | 0 | 0 |
| 3/29 | 0,18668155 | 1 | 0 | 0 |
| 3/29 | 0,18668155 | 1 | 0 | 0 |
| 11/157 | 0,188305993 | 1 | 0 | 0 |
| 7/91 | 0,189585156 | 1 | 0 | 0 |
| 6/75 | 0,189948359 | 1 | 0 | 0 |
| 6/75 | 0,189948359 | 1 | 0 | 0 |
| 48/816 | 0,190155496 | 1 | 0 | 0 |
| 4/44 | 0,190485643 | 1 | 0 | 0 |
| 4/44 | 0,190485643 | 1 | 0 | 0 |
| 4/44 | 0,190485643 | 1 | 0 | 0 |
| 4/44 | 0,190485643 | 1 | 0 | 0 |
| 4/44 | 0,190485643 | 1 | 0 | 0 |
| 4/44 | 0,190485643 | 1 | 0 | 0 |
| 9/124 | 0,190899161 | 1 | 0 | 0 |
| 7/92 | 0,196779476 | 1 | 0 | 0 |
| 6/76 | 0,197939526 | 1 | 0 | 0 |
| 2/16 | 0,198607672 | 1 | 0 | 0 |
| 2/16 | 0,198607672 | 1 | 0 | 0 |
| 2/16 | 0,198607672 | 1 | 0 | 0 |
| 2/16 | 0,198607672 | 1 | 0 | 0 |
| 2/16 | 0,198607672 | 1 | 0 | 0 |
| 2/16 | 0,198607672 | 1 | 0 | 0 |
| 2/16 | 0,198607672 | 1 | 0 | 0 |
| 2/16 | 0,198607672 | 1 | 0 | 0 |
| 2/16 | 0,198607672 | 1 | 0 | 0 |
| 2/16 | 0,198607672 | 1 | 0 | 0 |
| 2/16 | 0,198607672 | 1 | 0 | 0 |
| 2/16 | 0,198607672 | 1 | 0 | 0 |
| 2/16 | 0,198607672 | 1 | 0 | 0 |
| 2/16 | 0,198607672 | 1 | 0 | 0 |
| 2/16 | 0,198607672 | 1 | 0 | 0 |
| 2/16 | 0,198607672 | 1 | 0 | 0 |
| 2/16 | 0,198607672 | 1 | 0 | 0 |
| 2/16 | 0,198607672 | 1 | 0 | 0 |
| 23/368 | 0,198713336 | 1 | 0 | 0 |
| 3/30 | 0,200028832 | 1 | 0 | 0 |
| 3/30 | 0,200028832 | 1 | 0 | 0 |
| 3/30 | 0,200028832 | 1 | 0 | 0 |
| 3/30 | 0,200028832 | 1 | 0 | 0 |
| 3/30 | 0,200028832 | 1 | 0 | 0 |
| 3/30 | 0,200028832 | 1 | 0 | 0 |
| 3/30 | 0,200028832 | 1 | 0 | 0 |
| 3/30 | 0,200028832 | 1 | 0 | 0 |
| 3/30 | 0,200028832 | 1 | 0 | 0 |
| 3/30 | 0,200028832 | 1 | 0 | 0 |
| 4/45 | 0,201187717 | 1 | 0 | 0 |

| | | | | |
|---|---|---|---|---|
| 4/45 | 0,201187717 | 1 | 0 | 0 |
| 4/45 | 0,201187717 | 1 | 0 | 0 |
| 9/126 | 0,203229051 | 1 | 0 | 0 |
| 7/93 | 0,20407947 | 1 | 0 | 0 |
| 6/77 | 0,206052961 | 1 | 0 | 0 |
| 6/77 | 0,206052961 | 1 | 0 | 0 |
| 6/77 | 0,206052961 | 1 | 0 | 0 |
| 8/110 | 0,20763815 | 1 | 0 | 0 |
| 4/46 | 0,212063518 | 1 | 0 | 0 |
| 4/46 | 0,212063518 | 1 | 0 | 0 |
| 4/46 | 0,212063518 | 1 | 0 | 0 |
| 4/46 | 0,212063518 | 1 | 0 | 0 |
| 3/31 | 0,213592618 | 1 | 0 | 0 |
| 3/31 | 0,213592618 | 1 | 0 | 0 |
| 3/31 | 0,213592618 | 1 | 0 | 0 |
| 3/31 | 0,213592618 | 1 | 0 | 0 |
| 3/31 | 0,213592618 | 1 | 0 | 0 |
| 3/31 | 0,213592618 | 1 | 0 | 0 |
| 3/31 | 0,213592618 | 1 | 0 | 0 |
| 8/111 | 0,214470356 | 1 | 0 | 0 |
| 12/179 | 0,214832843 | 1 | 0 | 0 |
| 5/62 | 0,21513122 | 1 | 0 | 0 |
| 5/62 | 0,21513122 | 1 | 0 | 0 |
| 5/62 | 0,21513122 | 1 | 0 | 0 |
| 2/17 | 0,217857207 | 1 | 0 | 0 |
| 2/17 | 0,217857207 | 1 | 0 | 0 |
| 2/17 | 0,217857207 | 1 | 0 | 0 |
| 2/17 | 0,217857207 | 1 | 0 | 0 |
| 2/17 | 0,217857207 | 1 | 0 | 0 |
| 2/17 | 0,217857207 | 1 | 0 | 0 |
| 2/17 | 0,217857207 | 1 | 0 | 0 |
| 2/17 | 0,217857207 | 1 | 0 | 0 |
| 2/17 | 0,217857207 | 1 | 0 | 0 |
| 2/17 | 0,217857207 | 1 | 0 | 0 |
| 2/17 | 0,217857207 | 1 | 0 | 0 |
| 2/17 | 0,217857207 | 1 | 0 | 0 |
| 2/17 | 0,217857207 | 1 | 0 | 0 |
| 2/17 | 0,217857207 | 1 | 0 | 0 |
| 2/17 | 0,217857207 | 1 | 0 | 0 |
| 2/17 | 0,217857207 | 1 | 0 | 0 |
| 2/17 | 0,217857207 | 1 | 0 | 0 |
| 2/17 | 0,217857207 | 1 | 0 | 0 |
| 2/17 | 0,217857207 | 1 | 0 | 0 |
| 2/17 | 0,217857207 | 1 | 0 | 0 |
| 2/17 | 0,217857207 | 1 | 0 | 0 |
| 2/17 | 0,217857207 | 1 | 0 | 0 |
| 2/17 | 0,217857207 | 1 | 0 | 0 |
| 2/17 | 0,217857207 | 1 | 0 | 0 |
| 2/17 | 0,217857207 | 1 | 0 | 0 |
| 2/17 | 0,217857207 | 1 | 0 | 0 |
| 4/47 | 0,2230987 | 1 | 0 | 0 |
| 4/47 | 0,2230987 | 1 | 0 | 0 |
| 4/47 | 0,2230987 | 1 | 0 | 0 |
| 5/63 | 0,224585398 | 1 | 0 | 0 |

| | | | | |
|---|---|---|---|---|
| 7/96 | 0,226572868 | 1 | 0 | 0 |
| 31/520 | 0,226624629 | 1 | 0 | 0 |
| 11/164 | 0,227228505 | 1 | 0 | 0 |
| 3/32 | 0,227344696 | 1 | 0 | 0 |
| 3/32 | 0,227344696 | 1 | 0 | 0 |
| 3/32 | 0,227344696 | 1 | 0 | 0 |
| 3/32 | 0,227344696 | 1 | 0 | 0 |
| 3/32 | 0,227344696 | 1 | 0 | 0 |
| 3/32 | 0,227344696 | 1 | 0 | 0 |
| 3/32 | 0,227344696 | 1 | 0 | 0 |
| 3/32 | 0,227344696 | 1 | 0 | 0 |
| 3/32 | 0,227344696 | 1 | 0 | 0 |
| 6/80 | 0,231069631 | 1 | 0 | 0 |
| 6/80 | 0,231069631 | 1 | 0 | 0 |
| 5/64 | 0,234160515 | 1 | 0 | 0 |
| 5/64 | 0,234160515 | 1 | 0 | 0 |
| 5/64 | 0,234160515 | 1 | 0 | 0 |
| 4/48 | 0,234278972 | 1 | 0 | 0 |
| 4/48 | 0,234278972 | 1 | 0 | 0 |
| 4/48 | 0,234278972 | 1 | 0 | 0 |
| 4/48 | 0,234278972 | 1 | 0 | 0 |
| 4/48 | 0,234278972 | 1 | 0 | 0 |
| 37/632 | 0,234725226 | 1 | 0 | 0 |
| 8/114 | 0,235460725 | 1 | 0 | 0 |
| 14/218 | 0,236725168 | 1 | 0 | 0 |
| 2/18 | 0,237255632 | 1 | 0 | 0 |
| 2/18 | 0,237255632 | 1 | 0 | 0 |
| 2/18 | 0,237255632 | 1 | 0 | 0 |
| 2/18 | 0,237255632 | 1 | 0 | 0 |
| 2/18 | 0,237255632 | 1 | 0 | 0 |
| 2/18 | 0,237255632 | 1 | 0 | 0 |
| 2/18 | 0,237255632 | 1 | 0 | 0 |
| 2/18 | 0,237255632 | 1 | 0 | 0 |
| 2/18 | 0,237255632 | 1 | 0 | 0 |
| 2/18 | 0,237255632 | 1 | 0 | 0 |
| 2/18 | 0,237255632 | 1 | 0 | 0 |
| 2/18 | 0,237255632 | 1 | 0 | 0 |
| 2/18 | 0,237255632 | 1 | 0 | 0 |
| 2/18 | 0,237255632 | 1 | 0 | 0 |
| 2/18 | 0,237255632 | 1 | 0 | 0 |
| 2/18 | 0,237255632 | 1 | 0 | 0 |
| 2/18 | 0,237255632 | 1 | 0 | 0 |
| 2/18 | 0,237255632 | 1 | 0 | 0 |
| 2/18 | 0,237255632 | 1 | 0 | 0 |
| 2/18 | 0,237255632 | 1 | 0 | 0 |
| 2/18 | 0,237255632 | 1 | 0 | 0 |
| 2/18 | 0,237255632 | 1 | 0 | 0 |
| 2/18 | 0,237255632 | 1 | 0 | 0 |
| 6/81 | 0,239614479 | 1 | 0 | 0 |
| 6/81 | 0,239614479 | 1 | 0 | 0 |
| 6/81 | 0,239614479 | 1 | 0 | 0 |
| 6/81 | 0,239614479 | 1 | 0 | 0 |
| 3/33 | 0,241257655 | 1 | 0 | 0 |

| | | | | |
|---|---|---|---|---|
| 3/33 | 0,241257655 | 1 | 0 | 0 |
| 3/33 | 0,241257655 | 1 | 0 | 0 |
| 3/33 | 0,241257655 | 1 | 0 | 0 |
| 3/33 | 0,241257655 | 1 | 0 | 0 |
| 3/33 | 0,241257655 | 1 | 0 | 0 |
| 3/33 | 0,241257655 | 1 | 0 | 0 |
| 3/33 | 0,241257655 | 1 | 0 | 0 |
| 3/33 | 0,241257655 | 1 | 0 | 0 |
| 3/33 | 0,241257655 | 1 | 0 | 0 |
| 3/33 | 0,241257655 | 1 | 0 | 0 |
| 17/272 | 0,241314695 | 1 | 0 | 0 |
| 7/98 | 0,242020994 | 1 | 0 | 0 |
| 5/65 | 0,243847828 | 1 | 0 | 0 |
| 5/65 | 0,243847828 | 1 | 0 | 0 |
| 5/65 | 0,243847828 | 1 | 0 | 0 |
| 4/49 | 0,245590145 | 1 | 0 | 0 |
| 4/49 | 0,245590145 | 1 | 0 | 0 |
| 6/82 | 0,248252399 | 1 | 0 | 0 |
| 9/133 | 0,248762671 | 1 | 0 | 0 |
| 5/66 | 0,253638584 | 1 | 0 | 0 |
| 17/275 | 0,255248227 | 1 | 0 | 0 |
| 3/34 | 0,255304963 | 1 | 0 | 0 |
| 3/34 | 0,255304963 | 1 | 0 | 0 |
| 3/34 | 0,255304963 | 1 | 0 | 0 |
| 3/34 | 0,255304963 | 1 | 0 | 0 |
| 3/34 | 0,255304963 | 1 | 0 | 0 |
| 3/34 | 0,255304963 | 1 | 0 | 0 |
| 3/34 | 0,255304963 | 1 | 0 | 0 |
| 3/34 | 0,255304963 | 1 | 0 | 0 |
| 3/34 | 0,255304963 | 1 | 0 | 0 |
| 3/34 | 0,255304963 | 1 | 0 | 0 |
| 3/34 | 0,255304963 | 1 | 0 | 0 |
| 2/19 | 0,256736405 | 1 | 0 | 0 |
| 2/19 | 0,256736405 | 1 | 0 | 0 |
| 2/19 | 0,256736405 | 1 | 0 | 0 |
| 2/19 | 0,256736405 | 1 | 0 | 0 |
| 2/19 | 0,256736405 | 1 | 0 | 0 |
| 2/19 | 0,256736405 | 1 | 0 | 0 |
| 2/19 | 0,256736405 | 1 | 0 | 0 |
| 2/19 | 0,256736405 | 1 | 0 | 0 |
| 2/19 | 0,256736405 | 1 | 0 | 0 |
| 2/19 | 0,256736405 | 1 | 0 | 0 |
| 2/19 | 0,256736405 | 1 | 0 | 0 |
| 2/19 | 0,256736405 | 1 | 0 | 0 |
| 2/19 | 0,256736405 | 1 | 0 | 0 |
| 2/19 | 0,256736405 | 1 | 0 | 0 |
| 2/19 | 0,256736405 | 1 | 0 | 0 |
| 2/19 | 0,256736405 | 1 | 0 | 0 |
| 2/19 | 0,256736405 | 1 | 0 | 0 |
| 2/19 | 0,256736405 | 1 | 0 | 0 |
| 2/19 | 0,256736405 | 1 | 0 | 0 |
| 2/19 | 0,256736405 | 1 | 0 | 0 |
| 2/19 | 0,256736405 | 1 | 0 | 0 |

| | | | | |
|---|---|---|---|---|
| 2/19 | 0,256736405 | 1 | 0 | 0 |
| 6/83 | 0,25697738 | 1 | 0 | 0 |
| 6/83 | 0,25697738 | 1 | 0 | 0 |
| 4/50 | 0,257018171 | 1 | 0 | 0 |
| 7/100 | 0,257790801 | 1 | 0 | 0 |
| 6/84 | 0,265783387 | 1 | 0 | 0 |
| 4/51 | 0,268549185 | 1 | 0 | 0 |
| 4/51 | 0,268549185 | 1 | 0 | 0 |
| 4/51 | 0,268549185 | 1 | 0 | 0 |
| 4/51 | 0,268549185 | 1 | 0 | 0 |
| 11/171 | 0,268994742 | 1 | 0 | 0 |
| 3/35 | 0,269461014 | 1 | 0 | 0 |
| 3/35 | 0,269461014 | 1 | 0 | 0 |
| 3/35 | 0,269461014 | 1 | 0 | 0 |
| 3/35 | 0,269461014 | 1 | 0 | 0 |
| 3/35 | 0,269461014 | 1 | 0 | 0 |
| 1/6 | 0,272334438 | 1 | 0 | 0 |
| 1/6 | 0,272334438 | 1 | 0 | 0 |
| 1/6 | 0,272334438 | 1 | 0 | 0 |
| 1/6 | 0,272334438 | 1 | 0 | 0 |
| 1/6 | 0,272334438 | 1 | 0 | 0 |
| 1/6 | 0,272334438 | 1 | 0 | 0 |
| 1/6 | 0,272334438 | 1 | 0 | 0 |
| 1/6 | 0,272334438 | 1 | 0 | 0 |
| 1/6 | 0,272334438 | 1 | 0 | 0 |
| 1/6 | 0,272334438 | 1 | 0 | 0 |
| 1/6 | 0,272334438 | 1 | 0 | 0 |
| 1/6 | 0,272334438 | 1 | 0 | 0 |
| 1/6 | 0,272334438 | 1 | 0 | 0 |
| 1/6 | 0,272334438 | 1 | 0 | 0 |
| 1/6 | 0,272334438 | 1 | 0 | 0 |
| 1/6 | 0,272334438 | 1 | 0 | 0 |
| 1/6 | 0,272334438 | 1 | 0 | 0 |
| 1/6 | 0,272334438 | 1 | 0 | 0 |
| 1/6 | 0,272334438 | 1 | 0 | 0 |
| 1/6 | 0,272334438 | 1 | 0 | 0 |
| 1/6 | 0,272334438 | 1 | 0 | 0 |
| 1/6 | 0,272334438 | 1 | 0 | 0 |
| 1/6 | 0,272334438 | 1 | 0 | 0 |
| 1/6 | 0,272334438 | 1 | 0 | 0 |
| 1/6 | 0,272334438 | 1 | 0 | 0 |
| 1/6 | 0,272334438 | 1 | 0 | 0 |
| 1/6 | 0,272334438 | 1 | 0 | 0 |
| 1/6 | 0,272334438 | 1 | 0 | 0 |
| 1/6 | 0,272334438 | 1 | 0 | 0 |
| 1/6 | 0,272334438 | 1 | 0 | 0 |
| 1/6 | 0,272334438 | 1 | 0 | 0 |
| 1/6 | 0,272334438 | 1 | 0 | 0 |
| 1/6 | 0,272334438 | 1 | 0 | 0 |
| 1/6 | 0,272334438 | 1 | 0 | 0 |
| 1/6 | 0,272334438 | 1 | 0 | 0 |
| 1/6 | 0,272334438 | 1 | 0 | 0 |
| 1/6 | 0,272334438 | 1 | 0 | 0 |

| | | | | |
|---|---|---|---|---|
| 1/6 | 0,272334438 | 1 | 0 | 0 |
| 1/6 | 0,272334438 | 1 | 0 | 0 |
| 1/6 | 0,272334438 | 1 | 0 | 0 |
| 1/6 | 0,272334438 | 1 | 0 | 0 |
| 1/6 | 0,272334438 | 1 | 0 | 0 |
| 1/6 | 0,272334438 | 1 | 0 | 0 |
| 1/6 | 0,272334438 | 1 | 0 | 0 |
| 1/6 | 0,272334438 | 1 | 0 | 0 |
| 1/6 | 0,272334438 | 1 | 0 | 0 |
| 1/6 | 0,272334438 | 1 | 0 | 0 |
| 1/6 | 0,272334438 | 1 | 0 | 0 |
| 1/6 | 0,272334438 | 1 | 0 | 0 |
| 1/6 | 0,272334438 | 1 | 0 | 0 |
| 1/6 | 0,272334438 | 1 | 0 | 0 |
| 1/6 | 0,272334438 | 1 | 0 | 0 |
| 1/6 | 0,272334438 | 1 | 0 | 0 |
| 1/6 | 0,272334438 | 1 | 0 | 0 |
| 1/6 | 0,272334438 | 1 | 0 | 0 |
| 1/6 | 0,272334438 | 1 | 0 | 0 |
| 1/6 | 0,272334438 | 1 | 0 | 0 |
| 1/6 | 0,272334438 | 1 | 0 | 0 |
| 1/6 | 0,272334438 | 1 | 0 | 0 |
| 1/6 | 0,272334438 | 1 | 0 | 0 |
| 1/6 | 0,272334438 | 1 | 0 | 0 |
| 1/6 | 0,272334438 | 1 | 0 | 0 |
| 1/6 | 0,272334438 | 1 | 0 | 0 |
| 1/6 | 0,272334438 | 1 | 0 | 0 |
| 1/6 | 0,272334438 | 1 | 0 | 0 |
| 1/6 | 0,272334438 | 1 | 0 | 0 |
| 1/6 | 0,272334438 | 1 | 0 | 0 |
| 1/6 | 0,272334438 | 1 | 0 | 0 |
| 1/6 | 0,272334438 | 1 | 0 | 0 |
| 1/6 | 0,272334438 | 1 | 0 | 0 |
| 1/6 | 0,272334438 | 1 | 0 | 0 |
| 1/6 | 0,272334438 | 1 | 0 | 0 |
| 1/6 | 0,272334438 | 1 | 0 | 0 |
| 1/6 | 0,272334438 | 1 | 0 | 0 |
| 1/6 | 0,272334438 | 1 | 0 | 0 |
| 1/6 | 0,272334438 | 1 | 0 | 0 |
| 1/6 | 0,272334438 | 1 | 0 | 0 |
| 1/6 | 0,272334438 | 1 | 0 | 0 |
| 1/6 | 0,272334438 | 1 | 0 | 0 |
| 1/6 | 0,272334438 | 1 | 0 | 0 |
| 1/6 | 0,272334438 | 1 | 0 | 0 |
| 1/6 | 0,272334438 | 1 | 0 | 0 |
| 1/6 | 0,272334438 | 1 | 0 | 0 |
| 1/6 | 0,272334438 | 1 | 0 | 0 |
| 1/6 | 0,272334438 | 1 | 0 | 0 |
| 1/6 | 0,272334438 | 1 | 0 | 0 |
| 1/6 | 0,272334438 | 1 | 0 | 0 |
| 1/6 | 0,272334438 | 1 | 0 | 0 |
| 1/6 | 0,272334438 | 1 | 0 | 0 |
| 1/6 | 0,272334438 | 1 | 0 | 0 |

| | | | | |
|---|---|---|---|---|
| 1/6 | 0,272334438 | 1 | 0 | 0 |
| 1/6 | 0,272334438 | 1 | 0 | 0 |
| 1/6 | 0,272334438 | 1 | 0 | 0 |
| 1/6 | 0,272334438 | 1 | 0 | 0 |
| 1/6 | 0,272334438 | 1 | 0 | 0 |
| 1/6 | 0,272334438 | 1 | 0 | 0 |
| 1/6 | 0,272334438 | 1 | 0 | 0 |
| 1/6 | 0,272334438 | 1 | 0 | 0 |
| 1/6 | 0,272334438 | 1 | 0 | 0 |
| 1/6 | 0,272334438 | 1 | 0 | 0 |
| 1/6 | 0,272334438 | 1 | 0 | 0 |
| 1/6 | 0,272334438 | 1 | 0 | 0 |
| 1/6 | 0,272334438 | 1 | 0 | 0 |
| 1/6 | 0,272334438 | 1 | 0 | 0 |
| 1/6 | 0,272334438 | 1 | 0 | 0 |
| 1/6 | 0,272334438 | 1 | 0 | 0 |
| 1/6 | 0,272334438 | 1 | 0 | 0 |
| 1/6 | 0,272334438 | 1 | 0 | 0 |
| 1/6 | 0,272334438 | 1 | 0 | 0 |
| 1/6 | 0,272334438 | 1 | 0 | 0 |
| 1/6 | 0,272334438 | 1 | 0 | 0 |
| 1/6 | 0,272334438 | 1 | 0 | 0 |
| 1/6 | 0,272334438 | 1 | 0 | 0 |
| 1/6 | 0,272334438 | 1 | 0 | 0 |
| 1/6 | 0,272334438 | 1 | 0 | 0 |
| 1/6 | 0,272334438 | 1 | 0 | 0 |
| 1/6 | 0,272334438 | 1 | 0 | 0 |
| 1/6 | 0,272334438 | 1 | 0 | 0 |
| 5/68 | 0,27349548 | 1 | 0 | 0 |
| 5/68 | 0,27349548 | 1 | 0 | 0 |
| 17/279 | 0,274289908 | 1 | 0 | 0 |
| 2/20 | 0,276239439 | 1 | 0 | 0 |
| 2/20 | 0,276239439 | 1 | 0 | 0 |
| 2/20 | 0,276239439 | 1 | 0 | 0 |
| 2/20 | 0,276239439 | 1 | 0 | 0 |
| 2/20 | 0,276239439 | 1 | 0 | 0 |
| 2/20 | 0,276239439 | 1 | 0 | 0 |
| 2/20 | 0,276239439 | 1 | 0 | 0 |
| 2/20 | 0,276239439 | 1 | 0 | 0 |
| 2/20 | 0,276239439 | 1 | 0 | 0 |
| 2/20 | 0,276239439 | 1 | 0 | 0 |
| 2/20 | 0,276239439 | 1 | 0 | 0 |
| 2/20 | 0,276239439 | 1 | 0 | 0 |
| 2/20 | 0,276239439 | 1 | 0 | 0 |
| 2/20 | 0,276239439 | 1 | 0 | 0 |
| 2/20 | 0,276239439 | 1 | 0 | 0 |
| 2/20 | 0,276239439 | 1 | 0 | 0 |
| 2/20 | 0,276239439 | 1 | 0 | 0 |
| 2/20 | 0,276239439 | 1 | 0 | 0 |
| 2/20 | 0,276239439 | 1 | 0 | 0 |
| 2/20 | 0,276239439 | 1 | 0 | 0 |
| 2/20 | 0,276239439 | 1 | 0 | 0 |
| 2/20 | 0,276239439 | 1 | 0 | 0 |

| | | | | |
|---|---|---|---|---|
| 16/262 | 0,279259634 | 1 | 0 | 0 |
| 5/69 | 0,283544244 | 1 | 0 | 0 |
| 6/86 | 0,283614281 | 1 | 0 | 0 |
| 6/86 | 0,283614281 | 1 | 0 | 0 |
| 3/36 | 0,283701185 | 1 | 0 | 0 |
| 3/36 | 0,283701185 | 1 | 0 | 0 |
| 3/36 | 0,283701185 | 1 | 0 | 0 |
| 3/36 | 0,283701185 | 1 | 0 | 0 |
| 3/36 | 0,283701185 | 1 | 0 | 0 |
| 3/36 | 0,283701185 | 1 | 0 | 0 |
| 3/36 | 0,283701185 | 1 | 0 | 0 |
| 3/36 | 0,283701185 | 1 | 0 | 0 |
| 3/36 | 0,283701185 | 1 | 0 | 0 |
| 3/36 | 0,283701185 | 1 | 0 | 0 |
| 12/192 | 0,288752161 | 1 | 0 | 0 |
| 16/264 | 0,289359553 | 1 | 0 | 0 |
| 7/104 | 0,290153472 | 1 | 0 | 0 |
| 4/53 | 0,29186584 | 1 | 0 | 0 |
| 4/53 | 0,29186584 | 1 | 0 | 0 |
| 4/53 | 0,29186584 | 1 | 0 | 0 |
| 6/87 | 0,292627078 | 1 | 0 | 0 |
| 5/70 | 0,29366174 | 1 | 0 | 0 |
| 5/70 | 0,29366174 | 1 | 0 | 0 |
| 2/21 | 0,295710622 | 1 | 0 | 0 |
| 2/21 | 0,295710622 | 1 | 0 | 0 |
| 2/21 | 0,295710622 | 1 | 0 | 0 |
| 2/21 | 0,295710622 | 1 | 0 | 0 |
| 2/21 | 0,295710622 | 1 | 0 | 0 |
| 2/21 | 0,295710622 | 1 | 0 | 0 |
| 2/21 | 0,295710622 | 1 | 0 | 0 |
| 2/21 | 0,295710622 | 1 | 0 | 0 |
| 2/21 | 0,295710622 | 1 | 0 | 0 |
| 2/21 | 0,295710622 | 1 | 0 | 0 |
| 2/21 | 0,295710622 | 1 | 0 | 0 |
| 2/21 | 0,295710622 | 1 | 0 | 0 |
| 2/21 | 0,295710622 | 1 | 0 | 0 |
| 2/21 | 0,295710622 | 1 | 0 | 0 |
| 2/21 | 0,295710622 | 1 | 0 | 0 |
| 2/21 | 0,295710622 | 1 | 0 | 0 |
| 2/21 | 0,295710622 | 1 | 0 | 0 |
| 2/21 | 0,295710622 | 1 | 0 | 0 |
| 2/21 | 0,295710622 | 1 | 0 | 0 |
| 2/21 | 0,295710622 | 1 | 0 | 0 |
| 2/21 | 0,295710622 | 1 | 0 | 0 |
| 2/21 | 0,295710622 | 1 | 0 | 0 |
| 2/21 | 0,295710622 | 1 | 0 | 0 |
| 3/37 | 0,29800186 | 1 | 0 | 0 |
| 3/37 | 0,29800186 | 1 | 0 | 0 |
| 3/37 | 0,29800186 | 1 | 0 | 0 |
| 3/37 | 0,29800186 | 1 | 0 | 0 |
| 3/37 | 0,29800186 | 1 | 0 | 0 |
| 3/37 | 0,29800186 | 1 | 0 | 0 |
| 3/37 | 0,29800186 | 1 | 0 | 0 |

| | | | | |
|---|---|---|---|---|
| 11/176 | 0,300226355 | 1 | 0 | 0 |
| 8/123 | 0,302097423 | 1 | 0 | 0 |
| 5/71 | 0,303839462 | 1 | 0 | 0 |
| 5/71 | 0,303839462 | 1 | 0 | 0 |
| 5/71 | 0,303839462 | 1 | 0 | 0 |
| 1/7 | 0,309893194 | 1 | 0 | 0 |
| 1/7 | 0,309893194 | 1 | 0 | 0 |
| 1/7 | 0,309893194 | 1 | 0 | 0 |
| 1/7 | 0,309893194 | 1 | 0 | 0 |
| 1/7 | 0,309893194 | 1 | 0 | 0 |
| 1/7 | 0,309893194 | 1 | 0 | 0 |
| 1/7 | 0,309893194 | 1 | 0 | 0 |
| 1/7 | 0,309893194 | 1 | 0 | 0 |
| 1/7 | 0,309893194 | 1 | 0 | 0 |
| 1/7 | 0,309893194 | 1 | 0 | 0 |
| 1/7 | 0,309893194 | 1 | 0 | 0 |
| 1/7 | 0,309893194 | 1 | 0 | 0 |
| 1/7 | 0,309893194 | 1 | 0 | 0 |
| 1/7 | 0,309893194 | 1 | 0 | 0 |
| 1/7 | 0,309893194 | 1 | 0 | 0 |
| 1/7 | 0,309893194 | 1 | 0 | 0 |
| 1/7 | 0,309893194 | 1 | 0 | 0 |
| 1/7 | 0,309893194 | 1 | 0 | 0 |
| 1/7 | 0,309893194 | 1 | 0 | 0 |
| 1/7 | 0,309893194 | 1 | 0 | 0 |
| 1/7 | 0,309893194 | 1 | 0 | 0 |
| 1/7 | 0,309893194 | 1 | 0 | 0 |
| 1/7 | 0,309893194 | 1 | 0 | 0 |
| 1/7 | 0,309893194 | 1 | 0 | 0 |
| 1/7 | 0,309893194 | 1 | 0 | 0 |
| 1/7 | 0,309893194 | 1 | 0 | 0 |
| 1/7 | 0,309893194 | 1 | 0 | 0 |
| 1/7 | 0,309893194 | 1 | 0 | 0 |
| 1/7 | 0,309893194 | 1 | 0 | 0 |
| 1/7 | 0,309893194 | 1 | 0 | 0 |
| 1/7 | 0,309893194 | 1 | 0 | 0 |
| 1/7 | 0,309893194 | 1 | 0 | 0 |
| 1/7 | 0,309893194 | 1 | 0 | 0 |
| 1/7 | 0,309893194 | 1 | 0 | 0 |
| 1/7 | 0,309893194 | 1 | 0 | 0 |
| 1/7 | 0,309893194 | 1 | 0 | 0 |
| 1/7 | 0,309893194 | 1 | 0 | 0 |
| 1/7 | 0,309893194 | 1 | 0 | 0 |
| 1/7 | 0,309893194 | 1 | 0 | 0 |
| 1/7 | 0,309893194 | 1 | 0 | 0 |
| 1/7 | 0,309893194 | 1 | 0 | 0 |
| 1/7 | 0,309893194 | 1 | 0 | 0 |
| 1/7 | 0,309893194 | 1 | 0 | 0 |
| 1/7 | 0,309893194 | 1 | 0 | 0 |
| 1/7 | 0,309893194 | 1 | 0 | 0 |
| 1/7 | 0,309893194 | 1 | 0 | 0 |
| 1/7 | 0,309893194 | 1 | 0 | 0 |
| 1/7 | 0,309893194 | 1 | 0 | 0 |

| | | | | |
|---|---|---|---|---|
| 1/7 | 0,309893194 | 1 | 0 | 0 |
| 1/7 | 0,309893194 | 1 | 0 | 0 |
| 1/7 | 0,309893194 | 1 | 0 | 0 |
| 1/7 | 0,309893194 | 1 | 0 | 0 |
| 1/7 | 0,309893194 | 1 | 0 | 0 |
| 1/7 | 0,309893194 | 1 | 0 | 0 |
| 1/7 | 0,309893194 | 1 | 0 | 0 |
| 1/7 | 0,309893194 | 1 | 0 | 0 |
| 1/7 | 0,309893194 | 1 | 0 | 0 |
| 1/7 | 0,309893194 | 1 | 0 | 0 |
| 1/7 | 0,309893194 | 1 | 0 | 0 |
| 1/7 | 0,309893194 | 1 | 0 | 0 |
| 1/7 | 0,309893194 | 1 | 0 | 0 |
| 1/7 | 0,309893194 | 1 | 0 | 0 |
| 1/7 | 0,309893194 | 1 | 0 | 0 |
| 1/7 | 0,309893194 | 1 | 0 | 0 |
| 1/7 | 0,309893194 | 1 | 0 | 0 |
| 1/7 | 0,309893194 | 1 | 0 | 0 |
| 1/7 | 0,309893194 | 1 | 0 | 0 |
| 1/7 | 0,309893194 | 1 | 0 | 0 |
| 1/7 | 0,309893194 | 1 | 0 | 0 |
| 1/7 | 0,309893194 | 1 | 0 | 0 |
| 1/7 | 0,309893194 | 1 | 0 | 0 |
| 1/7 | 0,309893194 | 1 | 0 | 0 |
| 1/7 | 0,309893194 | 1 | 0 | 0 |
| 1/7 | 0,309893194 | 1 | 0 | 0 |
| 1/7 | 0,309893194 | 1 | 0 | 0 |
| 1/7 | 0,309893194 | 1 | 0 | 0 |
| 1/7 | 0,309893194 | 1 | 0 | 0 |
| 1/7 | 0,309893194 | 1 | 0 | 0 |
| 1/7 | 0,309893194 | 1 | 0 | 0 |
| 1/7 | 0,309893194 | 1 | 0 | 0 |
| 1/7 | 0,309893194 | 1 | 0 | 0 |
| 1/7 | 0,309893194 | 1 | 0 | 0 |
| 1/7 | 0,309893194 | 1 | 0 | 0 |
| 1/7 | 0,309893194 | 1 | 0 | 0 |
| 1/7 | 0,309893194 | 1 | 0 | 0 |
| 1/7 | 0,309893194 | 1 | 0 | 0 |
| 1/7 | 0,309893194 | 1 | 0 | 0 |
| 1/7 | 0,309893194 | 1 | 0 | 0 |
| 1/7 | 0,309893194 | 1 | 0 | 0 |
| 1/7 | 0,309893194 | 1 | 0 | 0 |
| 1/7 | 0,309893194 | 1 | 0 | 0 |
| 1/7 | 0,309893194 | 1 | 0 | 0 |
| 1/7 | 0,309893194 | 1 | 0 | 0 |
| 1/7 | 0,309893194 | 1 | 0 | 0 |
| 1/7 | 0,309893194 | 1 | 0 | 0 |
| 1/7 | 0,309893194 | 1 | 0 | 0 |
| 1/7 | 0,309893194 | 1 | 0 | 0 |
| 1/7 | 0,309893194 | 1 | 0 | 0 |
| 1/7 | 0,309893194 | 1 | 0 | 0 |
| 1/7 | 0,309893194 | 1 | 0 | 0 |
| 1/7 | 0,309893194 | 1 | 0 | 0 |

| | | | | |
|---|---|---|---|---|
| 1/7 | 0,309893194 | 1 | 0 | 0 |
| 1/7 | 0,309893194 | 1 | 0 | 0 |
| 1/7 | 0,309893194 | 1 | 0 | 0 |
| 1/7 | 0,309893194 | 1 | 0 | 0 |
| 1/7 | 0,309893194 | 1 | 0 | 0 |
| 1/7 | 0,309893194 | 1 | 0 | 0 |
| 1/7 | 0,309893194 | 1 | 0 | 0 |
| 1/7 | 0,309893194 | 1 | 0 | 0 |
| 1/7 | 0,309893194 | 1 | 0 | 0 |
| 1/7 | 0,309893194 | 1 | 0 | 0 |
| 1/7 | 0,309893194 | 1 | 0 | 0 |
| 1/7 | 0,309893194 | 1 | 0 | 0 |
| 1/7 | 0,309893194 | 1 | 0 | 0 |
| 1/7 | 0,309893194 | 1 | 0 | 0 |
| 1/7 | 0,309893194 | 1 | 0 | 0 |
| 1/7 | 0,309893194 | 1 | 0 | 0 |
| 6/89 | 0,310817292 | 1 | 0 | 0 |
| 6/89 | 0,310817292 | 1 | 0 | 0 |
| 3/38 | 0,312340461 | 1 | 0 | 0 |
| 3/38 | 0,312340461 | 1 | 0 | 0 |
| 3/38 | 0,312340461 | 1 | 0 | 0 |
| 3/38 | 0,312340461 | 1 | 0 | 0 |
| 3/38 | 0,312340461 | 1 | 0 | 0 |
| 3/38 | 0,312340461 | 1 | 0 | 0 |
| 3/38 | 0,312340461 | 1 | 0 | 0 |
| 3/38 | 0,312340461 | 1 | 0 | 0 |
| 11/178 | 0,312982306 | 1 | 0 | 0 |
| 5/72 | 0,314069009 | 1 | 0 | 0 |
| 7/107 | 0,315004196 | 1 | 0 | 0 |
| 2/22 | 0,315101353 | 1 | 0 | 0 |
| 2/22 | 0,315101353 | 1 | 0 | 0 |
| 2/22 | 0,315101353 | 1 | 0 | 0 |
| 2/22 | 0,315101353 | 1 | 0 | 0 |
| 2/22 | 0,315101353 | 1 | 0 | 0 |
| 2/22 | 0,315101353 | 1 | 0 | 0 |
| 2/22 | 0,315101353 | 1 | 0 | 0 |
| 2/22 | 0,315101353 | 1 | 0 | 0 |
| 2/22 | 0,315101353 | 1 | 0 | 0 |
| 2/22 | 0,315101353 | 1 | 0 | 0 |
| 2/22 | 0,315101353 | 1 | 0 | 0 |
| 2/22 | 0,315101353 | 1 | 0 | 0 |
| 2/22 | 0,315101353 | 1 | 0 | 0 |
| 2/22 | 0,315101353 | 1 | 0 | 0 |
| 2/22 | 0,315101353 | 1 | 0 | 0 |
| 2/22 | 0,315101353 | 1 | 0 | 0 |
| 2/22 | 0,315101353 | 1 | 0 | 0 |
| 2/22 | 0,315101353 | 1 | 0 | 0 |
| 2/22 | 0,315101353 | 1 | 0 | 0 |
| 2/22 | 0,315101353 | 1 | 0 | 0 |
| 4/55 | 0,315434093 | 1 | 0 | 0 |
| 4/55 | 0,315434093 | 1 | 0 | 0 |
| 6/90 | 0,319982781 | 1 | 0 | 0 |
| 7/108 | 0,323374032 | 1 | 0 | 0 |

| | | | | |
|---|---|---|---|---|
| 7/108 | 0,323374032 | 1 | 0 | 0 |
| 5/73 | 0,324342097 | 1 | 0 | 0 |
| 8/126 | 0,3252207 | 1 | 0 | 0 |
| 10/162 | 0,326206072 | 1 | 0 | 0 |
| 3/39 | 0,326695464 | 1 | 0 | 0 |
| 2/23 | 0,334368124 | 1 | 0 | 0 |
| 2/23 | 0,334368124 | 1 | 0 | 0 |
| 2/23 | 0,334368124 | 1 | 0 | 0 |
| 2/23 | 0,334368124 | 1 | 0 | 0 |
| 2/23 | 0,334368124 | 1 | 0 | 0 |
| 2/23 | 0,334368124 | 1 | 0 | 0 |
| 2/23 | 0,334368124 | 1 | 0 | 0 |
| 2/23 | 0,334368124 | 1 | 0 | 0 |
| 2/23 | 0,334368124 | 1 | 0 | 0 |
| 2/23 | 0,334368124 | 1 | 0 | 0 |
| 2/23 | 0,334368124 | 1 | 0 | 0 |
| 2/23 | 0,334368124 | 1 | 0 | 0 |
| 2/23 | 0,334368124 | 1 | 0 | 0 |
| 2/23 | 0,334368124 | 1 | 0 | 0 |
| 2/23 | 0,334368124 | 1 | 0 | 0 |
| 2/23 | 0,334368124 | 1 | 0 | 0 |
| 2/23 | 0,334368124 | 1 | 0 | 0 |
| 5/74 | 0,334650579 | 1 | 0 | 0 |
| 6/92 | 0,338425072 | 1 | 0 | 0 |
| 11/182 | 0,338857917 | 1 | 0 | 0 |
| 4/57 | 0,339152763 | 1 | 0 | 0 |
| 4/57 | 0,339152763 | 1 | 0 | 0 |
| 10/164 | 0,339922468 | 1 | 0 | 0 |
| 10/164 | 0,339922468 | 1 | 0 | 0 |
| 7/110 | 0,340216351 | 1 | 0 | 0 |
| 9/146 | 0,340625203 | 1 | 0 | 0 |
| 8/128 | 0,34080738 | 1 | 0 | 0 |
| 3/40 | 0,341046404 | 1 | 0 | 0 |
| 3/40 | 0,341046404 | 1 | 0 | 0 |
| 3/40 | 0,341046404 | 1 | 0 | 0 |
| 3/40 | 0,341046404 | 1 | 0 | 0 |
| 5/75 | 0,34498645 | 1 | 0 | 0 |
| 5/75 | 0,34498645 | 1 | 0 | 0 |
| 5/75 | 0,34498645 | 1 | 0 | 0 |
| 1/8 | 0,345515118 | 1 | 0 | 0 |
| 1/8 | 0,345515118 | 1 | 0 | 0 |
| 1/8 | 0,345515118 | 1 | 0 | 0 |
| 1/8 | 0,345515118 | 1 | 0 | 0 |
| 1/8 | 0,345515118 | 1 | 0 | 0 |
| 1/8 | 0,345515118 | 1 | 0 | 0 |
| 1/8 | 0,345515118 | 1 | 0 | 0 |
| 1/8 | 0,345515118 | 1 | 0 | 0 |
| 1/8 | 0,345515118 | 1 | 0 | 0 |
| 1/8 | 0,345515118 | 1 | 0 | 0 |
| 1/8 | 0,345515118 | 1 | 0 | 0 |
| 1/8 | 0,345515118 | 1 | 0 | 0 |
| 1/8 | 0,345515118 | 1 | 0 | 0 |
| 1/8 | 0,345515118 | 1 | 0 | 0 |

| | | | | |
|---|---|---|---|---|
| 1/8 | 0,345515118 | 1 | 0 | 0 |
| 1/8 | 0,345515118 | 1 | 0 | 0 |
| 1/8 | 0,345515118 | 1 | 0 | 0 |
| 1/8 | 0,345515118 | 1 | 0 | 0 |
| 1/8 | 0,345515118 | 1 | 0 | 0 |
| 1/8 | 0,345515118 | 1 | 0 | 0 |
| 1/8 | 0,345515118 | 1 | 0 | 0 |
| 1/8 | 0,345515118 | 1 | 0 | 0 |
| 1/8 | 0,345515118 | 1 | 0 | 0 |
| 1/8 | 0,345515118 | 1 | 0 | 0 |
| 1/8 | 0,345515118 | 1 | 0 | 0 |
| 1/8 | 0,345515118 | 1 | 0 | 0 |
| 1/8 | 0,345515118 | 1 | 0 | 0 |
| 1/8 | 0,345515118 | 1 | 0 | 0 |
| 1/8 | 0,345515118 | 1 | 0 | 0 |
| 1/8 | 0,345515118 | 1 | 0 | 0 |
| 1/8 | 0,345515118 | 1 | 0 | 0 |
| 1/8 | 0,345515118 | 1 | 0 | 0 |
| 1/8 | 0,345515118 | 1 | 0 | 0 |
| 1/8 | 0,345515118 | 1 | 0 | 0 |
| 1/8 | 0,345515118 | 1 | 0 | 0 |
| 1/8 | 0,345515118 | 1 | 0 | 0 |
| 1/8 | 0,345515118 | 1 | 0 | 0 |
| 1/8 | 0,345515118 | 1 | 0 | 0 |
| 1/8 | 0,345515118 | 1 | 0 | 0 |
| 1/8 | 0,345515118 | 1 | 0 | 0 |
| 1/8 | 0,345515118 | 1 | 0 | 0 |
| 1/8 | 0,345515118 | 1 | 0 | 0 |
| 1/8 | 0,345515118 | 1 | 0 | 0 |
| 1/8 | 0,345515118 | 1 | 0 | 0 |
| 1/8 | 0,345515118 | 1 | 0 | 0 |
| 1/8 | 0,345515118 | 1 | 0 | 0 |
| 1/8 | 0,345515118 | 1 | 0 | 0 |
| 1/8 | 0,345515118 | 1 | 0 | 0 |
| 1/8 | 0,345515118 | 1 | 0 | 0 |
| 1/8 | 0,345515118 | 1 | 0 | 0 |
| 1/8 | 0,345515118 | 1 | 0 | 0 |
| 1/8 | 0,345515118 | 1 | 0 | 0 |
| 1/8 | 0,345515118 | 1 | 0 | 0 |
| 1/8 | 0,345515118 | 1 | 0 | 0 |
| 1/8 | 0,345515118 | 1 | 0 | 0 |
| 1/8 | 0,345515118 | 1 | 0 | 0 |
| 1/8 | 0,345515118 | 1 | 0 | 0 |
| 1/8 | 0,345515118 | 1 | 0 | 0 |
| 1/8 | 0,345515118 | 1 | 0 | 0 |
| 1/8 | 0,345515118 | 1 | 0 | 0 |
| 1/8 | 0,345515118 | 1 | 0 | 0 |
| 1/8 | 0,345515118 | 1 | 0 | 0 |
| 1/8 | 0,345515118 | 1 | 0 | 0 |
| 1/8 | 0,345515118 | 1 | 0 | 0 |
| 1/8 | 0,345515118 | 1 | 0 | 0 |
| 1/8 | 0,345515118 | 1 | 0 | 0 |
| 1/8 | 0,345515118 | 1 | 0 | 0 |

| | | | | |
|---|---|---|---|---|
| 1/8 | 0,345515118 | 1 | 0 | 0 |
| 1/8 | 0,345515118 | 1 | 0 | 0 |
| 1/8 | 0,345515118 | 1 | 0 | 0 |
| 1/8 | 0,345515118 | 1 | 0 | 0 |
| 1/8 | 0,345515118 | 1 | 0 | 0 |
| 1/8 | 0,345515118 | 1 | 0 | 0 |
| 1/8 | 0,345515118 | 1 | 0 | 0 |
| 1/8 | 0,345515118 | 1 | 0 | 0 |
| 1/8 | 0,345515118 | 1 | 0 | 0 |
| 1/8 | 0,345515118 | 1 | 0 | 0 |
| 1/8 | 0,345515118 | 1 | 0 | 0 |
| 1/8 | 0,345515118 | 1 | 0 | 0 |
| 1/8 | 0,345515118 | 1 | 0 | 0 |
| 1/8 | 0,345515118 | 1 | 0 | 0 |
| 1/8 | 0,345515118 | 1 | 0 | 0 |
| 7/111 | 0,348680023 | 1 | 0 | 0 |
| 12/202 | 0,349995105 | 1 | 0 | 0 |
| 4/58 | 0,351038378 | 1 | 0 | 0 |
| 4/58 | 0,351038378 | 1 | 0 | 0 |
| 2/24 | 0,353472123 | 1 | 0 | 0 |
| 5/76 | 0,355341865 | 1 | 0 | 0 |
| 5/76 | 0,355341865 | 1 | 0 | 0 |
| 5/76 | 0,355341865 | 1 | 0 | 0 |
| 3/41 | 0,355373883 | 1 | 0 | 0 |
| 3/41 | 0,355373883 | 1 | 0 | 0 |
| 3/41 | 0,355373883 | 1 | 0 | 0 |
| 3/41 | 0,355373883 | 1 | 0 | 0 |
| 3/41 | 0,355373883 | 1 | 0 | 0 |
| 7/112 | 0,357166224 | 1 | 0 | 0 |
| 13/222 | 0,359921265 | 1 | 0 | 0 |
| 4/59 | 0,362926182 | 1 | 0 | 0 |
| 4/59 | 0,362926182 | 1 | 0 | 0 |
| 4/59 | 0,362926182 | 1 | 0 | 0 |
| 23/409 | 0,365344325 | 1 | 0 | 0 |
| 6/95 | 0,366280435 | 1 | 0 | 0 |
| 10/168 | 0,367629135 | 1 | 0 | 0 |
| 3/42 | 0,369659561 | 1 | 0 | 0 |
| 3/42 | 0,369659561 | 1 | 0 | 0 |
| 3/42 | 0,369659561 | 1 | 0 | 0 |
| 9/150 | 0,370038864 | 1 | 0 | 0 |
| 2/25 | 0,372378865 | 1 | 0 | 0 |
| 2/25 | 0,372378865 | 1 | 0 | 0 |
| 2/25 | 0,372378865 | 1 | 0 | 0 |
| 2/25 | 0,372378865 | 1 | 0 | 0 |
| 2/25 | 0,372378865 | 1 | 0 | 0 |
| 2/25 | 0,372378865 | 1 | 0 | 0 |
| 2/25 | 0,372378865 | 1 | 0 | 0 |
| 2/25 | 0,372378865 | 1 | 0 | 0 |
| 2/25 | 0,372378865 | 1 | 0 | 0 |
| 2/25 | 0,372378865 | 1 | 0 | 0 |
| 2/25 | 0,372378865 | 1 | 0 | 0 |
| 2/25 | 0,372378865 | 1 | 0 | 0 |
| 2/25 | 0,372378865 | 1 | 0 | 0 |

| | | | | |
|---|---|---|---|---|
| 2/25 | 0,372378865 | 1 | 0 | 0 |
| 2/25 | 0,372378865 | 1 | 0 | 0 |
| 2/25 | 0,372378865 | 1 | 0 | 0 |
| 2/25 | 0,372378865 | 1 | 0 | 0 |
| 1/9 | 0,379299997 | 1 | 0 | 0 |
| 1/9 | 0,379299997 | 1 | 0 | 0 |
| 1/9 | 0,379299997 | 1 | 0 | 0 |
| 1/9 | 0,379299997 | 1 | 0 | 0 |
| 1/9 | 0,379299997 | 1 | 0 | 0 |
| 1/9 | 0,379299997 | 1 | 0 | 0 |
| 1/9 | 0,379299997 | 1 | 0 | 0 |
| 1/9 | 0,379299997 | 1 | 0 | 0 |
| 1/9 | 0,379299997 | 1 | 0 | 0 |
| 1/9 | 0,379299997 | 1 | 0 | 0 |
| 1/9 | 0,379299997 | 1 | 0 | 0 |
| 1/9 | 0,379299997 | 1 | 0 | 0 |
| 1/9 | 0,379299997 | 1 | 0 | 0 |
| 1/9 | 0,379299997 | 1 | 0 | 0 |
| 1/9 | 0,379299997 | 1 | 0 | 0 |
| 1/9 | 0,379299997 | 1 | 0 | 0 |
| 1/9 | 0,379299997 | 1 | 0 | 0 |
| 1/9 | 0,379299997 | 1 | 0 | 0 |
| 1/9 | 0,379299997 | 1 | 0 | 0 |
| 1/9 | 0,379299997 | 1 | 0 | 0 |
| 1/9 | 0,379299997 | 1 | 0 | 0 |
| 1/9 | 0,379299997 | 1 | 0 | 0 |
| 1/9 | 0,379299997 | 1 | 0 | 0 |
| 1/9 | 0,379299997 | 1 | 0 | 0 |
| 1/9 | 0,379299997 | 1 | 0 | 0 |
| 1/9 | 0,379299997 | 1 | 0 | 0 |
| 1/9 | 0,379299997 | 1 | 0 | 0 |
| 1/9 | 0,379299997 | 1 | 0 | 0 |
| 1/9 | 0,379299997 | 1 | 0 | 0 |
| 1/9 | 0,379299997 | 1 | 0 | 0 |
| 1/9 | 0,379299997 | 1 | 0 | 0 |
| 1/9 | 0,379299997 | 1 | 0 | 0 |
| 1/9 | 0,379299997 | 1 | 0 | 0 |
| 1/9 | 0,379299997 | 1 | 0 | 0 |
| 1/9 | 0,379299997 | 1 | 0 | 0 |
| 1/9 | 0,379299997 | 1 | 0 | 0 |
| 1/9 | 0,379299997 | 1 | 0 | 0 |
| 1/9 | 0,379299997 | 1 | 0 | 0 |
| 1/9 | 0,379299997 | 1 | 0 | 0 |
| 1/9 | 0,379299997 | 1 | 0 | 0 |
| 1/9 | 0,379299997 | 1 | 0 | 0 |
| 1/9 | 0,379299997 | 1 | 0 | 0 |
| 1/9 | 0,379299997 | 1 | 0 | 0 |
| 1/9 | 0,379299997 | 1 | 0 | 0 |
| 1/9 | 0,379299997 | 1 | 0 | 0 |
| 1/9 | 0,379299997 | 1 | 0 | 0 |
| 1/9 | 0,379299997 | 1 | 0 | 0 |
| 1/9 | 0,379299997 | 1 | 0 | 0 |
| 1/9 | 0,379299997 | 1 | 0 | 0 |

| | | | | |
|---|---|---|---|---|
| 1/9 | 0,379299997 | 1 | 0 | 0 |
| 1/9 | 0,379299997 | 1 | 0 | 0 |
| 1/9 | 0,379299997 | 1 | 0 | 0 |
| 1/9 | 0,379299997 | 1 | 0 | 0 |
| 1/9 | 0,379299997 | 1 | 0 | 0 |
| 1/9 | 0,379299997 | 1 | 0 | 0 |
| 1/9 | 0,379299997 | 1 | 0 | 0 |
| 1/9 | 0,379299997 | 1 | 0 | 0 |
| 1/9 | 0,379299997 | 1 | 0 | 0 |
| 1/9 | 0,379299997 | 1 | 0 | 0 |
| 1/9 | 0,379299997 | 1 | 0 | 0 |
| 1/9 | 0,379299997 | 1 | 0 | 0 |
| 1/9 | 0,379299997 | 1 | 0 | 0 |
| 1/9 | 0,379299997 | 1 | 0 | 0 |
| 1/9 | 0,379299997 | 1 | 0 | 0 |
| 1/9 | 0,379299997 | 1 | 0 | 0 |
| 1/9 | 0,379299997 | 1 | 0 | 0 |
| 1/9 | 0,379299997 | 1 | 0 | 0 |
| 1/9 | 0,379299997 | 1 | 0 | 0 |
| 1/9 | 0,379299997 | 1 | 0 | 0 |
| 1/9 | 0,379299997 | 1 | 0 | 0 |
| 1/9 | 0,379299997 | 1 | 0 | 0 |
| 1/9 | 0,379299997 | 1 | 0 | 0 |
| 1/9 | 0,379299997 | 1 | 0 | 0 |
| 1/9 | 0,379299997 | 1 | 0 | 0 |
| 1/9 | 0,379299997 | 1 | 0 | 0 |
| 1/9 | 0,379299997 | 1 | 0 | 0 |
| 1/9 | 0,379299997 | 1 | 0 | 0 |
| 1/9 | 0,379299997 | 1 | 0 | 0 |
| 1/9 | 0,379299997 | 1 | 0 | 0 |
| 1/9 | 0,379299997 | 1 | 0 | 0 |
| 1/9 | 0,379299997 | 1 | 0 | 0 |
| 1/9 | 0,379299997 | 1 | 0 | 0 |
| 1/9 | 0,379299997 | 1 | 0 | 0 |
| 1/9 | 0,379299997 | 1 | 0 | 0 |
| 1/9 | 0,379299997 | 1 | 0 | 0 |
| 1/9 | 0,379299997 | 1 | 0 | 0 |
| 1/9 | 0,379299997 | 1 | 0 | 0 |
| 1/9 | 0,379299997 | 1 | 0 | 0 |
| 1/9 | 0,379299997 | 1 | 0 | 0 |
| 1/9 | 0,379299997 | 1 | 0 | 0 |
| 1/9 | 0,379299997 | 1 | 0 | 0 |
| 1/9 | 0,379299997 | 1 | 0 | 0 |
| 1/9 | 0,379299997 | 1 | 0 | 0 |
| 1/9 | 0,379299997 | 1 | 0 | 0 |
| 3/43 | 0,383886151 | 1 | 0 | 0 |
| 6/97 | 0,384913372 | 1 | 0 | 0 |
| 11/189 | 0,384971542 | 1 | 0 | 0 |
| 5/79 | 0,386449512 | 1 | 0 | 0 |
| 5/79 | 0,386449512 | 1 | 0 | 0 |
| 20/358 | 0,38967813 | 1 | 0 | 0 |
| 2/26 | 0,39105785 | 1 | 0 | 0 |
| 2/26 | 0,39105785 | 1 | 0 | 0 |

| | | | | |
|---|---|---|---|---|
| 2/26 | 0,39105785 | 1 | 0 | 0 |
| 2/26 | 0,39105785 | 1 | 0 | 0 |
| 2/26 | 0,39105785 | 1 | 0 | 0 |
| 2/26 | 0,39105785 | 1 | 0 | 0 |
| 2/26 | 0,39105785 | 1 | 0 | 0 |
| 2/26 | 0,39105785 | 1 | 0 | 0 |
| 2/26 | 0,39105785 | 1 | 0 | 0 |
| 2/26 | 0,39105785 | 1 | 0 | 0 |
| 2/26 | 0,39105785 | 1 | 0 | 0 |
| 2/26 | 0,39105785 | 1 | 0 | 0 |
| 2/26 | 0,39105785 | 1 | 0 | 0 |
| 2/26 | 0,39105785 | 1 | 0 | 0 |
| 2/26 | 0,39105785 | 1 | 0 | 0 |
| 2/26 | 0,39105785 | 1 | 0 | 0 |
| 2/26 | 0,39105785 | 1 | 0 | 0 |
| 9/153 | 0,39225613 | 1 | 0 | 0 |
| 6/98 | 0,394232438 | 1 | 0 | 0 |
| 6/98 | 0,394232438 | 1 | 0 | 0 |
| 8/135 | 0,396002874 | 1 | 0 | 0 |
| 3/44 | 0,398037404 | 1 | 0 | 0 |
| 3/44 | 0,398037404 | 1 | 0 | 0 |
| 3/44 | 0,398037404 | 1 | 0 | 0 |
| 3/44 | 0,398037404 | 1 | 0 | 0 |
| 3/44 | 0,398037404 | 1 | 0 | 0 |
| 3/44 | 0,398037404 | 1 | 0 | 0 |
| 3/44 | 0,398037404 | 1 | 0 | 0 |
| 11/191 | 0,398263196 | 1 | 0 | 0 |
| 4/62 | 0,398494592 | 1 | 0 | 0 |
| 4/62 | 0,398494592 | 1 | 0 | 0 |
| 4/62 | 0,398494592 | 1 | 0 | 0 |
| 4/62 | 0,398494592 | 1 | 0 | 0 |
| 7/117 | 0,399786482 | 1 | 0 | 0 |
| 6/99 | 0,403546277 | 1 | 0 | 0 |
| 8/136 | 0,403924514 | 1 | 0 | 0 |
| 5/81 | 0,407149855 | 1 | 0 | 0 |
| 33/608 | 0,407187018 | 1 | 0 | 0 |
| 7/118 | 0,408319465 | 1 | 0 | 0 |
| 13/230 | 0,4083821 | 1 | 0 | 0 |
| 2/27 | 0,409482246 | 1 | 0 | 0 |
| 2/27 | 0,409482246 | 1 | 0 | 0 |
| 2/27 | 0,409482246 | 1 | 0 | 0 |
| 2/27 | 0,409482246 | 1 | 0 | 0 |
| 2/27 | 0,409482246 | 1 | 0 | 0 |
| 2/27 | 0,409482246 | 1 | 0 | 0 |
| 2/27 | 0,409482246 | 1 | 0 | 0 |
| 2/27 | 0,409482246 | 1 | 0 | 0 |
| 2/27 | 0,409482246 | 1 | 0 | 0 |
| 4/63 | 0,410285043 | 1 | 0 | 0 |
| 4/63 | 0,410285043 | 1 | 0 | 0 |
| 4/63 | 0,410285043 | 1 | 0 | 0 |
| 1/10 | 0,411342481 | 1 | 0 | 0 |
| 1/10 | 0,411342481 | 1 | 0 | 0 |
| 1/10 | 0,411342481 | 1 | 0 | 0 |

| | | | | |
|---|---|---|---|---|
| 1/10 | 0,411342481 | 1 | 0 | 0 |
| 1/10 | 0,411342481 | 1 | 0 | 0 |
| 1/10 | 0,411342481 | 1 | 0 | 0 |
| 1/10 | 0,411342481 | 1 | 0 | 0 |
| 1/10 | 0,411342481 | 1 | 0 | 0 |
| 1/10 | 0,411342481 | 1 | 0 | 0 |
| 1/10 | 0,411342481 | 1 | 0 | 0 |
| 1/10 | 0,411342481 | 1 | 0 | 0 |
| 1/10 | 0,411342481 | 1 | 0 | 0 |
| 1/10 | 0,411342481 | 1 | 0 | 0 |
| 1/10 | 0,411342481 | 1 | 0 | 0 |
| 1/10 | 0,411342481 | 1 | 0 | 0 |
| 1/10 | 0,411342481 | 1 | 0 | 0 |
| 1/10 | 0,411342481 | 1 | 0 | 0 |
| 1/10 | 0,411342481 | 1 | 0 | 0 |
| 1/10 | 0,411342481 | 1 | 0 | 0 |
| 1/10 | 0,411342481 | 1 | 0 | 0 |
| 1/10 | 0,411342481 | 1 | 0 | 0 |
| 1/10 | 0,411342481 | 1 | 0 | 0 |
| 1/10 | 0,411342481 | 1 | 0 | 0 |
| 1/10 | 0,411342481 | 1 | 0 | 0 |
| 1/10 | 0,411342481 | 1 | 0 | 0 |
| 1/10 | 0,411342481 | 1 | 0 | 0 |
| 1/10 | 0,411342481 | 1 | 0 | 0 |
| 1/10 | 0,411342481 | 1 | 0 | 0 |
| 1/10 | 0,411342481 | 1 | 0 | 0 |
| 1/10 | 0,411342481 | 1 | 0 | 0 |
| 1/10 | 0,411342481 | 1 | 0 | 0 |
| 1/10 | 0,411342481 | 1 | 0 | 0 |
| 1/10 | 0,411342481 | 1 | 0 | 0 |
| 1/10 | 0,411342481 | 1 | 0 | 0 |
| 1/10 | 0,411342481 | 1 | 0 | 0 |
| 1/10 | 0,411342481 | 1 | 0 | 0 |
| 1/10 | 0,411342481 | 1 | 0 | 0 |
| 1/10 | 0,411342481 | 1 | 0 | 0 |
| 1/10 | 0,411342481 | 1 | 0 | 0 |
| 1/10 | 0,411342481 | 1 | 0 | 0 |
| 1/10 | 0,411342481 | 1 | 0 | 0 |
| 1/10 | 0,411342481 | 1 | 0 | 0 |
| 1/10 | 0,411342481 | 1 | 0 | 0 |
| 1/10 | 0,411342481 | 1 | 0 | 0 |
| 1/10 | 0,411342481 | 1 | 0 | 0 |
| 1/10 | 0,411342481 | 1 | 0 | 0 |
| 1/10 | 0,411342481 | 1 | 0 | 0 |
| 1/10 | 0,411342481 | 1 | 0 | 0 |
| 1/10 | 0,411342481 | 1 | 0 | 0 |
| 1/10 | 0,411342481 | 1 | 0 | 0 |
| 1/10 | 0,411342481 | 1 | 0 | 0 |
| 1/10 | 0,411342481 | 1 | 0 | 0 |
| 1/10 | 0,411342481 | 1 | 0 | 0 |
| 1/10 | 0,411342481 | 1 | 0 | 0 |
| 1/10 | 0,411342481 | 1 | 0 | 0 |
| 1/10 | 0,411342481 | 1 | 0 | 0 |

| | | | | |
|---|---|---|---|---|
| 1/10 | 0,411342481 | 1 | 0 | 0 |
| 1/10 | 0,411342481 | 1 | 0 | 0 |
| 1/10 | 0,411342481 | 1 | 0 | 0 |
| 1/10 | 0,411342481 | 1 | 0 | 0 |
| 1/10 | 0,411342481 | 1 | 0 | 0 |
| 1/10 | 0,411342481 | 1 | 0 | 0 |
| 1/10 | 0,411342481 | 1 | 0 | 0 |
| 1/10 | 0,411342481 | 1 | 0 | 0 |
| 1/10 | 0,411342481 | 1 | 0 | 0 |
| 1/10 | 0,411342481 | 1 | 0 | 0 |
| 1/10 | 0,411342481 | 1 | 0 | 0 |
| 1/10 | 0,411342481 | 1 | 0 | 0 |
| 1/10 | 0,411342481 | 1 | 0 | 0 |
| 1/10 | 0,411342481 | 1 | 0 | 0 |
| 1/10 | 0,411342481 | 1 | 0 | 0 |
| 1/10 | 0,411342481 | 1 | 0 | 0 |
| 1/10 | 0,411342481 | 1 | 0 | 0 |
| 1/10 | 0,411342481 | 1 | 0 | 0 |
| 1/10 | 0,411342481 | 1 | 0 | 0 |
| 1/10 | 0,411342481 | 1 | 0 | 0 |
| 1/10 | 0,411342481 | 1 | 0 | 0 |
| 1/10 | 0,411342481 | 1 | 0 | 0 |
| 1/10 | 0,411342481 | 1 | 0 | 0 |
| 1/10 | 0,411342481 | 1 | 0 | 0 |
| 1/10 | 0,411342481 | 1 | 0 | 0 |
| 1/10 | 0,411342481 | 1 | 0 | 0 |
| 1/10 | 0,411342481 | 1 | 0 | 0 |
| 1/10 | 0,411342481 | 1 | 0 | 0 |
| 1/10 | 0,411342481 | 1 | 0 | 0 |
| 1/10 | 0,411342481 | 1 | 0 | 0 |
| 1/10 | 0,411342481 | 1 | 0 | 0 |
| 3/45 | 0,412098092 | 1 | 0 | 0 |
| 10/175 | 0,416604554 | 1 | 0 | 0 |
| 4/64 | 0,422026674 | 1 | 0 | 0 |
| 4/64 | 0,422026674 | 1 | 0 | 0 |
| 4/64 | 0,422026674 | 1 | 0 | 0 |
| 3/46 | 0,426053988 | 1 | 0 | 0 |
| 3/46 | 0,426053988 | 1 | 0 | 0 |
| 2/28 | 0,42762859 | 1 | 0 | 0 |
| 2/28 | 0,42762859 | 1 | 0 | 0 |
| 2/28 | 0,42762859 | 1 | 0 | 0 |
| 2/28 | 0,42762859 | 1 | 0 | 0 |
| 2/28 | 0,42762859 | 1 | 0 | 0 |
| 2/28 | 0,42762859 | 1 | 0 | 0 |
| 2/28 | 0,42762859 | 1 | 0 | 0 |
| 2/28 | 0,42762859 | 1 | 0 | 0 |
| 2/28 | 0,42762859 | 1 | 0 | 0 |
| 2/28 | 0,42762859 | 1 | 0 | 0 |
| 2/28 | 0,42762859 | 1 | 0 | 0 |
| 2/28 | 0,42762859 | 1 | 0 | 0 |
| 2/28 | 0,42762859 | 1 | 0 | 0 |
| 2/28 | 0,42762859 | 1 | 0 | 0 |
| 2/28 | 0,42762859 | 1 | 0 | 0 |

| | | | | |
|---|---|---|---|---|
| 2/28 | 0,42762859 | 1 | 0 | 0 |
| 8/139 | 0,427672846 | 1 | 0 | 0 |
| 6/102 | 0,431406295 | 1 | 0 | 0 |
| 6/102 | 0,431406295 | 1 | 0 | 0 |
| 13/234 | 0,432757718 | 1 | 0 | 0 |
| 19/348 | 0,433496739 | 1 | 0 | 0 |
| 4/65 | 0,433710529 | 1 | 0 | 0 |
| 7/121 | 0,433864963 | 1 | 0 | 0 |
| 12/216 | 0,438607639 | 1 | 0 | 0 |
| 3/47 | 0,439891843 | 1 | 0 | 0 |
| 3/47 | 0,439891843 | 1 | 0 | 0 |
| 3/47 | 0,439891843 | 1 | 0 | 0 |
| 1/11 | 0,441732347 | 1 | 0 | 0 |
| 1/11 | 0,441732347 | 1 | 0 | 0 |
| 1/11 | 0,441732347 | 1 | 0 | 0 |
| 1/11 | 0,441732347 | 1 | 0 | 0 |
| 1/11 | 0,441732347 | 1 | 0 | 0 |
| 1/11 | 0,441732347 | 1 | 0 | 0 |
| 1/11 | 0,441732347 | 1 | 0 | 0 |
| 1/11 | 0,441732347 | 1 | 0 | 0 |
| 1/11 | 0,441732347 | 1 | 0 | 0 |
| 1/11 | 0,441732347 | 1 | 0 | 0 |
| 1/11 | 0,441732347 | 1 | 0 | 0 |
| 1/11 | 0,441732347 | 1 | 0 | 0 |
| 1/11 | 0,441732347 | 1 | 0 | 0 |
| 1/11 | 0,441732347 | 1 | 0 | 0 |
| 1/11 | 0,441732347 | 1 | 0 | 0 |
| 1/11 | 0,441732347 | 1 | 0 | 0 |
| 1/11 | 0,441732347 | 1 | 0 | 0 |
| 1/11 | 0,441732347 | 1 | 0 | 0 |
| 1/11 | 0,441732347 | 1 | 0 | 0 |
| 1/11 | 0,441732347 | 1 | 0 | 0 |
| 1/11 | 0,441732347 | 1 | 0 | 0 |
| 1/11 | 0,441732347 | 1 | 0 | 0 |
| 1/11 | 0,441732347 | 1 | 0 | 0 |
| 1/11 | 0,441732347 | 1 | 0 | 0 |
| 1/11 | 0,441732347 | 1 | 0 | 0 |
| 1/11 | 0,441732347 | 1 | 0 | 0 |
| 1/11 | 0,441732347 | 1 | 0 | 0 |
| 1/11 | 0,441732347 | 1 | 0 | 0 |
| 1/11 | 0,441732347 | 1 | 0 | 0 |
| 1/11 | 0,441732347 | 1 | 0 | 0 |
| 1/11 | 0,441732347 | 1 | 0 | 0 |
| 1/11 | 0,441732347 | 1 | 0 | 0 |
| 1/11 | 0,441732347 | 1 | 0 | 0 |
| 1/11 | 0,441732347 | 1 | 0 | 0 |
| 1/11 | 0,441732347 | 1 | 0 | 0 |
| 1/11 | 0,441732347 | 1 | 0 | 0 |
| 1/11 | 0,441732347 | 1 | 0 | 0 |
| 1/11 | 0,441732347 | 1 | 0 | 0 |
| 1/11 | 0,441732347 | 1 | 0 | 0 |
| 1/11 | 0,441732347 | 1 | 0 | 0 |
| 1/11 | 0,441732347 | 1 | 0 | 0 |

| | | | | |
|---|---|---|---|---|
| 1/11 | 0,441732347 | 1 | 0 | 0 |
| 1/11 | 0,441732347 | 1 | 0 | 0 |
| 1/11 | 0,441732347 | 1 | 0 | 0 |
| 1/11 | 0,441732347 | 1 | 0 | 0 |
| 1/11 | 0,441732347 | 1 | 0 | 0 |
| 1/11 | 0,441732347 | 1 | 0 | 0 |
| 1/11 | 0,441732347 | 1 | 0 | 0 |
| 1/11 | 0,441732347 | 1 | 0 | 0 |
| 1/11 | 0,441732347 | 1 | 0 | 0 |
| 1/11 | 0,441732347 | 1 | 0 | 0 |
| 1/11 | 0,441732347 | 1 | 0 | 0 |
| 1/11 | 0,441732347 | 1 | 0 | 0 |
| 1/11 | 0,441732347 | 1 | 0 | 0 |
| 1/11 | 0,441732347 | 1 | 0 | 0 |
| 1/11 | 0,441732347 | 1 | 0 | 0 |
| 1/11 | 0,441732347 | 1 | 0 | 0 |
| 1/11 | 0,441732347 | 1 | 0 | 0 |
| 1/11 | 0,441732347 | 1 | 0 | 0 |
| 1/11 | 0,441732347 | 1 | 0 | 0 |
| 1/11 | 0,441732347 | 1 | 0 | 0 |
| 1/11 | 0,441732347 | 1 | 0 | 0 |
| 1/11 | 0,441732347 | 1 | 0 | 0 |
| 1/11 | 0,441732347 | 1 | 0 | 0 |
| 1/11 | 0,441732347 | 1 | 0 | 0 |
| 1/11 | 0,441732347 | 1 | 0 | 0 |
| 1/11 | 0,441732347 | 1 | 0 | 0 |
| 1/11 | 0,441732347 | 1 | 0 | 0 |
| 1/11 | 0,441732347 | 1 | 0 | 0 |
| 1/11 | 0,441732347 | 1 | 0 | 0 |
| 1/11 | 0,441732347 | 1 | 0 | 0 |
| 1/11 | 0,441732347 | 1 | 0 | 0 |
| 1/11 | 0,441732347 | 1 | 0 | 0 |
| 1/11 | 0,441732347 | 1 | 0 | 0 |
| 1/11 | 0,441732347 | 1 | 0 | 0 |
| 1/11 | 0,441732347 | 1 | 0 | 0 |
| 1/11 | 0,441732347 | 1 | 0 | 0 |
| 1/11 | 0,441732347 | 1 | 0 | 0 |
| 1/11 | 0,441732347 | 1 | 0 | 0 |
| 1/11 | 0,441732347 | 1 | 0 | 0 |
| 1/11 | 0,441732347 | 1 | 0 | 0 |
| 1/11 | 0,441732347 | 1 | 0 | 0 |
| 1/11 | 0,441732347 | 1 | 0 | 0 |
| 1/11 | 0,441732347 | 1 | 0 | 0 |
| 8/141 | 0,443460326 | 1 | 0 | 0 |
| 8/141 | 0,443460326 | 1 | 0 | 0 |
| 9/160 | 0,444177789 | 1 | 0 | 0 |
| 4/66 | 0,44532807 | 1 | 0 | 0 |
| 2/29 | 0,445476512 | 1 | 0 | 0 |
| 2/29 | 0,445476512 | 1 | 0 | 0 |
| 2/29 | 0,445476512 | 1 | 0 | 0 |
| 2/29 | 0,445476512 | 1 | 0 | 0 |
| 2/29 | 0,445476512 | 1 | 0 | 0 |
| 2/29 | 0,445476512 | 1 | 0 | 0 |

| | | | | |
|---|---|---|---|---|
| 2/29 | 0,445476512 | 1 | 0 | 0 |
| 2/29 | 0,445476512 | 1 | 0 | 0 |
| 2/29 | 0,445476512 | 1 | 0 | 0 |
| 2/29 | 0,445476512 | 1 | 0 | 0 |
| 5/85 | 0,448216045 | 1 | 0 | 0 |
| 5/85 | 0,448216045 | 1 | 0 | 0 |
| 9/161 | 0,451566513 | 1 | 0 | 0 |
| 3/48 | 0,453599363 | 1 | 0 | 0 |
| 3/48 | 0,453599363 | 1 | 0 | 0 |
| 4/67 | 0,456871178 | 1 | 0 | 0 |
| 10/181 | 0,458580152 | 1 | 0 | 0 |
| 17/315 | 0,460034517 | 1 | 0 | 0 |
| 15/277 | 0,461595267 | 1 | 0 | 0 |
| 2/30 | 0,463008482 | 1 | 0 | 0 |
| 2/30 | 0,463008482 | 1 | 0 | 0 |
| 2/30 | 0,463008482 | 1 | 0 | 0 |
| 2/30 | 0,463008482 | 1 | 0 | 0 |
| 2/30 | 0,463008482 | 1 | 0 | 0 |
| 2/30 | 0,463008482 | 1 | 0 | 0 |
| 2/30 | 0,463008482 | 1 | 0 | 0 |
| 2/30 | 0,463008482 | 1 | 0 | 0 |
| 2/30 | 0,463008482 | 1 | 0 | 0 |
| 2/30 | 0,463008482 | 1 | 0 | 0 |
| 2/30 | 0,463008482 | 1 | 0 | 0 |
| 2/30 | 0,463008482 | 1 | 0 | 0 |
| 2/30 | 0,463008482 | 1 | 0 | 0 |
| 2/30 | 0,463008482 | 1 | 0 | 0 |
| 2/30 | 0,463008482 | 1 | 0 | 0 |
| 2/30 | 0,463008482 | 1 | 0 | 0 |
| 12/220 | 0,463971728 | 1 | 0 | 0 |
| 3/49 | 0,467165176 | 1 | 0 | 0 |
| 3/49 | 0,467165176 | 1 | 0 | 0 |
| 3/49 | 0,467165176 | 1 | 0 | 0 |
| 12/221 | 0,470290482 | 1 | 0 | 0 |
| 1/12 | 0,470554753 | 1 | 0 | 0 |
| 1/12 | 0,470554753 | 1 | 0 | 0 |
| 1/12 | 0,470554753 | 1 | 0 | 0 |
| 1/12 | 0,470554753 | 1 | 0 | 0 |
| 1/12 | 0,470554753 | 1 | 0 | 0 |
| 1/12 | 0,470554753 | 1 | 0 | 0 |
| 1/12 | 0,470554753 | 1 | 0 | 0 |
| 1/12 | 0,470554753 | 1 | 0 | 0 |
| 1/12 | 0,470554753 | 1 | 0 | 0 |
| 1/12 | 0,470554753 | 1 | 0 | 0 |
| 1/12 | 0,470554753 | 1 | 0 | 0 |
| 1/12 | 0,470554753 | 1 | 0 | 0 |
| 1/12 | 0,470554753 | 1 | 0 | 0 |
| 1/12 | 0,470554753 | 1 | 0 | 0 |
| 1/12 | 0,470554753 | 1 | 0 | 0 |
| 1/12 | 0,470554753 | 1 | 0 | 0 |
| 1/12 | 0,470554753 | 1 | 0 | 0 |
| 1/12 | 0,470554753 | 1 | 0 | 0 |
| 1/12 | 0,470554753 | 1 | 0 | 0 |

| | | | | |
|---|---|---|---|---|
| 1/12 | 0,470554753 | 1 | 0 | 0 |
| 1/12 | 0,470554753 | 1 | 0 | 0 |
| 1/12 | 0,470554753 | 1 | 0 | 0 |
| 1/12 | 0,470554753 | 1 | 0 | 0 |
| 1/12 | 0,470554753 | 1 | 0 | 0 |
| 1/12 | 0,470554753 | 1 | 0 | 0 |
| 1/12 | 0,470554753 | 1 | 0 | 0 |
| 1/12 | 0,470554753 | 1 | 0 | 0 |
| 1/12 | 0,470554753 | 1 | 0 | 0 |
| 1/12 | 0,470554753 | 1 | 0 | 0 |
| 1/12 | 0,470554753 | 1 | 0 | 0 |
| 1/12 | 0,470554753 | 1 | 0 | 0 |
| 1/12 | 0,470554753 | 1 | 0 | 0 |
| 1/12 | 0,470554753 | 1 | 0 | 0 |
| 1/12 | 0,470554753 | 1 | 0 | 0 |
| 1/12 | 0,470554753 | 1 | 0 | 0 |
| 1/12 | 0,470554753 | 1 | 0 | 0 |
| 1/12 | 0,470554753 | 1 | 0 | 0 |
| 1/12 | 0,470554753 | 1 | 0 | 0 |
| 1/12 | 0,470554753 | 1 | 0 | 0 |
| 1/12 | 0,470554753 | 1 | 0 | 0 |
| 1/12 | 0,470554753 | 1 | 0 | 0 |
| 1/12 | 0,470554753 | 1 | 0 | 0 |
| 1/12 | 0,470554753 | 1 | 0 | 0 |
| 1/12 | 0,470554753 | 1 | 0 | 0 |
| 1/12 | 0,470554753 | 1 | 0 | 0 |
| 1/12 | 0,470554753 | 1 | 0 | 0 |
| 1/12 | 0,470554753 | 1 | 0 | 0 |
| 1/12 | 0,470554753 | 1 | 0 | 0 |
| 1/12 | 0,470554753 | 1 | 0 | 0 |
| 1/12 | 0,470554753 | 1 | 0 | 0 |
| 1/12 | 0,470554753 | 1 | 0 | 0 |
| 1/12 | 0,470554753 | 1 | 0 | 0 |
| 1/12 | 0,470554753 | 1 | 0 | 0 |
| 1/12 | 0,470554753 | 1 | 0 | 0 |
| 1/12 | 0,470554753 | 1 | 0 | 0 |
| 1/12 | 0,470554753 | 1 | 0 | 0 |
| 1/12 | 0,470554753 | 1 | 0 | 0 |
| 1/12 | 0,470554753 | 1 | 0 | 0 |
| 1/12 | 0,470554753 | 1 | 0 | 0 |
| 1/12 | 0,470554753 | 1 | 0 | 0 |
| 1/12 | 0,470554753 | 1 | 0 | 0 |
| 1/12 | 0,470554753 | 1 | 0 | 0 |
| 1/12 | 0,470554753 | 1 | 0 | 0 |
| 1/12 | 0,470554753 | 1 | 0 | 0 |
| 1/12 | 0,470554753 | 1 | 0 | 0 |
| 1/12 | 0,470554753 | 1 | 0 | 0 |
| 1/12 | 0,470554753 | 1 | 0 | 0 |
| 1/12 | 0,470554753 | 1 | 0 | 0 |
| 1/12 | 0,470554753 | 1 | 0 | 0 |
| 1/12 | 0,470554753 | 1 | 0 | 0 |
| 1/12 | 0,470554753 | 1 | 0 | 0 |
| 1/12 | 0,470554753 | 1 | 0 | 0 |

| | | | | |
|---|---|---|---|---|
| 8/145 | 0,474821665 | 1 | 0 | 0 |
| 4/69 | 0,479703714 | 1 | 0 | 0 |
| 2/31 | 0,480209569 | 1 | 0 | 0 |
| 2/31 | 0,480209569 | 1 | 0 | 0 |
| 2/31 | 0,480209569 | 1 | 0 | 0 |
| 2/31 | 0,480209569 | 1 | 0 | 0 |
| 2/31 | 0,480209569 | 1 | 0 | 0 |
| 2/31 | 0,480209569 | 1 | 0 | 0 |
| 2/31 | 0,480209569 | 1 | 0 | 0 |
| 2/31 | 0,480209569 | 1 | 0 | 0 |
| 2/31 | 0,480209569 | 1 | 0 | 0 |
| 2/31 | 0,480209569 | 1 | 0 | 0 |
| 2/31 | 0,480209569 | 1 | 0 | 0 |
| 2/31 | 0,480209569 | 1 | 0 | 0 |
| 2/31 | 0,480209569 | 1 | 0 | 0 |
| 2/31 | 0,480209569 | 1 | 0 | 0 |
| 2/31 | 0,480209569 | 1 | 0 | 0 |
| 3/50 | 0,480578809 | 1 | 0 | 0 |
| 7/127 | 0,484385463 | 1 | 0 | 0 |
| 11/204 | 0,48454569 | 1 | 0 | 0 |
| 6/108 | 0,486339746 | 1 | 0 | 0 |
| 5/89 | 0,488513638 | 1 | 0 | 0 |
| 5/89 | 0,488513638 | 1 | 0 | 0 |
| 4/70 | 0,49097898 | 1 | 0 | 0 |
| 3/51 | 0,493830654 | 1 | 0 | 0 |
| 3/51 | 0,493830654 | 1 | 0 | 0 |
| 3/51 | 0,493830654 | 1 | 0 | 0 |
| 2/32 | 0,497067219 | 1 | 0 | 0 |
| 2/32 | 0,497067219 | 1 | 0 | 0 |
| 2/32 | 0,497067219 | 1 | 0 | 0 |
| 2/32 | 0,497067219 | 1 | 0 | 0 |
| 2/32 | 0,497067219 | 1 | 0 | 0 |
| 2/32 | 0,497067219 | 1 | 0 | 0 |
| 1/13 | 0,497890471 | 1 | 0 | 0 |
| 1/13 | 0,497890471 | 1 | 0 | 0 |
| 1/13 | 0,497890471 | 1 | 0 | 0 |
| 1/13 | 0,497890471 | 1 | 0 | 0 |
| 1/13 | 0,497890471 | 1 | 0 | 0 |
| 1/13 | 0,497890471 | 1 | 0 | 0 |
| 1/13 | 0,497890471 | 1 | 0 | 0 |
| 1/13 | 0,497890471 | 1 | 0 | 0 |
| 1/13 | 0,497890471 | 1 | 0 | 0 |
| 1/13 | 0,497890471 | 1 | 0 | 0 |
| 1/13 | 0,497890471 | 1 | 0 | 0 |
| 1/13 | 0,497890471 | 1 | 0 | 0 |
| 1/13 | 0,497890471 | 1 | 0 | 0 |
| 1/13 | 0,497890471 | 1 | 0 | 0 |
| 1/13 | 0,497890471 | 1 | 0 | 0 |
| 1/13 | 0,497890471 | 1 | 0 | 0 |
| 1/13 | 0,497890471 | 1 | 0 | 0 |
| 1/13 | 0,497890471 | 1 | 0 | 0 |
| 1/13 | 0,497890471 | 1 | 0 | 0 |
| 1/13 | 0,497890471 | 1 | 0 | 0 |

| | | | | |
|---|---|---|---|---|
| 1/13 | 0,497890471 | 1 | 0 | 0 |
| 1/13 | 0,497890471 | 1 | 0 | 0 |
| 1/13 | 0,497890471 | 1 | 0 | 0 |
| 1/13 | 0,497890471 | 1 | 0 | 0 |
| 1/13 | 0,497890471 | 1 | 0 | 0 |
| 1/13 | 0,497890471 | 1 | 0 | 0 |
| 1/13 | 0,497890471 | 1 | 0 | 0 |
| 1/13 | 0,497890471 | 1 | 0 | 0 |
| 1/13 | 0,497890471 | 1 | 0 | 0 |
| 1/13 | 0,497890471 | 1 | 0 | 0 |
| 1/13 | 0,497890471 | 1 | 0 | 0 |
| 1/13 | 0,497890471 | 1 | 0 | 0 |
| 1/13 | 0,497890471 | 1 | 0 | 0 |
| 1/13 | 0,497890471 | 1 | 0 | 0 |
| 1/13 | 0,497890471 | 1 | 0 | 0 |
| 1/13 | 0,497890471 | 1 | 0 | 0 |
| 1/13 | 0,497890471 | 1 | 0 | 0 |
| 1/13 | 0,497890471 | 1 | 0 | 0 |
| 1/13 | 0,497890471 | 1 | 0 | 0 |
| 1/13 | 0,497890471 | 1 | 0 | 0 |
| 1/13 | 0,497890471 | 1 | 0 | 0 |
| 1/13 | 0,497890471 | 1 | 0 | 0 |
| 1/13 | 0,497890471 | 1 | 0 | 0 |
| 1/13 | 0,497890471 | 1 | 0 | 0 |
| 1/13 | 0,497890471 | 1 | 0 | 0 |
| 1/13 | 0,497890471 | 1 | 0 | 0 |
| 1/13 | 0,497890471 | 1 | 0 | 0 |
| 1/13 | 0,497890471 | 1 | 0 | 0 |
| 1/13 | 0,497890471 | 1 | 0 | 0 |
| 1/13 | 0,497890471 | 1 | 0 | 0 |
| 1/13 | 0,497890471 | 1 | 0 | 0 |
| 1/13 | 0,497890471 | 1 | 0 | 0 |
| 1/13 | 0,497890471 | 1 | 0 | 0 |
| 1/13 | 0,497890471 | 1 | 0 | 0 |
| 1/13 | 0,497890471 | 1 | 0 | 0 |
| 4/71 | 0,502151483 | 1 | 0 | 0 |
| 3/52 | 0,50691194 | 1 | 0 | 0 |
| 5/91 | 0,508266738 | 1 | 0 | 0 |
| 4/72 | 0,513215153 | 1 | 0 | 0 |
| 2/33 | 0,513571053 | 1 | 0 | 0 |
| 2/33 | 0,513571053 | 1 | 0 | 0 |
| 2/33 | 0,513571053 | 1 | 0 | 0 |
| 2/33 | 0,513571053 | 1 | 0 | 0 |
| 2/33 | 0,513571053 | 1 | 0 | 0 |
| 2/33 | 0,513571053 | 1 | 0 | 0 |
| 2/33 | 0,513571053 | 1 | 0 | 0 |
| 2/33 | 0,513571053 | 1 | 0 | 0 |
| 2/33 | 0,513571053 | 1 | 0 | 0 |
| 13/248 | 0,517280285 | 1 | 0 | 0 |
| 7/131 | 0,51735239 | 1 | 0 | 0 |
| 5/92 | 0,518027749 | 1 | 0 | 0 |
| 5/92 | 0,518027749 | 1 | 0 | 0 |
| 5/92 | 0,518027749 | 1 | 0 | 0 |

| | | | | |
|---|---|---|---|---|
| 5/92 | 0,518027749 | 1 | 0 | 0 |
| 3/53 | 0,5198147 | 1 | 0 | 0 |
| 3/53 | 0,5198147 | 1 | 0 | 0 |
| 3/53 | 0,5198147 | 1 | 0 | 0 |
| 3/53 | 0,5198147 | 1 | 0 | 0 |
| 1/14 | 0,523816115 | 1 | 0 | 0 |
| 1/14 | 0,523816115 | 1 | 0 | 0 |
| 1/14 | 0,523816115 | 1 | 0 | 0 |
| 1/14 | 0,523816115 | 1 | 0 | 0 |
| 1/14 | 0,523816115 | 1 | 0 | 0 |
| 1/14 | 0,523816115 | 1 | 0 | 0 |
| 1/14 | 0,523816115 | 1 | 0 | 0 |
| 1/14 | 0,523816115 | 1 | 0 | 0 |
| 1/14 | 0,523816115 | 1 | 0 | 0 |
| 1/14 | 0,523816115 | 1 | 0 | 0 |
| 1/14 | 0,523816115 | 1 | 0 | 0 |
| 1/14 | 0,523816115 | 1 | 0 | 0 |
| 1/14 | 0,523816115 | 1 | 0 | 0 |
| 1/14 | 0,523816115 | 1 | 0 | 0 |
| 1/14 | 0,523816115 | 1 | 0 | 0 |
| 1/14 | 0,523816115 | 1 | 0 | 0 |
| 1/14 | 0,523816115 | 1 | 0 | 0 |
| 1/14 | 0,523816115 | 1 | 0 | 0 |
| 1/14 | 0,523816115 | 1 | 0 | 0 |
| 1/14 | 0,523816115 | 1 | 0 | 0 |
| 1/14 | 0,523816115 | 1 | 0 | 0 |
| 1/14 | 0,523816115 | 1 | 0 | 0 |
| 1/14 | 0,523816115 | 1 | 0 | 0 |
| 1/14 | 0,523816115 | 1 | 0 | 0 |
| 1/14 | 0,523816115 | 1 | 0 | 0 |
| 1/14 | 0,523816115 | 1 | 0 | 0 |
| 1/14 | 0,523816115 | 1 | 0 | 0 |
| 1/14 | 0,523816115 | 1 | 0 | 0 |
| 1/14 | 0,523816115 | 1 | 0 | 0 |
| 1/14 | 0,523816115 | 1 | 0 | 0 |
| 1/14 | 0,523816115 | 1 | 0 | 0 |
| 1/14 | 0,523816115 | 1 | 0 | 0 |
| 1/14 | 0,523816115 | 1 | 0 | 0 |
| 1/14 | 0,523816115 | 1 | 0 | 0 |
| 1/14 | 0,523816115 | 1 | 0 | 0 |
| 1/14 | 0,523816115 | 1 | 0 | 0 |
| 1/14 | 0,523816115 | 1 | 0 | 0 |
| 1/14 | 0,523816115 | 1 | 0 | 0 |
| 1/14 | 0,523816115 | 1 | 0 | 0 |
| 1/14 | 0,523816115 | 1 | 0 | 0 |
| 1/14 | 0,523816115 | 1 | 0 | 0 |
| 1/14 | 0,523816115 | 1 | 0 | 0 |
| 4/73 | 0,52416431 | 1 | 0 | 0 |
| 4/73 | 0,52416431 | 1 | 0 | 0 |
| 4/73 | 0,52416431 | 1 | 0 | 0 |
| 12/230 | 0,526449282 | 1 | 0 | 0 |
| 2/34 | 0,529712675 | 1 | 0 | 0 |
| 2/34 | 0,529712675 | 1 | 0 | 0 |
| 2/34 | 0,529712675 | 1 | 0 | 0 |

| | | | | |
|---|---|---|---|---|
| 2/34 | 0,529712675 | 1 | 0 | 0 |
| 2/34 | 0,529712675 | 1 | 0 | 0 |
| 2/34 | 0,529712675 | 1 | 0 | 0 |
| 2/34 | 0,529712675 | 1 | 0 | 0 |
| 2/34 | 0,529712675 | 1 | 0 | 0 |
| 2/34 | 0,529712675 | 1 | 0 | 0 |
| 2/34 | 0,529712675 | 1 | 0 | 0 |
| 2/34 | 0,529712675 | 1 | 0 | 0 |
| 6/113 | 0,530783321 | 1 | 0 | 0 |
| 3/54 | 0,532531736 | 1 | 0 | 0 |
| 4/74 | 0,53499366 | 1 | 0 | 0 |
| 5/94 | 0,537297506 | 1 | 0 | 0 |
| 6/114 | 0,539482998 | 1 | 0 | 0 |
| 7/134 | 0,541567612 | 1 | 0 | 0 |
| 3/55 | 0,545056593 | 1 | 0 | 0 |
| 3/55 | 0,545056593 | 1 | 0 | 0 |
| 3/55 | 0,545056593 | 1 | 0 | 0 |
| 2/35 | 0,545485494 | 1 | 0 | 0 |
| 2/35 | 0,545485494 | 1 | 0 | 0 |
| 2/35 | 0,545485494 | 1 | 0 | 0 |
| 2/35 | 0,545485494 | 1 | 0 | 0 |
| 2/35 | 0,545485494 | 1 | 0 | 0 |
| 2/35 | 0,545485494 | 1 | 0 | 0 |
| 2/35 | 0,545485494 | 1 | 0 | 0 |
| 2/35 | 0,545485494 | 1 | 0 | 0 |
| 4/75 | 0,545698286 | 1 | 0 | 0 |
| 5/95 | 0,546798449 | 1 | 0 | 0 |
| 5/95 | 0,546798449 | 1 | 0 | 0 |
| 1/15 | 0,548404355 | 1 | 0 | 0 |
| 1/15 | 0,548404355 | 1 | 0 | 0 |
| 1/15 | 0,548404355 | 1 | 0 | 0 |
| 1/15 | 0,548404355 | 1 | 0 | 0 |
| 1/15 | 0,548404355 | 1 | 0 | 0 |
| 1/15 | 0,548404355 | 1 | 0 | 0 |
| 1/15 | 0,548404355 | 1 | 0 | 0 |
| 1/15 | 0,548404355 | 1 | 0 | 0 |
| 1/15 | 0,548404355 | 1 | 0 | 0 |
| 1/15 | 0,548404355 | 1 | 0 | 0 |
| 1/15 | 0,548404355 | 1 | 0 | 0 |
| 1/15 | 0,548404355 | 1 | 0 | 0 |
| 1/15 | 0,548404355 | 1 | 0 | 0 |
| 1/15 | 0,548404355 | 1 | 0 | 0 |
| 1/15 | 0,548404355 | 1 | 0 | 0 |
| 1/15 | 0,548404355 | 1 | 0 | 0 |
| 1/15 | 0,548404355 | 1 | 0 | 0 |
| 1/15 | 0,548404355 | 1 | 0 | 0 |
| 1/15 | 0,548404355 | 1 | 0 | 0 |
| 1/15 | 0,548404355 | 1 | 0 | 0 |
| 1/15 | 0,548404355 | 1 | 0 | 0 |
| 1/15 | 0,548404355 | 1 | 0 | 0 |
| 1/15 | 0,548404355 | 1 | 0 | 0 |
| 1/15 | 0,548404355 | 1 | 0 | 0 |
| 1/15 | 0,548404355 | 1 | 0 | 0 |

| | | | | |
|---|---|---|---|---|
| 1/15 | 0,548404355 | 1 | 0 | 0 |
| 1/15 | 0,548404355 | 1 | 0 | 0 |
| 1/15 | 0,548404355 | 1 | 0 | 0 |
| 1/15 | 0,548404355 | 1 | 0 | 0 |
| 1/15 | 0,548404355 | 1 | 0 | 0 |
| 1/15 | 0,548404355 | 1 | 0 | 0 |
| 1/15 | 0,548404355 | 1 | 0 | 0 |
| 1/15 | 0,548404355 | 1 | 0 | 0 |
| 1/15 | 0,548404355 | 1 | 0 | 0 |
| 1/15 | 0,548404355 | 1 | 0 | 0 |
| 1/15 | 0,548404355 | 1 | 0 | 0 |
| 1/15 | 0,548404355 | 1 | 0 | 0 |
| 1/15 | 0,548404355 | 1 | 0 | 0 |
| 1/15 | 0,548404355 | 1 | 0 | 0 |
| 1/15 | 0,548404355 | 1 | 0 | 0 |
| 1/15 | 0,548404355 | 1 | 0 | 0 |
| 1/15 | 0,548404355 | 1 | 0 | 0 |
| 1/15 | 0,548404355 | 1 | 0 | 0 |
| 1/15 | 0,548404355 | 1 | 0 | 0 |
| 1/15 | 0,548404355 | 1 | 0 | 0 |
| 1/15 | 0,548404355 | 1 | 0 | 0 |
| 1/15 | 0,548404355 | 1 | 0 | 0 |
| 1/15 | 0,548404355 | 1 | 0 | 0 |
| 1/15 | 0,548404355 | 1 | 0 | 0 |
| 1/15 | 0,548404355 | 1 | 0 | 0 |
| 1/15 | 0,548404355 | 1 | 0 | 0 |
| 1/15 | 0,548404355 | 1 | 0 | 0 |
| 1/15 | 0,548404355 | 1 | 0 | 0 |
| 1/15 | 0,548404355 | 1 | 0 | 0 |
| 1/15 | 0,548404355 | 1 | 0 | 0 |
| 1/15 | 0,548404355 | 1 | 0 | 0 |
| 1/15 | 0,548404355 | 1 | 0 | 0 |
| 1/15 | 0,548404355 | 1 | 0 | 0 |
| 1/15 | 0,548404355 | 1 | 0 | 0 |
| 1/15 | 0,548404355 | 1 | 0 | 0 |
| 1/15 | 0,548404355 | 1 | 0 | 0 |
| 1/15 | 0,548404355 | 1 | 0 | 0 |
| 1/15 | 0,548404355 | 1 | 0 | 0 |
| 1/15 | 0,548404355 | 1 | 0 | 0 |
| 1/15 | 0,548404355 | 1 | 0 | 0 |
| 1/15 | 0,548404355 | 1 | 0 | 0 |
| 1/15 | 0,548404355 | 1 | 0 | 0 |
| 1/15 | 0,548404355 | 1 | 0 | 0 |
| 1/15 | 0,548404355 | 1 | 0 | 0 |
| 1/15 | 0,548404355 | 1 | 0 | 0 |
| 1/15 | 0,548404355 | 1 | 0 | 0 |
| 7/135 | 0,54952818 | 1 | 0 | 0 |
| 6/116 | 0,556668364 | 1 | 0 | 0 |
| 6/116 | 0,556668364 | 1 | 0 | 0 |
| 6/116 | 0,556668364 | 1 | 0 | 0 |
| 3/56 | 0,557383521 | 1 | 0 | 0 |
| 3/56 | 0,557383521 | 1 | 0 | 0 |
| 7/136 | 0,55742915 | 1 | 0 | 0 |

| | | | | |
|---|---|---|---|---|
| 2/36 | 0,560884561 | 1 | 0 | 0 |
| 2/36 | 0,560884561 | 1 | 0 | 0 |
| 2/36 | 0,560884561 | 1 | 0 | 0 |
| 2/36 | 0,560884561 | 1 | 0 | 0 |
| 2/36 | 0,560884561 | 1 | 0 | 0 |
| 2/36 | 0,560884561 | 1 | 0 | 0 |
| 2/36 | 0,560884561 | 1 | 0 | 0 |
| 2/36 | 0,560884561 | 1 | 0 | 0 |
| 2/36 | 0,560884561 | 1 | 0 | 0 |
| 2/36 | 0,560884561 | 1 | 0 | 0 |
| 2/36 | 0,560884561 | 1 | 0 | 0 |
| 6/117 | 0,565148461 | 1 | 0 | 0 |
| 6/117 | 0,565148461 | 1 | 0 | 0 |
| 5/97 | 0,565514604 | 1 | 0 | 0 |
| 3/57 | 0,569507444 | 1 | 0 | 0 |
| 3/57 | 0,569507444 | 1 | 0 | 0 |
| 3/57 | 0,569507444 | 1 | 0 | 0 |
| 3/57 | 0,569507444 | 1 | 0 | 0 |
| 3/57 | 0,569507444 | 1 | 0 | 0 |
| 1/16 | 0,571724118 | 1 | 0 | 0 |
| 1/16 | 0,571724118 | 1 | 0 | 0 |
| 1/16 | 0,571724118 | 1 | 0 | 0 |
| 1/16 | 0,571724118 | 1 | 0 | 0 |
| 1/16 | 0,571724118 | 1 | 0 | 0 |
| 1/16 | 0,571724118 | 1 | 0 | 0 |
| 1/16 | 0,571724118 | 1 | 0 | 0 |
| 1/16 | 0,571724118 | 1 | 0 | 0 |
| 1/16 | 0,571724118 | 1 | 0 | 0 |
| 1/16 | 0,571724118 | 1 | 0 | 0 |
| 1/16 | 0,571724118 | 1 | 0 | 0 |
| 1/16 | 0,571724118 | 1 | 0 | 0 |
| 1/16 | 0,571724118 | 1 | 0 | 0 |
| 1/16 | 0,571724118 | 1 | 0 | 0 |
| 1/16 | 0,571724118 | 1 | 0 | 0 |
| 1/16 | 0,571724118 | 1 | 0 | 0 |
| 1/16 | 0,571724118 | 1 | 0 | 0 |
| 1/16 | 0,571724118 | 1 | 0 | 0 |
| 1/16 | 0,571724118 | 1 | 0 | 0 |
| 1/16 | 0,571724118 | 1 | 0 | 0 |
| 1/16 | 0,571724118 | 1 | 0 | 0 |
| 1/16 | 0,571724118 | 1 | 0 | 0 |
| 1/16 | 0,571724118 | 1 | 0 | 0 |
| 1/16 | 0,571724118 | 1 | 0 | 0 |
| 1/16 | 0,571724118 | 1 | 0 | 0 |
| 1/16 | 0,571724118 | 1 | 0 | 0 |
| 1/16 | 0,571724118 | 1 | 0 | 0 |
| 1/16 | 0,571724118 | 1 | 0 | 0 |
| 1/16 | 0,571724118 | 1 | 0 | 0 |
| 1/16 | 0,571724118 | 1 | 0 | 0 |
| 1/16 | 0,571724118 | 1 | 0 | 0 |
| 1/16 | 0,571724118 | 1 | 0 | 0 |
| 1/16 | 0,571724118 | 1 | 0 | 0 |
| 1/16 | 0,571724118 | 1 | 0 | 0 |

| | | | | |
|---|---|---|---|---|
| 1/16 | 0,571724118 | 1 | 0 | 0 |
| 1/16 | 0,571724118 | 1 | 0 | 0 |
| 7/138 | 0,573043372 | 1 | 0 | 0 |
| 5/98 | 0,574723317 | 1 | 0 | 0 |
| 5/98 | 0,574723317 | 1 | 0 | 0 |
| 2/37 | 0,57590642 | 1 | 0 | 0 |
| 2/37 | 0,57590642 | 1 | 0 | 0 |
| 2/37 | 0,57590642 | 1 | 0 | 0 |
| 2/37 | 0,57590642 | 1 | 0 | 0 |
| 2/37 | 0,57590642 | 1 | 0 | 0 |
| 2/37 | 0,57590642 | 1 | 0 | 0 |
| 2/37 | 0,57590642 | 1 | 0 | 0 |
| 2/37 | 0,57590642 | 1 | 0 | 0 |
| 2/37 | 0,57590642 | 1 | 0 | 0 |
| 2/37 | 0,57590642 | 1 | 0 | 0 |
| 2/37 | 0,57590642 | 1 | 0 | 0 |
| 4/78 | 0,577020093 | 1 | 0 | 0 |
| 4/78 | 0,577020093 | 1 | 0 | 0 |
| 23/457 | 0,580034647 | 1 | 0 | 0 |
| 7/139 | 0,580752411 | 1 | 0 | 0 |
| 3/58 | 0,581423931 | 1 | 0 | 0 |
| 3/58 | 0,581423931 | 1 | 0 | 0 |
| 3/58 | 0,581423931 | 1 | 0 | 0 |
| 9/180 | 0,58672537 | 1 | 0 | 0 |
| 4/79 | 0,587183851 | 1 | 0 | 0 |
| 6/120 | 0,590107827 | 1 | 0 | 0 |
| 2/38 | 0,590548963 | 1 | 0 | 0 |
| 2/38 | 0,590548963 | 1 | 0 | 0 |
| 2/38 | 0,590548963 | 1 | 0 | 0 |
| 2/38 | 0,590548963 | 1 | 0 | 0 |
| 2/38 | 0,590548963 | 1 | 0 | 0 |
| 12/241 | 0,592340436 | 1 | 0 | 0 |
| 11/221 | 0,592467221 | 1 | 0 | 0 |
| 3/59 | 0,593129159 | 1 | 0 | 0 |
| 3/59 | 0,593129159 | 1 | 0 | 0 |
| 3/59 | 0,593129159 | 1 | 0 | 0 |
| 3/59 | 0,593129159 | 1 | 0 | 0 |
| 24/480 | 0,593580251 | 1 | 0 | 0 |
| 1/17 | 0,593840785 | 1 | 0 | 0 |
| 1/17 | 0,593840785 | 1 | 0 | 0 |
| 1/17 | 0,593840785 | 1 | 0 | 0 |
| 1/17 | 0,593840785 | 1 | 0 | 0 |
| 1/17 | 0,593840785 | 1 | 0 | 0 |
| 1/17 | 0,593840785 | 1 | 0 | 0 |
| 1/17 | 0,593840785 | 1 | 0 | 0 |
| 1/17 | 0,593840785 | 1 | 0 | 0 |
| 1/17 | 0,593840785 | 1 | 0 | 0 |
| 1/17 | 0,593840785 | 1 | 0 | 0 |
| 1/17 | 0,593840785 | 1 | 0 | 0 |
| 1/17 | 0,593840785 | 1 | 0 | 0 |
| 1/17 | 0,593840785 | 1 | 0 | 0 |
| 1/17 | 0,593840785 | 1 | 0 | 0 |
| 1/17 | 0,593840785 | 1 | 0 | 0 |

| | | | | |
|---|---|---|---|---|
| 1/17 | 0,593840785 | 1 | 0 | 0 |
| 1/17 | 0,593840785 | 1 | 0 | 0 |
| 1/17 | 0,593840785 | 1 | 0 | 0 |
| 1/17 | 0,593840785 | 1 | 0 | 0 |
| 1/17 | 0,593840785 | 1 | 0 | 0 |
| 1/17 | 0,593840785 | 1 | 0 | 0 |
| 1/17 | 0,593840785 | 1 | 0 | 0 |
| 1/17 | 0,593840785 | 1 | 0 | 0 |
| 1/17 | 0,593840785 | 1 | 0 | 0 |
| 1/17 | 0,593840785 | 1 | 0 | 0 |
| 1/17 | 0,593840785 | 1 | 0 | 0 |
| 1/17 | 0,593840785 | 1 | 0 | 0 |
| 1/17 | 0,593840785 | 1 | 0 | 0 |
| 1/17 | 0,593840785 | 1 | 0 | 0 |
| 1/17 | 0,593840785 | 1 | 0 | 0 |
| 1/17 | 0,593840785 | 1 | 0 | 0 |
| 1/17 | 0,593840785 | 1 | 0 | 0 |
| 1/17 | 0,593840785 | 1 | 0 | 0 |
| 1/17 | 0,593840785 | 1 | 0 | 0 |
| 1/17 | 0,593840785 | 1 | 0 | 0 |
| 1/17 | 0,593840785 | 1 | 0 | 0 |
| 1/17 | 0,593840785 | 1 | 0 | 0 |
| 1/17 | 0,593840785 | 1 | 0 | 0 |
| 1/17 | 0,593840785 | 1 | 0 | 0 |
| 7/141 | 0,595964551 | 1 | 0 | 0 |
| 12/242 | 0,598131919 | 1 | 0 | 0 |
| 5/101 | 0,601717403 | 1 | 0 | 0 |
| 2/39 | 0,604811304 | 1 | 0 | 0 |
| 6/122 | 0,606323923 | 1 | 0 | 0 |
| 5/102 | 0,61049632 | 1 | 0 | 0 |
| 1/18 | 0,614816365 | 1 | 0 | 0 |
| 1/18 | 0,614816365 | 1 | 0 | 0 |
| 1/18 | 0,614816365 | 1 | 0 | 0 |
| 1/18 | 0,614816365 | 1 | 0 | 0 |
| 1/18 | 0,614816365 | 1 | 0 | 0 |
| 1/18 | 0,614816365 | 1 | 0 | 0 |
| 1/18 | 0,614816365 | 1 | 0 | 0 |
| 1/18 | 0,614816365 | 1 | 0 | 0 |
| 1/18 | 0,614816365 | 1 | 0 | 0 |
| 1/18 | 0,614816365 | 1 | 0 | 0 |
| 1/18 | 0,614816365 | 1 | 0 | 0 |
| 1/18 | 0,614816365 | 1 | 0 | 0 |
| 1/18 | 0,614816365 | 1 | 0 | 0 |
| 1/18 | 0,614816365 | 1 | 0 | 0 |
| 1/18 | 0,614816365 | 1 | 0 | 0 |
| 1/18 | 0,614816365 | 1 | 0 | 0 |
| 1/18 | 0,614816365 | 1 | 0 | 0 |
| 1/18 | 0,614816365 | 1 | 0 | 0 |
| 1/18 | 0,614816365 | 1 | 0 | 0 |
| 1/18 | 0,614816365 | 1 | 0 | 0 |
| 1/18 | 0,614816365 | 1 | 0 | 0 |
| 1/18 | 0,614816365 | 1 | 0 | 0 |
| 1/18 | 0,614816365 | 1 | 0 | 0 |

| | | | | |
|---|---|---|---|---|
| 1/18 | 0,614816365 | 1 | 0 | 0 |
| 1/18 | 0,614816365 | 1 | 0 | 0 |
| 1/18 | 0,614816365 | 1 | 0 | 0 |
| 3/61 | 0,615893424 | 1 | 0 | 0 |
| 3/61 | 0,615893424 | 1 | 0 | 0 |
| 3/61 | 0,615893424 | 1 | 0 | 0 |
| 2/40 | 0,618693656 | 1 | 0 | 0 |
| 2/40 | 0,618693656 | 1 | 0 | 0 |
| 2/40 | 0,618693656 | 1 | 0 | 0 |
| 2/40 | 0,618693656 | 1 | 0 | 0 |
| 2/40 | 0,618693656 | 1 | 0 | 0 |
| 2/40 | 0,618693656 | 1 | 0 | 0 |
| 2/40 | 0,618693656 | 1 | 0 | 0 |
| 2/40 | 0,618693656 | 1 | 0 | 0 |
| 8/165 | 0,622211052 | 1 | 0 | 0 |
| 4/83 | 0,626371905 | 1 | 0 | 0 |
| 6/125 | 0,629973485 | 1 | 0 | 0 |
| 6/125 | 0,629973485 | 1 | 0 | 0 |
| 2/41 | 0,632197223 | 1 | 0 | 0 |
| 2/41 | 0,632197223 | 1 | 0 | 0 |
| 2/41 | 0,632197223 | 1 | 0 | 0 |
| 2/41 | 0,632197223 | 1 | 0 | 0 |
| 1/19 | 0,63470968 | 1 | 0 | 0 |
| 1/19 | 0,63470968 | 1 | 0 | 0 |
| 1/19 | 0,63470968 | 1 | 0 | 0 |
| 1/19 | 0,63470968 | 1 | 0 | 0 |
| 1/19 | 0,63470968 | 1 | 0 | 0 |
| 1/19 | 0,63470968 | 1 | 0 | 0 |
| 1/19 | 0,63470968 | 1 | 0 | 0 |
| 1/19 | 0,63470968 | 1 | 0 | 0 |
| 1/19 | 0,63470968 | 1 | 0 | 0 |
| 1/19 | 0,63470968 | 1 | 0 | 0 |
| 1/19 | 0,63470968 | 1 | 0 | 0 |
| 1/19 | 0,63470968 | 1 | 0 | 0 |
| 1/19 | 0,63470968 | 1 | 0 | 0 |
| 1/19 | 0,63470968 | 1 | 0 | 0 |
| 1/19 | 0,63470968 | 1 | 0 | 0 |
| 1/19 | 0,63470968 | 1 | 0 | 0 |
| 1/19 | 0,63470968 | 1 | 0 | 0 |
| 1/19 | 0,63470968 | 1 | 0 | 0 |
| 1/19 | 0,63470968 | 1 | 0 | 0 |
| 1/19 | 0,63470968 | 1 | 0 | 0 |
| 1/19 | 0,63470968 | 1 | 0 | 0 |
| 1/19 | 0,63470968 | 1 | 0 | 0 |
| 1/19 | 0,63470968 | 1 | 0 | 0 |
| 1/19 | 0,63470968 | 1 | 0 | 0 |
| 1/19 | 0,63470968 | 1 | 0 | 0 |
| 1/19 | 0,63470968 | 1 | 0 | 0 |
| 1/19 | 0,63470968 | 1 | 0 | 0 |
| 1/19 | 0,63470968 | 1 | 0 | 0 |
| 1/19 | 0,63470968 | 1 | 0 | 0 |
| 1/19 | 0,63470968 | 1 | 0 | 0 |
| 1/19 | 0,63470968 | 1 | 0 | 0 |
| 1/19 | 0,63470968 | 1 | 0 | 0 |

| | | | | |
|---|---|---|---|---|
| 1/19 | 0,63470968 | 1 | 0 | 0 |
| 1/19 | 0,63470968 | 1 | 0 | 0 |
| 1/19 | 0,63470968 | 1 | 0 | 0 |
| 1/19 | 0,63470968 | 1 | 0 | 0 |
| 4/84 | 0,635790137 | 1 | 0 | 0 |
| 5/105 | 0,636145778 | 1 | 0 | 0 |
| 5/105 | 0,636145778 | 1 | 0 | 0 |
| 3/63 | 0,637780712 | 1 | 0 | 0 |
| 3/63 | 0,637780712 | 1 | 0 | 0 |
| 3/63 | 0,637780712 | 1 | 0 | 0 |
| 3/63 | 0,637780712 | 1 | 0 | 0 |
| 3/63 | 0,637780712 | 1 | 0 | 0 |
| 10/209 | 0,642038163 | 1 | 0 | 0 |
| 5/106 | 0,644460603 | 1 | 0 | 0 |
| 4/85 | 0,645053048 | 1 | 0 | 0 |
| 2/42 | 0,645324095 | 1 | 0 | 0 |
| 2/42 | 0,645324095 | 1 | 0 | 0 |
| 2/42 | 0,645324095 | 1 | 0 | 0 |
| 2/42 | 0,645324095 | 1 | 0 | 0 |
| 2/42 | 0,645324095 | 1 | 0 | 0 |
| 2/42 | 0,645324095 | 1 | 0 | 0 |
| 3/64 | 0,648391557 | 1 | 0 | 0 |
| 3/64 | 0,648391557 | 1 | 0 | 0 |
| 1/20 | 0,653576518 | 1 | 0 | 0 |
| 1/20 | 0,653576518 | 1 | 0 | 0 |
| 1/20 | 0,653576518 | 1 | 0 | 0 |
| 1/20 | 0,653576518 | 1 | 0 | 0 |
| 1/20 | 0,653576518 | 1 | 0 | 0 |
| 1/20 | 0,653576518 | 1 | 0 | 0 |
| 1/20 | 0,653576518 | 1 | 0 | 0 |
| 1/20 | 0,653576518 | 1 | 0 | 0 |
| 1/20 | 0,653576518 | 1 | 0 | 0 |
| 1/20 | 0,653576518 | 1 | 0 | 0 |
| 1/20 | 0,653576518 | 1 | 0 | 0 |
| 1/20 | 0,653576518 | 1 | 0 | 0 |
| 1/20 | 0,653576518 | 1 | 0 | 0 |
| 1/20 | 0,653576518 | 1 | 0 | 0 |
| 1/20 | 0,653576518 | 1 | 0 | 0 |
| 1/20 | 0,653576518 | 1 | 0 | 0 |
| 1/20 | 0,653576518 | 1 | 0 | 0 |
| 1/20 | 0,653576518 | 1 | 0 | 0 |
| 1/20 | 0,653576518 | 1 | 0 | 0 |
| 1/20 | 0,653576518 | 1 | 0 | 0 |
| 1/20 | 0,653576518 | 1 | 0 | 0 |
| 1/20 | 0,653576518 | 1 | 0 | 0 |
| 1/20 | 0,653576518 | 1 | 0 | 0 |
| 1/20 | 0,653576518 | 1 | 0 | 0 |
| 1/20 | 0,653576518 | 1 | 0 | 0 |
| 1/20 | 0,653576518 | 1 | 0 | 0 |
| 1/20 | 0,653576518 | 1 | 0 | 0 |
| 1/20 | 0,653576518 | 1 | 0 | 0 |
| 7/149 | 0,653826066 | 1 | 0 | 0 |
| 2/43 | 0,658077154 | 1 | 0 | 0 |

| | | | | |
|---|---|---|---|---|
| 2/43 | 0,658077154 | 1 | 0 | 0 |
| 2/43 | 0,658077154 | 1 | 0 | 0 |
| 3/65 | 0,65877934 | 1 | 0 | 0 |
| 6/129 | 0,660180622 | 1 | 0 | 0 |
| 5/108 | 0,660728995 | 1 | 0 | 0 |
| 7/151 | 0,667490999 | 1 | 0 | 0 |
| 5/109 | 0,668680226 | 1 | 0 | 0 |
| 5/109 | 0,668680226 | 1 | 0 | 0 |
| 9/193 | 0,669505094 | 1 | 0 | 0 |
| 2/44 | 0,670459987 | 1 | 0 | 0 |
| 2/44 | 0,670459987 | 1 | 0 | 0 |
| 2/44 | 0,670459987 | 1 | 0 | 0 |
| 2/44 | 0,670459987 | 1 | 0 | 0 |
| 2/44 | 0,670459987 | 1 | 0 | 0 |
| 2/44 | 0,670459987 | 1 | 0 | 0 |
| 1/21 | 0,671469795 | 1 | 0 | 0 |
| 1/21 | 0,671469795 | 1 | 0 | 0 |
| 1/21 | 0,671469795 | 1 | 0 | 0 |
| 1/21 | 0,671469795 | 1 | 0 | 0 |
| 1/21 | 0,671469795 | 1 | 0 | 0 |
| 1/21 | 0,671469795 | 1 | 0 | 0 |
| 1/21 | 0,671469795 | 1 | 0 | 0 |
| 1/21 | 0,671469795 | 1 | 0 | 0 |
| 1/21 | 0,671469795 | 1 | 0 | 0 |
| 1/21 | 0,671469795 | 1 | 0 | 0 |
| 1/21 | 0,671469795 | 1 | 0 | 0 |
| 1/21 | 0,671469795 | 1 | 0 | 0 |
| 1/21 | 0,671469795 | 1 | 0 | 0 |
| 1/21 | 0,671469795 | 1 | 0 | 0 |
| 1/21 | 0,671469795 | 1 | 0 | 0 |
| 1/21 | 0,671469795 | 1 | 0 | 0 |
| 1/21 | 0,671469795 | 1 | 0 | 0 |
| 1/21 | 0,671469795 | 1 | 0 | 0 |
| 1/21 | 0,671469795 | 1 | 0 | 0 |
| 1/21 | 0,671469795 | 1 | 0 | 0 |
| 1/21 | 0,671469795 | 1 | 0 | 0 |
| 1/21 | 0,671469795 | 1 | 0 | 0 |
| 1/21 | 0,671469795 | 1 | 0 | 0 |
| 3/67 | 0,678884566 | 1 | 0 | 0 |
| 7/153 | 0,68081926 | 1 | 0 | 0 |
| 2/45 | 0,682476808 | 1 | 0 | 0 |
| 2/45 | 0,682476808 | 1 | 0 | 0 |
| 2/45 | 0,682476808 | 1 | 0 | 0 |
| 2/45 | 0,682476808 | 1 | 0 | 0 |
| 2/45 | 0,682476808 | 1 | 0 | 0 |
| 2/45 | 0,682476808 | 1 | 0 | 0 |
| 2/45 | 0,682476808 | 1 | 0 | 0 |
| 2/45 | 0,682476808 | 1 | 0 | 0 |
| 2/45 | 0,682476808 | 1 | 0 | 0 |
| 2/45 | 0,682476808 | 1 | 0 | 0 |
| 2/45 | 0,682476808 | 1 | 0 | 0 |
| 5/111 | 0,684212105 | 1 | 0 | 0 |
| 5/111 | 0,684212105 | 1 | 0 | 0 |

| | | | | |
|---|---|---|---|---|
| 1/22 | 0,688439703 | 1 | 0 | 0 |
| 1/22 | 0,688439703 | 1 | 0 | 0 |
| 1/22 | 0,688439703 | 1 | 0 | 0 |
| 1/22 | 0,688439703 | 1 | 0 | 0 |
| 1/22 | 0,688439703 | 1 | 0 | 0 |
| 1/22 | 0,688439703 | 1 | 0 | 0 |
| 1/22 | 0,688439703 | 1 | 0 | 0 |
| 1/22 | 0,688439703 | 1 | 0 | 0 |
| 1/22 | 0,688439703 | 1 | 0 | 0 |
| 1/22 | 0,688439703 | 1 | 0 | 0 |
| 1/22 | 0,688439703 | 1 | 0 | 0 |
| 1/22 | 0,688439703 | 1 | 0 | 0 |
| 1/22 | 0,688439703 | 1 | 0 | 0 |
| 1/22 | 0,688439703 | 1 | 0 | 0 |
| 1/22 | 0,688439703 | 1 | 0 | 0 |
| 1/22 | 0,688439703 | 1 | 0 | 0 |
| 1/22 | 0,688439703 | 1 | 0 | 0 |
| 1/22 | 0,688439703 | 1 | 0 | 0 |
| 1/22 | 0,688439703 | 1 | 0 | 0 |
| 1/22 | 0,688439703 | 1 | 0 | 0 |
| 1/22 | 0,688439703 | 1 | 0 | 0 |
| 1/22 | 0,688439703 | 1 | 0 | 0 |
| 1/22 | 0,688439703 | 1 | 0 | 0 |
| 1/22 | 0,688439703 | 1 | 0 | 0 |
| 3/68 | 0,688602361 | 1 | 0 | 0 |
| 3/68 | 0,688602361 | 1 | 0 | 0 |
| 3/68 | 0,688602361 | 1 | 0 | 0 |
| 2/46 | 0,694132378 | 1 | 0 | 0 |
| 2/46 | 0,694132378 | 1 | 0 | 0 |
| 2/46 | 0,694132378 | 1 | 0 | 0 |
| 2/46 | 0,694132378 | 1 | 0 | 0 |
| 2/46 | 0,694132378 | 1 | 0 | 0 |
| 4/91 | 0,697305711 | 1 | 0 | 0 |
| 3/69 | 0,698097744 | 1 | 0 | 0 |
| 3/69 | 0,698097744 | 1 | 0 | 0 |
| 3/69 | 0,698097744 | 1 | 0 | 0 |
| 1/23 | 0,704533848 | 1 | 0 | 0 |
| 1/23 | 0,704533848 | 1 | 0 | 0 |
| 1/23 | 0,704533848 | 1 | 0 | 0 |
| 1/23 | 0,704533848 | 1 | 0 | 0 |
| 1/23 | 0,704533848 | 1 | 0 | 0 |
| 1/23 | 0,704533848 | 1 | 0 | 0 |
| 1/23 | 0,704533848 | 1 | 0 | 0 |
| 1/23 | 0,704533848 | 1 | 0 | 0 |
| 1/23 | 0,704533848 | 1 | 0 | 0 |
| 1/23 | 0,704533848 | 1 | 0 | 0 |
| 1/23 | 0,704533848 | 1 | 0 | 0 |
| 1/23 | 0,704533848 | 1 | 0 | 0 |
| 1/23 | 0,704533848 | 1 | 0 | 0 |
| 1/23 | 0,704533848 | 1 | 0 | 0 |
| 1/23 | 0,704533848 | 1 | 0 | 0 |
| 1/23 | 0,704533848 | 1 | 0 | 0 |
| 1/23 | 0,704533848 | 1 | 0 | 0 |

| | | | | |
|---|---|---|---|---|
| 1/23 | 0,704533848 | 1 | 0 | 0 |
| 1/23 | 0,704533848 | 1 | 0 | 0 |
| 1/23 | 0,704533848 | 1 | 0 | 0 |
| 1/23 | 0,704533848 | 1 | 0 | 0 |
| 2/47 | 0,705431946 | 1 | 0 | 0 |
| 2/47 | 0,705431946 | 1 | 0 | 0 |
| 2/47 | 0,705431946 | 1 | 0 | 0 |
| 2/47 | 0,705431946 | 1 | 0 | 0 |
| 2/47 | 0,705431946 | 1 | 0 | 0 |
| 4/92 | 0,705454701 | 1 | 0 | 0 |
| 2/48 | 0,716381176 | 1 | 0 | 0 |
| 2/48 | 0,716381176 | 1 | 0 | 0 |
| 2/48 | 0,716381176 | 1 | 0 | 0 |
| 2/48 | 0,716381176 | 1 | 0 | 0 |
| 3/71 | 0,716425533 | 1 | 0 | 0 |
| 3/71 | 0,716425533 | 1 | 0 | 0 |
| 3/71 | 0,716425533 | 1 | 0 | 0 |
| 15/327 | 0,717092152 | 1 | 0 | 0 |
| 1/24 | 0,719797384 | 1 | 0 | 0 |
| 1/24 | 0,719797384 | 1 | 0 | 0 |
| 5/116 | 0,720853697 | 1 | 0 | 0 |
| 3/72 | 0,725260757 | 1 | 0 | 0 |
| 3/72 | 0,725260757 | 1 | 0 | 0 |
| 2/49 | 0,7269861 | 1 | 0 | 0 |
| 2/49 | 0,7269861 | 1 | 0 | 0 |
| 2/49 | 0,7269861 | 1 | 0 | 0 |
| 2/49 | 0,7269861 | 1 | 0 | 0 |
| 4/95 | 0,728941259 | 1 | 0 | 0 |
| 13/289 | 0,733369083 | 1 | 0 | 0 |
| 3/73 | 0,733879166 | 1 | 0 | 0 |
| 3/73 | 0,733879166 | 1 | 0 | 0 |
| 1/25 | 0,734273137 | 1 | 0 | 0 |
| 1/25 | 0,734273137 | 1 | 0 | 0 |
| 1/25 | 0,734273137 | 1 | 0 | 0 |
| 1/25 | 0,734273137 | 1 | 0 | 0 |
| 1/25 | 0,734273137 | 1 | 0 | 0 |
| 1/25 | 0,734273137 | 1 | 0 | 0 |
| 1/25 | 0,734273137 | 1 | 0 | 0 |
| 1/25 | 0,734273137 | 1 | 0 | 0 |
| 1/25 | 0,734273137 | 1 | 0 | 0 |
| 1/25 | 0,734273137 | 1 | 0 | 0 |
| 1/25 | 0,734273137 | 1 | 0 | 0 |
| 1/25 | 0,734273137 | 1 | 0 | 0 |
| 1/25 | 0,734273137 | 1 | 0 | 0 |
| 1/25 | 0,734273137 | 1 | 0 | 0 |
| 1/25 | 0,734273137 | 1 | 0 | 0 |
| 1/25 | 0,734273137 | 1 | 0 | 0 |
| 1/25 | 0,734273137 | 1 | 0 | 0 |
| 1/25 | 0,734273137 | 1 | 0 | 0 |
| 1/25 | 0,734273137 | 1 | 0 | 0 |
| 1/25 | 0,734273137 | 1 | 0 | 0 |
| 1/25 | 0,734273137 | 1 | 0 | 0 |
| 1/25 | 0,734273137 | 1 | 0 | 0 |

| | | | | |
|---|---|---|---|---|
| 1/25 | 0,734273137 | 1 | 0 | 0 |
| 5/118 | 0,734630243 | 1 | 0 | 0 |
| 4/96 | 0,736451512 | 1 | 0 | 0 |
| 4/96 | 0,736451512 | 1 | 0 | 0 |
| 2/50 | 0,737253058 | 1 | 0 | 0 |
| 2/50 | 0,737253058 | 1 | 0 | 0 |
| 2/50 | 0,737253058 | 1 | 0 | 0 |
| 2/50 | 0,737253058 | 1 | 0 | 0 |
| 6/141 | 0,741122664 | 1 | 0 | 0 |
| 6/141 | 0,741122664 | 1 | 0 | 0 |
| 5/119 | 0,741330221 | 1 | 0 | 0 |
| 3/74 | 0,742282769 | 1 | 0 | 0 |
| 4/97 | 0,743803628 | 1 | 0 | 0 |
| 4/97 | 0,743803628 | 1 | 0 | 0 |
| 8/185 | 0,744195448 | 1 | 0 | 0 |
| 2/51 | 0,747188653 | 1 | 0 | 0 |
| 2/51 | 0,747188653 | 1 | 0 | 0 |
| 22/480 | 0,747449801 | 1 | 0 | 0 |
| 1/26 | 0,74800173 | 1 | 0 | 0 |
| 1/26 | 0,74800173 | 1 | 0 | 0 |
| 1/26 | 0,74800173 | 1 | 0 | 0 |
| 1/26 | 0,74800173 | 1 | 0 | 0 |
| 1/26 | 0,74800173 | 1 | 0 | 0 |
| 1/26 | 0,74800173 | 1 | 0 | 0 |
| 1/26 | 0,74800173 | 1 | 0 | 0 |
| 1/26 | 0,74800173 | 1 | 0 | 0 |
| 1/26 | 0,74800173 | 1 | 0 | 0 |
| 1/26 | 0,74800173 | 1 | 0 | 0 |
| 1/26 | 0,74800173 | 1 | 0 | 0 |
| 1/26 | 0,74800173 | 1 | 0 | 0 |
| 1/26 | 0,74800173 | 1 | 0 | 0 |
| 1/26 | 0,74800173 | 1 | 0 | 0 |
| 1/26 | 0,74800173 | 1 | 0 | 0 |
| 1/26 | 0,74800173 | 1 | 0 | 0 |
| 1/26 | 0,74800173 | 1 | 0 | 0 |
| 1/26 | 0,74800173 | 1 | 0 | 0 |
| 1/26 | 0,74800173 | 1 | 0 | 0 |
| 1/26 | 0,74800173 | 1 | 0 | 0 |
| 1/26 | 0,74800173 | 1 | 0 | 0 |
| 1/26 | 0,74800173 | 1 | 0 | 0 |
| 1/26 | 0,74800173 | 1 | 0 | 0 |
| 1/26 | 0,74800173 | 1 | 0 | 0 |
| 1/26 | 0,74800173 | 1 | 0 | 0 |
| 1/26 | 0,74800173 | 1 | 0 | 0 |
| 1/26 | 0,74800173 | 1 | 0 | 0 |
| 1/26 | 0,74800173 | 1 | 0 | 0 |
| 1/26 | 0,74800173 | 1 | 0 | 0 |
| 1/26 | 0,74800173 | 1 | 0 | 0 |
| 1/26 | 0,74800173 | 1 | 0 | 0 |
| 17/377 | 0,750610745 | 1 | 0 | 0 |
| 4/98 | 0,750998527 | 1 | 0 | 0 |
| 6/143 | 0,753165416 | 1 | 0 | 0 |
| 2/52 | 0,756799709 | 1 | 0 | 0 |

| | | | | |
|---|---|---|---|---|
| 2/52 | 0,756799709 | 1 | 0 | 0 |
| 2/52 | 0,756799709 | 1 | 0 | 0 |
| 17/379 | 0,758037054 | 1 | 0 | 0 |
| 4/99 | 0,758037257 | 1 | 0 | 0 |
| 4/99 | 0,758037257 | 1 | 0 | 0 |
| 3/76 | 0,758454597 | 1 | 0 | 0 |
| 5/122 | 0,76068145 | 1 | 0 | 0 |
| 22/484 | 0,760686433 | 1 | 0 | 0 |
| 1/27 | 0,761021692 | 1 | 0 | 0 |
| 1/27 | 0,761021692 | 1 | 0 | 0 |
| 1/27 | 0,761021692 | 1 | 0 | 0 |
| 1/27 | 0,761021692 | 1 | 0 | 0 |
| 1/27 | 0,761021692 | 1 | 0 | 0 |
| 1/27 | 0,761021692 | 1 | 0 | 0 |
| 1/27 | 0,761021692 | 1 | 0 | 0 |
| 1/27 | 0,761021692 | 1 | 0 | 0 |
| 1/27 | 0,761021692 | 1 | 0 | 0 |
| 1/27 | 0,761021692 | 1 | 0 | 0 |
| 1/27 | 0,761021692 | 1 | 0 | 0 |
| 1/27 | 0,761021692 | 1 | 0 | 0 |
| 1/27 | 0,761021692 | 1 | 0 | 0 |
| 19/423 | 0,76565639 | 1 | 0 | 0 |
| 2/53 | 0,766093226 | 1 | 0 | 0 |
| 2/53 | 0,766093226 | 1 | 0 | 0 |
| 3/77 | 0,766227792 | 1 | 0 | 0 |
| 3/77 | 0,766227792 | 1 | 0 | 0 |
| 3/77 | 0,766227792 | 1 | 0 | 0 |
| 5/124 | 0,772963985 | 1 | 0 | 0 |
| 1/28 | 0,773369566 | 1 | 0 | 0 |
| 1/28 | 0,773369566 | 1 | 0 | 0 |
| 1/28 | 0,773369566 | 1 | 0 | 0 |
| 1/28 | 0,773369566 | 1 | 0 | 0 |
| 1/28 | 0,773369566 | 1 | 0 | 0 |
| 1/28 | 0,773369566 | 1 | 0 | 0 |
| 1/28 | 0,773369566 | 1 | 0 | 0 |
| 1/28 | 0,773369566 | 1 | 0 | 0 |
| 1/28 | 0,773369566 | 1 | 0 | 0 |
| 1/28 | 0,773369566 | 1 | 0 | 0 |
| 1/28 | 0,773369566 | 1 | 0 | 0 |
| 1/28 | 0,773369566 | 1 | 0 | 0 |
| 1/28 | 0,773369566 | 1 | 0 | 0 |
| 1/28 | 0,773369566 | 1 | 0 | 0 |
| 22/488 | 0,773466249 | 1 | 0 | 0 |
| 3/78 | 0,77379609 | 1 | 0 | 0 |
| 3/78 | 0,77379609 | 1 | 0 | 0 |
| 3/78 | 0,77379609 | 1 | 0 | 0 |
| 2/54 | 0,775076347 | 1 | 0 | 0 |
| 2/54 | 0,775076347 | 1 | 0 | 0 |
| 4/102 | 0,778228766 | 1 | 0 | 0 |
| 5/125 | 0,778921915 | 1 | 0 | 0 |
| 5/125 | 0,778921915 | 1 | 0 | 0 |
| 3/79 | 0,781162352 | 1 | 0 | 0 |
| 3/79 | 0,781162352 | 1 | 0 | 0 |

| | | | | |
|---|---|---|---|---|
| 3/79 | 0,781162352 | 1 | 0 | 0 |
| 3/79 | 0,781162352 | 1 | 0 | 0 |
| 2/55 | 0,783756324 | 1 | 0 | 0 |
| 2/55 | 0,783756324 | 1 | 0 | 0 |
| 2/55 | 0,783756324 | 1 | 0 | 0 |
| 2/55 | 0,783756324 | 1 | 0 | 0 |
| 5/126 | 0,78475876 | 1 | 0 | 0 |
| 1/29 | 0,785080013 | 1 | 0 | 0 |
| 1/29 | 0,785080013 | 1 | 0 | 0 |
| 1/29 | 0,785080013 | 1 | 0 | 0 |
| 1/29 | 0,785080013 | 1 | 0 | 0 |
| 1/29 | 0,785080013 | 1 | 0 | 0 |
| 1/29 | 0,785080013 | 1 | 0 | 0 |
| 1/29 | 0,785080013 | 1 | 0 | 0 |
| 1/29 | 0,785080013 | 1 | 0 | 0 |
| 1/29 | 0,785080013 | 1 | 0 | 0 |
| 1/29 | 0,785080013 | 1 | 0 | 0 |
| 1/29 | 0,785080013 | 1 | 0 | 0 |
| 1/29 | 0,785080013 | 1 | 0 | 0 |
| 1/29 | 0,785080013 | 1 | 0 | 0 |
| 1/29 | 0,785080013 | 1 | 0 | 0 |
| 3/80 | 0,788329565 | 1 | 0 | 0 |
| 3/80 | 0,788329565 | 1 | 0 | 0 |
| 5/127 | 0,790475465 | 1 | 0 | 0 |
| 2/56 | 0,792140489 | 1 | 0 | 0 |
| 2/56 | 0,792140489 | 1 | 0 | 0 |
| 3/81 | 0,795300825 | 1 | 0 | 0 |
| 3/81 | 0,795300825 | 1 | 0 | 0 |
| 5/128 | 0,796073042 | 1 | 0 | 0 |
| 1/30 | 0,796185909 | 1 | 0 | 0 |
| 1/30 | 0,796185909 | 1 | 0 | 0 |
| 1/30 | 0,796185909 | 1 | 0 | 0 |
| 1/30 | 0,796185909 | 1 | 0 | 0 |
| 1/30 | 0,796185909 | 1 | 0 | 0 |
| 1/30 | 0,796185909 | 1 | 0 | 0 |
| 1/30 | 0,796185909 | 1 | 0 | 0 |
| 1/30 | 0,796185909 | 1 | 0 | 0 |
| 1/30 | 0,796185909 | 1 | 0 | 0 |
| 2/57 | 0,800236223 | 1 | 0 | 0 |
| 2/57 | 0,800236223 | 1 | 0 | 0 |
| 2/57 | 0,800236223 | 1 | 0 | 0 |
| 2/57 | 0,800236223 | 1 | 0 | 0 |
| 2/57 | 0,800236223 | 1 | 0 | 0 |
| 2/57 | 0,800236223 | 1 | 0 | 0 |
| 3/82 | 0,802079325 | 1 | 0 | 0 |
| 3/82 | 0,802079325 | 1 | 0 | 0 |
| 1/31 | 0,806718435 | 1 | 0 | 0 |
| 1/31 | 0,806718435 | 1 | 0 | 0 |
| 1/31 | 0,806718435 | 1 | 0 | 0 |
| 1/31 | 0,806718435 | 1 | 0 | 0 |
| 1/31 | 0,806718435 | 1 | 0 | 0 |
| 1/31 | 0,806718435 | 1 | 0 | 0 |
| 1/31 | 0,806718435 | 1 | 0 | 0 |

| | | | | |
|---|---|---|---|---|
| 1/31 | 0,806718435 | 1 | 0 | 0 |
| 1/31 | 0,806718435 | 1 | 0 | 0 |
| 1/31 | 0,806718435 | 1 | 0 | 0 |
| 1/31 | 0,806718435 | 1 | 0 | 0 |
| 1/31 | 0,806718435 | 1 | 0 | 0 |
| 1/31 | 0,806718435 | 1 | 0 | 0 |
| 1/31 | 0,806718435 | 1 | 0 | 0 |
| 2/58 | 0,808050934 | 1 | 0 | 0 |
| 2/58 | 0,808050934 | 1 | 0 | 0 |
| 3/83 | 0,808668342 | 1 | 0 | 0 |
| 3/83 | 0,808668342 | 1 | 0 | 0 |
| 3/83 | 0,808668342 | 1 | 0 | 0 |
| 4/108 | 0,814586956 | 1 | 0 | 0 |
| 4/108 | 0,814586956 | 1 | 0 | 0 |
| 4/108 | 0,814586956 | 1 | 0 | 0 |
| 3/84 | 0,815071228 | 1 | 0 | 0 |
| 3/84 | 0,815071228 | 1 | 0 | 0 |
| 2/59 | 0,815592036 | 1 | 0 | 0 |
| 2/59 | 0,815592036 | 1 | 0 | 0 |
| 1/32 | 0,816707165 | 1 | 0 | 0 |
| 1/32 | 0,816707165 | 1 | 0 | 0 |
| 1/32 | 0,816707165 | 1 | 0 | 0 |
| 1/32 | 0,816707165 | 1 | 0 | 0 |
| 1/32 | 0,816707165 | 1 | 0 | 0 |
| 1/32 | 0,816707165 | 1 | 0 | 0 |
| 1/32 | 0,816707165 | 1 | 0 | 0 |
| 1/32 | 0,816707165 | 1 | 0 | 0 |
| 1/32 | 0,816707165 | 1 | 0 | 0 |
| 1/32 | 0,816707165 | 1 | 0 | 0 |
| 1/32 | 0,816707165 | 1 | 0 | 0 |
| 1/32 | 0,816707165 | 1 | 0 | 0 |
| 1/32 | 0,816707165 | 1 | 0 | 0 |
| 1/32 | 0,816707165 | 1 | 0 | 0 |
| 1/32 | 0,816707165 | 1 | 0 | 0 |
| 1/32 | 0,816707165 | 1 | 0 | 0 |
| 1/32 | 0,816707165 | 1 | 0 | 0 |
| 12/289 | 0,818293399 | 1 | 0 | 0 |
| 4/109 | 0,820145299 | 1 | 0 | 0 |
| 3/85 | 0,821291395 | 1 | 0 | 0 |
| 3/85 | 0,821291395 | 1 | 0 | 0 |
| 3/85 | 0,821291395 | 1 | 0 | 0 |
| 6/156 | 0,821544839 | 1 | 0 | 0 |
| 12/290 | 0,821769496 | 1 | 0 | 0 |
| 12/290 | 0,821769496 | 1 | 0 | 0 |
| 2/60 | 0,822866924 | 1 | 0 | 0 |
| 2/60 | 0,822866924 | 1 | 0 | 0 |
| 2/60 | 0,822866924 | 1 | 0 | 0 |
| 8/202 | 0,823976554 | 1 | 0 | 0 |
| 1/33 | 0,826180149 | 1 | 0 | 0 |
| 1/33 | 0,826180149 | 1 | 0 | 0 |
| 1/33 | 0,826180149 | 1 | 0 | 0 |
| 1/33 | 0,826180149 | 1 | 0 | 0 |
| 1/33 | 0,826180149 | 1 | 0 | 0 |

| | | | | |
|---|---|---|---|---|
| 1/33 | 0,826180149 | 1 | 0 | 0 |
| 1/33 | 0,826180149 | 1 | 0 | 0 |
| 1/33 | 0,826180149 | 1 | 0 | 0 |
| 1/33 | 0,826180149 | 1 | 0 | 0 |
| 1/33 | 0,826180149 | 1 | 0 | 0 |
| 1/33 | 0,826180149 | 1 | 0 | 0 |
| 5/134 | 0,827220937 | 1 | 0 | 0 |
| 12/292 | 0,828572145 | 1 | 0 | 0 |
| 2/61 | 0,829882961 | 1 | 0 | 0 |
| 2/61 | 0,829882961 | 1 | 0 | 0 |
| 4/111 | 0,83084979 | 1 | 0 | 0 |
| 1/34 | 0,835163993 | 1 | 0 | 0 |
| 1/34 | 0,835163993 | 1 | 0 | 0 |
| 1/34 | 0,835163993 | 1 | 0 | 0 |
| 1/34 | 0,835163993 | 1 | 0 | 0 |
| 1/34 | 0,835163993 | 1 | 0 | 0 |
| 1/34 | 0,835163993 | 1 | 0 | 0 |
| 1/34 | 0,835163993 | 1 | 0 | 0 |
| 1/34 | 0,835163993 | 1 | 0 | 0 |
| 1/34 | 0,835163993 | 1 | 0 | 0 |
| 1/34 | 0,835163993 | 1 | 0 | 0 |
| 1/34 | 0,835163993 | 1 | 0 | 0 |
| 1/34 | 0,835163993 | 1 | 0 | 0 |
| 8/205 | 0,835823282 | 1 | 0 | 0 |
| 2/62 | 0,83664746 | 1 | 0 | 0 |
| 2/62 | 0,83664746 | 1 | 0 | 0 |
| 2/62 | 0,83664746 | 1 | 0 | 0 |
| 2/62 | 0,83664746 | 1 | 0 | 0 |
| 2/62 | 0,83664746 | 1 | 0 | 0 |
| 5/136 | 0,836705463 | 1 | 0 | 0 |
| 3/88 | 0,838890424 | 1 | 0 | 0 |
| 3/88 | 0,838890424 | 1 | 0 | 0 |
| 3/88 | 0,838890424 | 1 | 0 | 0 |
| 7/183 | 0,838917274 | 1 | 0 | 0 |
| 7/184 | 0,842876206 | 1 | 0 | 0 |
| 2/63 | 0,843167667 | 1 | 0 | 0 |
| 2/63 | 0,843167667 | 1 | 0 | 0 |
| 1/35 | 0,84368393 | 1 | 0 | 0 |
| 1/35 | 0,84368393 | 1 | 0 | 0 |
| 1/35 | 0,84368393 | 1 | 0 | 0 |
| 1/35 | 0,84368393 | 1 | 0 | 0 |
| 1/35 | 0,84368393 | 1 | 0 | 0 |
| 1/35 | 0,84368393 | 1 | 0 | 0 |
| 1/35 | 0,84368393 | 1 | 0 | 0 |
| 1/35 | 0,84368393 | 1 | 0 | 0 |
| 1/35 | 0,84368393 | 1 | 0 | 0 |
| 3/89 | 0,844414733 | 1 | 0 | 0 |
| 2/64 | 0,849450754 | 1 | 0 | 0 |
| 2/64 | 0,849450754 | 1 | 0 | 0 |
| 2/64 | 0,849450754 | 1 | 0 | 0 |
| 2/64 | 0,849450754 | 1 | 0 | 0 |
| 2/64 | 0,849450754 | 1 | 0 | 0 |
| 3/90 | 0,849773978 | 1 | 0 | 0 |

| | | | | |
|---|---|---|---|---|
| 1/36 | 0,851763893 | 1 | 0 | 0 |
| 1/36 | 0,851763893 | 1 | 0 | 0 |
| 1/36 | 0,851763893 | 1 | 0 | 0 |
| 1/36 | 0,851763893 | 1 | 0 | 0 |
| 1/36 | 0,851763893 | 1 | 0 | 0 |
| 1/36 | 0,851763893 | 1 | 0 | 0 |
| 1/36 | 0,851763893 | 1 | 0 | 0 |
| 1/36 | 0,851763893 | 1 | 0 | 0 |
| 1/36 | 0,851763893 | 1 | 0 | 0 |
| 1/36 | 0,851763893 | 1 | 0 | 0 |
| 1/36 | 0,851763893 | 1 | 0 | 0 |
| 2/65 | 0,855503801 | 1 | 0 | 0 |
| 2/65 | 0,855503801 | 1 | 0 | 0 |
| 2/65 | 0,855503801 | 1 | 0 | 0 |
| 2/65 | 0,855503801 | 1 | 0 | 0 |
| 2/65 | 0,855503801 | 1 | 0 | 0 |
| 6/164 | 0,855564427 | 1 | 0 | 0 |
| 1/37 | 0,859426581 | 1 | 0 | 0 |
| 1/37 | 0,859426581 | 1 | 0 | 0 |
| 1/37 | 0,859426581 | 1 | 0 | 0 |
| 1/37 | 0,859426581 | 1 | 0 | 0 |
| 1/37 | 0,859426581 | 1 | 0 | 0 |
| 1/37 | 0,859426581 | 1 | 0 | 0 |
| 1/37 | 0,859426581 | 1 | 0 | 0 |
| 1/37 | 0,859426581 | 1 | 0 | 0 |
| 2/66 | 0,861333792 | 1 | 0 | 0 |
| 2/66 | 0,861333792 | 1 | 0 | 0 |
| 2/66 | 0,861333792 | 1 | 0 | 0 |
| 2/66 | 0,861333792 | 1 | 0 | 0 |
| 2/66 | 0,861333792 | 1 | 0 | 0 |
| 3/93 | 0,864897235 | 1 | 0 | 0 |
| 1/38 | 0,866693525 | 1 | 0 | 0 |
| 1/38 | 0,866693525 | 1 | 0 | 0 |
| 1/38 | 0,866693525 | 1 | 0 | 0 |
| 1/38 | 0,866693525 | 1 | 0 | 0 |
| 1/38 | 0,866693525 | 1 | 0 | 0 |
| 2/67 | 0,8669476 | 1 | 0 | 0 |
| 2/67 | 0,8669476 | 1 | 0 | 0 |
| 3/94 | 0,869632119 | 1 | 0 | 0 |
| 2/68 | 0,872351988 | 1 | 0 | 0 |
| 2/68 | 0,872351988 | 1 | 0 | 0 |
| 1/39 | 0,873585143 | 1 | 0 | 0 |
| 1/39 | 0,873585143 | 1 | 0 | 0 |
| 1/39 | 0,873585143 | 1 | 0 | 0 |
| 1/39 | 0,873585143 | 1 | 0 | 0 |
| 3/95 | 0,874219853 | 1 | 0 | 0 |
| 7/193 | 0,875090765 | 1 | 0 | 0 |
| 3/96 | 0,878663982 | 1 | 0 | 0 |
| 1/40 | 0,880120803 | 1 | 0 | 0 |
| 1/40 | 0,880120803 | 1 | 0 | 0 |
| 1/40 | 0,880120803 | 1 | 0 | 0 |
| 1/40 | 0,880120803 | 1 | 0 | 0 |
| 1/40 | 0,880120803 | 1 | 0 | 0 |

| | | | | |
|---|---|---|---|---|
| 1/40 | 0,880120803 | 1 | 0 | 0 |
| 1/40 | 0,880120803 | 1 | 0 | 0 |
| 7/195 | 0,881454616 | 1 | 0 | 0 |
| 1/41 | 0,886318873 | 1 | 0 | 0 |
| 1/41 | 0,886318873 | 1 | 0 | 0 |
| 1/41 | 0,886318873 | 1 | 0 | 0 |
| 1/41 | 0,886318873 | 1 | 0 | 0 |
| 1/41 | 0,886318873 | 1 | 0 | 0 |
| 1/41 | 0,886318873 | 1 | 0 | 0 |
| 1/41 | 0,886318873 | 1 | 0 | 0 |
| 1/41 | 0,886318873 | 1 | 0 | 0 |
| 1/41 | 0,886318873 | 1 | 0 | 0 |
| 1/41 | 0,886318873 | 1 | 0 | 0 |
| 1/41 | 0,886318873 | 1 | 0 | 0 |
| 1/41 | 0,886318873 | 1 | 0 | 0 |
| 3/98 | 0,887135472 | 1 | 0 | 0 |
| 6/173 | 0,887273389 | 1 | 0 | 0 |
| 2/71 | 0,887374376 | 1 | 0 | 0 |
| 3/99 | 0,89116978 | 1 | 0 | 0 |
| 3/99 | 0,89116978 | 1 | 0 | 0 |
| 2/72 | 0,89200618 | 1 | 0 | 0 |
| 2/72 | 0,89200618 | 1 | 0 | 0 |
| 2/72 | 0,89200618 | 1 | 0 | 0 |
| 2/72 | 0,89200618 | 1 | 0 | 0 |
| 1/42 | 0,892196776 | 1 | 0 | 0 |
| 1/42 | 0,892196776 | 1 | 0 | 0 |
| 1/42 | 0,892196776 | 1 | 0 | 0 |
| 1/42 | 0,892196776 | 1 | 0 | 0 |
| 1/42 | 0,892196776 | 1 | 0 | 0 |
| 1/42 | 0,892196776 | 1 | 0 | 0 |
| 1/42 | 0,892196776 | 1 | 0 | 0 |
| 1/42 | 0,892196776 | 1 | 0 | 0 |
| 6/175 | 0,893458839 | 1 | 0 | 0 |
| 2/73 | 0,896460449 | 1 | 0 | 0 |
| 2/73 | 0,896460449 | 1 | 0 | 0 |
| 1/43 | 0,897771034 | 1 | 0 | 0 |
| 1/43 | 0,897771034 | 1 | 0 | 0 |
| 1/43 | 0,897771034 | 1 | 0 | 0 |
| 1/43 | 0,897771034 | 1 | 0 | 0 |
| 1/43 | 0,897771034 | 1 | 0 | 0 |
| 2/74 | 0,900743148 | 1 | 0 | 0 |
| 4/128 | 0,9019964 | 1 | 0 | 0 |
| 1/44 | 0,903057318 | 1 | 0 | 0 |
| 1/44 | 0,903057318 | 1 | 0 | 0 |
| 5/154 | 0,904174253 | 1 | 0 | 0 |
| 2/75 | 0,904860104 | 1 | 0 | 0 |
| 5/155 | 0,90708417 | 1 | 0 | 0 |
| 1/45 | 0,908070493 | 1 | 0 | 0 |
| 1/45 | 0,908070493 | 1 | 0 | 0 |
| 1/45 | 0,908070493 | 1 | 0 | 0 |
| 1/45 | 0,908070493 | 1 | 0 | 0 |
| 1/45 | 0,908070493 | 1 | 0 | 0 |
| 1/45 | 0,908070493 | 1 | 0 | 0 |

| | | | | |
|---|---|---|---|---|
| 2/76 | 0,908816996 | 1 | 0 | 0 |
| 2/76 | 0,908816996 | 1 | 0 | 0 |
| 9/253 | 0,910017897 | 1 | 0 | 0 |
| 2/77 | 0,912619361 | 1 | 0 | 0 |
| 3/105 | 0,912768376 | 1 | 0 | 0 |
| 1/46 | 0,912824654 | 1 | 0 | 0 |
| 2/78 | 0,916272592 | 1 | 0 | 0 |
| 1/47 | 0,917333173 | 1 | 0 | 0 |
| 1/47 | 0,917333173 | 1 | 0 | 0 |
| 1/47 | 0,917333173 | 1 | 0 | 0 |
| 1/47 | 0,917333173 | 1 | 0 | 0 |
| 8/233 | 0,918371274 | 1 | 0 | 0 |
| 3/107 | 0,919052811 | 1 | 0 | 0 |
| 3/107 | 0,919052811 | 1 | 0 | 0 |
| 3/107 | 0,919052811 | 1 | 0 | 0 |
| 2/79 | 0,919781935 | 1 | 0 | 0 |
| 2/79 | 0,919781935 | 1 | 0 | 0 |
| 2/79 | 0,919781935 | 1 | 0 | 0 |
| 1/48 | 0,921608729 | 1 | 0 | 0 |
| 1/48 | 0,921608729 | 1 | 0 | 0 |
| 1/48 | 0,921608729 | 1 | 0 | 0 |
| 1/48 | 0,921608729 | 1 | 0 | 0 |
| 1/48 | 0,921608729 | 1 | 0 | 0 |
| 1/48 | 0,921608729 | 1 | 0 | 0 |
| 2/80 | 0,923152495 | 1 | 0 | 0 |
| 4/136 | 0,925205852 | 1 | 0 | 0 |
| 1/49 | 0,925663348 | 1 | 0 | 0 |
| 1/49 | 0,925663348 | 1 | 0 | 0 |
| 1/49 | 0,925663348 | 1 | 0 | 0 |
| 2/81 | 0,926389233 | 1 | 0 | 0 |
| 2/81 | 0,926389233 | 1 | 0 | 0 |
| 6/189 | 0,929144567 | 1 | 0 | 0 |
| 2/82 | 0,929496968 | 1 | 0 | 0 |
| 2/82 | 0,929496968 | 1 | 0 | 0 |
| 2/82 | 0,929496968 | 1 | 0 | 0 |
| 1/50 | 0,929508438 | 1 | 0 | 0 |
| 1/50 | 0,929508438 | 1 | 0 | 0 |
| 1/50 | 0,929508438 | 1 | 0 | 0 |
| 1/50 | 0,929508438 | 1 | 0 | 0 |
| 2/83 | 0,932480377 | 1 | 0 | 0 |
| 1/51 | 0,933154815 | 1 | 0 | 0 |
| 1/51 | 0,933154815 | 1 | 0 | 0 |
| 1/51 | 0,933154815 | 1 | 0 | 0 |
| 3/113 | 0,935503929 | 1 | 0 | 0 |
| 1/52 | 0,93661274 | 1 | 0 | 0 |
| 1/52 | 0,93661274 | 1 | 0 | 0 |
| 1/52 | 0,93661274 | 1 | 0 | 0 |
| 1/52 | 0,93661274 | 1 | 0 | 0 |
| 1/53 | 0,939891944 | 1 | 0 | 0 |
| 1/53 | 0,939891944 | 1 | 0 | 0 |
| 2/86 | 0,940729373 | 1 | 0 | 0 |
| 1/54 | 0,943001654 | 1 | 0 | 0 |
| 1/54 | 0,943001654 | 1 | 0 | 0 |

| | | | | |
|---|---|---|---|---|
| 1/54 | 0,943001654 | 1 | 0 | 0 |
| 2/88 | 0,945686698 | 1 | 0 | 0 |
| 1/55 | 0,945950624 | 1 | 0 | 0 |
| 1/55 | 0,945950624 | 1 | 0 | 0 |
| 1/55 | 0,945950624 | 1 | 0 | 0 |
| 18/485 | 0,946897816 | 1 | 0 | 0 |
| 2/90 | 0,950247508 | 1 | 0 | 0 |
| 1/57 | 0,951399117 | 1 | 0 | 0 |
| 1/57 | 0,951399117 | 1 | 0 | 0 |
| 1/57 | 0,951399117 | 1 | 0 | 0 |
| 1/57 | 0,951399117 | 1 | 0 | 0 |
| 1/57 | 0,951399117 | 1 | 0 | 0 |
| 2/91 | 0,952388524 | 1 | 0 | 0 |
| 2/91 | 0,952388524 | 1 | 0 | 0 |
| 1/58 | 0,95391398 | 1 | 0 | 0 |
| 1/59 | 0,956298824 | 1 | 0 | 0 |
| 1/59 | 0,956298824 | 1 | 0 | 0 |
| 1/60 | 0,958560365 | 1 | 0 | 0 |
| 1/60 | 0,958560365 | 1 | 0 | 0 |
| 24/634 | 0,958739765 | 1 | 0 | 0 |
| 1/61 | 0,960704971 | 1 | 0 | 0 |
| 2/96 | 0,961836591 | 1 | 0 | 0 |
| 1/62 | 0,962738684 | 1 | 0 | 0 |
| 2/97 | 0,96349697 | 1 | 0 | 0 |
| 5/184 | 0,963793028 | 1 | 0 | 0 |
| 1/63 | 0,964667232 | 1 | 0 | 0 |
| 1/64 | 0,966496048 | 1 | 0 | 0 |
| 1/65 | 0,968230286 | 1 | 0 | 0 |
| 1/66 | 0,969874831 | 1 | 0 | 0 |
| 1/66 | 0,969874831 | 1 | 0 | 0 |
| 1/66 | 0,969874831 | 1 | 0 | 0 |
| 1/68 | 0,972913143 | 1 | 0 | 0 |
| 1/69 | 0,974315473 | 1 | 0 | 0 |
| 1/69 | 0,974315473 | 1 | 0 | 0 |
| 4/167 | 0,975424135 | 1 | 0 | 0 |
| 1/70 | 0,975645263 | 1 | 0 | 0 |
| 1/71 | 0,97690626 | 1 | 0 | 0 |
| 2/109 | 0,978717145 | 1 | 0 | 0 |
| 2/109 | 0,978717145 | 1 | 0 | 0 |
| 3/142 | 0,979574614 | 1 | 0 | 0 |
| 2/110 | 0,979661768 | 1 | 0 | 0 |
| 1/74 | 0,980311144 | 1 | 0 | 0 |
| 1/74 | 0,980311144 | 1 | 0 | 0 |
| 1/74 | 0,980311144 | 1 | 0 | 0 |
| 5/203 | 0,981307067 | 1 | 0 | 0 |
| 1/75 | 0,981330738 | 1 | 0 | 0 |
| 1/77 | 0,983214376 | 1 | 0 | 0 |
| 1/80 | 0,985689806 | 1 | 0 | 0 |
| 1/80 | 0,985689806 | 1 | 0 | 0 |
| 1/82 | 0,987133925 | 1 | 0 | 0 |
| 1/84 | 0,988432409 | 1 | 0 | 0 |
| 1/89 | 0,991134165 | 1 | 0 | 0 |
| 1/89 | 0,991134165 | 1 | 0 | 0 |

| | | | | |
|---|---|---|---|---|
| 2/129 | 0,991509729 | 1 | 0 | 0 |
| 3/164 | 0,991839589 | 1 | 0 | 0 |
| 5/227 | 0,992210191 | 1 | 0 | 0 |
| 5/228 | 0,992495679 | 1 | 0 | 0 |
| 1/94 | 0,993205206 | 1 | 0 | 0 |
| 1/94 | 0,993205206 | 1 | 0 | 0 |
| 4/201 | 0,993399269 | 1 | 0 | 0 |
| 1/98 | 0,994508015 | 1 | 0 | 0 |
| 1/100 | 0,995062553 | 1 | 0 | 0 |
| 1/104 | 0,996009353 | 1 | 0 | 0 |
| 1/105 | 0,996216204 | 1 | 0 | 0 |
| 1/105 | 0,996216204 | 1 | 0 | 0 |
| 1/109 | 0,996941817 | 1 | 0 | 0 |
| 1/110 | 0,997100339 | 1 | 0 | 0 |
| 1/111 | 0,997250643 | 1 | 0 | 0 |
| 1/114 | 0,997656398 | 1 | 0 | 0 |
| 1/114 | 0,997656398 | 1 | 0 | 0 |
| 1/115 | 0,997777873 | 1 | 0 | 0 |
| 1/122 | 0,998468975 | 1 | 0 | 0 |
| 1/124 | 0,998623528 | 1 | 0 | 0 |
| 1/126 | 0,998762462 | 1 | 0 | 0 |
| 1/129 | 0,998944982 | 1 | 0 | 0 |
| 3/242 | 0,999739865 | 1 | 0 | 0 |
| 3/252 | 0,999834478 | 1 | 0 | 0 |
| 3/258 | 0,999873704 | 1 | 0 | 0 |

| Odds.Ratio | Combined.Score |
| --- | --- |
| 1,999885721 | 41,58742742 |
| 1,780663721 | 33,49244629 |
| 2,234654091 | 41,56706491 |
| 1,637917931 | 28,84876449 |
| 2,16748266 | 33,71199338 |
| 3,402568199 | 52,00349346 |
| 1,780480456 | 25,78600799 |
| 1,539237217 | 22,17766996 |
| 1,782343254 | 23,75739245 |
| 1,878498704 | 24,62781983 |
| 1,926651253 | 23,21875582 |
| 2,003309816 | 24,07333149 |
| 2,613012804 | 28,89445341 |
| 1,814720465 | 19,55271052 |
| 4,15282392 | 44,4085336 |
| 4,07996736 | 42,85923903 |
| 2,76854928 | 28,48282178 |
| 3,812428517 | 37,33775784 |
| 2,673082064 | 26,13871038 |
| 4,714016342 | 44,23546722 |
| 1,794430089 | 16,32387574 |
| 2,047166721 | 17,83179976 |
| 4,254112309 | 36,30541227 |
| 11,07419712 | 93,3877138 |
| 2,456600066 | 19,34236013 |
| 1,86998504 | 14,50553172 |
| 1,84569952 | 14,23471225 |
| 2,186444047 | 16,70261823 |
| 3,22997416 | 24,44225275 |
| 3,4606866 | 26,15183161 |
| 2,642706131 | 19,96242502 |
| 2,458637047 | 18,49142464 |
| 4,677893611 | 35,13084943 |
| 2,862931642 | 21,43837342 |
| 5,285412262 | 38,54968664 |
| 2,310180194 | 16,47700387 |
| 2,063594602 | 14,67331267 |
| 3,875968992 | 27,35873171 |
| 2,044662542 | 14,28088926 |
| 6,05620155 | 41,94775145 |
| 6,05620155 | 41,94775145 |
| 6,05620155 | 41,94775145 |
| 1,64365549 | 11,37271362 |
| 7,751937984 | 52,45132753 |
| 7,751937984 | 52,45132753 |
| 7,751937984 | 52,45132753 |
| 7,751937984 | 52,45132753 |
| 3,69139904 | 24,81143421 |
| 2,920250611 | 19,5589131 |
| 1,858341298 | 12,44146708 |
| 5,6999544 | 37,74061275 |
| 1,95427008 | 12,89728 |

| | |
|---|---|
| 2,880787765 | 18,96581487 |
| 1,645846706 | 10,78778523 |
| 2,193944713 | 14,33423991 |
| 3,98996808 | 25,92538617 |
| 2,466525722 | 15,92441091 |
| 1,99232051 | 12,75993993 |
| 2,544828126 | 16,21041782 |
| 5,383290267 | 34,12379901 |
| 2,936340146 | 18,33062615 |
| 9,689922481 | 58,30911925 |
| 9,689922481 | 58,30911925 |
| 9,689922481 | 58,30911925 |
| 6,45994832 | 38,70648143 |
| 6,45994832 | 38,70648143 |
| 3,666457155 | 21,93687393 |
| 2,199131343 | 13,14173637 |
| 3,298697015 | 19,68990491 |
| 2,527805865 | 14,96302294 |
| 2,631830797 | 15,38448731 |
| 2,236135957 | 13,0646707 |
| 2,399409376 | 14,01072131 |
| 2,76854928 | 16,04799184 |
| 2,5997353 | 14,94357175 |
| 5,963029219 | 33,78303964 |
| 2,568413188 | 14,51800651 |
| 4,6142488 | 25,85182255 |
| 4,6142488 | 25,85182255 |
| 8,305647841 | 45,65548196 |
| 8,305647841 | 45,65548196 |
| 8,305647841 | 45,65548196 |
| 2,223916635 | 12,20288756 |
| 2,397506593 | 13,13443161 |
| 2,813203301 | 15,31253022 |
| 3,750937734 | 20,2582527 |
| 2,76854928 | 14,76934109 |
| 2,981514609 | 15,87053117 |
| 2,725290698 | 14,25012536 |
| 4,213009774 | 21,84523656 |
| 1,898026053 | 9,836974688 |
| 2,20998232 | 11,33761009 |
| 5,167958656 | 26,36331328 |
| 5,167958656 | 26,36331328 |
| 5,167958656 | 26,36331328 |
| 5,167958656 | 26,36331328 |
| 5,167958656 | 26,36331328 |
| 5,167958656 | 26,36331328 |
| 3,523608175 | 17,90028517 |
| 7,26744186 | 36,81572007 |
| 7,26744186 | 36,81572007 |
| 7,26744186 | 36,81572007 |
| 7,26744186 | 36,81572007 |
| 7,26744186 | 36,81572007 |
| 3,41997264 | 16,85925491 |

| | |
|---|---|
| 3,41997264 | 16,85925491 |
| 2,236135957 | 11,00115077 |
| 2,76854928 | 13,47801937 |
| 1,727660597 | 8,394494538 |
| 3,01464255 | 14,64132408 |
| 4,84496124 | 23,5210893 |
| 4,84496124 | 23,5210893 |
| 4,84496124 | 23,5210893 |
| 4,84496124 | 23,5210893 |
| 1,597239969 | 7,717089486 |
| 2,56497948 | 12,38755367 |
| 2,004811548 | 9,675452047 |
| 3,875968992 | 18,65774953 |
| 3,322259136 | 15,89781298 |
| 1,562103372 | 7,427390749 |
| 6,45994832 | 30,3565599 |
| 1,55427328 | 7,281922842 |
| 1,7012841 | 7,966061834 |
| 2,363395727 | 11,02433501 |
| 3,22997416 | 15,00812038 |
| 3,726893262 | 17,30271119 |
| 3,726893262 | 17,30271119 |
| 3,726893262 | 17,30271119 |
| 4,55996352 | 21,10141337 |
| 4,55996352 | 21,10141337 |
| 4,55996352 | 21,10141337 |
| 4,55996352 | 21,10141337 |
| 4,55996352 | 21,10141337 |
| 4,55996352 | 21,10141337 |
| 2,886359888 | 13,33263431 |
| 2,33492108 | 10,69892368 |
| 2,627775588 | 11,97723216 |
| 2,456600066 | 11,18511944 |
| 3,142677561 | 14,18329016 |
| 2,82622739 | 12,73466308 |
| 3,588860178 | 16,07931778 |
| 3,588860178 | 16,07931778 |
| 1,717201452 | 7,69321232 |
| 1,840543935 | 8,24164411 |
| 2,583979328 | 11,52394911 |
| 2,27998176 | 10,08191194 |
| 4,306632214 | 19,02322652 |
| 4,306632214 | 19,02322652 |
| 4,306632214 | 19,02322652 |
| 2,17528872 | 9,594409495 |
| 2,17528872 | 9,594409495 |
| 1,448960371 | 6,381252256 |
| 1,7303433 | 7,61695116 |
| 2,76854928 | 12,17060025 |
| 1,637733377 | 7,19123162 |
| 3,05997552 | 13,41725036 |
| 3,05997552 | 13,41725036 |
| 5,813953488 | 25,4722545 |

| | |
|---|---|
| 5,813953488 | 25,4722545 |
| 5,813953488 | 25,4722545 |
| 5,813953488 | 25,4722545 |
| 5,813953488 | 25,4722545 |
| 5,813953488 | 25,4722545 |
| 5,813953488 | 25,4722545 |
| 5,813953488 | 25,4722545 |
| 5,813953488 | 25,4722545 |
| 1,306925961 | 5,669747589 |
| 3,4606866 | 14,97105531 |
| 1,937984496 | 8,343661351 |
| 1,589514098 | 6,82902225 |
| 2,357008171 | 10,12220394 |
| 2,713178295 | 11,63800197 |
| 2,500625156 | 10,67962542 |
| 4,07996736 | 17,22434882 |
| 4,07996736 | 17,22434882 |
| 4,07996736 | 17,22434882 |
| 2,325581395 | 9,79537826 |
| 2,110676184 | 8,863559428 |
| 3,34135258 | 13,963954 |
| 3,34135258 | 13,963954 |
| 3,34135258 | 13,963954 |
| 3,34135258 | 13,963954 |
| 2,906976744 | 12,04066138 |
| 5,285412262 | 21,67743134 |
| 5,285412262 | 21,67743134 |
| 2,42248062 | 9,910446483 |
| 1,627315226 | 6,606907577 |
| 2,265176684 | 9,179018154 |
| 2,153316107 | 8,71516804 |
| 3,875968992 | 15,65636772 |
| 3,875968992 | 15,65636772 |
| 3,875968992 | 15,65636772 |
| 3,875968992 | 15,65636772 |
| 1,502313563 | 6,067766135 |
| 2,385211688 | 9,55140324 |
| 2,236135957 | 8,888308786 |
| 1,95427008 | 7,648746495 |
| 1,95427008 | 7,648746495 |
| 3,125781445 | 12,20740068 |
| 3,125781445 | 12,20740068 |
| 3,125781445 | 12,20740068 |
| 3,125781445 | 12,20740068 |
| 3,125781445 | 12,20740068 |
| 3,125781445 | 12,20740068 |
| 2,512202125 | 9,780514918 |
| 2,011115987 | 7,783786852 |
| 3,69139904 | 14,28108748 |
| 3,69139904 | 14,28108748 |
| 3,69139904 | 14,28108748 |
| 3,69139904 | 14,28108748 |
| 1,937984496 | 7,470458436 |

| | |
|---|---|
| 4,84496124 | 18,66362223 |
| 4,84496124 | 18,66362223 |
| 4,84496124 | 18,66362223 |
| 2,180232558 | 8,339146213 |
| 1,99232051 | 7,586407507 |
| 1,99232051 | 7,586407507 |
| 1,921968095 | 7,29682921 |
| 3,028100775 | 11,43902942 |
| 3,028100775 | 11,43902942 |
| 2,27998176 | 8,565508793 |
| 2,153316107 | 8,079710139 |
| 2,153316107 | 8,079710139 |
| 3,523608175 | 13,0680136 |
| 3,523608175 | 13,0680136 |
| 3,523608175 | 13,0680136 |
| 3,523608175 | 13,0680136 |
| 2,642706131 | 9,792955667 |
| 2,936340146 | 10,73345316 |
| 4,472271914 | 16,22619769 |
| 4,472271914 | 16,22619769 |
| 4,472271914 | 16,22619769 |
| 4,472271914 | 16,22619769 |
| 4,472271914 | 16,22619769 |
| 4,472271914 | 16,22619769 |
| 4,472271914 | 16,22619769 |
| 4,472271914 | 16,22619769 |
| 4,472271914 | 16,22619769 |
| 4,472271914 | 16,22619769 |
| 2,101428972 | 7,588874957 |
| 2,583979328 | 9,316902113 |
| 2,583979328 | 9,316902113 |
| 2,583979328 | 9,316902113 |
| 2,214839424 | 7,976336846 |
| 3,370407819 | 11,99253845 |
| 3,370407819 | 11,99253845 |
| 3,370407819 | 11,99253845 |
| 3,370407819 | 11,99253845 |
| 3,370407819 | 11,99253845 |
| 2,07641196 | 7,356648428 |
| 2,8499772 | 10,08411019 |
| 2,8499772 | 10,08411019 |
| 2,8499772 | 10,08411019 |
| 2,183644503 | 7,700064552 |
| 1,9775352 | 6,953376449 |
| 2,299303639 | 7,9489053 |
| 2,76854928 | 9,485280904 |
| 2,76854928 | 9,485280904 |
| 2,76854928 | 9,485280904 |
| 2,76854928 | 9,485280904 |
| 4,15282392 | 14,22455135 |
| 4,15282392 | 14,22455135 |
| 4,15282392 | 14,22455135 |
| 4,15282392 | 14,22455135 |

| | |
|---|---|
| 4,15282392 | 14,22455135 |
| 4,15282392 | 14,22455135 |
| 4,15282392 | 14,22455135 |
| 4,15282392 | 14,22455135 |
| 4,15282392 | 14,22455135 |
| 4,15282392 | 14,22455135 |
| 4,15282392 | 14,22455135 |
| 4,15282392 | 14,22455135 |
| 4,15282392 | 14,22455135 |
| 2,474022761 | 8,449535228 |
| 2,474022761 | 8,449535228 |
| 2,474022761 | 8,449535228 |
| 1,658168553 | 5,660385191 |
| 1,680333378 | 5,661825245 |
| 1,802776275 | 6,059183986 |
| 6,45994832 | 21,70645267 |
| 6,45994832 | 21,70645267 |
| 6,45994832 | 21,70645267 |
| 6,45994832 | 21,70645267 |
| 6,45994832 | 21,70645267 |
| 6,45994832 | 21,70645267 |
| 6,45994832 | 21,70645267 |
| 6,45994832 | 21,70645267 |
| 6,45994832 | 21,70645267 |
| 6,45994832 | 21,70645267 |
| 6,45994832 | 21,70645267 |
| 6,45994832 | 21,70645267 |
| 6,45994832 | 21,70645267 |
| 6,45994832 | 21,70645267 |
| 6,45994832 | 21,70645267 |
| 6,45994832 | 21,70645267 |
| 6,45994832 | 21,70645267 |
| 6,45994832 | 21,70645267 |
| 6,45994832 | 21,70645267 |
| 6,45994832 | 21,70645267 |
| 6,45994832 | 21,70645267 |
| 6,45994832 | 21,70645267 |
| 6,45994832 | 21,70645267 |
| 6,45994832 | 21,70645267 |
| 6,45994832 | 21,70645267 |
| 6,45994832 | 21,70645267 |
| 1,918796531 | 6,40130298 |
| 2,691645134 | 8,931959959 |
| 2,691645134 | 8,931959959 |
| 2,691645134 | 8,931959959 |
| 2,095118374 | 6,937220484 |
| 2,223916635 | 7,335798167 |
| 1,631986944 | 5,36759124 |
| 3,100775194 | 10,1777513 |
| 1,407736922 | 4,610232714 |
| 1,775252974 | 5,787364465 |
| 3,875968992 | 12,55918396 |

| | |
|---|---|
| 3,875968992 | 12,55918396 |
| 3,875968992 | 12,55918396 |
| 3,875968992 | 12,55918396 |
| 3,875968992 | 12,55918396 |
| 3,875968992 | 12,55918396 |
| 3,875968992 | 12,55918396 |
| 3,875968992 | 12,55918396 |
| 3,875968992 | 12,55918396 |
| 3,875968992 | 12,55918396 |
| 3,875968992 | 12,55918396 |
| 1,68520391 | 5,458140738 |
| 2,37304224 | 7,681324916 |
| 1,822036706 | 5,895525117 |
| 1,959759603 | 6,312793915 |
| 2,618897968 | 8,419750834 |
| 2,618897968 | 8,419750834 |
| 2,03998368 | 6,478296822 |
| 1,55730897 | 4,930064231 |
| 2,981514609 | 9,408299464 |
| 2,981514609 | 9,408299464 |
| 1,702283679 | 5,366161258 |
| 2,325581395 | 7,329921039 |
| 2,5499796 | 7,944778207 |
| 2,5499796 | 7,944778207 |
| 2,5499796 | 7,944778207 |
| 2,5499796 | 7,944778207 |
| 1,84569952 | 5,741009741 |
| 2,013490386 | 6,262292079 |
| 1,776485788 | 5,473936273 |
| 2,119670543 | 6,52001306 |
| 2,119670543 | 6,52001306 |
| 3,63372093 | 11,15790341 |
| 3,63372093 | 11,15790341 |
| 3,63372093 | 11,15790341 |
| 3,63372093 | 11,15790341 |
| 3,63372093 | 11,15790341 |
| 3,63372093 | 11,15790341 |
| 3,63372093 | 11,15790341 |
| 3,63372093 | 11,15790341 |
| 5,53709856 | 16,93240671 |
| 5,53709856 | 16,93240671 |
| 5,53709856 | 16,93240671 |
| 5,53709856 | 16,93240671 |
| 5,53709856 | 16,93240671 |
| 5,53709856 | 16,93240671 |
| 5,53709856 | 16,93240671 |
| 5,53709856 | 16,93240671 |
| 5,53709856 | 16,93240671 |
| 5,53709856 | 16,93240671 |
| 5,53709856 | 16,93240671 |
| 5,53709856 | 16,93240671 |
| 5,53709856 | 16,93240671 |
| 5,53709856 | 16,93240671 |

| | |
|---|---|
| 5,53709856 | 16,93240671 |
| 5,53709856 | 16,93240671 |
| 5,53709856 | 16,93240671 |
| 5,53709856 | 16,93240671 |
| 5,53709856 | 16,93240671 |
| 5,53709856 | 16,93240671 |
| 5,53709856 | 16,93240671 |
| 5,53709856 | 16,93240671 |
| 1,82828726 | 5,588187379 |
| 2,871088142 | 8,714854065 |
| 2,871088142 | 8,714854065 |
| 2,484595508 | 7,503614852 |
| 2,236135957 | 6,684916596 |
| 2,236135957 | 6,684916596 |
| 2,236135957 | 6,684916596 |
| 1,74736307 | 5,211434793 |
| 1,61498708 | 4,752181273 |
| 2,055438102 | 6,036443209 |
| 2,42248062 | 7,093220319 |
| 1,937984496 | 5,663107337 |
| 2,76854928 | 8,087831292 |
| 2,76854928 | 8,087831292 |
| 2,76854928 | 8,087831292 |
| 3,41997264 | 9,967191743 |
| 3,41997264 | 9,967191743 |
| 3,41997264 | 9,967191743 |
| 3,41997264 | 9,967191743 |
| 3,41997264 | 9,967191743 |
| 3,41997264 | 9,967191743 |
| 3,41997264 | 9,967191743 |
| 3,41997264 | 9,967191743 |
| 3,41997264 | 9,967191743 |
| 3,41997264 | 9,967191743 |
| 2,193944713 | 6,38866695 |
| 1,364115301 | 3,919632221 |
| 2,024759921 | 5,810753559 |
| 2,024759921 | 5,810753559 |
| 1,914058762 | 5,478404972 |
| 1,761804087 | 5,020845507 |
| 1,661129568 | 4,71840137 |
| 2,363395727 | 6,710889265 |
| 2,363395727 | 6,710889265 |
| 2,363395727 | 6,710889265 |
| 2,363395727 | 6,710889265 |
| 2,363395727 | 6,710889265 |
| 2,363395727 | 6,710889265 |
| 1,218493246 | 3,430122359 |
| 2,673082064 | 7,51912473 |
| 2,673082064 | 7,51912473 |
| 2,673082064 | 7,51912473 |
| 2,673082064 | 7,51912473 |
| 2,673082064 | 7,51912473 |
| 2,673082064 | 7,51912473 |

| | |
|---|---|
| 2,673082064 | 7,51912473 |
| 2,673082064 | 7,51912473 |
| 2,673082064 | 7,51912473 |
| 2,673082064 | 7,51912473 |
| 4,84496124 | 13,58747994 |
| 4,84496124 | 13,58747994 |
| 4,84496124 | 13,58747994 |
| 4,84496124 | 13,58747994 |
| 4,84496124 | 13,58747994 |
| 4,84496124 | 13,58747994 |
| 4,84496124 | 13,58747994 |
| 4,84496124 | 13,58747994 |
| 4,84496124 | 13,58747994 |
| 4,84496124 | 13,58747994 |
| 4,84496124 | 13,58747994 |
| 4,84496124 | 13,58747994 |
| 4,84496124 | 13,58747994 |
| 4,84496124 | 13,58747994 |
| 4,84496124 | 13,58747994 |
| 4,84496124 | 13,58747994 |
| 4,84496124 | 13,58747994 |
| 4,84496124 | 13,58747994 |
| 4,84496124 | 13,58747994 |
| 4,84496124 | 13,58747994 |
| 4,84496124 | 13,58747994 |
| 1,527475465 | 4,26916767 |
| 3,22997416 | 8,946625228 |
| 3,22997416 | 8,946625228 |
| 3,22997416 | 8,946625228 |
| 3,22997416 | 8,946625228 |
| 3,22997416 | 8,946625228 |
| 3,22997416 | 8,946625228 |
| 3,22997416 | 8,946625228 |
| 2,3071244 | 6,354207732 |
| 2,3071244 | 6,354207732 |
| 2,3071244 | 6,354207732 |
| 2,3071244 | 6,354207732 |
| 1,867936864 | 5,129506462 |
| 1,966071228 | 5,388686681 |
| 1,966071228 | 5,388686681 |
| 2,583979328 | 7,001837457 |
| 2,583979328 | 7,001837457 |
| 2,583979328 | 7,001837457 |
| 1,665455426 | 4,503278339 |
| 2,07641196 | 5,591286245 |
| 2,07641196 | 5,591286245 |
| 1,84569952 | 4,964697916 |
| 1,550387597 | 4,141601743 |
| 1,6999864 | 4,516796533 |
| 3,05997552 | 8,065164774 |
| 3,05997552 | 8,065164774 |
| 3,05997552 | 8,065164774 |
| 3,05997552 | 8,065164774 |

| | |
|---|---|
| 3,05997552 | 8,065164774 |
| 3,05997552 | 8,065164774 |
| 1,91068894 | 5,00229892 |
| 2,500625156 | 6,530069643 |
| 2,500625156 | 6,530069643 |
| 2,500625156 | 6,530069643 |
| 2,500625156 | 6,530069643 |
| 2,500625156 | 6,530069643 |
| 1,403368083 | 3,644334559 |
| 2,202255109 | 5,709376829 |
| 2,202255109 | 5,709376829 |
| 4,306632214 | 11,14179549 |
| 4,306632214 | 11,14179549 |
| 4,306632214 | 11,14179549 |
| 4,306632214 | 11,14179549 |
| 4,306632214 | 11,14179549 |
| 4,306632214 | 11,14179549 |
| 4,306632214 | 11,14179549 |
| 4,306632214 | 11,14179549 |
| 4,306632214 | 11,14179549 |
| 4,306632214 | 11,14179549 |
| 4,306632214 | 11,14179549 |
| 4,306632214 | 11,14179549 |
| 4,306632214 | 11,14179549 |
| 4,306632214 | 11,14179549 |
| 4,306632214 | 11,14179549 |
| 4,306632214 | 11,14179549 |
| 4,306632214 | 11,14179549 |
| 4,306632214 | 11,14179549 |
| 4,306632214 | 11,14179549 |
| 4,306632214 | 11,14179549 |
| 4,306632214 | 11,14179549 |
| 1,726916878 | 4,431448533 |
| 1,322389421 | 3,392898583 |
| 1,67067629 | 4,285902512 |
| 1,884151593 | 4,821330346 |
| 1,884151593 | 4,821330346 |
| 2,004811548 | 5,126250728 |
| 2,004811548 | 5,126250728 |
| 1,38427464 | 3,538611952 |
| 1,70998632 | 4,305721154 |
| 2,42248062 | 6,098748804 |
| 2,42248062 | 6,098748804 |
| 2,42248062 | 6,098748804 |
| 2,153316107 | 5,417548418 |
| 2,153316107 | 5,417548418 |
| 2,153316107 | 5,417548418 |
| 2,153316107 | 5,417548418 |
| 2,906976744 | 7,298627913 |
| 2,906976744 | 7,298627913 |
| 2,906976744 | 7,298627913 |
| 2,906976744 | 7,298627913 |
| 2,906976744 | 7,298627913 |

| | |
|---|---|
| 2,906976744 | 7,298627913 |
| 2,906976744 | 7,298627913 |
| 2,906976744 | 7,298627913 |
| 2,906976744 | 7,298627913 |
| 1,858341298 | 4,647954556 |
| 1,858341298 | 4,647954556 |
| 1,858341298 | 4,647954556 |
| 1,858341298 | 4,647954556 |
| 1,229220935 | 3,045666543 |
| 1,761804087 | 4,363338631 |
| 1,560792883 | 3,858824269 |
| 1,358952543 | 3,35574635 |
| 1,6285584 | 3,963416612 |
| 1,937984496 | 4,706864245 |
| 1,937984496 | 4,706864245 |
| 1,937984496 | 4,706864245 |
| 1,937984496 | 4,706864245 |
| 2,349072117 | 5,703493121 |
| 2,349072117 | 5,703493121 |
| 2,349072117 | 5,703493121 |
| 2,349072117 | 5,703493121 |
| 1,350716467 | 3,267893644 |
| 3,875968992 | 9,293806274 |
| 3,875968992 | 9,293806274 |
| 3,875968992 | 9,293806274 |
| 3,875968992 | 9,293806274 |
| 3,875968992 | 9,293806274 |
| 3,875968992 | 9,293806274 |
| 3,875968992 | 9,293806274 |
| 3,875968992 | 9,293806274 |
| 3,875968992 | 9,293806274 |
| 3,875968992 | 9,293806274 |
| 3,875968992 | 9,293806274 |
| 3,875968992 | 9,293806274 |
| 3,875968992 | 9,293806274 |
| 3,875968992 | 9,293806274 |
| 3,875968992 | 9,293806274 |
| 3,875968992 | 9,293806274 |
| 3,875968992 | 9,293806274 |
| 3,875968992 | 9,293806274 |
| 3,875968992 | 9,293806274 |
| 3,875968992 | 9,293806274 |
| 2,76854928 | 6,627928123 |
| 2,76854928 | 6,627928123 |
| 2,76854928 | 6,627928123 |
| 2,76854928 | 6,627928123 |
| 1,61498708 | 3,861872171 |
| 1,420074846 | 3,376792242 |
| 2,061685634 | 4,887196592 |
| 2,061685634 | 4,887196592 |
| 2,061685634 | 4,887196592 |
| 1,601640079 | 3,763143706 |
| 1,601640079 | 3,763143706 |

| | |
|---|---|
| 1,601640079 | 3,763143706 |
| 2,27998176 | 5,340500606 |
| 1,78498572 | 4,169646036 |
| 1,703722634 | 3,966383947 |
| 1,703722634 | 3,966383947 |
| 2,01873385 | 4,645971938 |
| 1,544770251 | 3,543768092 |
| 2,642706131 | 6,037824525 |
| 2,642706131 | 6,037824525 |
| 2,642706131 | 6,037824525 |
| 2,642706131 | 6,037824525 |
| 2,642706131 | 6,037824525 |
| 2,642706131 | 6,037824525 |
| 2,642706131 | 6,037824525 |
| 2,642706131 | 6,037824525 |
| 2,642706131 | 6,037824525 |
| 2,642706131 | 6,037824525 |
| 2,642706131 | 6,037824525 |
| 2,642706131 | 6,037824525 |
| 2,642706131 | 6,037824525 |
| 2,642706131 | 6,037824525 |
| 2,214839424 | 5,006458647 |
| 2,214839424 | 5,006458647 |
| 2,214839424 | 5,006458647 |
| 2,214839424 | 5,006458647 |
| 1,84569952 | 4,15171171 |
| 1,61498708 | 3,629539863 |
| 1,667083438 | 3,724347396 |
| 3,523608175 | 7,860423421 |
| 3,523608175 | 7,860423421 |
| 3,523608175 | 7,860423421 |
| 3,523608175 | 7,860423421 |
| 3,523608175 | 7,860423421 |
| 3,523608175 | 7,860423421 |
| 3,523608175 | 7,860423421 |
| 3,523608175 | 7,860423421 |
| 3,523608175 | 7,860423421 |
| 3,523608175 | 7,860423421 |
| 3,523608175 | 7,860423421 |
| 3,523608175 | 7,860423421 |
| 3,523608175 | 7,860423421 |
| 3,523608175 | 7,860423421 |
| 3,523608175 | 7,860423421 |
| 3,523608175 | 7,860423421 |
| 3,523608175 | 7,860423421 |
| 3,523608175 | 7,860423421 |
| 3,523608175 | 7,860423421 |
| 3,523608175 | 7,860423421 |
| 3,523608175 | 7,860423421 |
| 3,523608175 | 7,860423421 |
| 3,523608175 | 7,860423421 |
| 3,523608175 | 7,860423421 |
| 3,523608175 | 7,860423421 |

| | |
|---|---|
| 3,523608175 | 7,860423421 |
| 3,523608175 | 7,860423421 |
| 1,396745583 | 3,060199475 |
| 1,550387597 | 3,394637314 |
| 1,649348507 | 3,609557658 |
| 2,527805865 | 5,516018479 |
| 2,527805865 | 5,516018479 |
| 1,717201452 | 3,7471302 |
| 2,153316107 | 4,698469725 |
| 2,153316107 | 4,698469725 |
| 2,153316107 | 4,698469725 |
| 1,788908766 | 3,824347528 |
| 1,695736434 | 3,617326474 |
| 1,695736434 | 3,617326474 |
| 1,273128501 | 2,713835904 |
| 1,352082207 | 2,864523515 |
| 1,490757305 | 3,14802583 |
| 1,8999848 | 4,004416356 |
| 1,8999848 | 4,004416356 |
| 2,095118374 | 4,413990065 |
| 2,095118374 | 4,413990065 |
| 2,095118374 | 4,413990065 |
| 2,095118374 | 4,413990065 |
| 2,095118374 | 4,413990065 |
| 2,095118374 | 4,413990065 |
| 2,095118374 | 4,413990065 |
| 1,61498708 | 3,391605274 |
| 3,22997416 | 6,724672735 |
| 3,22997416 | 6,724672735 |
| 3,22997416 | 6,724672735 |
| 3,22997416 | 6,724672735 |
| 3,22997416 | 6,724672735 |
| 3,22997416 | 6,724672735 |
| 3,22997416 | 6,724672735 |
| 3,22997416 | 6,724672735 |
| 3,22997416 | 6,724672735 |
| 3,22997416 | 6,724672735 |
| 3,22997416 | 6,724672735 |
| 3,22997416 | 6,724672735 |
| 3,22997416 | 6,724672735 |
| 3,22997416 | 6,724672735 |
| 3,22997416 | 6,724672735 |
| 3,22997416 | 6,724672735 |
| 3,22997416 | 6,724672735 |
| 3,22997416 | 6,724672735 |
| 3,22997416 | 6,724672735 |
| 3,22997416 | 6,724672735 |
| 3,22997416 | 6,724672735 |
| 3,22997416 | 6,724672735 |
| 1,514050388 | 3,143734169 |
| 1,514050388 | 3,143734169 |
| 1,598337729 | 3,288134589 |

| | |
|---|---|
| 1,654377009 | 3,372740619 |
| 2,03998368 | 4,150778681 |
| 2,03998368 | 4,150778681 |
| 2,03998368 | 4,150778681 |
| 2,03998368 | 4,150778681 |
| 2,03998368 | 4,150778681 |
| 2,325581395 | 4,639006775 |
| 2,325581395 | 4,639006775 |
| 2,325581395 | 4,639006775 |
| 2,325581395 | 4,639006775 |
| 2,325581395 | 4,639006775 |
| 2,325581395 | 4,639006775 |
| 2,325581395 | 4,639006775 |
| 2,325581395 | 4,639006775 |
| 2,325581395 | 4,639006775 |
| 2,325581395 | 4,639006775 |
| 1,634444756 | 3,257498161 |
| 1,634444756 | 3,257498161 |
| 1,634444756 | 3,257498161 |
| 1,82828726 | 3,635692848 |
| 1,70998632 | 3,3880701 |
| 1,70998632 | 3,3880701 |
| 1,70998632 | 3,3880701 |
| 1,70998632 | 3,3880701 |
| 1,45019248 | 2,864979282 |
| 1,566048078 | 3,091488617 |
| 1,566048078 | 3,091488617 |
| 1,987676406 | 3,906854855 |
| 1,987676406 | 3,906854855 |
| 1,987676406 | 3,906854855 |
| 1,987676406 | 3,906854855 |
| 1,358400348 | 2,650933901 |
| 1,61498708 | 3,146670379 |
| 2,981514609 | 5,80867905 |
| 2,981514609 | 5,80867905 |
| 2,981514609 | 5,80867905 |
| 2,981514609 | 5,80867905 |
| 2,981514609 | 5,80867905 |
| 2,981514609 | 5,80867905 |
| 2,981514609 | 5,80867905 |
| 2,981514609 | 5,80867905 |
| 2,981514609 | 5,80867905 |
| 2,981514609 | 5,80867905 |
| 2,981514609 | 5,80867905 |
| 2,981514609 | 5,80867905 |
| 2,981514609 | 5,80867905 |
| 2,981514609 | 5,80867905 |
| 2,981514609 | 5,80867905 |
| 2,981514609 | 5,80867905 |
| 2,981514609 | 5,80867905 |
| 2,981514609 | 5,80867905 |
| 2,981514609 | 5,80867905 |
| 1,550387597 | 2,998047165 |

| | |
|---|---|
| 1,68520391 | 3,25566162 |
| 1,68520391 | 3,25566162 |
| 1,794430089 | 3,466608554 |
| 2,236135957 | 4,268746712 |
| 2,236135957 | 4,268746712 |
| 2,236135957 | 4,268746712 |
| 2,236135957 | 4,268746712 |
| 2,236135957 | 4,268746712 |
| 2,236135957 | 4,268746712 |
| 1,296538923 | 2,463698256 |
| 1,937984496 | 3,68046246 |
| 1,937984496 | 3,68046246 |
| 1,937984496 | 3,68046246 |
| 1,400952648 | 2,653822098 |
| 1,661129568 | 3,129176589 |
| 1,661129568 | 3,129176589 |
| 1,661129568 | 3,129176589 |
| 1,42118863 | 2,67019564 |
| 1,761804087 | 3,306791541 |
| 1,761804087 | 3,306791541 |
| 1,325117604 | 2,479356535 |
| 1,446257087 | 2,69918108 |
| 1,51998784 | 2,820307726 |
| 1,2967926 | 2,400177373 |
| 1,637733377 | 3,008298221 |
| 1,637733377 | 3,008298221 |
| 1,890716582 | 3,470039912 |
| 2,153316107 | 3,936019159 |
| 2,153316107 | 3,936019159 |
| 2,153316107 | 3,936019159 |
| 2,153316107 | 3,936019159 |
| 2,153316107 | 3,936019159 |
| 2,153316107 | 3,936019159 |
| 2,76854928 | 5,058804027 |
| 2,76854928 | 5,058804027 |
| 2,76854928 | 5,058804027 |
| 2,76854928 | 5,058804027 |
| 2,76854928 | 5,058804027 |
| 2,76854928 | 5,058804027 |
| 2,76854928 | 5,058804027 |
| 2,76854928 | 5,058804027 |
| 2,76854928 | 5,058804027 |
| 2,76854928 | 5,058804027 |
| 2,76854928 | 5,058804027 |
| 2,76854928 | 5,058804027 |
| 2,76854928 | 5,058804027 |
| 2,76854928 | 5,058804027 |
| 2,76854928 | 5,058804027 |
| 2,76854928 | 5,058804027 |
| 2,76854928 | 5,058804027 |
| 2,76854928 | 5,058804027 |
| 2,76854928 | 5,058804027 |
| 1,7303433 | 3,155624087 |

| | |
|---|---|
| 1,7303433 | 3,155624087 |
| 1,55929787 | 2,838733042 |
| 1,61498708 | 2,892730146 |
| 1,61498708 | 2,892730146 |
| 1,541578576 | 2,743673332 |
| 1,84569952 | 3,274194731 |
| 1,84569952 | 3,274194731 |
| 1,32998936 | 2,355058598 |
| 2,07641196 | 3,636043216 |
| 2,07641196 | 3,636043216 |
| 2,07641196 | 3,636043216 |
| 2,07641196 | 3,636043216 |
| 2,07641196 | 3,636043216 |
| 2,07641196 | 3,636043216 |
| 1,592863969 | 2,782194858 |
| 1,404336591 | 2,439987384 |
| 1,196286726 | 2,065715109 |
| 1,67067629 | 2,877011543 |
| 1,67067629 | 2,877011543 |
| 2,583979328 | 4,437032068 |
| 2,583979328 | 4,437032068 |
| 2,583979328 | 4,437032068 |
| 2,583979328 | 4,437032068 |
| 2,583979328 | 4,437032068 |
| 2,583979328 | 4,437032068 |
| 2,583979328 | 4,437032068 |
| 2,583979328 | 4,437032068 |
| 2,583979328 | 4,437032068 |
| 2,583979328 | 4,437032068 |
| 2,583979328 | 4,437032068 |
| 2,583979328 | 4,437032068 |
| 2,583979328 | 4,437032068 |
| 2,583979328 | 4,437032068 |
| 2,583979328 | 4,437032068 |
| 2,583979328 | 4,437032068 |
| 2,583979328 | 4,437032068 |
| 2,583979328 | 4,437032068 |
| 2,583979328 | 4,437032068 |
| 2,583979328 | 4,437032068 |
| 2,583979328 | 4,437032068 |
| 2,583979328 | 4,437032068 |
| 2,583979328 | 4,437032068 |
| 2,583979328 | 4,437032068 |
| 1,802776275 | 3,091681929 |
| 1,802776275 | 3,091681929 |
| 1,802776275 | 3,091681929 |
| 1,802776275 | 3,091681929 |
| 1,39423345 | 2,379347858 |
| 1,571338781 | 2,676432288 |
| 1,571338781 | 2,676432288 |
| 1,507321275 | 2,563957584 |
| 1,310714152 | 2,221675742 |
| 2,004811548 | 3,364777581 |

| | |
|---|---|
| 2,004811548 | 3,364777581 |
| 2,004811548 | 3,364777581 |
| 2,004811548 | 3,364777581 |
| 2,004811548 | 3,364777581 |
| 2,004811548 | 3,364777581 |
| 2,004811548 | 3,364777581 |
| 2,004811548 | 3,364777581 |
| 2,004811548 | 3,364777581 |
| 2,004811548 | 3,364777581 |
| 1,357823532 | 2,267140325 |
| 1,490757305 | 2,479005637 |
| 1,550387597 | 2,575198513 |
| 1,550387597 | 2,575198513 |
| 1,13999088 | 1,892285846 |
| 1,761804087 | 2,921385574 |
| 1,761804087 | 2,921385574 |
| 1,761804087 | 2,921385574 |
| 1,761804087 | 2,921385574 |
| 1,761804087 | 2,921385574 |
| 1,761804087 | 2,921385574 |
| 1,40660165 | 2,329346317 |
| 1,474553421 | 2,397139599 |
| 1,52998776 | 2,478264567 |
| 2,42248062 | 3,915755568 |
| 2,42248062 | 3,915755568 |
| 2,42248062 | 3,915755568 |
| 2,42248062 | 3,915755568 |
| 2,42248062 | 3,915755568 |
| 2,42248062 | 3,915755568 |
| 2,42248062 | 3,915755568 |
| 2,42248062 | 3,915755568 |
| 2,42248062 | 3,915755568 |
| 2,42248062 | 3,915755568 |
| 2,42248062 | 3,915755568 |
| 2,42248062 | 3,915755568 |
| 2,42248062 | 3,915755568 |
| 2,42248062 | 3,915755568 |
| 2,42248062 | 3,915755568 |
| 2,42248062 | 3,915755568 |
| 2,42248062 | 3,915755568 |
| 2,42248062 | 3,915755568 |
| 1,21124031 | 1,957233548 |
| 1,937984496 | 3,118786362 |
| 1,937984496 | 3,118786362 |
| 1,937984496 | 3,118786362 |
| 1,937984496 | 3,118786362 |
| 1,937984496 | 3,118786362 |
| 1,937984496 | 3,118786362 |
| 1,937984496 | 3,118786362 |
| 1,937984496 | 3,118786362 |
| 1,937984496 | 3,118786362 |
| 1,937984496 | 3,118786362 |
| 1,722652885 | 2,762303 |

| | |
|---|---|
| 1,722652885 | 2,762303 |
| 1,722652885 | 2,762303 |
| 1,38427464 | 2,205733118 |
| 1,458698008 | 2,318229682 |
| 1,510117789 | 2,38541536 |
| 1,510117789 | 2,38541536 |
| 1,510117789 | 2,38541536 |
| 1,40944327 | 2,215586155 |
| 1,68520391 | 2,613531239 |
| 1,68520391 | 2,613531239 |
| 1,68520391 | 2,613531239 |
| 1,68520391 | 2,613531239 |
| 1,875468867 | 2,895132655 |
| 1,875468867 | 2,895132655 |
| 1,875468867 | 2,895132655 |
| 1,875468867 | 2,895132655 |
| 1,875468867 | 2,895132655 |
| 1,875468867 | 2,895132655 |
| 1,875468867 | 2,895132655 |
| 1,396745583 | 2,150406805 |
| 1,299207483 | 1,998044726 |
| 1,562890723 | 2,401392711 |
| 1,562890723 | 2,401392711 |
| 1,562890723 | 2,401392711 |
| 2,27998176 | 3,474499418 |
| 2,27998176 | 3,474499418 |
| 2,27998176 | 3,474499418 |
| 2,27998176 | 3,474499418 |
| 2,27998176 | 3,474499418 |
| 2,27998176 | 3,474499418 |
| 2,27998176 | 3,474499418 |
| 2,27998176 | 3,474499418 |
| 2,27998176 | 3,474499418 |
| 2,27998176 | 3,474499418 |
| 2,27998176 | 3,474499418 |
| 2,27998176 | 3,474499418 |
| 2,27998176 | 3,474499418 |
| 2,27998176 | 3,474499418 |
| 2,27998176 | 3,474499418 |
| 2,27998176 | 3,474499418 |
| 2,27998176 | 3,474499418 |
| 2,27998176 | 3,474499418 |
| 2,27998176 | 3,474499418 |
| 2,27998176 | 3,474499418 |
| 2,27998176 | 3,474499418 |
| 2,27998176 | 3,474499418 |
| 2,27998176 | 3,474499418 |
| 2,27998176 | 3,474499418 |
| 1,649348507 | 2,474255328 |
| 1,649348507 | 2,474255328 |
| 1,649348507 | 2,474255328 |
| 1,538082933 | 2,297125709 |

| | |
|---|---|
| 1,413113695 | 2,098033897 |
| 1,155336911 | 1,715051716 |
| 1,29986765 | 1,926142764 |
| 1,816860465 | 2,691293478 |
| 1,816860465 | 2,691293478 |
| 1,816860465 | 2,691293478 |
| 1,816860465 | 2,691293478 |
| 1,816860465 | 2,691293478 |
| 1,816860465 | 2,691293478 |
| 1,816860465 | 2,691293478 |
| 1,816860465 | 2,691293478 |
| 1,816860465 | 2,691293478 |
| 1,453488372 | 2,129413051 |
| 1,453488372 | 2,129413051 |
| 1,514050388 | 2,198020285 |
| 1,514050388 | 2,198020285 |
| 1,514050388 | 2,198020285 |
| 1,61498708 | 2,343738185 |
| 1,61498708 | 2,343738185 |
| 1,61498708 | 2,343738185 |
| 1,61498708 | 2,343738185 |
| 1,61498708 | 2,343738185 |
| 1,134579531 | 1,644391157 |
| 1,35998912 | 1,966831431 |
| 1,244577199 | 1,793255828 |
| 2,153316107 | 3,097797377 |
| 2,153316107 | 3,097797377 |
| 2,153316107 | 3,097797377 |
| 2,153316107 | 3,097797377 |
| 2,153316107 | 3,097797377 |
| 2,153316107 | 3,097797377 |
| 2,153316107 | 3,097797377 |
| 2,153316107 | 3,097797377 |
| 2,153316107 | 3,097797377 |
| 2,153316107 | 3,097797377 |
| 2,153316107 | 3,097797377 |
| 2,153316107 | 3,097797377 |
| 2,153316107 | 3,097797377 |
| 2,153316107 | 3,097797377 |
| 2,153316107 | 3,097797377 |
| 2,153316107 | 3,097797377 |
| 2,153316107 | 3,097797377 |
| 2,153316107 | 3,097797377 |
| 2,153316107 | 3,097797377 |
| 2,153316107 | 3,097797377 |
| 2,153316107 | 3,097797377 |
| 2,153316107 | 3,097797377 |
| 2,153316107 | 3,097797377 |
| 1,435544071 | 2,050996245 |
| 1,435544071 | 2,050996245 |
| 1,435544071 | 2,050996245 |
| 1,435544071 | 2,050996245 |
| 1,761804087 | 2,505091275 |

| | |
|---|---|
| 1,761804087 | 2,505091275 |
| 1,761804087 | 2,505091275 |
| 1,761804087 | 2,505091275 |
| 1,761804087 | 2,505091275 |
| 1,761804087 | 2,505091275 |
| 1,761804087 | 2,505091275 |
| 1,761804087 | 2,505091275 |
| 1,761804087 | 2,505091275 |
| 1,761804087 | 2,505091275 |
| 1,761804087 | 2,505091275 |
| 1,21124031 | 1,721963917 |
| 1,38427464 | 1,963913075 |
| 1,490757305 | 2,103772963 |
| 1,490757305 | 2,103772963 |
| 1,490757305 | 2,103772963 |
| 1,58202816 | 2,221311833 |
| 1,58202816 | 2,221311833 |
| 1,418037436 | 1,975764768 |
| 1,31141808 | 1,824518227 |
| 1,468170073 | 2,014101658 |
| 1,198026779 | 1,635928053 |
| 1,70998632 | 2,334638367 |
| 1,70998632 | 2,334638367 |
| 1,70998632 | 2,334638367 |
| 1,70998632 | 2,334638367 |
| 1,70998632 | 2,334638367 |
| 1,70998632 | 2,334638367 |
| 1,70998632 | 2,334638367 |
| 1,70998632 | 2,334638367 |
| 1,70998632 | 2,334638367 |
| 1,70998632 | 2,334638367 |
| 1,70998632 | 2,334638367 |
| 2,03998368 | 2,773776792 |
| 2,03998368 | 2,773776792 |
| 2,03998368 | 2,773776792 |
| 2,03998368 | 2,773776792 |
| 2,03998368 | 2,773776792 |
| 2,03998368 | 2,773776792 |
| 2,03998368 | 2,773776792 |
| 2,03998368 | 2,773776792 |
| 2,03998368 | 2,773776792 |
| 2,03998368 | 2,773776792 |
| 2,03998368 | 2,773776792 |
| 2,03998368 | 2,773776792 |
| 2,03998368 | 2,773776792 |
| 2,03998368 | 2,773776792 |
| 2,03998368 | 2,773776792 |
| 2,03998368 | 2,773776792 |
| 2,03998368 | 2,773776792 |
| 2,03998368 | 2,773776792 |
| 2,03998368 | 2,773776792 |
| 2,03998368 | 2,773776792 |

| | |
|---|---|
| 2,03998368 | 2,773776792 |
| 1,400952648 | 1,903568527 |
| 1,400952648 | 1,903568527 |
| 1,550387597 | 2,106369756 |
| 1,356589147 | 1,839001571 |
| 1,38427464 | 1,834265832 |
| 1,51998784 | 1,998360232 |
| 1,51998784 | 1,998360232 |
| 1,51998784 | 1,998360232 |
| 1,51998784 | 1,998360232 |
| 1,246656693 | 1,636939333 |
| 1,661129568 | 2,178291625 |
| 1,661129568 | 2,178291625 |
| 1,661129568 | 2,178291625 |
| 1,661129568 | 2,178291625 |
| 1,661129568 | 2,178291625 |
| 3,22997416 | 4,201306255 |
| 3,22997416 | 4,201306255 |
| 3,22997416 | 4,201306255 |
| 3,22997416 | 4,201306255 |
| 3,22997416 | 4,201306255 |
| 3,22997416 | 4,201306255 |
| 3,22997416 | 4,201306255 |
| 3,22997416 | 4,201306255 |
| 3,22997416 | 4,201306255 |
| 3,22997416 | 4,201306255 |
| 3,22997416 | 4,201306255 |
| 3,22997416 | 4,201306255 |
| 3,22997416 | 4,201306255 |
| 3,22997416 | 4,201306255 |
| 3,22997416 | 4,201306255 |
| 3,22997416 | 4,201306255 |
| 3,22997416 | 4,201306255 |
| 3,22997416 | 4,201306255 |
| 3,22997416 | 4,201306255 |
| 3,22997416 | 4,201306255 |
| 3,22997416 | 4,201306255 |
| 3,22997416 | 4,201306255 |
| 3,22997416 | 4,201306255 |
| 3,22997416 | 4,201306255 |
| 3,22997416 | 4,201306255 |
| 3,22997416 | 4,201306255 |
| 3,22997416 | 4,201306255 |
| 3,22997416 | 4,201306255 |
| 3,22997416 | 4,201306255 |
| 3,22997416 | 4,201306255 |
| 3,22997416 | 4,201306255 |
| 3,22997416 | 4,201306255 |
| 3,22997416 | 4,201306255 |
| 3,22997416 | 4,201306255 |
| 3,22997416 | 4,201306255 |
| 3,22997416 | 4,201306255 |
| 3,22997416 | 4,201306255 |

| | |
|---|---|
| 3,22997416 | 4,201306255 |
| 3,22997416 | 4,201306255 |
| 3,22997416 | 4,201306255 |
| 3,22997416 | 4,201306255 |
| 3,22997416 | 4,201306255 |
| 3,22997416 | 4,201306255 |
| 3,22997416 | 4,201306255 |
| 3,22997416 | 4,201306255 |
| 3,22997416 | 4,201306255 |
| 3,22997416 | 4,201306255 |
| 3,22997416 | 4,201306255 |
| 3,22997416 | 4,201306255 |
| 3,22997416 | 4,201306255 |
| 3,22997416 | 4,201306255 |
| 3,22997416 | 4,201306255 |
| 3,22997416 | 4,201306255 |
| 3,22997416 | 4,201306255 |
| 3,22997416 | 4,201306255 |
| 3,22997416 | 4,201306255 |
| 3,22997416 | 4,201306255 |
| 3,22997416 | 4,201306255 |
| 3,22997416 | 4,201306255 |
| 3,22997416 | 4,201306255 |
| 3,22997416 | 4,201306255 |
| 3,22997416 | 4,201306255 |
| 3,22997416 | 4,201306255 |
| 3,22997416 | 4,201306255 |
| 3,22997416 | 4,201306255 |
| 3,22997416 | 4,201306255 |
| 3,22997416 | 4,201306255 |
| 3,22997416 | 4,201306255 |
| 3,22997416 | 4,201306255 |
| 3,22997416 | 4,201306255 |
| 3,22997416 | 4,201306255 |
| 3,22997416 | 4,201306255 |
| 3,22997416 | 4,201306255 |
| 3,22997416 | 4,201306255 |
| 3,22997416 | 4,201306255 |
| 3,22997416 | 4,201306255 |
| 3,22997416 | 4,201306255 |
| 3,22997416 | 4,201306255 |
| 3,22997416 | 4,201306255 |
| 3,22997416 | 4,201306255 |
| 3,22997416 | 4,201306255 |
| 3,22997416 | 4,201306255 |
| 3,22997416 | 4,201306255 |
| 3,22997416 | 4,201306255 |
| 3,22997416 | 4,201306255 |
| 3,22997416 | 4,201306255 |
| 3,22997416 | 4,201306255 |
| 3,22997416 | 4,201306255 |
| 3,22997416 | 4,201306255 |
| 3,22997416 | 4,201306255 |

| | |
|---|---|
| 3,22997416 | 4,201306255 |
| 3,22997416 | 4,201306255 |
| 3,22997416 | 4,201306255 |
| 3,22997416 | 4,201306255 |
| 3,22997416 | 4,201306255 |
| 3,22997416 | 4,201306255 |
| 3,22997416 | 4,201306255 |
| 3,22997416 | 4,201306255 |
| 3,22997416 | 4,201306255 |
| 3,22997416 | 4,201306255 |
| 3,22997416 | 4,201306255 |
| 3,22997416 | 4,201306255 |
| 3,22997416 | 4,201306255 |
| 3,22997416 | 4,201306255 |
| 3,22997416 | 4,201306255 |
| 3,22997416 | 4,201306255 |
| 3,22997416 | 4,201306255 |
| 3,22997416 | 4,201306255 |
| 3,22997416 | 4,201306255 |
| 3,22997416 | 4,201306255 |
| 3,22997416 | 4,201306255 |
| 3,22997416 | 4,201306255 |
| 3,22997416 | 4,201306255 |
| 3,22997416 | 4,201306255 |
| 3,22997416 | 4,201306255 |
| 3,22997416 | 4,201306255 |
| 3,22997416 | 4,201306255 |
| 3,22997416 | 4,201306255 |
| 1,4249886 | 1,847455232 |
| 1,4249886 | 1,847455232 |
| 1,180850768 | 1,527512742 |
| 1,937984496 | 2,493192356 |
| 1,937984496 | 2,493192356 |
| 1,937984496 | 2,493192356 |
| 1,937984496 | 2,493192356 |
| 1,937984496 | 2,493192356 |
| 1,937984496 | 2,493192356 |
| 1,937984496 | 2,493192356 |
| 1,937984496 | 2,493192356 |
| 1,937984496 | 2,493192356 |
| 1,937984496 | 2,493192356 |
| 1,937984496 | 2,493192356 |
| 1,937984496 | 2,493192356 |
| 1,937984496 | 2,493192356 |
| 1,937984496 | 2,493192356 |
| 1,937984496 | 2,493192356 |
| 1,937984496 | 2,493192356 |
| 1,937984496 | 2,493192356 |
| 1,937984496 | 2,493192356 |
| 1,937984496 | 2,493192356 |
| 1,937984496 | 2,493192356 |
| 1,937984496 | 2,493192356 |
| 1,937984496 | 2,493192356 |

| | |
|---|---|
| 1,183501982 | 1,509690918 |
| 1,404336591 | 1,770007728 |
| 1,352082207 | 1,703813049 |
| 1,352082207 | 1,703813049 |
| 1,61498708 | 2,034615246 |
| 1,61498708 | 2,034615246 |
| 1,61498708 | 2,034615246 |
| 1,61498708 | 2,034615246 |
| 1,61498708 | 2,034615246 |
| 1,61498708 | 2,034615246 |
| 1,61498708 | 2,034615246 |
| 1,61498708 | 2,034615246 |
| 1,61498708 | 2,034615246 |
| 1,61498708 | 2,034615246 |
| 1,21124031 | 1,5045864 |
| 1,174536058 | 1,456524825 |
| 1,304412642 | 1,614008827 |
| 1,462629808 | 1,801171619 |
| 1,462629808 | 1,801171619 |
| 1,462629808 | 1,801171619 |
| 1,336541032 | 1,642416805 |
| 1,38427464 | 1,696188701 |
| 1,38427464 | 1,696188701 |
| 1,84569952 | 2,248752181 |
| 1,84569952 | 2,248752181 |
| 1,84569952 | 2,248752181 |
| 1,84569952 | 2,248752181 |
| 1,84569952 | 2,248752181 |
| 1,84569952 | 2,248752181 |
| 1,84569952 | 2,248752181 |
| 1,84569952 | 2,248752181 |
| 1,84569952 | 2,248752181 |
| 1,84569952 | 2,248752181 |
| 1,84569952 | 2,248752181 |
| 1,84569952 | 2,248752181 |
| 1,84569952 | 2,248752181 |
| 1,84569952 | 2,248752181 |
| 1,84569952 | 2,248752181 |
| 1,84569952 | 2,248752181 |
| 1,84569952 | 2,248752181 |
| 1,84569952 | 2,248752181 |
| 1,84569952 | 2,248752181 |
| 1,84569952 | 2,248752181 |
| 1,84569952 | 2,248752181 |
| 1,84569952 | 2,248752181 |
| 1,84569952 | 2,248752181 |
| 1,571338781 | 1,902350018 |
| 1,571338781 | 1,902350018 |
| 1,571338781 | 1,902350018 |
| 1,571338781 | 1,902350018 |
| 1,571338781 | 1,902350018 |
| 1,571338781 | 1,902350018 |
| 1,571338781 | 1,902350018 |

| | |
|---|---|
| 1,21124031 | 1,457386838 |
| 1,260477721 | 1,508799043 |
| 1,364777814 | 1,62579949 |
| 1,364777814 | 1,62579949 |
| 1,364777814 | 1,62579949 |
| 2,76854928 | 3,243431827 |
| 2,76854928 | 3,243431827 |
| 2,76854928 | 3,243431827 |
| 2,76854928 | 3,243431827 |
| 2,76854928 | 3,243431827 |
| 2,76854928 | 3,243431827 |
| 2,76854928 | 3,243431827 |
| 2,76854928 | 3,243431827 |
| 2,76854928 | 3,243431827 |
| 2,76854928 | 3,243431827 |
| 2,76854928 | 3,243431827 |
| 2,76854928 | 3,243431827 |
| 2,76854928 | 3,243431827 |
| 2,76854928 | 3,243431827 |
| 2,76854928 | 3,243431827 |
| 2,76854928 | 3,243431827 |
| 2,76854928 | 3,243431827 |
| 2,76854928 | 3,243431827 |
| 2,76854928 | 3,243431827 |
| 2,76854928 | 3,243431827 |
| 2,76854928 | 3,243431827 |
| 2,76854928 | 3,243431827 |
| 2,76854928 | 3,243431827 |
| 2,76854928 | 3,243431827 |
| 2,76854928 | 3,243431827 |
| 2,76854928 | 3,243431827 |
| 2,76854928 | 3,243431827 |
| 2,76854928 | 3,243431827 |
| 2,76854928 | 3,243431827 |
| 2,76854928 | 3,243431827 |
| 2,76854928 | 3,243431827 |
| 2,76854928 | 3,243431827 |
| 2,76854928 | 3,243431827 |
| 2,76854928 | 3,243431827 |
| 2,76854928 | 3,243431827 |
| 2,76854928 | 3,243431827 |
| 2,76854928 | 3,243431827 |
| 2,76854928 | 3,243431827 |
| 2,76854928 | 3,243431827 |
| 2,76854928 | 3,243431827 |
| 2,76854928 | 3,243431827 |
| 2,76854928 | 3,243431827 |
| 2,76854928 | 3,243431827 |
| 2,76854928 | 3,243431827 |
| 2,76854928 | 3,243431827 |
| 2,76854928 | 3,243431827 |
| 2,76854928 | 3,243431827 |
| 2,76854928 | 3,243431827 |

| | |
|---|---|
| 2,76854928 | 3,243431827 |
| 2,76854928 | 3,243431827 |
| 2,76854928 | 3,243431827 |
| 2,76854928 | 3,243431827 |
| 2,76854928 | 3,243431827 |
| 2,76854928 | 3,243431827 |
| 2,76854928 | 3,243431827 |
| 2,76854928 | 3,243431827 |
| 2,76854928 | 3,243431827 |
| 2,76854928 | 3,243431827 |
| 2,76854928 | 3,243431827 |
| 2,76854928 | 3,243431827 |
| 2,76854928 | 3,243431827 |
| 2,76854928 | 3,243431827 |
| 2,76854928 | 3,243431827 |
| 2,76854928 | 3,243431827 |
| 2,76854928 | 3,243431827 |
| 2,76854928 | 3,243431827 |
| 2,76854928 | 3,243431827 |
| 2,76854928 | 3,243431827 |
| 2,76854928 | 3,243431827 |
| 2,76854928 | 3,243431827 |
| 2,76854928 | 3,243431827 |
| 2,76854928 | 3,243431827 |
| 2,76854928 | 3,243431827 |
| 2,76854928 | 3,243431827 |
| 2,76854928 | 3,243431827 |
| 2,76854928 | 3,243431827 |
| 2,76854928 | 3,243431827 |
| 2,76854928 | 3,243431827 |
| 2,76854928 | 3,243431827 |
| 2,76854928 | 3,243431827 |
| 2,76854928 | 3,243431827 |
| 2,76854928 | 3,243431827 |
| 2,76854928 | 3,243431827 |
| 2,76854928 | 3,243431827 |
| 2,76854928 | 3,243431827 |
| 2,76854928 | 3,243431827 |
| 2,76854928 | 3,243431827 |
| 2,76854928 | 3,243431827 |
| 2,76854928 | 3,243431827 |
| 2,76854928 | 3,243431827 |
| 2,76854928 | 3,243431827 |
| 2,76854928 | 3,243431827 |
| 2,76854928 | 3,243431827 |
| 2,76854928 | 3,243431827 |
| 2,76854928 | 3,243431827 |
| 2,76854928 | 3,243431827 |
| 2,76854928 | 3,243431827 |
| 2,76854928 | 3,243431827 |
| 2,76854928 | 3,243431827 |
| 2,76854928 | 3,243431827 |
| 2,76854928 | 3,243431827 |

| | |
|---|---|
| 2,76854928 | 3,243431827 |
| 2,76854928 | 3,243431827 |
| 2,76854928 | 3,243431827 |
| 2,76854928 | 3,243431827 |
| 2,76854928 | 3,243431827 |
| 2,76854928 | 3,243431827 |
| 2,76854928 | 3,243431827 |
| 2,76854928 | 3,243431827 |
| 2,76854928 | 3,243431827 |
| 2,76854928 | 3,243431827 |
| 2,76854928 | 3,243431827 |
| 2,76854928 | 3,243431827 |
| 2,76854928 | 3,243431827 |
| 2,76854928 | 3,243431827 |
| 2,76854928 | 3,243431827 |
| 2,76854928 | 3,243431827 |
| 1,306506402 | 1,526718088 |
| 1,306506402 | 1,526718088 |
| 1,52998776 | 1,780387798 |
| 1,52998776 | 1,780387798 |
| 1,52998776 | 1,780387798 |
| 1,52998776 | 1,780387798 |
| 1,52998776 | 1,780387798 |
| 1,52998776 | 1,780387798 |
| 1,52998776 | 1,780387798 |
| 1,52998776 | 1,780387798 |
| 1,197630868 | 1,391178343 |
| 1,345822567 | 1,558654372 |
| 1,267840325 | 1,464570245 |
| 1,761804087 | 2,034638719 |
| 1,761804087 | 2,034638719 |
| 1,761804087 | 2,034638719 |
| 1,761804087 | 2,034638719 |
| 1,761804087 | 2,034638719 |
| 1,761804087 | 2,034638719 |
| 1,761804087 | 2,034638719 |
| 1,761804087 | 2,034638719 |
| 1,761804087 | 2,034638719 |
| 1,761804087 | 2,034638719 |
| 1,761804087 | 2,034638719 |
| 1,761804087 | 2,034638719 |
| 1,761804087 | 2,034638719 |
| 1,761804087 | 2,034638719 |
| 1,761804087 | 2,034638719 |
| 1,761804087 | 2,034638719 |
| 1,761804087 | 2,034638719 |
| 1,761804087 | 2,034638719 |
| 1,761804087 | 2,034638719 |
| 1,761804087 | 2,034638719 |
| 1,40944327 | 1,62622342 |
| 1,40944327 | 1,62622342 |
| 1,291989664 | 1,472206841 |
| 1,256101062 | 1,418069809 |

| | |
|---|---|
| 1,256101062 | 1,418069809 |
| 1,327386641 | 1,49457957 |
| 1,230466347 | 1,382122861 |
| 1,196286726 | 1,340111462 |
| 1,490757305 | 1,667750218 |
| 1,68520391 | 1,846162327 |
| 1,68520391 | 1,846162327 |
| 1,68520391 | 1,846162327 |
| 1,68520391 | 1,846162327 |
| 1,68520391 | 1,846162327 |
| 1,68520391 | 1,846162327 |
| 1,68520391 | 1,846162327 |
| 1,68520391 | 1,846162327 |
| 1,68520391 | 1,846162327 |
| 1,68520391 | 1,846162327 |
| 1,68520391 | 1,846162327 |
| 1,68520391 | 1,846162327 |
| 1,68520391 | 1,846162327 |
| 1,68520391 | 1,846162327 |
| 1,68520391 | 1,846162327 |
| 1,68520391 | 1,846162327 |
| 1,68520391 | 1,846162327 |
| 1,309448984 | 1,433412345 |
| 1,263902932 | 1,369378871 |
| 1,171309311 | 1,267560931 |
| 1,35998912 | 1,470562551 |
| 1,35998912 | 1,470562551 |
| 1,181697863 | 1,275096572 |
| 1,181697863 | 1,275096572 |
| 1,233262861 | 1,32967138 |
| 1,194647977 | 1,286603041 |
| 1,21124031 | 1,303824891 |
| 1,453488372 | 1,563570827 |
| 1,453488372 | 1,563570827 |
| 1,453488372 | 1,563570827 |
| 1,453488372 | 1,563570827 |
| 1,291989664 | 1,375000179 |
| 1,291989664 | 1,375000179 |
| 1,291989664 | 1,375000179 |
| 2,42248062 | 2,574415889 |
| 2,42248062 | 2,574415889 |
| 2,42248062 | 2,574415889 |
| 2,42248062 | 2,574415889 |
| 2,42248062 | 2,574415889 |
| 2,42248062 | 2,574415889 |
| 2,42248062 | 2,574415889 |
| 2,42248062 | 2,574415889 |
| 2,42248062 | 2,574415889 |
| 2,42248062 | 2,574415889 |
| 2,42248062 | 2,574415889 |
| 2,42248062 | 2,574415889 |
| 2,42248062 | 2,574415889 |
| 2,42248062 | 2,574415889 |

| | |
|---|---|
| 2,42248062 | 2,574415889 |
| 2,42248062 | 2,574415889 |
| 2,42248062 | 2,574415889 |
| 2,42248062 | 2,574415889 |
| 2,42248062 | 2,574415889 |
| 2,42248062 | 2,574415889 |
| 2,42248062 | 2,574415889 |
| 2,42248062 | 2,574415889 |
| 2,42248062 | 2,574415889 |
| 2,42248062 | 2,574415889 |
| 2,42248062 | 2,574415889 |
| 2,42248062 | 2,574415889 |
| 2,42248062 | 2,574415889 |
| 2,42248062 | 2,574415889 |
| 2,42248062 | 2,574415889 |
| 2,42248062 | 2,574415889 |
| 2,42248062 | 2,574415889 |
| 2,42248062 | 2,574415889 |
| 2,42248062 | 2,574415889 |
| 2,42248062 | 2,574415889 |
| 2,42248062 | 2,574415889 |
| 2,42248062 | 2,574415889 |
| 2,42248062 | 2,574415889 |
| 2,42248062 | 2,574415889 |
| 2,42248062 | 2,574415889 |
| 2,42248062 | 2,574415889 |
| 2,42248062 | 2,574415889 |
| 2,42248062 | 2,574415889 |
| 2,42248062 | 2,574415889 |
| 2,42248062 | 2,574415889 |
| 2,42248062 | 2,574415889 |
| 2,42248062 | 2,574415889 |
| 2,42248062 | 2,574415889 |
| 2,42248062 | 2,574415889 |
| 2,42248062 | 2,574415889 |
| 2,42248062 | 2,574415889 |
| 2,42248062 | 2,574415889 |
| 2,42248062 | 2,574415889 |
| 2,42248062 | 2,574415889 |
| 2,42248062 | 2,574415889 |
| 2,42248062 | 2,574415889 |
| 2,42248062 | 2,574415889 |
| 2,42248062 | 2,574415889 |
| 2,42248062 | 2,574415889 |
| 2,42248062 | 2,574415889 |
| 2,42248062 | 2,574415889 |
| 2,42248062 | 2,574415889 |
| 2,42248062 | 2,574415889 |
| 2,42248062 | 2,574415889 |
| 2,42248062 | 2,574415889 |
| 2,42248062 | 2,574415889 |
| 2,42248062 | 2,574415889 |
| 2,42248062 | 2,574415889 |

| | |
|---|---|
| 2,42248062 | 2,574415889 |
| 2,42248062 | 2,574415889 |
| 2,42248062 | 2,574415889 |
| 2,42248062 | 2,574415889 |
| 2,42248062 | 2,574415889 |
| 2,42248062 | 2,574415889 |
| 2,42248062 | 2,574415889 |
| 2,42248062 | 2,574415889 |
| 2,42248062 | 2,574415889 |
| 2,42248062 | 2,574415889 |
| 2,42248062 | 2,574415889 |
| 2,42248062 | 2,574415889 |
| 2,42248062 | 2,574415889 |
| 2,42248062 | 2,574415889 |
| 2,42248062 | 2,574415889 |
| 1,222152385 | 1,287660508 |
| 1,151277918 | 1,208653131 |
| 1,336541032 | 1,399170972 |
| 1,336541032 | 1,399170972 |
| 1,61498708 | 1,679506874 |
| 1,2749898 | 1,319200012 |
| 1,2749898 | 1,319200012 |
| 1,2749898 | 1,319200012 |
| 1,418037436 | 1,467080051 |
| 1,418037436 | 1,467080051 |
| 1,418037436 | 1,467080051 |
| 1,418037436 | 1,467080051 |
| 1,418037436 | 1,467080051 |
| 1,21124031 | 1,247037298 |
| 1,134855786 | 1,159675058 |
| 1,313887794 | 1,33169862 |
| 1,313887794 | 1,33169862 |
| 1,313887794 | 1,33169862 |
| 1,089820132 | 1,097356253 |
| 1,223990208 | 1,229321937 |
| 1,1535622 | 1,154347354 |
| 1,38427464 | 1,377592472 |
| 1,38427464 | 1,377592472 |
| 1,38427464 | 1,377592472 |
| 1,162790698 | 1,155985164 |
| 1,550387597 | 1,531540293 |
| 1,550387597 | 1,531540293 |
| 1,550387597 | 1,531540293 |
| 1,550387597 | 1,531540293 |
| 1,550387597 | 1,531540293 |
| 1,550387597 | 1,531540293 |
| 1,550387597 | 1,531540293 |
| 1,550387597 | 1,531540293 |
| 1,550387597 | 1,531540293 |
| 1,550387597 | 1,531540293 |
| 1,550387597 | 1,531540293 |
| 1,550387597 | 1,531540293 |
| 1,550387597 | 1,531540293 |

| | |
|---|---|
| 1,550387597 | 1,531540293 |
| 1,550387597 | 1,531540293 |
| 1,550387597 | 1,531540293 |
| 1,550387597 | 1,531540293 |
| 2,153316107 | 2,087484578 |
| 2,153316107 | 2,087484578 |
| 2,153316107 | 2,087484578 |
| 2,153316107 | 2,087484578 |
| 2,153316107 | 2,087484578 |
| 2,153316107 | 2,087484578 |
| 2,153316107 | 2,087484578 |
| 2,153316107 | 2,087484578 |
| 2,153316107 | 2,087484578 |
| 2,153316107 | 2,087484578 |
| 2,153316107 | 2,087484578 |
| 2,153316107 | 2,087484578 |
| 2,153316107 | 2,087484578 |
| 2,153316107 | 2,087484578 |
| 2,153316107 | 2,087484578 |
| 2,153316107 | 2,087484578 |
| 2,153316107 | 2,087484578 |
| 2,153316107 | 2,087484578 |
| 2,153316107 | 2,087484578 |
| 2,153316107 | 2,087484578 |
| 2,153316107 | 2,087484578 |
| 2,153316107 | 2,087484578 |
| 2,153316107 | 2,087484578 |
| 2,153316107 | 2,087484578 |
| 2,153316107 | 2,087484578 |
| 2,153316107 | 2,087484578 |
| 2,153316107 | 2,087484578 |
| 2,153316107 | 2,087484578 |
| 2,153316107 | 2,087484578 |
| 2,153316107 | 2,087484578 |
| 2,153316107 | 2,087484578 |
| 2,153316107 | 2,087484578 |
| 2,153316107 | 2,087484578 |
| 2,153316107 | 2,087484578 |
| 2,153316107 | 2,087484578 |
| 2,153316107 | 2,087484578 |
| 2,153316107 | 2,087484578 |
| 2,153316107 | 2,087484578 |
| 2,153316107 | 2,087484578 |
| 2,153316107 | 2,087484578 |
| 2,153316107 | 2,087484578 |
| 2,153316107 | 2,087484578 |
| 2,153316107 | 2,087484578 |
| 2,153316107 | 2,087484578 |
| 2,153316107 | 2,087484578 |
| 2,153316107 | 2,087484578 |
| 2,153316107 | 2,087484578 |
| 2,153316107 | 2,087484578 |
| 2,153316107 | 2,087484578 |

| | |
|---|---|
| 2,153316107 | 2,087484578 |
| 2,153316107 | 2,087484578 |
| 2,153316107 | 2,087484578 |
| 2,153316107 | 2,087484578 |
| 2,153316107 | 2,087484578 |
| 2,153316107 | 2,087484578 |
| 2,153316107 | 2,087484578 |
| 2,153316107 | 2,087484578 |
| 2,153316107 | 2,087484578 |
| 2,153316107 | 2,087484578 |
| 2,153316107 | 2,087484578 |
| 2,153316107 | 2,087484578 |
| 2,153316107 | 2,087484578 |
| 2,153316107 | 2,087484578 |
| 2,153316107 | 2,087484578 |
| 2,153316107 | 2,087484578 |
| 2,153316107 | 2,087484578 |
| 2,153316107 | 2,087484578 |
| 2,153316107 | 2,087484578 |
| 2,153316107 | 2,087484578 |
| 2,153316107 | 2,087484578 |
| 2,153316107 | 2,087484578 |
| 2,153316107 | 2,087484578 |
| 2,153316107 | 2,087484578 |
| 2,153316107 | 2,087484578 |
| 2,153316107 | 2,087484578 |
| 2,153316107 | 2,087484578 |
| 2,153316107 | 2,087484578 |
| 2,153316107 | 2,087484578 |
| 2,153316107 | 2,087484578 |
| 2,153316107 | 2,087484578 |
| 2,153316107 | 2,087484578 |
| 2,153316107 | 2,087484578 |
| 2,153316107 | 2,087484578 |
| 2,153316107 | 2,087484578 |
| 2,153316107 | 2,087484578 |
| 2,153316107 | 2,087484578 |
| 2,153316107 | 2,087484578 |
| 2,153316107 | 2,087484578 |
| 2,153316107 | 2,087484578 |
| 2,153316107 | 2,087484578 |
| 2,153316107 | 2,087484578 |
| 2,153316107 | 2,087484578 |
| 2,153316107 | 2,087484578 |
| 2,153316107 | 2,087484578 |
| 1,352082207 | 1,294496013 |
| 1,198753297 | 1,144494099 |
| 1,127927485 | 1,076703631 |
| 1,226572466 | 1,166168737 |
| 1,226572466 | 1,166168737 |
| 1,082672903 | 1,020347958 |
| 1,490757305 | 1,399671698 |
| 1,490757305 | 1,399671698 |

| | |
|---|---|
| 1,490757305 | 1,399671698 |
| 1,490757305 | 1,399671698 |
| 1,490757305 | 1,399671698 |
| 1,490757305 | 1,399671698 |
| 1,490757305 | 1,399671698 |
| 1,490757305 | 1,399671698 |
| 1,490757305 | 1,399671698 |
| 1,490757305 | 1,399671698 |
| 1,490757305 | 1,399671698 |
| 1,490757305 | 1,399671698 |
| 1,490757305 | 1,399671698 |
| 1,490757305 | 1,399671698 |
| 1,490757305 | 1,399671698 |
| 1,490757305 | 1,399671698 |
| 1,490757305 | 1,399671698 |
| 1,13999088 | 1,066849362 |
| 1,18652112 | 1,104431181 |
| 1,18652112 | 1,104431181 |
| 1,148435257 | 1,063834406 |
| 1,321353066 | 1,217242729 |
| 1,321353066 | 1,217242729 |
| 1,321353066 | 1,217242729 |
| 1,321353066 | 1,217242729 |
| 1,321353066 | 1,217242729 |
| 1,321353066 | 1,217242729 |
| 1,321353066 | 1,217242729 |
| 1,116116726 | 1,027544153 |
| 1,250312578 | 1,150364279 |
| 1,250312578 | 1,150364279 |
| 1,250312578 | 1,150364279 |
| 1,250312578 | 1,150364279 |
| 1,159477904 | 1,063037947 |
| 1,174536058 | 1,065849316 |
| 1,13999088 | 1,033432816 |
| 1,196286726 | 1,074952109 |
| 1,051866585 | 0,945083926 |
| 1,14965182 | 1,029749353 |
| 1,095382541 | 0,980972052 |
| 1,435544071 | 1,281742365 |
| 1,435544071 | 1,281742365 |
| 1,435544071 | 1,281742365 |
| 1,435544071 | 1,281742365 |
| 1,435544071 | 1,281742365 |
| 1,435544071 | 1,281742365 |
| 1,435544071 | 1,281742365 |
| 1,435544071 | 1,281742365 |
| 1,435544071 | 1,281742365 |
| 1,230466347 | 1,096226325 |
| 1,230466347 | 1,096226325 |
| 1,230466347 | 1,096226325 |
| 1,937984496 | 1,721568072 |
| 1,937984496 | 1,721568072 |
| 1,937984496 | 1,721568072 |

| | |
|---|---|
| 1,937984496 | 1,721568072 |
| 1,937984496 | 1,721568072 |
| 1,937984496 | 1,721568072 |
| 1,937984496 | 1,721568072 |
| 1,937984496 | 1,721568072 |
| 1,937984496 | 1,721568072 |
| 1,937984496 | 1,721568072 |
| 1,937984496 | 1,721568072 |
| 1,937984496 | 1,721568072 |
| 1,937984496 | 1,721568072 |
| 1,937984496 | 1,721568072 |
| 1,937984496 | 1,721568072 |
| 1,937984496 | 1,721568072 |
| 1,937984496 | 1,721568072 |
| 1,937984496 | 1,721568072 |
| 1,937984496 | 1,721568072 |
| 1,937984496 | 1,721568072 |
| 1,937984496 | 1,721568072 |
| 1,937984496 | 1,721568072 |
| 1,937984496 | 1,721568072 |
| 1,937984496 | 1,721568072 |
| 1,937984496 | 1,721568072 |
| 1,937984496 | 1,721568072 |
| 1,937984496 | 1,721568072 |
| 1,937984496 | 1,721568072 |
| 1,937984496 | 1,721568072 |
| 1,937984496 | 1,721568072 |
| 1,937984496 | 1,721568072 |
| 1,937984496 | 1,721568072 |
| 1,937984496 | 1,721568072 |
| 1,937984496 | 1,721568072 |
| 1,937984496 | 1,721568072 |
| 1,937984496 | 1,721568072 |
| 1,937984496 | 1,721568072 |
| 1,937984496 | 1,721568072 |
| 1,937984496 | 1,721568072 |
| 1,937984496 | 1,721568072 |
| 1,937984496 | 1,721568072 |
| 1,937984496 | 1,721568072 |
| 1,937984496 | 1,721568072 |
| 1,937984496 | 1,721568072 |
| 1,937984496 | 1,721568072 |
| 1,937984496 | 1,721568072 |
| 1,937984496 | 1,721568072 |
| 1,937984496 | 1,721568072 |
| 1,937984496 | 1,721568072 |
| 1,937984496 | 1,721568072 |
| 1,937984496 | 1,721568072 |
| 1,937984496 | 1,721568072 |
| 1,937984496 | 1,721568072 |
| 1,937984496 | 1,721568072 |
| 1,937984496 | 1,721568072 |
| 1,937984496 | 1,721568072 |

| | |
|---|---|
| 1,937984496 | 1,721568072 |
| 1,937984496 | 1,721568072 |
| 1,937984496 | 1,721568072 |
| 1,937984496 | 1,721568072 |
| 1,937984496 | 1,721568072 |
| 1,937984496 | 1,721568072 |
| 1,937984496 | 1,721568072 |
| 1,937984496 | 1,721568072 |
| 1,937984496 | 1,721568072 |
| 1,937984496 | 1,721568072 |
| 1,937984496 | 1,721568072 |
| 1,937984496 | 1,721568072 |
| 1,937984496 | 1,721568072 |
| 1,937984496 | 1,721568072 |
| 1,937984496 | 1,721568072 |
| 1,937984496 | 1,721568072 |
| 1,937984496 | 1,721568072 |
| 1,937984496 | 1,721568072 |
| 1,937984496 | 1,721568072 |
| 1,937984496 | 1,721568072 |
| 1,937984496 | 1,721568072 |
| 1,937984496 | 1,721568072 |
| 1,937984496 | 1,721568072 |
| 1,937984496 | 1,721568072 |
| 1,937984496 | 1,721568072 |
| 1,937984496 | 1,721568072 |
| 1,937984496 | 1,721568072 |
| 1,937984496 | 1,721568072 |
| 1,937984496 | 1,721568072 |
| 1,937984496 | 1,721568072 |
| 1,937984496 | 1,721568072 |
| 1,291989664 | 1,145340919 |
| 1,107419712 | 0,969676434 |
| 1,21124031 | 1,044920976 |
| 1,21124031 | 1,044920976 |
| 1,21124031 | 1,044920976 |
| 1,263902932 | 1,078348343 |
| 1,263902932 | 1,078348343 |
| 1,38427464 | 1,17594164 |
| 1,38427464 | 1,17594164 |
| 1,38427464 | 1,17594164 |
| 1,38427464 | 1,17594164 |
| 1,38427464 | 1,17594164 |
| 1,38427464 | 1,17594164 |
| 1,38427464 | 1,17594164 |
| 1,38427464 | 1,17594164 |
| 1,38427464 | 1,17594164 |
| 1,38427464 | 1,17594164 |
| 1,38427464 | 1,17594164 |
| 1,38427464 | 1,17594164 |
| 1,38427464 | 1,17594164 |
| 1,38427464 | 1,17594164 |
| 1,38427464 | 1,17594164 |

| | |
|---|---|
| 1,38427464 | 1,17594164 |
| 1,11538676 | 0,947405895 |
| 1,13999088 | 0,958395979 |
| 1,13999088 | 0,958395979 |
| 1,076658053 | 0,901784291 |
| 1,058094984 | 0,884430916 |
| 1,192605844 | 0,996276626 |
| 1,121148056 | 0,936183222 |
| 1,076658053 | 0,887327762 |
| 1,237011381 | 1,015866394 |
| 1,237011381 | 1,015866394 |
| 1,237011381 | 1,015866394 |
| 1,761804087 | 1,43948402 |
| 1,761804087 | 1,43948402 |
| 1,761804087 | 1,43948402 |
| 1,761804087 | 1,43948402 |
| 1,761804087 | 1,43948402 |
| 1,761804087 | 1,43948402 |
| 1,761804087 | 1,43948402 |
| 1,761804087 | 1,43948402 |
| 1,761804087 | 1,43948402 |
| 1,761804087 | 1,43948402 |
| 1,761804087 | 1,43948402 |
| 1,761804087 | 1,43948402 |
| 1,761804087 | 1,43948402 |
| 1,761804087 | 1,43948402 |
| 1,761804087 | 1,43948402 |
| 1,761804087 | 1,43948402 |
| 1,761804087 | 1,43948402 |
| 1,761804087 | 1,43948402 |
| 1,761804087 | 1,43948402 |
| 1,761804087 | 1,43948402 |
| 1,761804087 | 1,43948402 |
| 1,761804087 | 1,43948402 |
| 1,761804087 | 1,43948402 |
| 1,761804087 | 1,43948402 |
| 1,761804087 | 1,43948402 |
| 1,761804087 | 1,43948402 |
| 1,761804087 | 1,43948402 |
| 1,761804087 | 1,43948402 |
| 1,761804087 | 1,43948402 |
| 1,761804087 | 1,43948402 |
| 1,761804087 | 1,43948402 |
| 1,761804087 | 1,43948402 |
| 1,761804087 | 1,43948402 |
| 1,761804087 | 1,43948402 |
| 1,761804087 | 1,43948402 |
| 1,761804087 | 1,43948402 |
| 1,761804087 | 1,43948402 |
| 1,761804087 | 1,43948402 |
| 1,761804087 | 1,43948402 |
| 1,761804087 | 1,43948402 |
| 1,761804087 | 1,43948402 |
| 1,761804087 | 1,43948402 |
| 1,761804087 | 1,43948402 |

| | |
|---|---|
| 1,761804087 | 1,43948402 |
| 1,761804087 | 1,43948402 |
| 1,761804087 | 1,43948402 |
| 1,761804087 | 1,43948402 |
| 1,761804087 | 1,43948402 |
| 1,761804087 | 1,43948402 |
| 1,761804087 | 1,43948402 |
| 1,761804087 | 1,43948402 |
| 1,761804087 | 1,43948402 |
| 1,761804087 | 1,43948402 |
| 1,761804087 | 1,43948402 |
| 1,761804087 | 1,43948402 |
| 1,761804087 | 1,43948402 |
| 1,761804087 | 1,43948402 |
| 1,761804087 | 1,43948402 |
| 1,761804087 | 1,43948402 |
| 1,761804087 | 1,43948402 |
| 1,761804087 | 1,43948402 |
| 1,761804087 | 1,43948402 |
| 1,761804087 | 1,43948402 |
| 1,761804087 | 1,43948402 |
| 1,761804087 | 1,43948402 |
| 1,761804087 | 1,43948402 |
| 1,761804087 | 1,43948402 |
| 1,761804087 | 1,43948402 |
| 1,761804087 | 1,43948402 |
| 1,761804087 | 1,43948402 |
| 1,761804087 | 1,43948402 |
| 1,761804087 | 1,43948402 |
| 1,761804087 | 1,43948402 |
| 1,761804087 | 1,43948402 |
| 1,761804087 | 1,43948402 |
| 1,761804087 | 1,43948402 |
| 1,761804087 | 1,43948402 |
| 1,761804087 | 1,43948402 |
| 1,761804087 | 1,43948402 |
| 1,761804087 | 1,43948402 |
| 1,761804087 | 1,43948402 |
| 1,761804087 | 1,43948402 |
| 1,761804087 | 1,43948402 |
| 1,761804087 | 1,43948402 |
| 1,761804087 | 1,43948402 |
| 1,761804087 | 1,43948402 |
| 1,099565672 | 0,894108459 |
| 1,099565672 | 0,894108459 |
| 1,090116279 | 0,884662469 |
| 1,174536058 | 0,950133936 |
| 1,336541032 | 1,080741454 |
| 1,336541032 | 1,080741454 |
| 1,336541032 | 1,080741454 |
| 1,336541032 | 1,080741454 |
| 1,336541032 | 1,080741454 |
| 1,336541032 | 1,080741454 |

| | |
|---|---|
| 1,336541032 | 1,080741454 |
| 1,336541032 | 1,080741454 |
| 1,336541032 | 1,080741454 |
| 1,336541032 | 1,080741454 |
| 1,13999088 | 0,914819788 |
| 1,13999088 | 0,914819788 |
| 1,083345371 | 0,861294888 |
| 1,21124031 | 0,957535043 |
| 1,21124031 | 0,957535043 |
| 1,157005669 | 0,906344803 |
| 1,070709666 | 0,834746872 |
| 1,045896395 | 0,812090184 |
| 1,049450088 | 0,811295039 |
| 1,291989664 | 0,994844838 |
| 1,291989664 | 0,994844838 |
| 1,291989664 | 0,994844838 |
| 1,291989664 | 0,994844838 |
| 1,291989664 | 0,994844838 |
| 1,291989664 | 0,994844838 |
| 1,291989664 | 0,994844838 |
| 1,291989664 | 0,994844838 |
| 1,291989664 | 0,994844838 |
| 1,291989664 | 0,994844838 |
| 1,291989664 | 0,994844838 |
| 1,291989664 | 0,994844838 |
| 1,291989664 | 0,994844838 |
| 1,291989664 | 0,994844838 |
| 1,291989664 | 0,994844838 |
| 1,291989664 | 0,994844838 |
| 1,057082452 | 0,811767082 |
| 1,18652112 | 0,903028463 |
| 1,18652112 | 0,903028463 |
| 1,18652112 | 0,903028463 |
| 1,052299274 | 0,793859548 |
| 1,61498708 | 1,217446632 |
| 1,61498708 | 1,217446632 |
| 1,61498708 | 1,217446632 |
| 1,61498708 | 1,217446632 |
| 1,61498708 | 1,217446632 |
| 1,61498708 | 1,217446632 |
| 1,61498708 | 1,217446632 |
| 1,61498708 | 1,217446632 |
| 1,61498708 | 1,217446632 |
| 1,61498708 | 1,217446632 |
| 1,61498708 | 1,217446632 |
| 1,61498708 | 1,217446632 |
| 1,61498708 | 1,217446632 |
| 1,61498708 | 1,217446632 |
| 1,61498708 | 1,217446632 |
| 1,61498708 | 1,217446632 |
| 1,61498708 | 1,217446632 |
| 1,61498708 | 1,217446632 |
| 1,61498708 | 1,217446632 |

| | |
|---|---|
| 1,61498708 | 1,217446632 |
| 1,61498708 | 1,217446632 |
| 1,61498708 | 1,217446632 |
| 1,61498708 | 1,217446632 |
| 1,61498708 | 1,217446632 |
| 1,61498708 | 1,217446632 |
| 1,61498708 | 1,217446632 |
| 1,61498708 | 1,217446632 |
| 1,61498708 | 1,217446632 |
| 1,61498708 | 1,217446632 |
| 1,61498708 | 1,217446632 |
| 1,61498708 | 1,217446632 |
| 1,61498708 | 1,217446632 |
| 1,61498708 | 1,217446632 |
| 1,61498708 | 1,217446632 |
| 1,61498708 | 1,217446632 |
| 1,61498708 | 1,217446632 |
| 1,61498708 | 1,217446632 |
| 1,61498708 | 1,217446632 |
| 1,61498708 | 1,217446632 |
| 1,61498708 | 1,217446632 |
| 1,61498708 | 1,217446632 |
| 1,61498708 | 1,217446632 |
| 1,61498708 | 1,217446632 |
| 1,61498708 | 1,217446632 |
| 1,61498708 | 1,217446632 |
| 1,61498708 | 1,217446632 |
| 1,61498708 | 1,217446632 |
| 1,61498708 | 1,217446632 |
| 1,61498708 | 1,217446632 |
| 1,61498708 | 1,217446632 |
| 1,61498708 | 1,217446632 |
| 1,61498708 | 1,217446632 |
| 1,61498708 | 1,217446632 |
| 1,61498708 | 1,217446632 |
| 1,61498708 | 1,217446632 |
| 1,61498708 | 1,217446632 |
| 1,61498708 | 1,217446632 |
| 1,61498708 | 1,217446632 |
| 1,61498708 | 1,217446632 |
| 1,61498708 | 1,217446632 |
| 1,61498708 | 1,217446632 |
| 1,61498708 | 1,217446632 |
| 1,61498708 | 1,217446632 |
| 1,61498708 | 1,217446632 |
| 1,61498708 | 1,217446632 |
| 1,61498708 | 1,217446632 |
| 1,61498708 | 1,217446632 |
| 1,61498708 | 1,217446632 |
| 1,61498708 | 1,217446632 |
| 1,61498708 | 1,217446632 |
| 1,61498708 | 1,217446632 |
| 1,61498708 | 1,217446632 |

| | |
|---|---|
| 1,069232825 | 0,796381703 |
| 1,123469273 | 0,825285506 |
| 1,250312578 | 0,917145122 |
| 1,250312578 | 0,917145122 |
| 1,250312578 | 0,917145122 |
| 1,250312578 | 0,917145122 |
| 1,250312578 | 0,917145122 |
| 1,250312578 | 0,917145122 |
| 1,250312578 | 0,917145122 |
| 1,250312578 | 0,917145122 |
| 1,250312578 | 0,917145122 |
| 1,250312578 | 0,917145122 |
| 1,250312578 | 0,917145122 |
| 1,250312578 | 0,917145122 |
| 1,250312578 | 0,917145122 |
| 1,250312578 | 0,917145122 |
| 1,250312578 | 0,917145122 |
| 1,162790698 | 0,852051221 |
| 1,068180431 | 0,774296519 |
| 1,04499164 | 0,757141951 |
| 1,076658053 | 0,776106625 |
| 1,088755335 | 0,779971136 |
| 1,088755335 | 0,779971136 |
| 1,107419712 | 0,787767401 |
| 1,13999088 | 0,804334959 |
| 1,13999088 | 0,804334959 |
| 1,13999088 | 0,804334959 |
| 1,21124031 | 0,84669333 |
| 1,21124031 | 0,84669333 |
| 1,21124031 | 0,84669333 |
| 1,21124031 | 0,84669333 |
| 1,21124031 | 0,84669333 |
| 1,21124031 | 0,84669333 |
| 1,490757305 | 1,03961712 |
| 1,490757305 | 1,03961712 |
| 1,490757305 | 1,03961712 |
| 1,490757305 | 1,03961712 |
| 1,490757305 | 1,03961712 |
| 1,490757305 | 1,03961712 |
| 1,490757305 | 1,03961712 |
| 1,490757305 | 1,03961712 |
| 1,490757305 | 1,03961712 |
| 1,490757305 | 1,03961712 |
| 1,490757305 | 1,03961712 |
| 1,490757305 | 1,03961712 |
| 1,490757305 | 1,03961712 |
| 1,490757305 | 1,03961712 |
| 1,490757305 | 1,03961712 |
| 1,490757305 | 1,03961712 |
| 1,490757305 | 1,03961712 |
| 1,490757305 | 1,03961712 |
| 1,490757305 | 1,03961712 |
| 1,490757305 | 1,03961712 |

| | |
|---|---|
| 1,490757305 | 1,03961712 |
| 1,490757305 | 1,03961712 |
| 1,490757305 | 1,03961712 |
| 1,490757305 | 1,03961712 |
| 1,490757305 | 1,03961712 |
| 1,490757305 | 1,03961712 |
| 1,490757305 | 1,03961712 |
| 1,490757305 | 1,03961712 |
| 1,490757305 | 1,03961712 |
| 1,490757305 | 1,03961712 |
| 1,490757305 | 1,03961712 |
| 1,490757305 | 1,03961712 |
| 1,490757305 | 1,03961712 |
| 1,490757305 | 1,03961712 |
| 1,490757305 | 1,03961712 |
| 1,490757305 | 1,03961712 |
| 1,490757305 | 1,03961712 |
| 1,490757305 | 1,03961712 |
| 1,490757305 | 1,03961712 |
| 1,490757305 | 1,03961712 |
| 1,490757305 | 1,03961712 |
| 1,490757305 | 1,03961712 |
| 1,490757305 | 1,03961712 |
| 1,490757305 | 1,03961712 |
| 1,490757305 | 1,03961712 |
| 1,490757305 | 1,03961712 |
| 1,490757305 | 1,03961712 |
| 1,490757305 | 1,03961712 |
| 1,490757305 | 1,03961712 |
| 1,490757305 | 1,03961712 |
| 1,490757305 | 1,03961712 |
| 1,490757305 | 1,03961712 |
| 1,490757305 | 1,03961712 |
| 1,490757305 | 1,03961712 |
| 1,490757305 | 1,03961712 |
| 1,091822251 | 0,752105519 |
| 1,118067979 | 0,759635486 |
| 1,064826646 | 0,720620255 |
| 1,076658053 | 0,718195651 |
| 1,174536058 | 0,782671939 |
| 1,174536058 | 0,782671939 |
| 1,174536058 | 0,782671939 |
| 1,174536058 | 0,782671939 |
| 1,174536058 | 0,782671939 |
| 1,174536058 | 0,782671939 |
| 1,174536058 | 0,782671939 |
| 1,174536058 | 0,782671939 |
| 1,174536058 | 0,782671939 |
| 1,01587897 | 0,669637361 |
| 1,035564235 | 0,682468966 |
| 1,053252444 | 0,692752011 |
| 1,053252444 | 0,692752011 |
| 1,053252444 | 0,692752011 |

| | |
|---|---|
| 1,053252444 | 0,692752011 |
| 1,096972356 | 0,71773023 |
| 1,096972356 | 0,71773023 |
| 1,096972356 | 0,71773023 |
| 1,096972356 | 0,71773023 |
| 1,38427464 | 0,895092168 |
| 1,38427464 | 0,895092168 |
| 1,38427464 | 0,895092168 |
| 1,38427464 | 0,895092168 |
| 1,38427464 | 0,895092168 |
| 1,38427464 | 0,895092168 |
| 1,38427464 | 0,895092168 |
| 1,38427464 | 0,895092168 |
| 1,38427464 | 0,895092168 |
| 1,38427464 | 0,895092168 |
| 1,38427464 | 0,895092168 |
| 1,38427464 | 0,895092168 |
| 1,38427464 | 0,895092168 |
| 1,38427464 | 0,895092168 |
| 1,38427464 | 0,895092168 |
| 1,38427464 | 0,895092168 |
| 1,38427464 | 0,895092168 |
| 1,38427464 | 0,895092168 |
| 1,38427464 | 0,895092168 |
| 1,38427464 | 0,895092168 |
| 1,38427464 | 0,895092168 |
| 1,38427464 | 0,895092168 |
| 1,38427464 | 0,895092168 |
| 1,38427464 | 0,895092168 |
| 1,38427464 | 0,895092168 |
| 1,38427464 | 0,895092168 |
| 1,38427464 | 0,895092168 |
| 1,38427464 | 0,895092168 |
| 1,38427464 | 0,895092168 |
| 1,38427464 | 0,895092168 |
| 1,38427464 | 0,895092168 |
| 1,38427464 | 0,895092168 |
| 1,38427464 | 0,895092168 |
| 1,38427464 | 0,895092168 |
| 1,38427464 | 0,895092168 |
| 1,38427464 | 0,895092168 |
| 1,38427464 | 0,895092168 |
| 1,38427464 | 0,895092168 |
| 1,38427464 | 0,895092168 |
| 1,38427464 | 0,895092168 |
| 1,38427464 | 0,895092168 |
| 1,061909313 | 0,685940401 |
| 1,061909313 | 0,685940401 |
| 1,061909313 | 0,685940401 |
| 1,011122346 | 0,648736382 |
| 1,13999088 | 0,724373623 |
| 1,13999088 | 0,724373623 |
| 1,13999088 | 0,724373623 |

| | |
|---|---|
| 1,13999088 | 0,724373623 |
| 1,13999088 | 0,724373623 |
| 1,13999088 | 0,724373623 |
| 1,13999088 | 0,724373623 |
| 1,13999088 | 0,724373623 |
| 1,13999088 | 0,724373623 |
| 1,13999088 | 0,724373623 |
| 1,13999088 | 0,724373623 |
| 1,029018317 | 0,651781641 |
| 1,076658053 | 0,678416004 |
| 1,047559187 | 0,655248672 |
| 1,030842817 | 0,640362983 |
| 1,01999184 | 0,629481853 |
| 1,012379961 | 0,620879833 |
| 1,057082452 | 0,641507029 |
| 1,057082452 | 0,641507029 |
| 1,057082452 | 0,641507029 |
| 1,107419712 | 0,671183906 |
| 1,107419712 | 0,671183906 |
| 1,107419712 | 0,671183906 |
| 1,107419712 | 0,671183906 |
| 1,107419712 | 0,671183906 |
| 1,107419712 | 0,671183906 |
| 1,107419712 | 0,671183906 |
| 1,107419712 | 0,671183906 |
| 1,033591731 | 0,626035189 |
| 1,01999184 | 0,615743584 |
| 1,01999184 | 0,615743584 |
| 1,291989664 | 0,776152959 |
| 1,291989664 | 0,776152959 |
| 1,291989664 | 0,776152959 |
| 1,291989664 | 0,776152959 |
| 1,291989664 | 0,776152959 |
| 1,291989664 | 0,776152959 |
| 1,291989664 | 0,776152959 |
| 1,291989664 | 0,776152959 |
| 1,291989664 | 0,776152959 |
| 1,291989664 | 0,776152959 |
| 1,291989664 | 0,776152959 |
| 1,291989664 | 0,776152959 |
| 1,291989664 | 0,776152959 |
| 1,291989664 | 0,776152959 |
| 1,291989664 | 0,776152959 |
| 1,291989664 | 0,776152959 |
| 1,291989664 | 0,776152959 |
| 1,291989664 | 0,776152959 |
| 1,291989664 | 0,776152959 |
| 1,291989664 | 0,776152959 |
| 1,291989664 | 0,776152959 |
| 1,291989664 | 0,776152959 |
| 1,291989664 | 0,776152959 |
| 1,291989664 | 0,776152959 |
| 1,291989664 | 0,776152959 |

| | |
|---|---|
| 1,291989664 | 0,776152959 |
| 1,291989664 | 0,776152959 |
| 1,291989664 | 0,776152959 |
| 1,291989664 | 0,776152959 |
| 1,291989664 | 0,776152959 |
| 1,291989664 | 0,776152959 |
| 1,291989664 | 0,776152959 |
| 1,291989664 | 0,776152959 |
| 1,291989664 | 0,776152959 |
| 1,291989664 | 0,776152959 |
| 1,291989664 | 0,776152959 |
| 1,291989664 | 0,776152959 |
| 1,291989664 | 0,776152959 |
| 1,291989664 | 0,776152959 |
| 1,291989664 | 0,776152959 |
| 1,291989664 | 0,776152959 |
| 1,291989664 | 0,776152959 |
| 1,291989664 | 0,776152959 |
| 1,291989664 | 0,776152959 |
| 1,291989664 | 0,776152959 |
| 1,291989664 | 0,776152959 |
| 1,291989664 | 0,776152959 |
| 1,291989664 | 0,776152959 |
| 1,291989664 | 0,776152959 |
| 1,291989664 | 0,776152959 |
| 1,291989664 | 0,776152959 |
| 1,291989664 | 0,776152959 |
| 1,291989664 | 0,776152959 |
| 1,291989664 | 0,776152959 |
| 1,291989664 | 0,776152959 |
| 1,291989664 | 0,776152959 |
| 1,291989664 | 0,776152959 |
| 1,291989664 | 0,776152959 |
| 1,291989664 | 0,776152959 |
| 1,291989664 | 0,776152959 |
| 1,291989664 | 0,776152959 |
| 1,291989664 | 0,776152959 |
| 1,291989664 | 0,776152959 |
| 1,291989664 | 0,776152959 |
| 1,291989664 | 0,776152959 |
| 1,291989664 | 0,776152959 |
| 1,291989664 | 0,776152959 |
| 1,291989664 | 0,776152959 |
| 1,291989664 | 0,776152959 |
| 1,291989664 | 0,776152959 |
| 1,291989664 | 0,776152959 |
| 1,00488085 | 0,601617365 |
| 1,002405774 | 0,587194881 |
| 1,002405774 | 0,587194881 |
| 1,002405774 | 0,587194881 |
| 1,03820598 | 0,60683319 |
| 1,03820598 | 0,60683319 |
| 0,99749202 | 0,582954156 |

| | |
|---|---|
| 1,076658053 | 0,622566934 |
| 1,076658053 | 0,622566934 |
| 1,076658053 | 0,622566934 |
| 1,076658053 | 0,622566934 |
| 1,076658053 | 0,622566934 |
| 1,076658053 | 0,622566934 |
| 1,076658053 | 0,622566934 |
| 1,076658053 | 0,622566934 |
| 1,076658053 | 0,622566934 |
| 1,076658053 | 0,622566934 |
| 1,076658053 | 0,622566934 |
| 0,993838203 | 0,567150486 |
| 0,993838203 | 0,567150486 |
| 0,99896108 | 0,569426955 |
| 1,01999184 | 0,5742385 |
| 1,01999184 | 0,5742385 |
| 1,01999184 | 0,5742385 |
| 1,01999184 | 0,5742385 |
| 1,01999184 | 0,5742385 |
| 1,21124031 | 0,6772029 |
| 1,21124031 | 0,6772029 |
| 1,21124031 | 0,6772029 |
| 1,21124031 | 0,6772029 |
| 1,21124031 | 0,6772029 |
| 1,21124031 | 0,6772029 |
| 1,21124031 | 0,6772029 |
| 1,21124031 | 0,6772029 |
| 1,21124031 | 0,6772029 |
| 1,21124031 | 0,6772029 |
| 1,21124031 | 0,6772029 |
| 1,21124031 | 0,6772029 |
| 1,21124031 | 0,6772029 |
| 1,21124031 | 0,6772029 |
| 1,21124031 | 0,6772029 |
| 1,21124031 | 0,6772029 |
| 1,21124031 | 0,6772029 |
| 1,21124031 | 0,6772029 |
| 1,21124031 | 0,6772029 |
| 1,21124031 | 0,6772029 |
| 1,21124031 | 0,6772029 |
| 1,21124031 | 0,6772029 |
| 1,21124031 | 0,6772029 |
| 1,21124031 | 0,6772029 |
| 1,21124031 | 0,6772029 |
| 1,21124031 | 0,6772029 |
| 1,21124031 | 0,6772029 |
| 1,21124031 | 0,6772029 |
| 1,21124031 | 0,6772029 |
| 1,21124031 | 0,6772029 |
| 1,21124031 | 0,6772029 |
| 1,21124031 | 0,6772029 |
| 1,21124031 | 0,6772029 |

| | |
|---|---|
| 1,21124031 | 0,6772029 |
| 1,21124031 | 0,6772029 |
| 0,983035614 | 0,547348206 |
| 0,9887676 | 0,547645291 |
| 0,9887676 | 0,547645291 |
| 1,047559187 | 0,578053737 |
| 1,047559187 | 0,578053737 |
| 1,047559187 | 0,578053737 |
| 1,047559187 | 0,578053737 |
| 1,047559187 | 0,578053737 |
| 1,047559187 | 0,578053737 |
| 1,047559187 | 0,578053737 |
| 1,047559187 | 0,578053737 |
| 1,047559187 | 0,578053737 |
| 1,047559187 | 0,578053737 |
| 1,047559187 | 0,578053737 |
| 0,993838203 | 0,546489952 |
| 0,993838203 | 0,546489952 |
| 0,975353248 | 0,531243158 |
| 0,975963415 | 0,530368537 |
| 1,002405774 | 0,543579722 |
| 1,002405774 | 0,543579722 |
| 1,002405774 | 0,543579722 |
| 0,968992248 | 0,516665138 |
| 0,981257973 | 0,522438724 |
| 0,968992248 | 0,511094962 |
| 1,01999184 | 0,537232486 |
| 1,01999184 | 0,537232486 |
| 1,01999184 | 0,537232486 |
| 1,01999184 | 0,537232486 |
| 1,01999184 | 0,537232486 |
| 0,964971533 | 0,50533026 |
| 0,964607668 | 0,504933269 |
| 0,985415845 | 0,514725165 |
| 0,985415845 | 0,514725165 |
| 0,985415845 | 0,514725165 |
| 0,985415845 | 0,514725165 |
| 0,968992248 | 0,505409746 |
| 1,13999088 | 0,594099447 |
| 1,13999088 | 0,594099447 |
| 1,13999088 | 0,594099447 |
| 1,13999088 | 0,594099447 |
| 1,13999088 | 0,594099447 |
| 1,13999088 | 0,594099447 |
| 1,13999088 | 0,594099447 |
| 1,13999088 | 0,594099447 |
| 1,13999088 | 0,594099447 |
| 1,13999088 | 0,594099447 |
| 1,13999088 | 0,594099447 |
| 1,13999088 | 0,594099447 |
| 1,13999088 | 0,594099447 |
| 1,13999088 | 0,594099447 |
| 1,13999088 | 0,594099447 |

| | |
|---|---|
| 1,13999088 | 0,594099447 |
| 1,13999088 | 0,594099447 |
| 1,13999088 | 0,594099447 |
| 1,13999088 | 0,594099447 |
| 1,13999088 | 0,594099447 |
| 1,13999088 | 0,594099447 |
| 1,13999088 | 0,594099447 |
| 1,13999088 | 0,594099447 |
| 1,13999088 | 0,594099447 |
| 1,13999088 | 0,594099447 |
| 1,13999088 | 0,594099447 |
| 1,13999088 | 0,594099447 |
| 1,13999088 | 0,594099447 |
| 1,13999088 | 0,594099447 |
| 1,13999088 | 0,594099447 |
| 1,13999088 | 0,594099447 |
| 1,13999088 | 0,594099447 |
| 1,13999088 | 0,594099447 |
| 1,13999088 | 0,594099447 |
| 1,13999088 | 0,594099447 |
| 1,13999088 | 0,594099447 |
| 1,13999088 | 0,594099447 |
| 1,13999088 | 0,594099447 |
| 1,13999088 | 0,594099447 |
| 0,962119963 | 0,497968366 |
| 0,960984048 | 0,493891937 |
| 0,959398265 | 0,487343017 |
| 0,993838203 | 0,499740374 |
| 0,953107129 | 0,476878487 |
| 0,9499924 | 0,468805112 |
| 1,076658053 | 0,523720552 |
| 1,076658053 | 0,523720552 |
| 1,076658053 | 0,523720552 |
| 1,076658053 | 0,523720552 |
| 1,076658053 | 0,523720552 |
| 1,076658053 | 0,523720552 |
| 1,076658053 | 0,523720552 |
| 1,076658053 | 0,523720552 |
| 1,076658053 | 0,523720552 |
| 1,076658053 | 0,523720552 |
| 1,076658053 | 0,523720552 |
| 1,076658053 | 0,523720552 |
| 1,076658053 | 0,523720552 |
| 1,076658053 | 0,523720552 |
| 1,076658053 | 0,523720552 |
| 1,076658053 | 0,523720552 |
| 1,076658053 | 0,523720552 |
| 1,076658053 | 0,523720552 |
| 1,076658053 | 0,523720552 |
| 1,076658053 | 0,523720552 |
| 1,076658053 | 0,523720552 |
| 1,076658053 | 0,523720552 |
| 1,076658053 | 0,523720552 |

| | |
|---|---|
| 1,076658053 | 0,523720552 |
| 1,076658053 | 0,523720552 |
| 1,076658053 | 0,523720552 |
| 0,953107129 | 0,461953244 |
| 0,953107129 | 0,461953244 |
| 0,953107129 | 0,461953244 |
| 0,968992248 | 0,465256812 |
| 0,968992248 | 0,465256812 |
| 0,968992248 | 0,465256812 |
| 0,968992248 | 0,465256812 |
| 0,968992248 | 0,465256812 |
| 0,968992248 | 0,465256812 |
| 0,968992248 | 0,465256812 |
| 0,968992248 | 0,465256812 |
| 0,939628847 | 0,445831273 |
| 0,933968432 | 0,436920693 |
| 0,930232558 | 0,429839579 |
| 0,930232558 | 0,429839579 |
| 0,945358291 | 0,433497705 |
| 0,945358291 | 0,433497705 |
| 0,945358291 | 0,433497705 |
| 0,945358291 | 0,433497705 |
| 1,01999184 | 0,463675624 |
| 1,01999184 | 0,463675624 |
| 1,01999184 | 0,463675624 |
| 1,01999184 | 0,463675624 |
| 1,01999184 | 0,463675624 |
| 1,01999184 | 0,463675624 |
| 1,01999184 | 0,463675624 |
| 1,01999184 | 0,463675624 |
| 1,01999184 | 0,463675624 |
| 1,01999184 | 0,463675624 |
| 1,01999184 | 0,463675624 |
| 1,01999184 | 0,463675624 |
| 1,01999184 | 0,463675624 |
| 1,01999184 | 0,463675624 |
| 1,01999184 | 0,463675624 |
| 1,01999184 | 0,463675624 |
| 1,01999184 | 0,463675624 |
| 1,01999184 | 0,463675624 |
| 1,01999184 | 0,463675624 |
| 1,01999184 | 0,463675624 |
| 1,01999184 | 0,463675624 |
| 1,01999184 | 0,463675624 |
| 1,01999184 | 0,463675624 |
| 1,01999184 | 0,463675624 |
| 1,01999184 | 0,463675624 |
| 1,01999184 | 0,463675624 |
| 1,01999184 | 0,463675624 |
| 1,01999184 | 0,463675624 |
| 1,01999184 | 0,463675624 |
| 1,01999184 | 0,463675624 |
| 1,01999184 | 0,463675624 |

| | |
|---|---|
| 1,01999184 | 0,463675624 |
| 1,01999184 | 0,463675624 |
| 1,01999184 | 0,463675624 |
| 1,01999184 | 0,463675624 |
| 0,92284976 | 0,417946422 |
| 0,92284976 | 0,417430354 |
| 0,92284976 | 0,417430354 |
| 0,92284976 | 0,415061615 |
| 0,92284976 | 0,415061615 |
| 0,92284976 | 0,415061615 |
| 0,92284976 | 0,415061615 |
| 0,92284976 | 0,415061615 |
| 0,927265309 | 0,410878244 |
| 0,91414363 | 0,401621313 |
| 0,911992704 | 0,399838323 |
| 0,92284976 | 0,404210609 |
| 0,92284976 | 0,404210609 |
| 0,92284976 | 0,404210609 |
| 0,92284976 | 0,404210609 |
| 0,92284976 | 0,404210609 |
| 0,92284976 | 0,404210609 |
| 0,908430233 | 0,393586946 |
| 0,908430233 | 0,393586946 |
| 0,968992248 | 0,412108201 |
| 0,968992248 | 0,412108201 |
| 0,968992248 | 0,412108201 |
| 0,968992248 | 0,412108201 |
| 0,968992248 | 0,412108201 |
| 0,968992248 | 0,412108201 |
| 0,968992248 | 0,412108201 |
| 0,968992248 | 0,412108201 |
| 0,968992248 | 0,412108201 |
| 0,968992248 | 0,412108201 |
| 0,968992248 | 0,412108201 |
| 0,968992248 | 0,412108201 |
| 0,968992248 | 0,412108201 |
| 0,968992248 | 0,412108201 |
| 0,968992248 | 0,412108201 |
| 0,968992248 | 0,412108201 |
| 0,968992248 | 0,412108201 |
| 0,968992248 | 0,412108201 |
| 0,968992248 | 0,412108201 |
| 0,968992248 | 0,412108201 |
| 0,968992248 | 0,412108201 |
| 0,968992248 | 0,412108201 |
| 0,968992248 | 0,412108201 |
| 0,968992248 | 0,412108201 |
| 0,968992248 | 0,412108201 |
| 0,968992248 | 0,412108201 |
| 0,968992248 | 0,412108201 |
| 0,910462515 | 0,386868193 |
| 0,901388138 | 0,377170632 |

| | |
|---|---|
| 0,901388138 | 0,377170632 |
| 0,901388138 | 0,377170632 |
| 0,894454383 | 0,373315421 |
| 0,901388138 | 0,374294043 |
| 0,897215045 | 0,371816246 |
| 0,898403409 | 0,363161047 |
| 0,888983714 | 0,357770892 |
| 0,888983714 | 0,357770892 |
| 0,90372334 | 0,36258872 |
| 0,880902044 | 0,352176933 |
| 0,880902044 | 0,352176933 |
| 0,880902044 | 0,352176933 |
| 0,880902044 | 0,352176933 |
| 0,880902044 | 0,352176933 |
| 0,880902044 | 0,352176933 |
| 0,92284976 | 0,367558366 |
| 0,92284976 | 0,367558366 |
| 0,92284976 | 0,367558366 |
| 0,92284976 | 0,367558366 |
| 0,92284976 | 0,367558366 |
| 0,92284976 | 0,367558366 |
| 0,92284976 | 0,367558366 |
| 0,92284976 | 0,367558366 |
| 0,92284976 | 0,367558366 |
| 0,92284976 | 0,367558366 |
| 0,92284976 | 0,367558366 |
| 0,92284976 | 0,367558366 |
| 0,92284976 | 0,367558366 |
| 0,92284976 | 0,367558366 |
| 0,92284976 | 0,367558366 |
| 0,92284976 | 0,367558366 |
| 0,92284976 | 0,367558366 |
| 0,92284976 | 0,367558366 |
| 0,92284976 | 0,367558366 |
| 0,92284976 | 0,367558366 |
| 0,92284976 | 0,367558366 |
| 0,92284976 | 0,367558366 |
| 0,867754252 | 0,336084841 |
| 0,886659573 | 0,340883731 |
| 0,861326443 | 0,329049728 |
| 0,861326443 | 0,329049728 |
| 0,861326443 | 0,329049728 |
| 0,861326443 | 0,329049728 |
| 0,861326443 | 0,329049728 |
| 0,861326443 | 0,329049728 |
| 0,861326443 | 0,329049728 |
| 0,861326443 | 0,329049728 |
| 0,861326443 | 0,329049728 |
| 0,861326443 | 0,329049728 |
| 0,861326443 | 0,329049728 |
| 0,872965989 | 0,331279519 |
| 0,872965989 | 0,331279519 |

| | |
|---|---|
| 0,880902044 | 0,328864994 |
| 0,880902044 | 0,328864994 |
| 0,880902044 | 0,328864994 |
| 0,880902044 | 0,328864994 |
| 0,880902044 | 0,328864994 |
| 0,880902044 | 0,328864994 |
| 0,880902044 | 0,328864994 |
| 0,880902044 | 0,328864994 |
| 0,880902044 | 0,328864994 |
| 0,880902044 | 0,328864994 |
| 0,880902044 | 0,328864994 |
| 0,880902044 | 0,328864994 |
| 0,880902044 | 0,328864994 |
| 0,880902044 | 0,328864994 |
| 0,880902044 | 0,328864994 |
| 0,880902044 | 0,328864994 |
| 0,880902044 | 0,328864994 |
| 0,880902044 | 0,328864994 |
| 0,880902044 | 0,328864994 |
| 0,880902044 | 0,328864994 |
| 0,880902044 | 0,328864994 |
| 0,880902044 | 0,328864994 |
| 0,880902044 | 0,328864994 |
| 0,880902044 | 0,328864994 |
| 0,85499316 | 0,318990509 |
| 0,85499316 | 0,318990509 |
| 0,85499316 | 0,318990509 |
| 0,842601955 | 0,30762773 |
| 0,842601955 | 0,30762773 |
| 0,842601955 | 0,30762773 |
| 0,842601955 | 0,30762773 |
| 0,842601955 | 0,30762773 |
| 0,851861317 | 0,307122715 |
| 0,842601955 | 0,3028279 |
| 0,842601955 | 0,3028279 |
| 0,842601955 | 0,3028279 |
| 0,842601955 | 0,295095132 |
| 0,842601955 | 0,295095132 |
| 0,842601955 | 0,295095132 |
| 0,842601955 | 0,295095132 |
| 0,842601955 | 0,295095132 |
| 0,842601955 | 0,295095132 |
| 0,842601955 | 0,295095132 |
| 0,842601955 | 0,295095132 |
| 0,842601955 | 0,295095132 |
| 0,842601955 | 0,295095132 |
| 0,842601955 | 0,295095132 |
| 0,842601955 | 0,295095132 |
| 0,842601955 | 0,295095132 |
| 0,842601955 | 0,295095132 |
| 0,842601955 | 0,295095132 |
| 0,842601955 | 0,295095132 |
| 0,842601955 | 0,295095132 |

| | |
|---|---|
| 0,842601955 | 0,295095132 |
| 0,842601955 | 0,295095132 |
| 0,842601955 | 0,295095132 |
| 0,842601955 | 0,295095132 |
| 0,824674254 | 0,287765937 |
| 0,824674254 | 0,287765937 |
| 0,824674254 | 0,287765937 |
| 0,824674254 | 0,287765937 |
| 0,824674254 | 0,287765937 |
| 0,842601955 | 0,293994538 |
| 0,80749354 | 0,269333725 |
| 0,80749354 | 0,269333725 |
| 0,80749354 | 0,269333725 |
| 0,80749354 | 0,269333725 |
| 0,818866689 | 0,273076457 |
| 0,818866689 | 0,273076457 |
| 0,818866689 | 0,273076457 |
| 0,888983714 | 0,295632354 |
| 0,80749354 | 0,265492182 |
| 0,80749354 | 0,265492182 |
| 0,835338145 | 0,273422113 |
| 0,80749354 | 0,259386324 |
| 0,80749354 | 0,259386324 |
| 0,79101408 | 0,252213195 |
| 0,79101408 | 0,252213195 |
| 0,79101408 | 0,252213195 |
| 0,79101408 | 0,252213195 |
| 0,815993472 | 0,257986232 |
| 0,871757732 | 0,270337461 |
| 0,796431985 | 0,246424727 |
| 0,796431985 | 0,246424727 |
| 0,775193798 | 0,239437363 |
| 0,775193798 | 0,239437363 |
| 0,775193798 | 0,239437363 |
| 0,775193798 | 0,239437363 |
| 0,775193798 | 0,239437363 |
| 0,775193798 | 0,239437363 |
| 0,775193798 | 0,239437363 |
| 0,775193798 | 0,239437363 |
| 0,775193798 | 0,239437363 |
| 0,775193798 | 0,239437363 |
| 0,775193798 | 0,239437363 |
| 0,775193798 | 0,239437363 |
| 0,775193798 | 0,239437363 |
| 0,775193798 | 0,239437363 |
| 0,775193798 | 0,239437363 |
| 0,775193798 | 0,239437363 |
| 0,775193798 | 0,239437363 |
| 0,775193798 | 0,239437363 |
| 0,775193798 | 0,239437363 |
| 0,775193798 | 0,239437363 |
| 0,775193798 | 0,239437363 |
| 0,775193798 | 0,239437363 |

| | |
|---|---|
| 0,775193798 | 0,239437363 |
| 0,821179871 | 0,253242 |
| 0,80749354 | 0,247021868 |
| 0,80749354 | 0,247021868 |
| 0,775193798 | 0,23629774 |
| 0,775193798 | 0,23629774 |
| 0,775193798 | 0,23629774 |
| 0,775193798 | 0,23629774 |
| 0,824674254 | 0,247063441 |
| 0,824674254 | 0,247063441 |
| 0,8142792 | 0,243721183 |
| 0,78566939 | 0,234149134 |
| 0,799168864 | 0,236536578 |
| 0,799168864 | 0,236536578 |
| 0,83804735 | 0,247602413 |
| 0,75999392 | 0,221490787 |
| 0,75999392 | 0,221490787 |
| 0,888242894 | 0,258556964 |
| 0,745378652 | 0,216420683 |
| 0,745378652 | 0,216420683 |
| 0,745378652 | 0,216420683 |
| 0,745378652 | 0,216420683 |
| 0,745378652 | 0,216420683 |
| 0,745378652 | 0,216420683 |
| 0,745378652 | 0,216420683 |
| 0,745378652 | 0,216420683 |
| 0,745378652 | 0,216420683 |
| 0,745378652 | 0,216420683 |
| 0,745378652 | 0,216420683 |
| 0,745378652 | 0,216420683 |
| 0,745378652 | 0,216420683 |
| 0,745378652 | 0,216420683 |
| 0,745378652 | 0,216420683 |
| 0,745378652 | 0,216420683 |
| 0,745378652 | 0,216420683 |
| 0,745378652 | 0,216420683 |
| 0,745378652 | 0,216420683 |
| 0,745378652 | 0,216420683 |
| 0,745378652 | 0,216420683 |
| 0,745378652 | 0,216420683 |
| 0,745378652 | 0,216420683 |
| 0,745378652 | 0,216420683 |
| 0,745378652 | 0,216420683 |
| 0,745378652 | 0,216420683 |
| 0,745378652 | 0,216420683 |
| 0,745378652 | 0,216420683 |
| 0,745378652 | 0,216420683 |
| 0,745378652 | 0,216420683 |
| 0,873892213 | 0,250691779 |
| 0,79101408 | 0,226508139 |
| 0,813140348 | 0,230501219 |
| 0,745378652 | 0,207704715 |

| | |
|---|---|
| 0,745378652 | 0,207704715 |
| 0,745378652 | 0,207704715 |
| 0,869280645 | 0,240810741 |
| 0,783024039 | 0,216915467 |
| 0,783024039 | 0,216915467 |
| 0,76499388 | 0,211499649 |
| 0,794255941 | 0,217261249 |
| 0,880902044 | 0,240956706 |
| 0,717772036 | 0,196018818 |
| 0,717772036 | 0,196018818 |
| 0,717772036 | 0,196018818 |
| 0,717772036 | 0,196018818 |
| 0,717772036 | 0,196018818 |
| 0,717772036 | 0,196018818 |
| 0,717772036 | 0,196018818 |
| 0,717772036 | 0,196018818 |
| 0,717772036 | 0,196018818 |
| 0,717772036 | 0,196018818 |
| 0,717772036 | 0,196018818 |
| 0,717772036 | 0,196018818 |
| 0,717772036 | 0,196018818 |
| 0,87048949 | 0,232439659 |
| 0,731314904 | 0,194859889 |
| 0,731314904 | 0,194859889 |
| 0,755058895 | 0,201053892 |
| 0,755058895 | 0,201053892 |
| 0,755058895 | 0,201053892 |
| 0,781445361 | 0,201240015 |
| 0,69213732 | 0,177878081 |
| 0,69213732 | 0,177878081 |
| 0,69213732 | 0,177878081 |
| 0,69213732 | 0,177878081 |
| 0,69213732 | 0,177878081 |
| 0,69213732 | 0,177878081 |
| 0,69213732 | 0,177878081 |
| 0,69213732 | 0,177878081 |
| 0,69213732 | 0,177878081 |
| 0,69213732 | 0,177878081 |
| 0,69213732 | 0,177878081 |
| 0,69213732 | 0,177878081 |
| 0,69213732 | 0,177878081 |
| 0,69213732 | 0,177878081 |
| 0,873681535 | 0,224425411 |
| 0,745378652 | 0,191150037 |
| 0,745378652 | 0,191150037 |
| 0,745378652 | 0,191150037 |
| 0,717772036 | 0,182883823 |
| 0,717772036 | 0,182883823 |
| 0,75999392 | 0,190556889 |
| 0,775193798 | 0,193677888 |
| 0,775193798 | 0,193677888 |
| 0,73594348 | 0,181757635 |
| 0,73594348 | 0,181757635 |

| | |
|---|---|
| 0,73594348 | 0,181757635 |
| 0,73594348 | 0,181757635 |
| 0,704721635 | 0,171710442 |
| 0,704721635 | 0,171710442 |
| 0,704721635 | 0,171710442 |
| 0,704721635 | 0,171710442 |
| 0,769041467 | 0,186399441 |
| 0,668270516 | 0,161701175 |
| 0,668270516 | 0,161701175 |
| 0,668270516 | 0,161701175 |
| 0,668270516 | 0,161701175 |
| 0,668270516 | 0,161701175 |
| 0,668270516 | 0,161701175 |
| 0,668270516 | 0,161701175 |
| 0,668270516 | 0,161701175 |
| 0,668270516 | 0,161701175 |
| 0,668270516 | 0,161701175 |
| 0,668270516 | 0,161701175 |
| 0,668270516 | 0,161701175 |
| 0,668270516 | 0,161701175 |
| 0,668270516 | 0,161701175 |
| 0,726744186 | 0,172848145 |
| 0,726744186 | 0,172848145 |
| 0,762986022 | 0,179393778 |
| 0,69213732 | 0,161279428 |
| 0,69213732 | 0,161279428 |
| 0,717772036 | 0,164394803 |
| 0,717772036 | 0,164394803 |
| 0,757025194 | 0,172650448 |
| 0,645994832 | 0,1472368 |
| 0,645994832 | 0,1472368 |
| 0,645994832 | 0,1472368 |
| 0,645994832 | 0,1472368 |
| 0,645994832 | 0,1472368 |
| 0,645994832 | 0,1472368 |
| 0,645994832 | 0,1472368 |
| 0,645994832 | 0,1472368 |
| 0,645994832 | 0,1472368 |
| 0,67999456 | 0,151535643 |
| 0,67999456 | 0,151535643 |
| 0,67999456 | 0,151535643 |
| 0,67999456 | 0,151535643 |
| 0,67999456 | 0,151535643 |
| 0,67999456 | 0,151535643 |
| 0,709018718 | 0,156372495 |
| 0,709018718 | 0,156372495 |
| 0,625156289 | 0,134271427 |
| 0,625156289 | 0,134271427 |
| 0,625156289 | 0,134271427 |
| 0,625156289 | 0,134271427 |
| 0,625156289 | 0,134271427 |
| 0,625156289 | 0,134271427 |
| 0,625156289 | 0,134271427 |

| | |
|---|---|
| 0,625156289 | 0,134271427 |
| 0,625156289 | 0,134271427 |
| 0,625156289 | 0,134271427 |
| 0,625156289 | 0,134271427 |
| 0,625156289 | 0,134271427 |
| 0,625156289 | 0,134271427 |
| 0,625156289 | 0,134271427 |
| 0,668270516 | 0,142428619 |
| 0,668270516 | 0,142428619 |
| 0,700476324 | 0,14875764 |
| 0,700476324 | 0,14875764 |
| 0,700476324 | 0,14875764 |
| 0,717772036 | 0,147196452 |
| 0,717772036 | 0,147196452 |
| 0,717772036 | 0,147196452 |
| 0,69213732 | 0,141528082 |
| 0,69213732 | 0,141528082 |
| 0,656943897 | 0,133912104 |
| 0,656943897 | 0,133912104 |
| 0,605620155 | 0,122622744 |
| 0,605620155 | 0,122622744 |
| 0,605620155 | 0,122622744 |
| 0,605620155 | 0,122622744 |
| 0,605620155 | 0,122622744 |
| 0,605620155 | 0,122622744 |
| 0,605620155 | 0,122622744 |
| 0,605620155 | 0,122622744 |
| 0,605620155 | 0,122622744 |
| 0,605620155 | 0,122622744 |
| 0,605620155 | 0,122622744 |
| 0,605620155 | 0,122622744 |
| 0,605620155 | 0,122622744 |
| 0,605620155 | 0,122622744 |
| 0,605620155 | 0,122622744 |
| 0,605620155 | 0,122622744 |
| 0,605620155 | 0,122622744 |
| 0,804699445 | 0,161369862 |
| 0,711186971 | 0,141009715 |
| 0,683994528 | 0,134663 |
| 0,683994528 | 0,134663 |
| 0,683994528 | 0,134663 |
| 0,745378652 | 0,146518158 |
| 0,801924619 | 0,157414067 |
| 0,801924619 | 0,157414067 |
| 0,645994832 | 0,125943661 |
| 0,645994832 | 0,125943661 |
| 0,645994832 | 0,125943661 |
| 0,767518612 | 0,148601737 |
| 0,587268029 | 0,112134385 |
| 0,587268029 | 0,112134385 |
| 0,587268029 | 0,112134385 |
| 0,587268029 | 0,112134385 |
| 0,587268029 | 0,112134385 |

| | |
|---|---|
| 0,587268029 | 0,112134385 |
| 0,587268029 | 0,112134385 |
| 0,587268029 | 0,112134385 |
| 0,587268029 | 0,112134385 |
| 0,587268029 | 0,112134385 |
| 0,587268029 | 0,112134385 |
| 0,723128543 | 0,137165527 |
| 0,796431985 | 0,149770123 |
| 0,635404753 | 0,118484305 |
| 0,635404753 | 0,118484305 |
| 0,698372791 | 0,129412849 |
| 0,56999544 | 0,102671668 |
| 0,56999544 | 0,102671668 |
| 0,56999544 | 0,102671668 |
| 0,56999544 | 0,102671668 |
| 0,56999544 | 0,102671668 |
| 0,56999544 | 0,102671668 |
| 0,56999544 | 0,102671668 |
| 0,56999544 | 0,102671668 |
| 0,56999544 | 0,102671668 |
| 0,56999544 | 0,102671668 |
| 0,56999544 | 0,102671668 |
| 0,56999544 | 0,102671668 |
| 0,756286633 | 0,135630987 |
| 0,625156289 | 0,111498182 |
| 0,625156289 | 0,111498182 |
| 0,625156289 | 0,111498182 |
| 0,625156289 | 0,111498182 |
| 0,625156289 | 0,111498182 |
| 0,7124943 | 0,12702574 |
| 0,660676533 | 0,116064472 |
| 0,660676533 | 0,116064472 |
| 0,660676533 | 0,116064472 |
| 0,741305545 | 0,130205262 |
| 0,73727671 | 0,126026528 |
| 0,615233173 | 0,104952287 |
| 0,615233173 | 0,104952287 |
| 0,553709856 | 0,094118131 |
| 0,553709856 | 0,094118131 |
| 0,553709856 | 0,094118131 |
| 0,553709856 | 0,094118131 |
| 0,553709856 | 0,094118131 |
| 0,553709856 | 0,094118131 |
| 0,553709856 | 0,094118131 |
| 0,553709856 | 0,094118131 |
| 0,553709856 | 0,094118131 |
| 0,653253201 | 0,110472639 |
| 0,605620155 | 0,0988162 |
| 0,605620155 | 0,0988162 |
| 0,605620155 | 0,0988162 |
| 0,605620155 | 0,0988162 |
| 0,605620155 | 0,0988162 |
| 0,645994832 | 0,105158187 |

| | |
|---|---|
| 0,538329027 | 0,086372691 |
| 0,538329027 | 0,086372691 |
| 0,538329027 | 0,086372691 |
| 0,538329027 | 0,086372691 |
| 0,538329027 | 0,086372691 |
| 0,538329027 | 0,086372691 |
| 0,538329027 | 0,086372691 |
| 0,538329027 | 0,086372691 |
| 0,538329027 | 0,086372691 |
| 0,538329027 | 0,086372691 |
| 0,538329027 | 0,086372691 |
| 0,596302922 | 0,093061862 |
| 0,596302922 | 0,093061862 |
| 0,596302922 | 0,093061862 |
| 0,596302922 | 0,093061862 |
| 0,596302922 | 0,093061862 |
| 0,709018718 | 0,110602581 |
| 0,523779594 | 0,079347307 |
| 0,523779594 | 0,079347307 |
| 0,523779594 | 0,079347307 |
| 0,523779594 | 0,079347307 |
| 0,523779594 | 0,079347307 |
| 0,523779594 | 0,079347307 |
| 0,523779594 | 0,079347307 |
| 0,523779594 | 0,079347307 |
| 0,587268029 | 0,087663361 |
| 0,587268029 | 0,087663361 |
| 0,587268029 | 0,087663361 |
| 0,587268029 | 0,087663361 |
| 0,587268029 | 0,087663361 |
| 0,625156289 | 0,090738049 |
| 0,50999592 | 0,072965042 |
| 0,50999592 | 0,072965042 |
| 0,50999592 | 0,072965042 |
| 0,50999592 | 0,072965042 |
| 0,50999592 | 0,072965042 |
| 0,578502835 | 0,08259675 |
| 0,578502835 | 0,08259675 |
| 0,61850569 | 0,086395973 |
| 0,56999544 | 0,077839877 |
| 0,56999544 | 0,077839877 |
| 0,496919102 | 0,067158458 |
| 0,496919102 | 0,067158458 |
| 0,496919102 | 0,067158458 |
| 0,496919102 | 0,067158458 |
| 0,611995104 | 0,082266455 |
| 0,702895931 | 0,093785764 |
| 0,605620155 | 0,078338619 |
| 0,484496124 | 0,061868268 |
| 0,484496124 | 0,061868268 |
| 0,484496124 | 0,061868268 |
| 0,484496124 | 0,061868268 |
| 0,484496124 | 0,061868268 |

| | |
|---|---|
| 0,484496124 | 0,061868268 |
| 0,484496124 | 0,061868268 |
| 0,695686742 | 0,08778298 |
| 0,472679145 | 0,057042206 |
| 0,472679145 | 0,057042206 |
| 0,472679145 | 0,057042206 |
| 0,472679145 | 0,057042206 |
| 0,472679145 | 0,057042206 |
| 0,472679145 | 0,057042206 |
| 0,472679145 | 0,057042206 |
| 0,472679145 | 0,057042206 |
| 0,472679145 | 0,057042206 |
| 0,472679145 | 0,057042206 |
| 0,472679145 | 0,057042206 |
| 0,472679145 | 0,057042206 |
| 0,59326056 | 0,071047448 |
| 0,672133351 | 0,080388578 |
| 0,545911126 | 0,065230001 |
| 0,587268029 | 0,06766521 |
| 0,587268029 | 0,06766521 |
| 0,538329027 | 0,061521435 |
| 0,538329027 | 0,061521435 |
| 0,538329027 | 0,061521435 |
| 0,538329027 | 0,061521435 |
| 0,46142488 | 0,052634076 |
| 0,46142488 | 0,052634076 |
| 0,46142488 | 0,052634076 |
| 0,46142488 | 0,052634076 |
| 0,46142488 | 0,052634076 |
| 0,46142488 | 0,052634076 |
| 0,46142488 | 0,052634076 |
| 0,46142488 | 0,052634076 |
| 0,664451827 | 0,074853829 |
| 0,530954656 | 0,05803393 |
| 0,530954656 | 0,05803393 |
| 0,450694069 | 0,048602946 |
| 0,450694069 | 0,048602946 |
| 0,450694069 | 0,048602946 |
| 0,450694069 | 0,048602946 |
| 0,450694069 | 0,048602946 |
| 0,523779594 | 0,054753371 |
| 0,605620155 | 0,062466539 |
| 0,440451022 | 0,044912461 |
| 0,440451022 | 0,044912461 |
| 0,629215745 | 0,063382902 |
| 0,516795866 | 0,05166663 |
| 0,625156289 | 0,060965261 |
| 0,430663221 | 0,041530262 |
| 0,430663221 | 0,041530262 |
| 0,430663221 | 0,041530262 |
| 0,430663221 | 0,041530262 |
| 0,430663221 | 0,041530262 |
| 0,430663221 | 0,041530262 |

| | |
|---|---|
| 0,50999592 | 0,04876149 |
| 0,50999592 | 0,04876149 |
| 0,689401599 | 0,065004375 |
| 0,503372596 | 0,046026576 |
| 0,553709856 | 0,05053883 |
| 0,421300977 | 0,038427482 |
| 0,496919102 | 0,043451287 |
| 0,412337127 | 0,035578321 |
| 0,412337127 | 0,035578321 |
| 0,412337127 | 0,035578321 |
| 0,412337127 | 0,035578321 |
| 0,665402402 | 0,056661365 |
| 0,543360139 | 0,045865949 |
| 0,543360139 | 0,045865949 |
| 0,543360139 | 0,045865949 |
| 0,490628986 | 0,04102574 |
| 0,490628986 | 0,04102574 |
| 0,490628986 | 0,04102574 |
| 0,40374677 | 0,032959673 |
| 0,40374677 | 0,032959673 |
| 0,40374677 | 0,032959673 |
| 0,40374677 | 0,032959673 |
| 0,40374677 | 0,032959673 |
| 0,40374677 | 0,032959673 |
| 0,484496124 | 0,038740718 |
| 0,56999544 | 0,044310889 |
| 0,39550704 | 0,030550809 |
| 0,39550704 | 0,030550809 |
| 0,39550704 | 0,030550809 |
| 0,47851469 | 0,036587613 |
| 0,47851469 | 0,036587613 |
| 0,615233173 | 0,045214062 |
| 0,472679145 | 0,034558392 |
| 0,472679145 | 0,034558392 |
| 0,472679145 | 0,034558392 |
| 0,387596899 | 0,028333098 |
| 0,387596899 | 0,028333098 |
| 0,387596899 | 0,028333098 |
| 0,387596899 | 0,028333098 |
| 0,466984216 | 0,032645545 |
| 0,37999696 | 0,02628977 |
| 0,37999696 | 0,02628977 |
| 0,37999696 | 0,02628977 |
| 0,514509158 | 0,034302291 |
| 0,372689326 | 0,024405702 |
| 0,372689326 | 0,024405702 |
| 0,372689326 | 0,024405702 |
| 0,372689326 | 0,024405702 |
| 0,365657452 | 0,022667239 |
| 0,365657452 | 0,022667239 |
| 0,450694069 | 0,027537307 |
| 0,358886018 | 0,021062031 |
| 0,358886018 | 0,021062031 |

| | |
|---|---|
| 0,358886018 | 0,021062031 |
| 0,440451022 | 0,024596525 |
| 0,352360817 | 0,019578896 |
| 0,352360817 | 0,019578896 |
| 0,352360817 | 0,019578896 |
| 0,719251978 | 0,039245333 |
| 0,430663221 | 0,021977947 |
| 0,33999728 | 0,016939216 |
| 0,33999728 | 0,016939216 |
| 0,33999728 | 0,016939216 |
| 0,33999728 | 0,016939216 |
| 0,33999728 | 0,016939216 |
| 0,425930658 | 0,020777841 |
| 0,425930658 | 0,020777841 |
| 0,334135258 | 0,015765096 |
| 0,328471948 | 0,014677716 |
| 0,328471948 | 0,014677716 |
| 0,322997416 | 0,013670136 |
| 0,322997416 | 0,013670136 |
| 0,733621891 | 0,030911599 |
| 0,317702376 | 0,012736027 |
| 0,40374677 | 0,015710072 |
| 0,312578145 | 0,011869611 |
| 0,399584432 | 0,014858921 |
| 0,526626222 | 0,019421295 |
| 0,307616587 | 0,011065607 |
| 0,302810078 | 0,010319183 |
| 0,298151461 | 0,009625916 |
| 0,293634015 | 0,008981752 |
| 0,293634015 | 0,008981752 |
| 0,293634015 | 0,008981752 |
| 0,28499772 | 0,007826171 |
| 0,280867318 | 0,007308205 |
| 0,280867318 | 0,007308205 |
| 0,464187903 | 0,011550338 |
| 0,276854928 | 0,006826196 |
| 0,272955563 | 0,006377492 |
| 0,355593486 | 0,007649741 |
| 0,355593486 | 0,007649741 |
| 0,409433344 | 0,008449422 |
| 0,352360817 | 0,007240276 |
| 0,261889797 | 0,005207748 |
| 0,261889797 | 0,005207748 |
| 0,261889797 | 0,005207748 |
| 0,477336083 | 0,009007262 |
| 0,258397933 | 0,004869698 |
| 0,251686298 | 0,00426057 |
| 0,242248062 | 0,00349166 |
| 0,242248062 | 0,00349166 |
| 0,236339573 | 0,003060493 |
| 0,23071244 | 0,002684343 |
| 0,217751067 | 0,001939154 |
| 0,217751067 | 0,001939154 |

| | |
|---|---|
| 0,300462713 | 0,002561901 |
| 0,354509359 | 0,00290481 |
| 0,426868832 | 0,003338246 |
| 0,4249966 | 0,003201338 |
| 0,206168563 | 0,001405654 |
| 0,206168563 | 0,001405654 |
| 0,385668556 | 0,002554133 |
| 0,19775352 | 0,001089053 |
| 0,19379845 | 0,00095924 |
| 0,186344663 | 0,000745124 |
| 0,184569952 | 0,0006997 |
| 0,184569952 | 0,0006997 |
| 0,177796743 | 0,000544568 |
| 0,176180409 | 0,000511606 |
| 0,174593198 | 0,00048068 |
| 0,16999864 | 0,000398877 |
| 0,16999864 | 0,000398877 |
| 0,168520391 | 0,00037489 |
| 0,158851188 | 0,000243392 |
| 0,156289072 | 0,000215276 |
| 0,153808293 | 0,000190461 |
| 0,150231356 | 0,00015858 |
| 0,240246012 | 6,25044E-05 |
| 0,23071244 | 3,81911E-05 |
| 0,225347034 | 2,84623E-05 |

**Genes**

CREBZF;TCERG1;HNRNPU;RSF1;RORB;BMI1;BACH1;CDC73;ELK4;ING4;SDR16C5;MECOM;NKRF;SIN3A;
MAML1;HNRNPU;RORB;IKZF3;ELK4;EPC1;BTRC;RNF111;MEF2A;EOMES;MAP2K1;EBF1;POU3F1;RUNX3;
SIX1;RSF1;RORB;MED17;PHF8;CKS1B;MED14;MECOM;EPC1;BTRC;TEAD1;RNF111;EOMES;MAP2K1;PRC
TCERG1;MAML1;HNRNPU;RORB;IKZF3;BACH1;ELK4;TIAL1;RUVBL1;EPC1;RNF111;MEF2A;EOMES;USP2;
NCKAP1;CHRM3;COX7B;RPS6KA6;CDH2;DPYSL5;SALL4;TRIM3;PCDHAC2;SH3GL2;PCDHAC1;TMOD2;DY
GPM6A;MAP2K1;FZD3;FZD5;USP9X;SEMA3A;SIX1;NDNF;ATP2B2;PROX1;SMARCA1;RUNX3;PHOX2B;RUI
BMPR2;ONECUT2;MAML1;HNRNPU;SIX1;RORB;LITAF;IKZF3;CDC73;MED17;ELK4;MED14;MECOM;NKRF;
RORB;IKZF3;BACH1;ELK4;TIAL1;EFEMP1;SALL4;RUVBL1;EPC1;SOX6;BTRC;RNF111;EOMES;MAP2K1;AR
SIX1;RSF1;RORB;MED17;PHF8;CKS1B;RBM3;MED14;MECOM;EPC1;BTRC;TEAD1;RNF111;EOMES;UTP15
CREBZF;RSF1;RORB;BMI1;ING4;MECOM;NKRF;SIN3A;EPC1;METTL16;BTRC;KLF10;USP47;MAP2K1;SFM
CREBZF;BMPR2;RSF1;RORB;ING4;MECOM;NKRF;SIN3A;EPC1;BTRC;KLF10;USP47;ARID5B;PROX1;CBF
CREBZF;HDAC5;SET;TSHZ3;GATA6;RSF1;RORB;PRICKLE1;ZBTB4;FOXO1;ING4;BCLAF1;MECOM;ZNF706
BTG1;ROCK1;PDE3B;VASH2;GATA6;GAB1;FOXJ2;TNFAIP3;PTPRM;HIPK1;SYNJ2BP;HIF1A;HK2;AMOT;RU
TCERG1;HNRNPU;BMI1;BACH1;CDC73;ELK4;SDR16C5;SIN3A;C1QBP;TRPS1;EPC1;ZNF366;MEF2A;EOM
DLX1;SMURF2;GPC1;SPRY3;SOSTDC1;SPRY1;AGTR2;SLIT2;DAND5;HIPK2;SULF2;SMAD7
DLX1;TFAP2B;BMPR2;SMURF2;GATA6;SOSTDC1;XIAP;ZNF423;DAND5;RNF165;HIPK2;SMAD7
MAP2K1;FZD3;ATP8A2;TENM3;FZD5;ATP2B2;RORB;INHBA;PROX1;SMARCA1;RUNX3;PHOX2B;RUNX1;LR
SRPK2;MBNL2;YTHDC1;C1QBP;TRA2B;CELF3;HNRNPU;METTL16;RBM7;MBNL3;SRSF10;SRSF9
LYN;GPM6A;ATP8A2;TENM3;ROCK1;ARL3;PTPRM;L1CAM;PPP1R9A;RUNX3;PHOX2B;RAB11A;RUNX1;MA
ING4;KAT2B;ZDHHC18;CREBBP;ABHD17B;ZDHHC20;ZDHHC21;ABHD17C;FOXO1
TCERG1;BMPR2;MAML1;SRSF1;GATA6;GLIS3;DTX1;PHAX;RORB;HIF1A;AFF4;CDC73;MED17;CTGF;MED1
PSMD11;TNKS;CUL3;RNF180;TNFAIP3;FBXO40;ZNRF2;TNKS2;RNF217;LONRF3;BTRC;UNKL;RNF111;FBX
GPM6A;USP9X;SEMA3A;DCX;NDNF;PHOX2B;LRP12;MARK1;FGFR1
RXRA;VAPB;DAG1;HIPK2
FBXW4;PSMD11;RANBP3;SMURF2;UBA6;CUL3;BFAR;UBE2G1;RNF126;ITCH;CDC34;ZNRF3;RNF217;RLIM
AMER1;PSMD11;UBA6;CUL3;LTN1;TNFAIP3;ZNRF2;ZNRF3;RNF217;BTRC;RNF111;FBXW4;RANBP3;CRBN
CREBZF;TCERG1;BMPR2;GATA6;GLIS3;DTX1;RSF1;HIF1A;AFF4;CDC73;SALL4;TRPS1;TRIM24;E2F1;EPC
MEF2A;CCDC71L;DLX1;BMPR2;ZBTB16;CREBL2;TMEM64;GDF6;PAX2;CTGF;SFRP1;RAP1A;CREB1;CDH2
LYN;KAT2B;CREBBP;SIN3A;RBBP5;ZBTB16;AGO1;PTK2B;CDC73;RUNX1;TNRC6B
SRPK2;MBNL2;CELF1;YTHDC1;MYOD1;TRA2B;DYRK1A;METTL16;MBNL3;SRSF10
AMER1;CSNK1G3;FZD3;FZD5;TNKS;CSNK1A1;RBX1;LRP6;SFRP1;TNKS2;APC;CTNNB1;STRN;BTRC;WLS
NCOA2;NPFFR1;MME;TIPARP;PTPN11;ADRA2A;IL18BP;MAPK10;SFRP1;DPYSL3;IL1RAPL1;GNRHR;MAPK
TGFBR3;HEYL;BMPR2;PROX1;TBX5;DAND5;SMAD7
DLX1;USP15;BMPR2;SMURF2;USP9X;SMAD9;GDF6;PHOX2B;SMAD7;TGFBR3;SFRP1;PDCD4;BMPR1A
AMER1;FZD5;APC;CSNK1A1;CTNNB1;LRP6
ABCA1;CRP;ZBTB16;HNRNPU;RORB;LDLRAD4;TMEM64;TBX5;FOXO1;CDC73;SMAD7;PPP2CA;SFRP1;EF
SRPK2;MAP3K2;MAP2K1;HPGD;NEK7;HNRNPU;PROX1;INHBA;RUNX3;HIPK2;CKS1B;LRP6;SFRP1;RBL1;
CCDC71L;SFRP1;CREB1;ZBTB16;CARM1;CREBL2;TMEM64;WDFY2
LYN;EOMES;NCKAP1;COX7B;EIF2B2;MYRF;ZBTB16;PTPN11;PROX1;SMARCA1;S100B;HPCAL4;GRIN2B;F
HEYL;TMEM65;SCN5A;TBX5;DAND5
TFAP2B;FZD3;SEMA3A;CTNNB1;PHOX2B
TGFBR3;HEYL;PDCD4;HAS2;SPRY1
PSMD11;TNKS;UBA6;CUL3;RNF180;KLHL32;XIAP;BACH1;HIF1A;CDC73;FBXO40;ZNRF2;ZNRF3;TNKS2;RN
FZD3;SEMA3A;CTNNB1;PHOX2B
RAP2C;RAP2A;RAP2B;RAP1A
PLXNA2;SLIT2;PLXNA3;RNF165
FZD3;SEMA3A;CTNNB1;PHOX2B
SRPK2;SART3;CELF1;YTHDC1;SRSF1;SNRPD3;SRSF10;SRSF9
CCDC71L;SFRP1;CREB1;ZBTB16;CARM1;E2F1;CREBL2;WDFY2;TMEM64;TRIB2;FOXO1
AMER1;PSMD11;CUL3;LTN1;UBE2J1;ZNRF2;BTRC;RNF111;FBXW4;CRBN;SMURF2;CSNK1A1;EDEM1;YOD
LYN;SFRP1;RC3H1;TNFAIP3;INHBA
AMER1;PSMD11;CRBN;SMURF2;CSNK1A1;CUL3;LTN1;BFAR;BTBD9;RBX1;RNF126;ITCH;ZNRF2;CDC34;A

USP13;CSNK1A1;RNF180;RNF217;TMTC3;LONRF3;PRICKLE1;TRIB2;RBX1;PIAS1;SMAD7
DYRK3;USP15;MAML1;TNKS;RPS6KA6;EFEMP1;EEF2K;ERBB4;PTK2B;MAPK1;PIM3;MAP3K9;CLSPN;MAF
SH3KBP1;SEMA3A;GAB1;NRXN3;PTPRM;PTPN11;L1CAM;ENAH;EFNA3;EFNB3;CREB1;KIF5C;GPC1;PLXN
ING4;KAT2B;CREBBP;NAA30;ESCO1;NAA35;FOXO1
AMER1;APCDD1;SENP2;ESR1;CDC73;RUNX1;LRP6;SULF2;PPP2CA;SFRP1;RSPO2;SOSTDC1;BTRC;WLS
AMER1;USP47;PSMD11;SMURF2;CSNK1A1;TNKS;CUL3;GSKIP;XIAP;PRICKLE1;TMEM64;DKK3;RBX1;LRP
LYN;DLX1;TENM3;NDNF;PROX1;CBFA2T2;ADRA2B;HEYL;RAP1A;DPYSL3;PTK2B;ATOH1;FGFR1
RXRA;VAPB;DAG1;HIPK2;KPNA1
SPRED1;EFNA3;ROCK1;AGO1;PDE3B;FOXJ2;PTPRM;CTNNB1;SYNJ2BP;AMOT
ALAS2;SOD2;HIF1A
PCGF3;RLIM;HNRNPU
USP47;USP15;USP1
LYN;RC3H1;PAX5;RUNX1
GPC1;SPRY3;SPRY1;SULF2
NPR3;NOS1AP;ATP2B4;ZDHHC21;AGTR2;CALM1;HIF1A
AMER1;USP47;PSMD11;SMURF2;TNKS;GSKIP;XIAP;CDC73;LRP6;SULF2;SFRP1;TNKS2;TBL1XR1;RUVBL
LYN;IGBP1;TGFBR3;ROCK1;ERBB4;ACTN4;RAF1;EREG
ITCH;SF3B3;SMURF2;APC;TIPARP;HECW2;AGAP2;TNFAIP3;DTL;SORL1;FOXO1;AZIN1
LYN;IGBP1;SPRED1;RPS6KA6;CNKSR3;DAG1;SPRY3;SPRY1;SYNJ2BP;SORL1;FOXO1
SRPK2;MTMR3;SORT1;MTMR9;PROX1;BMI1;HIF1A;PHOX2B;MAPK10;NPAT;DDX19B;SART3;CDK1;MAPK1
BTG1;VASH2;GATA6;GAB1;HIPK1;HIF1A;HK2;RUNX1;MTDH;HIPK2;RAP1A;SP1;ADAM12
SPRED1;EFNA3;ROCK1;AGO1;PDE3B;FOXJ2;PTPRM;CTNNB1;SYNJ2BP;AMOT
AMER1;ITCH;SMURF2;APC;TIPARP;HECW2;TNFAIP3;DTL;SORL1;ADRA2A;FOXO1
TGFBR3;USP15;GDF6;SMAD7
LYN;EFNA3;SIPA1L1;APH1B;EFNB3;ROCK1;MMP2;PTPN11;CRK;GRIN2B;AP2M1
TGFBR3;HEYL;BMPR2;PROX1;SMAD7
NAPA;NAPB;GRID1;SLC17A6;SLC1A4
ALAS2;SOD2;HIF1A
TFAP2B;NPR3;HAS2
BHLHB9;CAPRIN1;LRP8
DLX1;TFAP2B;CREBBP;RUNX3;CTGF;RUNX1;SMAD7;PPP2CA;APC;TRPS1;DAG1;OCIAD1;RAF1;FGFR1
ITCH;HNRNPK;SMURF2;APC;TIPARP;HECW2;TNFAIP3;INHBA;DTL;SORL1;FOXO1;EREG
RAP2A;RALA;SIPA1L1;CXADR;EFS;GAB1;S1PR1;PARVA;CDC42BPA
KANK2;GPAM;CARM1;ESR1;ZNF366;RUNX1
ITCH;SMURF2;APC;TIPARP;HECW2;TNFAIP3;DTL;SORL1;FOXO1
TNKS2;TNKS;XRCC5;NEK7;HNRNPU;MAPK1;HNRNPC;DCP2
TFAP2B;MME;ARL3;ZBTB16;SIX1;HAS2;PROX1;PAX2;SULF2
EFEMP1;ZBTB16;TRPS1;GDF6;SOX6
SRPK2;SF3B3;CELF1;CELF3;SRSF1;HNRNPU;HNRNPR;LSM5;DHX40;PPP2CA;RBM3;DDX19B;HNRNPK;P/
AMER1;USP47;PSMD11;SMURF2;TNKS;GSKIP;XIAP;LRP6;SFRP1;TNKS2;TBL1XR1;RUVBL1;WLS
SORT1;MYOD1;ADAM12;KCNH1
TFAP2B;SEMA3A;PHOX2B;PHACTR4
KAT2B;CREBBP;NAA30;NAA35
OCLN;RAP1A;MYO1C;RUNX1
BMPR2;ZBTB16;TRPS1;SOX6
TNKS2;XRCC5;TNKS;TERF2IP
DLX1;SMURF2;SOSTDC1;DAND5;HIPK2;SMAD7
GJC1;SCN5A;SCN3B
LYN;EFNA3;SORL1
KAT2B;CREBBP;ESCO1
LYN;MAP2K1;MAPK1
EPS8;CAPZB;ADD2
ID2;ID4;DTX1;HOOK3;SRGAP2;SORL1

ANGEL2;E2F1;IGF2BP1;HNRNPU;HNRNPC;TARDBP
BTG1;SP1;ADAM12;VASH2;GATA6;GAB1;HIPK1;HIF1A;HK2;RUNX1;MTDH;HIPK2
KAT2B;HEYL;CREBBP;ITCH;APH1B;MAML1;DTX1;ZNF423
OTUD4;USP13;USP47;USP37;USP15;PSMD11;SMURF2;USP9X;TNKS;USP2;TNFAIP3;YOD1;HIF1A;ESR1;S
RBM3;GRM5;YTHDC1;RC3H1;METTL16;EIF4E;PUM2
BHLHB9;CAPRIN1;IL1RAPL1;LRP8
OCLN;MYO1C;ROCK1;RUNX1
NPR3;NOS1AP;AGTR2;HIF1A
CDK1;DTL;RAB11A;PHOX2B
KANK2;BTG1;ATP8A2;BMPR2;TNFAIP3;CDC73;ING4;ERBB4;E2F1;PTK2B;LYN;KLF10;TFAP2B;MAP2K1;PR
CCND3;APC;CDC37;CCNYL1;MAPRE3;GTF2H1;PROX1;GTPBP4;CKS1B
KLF10;USP15;BMPR2;SMURF2;HPGD;USP9X;PALM2;SMAD9;INHBA;GDF6;SMAD7;TGFBR3;PDCD4;RNF1
CELF1;YTHDC1;SRSF1;SRSF10;SRSF9
TNKS;SIN3A;ID2;E2F1;ID4;RSF1
SHC4;NCKAP1;SHC2;BMPR2;ROCK1;IRS4;GIGYF2;FOXO1;TIAL1;SPRED1;APH1B;SIPA1L1;EFNB3;EFS;E
PDE1B;PDE3B;PDE5A
MOCS3;DCUN1D5;UBA6;CUL3;DCUN1D1;KLHL32;XIAP;BACH1;HIF1A;CDC73;ZNRF3;TRIM24;BTRC;RNF1
USP13;USP47;USP37;USP15;PSMD11;SMURF2;USP9X;TNKS;USP2;TNFAIP3;YOD1;SENP2;HIF1A;ESR1;S
TGFBR3;USP15;BMPR2;SMURF2;USP9X;PDCD4;SMAD9;GDF6;SMAD7;BMPR1A
LYN;TGFBR3;DYRK3;ALAS2;INHBA;HIPK2
MYRF;CDH2;GPC1;PHOX2B;PAX2
PPP2CA;SFRP1;LDLRAD4;TBX5;SMAD7
MMP2;FOXJ2;PDCD4;PRKG1;BMPR1A
TNKS2;XRCC5;TNKS;TERF2IP
EDEM3;MAN1A2;EDEM1;ST8SIA3
TNKS2;TNKS;NEK7;MAPK1
BMPR2;ZBTB16;GDF6;SOX6
HEYL;KAT2B;CREBBP;MAML1
CDK1;DTL;RAB11A;PHOX2B
PPP2CA;USP37;ESCO1;XRCC5;USP1;TERF2IP;KPNA1
SRPK2;CCND3;USP2;CCNYL1;MAPRE3;PROX1;EIF4E;PHOX2B;CKS1B;LRP6
DOCK5;C1QBP;CAPRIN1;IL1RAPL1;PLXNA2;SLIT2;CRK;PLXNA3
CSNK1A1;RNF180;RNF217;LONRF3;PRICKLE1;TRIB2;RBX1;PIAS1;SMAD7
FZD3;FZD5;AGO1;CTNNB1;CALM1;TNRC6B
ING4;KANSL1;PHF20;MYOD1;RUVBL1;MSL2;EPC1
CCND3;CCNYL1;MAPRE3;PROX1;CKS1B
TNKS2;TNKS;HNRNPU;HNRNPC;DCP2
CPSF7;SF3B3;SRSF1;HNRNPU;PPWD1;HNRNPR;LSM5;ELAVL2;U2SURP;DHX40;HNRNPK;SART3;PABPN
LYN;DUSP19;FAM129A;LDLRAD4;PPM1E;SMAD7;LRP6;SFRP1;SPRED1;CHAD;CNKSR3;DAG1;TERF2IP;S
TFAP2B;MME;ARL3;SIX1;HAS2;PROX1;PAX2;SULF2
HEYL;ROCK1;ID2;IL1RAPL1;ID4;SIX1;DTX1;ADRA2B;ATOH1;FGFR1
IGBP1;MTMR3;MTMR9;SMG7
TGFBR3;ERBB4;TBX5;BMPR1A
DLX1;SMURF2;SMAD7;DKK3
LYN;RAP1A;TENM3;DPYSL3;PLXNA2;PTK2B;NDNF;SLIT2;CBFA2T2;LRP8;PLXNA3
RAP2C;SFRP1;DPYSL3;DAG1;LDLRAD4;SLIT2;SRGAP2;TBX5;CRK;GTPBP4;SMAD7
KANK2;BTG1;ATP8A2;BMPR2;HPGD;PDE3B;CDC73;CTGF;ING4;PPP2CA;SERTAD2;ERBB4;PLXNA2;PTK2
NCKAP1;RAB3C;RAB2B;SHC2;ROCK1;RHOBTB3;RND3;RAB11A;ADRA2A;EPS8;RAP2C;GNA13;RAP2A;RA
RNF126;ITCH;CDC34;TNFAIP3;BFAR;UBE2G1;UBE2K
CPSF7;SF3B3;CELF1;SRSF1;HNRNPU;PPWD1;HNRNPR;GTF2H1;LSM5;GTF2H5;ELAVL2;U2SURP;DHX40;
MMP2;ID2;FOXJ2;IL6R;EREG;BMPR1A
ARHGEF10;SFRP1;PPM1E;SORBS3;ARHGEF5;CTGF
SFRP1;FZD5;LRP6

USP37;TNFAIP3;YOD1
TFAP2B;NPR3;HAS2
SMURF2;C1QBP;INHBA
EPS8;CAPZB;ADD2
RPS6KA6;FZD3;CTNNB1
CHURC1-FNTB;FNTB;AIPL1
ABCA1;SYT7;LRP6
ADRA2B;ADRA2A;EREG
BTG1;CELF1;SIX1;RORB;BMI1;BACH1;ELAVL2;EFEMP1;SART3;SALL4;METTL16;SOX6;NCOA2;MAP2K1;M
TNKS2;TNKS;XRCC5;TERF2IP;ESR1
DLX1;BMPR2;SMAD9;MTSS1L;PTPN11;PHOX2B;SMAD7;ACAP2;SFRP1;RAP1A;HAS2;CTNNB1;SOX6;BMP
SEMA3A;LDLRAD4;RND3;MYPN;AMOT;ERBB4;DPYSL3;DAG1;PLXNA2;HAS2;PTK2B;SLIT2;SRGAP2;PLXN
AMER1;SFRP1;FZD3;FZD5;APC;CSNK1A1;CTNNB1;TMEM64;LRP6
SEPT11;CPSF7;MAT2A;KCNC2;TRPS1;CLDN1;GRIN2B
PPP2CA;LYN;SPRED1;DUSP19;PDCD4;SPRY3;SPRY1;SORL1
CNOT6;CELF1;AGO1;TNRC6B
PDE1B;PDE3B;PDE5A;NUDT4
JPH3;ATP2B4;JPH1;GRIN2B
CREBZF;KAT2B;HDAC5;MYO1C;TDG;DNMT3A;EPC1;DEK;BMI1
ABCA1;NCOA2;CREBBP;RBL1;RXRA;TBL1XR1;SIN3A;ID2;CARM1;IRS4;CTGF
SRPK2;PABPN1;YTHDC1;METTL16;SRSF10
TFAP2B;SMURF2;XIAP;SMAD7;DKK3
OCLN;APC;FRMPD2;STRN;CLDN1
USP9X;SEMA3A;TMEM108;CTNNB1;SH3GL2
MBNL2;TRA2B;CELF3;HNRNPU;MBNL3;RBM7
SDR16C5;SET;SIN3A
ARL3;RORB;MAPRE2
BHLHB9;SET;AGAP2;SIX1;PTK2B;NDNF;SOD2;HIF1A
SRPK2;CPSF7;SF3B3;SRSF1;HNRNPU;PPWD1;HNRNPR;LSM5;ELAVL2;U2SURP;DHX40;HNRNPK;SART3;
TGFBR3;SFRP1;ZBTB16;PGM3;INHBA;BMI1;HIPK1;RUNX3;RUNX1
SRPK2;TFAP2B;BHLHB9;SET;AGAP2;SIX1;PTK2B;CTNNB1;NDNF;SOD2
TMOD3;MYOD1;TMOD2;LMOD3
MCTS1;DYRK3;CDK1;PELO
GPC1;SPRY3;SPRY1;SULF2
SFRP1;MYOD1;NRIP1;ESR1
DYRK3;MAML1;ADK;RPS6KA6;PTK2B;MAPK1;PIM3;MAP3K9;MARK1;LYN;SRPK2;MAP3K2;MAP2K1;CSNK1
DLX1;SEMA3A;ID2;DPYSL3;ID4;DTX1;CBFA2T2;ITM2C
DLX1;SMURF2;PRDM16;SOSTDC1;LDLRAD4;DAND5;SNX6;HIPK2;SMAD7
LYN;GPM6A;ROCK1;VAPA;TMEM108;PTPRM;NPTXR;L1CAM;PPP1R9A;SH3GL2;LRP12;RAB11A
PPP2CA;CCND3;MAP2K1;ATP2B4;ZFP91;CCNYL1;MAPRE3;PROX1;CALM1;ADRA2A;FGFR1;CKS1B
CREBBP;RBBP5;RUVBL1;CTNNB1;CDC73
ZDHHC18;ABHD17B;ZDHHC20;ZDHHC21;ABHD17C
TFAP2B;BMPR2;HPGD;SMAD7;BMPR1A
SFRP1;MYOD1;NRIP1;CTNNB1;ESR1
IGBP1;ITCH;MECOM;DUSP19;FOXO1
CCND3;CCNYL1;MAPRE3;PROX1;CKS1B
DLX1;SMURF2;PRDM16;LDLRAD4;DAND5;SNX6;SMAD7
NPAT;USP37;CDC34;RANBP3;CUL3;RPA4;E2F1;INHBA;BACH1;EIF4E;PHF8
BMPR2;GATA6;ZNF423;RNF165
MED14;ABHD2;ESR1;MED17
FBXW4;TFAP2B;TBX5;LRP6
SLC24A2;TSHZ3;PTK2B;GRIN2B
LYN;RAP1A;TENM3;SIPA1L1;CDH2;SEMA3A;DPYSL3;IL1RAPL1;PTK2B;NDNF;CBFA2T2;ITM2C

PRDM8;EIF2B2;MYRF
SFRP1;S1PR1;SYT7
ZBTB16;GDF6;SOX6
SMURF2;PRDM16;LDLRAD4;RNF111;DAND5;DKK3;SNX6;HIPK2;SMAD7
AMER1;ITCH;PFKFB3;ROCK1;TFEB;RC3H1;HIF1A;FOXO1;ADRA2A;MTDH;TPCN1
SRPK2;PPP2CA;DDX19B;SF3B3;TRA2B;CELF3;SNRPD3;HNRNPC;TARDBP;SRSF10;SMNDC1
EPS8;EFS;TPM3;TMOD3;PALM2;DPYSL3;TMOD2;RHOBTB3;PROX1;PPP1R9A;RND3;LMOD3
SRPK2;STAU1;VAPA;VAPB;FKBP6
CREBBP;CUL3;BTRC;SENP2;CDC73
SCOC;SESN3;ROCK1;TFEB;PAFAH1B2;FOXO1;MTDH;TPCN1
EPS8;EFEMP1;ERBB4;CDC37;GAB1;PTK2B;MAPK1;PTPN11;EREG
SIPA1L1;TDG;PLXNA2;RAB3GAP2;RAPGEF6;CRK;PRKG1;ARHGEF5;PLXNA3
ACAP2;RAP1A;TMEM108;SH3GL2
NCKIPSD;TRPS1;DAG1;KPNA1
KANK2;DYRK1A;SLIT2;KANK4
BMPR2;GATA6;PARVA;BMPR1A
RNF126;SH3KBP1;ERBB4;SPRY1;SH3GL2;EREG
TNKS2;TNKS;XRCC5;NEK7;MAPK1
GJC1;SCN5A;SCN3B
FBXW4;TBX5;LRP6
DCUN1D5;DCUN1D1;RBX1
ADRA2B;ADRA2A;RAB11FIP5
SEMA3A;PLXNA2;PLXNA3
MCTS1;DYRK3;PELO
CNKSR3;SCN5A;SCN3B
C1QBP;SRSF10;SRSF9
MYOD1;GPC1;LMOD3
SEMA3A;PLXNA2;PLXNA3
BMPR2;GATA6;INHBA;ZNF423;GDF6;RNF111;RNF165;HIPK2;BMPR1A
XRCC5;ID2;TNFAIP3;CTNNB1;IL6R;EREG
ARHGEF10;SFRP1;PPM1E;SORBS3;ARHGEF5;CTGF
LYN;DYRK3;ALAS2;ZBTB16;INHBA;HIPK2
HEYL;FZD3;BHLHB9;SEMA3A;CTNNB1;ADRA2B;ATOH1;FGFR1
TNKS2;TNKS;NEK7;MAPK1
OTUD4;USP37;TNFAIP3;YOD1
BHLHB9;SIPA1L1;CAPRIN1;LRP8
ARL5B;VPS13C;SORL1;ARL5A
NPR3;NOS1AP;AGTR2;HIF1A
AMER1;DCUN1D5;CUL3;DCUN1D1;XIAP;PRICKLE1;BMI1;SENP2;SMAD7
TRA2B;ZNF236;FOXO1;HK2;PAX2
ATL3;CALCOCO2;RAB3GAP2;SORBS3;PPARGC1B
GPM6A;SFRP1;PSMD11;PAX2;LRP6
ITCH;SMURF2;HECW2;CUL3;RNF217;TRIP12;BTBD9;RBX1
SF3B3;CELF1;SRSF1;HNRNPR;SNRPD3;LSM5;METTL16;DCP2;EREG;SRSF9
SORT1;VPS13C;ARL3;SNAP23;VTI1A;VPS54;SORL1
MEF2A;BTG1;CDH2;MYOD1;CTNNB1
TNKS2;TNKS;XRCC5;NEK7;MAPK1
REEP1;ATL3;VAPB;TMEM33;ATL2
FBXW4;CREBBP;CTNNB1;TBX5;LRP6
TGFBR3;ERBB4;BMPR1A
FZD3;CTNNB1;PROX1
RCAN1;RCAN2;ATP2B4
ZBTB16;PTK2B;CDC73

PRDM8;EIF2B2;MYRF
ITCH;C1QBP;TNFAIP3
MMP2;FOXJ2;BMPR1A
SLIT2;PRKG1;BMPR1A
KANSL1;PHF20;MSL2
CDH2;PHOX2B;PAX2
NDC1;TMEM33;ANO6
REEP1;TMEM33;ATL2
ING4;RPS6KA6;HIPK2
AMER1;FZD5;APC;CSNK1A1;CTNNB1;LRP6
STIM2;CNKSR3;ANO6;SCN5A;SCN3B;ADRA2A
SFRP1;E2F1;CTNNB1;BMI1;CDC73;EREG
CREBBP;MAML1;RSF1;GTF2H1;RORB;TBX5;ESR1;GTF2H5;CTGF;MED17;KAT2B;MED14;RXRA;CDK1;TFA
MAP3K2;MAP2K1;XRCC5;DUSP19;LRP8;ADRA2A;EREG;PPP2CA;GRM5;RAP1A;VAPB;PTK2B;MAPRE3;CL
SET;PSMD11;SERBP1;E2F1;IGF2BP1;HNRNPU;RC3H1;METTL16;ZC3H14;GIGYF2;DCP2;PUM2
MYOD1;LMOD3
LYN;EFNA3
RUNX3;RUNX1
MEF2A;PROX1
SFRP1;INHBA
TMEM108;SH3GL2
SCN5A;SCN3B
CDC34;PDE3B
ARL3;MAPRE2
SLIT2;HIF1A
TGFBR3;HIPK1
SFRP1;SLIT2
GATA6;HIF1A
TGFBR3;BMPR1A
BMPR2;SOD2
TSHZ3;PHOX2B
ATP2B4;FOXO1
PTK2B;CD244
PHOX2B;PHACTR4
SMURF2;C1QBP
TFAP2B;PAX2
PDCD4;EREG
BMPR2;SULF2
SFRP1;BTG1
LYN;EFNA3
ADRA2B;ADRA2A
PTPRM;SLIT2
KLF10;TGFBR3;DLX1;SFRP1;USP15;HPGD;USP9X;SOX6;SMAD7;BMPR1A
NKAIN1;CNKSR3;ATP2B4;SCN5A;SCN3B
PDCD4;ZFP91;TERF2IP;ACTN4;TRIM44
OCLN;APC;FRMPD2;STRN;CLDN1
ST6GAL2;ALG6;MGAT5;OSTC;GFPT1;PGM3;ST6GALNAC3;UBE2J1
ARHGEF10;SFRP1;ROCK1;PPM1E;SORBS3;ARHGEF5;CTGF
NAPA;AMER1;CREBBP;ST13;CSNK1A1;CUL3;TNFAIP3;ANO6;CDC73;SH3PXD2A;APC;RBBP5;TRA2B;RUV
ID2;E2F1;ATP2B4;CDC73
BMPR2;FAM129A;DUSP19;CREBL2;LRP8;CCND3;RAP1A;ERBB4;PTK2B;MAPK1;IL6R;LYN;GPR37;XRCC5;
B3GALNT2;GALNT7;SERP1;ALG6;TNKS2;TNKS;MGAT5;ST8SIA3;GFPT1;TET3;DAG1;PGM3
ING4;KANSL1;PHF20

SFRP1;PIM3;ADRA2A
KCNC2;NDNF;AGTR2
ING4;RPS6KA6;HIPK2
TSFM;C1QBP;NSUN3
C1QBP;SRSF10;SRSF9
ING4;KANSL1;PHF20
HEYL;BMPR2;BMPR1A
SPRED1;CALM1;SMAD7
SEPT10;SEPT11;SEPT12
SFRP1;INHBA;IKZF3
CREBBP;MAML1;GTF2H1;RORB;TBX5;ESR1;GTF2H5;CTGF;MED17;KAT2B;MED14;RXRA;CDK1;TEAD1
EDEM3;MAN1A2;EDEM1;ST8SIA3;ST6GALNAC3;HIF1A
KAT2B;HDAC5;RUVBL1;TFAM;RSF1;HNRNPC;DEK;NUDT5;SMARCA1;ESR1;GATAD2B
CSNK1A1;RNF180;RNF217;LONRF3;PRICKLE1;TRIB2;RBX1;PIAS1;SMAD7
AGO1;RC3H1;METTL16;LSM5;DCP2
AP3M2;TMEM108;KIF1B;HIF1A;TRAK2
LYN;FAM129A;HNRNPU;TERF2IP;SLIT2;INHBA;TARDBP;LRP6
SH3KBP1;USP9X;SEMA3A;OMD;GAB1;NRXN3;PTPN11;L1CAM;S100B;PAX2;ENAH;EFNA3;EFNB3;CREB1;I
CREBZF;DNMT3A;EPC1;BMI1
SF3B3;TRA2B;SRSF10;SMNDC1
XRCC5;DUSP19;PPM1E;EREG;LRP6;RAP2C;SPRED1;RAP1A;RAP2B;CHAD;PTK2B;MAPRE3;TRIB2
KAT2B;SIN3A;RBBP5;AGO1;RUNX1;TNRC6B
EOMES;TGFBR3;HEYL;CTNNB1;HIF1A
RNF126;CUL3;DTL;CDC73;RBX1
ITCH;RNF126;ZFP91;BFAR;UBE2G1
SPRED1;MAPK1;CALM1;UBE2K;SMAD7
NDC1;FYTTD1;NXF1;DDX19B;KIF5C;SRSF1;SMG7;EIF4E;SRSF10;SRSF9
CREBZF;MEF2A;E2F1;EPC1;DTX1;SMARCA1;ESR1;PAX2
NDC1;FYTTD1;NXF1;DDX19B;RANBP3;SRSF1;PHAX;SMG7;EIF4E;SRSF10;SRSF9
LYN;TENM3;RAP1A;DPYSL3;PTK2B;NDNF;CBFA2T2
LYN;FAM129A;ATP2B4;TERF2IP;SLIT2;TARDBP;LRP6
ROCK1;SELE;ADD2
ALAD;ALAS2;NFE2L1
MAML1;MYOD1;LMOD3
TFAP2B;PROX1;BMPR1A
BCLAF1;SUB1;CTNNB1
TGFBR3;ERBB4;BMPR1A
ROCK1;SELE;ADD2
TRA2B;CELF3;CDC73
CTNNB1;SMAD7
ANO6;AZIN1
BMPR2;BMPR1A
IGBP1;FOXO1
BMPR2;PROX1
SLIT2;PHOX2B
RXRA;ACTN4
MYO1C;GAB1
RXRA;ACTN4
FZD3;CTNNB1
MYO1C;GAB1
ADRA2B;ADRA2A
BMPR2;BMPR1A
ERBB4;INHBA

SCN5A;SCN3B
OTUD4;ITCH
HAS2;MTSS1L
MYOD1;LMOD3
ADAM12;KCNH1
PDE1B;PDE3B
BMPR2;PROX1
SRPK2;HIPK2
NDC1;FYTTD1;NXF1;DDX19B;PABPN1;SRSF1;SMG7;EIF4E;SRSF10;SRSF9
NPAT;ID2;E2F1;BACH1
KIF5C;KIF1B;HOOK3;DYNLL2
SFRP1;BMPR2;ZBTB16;S1PR1;LRP6
EFNB3;SEMA3A;DPYSL3;OSTN;CBFA2T2;ITM2C
LYN;EFEMP1;ERBB4;DYRK1A;PTK2B;FGFR1
RAB3C;RAB2B;RALA;IL1RAPL1;SYTL4;STXBP5L
RAP2C;SFRP1;DPYSL3;DAG1;LDLRAD4;SLIT2;SRGAP2;TBX5;GTPBP4;SMAD7;BMPR1A
LYN;NCKAP1;GPM6A;ROCK1;PTPRM;L1CAM;PPP1R9A;RAB11A;VAPA;TMEM108;NPTXR;SRGAP2;SH3GL[illegible]
AMER1;CUL3;XIAP;PRICKLE1;SENP2;PIAS1;SMAD7
MBNL2;CELF1;TRA2B;MYOD1;MBNL3
CDH6;GJC1;OCLN;CDH2;APC;FRMPD2;STRN;CLDN1
TRA2B;ZNF236;PAX2;RAB11FIP5
JPH3;CALM1;JPH1;ADRA2A
GPAM;CARM1;ESR1;RUNX1
ITCH;C1QBP;RC3H1
SCN5A;SCN3B;SMAD7
ST6GAL2;ST8SIA3;ST6GALNAC3
FZD3;HNRNPU;CTNNB1
ANGEL2;HNRNPC;TARDBP
CELF1;RAI2;FGFR1
NAPA;NAPB;RIMS3
LYN;PAX5;RUNX1
SEPT10;SEPT11;SEPT12
NAA30;NAA35;METAP2
DOCK5;SFRP1;C1QBP;PTK2B;NDNF;CRK
FYTTD1;CREBZF;SRSF1;HNRNPU;DTX1;SMG7;RBM3;MRPL42;NXF1;PABPN1;E2F1;EIF4EBP2;EPC1;SRS[illegible]
MED14;NRIP1;CTNNB1;ESR1;PPARGC1B;MED17;PIAS1
TGFBR3;SFRP1;PTPRM;SYNJ2BP;RUNX3;CDC73;EREG
PPP2CA;MAP2K1;CDC37;GTF2H1;CALM1;TRIB2;GTPBP4;LRP6
ROCK1;ERBB4;PLXNA2;RAF1;RND3;CRK;MYPN;AMOT;EREG;PLXNA3
ABCA1;NCOA2;CREBBP;PSMD11;RXRA;TBL1XR1;SIN3A;ID2;CARM1;IRS4;AZIN1;CTGF
RNF126;SH3KBP1;SPRY1;SH3GL2;SNX6
ERBB4;SEMA3A;SRGAP2;AMOT;PHACTR4
CUL3;CDK1;DTL;RAB11A;PHOX2B
MED14;NRIP1;CTNNB1;MED17;PIAS1
LYN;RPS6KA6;CNKSR3;SPRY1;SYNJ2BP
SF3B3;PDE3B;AGAP2;AZIN1;ADRA2A
CHRM3;DYRK3;MAML1;CHURC1-FNTB;PTPRM;DCAF7;RPS6KA6;SERP1;CDH2;FNTB;UBXN7;PIM3;MAP3[illegible]
KANK2;HEYL;SFRP1;ZNF366
TNFAIP3;TERF2IP;PIM3;ADRA2A
BMPR2;RUNX3;RUNX1;SULF2
GRM5;LRP8;ADRA2A;EREG
PDCD4;TERF2IP;ACTN4;TRIM44
ITCH;MECOM;DUSP19;PDCD4

GJC1;SCN5A;SCN3B;SCN1A
ERBB4;SEMA3A;PHOX2B;PHACTR4
OTUD4;USP13;TNFAIP3;YOD1
PRICKLE1;PAX2;LRP6;PHACTR4
KCNC2;FOXO1
SFRP1;BTG1
RAP2A;RAPGEF6
MAP2K1;MAPK1
SORT1;RAB11A
SESTD1;ADRA2A
LYN;TNFAIP3
SFRP1;TNFAIP3
ERBB4;ID2
MCTS1;PELO
SIX1;PAX2
DOCK5;PRKG1
SEMA3A;SLIT2
PCGF3;BMI1
SESTD1;NOS1AP
RAP2A;RAPGEF6
TGFBR3;SPRED1
SPRED1;DYRK1A
RC3H1;SMAD7
DFFA;FZD3
SEMA3A;SLIT2
DOCK5;BMPR2;SMURF2;ROCK1;SGIP1;ZBTB16;INHBA;HIF1A;RAB11A;ADRA2A;EREG;LRP6;C1QBP;CTNI
C1QBP;SRSF10;SRSF9
TGFBR3;S1PR1;BMPR1A
TFAP2B;XIAP;SLIT2
SLIT2;SORL1;BMPR1A
RC3H1;METTL16;GIGYF2
ALAD;ALAS2;NFE2L1
SCN5A;SCN3B;SCN1A
TGFBR3;HEYL;CTNNB1;HIF1A;LRP6
RCAN1;JPH3;RCAN2;CALM1;JPH1
SEMA3A;DPYSL3;CBFA2T2;GRIN2B;ITM2C
IGBP1;MAP2K1;RAP2A;MAPK1;FOXO1
GRM5;RIMS3;CDH2;GRID1;TMEM108;EIF4EBP2;BTBD9;LRP8
PPP2CA;SFRP1;DAG1;CTNNB1;LDLRAD4;TBX5;SMAD7
EPS8;KANK2;NCKAP1;FCHSD2;PTK2B;SLIT2;KANK4
RPS6KA6;DAG1;CDK1;TRIP12
PRICKLE1;PAX2;LRP6;PHACTR4
KCNIP2;SCN5A;SCN3B;SCN1A
SFRP1;FZD3;PSMD11;FZD5;SMURF2;AGO1;CTNNB1;PRICKLE1;CALM1;AP2M1;TNRC6B
SMURF2;PRDM16;LDLRAD4;DAND5;SNX6;SMAD7
SIPA1L1;EFNB3;CDH2;PLXNA2;SLIT2;PLXNA3
BHLHB9;SET;AGAP2;SIX1;PTK2B;NDNF;SOD2;SORL1
AMER1;PSMD11;CSNK1A1;CUL3;PRICKLE1;TMEM64;APCDD1;DKK3;RBX1;LRP6;SFRP1;ZNRF3;APC;SOS
DLX1;BMPR2;SMURF2;ZBTB16;C1QBP;MIEF1;SOX6;CTGF;BMPR1A;LRP6
EPS8;NCKAP1;ELMO1
HEYL;BMPR2;BMPR1A
GABRA1;GABRB1;GABRA3
GNA13;ESR1;FGFR1

SRPK2;TFAP2B;CTNNB1
AGO1;SNIP1;PUM2
NCKAP1;SHC2;BMPR2;ROCK1;ELMO1;PTK2B;CRK
ZBTB16;ARID5B;SLC39A13;SULF2
LYN;PTK2B;SLIT2;IL6R
BMPR2;HNRNPU;HNRNPC;DCP2
ACAP2;AGAP2;AGFG2;APPL1
CHST7;B3GAT2;NDNF;CHST3
NAPA;SYT5;NAPB;GABRA1;CHRM3;GABRB1;GRID1;KCNIP2;GABRA3;PCDHB15;SLC1A4;NSG1;GRIN2B;L
TGFBR3;HEYL;PROX1;SMAD7;BMPR1A
SIX1;CTNNB1;SLIT2;PAX2;LRP6
NECAB3;ITM2C
LYN;TNFAIP3
KCNC2;FOXO1
AGTR2;SOD2
IGBP1;C21ORF59
PLXNA2;PLXNA3
SNX1;SNX8
VAPA;VAPB
LETM1;MAT2A
LYN;TNFAIP3
BMPR2;BMPR1A
ROCK1;SORL1
CREB1;SMAD7
SP1;FGFR1
HIF1A;TRAK2
CTNNB1;PAX2
SDR16C5;SET
METTL16;GIGYF2
AZIN1;OTC
EPS8;DPYSL3
TBX5;LRP6
NDC1;FYTTD1;NXF1;DDX19B;SRSF1;SMG7;EIF4E;SRSF10;SRSF9
PTGFR;CUL3;HNRNPU;BMI1;ERBB4;E2F1;HAS2;PTK2B;S1PR3;SCN5A;IL6R;MCTS1;LYN;SRPK2;TFAP2B;
PPP2CA;SFRP1;BMPR2;BTG1;SERTAD2;APBB2;AGTR2;INHBA;OSTN;SLIT2
TMOD3;CUL3;TMOD2;CDC42BPA;MYH10;LMOD3;MYPN
CXADR;GRID1;XIAP;EIF4EBP2;BTBD9;CRK;LRP8
BHLHB9;ROCK1;SIX1;CTNNB1;HOOK3;SORL1
EPS8;EFEMP1;GAB1;PTK2B;PTPN11;EREG
SRPK2;HRK;TFAP2B;TNFAIP8;BTG1;ANO6;GRAMD4;FOXO1;ING4;GNA13;SFRP1;BCLAF1;APH1B;APC;C1
AMER1;CUL3;TNFAIP3;XIAP;PTK2B;PRICKLE1;SENP2;GTPBP4;SMAD7
DCUN1D5;DCUN1D1;BMI1;SMAD7
CNOT6;AGO1;SNIP1;PUM2
RAP2C;GRM5;RAP2B;LRP8
RAP2A;SIPA1L1;CAPRIN1;HECW2;IL1RAPL1
SCOC;SESN3;CALCOCO2;RAB3GAP2;PAFAH1B2
CARNS1;HAS2;RIMKLA;NAT8L;GLS
CDH6;CADM3;CDH2;CTNNB1;SMAD7
BHLHB9;CAPRIN1;LRP8
PRDM8;EIF2B2;MYRF
HNRNPU;HNRNPC;DCP2
ADCY9;ADRA2B;ADRA2A
HS3ST3B1;GPC1;SULF2

CTNNB1;PHOX2B;LRP6
NCKAP1;RHOBTB3;PARVA
USP9X;SEMA3A;SLIT2
ADCY9;ADRA2B;ADRA2A
EPS8;GNA13;ROCK1;RHOBTB3;RND3;ADRA2A;PHACTR4
TGFBR3;SFRP1;SCN5A;RUNX3;CDC73;PAX2;EREG
BTG1;HPGD;APC;ANGEL2;PDCD4;INHBA;RUNX3
CDH6;GJC1;CADM3;OCLN;CXADR;CDH2;CLDN1
KANK2;PTGFR;BTG1;ATP8A2;BMPR2;CUL3;NPR3;XIAP;CDC73;ING4;ERBB4;HAS2;PTK2B;S1PR3;RELT;IL6
SLC24A2;TFAP2B;TMEM203;STIM2;VAPB;ATP2B4;ATP2B2;ATP2B1
AMER1;SFRP1;PSMD11;ZNRF3;APC;CSNK1A1;CUL3;PRICKLE1;TMEM64;DKK3;RBX1;LRP6
NAPA;NAPB;RAB3C;RAB2B;SEPT10;RABGAP1L;SEPT11;SEPT12;TRAPPC8;RAB11A;FBLN5;AMOT;SMAD7
RNF126;SNX1;SORT1;VPS13C;RHOBTB3;VTI1A;VPS54;HOOK3;SYT7;SNX6
SEPT10;SEPT11;SEPT12;APC;ARL3;MYH10
CDH6;GJC1;CDH2;CAPZA1;CTNNB1;SMAD7
BMPR2;LDLRAD4;INHBA;GDF6;SMAD7;BMPR1A
CDH6;NCKAP1;TENM3;CDH2;CAPZB;C21ORF59
CTNNB1;PROX1;PHOX2B;LRP6
TNKS2;TNKS;NEK7;MAPK1
HPSE2;GPC1;GPC5;CTBS
GTF2H1;RNF111;GTF2H5;RBX1
LYN;USP47;UBA6;XIAP;GTF2H1;SMC1A;BACH1;ZBTB4;GNL1;FOXO1;APC;RBBP5;PITHD1;RFWD3;RPA4;C
ESR1;FGFR1
BMPR2;HIPK1
EOMES;BMPR2
ABCA1;SMAD7
NDC1;TMEM33
LYN;RC3H1
EDEM1;SORL1
DNMT3A;PCGF3
CRP;VAPA
KANK2;ZNF366
ST8SIA3;ST6GALNAC3
LDLRAD4;SMAD7
GLUD2;GLS
CTNNB1;LRP6
FZD3;CTNNB1
TRA2B;CELF3
MTMR3;MTMR9
TFAP2B;TBX5
PROX1;LMOD3
BCLAF1;CTNNB1
TGFBR3;PROX1;SMAD7
ABCA1;ATP8A2;ATP11A
GTF2H1;GTF2H5;RBX1
TGFBR3;ARID5B;PROX1
EOMES;CDH2;PTPN11;AGTR2;PROX1;SMARCA1;GRIN2B;PAX2;PAFAH1B2;PHF8
NCKAP1;PDE1B;PLAG1;ZBTB16;PRUNE2;NSG1;GDF6;HK2;ING4;PPP2CA;BCL2L13;BCLAF1;ELMO1;PDCD
KANK2;RBL1;ID2;E2F1;CDC73
PCMT1;NSD1;CARM1;SNRPD3;CALM1
TNFAIP3;CTNNB1;SENP2;GTPBP4;SMAD7
CREBBP;OCLN;SF3B3;APC;ST13;CAPZA1;CNKSR3;PTK2B;TEAD1;ADD2
ING4;PPP2CA;SFRP1;BMPR2;BTG1;SERTAD2;APBB2;AGTR2;INHBA;SLIT2

SHC4;LYN;SHC2;BMPR2;EFS;ERBB4;PALM2;CNPY1;PTK2B;FRK
PRICKLE1;PAX2;LRP6;PHACTR4
EPS8;ROCK1;RHOBTB3;PTK2B;RND3;CRK;ARHGEF5
EPS8;ACAP2;AGAP2;SPRY3;SPRY1;RAF1;AGFG2;APPL1
RAP2A;ITCH;MECOM;SEMA3A;DUSP19;PTK2B;CTGF;HIPK2
CADM3;TENM3;CXADR;IL1RAPL1;SELE
DCUN1D5;PABPN1;TNKS;XRCC5;ERBB4;NEK7;DCUN1D1;MAPK1;BMI1;SORL1;FGFR1
RAP2C;RAP2A;CALM1
ZDHHC18;ZDHHC20;ZDHHC21
ZDHHC18;ZDHHC20;ZDHHC21
TRA2B;ZNF236;PAX2
GTF2H1;GTF2H5;RBX1
KANK2;RBL1;CDC73
ZBTB16;PTK2B;CDC73
KANK2;SLIT2;KANK4
GTF2H1;GTF2H5;RBX1
HSPH1;HSPA4L;TM7SF3
TRA2B;CELF3;DYRK1A
LYN;ANO6;SLIT2
MTMR9;AGTR2;CALM1
ABCA1;ATP8A2;ATP11A
TGFBR3;PROX1;SMAD7;BMPR1A
SEMA3A;PLXNA2;L1CAM;PLXNA3
PTPRM;SLIT2;AGTR2;SYNJ2BP
BHLHB9;MYOD1;PROX1;LMOD3
PPP2CA;NDNF;AGTR2;PPP1R9A;SELE;NUDT4
TFAP2B;MARCKS;RAP1A;KCNC2;SYTL4;MYH10;SYT7;ADRA2A;STXBP5L
USP37;CDC34;RANBP3;CUL3;RPA4;INHBA;EIF4E;PHF8
ITCH;C1QBP
PDCD4;DUSP19
SDR16C5;DYRK1A
RIMS3;SYT7
CREBBP;RUNX1
MEF2A;TRAK2
ROCK1;SORL1
CDC34;UBE2K
TFAP2B;GPCPD1
TENM3;LRP6
SIX1;PAX2
KCNC2;FOXO1
ATP2B4;FOXO1
GABRA1;GABRA3
ITPK1;ADK
CALCOCO2;TBC1D25
CTNNB1;LRP6
GFPT1;PGM3
PDCD4;ANO6
PTGFR;SFRP1
PHACTR4;LRP6
SMURF2;C1QBP
EFEMP1;LRP6
ZBTB16;PAX2
HNRNPK;HIF1A

ROCK1;SIX1
SFRP1;ZNRF3
DOCK5;BMPR2;SMURF2;SEMA3A;GAB1;ACTN4;SOD2;HIF1A;RAB11A;ADRA2A;PTP4A1;MYO1C;APC;C1Q
MAP2K1;SFRP1;RBL1;TIPARP;SFMBT2;ID2;ZNF148;TARDBP;HIF1A;ESR1
IGBP1;USP47;TFAP2B;KIAA0141;XIAP;RAF1;GRAMD4;PAX2
NOS1AP;ZDHHC21;CALM1
SEMA3A;PLXNA2;PLXNA3
GNA13;ABCA1;PTGFR;ADCY9;ADRA2B;ADRA2A;TSHR
TNKS;XRCC5;NEK7;MAPK1
EPS8;DPYSL3;RND3;ADD2
PDCD4;TNFAIP3;NDNF;ANO6
CDH6;CADM3;TENM3;CDH2;PTPRM;PCDHA7
LYN;EFEMP1;ERBB4;DYRK1A;PTK2B;FRK;FGFR1
MEF2A;KCNIP2;SCN5A;SLC25A12;CALM1;KCNH1;SLC25A24
NAPA;NAPB;SNAP23;AGAP2;RHOBTB3;LRP8;CYTH3;SNX1;VTI1A;VPS54;KIF1B;SNX8;AGFG2;APPL1;SNX
HRK;TNFAIP8;ANO6;FOXO1;ING4;GNA13;SFRP1;BCLAF1;APH1B;APC;C1QBP;JMY;E2F1;CTNNB1;SLIT2;S
CDH6;CADM3;CXADR;TENM3;CDH2;IL1RAPL1;PTPRM;CLDN18;CLDN1;SELE;PCDHA7
TNFAIP3;SENP2;GTPBP4;HIPK2;SMAD7
BMPR2;S1PR1;PTK2B;ANO6;BMPR1A
CREBBP;SFRP1;TUBD1;BTRC
ANO6;SCN5A;SCN3B;SCN1A
ELK4;HDAC5;SIN3A;TBL1XR1
RAP2A;SIPA1L1;CDH2;HECW2
TGFBR3;S1PR1;PROX1;SMAD7
MEF2A;CDH2;MYOD1;CTNNB1
GPM6A;RALA;CAPZB;DPYSL3
ROCK1;RHOBTB3;MAPK1;RND3;CRK;SORBS3;MYPN;ARHGEF5
IGF2BP1;EREG
GFPT1;PGM3
LYN;EFNA3
SIX1;PAX2
RORB;MAPRE2
TGFBR3;SFRP1
SART3;SRSF10
ROCK1;S1PR3
LYN;SLIT2
SDR16C5;SET
PDCD4;PRKG1
CXADR;MYPN
NAA30;METAP2
SERINC3;PSPH
SP1;FGFR1
TBX5;SOX6
SOSTDC1;PRICKLE1
HEYL;SFRP1
EDEM1;UBE2J1
PROX1;LMOD3
TMEM33;BFAR
GFPT1;PGM3
PROX1;PHOX2B
SYT5;SNX1;SORT1;SNAP23;HOOK3;SYTL4;SNX8;AP2M1;SYT7;SNX6
LYN;RBL1;ERBB4;VPS13C;PIM3;SLIT2;RAF1;ESR1;ADRA2A;FGFR1
SLC24A2;LETM1;TMEM203;STIM2;VAPB;ATP2B4;ATP2B2;ATP2B1

KANK2;MECOM;CTNNB1;TM7SF3;SORL1;PAX2;CTGF
PLXNA2;SLIT2;L1CAM;PLXNA3
CBX5;E2F1;EPC1;BMI1
EPS8;DPYSL3;RND3;ADD2
SMURF2;C1QBP;INHBA;PUM2
ARHGEF10;EIF2B2;MYRF;GPC1
KANK2;SLIT2;KANK4
XRCC5;HNRNPU;TERF2IP
KANK2;RBL1;CDC73
ADRA2A;SNX6;EREG
LYN;EFNA3;GRIN2B
PURA;CDC34;RPA4
EDEM3;MAN1A2;EDEM1
HS3ST3B1;BMPR2;B3GAT2
ADCY9;PDE1B;PDE3B
TENM3;EFEMP1;PAX2
SFRP1;CAPZB;DAG1;VASH2;TNFAIP3;RC3H1;CTNNB1
ING4;RPS6KA6;CNOT6;CARM1;E2F1;CDK1;HIPK2
RNF126;SNX1;RHOBTB3;VTI1A;VPS54;SNX8;SNX6
BMPR2;ZBTB16;ANO6;IL6R;BMPR1A
LYN;APC;SPRY3;SPRY1;SORL1;LRP6
BMPR2;SP1;VASH2;PROX1;HIF1A;FGFR1
SORT1;KIAA0141;INHBA;HIPK1;IL6R;APPL1
CXADR;TMEM65;ATP2B4;ATP2B2;ATP2B1;CALM1
KLF10;VKORC1;DLX6;ZBTB16;TRPS1;SHOX2;NPR3;PAPSS2;COL19A1;FGFR1;SULF2
TFAP2B;SFRP1;MARCKS;RAP1A;KCNC2;TM7SF3;SYT7;ADRA2A
HDAC5;CREBBP;TBL1XR1;ATXN7;SIN3A;NSD1;CARM1;RUVBL1
RBM3;GRM5;ROCK1;EIF4E
SCN5A;SCN3B;SCN1A;TPCN1
RPA4;E2F1;CLSPN;RAD9A
SRPK2;TFAP2B;CTNNB1;GRIN2B
NDC1;TSFM;EIF2B2;NSUN3;FAM129A;LTN1;GIGYF2;PUM2;RBM3;CTIF;GRM5;AGO1;CAPRIN1;IGF2BP1;EI
REEP1;ZDHHC18;MYO1C;ZDHHC20;RAB3GAP2;ZDHHC21;RAB11A
PDP2;PDHB
SORT1;SYTL4
SERINC3;PSPH
BMPR2;DAND5
MAP2K1;ESR1
GLUD2;GLS
GATA6;PROX1
NXF1;PABPN1
DFFA;FZD3
USP13;EDEM1
NUDT5;NUDT4
FZD5;SMAD7
BMPR2;PROX1
SFRP1;TUBD1
KCNIP2;CALM1
VAPA;KPNA1
CRP;ABCA1
ABHD2;GPCPD1
SLC17A6;SYT7
HS3ST3B1;CHST7;GPC1;B3GAT2;OMD;HAS2;GPC5;CHST3

IGBP1;USP47;TFAP2B;XIAP;RAF1;PAX2
MAP2K1;TNKS;DYRK1A;CDK1;MAPK1;HIPK2
SYT5;RIMS3;SYTL4;ADRA2A;SYT7
CHST7;B3GAT2;CHST3
GPM6A;RALA;DPYSL3
TGFBR3;ABCA2;GRIN2B
NCKAP1;CAPZB;SLIT2
MAPK1;CALM1;UBE2K
JPH3;CALM1;JPH1
LYN;KANK2;OTUD4;TNFAIP3;APCDD1;EREG;DKK3;SULF2;SFRP1;ERBB4;GPC1;SPRY3;SOSTDC1;SPRY1
SF3B3;ROCK1;AGAP2;AZIN1
ERBB4;CDC37;GAB1;EREG
CBX5;E2F1;EPC1;BMI1
MAP3K2;MAP2K1;BTG1;RBL1;ANGEL2;USP2;NEK7;CDK1;HNRNPU;PIM3;EIF4E;CKS1B
BMPR2;PTPRM;PTK2B;PROX1;SLIT2;SYNJ2BP
ABCA2;SFRP1;TIPARP;MAPK1;CTNNB1;ESR1
EPS8;NCKAP1;GPM6A;RALA;APC;DPYSL3
MEF2A;TGFBR3;KAT2B;CXADR;BMPR2;ERBB4;SHOX2;GATA6;PTPN11;TBX5;DAND5
NAPA;NAPB;VAPA;SNAP23;VASH2
KANK2;APC;DYRK1A;SLIT2;KANK4
CREBBP;XIAP;DTX1;SYNJ2BP;SENP2;ESR1;RUNX1;PPP2CA;RIC8B;FRRS1L;ERBB4;TMEM108;SIK2;RAF
ZDHHC18;MYO1C;ZDHHC20;CDC37;VPS13C;VTI1A;ZDHHC21;SORL1;TRAK2;OTC
SLC24A2;TMEM203;STIM2;VAPB;ATP2B4;SLC39A13;ATP2B2;ATP2B1
KANK2;MAP3K2;USP47;CREBBP;DFFA;MAP2K1;ACTN4;INHBA;GDF6;TM7SF3;IKZF3;PAX2;MECOM;NOS1
BMPR2;SP1;PTK2B;PROX1;HIF1A;FGFR1
SEPT10;SEPT11;SEPT12;APC;ARL3;MYH10
TGFBR3;EIF2B2;CREB1;SORT1
SFRP1;GATA6;CTNNB1
RRM1;ADCY9;RRM2B
NFATC3;HIF1A;TEAD1
TGFBR3;ABHD2;SLIT2
KAT2B;CREBBP;RUVBL1
FNTB;CALM1;METAP2
MAP2K1;MAPK1
LYN;PRKG1
PDP2;PDHB
ID2;PROX1
NAPA;NAPB
ADK;PRTFDC1
MME;DYRK1A
NOS1AP;SCN5A
VTI1A;VPS54
ALG6;ALG14
GNL1;IL18BP
PTPN11;IL6R
CDC37;PIAS1
BMPR2;AGTR2
ADK;PFAS
CHST7;PGM3
BTG1;MYOD1
MYOD1;LMOD3
IGBP1;SMG7
TNKS2;TNKS;XRCC5;NEK7;MAPK1

TFAP2B;PRDM16;SENP2;GDF6;FOXO1
NSD1;CNKSR3;ATP2B4;TERF2IP;CREBL2;RAF1;SMAD7
ING4;KAT2B;CREBBP;MYOD1;RUVBL1;EPC1
JPH3;KCNIP2;PTK2B;SHISA9;CALM1;JPH1
LYN;SPRY3;SPRY1;TRIB2;SORL1;ADRA2A;FGFR1
DOCK5;C1QBP;ACTN4;CRK
PURA;GTF2H1;GTF2H5;RBX1
LYN;MAP2K1;GPR37;DUSP19;INHBA;GDF6;ADRA2B;ADRA2A;SPRED1;ERBB4;DAG1;MAPK1;TRIB2;FGFR
SPRY3;SPRY1;SLIT2
DOCK5;C1QBP;CRK
VPS13C;VTI1A;SORL1
CXADR;MYPN;LMOD3
HSPH1;HSPA4L;TM7SF3
PDCD4;TNFAIP3;PRKG1
IGBP1;USP47;TFAP2B;XIAP;RAF1;PAX2
GJC1;ROCK1;PABPN1;TPM3;KCNIP2;TMOD3;TMOD2;CALM1;SORBS3;LMOD3
TNFAIP8;SET;BHLHB9;GATA6;AGAP2;XIAP;SIX1;NDNF;FOXO1;MTDH;ERBB4;PTK2B;PIM3;IGBP1;TFAP2B;
LYN;RXRA;PTK2B;RORB;PAX2
GABRA1;GABRB1;CLCN5;GABRA3;ANO6
DFFA;KPNA1
NDC1;TMEM33
HEYL;BMPR1A
PFKFB3;HIF1A
SIX1;PAX2
PFKFB3;HIF1A
CUL3;HECW2
NOS1AP;ANO6
ROCK1;PTK2B
MEF2A;TRAK2
HEYL;BMPR1A
PDE3B;ADRA2A
AGTR2;CALM1
TGFBR3;CRK
ABCA2;LRP6
ST8SIA3;ST6GALNAC3
NOS1AP;KCNRG
SOSTDC1;EREG
CA12;TFAP2B
LYN;MAPK1
NOS1AP;ADRA2A
ENTPD1;NUDT5
SIX1;PAX2
CALCOCO2;WDFY3
TNKS;XRCC5;NEK7;MAPK1
LYN;PTK2B;RORB;PAX2
MEF2A;SCN5A;SLC25A24;KCNH1
PPP2CA;LYN;SPRED1;GRM5
IGBP1;MME;CALCOCO2;DPYSL3;IL1RAPL1;PTPN11;PTPN4;IL6R;SELE;IL18BP
GABRA1;GABRB1;CLCN5;GABRA3;SLC4A10;ANO6
TFAP2B;MARCKS;RAP1A;KCNC2;ADRA2A;SYT7
GALNT7;MAP2K1;TNKS;DYRK1A;CDK1;MAPK1;HIPK2
CREBBP;MAML1;TUBD1;HIF1A;CDC73;SULF2;KAT2B;SFRP1;MYO1C;RSPO2;TMEM108;PTK2B;TRIM44;WI
SFRP1;CTNNB1;LRP6

CRP;ANO6;IL6R
RIMKLA;NAT8L;GLS
SFRP1;INHBA;AGTR2
ABCA1;TFEB;HOOK3
AGO1;SNIP1;PUM2
PRKAB2;CREB1;CREBL2
ADCY9;PDE1B;PDE5A
E2F1;BMI1;EREG
SFRP1;VASH2;CTNNB1
MCTS1;SRPK2;SART3;YTHDC1;CELF1;SRSF1;RC3H1;SNRPD3;SRSF10;CDC73;SRSF9
CDC37;TNFAIP3;PTPN11;HIPK1;TRIM44;RUNX1;PIAS1
DOCK5;BMPR2;PTK2B;PROX1;HIF1A;RAB11A
CPSF7;PABPN1;PAPOLG;SRSF1;CDC73;SRSF9
HRK;TNFAIP8;GATA6;KIAA0141;XIAP;IKZF3;FOXO1;MTDH;ING4;GNA13;BCLAF1;APH1B;ERBB4;C1QBP;E2
SCN5A;SCN3B;SCN1A;TPCN1
EFEMP1;HIPK1;PAX2;HIPK2
ZMAT3;JMY;CDIP1;HIPK2
DFFA;BMPR2;TERF2IP;GTPBP4
DAG1;CDK1;TRIP12;SMAD7
SF3B3;ROCK1;AGAP2;AZIN1
SLC24A2;LETM1;TMEM203;STIM2;MB;VAPB;ATP2B4;ATP2B2;ATP2B1
SPRED1;CHAD;DUSP19;HNRNPU;TRIB2;PPM1E;LRP6
TGFBR3;IGSF3;SIX1;ARID5B;PROX1;DKK3
DCTN2;HOOK3
PPP2CA;PPM1E
C1QBP;PUM2
HDAC5;SIN3A
HDAC5;SOX6
SCN5A;SCN3B
HAS2;FBLN5
SGIP1;FOXO1
GLUD2;GLS
SFRP1;NPR3
SRD5A1;PTPN11
SFRP1;ADRA2A
SCN5A;LRP6
BMPR2;SMAD7
CRP;ABCA1
PDCD4;ANO6
LYN;MTDH
RUVBL1;EPC1
MEF2A;CREBZF;TSFM;ALAS2;SET;COA1;MRPL27;DTX1;INHBA;SMARCA1;ESR1;PAX2;ING4;MRPL42;EEF
GTF2H1;GTF2H5;RBX1
CYB5R4;SH3PXD2A;SOD2
VPS13C;VTI1A;VPS54
NCKAP1;ROCK1;RHOBTB3
PCMT1;SNRPD3;CALM1
MOCS3;MAT2A;MAT2B
SEMA3A;PLXNA2;PLXNA3
PRKAB2;NOS1AP;IGF2BP1
RRM1;ALG6;RRM2B
BTG1;MYOD1;SOSTDC1
ST6GAL2;OSTC;ST6GALNAC3;UBE2J1

LOXL4;PTK2B;PARVA;SLIT2
SRPK2;STAU1;VAPB;FKBP6
PPP2CA;RPS6KA6;SFRP1;ZNF706;SOSTDC1;LDLRAD4;TBX5;RAB11FIP5;SMAD7
NXF1;RANBP3;XPO4;SRSF1;PHAX;EIF4E;SRSF9
KLF10;TGFBR3;USP15;HPGD;USP9X;BMPR1A
IGBP1;SFRP1;RC3H1;HAS2;HIF1A;SELE
SEMA3A;DUSP19;PTK2B;ARHGEF5;CTGF;HIPK2
MEF2A;KAT2B;CXADR;ERBB4;SHOX2;GATA6;PTPN11;TBX5
ST6GAL2;OSTC;ST6GALNAC3;UBE2J1
GRM5;TSHZ3;CDH2;PTK2B
LYN;DYRK1A;PTK2B;FRK
SFRP1;PTK2B;RORB;TMEM64
SIN3A;TNFAIP3;TRIM44
LYN;PDE3B;PTPN11
KCNIP2;ATP2B4;SCN5A
SELE;ESR1;FGFR1
GPC1;B3GAT2;GPC5
HNRNPC;SMARCA1;GATAD2B
ATP2B4;SCN5A;SCN3B
KAT2B;HDAC5;APC;PDE3B;IRS4;GAB1;FOXO1;APPL1
LYN;SFRP1;PABPN1;MYOD1;NRIP1;PDCD4;TNFAIP3;PTK2B;RORB;ESR1;CDC73;PAX2
HS3ST3B1;HPSE2;GPC1;B3GAT2;GPC5
ZDHHC18;MYO1C;ZDHHC20;ZDHHC21;SEC62
PPP2CA;USP37;ESCO1;GTPBP4;EREG
CTNNB1;PIAS1
MEF2A;PROX1
SUB1;ESR1
LYN;TNFAIP3
SIX1;SLIT2
E2F1;HNRNPU
MAML1;PROX1
EOMES;LRP6
PCGF3;BMI1
HOOK3;SORL1
BMPR2;PROX1
ROCK1;PPP1R1B
CLSPN;RAD9A
BMPR2;BMPR1A
UTP15;PHF8
RAP1A;SORT1
ROCK1;PTK2B
KIF5C;SYT7
SIX1;SLIT2
MAP2K1;MAPK1
KCNC2;FOXO1
SCN5A;SCN3B
SFRP1;ROCK1
SEMA3A;SLIT2
TNKS2;TNKS
ZBTB16;RUNX3;RUNX1;SULF2
RALA;CAPZA1;PDCD4;SOD2
RALA;CAPZA1;PDCD4;SOD2
ROCK1;RHOBTB3;RND3;CRK;ARHGEF5

TMEM65;KCNIP2;ATP2B4;ATP2B2;ATP2B1;CALM1;SMAD7
DLX1;PTGFR;MYRF;CUL3;CTGF;LRP6;BCLAF1;ERBB4;HAS2;PTK2B;S1PR3;IL6R;MCTS1;LYN;SRPK2;TFAI
LYN;CCND3;RAP2A;ERBB4;NOS1AP;DYRK1A;FAM129A;TERF2IP;RAB3GAP2;WDFY2;CALM1
DCUN1D5;DCUN1D1;BMI1
TFAP2B;ADCY9;RAB11A
AP3M2;KIF1B;TRAK2
RAP2C;RAP2A;CALM1
ABCA1;TFEB;HOOK3
RRM1;RRM2B;CMPK1
TGFBR3;ARID5B;PROX1
BMPR2;ANO6;BMPR1A
OTUD4;LYN;TNFAIP3
KANK2;USP47;RBL1;PSMD11;CDC73;RBX1
BMPR2;VASH2;PTPRM;PROX1;SYNJ2BP;HIF1A
CHRM3;GRM5;GPR37;ADCY9;ADRA2A
SRPK2;STAU1;VAPB;PROX1;FKBP6
CNOT6;RC3H1;LSM5;EIF4E;DCP2
ITPK1;IP6K1;CALM1;NUDT4
ITPK1;IP6K1;CALM1;NUDT4
BMPR2;INHBA;GDF6;BMPR1A
EIF2B2;TRA2B;ZNF236;PAX2
TMOD3;TMOD2;MYPN;LMOD3
USP13;ZNF275;USP37;BTG1;SIX1;RORB;BACH1;HIF1A;ELAVL2;PPP2CA;RBM3;GRM5;EFEMP1;ZNF629;SA
RAP1A;XRCC5;ERBB4;DUSP19;PTK2B;MAPRE3;EREG;FGFR1
CPSF7;SEPT11;ATL3;KCNC2;ATL2;TNFAIP3;CLDN1;LETM1;ALAD;MAT2A;APC;TRPS1;TRA2B;DPYSL3
MGAM;CALM1
RUVBL1;DCP2
ROCK1;SORL1
DCTN2;HOOK3
PDE3B;ADRA2A
FZD5;WLS
CALM1;TSHR
SFRP1;CDC73
HIF1A;IL6R
NOS1AP;SCN5A
CNKSR3;SMAD7
ALG6;ALG14
CRP;IL6R
CDC34;PDE3B
SOD2;HIF1A
PFKFB3;HIF1A
TDRKH;FKBP6
SIX1;PAX2
ATP2B4;CALM1
TSHZ3;PTK2B
ST8SIA3;ST6GALNAC3
PTGFR;GRM5
HNRNPU;HK2
SEMA3A;DUSP19;PTK2B;GDF6;CTGF;HIPK2
NCOA2;NPFFR1;GNRHR;SIK2;SLIT2;ADRA2A
NXF1;RANBP3;KIF5C;SRSF1;EIF4E;SRSF9
ARHGEF10;TNKS;NEK7;HAUS3;XIAP;RAB11A
FRRS1L;PTK2B;SHISA9

HEYL;CALM1;SNX6
ADCY9;RAF1;TSHR
MMP2;INHBA;CDC73
CD47;GTPBP4;SMAD7
E2F1;TRIB2;FOXO1
LYN;INHBA;HIF1A
NOS1AP;ATP2B4;AGTR2
TGFBR3;SPRED1;HAS2
HDAC5;SIN3A;TBL1XR1
CAPRIN1;IL1RAPL1;LRP8
NAPA;DCTN2;CUL3;ARL3;SNAP23;TRAPPC8;DYNLL2;SORL1;CYTH3;SNX1;CAPZB;VAPA;VAPB;CAPZA1;V
SFRP1;GABRB1;RAP1A;SESN3;CTNNB1;SLIT2;TSHR
GABRA1;GABRB1;CLCN5;GABRA3;ANO6
SFRP1;GATA6;NDNF;HIF1A;HIPK2
C1QBP;NDNF;CD47;TGM2;SMAD7
FZD3;BTG1;ANGEL2;CTNNB1
DOCK5;BMPR2;HIF1A;RAB11A
CBX5;E2F1;EPC1;BMI1;SMC1A;PHOX2B
SFRP1;CREBBP;PSMD11;GATA6;NDNF;BACH1;HIF1A;RBX1;HIPK2
KCNC2;TNFAIP3;MAPK1;PAX2;FOXO1
LYN;TNFAIP3;RORB;ESR1;PAX2;CDC73;TSHR;SFRP1;RAP1A;SESN3;PABPN1;MYOD1;NRIP1;PDCD4;PTK
DENND6A;CD47;SMAD7
GABRA1;GABRA3;GRIN2B
SFRP1;ROCK1;PTK2B
EFEMP1;ID2;SIX1
GABRA1;GABRA3;GRIN2B
CNOT6;LSM5;DCP2
BMPR2;ANO6;BMPR1A
KAT2B;CREBBP;MAML1
ADCY9;RAF1;TSHR
RAP1A;RAF1;CRK
HIF1A;UBE2K;TRIM44
HNRNPU;SMC1A
RNF111;HIPK2
TNKS2;TNKS
ALAD;HIPK2
TGFBR3;PROX1
PDE3B;ADRA2A
ESR1;PPARGC1B
SORT1;PROX1
USP15;ATXN7
CYB5R4;RAB11FIP5
SEMA3A;SRGAP2
GFPT1;PGM3
HIF1A;TEAD1
SFRP1;ROCK1
CPSF7;GRIN2B
LETM1;TGM2
CALM1;TSHR
RNF111;HIPK2
CDC34;UBE2K
INHBA;GDF6
NSD1;CARM1

HDAC5;MAML1
ROCK1;ID2;E2F1;ID4;RSF1;SORL1
CNOT6;ID2;CARM1;GATA6;E2F1;CDK1
JPH3;PTK2B;CALM1;JPH1
SHC2;ARHGAP31;ARL3;G3BP1;CNKSR3;RAPGEF6;ADRA2A
APH1B;DDI2;KLK13;ASPRV1;SORL1;METAP2
SP1;AGTR2;HIF1A;FGFR1
BCL11B;SALL4;SIX1;TRAK2
RBM3;GRM5;INHBA;EIF4E
MAP3K2;DUSP19;MAP3K9;ARHGEF5
GALNT7;CSNK1G3;TNKS;CSNK1A1;CAMK4;DYRK1A;CDK1;MAPK1;NDNF;CLSPN;HIPK2
ERBB4;SOD2;HK2
TNFAIP3;CRK;ADRA2A
DUSP19;PDCD4;ARHGEF5
BMPR2;LRP6;WLS
TERF2IP;TRIP12;DEK
SORL1
ABCA1
PCMT1
TFAP2B
PROX1
HIF1A
LRP6
HEYL
OTC
TERF2IP
PDE1B
GATA6
LYN
VKORC1
HPGD
GATA6
MYRF
RCSD1
ANO6
RAB11A
CHRM3
RAP2C
TBX5
SMC1A
TGM2
TNFAIP3
LRP6
ING4
SRD5A1
ID2
SOX6
PAPSS2
GTF2H1
SFRP1
ABCA1
RUNX1
GJC1

LTN1
LYN
OTC
FBLN5
HSPH1
CTNNB1
PDCD4
PROX1
PAX2
ID2
CDK1
MME
PDHB
LRP6
SCN5A
PPP2CA
GRM5
DYNLL2
CDK1
SRSF1
LHX6
NUDT4
SLC17A6
SORL1
VTI1A
ADRA2A
DKK3
HS3ST3B1
RFWD3
DKK3
INSIG2
WDFY3
VASH2
PRUNE2
GATA6
PAX2
TGFBR3
GRM5
C1QBP
KCNRG
CALCOCO2
TRIP12
PAX2
PAX2
CTNNB1
RANBP3
DAND5
PROX1
FOXO1
RAB11FIP5
SLIT2
PDE1B
DYRK1A

PROX1
GLUD2
XRCC5
ATL2
CHRM3
APC
GFPT1
S1PR1
CD244
E2F1
LRP6
DLST
ROCK1
PAX2
HIF1A
RBX1
FBLN5
NAA30
SORL1
ROCK1
TGFBR3
METTL16
TNFAIP3
RAB11FIP5
SFRP1
HSPH1
SYT7
TERF2IP
SLC24A2;RIMS3;TSHZ3;PTK2B;GRIN2B
APC;IRS4;GAB1;FOXO1;APPL1
MEF2A;MAP2K1;SHC2;PSMD11;CUL3;IL17RD;GRIN2B;EREG;RBX1;SPRED1;PABPN1;ERBB4;MAPK1;BTR
SORT1;VPS13C
ROCK1;RUNX1
PTK2B;ADRA2A
MGAM;CALM1
CLDN18;CLDN1
ITCH;C1QBP
MAP2K1;PUM2
SIX1;PAX2
LYN;MAPK1
PPP2CA;SPRED1
TERF2IP;SMG7
ACTN4;TRAK2
TENM3;EFEMP1
CAPZB;SLIT2
C1QBP;INHBA
CHST7;CHST3
ID2;USP2
CALM1;TSHR
EOMES;BMPR2
BMPR2;SULF2
SIX1;PAX2
NOS1AP;PTK2B

LYN;FAM129A;INHBA;GDF6;LRP6;PPP2CA;RAP2A;CCND3;SPRED1;GRM5;ERBB4;NSD1;TERF2IP;WDFY2;
CPSF7;PABPN1;SRSF1;SNRPD3;SRSF9
CREBBP;ITCH;XRCC5;TNFAIP3;CTNNB1;DTX4
RAP2C;SFRP1;RAP2B;PTK2B;IL6R;LRP8
UTP15;MAP2K1;DYRK1A
CDC37;GTF2H1;GTPBP4
CNOT6;LSM5;DCP2
LDLRAD4;RAF1;SORL1
PTPRM;SLIT2;SYNJ2BP
PPP1R1B;AGTR2;CALM1
SCN5A;CALM1;SMAD7
ERBB4;SEMA3A;PHACTR4
BMPR2;IL6R;BMPR1A
CREB1;FOXO1;PAX2
DHX40;RBM3;DDX19B;HNRNPK;PABPN1;PAPOLG;AGO1;RNASE6;HNRNPU;RBMS1;HNRNPR;HNRNPC
CSNK1G3;CSNK1A1;SORT1;RHOBTB3;LRP8;SORL1;SYT7;NEURL1B;SNX1;NECAP2;HSPH1;TMEM108;MA
NCKIPSD;RANBP3;TRPS1;DAG1;CTNNB1;PRICKLE1;KPNA1
NCKAP1;GPM6A;SNX1;SRGAP2
BHLHB9;MYRF;IL1RAPL1;PAX2
ID2;E2F1;ID4;RSF1
SLC24A2;LETM1;ATP2B4;ANO6;MICU3;GRIN2B
CDC37;TNFAIP3;XIAP;PTPN11;EREG
BMPR2;RORB;TMEM64;IL6R;BMPR1A
UTP15;PHF8
LYN;ACTN4
HIF1A;EREG
LYN;CAMK4
NCKAP1;ROCK1
CDIP1;HIPK2
SLC30A4;SLC39A13
INHBA;TRIB2
RAP2C;RAP1A
ALDH6A1;PPM1K
TNFAIP3;RUNX1
SIN3A;TRIM44
RNF126;CUL3
CREB1;CREBL2
LETM1;MICU3
SIX1;SULF2
ID2;USP2
MAP2K1;MAPK1
GLUD2;GLS
SMAD9;HIF1A
TERF2IP;SMG7
HAS2;CD47
HECW2;ADRA2A
KCNIP2;PTK2B;ADRA2A
FBXO40;MYOD1;GATA6
EOMES;MMP2;INHBA
BCLAF1;RFWD3;USP1
SERP1;ANO6;SYT7
GRM5;PTK2B;GRIN2B
MAML1;PROX1;BMPR1A

LYN;EEF2K;ERBB4;CAMK4;DYRK1A;PTK2B;PIM3;MAP3K9;SIK2;FRK;FGFR1
HS3ST3B1;CHST7;MAT2A;B3GAT2;OMD;MAT2B;PAPSS2;CHST3
KAT2B;CREBBP;MAML1;DTX1;SYNJ2BP
RPS6KA6;SFRP1;ZNF706;SOSTDC1;RC3H1
PTGFR;GRM5;PPP1R9A;SELE;NUDT4
CALM1
ABCA1
KPNA1
PARVA
IL18BP
ANO6
SCN5A
ROCK1
DPY19L2
PAPSS2
C1QBP
HNRNPU
SMC1A
SORL1
FBLN5
SRD5A1
SOX6
AGO1
DIO2
INSIG2
ALDH6A1
SLC1A4
MOCS3
SMAD7
CTBS
MCTS1
RUVBL1
SART3
PDE3B
EREG
SORD
SLC25A24
DPY19L2
ST6GALNAC3
HNRNPK
SCN5A
HSPH1
NOS1AP
CD244
ROCK1
BMPR1A
ATP2B4
STAU1
SOX6
ACTN4
SMAD7
PUM2
TERF2IP

LRP6
SLC25A24
SRD5A1
TNFAIP3
VAPB
GAPVD1
GABRB1
BMPR1A
HAS2
PAX2
ATP2B4
MOCS3
PHOX2B
HNRNPU
SMAD7
MAF
PPP2CA
ITM2C
NSD1
UBE2K
C1QBP
PAX2
APC
SOX6
GRIN2B
CTBS
STIM2
IL18BP
SLC24A2
PROX1
PAX2
AGTR2
LRP6
AGTR2
PTK2B
DIO2
GPCPD1
RCSD1
SIK2
MYRF
B3GAT2
ABCA1
CALM1
CXADR
DAND5
GPCPD1
ESR1
HIF1A
ERI1
SLIT2
CUL3
GPCPD1
ERI1

PRDM16
SORL1
GRIN2B
C1QBP
MB
BMPR1A
PAX2
FGFR1
USP13
ACTN4
HNRNPK
CMPK1
SEC62
TMEM108
SFRP1
HK2
CNOT6;CAPRIN1;IGF2BP1;EIF4EBP2;METTL16;GIGYF2
FZD3;PSMD11;SMURF2;FZD5;PRICKLE1;AP2M1
SART3;SNRPD3;SRSF10
USP1;TRIP12;DEK
CALM1;ADRA2A;EREG
RAP2A;HECW2;LRP8
CUL3;SPRY3;SPRY1
GTF2H1;GTF2H5;RBX1
PPWD1;FKBP6;PPIC
SYT5;SYTL4;SYT7
MAP3K2;MAP2K1;RAP1A;CDK1;DUSP19;MAPK1;RAF1;CRK;ADRA2A;ARHGEF5;FGFR1
KAT2B;HDAC5;SORT1;PDE3B;FOXO1
CPSF7;PABPN1;SRSF1;GTF2H1;SNRPD3;GTF2H5;SRSF9
VPS13C;VTI1A
MYO1C;MIEF1
BMPR2;TNKS
DFFA;KPNA1
JPH3;JPH1
INHBA;HIF1A
HDAC5;SYNJ2BP
CTNNB1;FBLN5
PLA2G12A;CDS2
ALDH6A1;PPM1K
ROCK1;ADRA2A
CREB1;CREBL2
ERBB4;EREG
NFIA;DEK
SIX1;PAX2
HOOK3;SORL1
S1PR1;TNFAIP3
PIM3;ADRA2A
CDC37;PIAS1
SFRP1;INHBA
KLF10;SFRP1;BMPR2;FOXO1
SCN5A;SCN3B;SCN1A;TPCN1
FZD3;PSMD11;SMURF2;FZD5;PRICKLE1;AP2M1
KLF10;SFRP1;MTMR3;BMPR2;SESN3;MAPK1;FOXO1

SFRP1;BMPR2;VASH2;SCN5A;PROX1;HIF1A;PAX2
PPP2CA;SLC30A4;SLC25A12;CALM1;HIF1A
PHLPP2;PPP2CA;MTMR3;PTPRM;PTPN11;BTRC;PTPN4;PPM1E
LYN;PHLPP2;SPRED1;CDC34;PDE3B;DAG1;ATP2B4;TNFAIP3;SLIT2;ESR1
UBE2K;PUM2;EREG
ALDH6A1;PPM1K
S1PR1;SRGAP2
SLC30A4;SLC39A13
SIX1;PAX2
PFKFB3;HIF1A
GABRB1;MAPK1
DFFA;KPNA1
CRP;CD244
PTPN11;IL6R
NSUN3;METTL16
RBBP5;TET3
SFRP1;ROCK1
ATP2B4;MAPK1
SCN5A;SCN1A
CDH6;CDH2
HNRNPU;CDC73
RC3H1;TNRC6B
ERI1;RNASE6;GTF2H1;GTF2H5;RBX1
LYN;PABPN1;PDCD4;TNFAIP3;CDC73;MTDH
ALAS2;PCYT1B;MOCS3;PPM1L;VAPA;VAPB;INHBA;RIMKLA;NAT8L;GLS;OTC
PURA;CDC34;RPA4;MCMBP
HPSE2;GPC1;OMD;GPC5
PHLPP2;ERBB4;C1QBP;DAG1;GAB1;PTPN11;ESR1;EREG;MTDH;FGFR1
MEF2A;NCKAP1;GPM6A;ROCK1;OMD;CTNNB1;SLIT2;SRGAP2;S100B;PAX2
SPRED1;CHAD;DUSP19;TRIB2;PPM1E;SORL1;LRP6
CSNK1G3;TNKS;CSNK1A1;CAMK4;DYRK1A;CDK1;MAPK1;CLSPN;HIPK2
ERBB4;C1QBP;GAB1;PTPN11;ESR1;EREG;MTDH;FGFR1
MYOD1;SIX1;LMOD3
RFWD3;CLSPN;RAD9A
ALDH6A1;CARNS1;OTC
CDC37;PTPN11;UBE2K
ARHGEF10;DCTN2;TNKS;XIAP;RAB11A
TFAP2B;CYB5R4;HK2;ADRA2A;FOXO1
EPS8;ARHGEF10;XIAP;SMC1A;RAB11A
TBX5
TGM2
IL18BP
TYRP1
CDC73
SORT1
RC3H1
CXADR
CARNS1
HNRNPU
DAND5
MME
CALM1
HIF1A

KCNRG
HDAC5
MYRF
UBE2J1
DCP2
TMEM33
PAX2
DNMT3A
HECW2
ATP2B4
KAT2B
CARNS1
NDNF
ACAP2
ZC3H14
DYRK1A
CDC73
IGSF3
RAP1A
ABCA1
NOS1AP
CARNS1
OTC
CUL3
ANO6
KCNC2
CNOT6
BMPR1A
IL1RAPL1
ARHGEF10
ALDH6A1
SLIT2
LRP6
ATP2B4
RNF180
USP13
CMPK1
MAP2K1
PTK2B
C21ORF59
SIN3A
ATP2B4
GPAM
PROX1
PHF8
TDG
ABCA1
SFRP1
SFRP1
GPCPD1
PDCD4
DAG1
SFRP1

ROCK1
CMPK1
ATP2B4
MYRF
TFAP2B
XRCC5
PRKG1
CARM1
SGIP1
ATP2B4
LETM1
TMEM33
ESR1
SENP2
ARHGEF10
KAT2B;HDAC5;ADCY9;PDE3B;GNRHR;FOXO1;TSHR
PPP2CA;USP47;SFRP1;ITCH;BTG1;BMPR2;NRG3;SERTAD2;APBB2;AGTR2;INHBA;SLIT2
SERP1;EDEM1;GFPT1;SEC62
EPS8;ABCA1;CUL3;RAF1
HEYL;SFRP1
SDR16C5;GPC1;E2F1;GPC5;LRP8
ROCK1;CAPRIN1;IGF2BP1;SORL1;GIGYF2
GNA13;ABCA1;PTGFR;ADCY9;TSHR
GTF2H1;GTF2H5;RBX1
GRM5;SLC1A4;DGCR2
RANBP3;HNRNPU;EREG
PPWD1;FKBP6;PPIC
LYN;MAPK1;PIAS1
PPP2CA;HDAC5;ROCK1;CRK;SORL1;ADD2;HIPK2
USP37;RANBP3;DCTN2;CUL3;HAUS3;INHBA;PHF8;EPS8;CDC34;RPA4;CDK1;BTRC;EIF4E
ABCA1;ATP8A2;ATP11A;PITPNC1
RNF126;SH3KBP1;SPRY1;SH3GL2
ANO6;SCN5A;SCN3B;SCN1A
KANK2;TFAP2B;USP47;TNFAIP8;GATA6;XIAP;BFAR;TM7SF3;PAX2;FOXO1;MTDH;SFRP1;ITCH;HNRNPK;M
TIAL1;SPRED1;SFRP1;MAPK1;PTPN11;FGFR1
MEF2A;CXADR;VPS13C;TFAM;MIEF1;SOD2;NFE2L1;HK2;TRAK2;OTC
SCN5A;INHBA;LRP6
CA12;CYB5R4;SLC4A10
HNRNPK;SGIP1;SELE
USP47;PSMD11;DCTN2;CDK1;HAUS3;DTL;PHOX2B;RAB11A;RBX1
CALM1;TSHR
ST6GAL2;ST6GALNAC3
ZNRF3;AP2M1
FCHSD2;KIF1B
MME;SCN1A
GPR37;SOD2
MAP2K1;ID2
TNFAIP3;NDNF
MTMR9;CALM1
HIF1A;SULF2
HSPH1;HSPA4L
JMY;LMOD3
CXADR;NOS1AP

ITCH;TNFAIP3
HNRNPU;SLIT2
SIX1;PAX2
SNRPD3;DCP2
SIN3A
ITPK1
ABCA1
SLC1A4
PDCD4
TERF2IP
CD244
NOS1AP
EREG
ERBB4
NFATC3
CUL3
KCNIP2
TBX5
UQCR10
SYT7
HOOK3
ARID5B
RHOBTB3
PPP2CA
RUVBL1
CARM1
LYN
MAPK1
UBE2J1
XRCC5
GPC1
SESN3
GPC1
CMPK1
UQCR10
GATA6
ABCA1
METAP2
RAB3GAP2
NSG1
KAT2B
SH3PXD2A
CMPK1
DAND5
CARM1
CLSPN
UTP15
CTNNB1
HEYL
PTK2B
PROX1
UTP15
SOD2

TMEM108
DENND6A
LYN
LYN
SORT1
TMEM86A
PHF8
SESN3
CRP
CALM1
LYN
GNL1
SLC1A4
MYOD1
HRK
RAB11FIP5
DYRK1A
LNPEP
SCN1A
ERBB4
MOCS3
DAG1
HNRNPU
CDK1
NECAB3
TET3
SOD2
AGO1
SLIT2
SLC25A12
PIAS1
GPR37
EFS
KCNIP2
HOOK3
CD244
BTG1
C1QBP
LYN
PTK2B
PTK2B
SORT1
ZC3H14
ABCA1
ANO6
SFRP1;ADRA2A;RAB11FIP5
MAF;ERBB4;CDK1;SIX1;PROX1;PAX2
SFRP1;RAP1A;SIPA1L1;PLXNA2;RAB3GAP2;SRGAP2;RAPGEF6;CRK;PRKG1;ARHGEF5;PLXNA3
TIAL1;SPRED1;MAPK1;PTPN11;FGFR1
CREBBP;PSMD11;BACH1;HIF1A;RBX1
FBXW4;PSMD11;ESCO1;CUL3;ZBTB16;DCUN1D1;HIF1A;DCAF7;RAB11A;FBXO30;SUMF1;RBX1;FBXO40;C
CXADR;PARVA
HNRNPU;SMC1A

XRCC5;TERF2IP
SNX1;S1PR1
PTK2B;HIPK2
SPRED1;DYRK1A
TMEM108;PTK2B
DIAPH2;USP9X
DAG1;SIX1
CTNNB1;RAB11FIP5
SLC1A4;SLC25A12
NOS1AP;AGTR2
NOS1AP;AGTR2
ITCH;TNFAIP3
ADCY9;NUDT4
RNF126;CUL3
LNPEP;TRHDE
USP13;CREB1;CDC37;USP2;MTMR9;CREBL2;GTPBP4;TRIM44;SMAD7
CAPZB;DCTN2;CAPZA1;DYNLL2;SH3GL2;AP2M1
CAPZB;DCTN2;CAPZA1;DYNLL2;SH3GL2;AP2M1
ABCA1;GNA13;PTGFR;ADCY9;GPR37;ADRA2B;ADRA2A;TSHR
LYN;RC3H1;IKZF3
GATA6;KLHL32;INHBA
HSPH1;HSPA4L;TM7SF3
ING4;KAT2B;MYOD1
ENTPD1;RNASE6;NUDT5
GATA6;KLHL32;INHBA
RNASE6;METTL16;DCP2
ALAD;LETM1;RXRA;ATL3;APC;KCNC2;DPYSL3;ATL2;SOD2;CLDN1;GLS
C1QBP;PTK2B;ANO6;IL6R
CPSF7;PABPN1;SRSF1;SRSF9
SCN5A;RCSD1;SCN3B;LMOD3
PPP2CA;LYN;ERBB4;IL6R
SCOC;PRKAB2;MTMR3;SESN3;TBC1D25;PAFAH1B2;SNX6
CAPZB;DCTN2;CAPZA1;DYNLL2;SH3GL2;AP2M1
SFRP1;GABRB1;RAP1A;TIPARP;MYOD1;NRIP1;CTNNB1;ESR1
HNRNPK;ERI1;HNRNPU;HNRNPR;HNRNPC
USP13;ZNF275;BTG1;SIX1;RORB;BACH1;HIF1A;ELAVL2;PPP2CA;EFEMP1;ZNF629;SALL4;E2F1;APBB2;HI
EFR3A;RAP2A;ROCK1;SCN3B;RAPGEF6;RAB11A;LRP6
MMP2;LOXL4;NDNF;COL19A1;FBLN5;ADAM19;ADAMTS5;SH3PXD2A;ADAM12;DAG1;HAS2;CD47;JAM2
BHLHB9;IL1RAPL1
SIN3A;NSD1
SEMA3A;SLIT2
CALM1;TSHR
IGBP1;APC
SDR16C5;SET
PLA2G12A;ABHD4
SIX1;DKK3
CREBL2;PTPN11
GPC1;E2F1;GPC5;LRP8
CNOT6;CARM1;E2F1;CDK1
RANBP3;PRICKLE1;HK2;RAB11A
C1QBP
DYRK3
SULF2

HNRNPU
SIX1
RC3H1
SLC30A4
HIF1A
SORL1
CA12
NCKAP1
PPP2CA
RAP1A
ID2
CTNNB1
PTPN11
GLS
SORL1
ROCK1
TDG
FGFR1
SRD5A1
PAX2
NSG1
RFWD3
ESR1
RUNX1
EDEM1
TERF2IP
RFWD3
KIF5C
MACROD2
SLIT2
CADM3
UQCR10
CAMK4
GIGYF2
BTRC
MCTS1
RFWD3
DOCK5
KAT2B
HNRNPU
ACTN4
FGFR1
MOCS3
HECW2
CUL3
CDC73
RFWD3
SESN3
PRKG1
BFAR
CTNNB1
GABRB1
ABHD2

RAB3GAP2
LYN
CDC73
VAPB
HEYL
GATA6
DIO2
LYN
AP2M1
DKK3
PPP2CA
HECW2
GFPT1
CAMK4
CTBS
PRDM16
EPS8
APC
CDS2
IL1RAPL1
CD244
RAB11FIP5
CUL3
LYN
CADM3
SLC25A12
MAPK1
SCN5A
PAX2
SORL1
HIF1A
LYN;TNFAIP3;RC3H1
PPP2CA;CTIF;CNOT6;AGO1;RC3H1;LSM5;SMG7;PELO;EIF4E;DCP2
GALNT7;ST6GAL2;ST8SIA3;ST6GALNAC3
CNOT6;CARM1;E2F1;CDK1
GPM6A;CDH2;FZD5;PCDHB15
SP1;HIF1A;FGFR1
NDC1;ST13;LOXL4
PAPOLG;CDC73
EFR3A;IP6K1
SP1;TARDBP
SIN3A;CDK1
RORB;TMEM64
SORT1;KIAA0141
GAPVD1;STXBP5L
MYO1C;MIEF1
HIF1A;SULF2
FZD5;RUVBL1
CTNNB1;RAB11FIP5
TERF2IP;SMG7
PPP2CA;SFRP1
ROCK1;SELE
APC;DYRK1A

CXADR;MYPN
RAB3C;MAP2K1;RABGAP1L;RALA;TBC1D25;MAPK1;RAB11A;STXBP5L
APH1B;DDI2;KLK13;ASPRV1;ZMPSTE24;METAP2
ZMAT3;CUL3;JMY;E2F1;GSKIP;HIPK2
MAP3K2;MAP2K1;ZFP91;ADRA2B;ADRA2A;ADCY9;RAP1A;CDK1;PTK2B;MAPK1;CLSPN;RAF1;CRK
NAPA;NAPB;RAB3C;RAB2B;RABGAP1L;RANBP3;PRICKLE1;SORL1;RAB11A;TRAK2;SNX1;MYO1C;XPO4;C
LYN;ERBB4;PTK2B;IL6R
ING4;SET;CDK1;MCMBP;RBMS1;CLSPN;RAD9A
HDAC5;JMY;CTNNB1;ARID5B;TERF2IP;ESR1;LRP8;PPARGC1B;ARHGEF5;MTDH;HIPK2;LRP6
ARHGEF10;XIAP;RAB11A
HS3ST3B1;GPC1;GPC5
SET;PHAX;SNRPD3
PUM2
BMPR2
MME
LYN
CDC73
SGIP1
PDE1B
VAPA
MB
LYN
TNRC6B
NFATC3
CRP
OTC
PAX2
RANBP3
SDR16C5
PRTFDC1
PHACTR4
MTMR3
METTL16
GNL1
HDAC5
PDE1B
SULF2
SHISA9
RBL1
CADM3
TNRC6B
PTK2B
PAX2
ABCA2
RC3H1
DCP2
ESR1
HPGD
SRD5A1
C21ORF59
PTK2B
PRKAB2
HK2

AGTR2
RIMS3
VAPA
MME
SLIT2
KIF5C
FOXO1
SDR16C5
PPARGC1B
XIAP
ACTN4
AP2M1
CALM1
AGO1
SOX6
HIF1A
IL1RAPL1
AGO1
VKORC1
SIN3A
ZC3H14
VKORC1
KCNIP2
ZNF706
HOOK3
CRP
SDR16C5
RBL1
SIN3A
CRP
GPR37
HIPK2
TRAPPC8
PTK2B
MYO1C
PRKAB2
VTI1A
PSMD11
LRP6
GSKIP
FZD5
KAT2B
MYOD1
SLC24A2;SLC30A4;ATP2B4;SLC39A13;ANO6;SCN5A;SCN3B;SCN1A
HEYL;ITCH;ID2;TNFAIP3;PROX1;BTRC;ESR1;SMAD7
RBM3;SP1;NOS1AP;TYRP1;FAM129A;AGTR2;HIF1A;CD244;EREG
PDE3B;PLXNA2;GTPBP4;PLXNA3
DYRK3;SESN3
RIMS3;WLS
ELMO1;AP2M1
CREB1;CREBL2
ITCH;TNFAIP3
EPS8;FCHSD2

GTF2H1;GTF2H5
PAPOLG;CDC73
TNFAIP3;PAX2
CD244;EREG
ARHGEF10;RABGAP1L;SIPA1L1;TBC1D25;MTSS1L
PABPN1;FZD5;PDCD4;TNFAIP3;CDC73
EFR3A;ARL5B;RAP2A;ROCK1;SCN3B;ARL5A;RAPGEF6;RAB11A;LRP6
CNOT6;RAB3GAP2;PROX1
KAT2B;MYO1C;DEK
PPM1L;VAPA;VAPB;ST8SIA3
NAPA;CAPZB;DCTN2;VAPA;VAPB;CUL3;CAPZA1;VTI1A;TRAPPC8;DYNLL2
SET;XRCC5;GTF2H1;SMC1A;BACH1;ING4;PURA;CDC34;ERI1;TDG;RPA4;CDK1;NABP1;RBMS1;TERF2IP;C
SRPK2;CRP;MAP2K1;ROCK1;INHBA;POU3F1;HIF1A;SELE;ITCH;SP1;FUBP1;ID2;NOS1AP;E2F1;MAPK1
CALM1;SMAD7
CHRM3;BMPR2
INHBA;HIF1A
SDR16C5;SART3
C1QBP;SLIT2
LYN;RC3H1
APC;PUM2
ROCK1;RUNX1
TNFAIP3;NDNF
CNOT6;EIF4E
SYT5;SYT7
ADRA2A;EREG
MYOD1;SIX1
PROX1;PHOX2B
MYOD1;SIX1
CRP;ABCA1
SLC24A2;GABRA1;GABRB1;CLCN5;KCNIP2;GABRA3;ATP2B4;ANO6;ATP2B2;ATP2B1;RAF1;TPCN1
FZD5;RUVBL1;PPARGC1B
E2F1;CDIP1;HIPK2
ID2;GATA6;PHOX2B
MCTS1;NDC1;RXRA;NFIA;CALCOCO2;VAPB;DAG1;ELMO1;DEK;HCFC2;AP2M1;HIPK2
HEYL
BMPR2
MYOD1
EFEMP1
CALM1
PTK2B
ABCA1
UTP15
HIF1A
DPYSL3
SRSF1
ACTN4
EPS8
TBX5
CALM1
EPS8
ELK4
APPL1
RPP14

TARDBP
APC
HAS2
MCTS1
DLST
APC
PFAS
HIF1A
TMEM33
MME
DYRK1A
MYOD1
TSHR
TFAM
HPGD
CAMK4
TDG
SORT1
RC3H1
C1QBP
OMD
CTNNB1
KCNIP2
HIF1A
SEMA3A
FZD5
NFATC3
DAG1
BMPR1A
RORB
ERI1
TSHR
BMPR1A
RAP1A
DTL
PHF8
SORL1
CDK1
KAT2B
SDR16C5
UBE2J1
REEP1
CTNNB1
HOOK3
ABCA1
BMPR2
CXADR
CARM1
ROCK1
LMOD3
DLST
NSD1
CPSF7

NDC1;SEPT10;SEPT11;PSMD11;SEPT12;TMEM33;NSG1;RBX1
NAPA;VAPA;VAPB;CUL3
NCOA2;RXRA
RAP2A;PPM1E
ADRA2B;ADRA2A
MAP3K2;MAP3K9
HSPH1;HSPA4L
E2F1;DYNLL2
EPS8;FCHSD2
RUVBL1;RSF1
RUVBL1;RSF1
EFNB3;ACTN4
NOS1AP;AGTR2
E2F1;DYNLL2
PTPRM;SYNJ2BP
TEAD1;AMOT
HNRNPU;SMAD7
KLHL32;GATA6;INHBA
NCKIPSD;CAPZB;SH3KBP1;DAG1;RHOBTB3;PTK2B;MARK1
USP13;PRKAB2;FZD5;ROCK1;RUVBL1;TFEB;MTMR9;FOXO1;MTDH;SNX6;TPCN1
FNTB;TNFAIP3;XIAP;CALM1;SELE;METAP2
BCLAF1;CTNNB1;GTF2H1;GTF2H5;RBX1
ITCH;PSMD11;OCIAD1;HNRNPU;RUNX1
USP47;PSMD11;DTL;RBX1
RAF1;SYT7;EREG
SRPK2;VAPA;PROX1
TGFBR3;HEYL;BMPR1A
ABCA1;RXRA
SIX1;FGFR1
GTF2H1;GTF2H5
HDAC5;AGTR2
CREBL2;APPL1
AGTR2;CALM1
CTNNB1
DTL
IL1RAPL1
MEF2A
PTPN11
SNX6
ARHGEF10
ABCA1
FGFR1
CALCOCO2
INHBA
XIAP
MME
KIF5C
LYN
ID2
KCNIP2
ATP2B4
VAPA
ABCA1

GLUD2
PAX2
NCKAP1
CELF1
FBLN5
ERBB4
ATP2B4
BMPR2
PAX2
SUB1
PPARGC1B
EFEMP1
SMAD7
ABCA1
NSG1
TRIB2
CLSPN
RIMS3
AGO1
SIK3
HIF1A
GFPT1
IKZF3
ADRA2A
CRP
PTK2B
APH1B
CD244
HAS2
MCTS1
CPSF7
BCLAF1
S1PR1
IGF2BP1
NCKAP1
MAP3K2;MAP2K1;MAPK1;BTRC
NCKAP1;PTK2B;LMOD3
CPSF7;RXRA;SOD2;GRIN2B;GLS
SYT5;SNAP23;SYTL4;SYT7
CALM1;ADRA2A
GTF2H1;GTF2H5
KCNIP2;NOS1AP
PARVA;SRGAP2
CLSPN;DTL
GTF2H1;GTF2H5
B3GAT2;SULF2
RUVBL1;RSF1
ST8SIA3;ST6GALNAC3
LYN;MAP2K1;PTPN11;SYNJ2BP;CTGF;RPS6KA6;RAP1A;ERBB4;CNKSR3;PTK2B;SPRY1;RAF1;FGFR1
EFR3A;RAP2A;ROCK1;SCN3B;RAPGEF6;RAB11A;LRP6
RBM3;AMER1;FAM129A;INHBA;BTRC
LYN;SESN3;PTK2B;RORB;PAX2
SART3;RSF1;GTF2H1;GTF2H5;RBX1

ABCA1;NCOA2;ABCA2;RXRA;PITPNC1
STIM2;CTNNB1;ADRA2A
SFRP1;ROCK1;PTK2B
USP2;EIF4E;EREG
RAB2B;SYTL4;MYH10
CTNNB1
PRTFDC1
CAMK4
RND3
TRIM44
SLC1A4
GJC1
BMPR1A
TET3
SIX1
PROX1
TBX5
IKZF3
MIEF1
PTK2B
SLIT2
ROCK1
GRIN2B
RCSD1
PRKAB2
ADCY9
SPRED1
HDAC5
PGAM5
TDG
DAG1
CMPK1
SCN3B
MEF2A
ITCH
INHBA
RC3H1
CD244
NRXN3
GPR37
TNRC6B
NSG1
SMAD7
NUDT4
UQCR10
CUL3
SORT1;VPS54;HOOK3;SORL1
FBXW4;PSMD11;BTRC;RBX1
B3GALNT2;BMPR2;SERP1;ST8SIA3
ABCA1;RNF126;SNX1;AP5M1;SORT1;VPS13C;VTI1A;RHOBTB3;VPS54;NSG1;HOOK3;SNX6
CAMK4;RUNX1
ITCH;CTNNB1
NAPA;VTI1A

MTMR3;RAB3GAP2
MTMR3;SESN3
GTF2H1;GTF2H5
XRCC5;PHACTR4
TERF2IP;KPNA1
GTF2H1;GTF2H5
ITCH;C1QBP
PTK2B;SHISA9
EFR3A;PLA2G12A;MTMR3;MTMR9;PLEKHA3;IP6K1
LYN;ERBB4;IL6R
NAPA;NAPB;PTPN11;RAF1
CREBBP;PSMD11;BACH1;HIF1A;RBX1
RABGAP1L;TMEM33;TBC1D25;MAPK1;MYPN;ARHGEF5
NCKIPSD;LYN;NCKAP1;MYO1C;ELMO1;MAPK1;CRK
PARVA;PROX1;SRGAP2
NSUN3;C9ORF64;ADAT2
ST8SIA3;ST6GALNAC3;SUMF1
CTNNB1;SLIT2
NPFFR1;EIF2B2
VKORC1;SULF2
GPAM;SP1
HIF1A;IL6R
CREB1;ADCY9
TDRKH;FKBP6
RAB2B;SYTL4
RIC8B;FNTB;CALM1;METAP2
SYT5;SNAP23;VTI1A;SYTL4;SYT7
OMD;SLIT2;L1CAM;S100B;PAX2
SRPK2
RNF180
HNRNPU
ETNK1
HRK
SMC1A
SESN3
LRP8
SLC25A12
GRM5
HPGD
AP3M2
CDC73
HPGD
FZD5
PRKAB2
PROX1
GPCPD1
RUVBL1
KAT2B
RBL1
IL1RAPL1
CHST7
TNFAIP3
ROCK1

ROCK1
RAD9A
ETFA
LRP6
HECW2
HEYL
VAPA
BMPR2
ADRA2A
PTK2B
DYRK1A
SEMA3A
PTPN11
ERBB4
PRKG1
SMAD7
SMC1A
SRGAP2
ESR1
MYOD1
ADAMTS5
TBC1D25
NUDT4
IKZF3
AP3M2
SIX1
XPO4
STXBP5L
EOMES
XIAP
RAB3GAP2
HNRNPU
HSPH1
KCNIP2
LYN
HS3ST3B1
ID2
ADRA2A
FZD5
XRCC5
MAPRE2
DYNLL2
ARHGEF5
LYN
CDK1
FZD5
NCKIPSD;LYN;NCKAP1;MYO1C;ELMO1;MAPK1;CRK
NDC1;KAT2B;ESCO1;LOXL4;SENP2;PIAS1
B3GALNT2;GALNT7;ADAMTS5;TET3;DAG1;PGM3
TXNL1;MAPK1;SOD2;HIF1A;FOXO1;SLC25A24
NDC1;AGO1;PUM2
NDC1;AGO1;PUM2
NCKIPSD;LYN;NCKAP1;MYO1C;ELMO1;MAPK1;CRK

SCN5A;SCN3B
MYH10;SYTL4
PHLPP2;DAG1
XRCC5;TERF2IP
HNRNPU;SMC1A
BHLHB9;IL1RAPL1
HNRNPU;DYNLL2
APH1B;DAG1
ATP2B4;SORD
GPC1;OMD
EFR3A;IP6K1
PPP2CA;PPM1L;VAPA;VAPB;ST6GALNAC3;SUMF1
LYN;GRM5;ERBB4;PTK2B;IL6R;LRP8
APC;GPR75;CTNNB1;CTGF;SLC25A24
PROX1;SULF2;FGFR1
TRIM24;LNPEP;TRHDE
SYTL4;MYH10;ARHGEF5
C1QBP;KCNH1;FGFR1
RAB3C;RALA;STXBP5L
TNRC6B
APH1B
CREBBP
CRK
ATP2B4
CLDN1
MEF2A
DNMT3A
SIN3A
EIF4EBP2
RAB11FIP5
TERF2IP
GTPBP4
S1PR1
BMPR2
BMPR1A
CALM1
HIF1A
CHRM3
HNRNPU
ADCY9
ESR1
MAPRE2
SLC17A6
APPL1
BMPR2
USP13
PGAM5
PROX1
DNMT3A
CLDN1
PLA2G12A
ZFP91
NOS1AP

PROX1
CTNNB1
PTGFR;MB;ATP2B4;S1PR3;ATP2B2;ATP2B1;ESR1
MEF2A;OMD;SLIT2;S100B;PAX2
ACAP2;VAPA;SIX1;SORL1;RAB11A
HNRNPK;SGIP1
RUVBL1;RSF1
MOCS3;GPAM
ATP2B4;GRIN2B
SYT5;KIF5C
RUVBL1;RSF1
SCN5A;SCN3B
KIAA0141;GRIN2B
DPY19L2;KLHL32
NCOA2;RXRA
RIMS3;WLS
USP13;EDEM1;YOD1;UBE2J1
NCKAP1;PTK2B;PROX1;SORBS3
MME;PDE1B;MMP2;HNRNPU;RC3H1;PTPN11;HIF1A;LRP8;FOXO1;IL18BP;EREG;SFRP1;CDC34;DPYSL3;IL
PRKAB2;PHF20;SETD9;JMY;HIPK1;RAD9A;HIPK2
RXRA;SOD2;GLS
NDC1;PFKFB3;HIF1A
MOCS3;C9ORF64;ADAT2
PTP4A1;MYO1C;APC;SEMA3A;HAS2;PTK2B;ACTN4;SOD2;ADRA2A
NDC1;CREBBP;HSPH1;MAPK1
LYN;NDC1;PPP2CA;GRM5;INHBA;EREG
HECW2;SCN3B
ABCA2;ABHD2
MOCS3;SRD5A1
USP47;TDG
E2F1;DYNLL2
SYT5;GABRA1;GABRB1;GRM5;KCNIP2;GABRA3;PCDHB15;MAPK1;PDE7B;BSN;GRIN2B;LRP6
CREBBP;FZD5;XRCC5;CTNNB1;HIF1A;IL6R;ADRA2A;RUNX1;EREG;PUM2;SULF2
SNX1;NSG1;AP2M1
EDEM1;YOD1;UBE2J1
CUL3;PTK2B;CD47
MAPK10;USP1;DTL
LYN;GPR37;DUSP19;GAB1;PTPN11;ACTN4;S100B;LITAF;ADRA2B;ESR1;ADRA2A;MTDH;EREG;PUM2;BCL
ABCA1
SYT7
ABCA1
PPARGC1B
ANO6
SRSF1
PUM2
RAB11A
MYRF
PAX2
APC
WDR37
AMER1
TET3
PELO

APPL1
GPCPD1
CALM1
SPRED1
CAMK4
MOCS3
CALM1
PSPH
SP1
CAPZB
MACROD2
DENND5B
RAB11A
ADK
NOS1AP
HNRNPU
ITCH
APC
APC
CLSPN
AMER1
ERI1
PTK2B
DLST
GNA13;ARHGAP20;ARHGAP31;SLIT2;SRGAP2;SOS2;ARHGEF5
SEPT10;RAP2A;SEPT11;SEPT12;C21ORF59;ARL3;S1PR1;PARVA;ANO6;SRGAP2;DYNLL2;RAPGEF6
MEF2A;MAPK1;SCN5A;KCNH1;SLC25A24
ABHD4;OTC
ATL3;CSNK1A1;ATL2;VTI1A;CDK1;TRAPPC8
KCNIP2;SCN5A;SCN3B;KCNH1;SCN1A
ROCK1
ACSL6
RAB11A
PPP2CA
XRCC5
DPY19L2
RNF126
ABCA1
SOD2
PPP1R1B
DNAL1
APC
ABCA1
RCSD1
CADM3
GDF6
CALM1
PLA2G12A
SLIT2
DNMT3A
CYTH3
OSTN
DAG1

KIF5C
SOD2
TGM2
PPP2CA;ADD2;HIPK2
MEF2A;MYOD1;SOX6
PPM1L;VAPA;VAPB
ZMAT3;JMY
ALAD;TMTC3
KCNH1;FGFR1
RUVBL1;RSF1
SESN3;MAPK1
ACSL6;DLST
SFRP1;TNFAIP3
SYT5;AP2M1
PCYT1B;PLA2G12A;MTMR3;GPAM;ETNK1;MTMR9;PLEKHA3;CDS2
TM7SF3;SYTL4;MYH10;WLS
PHLPP2;PPP2CA;MTMR3;PTPRM;BTRC;PTPN4
CA12;CYB5R4;SLC22A17;SLC4A10;PITPNC1;SLC25A24
HIPK1;HIPK2
PLA2G12A;GPAM
OTUD4;SLIT2
GPAM;SP1
BTRC
HNRNPU
SDR16C5
TNFAIP3
SYNJ2BP
SORL1
CHRM3
ERI1
TRAK2
RAP1A
LRP6
ANO6
ADCY9
MIEF1
SET
SET
APPL1
HAS2
PTK2B
AP2M1
ANO6
LYN
DLST
ADRA2A
TERF2IP
BTG1
CTNNB1
GPAM
L1CAM
ROCK1
AP2M1

SCN3B
NSG1
TRIP12
GPAM
ATP2B4;TERF2IP;CREBL2;RAF1
NDC1;CREBBP;DYRK3;HSPH1;MAPK1
KCNIP2;SCN5A;SCN3B;KCNH1;SCN1A
MAPK10;MAPK1;BTRC
RPP14;C9ORF64;ADAT2
CTIF;EIF2B2;EIF4EBP2
CREBBP;XRCC5;CTNNB1
USP47;PSMD11;RBX1
LYN;RAP2A;CCND3;PFKFB3;ERBB4;FAM129A;WDFY2;HIF1A;EREG;FGFR1
MRPL42;TSFM;EEF2K;LTN1;MRPL27
CHRM3;CSNK1G3;SERP1;CDC34
GPAM;SP1
CDH6;CDH2
SP1;RSF1
PLA2G12A;GPAM
NDC1;RAP1A
XRCC5;TERF2IP
TFAP2B;CYB5R4;ADRA2A
ITCH;BTRC;BTBD9
TNFAIP3
MTMR3
INHBA
RUNX1
ABHD2
ETNK1
TSFM
SRD5A1
GRIN2B
CTNNB1
APC
RUNX1
OTUD4
SFRP1
RAB11A
NFATC3
ABHD2
LYN
RAB11A
TFAM
RUNX1
CD244
SORT1
ID2
ABCA2
COA1
JMY
TRAPPC8
LYN;RIMS3;SCG3;ACTN4;CALM1;SYTL4;RAB11FIP5
KCNIP2;SCN3B

PPP2CA;ST6GALNAC3
TNFAIP3;IL6R
TFAP2B;SORD;SLC25A12
HRK;FZD5;RUVBL1;SNIP1;MIEF1;PPARGC1B
NDC1;ITCH;NFIA;DEK;KPNA1
SFRP1;PSMD11;RC3H1;HAS2;BTRC;HIF1A;RBX1
RPA4;GTF2H1;RNF111;GTF2H5;RBX1
LYN;IGF2BP1;DTX4;ADRA2A;EREG
ARHGEF10;SFRP1;RABGAP1L;SIPA1L1;RAP1A;MTSS1L;TBC1D25;SRGAP2;RAPGEF6
TNFAIP3;PAX2
IL6R;EREG
ZNF148;CALM1
FZD5;C1QBP
FZD5;CD244
CUL3;RAB11A
HAS2
GFPT1
PHF8
IKZF3
FZD5
MIEF1
MEF2A
NCKAP1
GALNT7
ADRA2A
SNX6
MAPK1
CRP
SET
COA1
APC
SELE
PLA2G12A
PHOX2B
EPS8
COX7B
SMAD7
CDC73
MAP3K2;MAPK10;MAP3K9
MYO1C;FZD5;XPO4;RUVBL1;SNIP1;MIEF1;SORL1
SORD;SLC25A12
ITCH;TNFAIP3
SCN5A;SCN3B
DCTN2;TNKS
NDC1;CDK1
CUL3;RAB11A
SLC4A10;ANO6
GPAM;SP1
STAU1;RSF1
SFRP1;ROCK1
OTUD4;LYN
PRKAB2;MTMR3;YOD1;PGAM5;DYNLL2
FZD5;RUVBL1;E2F1;SNIP1;DYNLL2

HIF1A
MOCS3
CALM1
HOOK3
SET
GPC1
RBBP5
SIX1
NSUN3
PDCD4
ADK
PIAS1
APC
GALNT7
PAX2
SMAD7
SRD5A1
GATA6
SORT1
PTK2B
CTNNB1
TMTC3
CD244
SELE
ROCK1;SORL1;GTPBP4
USP47;CREBBP;MME
NDC1;AGO1;PUM2
NDC1;ATXN7
GATC;TFAM
LETM1;TFAP2B
TFAP2B;SORD
PDHB;HK2
SFRP1;APC;CALM1;RAB11A
SPRED1;PPP1R1B;SLC7A14
ABCA1;RXRA;CNBP
NDC1;SENP2;PIAS1
SORL1
HK2
PTK2B
HOOK3
RBX1
IL1RAPL1
CHRM3
MAPK1
WDR37
EFNB3
TFAP2B
CALM1
GRM5
SLC30A4
SEMA3A
CLSPN
ABCA1

TARDBP
HRK
EIF4EBP2
GLUD2
PDHB;HK2
DDX19B;AGO1
NDC1;PRDM16
XRCC5;RAD9A
NDC1;CDK1
CHRM3;GRM5;ESR1;ADRA2A
TNFAIP3;ESR1
ATP2B4;PROX1
PPP2CA;CHAD
HSPH1;HSPA4L
RAB3C;RAB2B;RAB11A
SP1;NABP1;PHAX
SP1;NABP1;PHAX
NDC1;NAPA;NAPB;RAB3C;RAB2B;RABGAP1L;RAB11A;NEURL1B;SNX1;RAP1A;RAB3GAP2;TBC1D25;SNX
CDC73
MTMR3
CDK1;SIX1;PROX1;TBX5;PAX2
PLA2G12A;PCYT1B;ABHD4
RANBP3;SNRPD3;PRICKLE1
HOOK3;RAB11A
RFWD3;USP1
RNASE6;UBE2G1
SORT1;HOOK3
COX7B;ETFA;UQCR10;SLC25A12
USP47;XRCC5;GTF2H1;SMC1A;BACH1;GTF2H5;RFWD3;TDG;RPA4;USP1;CDK1;NABP1;RAD9A
GPR75;SORL1;GRIN2B
SPRED1;SLC7A14;MTMR9
CTNNB1
ACBD5
AP2M1
CARNS1
CDC73
APC
MAPRE2
SHISA9
KAT2B
SHISA9
TNFAIP3
ERBB4
XIAP
RC3H1
ADCY9
RBL1
C1QBP
APH1B
PUM2
CDC73
OSTN
ROCK1

HK2
MAP3K2;MAP2K1;CDK1;MAPK1;MAP3K9
MYO1C;E2F1;MIEF1;DYNLL2
GTF2H1;AFF4;CDC73;GTF2H5
ITCH;BFAR
HK2;OTC
SPRED1;BCLAF1
ABCA1;ABHD4
ABCA2;RXRA;CTNNB1;ESR1;SELE;RELT
ABCA1;ITCH;RAP1A;CAPZB;NRG3;PTPN11
SLC24A2;STIM2;ATP2B4;ANO6;ATP2B2
GTF2H1;GTF2H5;RBX1
RANBP3;HNRNPU;SIX1;PRICKLE1
DCTN2;CDK1;HAUS3;RAB11A
PSMD11;DCTN2;HECW2;ID2;CDK1;E2F1;HAUS3;BTRC
PDE1B;CAMK4
SNX1;AP2M1
KCMF1;MGAM;PSMD11;ROCK1;MME;XRCC5;SNAP23;PDAP1;ANO6;ATP11A;ALAD;RAP2C;PGRMC1;RAP1
CRK
PITPNC1
HK2
COA1
GRM5
EREG
ACBD5
CREBL2
SIK3
FZD5
SIN3A
PTPN11
GAB1
ERI1
PRKAB2
ACSL6
PROX1
C1QBP
CYB5R4
PAPSS2
CRK
CTNNB1
PTPN11
HK2
NOS1AP
HK2
PTK2B
ATP2B4
TFAP2B
SLC1A4
HPGD
SEPT10;SEPT11;SEPT12;TNKS;TMOD3;C21ORF59;ARL3;TMOD2;NEK7;RC3H1;HAUS3;PARVA;TRAPPC8;[illegible]
IGBP1;SFRP1;HAS2;SELE
RBM3;HSPH1;FAM129A;RAB3GAP2;HIF1A;EREG
PSMD11;AZIN1

XRCC5;TERF2IP
PSMD11;AZIN1
SLC24A2;ABCA2;GABRA1;GABRB1;PSMD11;GABRA3;ATP2B4;ANO6;ATP2B2;ATP2B1;ADD3;ADD2;TPCN1
USP47;CREBBP;USP1;DTL
FZD5;TNFAIP3;SELE;RELT
TNFAIP3;XIAP;SELE
LYN;CREBBP;PSMD11;BTRC;RAF1
KCMF1;MGAM;PSMD11;ROCK1;MME;XRCC5;SNAP23;PDAP1;ANO6;ATP11A;ALAD;RAP2C;PGRMC1;RAP1
XRCC5
GDF6
C1QBP
ADRA2A
SCN5A
VKORC1
HRK
RC3H1
RSF1
TFAP2B
TGM2
TFAP2B
CTNNB1
LYN;PRKAB2;PHF20;SETD9;XIAP;INHBA;HIPK1;GDF6;HIPK2;GNA13;ARHGAP20;ARHGAP31;SPRED1;JMY
GPR75;PTK2B
BTRC;ADRA2A
ITCH;PSMD11;RUNX1
PSMD11;CDK1;BTRC
HRK;BCLAF1;AGTR2
PSMD11;CDIP1;PTK2B;RELT;TXNDC17
PTK2B
SLC24A2
LETM1
VKORC1
TERF2IP
INHBA
LETM1
INHBA
PLA2G12A
RIMS3
CRP
KAT2B
PLA2G12A
EIF2B2
KCMF1;MGAM;PSMD11;ROCK1;MME;XRCC5;SNAP23;PDAP1;ANO6;ATP11A;ALAD;RAP2C;PGRMC1;RAP1
RFWD3;NABP1;RAD9A
ITCH;PSMD11;RUNX1
CUL3;CALM1;RAB11A
NCOA2;RXRA
ERBB4;BMPR1A
RBL1;ERBB4;HNRNPU;FGFR1
LYN;CREBBP;PSMD11;BTRC;RAF1
LYN;SCG3;ACTN4;CALM1;SYTL4
RBM3;C1QBP;FAM129A
ERBB4;RAF1;FGFR1

PSMD11;SNAP23;LNPEP
EFEMP1;ATXN7;PAX2
NDC1;KPNA1
DFFA;RAF1
MAP2K1;BCLAF1
NDC1;KPNA1
GNA13;AGTR2;ADRA2B;ADRA2A;TSHR
SFRP1
CHRM3
DYRK1A
MIEF1
PTK2B
CALM1
PRKAB2
TSHR
CAPZB
XPO4
RNASE6
ALAD
SELE
UQCR10
PSMD11;CUL3;CDK1
SART3;SET;RSF1
DCTN2;CDK1;HAUS3;BTRC;BACH1
USP13;ROCK1
GRAMD4;GRIN2B
PSMD11;ZFP91;BTRC
ITCH;C1QBP;TNFAIP3
ZDHHC18;ABHD17B;ZDHHC20;ZDHHC21;ABHD17C
SLC25A12
LOXL4
SYT5
OMD
HIF1A
FZD5
RC3H1
MAPK1
PROX1
RANBP3;PRICKLE1
ROCK1;MTMR9
ALAS2;SLC22A17
TNFAIP3;HIPK1
SLC22A17;SLC17A6
PTK2B;ZNF148
FZD5;RUVBL1;SNIP1
EFEMP1;ATXN7;PAX2
PTK2B
CALM1
IL1RAPL1
ADCY9
RAB11A
ACSL6
CALM1

SORL1
SIX1
C1QBP
HDAC5
RFWD3
ABHD2
RAP1A
ETFA;UQCR10
APC;GRIN2B
CUL3;SMC1A;RAB11A
OTUD4;USP9X;EREG
PSMD11;CDK1;BTRC
MRPL42;TSFM;MRPL27;GATC
CUL3;ID2;GATA6;EREG
ITCH;C1QBP;TNFAIP3;CRK
ERBB4;RAF1;FGFR1
SGIP1;INHBA;HIF1A
ABCA1;ABCA2
ABCA1;ABCA2
SIPA1L1
GTPBP4
TNFAIP3
ALDH6A1
TRAK2
XPO4
SLC39A13
TNFAIP3
RAB11A
MOCS3
FZD5
SLC39A13
TM7SF3
MAPK1
CRP
MYPN
CALM1
SEPT10;SEPT11;SEPT12;DCTN2;C21ORF59;ARL3;CDK1;HAUS3;PARVA;DYNLL2;MAPRE2;RAB11A
GTF2H1;AFF4;CDC73;GTF2H5
NCKAP1;PTK2B;PROX1
HNRNPU;SMC1A;EREG
DCTN2;CDK1;HAUS3
PABPN1;PDCD4;TNFAIP3;SELE;RELT;CDC73
SLC24A2;GABRA1;GABRB1;CLCN5;GABRA3;ATP2B4;SLC17A6;ANO6;ATP2B2;ATP2B1;RAF1;TPCN1
MAP2K1;GPR37;RAP1A;ERBB4;DUSP19;PTK2B;PTPN11;RAF1;ADRA2B;ADRA2A;CTGF;FGFR1
ABCA1;RXRA
GLUD2;DLST
IGF2BP1;BTBD9
MAP2K1;RAP1A;ERBB4;PTK2B;PTPN11;RAF1;CTGF;FGFR1
MEF2A
SESN3
ADCY9
MAPRE2
NUDT4

PRKAB2
TDG
RUNX1
SLC30A4
PCYT1B
SMC1A
PTGFR;JPH3;S1PR3;ESR1;JPH1
APH1B;MME;DDI2;MMP2;KLK13;FBXO3;LNPEP;ASPRV1;TRHDE;LRP8;ZMPSTE24;METAP2
MAPK1;SOD2
SART3;RSF1
ITCH;C1QBP;PDCD4;TNFAIP3
CALM1
DNAL1
GALNT7
NDC1
NDNF
NDC1
RAP2B
BCLAF1
PTK2B
XPO4
C1QBP
STIM2
VAPA;TNFAIP3;TERF2IP;S100B;LITAF;ESR1;WLS;MTDH
KCNIP2;KCNH1
RABGAP1L;TBC1D25
PSMD11;AZIN1
SRPK2;PROX1
RBM3;FAM129A
SLC25A51;SLC25A45;SLC25A12;SLC25A24;OTC
MRPL42;TSFM;MRPL27
ABCA1;ABCA2;SLC22A17
PSMD11;CDK1;BTRC
LYN;MAPK10;PSMD11;NFATC3;MAPK1;BTRC;CALM1
LYN;MAPK10;PSMD11;NFATC3;MAPK1;BTRC;CALM1
PPP2CA;MME
UBE2G1;AP2M1
IL6R
CUL3
SIK2
FZD5
TRIM24
CRP
PAX2
OMD
SESN3
FZD5;RUVBL1;SNIP1
NAPA;CUL3
ALAS2;SLC22A17
NAPA;CUL3
SPRED1;SLC7A14
NAPA;CUL3
CAPRIN1;IGF2BP1;GIGYF2

ACTN4
DYRK1A
DIEXF
NDC1
CNBP
ANO6
TNFAIP3
HK2
RFWD3
RNASE6
EREG
NCKAP1;PTK2B
COA1;TIMMDC1
COA1;TIMMDC1
COA1;TIMMDC1
HNRNPK;SGIP1
VAPA;TERF2IP;S100B;LITAF;WLS;MTDH
GALNT7
CD244
KCNIP2
HOOK3
ABHD2
CDK1
LETM1
CNBP
ST6GALNAC3;SUMF1
CALM1;RAB11A
CRP;RNASE6
ACBD5;ABHD2
SART3;RSF1
INHBA;HIF1A;SELE
SLC22A17
GPAM
CAMK4
NDC1
NKAIN1
APC;CDK1
USP47;CREBBP
ALAS2;SLC22A17;SLC39A13
AP2M1;APPL1
CTNNB1;EREG
TPM3
TPM3
RAP2B
SLC4A10
CLDN1;ASPRV1;EREG
SEPT10;SEPT11;SEPT12;C21ORF59;ARL3;PARVA;DYNLL2
PSMD11;BTRC;RBX1
PFAS
ANO6
PFAS
TM7SF3
CRP

CAPZB
NDC1
SFRP1;PSMD11;CDIP1;HAS2;PTK2B;RELT;TXNDC17
SESN3
RAP1A
GATC
CNBP
HRK
LYN
EREG
RSF1
DAND5
DYNLL2
CTNNB1
CALM1
COA1;TIMMDC1;UQCR10
SFRP1;RAP1A;MTMR9;SRGAP2;CALM1;RAPGEF6
SORT1;SORCS1
USP47;SFRP1;L1CAM
MTMR3;MTMR9;PLEKHA3
RFWD3;NABP1
PSMD11;CDK1
RNASE6;IL6R
USP47;SFRP1
SYT7
GRM5
CHAD
IL6R
RABGAP1L
ROCK1
SLC25A12
CUL3
MRPL42;COA1;CARNS1;MRPL27;EIF4EBP2;PELO
GPAM;SRD5A1
PSMD11;CDK1
DAND5
RAD9A
CDK1
ST8SIA3
DTL
FZD5;CD244
DCTN2;CDK1;HAUS3;BTRC
NDC1
GATA6
TDRKH;TMEM203;RUVBL1;SPATA2;FKBP6
PDCD4;EREG
TDRKH;TMEM203;RUVBL1;SPATA2;FKBP6
OTC
CD244
CD47
SORL1
SLC4A10
ABCA1

PSMD11;SNAP23
ADCY9;PAPSS2
LYN;CRP;PTGFR;HDAC5;ITCH;NFATC3;RELT;SELE;NFE2L1
RFWD3;NABP1
MME;LNPEP;TRHDE
UBE2K
PSMD11;CDK1
DYNLL2
DIEXF
HPGD
MTMR9
MRPL42;TSFM;COA1;EEF2K;MRPL27;GATC;EIF4EBP2;PELO
TMEM33;TMTC3;FAM129A
ACBD5;HPGD;GPAM
BHLHB9;FRMPD4;IL1RAPL1
ADAMTS5;MMP2
PDCD4;TNFAIP3
ATP2B2;CCDC50
NDNF
BCLAF1
FZD5
MTMR3
DTL
ABHD2
ANO6;SLC1A4
CSNK1A1;ELMO1;SYT7;TGM2
IL6R
SLC1A4
MAP3K2
MAPK1;FGFR1
NAPA;ERGIC2
SNX1;HSPH1;TMEM108;SORL1;AP2M1;LRP6
ATP2B2;CCDC50
ADD2;HIPK2
TRIM24;UBE2G1
NSUN3
RNASE6
PAX2
XRCC5
ITCH;TNFAIP3
ETFA
XRCC5
RCAN1
PPP2CA;CTIF;SMG7
NPFFR1
SLC1A4
ACSL6
L1CAM
HDAC5
GPAM
COX7B;UQCR10
FGFR1
TEAD1

CXADR
BHLHB9;IL1RAPL1
EREG
MME
OTC
CHRM3;CSNK1G3;COA1;USP9X;MMP2;GATA6;MRPL27;UBE2G1;SORL1;PCMT1;MRPL42;CDC34;APH1B;S
MRPL42;MRPL27
RBX1
CXADR
TRAPPC8
NUDT5
C1QBP
L1CAM;FBLN5
PDCD4;PUM2
ROCK1
TRAPPC8
CXADR
AGTR2
EREG
RALA;PSMD11;MMP2;GPR75;PTPN11;SOD2;HIF1A;LRP8;FOXO1;IL18BP;RBX1;TXNDC17;EREG;CAPZA1;C
PARVA
TNFAIP3;IKZF3
TNFAIP3
MRPL42;MRPL27
GFPT1;ITPK1;IP6K1;CALM1;NUDT4
SOD2
WDR37
GPCPD1
CHAD
DTL
GALNT7
DYRK3
CALCOCO2
CD47
PDCD4;TNFAIP3;XIAP;SELE
SYT7
GPAM
SPRED1;SLC7A14
GPAM;PDHB
XRCC5;RFWD3;NABP1
BCL2L13;GRAMD4
HDAC5
HDAC5
PDCD4
UTP15;WDR37;ERI1;RPP14;DIEXF
SLC1A4
TNFAIP3
BCL2L13
SRD5A1
CTGF
SORL1
CXADR
ATP2B2

TERF2IP;MTDH
PSMD11;EIF2B2;BTRC
UTP15;ERI1;RPP14;GNL1;DIEXF
UTP15;ERI1;AGO1;RPP14;DIEXF
SEC62
TNFAIP3
UTP15;ERI1;RPP14;DIEXF
SEC62
SRD5A1
ELMO1
CD47
ELMO1
C1QBP
C1QBP
NDC1
C1QBP
NDC1
C1QBP
C1QBP
CRP
CRP
C1QBP
CRP;RNASE6;IL6R
LYN;CXADR;XIAP
PSMD11;EIF2B2;BTRC

;C1QBP;TRPS1;EPC1;BTRC;ZNF366;MEF2A;EOMES;KLF10;USP47;USP2;ARID5B;PROX1;HCFC2;CBF/
PIAS1;RUNX1;SFRP1;TBL1XR1;ZNF318;TFAM;DLX1;TNKS;GATA6;GLIS3;CREBL2;HIF1A;FOXO1;NPAT;
OX1;POU3F1;RUNX3;PAX2;PIAS1;RUNX1;SFRP1;CREB1;TBL1XR1;ZNF318;TFAM;MAPRE3;MYRF;GAT/
;EBF1;ARID5B;HCFC2;RUNX3;FOXP2;RUNX1;TBL1XR1;SUB1;TFAM;CGGBP1;DLX1;TSHZ3;TNKS;GAT/
YRK1A;PROX1;PCDHA13;PCDHA12;HPCAL4;PCDHA11;PCDHA10;DCX;GPM6A;DLX6;SHOX2;TRAK2;E
NX1;ID2;IL1RAPL1;ID4;DCX;CTNNB1;LRP12;MARK1;FGFR1;ITM2C
SIN3A;EPC1;TEAD1;RNF111;MEF2A;EOMES;KLF10;NCOA2;EBF1;TFEB;PROX1;PAX2;RUNX1;KAT2B;C
ID5B;POU3F1;HCFC2;RUNX3;FOXP2;PIAS1;EREG;DKK3;RUNX1;SFRP1;TBL1XR1;SUB1;ZNF318;TFAM
;MAP2K1;DYRK1A;PROX1;POU3F1;RUNX3;PAX2;PIAS1;RUNX1;KAT2B;SFRP1;CREB1;TBL1XR1;ZNF3
BT2;DNMT3A;ARID5B;PROX1;CBFA2T2;PAX2;FOXP2;DKK3;EREG;SFRP1;RBL1;ZNF318;DCP2;HDAC5
A2T2;GTPBP4;PAX2;FOXP2;DKK3;EREG;SFRP1;ZNF318;HDAC5;SET;TSHZ3;GATA6;PRICKLE1;ZBTB4;
;NKRF;SIN3A;PRDM16;E2F1;EPC1;ZNF148;ZNF423;BTRC;SNX6;KLF10;TFAP2B;USP47;CBX5;ZBTB14
NX1;MTDH;HIPK2;SFRP1;EFNA3;SPRED1;SP1;AGO1;ADAM12;CTNNB1
ES;KLF10;USP2;ARID5B;PROX1;HCFC2;RUNX3;TBL1XR1;CGGBP1;KANK2;DLX1;HDAC5;GATA6;GLIS

RP6;MAPK10;ID2;IL1RAPL1;ID4;CTNNB1;ITM2C

PK10;VAPA;TMEM108;NPTXR;MAPRE2;SH3GL2;LRP12

14;RXRA;PABPN1;SALL4;TRPS1;TRIM24;SNRPD3;HIVEP2;ZNF148;TEAD1;ATOH1;CREBBP;CPSF7;NF/
W4;SMURF2;ZFP91;LNPEP;UBE2G1;BFAR;FBXO30;RBX1;RNF126;ITCH;CDC34;HECW2;RLIM;TRIP12

;TRIP12;BTRC;UBE2K;DTL
;SMURF2;CSNK1A1;UBE2G1;BFAR;BTBD9;RBX1;RNF126;ITCH;CDC34;APC;TBL1XR1;HECW2;RLIM;C
1;HIVEP2;ZNF148;ATOH1;MEF2A;NFATC3;GTF2H1;PAX5;DEK;SMARCA1;RUNX3;ESR1;PAX2;GTF2H5;
;GPC1;MYOD1;CARM1;CTNNB1;WDFY2;SOX6;IL6R;BMPR1A

;
1;CTNNB1;SIK2;SLIT2;PTPN4

EMP1;E2F1;PDCD4;PTK2B;TRIB2
MECOM;ID2;ID4;CDK1;PDCD4;PIM3;TARDBP;DTL;PHACTR4

PHF8;RCAN1;RPS6KA6;CDH2;DCX;AGTR2;PAFAH1B2;SH3GL2;ATOH1;FGFR1

NF217;TRIM24;LONRF3;BTRC;UNKL;RNF111;FBXW4;CRBN;SMURF2;USP9X;MSL2;LNPEP;UBE2G1;KL

1;BFAR;BTBD9;RBX1;RNF126;ITCH;CDC34;APC;TBL1XR1;HECW2;RLIM;CDK1;CTNNB1;UBE2K;RNF1

PC;TBL1XR1;HECW2;RLIM;CDK1;CTNNB1;BTRC;UBE2K;RNF111;RNF165

RK1;LYN;SRPK2;MAP3K2;CSNK1G3;MAP2K1;CSNK1A1;NEK7;DYRK1A;GTF2H1;CDC42BPA;GDF6;RUN
IA2;MAPK1;SLIT2;PLXNA3;RNF165

S
P6;SFRP1;ZNRF3;TNKS2;APC;TBL1XR1;RUVBL1;BTRC;WLS

1;RSPO2;WLS

I;TRIM44

ABPN1;RBMS1;SNRPD3;HNRNPC;TARDBP;SRSF9

;MAD7;KAT2B;TNKS2;APC;ATXN7;RUVBL1;USP1;CDK1;CLSPN

OX1;INHBA;TBX5;SOD2;RUNX3;GTPBP4;EREG;TGFBR3;KAT2B;SFRP1;APC;ID2;CTNNB1;STRN;RAF1

11;BMPR1A

RBB4;PTK2B;MAPK1;AP2M1;APPL1;LYN;SORT1;MMP2;GAB1;PTPN11;GRIN2B;SULF2;EFNA3;APC;ELN

11;NDC1;CRBN;SMURF2;USP9X;MSL2;UBE2G1;KLHL23;SENP2;RBX1;PIAS1;ITCH;CDC34;HECW2;RF
;MAD7;KAT2B;TNKS2;APC;ATXN7;RUVBL1;USP1;CDK1;CLSPN

1;TRA2B;RAVER2;NAA38;SNRPD3;HNRNPC;SRSF10;SMNDC1;SRSF9
LIT2;CALM1;TARDBP;TRIB2

B;APBB2;SLIT2;PLXNA3;LYN;KLF10;TFAP2B;MAP2K1;VPS13C;PROX1;INHBA;TBX5;SOD2;RUNX3;GTF
P2B;RAP1A;CNKSR3;G3BP1;ELMO1;RAPGEF6;PHACTR4

;HNRNPK;SART3;PABPN1;TRA2B;RAVER2;NAA38;SNRPD3;HNRNPC;SRSF10;SMNDC1;SRSF9

IBNL2;SFMBT2;TFEB;RC3H1;PROX1;POU3F1;RUNX3;SFRP1;DDX19B;RBL1;MYOD1;NOS1AP;TFAM;TI

'R1A
IA3;ACTN4;TBX5;SOD2;SORL1;GTPBP4;ADRA2A;SMAD7;RAP2C;PTP4A1;SFRP1;MYO1C;APC

;PABPN1;TRA2B;RAVER2;NAA38;SNRPD3;HNRNPC;SRSF10;SMNDC1;SRSF9

1A1;NEK7;DYRK1A;CDC42BPA;RUNX3;HIPK2;MAPK10;CREB1;CAMK4;MYOD1;CDK1;SIK3;SIK2;RAF1;

\M;TEAD1
.SPN;CALM1

BL1;CTNNB1;CRK

ATP2B4;INHBA;GDF6;ADRA2B;ADRA2A;EREG;RAP2C;RAP2A;TERF2IP;MAPRE3;WDFY2;CALM1;RAF1

KIF5C;GPC1;MAPK1;SLIT2

2;LRP12

F10;EIF4E;PELO;MEF2A;NDC1;COA1;MRPL27;SMARCA1;SORL1;ESR1;PAX2;DHX40;DDX19B;HNRNPK

K9;MACROD2;BTRC;FBXW4;CSNK1G3;MAP2K1;DYRK1A;RUNX3;SUMF1;RBX1;CDC34;CREB1;MYOD1

NB1;SOX6;BMPR1A

TDC1

RP6;GRM5;FCHSD2;SLC17A6;MAPK1;KIF1B;PDE7B;BSN

EGR4;CNBP;PROX1;PAX2;ADRA2A;TSHR;EREG;TGFBR3;CNOT6;SFRP1;ID2;CD47;FGFR1;BMPR1A

QBP;JMY;E2F1;CTNNB1;SLIT2;SOS2;ARHGEF5;TGM2

6R;KCNH1;MCTS1;LYN;SRPK2;KLF10;TFAP2B;MAP2K1;EGR4;XRCC5;CNBP;PROX1;INHBA;TBX5;SOD

';REEP1;SNX1;RAP2A;TMEM33;RAB3GAP2;CTNNB1;TBC1D25;SNX8;WLS;SNX6

CDK1;E2F1;NABP1;TRIP12;MACROD2;CLSPN;DTL;RAD9A

)4;CDIP1;PTK2B;RAF1

BP;HAS2;PTK2B

6;SYT5;CSNK1G3;CCZ1B;SORT1;HOOK3;SYTL4;RAB11A;SYT7;RNF126;ACAP2;NECAP2;LRP12
OS2;ARHGEF5;TGM2

F4E

;AGTR2;BTRC;RAF1;FGFR1;PHACTR4

1;CRK;FGFR1

AP;TRIM24;TARDBP;RAF1;RELT

1

;USP47;FZD3;DFFA;BFAR;SOD2;PAX2;SFRP1;ITCH;HNRNPK;CDK1;CTNNB1;NAA35;NAA38;RAF1;FGF

LS

2F1;TRIM24;PTK2B;PIM3;SLIT2;RELT;TGM2;MAP3K2;TFAP2B;USP47;CREBBP;DFFA;MAP2K1;ANO6;BF

2K;CDK1;E2F1;EPC1;EIF4EBP2;RBMS1;CLSPN;PELO;RAD9A

P2B;USP47;EGR4;CNBP;ACTN4;PROX1;SORL1;ADRA2A;TSHR;EREG;CNOT6;SFRP1;APC;NOS1AP;CI

ALL4;E2F1;APBB2;HIVEP2;SOX6;SRSF10;EIF4E;PPARGC1B;NCOA2;TFAP2B;CREBBP;ESCO1;BCL11E

TI1A;SNX8;ERGIC2

2B;MAPK1;SLIT2

C;CALM1;RAF1;FGFR1

;SLIT2;TARDBP

\PK1;SNX8;LRP12;SNX6

ECOM;ERBB4;CDK1;NAA35;PTK2B;PIM3;NAA38;RAF1;FGFR1

:DH2;RBBP5;UBXN7;ASB7;SCG3;BTRC;DTL

VEP2;SOX6;SRSF10;PPARGC1B;NCOA2;TFAP2B;CREBBP;BCL11B;CNBP;TFEB;ACTN4;RUNX3;ESR1;

:DC37;RAB3GAP2;TBC1D25;SNX8;WLS;SNX6

[illegible]LSPN;RAD9A

L1RAPL1;CHAD;S1PR1;HAS2;PTPN4;CRK;IL6R;UBE2K;IL13RA1

AF1;VAPA;ERBB4;C1QBP;NOS1AP;PDCD4;TERF2IP;TRIM44;WLS;FGFR1

:8;WLS;SNX6

A;RAP2B;VAPA;CRISPLD2;MLEC;MAPK1;CD47;PAFAH1B2;FRK

YNLL2;RAB11A;LMOD3;SH3PXD2A

;CLCN5;SLC22A17;RAF1;SLC28A3

A;RAP2B;VAPA;CRISPLD2;MLEC;MAPK1;CD47;PAFAH1B2;FRK

;MAPK1;SRGAP2;SOS2;ARHGEF5;RAD9A

A;RAP2B;VAPA;CRISPLD2;MLEC;MAPK1;CD47;PAFAH1B2;FRK

;ERP1;CDH2;EIF4EBP2;SCG3;PELO

:HAD;PDCD4;S1PR1;CDIP1;PTK2B;BTRC;CRK;RELT;IL6R;IL13RA1

A2T2;RUNX3;FOXP2;PAX2;EREG;DKK3;SFRP1;CREB1;TBL1XR1;ZNF318;CGGBP1;KANK2;DLX1;HDAC
;PRDM16;ZNF423;PPARGC1B;TFAP2B;CREBBP;EGR4;FZD5;BCL11B;ZBTB16;SS18L2;NFATC3;INHBA;
A6;CREBL2;HIF1A;FOXO1;LRP6;NPAT;ERBB4;NSD1;PRDM16;NRIP1;E2F1;PCBD2;ZNF423;TRIM44;MA
A6;GLIS3;ZBTB4;HIF1A;FOXO1;MED12L;MTDH;PRDM16;ZNF148;PPARGC1B;ABCA2;TFAP2B;CREBBP
FNB3;PCDHA1;GBX2;ERBB4;PCDHA5;PCDHA4;PCDHA3;PCDHA2;PCDHA9;PCDHA8;PCDHA7;ATOH1;

CREB1;RBL1;TBL1XR1;SUB1;MYOD1;TET3;TFAM;DLX1;HDAC5;INSIG2;TNKS;PLAG1;GLIS3;HIF1A;FOX
M;ZNF275;SET;TSHZ3;GATA6;CREBL2;ZBTB4;HIF1A;FOXO1;MED12L;NPAT;ZNF706;PRDM16;HIVEP2;Z
318;NOS1AP;TFAM;MAPRE3;CRP;MYRF;ROCK1;GATA6;FAM129A;CREBL2;HIF1A;FOXO1;LRP6;NPAT;E
;SET;TSHZ3;GATA6;PRICKLE1;HIF1A;ZBTB4;GIGYF2;FOXO1;BCLAF1;ZNF706;PRDM16;IGF2BP1;E2F
GIGYF2;FOXO1;BCLAF1;ZNF706;PRDM16;IGF2BP1;E2F1;ZNF148;ZNF423;SNX6;TFAP2B;CBX5;ZBTB1
;SMURF2;XRCC5;ZBTB16;ARID5B;PROX1;CBFA2T2;PAX2;FOXP2;DKK3;EREG;HEYL;SFRP1;ID2;ZNF3

3;LRP8;ZBTB4;MTDH;RXRA;NSD1;PRDM16;NRIP1;E2F1;ZNF148;IGBP1;TFAP2B;CREBBP;CBX6;CBX5;

ATC3;GTF2H1;PAX5;DEK;TBX5;RUNX3;ESR1;PAX2;GTF2H5;KAT2B;MAF;SP1;FUBP1;CDK1;NABP1;TAF
;UBE2K;DTL;RNF165

DK1;CTNNB1;TRIP12;UBE2K;DTL;RNF165
;EREG;MAF;FUBP1;TFAM;TARDBP;NFE2L1

HL23;BFAR;BTBD9;FBXO30;RBX1;RNF126;ITCH;CDC34;HECW2;RFWD3;KLHL7;RLIM;TRIP12;DTL;UBI

165

NX3;HIPK2;SMAD7;TGFBR3;MAPK10;CREB1;CAMK4;MYOD1;CDK1;SIK3;SIK2;RAF1;FRK;FGFR1;BMPR

;FRK

MO1;CRK;FRK;FGFR1

WD3;KLHL7;RLIM;TRIP12;UBE2K

PBP4;PAX2;EREG;TGFBR3;KAT2B;SFRP1;APC;PDCD4;CTNNB1;STRN;AGTR2;RAF1;FRK

ERF2IP;CRP;USP13;ZNF275;HDAC5;ROCK1;YTHDC1;HIF1A;PPP2CA;NPAT;ZNF629;TDG;TRA2B;E2F1;

FRK;FGFR1;BMPR1A

1;UBE2K;FGFR1;BMPR1A

K;RBMS1;SRSF9

1;SIK3;SCG3;SIK2;RAF1;DTL;PHLPP2;MTMR3;PSMD11;CUL3;DCUN1D1;HIF1A;PPP2CA;FBXO40;RBBF

)2;GTPBP4;ADRA2A;TSHR;PIAS1;EREG;KAT2B;RNF126;CNOT6;SFRP1;APC;CTNNB1;STRN;CD47;RAF

R1

FAR;ACTN4;INHBA;GDF6;PAX2;SFRP1;ITCH;HNRNPK;APC;NOS1AP;JMY;CDK1;NAA35;CTNNB1;NAA3[illegible]

D47;FGFR1

3;CNBP;TFEB;RUNX3;ESR1;NFIA;SP1;CARM1;TFAM;TERF2IP;SSBP2

;NFIA;SP1;CARM1;TFAM;TERF2IP;SSBP2

C5;SET;TSHZ3;GATA6;GLIS3;PRICKLE1;LRP8;ZBTB4;FOXO1;MTDH;BCLAF1;RXRA;ZNF706;NSD1;PRD
SMARCA1;GDF6;ESR1;PHOX2B;SMAD7;HNRNPK;SP1;AGO1;ID2;LHX4;NFE2L1;BMPR1A;BMPR2;ONE
.P3K2;TFAP2B;CREBBP;ZBTB16;CNBP;SS18L2;INHBA;TBX5;SMARCA1;GDF6;ESR1;HIPK2;SP1;ID2;CA
';CBX6;CBX5;EGR4;FZD5;BCL11B;SMURF2;ZBTB16;SS18L2;NFATC3;SMAD9;INHBA;GDF6;ESR1;PHO)
;PCDHA6;LYN;EIF2B2;FZD5;BCL11B;ZBTB16;SLC4A10;PCDHB15;INHBA;L1CAM;S100B;PHOX2B;TSHR

(O1;LRP6;RXRA;PRDM16;NRIP1;E2F1;PPARGC1B;ZNF462;TFAP2B;CREBBP;EGR4;FZD5;BCL11B;CNE
ZNF423;ZNF148;PPARGC1B;SNX6;ABCA2;TFAP2B;CREBBP;CBX5;ZBTB14;BCL11B;SMURF2;XRCC5;ZI
ERBB4;NSD1;PRDM16;NRIP1;E2F1;MAPK1;PCBD2;ZNF423;TRIM44;MAP3K2;SRPK2;TFAP2B;CREBBP;
1;ZNF148;ZNF423;SNX6;TFAP2B;CBX5;ZBTB14;SMURF2;TIPARP;XRCC5;ZBTB16;ESR1;HEYL;ID2;CAF
14;SMURF2;XRCC5;ZBTB16;HEYL;ID2;CAPRIN1;RLIM;PDCD4;ID4;CTNNB1
318;RLIM;PDCD4;ID4;CTNNB1

;SMURF2;USP9X;ZBTB16;CNBP;NFATC3;ESR1;GATAD2B;SMAD7;NFIA;MXD1;FRK;CC2D1B

RDBP;NFE2L1;SRSF9

E2K;RNF165

R1A

;MAPK1;APBB2;HIVEP2;ZNF148;MBNL3;SRSF10;PPARGC1B;TRIM44;SRPK2;TFAP2B;CREBBP;BCL11[illegible]

P5;PTK2B;MAPK1;MARK1;B3GALNT2;LYN;MAP3K2;SRPK2;CSNK1A1;ZBTB16;NEK7;ST8SIA3;CDC42BP

F1;FRK;FGFR1;BMPR1A

8;TARDBP;RAF1;SOS2;ARHGEF5;FGFR1

M16;NRIP1;E2F1;ZNF423;ZNF148;SNX6;IGBP1;TFAP2B;CREBBP;CBX6;CBX5;ZBTB14;SMURF2;USP9
CUT2;SIX1;RSF1;LITAF;CDC73;MED17;PHF8;CKS1B;MED14;MECOM;NKRF;SIN3A;TEAD1;UTP15;KLF
MK4;CTNNB1;NFE2L1
X2B;SMAD7;HNRNPK;SP1;AGO1;SP4;JMY;ID4;LHX4;MXD1;NFE2L1;CC2D1B;BMPR1A;BMPR2;ONECU
;RCAN1;FGFR1

P;SS18L2;FOXJ2;NFATC3;SMAD9;GTF2H1;INHBA;TBX5;ESR1;PHOX2B;HIPK2;SMAD7;HEYL;HNRNP
BTB16;SS18L2;NFATC3;INHBA;SMARCA1;GDF6;ESR1;SP1;SP4;ID2;JMY;ID4;NFE2L1;CREBZF;BTG1;S
;ZBTB16;CNBP;SS18L2;INHBA;DEK;TBX5;SMARCA1;GDF6;ESR1;HIPK2;MYO1C;SP1;ID2;FUBP1;CAM
RIN1;RLIM;PDCD4;ID4;CTNNB1;TARDBP

3;TIPARP;SORT1;CNBP;INHBA;ESR1;PHOX2B;PUM2;MAPK10;NFIA;SP1;ID2;CARM1;FUBP1;RLIM;CDK

PA;FBXO30;RAB11A;HIPK2;MAPK10;CAMK4;CDK1;ASB7;PTPN4;FRK;METAP2;FGFR1;BMPR1A

X;XRCC5;ZBTB16;CNBP;ESR1;GATAD2B;SMAD7;HEYL;NFIA;ID2;PDCD4;ID4;RLIM;CTNNB1;MXD1;FRK
F10;NCOA2;TFEB;PROX1;PAX2;KAT2B;CREB1;RBL1;MYOD1;TET3;MAPRE3;HDAC5;MYRF;PLAG1;LRP

IT2;SIX1;BMI1;LITAF;CDC73;MED17;SDR16C5;MED14;MECOM;NKRF;SIN3A;C1QBP;TRPS1;ZNF367;TE

K;NFIA;SP1;AGO1;ASXL3;CTNNB1;LHX4;SSBP2;NFE2L1;BMPR1A
SIX1;RSF1;LITAF;ELAVL2;MED17;PHF8;CKS1B;ING4;MED14;MECOM;NKRF;SIN3A;ZNF367;TEAD1;KLF1
K4;CTNNB1;NFE2L1

‹1;TARDBP;SSBP2

<;CC2D1B
'6;BCLAF1;RXRA;ERBB4;NSD1;NRIP1;E2F1;PCBD2;TRIM44;MAP3K2;ZNF462;CNBP;FOXJ2;GTF2H1;TE

EAD1;ZNF366;KLF10;NCOA2;TFEB;PROX1;PAX2;TGFBR3;KAT2B;CREB1;RBL1;MYOD1;TET3;CRK;KAN

10;NCOA2;TSFM;USP47;TFEB;PROX1;CBFA2T2;PAX2;CREB1;MYOD1;TERF2IP;MAPRE3;CRK;USP13;

3X5;HIPK2;HEYL;NFIA;ASXL3;CAMK4;CTNNB1;SSBP2

NK2;HDAC5;USP15;PLAG1;XIAP;LRP8;LRP6;RXRA;NSD1;NRIP1;E2F1;IGBP1;ZNF462;USP9X;CNBP;FO

HDAC5;MYRF;PRICKLE1;LRP6;PPP2CA;BCLAF1;ZNF629;ERBB4;NSD1;NRIP1;E2F1;APBB2;PCBD2;SF

)XJ2;ATP2B4;GTF2H1;DEK;TBX5;SOD2;GATAD2B;HIPK2;HEYL;NFIA;ASXL3;PDCD4;CTNNB1;SSBP2;FI

RSF10;TRIM44;MAP3K2;CNBP;FOXJ2;ATP2B4;DEK;TBX5;SOD2;HIPK2;HEYL;NFIA;CARM1;CAMK4;PD(

RK

CD4;RLIM;CTNNB1;SSBP2
