# Supplementary material for: Circulating miR-330-3p in Late Pregnancy is Associated with Pregnancy Outcomes Among Lean Women with GDM
Source: Sci Rep. 2020 Jan 22;10:908. doi: 10.1038/s41598-020-57838-6 (PMC6976655; doi:10.1038/s41598-020-57838-6)
Supplement: Supplementary file 1 — Supplementary data. [file 41598_2020_57838_MOESM1_ESM.zip › Supplimentary File_EnrichR_Analysis GO_Cellular_Component_2018.pdf]

Term	Overlap
cytoplasmic stress granule (GO:0010494)	10/42
bicellular tight junction (GO:0005923)	13/73
nuclear body (GO:0016604)	54/619
caveola (GO:0005901)	9/57
early endosome (GO:0005769)	22/223
nucleoplasm part (GO:0044451)	34/408
cytoplasmic vesicle (GO:0031410)	20/216
RISC complex (GO:0016442)	3/9
RNAi effector complex (GO:0031332)	3/9
chromatin (GO:0000785)	25/297
nuclear chromatin (GO:0000790)	22/254
N-terminal protein acetyltransferase complex (GO:0031414)	3/10
early endosome membrane (GO:0031901)	9/74
spindle microtubule (GO:0005876)	7/50
catenin complex (GO:0016342)	5/29
nuclear speck (GO:0016607)	24/297
perinuclear region of cytoplasm (GO:0048471)	29/379
telomerase holoenzyme complex (GO:0005697)	4/22
Cul4A-RING E3 ubiquitin ligase complex (GO:0031464)	3/13
septin cytoskeleton (GO:0032156)	3/14
septin ring (GO:0005940)	3/14
septin filament array (GO:0032160)	3/14
sex chromosome (GO:0000803)	2/6
integral component of plasma membrane (GO:0005887)	91/1464
RNA polymerase II transcription factor complex (GO:0090575)	13/148
pericentriolar material (GO:0000242)	3/16
nuclear chromosome part (GO:0044454)	28/393
MLL1/2 complex (GO:0044665)	4/28
MLL1 complex (GO:0071339)	4/28
dendrite (GO:0030425)	17/216
core mediator complex (GO:0070847)	2/8
unconventional myosin complex (GO:0016461)	2/8
SCF ubiquitin ligase complex (GO:0019005)	6/55
aggresome (GO:0016235)	5/42
GABA-A receptor complex (GO:1902711)	3/19
striated muscle thin filament (GO:0005865)	3/19
endoplasmic reticulum quality control compartment (GO:0044322)	2/9
junctional sarcoplasmic reticulum membrane (GO:0014701)	2/9
NMDA selective glutamate receptor complex (GO:0017146)	2/9
fibrillar center (GO:0001650)	11/132
intercalated disc (GO:0014704)	4/32
spliceosomal tri-snRNP complex (GO:0097526)	4/32
dendrite membrane (GO:0032590)	3/20
tertiary granule membrane (GO:0070821)	7/74
euchromatin (GO:0000791)	3/21
recycling endosome (GO:0055037)	10/120
clathrin-coated endocytic vesicle membrane (GO:0030669)	4/34
recycling endosome membrane (GO:0055038)	5/48
histone acetyltransferase complex (GO:0000123)	3/22
specific granule membrane (GO:0035579)	8/92
PML body (GO:0016605)	6/63
nuclear chromosome, telomeric region (GO:0000784)	9/108

actomyosin (GO:0042641)	5/49
ISWI-type complex (GO:0031010)	2/11
axonal growth cone (GO:0044295)	2/11
endocytic vesicle (GO:0030139)	9/109
polysome (GO:0005844)	6/64
Cul4-RING E3 ubiquitin ligase complex (GO:0080008)	4/37
cation channel complex (GO:0034703)	6/66
PRC1 complex (GO:0035102)	2/12
U4 snRNP (GO:0005687)	2/12
cytoskeleton (GO:0005856)	33/521
cytoplasmic microtubule (GO:0005881)	4/39
clathrin coat of endocytic vesicle (GO:0030128)	2/13
AP-2 adaptor complex (GO:0030122)	2/13
nuclear telomere cap complex (GO:0000783)	2/13
ruffle membrane (GO:0032587)	5/54
cullin-RING ubiquitin ligase complex (GO:0031461)	13/182
U4/U6 x U5 tri-snRNP complex (GO:0046540)	3/26
mitotic spindle (GO:0072686)	7/85
cytoplasmic vesicle membrane (GO:0030659)	5/55
Cul3-RING ubiquitin ligase complex (GO:0031463)	6/71
contractile actin filament bundle (GO:0097517)	4/41
stress fiber (GO:0001725)	4/41
contractile fiber (GO:0043292)	3/27
endoplasmic reticulum subcompartment (GO:0098827)	2/14
nucleolus (GO:0005730)	41/677
nuclear transcription factor complex (GO:0044798)	6/72
nucleolar part (GO:0044452)	11/154
myofibril (GO:0030016)	3/28
cytoplasmic ribonucleoprotein granule (GO:0036464)	12/171
actin cytoskeleton (GO:0015629)	19/295
chromosome, telomeric region (GO:0000781)	9/125
secretory vesicle (GO:0099503)	2/16
CHD-type complex (GO:0090545)	2/16
NuRD complex (GO:0016581)	2/16
exocytic vesicle (GO:0070382)	2/16
cell cortex part (GO:0044448)	4/45
centrosome (GO:0005813)	28/462
mRNA cleavage and polyadenylation specificity factor complex (GO:0005847)	2/17
endoplasmic reticulum tubular network (GO:0071782)	2/17
clathrin adaptor complex (GO:0030131)	2/17
focal adhesion (GO:0005925)	22/357
nuclear euchromatin (GO:0005719)	2/18
microtubule organizing center (GO:0005815)	30/508
small nuclear ribonucleoprotein complex (GO:0030532)	2/19
clathrin-coated endocytic vesicle (GO:0045334)	4/51
Cajal body (GO:0015030)	3/35
clathrin coat (GO:0030118)	1/6
gamma-secretase complex (GO:0070765)	1/6
spanning component of plasma membrane (GO:0044214)	1/6
spindle pole body (GO:0005816)	1/6
meiotic cohesin complex (GO:0030893)	1/6
Cul5-RING ubiquitin ligase complex (GO:0031466)	1/6
clathrin vesicle coat (GO:0030125)	1/6

U2 snRNP (GO:0005686)	2/21
pigment granule (GO:0048770)	2/21
melanosome (GO:0042470)	2/21
U1 snRNP (GO:0005685)	2/21
actin-based cell projection (GO:0098858)	5/71
NURF complex (GO:0016589)	1/7
early phagosome (GO:0032009)	1/7
kinetochore microtubule (GO:0005828)	1/7
oxoglutarate dehydrogenase complex (GO:0045252)	1/7
L-type voltage-gated calcium channel complex (GO:1990454)	1/7
Gemini of coiled bodies (GO:0097504)	1/7
micro-ribonucleoprotein complex (GO:0035068)	1/7
pi-body (GO:0071546)	1/7
U7 snRNP (GO:0005683)	1/7
intermediate filament cytoskeleton (GO:0045111)	5/72
transcriptionally active chromatin (GO:0035327)	2/22
actin filament (GO:0005884)	4/56
U5 snRNP (GO:0005682)	2/23
ionotropic glutamate receptor complex (GO:0008328)	3/40
Cdc73/Paf1 complex (GO:0016593)	1/8
commitment complex (GO:0000243)	1/8
apical dendrite (GO:0097440)	1/8
U6 snRNP (GO:0005688)	1/8
HAUS complex (GO:0070652)	1/8
HFE-transferrin receptor complex (GO:1990712)	1/8
shelterin complex (GO:0070187)	1/8
prespliceosome (GO:0071010)	2/24
cytoplasmic vesicle lumen (GO:0060205)	8/130
nuclear ubiquitin ligase complex (GO:0000152)	3/42
calcium channel complex (GO:0034704)	2/25
nuclear matrix (GO:0016363)	4/60
spliceosomal snRNP complex (GO:0097525)	4/60
RISC-loading complex (GO:0070578)	1/9
ATP-binding cassette (ABC) transporter complex (GO:0043190)	1/9
Swr1 complex (GO:0000812)	1/9
pseudopodium (GO:0031143)	1/9
outer dynein arm (GO:0036157)	1/9
filopodium (GO:0030175)	4/61
mitotic spindle pole (GO:0097431)	2/26
mitochondrial outer membrane (GO:0005741)	7/116
vacuole (GO:0005773)	4/63
primary lysosome (GO:0005766)	1/10
myosin filament (GO:0032982)	1/10
multimeric ribonuclease P complex (GO:0030681)	1/10
mitotic spindle midzone (GO:1990023)	1/10
clathrin-coated vesicle membrane (GO:0030665)	5/82
membrane raft (GO:0045121)	7/120
U12-type spliceosomal complex (GO:0005689)	2/28
mitochondrial matrix (GO:0005759)	17/309
Golgi membrane (GO:0000139)	24/443
autophagosome (GO:0005776)	4/65
phagocytic vesicle (GO:0045335)	5/84
intermediate filament (GO:0005882)	3/47

MLL3/4 complex (GO:0044666)	1/11
beta-catenin-TCF complex (GO:1990907)	1/11
nuclear inclusion body (GO:0042405)	1/11
spanning component of membrane (GO:0089717)	1/11
ribonuclease P complex (GO:0030677)	1/11
late endosome (GO:0005770)	8/141
microtubule cytoskeleton (GO:0015630)	21/389
axon (GO:0030424)	8/142
specific granule (GO:0042581)	9/161
ficolin-1-rich granule lumen (GO:1904813)	7/124
late endosome membrane (GO:0031902)	3/49
pericentric heterochromatin (GO:0005721)	1/12
methylosome (GO:0034709)	1/12
holo TFIIH complex (GO:0005675)	1/12
neurotransmitter receptor complex (GO:0098878)	1/12
P-body (GO:0000932)	4/69
kinesin complex (GO:0005871)	3/51
endoribonuclease complex (GO:1902555)	1/13
Sin3 complex (GO:0016580)	1/13
Set1C/COMPASS complex (GO:0048188)	1/13
spindle (GO:0005819)	10/187
DNA-directed RNA polymerase II, holoenzyme (GO:0016591)	5/91
mitochondrial large ribosomal subunit (GO:0005762)	3/53
nuclear chromosome (GO:0000228)	3/53
potassium channel complex (GO:0034705)	1/14
dystrophin-associated glycoprotein complex (GO:0016010)	1/14
microtubule (GO:0005874)	11/211
spliceosomal complex (GO:0005681)	6/115
keratin filament (GO:0045095)	1/15
synaptonemal structure (GO:0099086)	1/15
endolysosome membrane (GO:0036020)	1/15
mitochondrial respiratory chain complex III (GO:0005750)	1/15
Cul2-RING ubiquitin ligase complex (GO:0031462)	1/15
Ino80 complex (GO:0031011)	1/15
axonemal dynein complex (GO:0005858)	1/16
Sin3-type complex (GO:0070822)	1/16
P granule (GO:0043186)	1/16
nuclear periphery (GO:0034399)	4/79
Golgi subcompartment (GO:0098791)	24/480
platelet alpha granule membrane (GO:0031092)	1/17
polymeric cytoskeletal fiber (GO:0099513)	11/222
ribonucleoprotein granule (GO:0035770)	4/81
AMPA glutamate receptor complex (GO:0032281)	1/18
endocytic vesicle lumen (GO:0071682)	1/18
U2-type prespliceosome (GO:0071004)	1/19
nuclear heterochromatin (GO:0005720)	1/20
trans-Golgi network membrane (GO:0032588)	4/86
spindle pole (GO:0000922)	5/108
endolysosome (GO:0036019)	1/21
cytoplasmic dynein complex (GO:0005868)	1/21
mitochondrion (GO:0005739)	50/1027
INO80-type complex (GO:0097346)	1/22
pre-autophagosomal structure (GO:0000407)	1/24

coated vesicle (GO:0030135)	3/72
endosome lumen (GO:0031904)	1/25
histone methyltransferase complex (GO:0035097)	2/50
chromosome, centromeric region (GO:0000775)	2/50
lytic vacuole (GO:0000323)	8/184
ficolin-1-rich granule (GO:0101002)	8/185
Golgi cisterna (GO:0031985)	2/51
nuclear inner membrane (GO:0005637)	1/26
centriolar satellite (GO:0034451)	1/26
trans-Golgi network (GO:0005802)	8/186
tertiary granule (GO:0070820)	7/165
Golgi lumen (GO:0005796)	4/99
preribosome, large subunit precursor (GO:0030687)	1/27
cortical cytoskeleton (GO:0030863)	2/53
chromosomal region (GO:0098687)	3/77
ribosome (GO:0005840)	3/77
clathrin-coated vesicle (GO:0030136)	4/101
mitochondrial small ribosomal subunit (GO:0005763)	1/28
mitochondrial inner membrane (GO:0005743)	15/342
synaptonemal complex (GO:0000795)	1/29
Golgi stack (GO:0005795)	1/29
polysomal ribosome (GO:0042788)	1/29
brush border membrane (GO:0031526)	1/29
rough endoplasmic reticulum (GO:0005791)	1/29
Golgi cis cisterna (GO:0000137)	1/29
microtubule organizing center part (GO:0044450)	5/127
sarcoplasmic reticulum (GO:0016529)	1/30
multivesicular body (GO:0005771)	1/30
intrinsic component of mitochondrial inner membrane (GO:0031304)	1/30
U2-type catalytic step 2 spliceosome (GO:0071007)	1/30
spindle midzone (GO:0051233)	1/31
endosomal part (GO:0044440)	1/32
sarcoplasm (GO:0016528)	1/32
vesicle coat (GO:0030120)	1/32
U2-type spliceosomal complex (GO:0005684)	2/61
lysosomal lumen (GO:0043202)	3/87
precatalytic spliceosome (GO:0071011)	1/34
integral component of mitochondrial inner membrane (GO:0031305)	1/34
condensed nuclear chromosome (GO:0000794)	1/35
site of double-strand break (GO:0035861)	1/35
transcription factor TFIID complex (GO:0005669)	1/36
small-subunit processome (GO:0032040)	1/36
lipid droplet (GO:0005811)	2/65
site of DNA damage (GO:0090734)	1/38
phagocytic vesicle membrane (GO:0030670)	1/39
lysosome (GO:0005764)	17/423
integral component of Golgi membrane (GO:0030173)	1/41
peroxisomal membrane (GO:0005778)	1/41
condensed chromosome (GO:0000793)	1/41
chromosome (GO:0005694)	3/98
heterochromatin (GO:0000792)	1/42
large ribosomal subunit (GO:0015934)	2/73
microvillus (GO:0005902)	1/43

integral component of endoplasmic reticulum membrane (GO:0030176)	4/129
preribosome (GO:0030684)	2/76
lytic vacuole membrane (GO:0098852)	8/234
cis-Golgi network (GO:0005801)	1/48
vacuolar lumen (GO:0005775)	5/162
cytosolic small ribosomal subunit (GO:0022627)	1/50
lysosomal membrane (GO:0005765)	10/292
cortical actin cytoskeleton (GO:0030864)	1/53
small ribosomal subunit (GO:0015935)	1/54
ER to Golgi transport vesicle membrane (GO:0012507)	1/55
integral component of mitochondrial membrane (GO:0032592)	1/57
platelet alpha granule (GO:0031091)	2/91
azurophil granule lumen (GO:0035578)	2/91
azurophil granule membrane (GO:0035577)	1/59
peroxisome (GO:0005777)	2/93
microbody (GO:0042579)	2/93
azurophil granule (GO:0042582)	4/155
ficolin-1-rich granule membrane (GO:0101003)	1/62
specific granule lumen (GO:0035580)	1/63
secretory granule lumen (GO:0034774)	10/318
platelet alpha granule lumen (GO:0031093)	1/68
cytosolic large ribosomal subunit (GO:0022625)	1/70
peroxisomal part (GO:0044439)	1/76
COPII-coated ER to Golgi transport vesicle (GO:0030134)	1/76
cytosolic ribosome (GO:0022626)	2/125
centriole (GO:0005814)	1/96
endoplasmic reticulum lumen (GO:0005788)	6/271
cytosolic part (GO:0044445)	2/160

P.value		Adjusted.P.value	Old.P.value	Old.Adjusted
	4,15801E-05	0,018544707	0	0
	8,21235E-05	0,01831354	0	0
	0,000116895	0,017378458	0	0
	0,002412653	0,269010844	0	0
	0,002772087	0,247270159	0	0
	0,004191642	0,311578744	0	0
	0,008403976	0,535453326	0	0
	0,009102597	0,507469807	0	0
	0,009102597	0,451084273	0	0
	0,011373954	0,507278337	0	0
	0,012436623	0,504248523	0	0
	0,01250999	0,464954619	0	0
	0,013643204	0,468066859	0	0
	0,013712671	0,436846506	0	0
	0,01531179	0,455270558	0	0
	0,020267414	0,564954166	0	0
	0,022594363	0,59276975	0	0
	0,024509309	0,607286222	0	0
	0,026564546	0,623567768	0	0
	0,03254043	0,725651587	0	0
	0,03254043	0,691096749	0	0
	0,03254043	0,659683261	0	0
	0,034729741	0,673454976	0	0
	0,035773636	0,664793401	0	0
	0,042752364	0,76270217	0	0
	0,046390758	0,795779926	0	0
	0,05322037	0,879121669	0	0
	0,053862291	0,857949344	0	0
	0,053862291	0,828364884	0	0
	0,055468982	0,824638872	0	0
	0,060539704	0,870990584	0	0
	0,060539704	0,843772128	0	0
	0,06305942	0,852257615	0	0
	0,063661977	0,835095347	0	0
	0,071669081	0,913268864	0	0
	0,071669081	0,887900285	0	0
	0,07523604	0,906899288	0	0
	0,07523604	0,883033518	0	0
	0,07523604	0,860391633	0	0
	0,079476631	0,886164438	0	0
	0,080655885	0,877378652	0	0
	0,080655885	0,856488684	0	0
	0,081209097	0,842308312	0	0
	0,086747796	0,8793072	0	0
	0,0912632	0,904519717	0	0
	0,091513274	0,887280872	0	0
	0,096102152	0,911948084	0	0
	0,10011571	0,930241808	0	0
	0,101803296	0,926617753	0	0
	0,102225566	0,91185205	0	0
	0,105462757	0,922282148	0	0
	0,105672448	0,906344457	0	0

0,107028285	0,900653113	0	0
0,107443713	0,887405483	0	0
0,107443713	0,871270838	0	0
0,110231692	0,877916692	0	0
0,111599028	0,873213444	0	0
0,121626858	0,935268602	0	0
0,124400603	0,940384221	0	0
0,124685754	0,926830768	0	0
0,124685754	0,911636821	0	0
0,131078967	0,942922894	0	0
0,140080408	0,99168035	0	0
0,142525984	0,99322795	0	0
0,142525984	0,97794752	0	0
0,142525984	0,963130133	0	0
0,144876791	0,964403713	0	0
0,14780046	0,969397136	0	0
0,148230982	0,958130698	0	0
0,148850429	0,948389875	0	0
0,15305853	0,961466262	0	0
0,159315798	0,986872862	0	0
0,159564969	0,974876387	0	0
0,159564969	0,961702382	0	0
0,160752832	0,955943509	0	0
0,160856918	0,943976127	0	0
0,162173757	0,939344101	0	0
0,166763522	0,953545266	0	0
0,172632767	0,974610305	0	0
0,173579701	0,967706834	0	0
0,173890527	0,957471298	0	0
0,189499563	1	0	0
0,197023122	1	0	0
0,198607672	1	0	0
0,198607672	1	0	0
0,198607672	1	0	0
0,198607672	1	0	0
0,201187717	1	0	0
0,214392348	1	0	0
0,217857207	1	0	0
0,217857207	1	0	0
0,217857207	1	0	0
0,223857998	1	0	0
0,237255632	1	0	0
0,247024006	1	0	0
0,256736405	1	0	0
0,268549185	1	0	0
0,269461014	1	0	0
0,272334438	1	0	0
0,272334438	1	0	0
0,272334438	1	0	0
0,272334438	1	0	0
0,272334438	1	0	0
0,272334438	1	0	0
0,272334438	1	0	0

0,295710622	1	0	0
0,295710622	1	0	0
0,295710622	1	0	0
0,295710622	1	0	0
0,303839462	1	0	0
0,309893194	1	0	0
0,309893194	1	0	0
0,309893194	1	0	0
0,309893194	1	0	0
0,309893194	1	0	0
0,309893194	1	0	0
0,309893194	1	0	0
0,309893194	1	0	0
0,309893194	1	0	0
0,314069009	1	0	0
0,315101353	1	0	0
0,327280741	1	0	0
0,334368124	1	0	0
0,341046404	1	0	0
0,345515118	1	0	0
0,345515118	1	0	0
0,345515118	1	0	0
0,345515118	1	0	0
0,345515118	1	0	0
0,345515118	1	0	0
0,345515118	1	0	0
0,353472123	1	0	0
0,356498409	1	0	0
0,369659561	1	0	0
0,372378865	1	0	0
0,374805161	1	0	0
0,374805161	1	0	0
0,379299997	1	0	0
0,379299997	1	0	0
0,379299997	1	0	0
0,379299997	1	0	0
0,379299997	1	0	0
0,386664701	1	0	0
0,39105785	1	0	0
0,391251074	1	0	0
0,410285043	1	0	0
0,411342481	1	0	0
0,411342481	1	0	0
0,411342481	1	0	0
0,411342481	1	0	0
0,417467868	1	0	0
0,425362518	1	0	0
0,42762859	1	0	0
0,428260852	1	0	0
0,432624992	1	0	0
0,433710529	1	0	0
0,438007127	1	0	0
0,439891843	1	0	0

0,441732347	1	0	0
0,441732347	1	0	0
0,441732347	1	0	0
0,441732347	1	0	0
0,441732347	1	0	0
0,443460326	1	0	0
0,447504801	1	0	0
0,451331657	1	0	0
0,451566513	1	0	0
0,459251941	1	0	0
0,467165176	1	0	0
0,470554753	1	0	0
0,470554753	1	0	0
0,470554753	1	0	0
0,470554753	1	0	0
0,479703714	1	0	0
0,493830654	1	0	0
0,497890471	1	0	0
0,497890471	1	0	0
0,497890471	1	0	0
0,500072467	1	0	0
0,508266738	1	0	0
0,5198147	1	0	0
0,5198147	1	0	0
0,523816115	1	0	0
0,523816115	1	0	0
0,530016495	1	0	0
0,548112302	1	0	0
0,548404355	1	0	0
0,548404355	1	0	0
0,548404355	1	0	0
0,548404355	1	0	0
0,548404355	1	0	0
0,548404355	1	0	0
0,571724118	1	0	0
0,571724118	1	0	0
0,571724118	1	0	0
0,587183851	1	0	0
0,593580251	1	0	0
0,593840785	1	0	0
0,598507831	1	0	0
0,607076533	1	0	0
0,614816365	1	0	0
0,614816365	1	0	0
0,63470968	1	0	0
0,653576518	1	0	0
0,654159206	1	0	0
0,660728995	1	0	0
0,671469795	1	0	0
0,671469795	1	0	0
0,688379321	1	0	0
0,688439703	1	0	0
0,719797384	1	0	0

0,725260757	1	0	0
0,734273137	1	0	0
0,737253058	1	0	0
0,737253058	1	0	0
0,738812674	1	0	0
0,744195448	1	0	0
0,747188653	1	0	0
0,74800173	1	0	0
0,74800173	1	0	0
0,749500794	1	0	0
0,753472346	1	0	0
0,758037257	1	0	0
0,761021692	1	0	0
0,766093226	1	0	0
0,766227792	1	0	0
0,766227792	1	0	0
0,771651031	1	0	0
0,773369566	1	0	0
0,776952424	1	0	0
0,785080013	1	0	0
0,785080013	1	0	0
0,785080013	1	0	0
0,785080013	1	0	0
0,785080013	1	0	0
0,785080013	1	0	0
0,790475465	1	0	0
0,796185909	1	0	0
0,796185909	1	0	0
0,796185909	1	0	0
0,796185909	1	0	0
0,806718435	1	0	0
0,816707165	1	0	0
0,816707165	1	0	0
0,816707165	1	0	0
0,829882961	1	0	0
0,833197469	1	0	0
0,835163993	1	0	0
0,835163993	1	0	0
0,84368393	1	0	0
0,84368393	1	0	0
0,851763893	1	0	0
0,851763893	1	0	0
0,855503801	1	0	0
0,866693525	1	0	0
0,873585143	1	0	0
0,884831035	1	0	0
0,886318873	1	0	0
0,886318873	1	0	0
0,886318873	1	0	0
0,887135472	1	0	0
0,892196776	1	0	0
0,896460449	1	0	0
0,897771034	1	0	0

0,905210594	1	0	0
0,908816996	1	0	0
0,920503574	1	0	0
0,921608729	1	0	0
0,925371797	1	0	0
0,929508438	1	0	0
0,938598118	1	0	0
0,939891944	1	0	0
0,943001654	1	0	0
0,945950624	1	0	0
0,951399117	1	0	0
0,952388524	1	0	0
0,952388524	1	0	0
0,956298824	1	0	0
0,956409321	1	0	0
0,956409321	1	0	0
0,961762637	1	0	0
0,962738684	1	0	0
0,964667232	1	0	0
0,969022954	1	0	0
0,972913143	1	0	0
0,975645263	1	0	0
0,982297573	1	0	0
0,982297573	1	0	0
0,989779057	1	0	0
0,993891226	1	0	0
0,99544316	1	0	0
0,998029906	1	0	0

Odds.Ratio	Combined.Score	Genes
4,6142488	46,5480335	TIAL1;DYRK3;STAU1;NUFIP2;CAPRIN1;IGF2BP1;RC3H1;EIF
3,451205267	32,46647627	CXADR;FRMPD2;CLDN1;AMOT;MTDH;RAP2C;CYTH3;OCLN;
1,690648833	15,30752432	HNRNPU;ZBTB20;PPWD1;BMI1;DCAF7;SART3;MECOM;PAP
3,05997552	18,44255869	BMPR2;NOS1AP;ATP2B4;MAPK1;SCN5A;LRP8;SELE;LRP6;E
1,911912956	11,25763948	RABGAP1L;MAP2K1;FZD5;SORT1;LNPEP;NSG1;SORL1;LRF
1,61498708	8,841509471	AMER1;NUFIP2;PHF20;TNKS;ZBTB20;PPWD1;DTX1;BMI1;HI
1,794430089	8,575671756	ABCA1;ABCA2;RAB3C;RALA;SH3KBP1;SORT1;RNASE6;RH(
6,45994832	30,3565599	AGO1;EIF4E;DCP2
6,45994832	30,3565599	AGO1;EIF4E;DCP2
1,631300081	7,302399476	AFF4;SDR16C5;RXRA;SIN3A;TRPS1;NRIP1;E2F1;MCMBP;M
1,678569249	7,364067451	MEF2A;CREBBP;DFFA;CBX5;RUNX3;ESR1;GATAD2B;PHOX:
5,813953488	25,4722545	NAA30;NAA35;NAA38
2,357008171	10,12220394	SNX1;FZD5;NSG1;LITAF;SNX8;WLS;KCNH1;LRP6;SNX6
2,713178295	11,63800197	TBL1XR1;CUL3;ARL3;CDK1;HNRNPU;CALM1;RAB11A
3,34135258	13,963954	CDH6;CDH2;APC;CTNNB1;SMAD7
1,566048078	6,105615698	FNBP4;FYTTD1;HDAC5;SMURF2;CSNK1A1;YTHDC1;PLAG1
1,482890512	5,620236364	RAB3C;SET;CALCOCO2;PTPRM;MTDH;CTIF;CYB5R4;RAP1/
3,523608175	13,0680136	HNRNPU;SNRPD3;HNRNPC;SMG7
4,472271914	16,22619769	CRBN;DTL;RBX1
4,15282392	14,22455135	SEPT10;SEPT11;SEPT12
4,15282392	14,22455135	SEPT10;SEPT11;SEPT12
4,15282392	14,22455135	SEPT10;SEPT11;SEPT12
6,45994832	21,70645267	PCGF3;HNRNPU
1,204621511	4,012045046	CHRM3;GABRB1;NPFFR1;BMPR2;TENM3;KCNC2;PTPRM;CI
1,702283679	5,366161258	HNRNPU;GTF2H1;RORB;HIF1A;GTF2H5;MED17;HIPK2;MED
3,63372093	11,15790341	TNKS2;TNKS;HOOK3
1,380752313	4,050180171	TNKS;CDC73;PURA;RXRA;TNKS2;TRPS1;RUVBL1;NRIP1;E2
2,76854928	8,087831292	KANSL1;PHF20;RBBP5;RUVBL1
2,76854928	8,087831292	KANSL1;PHF20;RBBP5;RUVBL1
1,525265576	4,410963243	GABRA1;MME;STAU1;KCNC2;GABRA3;DYRK1A;NSG1;PPP1
4,84496124	13,58747994	MED14;MED17
4,84496124	13,58747994	MYO1C;DYNLL2
2,114164905	5,842870663	FBXW4;USP47;BTRC;BTBD9;CKS1B;RBX1
2,3071244	6,354207732	CCZ1B;ZBTB14;EDEM1;SFMBT2;PRDM16
3,05997552	8,065164774	GABRA1;GABRB1;GABRA3
3,05997552	8,065164774	TMOD3;TMOD2;LMOD3
4,306632214	11,14179549	EDEM3;EDEM1
4,306632214	11,14179549	JPH3;JPH1
4,306632214	11,14179549	PTK2B;GRIN2B
1,61498708	4,089619264	UTP15;RPS6KA6;CAMK4;FOXJ2;SPATA2;PAX5;IP6K1;AFF4;F
2,42248062	6,098748804	GJC1;TMEM65;CDH2;SCN5A
2,42248062	6,098748804	SART3;NAA38;SNRPD3;LSM5
2,906976744	7,298627913	GABRA1;KCNC2;GABRA3
1,833228577	4,481786064	RAP2C;MGAM;RAP2B;SNAP23;ANO6;ATP11A;CD47
2,76854928	6,627928123	CREB1;DNMT3A;CTNNB1
1,61498708	3,861872171	RAP2C;RAP2A;FCHSD2;RAP2B;ATP11A;DENND6A;NSG1;SC
2,27998176	5,340500606	FZD5;TYRP1;SH3GL2;AP2M1
2,01873385	4,645971938	RAP2C;RAP2A;RAP2B;NSG1;RAB11A
2,642706131	6,037824525	ING4;KANSL1;PHF20
1,68520391	3,843231336	PGRMC1;RAP2B;RAP1A;SNAP23;MLEC;ANO6;ATP11A;CD47
1,84569952	4,15171171	CBX5;ZBTB16;WDFY3;SENP2;PIAS1;HIPK2
1,61498708	3,629539863	PURA;CBX5;TNKS2;TNKS;XRCC5;CDK1;NABP1;TERF2IP;CI

1,9775352	4,419123035	SEPT11;SEPT12;TPM3;CDC42BPA;MYH10
3,523608175	7,860423421	RSF1;SMARCA1
3,523608175	7,860423421	GPM6A;L1CAM
1,600170685	3,528649726	ABCA1;RALA;HSPH1;SH3KBP1;SRGAP2;RAPGEF6;AP2M1;F
1,816860465	3,984089648	NAA30;NUFIP2;AGO1;NAA35;NAA38;MYH10
2,095118374	4,413990065	CRBN;DTL;DCAF7;RBX1
1,761804087	3,672037086	KCNIP2;SESTD1;PTK2B;SHISA9;CALM1;GRIN2B
3,22997416	6,724672735	PHC2;BMI1
3,22997416	6,724672735	NAA38;SNRPD3
1,227514172	2,494253969	KANK2;ONECUT2;ROCK1;RHOBTB3;SMG7;PPP1R9A;LMOD
1,987676406	3,906854855	SERP1;APC;SNPH;ARL3
2,981514609	5,80867905	SGIP1;AP2M1
2,981514609	5,80867905	SGIP1;AP2M1
2,981514609	5,80867905	XRCC5;TERF2IP
1,794430089	3,466608554	EPS8;APC;PSD4;PSD3;MTSS1L
1,38427464	2,646583827	FBXW4;USP47;CUL3;KLHL32;KLHL23;BTBD9;BACH1;DCAF7
2,236135957	4,268746712	SART3;NAA38;LSM5
1,595987232	3,040057723	TNKS;HECW2;CDK1;HNRNPU;HAUS3;MAPK1;SMC1A
1,761804087	3,306791541	RALA;ZNRF2;KIF1B;LNPEP;RAB11A
1,637733377	3,008298221	CUL3;KLHL7;KLHL32;KLHL23;BACH1;RBX1
1,890716582	3,470039912	SEPT11;SEPT12;TPM3;MYH10
1,890716582	3,470039912	SEPT11;SEPT12;TPM3;MYH10
2,153316107	3,936019159	TMOD3;TMOD2;LMOD3
2,76854928	5,058804027	REEP1;ATL3
1,173668602	2,135005229	RBM28;SF3B3;AGAP2;SIX1;PPM1E;PHF8;RBM3;RPS6KA6;N
1,61498708	2,892730146	RXRA;NFATC3;HNRNPU;RORB;HIF1A;HIPK2
1,38427464	2,431601156	UTP15;RPS6KA6;CAMK4;FOXJ2;SPATA2;PAX5;IP6K1;AFF4;F
2,07641196	3,636043216	TMOD3;TMOD2;LMOD3
1,35998912	2,379068859	TIAL1;DYRK3;STAU1;NUFIP2;CAPRIN1;IGF2BP1;HNRNPU;R
1,248193404	2,076205664	NDC1;ONECUT2;DCTN2;TPM3;TMOD3;TMOD2;PARVA;ACTN
1,395348837	2,266652355	PURA;CBX5;TNKS2;TNKS;XRCC5;CDK1;NABP1;TERF2IP;CD
2,42248062	3,915755568	DPYSL3;SYTL4
2,42248062	3,915755568	GATAD2B;APPL1
2,42248062	3,915755568	GATAD2B;APPL1
2,42248062	3,915755568	DPYSL3;SYTL4
1,722652885	2,762303	SEPT10;SEPT11;SEPT12;SELE
1,174536058	1,808723917	DCTN2;TNKS;ARL3;HNRNPU;PHAX;SLC1A4;RIC8B;TNKS2;E
2,27998176	3,474499418	CPSF7;CSNK1A1
2,27998176	3,474499418	REEP1;ATL3
2,27998176	3,474499418	SGIP1;AP2M1
1,19427616	1,787524918	NCKAP1;MAP2K1;RALA;MME;SNAP23;PARVA;ACTN4;L1CAM
2,153316107	3,097797377	CREB1;CTNNB1
1,144479033	1,600290419	DCTN2;ARL3;HNRNPU;PHAX;SLC1A4;RIC8B;E2F1;CEP170;I
2,03998368	2,773776792	SF3B3;SNRPD3
1,51998784	1,998360232	FZD5;TYRP1;SH3GL2;AP2M1
1,661129568	2,178291625	NPAT;SART3;ANGEL2
3,22997416	4,201306255	NECAP2
3,22997416	4,201306255	APH1B
3,22997416	4,201306255	BMPR2
3,22997416	4,201306255	NDC1
3,22997416	4,201306255	SMC1A
3,22997416	4,201306255	RBX1
3,22997416	4,201306255	NECAP2

1,84569952	2,248752181	NAA38;SNRPD3
1,84569952	2,248752181	TMEM33;TYRP1
1,84569952	2,248752181	TMEM33;TYRP1
1,84569952	2,248752181	NAA38;SNRPD3
1,364777814	1,62579949	GPM6A;CXADR;MYO1C;DAG1;NPTXR
2,76854928	3,243431827	SMARCA1
2,76854928	3,243431827	SYT7
2,76854928	3,243431827	RAB11A
2,76854928	3,243431827	DLST
2,76854928	3,243431827	NOS1AP
2,76854928	3,243431827	NPAT
2,76854928	3,243431827	AGO1
2,76854928	3,243431827	TDRKH
2,76854928	3,243431827	SNRPD3
1,345822567	1,558654372	ING4;NCKIPSD;SESTD1;SLC1A4;SMG7
1,761804087	2,034638719	AFF4;ESR1
1,38427464	1,546147481	CAPZB;TPM3;RCSD1;AMOT
1,68520391	1,846162327	NAA38;SNRPD3
1,453488372	1,563570827	PTK2B;SHISA9;GRIN2B
2,42248062	2,574415889	CDC73
2,42248062	2,574415889	SNRPD3
2,42248062	2,574415889	PTK2B
2,42248062	2,574415889	LSM5
2,42248062	2,574415889	HAUS3
2,42248062	2,574415889	BMPR1A
2,42248062	2,574415889	TERF2IP
1,61498708	1,679506874	NAA38;SNRPD3
1,192605844	1,23008408	ALAD;PSMD11;HSPH1;ROCK1;CRISPLD2;XRCC5;SCG3;PAF
1,38427464	1,377592472	PHC2;BMI1;RBX1
1,550387597	1,531540293	SESTD1;CALM1
1,291989664	1,26789271	DNMT3A;HNRNPU;SMC1A;DCAF7
1,291989664	1,26789271	SART3;NAA38;SNRPD3;LSM5
2,153316107	2,087484578	AGO1
2,153316107	2,087484578	ABCA2
2,153316107	2,087484578	RUVBL1
2,153316107	2,087484578	ACTN4
2,153316107	2,087484578	DNAL1
1,270809506	1,207519848	GPM6A;CXADR;DAG1;NPTXR
1,490757305	1,399671698	TNKS;SMC1A
1,169473403	1,097440616	GPAM;VPS13C;PGAM5;MIEF1;RAF1;HK2;RAB11FIP5
1,230466347	1,096226325	CALCOCO2;VTI1A;TBC1D25;WDFY3
1,937984496	1,721568072	SNAP23
1,937984496	1,721568072	MYH10
1,937984496	1,721568072	RPP14
1,937984496	1,721568072	HNRNPU
1,181697863	1,03226945	NECAP2;FZD5;TYRP1;SH3GL2;AP2M1
1,130490956	0,966358918	ABCA1;LYN;CXADR;RAP2B;MYO1C;GPC1;SELE
1,38427464	1,17594164	SF3B3;SNRPD3
1,066205063	0,904166204	TSFM;ALAS2;NSUN3;DLST;ETFA;PDHB;PPM1K;SOD2;GLS;A
1,049923881	0,879714415	HS3ST3B1;GALNT7;RAB2B;CHST7;ST6GAL2;EDEM1;B3GAT
1,192605844	0,996276626	CALCOCO2;VTI1A;TBC1D25;WDFY3
1,1535622	0,95228878	ABCA1;SNAP23;SRGAP2;RAB11A;SYT7
1,237011381	1,015866394	NCKIPSD;CSNK1A1;SLC1A4

1,761804087	1,43948402	RBBP5
1,761804087	1,43948402	CTNNB1
1,761804087	1,43948402	PABPN1
1,761804087	1,43948402	BMPR2
1,761804087	1,43948402	RPP14
1,099565672	0,894108459	MAP2K1;AP5M1;RAP1A;SLC30A4;VTI1A;MAPK1;NSG1;RAB1
1,04621271	0,841226175	ABCA2;SEPT10;SEPT11;SEPT12;HAUS3;TUBD1;SLC1A4;SY
1,091822251	0,868602281	MME;KCNC2;SEMA3A;DYRK1A;TMEM108;KIF1B;L1CAM;RAI
1,083345371	0,861294888	PGRMC1;RAP1A;RAP2B;SNAP23;MLEC;ANO6;ATP11A;CD47
1,094023506	0,851321313	KCMF1;ALAD;PSMD11;CRISPLD2;MAPK1;PDAP1;PAFAH1B2
1,18652112	0,903028463	VTI1A;CDIP1;LITAF
1,61498708	1,217446632	CBX5
1,61498708	1,217446632	SNRPD3
1,61498708	1,217446632	GTF2H1
1,61498708	1,217446632	SHISA9
1,123469273	0,825285506	CAPRIN1;RC3H1;EIF4E;DCP2
1,13999088	0,804334959	KIF5C;KIF26B;KIF1B
1,490757305	1,03961712	AGO1
1,490757305	1,03961712	SIN3A
1,490757305	1,03961712	RBBP5
1,036355346	0,718196593	SEPT12;TBL1XR1;HECW2;ARL3;CDK1;HNRNPU;HAUS3;MAI
1,064826646	0,720620255	MED14;GTF2H1;CDC73;GTF2H5;MED17
1,096972356	0,71773023	MRPL42;NSUN3;MRPL27
1,096972356	0,71773023	HNRNPU;TERF2IP;SMC1A
1,38427464	0,895092168	KCNIP2
1,38427464	0,895092168	DAG1
1,010323671	0,641401104	SERP1;APC;KIF5C;TBL1XR1;SNPH;KIF26B;ARL3;CDK1;TUB
1,011122346	0,607962672	DHX40;SF3B3;TRA2B;HNRNPR;SNRPD3;HNRNPC
1,291989664	0,776152959	CSNK1A1
1,291989664	0,776152959	FKBP6
1,291989664	0,776152959	AP2M1
1,291989664	0,776152959	UQCR10
1,291989664	0,776152959	RBX1
1,291989664	0,776152959	RUVBL1
1,21124031	0,6772029	DNAL1
1,21124031	0,6772029	SIN3A
1,21124031	0,6772029	TDRKH
0,981257973	0,522438724	DNMT3A;HNRNPU;SMC1A;DCAF7
0,968992248	0,505409746	HS3ST3B1;GALNT7;RAB2B;SCOC;CHST7;ST6GAL2;EDEM1;
1,13999088	0,594099447	SYTL4
0,960262588	0,492917834	NCKIPSD;CAPZB;APC;KIF5C;TPM3;KIF26B;TUBD1;KIF1B;SL
0,957029381	0,477653758	STAU1;HNRNPU;EIF4E;DCP2
1,076658053	0,523720552	SHISA9
1,076658053	0,523720552	HSPH1
1,01999184	0,463675624	NAA38
0,968992248	0,412108201	CBX5
0,901388138	0,382553202	RHOBTB3;VTI1A;VPS54;NSG1
0,897215045	0,371816246	TNKS;CUL3;CALM1;SMC1A;RAB11A
0,92284976	0,367558366	AP2M1
0,92284976	0,367558366	DYNLL2
0,943517281	0,352323745	TDRKH;HRK;CREBZF;ALAS2;COX7B;KIAA0141;ETFA;GLS;M
0,880902044	0,328864994	RUVBL1
0,80749354	0,265492182	TRAPPC8

0,80749354	0,259386324	SGIP1;SORT1;VTI1A
0,775193798	0,239437363	LNPEP
0,775193798	0,23629774	CBX5;RBBP5
0,775193798	0,23629774	CBX5;SMC1A
0,842601955	0,255064776	TIAL1;ABCA2;SNX1;AP5M1;LAPTM5;SYT7;ITM2C;TPCN1
0,83804735	0,247602413	KCMF1;ALAD;MGAM;PSMD11;CRISPLD2;MAPK1;PDAP1;PAI
0,75999392	0,221490787	NECAB3;SORL1
0,745378652	0,216420683	DPY19L2
0,745378652	0,216420683	PAX2
0,833541719	0,240350006	SCOC;RHOBTB3;VTI1A;VPS54;NSG1;SORL1;RAB11A;WLS
0,822175241	0,232727359	RAP2C;MGAM;RAP2B;SNAP23;ANO6;ATP11A;CD47
0,783024039	0,216915467	GPC1;OMD;DAG1;GPC5
0,717772036	0,196018818	WDR37
0,731314904	0,194859889	CDH2;SELE
0,755058895	0,201053892	TNKS;TERF2IP;SMC1A
0,755058895	0,201053892	SERP1;NUFIP2;LTN1
0,767518612	0,198958372	SGIP1;SORT1;VTI1A;AP2M1
0,69213732	0,177878081	MRPL42
0,8499932	0,21451802	COX7B;ALAS2;TIMMDC1;MRPL27;UQCR10;SOD2;LETM1;MI
0,668270516	0,161701175	FKBP6
0,668270516	0,161701175	SORL1
0,668270516	0,161701175	NUFIP2
0,668270516	0,161701175	GNA13
0,668270516	0,161701175	SEC62
0,668270516	0,161701175	NECAB3
0,762986022	0,179393778	TNKS2;TNKS;CEP170;HOOK3;PAX2
0,645994832	0,1472368	JPH1
0,645994832	0,1472368	RAB11A
0,645994832	0,1472368	COA1
0,645994832	0,1472368	SNRPD3
0,625156289	0,134271427	HNRNPU
0,605620155	0,122622744	SNX6
0,605620155	0,122622744	JPH1
0,605620155	0,122622744	NECAP2
0,635404753	0,118484305	NAA38;SNRPD3
0,668270516	0,121949082	GPC1;OMD;GPC5
0,56999544	0,102671668	SNRPD3
0,56999544	0,102671668	COA1
0,553709856	0,094118131	SMC1A
0,553709856	0,094118131	RFWD3
0,538329027	0,086372691	GTF2H5
0,538329027	0,086372691	DIEXF
0,596302922	0,093061862	SDR16C5;SET
0,50999592	0,072965042	RFWD3
0,496919102	0,067158458	SNAP23
0,778859017	0,095300078	ABCA2;OMD;LAPTM5;LNPEP;ATP11A;LITAF;SYT7;TPCN1;TI
0,472679145	0,057042206	SLC39A13
0,472679145	0,057042206	ACBD5
0,472679145	0,057042206	SMC1A
0,59326056	0,071047448	HNRNPU;TERF2IP;SMC1A
0,46142488	0,052634076	CBX5
0,530954656	0,05803393	RBM3;COA1
0,450694069	0,048602946	MYO1C

0,600925425	0,059844758	TMEM33;EDEM1;BFAR;ZMPSTE24
0,50999592	0,04876149	WDR37;DIEXF
0,662558802	0,054882658	ABCA2;CLCN5;LNPEP;ATP11A;LITAF;SLC29A3;AP2M1;TPCN
0,40374677	0,032959673	HOOK3
0,598143363	0,046391807	GPC1;OMD;MAPK1;GPC5;FRK
0,387596899	0,028333098	MCTS1
0,663693321	0,042056839	ABCA2;CLCN5;VAPA;CDIP1;LNPEP;ATP11A;LITAF;SLC29A3;
0,365657452	0,022667239	CDH2
0,358886018	0,021062031	MCTS1
0,352360817	0,019578896	VTI1A
0,33999728	0,016939216	COA1
0,425930658	0,020777841	ACTN4;SYTL4
0,425930658	0,020777841	MAPK1;FRK
0,328471948	0,014677716	VAPA
0,416770859	0,018575185	ACBD5;SYT7
0,416770859	0,018575185	ACBD5;SYT7
0,500125031	0,019498674	VAPA;SNAP23;MAPK1;FRK
0,312578145	0,011869611	MGAM
0,307616587	0,011065607	FRK
0,609429087	0,019176893	ALAD;PSMD11;ROCK1;CRISPLD2;XRCC5;MAPK1;SCG3;ACT
0,28499772	0,007826171	ACTN4
0,276854928	0,006826196	COA1
0,25499796	0,004554516	ACBD5
0,25499796	0,004554516	VTI1A
0,310077519	0,003185593	MCTS1;COA1
0,201873385	0,001236981	CEP170
0,429074058	0,00195969	ADAMTS5;CDH2;DAG1;SCG3;COL19A1;SUMF1
0,242248062	0,000477722	MCTS1;COA1

4E;GIGYF2;PUM2
;RAP2B;APC;VAPA;CLDN18;STRN
'OLG;UBXN7;SNRPD3;SCN1A;FNBP4;NCOA2;SFMBT2;ANGEL2;DYRK1A;ACTN4;SENP2;PIAS1;CDC34
3MPR1A
'6;NEURL1B;SNX1;ITCH;RAP1A;TMEM108;MAPK1;WDFY2;SNX8;SH3GL2;WLS;RAB11FIP5;SNX6;APPL
IF1A;ZBTB4;DCAF7;MTDH;MED17;ING4;MED14;RBBP5;PAPOLG;UBXN7;SNRPD3;SCN1A;NCOA2;CRE
OBTB3;TRAPPC8;RND3;RAB11A;TRAK2;AMOT;LRP6;ADD2;OCLN;SPRED1;KIF1B;RAPGEF6;WLS

EF2A;CREBBP;DFFA;ESCO1;DNMT3A;RUNX3;ESR1;GATAD2B;PHOX2B;HNRNPK;MAF;RBL1;SP1;MY(
2B;HNRNPK;RXRA;CREB1;SP1;TRPS1;MYOD1;ASXL3;RUVBL1;NRIP1;E2F1;MCMBP;CTNNB1;HNRNP

;SFMBT2;ZBTB16;SRSF1;YLPM1;DYRK1A;HNRNPU;HIF1A;NXF1;BCLAF1;CDC34;MECOM;TRIP12;RAF
A;TNKS2;VTI1A;VPS54;PTK2B;EIF4E;ABCA1;LYN;SEPT12;SORT1;SLC39A13;ACTN4;S100B;SELE;GTPI

LDN1;GRM5;SLC22A17;GNRHR;EMB;IL6R;PCDHAC2;KCNH1;IL13RA1;PCDHAC1;HS3ST3B1;ENTPD1;(
)14;CREB1;RXRA;E2F1;CTNNB1;TEAD1

2F1;MCMBP;MEF2A;CREBBP;DFFA;CBX5;XRCC5;RUNX3;ESR1;GATAD2B;PHOX2B;HNRNPK;SP1;ASX

IR9A;TRAK2;RBM3;IL1RAPL1;PTK2B;STRN;KIF1B;BSN;NPTXR;KPNA1

PAFAH1B2;MTDH;SMAD7

ORL1;RAB11A;RAB11FIP5

7

OC73

[illegible]RAB11A;AMOT

[illegible]3;ING4;PPP2CA;CAPZB;ATXN7;MAPK1;HOXC8;MARK1;NDC1;SEPT10;SEPT11;SEPT12;TPM3;TMOD3

[illegible];RBX1;CKS1B;KLHL7;BTRC;DTL

[illegible]IKRF;RBBP5;ERI1;ATXN1L;NRIP1;MACROD2;ZC3H14;SRPK2;UTP15;TRIM41;CBX5;CRBN;ZBTB14;ZFP

[illegible]PAFAH1B2;MTDH;SMAD7

[illegible]C3H1;EIF4E;GIGYF2;DCP2;PUM2
[illegible]N4;RCSD1;PPP1R9A;CDC42BPA;LMOD3;AMOT;MARCKS;CDH2;CAPZB;CAPZA1;HNRNPC;MYH10
[illegible]C73

[illegible]E2F1;CEP170;MACROD2;ARHGEF10;RANBP3;CSNK1A1;HAUS3;HOOK3;HIPK1;DYNLL2;PAX2;RAB11A

[illegible]M;SORBS3;RND3;ENAH;GNA13;MARCKS;HNRNPK;CDH2;G3BP1;DAG1;CTNNB1;PTK2B;MAPK1;TNS1;

MACROD2;RAB11FIP5;NDC1;ABCA2;ARHGEF10;RANBP3;CSNK1A1;HAUS3;HOOK3;HIPK1;SYTL4;DY[illegible]

FAH1B2

ALDH6A1;PDP2;ALDH2;ERBB4;CDK1;TFAM;NAT8L;OTC
T2;FUT11;ST8SIA3;RHOBTB3;ZDHHC21;PLEKHA3;NSG1;IL17RD;SORL1;CYTH3;MAN1A2;MGAT5;VTI1A

11A
TL4;PAX2;RAB11A;PPP2CA;APC;ATXN7;KIF5C;KIF26B;KIF1B;HOXC8;MAPRE2;KANK4;RAB11FIP5;MA
B11A
7;FRK
2

PK1;CALM1;RAB11A

D1;KIF1B;CALM1

;B3GAT2;FUT11;ST8SIA3;ZDHHC21;PLEKHA3;IL17RD;SORL1;RAB11A;CYTH3;MAN1A2;MGAT5;VTI1A;

C1A4;RCSD1;AMOT

IRPL42;RPS6KA6;C1QBP;TGM2;TSFM;MAP2K1;NSUN3;CMC1;HAUS3;NOS1AP;TFAM;PGAM5;SLC25A

FAH1B2

RPL42;TMEM65;SLC25A51;MICU3;SLC25A45;SLC25A12;CDS2;SLC25A24

AL1;SNX1;CLCN5;AP5M1;GPC1;GPC5;SLC29A3;AP2M1;ITM2C

N1

AP2M1;TPCN1

TN4;PAFAH1B2;FRK

-;MYOD1;COMMD3-BMI1;TERF2IP;WDFY3;RAF1;GLYR1;AMER1;FYTTD1;HDAC5;NUFIP2;TNKS;PLAG1

L1;KCNH1
EBBP;TRIM41;CBX5;ZBTB16;GTF2H1;ACTN4;GTF2H5;HIPK2;MYO1C;KANSL1;MYOD1;COMMD3-BMI1;T

OD1;ASXL3;HNRNPC;MXD1
C;MXD1

F1;SRSF10;ZC3H14;S100PBP;GLYR1
BP4;PUM2;APC;VAPA;KLHL7;NOS1AP;CTNNB1;ITM2C

CXADR;GPR37;MME;GPR75;PCDHA13;IL17RD;SHISA9;PCDHA12;ADRA2B;PCDHA11;ADRA2A;PCDHA

L3;MYOD1;CDK1;NABP1;TERF2IP;HNRNPC;MXD1

;TMOD2;DYRK1A;HAUS3;PARVA;ACTN4;SELE;MARCKS;SESTD1;CAPZA1;DCX;HNRNPC;MAPRE2;KA

?91;ZFY;RUNX3;ZFX;GTPBP4;DIEXF;PAX2;DIAPH2;DDX19B;ZZZ3;CDKN2AIPNL;SUB1;KLHL7;CMPK1;V

.;SMAD7;KAT2B;APC;CDK1;CTNNB1;CALM1;RAPGEF6;DTL

;TGM2

NLL2;PAX2;RAB11A;SMAD7;KAT2B;APC;CDK1;CTNNB1;CALM1;RAPGEF6;DTL

A;VPS54;ST6GALNAC3;SH3GL2;WLS;CHST3

RK1

VPS54;ST6GALNAC3;SH3GL2;WLS;CHST3

.12;RAF1;NAT8L;KANK2;AGAP2;DLST;MIEF1;PPM1K;PDHB;GRAMD4;FOXO1;TRAK2;PPP2CA;BCL2L13

1;YTHDC1;SRSF1;DTX1;HIF1A;ZBTB4;MTDH;NPAT;NXF1;BCLAF1;SRSF10;ZC3H14;S100PBP;CREBBP;

TERF2IP

A10;EREG;TGFBR3;ADCY9;NRG3;AGTR2;SLC24A2;PTGFR;ADCYAP1R1;NPR3;NRXN3;SLC1A4;APCDD

NK4

WDFY3;ERGIC2;DTL;PAFAH1B2;SRSF9;FGFR1

;ERBB4;MAPK1;SLC25A24;RAB11FIP5;TIMMDC1;COA1;GATC;SOD2;SYNJ2BP;LETM1;MAPK10;ALDH

TRIM41;CBX5;SMURF2;CSNK1A1;ZBTB16;YLPM1;HIPK2;MYO1C;TRIP12

)1;TRHDE;PGRMC1;ZNRF3;APH1B;EFNB3;PCDHA1;ERBB4;GPC1;PCDHA5;PLXNA2;HAS2;PCDHA4;G

6A1;GLUD2;CDK1;MXD1;OTC

PC5;PCDHA3;S1PR3;PCDHA2;PCDHA9;RELT;PCDHA8;JAM2;PCDHA7;PCDHA6;PLXNA3;ABCA1;GABF

RA1;CADM3;FZD5;TPBG;GABRA3;PCDHB15;ATP2B4;LAPTM5;SLC4A10;LNPEP;ATP2B2;BFAR;ATP2B1;

;GRIN2B;SORL1;SELE;EPOR;TSHR;CLCN5;EFNA3;CAPRIN1;LRP12;SLC28A3;FGFR1
