# Supplementary material for: Circulating miR-330-3p in Late Pregnancy is Associated with Pregnancy Outcomes Among Lean Women with GDM
Source: Sci Rep. 2020 Jan 22;10:908. doi: 10.1038/s41598-020-57838-6 (PMC6976655; doi:10.1038/s41598-020-57838-6)
Supplement: Supplementary file 1 — Supplementary data. [file 41598_2020_57838_MOESM1_ESM.zip › Supplimentary File_EnrichR_Analysis GO_Molecular_Function_2018.pdf]

Term

ubiquitin-like protein ligase activity (GO:0061659)
transcription coactivator activity (GO:0003713)
ubiquitin protein ligase activity (GO:0061630)
transcription regulatory region DNA binding (GO:0044212)
transcriptional activator activity, RNA polymerase II core promoter proximal region sequence-specific binding (G
core promoter proximal region sequence-specific DNA binding (GO:0000987)
ubiquitin-protein transferase activity (GO:0004842)
nuclear hormone receptor binding (GO:0035257)
transcription factor activity, RNA polymerase II core promoter proximal region sequence-specific binding (GO:00
RNA polymerase II transcription factor binding (GO:0001085)
RNA polymerase II regulatory region sequence-specific DNA binding (GO:0000977)
transcriptional activator activity, RNA polymerase II transcription regulatory region sequence-specific binding (G
core promoter binding (GO:0001047)
RNA polymerase II core promoter proximal region sequence-specific DNA binding (GO:0000978)
peptide N-acetyltransferase activity (GO:0034212)
estrogen receptor binding (GO:0030331)
transcription regulatory region sequence-specific DNA binding (GO:0000976)
RNA binding (GO:0003723)
regulatory region DNA binding (GO:0000975)
syntaxin binding (GO:0019905)
BMP receptor activity (GO:0098821)
sequence-specific DNA binding (GO:0043565)
activating transcription factor binding (GO:0033613)
RNA polymerase II activating transcription factor binding (GO:0001102)
transcriptional repressor activity, RNA polymerase II transcription factor binding (GO:0001191)
low-density lipoprotein receptor activity (GO:0005041)
RNA polymerase II regulatory region DNA binding (GO:0001012)
RNA polymerase II transcription cofactor activity (GO:0001104)
telomerase RNA binding (GO:0070034)
transmembrane receptor protein serine/threonine kinase activity (GO:0004675)
ubiquitin-like protein-specific protease activity (GO:0019783)
mRNA 3'-UTR binding (GO:0003730)
lipoprotein particle receptor activity (GO:0030228)
mannosyl-oligosaccharide 1,2-alpha-mannosidase activity (GO:0004571)
guanyl ribonucleotide binding (GO:0032561)
thiol-dependent ubiquitinyl hydrolase activity (GO:0036459)
transcriptional repressor activity, RNA polymerase II transcription regulatory region sequence-specific binding (G
purine ribonucleoside binding (GO:0032550)
transcription corepressor activity (GO:0003714)
GTP binding (GO:0005525)
phosphatidic acid binding (GO:0070300)
protein phosphatase regulator activity (GO:0019888)
ubiquitin-like protein ligase binding (GO:0044389)
mRNA binding (GO:0003729)
NAD+ ADP-ribosyltransferase activity (GO:0003950)
mannosyl-oligosaccharide mannosidase activity (GO:0015924)
ubiquitin protein ligase binding (GO:0031625)
double-stranded DNA binding (GO:0003690)
purine ribonucleoside triphosphate binding (GO:0035639)
ephrin receptor binding (GO:0046875)
transcriptional activator activity, RNA polymerase II transcription factor binding (GO:0001190)
RNA polymerase II transcription coactivator activity (GO:0001105)

Lys63-specific deubiquitinase activity (GO:0061578)
nitric-oxide synthase binding (GO:0050998)
3',5'-cyclic-GMP phosphodiesterase activity (GO:0047555)
GDP binding (GO:0019003)
thiol-dependent ubiquitin-specific protease activity (GO:0004843)
protein phosphatase 2A binding (GO:0051721)
3',5'-cyclic-nucleotide phosphodiesterase activity (GO:0004114)
ubiquitin conjugating enzyme binding (GO:0031624)
hormone receptor binding (GO:0051427)
pre-mRNA binding (GO:0036002)
GABA-gated chloride ion channel activity (GO:0022851)
SH3 domain binding (GO:0017124)
Wnt-activated receptor activity (GO:0042813)
N-acetyltransferase activity (GO:0008080)
core promoter proximal region DNA binding (GO:0001159)
phosphoprotein phosphatase activity (GO:0004721)
RNA stem-loop binding (GO:0035613)
tropomyosin binding (GO:0005523)
calcium-transporting ATPase activity (GO:0005388)
vitamin D receptor binding (GO:0042809)
protein-disulfide reductase activity (GO:0047134)
chondroitin sulfotransferase activity (GO:0034481)
alpha-adrenergic receptor activity (GO:0004936)
inositol trisphosphate kinase activity (GO:0051766)
exoribonuclease activity (GO:0004532)
LBD domain binding (GO:0050693)
ubiquitin-like protein conjugating enzyme binding (GO:0044390)
single-stranded DNA binding (GO:0003697)
acetyltransferase activity (GO:0016407)
protein kinase binding (GO:0019901)
3',5'-cyclic-AMP phosphodiesterase activity (GO:0004115)
RNA polymerase II carboxy-terminal domain kinase activity (GO:0008353)
protein serine/threonine phosphatase activity (GO:0004722)
epidermal growth factor receptor binding (GO:0005154)
steroid hormone receptor binding (GO:0035258)
small GTPase binding (GO:0031267)
nucleotidyltransferase activity (GO:0016779)
N6-methyladenosine-containing RNA binding (GO:1990247)
N-acetylglucosamine 6-O-sulfotransferase activity (GO:0001517)
chloride ion binding (GO:0031404)
palmitoyl-(protein) hydrolase activity (GO:0008474)
NMDA glutamate receptor activity (GO:0004972)
leucine binding (GO:0070728)
transcription factor activity, RNA polymerase II distal enhancer sequence-specific binding (GO:0003705)
protein phosphatase binding (GO:0019903)
ligand-dependent nuclear receptor transcription coactivator activity (GO:0030374)
ligand-dependent nuclear receptor binding (GO:0016922)
receptor antagonist activity (GO:0048019)
protein kinase B binding (GO:0043422)
tau protein binding (GO:0048156)
m7G(5')pppN diphosphatase activity (GO:0050072)
oxidoreductase activity, acting on a sulfur group of donors, NAD(P) as acceptor (GO:0016668)
epinephrine binding (GO:0051379)

ligand-gated anion channel activity (GO:0099095)
pyrophosphatase activity (GO:0016462)
magnesium ion binding (GO:0000287)
protein serine/threonine kinase activity (GO:0004674)
transferase activity, transferring pentosyl groups (GO:0016763)
phosphotransferase activity, alcohol group as acceptor (GO:0016773)
GABA-A receptor activity (GO:0004890)
insulin receptor binding (GO:0005158)
double-stranded telomeric DNA binding (GO:0003691)
complement component C1q binding (GO:0001849)
protein phosphatase activator activity (GO:0072542)
phosphatase activator activity (GO:0019211)
BMP binding (GO:0036122)
cytidylyltransferase activity (GO:0070567)
DNA binding (GO:0003677)
metal ion binding (GO:0046872)
transforming growth factor beta receptor binding (GO:0005160)
ubiquitin conjugating enzyme activity (GO:0061631)
ligand-gated calcium channel activity (GO:0099604)
phospholipid-translocating ATPase activity (GO:0004012)
core promoter sequence-specific DNA binding (GO:0001046)
exopeptidase activity (GO:0008238)
telomeric DNA binding (GO:0042162)
adenyl ribonucleotide binding (GO:0032559)
Lys48-specific deubiquitinase activity (GO:1990380)
prostaglandin receptor activity (GO:0004955)
benzodiazepine receptor activity (GO:0008503)
small protein activating enzyme activity (GO:0008641)
carbon-nitrogen ligase activity, with glutamine as amido-N-donor (GO:0016884)
protein-hormone receptor activity (GO:0016500)
histone acetyltransferase activity (H4-K8 specific) (GO:0043996)
vinculin binding (GO:0017166)
cyclic-nucleotide phosphodiesterase activity (GO:0004112)
histone acetyltransferase activity (H4-K5 specific) (GO:0043995)
I-SMAD binding (GO:0070411)
histone acetyltransferase activity (H4-K16 specific) (GO:0046972)
voltage-gated ion channel activity (GO:0005244)
sialyltransferase activity (GO:0008373)
neurotransmitter receptor activity involved in regulation of postsynaptic membrane potential (GO:0099529)
ubiquitin-like protein conjugating enzyme activity (GO:0061650)
GABA receptor activity (GO:0016917)
kinase activity (GO:0016301)
protein heterodimerization activity (GO:0046982)
transmitter-gated ion channel activity (GO:0022824)
kinase binding (GO:0019900)
chloride channel activity (GO:0005254)
NAD binding (GO:0051287)
ATPase activity, coupled to transmembrane movement of ions, phosphorylative mechanism (GO:0015662)
histone acetyltransferase binding (GO:0035035)
PDZ domain binding (GO:0030165)
histone methyltransferase activity (GO:0042054)
protein tyrosine kinase binding (GO:1990782)
semaphorin receptor activity (GO:0017154)

U6 snRNA binding (GO:0017070)
prenyltransferase activity (GO:0004659)
peptide alpha-N-acetyltransferase activity (GO:0004596)
ADP binding (GO:0043531)
fibroblast growth factor binding (GO:0017134)
voltage-gated ion channel activity involved in regulation of postsynaptic membrane potential (GO:1905030)
calcium-release channel activity (GO:0015278)
voltage-gated sodium channel activity (GO:0005248)
ATP binding (GO:0005524)
enhancer sequence-specific DNA binding (GO:0001158)
cysteine-type peptidase activity (GO:0008234)
calcium:cation antiporter activity (GO:0015368)
SUMO binding (GO:0032183)
RNA polymerase II basal transcription factor binding (GO:0001091)
sodium ion binding (GO:0031402)
phosphatidylinositol-3,5-bisphosphate 3-phosphatase activity (GO:0052629)
oxidoreductase activity, acting on NAD(P)H, oxygen as acceptor (GO:0050664)
voltage-gated chloride channel activity (GO:0005247)
Rab guanyl-nucleotide exchange factor activity (GO:0017112)
SH3/SH2 adaptor activity (GO:0005070)
protein homodimerization activity (GO:0042803)
protein-cysteine S-palmitoyltransferase activity (GO:0019706)
protein-cysteine S-acyltransferase activity (GO:0019707)
cysteine-type endopeptidase inhibitor activity involved in apoptotic process (GO:0043027)
ATPase coupled ion transmembrane transporter activity (GO:0042625)
RNA polymerase II distal enhancer sequence-specific DNA binding (GO:0000980)
methylation-dependent protein binding (GO:0140034)
microtubule binding (GO:0008017)
carbohydrate kinase activity (GO:0019200)
glycine binding (GO:0016594)
ubiquitin-ubiquitin ligase activity (GO:0034450)
acidic amino acid transmembrane transporter activity (GO:0015172)
nucleoside-diphosphatase activity (GO:0017110)
thiolester hydrolase activity (GO:0016790)
phosphatidylinositol-4,5-bisphosphate 3-kinase activity (GO:0046934)
repressing transcription factor binding (GO:0070491)
amino acid binding (GO:0016597)
peptide-lysine-N-acetyltransferase activity (GO:0061733)
phosphatidylinositol binding (GO:0035091)
nucleoside-triphosphatase activity (GO:0017111)
protein kinase activity (GO:0004672)
thyroid hormone receptor binding (GO:0046966)
calcium channel regulator activity (GO:0005246)
RNA polymerase II core binding (GO:0000993)
RNA polymerase II transcription corepressor activity (GO:0001106)
MAP kinase activity (GO:0004707)
phosphatidylinositol monophosphate phosphatase activity (GO:0052744)
1-phosphatidylinositol binding (GO:0005545)
poly(A) binding (GO:0008143)
transcription coactivator binding (GO:0001223)
L-glutamate transmembrane transporter activity (GO:0005313)
nucleoside kinase activity (GO:0019206)
phosphatidylinositol-3-phosphatase activity (GO:0004438)

methylated histone binding (GO:0035064)
metallopeptidase activity (GO:0008237)
phosphatidylinositol bisphosphate kinase activity (GO:0052813)
histone-lysine N-methyltransferase activity (GO:0018024)
transferase activity, transferring acyl groups other than amino-acyl groups (GO:0016747)
tubulin binding (GO:0015631)
basal RNA polymerase II transcription machinery binding (GO:0001099)
cyclin-dependent protein serine/threonine kinase regulator activity (GO:0016538)
calmodulin-dependent protein kinase activity (GO:0004683)
phosphatidylinositol-3,5-bisphosphate phosphatase activity (GO:0106018)
inhibitory extracellular ligand-gated ion channel activity (GO:0005237)
intracellular ligand-gated ion channel activity (GO:0005217)
adrenergic receptor binding (GO:0031690)
myosin V binding (GO:0031489)
androgen receptor binding (GO:0050681)
palmitoyltransferase activity (GO:0016409)
non-membrane spanning protein tyrosine kinase activity (GO:0004715)
Rac GTPase binding (GO:0048365)
Rho guanyl-nucleotide exchange factor activity (GO:0005089)
kinase inhibitor activity (GO:0019210)
retinoic acid receptor binding (GO:0042974)
alkali metal ion binding (GO:0031420)
adrenergic receptor activity (GO:0004935)
ionotropic glutamate receptor activity (GO:0004970)
alpha-actinin binding (GO:0051393)
cation-transporting ATPase activity (GO:0019829)
phosphatidylinositol-4,5-bisphosphate binding (GO:0005546)
phosphatidylinositol 3-kinase activity (GO:0035004)
protein serine/threonine kinase inhibitor activity (GO:0030291)
snoRNA binding (GO:0030515)
Notch binding (GO:0005112)
ephrin receptor activity (GO:0005003)
H4 histone acetyltransferase activity (GO:0010485)
metalloaminopeptidase activity (GO:0070006)
aspartic-type peptidase activity (GO:0070001)
low-density lipoprotein particle binding (GO:0030169)
transmembrane-ephrin receptor activity (GO:0005005)
calcium ion binding (GO:0005509)
myosin binding (GO:0017022)
ubiquitin binding (GO:0043130)
protein serine/threonine kinase activator activity (GO:0043539)
ATP-dependent microtubule motor activity, plus-end-directed (GO:0008574)
protein-lysine N-methyltransferase activity (GO:0016279)
Ran GTPase binding (GO:0008536)
phosphatase inhibitor activity (GO:0019212)
lysine-acetylated histone binding (GO:0070577)
voltage-gated anion channel activity (GO:0008308)
cation transmembrane transporter activity (GO:0008324)
transcriptional repressor activity, RNA polymerase II core promoter proximal region sequence-specific binding (
G-protein coupled peptide receptor activity (GO:0008528)
phospholipid transporter activity (GO:0005548)
cysteine-type endopeptidase activity (GO:0004197)
metalloendopeptidase activity (GO:0004222)

RNA polymerase binding (GO:0070063)
R-SMAD binding (GO:0070412)
spectrin binding (GO:0030507)
acetylation-dependent protein binding (GO:0140033)
amyloid-beta binding (GO:0001540)
GTPase activity (GO:0003924)
aminopeptidase activity (GO:0004177)
RNA polymerase core enzyme binding (GO:0043175)
alcohol dehydrogenase activity, zinc-dependent (GO:0004024)
purine nucleobase transmembrane transporter activity (GO:0005345)
protein phosphatase 2B binding (GO:0030346)
clathrin light chain binding (GO:0032051)
histone methyltransferase activity (H3-K36 specific) (GO:0046975)
prostaglandin E receptor activity (GO:0004957)
nerve growth factor binding (GO:0048406)
G-protein coupled neurotransmitter receptor activity (GO:0099528)
protein-lysine 6-oxidase activity (GO:0004720)
alpha-N-acetylgalactosaminide alpha-2,6-sialyltransferase activity (GO:0001665)
NEDD8 transferase activity (GO:0019788)
ubiquitin-dependent protein binding (GO:0140036)
cytochrome-b5 reductase activity, acting on NAD(P)H (GO:0004128)
alpha-N-acetylneuraminate alpha-2,8-sialyltransferase activity (GO:0003828)
G-protein coupled acetylcholine receptor activity (GO:0016907)
ADP-ribosylation factor binding (GO:0030306)
NAD(P)H oxidase activity (GO:0016174)
olfactory receptor binding (GO:0031849)
transforming growth factor beta-activated receptor activity (GO:0005024)
U4 snRNA binding (GO:0030621)
peroxisome proliferator activated receptor binding (GO:0042975)
retinol binding (GO:0019841)
glucosyltransferase activity (GO:0046527)
polynucleotide adenylyltransferase activity (GO:0004652)
U3 snoRNA binding (GO:0034511)
aldehyde dehydrogenase [NAD(P)+] activity (GO:0004030)
protein kinase inhibitor activity (GO:0004860)
translation factor activity, RNA binding (GO:0008135)
low-density lipoprotein particle receptor binding (GO:0050750)
glutamate receptor activity (GO:0008066)
poly-pyrimidine tract binding (GO:0008187)
poly-purine tract binding (GO:0070717)
TBP-class protein binding (GO:0017025)
transcription factor activity, RNA polymerase II core promoter sequence-specific (GO:0000983)
aspartic-type endopeptidase activity (GO:0004190)
exonuclease activity (GO:0004527)
transmitter-gated ion channel activity involved in regulation of postsynaptic membrane potential (GO:1904315)
snRNA binding (GO:0017069)
translation initiation factor activity (GO:0003743)
phosphatase activity (GO:0016791)
phosphatidylinositol bisphosphate binding (GO:1902936)
calcium-dependent phospholipase A2 activity (GO:0047498)
interleukin-17 receptor activity (GO:0030368)
satellite DNA binding (GO:0003696)
superoxide-generating NADPH oxidase activity (GO:0016175)

annealing helicase activity (GO:0036310)
phospholipase binding (GO:0043274)
ribosomal protein S6 kinase activity (GO:0004711)
interleukin-6 receptor binding (GO:0005138)
cAMP response element binding (GO:0035497)
activin-activated receptor activity (GO:0017002)
inositol-1,3,4,5-tetrakisphosphate 5-phosphatase activity (GO:0052659)
NAADP-sensitive calcium-release channel activity (GO:0072345)
Arp2/3 complex binding (GO:0071933)
protein-glutamine gamma-glutamyltransferase activity (GO:0003810)
alanine transmembrane transporter activity (GO:0022858)
[heparan sulfate]-glucosamine 3-sulfotransferase 1 activity (GO:0008467)
poly(G) binding (GO:0034046)
SUMO-specific protease activity (GO:0016929)
C4-dicarboxylate transmembrane transporter activity (GO:0015556)
L-proline transmembrane transporter activity (GO:0015193)
protein binding involved in heterotypic cell-cell adhesion (GO:0086080)
mRNA methyltransferase activity (GO:0008174)
L-alanine transmembrane transporter activity (GO:0015180)
chitinase activity (GO:0004568)
protein carboxyl O-methyltransferase activity (GO:0051998)
frizzled binding (GO:0005109)
phosphatidylinositol-4-phosphate binding (GO:0070273)
actinin binding (GO:0042805)
mRNA 5'-UTR binding (GO:0048027)
bHLH transcription factor binding (GO:0043425)
zinc ion transmembrane transporter activity (GO:0005385)
metalloexopeptidase activity (GO:0008235)
protein kinase regulator activity (GO:0019887)
peptidyl-prolyl cis-trans isomerase activity (GO:0003755)
divalent inorganic cation transmembrane transporter activity (GO:0072509)
ribosome binding (GO:0043022)
protein kinase activator activity (GO:0030295)
protein phosphatase inhibitor activity (GO:0004864)
lipoprotein particle binding (GO:0071813)
chloride transmembrane transporter activity (GO:0015108)
ATPase activity, coupled to movement of substances (GO:0043492)
actin filament binding (GO:0051015)
receptor tyrosine kinase binding (GO:0030971)
signal sequence binding (GO:0005048)
type II transforming growth factor beta receptor binding (GO:0005114)
aryl hydrocarbon receptor binding (GO:0017162)
transferrin receptor binding (GO:1990459)
ether hydrolase activity (GO:0016803)
alpha-(1->3)-fucosyltransferase activity (GO:0046920)
mismatched DNA binding (GO:0030983)
cyclic nucleotide-dependent protein kinase activity (GO:0004690)
calcium-dependent protein kinase activity (GO:0010857)
phosphatidylinositol transporter activity (GO:0008526)
phosphofructokinase activity (GO:0008443)
sodium:bicarbonate symporter activity (GO:0008510)
activin receptor binding (GO:0070697)
type I transforming growth factor beta receptor binding (GO:0034713)

phosphatidylcholine transporter activity (GO:0008525)
sulfurtransferase activity (GO:0016783)
oxidoreductase activity, acting on diphenols and related substances as donors, cytochrome as acceptor (GO:00
protein-arginine omega-N asymmetric methyltransferase activity (GO:0035242)
bioactive lipid receptor activity (GO:0045125)
NAD+ binding (GO:0070403)
tRNA (cytosine) methyltransferase activity (GO:0016427)
ubiquinol-cytochrome-c reductase activity (GO:0008121)
alcohol dehydrogenase (NAD) activity (GO:0004022)
coreceptor activity involved in Wnt signaling pathway (GO:0071936)
protein tyrosine kinase activity (GO:0004713)
sodium channel activity (GO:0005272)
cadherin binding (GO:0045296)
ion channel activity (GO:0005216)
Ras GTPase binding (GO:0017016)
ATPase activity (GO:0016887)
GTPase binding (GO:0051020)
integrin binding (GO:0005178)
histone acetyltransferase activity (GO:0004402)
retinoid binding (GO:0005501)
disulfide oxidoreductase activity (GO:0015036)
miRNA binding (GO:0035198)
calcium ion transmembrane transporter activity (GO:0015085)
RNA 7-methylguanosine cap binding (GO:0000340)
ribonuclease P activity (GO:0004526)
ATP transmembrane transporter activity (GO:0005347)
GABA receptor binding (GO:0050811)
armadillo repeat domain binding (GO:0070016)
RAGE receptor binding (GO:0050786)
superoxide-generating NADPH oxidase activator activity (GO:0016176)
histone-arginine N-methyltransferase activity (GO:0008469)
histone demethylase activity (H3-K36 specific) (GO:0051864)
sterol esterase activity (GO:0004771)
dolichyl-diphosphooligosaccharide-protein glycotransferase activity (GO:0004579)
retinoid X receptor binding (GO:0046965)
actin-dependent ATPase activity (GO:0030898)
neurotrophin binding (GO:0043121)
glucocorticoid receptor binding (GO:0035259)
inositol tetrakisphosphate phosphatase activity (GO:0052743)
cAMP-dependent protein kinase inhibitor activity (GO:0004862)
oxidoreductase activity, acting on the aldehyde or oxo group of donors, NAD or NADP as acceptor (GO:001662
lipoprotein particle receptor binding (GO:0070325)
transcription cofactor binding (GO:0001221)
exoribonuclease activity, producing 5'-phosphomonoesters (GO:0016896)
zinc ion binding (GO:0008270)
1-phosphatidylinositol-3-kinase activity (GO:0016303)
enhancer binding (GO:0035326)
SH2 domain binding (GO:0042169)
protein tyrosine phosphatase activity (GO:0004725)
G-protein coupled receptor activity (GO:0004930)
histone kinase activity (GO:0035173)
store-operated calcium channel activity (GO:0015279)
protein serine/threonine phosphatase inhibitor activity (GO:0004865)

phosphatidylinositol-5-phosphate binding (GO:0010314)
DNA N-glycosylase activity (GO:0019104)
endocytic adaptor activity (GO:0098748)
phosphatidylglycerol binding (GO:1901611)
tau-protein kinase activity (GO:0050321)
neurexin family protein binding (GO:0042043)
oxidoreductase activity, acting on a sulfur group of donors, disulfide as acceptor (GO:0016671)
arginine N-methyltransferase activity (GO:0016273)
adenylate cyclase binding (GO:0008179)
oligosaccharyl transferase activity (GO:0004576)
calcium channel inhibitor activity (GO:0019855)
eukaryotic initiation factor 4E binding (GO:0008190)
hydrolase activity, acting on acid anhydrides, in phosphorus-containing anhydrides (GO:0016818)
clathrin adaptor activity (GO:0035615)
long-chain fatty acid-CoA ligase activity (GO:0004467)
activin binding (GO:0048185)
cGMP binding (GO:0030553)
G-rich strand telomeric DNA binding (GO:0098505)
cyclin-dependent protein serine/threonine kinase inhibitor activity (GO:0004861)
adenine nucleotide transmembrane transporter activity (GO:0000295)
ADP transmembrane transporter activity (GO:0015217)
co-SMAD binding (GO:0070410)
GTPase activator activity (GO:0005096)
single-stranded RNA binding (GO:0003727)
phosphatase binding (GO:0019902)
filamin binding (GO:0031005)
protein phosphatase 1 binding (GO:0008157)
ribosomal large subunit binding (GO:0043023)
gap junction channel activity (GO:0005243)
thioesterase binding (GO:0031996)
nucleotide diphosphatase activity (GO:0004551)
caspase binding (GO:0089720)
adenyl nucleotide binding (GO:0030554)
intramolecular transferase activity, phosphotransferases (GO:0016868)
dynactin binding (GO:0034452)
adenyl-nucleotide exchange factor activity (GO:0000774)
mitogen-activated protein kinase kinase kinase binding (GO:0031435)
calcium-dependent protein serine/threonine kinase activity (GO:0009931)
acylglycerol lipase activity (GO:0047372)
actin binding (GO:0003779)
mitogen-activated protein kinase kinase binding (GO:0031434)
ATP-dependent DNA helicase activity (GO:0004003)
inorganic anion exchanger activity (GO:0005452)
kinesin binding (GO:0019894)
dicarboxylic acid transmembrane transporter activity (GO:0005310)
RNA polymerase II repressing transcription factor binding (GO:0001103)
S-adenosylmethionine-dependent methyltransferase activity (GO:0008757)
calcium-dependent phospholipid binding (GO:0005544)
GTPase regulator activity (GO:0030695)
anion channel activity (GO:0005253)
histone deacetylase binding (GO:0042826)
delayed rectifier potassium channel activity (GO:0005251)
WW domain binding (GO:0050699)

nucleosomal DNA binding (GO:0031492)
ATP-dependent microtubule motor activity (GO:1990939)
arylsulfatase activity (GO:0004065)
voltage-gated potassium channel activity involved in cardiac muscle cell action potential repolarization (GO:008
Ral GTPase binding (GO:0017160)
single-stranded telomeric DNA binding (GO:0043047)
wide pore channel activity (GO:0022829)
protein tyrosine kinase activator activity (GO:0030296)
potassium ion binding (GO:0030955)
protein-arginine N-methyltransferase activity (GO:0016274)
poly(A)-specific ribonuclease activity (GO:0004535)
transcriptional repressor activity, RNA polymerase II activating transcription factor binding (GO:0098811)
annealing activity (GO:0097617)
ligand-gated ion channel activity (GO:0015276)
phospholipase A2 activity (GO:0004623)
Hsp90 protein binding (GO:0051879)
ribonuclease activity (GO:0004540)
L-amino acid transmembrane transporter activity (GO:0015179)
acetylglucosaminyltransferase activity (GO:0008375)
3'-5'-exoribonuclease activity (GO:0000175)
oxidoreductase activity, acting on paired donors, with incorporation or reduction of molecular oxygen, 2-oxogluta
ligand-gated channel activity (GO:0022834)
microtubule plus-end binding (GO:0051010)
fucosyltransferase activity (GO:0008417)
tRNA-specific ribonuclease activity (GO:0004549)
ammonium ion binding (GO:0070405)
histone demethylase activity (H3-K9 specific) (GO:0032454)
GTPase inhibitor activity (GO:0005095)
oxidoreductase activity, acting on NAD(P)H, heme protein as acceptor (GO:0016653)
carbonate dehydratase activity (GO:0004089)
translation elongation factor activity (GO:0003746)
phosphatidylinositol kinase activity (GO:0052742)
cysteine-type endopeptidase inhibitor activity (GO:0004869)
transition metal ion transmembrane transporter activity (GO:0046915)
hydrolase activity, hydrolyzing O-glycosyl compounds (GO:0004553)
Rab GTPase binding (GO:0017137)
extracellular ligand-gated ion channel activity (GO:0005230)
outward rectifier potassium channel activity (GO:0015271)
Rac guanyl-nucleotide exchange factor activity (GO:0030676)
acid-amino acid ligase activity (GO:0016881)
5'-nucleotidase activity (GO:0008253)
retinoic acid binding (GO:0001972)
aldehyde dehydrogenase (NAD) activity (GO:0004029)
heparan sulfate sulfotransferase activity (GO:0034483)
hydrolase activity, hydrolyzing N-glycosyl compounds (GO:0016799)
titin binding (GO:0031432)
DNA-dependent ATPase activity (GO:0008094)
Rho GTPase binding (GO:0017048)
growth factor receptor binding (GO:0070851)
sequence-specific double-stranded DNA binding (GO:1990837)
calcium channel activity (GO:0005262)
G-protein coupled receptor binding (GO:0001664)
helicase activity (GO:0004386)

ATPase activity, coupled (GO:0042623)
MHC class I protein binding (GO:0042288)
protein serine/threonine/tyrosine kinase activity (GO:0004712)
oxidoreductase activity, acting on the CH-NH2 group of donors, oxygen as acceptor (GO:0016641)
protein disulfide oxidoreductase activity (GO:0015035)
syntaxin-1 binding (GO:0017075)
FK506 binding (GO:0005528)
transmembrane receptor protein tyrosine phosphatase activity (GO:0005001)
transmembrane receptor protein phosphatase activity (GO:0019198)
5'-3' exonuclease activity (GO:0008409)
neuropeptide receptor activity (GO:0008188)
ion transmembrane transporter activity (GO:0015075)
G-protein coupled amine receptor activity (GO:0008227)
histone methyltransferase activity (H3-K4 specific) (GO:0042800)
ubiquitin-specific protease binding (GO:1990381)
muscle alpha-actinin binding (GO:0051371)
sulfuric ester hydrolase activity (GO:0008484)
transferase activity, transferring amino-acyl groups (GO:0016755)
potassium ion antiporter activity (GO:0022821)
nucleotidase activity (GO:0008252)
cysteine-type endopeptidase activator activity involved in apoptotic process (GO:0008656)
poly(U) RNA binding (GO:0008266)
MAP kinase kinase kinase activity (GO:0004709)
voltage-gated calcium channel activity (GO:0005245)
transition metal ion binding (GO:0046914)
RNA cap binding (GO:0000339)
cholesterol transporter activity (GO:0017127)
antiporter activity (GO:0015297)
CXCR chemokine receptor binding (GO:0045236)
DNA binding, bending (GO:0008301)
G-protein beta/gamma-subunit complex binding (GO:0031683)
retinol dehydrogenase activity (GO:0004745)
nuclease activity (GO:0004518)
double-stranded RNA binding (GO:0003725)
3'-5' exonuclease activity (GO:0008408)
microfilament motor activity (GO:0000146)
SUMO transferase activity (GO:0019789)
fatty acid ligase activity (GO:0015645)
ankyrin binding (GO:0030506)
hyaluronic acid binding (GO:0005540)
semaphorin receptor binding (GO:0030215)
peptidase activator activity involved in apoptotic process (GO:0016505)
nucleoside diphosphate kinase activity (GO:0004550)
transcriptional activator activity, RNA polymerase II distal enhancer sequence-specific binding (GO:0001205)
microtubule motor activity (GO:0003777)
phosphatidylinositol phosphate binding (GO:1901981)
sulfotransferase activity (GO:0008146)
peptidase activity, acting on L-amino acid peptides (GO:0070011)
transmembrane receptor protein tyrosine kinase activity (GO:0004714)
carboxylic ester hydrolase activity (GO:0052689)
histone deacetylase activity (GO:0004407)
DNA helicase activity (GO:0003678)
RNA methyltransferase activity (GO:0008173)

intracellular chloride channel activity (GO:0061778)
transforming growth factor beta binding (GO:0050431)
intracellular calcium activated chloride channel activity (GO:0005229)
gamma-tubulin binding (GO:0043015)
protein kinase A regulatory subunit binding (GO:0034237)
cadherin binding involved in cell-cell adhesion (GO:0098641)
neuropilin binding (GO:0038191)
dynein complex binding (GO:0070840)
FAD binding (GO:0071949)
RNA polymerase II transcription factor activity, sequence-specific transcription regulatory region DNA binding (G
transmembrane receptor protein kinase activity (GO:0019199)
anion transmembrane transporter activity (GO:0008509)
phosphotransferase activity, phosphate group as acceptor (GO:0016776)
ATPase binding (GO:0051117)
methyl-CpG binding (GO:0008327)
proteasome binding (GO:0070628)
K63-linked polyubiquitin modification-dependent protein binding (GO:0070530)
motor activity (GO:0003774)
ATP-dependent helicase activity (GO:0008026)
protein deacetylase activity (GO:0033558)
hydrolase activity, acting on acid anhydrides, catalyzing transmembrane movement of substances (GO:001682(
lipase activity (GO:0016298)
rRNA binding (GO:0019843)
adenylyltransferase activity (GO:0070566)
N-methyltransferase activity (GO:0008170)
cyclic nucleotide binding (GO:0030551)
polypeptide N-acetylgalactosaminyltransferase activity (GO:0004653)
chemokine receptor activity (GO:0004950)
phosphatidylinositol-3,5-bisphosphate binding (GO:0080025)
secondary active transmembrane transporter activity (GO:0015291)
oxidoreductase activity, acting on the CH-OH group of donors, NAD or NADP as acceptor (GO:0016616)
solute:sodium symporter activity (GO:0015370)
lysine N-methyltransferase activity (GO:0016278)
bicarbonate transmembrane transporter activity (GO:0015106)
monocarboxylic acid binding (GO:0033293)
protein binding involved in cell-cell adhesion (GO:0098632)
methyltransferase activity (GO:0008168)
RNA helicase activity (GO:0003724)
ATP-dependent RNA helicase activity (GO:0004004)
hydro-lyase activity (GO:0016836)
ATPase activity, coupled to transmembrane movement of substances (GO:0042626)
P-P-bond-hydrolysis-driven transmembrane transporter activity (GO:0015405)
RNA-dependent ATPase activity (GO:0008186)
disordered domain specific binding (GO:0097718)
acetylcholine receptor activity (GO:0015464)
calcium activated cation channel activity (GO:0005227)
damaged DNA binding (GO:0003684)
growth factor activity (GO:0008083)
cytokine receptor binding (GO:0005126)
flavin adenine dinucleotide binding (GO:0050660)
acetylgalactosaminyltransferase activity (GO:0008376)
inorganic anion transmembrane transporter activity (GO:0015103)
NF-kappaB binding (GO:0051059)

mannosyltransferase activity (GO:0000030)
endoribonuclease activity, producing 5'-phosphomonoesters (GO:0016891)
kinase activator activity (GO:0019209)
death receptor activity (GO:0005035)
tumor necrosis factor-activated receptor activity (GO:0005031)
phosphatidylinositol-3,4-bisphosphate binding (GO:0043325)
phosphatidylinositol phospholipase C activity (GO:0004435)
neutral amino acid transmembrane transporter activity (GO:0015175)
cytokine receptor activity (GO:0004896)
endopeptidase activity (GO:0004175)
ion gated channel activity (GO:0022839)
metal ion transmembrane transporter activity (GO:0046873)
N-acyltransferase activity (GO:0016410)
actin monomer binding (GO:0003785)
hydrolase activity, acting on carbon-nitrogen (but not peptide) bonds, in linear amides (GO:0016811)
carboxy-lyase activity (GO:0016831)
alpha-tubulin binding (GO:0043014)
voltage-gated cation channel activity (GO:0022843)
transferase activity, transferring hexosyl groups (GO:0016758)
beta-tubulin binding (GO:0048487)
phospholipase C activity (GO:0004629)
Hsp70 protein binding (GO:0030544)
RNA polymerase II core promoter sequence-specific DNA binding (GO:0000979)
cation channel activity (GO:0005261)
iron ion binding (GO:0005506)
nuclear localization sequence binding (GO:0008139)
sterol transporter activity (GO:0015248)
phosphotyrosine residue binding (GO:0001784)
phosphoric ester hydrolase activity (GO:0042578)
amino acid transmembrane transporter activity (GO:0015171)
nucleobase-containing compound kinase activity (GO:0019205)
MHC protein binding (GO:0042287)
O-acyltransferase activity (GO:0008374)
phosphatidylinositol-3-phosphate binding (GO:0032266)
monocarboxylic acid transmembrane transporter activity (GO:0008028)
phosphatidylinositol-3,4,5-trisphosphate binding (GO:0005547)
voltage-gated potassium channel activity (GO:0005249)
ligand-gated cation channel activity (GO:0099094)
phosphoric diester hydrolase activity (GO:0008081)
cyclin-dependent protein serine/threonine kinase activity (GO:0004693)
sodium-independent organic anion transmembrane transporter activity (GO:0015347)
ion channel inhibitor activity (GO:0008200)
glucuronosyltransferase activity (GO:0015020)
protein tyrosine/serine/threonine phosphatase activity (GO:0008138)
cyclin-dependent protein kinase activity (GO:0097472)
carboxylic acid binding (GO:0031406)
E-box binding (GO:0070888)
protein phosphorylated amino acid binding (GO:0045309)
UDP-glycosyltransferase activity (GO:0008194)
manganese ion binding (GO:0030145)
carboxylic acid transmembrane transporter activity (GO:0046943)
ATPase regulator activity (GO:0060590)
phospholipase activity (GO:0004620)

protein kinase C binding (GO:0005080)
organic anion transmembrane transporter activity (GO:0008514)
sodium ion transmembrane transporter activity (GO:0015081)
copper ion binding (GO:0005507)
protein methyltransferase activity (GO:0008276)
heme binding (GO:0020037)
endonuclease activity (GO:0004519)
potassium channel regulator activity (GO:0015459)
anion:cation symporter activity (GO:0015296)
polyubiquitin modification-dependent protein binding (GO:0031593)
cholesterol binding (GO:0015485)
sterol binding (GO:0032934)
protein transporter activity (GO:0008565)
chemokine receptor binding (GO:0042379)
hydrogen ion transmembrane transporter activity (GO:0015078)
protease binding (GO:0002020)
potassium channel activity (GO:0005267)
hormone activity (GO:0005179)
cytokine activity (GO:0005125)
serine-type endopeptidase activity (GO:0004252)
serine-type peptidase activity (GO:0008236)

Overlap	P.value	Adjusted.P.value	Old.P.value	Old.Adjusted
25/187	1,22731E-05	0,014126389	0	0
33/292	2,06855E-05	0,011904522	0	0
25/193	2,12508E-05	0,008153216	0	0
39/375	2,64135E-05	0,007600497	0	0
22/176	0,00011098	0,025547517	0	0
30/279	0,000120193	0,0230571	0	0
40/418	0,00013251	0,021788393	0	0
11/57	0,000135674	0,019520149	0	0
30/281	0,000136555	0,017463899	0	0
17/122	0,000175777	0,020231989	0	0
42/461	0,000264392	0,027664979	0	0
29/285	0,000394189	0,03780927	0	0
14/98	0,000493909	0,043729936	0	0
27/263	0,000536332	0,044094141	0	0
5/15	0,000704559	0,054063143	0	0
7/33	0,001251733	0,090046513	0	0
28/293	0,001302482	0,088185679	0	0
97/1388	0,001324035	0,084664656	0	0
23/225	0,001416458	0,085807559	0	0
11/78	0,002134777	0,122856408	0	0
3/6	0,002435749	0,133502225	0	0
34/395	0,002516983	0,131683951	0	0
10/69	0,002727401	0,136488653	0	0
8/48	0,002932021	0,140614847	0	0
8/48	0,002932021	0,134990253	0	0
4/13	0,003463707	0,153335664	0	0
20/202	0,004042654	0,172336832	0	0
11/85	0,004225305	0,173690198	0	0
5/22	0,004573051	0,181502807	0	0
4/14	0,004653013	0,178520592	0	0
10/75	0,005038919	0,187090187	0	0
9/64	0,005359707	0,192781956	0	0
4/15	0,006088819	0,212370631	0	0
3/8	0,00630859	0,213564326	0	0
17/171	0,007355661	0,241896176	0	0
11/93	0,008328495	0,266280508	0	0
16/160	0,008657872	0,269330022	0	0
16/160	0,008657872	0,26224239	0	0
19/204	0,009456937	0,279100872	0	0
15/148	0,009609226	0,27650547	0	0
4/17	0,009778786	0,274521524	0	0
7/48	0,011043783	0,30265224	0	0
25/298	0,01182894	0,316630465	0	0
17/180	0,011960484	0,312875382	0	0
4/18	0,012068057	0,308674092	0	0
3/10	0,01250999	0,3130217	0	0
24/285	0,012890152	0,315671586	0	0
11/100	0,014001305	0,335739637	0	0
31/397	0,014604323	0,343052578	0	0
4/19	0,014673856	0,337792157	0	0
7/51	0,015207159	0,343204709	0	0
6/40	0,015891236	0,351746397	0	0

3/11	0,016549986	0,359415739	0	0
3/11	0,016549986	0,352759892	0	0
3/11	0,016549986	0,346346075	0	0
7/52	0,016813887	0,345585418	0	0
9/77	0,017383557	0,351025852	0	0
4/20	0,01760904	0,349448363	0	0
4/20	0,01760904	0,343525509	0	0
5/30	0,017613657	0,337888658	0	0
6/41	0,01782629	0,336361644	0	0
5/31	0,020133068	0,373760672	0	0
3/12	0,021233575	0,387934043	0	0
7/56	0,024441178	0,439559312	0	0
4/22	0,024509309	0,434003309	0	0
4/22	0,024509309	0,427427502	0	0
4/22	0,024509309	0,421047987	0	0
13/137	0,024919108	0,421792543	0	0
3/13	0,026564546	0,44312743	0	0
3/14	0,03254043	0,53505764	0	0
3/14	0,03254043	0,527521617	0	0
3/14	0,03254043	0,520194928	0	0
2/6	0,034729741	0,547588106	0	0
2/6	0,034729741	0,540188267	0	0
2/6	0,034729741	0,532985757	0	0
2/6	0,034729741	0,525972786	0	0
2/6	0,034729741	0,519141971	0	0
2/6	0,034729741	0,512486305	0	0
5/36	0,036210659	0,527575547	0	0
9/88	0,03751493	0,53974606	0	0
4/25	0,03754088	0,533451277	0	0
35/496	0,038155222	0,535569032	0	0
3/15	0,039153343	0,54295781	0	0
3/15	0,039153343	0,536494027	0	0
7/62	0,03985778	0,539721239	0	0
4/26	0,042615227	0,57035031	0	0
8/77	0,044593491	0,589966767	0	0
7/64	0,046145498	0,603562138	0	0
3/16	0,046390758	0,599952388	0	0
2/7	0,04698191	0,600846423	0	0
2/7	0,04698191	0,594243715	0	0
2/7	0,04698191	0,587784544	0	0
2/7	0,04698191	0,58146428	0	0
2/7	0,04698191	0,57527849	0	0
2/7	0,04698191	0,569222927	0	0
7/65	0,049514039	0,593652692	0	0
9/93	0,050500138	0,599233595	0	0
7/66	0,053034329	0,622882784	0	0
3/17	0,054236191	0,630564199	0	0
3/17	0,054236191	0,624258557	0	0
2/8	0,060539704	0,689912868	0	0
2/8	0,060539704	0,683149016	0	0
2/8	0,060539704	0,676516501	0	0
2/8	0,060539704	0,670011535	0	0
2/8	0,060539704	0,663630473	0	0

3/18	0,062669827	0,680499728	0	0
5/42	0,063661977	0,684812483	0	0
11/128	0,066941775	0,713425767	0	0
26/369	0,067325909	0,710936893	0	0
5/43	0,069121735	0,723264698	0	0
19/255	0,069651275	0,722239795	0	0
3/19	0,071669081	0,736527791	0	0
3/19	0,071669081	0,730009845	0	0
2/9	0,07523604	0,759620014	0	0
2/9	0,07523604	0,753014623	0	0
2/9	0,07523604	0,746523117	0	0
2/9	0,07523604	0,740142578	0	0
2/9	0,07523604	0,733870183	0	0
2/9	0,07523604	0,727703207	0	0
56/894	0,076523208	0,733985105	0	0
30/443	0,078632319	0,747981809	0	0
5/45	0,080789402	0,762201653	0	0
3/20	0,081209097	0,759932285	0	0
3/20	0,081209097	0,753803799	0	0
3/20	0,081209097	0,747773368	0	0
7/74	0,086747796	0,792434227	0	0
5/46	0,086991823	0,788406211	0	0
4/33	0,088215109	0,793246804	0	0
20/280	0,089065745	0,794687386	0	0
2/10	0,090917569	0,804970168	0	0
2/10	0,090917569	0,798825358	0	0
2/10	0,090917569	0,79277365	0	0
2/10	0,090917569	0,786812946	0	0
2/10	0,090917569	0,780941208	0	0
2/10	0,090917569	0,775156458	0	0
2/10	0,090917569	0,769456778	0	0
2/10	0,090917569	0,763840306	0	0
2/10	0,090917569	0,758305231	0	0
2/10	0,090917569	0,752849798	0	0
2/10	0,090917569	0,747472299	0	0
2/10	0,090917569	0,742171077	0	0
3/21	0,0912632	0,73974608	0	0
3/21	0,0912632	0,73457303	0	0
3/21	0,0912632	0,729471829	0	0
3/21	0,0912632	0,724440989	0	0
3/21	0,0912632	0,719479064	0	0
20/281	0,091491163	0,71636958	0	0
19/266	0,095509446	0,742779543	0	0
4/34	0,096102152	0,742373003	0	0
28/419	0,097946229	0,751574061	0	0
6/62	0,099506996	0,758493725	0	0
3/22	0,101803296	0,770892062	0	0
3/22	0,101803296	0,765853552	0	0
3/22	0,101803296	0,760880477	0	0
6/63	0,105462757	0,78314602	0	0
5/49	0,107028285	0,789676639	0	0
5/49	0,107028285	0,784646852	0	0
2/11	0,107443713	0,78270705	0	0

2/11	0,107443713	0,777784364	0	0
2/11	0,107443713	0,772923212	0	0
2/11	0,107443713	0,768122446	0	0
3/23	0,112800229	0,801438664	0	0
3/23	0,112800229	0,796521862	0	0
3/23	0,112800229	0,791665021	0	0
3/23	0,112800229	0,786867051	0	0
3/23	0,112800229	0,782126888	0	0
18/256	0,114104132	0,786430277	0	0
6/65	0,117912758	0,807842764	0	0
6/66	0,124400603	0,847249078	0	0
2/12	0,124685754	0,844195896	0	0
2/12	0,124685754	0,839259078	0	0
2/12	0,124685754	0,834379665	0	0
2/12	0,124685754	0,829556661	0	0
2/12	0,124685754	0,824789094	0	0
2/12	0,124685754	0,820076013	0	0
2/12	0,124685754	0,81541649	0	0
5/52	0,129104912	0,839546634	0	0
5/52	0,129104912	0,83483008	0	0
41/665	0,135916578	0,873966377	0	0
3/25	0,136044545	0,869929282	0	0
3/25	0,136044545	0,865123043	0	0
3/25	0,136044545	0,86036962	0	0
3/25	0,136044545	0,855668147	0	0
5/53	0,136889868	0,856305641	0	0
5/53	0,136889868	0,851676962	0	0
14/196	0,137065065	0,848182204	0	0
2/13	0,142525984	0,877258863	0	0
2/13	0,142525984	0,872592592	0	0
2/13	0,142525984	0,8679757	0	0
2/13	0,142525984	0,863407407	0	0
2/13	0,142525984	0,85888695	0	0
2/13	0,142525984	0,85441358	0	0
6/69	0,144871287	0,863973324	0	0
5/54	0,144876791	0,859552507	0	0
4/40	0,149700503	0,883616814	0	0
4/40	0,149700503	0,879108565	0	0
8/101	0,150436148	0,878944193	0	0
27/425	0,155380113	0,903244999	0	0
32/514	0,157112325	0,908725057	0	0
3/27	0,160752832	0,925132549	0	0
3/27	0,160752832	0,9205299	0	0
3/27	0,160752832	0,915972821	0	0
3/27	0,160752832	0,91146064	0	0
2/14	0,160856918	0,907579966	0	0
2/14	0,160856918	0,903152747	0	0
2/14	0,160856918	0,89876851	0	0
2/14	0,160856918	0,894426633	0	0
2/14	0,160856918	0,890126505	0	0
2/14	0,160856918	0,885867527	0	0
2/14	0,160856918	0,88164911	0	0
2/14	0,160856918	0,877470678	0	0

5/56	0,161427642	0,876430265	0	0
8/103	0,162428941	0,877726341	0	0
6/72	0,166763522	0,896938382	0	0
4/42	0,169660024	0,908272964	0	0
5/57	0,169976421	0,905753984	0	0
17/256	0,172923628	0,917212425	0	0
3/28	0,173579701	0,916468972	0	0
3/28	0,173579701	0,912284182	0	0
3/28	0,173579701	0,908137436	0	0
2/15	0,179580553	0,935281521	0	0
2/15	0,179580553	0,931068541	0	0
2/15	0,179580553	0,926893346	0	0
2/15	0,179580553	0,922755429	0	0
2/15	0,179580553	0,918654294	0	0
4/43	0,179971645	0,916581256	0	0
3/29	0,18668155	0,946565919	0	0
4/44	0,190485643	0,961618312	0	0
4/44	0,190485643	0,957419105	0	0
5/60	0,196620438	0,983957063	0	0
5/60	0,196620438	0,979697508	0	0
2/16	0,198607672	0,985333751	0	0
2/16	0,198607672	0,981104851	0	0
2/16	0,198607672	0,976912095	0	0
2/16	0,198607672	0,972755023	0	0
3/30	0,200028832	0,975564346	0	0
3/30	0,200028832	0,971448041	0	0
5/61	0,205806685	0,995308799	0	0
6/77	0,206052961	0,992330368	0	0
3/31	0,213592618	1	0	0
3/31	0,213592618	1	0	0
2/17	0,217857207	1	0	0
2/17	0,217857207	1	0	0
2/17	0,217857207	1	0	0
2/17	0,217857207	1	0	0
2/17	0,217857207	1	0	0
2/17	0,217857207	1	0	0
2/17	0,217857207	1	0	0
18/285	0,220345536	1	0	0
4/47	0,2230987	1	0	0
5/63	0,224585398	1	0	0
3/32	0,227344696	1	0	0
3/32	0,227344696	1	0	0
4/48	0,234278972	1	0	0
2/18	0,237255632	1	0	0
2/18	0,237255632	1	0	0
2/18	0,237255632	1	0	0
2/18	0,237255632	1	0	0
7/98	0,242020994	1	0	0
7/98	0,242020994	1	0	0
5/65	0,243847828	1	0	0
4/49	0,245590145	1	0	0
6/82	0,248252399	1	0	0
5/66	0,253638584	1	0	0

2/19	0,256736405	1	0	0
2/19	0,256736405	1	0	0
2/19	0,256736405	1	0	0
2/19	0,256736405	1	0	0
4/50	0,257018171	1	0	0
14/223	0,262719251	1	0	0
3/35	0,269461014	1	0	0
3/35	0,269461014	1	0	0
1/6	0,272334438	1	0	0
1/6	0,272334438	1	0	0
1/6	0,272334438	1	0	0
1/6	0,272334438	1	0	0
1/6	0,272334438	1	0	0
1/6	0,272334438	1	0	0
1/6	0,272334438	1	0	0
1/6	0,272334438	1	0	0
1/6	0,272334438	1	0	0
1/6	0,272334438	1	0	0
1/6	0,272334438	1	0	0
1/6	0,272334438	1	0	0
1/6	0,272334438	1	0	0
1/6	0,272334438	1	0	0
1/6	0,272334438	1	0	0
1/6	0,272334438	1	0	0
1/6	0,272334438	1	0	0
1/6	0,272334438	1	0	0
1/6	0,272334438	1	0	0
1/6	0,272334438	1	0	0
1/6	0,272334438	1	0	0
1/6	0,272334438	1	0	0
1/6	0,272334438	1	0	0
1/6	0,272334438	1	0	0
1/6	0,272334438	1	0	0
1/6	0,272334438	1	0	0
5/68	0,27349548	1	0	0
5/68	0,27349548	1	0	0
2/20	0,276239439	1	0	0
2/20	0,276239439	1	0	0
2/20	0,276239439	1	0	0
2/20	0,276239439	1	0	0
2/21	0,295710622	1	0	0
2/21	0,295710622	1	0	0
2/21	0,295710622	1	0	0
3/37	0,29800186	1	0	0
3/37	0,29800186	1	0	0
3/37	0,29800186	1	0	0
3/37	0,29800186	1	0	0
7/105	0,298389432	1	0	0
6/88	0,301696743	1	0	0
1/7	0,309893194	1	0	0
1/7	0,309893194	1	0	0
1/7	0,309893194	1	0	0
1/7	0,309893194	1	0	0

1/7	0,309893194	1	0	0
1/7	0,309893194	1	0	0
1/7	0,309893194	1	0	0
1/7	0,309893194	1	0	0
1/7	0,309893194	1	0	0
1/7	0,309893194	1	0	0
1/7	0,309893194	1	0	0
1/7	0,309893194	1	0	0
1/7	0,309893194	1	0	0
1/7	0,309893194	1	0	0
1/7	0,309893194	1	0	0
1/7	0,309893194	1	0	0
1/7	0,309893194	1	0	0
1/7	0,309893194	1	0	0
1/7	0,309893194	1	0	0
1/7	0,309893194	1	0	0
1/7	0,309893194	1	0	0
1/7	0,309893194	1	0	0
1/7	0,309893194	1	0	0
1/7	0,309893194	1	0	0
1/7	0,309893194	1	0	0
3/38	0,312340461	1	0	0
2/22	0,315101353	1	0	0
2/22	0,315101353	1	0	0
2/22	0,315101353	1	0	0
2/22	0,315101353	1	0	0
2/22	0,315101353	1	0	0
4/55	0,315434093	1	0	0
7/108	0,323374032	1	0	0
3/39	0,326695464	1	0	0
3/39	0,326695464	1	0	0
3/39	0,326695464	1	0	0
4/56	0,327280741	1	0	0
2/23	0,334368124	1	0	0
2/23	0,334368124	1	0	0
4/57	0,339152763	1	0	0
4/57	0,339152763	1	0	0
8/128	0,34080738	1	0	0
3/40	0,341046404	1	0	0
3/40	0,341046404	1	0	0
1/8	0,345515118	1	0	0
1/8	0,345515118	1	0	0
1/8	0,345515118	1	0	0
1/8	0,345515118	1	0	0
1/8	0,345515118	1	0	0
1/8	0,345515118	1	0	0
1/8	0,345515118	1	0	0
1/8	0,345515118	1	0	0
1/8	0,345515118	1	0	0
1/8	0,345515118	1	0	0
1/8	0,345515118	1	0	0
1/8	0,345515118	1	0	0
1/8	0,345515118	1	0	0

1/8	0,345515118	1	0	0
1/8	0,345515118	1	0	0
1/8	0,345515118	1	0	0
1/8	0,345515118	1	0	0
1/8	0,345515118	1	0	0
1/8	0,345515118	1	0	0
1/8	0,345515118	1	0	0
1/8	0,345515118	1	0	0
1/8	0,345515118	1	0	0
1/8	0,345515118	1	0	0
9/148	0,355292848	1	0	0
3/41	0,355373883	1	0	0
18/314	0,356967482	1	0	0
6/94	0,356977271	1	0	0
11/185	0,358519476	1	0	0
12/204	0,362531681	1	0	0
5/77	0,365709148	1	0	0
6/95	0,366280435	1	0	0
3/42	0,369659561	1	0	0
2/25	0,372378865	1	0	0
2/25	0,372378865	1	0	0
2/25	0,372378865	1	0	0
5/78	0,3760808	1	0	0
1/9	0,379299997	1	0	0
1/9	0,379299997	1	0	0
1/9	0,379299997	1	0	0
1/9	0,379299997	1	0	0
1/9	0,379299997	1	0	0
1/9	0,379299997	1	0	0
1/9	0,379299997	1	0	0
1/9	0,379299997	1	0	0
1/9	0,379299997	1	0	0
1/9	0,379299997	1	0	0
1/9	0,379299997	1	0	0
1/9	0,379299997	1	0	0
1/9	0,379299997	1	0	0
1/9	0,379299997	1	0	0
1/9	0,379299997	1	0	0
1/9	0,379299997	1	0	0
1/9	0,379299997	1	0	0
2/26	0,39105785	1	0	0
2/26	0,39105785	1	0	0
2/26	0,39105785	1	0	0
2/26	0,39105785	1	0	0
17/303	0,396534729	1	0	0
3/44	0,398037404	1	0	0
2/27	0,409482246	1	0	0
2/27	0,409482246	1	0	0
4/63	0,410285043	1	0	0
15/268	0,410745544	1	0	0
1/10	0,411342481	1	0	0
1/10	0,411342481	1	0	0
1/10	0,411342481	1	0	0

1/10	0,411342481	1	0	0
1/10	0,411342481	1	0	0
1/10	0,411342481	1	0	0
1/10	0,411342481	1	0	0
1/10	0,411342481	1	0	0
1/10	0,411342481	1	0	0
1/10	0,411342481	1	0	0
1/10	0,411342481	1	0	0
1/10	0,411342481	1	0	0
1/10	0,411342481	1	0	0
1/10	0,411342481	1	0	0
1/10	0,411342481	1	0	0
1/10	0,411342481	1	0	0
1/10	0,411342481	1	0	0
1/10	0,411342481	1	0	0
1/10	0,411342481	1	0	0
1/10	0,411342481	1	0	0
1/10	0,411342481	1	0	0
1/10	0,411342481	1	0	0
1/10	0,411342481	1	0	0
1/10	0,411342481	1	0	0
1/10	0,411342481	1	0	0
14/250	0,415530985	1	0	0
3/46	0,426053988	1	0	0
6/103	0,440649705	1	0	0
1/11	0,441732347	1	0	0
1/11	0,441732347	1	0	0
1/11	0,441732347	1	0	0
1/11	0,441732347	1	0	0
1/11	0,441732347	1	0	0
1/11	0,441732347	1	0	0
1/11	0,441732347	1	0	0
1/11	0,441732347	1	0	0
1/11	0,441732347	1	0	0
1/11	0,441732347	1	0	0
1/11	0,441732347	1	0	0
1/11	0,441732347	1	0	0
1/11	0,441732347	1	0	0
1/11	0,441732347	1	0	0
14/255	0,444834927	1	0	0
4/66	0,44532807	1	0	0
2/29	0,445476512	1	0	0
2/29	0,445476512	1	0	0
2/29	0,445476512	1	0	0
2/29	0,445476512	1	0	0
2/29	0,445476512	1	0	0
3/48	0,453599363	1	0	0
3/48	0,453599363	1	0	0
15/276	0,455954716	1	0	0
4/67	0,456871178	1	0	0
5/86	0,458376644	1	0	0
2/30	0,463008482	1	0	0
2/30	0,463008482	1	0	0

2/30	0,463008482	1	0	0
3/49	0,467165176	1	0	0
1/12	0,470554753	1	0	0
1/12	0,470554753	1	0	0
1/12	0,470554753	1	0	0
1/12	0,470554753	1	0	0
1/12	0,470554753	1	0	0
1/12	0,470554753	1	0	0
1/12	0,470554753	1	0	0
1/12	0,470554753	1	0	0
1/12	0,470554753	1	0	0
1/12	0,470554753	1	0	0
1/12	0,470554753	1	0	0
4/69	0,479703714	1	0	0
2/31	0,480209569	1	0	0
2/31	0,480209569	1	0	0
3/50	0,480578809	1	0	0
3/50	0,480578809	1	0	0
3/51	0,493830654	1	0	0
2/32	0,497067219	1	0	0
2/32	0,497067219	1	0	0
2/32	0,497067219	1	0	0
1/13	0,497890471	1	0	0
1/13	0,497890471	1	0	0
1/13	0,497890471	1	0	0
1/13	0,497890471	1	0	0
1/13	0,497890471	1	0	0
1/13	0,497890471	1	0	0
1/13	0,497890471	1	0	0
1/13	0,497890471	1	0	0
1/13	0,497890471	1	0	0
3/52	0,50691194	1	0	0
3/52	0,50691194	1	0	0
2/33	0,513571053	1	0	0
2/33	0,513571053	1	0	0
7/131	0,51735239	1	0	0
3/53	0,5198147	1	0	0
1/14	0,523816115	1	0	0
1/14	0,523816115	1	0	0
1/14	0,523816115	1	0	0
1/14	0,523816115	1	0	0
1/14	0,523816115	1	0	0
1/14	0,523816115	1	0	0
1/14	0,523816115	1	0	0
1/14	0,523816115	1	0	0
1/14	0,523816115	1	0	0
4/73	0,52416431	1	0	0
4/73	0,52416431	1	0	0
5/93	0,527706009	1	0	0
6/113	0,530783321	1	0	0
5/94	0,537297506	1	0	0
7/134	0,541567612	1	0	0
2/35	0,545485494	1	0	0

5/95	0,546798449	1	0	0
1/15	0,548404355	1	0	0
1/15	0,548404355	1	0	0
1/15	0,548404355	1	0	0
1/15	0,548404355	1	0	0
1/15	0,548404355	1	0	0
1/15	0,548404355	1	0	0
1/15	0,548404355	1	0	0
1/15	0,548404355	1	0	0
1/15	0,548404355	1	0	0
2/36	0,560884561	1	0	0
2/36	0,560884561	1	0	0
3/57	0,569507444	1	0	0
1/16	0,571724118	1	0	0
1/16	0,571724118	1	0	0
1/16	0,571724118	1	0	0
1/16	0,571724118	1	0	0
1/16	0,571724118	1	0	0
1/16	0,571724118	1	0	0
1/16	0,571724118	1	0	0
1/16	0,571724118	1	0	0
1/16	0,571724118	1	0	0
4/79	0,587183851	1	0	0
2/38	0,590548963	1	0	0
20/400	0,590649368	1	0	0
1/17	0,593840785	1	0	0
1/17	0,593840785	1	0	0
1/17	0,593840785	1	0	0
1/17	0,593840785	1	0	0
1/17	0,593840785	1	0	0
1/17	0,593840785	1	0	0
1/17	0,593840785	1	0	0
3/60	0,604619888	1	0	0
3/60	0,604619888	1	0	0
2/39	0,604811304	1	0	0
1/18	0,614816365	1	0	0
1/18	0,614816365	1	0	0
1/18	0,614816365	1	0	0
1/18	0,614816365	1	0	0
1/18	0,614816365	1	0	0
1/18	0,614816365	1	0	0
1/18	0,614816365	1	0	0
1/18	0,614816365	1	0	0
1/18	0,614816365	1	0	0
3/61	0,615893424	1	0	0
4/82	0,616800048	1	0	0
2/40	0,618693656	1	0	0
9/186	0,62615138	1	0	0
3/62	0,626947593	1	0	0
5/104	0,627712366	1	0	0
2/41	0,632197223	1	0	0
2/41	0,632197223	1	0	0
1/19	0,63470968	1	0	0

1/19	0,63470968	1	0	0
1/19	0,63470968	1	0	0
1/19	0,63470968	1	0	0
1/19	0,63470968	1	0	0
1/19	0,63470968	1	0	0
1/19	0,63470968	1	0	0
1/19	0,63470968	1	0	0
1/19	0,63470968	1	0	0
1/19	0,63470968	1	0	0
1/19	0,63470968	1	0	0
3/63	0,637780712	1	0	0
3/63	0,637780712	1	0	0
2/42	0,645324095	1	0	0
3/64	0,648391557	1	0	0
1/20	0,653576518	1	0	0
1/20	0,653576518	1	0	0
1/20	0,653576518	1	0	0
4/86	0,654159206	1	0	0
4/86	0,654159206	1	0	0
2/43	0,658077154	1	0	0
2/43	0,658077154	1	0	0
2/44	0,670459987	1	0	0
2/44	0,670459987	1	0	0
1/21	0,671469795	1	0	0
1/21	0,671469795	1	0	0
1/21	0,671469795	1	0	0
1/21	0,671469795	1	0	0
1/21	0,671469795	1	0	0
1/21	0,671469795	1	0	0
3/67	0,678884566	1	0	0
4/89	0,680526583	1	0	0
1/22	0,688439703	1	0	0
1/22	0,688439703	1	0	0
1/22	0,688439703	1	0	0
1/22	0,688439703	1	0	0
1/22	0,688439703	1	0	0
3/68	0,688602361	1	0	0
3/68	0,688602361	1	0	0
3/68	0,688602361	1	0	0
2/46	0,694132378	1	0	0
2/46	0,694132378	1	0	0
2/46	0,694132378	1	0	0
3/69	0,698097744	1	0	0
1/23	0,704533848	1	0	0
1/23	0,704533848	1	0	0
1/23	0,704533848	1	0	0
2/47	0,705431946	1	0	0
3/70	0,707371709	1	0	0
6/138	0,722280216	1	0	0
2/49	0,7269861	1	0	0
2/49	0,7269861	1	0	0
2/49	0,7269861	1	0	0
1/25	0,734273137	1	0	0

1/25	0,734273137	1	0	0
1/25	0,734273137	1	0	0
1/25	0,734273137	1	0	0
1/25	0,734273137	1	0	0
1/25	0,734273137	1	0	0
1/25	0,734273137	1	0	0
1/25	0,734273137	1	0	0
1/25	0,734273137	1	0	0
3/74	0,742282769	1	0	0
16/355	0,746420559	1	0	0
1/26	0,74800173	1	0	0
1/26	0,74800173	1	0	0
1/26	0,74800173	1	0	0
1/26	0,74800173	1	0	0
3/76	0,758454597	1	0	0
1/27	0,761021692	1	0	0
1/27	0,761021692	1	0	0
4/101	0,771651031	1	0	0
4/101	0,771651031	1	0	0
1/28	0,773369566	1	0	0
1/28	0,773369566	1	0	0
1/28	0,773369566	1	0	0
1/28	0,773369566	1	0	0
6/147	0,77601399	1	0	0
2/55	0,783756324	1	0	0
1/29	0,785080013	1	0	0
1/29	0,785080013	1	0	0
1/30	0,796185909	1	0	0
2/57	0,800236223	1	0	0
2/57	0,800236223	1	0	0
1/31	0,806718435	1	0	0
1/31	0,806718435	1	0	0
1/32	0,816707165	1	0	0
1/32	0,816707165	1	0	0
1/33	0,826180149	1	0	0
1/33	0,826180149	1	0	0
3/86	0,827332306	1	0	0
2/61	0,829882961	1	0	0
1/34	0,835163993	1	0	0
1/34	0,835163993	1	0	0
1/34	0,835163993	1	0	0
1/34	0,835163993	1	0	0
1/34	0,835163993	1	0	0
1/35	0,84368393	1	0	0
1/35	0,84368393	1	0	0
1/35	0,84368393	1	0	0
1/35	0,84368393	1	0	0
1/35	0,84368393	1	0	0
2/64	0,849450754	1	0	0
1/36	0,851763893	1	0	0
2/65	0,855503801	1	0	0
1/39	0,873585143	1	0	0
2/70	0,882558929	1	0	0

1/41	0,886318873	1	0	0
2/71	0,887374376	1	0	0
2/72	0,89200618	1	0	0
1/42	0,892196776	1	0	0
1/42	0,892196776	1	0	0
2/73	0,896460449	1	0	0
2/74	0,900743148	1	0	0
1/44	0,903057318	1	0	0
1/44	0,903057318	1	0	0
1/44	0,903057318	1	0	0
1/44	0,903057318	1	0	0
1/49	0,925663348	1	0	0
2/81	0,926389233	1	0	0
1/50	0,929508438	1	0	0
1/52	0,93661274	1	0	0
3/119	0,948819231	1	0	0
2/101	0,969470187	1	0	0
1/84	0,988432409	1	0	0
2/156	0,997616537	1	0	0
2/197	0,999667576	1	0	0
2/221	0,999895251	1	0	0

Odds.Ratio	Combined.Score
2,590888364	29,2980171
2,190187958	23,62353424
2,510342612	27,00906981
2,015503876	21,24670369
2,42248062	22,05950491
2,083854297	18,8097201
1,854530618	16,55883341
3,73997008	33,30537957
2,069022594	18,41177956
2,7004702	23,34905293
1,765625788	14,54536488
1,971984224	15,45775473
2,76854928	21,07740703
1,989565833	14,9829377
6,45994832	46,88590967
4,110876204	27,47391728
1,851998836	12,30372419
1,354355159	8,975408741
1,981050818	12,99489225
2,733055059	16,80663008
9,689922481	58,30911925
1,668138554	9,983299617
2,808673183	16,58354675
3,22997416	18,83741371
3,22997416	18,83741371
5,963029219	33,78303964
1,918796531	10,57420741
2,507979936	13,71028347
4,404510218	23,72962798
5,53709856	29,73555012
2,583979328	13,67070724
2,725290698	14,25012536
5,167958656	26,36331328
7,26744186	36,81572007
1,926651253	9,464260083
2,292239727	10,97540989
1,937984496	9,204043217
1,937984496	9,204043217
1,80498556	8,41304993
1,964173476	9,12364792
4,55996352	21,10141337
2,82622739	12,73466308
1,62582592	7,214124856
1,830318691	8,101259733
4,306632214	19,02322652
5,813953488	25,4722545
1,631986944	7,101251243
2,131782946	9,099738717
1,513287642	6,395815888
4,07996736	17,22434882
2,65997872	11,13464158
2,906976744	12,04066138

5,285412262	21,67743134
5,285412262	21,67743134
5,285412262	21,67743134
2,608825283	10,65848654
2,265176684	9,179018154
3,875968992	15,65636772
3,875968992	15,65636772
3,22997416	13,04612629
2,836074872	11,42110301
3,125781445	12,20740068
4,84496124	18,66362223
2,42248062	8,991002767
3,523608175	13,0680136
3,523608175	13,0680136
3,523608175	13,0680136
1,83896339	6,789674235
4,472271914	16,22619769
4,15282392	14,22455135
4,15282392	14,22455135
4,15282392	14,22455135
6,45994832	21,70645267
6,45994832	21,70645267
6,45994832	21,70645267
6,45994832	21,70645267
6,45994832	21,70645267
6,45994832	21,70645267
2,691645134	8,931959959
1,982029598	6,507035447
3,100775194	10,1777513
1,367529382	4,466477662
3,875968992	12,55918396
3,875968992	12,55918396
2,188047012	7,05084507
2,981514609	9,408299464
2,013490386	6,262292079
2,119670543	6,52001306
3,63372093	11,15790341
5,53709856	16,93240671
5,53709856	16,93240671
5,53709856	16,93240671
5,53709856	16,93240671
5,53709856	16,93240671
5,53709856	16,93240671
2,087060227	6,27265751
1,875468867	5,599735958
2,055438102	6,036443209
3,41997264	9,967191743
3,41997264	9,967191743
4,84496124	13,58747994
4,84496124	13,58747994
4,84496124	13,58747994
4,84496124	13,58747994
4,84496124	13,58747994

3,22997416	8,946625228
2,3071244	6,354207732
1,665455426	4,503278339
1,365517531	3,684453245
2,253470344	6,02101599
1,443988448	3,847152398
3,05997552	8,065164774
3,05997552	8,065164774
4,306632214	11,14179549
4,306632214	11,14179549
4,306632214	11,14179549
4,306632214	11,14179549
4,306632214	11,14179549
4,306632214	11,14179549
1,21395002	3,120047251
1,312404851	3,337409424
2,153316107	5,417548418
2,906976744	7,298627913
2,906976744	7,298627913
2,906976744	7,298627913
1,833228577	4,481786064
2,106504887	5,143960963
2,349072117	5,703493121
1,38427464	3,347702757
3,875968992	9,293806274
3,875968992	9,293806274
3,875968992	9,293806274
3,875968992	9,293806274
3,875968992	9,293806274
3,875968992	9,293806274
3,875968992	9,293806274
3,875968992	9,293806274
3,875968992	9,293806274
3,875968992	9,293806274
3,875968992	9,293806274
3,875968992	9,293806274
2,76854928	6,627928123
2,76854928	6,627928123
2,76854928	6,627928123
2,76854928	6,627928123
2,76854928	6,627928123
1,379348396	3,298729473
1,38427464	3,251010693
2,27998176	5,340500606
1,295073172	3,008890949
1,875468867	4,327695658
2,642706131	6,037824525
2,642706131	6,037824525
2,642706131	6,037824525
1,84569952	4,15171171
1,9775352	4,419123035
1,9775352	4,419123035
3,523608175	7,860423421

3,523608175	7,860423421
3,523608175	7,860423421
3,523608175	7,860423421
2,527805865	5,516018479
2,527805865	5,516018479
2,527805865	5,516018479
2,527805865	5,516018479
2,527805865	5,516018479
1,362645349	2,957817688
1,788908766	3,824347528
1,761804087	3,672037086
3,22997416	6,724672735
3,22997416	6,724672735
3,22997416	6,724672735
3,22997416	6,724672735
3,22997416	6,724672735
3,22997416	6,724672735
3,22997416	6,724672735
1,863446631	3,814717371
1,863446631	3,814717371
1,194847584	2,384574025
2,325581395	4,639006775
2,325581395	4,639006775
2,325581395	4,639006775
2,325581395	4,639006775
1,82828726	3,635692848
1,82828726	3,635692848
1,38427464	2,750968353
2,981514609	5,80867905
2,981514609	5,80867905
2,981514609	5,80867905
2,981514609	5,80867905
2,981514609	5,80867905
2,981514609	5,80867905
1,68520391	3,25566162
1,794430089	3,466608554
1,937984496	3,68046246
1,937984496	3,68046246
1,535037225	2,907692919
1,23119015	2,292329331
1,206527313	2,233033851
2,153316107	3,936019159
2,153316107	3,936019159
2,153316107	3,936019159
2,153316107	3,936019159
2,76854928	5,058804027
2,76854928	5,058804027
2,76854928	5,058804027
2,76854928	5,058804027
2,76854928	5,058804027
2,76854928	5,058804027
2,76854928	5,058804027
2,76854928	5,058804027

1,7303433	3,155624087
1,505230677	2,735778823
1,61498708	2,892730146
1,84569952	3,274194731
1,6999864	3,012538338
1,286942829	2,258462711
2,07641196	3,636043216
2,07641196	3,636043216
2,07641196	3,636043216
2,583979328	4,437032068
2,583979328	4,437032068
2,583979328	4,437032068
2,583979328	4,437032068
2,583979328	4,437032068
1,802776275	3,091681929
2,004811548	3,364777581
1,761804087	2,921385574
1,761804087	2,921385574
1,61498708	2,626744377
1,61498708	2,626744377
2,42248062	3,915755568
2,42248062	3,915755568
2,42248062	3,915755568
2,42248062	3,915755568
1,937984496	3,118786362
1,937984496	3,118786362
1,588511882	2,511148135
1,510117789	2,38541536
1,875468867	2,895132655
1,875468867	2,895132655
2,27998176	3,474499418
2,27998176	3,474499418
2,27998176	3,474499418
2,27998176	3,474499418
2,27998176	3,474499418
2,27998176	3,474499418
2,27998176	3,474499418
1,223990208	1,851356608
1,649348507	2,474255328
1,538082933	2,297125709
1,816860465	2,691293478
1,816860465	2,691293478
1,61498708	2,343738185
2,153316107	3,097797377
2,153316107	3,097797377
2,153316107	3,097797377
2,153316107	3,097797377
1,38427464	1,963913075
1,38427464	1,963913075
1,490757305	2,103772963
1,58202816	2,221311833
1,418037436	1,975764768
1,468170073	2,014101658

2,03998368	2,773776792
2,03998368	2,773776792
2,03998368	2,773776792
2,03998368	2,773776792
1,550387597	2,106369756
1,216671881	1,626287956
1,661129568	2,178291625
1,661129568	2,178291625
3,22997416	4,201306255
3,22997416	4,201306255
3,22997416	4,201306255
3,22997416	4,201306255
3,22997416	4,201306255
3,22997416	4,201306255
3,22997416	4,201306255
3,22997416	4,201306255
3,22997416	4,201306255
3,22997416	4,201306255
3,22997416	4,201306255
3,22997416	4,201306255
3,22997416	4,201306255
3,22997416	4,201306255
3,22997416	4,201306255
3,22997416	4,201306255
3,22997416	4,201306255
3,22997416	4,201306255
3,22997416	4,201306255
3,22997416	4,201306255
3,22997416	4,201306255
3,22997416	4,201306255
3,22997416	4,201306255
3,22997416	4,201306255
3,22997416	4,201306255
3,22997416	4,201306255
1,4249886	1,847455232
1,4249886	1,847455232
1,937984496	2,493192356
1,937984496	2,493192356
1,937984496	2,493192356
1,937984496	2,493192356
1,84569952	2,248752181
1,84569952	2,248752181
1,84569952	2,248752181
1,571338781	1,902350018
1,571338781	1,902350018
1,571338781	1,902350018
1,571338781	1,902350018
1,291989664	1,562475228
1,321353066	1,583420887
2,76854928	3,243431827
2,76854928	3,243431827
2,76854928	3,243431827
2,76854928	3,243431827

2,76854928	3,243431827
2,76854928	3,243431827
2,76854928	3,243431827
2,76854928	3,243431827
2,76854928	3,243431827
2,76854928	3,243431827
2,76854928	3,243431827
2,76854928	3,243431827
2,76854928	3,243431827
2,76854928	3,243431827
2,76854928	3,243431827
2,76854928	3,243431827
2,76854928	3,243431827
2,76854928	3,243431827
2,76854928	3,243431827
2,76854928	3,243431827
2,76854928	3,243431827
2,76854928	3,243431827
2,76854928	3,243431827
2,76854928	3,243431827
2,76854928	3,243431827
1,52998776	1,780387798
1,761804087	2,034638719
1,761804087	2,034638719
1,761804087	2,034638719
1,761804087	2,034638719
1,761804087	2,034638719
1,40944327	1,62622342
1,256101062	1,418069809
1,490757305	1,667750218
1,490757305	1,667750218
1,490757305	1,667750218
1,38427464	1,546147481
1,68520391	1,846162327
1,68520391	1,846162327
1,35998912	1,470562551
1,35998912	1,470562551
1,21124031	1,303824891
1,453488372	1,563570827
1,453488372	1,563570827
2,42248062	2,574415889
2,42248062	2,574415889
2,42248062	2,574415889
2,42248062	2,574415889
2,42248062	2,574415889
2,42248062	2,574415889
2,42248062	2,574415889
2,42248062	2,574415889
2,42248062	2,574415889
2,42248062	2,574415889
2,42248062	2,574415889
2,42248062	2,574415889
2,42248062	2,574415889

2,42248062	2,574415889
2,42248062	2,574415889
2,42248062	2,574415889
2,42248062	2,574415889
2,42248062	2,574415889
2,42248062	2,574415889
2,42248062	2,574415889
2,42248062	2,574415889
2,42248062	2,574415889
2,42248062	2,574415889
1,178504085	1,219531236
1,418037436	1,467080051
1,110946526	1,14439778
1,237011381	1,274224598
1,152315106	1,18201291
1,13999088	1,156684238
1,258431491	1,265877555
1,223990208	1,229321937
1,38427464	1,377592472
1,550387597	1,531540293
1,550387597	1,531540293
1,550387597	1,531540293
1,242297754	1,214906659
2,153316107	2,087484578
2,153316107	2,087484578
2,153316107	2,087484578
2,153316107	2,087484578
2,153316107	2,087484578
2,153316107	2,087484578
2,153316107	2,087484578
2,153316107	2,087484578
2,153316107	2,087484578
2,153316107	2,087484578
2,153316107	2,087484578
2,153316107	2,087484578
2,153316107	2,087484578
2,153316107	2,087484578
2,153316107	2,087484578
2,153316107	2,087484578
2,153316107	2,087484578
1,490757305	1,399671698
1,490757305	1,399671698
1,490757305	1,399671698
1,490757305	1,399671698
1,087318034	1,005760106
1,321353066	1,217242729
1,435544071	1,281742365
1,435544071	1,281742365
1,230466347	1,096226325
1,084692815	0,965139461
1,937984496	1,721568072
1,937984496	1,721568072
1,937984496	1,721568072

1,937984496	1,721568072
1,937984496	1,721568072
1,937984496	1,721568072
1,937984496	1,721568072
1,937984496	1,721568072
1,937984496	1,721568072
1,937984496	1,721568072
1,937984496	1,721568072
1,937984496	1,721568072
1,937984496	1,721568072
1,937984496	1,721568072
1,937984496	1,721568072
1,937984496	1,721568072
1,937984496	1,721568072
1,937984496	1,721568072
1,937984496	1,721568072
1,937984496	1,721568072
1,937984496	1,721568072
1,937984496	1,721568072
1,937984496	1,721568072
1,937984496	1,721568072
1,937984496	1,721568072
1,085271318	0,953083202
1,263902932	1,078348343
1,128923007	0,925158094
1,761804087	1,43948402
1,761804087	1,43948402
1,761804087	1,43948402
1,761804087	1,43948402
1,761804087	1,43948402
1,761804087	1,43948402
1,761804087	1,43948402
1,761804087	1,43948402
1,761804087	1,43948402
1,761804087	1,43948402
1,761804087	1,43948402
1,761804087	1,43948402
1,761804087	1,43948402
1,761804087	1,43948402
1,063991488	0,861888451
1,174536058	0,950133936
1,336541032	1,080741454
1,336541032	1,080741454
1,336541032	1,080741454
1,336541032	1,080741454
1,336541032	1,080741454
1,21124031	0,957535043
1,21124031	0,957535043
1,053252444	0,827184216
1,157005669	0,906344803
1,126735172	0,878925619
1,291989664	0,994844838
1,291989664	0,994844838

1,291989664	0,994844838
1,18652112	0,903028463
1,61498708	1,217446632
1,61498708	1,217446632
1,61498708	1,217446632
1,61498708	1,217446632
1,61498708	1,217446632
1,61498708	1,217446632
1,61498708	1,217446632
1,61498708	1,217446632
1,61498708	1,217446632
1,61498708	1,217446632
1,61498708	1,217446632
1,123469273	0,825285506
1,250312578	0,917145122
1,250312578	0,917145122
1,162790698	0,852051221
1,162790698	0,852051221
1,13999088	0,804334959
1,21124031	0,84669333
1,21124031	0,84669333
1,21124031	0,84669333
1,490757305	1,03961712
1,490757305	1,03961712
1,490757305	1,03961712
1,490757305	1,03961712
1,490757305	1,03961712
1,490757305	1,03961712
1,490757305	1,03961712
1,490757305	1,03961712
1,490757305	1,03961712
1,118067979	0,759635486
1,118067979	0,759635486
1,174536058	0,782671939
1,174536058	0,782671939
1,035564235	0,682468966
1,096972356	0,71773023
1,38427464	0,895092168
1,38427464	0,895092168
1,38427464	0,895092168
1,38427464	0,895092168
1,38427464	0,895092168
1,38427464	0,895092168
1,38427464	0,895092168
1,38427464	0,895092168
1,38427464	0,895092168
1,061909313	0,685940401
1,061909313	0,685940401
1,041927148	0,666016454
1,029018317	0,651781641
1,030842817	0,640362983
1,012379961	0,620879833
1,107419712	0,671183906

1,01999184	0,615743584
1,291989664	0,776152959
1,291989664	0,776152959
1,291989664	0,776152959
1,291989664	0,776152959
1,291989664	0,776152959
1,291989664	0,776152959
1,291989664	0,776152959
1,291989664	0,776152959
1,291989664	0,776152959
1,076658053	0,622566934
1,076658053	0,622566934
1,01999184	0,5742385
1,21124031	0,6772029
1,21124031	0,6772029
1,21124031	0,6772029
1,21124031	0,6772029
1,21124031	0,6772029
1,21124031	0,6772029
1,21124031	0,6772029
1,21124031	0,6772029
1,21124031	0,6772029
0,981257973	0,522438724
1,01999184	0,537232486
0,968992248	0,510206127
1,13999088	0,594099447
1,13999088	0,594099447
1,13999088	0,594099447
1,13999088	0,594099447
1,13999088	0,594099447
1,13999088	0,594099447
1,13999088	0,594099447
0,968992248	0,487553588
0,968992248	0,487553588
0,993838203	0,499740374
1,076658053	0,523720552
1,076658053	0,523720552
1,076658053	0,523720552
1,076658053	0,523720552
1,076658053	0,523720552
1,076658053	0,523720552
1,076658053	0,523720552
1,076658053	0,523720552
1,076658053	0,523720552
0,953107129	0,461953244
0,945358291	0,456806939
0,968992248	0,465256812
0,937734434	0,439012674
0,937734434	0,43782101
0,931723315	0,433878609
0,945358291	0,433497705
0,945358291	0,433497705
1,01999184	0,463675624

1,01999184	0,463675624
1,01999184	0,463675624
1,01999184	0,463675624
1,01999184	0,463675624
1,01999184	0,463675624
1,01999184	0,463675624
1,01999184	0,463675624
1,01999184	0,463675624
1,01999184	0,463675624
1,01999184	0,463675624
0,92284976	0,415061615
0,92284976	0,415061615
0,92284976	0,404210609
0,908430233	0,393586946
0,968992248	0,412108201
0,968992248	0,412108201
0,968992248	0,412108201
0,901388138	0,382553202
0,901388138	0,382553202
0,901388138	0,377170632
0,901388138	0,377170632
0,880902044	0,352176933
0,880902044	0,352176933
0,92284976	0,367558366
0,92284976	0,367558366
0,92284976	0,367558366
0,92284976	0,367558366
0,92284976	0,367558366
0,92284976	0,367558366
0,867754252	0,336084841
0,871004268	0,335239434
0,880902044	0,328864994
0,880902044	0,328864994
0,880902044	0,328864994
0,880902044	0,328864994
0,880902044	0,328864994
0,85499316	0,318990509
0,85499316	0,318990509
0,85499316	0,318990509
0,842601955	0,30762773
0,842601955	0,30762773
0,842601955	0,30762773
0,842601955	0,3028279
0,842601955	0,295095132
0,842601955	0,295095132
0,842601955	0,295095132
0,824674254	0,287765937
0,830564784	0,287540694
0,842601955	0,274133894
0,79101408	0,252213195
0,79101408	0,252213195
0,79101408	0,252213195
0,775193798	0,239437363

0,775193798	0,239437363
0,775193798	0,239437363
0,775193798	0,239437363
0,775193798	0,239437363
0,775193798	0,239437363
0,775193798	0,239437363
0,775193798	0,239437363
0,775193798	0,239437363
0,78566939	0,234149134
0,873457801	0,255456784
0,745378652	0,216420683
0,745378652	0,216420683
0,745378652	0,216420683
0,745378652	0,216420683
0,76499388	0,211499649
0,717772036	0,196018818
0,717772036	0,196018818
0,767518612	0,198958372
0,767518612	0,198958372
0,69213732	0,177878081
0,69213732	0,177878081
0,69213732	0,177878081
0,69213732	0,177878081
0,79101408	0,200589093
0,704721635	0,171710442
0,668270516	0,161701175
0,668270516	0,161701175
0,645994832	0,1472368
0,67999456	0,151535643
0,67999456	0,151535643
0,625156289	0,134271427
0,625156289	0,134271427
0,605620155	0,122622744
0,605620155	0,122622744
0,587268029	0,112134385
0,587268029	0,112134385
0,676041103	0,128142809
0,635404753	0,118484305
0,56999544	0,102671668
0,56999544	0,102671668
0,56999544	0,102671668
0,56999544	0,102671668
0,56999544	0,102671668
0,553709856	0,094118131
0,553709856	0,094118131
0,553709856	0,094118131
0,553709856	0,094118131
0,553709856	0,094118131
0,605620155	0,0988162
0,538329027	0,086372691
0,596302922	0,093061862
0,496919102	0,067158458
0,553709856	0,069174816

0,472679145	0,057042206
0,545911126	0,065230001
0,538329027	0,061521435
0,46142488	0,052634076
0,46142488	0,052634076
0,530954656	0,05803393
0,523779594	0,054753371
0,440451022	0,044912461
0,440451022	0,044912461
0,440451022	0,044912461
0,440451022	0,044912461
0,39550704	0,030550809
0,47851469	0,036587613
0,387596899	0,028333098
0,372689326	0,024405702
0,48856752	0,025667863
0,383759306	0,011898671
0,23071244	0,002684343
0,248459551	0,000592901
0,196749695	6,54152E-05
0,175383212	1,83722E-05

Genes

CUL3;RNF180;LTN1;XIAP;UBE2J1;ZNRF2;ZNRF3;RNF217;LONRF3;BTRC;RNF111;SMURF2;MSL2;RC3H1;
TCERG1;MAML1;DTX1;RORB;MED12L;MTDH;MED17;ING4;NPAT;MED14;RXRA;PRDM16;RUVBL1;NRIP1;
CUL3;RNF180;LTN1;XIAP;UBE2J1;ZNRF2;ZNRF3;RNF217;LONRF3;BTRC;RNF111;SMURF2;MSL2;RC3H1;
HDAC5;GATA6;HNRNPU;SIX1;RORB;IKZF3;ELK4;PURA;RXRA;SIN3A;RBBP5;ERBB4;SALL4;TRIM24;E2F1
MEF2A;KLF10;BCL11B;ONECUT2;PLAG1;EBF1;NFATC3;TFEB;FOXJ2;SIX1;ESR1;LITAF;PHOX2B;ELK4;HN
ONECUT2;PLAG1;SIX1;LITAF;ELK4;NKRF;NSD1;ZNF148;TEAD1;MEF2A;KLF10;CREBBP;NFATC3;TFEB;F
CUL3;RNF180;LTN1;KLHL32;XIAP;TNFAIP3;DTX4;BACH1;UBE2J1;FBXO40;ZNRF2;ZNRF3;RNF217;TRIM2
NCOA2;MED14;RXRA;MYOD1;NSD1;NRIP1;CTNNB1;TRIP12;ACTN4;HIF1A;MED17
TSHZ3;ONECUT2;PLAG1;SIX1;BACH1;LITAF;ELK4;NKRF;ZNF148;TEAD1;MEF2A;KLF10;CREBBP;BCL11B
EOMES;MEF2A;TCERG1;HDAC5;CREBBP;GATA6;MTDH;HIPK2;CREB1;RBL1;SP1;SIN3A;ID2;TRPS1;ID4;
ONECUT2;PLAG1;GATA6;SIX1;RORB;BACH1;LITAF;ZBTB4;ELK4;PURA;RXRA;NKRF;NSD1;TRPS1;ZNF14
ONECUT2;PLAG1;GATA6;SIX1;HIF1A;LITAF;ELK4;NKRF;TRPS1;MEF2A;KLF10;EGR4;BCL11B;EBF1;NFAT
KLF10;HDAC5;HNRNPU;PROX1;ESR1;RUNX1;ELK4;KANSL1;SP1;AGO1;MYOD1;NRIP1;E2F1;NFE2L1
ONECUT2;PLAG1;SIX1;LITAF;ELK4;NKRF;NSD1;ZNF148;MEF2A;KLF10;NFATC3;TFEB;FOXJ2;TBX5;ESR1
KAT2B;CREBBP;NAA30;ESCO1;NAA35
NSD1;NRIP1;CTNNB1;STRN;ESR1;PPARGC1B;ZNF366
GATA6;RORB;ZBTB4;PURA;RXRA;SIN3A;TRPS1;NRIP1;TEAD1;MEF2A;KLF10;TFAP2B;CREBBP;CNBP;A
OTUD4;TCERG1;HNRNPU;HNRNPR;NUDT5;NUDT4;TIAL1;RAVER2;SNIP1;SNRPD3;TNS1;MBNL2;FNDC3
MEF2A;HDAC5;BCL11B;ZBTB14;XRCC5;GATA6;TFEB;ARID5B;SIX1;PROX1;IKZF3;PAX2;RUNX1;SMAD7;R
NAPA;SYT5;ABCA1;NAPB;SNPH;SNAP23;VPS54;SYTL4;RAB11A;SYT7;STXBP5L
BMPR2;SOSTDC1;BMPR1A
MYRF;HNRNPU;SIX1;SMG7;HIF1A;ZNF25;IKZF3;ZBTB4;FOXO1;PURA;RXRA;MECOM;SALL4;PRDM16;E2
EOMES;MEF2A;SDR16C5;CREBBP;RBL1;CREB1;SIN3A;PRDM16;CTNNB1;HIPK2
MEF2A;EOMES;CREBBP;CREB1;RBL1;SIN3A;CTNNB1;HIPK2
EOMES;TCERG1;SDR16C5;TFAP2B;CREBBP;SIN3A;ID2;ID4
SORL1;LRP8;LRP12;LRP6
MEF2A;TFAP2B;CNBP;GATA6;ARID5B;ACTN4;RORB;RUNX3;ZBTB4;GATAD2B;PHOX2B;RUNX1;PURA;RX
MEF2A;TCERG1;TFAP2B;MED14;CREBBP;SIN3A;CTNNB1;MED12L;PPARGC1B;MED17;HIPK2
HNRNPU;SNRPD3;HNRNPC;SMG7;DCP2
TGFBR3;BMPR2;SOSTDC1;BMPR1A
USP13;USP47;USP37;USP15;ATXN7;USP9X;USP2;USP1;TNFAIP3;SENP2
ANGEL2;SERBP1;IGF2BP1;HNRNPU;RC3H1;HNRNPC;TARDBP;ELAVL2;PUM2
SORL1;LRP8;LRP12;LRP6
EDEM3;MAN1A2;EDEM1
RALA;EIF2B2;SEPT12;ARL3;DIRAS2;RHOBTB3;TUBD1;MIEF1;RND3;GNL1;GTPBP4;RAP2C;GLUD2;RAP2
OTUD4;USP13;USP47;USP37;USP15;ATXN7;USP9X;USP2;USP1;TNFAIP3;YOD1
CREBBP;TSHZ3;ZBTB16;CNBP;NFATC3;ARID5B;PROX1;BACH1;ZBTB4;PURA;RBL1;TRPS1;E2F1;ZNF14
RALA;EIF2B2;SEPT12;ARL3;DIRAS2;RHOBTB3;TUBD1;MIEF1;RND3;GNL1;GTPBP4;RAP2C;GLUD2;RAP2
TCERG1;TFAP2B;HNRNPU;PROX1;CBFA2T2;CDYL2;HIPK2;PIAS1;HEYL;NPAT;TBL1XR1;SIN3A;C1QBP;N
RALA;EIF2B2;SEPT12;ARL3;DIRAS2;RHOBTB3;TUBD1;RND3;GNL1;GTPBP4;RAP2C;GLUD2;RAP2A;RAP
SESTD1;RAPGEF6;PITPNC1;MARK1
IGBP1;RCAN1;SET;RCAN2;PPP1R1B;CALM1;PHACTR4
USP13;RALA;CUL3;DTX1;HIF1A;FOXO1;UBE2J1;UBXN7;SCN5A;CCDC50;GPR37;FZD5;XRCC5;USP2;DIO
FYTTD1;CELF1;ANGEL2;SRSF1;HNRNPU;RC3H1;ELAVL2;PUM2;C1QBP;TRA2B;G3BP1;SERBP1;IGF2BP
PARP11;TNKS2;TIPARP;TNKS
EDEM3;MAN1A2;EDEM1
USP13;RALA;GPR37;FZD5;XRCC5;CUL3;USP2;DIO2;DTX1;YOD1;UBE2G1;BTBD9;HIF1A;FOXO1;UBE2J1;
PURA;RXRA;XRCC5;SP1;TDG;HNRNPU;RBMS1;TARDBP;AFF4;CGGBP1;AFF1
DYRK3;RALA;ARL3;HSPA4L;DIRAS2;RHOBTB3;HNRNPU;TUBD1;RND3;HSPH1;TDG;N4BP2;MYH10;MAR
LYN;EFNA3;EFNB3;CRK
MEF2A;TFAP2B;CREBBP;CREB1;CTNNB1;MED12L;HIPK2
MEF2A;TFAP2B;CREBBP;CTNNB1;MED12L;HIPK2

OTUD4;TNFAIP3;YOD1
NOS1AP;ATP2B4;SCN5A
PDE1B;PDE3B;PDE5A
RAP2C;RAP2A;RALA;RAP2B;SEPT12;ARL3;MIEF1
OTUD4;USP13;USP47;USP37;USP15;USP2;USP1;TNFAIP3;YOD1
IGBP1;STRN;SMG7;FOXO1
PDE1B;PDE3B;PDE5A;PDE7B
DCUN1D5;RNF180;RNF217;DCUN1D1;LONRF3
NCOA2;MYOD1;NRIP1;CTNNB1;ACTN4;HIF1A
CELF1;ERI1;TRA2B;CELF3;HNRNPU
GABRA1;GABRB1;GABRA3
SGIP1;DPYSL3;ADAM12;ELMO1;CRK;MYPN;RAD9A
FZD3;SFRP1;FZD5;LRP6
KAT2B;CREBBP;ESCO1;NAT8L
CREBBP;SP1;TEAD1;PAX2
PHLPP2;MTMR3;PPM1L;DUSP19;PPM1H;PTPRM;PTPN11;CTDSPL2;PPM1K;PPM1E;PTP4A1;PPP2CA;PG
RC3H1;METTL16;MYH10
TMOD3;TMOD2;LMOD3
ATP2B4;ATP2B2;ATP2B1
MED14;RXRA;MED17
TXNL1;TXNDC17
CHST7;CHST3
ADRA2B;ADRA2A
ITPK1;CALM1
CNOT6;DCP2
RXRA;PROX1
DCUN1D5;RNF180;RNF217;DCUN1D1;LONRF3
PURA;HNRNPK;SUB1;FUBP1;RPA4;HNRNPU;RBMS1;NABP1;SSBP2
KAT2B;CREBBP;ESCO1;PAFAH1B2
SHC4;HDAC5;SHC2;USP37;DCTN2;MAML1;PDE3B;GATA6;RHOBTB3;DUSP19;HIF1A;ZBTB4;RND3;CKS1E
PDE1B;PDE3B;PDE7B
CDK1;MAPK1;GTF2H1
PHLPP2;PPP2CA;MTMR3;PPM1L;PGAM5;PPM1K;PPM1E
SNX1;EFEMP1;ERBB4;EREG
NSD1;NRIP1;CTNNB1;STRN;ESR1;ZNF366;PPARGC1B;PIAS1
ABCA1;ACAP2;AGAP2;RAPGEF6;SORL1;AGFG2;APPL1
MOCS3;NUDT5;PAPSS2
YTHDC1;HNRNPC
CHST7;CHST3
TDG;NPR3
ABHD17B;ABHD17C
PTK2B;GRIN2B
GLUD2;SESN3
MEF2A;PURA;CREB1;MYOD1;ZBTB16;PROX1;HIF1A
IGBP1;MTMR3;ATP2B4;MTMR9;CTNNB1;STRN;SMG7;FOXO1;PHACTR4
NCOA2;MED14;CARM1;RORB;ACTN4;PPARGC1B;MED17
NCOA2;RXRA;PROX1
AGTR2;IL18BP;DKK3
PDE3B;APPL1
DYRK1A;S100B
DCP2;NUDT4
TXNL1;TXNDC17
ADRA2B;ADRA2A

GABRA1;GABRB1;GABRA3
ENTPD1;PRUNE2;NUDT5;DCP2;NUDT4
SRPK2;DYRK3;RAP2A;TDG;SIK3;SIK2;NUDT5;CDC42BPA;PSPH;PRTFDC1;MARK1
DYRK3;ROCK1;RPS6KA6;PTK2B;MAPK1;PIM3;MAP3K9;PRKG1;MARK1;SRPK2;MAP3K2;CSNK1G3;PRKA
PARP11;TNKS2;TNKS;TIPARP;PRTFDC1
MAP3K2;CSNK1G3;MAP2K1;DYRK3;ROCK1;CSNK1A1;ADK;DYRK1A;GTF2H1;PAPSS2;HK2;HIPK2;RPS6K
GABRA1;GABRB1;GABRA3
SNX1;IRS4;PTPN11
PURA;XRCC5
CRP;C1QBP
CALM1;PHACTR4
CALM1;PHACTR4
BMPR2;SOSTDC1
PCYT1B;CDS2
CREBZF;ONECUT2;HNRNPU;SIX1;IKZF3;AFF4;AFF1;ELK4;MECOM;SALL4;SOX6;TEAD1;MEF2A;EOMES;
CRP;SLC24A2;DYRK3;NUDT5;CDH6;CDH2;MB;PAPOLG;TDG;PCDHA4;SLIT2;PCDHA7;PSPH;SLC25A24;M
TGFBR3;USP15;INHBA;GDF6;SMAD7
CDC34;UBE2G1;UBE2J1
JPH3;JPH1;GRIN2B
ABCA1;ATP8A2;ATP11A
KLF10;TFAP2B;SP1;NRIP1;PROX1;ESR1;NFE2L1
MME;LNPEP;TRHDE;ZMPSTE24;METAP2
PURA;XRCC5;TERF2IP;SMG7
ABCA1;SRPK2;ABCA2;DYRK3;EIF2B2;HSPA4L;RHOBTB3;HNRNPU;MIEF1;CDC42BPA;RUNX3;GLUD2;HS
USP9X;YOD1
PTGFR;HPGD
GABRA1;GABRA3
MOCS3;UBA6
GATC;PFAS
GNRHR;TSHR
KANSL1;PHF20
DAG1;SORBS3
PDE3B;PDE5A
KANSL1;PHF20
CTNNB1;SMAD7
KANSL1;PHF20
PTK2B;ANO6;GRIN2B
ST6GAL2;ST8SIA3;ST6GALNAC3
GABRA1;GRID1;GABRA3
CDC34;UBE2G1;UBE2J1
GABRA1;GABRB1;GABRA3
LYN;MAP3K2;CSNK1G3;MAP2K1;DYRK3;ROCK1;CSNK1A1;ADK;DYRK1A;GTF2H1;PAPSS2;HIPK2;RPS6K
MEF2A;TFAP2B;TENM3;TYRP1;SMC1A;IKZF3;HIF1A;ADRA2A;ADD2;PPP2CA;HEYL;SNX1;RXRA;VAPA;VA
GABRA1;GABRB1;GRID1;GABRA3
SHC4;SHC2;USP37;BTG1;DCTN2;MAML1;GATA6;RHOBTB3;HIF1A;ZBTB4;RND3;CKS1B;ADD2;CCND3;SF
GABRA1;GABRB1;CLCN5;GABRA3;ANO6;SLC1A4
HPGD;ALDH2;SORD
ATP2B4;ATP2B2;ATP2B1
MEF2A;KANSL1;HIF1A
TGFBR3;FZD3;CXADR;ATP2B4;ATP2B2;ATP2B1
MECOM;RBBP5;NSD1;CARM1;PRDM16
SHC4;GRM5;SHC2;NRG3;CRK
PLXNA2;PLXNA3

SART3;METTL16
CHURC1-FNTB;FNTB
NAA30;NAA35
GLUD2;MIEF1;MYH10
GPC1;SCN5A;FGFR1
SCN5A;SCN3B;SCN1A
JPH3;JPH1;TPCN1
SCN5A;SCN3B;SCN1A
ABCA1;SRPK2;ABCA2;DYRK3;EIF2B2;HSPA4L;RHOBTB3;HNRNPU;CDC42BPA;RUNX3;HSPH1;TDG;SIK3
TFAP2B;CREB1;ZBTB16;HNRNPC;BACH1;GATAD2B
USP13;USP37;SFRP1;USP15;USP9X;USP2
SLC24A2;LETM1
TDG;RNF111
HNRNPU;ESR1
SLC24A2;TDG
MTMR3;MTMR9
CYB5R4;PAX2
CLCN5;ANO6
DENND5B;RAB3GAP2;TRAPPC8;DENND6A;GAPVD1
EPS8;IRS4;GAB1;PTPN11;CRK
TENM3;BHLHB9;HPGD;CALCOCO2;TYRP1;NPR3;NUDT5;IKZF3;ZBTB4;FBLN5;LRP6;ADD2;CDH6;SNX1;C
ZDHHC18;ZDHHC20;ZDHHC21
ZDHHC18;ZDHHC20;ZDHHC21
TFAP2B;TNFAIP8;XIAP
ATP2B4;ATP2B2;ATP2B1
CREB1;ZBTB16;HNRNPC;BACH1;GATAD2B
ING4;CBX5;RBBP5;PHF8;GLYR1
SGIP1;ARL3;HOOK3;RAB11A;REEP1;APC;VAPA;KIF5C;VAPB;KIF26B;DCX;KIF1B;MAPRE3;MAPRE2
ADK;HK2
ALAS2;GRIN2B
UBE2K;RBX1
SLC17A6;SLC25A12
ENTPD1;NUDT5
ABHD17B;ABHD17C
ERBB4;GAB1;PTPN11;ESR1;EREG;FGFR1
TCERG1;HDAC5;CBX5;SP1;EIF4E
ALAS2;GLUD2;SESN3;GRIN2B
KAT2B;CREBBP;ESCO1;EPC1
SNX1;SEPT12;SH3PXD2A;FRMPD2;WDFY3;SNX8;PITPNC1;SNX6
RAB3C;ATL3;HSPA4L;ATL2;RHOBTB3;RND3;GNA13;HSPH1;KIF5C;CARNS1;RUVBL1;KIF1B;MYH10;ABCA
DYRK3;ROCK1;RPS6KA6;EEF2K;ERBB4;TRIM24;PTK2B;MAPK1;PIM3;MAP3K9;PRKG1;MARK1;LYN;SRPK
NSD1;TRIP12;MED17
STIM2;CALM1;PRKG1
AGO1;HNRNPU;CDC73
TCERG1;TFAP2B;SIN3A
MAPK10;MAPK1
MTMR3;MTMR9
FRMPD2;WDFY3
HNRNPU;ZC3H14
ESR1;TEAD1
SLC17A6;SLC25A12
ADK;CMPK1
MTMR3;MTMR9

ING4;CBX5;RBBP5;PHF8;GLYR1
ADAMTS5;ADAMTS2;MME;MMP2;ADAM12;LNPEP;ZMPSTE24;METAP2
ERBB4;GAB1;PTPN11;ESR1;EREG;FGFR1
MECOM;RBBP5;NSD1;PRDM16
KAT2B;ZDHHC18;CREBBP;ZDHHC20;ZDHHC21
SGIP1;ARL3;HOOK3;RAB11A;REEP1;APC;VAPA;KIF5C;VAPB;KIF26B;DAG1;DCX;KIF1B;MAPRE3;MAPRE2
AGO1;HNRNPU;CDC73
KAT2B;CCNYL1;CKS1B
EEF2K;CAMK4;PTK2B
MTMR3;MTMR9
GABRA1;GABRA3
JPH3;JPH1
C1QBP;ADRA2A
RAB3C;RAB11A
NSD1;NRIP1;CTNNB1;PIAS1
ZDHHC18;ZDHHC20;ZDHHC21
LYN;DYRK1A;PTK2B;FRK
EPS8;NCKAP1;MTSS1L;SRGAP2
EPS8;GNA13;ARHGEF10;SOS2;ARHGEF5
CHAD;PPP1R1B;DUSP19;GSKIP;LRP6
NSD1;ACTN4
SLC24A2;TDG
ADRA2B;ADRA2A
PTK2B;GRIN2B
CNKSR3;DAG1;MYPN
ATP2B4;ATP2B2;ATP2B1
AMER1;FRMPD4;SESTD1;SYT7;MARK1
ERBB4;GAB1;PTPN11;ESR1;EREG;FGFR1
KAT2B;SPRED1;PPP1R1B
NUDT5;DIEXF;NUDT4
CUL3;DTX1
EFNA3;EFNB3
KANSL1;PHF20
LNPEP;TRHDE
DDI2;ASPRV1
CRP;SORL1
EFNA3;EFNB3
SYT5;CRP;SLC24A2;KCNIP2;S100B;SYTL4;SYT7;CDH6;CDH2;STIM2;MB;PCDHA4;SLIT2;CALM1;SLC25A1
RAB3C;RALA;RAB11A;TRAK2
USP13;DDI2;TNFAIP3;UBXN7;CKS1B
MAP2K1;CALM1;CKS1B
KIF5C;KIF1B;DYNLL2
MECOM;RBBP5;NSD1;PRDM16
RANBP3;XPO4
SET;PPP1R1B
CARM1;TRIM24
CLCN5;ANO6
ATP2B4;SLC1A4;ATP2B2;SCN5A;ATP2B1;SCN1A;TPCN1
CREBBP;RBL1;TSHZ3;PROX1;ZNF148;MXD1;BACH1
NPFFR1;GPR37;NPR3;SORCS1;TSHR
ABCA1;ATP8A2;ATP11A;PITPNC1
USP13;USP37;SFRP1;USP15;USP9X;USP2
ADAMTS5;ADAMTS2;MME;MMP2;ZMPSTE24

TCERG1;PABPN1
RANBP3;LDLRAD4
DCTN2;ADD2
CARM1;TRIM24
FZD5;APBB2;SORL1;ITM2C
RAB3C;RAB2B;SEPT10;SEPT11;SEPT12;ATL3;ATL2;RHOBTB3;GNL1;RND3;GTPBP4;RAB11A;GNA13;RAF
LNPEP;TRHDE;METAP2
AGO1;HNRNPU;CDC73
ADH1B
SLC28A3
ATP2B4
NSG1
NSD1
HPGD
SORT1
CHRM3
LOXL4
ST6GALNAC3
RBX1
USP15
CYB5R4
ST8SIA3
CHRM3
SORL1
CYB5R4
REEP1
TGFBR3
SART3
ASXL3
E2F1
ALG6
PAPOLG
DIEXF
ALDH2
SPRED1;CHAD;PPP1R1B;DUSP19;GSKIP
TSFM;EEF2K;EIF2B2;EIF3J;EIF4E
CRP;AP2M1
GRM5;GRID1
HNRNPU;HNRNPC
HNRNPU;ZC3H14
RUVBL1;ESR1
TFAP2B;HEYL
DDI2;ASPRV1
CNOT6;ERI1;RAD9A
GABRA1;GRID1;GABRA3
SART3;HNRNPU;SNRPD3
EIF2B2;EIF3J;EIF4E
PPP2CA;PDP2;PPM1H;PGAM5;PTPN11;CTDSPL2;PSPH
AMER1;FRMPD4;SESTD1;SYT7;KCNH1;MARK1
PLA2G12A
IL17RD
HEYL
PAX2

SMARCA1
SELE
RPS6KA6
IL6R
CREB1
BMPR2
ITPK1
TPCN1
JMY
TGM2
SLC1A4
HS3ST3B1
HNRNPU
SENP2
SLC25A12
SLC1A4
CXADR
METTL16
SLC1A4
CTBS
PCMT1
SFRP1;ZNRF3;LRP6
SESTD1;PLEKHA3
CNKSR3;DAG1
IGF2BP1;MYH10
CREBBP;SP1
SLC30A4;SLC39A13
LNPEP;TRHDE;ZMPSTE24;METAP2
APC;CHAD;PPP1R1B;CNKSR3;DUSP19;GSKIP;CCNYL1
PPWD1;FKBP6;PPIC
SLC30A4;SLC39A13;ATP2B1
ERI1;C1QBP;PELO
GRM5;MAP2K1;DUSP19;CALM1
SET;PPP1R1B
CRP;SORL1
GABRA1;CLCN5;GABRA3;SLC1A4
ABCA1;ABCA2;ATP8A2;ATP11A
CAPZB;TPM3;ACTN4;PPP1R9A;RCSD1;MYH10;MYPN;ADD2
SHC4;SHC2;NRG3
KCNIP2;AP2M1;KPNA1
TGFBR3
NCOA2
SNX1
TMEM86A
FUT11
TDG
PRKG1
CAMK4
PITPNC1
PFKFB3
SLC4A10
INHBA
SMAD7

ABCA1
MOCS3
UQCR10
CARM1
S1PR1
HPGD
NSUN3
UQCR10
ADH1B
LRP6
LYN;ERBB4;CDC37;TRIM24;DYRK1A;PTK2B;FRK;EREG;FGFR1
SCN5A;SCN3B;SCN1A
TMOD3;SWAP70;PTPRM;PARVA;GAPVD1;GIGYF2;CDH6;PCMT1;SNX1;HNRNPK;CDH2;CAPZB;VAPA;VAP
GABRB1;ANO6;SCN5A;CALM1;SCN1A;TPCN1
RABGAP1L;RIMS3;RANBP3;MYO1C;XPO4;RHOBTB3;TBC1D25;RAB3GAP2;RAPGEF6;RAB11FIP5;STXBP
ABCA1;ABCA2;HSPH1;KIF5C;KIF26B;HSPA4L;CARNS1;RUVBL1;RHOBTB3;KIF1B;DYNLL2;MYH10
ABCA1;ACAP2;AGAP2;AGFG2;APPL1
ADAMTS5;CXADR;S1PR3;ACTN4;FBLN5;CTGF
KAT2B;CREBBP;EPC1
RXRA;E2F1
TXNL1;TXNDC17
AGO1;PUM2
SLC24A2;DENND5B;ATP2B4;ATP2B2;ATP2B1
EIF4E
RPP14
SLC25A24
TRAK2
STRN
S100B
SH3PXD2A
CARM1
PHF8
NCEH1
OSTC
NSD1
MYH10
SORT1
NRIP1
ITPK1
PPP1R1B
ALDH6A1;ALDH2
CRP;AP2M1
TEAD1;ESR1
ERI1;DCP2
ESCO1;MME;ADH1B;TNKS;SORD;LNPEP;BMI1;S100B;GRIN2B;LITAF;TRHDE;PHF8;ALAD;RXRA;NSD1;FN
GAB1;PTPN11;FGFR1
TFAP2B;TIPARP
DAG1;CRK
PTP4A1;MTMR3;PTPRM;PTPN11
GPR27;PTGFR;FZD3;NPFFR1;GPR37;FZD5;NPR3;GPR75;RORB;TSHR;SFRP1;GRM5;GNRHR;S1PR1;S1F
CDK1
STIM2
PPP1R1B

SESTD1
TDG
AP2M1
PITPNC1
MARK1
SYTL4
TXNL1
CARM1
CALM1
OSTC
CALM1
EIF4EBP2
NUDT4
AP2M1
ACSL6
SMAD7
PDE5A
TERF2IP
KAT2B
SLC25A24
SLC25A24
USP9X
RABGAP1L;RANBP3;AGAP2;MTSS1L;STXBP5L;ACAP2;ARHGAP20;ARHGAP31;RIC8B;TBC1D25;PGAM5;
AGO1;HNRNPU;RBM7
SPRED1;MTMR3;MTMR9;TERF2IP;MAPK1;CTNNB1
DPYSL3
PHACTR4
LTN1
GJA9
ADRA2A
NUDT4
BFAR
HSPH1
PGM3
SNX6
HSPH1
DUSP19
CAMK4
ABHD2
TPM3;HNRNPU;PARVA;ACTN4;RCSD1;PPP1R9A;MYPN;LMOD3;ADD2;EPS8;CAPZB;DAG1;MYH10;PHAC
ERBB4;RAF1;TRIB2;FGFR1
RUVBL1;G3BP1
SLC22A17;SLC4A10
ARHGEF10;KIF1B
SLC17A6;SLC25A12
TCERG1;SP1
PCMT1;SETD9;DNMT3A
SYT5;SYTL4;SYT7
RABGAP1L;RANBP3;AGAP2;MTSS1L;STXBP5L;ACAP2;ARHGAP20;ARHGAP31;RIC8B;TBC1D25;PGAM5;
GABRA1;CLCN5;GABRA3;SLC1A4
MEF2A;KAT2B;HDAC5;SP1;RAD9A
KCNC2;KCNH1
ENAH;LITAF

HNRNPC;GATAD2B
KIF5C;KIF1B;DYNLL2
SULF2
KCNIP2
MYO1C
TERF2IP
GJA9
GRM5
SLC24A2
CARM1
CNOT6
CREB1
SMARCA1
GABRA1;GABRB1;GABRA3;CALM1
PLA2G12A;PAFAH1B2
HIF1A;FKBP6
CNOT6;RNASE6;SMG7
SLC17A6;SLC1A4;SLC25A12
B3GALNT2;MGAT5;ALG14
CNOT6;ERI1
TET3;PHF8
GABRB1;CALM1
APC
FUT11
RPP14
CRP
PHF8
SLIT2
CYB5R4
CA12
TSFM
GAB1;PTPN11;FGFR1
TFAP2B;TNFAIP8;XIAP
SLC30A4;SLC39A13
HPSE2;CTBS
RABGAP1L;RIMS3;RHOBTB3;TBC1D25;RAB3GAP2;RAB11FIP5;STXBP5L
GABRA1;GABRB1;GABRA3
KCNIP2
EPS8
CARNS1
NT5DC3
RXRA
ALDH2
HS3ST3B1
TDG
CALM1
RUVBL1;G3BP1;GTF2H1;SMARCA1
EPS8;NCKAP1;MTSS1L;SRGAP2
SNX1;EFEMP1;ERBB4;IL6R;EREG
MEF2A;PURA;SIN3A;HNRNPU;RORB;NFE2L1
SLC24A2;DENND5B;STIM2;IL1RAPL1;TPCN1
REEP1;GNA13;SFRP1;ZNRF3;C1QBP;S1PR1;LRP6
DDX19B;RUVBL1

HSPH1;HSPA4L;GTF2H1;SMARCA1;MYH10
CD244
MAP2K1
LOXL4
TXNL1
SNPH
FKBP6
PTPRM
PTPRM
DCP2
NPFFR1;SORCS1
ABCA1;SLC4A10
CHRM3;ADRA2B;ADRA2A
RBBP5
SART3
MYPN
SULF2
TGM2
SLC24A2
NT5DC3
BCL2L13
HNRNPC
ERBB4;MAP3K9;RAF1;FGFR1
IL1RAPL1;TPCN1
ESCO1;MME;TNKS;ADH1B;SORD;LNPEP;BMI1;S100B;SOD2;GRIN2B;LITAF;TRHDE;PHF8;ALAD;RXRA;N[illegible]
EIF4E
ABCA1
CLCN5
ITCH
TFAM
GNA13
SDR16C5
RNASE6;SMG7;N4BP2
AGO1;HNRNPU;MTDH
ERI1;RAD9A
MYH10
PIAS1
ACSL6
SCN5A
C1QBP
SEMA3A
BCL2L13
CMPK1
MEF2A
KIF5C;KIF26B;KIF1B
CYTH3;SESTD1;PLEKHA3;KCNH1
CHST7;CHST3
ADAMTS5;ADAMTS2;APH1B;MME;USP9X;MMP2;ADAM12;LNPEP;SENP2
EFEMP1;ERBB4;FGFR1
PLA2G12A;NCEH1;ABHD4;ABHD2;PAFAH1B2
HDAC5;SIN3A
RUVBL1;G3BP1
METTL16

ANO6
TGFBR3
ANO6
RAB11FIP5
GSKIP
TMOD3
SEMA3A
APC
CYB5R4
RORB
ERBB4;BMPR1A;FGFR1
ABCA1;SLC22A17;SLC4A10
CMPK1;IP6K1
ABCA1;RALA;ESR1
ZBTB4
USP13
TNFAIP3
KIF5C;KIF26B;KIF1B;MYH10
DHX40;DDX19B;RUVBL1;G3BP1
HDAC5;SIN3A
ABCA1;ABCA2
NCEH1;ABHD2
ERI1;DIEXF
PAPOLG
SETD9
PDE5A
GALNT7
GPR75
SESTD1
CLCN5;SLC22A17;SLC4A10
SDR16C5;HPGD;SORD;GLYR1
SLC28A3
SETD9
SLC4A10
RXRA
TMOD3
CARM1;NSUN3;DNMT3A
DHX40;DDX19B;G3BP1
DHX40;DDX19B;G3BP1
ALAD;CA12
ABCA1;ABCA2
ABCA1;ABCA2
DHX40;DDX19B;G3BP1
CALM1
CHRM3
ANO6
CREBBP;TDG
NRG3;INHBA;EREG
TGFBR3;SPRED1;USP15;INHBA;GDF6;IL6R
CYB5R4;ETFA
B3GALNT2;GALNT7
SLC22A17;SLC4A10
MTDH

DPY19L2
RPP14
DUSP19
RELT
RELT
SESTD1
CHRM3
SLC1A4
IL17RD;IL6R;EPOR
USP13;USP37;USP15;MME;USP9X;MMP2;USP2;KLK13;SENP2;ASPRV1;ZMPSTE24;ADAMTS5;SFRP1;AD
ANO6
ATP2B1
ESCO1
LMOD3
HDAC5;SIN3A;GLS
AZIN1
DNAL1
KCNC2;IL1RAPL1;KCNH1;TPCN1
B3GALNT2;DPY19L2;ALG6;HAS2
VAPB
CHRM3
GPR37
TFAP2B
SLC24A2;DENND5B;ANO6;SCN5A;SCN1A;TPCN1
TET3;PHF8
KPNA1
ABCA1
CRK
PGAM5;PSPH
SLC1A4;SLC25A12
CMPK1
CD244
GPAM
SESTD1
EMB
CYTH3
KCNC2;KCNIP2;KCNH1
PTK2B;GRIN2B
GPCPD1
CDK1
SLC22A17
CALM1
B3GAT2
DUSP19
CDK1
C1QBP
MYOD1
CRK
B3GALNT2;HAS2
SOD2
SLC1A4;EMB
HSPH1
PLA2G12A;PAFAH1B2

HDAC5
SLC22A17;SLC25A24
SLC24A2;SLC4A10
GPC1
CARM1
PGRMC1;BACH1
RNASE6;N4BP2
KCNIP2
SLC4A10
TNFAIP3
ABCA1
ABCA1
RAP1A;KPNA1
ITCH
UQCR10
SART3;TNFAIP3;BFAR
KCNC2;KCNH1
INHBA
INHBA;GDF6
MMP2;KLK13
MMP2;KLK13

;BFAR;UBE2G1;RBX1;PIAS1;RNF126;ITCH;CDC34;HECW2;RLIM;TRIP12;RNF165
TRIM24;PPARGC1B;MEF2A;NCOA2;TFAP2B;CREBBP;PRRX1;ARID5B;ACTN4;HCFC2;ZFX;HIPK2;PIAS
;UBE2G1;BFAR;RBX1;RNF126;ITCH;CDC34;HECW2;RLIM;TRIP12;UBE2K;RNF165
;HIVEP2;TEAD1;MEF2A;BCL11B;ZBTB14;XRCC5;TIPARP;TFEB;ARID5B;PROX1;ESR1;PAX2;RUNX1;SM
IRNPK;CREB1;NFIA;SP1;NKRF;TFAM;SSBP2;NFE2L1
OXJ2;TBX5;ESR1;PAX2;GATAD2B;PHOX2B;HNRNPK;CREB1;NFIA;SP1;MYOD1;TFAM;HNRNPC;MXD1;
4;FBXO3;LONRF3;BTRC;UNKL;RNF111;FBXW4;SMURF2;MSL2;RC3H1;ZFP91;UBE2G1;KLHL23;BFAR;I

3;EBF1;NFATC3;TFEB;FOXJ2;PROX1;ESR1;PHOX2B;HNRNPK;CREB1;RBL1;NFIA;SP1;TFAM;MXD1;SSI
CTNNB1;TEAD1
8;MEF2A;KLF10;TFAP2B;ZBTB16;CNBP;NFATC3;FOXJ2;TFEB;ARID5B;ACTN4;TBX5;RUNX3;ESR1;GAT
C3;TFEB;FOXJ2;ESR1;PHOX2B;RUNX1;HNRNPK;CREB1;NFIA;SP1;MYOD1;TFAM;LHX4;SSBP2;NFE2L

1;GATAD2B;PHOX2B;HNRNPK;CREB1;NFIA;SP1;MYOD1;TFAM;HNRNPC;MXD1;SSBP2;NFE2L1

RID5B;ACTN4;PROX1;RUNX3;ESR1;PAX2;GATAD2B;PHOX2B;RUNX1;CREB1;SP1;MYOD1;CGGBP1;NF
B;RC3H1;ACTN4;FNDC3A;GTPBP4;FAM133B;DHX40;SUB1;TFAM;SCG3;SRSF9;YTHDC1;C1ORF52;GI
XRA;TBL1XR1;SP1;RBBP5;ERBB4;SALL4;CARM1;HIVEP2;TEAD1

F1;HIVEP2;ZNF148;TEAD1;MEF2A;EOMES;TFAP2B;ZBTB14;EGR4;BCL11B;XRCC5;TBX5;POU3F1;FOX

XRA;CREB1;RBL1;SP1;TRPS1;MYOD1;CGGBP1

A;RAP2B;PDE5A;ARHGEF5

3;MXD1;CGGBP1
A;RAP2B;ARHGEF5
SD1;NRIP1;ID4;RLIM;MXD1;ZNF366
2B;ARHGEF5

2;YOD1;UBE2G1;BTBD9;RBX1;SMAD7;ITCH;CDC34;APC;TRIB2;UBE2K
1;SNIP1;HNRNPC;TARDBP;MYH10

;RBX1;SMAD7;CDC34;APC;UBXN7;SCN5A;TRIB2;UBE2K;CCDC50

K1;ABCA1;SRPK2;ABCA2;EIF2B2;SEPT12;CDC42BPA;GNL1;RUNX3;GTPBP4;RAP2C;GLUD2;RAP2A;R

;AM5

3;ADD2;CCND3;SPRED1;GRM5;ERBB4;SCN5A;APPL1;MAP3K2;FZD5;ESR1;ADRA2A;KAT2B;APC;DCX

\B2;MAP2K1;CSNK1A1;NEK7;DYRK1A;GTF2H1;CDC42BPA;HIPK2;CAMK4;SIK3;CDK1;SIK2;RAF1;BMPF

(A6;EEF2K;ETNK1;CDK1;RAF1;IP6K1;PRKG1

DNMT3A;ARID5B;PROX1;POU3F1;FOXP2;PAX2;SUB1;TFAM;RBMS1;CGGBP1;MYRF;ZNF25;ZBTB4;HI
/ARK1;SRPK2;SYT5;KCNIP2;CDC42BPA;S100B;SOD2;SYTL4;SYT7;RAP2A;STIM2;SIK3;SIK2;SLC25A1

PH1;TDG;SIK3;SIK2;N4BP2;MYH10;MARK1;BMPR1A

(A6;EEF2K;ETNK1;CDK1;MAPK1;RAF1;IP6K1;PRKG1
\PB;MYOD1;RAB3GAP2;TEAD1;SNX6

PRED1;SCN5A;MAP3K2;FZD5;ESR1;ADRA2A;KAT2B;APC;CDC37;DCX;CTNNB1;MAPRE3;CALM1;RAD9

;SIK2;N4BP2;MYH10;MARK1;BMPR1A

:DH2;ERBB4;TDG;MAP3K9;SLIT2;SRGAP2;IL6R;PSPH;SNX6;TFAP2B;CADM3;SEPT12;ZBTB16;ACSL6;[illegible]

\1;ABCA2;RAB2B;SEPT10;SEPT11;ENTPD1;SEPT12;SMARCA1;GNL1;GTPBP4;RAB11A;RAP2A;DDX19[illegible]
<2;MAP3K2;CSNK1G3;MAP2K1;CSNK1A1;NEK7;DYRK1A;GTF2H1;CDC42BPA;HIPK2;EREG;CDC37;CD[illegible]

2;DNAL1;RAB11FIP5

12;PCDHA7;PSPH;SLC25A24

ʾ2A

'B;CAPZA1;SERBP1;RUVBL1;CTNNB1

'5L

ITB;TRIM24

'R3

SRGAP2;AGFG2;APPL1

TR4

SLIT2;SRGAP2;AGFG2;APPL1

SD1;GPC1;TET3;FNTB;TRIM24

)AMTS2;APH1B;DDI2

1;RAP2C;KAT2B;SUB1;CARM1;JMY;CTNNB1

MAD7;CREB1;RBL1;KANSL1;TBL1XR1;SP1;AGO1;MYOD1;CARM1;TFAM;NFE2L1

;SSBP2;NFE2L1
FBXO30;RBX1;RNF126;ITCH;CDC34;HECW2;RFWD3;KLHL7;RLIM;TRIP12;DTL;UBE2K;RNF165

BP2;NFE2L1

TAD2B;PHOX2B;RUNX1;HNRNPK;CREB1;NFIA;SP1;MYOD1;TFAM;HNRNPC;MXD1;SSBP2;CGGBP1;NF
-1

FE2L1
GYF2;NOP9;MTDH;ERI1;ZMAT3;G3BP1;RBM12;MYH10;ZC3H14;PPARGC1B;SRPK2;CPSF7;COA1;XRC

KP2;ZNF70;SP1;SP4;TFAM;TERF2IP;LHX4

AP2B;SIK3;SIK2;ARHGEF5;BMPR1A

;MAPRE3;CALM1;RAF1;CRK;TRIB2;RAD9A;FGFR1

R1A

F1A;FOXO1;PURA;BCLAF1;RXRA;TDG;PRDM16;E2F1;HIVEP2;ZNF148;TFAP2B;CREBBP;ZBTB14;EGR
2;CALM1;PRTFDC1

9A

SLC39A13;ACTN4;S100B;FOXP2;ADRA2A;SP1;VAPB;KLHL7;ZNF318;NFE2L1;PRTFDC1;FGFR1;BMPR[illegible]

B;KIF26B
[illegible]K1;SIK3;SIK2;RAF1;FRK;FGFR1;BMPR1A

FE2L1

C5;MRPL27;HELZ;LSM5;PUM2;ALDH6A1;HNRNPK;AGO1;FUBP1;CAPRIN1;HNRNPC;LSM14B;RBM28;(

[illegible]4;BCL11B;XRCC5;ZBTB16;TBX5;RCAN1;ZNF70;HNRNPK;SP1;SP4;FUBP1;RPA4;NABP1;LHX4;TARDB[illegible]

1A

CELF1;PRR3;CELF3;ADK;SMG7;ELAVL2;RBM3;MRPL42;EEF2K;SART3;NKRF;C1QBP;METTL16;RBM7;

P;SSBP2

UTP15;ZCCHC24;TSFM;SMC1A;DIEXF;RBMS1;DCP2;FYTTD1;SF3B3;STAU1;NUFIP2;SRSF1;PURA;NX

‹F1;BCLAF1;PABPN1;TRA2B;IGF2BP1;RPP14;MBNL3;SRSF10;EIF4E;SMNDC1;CNBP;YLPM1;PDAP1;DI

EK;U2SURP;SERBP1;TARDBP;METAP2;TNRC6B
