# Supplementary material for: Circulating miR-330-3p in Late Pregnancy is Associated with Pregnancy Outcomes Among Lean Women with GDM
Source: Sci Rep. 2020 Jan 22;10:908. doi: 10.1038/s41598-020-57838-6 (PMC6976655; doi:10.1038/s41598-020-57838-6)
Supplement: Supplementary file 1 — Supplementary data. [file 41598_2020_57838_MOESM1_ESM.zip › Supplimentary File_EnrichR_Analysis InterPro_Domains_2019.pdf]

Term	Overlap
Cadherin, C-terminal catenin-binding domain	15/37
Cadherin, N-terminal	17/65
Cadherin-like	19/115
WWE domain	5/12
PPM-type phosphatase domain	6/20
RNA recognition motif domain	24/223
PTB/PI domain	8/41
Sortilin, C-terminal	3/5
Sortilin, N-terminal	3/5
VPS10	3/5
Zinc finger, RING-type	27/290
Mu homology domain	4/13
WW domain	8/52
PH-BEACH domain	3/8
Serine aminopeptidase, S33	3/8
BEACH domain	3/9
Poly(ADP-ribose) polymerase, catalytic domain	4/17
Zinc finger, CCCH-type	8/59
Zinc finger, ZZ-type	4/18
Armadillo repeat-containing domain	3/10
3'5'-cyclic nucleotide phosphodiesterase, catalytic domain	4/21
MAD homology 1, Dwarfin-type	3/12
BTB/POZ domain	16/178
Deltex, C-terminal	2/5
Potentiating neddylation domain	2/5
Sorting nexin Vps5-like, C-terminal	2/5
Ubiquitin-associated domain	6/44
ATP-grasp fold	3/13
Basic leucine zipper domain, Maf-type	3/13
Septin-type guanine nucleotide-binding (G) domain	3/13
SAP domain	4/23
HD/PDEase domain	4/24
Kinesin-like	2/6
LCCL domain	2/6
Lon, substrate-binding domain	2/6
Major sperm protein (MSP) domain	2/6
Voltage-gated Na+ ion channel, cytoplasmic domain	2/6
Zinc finger, GATA-type	3/15
OTU domain	3/15
Small GTP-binding protein domain	14/162
Cation-transporting P-type ATPase, C-terminal	3/16
Sec7 domain	3/16
Synaptotagmin	3/16
SET domain	6/51
RAWUL domain	2/7
HECT domain	4/28
Zinc finger, RING-CH-type	3/17
Basic-leucine zipper domain	6/54
Yippee/Mis18/Cereblon	2/8
ERAP1-like C-terminal domain	2/8
Ephrin receptor-binding domain	2/8
MAD homology, MH1	2/8

SMAD domain, Dwarfin-type	2/8
Tensin/EPS8 phosphotyrosine-binding domain	2/8
Transcription elongation factor S-II, central domain	2/8
Vacuolar protein sorting-associated protein 13, N-terminal domain	2/8
Cation-transporting P-type ATPase, N-terminal	3/18
IPT domain	4/30
Immunoglobulin	7/70
Activin types I and II receptor domain	2/9
Guanylate-binding protein/Atlastin, C-terminal	2/9
Nuclear transport factor 2	2/9
Nuclear transport factor 2, eukaryote	2/9
Paired domain	2/9
Plexin, cytoplasmic RasGAP domain	2/9
LSM domain, eukaryotic/archaea-type	3/20
Protein kinase domain	32/482
Chromo domain subgroup	2/10
Sodium ion transport-associated	2/10
THIF-type NAD/FAD binding fold	2/10
Type I cytokine receptor, cytokine-binding domain	2/10
Connexin, N-terminal	3/21
Gap junction protein, cysteine-rich domain	3/21
Protein-tyrosine phosphatase, catalytic	6/62
Pleckstrin homology domain	19/269
Sterile alpha motif domain	8/93
Amidohydrolase-related	2/11
Frizzled/Smoothened, transmembrane domain	2/11
GB1/RHD3-type guanine nucleotide-binding (G) domain	2/11
Guanylate-binding protein, N-terminal	2/11
Transcription elongation factor, TFIIS/CRSP70, N-terminal, sub-type	2/11
WH1/EVH1 domain	2/11
Frizzled domain	3/23
PWWP domain	3/23
K Homology domain	4/36
Receptor, ligand binding region	4/36
F-box domain	6/65
Peptidase C19, ubiquitin carboxyl-terminal hydrolase	7/80
K Homology domain, type 1	4/37
GNAT domain	3/24
Palmitoyltransferase, DHHC domain	3/24
Zinc finger, TRAF-type	2/12
Ubiquitin specific protease domain	7/82
Myc-type, basic helix-loop-helix (bHLH) domain	9/114
Cystine knot, C-terminal	3/25
Peptidase M1, membrane alanine aminopeptidase	2/13
Transcription factor IIS, N-terminal	2/13
Chromo domain	3/26
Peptidase M12B, ADAM/reprolysin	4/40
Peptidase M12B, propeptide	4/40
Ubiquitin-conjugating enzyme E2	4/40
NUDIX hydrolase domain	3/27
Myotubularin-like phosphatase domain	2/14
Neurexin/syndecan/glycophorin C	2/14
P-type ATPase, C-terminal	2/14

P-type ATPase, N-terminal	2/14
ADAM, cysteine-rich domain	3/28
LIS1 homology motif	3/28
AP complex, mu/sigma subunit	2/15
Alcohol dehydrogenase, C-terminal	2/15
Alcohol dehydrogenase, N-terminal	2/15
B-box, C-terminal	2/15
CAP domain	2/15
Cytochrome b5-like heme/steroid binding domain	2/15
Formin, FH2 domain	2/15
Arf GTPase activating protein	3/29
FYVE zinc finger	3/29
SH3 domain	15/225
OAR domain	2/16
Polyketide synthase, enoylreductase domain	2/16
Tudor domain	3/30
Sema domain	3/31
C2 domain	9/128
FKBP-type peptidyl-prolyl cis-trans isomerase domain	2/17
PA domain	2/17
Phospholipid/glycerol acyltransferase	2/17
Yippee domain	1/5
3-oxo-5-alpha-steroid 4-dehydrogenase, C-terminal	1/5
Zinc finger, FCS-type	1/5
AWS domain	1/5
Alpha-D-phosphohexomutase, alpha/beta/alpha domain I	1/5
CIDE-N domain	1/5
Cationic amino acid transporter, C-terminal	1/5
DAN	1/5
DOMON domain	1/5
Dedicator of cytokinesis, N-terminal domain	1/5
Domain of unknown function DUF4704	1/5
Dullard phosphatase domain, eukaryotic	1/5
Ephrin-A ectodomain	1/5
Flavoprotein pyridine nucleotide cytochrome reductase-like, FAD-binding domain	1/5
Glutamine amidotransferase type 2 domain	1/5
Growth hormone/erythropoietin receptor, ligand binding	1/5
Hexokinase, C-terminal	1/5
Hexokinase, N-terminal	1/5
IMD/I-BAR domain	1/5
Inhibitor of growth protein, N-terminal histone-binding	1/5
KIND domain	1/5
Kinase associated domain 1 (KA1)	1/5
Kinesin-like KIF1-type	1/5
Neuralized homology repeat (NHR) domain	1/5
Nuclear RNA export factor Tap, RNA-binding domain	1/5
PUA domain	1/5
Protocadherin	1/5
Putative zinc-RING and/or ribbon	1/5
SERTA domain	1/5
SLED domain	1/5
TAFH/NHR1	1/5
Transcription factor AP-2, C-terminal	1/5

Transcription factor, MADS-box	1/5
Transketolase, C-terminal domain	1/5
YTH domain	1/5
FERM central domain	4/48
FERM domain	4/48
Serine-threonine/tyrosine-protein kinase, catalytic domain	9/131
Ionotropic glutamate receptor	2/18
Ionotropic glutamate receptor, L-glutamate and glycine-binding domain	2/18
MAM domain	2/18
Calponin homology domain	6/81
Chromo/chromo shadow domain	3/33
Sulfotransferase domain	3/33
Zinc finger, FYVE-related	3/34
Forkhead-associated (FHA) domain	3/34
PAS domain	3/34
Aldehyde dehydrogenase domain	2/19
GTP binding domain	2/19
PAS fold	2/19
Reprolysin domain, adamalysin-type	2/19
Band 4.1 domain	4/50
Phox homologous domain	4/50
WD40-repeat-containing domain	15/240
Cyclic nucleotide-binding domain	3/35
Zinc finger, TFIIS-type	1/6
Anti-proliferative protein	1/6
Argonaute, linker 1 domain	1/6
BRO1 domain	1/6
DMAP1-binding domain	1/6
Domain of unknown function DUF3456	1/6
E2F transcription factor, CC-MB domain	1/6
EF-hand domain, type 1	1/6
EF-hand domain, type 2	1/6
EF-hand, Ca insensitive	1/6
ELMO domain	1/6
FF domain	1/6
Glutamine amidotransferase	1/6
Growth factor receptor domain 4	1/6
Initiation factor eIF-4 gamma, MA3	1/6
Kinesin-associated	1/6
MHCK/EF2 kinase	1/6
OBG-type guanine nucleotide-binding (G) domain	1/6
Occludin homology domain	1/6
PAS-associated, C-terminal	1/6
PET domain	1/6
Pleckstrin homology domain, spectrin-type	1/6
Rab-binding domain FIP-RBD	1/6
RecF/RecN/SMC, N-terminal	1/6
SEFIR domain	1/6
SMCs flexible hinge	1/6
SWAP/Surp	1/6
Tesmin/TSO1-like CXC domain	1/6
Zinc finger C2H2-type	45/797
Homeobox domain	15/247

EGF-like domain	14/229
Double-stranded RNA-binding domain	2/21
Zinc finger, CCHC-type	3/37
Cyclin-like	3/37
Zinc finger, PHD-type	6/88
Leucine-rich repeat N-terminal domain	4/54
Zinc finger, A20-type	1/7
Zinc finger, MIZ-type	1/7
Acyl-CoA-binding protein, ACBP	1/7
Alpha/beta hydrolase fold-3	1/7
Anoctamin, dimerisation domain	1/7
Aspartic peptidase, N-terminal	1/7
CUT domain	1/7
Chitinase II	1/7
Circularly permuted (CP)-type guanine nucleotide-binding (G) domain	1/7
Cytidyltransferase-like domain	1/7
Drought induced 19 protein type, zinc-binding domain	1/7
Furin-like cysteine-rich domain	1/7
GAF domain	1/7
GS domain	1/7
Glycerophosphodiester phosphodiesterase domain	1/7
Glycoside hydrolase family 18, catalytic domain	1/7
Homeobox protein SIX1, N-terminal SD domain	1/7
Peptidase C19, ubiquitin-specific peptidase, DUSP domain	1/7
Raf-like Ras-binding	1/7
Receptor L-domain	1/7
Syndecan/Neurexin domain	1/7
Ulp1 protease family, C-terminal catalytic domain	1/7
PTP type protein phosphatase	3/38
Disintegrin domain	2/22
Ras-like guanine nucleotide exchange factor, N-terminal	2/22
Tripartite DENN domain	2/22
AGC-kinase, C-terminal	4/55
Homeobox domain, metazoa	6/90
Ras-associating (RA) domain	3/39
SH2 domain	7/109
ADAM-TS Spacer 1	2/23
Coagulation factor 5/8 C-terminal domain	2/23
FCH domain	2/23
TGF-beta, propeptide	2/23
Zinc-binding domain	1/8
CID domain	1/8
Cadherin prodomain	1/8
Class I myosin tail homology domain	1/8
Class I myosin, motor domain	1/8
Cullin protein, neddylation domain	1/8
Diaphanous autoregulatory (DAD) domain	1/8
Enhancer of polycomb-like, N-terminal	1/8
FCP1 homology domain	1/8
Fatty acid desaturase domain	1/8
Fucosyltransferase, N-terminal	1/8
LRAT domain	1/8
Peptidase M13, C-terminal domain	1/8

Peptidase M13, N-terminal domain	1/8
Piwi domain	1/8
Tensin-type phosphatase domain	1/8
Transketolase-like, pyrimidine-binding domain	1/8
Ubiquitin domain	4/58
Cadherin, cytoplasmic domain	2/24
Cyclophilin-type peptidyl-prolyl cis-trans isomerase domain	2/24
Endonuclease/exonuclease/phosphatase	2/24
Zinc finger, PHD-finger	5/76
Bromodomain	3/41
Cadherin, cytoplasmic C-terminal domain	3/41
VWFC domain	3/41
PSI domain	3/42
F-BAR domain	2/25
BTB/Kelch-associated	4/60
Band 3 cytoplasmic domain	1/9
Cullin homology domain	1/9
Doublecortin domain	1/9
Follistatin-like, N-terminal	1/9
Formin, FH3 domain	1/9
Formin, GTPase-binding domain	1/9
Importin-alpha, importin-beta-binding domain	1/9
Myb domain	1/9
Na-Ca exchanger/integrin-beta4	1/9
P-type trefoil domain	1/9
PAZ domain	1/9
Peptidase M24	1/9
SAM-dependent methyltransferase RsmB/NOP2-type	1/9
Sodium/calcium exchanger membrane region	1/9
Tensin phosphatase, C2 domain	1/9
Transglutaminase, C-terminal	1/9
Transglutaminase, N-terminal	1/9
PDZ domain	9/152
Tyrosine-protein kinase, catalytic domain	5/79
SANT domain	2/26
EF-hand domain	13/228
SANT/Myb domain	3/44
Zinc finger, C3HC4 RING-type	2/27
CRAL-TRIO lipid binding domain	2/27
Complement Clr-like EGF domain	2/27
FERM, C-terminal PH-like domain	2/27
Target SNARE coiled-coil homology domain	2/27
BRICHOS domain	1/10
Bicarbonate transporter, C-terminal	1/10
Biotin/lipoyl attachment	1/10
Cullin, N-terminal	1/10
Heat shock chaperonin-binding	1/10
MIF4G-like, type 3	1/10
Macro domain	1/10
Peptidase family A1 domain	1/10
Polymerase, nucleotidyl transferase domain	1/10
Rel homology dimerisation domain	1/10
Rel homology domain (RHD), DNA-binding domain	1/10

Ubiquitin system component CUE	1/10
VPS9 domain	1/10
Kinesin motor domain	3/45
Tyrosine specific protein phosphatases domain	5/82
Zinc finger, nuclear hormone receptor-type	3/46
Neurotransmitter-gated ion-channel transmembrane domain	3/46
Alpha/beta hydrolase fold-1	2/28
Cysteine-rich flanking region, C-terminal	5/83
Neurotransmitter-gated ion-channel ligand-binding domain	3/47
DHR-1 domain	1/11
DHR-2 domain	1/11
Dedicator of cytokinesis, C-terminal	1/11
E2F/DP family, winged-helix DNA-binding domain	1/11
Histone deacetylase domain	1/11
PKD domain	1/11
Phosphoribosyltransferase domain	1/11
R3H domain	1/11
Rap GTPase activating protein domain	1/11
Rho GTPase-binding/formin homology 3 (GBD/FH3) domain	1/11
Transglutaminase-like	1/11
FERM, N-terminal	2/29
Nuclear hormone receptor, ligand-binding domain	3/48
AMP-dependent synthetase/ligase	2/30
Matrin/U1-C-like, C2H2-type zinc finger	2/30
Protein kinase, C-terminal	2/30
Ras guanine-nucleotide exchange factors catalytic domain	2/30
ABC transporter-like	3/49
Fork head domain	3/49
DDE superfamily endonuclease domain	1/12
IRS-type PTB domain	1/12
Mab-21 domain	1/12
Orange domain	1/12
Oxidoreductase FAD/NAD(P)-binding	1/12
Peptidase M28	1/12
Rab-binding domain	1/12
Ran binding domain	1/12
Synaptobrevin	1/12
Potassium channel tetramerisation-type BTB domain	3/50
Cyclin, N-terminal	2/32
DNA binding HTH domain, Psq-type	1/13
ELM2 domain	1/13
FERM adjacent (FA)	1/13
Fibronectin type II domain	1/13
GPCR, family 3, nine cysteines domain	1/13
Globin	1/13
HTH CenpB-type DNA-binding domain	1/13
PAS fold-3	1/13
Peptidase A2A, retrovirus, catalytic	1/13
Serine/threonine-specific protein phosphatase/bis(5-nucleosyl)-tetraphosphatase	1/13
UBX domain	1/13
Tetratricopeptide repeat-containing domain	7/131
Zinc finger, UBP-type	1/14
Zinc finger, double-stranded RNA binding	1/14

Exonuclease, RNase T/DNA polymerase III	1/14
PB1 domain	1/14
Rab-GTPase-TBC domain	3/54
ARID DNA-binding domain	1/15
uDENN domain	1/15
Citron homology (CNH) domain	1/15
IBR domain	1/15
Ras GTPase-activating domain	1/15
Ribonuclease A-domain	1/15
cDENN domain	1/16
dDENN domain	1/16
BAR domain	1/16
CS domain	1/16
Cytidine and deoxycytidylate deaminase domain	1/16
Glycoprotein hormone subunit beta	1/16
POU-specific domain	1/16
Post-SET domain	1/16
TRAM/LAG1/CLN8 homology domain	1/16
Transforming growth factor-beta, C-terminal	2/37
Laminin G domain	3/58
RING-type zinc-finger, LisH dimerisation motif	2/38
Zona pellucida domain	1/17
Aminotransferase, class I/classII	1/17
Cyclin, C-terminal domain	1/17
Domain of unknown function DUF1605	1/17
POU domain	1/17
Sulfatase, N-terminal	1/17
B-box-type zinc finger	4/80
GPCR, family 2-like	3/60
Myosin head, motor domain	2/39
Alpha carbonic anhydrase domain	1/18
CRIB domain	1/18
GOLD domain	1/18
GRAM domain	1/18
Helicase-associated domain	1/18
Netrin module, non-TIMP type	1/18
PLAC	1/18
Fibronectin type III	10/205
Adenylyl cyclase class-3/4/guanylyl cyclase	1/19
FAD-binding domain, ferredoxin reductase-type	1/19
Myosin tail	1/19
Myosin, N-terminal, SH3-like	1/19
RUN domain	1/19
Ion transport domain	5/106
Dual specificity phosphatase, catalytic domain	2/42
Protein kinase C-like, phorbol ester/diacylglycerol-binding domain	3/64
CBS domain	1/20
Insulin-like growth factor-binding protein, IGFBP	1/20
Peptidoglycan binding-like	1/20
Ankyrin repeat-containing domain	12/254
Rho GTPase-activating protein domain	3/66
Zinc finger, MYND-type	1/21
Diacylglycerol/phorbol-ester binding	1/21

PLAT/LH2 domain	1/21
WH2 domain	1/21
GPCR family 3, C-terminal	1/22
Hyaluronan/mRNA-binding protein	1/22
Hemopexin-like domain	1/23
Netrin domain	1/23
Peptidase M10, metallopeptidase	1/23
Peptidase M10A, catalytic domain	1/23
Toll/interleukin-1 receptor homology (TIR) domain	1/23
Tubulin/FtsZ, GTPase domain	1/23
Dbl homology (DH) domain	3/70
Immunoglobulin subtype 2	11/242
Zinc finger, LIM-type	3/71
Proteasome component (PCI) domain	1/24
Rhodanese-like domain	1/24
Glycosyltransferase 2-like	1/25
SRCR domain	1/25
CUB domain	2/50
BRCT domain	1/26
RNA recognition motif domain, eukaryote	1/26
SRCR-like domain	1/26
ABC transporter type 1, transmembrane domain	1/27
GPCR, family 2, extracellular hormone receptor domain	1/27
von Willebrand factor, type A	3/77
EGF-like calcium-binding domain	5/124
CD80-like, immunoglobulin C2-set	1/28
Calcineurin-like phosphoesterase domain, ApaH type	1/28
Ets domain	1/28
Marvel domain	1/28
High mobility group box domain	2/55
Sushi/SCR/CCP domain	2/55
Peptidase, metallopeptidase	1/29
Ricin B, lectin domain	1/29
S100/CaBP-9k-type, calcium binding, subdomain	1/29
JmjC domain	1/32
SNF2-related, N-terminal domain	1/32
Immunoglobulin I-set	5/134
G-patch domain	1/34
Dual specificity protein phosphatase domain	1/36
RNA helicase, DEAD-box type, Q motif	1/37
SPRY domain	3/92
MAGE homology domain	1/38
SOCS box domain	1/38
DEAD/DEAH box helicase domain	2/67
B30.2/SPRY domain	3/96
Thioredoxin domain	1/40
SPRY-associated	1/48
Helicase, C-terminal	3/108
AAA+ ATPase domain	4/135
Helicase superfamily 1/2, ATP-binding domain	3/110
C-type lectin-like	2/86
Immunoglobulin subtype	17/460
Histone H2A/H2B/H3	1/66

Butyrophylin-like, SPRY domain	1/70
Intermediate filament, rod domain	1/74
Major facilitator superfamily domain	2/115
Serine proteases, trypsin domain	1/121
Immunoglobulin-like domain	20/689
Immunoglobulin V-set domain	7/402
GPCR, rhodopsin-like, 7TM	14/728
Krueppel-associated box	3/380

P.value	Adjusted.P.value	Old.P.value	Old.Adjusted
1,42144E-10	1,52236E-07	0	0
2,04148E-08	1,09321E-05	0	0
6,43229E-06	0,002296328	0	0
0,000211584	0,056651707	0	0
0,000385926	0,082665249	0	0
0,000545548	0,097380356	0	0
0,001020845	0,156189281	0	0
0,001266489	0,169551196	0	0
0,001266489	0,150712174	0	0
0,001266489	0,135640957	0	0
0,002285911	0,222564646	0	0
0,003463707	0,309135889	0	0
0,004878214	0,401889766	0	0
0,00630859	0,482607135	0	0
0,00630859	0,450433326	0	0
0,009102597	0,609305116	0	0
0,009778786	0,616063506	0	0
0,010483678	0,623778847	0	0
0,012068057	0,680257344	0	0
0,01250999	0,669909951	0	0
0,02088453	1	0	0
0,021233575	1	0	0
0,022060742	1	0	0
0,02396506	1	0	0
0,02396506	1	0	0
0,02396506	0,987176108	0	0
0,024584124	0,975170238	0	0
0,026564546	1	0	0
0,026564546	0,981056171	0	0
0,026564546	0,948354299	0	0
0,028490455	0,984299271	0	0
0,032833178	1	0	0
0,034729741	1	0	0
0,034729741	1	0	0
0,034729741	1	0	0
0,034729741	1	0	0
0,034729741	1	0	0
0,039153343	1	0	0
0,039153343	1	0	0
0,04097051	1	0	0
0,046390758	1	0	0
0,046390758	1	0	0
0,046390758	1	0	0
0,046447585	1	0	0
0,04698191	1	0	0
0,053862291	1	0	0
0,054236191	1	0	0
0,058618048	1	0	0
0,060539704	1	0	0
0,060539704	1	0	0
0,060539704	1	0	0
0,060539704	1	0	0

0,060539704	1	0	0
0,060539704	1	0	0
0,060539704	1	0	0
0,060539704	1	0	0
0,062669827	1	0	0
0,066556032	1	0	0
0,068652616	1	0	0
0,07523604	1	0	0
0,07523604	1	0	0
0,07523604	1	0	0
0,07523604	1	0	0
0,07523604	1	0	0
0,07523604	1	0	0
0,081209097	1	0	0
0,087073446	1	0	0
0,090917569	1	0	0
0,090917569	1	0	0
0,090917569	1	0	0
0,090917569	1	0	0
0,0912632	1	0	0
0,0912632	1	0	0
0,099506996	1	0	0
0,103502001	1	0	0
0,107093844	1	0	0
0,107443713	1	0	0
0,107443713	1	0	0
0,107443713	1	0	0
0,107443713	1	0	0
0,107443713	1	0	0
0,107443713	1	0	0
0,112800229	1	0	0
0,112800229	1	0	0
0,112819132	1	0	0
0,112819132	1	0	0
0,117912758	1	0	0
0,118458914	1	0	0
0,121626858	1	0	0
0,1242241	1	0	0
0,1242241	1	0	0
0,124685754	1	0	0
0,130200832	1	0	0
0,134629558	1	0	0
0,136044545	1	0	0
0,142525984	1	0	0
0,142525984	1	0	0
0,148230982	1	0	0
0,149700503	1	0	0
0,149700503	1	0	0
0,149700503	1	0	0
0,160752832	1	0	0
0,160856918	1	0	0
0,160856918	1	0	0
0,160856918	1	0	0

0,160856918	1	0	0
0,173579701	1	0	0
0,173579701	1	0	0
0,179580553	1	0	0
0,179580553	1	0	0
0,179580553	1	0	0
0,179580553	1	0	0
0,179580553	1	0	0
0,179580553	1	0	0
0,179580553	1	0	0
0,18668155	1	0	0
0,18668155	1	0	0
0,187324757	1	0	0
0,198607672	1	0	0
0,198607672	1	0	0
0,200028832	1	0	0
0,213592618	1	0	0
0,215877432	1	0	0
0,217857207	1	0	0
0,217857207	1	0	0
0,217857207	1	0	0
0,232733649	1	0	0
0,232733649	1	0	0
0,232733649	1	0	0
0,232733649	1	0	0
0,232733649	1	0	0
0,232733649	1	0	0
0,232733649	1	0	0
0,232733649	1	0	0
0,232733649	1	0	0
0,232733649	1	0	0
0,232733649	1	0	0
0,232733649	1	0	0
0,232733649	1	0	0
0,232733649	1	0	0
0,232733649	1	0	0
0,232733649	1	0	0
0,232733649	1	0	0
0,232733649	1	0	0
0,232733649	1	0	0
0,232733649	1	0	0
0,232733649	1	0	0
0,232733649	1	0	0
0,232733649	1	0	0
0,232733649	1	0	0
0,232733649	1	0	0
0,232733649	1	0	0
0,232733649	1	0	0
0,232733649	1	0	0
0,232733649	1	0	0
0,232733649	1	0	0
0,232733649	1	0	0
0,232733649	1	0	0

0,232733649	1	0	0
0,232733649	1	0	0
0,232733649	1	0	0
0,234278972	1	0	0
0,234278972	1	0	0
0,235404591	1	0	0
0,237255632	1	0	0
0,237255632	1	0	0
0,237255632	1	0	0
0,239614479	1	0	0
0,241257655	1	0	0
0,241257655	1	0	0
0,255304963	1	0	0
0,255304963	1	0	0
0,255304963	1	0	0
0,256736405	1	0	0
0,256736405	1	0	0
0,256736405	1	0	0
0,256736405	1	0	0
0,257018171	1	0	0
0,257018171	1	0	0
0,258616455	1	0	0
0,269461014	1	0	0
0,272334438	1	0	0
0,272334438	1	0	0
0,272334438	1	0	0
0,272334438	1	0	0
0,272334438	1	0	0
0,272334438	1	0	0
0,272334438	1	0	0
0,272334438	1	0	0
0,272334438	1	0	0
0,272334438	1	0	0
0,272334438	1	0	0
0,272334438	1	0	0
0,272334438	1	0	0
0,272334438	1	0	0
0,272334438	1	0	0
0,272334438	1	0	0
0,272334438	1	0	0
0,272334438	1	0	0
0,272334438	1	0	0
0,272334438	1	0	0
0,272334438	1	0	0
0,272334438	1	0	0
0,272334438	1	0	0
0,272334438	1	0	0
0,272334438	1	0	0
0,272334438	1	0	0
0,272334438	1	0	0
0,285336573	1	0	0
0,294916861	1	0	0

0,295159786	1	0	0
0,295710622	1	0	0
0,29800186	1	0	0
0,29800186	1	0	0
0,301696743	1	0	0
0,303624962	1	0	0
0,309893194	1	0	0
0,309893194	1	0	0
0,309893194	1	0	0
0,309893194	1	0	0
0,309893194	1	0	0
0,309893194	1	0	0
0,309893194	1	0	0
0,309893194	1	0	0
0,309893194	1	0	0
0,309893194	1	0	0
0,309893194	1	0	0
0,309893194	1	0	0
0,309893194	1	0	0
0,309893194	1	0	0
0,309893194	1	0	0
0,309893194	1	0	0
0,309893194	1	0	0
0,309893194	1	0	0
0,309893194	1	0	0
0,309893194	1	0	0
0,309893194	1	0	0
0,309893194	1	0	0
0,312340461	1	0	0
0,315101353	1	0	0
0,315101353	1	0	0
0,315101353	1	0	0
0,315434093	1	0	0
0,319982781	1	0	0
0,326695464	1	0	0
0,331779558	1	0	0
0,334368124	1	0	0
0,334368124	1	0	0
0,334368124	1	0	0
0,334368124	1	0	0
0,345515118	1	0	0
0,345515118	1	0	0
0,345515118	1	0	0
0,345515118	1	0	0
0,345515118	1	0	0
0,345515118	1	0	0
0,345515118	1	0	0
0,345515118	1	0	0
0,345515118	1	0	0
0,345515118	1	0	0
0,345515118	1	0	0
0,345515118	1	0	0
0,345515118	1	0	0

0,345515118	1	0	0
0,345515118	1	0	0
0,345515118	1	0	0
0,345515118	1	0	0
0,351038378	1	0	0
0,353472123	1	0	0
0,353472123	1	0	0
0,353472123	1	0	0
0,355341865	1	0	0
0,355373883	1	0	0
0,355373883	1	0	0
0,355373883	1	0	0
0,369659561	1	0	0
0,372378865	1	0	0
0,374805161	1	0	0
0,379299997	1	0	0
0,379299997	1	0	0
0,379299997	1	0	0
0,379299997	1	0	0
0,379299997	1	0	0
0,379299997	1	0	0
0,379299997	1	0	0
0,379299997	1	0	0
0,379299997	1	0	0
0,379299997	1	0	0
0,379299997	1	0	0
0,379299997	1	0	0
0,379299997	1	0	0
0,379299997	1	0	0
0,379299997	1	0	0
0,379299997	1	0	0
0,379299997	1	0	0
0,384840943	1	0	0
0,386449512	1	0	0
0,39105785	1	0	0
0,396212785	1	0	0
0,398037404	1	0	0
0,409482246	1	0	0
0,409482246	1	0	0
0,409482246	1	0	0
0,409482246	1	0	0
0,409482246	1	0	0
0,411342481	1	0	0
0,411342481	1	0	0
0,411342481	1	0	0
0,411342481	1	0	0
0,411342481	1	0	0
0,411342481	1	0	0
0,411342481	1	0	0
0,411342481	1	0	0
0,411342481	1	0	0
0,411342481	1	0	0
0,411342481	1	0	0

0,411342481	1	0	0
0,411342481	1	0	0
0,412098092	1	0	0
0,417467868	1	0	0
0,426053988	1	0	0
0,426053988	1	0	0
0,42762859	1	0	0
0,427755717	1	0	0
0,439891843	1	0	0
0,441732347	1	0	0
0,441732347	1	0	0
0,441732347	1	0	0
0,441732347	1	0	0
0,441732347	1	0	0
0,441732347	1	0	0
0,441732347	1	0	0
0,441732347	1	0	0
0,441732347	1	0	0
0,441732347	1	0	0
0,441732347	1	0	0
0,445476512	1	0	0
0,453599363	1	0	0
0,463008482	1	0	0
0,463008482	1	0	0
0,463008482	1	0	0
0,463008482	1	0	0
0,467165176	1	0	0
0,467165176	1	0	0
0,470554753	1	0	0
0,470554753	1	0	0
0,470554753	1	0	0
0,470554753	1	0	0
0,470554753	1	0	0
0,470554753	1	0	0
0,470554753	1	0	0
0,470554753	1	0	0
0,470554753	1	0	0
0,480578809	1	0	0
0,497067219	1	0	0
0,497890471	1	0	0
0,497890471	1	0	0
0,497890471	1	0	0
0,497890471	1	0	0
0,497890471	1	0	0
0,497890471	1	0	0
0,497890471	1	0	0
0,497890471	1	0	0
0,497890471	1	0	0
0,497890471	1	0	0
0,497890471	1	0	0
0,51735239	1	0	0
0,523816115	1	0	0
0,523816115	1	0	0

0,523816115	1	0	0
0,523816115	1	0	0
0,532531736	1	0	0
0,548404355	1	0	0
0,548404355	1	0	0
0,548404355	1	0	0
0,548404355	1	0	0
0,548404355	1	0	0
0,548404355	1	0	0
0,571724118	1	0	0
0,571724118	1	0	0
0,571724118	1	0	0
0,571724118	1	0	0
0,571724118	1	0	0
0,571724118	1	0	0
0,571724118	1	0	0
0,571724118	1	0	0
0,571724118	1	0	0
0,57590642	1	0	0
0,581423931	1	0	0
0,590548963	1	0	0
0,593840785	1	0	0
0,593840785	1	0	0
0,593840785	1	0	0
0,593840785	1	0	0
0,593840785	1	0	0
0,593840785	1	0	0
0,597203617	1	0	0
0,604619888	1	0	0
0,604811304	1	0	0
0,614816365	1	0	0
0,614816365	1	0	0
0,614816365	1	0	0
0,614816365	1	0	0
0,614816365	1	0	0
0,614816365	1	0	0
0,614816365	1	0	0
0,617822284	1	0	0
0,63470968	1	0	0
0,63470968	1	0	0
0,63470968	1	0	0
0,63470968	1	0	0
0,63470968	1	0	0
0,644460603	1	0	0
0,645324095	1	0	0
0,648391557	1	0	0
0,653576518	1	0	0
0,653576518	1	0	0
0,653576518	1	0	0
0,664475808	1	0	0
0,668943677	1	0	0
0,671469795	1	0	0
0,671469795	1	0	0

0,671469795	1	0	0
0,671469795	1	0	0
0,688439703	1	0	0
0,688439703	1	0	0
0,704533848	1	0	0
0,704533848	1	0	0
0,704533848	1	0	0
0,704533848	1	0	0
0,704533848	1	0	0
0,704533848	1	0	0
0,707371709	1	0	0
0,709482066	1	0	0
0,716425533	1	0	0
0,719797384	1	0	0
0,719797384	1	0	0
0,734273137	1	0	0
0,734273137	1	0	0
0,737253058	1	0	0
0,74800173	1	0	0
0,74800173	1	0	0
0,74800173	1	0	0
0,761021692	1	0	0
0,761021692	1	0	0
0,766227792	1	0	0
0,772963985	1	0	0
0,773369566	1	0	0
0,773369566	1	0	0
0,773369566	1	0	0
0,773369566	1	0	0
0,783756324	1	0	0
0,783756324	1	0	0
0,785080013	1	0	0
0,785080013	1	0	0
0,785080013	1	0	0
0,816707165	1	0	0
0,816707165	1	0	0
0,827220937	1	0	0
0,835163993	1	0	0
0,851763893	1	0	0
0,859426581	1	0	0
0,860011638	1	0	0
0,866693525	1	0	0
0,866693525	1	0	0
0,8669476	1	0	0
0,878663982	1	0	0
0,880120803	1	0	0
0,921608729	1	0	0
0,922037538	1	0	0
0,922602908	1	0	0
0,927706934	1	0	0
0,940729373	1	0	0
0,944522004	1	0	0
0,969874831	1	0	0

0,975645263	1	0	0
0,980311144	1	0	0
0,983807014	1	0	0
0,9983853	1	0	0
0,998770151	1	0	0
0,999896116	1	0	0
0,999993308	1	0	0
0,999995482	1	0	0

Odds.Ratio	Combined.Score
7,856693903	178,1441228
5,068574836	89,74928654
3,201887428	38,27593738
8,074935401	68,32111488
5,813953488	45,6968961
2,085723225	15,671539
3,781433163	26,04320132
11,62790698	77,57566148
11,62790698	77,57566148
11,62790698	77,57566148
1,804330393	10,97211593
5,963029219	33,78303964
2,981514609	15,87053117
7,26744186	36,81572007
7,26744186	36,81572007
6,45994832	30,3565599
4,55996352	21,10141337
2,627775588	11,97723216
4,306632214	19,02322652
5,813953488	25,4722545
3,69139904	14,28108748
4,84496124	18,66362223
1,742008536	6,64394323
7,751937984	28,92370822
7,751937984	28,92370822
7,751937984	28,92370822
2,642706131	9,792955667
4,472271914	16,22619769
4,472271914	16,22619769
4,472271914	16,22619769
3,370407819	11,99253845
3,22997416	11,03461162
6,45994832	21,70645267
6,45994832	21,70645267
6,45994832	21,70645267
6,45994832	21,70645267
6,45994832	21,70645267
3,875968992	12,55918396
3,875968992	12,55918396
1,674801416	5,350827645
3,63372093	11,15790341
3,63372093	11,15790341
3,63372093	11,15790341
2,27998176	6,998246266
5,53709856	16,93240671
2,76854928	8,087831292
3,41997264	9,967191743
2,153316107	6,108339039
4,84496124	13,58747994
4,84496124	13,58747994
4,84496124	13,58747994
4,84496124	13,58747994

4,84496124	13,58747994
4,84496124	13,58747994
4,84496124	13,58747994
4,84496124	13,58747994
3,22997416	8,946625228
2,583979328	7,001837457
1,937984496	5,191271411
4,306632214	11,14179549
4,306632214	11,14179549
4,306632214	11,14179549
4,306632214	11,14179549
4,306632214	11,14179549
4,306632214	11,14179549
2,906976744	7,298627913
1,286628711	3,140664942
3,875968992	9,293806274
3,875968992	9,293806274
3,875968992	9,293806274
3,875968992	9,293806274
2,76854928	6,627928123
2,76854928	6,627928123
1,875468867	4,327695658
1,368836633	3,104746429
1,667083438	3,724347396
3,523608175	7,860423421
3,523608175	7,860423421
3,523608175	7,860423421
3,523608175	7,860423421
3,523608175	7,860423421
3,523608175	7,860423421
2,527805865	5,516018479
2,527805865	5,516018479
2,153316107	4,698469725
2,153316107	4,698469725
1,788908766	3,824347528
1,695736434	3,617326474
2,095118374	4,413990065
2,42248062	5,052490523
2,42248062	5,052490523
3,22997416	6,724672735
1,654377009	3,372740619
1,52998776	3,067974735
2,325581395	4,639006775
2,981514609	5,80867905
2,981514609	5,80867905
2,236135957	4,268746712
1,937984496	3,68046246
1,937984496	3,68046246
1,937984496	3,68046246
2,153316107	3,936019159
2,76854928	5,058804027
2,76854928	5,058804027
2,76854928	5,058804027

2,76854928	5,058804027
2,07641196	3,636043216
2,07641196	3,636043216
2,583979328	4,437032068
2,583979328	4,437032068
2,583979328	4,437032068
2,583979328	4,437032068
2,583979328	4,437032068
2,583979328	4,437032068
2,583979328	4,437032068
2,004811548	3,364777581
2,004811548	3,364777581
1,291989664	2,163968346
2,42248062	3,915755568
2,42248062	3,915755568
1,937984496	3,118786362
1,875468867	2,895132655
1,362645349	2,088995923
2,27998176	3,474499418
2,27998176	3,474499418
2,27998176	3,474499418
3,875968992	5,650622543
3,875968992	5,650622543
3,875968992	5,650622543
3,875968992	5,650622543
3,875968992	5,650622543
3,875968992	5,650622543
3,875968992	5,650622543
3,875968992	5,650622543
3,875968992	5,650622543
3,875968992	5,650622543
3,875968992	5,650622543
3,875968992	5,650622543
3,875968992	5,650622543
3,875968992	5,650622543
3,875968992	5,650622543
3,875968992	5,650622543
3,875968992	5,650622543
3,875968992	5,650622543
3,875968992	5,650622543
3,875968992	5,650622543
3,875968992	5,650622543
3,875968992	5,650622543
3,875968992	5,650622543
3,875968992	5,650622543
3,875968992	5,650622543
3,875968992	5,650622543
3,875968992	5,650622543
3,875968992	5,650622543
3,875968992	5,650622543
3,875968992	5,650622543
3,875968992	5,650622543
3,875968992	5,650622543

3,875968992	5,650622543
3,875968992	5,650622543
3,875968992	5,650622543
1,61498708	2,343738185
1,61498708	2,343738185
1,33143973	1,925860438
2,153316107	3,097797377
2,153316107	3,097797377
2,153316107	3,097797377
1,435544071	2,050996245
1,761804087	2,505091275
1,761804087	2,505091275
1,70998632	2,334638367
1,70998632	2,334638367
1,70998632	2,334638367
2,03998368	2,773776792
2,03998368	2,773776792
2,03998368	2,773776792
2,03998368	2,773776792
1,550387597	2,106369756
1,550387597	2,106369756
1,21124031	1,638092519
1,661129568	2,178291625
3,22997416	4,201306255
3,22997416	4,201306255
3,22997416	4,201306255
3,22997416	4,201306255
3,22997416	4,201306255
3,22997416	4,201306255
3,22997416	4,201306255
3,22997416	4,201306255
3,22997416	4,201306255
3,22997416	4,201306255
3,22997416	4,201306255
3,22997416	4,201306255
3,22997416	4,201306255
3,22997416	4,201306255
3,22997416	4,201306255
3,22997416	4,201306255
3,22997416	4,201306255
3,22997416	4,201306255
3,22997416	4,201306255
3,22997416	4,201306255
3,22997416	4,201306255
3,22997416	4,201306255
3,22997416	4,201306255
3,22997416	4,201306255
3,22997416	4,201306255
3,22997416	4,201306255
3,22997416	4,201306255
3,22997416	4,201306255
1,094219603	1,372245307
1,176913662	1,4370843

1,184794015	1,445731177
1,84569952	2,248752181
1,571338781	1,902350018
1,571338781	1,902350018
1,321353066	1,583420887
1,435544071	1,711114005
2,76854928	3,243431827
2,76854928	3,243431827
2,76854928	3,243431827
2,76854928	3,243431827
2,76854928	3,243431827
2,76854928	3,243431827
2,76854928	3,243431827
2,76854928	3,243431827
2,76854928	3,243431827
2,76854928	3,243431827
2,76854928	3,243431827
2,76854928	3,243431827
2,76854928	3,243431827
2,76854928	3,243431827
2,76854928	3,243431827
2,76854928	3,243431827
2,76854928	3,243431827
2,76854928	3,243431827
2,76854928	3,243431827
2,76854928	3,243431827
2,76854928	3,243431827
2,76854928	3,243431827
1,52998776	1,780387798
1,761804087	2,034638719
1,761804087	2,034638719
1,761804087	2,034638719
1,40944327	1,62622342
1,291989664	1,472206841
1,490757305	1,667750218
1,244577199	1,373122748
1,68520391	1,846162327
1,68520391	1,846162327
1,68520391	1,846162327
1,68520391	1,846162327
2,42248062	2,574415889
2,42248062	2,574415889
2,42248062	2,574415889
2,42248062	2,574415889
2,42248062	2,574415889
2,42248062	2,574415889
2,42248062	2,574415889
2,42248062	2,574415889
2,42248062	2,574415889
2,42248062	2,574415889
2,42248062	2,574415889
2,42248062	2,574415889
2,42248062	2,574415889

2,42248062	2,574415889
2,42248062	2,574415889
2,42248062	2,574415889
2,42248062	2,574415889
1,336541032	1,399170972
1,61498708	1,679506874
1,61498708	1,679506874
1,61498708	1,679506874
1,2749898	1,319200012
1,418037436	1,467080051
1,418037436	1,467080051
1,418037436	1,467080051
1,38427464	1,377592472
1,550387597	1,531540293
1,291989664	1,26789271
2,153316107	2,087484578
2,153316107	2,087484578
2,153316107	2,087484578
2,153316107	2,087484578
2,153316107	2,087484578
2,153316107	2,087484578
2,153316107	2,087484578
2,153316107	2,087484578
2,153316107	2,087484578
2,153316107	2,087484578
2,153316107	2,087484578
2,153316107	2,087484578
2,153316107	2,087484578
2,153316107	2,087484578
2,153316107	2,087484578
2,153316107	2,087484578
2,153316107	2,087484578
1,14749082	1,095767861
1,226572466	1,166168737
1,490757305	1,399671698
1,10499116	1,023005098
1,321353066	1,217242729
1,435544071	1,281742365
1,435544071	1,281742365
1,435544071	1,281742365
1,435544071	1,281742365
1,435544071	1,281742365
1,937984496	1,721568072
1,937984496	1,721568072
1,937984496	1,721568072
1,937984496	1,721568072
1,937984496	1,721568072
1,937984496	1,721568072
1,937984496	1,721568072
1,937984496	1,721568072
1,937984496	1,721568072
1,937984496	1,721568072
1,937984496	1,721568072

1,937984496	1,721568072
1,937984496	1,721568072
1,291989664	1,145340919
1,181697863	1,03226945
1,263902932	1,078348343
1,263902932	1,078348343
1,38427464	1,17594164
1,16746054	0,991410994
1,237011381	1,015866394
1,761804087	1,43948402
1,761804087	1,43948402
1,761804087	1,43948402
1,761804087	1,43948402
1,761804087	1,43948402
1,761804087	1,43948402
1,761804087	1,43948402
1,761804087	1,43948402
1,761804087	1,43948402
1,761804087	1,43948402
1,761804087	1,43948402
1,336541032	1,080741454
1,21124031	0,957535043
1,291989664	0,994844838
1,291989664	0,994844838
1,291989664	0,994844838
1,291989664	0,994844838
1,18652112	0,903028463
1,18652112	0,903028463
1,61498708	1,217446632
1,61498708	1,217446632
1,61498708	1,217446632
1,61498708	1,217446632
1,61498708	1,217446632
1,61498708	1,217446632
1,61498708	1,217446632
1,61498708	1,217446632
1,61498708	1,217446632
1,162790698	0,852051221
1,21124031	0,84669333
1,490757305	1,03961712
1,490757305	1,03961712
1,490757305	1,03961712
1,490757305	1,03961712
1,490757305	1,03961712
1,490757305	1,03961712
1,490757305	1,03961712
1,490757305	1,03961712
1,490757305	1,03961712
1,490757305	1,03961712
1,490757305	1,03961712
1,035564235	0,682468966
1,38427464	0,895092168
1,38427464	0,895092168

1,38427464	0,895092168
1,38427464	0,895092168
1,076658053	0,678416004
1,291989664	0,776152959
1,291989664	0,776152959
1,291989664	0,776152959
1,291989664	0,776152959
1,291989664	0,776152959
1,291989664	0,776152959
1,21124031	0,6772029
1,21124031	0,6772029
1,21124031	0,6772029
1,21124031	0,6772029
1,21124031	0,6772029
1,21124031	0,6772029
1,21124031	0,6772029
1,21124031	0,6772029
1,21124031	0,6772029
1,047559187	0,578053737
1,002405774	0,543579722
1,01999184	0,537232486
1,13999088	0,594099447
1,13999088	0,594099447
1,13999088	0,594099447
1,13999088	0,594099447
1,13999088	0,594099447
1,13999088	0,594099447
0,968992248	0,499512748
0,968992248	0,487553588
0,993838203	0,499740374
1,076658053	0,523720552
1,076658053	0,523720552
1,076658053	0,523720552
1,076658053	0,523720552
1,076658053	0,523720552
1,076658053	0,523720552
1,076658053	0,523720552
0,945358291	0,455241473
1,01999184	0,463675624
1,01999184	0,463675624
1,01999184	0,463675624
1,01999184	0,463675624
1,01999184	0,463675624
0,91414363	0,401621313
0,92284976	0,404210609
0,908430233	0,393586946
0,968992248	0,412108201
0,968992248	0,412108201
0,968992248	0,412108201
0,915583227	0,374250877
0,880902044	0,354171434
0,92284976	0,367558366
0,92284976	0,367558366

0,92284976	0,367558366
0,92284976	0,367558366
0,880902044	0,328864994
0,880902044	0,328864994
0,842601955	0,295095132
0,842601955	0,295095132
0,842601955	0,295095132
0,842601955	0,295095132
0,842601955	0,295095132
0,842601955	0,295095132
0,830564784	0,287540694
0,880902044	0,302343252
0,818866689	0,273076457
0,80749354	0,265492182
0,80749354	0,265492182
0,775193798	0,239437363
0,775193798	0,239437363
0,775193798	0,23629774
0,745378652	0,216420683
0,745378652	0,216420683
0,745378652	0,216420683
0,717772036	0,196018818
0,717772036	0,196018818
0,755058895	0,201053892
0,781445361	0,201240015
0,69213732	0,177878081
0,69213732	0,177878081
0,69213732	0,177878081
0,69213732	0,177878081
0,704721635	0,171710442
0,704721635	0,171710442
0,668270516	0,161701175
0,668270516	0,161701175
0,668270516	0,161701175
0,605620155	0,122622744
0,605620155	0,122622744
0,723128543	0,137165527
0,56999544	0,102671668
0,538329027	0,086372691
0,523779594	0,079347307
0,631951466	0,095304194
0,50999592	0,072965042
0,50999592	0,072965042
0,578502835	0,08259675
0,605620155	0,078338619
0,484496124	0,061868268
0,40374677	0,032959673
0,538329027	0,043695813
0,574217628	0,04625688
0,528541226	0,039661417
0,450694069	0,027537307
0,716211662	0,040878708
0,293634015	0,008981752

0,276854928	0,006826196
0,261889797	0,005207748
0,337040782	0,005502368
0,160164008	0,000258826
0,562549926	0,000692277
0,337459987	3,50585E-05
0,372689326	2,49399E-06
0,152998776	6,91188E-07

Genes
PCDHA13;PCDHA12;PCDHA11;PCDHA10;PCDHA1;PCDHA5;PCDHA4;PCDHA3;PCDHA2;PCDHA9;PCDHA8
PCDH11Y;PCDHB15;PCDHA13;PCDHA12;PCDHA11;PCDHA10;PCDHA1;PCDHA5;PCDHA4;PCDHA3;PCDH
PCDH11Y;PCDHB15;PCDHA13;PCDHA12;PCDHA11;PCDHA10;CDH6;CDH2;PCDHA1;PCDHA5;PCDHA4;PC
PARP11;TIPARP;DTX1;TRIP12;DTX4
PHLPP2;PDP2;PPM1L;PPM1H;PPM1K;PPM1E
RBM28;CPSF7;CELF1;CELF3;SRSF1;HNRNPR;ELAVL2;U2SURP;TIAL1;RBM3;SART3;PABPN1;TRA2B;RAV
SHC4;EPS8;SHC2;RABGAP1L;NOS1AP;APBB2;TNS1;APPL1
SORT1;SORCS1;SORL1
SORT1;SORCS1;SORL1
SORT1;SORCS1;SORL1
RNF180;LTN1;XIAP;DTX1;BMI1;DTX4;NEURL1B;RNF214;ZNRF2;ZNRF3;TRIM3;TRIM24;LONRF3;UNKL;RNF
AP3M2;AP5M1;SGIP1;AP2M1
FNBP4;DRP2;TCERG1;ITCH;SMURF2;FRMPD4;HECW2;APBB2
NBEAL1;WDFY3;WDFY4
ABHD17B;ABHD17C;ABHD15
NBEAL1;WDFY3;WDFY4
PARP11;TNKS2;TIPARP;TNKS
MBNL2;TIPARP;PRR3;RC3H1;HELZ;MBNL3;UNKL;ZC3H14
KCMF1;DRP2;CREBBP;ZZZ3
BHLHB9;ARMCX6;GPRASP2
PDE1B;PDE3B;PDE5A;PDE7B
NFIA;SMAD9;SMAD7
ZBTB14;KCNC2;ZBTB16;KLHL32;RHOBTB3;ZBTB10;ZBTB20;ZBTB34;KLHL23;ZBTB44;KCNRG;BTBD9;BAC
DTX1;DTX4
DCUN1D5;DCUN1D1
SNX1;SNX6
USP13;SIK3;SIK2;UBE2K;UBL7;MARK1
ITPK1;CARNS1;RIMKLA
MAF;BACH1;NFE2L1
SEPT10;SEPT11;SEPT12
ERI1;HNRNPU;DEK;PIAS1
PDE1B;PDE3B;PDE5A;PDE7B
RABGAP1L;KIF1B
CRISPLD1;CRISPLD2
CRBN;LONRF3
VAPA;VAPB
SCN5A;SCN1A
TRPS1;GATA6;GATAD2B
OTUD4;TNFAIP3;YOD1
RAB3C;RAB2B;RALA;ARL3;DIRAS2;RND3;GTPBP4;RAB11A;RAP2C;ARL5B;RAP2A;RAP1A;RAP2B;ARL5A
ATP2B4;ATP2B2;ATP2B1
CYTH3;PSD4;PSD3
SYT5;SYTL4;SYT7
PRDM8;MECOM;SETD9;PRDM6;NSD1;PRDM16
PCGF3;BMI1
ITCH;SMURF2;HECW2;TRIP12
LTN1;MARCH7;RNF165
CREBZF;MAF;CREB1;CREBL2;BACH1;NFE2L1
CRBN;YPEL2
LNPEP;TRHDE
EFNA3;EFNB3
SMAD9;SMAD7

SMAD9;SMAD7
EPS8;TNS1
TCEA3;TCEANC2
UHRF1BP1L;VPS13C
ATP2B4;ATP2B2;ATP2B1
PLXNA2;EBF1;NFATC3;PLXNA3
CXADR;IGLON5;IL1RAPL1;PTPRM;EMB;IL6R;FGFR1
BMPR2;BMPR1A
ATL3;ATL2
NXF1;G3BP1
NXF1;G3BP1
PAX5;PAX2
PLXNA2;PLXNA3
NAA38;SNRPD3;LSM5
DYRK3;BMPR2;ROCK1;RPS6KA6;ERBB4;PTK2B;MAPK1;PIM3;MAP3K9;PRKG1;MARK1;LYN;SRPK2;MAP3I
CBX6;CBX5
SCN5A;SCN1A
MOCS3;UBA6
IL6R;IL13RA1
GJC1;GJA3;GJA9
GJC1;GJA3;GJA9
PTP4A1;MTMR3;PTPRM;PTPN11;PTPN4;TNS1
ROCK1;SWAP70;AGAP2;GAB1;IRS4;GAB3;PLEKHA3;CDC42BPA;CYTH3;ACAP2;ARHGAP20;PSD4;PSD3;E
PHC2;TNKS2;STIM2;TNKS;SFMBT2;CNKSR3;BFAR;PPP1R9A
DPYSL5;DPYSL3
FZD3;FZD5
ATL3;ATL2
ATL3;ATL2
TCEA3;TCEANC2
ENAH;SPRED1
FZD3;SFRP1;FZD5
NSD1;DNMT3A;GLYR1
TDRKH;HNRNPK;FUBP1;IGF2BP1
GRM5;GRID1;NPR3;GRIN2B
FBXW4;FBXO40;FBXL17;FBXO3;BTRC;FBXO30
USP13;USP47;USP37;USP15;USP9X;USP2;USP1
TDRKH;HNRNPK;FUBP1;IGF2BP1
KAT2B;NAA30;NAT8L
ZDHHC18;ZDHHC20;ZDHHC21
FBXO40;FBXO30
USP13;USP47;USP37;USP15;USP9X;USP2;USP1
NCOA2;HEYL;ID2;MYOD1;ID4;TFEB;MXD1;HIF1A;ATOH1
SOSTDC1;SLIT2;CTGF
LNPEP;TRHDE
TCEA3;TCEANC2
CBX6;CBX5;CDYL2
ADAM19;ADAMTS5;ADAMTS2;ADAM12
ADAM19;ADAMTS5;ADAMTS2;ADAM12
CDC34;UBE2G1;UBE2K;UBE2J1
NUDT5;DCP2;NUDT4
MTMR3;MTMR9
CADM3;NRXN3
ATP8A2;ATP11A

ATP8A2;ATP11A
ADAM19;ADAMTS5;ADAM12
NPAT;TBL1XR1;SSBP2
AP3M2;AP2M1
ADH1B;SORD
ADH1B;SORD
TRIM3;TRIM24
CRISPLD1;CRISPLD2
PGRMC1;CYB5R4
DIAPH2;FMN1
ACAP2;AGAP2;AGFG2
MTMR3;WDFY3;WDFY2
LYN;DOCK5;SH3KBP1;SORBS3;EPS8;NCKIPSD;FCHSD2;EFS;SH3PXD2A;MAP3K9;SRGAP2;CRK;ARHGEF
PRRX1;SHOX2
ADH1B;SORD
TDRKH;PHF20;SMNDC1
SEMA3A;PLXNA2;PLXNA3
SYT5;ITCH;RIMS3;SMURF2;HECW2;SYTL4;SYT7;RAB11FIP5;CC2D1B
AIPL1;FKBP6
EDEM3;RNF148
GPAM;TMEM68
YPEL2
SRD5A1
PHC2
NSD1
PGM3
DFFA
SLC7A14
DAND5
FRRS1L
DOCK5
NBEAL1
CTDSPL2
EFNA3
CYB5R4
GFPT1
EPOR
HK2
HK2
MTSS1L
ING4
FRMPD2
MARK1
KIF1B
NEURL1B
NXF1
MCTS1
PCDH11Y
PLEKHM3
SERTAD2
SFMBT2
CBFA2T2
TFAP2B

MEF2A
PDHB
YTHDC1
FRMPD4;FRMPD2;PTK2B;PTPN4
FRMPD4;FRMPD2;PTK2B;PTPN4
LYN;ERBB4;NEK7;PTK2B;MAP3K9;RAF1;FRK;FGFR1;BMPR1A
GRID1;GRIN2B
GRID1;GRIN2B
NRP2;PTPRM
MAPRE3;PARVA;ACTN4;HOOK3;SMTNL2;MAPRE2
CBX6;CBX5;CDYL2
HS3ST3B1;CHST7;CHST3
MTMR3;WDFY3;WDFY2
SNIP1;KIF1B;CEP170
NCOA2;HIF1A;KCNH1
ALDH6A1;ALDH2
GNL1;GTPBP4
NCOA2;HIF1A
ADAM19;ADAM12
FRMPD4;FRMPD2;PTK2B;PTPN4
SNX1;SH3PXD2A;SNX8;SNX6
FBXW4;UTP15;WDR37;NBEAL1;PPWD1;DCAF7;STXBP5L;TBL1XR1;RBBP5;STRN;WDFY3;WDFY2;BTRC;W
RAPGEF6;PRKG1;KCNH1
TCEA3
BTG1
AGO1
BROX
DIP2B
CNPY1
E2F1
DRP2
DRP2
ACTN4
ELMO1
TCERG1
PFAS
ERBB4
PDCD4
KIF1B
EEF2K
GTPBP4
OCLN
KCNH1
PRICKLE1
PSD4
RAB11FIP5
SMC1A
IL17RD
SMC1A
U2SURP
MSL2
PRDM8;ZNF275;TSHZ3;PHF20;PLAG1;ZNF292;PRDM6;GLIS3;ZBTB20;ZBTB44;ZNF25;IKZF3;ZBTB4;MECO
DLX1;TSHZ3;ONECUT2;PRRX1;DLX6;SHOX2;SIX1;HOXD12;POU3F1;PHOX2B;GBX2;HOXA3;LHX6;LHX4;H

TENM3;SUSD1;NRXN3;SELE;LRP8;FBLN5;EREG;LRP6;ADAM19;EFEMP1;FRAS1;NRG3;ADAM12;SLIT2
STAU1;NKRF
ZCCHC24;CNBP;SREK1IP1
CCND3;RBL1;CCNYL1
ING4;PHF20;NSD1;TRIM24;RSF1;PHF8
TPBG;OMD;CHAD;SLIT2
TNFAIP3
PIAS1
ACBD5
NCEH1
ANO6
REN
ONECUT2
CTBS
GNL1
PCYT1B
KCMF1
ERBB4
PDE5A
BMPR1A
GPCPD1
CTBS
SIX1
USP15
RAF1
ERBB4
NRXN3
SENP2
PTPRM;PTPN11;PTPN4
ADAM19;ADAM12
RAPGEF6;SOS2
DENND5B;DENND6A
RPS6KA6;ROCK1;CDC42BPA;PRKG1
DLX1;GBX2;DLX6;HOXA3;HOXD12;HOXC8
ARHGAP20;RASSF8;RAPGEF6
SHC4;LYN;SHC2;PTPN11;CRK;FRK;TNS1
ADAMTS5;ADAMTS2
NRP2;BTBD9
FCHSD2;SRGAP2
INHBA;GDF6
ZCCHC24
U2SURP
CDH2
MYO1C
MYO1C
CUL3
DIAPH2
EPC1
CTDSPL2
FADS6
FUT11
FAM84A
MME

MME
AGO1
TNS1
PDHB
TBCEL;UBL3;DDI2;UBL7
CDH6;CDH2
PPWD1;PPIC
CNOT6;ANGEL2
ING4;NSD1;TRIM24;RSF1;PHF8
KAT2B;CREBBP;TRIM24
PCDHB15;PCDHAC2;PCDHAC1
FRAS1;CTGF;DGCR2
SEMA3A;PLXNA2;PLXNA3
FCHSD2;SRGAP2
KLHL7;KLHL32;KLHL23;BTBD9
SLC4A10
CUL3
DCX
SLIT2
DIAPH2
DIAPH2
KPNA1
ZZZ3
FRAS1
MGAM
AGO1
METAP2
NSUN3
SLC24A2
TNS1
TGM2
TGM2
SIPA1L1;FRMPD4;FRMPD2;CNKSR3;PPP1R9A;SYNJ2BP;PTPN4;RAPGEF6;LNX2
LYN;ERBB4;PTK2B;FRK;FGFR1
MIER3;SMARCA1
NECAB3;KCNIP2;SWAP70;CALML4;ACTN4;S100B;HPCAL4;LETM1;CHP1;MICU3;CALM1;SLC25A12;SLC25
ZZZ3;MIER3;SMARCA1
DTX1;DTX4
SESTD1;PRUNE2
EFEMP1;FBLN5
FRMPD2;PTPN4
SNAP23;VTI1A
ITM2C
SLC4A10
DLST
CUL3
ST13
CTIF
MACROD2
REN
PAPOLG
NFATC3
NFATC3

N4BP2
GAPVD1
KIF5C;KIF26B;KIF1B
PTP4A1;DUSP19;PTPRM;PTPN11;PTPN4
RXRA;RORB;ESR1
GABRA1;GABRB1;GABRA3
ABHD4;ABHD2
TPBG;CHAD;LGI2;SLIT2;LRIG2
GABRA1;GABRB1;GABRA3
DOCK5
DOCK5
DOCK5
E2F1
HDAC5
SORCS1
PRTFDC1
NKRF
SIPA1L1
DIAPH2
TGM2
FRMPD2;PTPN4
RXRA;RORB;ESR1
ACSL6;DIP2B
ZMAT3;ZNF318
RPS6KA6;CDC42BPA
RAPGEF6;SOS2
ABCA1;ABCA2;TAP2
FOXJ2;FOXP2;FOXO1
POGK
IRS4
MIEF1
HEYL
CYB5R4
NAALADL2
SYTL4
RANBP3
STXBP5L
KCNC2;KCNRG;KCTD16
CCND3;CCNYL1
LCOR
MIER3
PTPN4
MMP2
GRM5
MB
POGK
HIF1A
ASPRV1
PPP2CA
UBXN7
TTC9;ST13;TTC33;TMTC3;LONRF3;AIPL1;FKBP6
USP13
ZMAT3

ERI1
MAP3K2
TBC1D19;RABGAP1L;TBC1D25
ARID5B
DENND5B
CDC42BPA
RNF217
GAPVD1
RNASE6
DENND5B
DENND5B
SH3GL2
CYB5R4
ADAT2
CTGF
POU3F1
NSD1
TMEM56
INHBA;GDF6
NRXN3;SLIT2;COL19A1
TRIM3;RC3H1
TGFBR3
ALAS2
CCND3
DHX40
POU3F1
SULF2
TRIM41;TRIM3;TRIM24;TRIM44
ADCYAP1R1;FZD3;FZD5
MYO1C;MYH10
CA12
CDC42BPA
TMED8
GRAMD4
DHX40
SFRP1
ADAMTS2
FNDC3B;PTPRM;NDNF;FNDC3A;HCFC2;L1CAM;IL6R;SORL1;EPOR;IL13RA1
ADCY9
CYB5R4
MYH10
MYH10
DENND5B
KCNC2;SCN5A;KCNH1;SCN1A;TPCN1
PTP4A1;DUSP19
ROCK1;RAF1;CDC42BPA
CLCN5
CTGF
MMP2
KANK2;ANKRD13B;ACAP2;GABPB2;ANKRD33B;TNKS2;TNKS;AGAP2;ASB7;ANKRD40;KANK4;GLS
ARHGAP20;ARHGAP31;SRGAP2
CBFA2T2
RAF1

DENND5B
JMY
GRM5
SERBP1
MMP2
SFRP1
MMP2
MMP2
IL1RAPL1
TUBD1
ARHGEF10;SOS2;ARHGEF5
CADM3;CXADR;IGSF3;IGLON5;IL1RAPL1;LRIG2;L1CAM;IL6R;MYPN;JAM2;FGFR1
LHX6;PRICKLE1;LHX4
PSMD11
MOCS3
GALNT7
LOXL4
NRP2;LRP12
TERF2IP
TIAL1
LOXL4
TAP2
ADCYAP1R1
XRCC5;COL6A5;ANTXR2
EFEMP1;SUSD1;SLIT2;LRP8;FBLN5
CADM3
PPP2CA
ELK4
OCLN
TFAM;SOX6
SUSD1;SELE
MMP2
GALNT7
S100B
PHF8
SMARCA1
EMB;LRIG2;L1CAM;MYPN;FGFR1
NKRF
DUSP19
DDX19B
TRIM41;SPRYD7;HNRNPU
MAGEE1
ASB7
DHX40;DDX19B
TRIM41;SPRYD7;HNRNPU
TXNL1
TRIM41
DHX40;DDX19B;SMARCA1
ABCA1;ABCA2;RUVBL1;TAP2
DHX40;DDX19B;SMARCA1
SELE;DGCR2
CADM3;CXADR;IGSF3;SEMA3A;PTPRM;L1CAM;MYPN;IGLON5;IL1RAPL1;EMB;PTGFRN;LRIG2;SCN3B;MF
SOS2

TRIM41
BFSP2
SLC22A17;SLC17A6
KLK13
CADM3;CXADR;IGSF3;SEMA3A;PTPRM;L1CAM;MYPN;IL18BP;IGLON5;IL1RAPL1;EMB;PTGFRN;CD47;LRI
CADM3;CXADR;IGSF3;PTGFRN;SCN3B;MPZL3;JAM2
GPR27;CHRM3;PTGFR;GPR37;NPFFR1;GPR75;ADRA2B;ADRA2A;TSHR;GNRHR;S1PR1;S1PR3;AGTR2;G
POGK;ZNF25;ZNF597

3;PCDHA7;PCDHAC2;PCDHAC1;PCDHA6
A2;PCDHA9;PCDHA8;PCDHAC2;PCDHA7;PCDHAC1;PCDHA6
CDHA3;PCDHA2;PCDHA9;PCDHA8;PCDHAC2;PCDHA7;PCDHAC1;PCDHA6

ER2;G3BP1;IGF2BP1;RBMS1;HNRNPC;RBM12;TARDBP;RBM7;SRSF10;PPARGC1B;SRSF9

F111;TRIM41;PCGF3;MSL2;RC3H1;BFAR;RBX1;RNF126;RNF148;RFWD3;RLIM;RNF165;LNX2

CH1;ZBTB4;KLHL7;KCTD16

K2;CSNK1G3;MAP2K1;CSNK1A1;NEK7;DYRK1A;HIPK1;CDC42BPA;HIPK2;MAPK10;CAMK4;CDK1;SIK3

[illegible]LMO1;KIF1B;SOS2;ARHGEF5;PLEKHM3;APPL1

=5;FRK;SH3GL2

VDFY4;DTL

M;ZNF629;ZMAT3;SALL4;TRPS1;PRDM16;ZNF800;HIVEP2;ZNF687;ZNF423;ZNF148;ZNF367;ZNF366;K
IOXC8

A24

ᵖZL3;IL6R;JAM2;FGFR1

G2;SCN3B;MPZL3;IL6R;JAM2;CD244;FGFR1

PR135

[illegible];SIK2;RAF1;TRIB2;FRK;FGFR1;BMPR1A

(CMF1;KLF10;ZNF462;ZBTB14;EGR4;BCL11B;ZBTB16;ZBTB10;ZFP91;ZBTB34;YOD1;ZFY;ZFX;ZNF70;S

SP1;ZFP62;SP4;ZNF236;ZNF597
