# Supplementary material for: Circulating miR-330-3p in Late Pregnancy is Associated with Pregnancy Outcomes Among Lean Women with GDM
Source: Sci Rep. 2020 Jan 22;10:908. doi: 10.1038/s41598-020-57838-6 (PMC6976655; doi:10.1038/s41598-020-57838-6)
Supplement: Supplementary file 1 — Supplementary data. [file 41598_2020_57838_MOESM1_ESM.zip › Supplimentary File_EnrichR_Analysis KEA_2015.pdf]

Term	Overlap	P.value	Adjusted.P.value	Old.P.value	
GSK3B	54/527	1,25017E-06	0,000535072		0
CSNK1G1	5/12	0,000211584	0,045279049		0
PRKCB	27/250	0,00023972	0,034200085		0
CDK2	48/553	0,000308248	0,032982508		0
KSR2	3/4	0,000526873	0,045100315		0
AKT1	23/210	0,000551991	0,03937537		0
MAPK12	5/15	0,000704559	0,043078736		0
CSNK1A1	12/84	0,00121882	0,065206886		0
CDK4	5/17	0,001331814	0,063335171		0
CSNK2A1	27/286	0,001876122	0,080298009		0
RPS6KA3	30/332	0,002136273	0,083120453		0
MAP2K1	5/19	0,002296337	0,081902689		0
RAF1	3/6	0,002435749	0,080192341		0
KSR1	3/6	0,002435749	0,074464317		0
MAP3K9	2/2	0,002660068	0,075900619		0
MAPK10	13/105	0,002911061	0,077870891		0
SRC	24/259	0,004122345	0,103786091		0
PRKACA	33/393	0,004226882	0,100505869		0
GSK3A	6/34	0,007229061	0,16284411		0
MAPK9	14/131	0,00774857	0,165819391		0
RPS6KA2	4/16	0,007791259	0,158793276		0
CAMK4	4/17	0,009778786	0,190241833		0
EGFR	9/72	0,011504736	0,214088138		0
CDK6	3/10	0,01250999	0,223094817		0
CSNK1G3	3/10	0,01250999	0,214171024		0
CSNK1A1L	3/10	0,01250999	0,205933677		0
PRKAA2	5/28	0,013219845	0,20955903		0
PRKCA	34/442	0,013464194	0,205809816		0
MAP3K8	4/19	0,014673856	0,216565869		0
NTRK3	2/4	0,014885615	0,212368101		0
MAP3K2	2/4	0,014885615	0,205517517		0
MAPK3	18/202	0,01718588	0,229861145		0
RPS6KA1	8/65	0,018234756	0,236499266		0
ATM	15/161	0,01957453	0,246408786		0
AKT2	5/31	0,020133068	0,246198664		0
CSNK1G2	3/12	0,021233575	0,252443613		0
MAPK13	3/12	0,021233575	0,245620812		0
ABL1	10/94	0,023006143	0,259121826		0
MOS	2/5	0,02396506	0,263001167		0
DYRK3	2/5	0,02396506	0,256426138		0
MAPK8	19/225	0,024278112	0,253439804		0
CHEK1	13/137	0,024919108	0,253937574		0
SGK3	3/13	0,026564546	0,264409902		0
MAP3K1	3/13	0,026564546	0,258400586		0
CDK5	10/97	0,027920253	0,265552625		0
CHUK	4/23	0,028490455	0,265085104		0
PRKCE	6/46	0,029929579	0,27255021		0
INSR	11/113	0,031637499	0,282101033		0
HIPK2	3/14	0,03254043	0,284230694		0
CSNK1E	12/128	0,032953233	0,282079677		0
MAPK14	29/396	0,037174622	0,311975261		0
PRKACG	15/176	0,039136624	0,322124521		0

MAPK11	3/15	0,039153343	0,316181716	0
MAP2K4	2/7	0,04698191	0,372375136	0
CSNK2A2	18/229	0,050380078	0,392048604	0
PKMYT1	1/1	0,051599784	0,394369776	0
TSSK4	1/1	0,051599784	0,387451008	0
MAP3K15	1/1	0,051599784	0,380770818	0
AAK1	1/1	0,051599784	0,374317076	0
WEE2	1/1	0,051599784	0,368078458	0
TRIM33	1/1	0,051599784	0,362044385	0
CDK1	34/494	0,054052295	0,373135195	0
HCK	5/41	0,058454216	0,39711753	0
SGK1	7/68	0,060534578	0,404824994	0
DYRK1A	3/18	0,062669827	0,412656709	0
CDK14	5/43	0,069121735	0,448243978	0
SGK2	2/10	0,090917569	0,580786858	0
CDK7	3/21	0,0912632	0,574421319	0
MAPKAPK2	5/47	0,093435321	0,579569818	0
JAK2	4/34	0,096102152	0,587596017	0
MAPK4	4/34	0,096102152	0,579320017	0
RPS6KB1	4/34	0,096102152	0,571273906	0
PRKG1	9/106	0,096881166	0,568015602	0
ARAF	1/2	0,10053959	0,581499252	0
TLK1	1/2	0,10053959	0,573745929	0
EEF2K	1/2	0,10053959	0,56619664	0
FRK	1/2	0,10053959	0,558843437	0
MAP3K13	1/2	0,10053959	0,551678778	0
CDK15	4/36	0,112819132	0,611222642	0
PRKCZ	5/50	0,114167849	0,610797991	0
CDK18	4/37	0,121626858	0,642670314	0
PRKCG	4/37	0,121626858	0,634832871	0
CDK11A	4/38	0,130718162	0,674064738	0
STK11	3/25	0,136044545	0,693179346	0
PRKAA1	5/53	0,136889868	0,689280747	0
PRKDC	13/180	0,139417514	0,693845305	0
FYN	9/115	0,139821705	0,687858503	0
SYK	4/39	0,140080408	0,681300167	0
PKN1	2/13	0,142525984	0,685405855	0
SGK494	2/13	0,142525984	0,677790234	0
CDK3	2/13	0,142525984	0,67034199	0
SGK223	2/13	0,142525984	0,663055664	0
MAPKAPK3	1/3	0,146956321	0,676315112	0
TAOK1	1/3	0,146956321	0,669120271	0
MKNK2	1/3	0,146956321	0,6620769	0
CAMKK2	1/3	0,146956321	0,655180265	0
ABL2	2/14	0,160856918	0,709760424	0
CSNK1D	6/72	0,166763522	0,728314157	0
MAPK1	21/326	0,174898629	0,756127405	0
CAMK2A	7/90	0,182500436	0,781101866	0
CDC7	1/4	0,190979919	0,809301041	0
MARK1	1/4	0,190979919	0,801366717	0
MAP3K4	1/4	0,190979919	0,793586458	0
MAP3K3	1/4	0,190979919	0,785955819	0
MAP2K7	1/4	0,190979919	0,778470525	0

SRPK1	1/4	0,190979919	0,771126464	0
WEE1	1/4	0,190979919	0,763919675	0
NEK9	1/4	0,190979919	0,756846344	0
CDK8	1/4	0,190979919	0,7499028	0
MAPK7	2/16	0,198607672	0,772764396	0
AURKB	6/77	0,206052961	0,794510516	0
BTK	2/17	0,217857207	0,832525755	0
ERBB2	2/17	0,217857207	0,82515827	0
LCK	7/96	0,226572868	0,850641995	0
BRAF	1/5	0,232733649	0,866173929	0
MET	1/5	0,232733649	0,858706912	0
ERBB4	1/5	0,232733649	0,851367537	0
TRPM7	1/5	0,232733649	0,844152558	0
NLK	1/5	0,232733649	0,837058839	0
RPS6KA4	1/5	0,232733649	0,830083349	0
MATK	1/5	0,232733649	0,823223156	0
MKNK1	1/5	0,232733649	0,816475425	0
PAK1	4/48	0,234278972	0,815214636	0
LYN	6/82	0,248252399	0,856871182	0
RPS6KB2	1/6	0,272334438	0,932473116	0
PAK6	1/6	0,272334438	0,925072535	0
GRK1	1/6	0,272334438	0,9177885	0
MAPKAPK5	1/6	0,272334438	0,910618277	0
CAMK1	2/20	0,276239439	0,916515349	0
IGF1R	6/86	0,283614281	0,933745478	0
AURKA	3/36	0,283701185	0,926901582	0
PRKCD	7/104	0,290153472	0,940800652	0
RPS6KL1	2/21	0,295710622	0,951610121	0
RPS6KC1	2/21	0,295710622	0,944508553	0
FGFR3	1/7	0,309893194	0,982476201	0
UHMK1	1/7	0,309893194	0,975252111	0
CAMKK1	1/7	0,309893194	0,968133482	0
PDGFRA	1/7	0,309893194	0,961118022	0
BMX	1/7	0,309893194	0,954203504	0
STK38	1/7	0,309893194	0,947387765	0
PAK4	1/7	0,309893194	0,940668703	0
RPS6KA6	2/23	0,334368124	1	0
PAK3	1/8	0,345515118	1	0
FER	1/8	0,345515118	1	0
DYRK1B	1/8	0,345515118	1	0
PRKD2	1/8	0,345515118	1	0
PTK2B	1/8	0,345515118	1	0
ATR	4/58	0,351038378	1	0
ROCK2	2/24	0,353472123	1	0
PRKD1	3/41	0,355373883	1	0
DAPK1	1/9	0,379299997	1	0
PRKG2	1/9	0,379299997	1	0
MELK	1/9	0,379299997	1	0
TYK2	1/9	0,379299997	1	0
MAP3K7	1/9	0,379299997	1	0
TEC	1/9	0,379299997	1	0
CAMK2B	2/26	0,39105785	1	0
CDC42BPA	1/10	0,411342481	1	0

IKBKE	1/10	0,411342481	1	0
EIF2AK2	1/11	0,441732347	1	0
PTK6	1/11	0,441732347	1	0
ALK	1/11	0,441732347	1	0
CDK9	1/11	0,441732347	1	0
MARK2	1/12	0,470554753	1	0
DYRK2	1/12	0,470554753	1	0
PRKCH	1/12	0,470554753	1	0
CAMK2D	2/31	0,480209569	1	0
ITK	1/13	0,497890471	1	0
PRKACB	6/111	0,513185032	1	0
ROCK1	2/35	0,545485494	1	0
JAK1	1/15	0,548404355	1	0
CSK	1/15	0,548404355	1	0
RET	1/15	0,548404355	1	0
MTOR	4/78	0,577020093	1	0
FGFR1	1/17	0,593840785	1	0
PTK2	1/17	0,593840785	1	0
PIM1	1/18	0,614816365	1	0
PLK3	1/18	0,614816365	1	0
PDGFRB	1/18	0,614816365	1	0
CHEK2	1/23	0,704533848	1	0
RPS6KA5	1/25	0,734273137	1	0
IKBKB	1/25	0,734273137	1	0
ADRBK1	1/25	0,734273137	1	0
PDPK1	1/27	0,761021692	1	0
CAMK2G	1/32	0,816707165	1	0
PDK1	1/33	0,826180149	1	0
PRKCQ	1/35	0,84368393	1	0
PLK1	2/78	0,916272592	1	0

Old.Adjusted	Odds.Ratio	Combined.Score	Genes
0	1,985790565	26,99132709	JPH1;AFF1;MED14;CCND3;GRM5;SIN3
0	8,074935401	68,32111488	APC;NFATC3;CTNNB1;FOXO1;LRP6
0	2,093023256	17,44752153	PDE3B;ADD3;ADD2;BCLAF1;GRM5;EEI
0	1,682156525	13,599574	BTG1;NUFIP2;SH3KBP1;ADD3;JPH1;GI
0	14,53488372	109,7173157	MAP2K1;MAPK1;RAF1
0	2,122554448	15,92335788	SRPK2;CREBBP;RANBP3;PFKFB3;PHF
0	6,45994832	46,88590967	MEF2A;C20ORF112;CCND3;EEF2K;RXI
0	2,76854928	18,57661089	SLC24A2;GJC1;CREB1;APC;PPP1R1B;
0	5,6999544	37,74061275	RBL1;CELF1;SOD2;RUNX3;FOXO1
0	1,829565783	11,4870176	HIF1A;HSPH1;MYH10;MEF2A;ABCA1;N
0	1,75119081	10,76753363	SLC24A2;GABRB1;PDE1B;PDE3B;RSF
0	5,0999592	30,98959607	MAP2K1;MYOD1;MAPK1;PARVA;RAF1
0	9,689922481	58,30911925	MAP2K1;MAPK1;RAF1
0	9,689922481	58,30911925	MAP2K1;MAPK1;RAF1
0	19,37984496	114,9109193	MAP2K1;MAP3K9
0	2,399409376	14,01072131	MEF2A;USP47;PCYT1B;NFATC3;GRM5
0	1,795815749	9,861422578	LYN;GAB1;ATP2B4;L1CAM;GRIN2B;ESI
0	1,627315226	8,895377911	HDAC5;GABRB1;PFKFB3;SRSF1;PDE3
0	3,41997264	16,85925491	RCAN1;ARHGAP31;CREB1;CTNNB1;F(
0	2,071128469	10,06619595	PCYT1B;CXADR;GFPT1;NFATC3;ADD2
0	4,84496124	23,5210893	EEF2K;CREB1;L1CAM;ESR1
0	4,55996352	21,10141337	HDAC5;CREBBP;CREB1;CAMK4
0	2,42248062	10,81636742	ENAH;PPP2CA;USP9X;GAB1;CTNNB1;
0	5,813953488	25,4722545	CELF1;CTNNB1;RUNX1
0	5,813953488	25,4722545	APC;NFATC3;CTNNB1
0	5,813953488	25,4722545	APC;NFATC3;CTNNB1
0	3,4606866	14,97105531	HDAC5;PFKFB3;EEF2K;HAS2;RAF1
0	1,490757305	6,421767209	GPM6A;GABRB1;PFKFB3;SNAP23;AG/
0	4,07996736	17,22434882	MAP2K1;CREB1;CDK1;MAPK1
0	9,689922481	40,76899225	SHC2;MAPK1
0	9,689922481	40,76899225	MAP3K2;MAPK1
0	1,726916878	7,017615406	MCTS1;NCOA2;MAP2K1;RANBP3;GAT/
0	2,385211688	9,55140324	SIPA1L1;EEF2K;CREB1;RANBP3;L1CAI
0	1,805575618	7,10227875	USP15;SMC1A;HIF1A;CREB1;RRM2B;S
0	3,125781445	12,20740068	TMOD3;XIAP;CTNNB1;SIK2;ESR1
0	4,84496124	18,66362223	APC;NFATC3;CTNNB1
0	4,84496124	18,66362223	CCND3;MARCKS;EEF2K
0	2,061685634	7,776665826	ENAH;ACAP2;MYOD1;DCX;CTNNB1;PT
0	7,751937984	28,92370822	MAP2K1;MYOD1
0	7,751937984	28,92370822	DYRK3;CREB1
0	1,636520241	6,084876952	MEF2A;CUL3;FUT11;ESR1;PAX2;ADD2;
0	1,83896339	6,789674235	FYTTD1;MAP2K1;SENP2;ADD3;PHF8;A
0	4,472271914	16,22619769	ITCH;CREB1;FOXO1
0	4,472271914	16,22619769	SRPK2;MAP2K1;MAPK1
0	1,997922161	7,149370558	MEF2A;MAPK10;MAP2K1;SIPA1L1;DPY
0	3,370407819	11,99253845	CREBBP;CTNNB1;ESR1;PIAS1
0	2,527805865	8,869838268	GRM5;OCLN;CREB1;NRIP1;RAB11A;F(
0	1,88653358	6,514978053	LYN;RBM3;PPP2CA;FUBP1;ADAM12;D'
0	4,15282392	14,22455135	CREB1;ZBTB4;RUNX1
0	1,816860465	6,200337743	OCLN;PGRMC1;CDC34;APC;G3BP1;PF
0	1,41923107	4,672291692	AGAP2;PPP1R9A;JPH1;GIGYF2;FOXO'
0	1,651691332	5,352630438	ABCA1;GABRB1;PFKFB3;GFPT1;PDE3

0	3,875968992	12,55918396	CCND3;EEF2K;E2F1
0	5,53709856	16,93240671	MAPK10;RXRA
0	1,523306591	4,551883012	XRCC5;TPM3;L1CAM;ZFY;PPP1R9A;ZF
0	19,37984496	57,44646892	CDK1
0	19,37984496	57,44646892	CREB1
0	19,37984496	57,44646892	MAP2K1
0	19,37984496	57,44646892	AP2M1
0	19,37984496	57,44646892	CDK1
0	19,37984496	57,44646892	TRIM24
0	1,333835483	3,891869552	FUT11;HIF1A;GIGYF2;FOXO1;PHF8;BC
0	2,363395727	6,710889265	ENAH;ACAP2;ELMO1;PTK2B;RAF1
0	1,99498404	5,595013601	GRM5;CREB1;PPP1R1B;MAPK1;BSN;F
0	3,22997416	8,946625228	RCAN1;DYRK1A;FOXO1
0	2,253470344	6,02101599	CCND3;MAP2K1;MYOD1;PPP1R1B;ESI
0	3,875968992	9,293806274	CREB1;FOXO1
0	2,76854928	6,627928123	E2F1;CDK1;ESR1
0	2,061685634	4,887196592	EEF2K;CREB1;RCSD1;ESR1;PIAS1
0	2,27998176	5,340500606	MAPK1;PTPN11;RAF1;EPOR
0	2,27998176	5,340500606	RCAN1;RXRA;DCX;ESR1
0	2,27998176	5,340500606	EEF2K;PDCD4;MXD1;ESR1
0	1,645458534	3,840944735	ABCA1;GNA13;GABRB1;CREB1;APC;D
0	9,689922481	22,25972573	MAP2K1
0	9,689922481	22,25972573	RAD9A
0	9,689922481	22,25972573	EEF2K
0	9,689922481	22,25972573	FRK
0	9,689922481	22,25972573	MAP2K1
0	2,153316107	4,698469725	MAP2K1;MYOD1;PPP1R1B;ESR1
0	1,937984496	4,205592161	GRM5;CREBBP;SP1;MYH10;ADD2
0	2,095118374	4,413990065	MAP2K1;MYOD1;PPP1R1B;ESR1
0	2,095118374	4,413990065	GRM5;OCLN;EIF4E;GRIN2B
0	2,03998368	4,150778681	MAP2K1;MYOD1;PPP1R1B;ESR1
0	2,325581395	4,639006775	SIK3;SIK2;MARK1
0	1,82828726	3,635692848	HDAC5;EEF2K;PFKFB3;GFPT1;RAF1
0	1,399655469	2,757716183	PHC2;GABRB1;XRCC5;HNRNPU;SMC1
0	1,516683519	2,983903746	ITCH;C21ORF59;AGAP2;PTK2B;CTNNE
0	1,987676406	3,906854855	SWAP70;NRIP1;PTK2B;EMB
0	2,981514609	5,80867905	HDAC5;MARCKS
0	2,981514609	5,80867905	CREB1;FOXO1
0	2,981514609	5,80867905	YLPM1;SLC4A10
0	2,981514609	5,80867905	CREB1;FOXO1
0	6,45994832	12,38772527	EEF2K
0	6,45994832	12,38772527	MARK1
0	6,45994832	12,38772527	EIF4E
0	6,45994832	12,38772527	CAMK4
0	2,76854928	5,058804027	PTPN11;CRK
0	1,61498708	2,892730146	RBL1;APC;AGAP2;NFATC3;CTNNB1;HI
0	1,248394921	2,176637388	MCTS1;NCOA2;MAP2K1;GATA6;GAB1;I
0	1,507321275	2,563957584	GJC1;GABRB1;CREB1;CAMK4;GFPT1;(
0	4,84496124	8,021254818	CLSPN
0	4,84496124	8,021254818	DCX
0	4,84496124	8,021254818	MAPK10
0	4,84496124	8,021254818	RCAN1
0	4,84496124	8,021254818	MAPK10

0	4,84496124	8,021254818	SRSF1
0	4,84496124	8,021254818	CDK1
0	4,84496124	8,021254818	NEK7
0	4,84496124	8,021254818	E2F1
0	2,42248062	3,915755568	MEF2A;RXRA
0	1,510117789	2,38541536	RBM3;HDAC5;DIAPH2;CBX5;MAPRE3;[illegible]
0	2,27998176	3,474499418	EFS;EMB
0	2,27998176	3,474499418	CDK1;CTNNB1
0	1,413113695	2,098033897	PPP2CA;ACAP2;EFS;G3BP1;MAPK1;E[illegible]
0	3,875968992	5,650622543	MAP2K1
0	3,875968992	5,650622543	GAB1
0	3,875968992	5,650622543	ERBB4
0	3,875968992	5,650622543	EEF2K
0	3,875968992	5,650622543	FOXO1
0	3,875968992	5,650622543	CREB1
0	3,875968992	5,650622543	LYN
0	3,875968992	5,650622543	EIF4E
0	1,61498708	2,343738185	MAP2K1;RAF1;ESR1;FOXO1
0	1,418037436	1,975764768	LYN;CDK1;PTK2B;EMB;RAF1;EPOR
0	3,22997416	4,201306255	EEF2K
0	3,22997416	4,201306255	RAF1
0	3,22997416	4,201306255	OCLN
0	3,22997416	4,201306255	EEF2K
0	1,937984496	2,493192356	HDAC5;CAMK4
0	1,352082207	1,703813049	EFS;CDK1;PTK2B;PTPN11;CRK;FGFR1
0	1,61498708	2,034615246	RALA;MAPRE3;ESR1
0	1,304412642	1,614008827	HDAC5;CREBBP;GRM5;HNRNPK;TBL1
0	1,84569952	2,248752181	CREB1;ESR1
0	1,84569952	2,248752181	CREB1;ESR1
0	2,76854928	3,243431827	DYRK1A
0	2,76854928	3,243431827	FAM64A
0	2,76854928	3,243431827	CAMK4
0	2,76854928	3,243431827	PTPN11
0	2,76854928	3,243431827	FGFR1
0	2,76854928	3,243431827	RAB11FIP5
0	2,76854928	3,243431827	CTNNB1
0	1,68520391	1,846162327	CREB1;ESR1
0	2,42248062	2,574415889	RAF1
0	2,42248062	2,574415889	CTNNB1
0	2,42248062	2,574415889	HDAC5
0	2,42248062	2,574415889	HDAC5
0	2,42248062	2,574415889	PTK2B
0	1,336541032	1,399170972	CREB1;SP1;E2F1;SMC1A
0	1,61498708	1,679506874	SORL1;SH3GL2
0	1,418037436	1,467080051	HDAC5;CREB1;CTNNB1
0	2,153316107	2,087484578	GRIN2B
0	2,153316107	2,087484578	PRKG1
0	2,153316107	2,087484578	SMAD7
0	2,153316107	2,087484578	IL13RA1
0	2,153316107	2,087484578	HDAC5
0	2,153316107	2,087484578	EMB
0	1,490757305	1,399671698	CREB1;GRIN2B
0	1,937984496	1,721568072	CDC42BPA

0	1,937984496	1,721568072	ESR1
0	1,761804087	1,43948402	CDK1
0	1,761804087	1,43948402	CTNNB1
0	1,761804087	1,43948402	PTPN11
0	1,761804087	1,43948402	NCOA2
0	1,61498708	1,217446632	HDAC5
0	1,61498708	1,217446632	DPYSL3
0	1,61498708	1,217446632	PTPN11
0	1,250312578	0,917145122	CREB1;SCN5A
0	1,490757305	1,03961712	EMB
0	1,047559187	0,698846441	RAP1A;HNRNPK;XK;TRIM3;RAF1;GRIN
0	1,107419712	0,671183906	MARCKS;RND3
0	1,291989664	0,776152959	PTPN11
0	1,291989664	0,776152959	LYN
0	1,291989664	0,776152959	MAPK1
0	0,993838203	0,546489952	EEF2K;DPYSL3;RC3H1;EIF4EBP2
0	1,13999088	0,594099447	FGFR1
0	1,13999088	0,594099447	ATP2B4
0	1,076658053	0,523720552	RUNX3
0	1,076658053	0,523720552	HIF1A
0	1,076658053	0,523720552	PTPN11
0	0,842601955	0,295095132	E2F1
0	0,775193798	0,239437363	CREB1
0	0,775193798	0,239437363	SNAP23
0	0,775193798	0,239437363	ADRA2A
0	0,717772036	0,196018818	MAP2K1
0	0,605620155	0,122622744	CREB1
0	0,587268029	0,112134385	MAP2K1
0	0,553709856	0,094118131	GRM5
0	0,496919102	0,043451287	CTNNB1;CLSPN

3A;PAPOLG;DPYSL3;KIF1B;BSN;FNBP4;MEF2A;MAP2K1;PCYT1B;CXADR;CBFA2T2;ADRA2A;RUNX1;C

F2K;ATXN7;EMB;CLSPN;BSN;EIF4E;ABCA1;PCYT1B;GFPT1;NFATC3;TFEB;PTPN11;GRIN2B;RAB11A;C
IGYF2;FOXO1;AFF1;AMOT;ADD2;NPAT;BCLAF1;RXRA;ATXN7;SIN3A;TRA2B;DPYSL3;E2F1;BSN;MARK

F20;CELF1;PDE3B;AGAP2;FAM129A;XIAP;ESR1;FOXO1;HK2;EEF2K;CREB1;TRA2B;S1PR1;PDCD4;CTN
RA
NFATC3;CTNNB1;HNRNPC;BSN;TARDBP;FOXO1;LRP6

ICOA2;CBX5;MME;DEK;L1CAM;ESR1;PIAS1;OCLN;ITCH;CDC34;XK;CREB1;SP1;SUB1;CDC37;CAPZA1
1;ADD3;FOXO1;BCLAF1;JPH3;EEF2K;ATXN7;TRA2B;E2F1;S1PR1;BSN;RBM7;MARK1;FNBP4;ABCA1;M

;;HSPH1;HNRNPK;RXRA;NFIA;SNPH;MYOD1;CCDC6;KIF1B
R1;PPP2CA;NCKIPSD;OCLN;BCLAF1;HNRNPK;EFS;SH3PXD2A;CDH2;FUBP1;ADAM12;G3BP1;DAG1;C
3B;PPP1R9A;ADD3;LRP6;ADD2;GNA13;GRM5;RAP1A;RXRA;EEF2K;TEAD1;APPL1;TGM2;ABCA1;CSNK
OXO1;LRP6
2;MAPK10;ADCY9;EEF2K;RXRA;PPP1R1B;DCX;CTNNB1;RAPGEF6;MARK1

CALM1;CRK;CCDC50;FGFR1

AP2;ADD3;FOXO1;PITPNC1;ADD2;GRM5;PLXNA2;E2F1;S1PR1;EIF4E;PRKG1;CREBBP;XRCC5;MMP2;C

A6;GAB1;ESR1;RUNX1;EPS8;RCAN1;ARHGAP31;HNRNPK;RXRA;SP1;CCDC6;BSN;RAF1;FGFR1
M;MXD1;ESR1;ADD2
SP1;C1QBP;RFWD3;DCX;E2F1;CCDC6;CTNNB1;BSN;ZNF148;RAD9A

TPN11;EMB;CRK;ESR1;RAD9A

;BCLAF1;ITCH;HNRNPK;RXRA;EEF2K;NFIA;ATXN7;SP1;FUBP1;PPP1R1B;DCX;CTNNB1;BSN
ADD2;DDX19B;SIN3A;CDK1;CCDC6;TRIP12;CLSPN;KPNA1

YSL3;PPP1R1B;AGAP2;DCX;CTNNB1;ADD2

GFR1
YRK1A;GAB1;PTK2B;CALM1;EPOR;FGFR1

PP1R1B;NFATC3;PDAP1;CTNNB1;KIF1B;BSN;LRP6
1;PGRMC1;CCND3;EEF2K;SIN3A;PAPOLG;TRA2B;PLXNA2;E2F1;ZNF148;FNBP4;MEF2A;NCOA2;YLPM
B;ATP2B1;ESR1;ADD2;GNA13;RAP1A;APC;PPP1R1B;PDE5A;RAF1;NFE2L1

FX;ESR1;OCLN;CDC34;HSPH1;XK;SP1;SIN3A;CDC37;PPP1R1B;CDK1;CTNNB1;HNRNPC

LAF1;GRM5;RXRA;EEF2K;TRA2B;USP1;PLXNA2;E2F1;KIF1B;MEF2A;MCTS1;MAP2K1;CXADR;NFATC3

FOXO1;MARK1

R1

CX;PDE5A;PRKG1;MARK1

1A;ENAH;ACAP2;DCX;E2F1;PTK2B;BSN;RAF1;RAD9A
31;PTPN11;SCN5A;GRIN2B;ADD2

F1A
L1CAM;HIF1A;SORBS3;ESR1;GIGYF2;FOXO1;RUNX1;RCAN1;RXRA;RBL1;SP1;E2F1;MAPK1;CEP170;F
CTNNB1;GRIN2B

DEK

SR1;CCDC50

I

XR1;ADRA2A;ADD2

ɿ2B

;REB1;RBL1;SNPH;DCX;TERF2IP;TNKS;CUL3;FUT11;ADD3;GIGYF2;FOXO1;LRP6;ADD2;BCLAF1;RXRA

OCLN;MARCKS;CREB1;APC;SP4;PPP1R1B;DCX;CTNNB1
1;FNBP4;CREBBP;PCYT1B;ZBTB16;GFPT1;NFATC3;SORBS3;ESR1;RUNX1;PUM2;RCAN1;MARCKS;AI

NNB1;SIK2;RAF1;MXD1;PPARGC1B

;PPP1R1B;CDK1;CTNNB1;HNRNPC;TARDBP;RAD9A
MAP2K1;RANBP3;GRIN2B;ESR1;PUM2;CREB1;SNPH;CDK1;DCX;TRIP12;RAF1

CDK1;CTNNB1;PTK2B;EMB;RAF1;TGM2
K1A1;GFPT1;ATP2B1;ESR1;MARCKS;ITCH;CREB1;APC;PPP1R1B;DCX;TFAM;CTNNB1;PDE5A;RAF1;NI

GFPT1;ATP2B4;PTPN11;ATP2B2;ATP2B1;L1CAM;SMC1A;GRIN2B;ESR1;ADRA2A;ENAH;MARCKS;OCLI

M1;SLC4A10;SMAD9;CBFA2T2;ESR1;RUNX1;RCAN1;CREB1;RBL1;ID2;TERF2IP;FGFR1

3;GAPVD1;CBFA2T2;ESR1;RUNX1;MAPK10;MARCKS;HNRNPK;SP1;FUBP1;ID2;MYOD1;PPP1R1B;CDK

RAF1;FGFR1

A;ATXN7;TRA2B;E2F1;GFPT1;NFATC3;YLPM1;SLC4A10;HELZ;ESR1;RCAN1;C20ORF112;MARCKS;ARH

DCY9;HNRNPK;RBL1;APC;SNPH;ID2;MYOD1;PPP1R1B;CCDC6;DCX;TFAM;TERF2IP;RAPGEF6;DTL;SF

FE2L1

N;RBL1;MYOD1;RAF1

‹1;DCX;RAD9A

ꟷGAP31;NFIA;APC;FUBP1;ID2;PPP1R1B;CCDC6;CTNNB1;NFE2L1

ꓤSF9;RAD9A
