# Supplementary material for: Circulating miR-330-3p in Late Pregnancy is Associated with Pregnancy Outcomes Among Lean Women with GDM
Source: Sci Rep. 2020 Jan 22;10:908. doi: 10.1038/s41598-020-57838-6 (PMC6976655; doi:10.1038/s41598-020-57838-6)
Supplement: Supplementary file 1 — Supplementary data. [file 41598_2020_57838_MOESM1_ESM.zip › Supplimentary File_EnrichR_Analysis KEGG_2019_Human.pdf]

Term	Overlap	P.value
TGF-beta signaling pathway	16/90	1,31565E-05
Thyroid hormone signaling pathway	18/116	2,70083E-05
cGMP-PKG signaling pathway	20/166	0,000365494
Neurotrophin signaling pathway	16/119	0,000406905
Wnt signaling pathway	19/158	0,00051929
Long-term potentiation	11/67	0,000590265
Renal cell carcinoma	11/69	0,000762684
Signaling pathways regulating pluripotency of stem cells	17/139	0,000825755
ErbB signaling pathway	12/85	0,001353898
Breast cancer	17/147	0,001541982
Chronic myeloid leukemia	11/76	0,001725691
Insulin signaling pathway	16/137	0,001888397
GnRH signaling pathway	12/93	0,002943556
Glioma	10/75	0,005038919
Melanogenesis	12/101	0,00579191
Mitophagy	9/65	0,00594332
cAMP signaling pathway	20/212	0,006885019
Phospholipase D signaling pathway	15/148	0,009609226
Gastric cancer	15/149	0,010191917
Pathways in cancer	40/530	0,01058531
Thyroid cancer	6/37	0,010964166
Estrogen signaling pathway	14/137	0,011300029
Axon guidance	17/181	0,012587856
Colorectal cancer	10/86	0,012982942
Longevity regulating pathway	11/102	0,016061171
Proteoglycans in cancer	18/201	0,016416756
Central carbon metabolism in cancer	8/65	0,018234756
Cellular senescence	15/160	0,018604954
Circadian rhythm	5/31	0,020133068
Parathyroid hormone synthesis, secretion and action	11/106	0,020850406
Spliceosome	13/134	0,021191986
D-Glutamine and D-glutamate metabolism	2/5	0,02396506
Apelin signaling pathway	13/137	0,024919108
Ubiquitin mediated proteolysis	13/137	0,024919108
Prolactin signaling pathway	8/70	0,027287786
Prostate cancer	10/97	0,027920253
Renin-angiotensin system	4/23	0,028490455
Focal adhesion	17/199	0,028920258
Cushing syndrome	14/155	0,029756494
Leukocyte transendothelial migration	11/112	0,029894298
Tight junction	15/170	0,030108716
Transcriptional misregulation in cancer	16/186	0,031551597
Alanine, aspartate and glutamate metabolism	5/35	0,032514034
HIF-1 signaling pathway	10/100	0,033538624
Long-term depression	7/60	0,03415701
Purine metabolism	12/129	0,034699522
Notch signaling pathway	6/48	0,035984006
Bacterial invasion of epithelial cells	8/74	0,036474739
Hippo signaling pathway	14/160	0,037501233
Gap junction	9/88	0,03751493
FoxO signaling pathway	12/132	0,040331335
Human cytomegalovirus infection	18/225	0,043739003

Hepatocellular carcinoma	14/168	0,052731059
Nicotine addiction	5/40	0,053499587
Oocyte meiosis	11/125	0,058431313
Bladder cancer	5/41	0,058454216
Calcium signaling pathway	15/188	0,062707986
Circadian entrainment	9/97	0,062791752
Renin secretion	7/69	0,06451613
Choline metabolism in cancer	9/99	0,069592032
Adrenergic signaling in cardiomyocytes	12/145	0,072073034
Vasopressin-regulated water reabsorption	5/44	0,074831689
Endometrial cancer	6/58	0,07753903
Glucagon signaling pathway	9/103	0,084520421
Arginine biosynthesis	3/21	0,0912632
Ras signaling pathway	17/232	0,092745382
Salivary secretion	8/90	0,092869453
Hedgehog signaling pathway	5/47	0,093435321
MicroRNAs in cancer	21/299	0,094750206
mRNA surveillance pathway	8/91	0,097483801
Oxytocin signaling pathway	12/153	0,097937036
Endocrine and other factor-regulated calcium reabsorption	5/48	0,10011571
Kaposi sarcoma-associated herpesvirus infection	14/186	0,101206653
Hepatitis C	12/155	0,105176893
Rap1 signaling pathway	15/206	0,113403303
Cholesterol metabolism	5/50	0,114167849
N-Glycan biosynthesis	5/50	0,114167849
Shigellosis	6/65	0,117912758
Acute myeloid leukemia	6/66	0,124400603
Non-small cell lung cancer	6/66	0,124400603
Huntington disease	14/193	0,125642471
Endocytosis	17/244	0,129222873
Aldosterone synthesis and secretion	8/98	0,133290542
Pancreatic secretion	8/98	0,133290542
Relaxin signaling pathway	10/130	0,134793004
Amphetamine addiction	6/68	0,13788388
Fc epsilon RI signaling pathway	6/68	0,13788388
Progesterone-mediated oocyte maturation	8/99	0,138890412
Alcoholism	13/180	0,139417514
Regulation of actin cytoskeleton	15/214	0,142059187
Vascular smooth muscle contraction	10/132	0,144565152
Adipocytokine signaling pathway	6/69	0,144871287
Regulation of lipolysis in adipocytes	5/55	0,15305853
B cell receptor signaling pathway	6/71	0,159315798
Sphingolipid signaling pathway	9/119	0,161585729
Adherens junction	6/72	0,166763522
Fluid shear stress and atherosclerosis	10/139	0,181488415
Chemokine signaling pathway	13/190	0,183899341
Morphine addiction	7/91	0,189585156
Pancreatic cancer	6/75	0,189948359
Insulin resistance	8/108	0,194239408
TNF signaling pathway	8/110	0,20763815
Type II diabetes mellitus	4/46	0,212063518
beta-Alanine metabolism	3/31	0,213592618
Autophagy	9/128	0,215877432

Cell adhesion molecules (CAMs)	10/145	0,216211076
Nitrogen metabolism	2/17	0,217857207
Nucleotide excision repair	4/47	0,2230987
Neomycin, kanamycin and gentamicin biosynthesis	1/5	0,232733649
Protein processing in endoplasmic reticulum	11/165	0,233034774
Amino sugar and nucleotide sugar metabolism	4/48	0,234278972
Dopaminergic synapse	9/131	0,235404591
Natural killer cell mediated cytotoxicity	9/131	0,235404591
Fructose and mannose metabolism	3/33	0,241257655
Human papillomavirus infection	20/330	0,260223217
Phosphonate and phosphinate metabolism	1/6	0,272334438
C-type lectin receptor signaling pathway	7/104	0,290153472
Human immunodeficiency virus 1 infection	13/212	0,300882855
Platelet activation	8/124	0,309766268
GABAergic synapse	6/89	0,310817292
Melanoma	5/72	0,314069009
p53 signaling pathway	5/72	0,314069009
Th17 cell differentiation	7/107	0,315004196
Terpenoid backbone biosynthesis	2/22	0,315101353
Pathogenic Escherichia coli infection	4/55	0,315434093
Fc gamma R-mediated phagocytosis	6/91	0,329187318
Hepatitis B	10/163	0,333050886
Histidine metabolism	2/23	0,334368124
Mannose type O-glycan biosynthesis	2/23	0,334368124
Proximal tubule bicarbonate reclamation	2/23	0,334368124
Th1 and Th2 cell differentiation	6/92	0,338425072
Pyrimidine metabolism	4/57	0,339152763
Human T-cell leukemia virus 1 infection	13/219	0,341971938
Viral carcinogenesis	12/201	0,343755004
Sulfur relay system	1/8	0,345515118
Lysine degradation	4/59	0,362926182
VEGF signaling pathway	4/59	0,362926182
Sulfur metabolism	1/9	0,379299997
mTOR signaling pathway	9/152	0,384840943
Glycerophospholipid metabolism	6/97	0,384913372
Fatty acid degradation	3/44	0,398037404
Phosphatidylinositol signaling system	6/99	0,403546277
Basal cell carcinoma	4/63	0,410285043
ABC transporters	3/45	0,412098092
AMPK signaling pathway	7/120	0,425362518
Phototransduction	2/28	0,42762859
Cortisol synthesis and secretion	4/65	0,433710529
Cysteine and methionine metabolism	3/47	0,439891843
Ubiquinone and other terpenoid-quinone biosynthesis	1/11	0,441732347
MAPK signaling pathway	16/295	0,455354527
JAK-STAT signaling pathway	9/162	0,458940654
Cell cycle	7/124	0,459251941
Citrate cycle (TCA cycle)	2/30	0,463008482
PI3K-Akt signaling pathway	19/354	0,463500067
Cocaine addiction	3/49	0,467165176
Epithelial cell signaling in Helicobacter pylori infection	4/68	0,468332155
Glycolysis / Gluconeogenesis	4/68	0,468332155
Neuroactive ligand-receptor interaction	18/338	0,479773933

Galactose metabolism	2/31	0,480209569
RNA transport	9/165	0,48095215
Fatty acid biosynthesis	1/13	0,497890471
Non-homologous end-joining	1/13	0,497890471
Retrograde endocannabinoid signaling	8/148	0,498068602
Glycosaminoglycan biosynthesis	3/53	0,5198147
Cholinergic synapse	6/112	0,522016281
Influenza A	9/171	0,524277119
SNARE interactions in vesicular transport	2/34	0,529712675
Thermogenesis	12/231	0,532584935
Glutamatergic synapse	6/114	0,539482998
Prion diseases	2/35	0,545485494
Gastric acid secretion	4/75	0,545698286
Pertussis	4/76	0,556273637
Starch and sucrose metabolism	2/36	0,560884561
Tyrosine metabolism	2/36	0,560884561
RNA degradation	4/79	0,587183851
Epstein-Barr virus infection	10/201	0,592808028
AGE-RAGE signaling pathway in diabetic complications	5/100	0,592827344
Selenocompound metabolism	1/17	0,593840785
T cell receptor signaling pathway	5/101	0,601717403
Pyruvate metabolism	2/39	0,604811304
Apoptosis	7/143	0,610890194
Lysosome	6/123	0,614298825
ECM-receptor interaction	4/82	0,616800048
Glycine, serine and threonine metabolism	2/40	0,618693656
Taste transduction	4/83	0,626371905
Fat digestion and absorption	2/41	0,632197223
Glycosaminoglycan degradation	1/19	0,63470968
Porphyrin and chlorophyll metabolism	2/42	0,645324095
Tryptophan metabolism	2/42	0,645324095
Non-alcoholic fatty liver disease (NAFLD)	7/149	0,653826066
Insulin secretion	4/86	0,654159206
Salmonella infection	4/86	0,654159206
Alzheimer disease	8/171	0,661883598
Carbohydrate digestion and absorption	2/44	0,670459987
Basal transcription factors	2/45	0,682476808
Other types of O-glycan biosynthesis	1/22	0,688439703
Mismatch repair	1/23	0,704533848
Protein export	1/23	0,704533848
Ether lipid metabolism	2/47	0,705431946
IL-17 signaling pathway	4/93	0,713443398
Small cell lung cancer	4/93	0,713443398
Valine, leucine and isoleucine degradation	2/48	0,716381176
Arrhythmogenic right ventricular cardiomyopathy (ARVC)	3/72	0,725260757
Bile secretion	3/72	0,725260757
Arginine and proline metabolism	2/49	0,7269861
alpha-Linolenic acid metabolism	1/25	0,734273137
Inositol phosphate metabolism	3/74	0,742282769
Thyroid hormone synthesis	3/74	0,742282769
Folate biosynthesis	1/26	0,74800173
Ascorbate and aldarate metabolism	1/27	0,761021692
Inflammatory mediator regulation of TRP channels	4/100	0,764920995

Ribosome biogenesis in eukaryotes	4/101	0,771651031
Cardiac muscle contraction	3/78	0,77379609
Synaptic vesicle cycle	3/78	0,77379609
Fanconi anemia pathway	2/54	0,775076347
Linoleic acid metabolism	1/29	0,785080013
Osteoclast differentiation	5/127	0,790475465
Glutathione metabolism	2/56	0,792140489
Mucin type O-glycan biosynthesis	1/31	0,806718435
Drug metabolism	4/108	0,814586956
Viral myocarditis	2/59	0,815592036
Propanoate metabolism	1/32	0,816707165
Hypertrophic cardiomyopathy (HCM)	3/85	0,821291395
Tuberculosis	7/179	0,822283088
Base excision repair	1/33	0,826180149
Glycerolipid metabolism	2/61	0,829882961
Pentose and glucuronate interconversions	1/34	0,835163993
Serotonergic synapse	4/113	0,841018605
Necroptosis	6/162	0,847599405
DNA replication	1/36	0,851763893
Dilated cardiomyopathy (DCM)	3/91	0,854971748
African trypanosomiasis	1/37	0,859426581
Aldosterone-regulated sodium reabsorption	1/37	0,859426581
Primary immunodeficiency	1/37	0,859426581
Parkinson disease	5/142	0,862623354
Retinol metabolism	2/67	0,8669476
NF-kappa B signaling pathway	3/95	0,874219853
Ferroptosis	1/40	0,880120803
Hematopoietic cell lineage	3/97	0,882968025
Homologous recombination	1/41	0,886318873
PPAR signaling pathway	2/74	0,900743148
NOD-like receptor signaling pathway	6/178	0,902193246
Chagas disease (American trypanosomiasis)	3/103	0,906043438
Glycosphingolipid biosynthesis	1/45	0,908070493
Proteasome	1/45	0,908070493
Toll-like receptor signaling pathway	3/104	0,909462536
Antigen processing and presentation	2/77	0,912619361
Malaria	1/49	0,925663348
Ovarian steroidogenesis	1/49	0,925663348
Vibrio cholerae infection	1/50	0,929508438
Peroxisome	2/83	0,932480377
Amyotrophic lateral sclerosis (ALS)	1/51	0,933154815
Mineral absorption	1/51	0,933154815
Toxoplasmosis	3/113	0,935503929
Autoimmune thyroid disease	1/53	0,939891944
Legionellosis	1/55	0,945950624
Protein digestion and absorption	2/90	0,950247508
Steroid hormone biosynthesis	1/60	0,958560365
Arachidonic acid metabolism	1/63	0,964667232
Inflammatory bowel disease (IBD)	1/65	0,968230286
Aminoacyl-tRNA biosynthesis	1/66	0,969874831
Systemic lupus erythematosus	3/133	0,970576475
RIG-I-like receptor signaling pathway	1/70	0,975645263
Measles	3/138	0,975957476

Leishmaniasis	1/74	0,980311144
Metabolism of xenobiotics by cytochrome P450	1/74	0,980311144
Cytokine-cytokine receptor interaction	8/294	0,98615442
Chemical carcinogenesis	1/82	0,987133925
Oxidative phosphorylation	2/133	0,992952764
Amoebiasis	1/96	0,993891226
Phagosome	1/152	0,999688632
Ribosome	1/153	0,999704658
Herpes simplex virus 1 infection	10/492	0,999884602
Olfactory transduction	4/444	0,999995499

Adjusted.P.value	Old.P.value	Old.Adjusted	Odds.Ratio
0,004052187	0	0	3,445305771
0,004159278	0	0	3,007217322
0,037524086	0	0	2,33492108
0,031331647	0	0	2,60569344
0,031988253	0	0	2,330487685
0,030300288	0	0	3,181765591
0,033558075	0	0	3,089540501
0,031791566	0	0	2,370196866
0,046333407	0	0	2,735978112
0,047493057	0	0	2,24120656
0,048319343	0	0	2,80497756
0,048468866	0	0	2,263339558
0,069739637	0	0	2,500625156
0,110856218	0	0	2,583979328
0,118927215	0	0	2,302555837
0,114408914	0	0	2,683363148
0,124740349	0	0	1,82828726
0,164424529	0	0	1,964173476
0,165216333	0	0	1,950991103
0,163013768	0	0	1,462629808
0,160807773	0	0	3,142677561
0,158200412	0	0	1,980422113
0,168567812	0	0	1,820206433
0,166614418	0	0	2,253470344
0,197873623	0	0	2,08998328
0,194475416	0	0	1,735508504
0,208011296	0	0	2,385211688
0,204654493	0	0	1,816860465
0,21382707	0	0	3,125781445
0,214064166	0	0	2,011115987
0,210552632	0	0	1,880134213
0,230663699	0	0	7,751937984
0,232578338	0	0	1,83896339
0,225737799	0	0	1,83896339
0,240132514	0	0	2,214839424
0,238873273	0	0	1,997922161
0,237163788	0	0	3,370407819
0,234406305	0	0	1,655564645
0,235000004	0	0	1,750437609
0,230186097	0	0	1,90337763
0,226182548	0	0	1,70998632
0,231378375	0	0	1,667083438
0,232891221	0	0	2,76854928
0,234770365	0	0	1,937984496
0,233785758	0	0	2,260981912
0,232335932	0	0	1,802776275
0,235810082	0	0	2,42248062
0,234046243	0	0	2,095118374
0,235722037	0	0	1,695736434
0,231091971	0	0	1,982029598
0,243569631	0	0	1,761804087
0,259069482	0	0	1,550387597

0,306437097	0	0	1,61498708
0,305145792	0	0	2,42248062
0,327215356	0	0	1,705426357
0,321498187	0	0	2,363395727
0,338843153	0	0	1,546264226
0,333445856	0	0	1,798129945
0,336796069	0	0	1,966071228
0,357239095	0	0	1,761804087
0,363909748	0	0	1,603849238
0,371744521	0	0	2,202255109
0,3790797	0	0	2,004811548
0,406754527	0	0	1,693384511
0,432447164	0	0	2,76854928
0,432811782	0	0	1,420074846
0,426922262	0	0	1,722652885
0,423207043	0	0	2,061685634
0,422942949	0	0	1,361126235
0,428928725	0	0	1,703722634
0,42485362	0	0	1,51998784
0,428272761	0	0	2,01873385
0,42700889	0	0	1,458698008
0,437763284	0	0	1,500375094
0,465709563	0	0	1,411153759
0,46268023	0	0	1,937984496
0,456671395	0	0	1,937984496
0,465604223	0	0	1,788908766
0,485004883	0	0	1,761804087
0,478942322	0	0	1,761804087
0,477751617	0	0	1,405791862
0,48537372	0	0	1,3502351
0,494620324	0	0	1,58202816
0,488731987	0	0	1,58202816
0,488426415	0	0	1,490757305
0,493816688	0	0	1,70998632
0,488140634	0	0	1,70998632
0,486116444	0	0	1,566048078
0,482478589	0	0	1,399655469
0,486158106	0	0	1,358400348
0,489297437	0	0	1,468170073
0,485003874	0	0	1,68520391
0,50690352	0	0	1,761804087
0,522013467	0	0	1,637733377
0,523877943	0	0	1,46570256
0,535032966	0	0	1,61498708
0,576272492	0	0	1,39423345
0,577969356	0	0	1,325989392
0,589820487	0	0	1,490757305
0,585040944	0	0	1,550387597
0,592334036	0	0	1,435544071
0,626985787	0	0	1,40944327
0,634131684	0	0	1,68520391
0,632562754	0	0	1,875468867
0,633240468	0	0	1,362645349

0,628235957	0	0	1,336541032
0,627102988	0	0	2,27998176
0,63624444	0	0	1,649348507
0,657632697	0	0	3,875968992
0,652497368	0	0	1,291989664
0,650071383	0	0	1,61498708
0,647362627	0	0	1,33143973
0,641633754	0	0	1,33143973
0,651818928	0	0	1,761804087
0,696945659	0	0	1,174536058
0,723094887	0	0	3,22997416
0,763822816	0	0	1,304412642
0,785355249	0	0	1,188386719
0,801747987	0	0	1,250312578
0,797764383	0	0	1,306506402
0,799448385	0	0	1,345822567
0,79289553	0	0	1,345822567
0,788790995	0	0	1,267840325
0,782671102	0	0	1,761804087
0,777229605	0	0	1,40944327
0,80468011	0	0	1,277791975
0,807713959	0	0	1,188947544
0,804573299	0	0	1,68520391
0,798336296	0	0	1,68520391
0,792195248	0	0	1,68520391
0,79568643	0	0	1,263902932
0,791356448	0	0	1,35998912
0,791935014	0	0	1,150401756
0,790123441	0	0	1,157005669
0,788286344	0	0	2,42248062
0,82192106	0	0	1,313887794
0,815921636	0	0	1,313887794
0,846553617	0	0	2,153316107
0,852741082	0	0	1,14749082
0,846809419	0	0	1,198753297
0,869471777	0	0	1,321353066
0,87529756	0	0	1,174536058
0,883690861	0	0	1,230466347
0,88143203	0	0	1,291989664
0,90352866	0	0	1,130490956
0,902120587	0	0	1,38427464
0,908726823	0	0	1,192605844
0,915450593	0	0	1,237011381
0,913111161	0	0	1,761804087
0,934994628	0	0	1,051110235
0,936117361	0	0	1,076658053
0,93058946	0	0	1,094023506
0,932069363	0	0	1,291989664
0,927000135	0	0	1,04016117
0,928302414	0	0	1,18652112
0,924655793	0	0	1,13999088
0,918766266	0	0	1,13999088
0,935255516	0	0	1,032062749

0,930217278	0	0	1,250312578
0,925832888	0	0	1,057082452
0,952486118	0	0	1,490757305
0,946606574	0	0	1,490757305
0,941135763	0	0	1,047559187
0,976237363	0	0	1,096972356
0,974430391	0	0	1,03820598
0,972755136	0	0	1,01999184
0,976955113	0	0	1,13999088
0,976405714	0	0	1,006745193
0,983199784	0	0	1,01999184
0,988291365	0	0	1,107419712
0,982895158	0	0	1,033591731
0,996117907	0	0	1,01999184
0,998569045	0	0	1,076658053
0,992830142	0	0	1,076658053
1	0	0	0,981257973
1	0	0	0,964171391
1	0	0	0,968992248
1	0	0	1,13999088
1	0	0	0,959398265
1	0	0	0,993838203
1	0	0	0,948663739
1	0	0	0,945358291
1	0	0	0,945358291
1	0	0	0,968992248
1	0	0	0,933968432
1	0	0	0,945358291
1	0	0	1,01999184
1	0	0	0,92284976
1	0	0	0,92284976
1	0	0	0,910462515
1	0	0	0,901388138
1	0	0	0,901388138
1	0	0	0,906659413
1	0	0	0,880902044
1	0	0	0,861326443
1	0	0	0,880902044
1	0	0	0,842601955
1	0	0	0,842601955
1	0	0	0,824674254
1	0	0	0,833541719
1	0	0	0,833541719
1	0	0	0,80749354
1	0	0	0,80749354
1	0	0	0,80749354
1	0	0	0,79101408
1	0	0	0,775193798
1	0	0	0,78566939
1	0	0	0,78566939
1	0	0	0,745378652
1	0	0	0,717772036
1	0	0	0,775193798

1	0	0	0,767518612
1	0	0	0,745378652
1	0	0	0,745378652
1	0	0	0,717772036
1	0	0	0,668270516
1	0	0	0,762986022
1	0	0	0,69213732
1	0	0	0,625156289
1	0	0	0,717772036
1	0	0	0,656943897
1	0	0	0,605620155
1	0	0	0,683994528
1	0	0	0,757871032
1	0	0	0,587268029
1	0	0	0,635404753
1	0	0	0,56999544
1	0	0	0,686012211
1	0	0	0,717772036
1	0	0	0,538329027
1	0	0	0,638895988
1	0	0	0,523779594
1	0	0	0,523779594
1	0	0	0,523779594
1	0	0	0,682388907
1	0	0	0,578502835
1	0	0	0,611995104
1	0	0	0,484496124
1	0	0	0,599376648
1	0	0	0,472679145
1	0	0	0,523779594
1	0	0	0,653253201
1	0	0	0,564461504
1	0	0	0,430663221
1	0	0	0,430663221
1	0	0	0,559033989
1	0	0	0,503372596
1	0	0	0,39550704
1	0	0	0,39550704
1	0	0	0,387596899
1	0	0	0,466984216
1	0	0	0,37999696
1	0	0	0,37999696
1	0	0	0,514509158
1	0	0	0,365657452
1	0	0	0,352360817
1	0	0	0,430663221
1	0	0	0,322997416
1	0	0	0,307616587
1	0	0	0,298151461
1	0	0	0,293634015
1	0	0	0,43713936
1	0	0	0,276854928
1	0	0	0,421300977

1	0	0	0,261889797
1	0	0	0,261889797
1	0	0	0,52734272
1	0	0	0,236339573
1	0	0	0,29142624
1	0	0	0,201873385
1	0	0	0,12749898
1	0	0	0,126665653
1	0	0	0,393899288
1	0	0	0,174593198

Combined.Score
38,7204073
31,63402076
18,47917191
20,3424716
17,62559117
23,65623106
22,17878363
16,82653117
18,07049865
14,51110974
17,84562579
14,19572628
14,57398567
13,67070724
11,86114021
13,75354385
9,101958734
9,12364792
8,947558062
6,652461791
14,18329016
8,87813321
7,963444521
9,789343256
8,634453839
7,131990219
9,55140324
7,238966923
12,20740068
7,783786852
7,246285813
28,92370822
6,789674235
6,789674235
7,976336846
7,149370558
11,99253845
5,866018087
6,152276868
6,681022044
5,989980486
5,76165889
9,485280904
6,579568918
7,634855326
6,059183986
8,053974604
6,937220484
5,567749576
6,507035447
5,656495011
4,851961316

4,752181273
7,093220319
4,843246011
6,710889265
4,282017671
4,977100603
5,388686681
4,695393248
4,218244278
5,709376829
5,126250728
4,183950275
6,627928123
3,376792242
4,093988808
4,887196592
3,207509299
3,966383947
3,531586106
4,645971938
3,341280218
3,37901223
3,071806226
4,205592161
4,205592161
3,824347528
3,672037086
3,672037086
2,916055061
2,762873562
3,188141129
3,188141129
2,987499969
3,3880701
3,3880701
3,091488617
2,757716183
2,650933901
2,839477618
3,25566162
3,306791541
3,008298221
2,671564559
2,892730146
2,379347858
2,245386325
2,479005637
2,575198513
2,352374132
2,215586155
2,613531239
2,895132655
2,088995923

2,046912783
3,474499418
2,474255328
5,650622543
1,881870271
2,343738185
1,925860438
1,925860438
2,505091275
1,581178635
4,201306255
1,614008827
1,427293183
1,465287873
1,526718088
1,558654372
1,558654372
1,464570245
2,034638719
1,62622342
1,419790872
1,307200255
1,846162327
1,846162327
1,846162327
1,369378871
1,470562551
1,234411683
1,235480821
2,574415889
1,33169862
1,33169862
2,087484578
1,095767861
1,144494099
1,217242729
1,065849316
1,096226325
1,145340919
0,966358918
1,17594164
0,996276626
1,015866394
1,43948402
0,826886332
0,838538298
0,851321313
0,994844838
0,79983063
0,903028463
0,864771434
0,864771434
0,757988432

0,917145122
0,773771136
1,03961712
1,03961712
0,730167041
0,71773023
0,674892548
0,65864431
0,724373623
0,63426245
0,629481853
0,671183906
0,626035189
0,598220067
0,622566934
0,622566934
0,522438724
0,504150432
0,506639611
0,594099447
0,487343017
0,499740374
0,467537589
0,460648311
0,456806939
0,465256812
0,436920693
0,433497705
0,463675624
0,404210609
0,404210609
0,386868193
0,382553202
0,382553202
0,374147126
0,352176933
0,329049728
0,328864994
0,295095132
0,295095132
0,287765937
0,281447174
0,281447174
0,269333725
0,259386324
0,259386324
0,252213195
0,239437363
0,234149134
0,234149134
0,216420683
0,196018818
0,207738547

0,198958372
0,191150037
0,191150037
0,182883823
0,161701175
0,179393778
0,161279428
0,134271427
0,147196452
0,133912104
0,122622744
0,134663
0,148293045
0,112134385
0,118484305
0,102671668
0,118777181
0,118681564
0,086372691
0,100106602
0,079347307
0,079347307
0,079347307
0,100841468
0,08259675
0,082266455
0,061868268
0,074602188
0,057042206
0,054753371
0,067237092
0,055694304
0,041530262
0,041530262
0,053053149
0,046026576
0,030550809
0,030550809
0,028333098
0,032645545
0,02628977
0,02628977
0,034302291
0,022667239
0,019578896
0,021977947
0,013670136
0,011065607
0,009625916
0,008981752
0,013055202
0,006826196
0,010252892

0,005207748
0,005207748
0,007352383
0,003060493
0,00206102
0,001236981
3,97053E-05
3,74153E-05
4,54576E-05
7,85785E-07

Genes

CREBBP;BMPR2;ROCK1;SMURF2;SMAD9;INHBA;GDF6;RBX1;SMAD7;PPP2CA;RBL1;SP1;ID2;ID4;MAPK1;
NCOA2;MAP2K1;CREBBP;DIO2;HIF1A;ESR1;FOXO1;MED12L;MED17;RCAN1;KAT2B;MED14;RXRA;SIN3A;
MEF2A;MAP2K1;ROCK1;PDE3B;IRS4;NFATC3;ATP2B4;ATP2B2;CALML4;ATP2B1;ADRA2B;ADRA2A;GNA13
SHC4;MAP2K1;SHC2;SORT1;GAB1;PTPN11;CALML4;MAPK10;RPS6KA6;RAP1A;CAMK4;MAPK1;RAF1;CAL
CREBBP;FZD3;FZD5;CSNK1A1;NFATC3;PRICKLE1;SENP2;RBX1;LRP6;MAPK10;CCND3;SFRP1;ZNRF3;AP
GRM5;RPS6KA6;MAP2K1;CREBBP;RAP1A;CAMK4;MAPK1;CALML4;RAF1;CALM1;GRIN2B
MAP2K1;CREBBP;RAP1A;GAB1;MAPK1;PTPN11;RAF1;SOS2;CRK;HIF1A;RBX1
MAP2K1;FZD3;BMPR2;FZD5;PCGF3;SMAD9;INHBA;BMI1;APC;ID2;ID4;COMMD3-BMI1;MAPK1;CTNNB1;RA
SHC4;MAPK10;MAP2K1;SHC2;NRG3;ERBB4;GAB1;MAPK1;RAF1;SOS2;CRK;EREG
SHC4;MAP2K1;SHC2;FZD3;FZD5;CSNK1A1;ESR1;LRP6;HEYL;APC;SP1;E2F1;MAPK1;CTNNB1;RAF1;SOS2
SHC4;MAP2K1;SHC2;MECOM;E2F1;MAPK1;PTPN11;RAF1;SOS2;CRK;RUNX1
SHC4;PRKAB2;MAP2K1;SHC2;PDE3B;IRS4;CALML4;FOXO1;HK2;MAPK10;MAPK1;RAF1;CALM1;SOS2;CRI
MAP3K2;MAPK10;MAP2K1;ADCY9;MMP2;GNRHR;PTK2B;MAPK1;CALML4;RAF1;CALM1;SOS2
SHC4;MAP2K1;SHC2;CAMK4;E2F1;MAPK1;CALML4;RAF1;CALM1;SOS2
MAP2K1;CREBBP;FZD3;ADCY9;CREB1;FZD5;TYRP1;MAPK1;CTNNB1;CALML4;RAF1;CALM1
MAPK10;BCL2L13;USP15;CALCOCO2;SP1;TFEB;E2F1;PGAM5;HIF1A
ADCYAP1R1;CREBBP;MAP2K1;ROCK1;PDE3B;ATP2B4;ATP2B2;CALML4;ATP2B1;GRIN2B;TSHR;MAPK10;
SHC4;PTGFR;MAP2K1;SHC2;RALA;GAB1;PTPN11;CYTH3;GNA13;GRM5;ADCY9;PTK2B;MAPK1;RAF1;SOS
SHC4;MAP2K1;SHC2;FZD3;FZD5;CSNK1A1;GAB1;LRP6;RXRA;APC;E2F1;MAPK1;CTNNB1;RAF1;SOS2
RALA;ROCK1;XIAP;CALML4;HIF1A;FOXO1;LRP6;CKS1B;GNA13;CCND3;RXRA;MECOM;E2F1;MAPK1;IL6R
MAP2K1;RXRA;TPM3;CCDC6;MAPK1;CTNNB1
SHC4;NCOA2;MAP2K1;SHC2;MMP2;CALML4;ESR1;ADCY9;CREB1;SP1;MAPK1;RAF1;CALM1;SOS2
FZD3;BMPR2;ROCK1;SEMA3A;NFATC3;PTPN11;L1CAM;ENAH;EFNA3;EFNB3;DPYSL5;PLXNA2;MAPK1;SL
MAPK10;MAP2K1;RALA;APC;MAPK1;CTNNB1;RAF1;SOS2;APPL1;EREG
PRKAB2;ADCY9;CREB1;SESN3;CAMK4;IRS4;EIF4EBP2;SOD2;EIF4E;FOXO1;APPL1
FZD3;MAP2K1;HPSE2;FZD5;ROCK1;MMP2;GAB1;PTPN11;HIF1A;ESR1;ERBB4;GPC1;PDCD4;CTNNB1;MAI
MAP2K1;MAPK1;PDHB;RAF1;HIF1A;HK2;GLS;FGFR1
MAP2K1;NFATC3;CALML4;HIPK1;FOXO1;HIPK2;CCND3;RBL1;CDK1;E2F1;MAPK1;BTRC;RAF1;CALM1;RAD
PRKAB2;CREB1;RORB;BTRC;RBX1
MEF2A;GNA13;MAP2K1;ADCY9;CREB1;RXRA;SP1;MAPK1;RAF1;FGFR1;LRP6
TCERG1;SF3B3;SRSF1;HNRNPU;LSM5;U2SURP;HNRNPK;TRA2B;SNRPD3;HNRNPC;SRSF10;SMNDC1;SI
GLUD2;GLS
MEF2A;HDAC5;PRKAB2;MAP2K1;PDE3B;CALML4;GNA13;ADCY9;CAMK4;TFAM;MAPK1;RAF1;CALM1
SMURF2;UBA6;CUL3;XIAP;UBE2G1;RBX1;PIAS1;UBE2J1;ITCH;CDC34;TRIP12;BTRC;UBE2K
SHC4;MAPK10;MAP2K1;SHC2;MAPK1;RAF1;SOS2;ESR1
MAP2K1;CREBBP;CREB1;E2F1;MAPK1;CTNNB1;RAF1;SOS2;FOXO1;FGFR1
MME;LNPEP;REN;AGTR2
SHC4;MAP2K1;SHC2;ROCK1;XIAP;PARVA;ACTN4;MAPK10;CCND3;RAP1A;CHAD;CTNNB1;MAPK1;COL6A
MAP2K1;FZD3;FZD5;ADCY9;RAP1A;NCEH1;CREB1;APC;SP1;RBBP5;E2F1;MAPK1;CTNNB1;AIPL1
OCLN;RAP1A;ROCK1;MMP2;CLDN18;PTK2B;CTNNB1;PTPN11;ACTN4;CLDN1;JAM2
PRKAB2;ROCK1;ACTN4;CLDN1;AMOT;RUNX1;RAP2C;PPP2CA;MAPK10;OCLN;RAP1A;CLDN18;MYH10;RA
BCL11B;HPGD;ZBTB16;SIX1;PAX5;BMI1;FOXO1;AFF1;RUNX1;ELK4;MAF;RXRA;SP1;SIN3A;ID2;COMMD3-E
GLUD2;GFPT1;RIMKLA;NAT8L;GLS
MAP2K1;CREBBP;PFKFB3;MAPK1;PDHB;IL6R;HIF1A;EIF4E;HK2;RBX1
PPP2CA;GNA13;LYN;MAP2K1;MAPK1;RAF1;PRKG1
ENTPD1;RRM1;ADCY9;RRM2B;PDE1B;PDE3B;ADK;PDE5A;PDE7B;NUDT5;PFAS;PAPSS2
KAT2B;CREBBP;APH1B;MAML1;DTX1;DTX4
SHC4;SHC2;SEPT11;SEPT12;GAB1;ELMO1;CTNNB1;CRK
FZD3;BMPR2;FZD5;GDF6;AMOT;SMAD7;PPP2CA;CCND3;APC;ID2;CTNNB1;BTRC;TEAD1;BMPR1A
MAP3K2;GRM5;MAP2K1;ADCY9;CDK1;MAPK1;RAF1;SOS2;PRKG1
MAPK10;PRKAB2;MAP2K1;CREBBP;IRS4;AGAP2;S1PR1;MAPK1;RAF1;SOD2;SOS2;FOXO1
MAP2K1;ROCK1;NFATC3;TAP2;CALML4;GNA13;ADCY9;CREB1;SP1;E2F1;CTNNB1;PTK2B;MAPK1;CALM1;

SHC4;MAP2K1;SHC2;FZD3;FZD5;CSNK1A1;GAB1;LRP6;APC;E2F1;MAPK1;CTNNB1;RAF1;SOS2
GABRA1;GABRB1;GABRA3;SLC17A6;GRIN2B
PPP2CA;RPS6KA6;MAP2K1;ADCY9;CDK1;MAPK1;CALML4;BTRC;CALM1;SMC1A;RBX1
MAP2K1;MMP2;E2F1;MAPK1;RAF1
CHRM3;PTGFR;PDE1B;ATP2B4;ATP2B2;CALML4;ATP2B1;TPCN1;GRM5;ADCY9;STIM2;ERBB4;CAMK4;PTI
ADCYAP1R1;ADCY9;CREB1;NOS1AP;MAPK1;CALML4;CALM1;GRIN2B;PRKG1
ADCYAP1R1;CREB1;PDE1B;PDE3B;REN;CALML4;CALM1
MAPK10;MAP2K1;PCYT1B;SP1;MAPK1;RAF1;GPCPD1;HIF1A;SOS2
PPP2CA;ADCY9;CREB1;TPM3;ATP2B4;MAPK1;ATP2B2;CALML4;AGTR2;SCN5A;ATP2B1;CALM1
CREB1;ADCY9;DCTN2;DYNLL2;RAB11A
MAP2K1;APC;MAPK1;CTNNB1;RAF1;SOS2
PRKAB2;CREBBP;CREB1;PDE3B;CALML4;SIK2;PDHB;CALM1;FOXO1
GLUD2;GLS;OTC
SHC4;MAP2K1;SHC2;PLA2G12A;RALA;GAB1;PTPN11;CALML4;GRIN2B;MAPK10;EFNA3;RAP1A;MAPK1;CA
CHRM3;ADCY9;ATP2B4;ATP2B2;CALML4;ATP2B1;CALM1;PRKG1
CSNK1G3;SMURF2;CSNK1A1;CUL3;BTRC
SHC4;CREBBP;FZD3;MAP2K1;BMPR2;ROCK1;DNMT3A;BMI1;GLS;EFNA3;MARCKS;HNRNPK;APC;E2F1;IG
PPP2CA;NXF1;CPSF7;PABPN1;PAPOLG;SMG7;BCL2L2-PABPN1;PELO
RCAN1;PRKAB2;MAP2K1;ADCY9;EEF2K;ROCK1;CAMK4;NFATC3;MAPK1;CALML4;RAF1;CALM1
ADCY9;ATP2B1;ESR1;AP2M1;RAB11A
LYN;MAP2K1;CREBBP;NFATC3;CALML4;HIF1A;MAPK10;RCAN1;CREB1;E2F1;MAPK1;CTNNB1;RAF1;CALN
PPP2CA;MAP2K1;OCLN;RXRA;E2F1;CLDN18;MAPK1;CTNNB1;RAF1;CLDN1;SOS2;PIAS1
MAP2K1;RALA;CALML4;GRIN2B;EFNA3;ADCY9;SIPA1L1;RAP1A;CTNNB1;MAPK1;CALM1;RAF1;RAPGEF6;
ABCA1;NCEH1;VAPA;SORT1;VAPB
ST6GAL2;ALG6;MAN1A2;MGAT5;ALG14
MAPK10;ROCK1;ELMO1;MAPK1;BTRC;CRK
MAP2K1;ZBTB16;MAPK1;RAF1;SOS2;RUNX1
MAP2K1;RXRA;E2F1;MAPK1;RAF1;SOS2
CREBBP;COX7B;DCTN2;UQCR10;SOD2;GRIN2B;GRM5;CREB1;SP1;SIN3A;TFAM;AP2M1;DNAL1;TGM2
SMURF2;SH3KBP1;AGAP2;RAB11A;CYTH3;SNX1;ITCH;ACAP2;CAPZB;KIF5C;CAPZA1;PSD4;PSD3;SH3GL
ADCY9;CREB1;CAMK4;ATP2B4;ATP2B2;CALML4;ATP2B1;CALM1
CHRM3;PLA2G12A;ADCY9;RAP1A;ATP2B4;ATP2B2;ATP2B1;RAB11A
SHC4;MAPK10;MAP2K1;SHC2;ADCY9;CREB1;MMP2;MAPK1;RAF1;SOS2
CREB1;CAMK4;PPP1R1B;CALML4;CALM1;GRIN2B
LYN;MAPK10;MAP2K1;MAPK1;RAF1;SOS2
MAPK10;RPS6KA6;MAP2K1;ADCY9;PDE3B;CDK1;MAPK1;RAF1
SHC4;HDAC5;MAP2K1;SHC2;CALML4;GRIN2B;CREB1;CAMK4;PPP1R1B;MAPK1;RAF1;CALM1;SOS2
NCKAP1;CHRM3;MAP2K1;ROCK1;ACTN4;ENAH;GNA13;DIAPH2;APC;MAPK1;RAF1;MYH10;SOS2;CRK;FG
GNA13;MAP2K1;PLA2G12A;ADCY9;ROCK1;MAPK1;CALML4;RAF1;CALM1;PRKG1
MAPK10;PRKAB2;RXRA;IRS4;ACSL6;PTPN11
ADCY9;PDE3B;IRS4;PRKG1;TSHR
LYN;MAP2K1;NFATC3;MAPK1;RAF1;SOS2
PPP2CA;GNA13;MAPK10;MAP2K1;ROCK1;S1PR1;MAPK1;S1PR3;RAF1
CREBBP;PTPRM;MAPK1;CTNNB1;ACTN4;FGFR1
MEF2A;MAPK10;BMPR2;MMP2;GPC1;CTNNB1;CALML4;CALM1;SELE;BMPR1A
SHC4;LYN;MAP2K1;SHC2;ROCK1;ADCY9;RAP1A;ELMO1;PTK2B;MAPK1;RAF1;SOS2;CRK
GABRA1;GABRB1;ADCY9;PDE1B;PDE3B;GABRA3;PDE7B
MAPK10;MAP2K1;RALA;E2F1;MAPK1;RAF1
MAPK10;RPS6KA6;PRKAB2;CREB1;GFPT1;PTPN11;FOXO1;PPARGC1B
MAPK10;MAP2K1;ITCH;CREB1;TNFAIP3;MAPK1;PGAM5;SELE
MAPK10;IRS4;MAPK1;HK2
ALDH6A1;ALDH2;CARNS1
PPP2CA;IGBP1;MAPK10;MAP2K1;MTMR3;IRS4;MAPK1;RAF1;HIF1A

CADM3;OCLN;CDH2;NRXN3;PTPRM;CLDN18;L1CAM;CLDN1;SELE;JAM2
CA12;GLUD2
RPA4;GTF2H1;GTF2H5;RBX1
HK2
EDEM3;MAPK10;HSPH1;MAN1A2;EDEM1;HSPA4L;YOD1;UBE2G1;SEC62;RBX1;UBE2J1
CYB5R4;GFPT1;PGM3;HK2
PPP2CA;MAPK10;CREB1;KIF5C;PPP1R1B;CALML4;CALM1;GRIN2B;SCN1A
SHC4;MAP2K1;SHC2;PTK2B;MAPK1;PTPN11;RAF1;SOS2;CD244
PFKFB3;SORD;HK2
CREBBP;FZD3;MAP2K1;FZD5;MAML1;CSNK1A1;FOXO1;PPP2CA;HEYL;CCND3;CREB1;RBL1;APC;CHAD;E
PCYT1B
MAPK10;NFATC3;MAPK1;PTPN11;CALML4;RAF1;CALM1
MAP2K1;NFATC3;TAP2;CALML4;RBX1;MAPK10;CDK1;PTK2B;MAPK1;BTRC;CALM1;RAF1;CRK
LYN;GNA13;ADCY9;RAP1A;ROCK1;SNAP23;MAPK1;PRKG1
GABRA1;GABRB1;ADCY9;GABRA3;GLS;TRAK2
MAP2K1;E2F1;MAPK1;RAF1;FGFR1
CCND3;SESN3;RRM2B;ZMAT3;CDK1
MAPK10;RXRA;NFATC3;MAPK1;IL6R;HIF1A;RUNX1
FNTB;ZMPSTE24
OCLN;ROCK1;CTNNB1;CLDN1
LYN;MAP2K1;MARCKS;MAPK1;RAF1;CRK
MAPK10;MAP2K1;CREBBP;CREB1;NFATC3;E2F1;PTK2B;MAPK1;RAF1;SOS2
ALDH2;CARNS1
B3GALNT2;B3GAT2
GLUD2;GLS
MAPK10;MAF;MAML1;NFATC3;MAPK1;RUNX3
ENTPD1;RRM1;RRM2B;CMPK1
CREBBP;MAP2K1;RANBP3;NFATC3;XIAP;ELK4;MAPK10;KAT2B;CCND3;ADCY9;CREB1;E2F1;MAPK1
LYN;KAT2B;HDAC5;CREBBP;CCND3;HNRNPK;CREB1;RBL1;CDK1;MAPK1;GTF2H1;ACTN4
MOCS3
ALDH2;PRDM6;NSD1;DLST
MAP2K1;SHC2;MAPK1;RAF1
PAPSS2
RPS6KA6;MAP2K1;FZD3;FZD5;MAPK1;RAF1;SOS2;EIF4E;LRP6
PCYT1B;PLA2G12A;GPAM;ETNK1;GPCPD1;CDS2
ALDH2;ADH1B;ACSL6
MTMR3;ITPK1;CALML4;IP6K1;CALM1;CDS2
FZD3;FZD5;APC;CTNNB1
ABCA1;ABCA2;TAP2
PPP2CA;PRKAB2;PFKFB3;EEF2K;CREB1;IRS4;FOXO1
CALML4;CALM1
ADCY9;CREB1;NCEH1;SP1
MAT2A;DNMT3A;MAT2B
VKORC1
MAP3K2;MAP2K1;NFATC3;EREG;ELK4;MAPK10;RPS6KA6;EFNA3;RAP1A;MECOM;ERBB4;MAPK1;RAF1;S
CCND3;CREBBP;PTPN11;RAF1;IL6R;SOS2;EPOR;IL13RA1;PIAS1
CCND3;CREBBP;RBL1;CDK1;E2F1;SMC1A;RBX1
DLST;PDHB
PHLPP2;MAP2K1;EPOR;EREG;PPP2CA;CCND3;EFNA3;RXRA;CREB1;ERBB4;CDC37;CHAD;MAPK1;COL6
CREB1;PPP1R1B;GRIN2B
LYN;MAPK10;PTPN11;JAM2
ALDH2;ADH1B;PDHB;HK2
PTGFR;GABRA1;CHRM3;ADCYAP1R1;GABRB1;NPFFR1;GRID1;GABRA3;GRIN2B;ADRA2B;ADRA2A;TSHR

MGAM;HK2
NDC1;NXF1;EIF2B2;EIF3J;EIF4EBP2;PHAX;SENP2;RPP14;EIF4E
ACSL6
XRCC5
MAPK10;GABRA1;GABRB1;GRM5;ADCY9;GABRA3;SLC17A6;MAPK1
HS3ST3B1;CHST7;CHST3
CHRM3;MAP2K1;ADCY9;CREB1;CAMK4;MAPK1
MAPK10;NXF1;MAP2K1;CREBBP;PABPN1;MAPK1;RAF1;BCL2L2-PABPN1;KPNA1
SNAP23;VTI1A
RPS6KA6;PRKAB2;COX7B;ADCY9;COA1;CREB1;PRDM16;ACSL6;UQCR10;SOS2;PRKG1;FGFR1
GRM5;ADCY9;SLC17A6;MAPK1;GRIN2B;GLS
MAP2K1;MAPK1
CHRM3;ADCY9;CALML4;CALM1
MAPK10;MAPK1;CALML4;CALM1
MGAM;HK2
ADH1B;TYRP1
CNOT6;BTG1;LSM5;DCP2
LYN;MAPK10;ENTPD1;CCND3;PSMD11;SIN3A;TAP2;E2F1;TNFAIP3;RUNX3
MAPK10;MMP2;MAPK1;SELE;FOXO1
PAPSS2
MAP2K1;NFATC3;MAPK1;RAF1;SOS2
ALDH2;PDHB
HRK;MAPK10;MAP2K1;DFFA;XIAP;MAPK1;RAF1
AP3M2;ABCA2;SORT1;LAPTM5;LITAF;SUMF1
CHAD;DAG1;COL6A5;CD47
ALAS2;PSPH
CHRM3;GABRA1;PDE1B;GABRA3
ABCA1;PLA2G12A
HPSE2
ALAD;ALAS2
ALDH2;DLST
MAPK10;PRKAB2;COX7B;ITCH;RXRA;UQCR10;IL6R
CHRM3;ADCYAP1R1;ADCY9;CREB1
MAPK10;ROCK1;MAPK1;MYH10
COX7B;APH1B;MME;MAPK1;CALML4;UQCR10;CALM1;GRIN2B
MGAM;HK2
GTF2H1;GTF2H5
ST6GAL2
RPA4
SEC62
PLA2G12A;PAFAH1B2
MAPK10;SRSF1;TNFAIP3;MAPK1
RXRA;E2F1;XIAP;CKS1B
ALDH6A1;ALDH2
CDH2;DAG1;CTNNB1
ADCY9;RXRA;NCEH1
ALDH2;CARNS1
PLA2G12A
ALDH6A1;MTMR3;ITPK1
ADCY9;CREB1;TSHR
PCBD2
ALDH2
MAPK10;ADCY9;CALML4;CALM1

UTP15;RBM28;NXF1;GTPBP4
COX7B;TPM3;UQCR10
NAPA;SLC17A6;AP2M1
RPA4;USP1
PLA2G12A
MAPK10;MAP2K1;CREB1;CAMK4;MAPK1
RRM1;RRM2B
GALNT7
RRM1;RRM2B;ADH1B;CMPK1
CXADR;DAG1
ALDH6A1
PRKAB2;TPM3;DAG1
MAPK10;CREBBP;CREB1;MAPK1;CALML4;RAF1;CALM1
TDG
ALDH2;GPAM
SORD
MAP2K1;GABRB1;MAPK1;RAF1
MAPK10;GLUD2;SPATA2;TNFAIP3;XIAP;PGAM5
RPA4
ADCY9;TPM3;DAG1
SELE
MAPK1
TAP2
COX7B;GPR37;UBE2G1;UQCR10;UBE2J1
SDR16C5;ADH1B
LYN;TNFAIP3;XIAP
ACSL6
MME;IL6R;EPOR
RPA4
RXRA;ACSL6
MAPK10;NEK7;TNFAIP3;XIAP;MAPK1;ANTXR2
PPP2CA;MAPK10;MAPK1
ST6GALNAC3
PSMD11
MAPK10;MAP2K1;MAPK1
CREB1;TAP2
SELE
ADCY9
ADCY9
ACSL6;SOD2
GRIN2B
ATP2B1
MAPK10;XIAP;MAPK1
TSHR
BCL2L13
MME;COL6A5
SRD5A1
PLA2G12A
MAF
GATC
SNRPD3;ACTN4;GRIN2B
MAPK10
MAPK10;CCND3;TNFAIP3

MAPK1
ADH1B
BMPR2;INHBA;GDF6;RELT;IL6R;EPOR;IL13RA1;BMPR1A
ADH1B
COX7B;UQCR10
ACTN4
TAP2
MRPL27
ZNF275;NXF1;EIF2B2;SRSF1;TAP2;PTPN11;HCFC2;ZNF25;ZNF597;SRSF9
PDE1B;CALML4;CALM1;PRKG1

;BMPR1A
RCAN2;MAPK1;CTNNB1;RAF1
3;ADCY9;CREB1;MAPK1;PDE5A;RAF1;CALM1;PRKG1
LM1;SOS2;CRK
PC;TBL1XR1;RUVBL1;RSPO2;CTNNB1;BTRC

AF1;FGFR1;BMPR1A

2;FGFR1

K;EIF4E

ADCY9;RAP1A;CREB1;CAMK4;PPP1R1B;MAPK1;CALM1;RAF1
S2

2;APPL1;IL13RA1;CREBBP;FZD3;MAP2K1;FZD5;TPM3;ZBTB16;MMP2;ESR1;EPOR;RBX1;RUNX1;MAPK

LIT2;RAF1;SRGAP2;PLXNA3

PK1;RAF1;SOS2;FGFR1

D9A

RSF9

L5;RAF1;SOS2;CRK

APGEF6;JAM2
BMI1

;RAF1;IL6R;SOS2;CRK

K2B;CALM1

ALM1;RAF1;SOS2;FGFR1

GF2BP1;PDCD4;COMMD3-BMI1;MAPK1;RAF1;SOS2;CRK

M1

;CRK;FGFR1

.2;AP2M1;RAB11FIP5;SNX6

FR1

E2F1;CTNNB1;MAPK1;COL6A5;RAF1;SOS2

OS2;CRK;FGFR1

A5;RAF1;IL6R;SOS2;EIF4E;FGFR1

;GRM5;GNRHR;S1PR1;TAC3;S1PR3;AGTR2

K10;HEYL;ADCY9;APC;SP1;CCDC6;CTNNB1;CALM1;RAF1;SOS2;CRK;FGFR1
