# Supplementary material for: Circulating miR-330-3p in Late Pregnancy is Associated with Pregnancy Outcomes Among Lean Women with GDM
Source: Sci Rep. 2020 Jan 22;10:908. doi: 10.1038/s41598-020-57838-6 (PMC6976655; doi:10.1038/s41598-020-57838-6)
Supplement: Supplementary file 1 — Supplementary data. [file 41598_2020_57838_MOESM1_ESM.zip › Supplimentary File_EnrichR_Analysis NCI-Nature_2016.pdf]

Term
Regulation of nuclear SMAD2/3 signaling_Homo sapiens_246aac04-6195-11e5-8ac5-06603eb7f303
TGF-beta receptor signaling_Homo sapiens_1f188fcc-6196-11e5-8ac5-06603eb7f303
Canonical Wnt signaling pathway_Homo sapiens_9dbe253d-618f-11e5-8ac5-06603eb7f303
Retinoic acid receptors-mediated signaling_Homo sapiens_5797691b-6195-11e5-8ac5-06603eb7f303
Signaling events mediated by Hepatocyte Growth Factor Receptor (c-Met)_Homo sapiens_ac39d2b9-6195-11e
Regulation of Telomerase_Homo sapiens_4dfe97ca-6195-11e5-8ac5-06603eb7f303
FoxO family signaling_Homo sapiens_d06dbbda-6192-11e5-8ac5-06603eb7f303
Coregulation of Androgen receptor activity_Homo sapiens_27e0e369-6191-11e5-8ac5-06603eb7f303
IFN-gamma pathway_Homo sapiens_51b1ed75-6193-11e5-8ac5-06603eb7f303
FOXM1 transcription factor network_Homo sapiens_c51cda49-6192-11e5-8ac5-06603eb7f303
BMP receptor signaling_Homo sapiens_2a3c66e7-618e-11e5-8ac5-06603eb7f303
Degradation of beta catenin_Homo sapiens_61b6f2fc-6191-11e5-8ac5-06603eb7f303
VEGFR1 specific signals_Homo sapiens_8b13143b-6196-11e5-8ac5-06603eb7f303
Presenilin action in Notch and Wnt signaling_Homo sapiens_f51de83a-6194-11e5-8ac5-06603eb7f303
Class IB PI3K non-lipid kinase events_Homo sapiens_263703f8-6191-11e5-8ac5-06603eb7f303
Signaling events mediated by Stem cell factor receptor (c-Kit)_Homo sapiens_c6b6861c-6195-11e5-8ac5-0660
Role of Calcineurin-dependent NFAT signaling in lymphocytes_Homo sapiens_61bdd46d-6195-11e5-8ac5-0660
PDGFR-beta signaling pathway_Homo sapiens_c901a3e4-6194-11e5-8ac5-06603eb7f303
N-cadherin signaling events_Homo sapiens_5fc9c1e1-6194-11e5-8ac5-06603eb7f303
Trk receptor signaling mediated by the MAPK pathway_Homo sapiens_4980bb81-6196-11e5-8ac5-06603eb7f3
Signaling events mediated by VEGFR1 and VEGFR2_Homo sapiens_d6f6ae1f-6195-11e5-8ac5-06603eb7f303
ALK1 signaling events_Homo sapiens_fcc72679-6186-11e5-8ac5-06603eb7f303
Notch-mediated HES/HEY network_Homo sapiens_8ee56389-6194-11e5-8ac5-06603eb7f303
E2F transcription factor network_Homo sapiens_bb4d0fd3-6191-11e5-8ac5-06603eb7f303
ErbB4 signaling events_Homo sapiens_6104ebb2-6192-11e5-8ac5-06603eb7f303
Signaling events regulated by Ret tyrosine kinase_Homo sapiens_e4431190-6195-11e5-8ac5-06603eb7f303
Neurotrophic factor-mediated Trk receptor signaling_Homo sapiens_774988f5-6194-11e5-8ac5-06603eb7f303
EPHB forward signaling_Homo sapiens_01c81f4a-6192-11e5-8ac5-06603eb7f303
Cellular roles of Anthrax toxin_Homo sapiens_bb877621-6190-11e5-8ac5-06603eb7f303
Nectin adhesion pathway_Homo sapiens_685baa82-6194-11e5-8ac5-06603eb7f303
Calcium signaling in the CD4+ TCR pathway_Homo sapiens_5294f70b-618f-11e5-8ac5-06603eb7f303
amb2 Integrin signaling_Homo sapiens_5d4f90b6-6188-11e5-8ac5-06603eb7f303
Regulation of Androgen receptor activity_Homo sapiens_094a8cb0-6195-11e5-8ac5-06603eb7f303
ErbB1 downstream signaling_Homo sapiens_30d60550-6192-11e5-8ac5-06603eb7f303
HIF-1-alpha transcription factor network_Homo sapiens_20ef2b81-6193-11e5-8ac5-06603eb7f303
Regulation of nuclear beta catenin signaling and target gene transcription_Homo sapiens_1590a3b3-6195-11e
C-MYB transcription factor network_Homo sapiens_61020228-618e-11e5-8ac5-06603eb7f303
AP-1 transcription factor network_Homo sapiens_3ce2f9c5-6189-11e5-8ac5-06603eb7f303
EPO signaling pathway_Homo sapiens_20fe3c0e-6192-11e5-8ac5-06603eb7f303
Integrin-linked kinase signaling_Homo sapiens_21738158-6194-11e5-8ac5-06603eb7f303
ATF-2 transcription factor network_Homo sapiens_c76c900a-618a-11e5-8ac5-06603eb7f303
IL6-mediated signaling events_Homo sapiens_e684d5d2-6193-11e5-8ac5-06603eb7f303
Calcineurin-regulated NFAT-dependent transcription in lymphocytes_Homo sapiens_0439e9da-618f-11e5-8ac5-
FGF signaling pathway_Homo sapiens_98ed0df6-6192-11e5-8ac5-06603eb7f303
Ras signaling in the CD4+ TCR pathway_Homo sapiens_03079d1e-6195-11e5-8ac5-06603eb7f303
Hedgehog signaling events mediated by Gli proteins_Homo sapiens_153b6970-6193-11e5-8ac5-06603eb7f303
GMCSF-mediated signaling events_Homo sapiens_095aa3ef-6193-11e5-8ac5-06603eb7f303
ErbB receptor signaling network_Homo sapiens_2c26d51f-6192-11e5-8ac5-06603eb7f303
Signaling events mediated by HDAC Class III_Homo sapiens_a7191c08-6195-11e5-8ac5-06603eb7f303
Signaling mediated by p38-alpha and p38-beta_Homo sapiens_e9dd8c21-6195-11e5-8ac5-06603eb7f303
Glypican 1 network_Homo sapiens_fb91e48c-6192-11e5-8ac5-06603eb7f303
E-cadherin signaling in the nascent adherens junction_Homo sapiens_aef0d8c2-6191-11e5-8ac5-06603eb7f30

LPA receptor mediated events_Homo sapiens_4b994cde-6194-11e5-8ac5-06603eb7f303
Downstream signaling in naive CD8+ T cells_Homo sapiens_92180cef-6191-11e5-8ac5-06603eb7f303
Signaling events mediated by HDAC Class I_Homo sapiens_97e84126-6195-11e5-8ac5-06603eb7f303
Lissencephaly gene (LIS1) in neuronal migration and development_Homo sapiens_41fc83ec-6194-11e5-8ac5-0
S1P3 pathway_Homo sapiens_7cb02d01-6195-11e5-8ac5-06603eb7f303
IL2-mediated signaling events_Homo sapiens_a2a1883c-6193-11e5-8ac5-06603eb7f303
Nongenotropic Androgen signaling_Homo sapiens_843e2f77-6194-11e5-8ac5-06603eb7f303
Thromboxane A2 receptor signaling_Homo sapiens_27d5800d-6196-11e5-8ac5-06603eb7f303
CDC42 signaling events_Homo sapiens_50b98ae0-6190-11e5-8ac5-06603eb7f303
p53 pathway_Homo sapiens_a0de862d-6194-11e5-8ac5-06603eb7f303
ErbB2/ErbB3 signaling events_Homo sapiens_51e35311-6192-11e5-8ac5-06603eb7f303
FOXA1 transcription factor network_Homo sapiens_aa3927b7-6192-11e5-8ac5-06603eb7f303
Signaling events mediated by focal adhesion kinase_Homo sapiens_8fb80085-6195-11e5-8ac5-06603eb7f303
Fc-epsilon receptor I signaling in mast cells_Homo sapiens_86cd7795-6192-11e5-8ac5-06603eb7f303
Noncanonical Wnt signaling pathway_Homo sapiens_7ff5fe76-6194-11e5-8ac5-06603eb7f303
Notch signaling pathway_Homo sapiens_88f83518-6194-11e5-8ac5-06603eb7f303
Sphingosine 1-phosphate (S1P) pathway_Homo sapiens_eff796f3-6195-11e5-8ac5-06603eb7f303
LKB1 signaling events_Homo sapiens_4623bf6d-6194-11e5-8ac5-06603eb7f303
IL8- and CXCR2-mediated signaling events_Homo sapiens_fe78e284-6193-11e5-8ac5-06603eb7f303
EPHA forward signaling_Homo sapiens_f25420e8-6191-11e5-8ac5-06603eb7f303
Posttranslational regulation of adherens junction stability and dissassembly_Homo sapiens_ebb21a59-6194-11e
Angiopoietin receptor Tie2-mediated signaling_Homo sapiens_ad60647c-6188-11e5-8ac5-06603eb7f303
Endothelins_Homo sapiens_dfb9dc47-6191-11e5-8ac5-06603eb7f303
Validated targets of C-MYC transcriptional repression_Homo sapiens_6bbdafa6-6196-11e5-8ac5-06603eb7f303
ALK2 signaling events_Homo sapiens_43d0451b-6187-11e5-8ac5-06603eb7f303
Regulation of retinoblastoma protein_Homo sapiens_407a3468-6195-11e5-8ac5-06603eb7f303
Signaling events mediated by PRL_Homo sapiens_bb67523a-6195-11e5-8ac5-06603eb7f303
Validated targets of C-MYC transcriptional activation_Homo sapiens_61d3b115-6196-11e5-8ac5-06603eb7f303
Trk receptor signaling mediated by PI3K and PLC-gamma_Homo sapiens_4037def0-6196-11e5-8ac5-06603eb7
VEGFR3 signaling in lymphatic endothelium_Homo sapiens_9048d98c-6196-11e5-8ac5-06603eb7f303
Plasma membrane estrogen receptor signaling_Homo sapiens_dcc37895-6194-11e5-8ac5-06603eb7f303
Stabilization and expansion of the E-cadherin adherens junction_Homo sapiens_f1a6cd94-6195-11e5-8ac5-066
S1P4 pathway_Homo sapiens_821b0c12-6195-11e5-8ac5-06603eb7f303
IL5-mediated signaling events_Homo sapiens_e1d816a1-6193-11e5-8ac5-06603eb7f303
Direct p53 effectors_Homo sapiens_67c3b75d-6191-11e5-8ac5-06603eb7f303
SHP2 signaling_Homo sapiens_85755aa4-6195-11e5-8ac5-06603eb7f303
IGF1 pathway_Homo sapiens_5e904cd6-6193-11e5-8ac5-06603eb7f303
PAR4-mediated thrombin signaling events_Homo sapiens_c3b87da1-6194-11e5-8ac5-06603eb7f303
LPA4-mediated signaling events_Homo sapiens_53b0602f-6194-11e5-8ac5-06603eb7f303
Arf6 downstream pathway_Homo sapiens_1e972087-618a-11e5-8ac5-06603eb7f303
Reelin signaling pathway_Homo sapiens_054f432f-6195-11e5-8ac5-06603eb7f303
IL2 signaling events mediated by STAT5_Homo sapiens_9938526b-6193-11e5-8ac5-06603eb7f303
Netrin-mediated signaling events_Homo sapiens_716a3d34-6194-11e5-8ac5-06603eb7f303
Osteopontin-mediated events_Homo sapiens_94bf7f2a-6194-11e5-8ac5-06603eb7f303
Regulation of cytoplasmic and nuclear SMAD2/3 signaling_Homo sapiens_13312fe2-6195-11e5-8ac5-06603eb7
Alpha4 beta1 integrin signaling events_Homo sapiens_aa07df5d-6187-11e5-8ac5-06603eb7f303
Alpha-synuclein signaling_Homo sapiens_588134bc-6187-11e5-8ac5-06603eb7f303
BCR signaling pathway_Homo sapiens_acbf44e2-618c-11e5-8ac5-06603eb7f303
Validated nuclear estrogen receptor alpha network_Homo sapiens_58949883-6196-11e5-8ac5-06603eb7f303
Hypoxic and oxygen homeostasis regulation of HIF-1-alpha_Homo sapiens_4c0f3584-6193-11e5-8ac5-06603eb
Glucocorticoid receptor regulatory network_Homo sapiens_dfba0dfb-6192-11e5-8ac5-06603eb7f303
EGF receptor (ErbB1) signaling pathway_Homo sapiens_NULL
Signaling events mediated by HDAC Class II_Homo sapiens_a1da5607-6195-11e5-8ac5-06603eb7f303

Class I PI3K signaling events mediated by Akt_Homo sapiens_046a84e6-6191-11e5-8ac5-06603eb7f303
CXCR4-mediated signaling events_Homo sapiens_46a5529b-6191-11e5-8ac5-06603eb7f303
Arf6 signaling events_Homo sapiens_3b3d91b8-618a-11e5-8ac5-06603eb7f303
IL2 signaling events mediated by PI3K_Homo sapiens_8bbf39aa-6193-11e5-8ac5-06603eb7f303
Validated transcriptional targets of AP1 family members Fra1 and Fra2_Homo sapiens_76d028a7-6196-11e5-8a
p75(NTR)-mediated signaling_Homo sapiens_b492782f-6194-11e5-8ac5-06603eb7f303
mTOR signaling pathway_Homo sapiens_559dd850-6194-11e5-8ac5-06603eb7f303
E-cadherin signaling in keratinocytes_Homo sapiens_a5f1af61-6191-11e5-8ac5-06603eb7f303
S1P1 pathway_Homo sapiens_7327884f-6195-11e5-8ac5-06603eb7f303
p38 signaling mediated by MAPKAP kinases_Homo sapiens_9d980dbc-6194-11e5-8ac5-06603eb7f303
ATR signaling pathway_Homo sapiens_8991cbac-618b-11e5-8ac5-06603eb7f303
C-MYC pathway_Homo sapiens_e3162de9-618e-11e5-8ac5-06603eb7f303
Internalization of ErbB1_Homo sapiens_3aa9aafa-6194-11e5-8ac5-06603eb7f303
S1P2 pathway_Homo sapiens_7796a240-6195-11e5-8ac5-06603eb7f303
RXR and RAR heterodimerization with other nuclear receptor_Homo sapiens_6ce0a11e-6195-11e5-8ac5-0660
Signaling events mediated by TCPTP_Homo sapiens_cd5ca44d-6195-11e5-8ac5-06603eb7f303
Alpha9 beta1 integrin signaling events_Homo sapiens_0f5519cf-6188-11e5-8ac5-06603eb7f303
p73 transcription factor network_Homo sapiens_a88c505e-6194-11e5-8ac5-06603eb7f303
Effects of Botulinum toxin_Homo sapiens_d236ff84-6191-11e5-8ac5-06603eb7f303
PAR1-mediated thrombin signaling events_Homo sapiens_be5084c0-6194-11e5-8ac5-06603eb7f303
CXCR3-mediated signaling events_Homo sapiens_3a38a0ca-6191-11e5-8ac5-06603eb7f303
Ceramide signaling pathway_Homo sapiens_d3747df2-6190-11e5-8ac5-06603eb7f303
Insulin-mediated glucose transport_Homo sapiens_145e3376-6194-11e5-8ac5-06603eb7f303
VEGF and VEGFR signaling network_Homo sapiens_8957818a-6196-11e5-8ac5-06603eb7f303
Validated transcriptional targets of deltaNp63 isoforms_Homo sapiens_7d7360a8-6196-11e5-8ac5-06603eb7f3
PLK1 signaling events_Homo sapiens_e5e87977-6194-11e5-8ac5-06603eb7f303
Wnt signaling network_Homo sapiens_987a2b9f-6196-11e5-8ac5-06603eb7f303
Sumoylation by RanBP2 regulates transcriptional repression_Homo sapiens_f70a59a5-6195-11e5-8ac5-06603
Class I PI3K signaling events_Homo sapiens_12b82bb7-6191-11e5-8ac5-06603eb7f303
CD40/CD40L signaling_Homo sapiens_1971792f-6190-11e5-8ac5-06603eb7f303
Regulation of p38-alpha and p38-beta_Homo sapiens_3062ca15-6195-11e5-8ac5-06603eb7f303
Arf6 trafficking events_Homo sapiens_7a5b8f09-618a-11e5-8ac5-06603eb7f303
Syndecan-4-mediated signaling events_Homo sapiens_076bc549-6196-11e5-8ac5-06603eb7f303
Regulation of Ras family activation_Homo sapiens_397d91c7-6195-11e5-8ac5-06603eb7f303
Signaling events mediated by PTP1B_Homo sapiens_be498a9b-6195-11e5-8ac5-06603eb7f303
Integrins in angiogenesis_Homo sapiens_2ddeac89-6194-11e5-8ac5-06603eb7f303
Syndecan-2-mediated signaling events_Homo sapiens_fe05ead7-6195-11e5-8ac5-06603eb7f303
JNK signaling in the CD4+ TCR pathway_Homo sapiens_400ebdab-6194-11e5-8ac5-06603eb7f303
HIF-2-alpha transcription factor network_Homo sapiens_37358832-6193-11e5-8ac5-06603eb7f303
ATM pathway_Homo sapiens_49bc3e2b-618b-11e5-8ac5-06603eb7f303
RAC1 signaling pathway_Homo sapiens_faffa4fc-6194-11e5-8ac5-06603eb7f303
Validated nuclear estrogen receptor beta network_Homo sapiens_5fb983a4-6196-11e5-8ac5-06603eb7f303
DNA-PK pathway in nonhomologous end joining_Homo sapiens_8dc10ede-6191-11e5-8ac5-06603eb7f303
Regulation of RAC1 activity_Homo sapiens_351aacd6-6195-11e5-8ac5-06603eb7f303
Syndecan-3-mediated signaling events_Homo sapiens_052075b8-6196-11e5-8ac5-06603eb7f303
Aurora B signaling_Homo sapiens_304a75af-618c-11e5-8ac5-06603eb7f303
Arf1 pathway_Homo sapiens_faef51c6-6189-11e5-8ac5-06603eb7f303
Beta3 integrin cell surface interactions_Homo sapiens_c2800165-618d-11e5-8ac5-06603eb7f303
RhoA signaling pathway_Homo sapiens_5c6b5f5c-6195-11e5-8ac5-06603eb7f303
FOXA2 and FOXA3 transcription factor networks_Homo sapiens_b6933be8-6192-11e5-8ac5-06603eb7f303
PDGFR-alpha signaling pathway_Homo sapiens_c66cc833-6194-11e5-8ac5-06603eb7f303
Regulation of RhoA activity_Homo sapiens_49ece019-6195-11e5-8ac5-06603eb7f303
Canonical NF-kappaB pathway_Homo sapiens_7a1a9c3c-618f-11e5-8ac5-06603eb7f303

Visual signal transduction: Cones_Homo sapiens_9433a84d-6196-11e5-8ac5-06603eb7f303
Fanconi anemia pathway_Homo sapiens_6befb873-6192-11e5-8ac5-06603eb7f303
IL3-mediated signaling events_Homo sapiens_c868db9f-6193-11e5-8ac5-06603eb7f303
p38 MAPK signaling pathway_Homo sapiens_99bea41b-6194-11e5-8ac5-06603eb7f303
Validated transcriptional targets of TAp63 isoforms_Homo sapiens_82e8ee19-6196-11e5-8ac5-06603eb7f303
IL8- and CXCR1-mediated signaling events_Homo sapiens_f6a58ef3-6193-11e5-8ac5-06603eb7f303
TRAIL signaling pathway_Homo sapiens_3a79fddf-6196-11e5-8ac5-06603eb7f303
Caspase Cascade in Apoptosis_Homo sapiens_b9d3ef2e-618f-11e5-8ac5-06603eb7f303
BARD1 signaling events_Homo sapiens_75b04491-618c-11e5-8ac5-06603eb7f303
Ephrin B reverse signaling_Homo sapiens_149a63dc-6192-11e5-8ac5-06603eb7f303
Regulation of CDC42 activity_Homo sapiens_0ffe6681-6195-11e5-8ac5-06603eb7f303
Nephrin/Neph1 signaling in the kidney podocyte_Homo sapiens_6cfb9873-6194-11e5-8ac5-06603eb7f303
IL12 signaling mediated by STAT4_Homo sapiens_72cf19b8-6193-11e5-8ac5-06603eb7f303
IL4-mediated signaling events_Homo sapiens_cff33f50-6193-11e5-8ac5-06603eb7f303
HIV-1 Nef: Negative effector of Fas and TNF-alpha_Homo sapiens_41a75463-6193-11e5-8ac5-06603eb7f303
TCR signaling in naive CD4+ T cells_Homo sapiens_0c2862fa-6196-11e5-8ac5-06603eb7f303
Beta1 integrin cell surface interactions_Homo sapiens_2fd0bc63-618d-11e5-8ac5-06603eb7f303
FAS (CD95) signaling pathway_Homo sapiens_79cc9c14-6192-11e5-8ac5-06603eb7f303
Urokinase-type plasminogen activator (uPA) and uPAR-mediated signaling_Homo sapiens_503076a2-6196-11e
Insulin Pathway_Homo sapiens_073b9f25-6194-11e5-8ac5-06603eb7f303
a6b1 and a6b4 Integrin signaling_Homo sapiens_73d1a893-6186-11e5-8ac5-06603eb7f303
TNF receptor signaling pathway_Homo sapiens_316be05e-6196-11e5-8ac5-06603eb7f303
Syndecan-1-mediated signaling events_Homo sapiens_f957cc16-6195-11e5-8ac5-06603eb7f303
TCR signaling in naive CD8+ T cells_Homo sapiens_15a017bb-6196-11e5-8ac5-06603eb7f303
IL12-mediated signaling events_Homo sapiens_7acdea19-6193-11e5-8ac5-06603eb7f303

Overlap	P.value	Adjusted.P.value	Old.P.value	Old.Adjusted
15/81	1,46368E-05	0,00305909	0	0
10/54	0,000385529	0,040287746	0	0
6/20	0,000385926	0,026886145	0	0
7/28	0,0004346	0,02270783	0	0
12/77	0,000551486	0,023052101	0	0
11/67	0,000590265	0,02056091	0	0
9/48	0,000680763	0,020325638	0	0
10/59	0,000801712	0,020944713	0	0
8/40	0,000860022	0,019971618	0	0
8/40	0,000860022	0,017974456	0	0
8/42	0,001204829	0,022891753	0	0
5/17	0,001331814	0,023195766	0	0
6/27	0,002162854	0,034772044	0	0
8/46	0,002220208	0,033144533	0	0
3/6	0,002435749	0,033938098	0	0
8/52	0,004878214	0,063721667	0	0
8/53	0,00549151	0,067513266	0	0
14/128	0,006341332	0,073629911	0	0
6/34	0,007229061	0,07951967	0	0
6/34	0,007229061	0,075543687	0	0
9/68	0,007990561	0,079525111	0	0
5/25	0,008117773	0,077118847	0	0
7/48	0,011043783	0,100354375	0	0
9/72	0,011504736	0,100187079	0	0
6/38	0,012465912	0,104215023	0	0
6/38	0,012465912	0,100206752	0	0
8/61	0,012726362	0,09851147	0	0
6/39	0,014106314	0,105293558	0	0
4/19	0,014673856	0,10575296	0	0
5/29	0,01531179	0,106672138	0	0
5/29	0,01531179	0,103231101	0	0
6/40	0,015891236	0,103789635	0	0
7/52	0,016813887	0,106487948	0	0
11/105	0,019565439	0,120269902	0	0
8/66	0,019843043	0,118491317	0	0
9/79	0,020260427	0,117623036	0	0
9/82	0,025196961	0,142328781	0	0
8/69	0,025269433	0,13898188	0	0
5/33	0,025851548	0,138537781	0	0
6/45	0,02717002	0,141963355	0	0
7/58	0,029025289	0,14795818	0	0
6/46	0,029929579	0,148935287	0	0
6/46	0,029929579	0,145471675	0	0
7/59	0,031521278	0,14972607	0	0
3/14	0,03254043	0,151132219	0	0
6/47	0,032866475	0,149328114	0	0
5/36	0,036210659	0,161021865	0	0
3/15	0,039153343	0,170480183	0	0
4/26	0,042615227	0,18176699	0	0
5/38	0,044350814	0,185386402	0	0
4/27	0,048056222	0,196936281	0	0
5/39	0,048798539	0,196132589	0	0

7/65	0,049514039	0,195253473	0	0
7/66	0,053034329	0,205262496	0	0
7/66	0,053034329	0,201530451	0	0
4/29	0,060030377	0,224041943	0	0
4/29	0,060030377	0,220111382	0	0
6/55	0,06305942	0,227231358	0	0
4/30	0,066556032	0,235766284	0	0
6/56	0,067693612	0,235799416	0	0
7/70	0,068652616	0,235219617	0	0
6/57	0,072520364	0,244463807	0	0
5/44	0,074831689	0,24825116	0	0
5/44	0,074831689	0,244372235	0	0
6/58	0,07753903	0,249317803	0	0
6/58	0,07753903	0,24554026	0	0
4/32	0,080655885	0,251598209	0	0
6/59	0,082748589	0,254330224	0	0
3/21	0,0912632	0,276434911	0	0
5/47	0,093435321	0,278971173	0	0
4/34	0,096102152	0,282892251	0	0
4/34	0,096102152	0,278963192	0	0
5/48	0,10011571	0,28663265	0	0
5/48	0,10011571	0,282759236	0	0
6/63	0,105462757	0,293889549	0	0
6/63	0,105462757	0,290022581	0	0
2/11	0,107443713	0,291632936	0	0
6/64	0,111599028	0,299028164	0	0
3/23	0,112800229	0,298420859	0	0
7/79	0,112802756	0,2946972	0	0
4/36	0,112819132	0,291101218	0	0
3/25	0,136044545	0,346747681	0	0
4/40	0,149700503	0,376956688	0	0
4/41	0,159564969	0,39701284	0	0
2/14	0,160856918	0,395518776	0	0
2/14	0,160856918	0,39091972	0	0
10/136	0,165162226	0,396769026	0	0
5/57	0,169976421	0,403694	0	0
3/28	0,173579701	0,407619748	0	0
2/15	0,179580553	0,41702595	0	0
2/15	0,179580553	0,412443247	0	0
2/15	0,179580553	0,407960169	0	0
3/29	0,18668155	0,419531654	0	0
3/30	0,200028832	0,444744956	0	0
3/30	0,200028832	0,44006343	0	0
3/31	0,213592618	0,465008929	0	0
2/17	0,217857207	0,469403673	0	0
3/32	0,227344696	0,484847361	0	0
3/32	0,227344696	0,479949913	0	0
5/64	0,234160515	0,489395476	0	0
5/64	0,234160515	0,484549976	0	0
2/18	0,237255632	0,486141443	0	0
6/82	0,248252399	0,503735449	0	0
3/34	0,255304963	0,513064781	0	0
3/34	0,255304963	0,508178449	0	0

3/34	0,255304963	0,503384313	0	0
7/100	0,257790801	0,503535303	0	0
3/35	0,269461014	0,521456963	0	0
3/35	0,269461014	0,516672954	0	0
3/35	0,269461014	0,511975927	0	0
5/68	0,27349548	0,514959958	0	0
5/69	0,283544244	0,529113813	0	0
2/21	0,295710622	0,546933805	0	0
2/21	0,295710622	0,54213614	0	0
2/21	0,295710622	0,537421913	0	0
3/39	0,326695464	0,588615103	0	0
2/23	0,334368124	0,597290068	0	0
3/40	0,341046404	0,604056767	0	0
2/24	0,353472123	0,620803981	0	0
2/24	0,353472123	0,615630614	0	0
3/42	0,369659561	0,638502879	0	0
2/25	0,372378865	0,637927728	0	0
5/78	0,3760808	0,639031604	0	0
1/9	0,379299997	0,639304027	0	0
3/43	0,383886151	0,641857645	0	0
3/43	0,383886151	0,636763537	0	0
3/44	0,398037404	0,655037933	0	0
2/27	0,409482246	0,66860773	0	0
1/10	0,411342481	0,666438593	0	0
3/45	0,412098092	0,662526932	0	0
3/46	0,426053988	0,679734988	0	0
2/28	0,42762859	0,677078601	0	0
1/11	0,441732347	0,694150831	0	0
3/48	0,453599363	0,707479603	0	0
2/30	0,463008482	0,716805724	0	0
2/30	0,463008482	0,711535094	0	0
3/49	0,467165176	0,71268264	0	0
2/31	0,480209569	0,727273912	0	0
2/31	0,480209569	0,722041726	0	0
3/52	0,50691194	0,756747111	0	0
4/72	0,513215153	0,76072317	0	0
2/33	0,513571053	0,755889789	0	0
1/14	0,523816115	0,765577399	0	0
2/34	0,529712675	0,768819091	0	0
2/34	0,529712675	0,76351689	0	0
3/54	0,532531736	0,762322828	0	0
1/15	0,548404355	0,779704151	0	0
1/16	0,571724118	0,807367167	0	0
2/38	0,590548963	0,828353914	0	0
1/17	0,593840785	0,82741816	0	0
2/39	0,604811304	0,83712293	0	0
1/19	0,63470968	0,87272581	0	0
2/43	0,658077154	0,898941994	0	0
2/45	0,682476808	0,926218525	0	0
2/45	0,682476808	0,920242921	0	0
1/22	0,688439703	0,92233268	0	0
2/46	0,694132378	0,924036096	0	0
1/23	0,704533848	0,931946673	0	0

1/23	0,704533848	0,926085373	0	0
2/47	0,705431946	0,921470479	0	0
1/26	0,74800173	0,971008457	0	0
1/27	0,761021692	0,981811936	0	0
2/53	0,766093226	0,982291314	0	0
1/28	0,773369566	0,98557463	0	0
1/28	0,773369566	0,97960145	0	0
2/55	0,783756324	0,986777541	0	0
1/29	0,785080013	0,982525286	0	0
1/30	0,796185909	0,990493185	0	0
1/30	0,796185909	0,984632278	0	0
1/31	0,806718435	0,991789135	0	0
1/32	0,816707165	0,998197646	0	0
2/60	0,822866924	0,999878995	0	0
1/35	0,84368393	1	0	0
2/64	0,849450754	1	0	0
2/66	0,861333792	1	0	0
1/38	0,866693525	1	0	0
1/42	0,892196776	1	0	0
1/43	0,897771034	1	0	0
1/45	0,908070493	1	0	0
1/46	0,912824654	1	0	0
1/46	0,912824654	1	0	0
1/53	0,939891944	1	0	0
1/62	0,962738684	1	0	0

Odds.Ratio	Combined.Score
3,588860178	39,95109164
3,588860178	28,211653
5,813953488	45,6968961
4,84496124	37,5052585
3,020235578	22,66050952
3,181765591	23,65623106
3,63372093	26,49816985
3,284719485	23,41598251
3,875968992	27,35873171
3,875968992	27,35873171
3,69139904	24,81143421
5,6999544	37,74061275
4,306632214	26,42690116
3,370407819	20,5937122
9,689922481	58,30911925
2,981514609	15,87053117
2,925259617	15,22466601
2,119670543	10,72694556
3,41997264	16,85925491
3,41997264	16,85925491
2,56497948	12,38755367
3,875968992	18,65774953
2,82622739	12,73466308
2,42248062	10,81636742
3,05997552	13,41725036
3,05997552	13,41725036
2,541619011	11,09182788
2,981514609	12,70462963
4,07996736	17,22434882
3,34135258	13,963954
3,34135258	13,963954
2,906976744	12,04066138
2,608825283	10,65848654
2,030269472	7,987061038
2,349072117	9,208131988
2,207830439	8,608520067
2,127056154	7,82976151
2,246938546	8,264599055
2,936340146	10,73345316
2,583979328	9,316902113
2,338946806	8,278907568
2,527805865	8,869838268
2,527805865	8,869838268
2,299303639	7,9489053
4,15282392	14,22455135
2,474022761	8,449535228
2,691645134	8,931959959
3,875968992	12,55918396
2,981514609	9,408299464
2,5499796	7,944778207
2,871088142	8,714854065
2,484595508	7,503614852

2,087060227	6,27265751
2,055438102	6,036443209
2,055438102	6,036443209
2,673082064	7,51912473
2,673082064	7,51912473
2,114164905	5,842870663
2,583979328	7,001837457
2,07641196	5,591286245
1,937984496	5,191271411
2,03998368	5,352688448
2,202255109	5,709376829
2,202255109	5,709376829
2,004811548	5,126250728
2,004811548	5,126250728
2,42248062	6,098748804
1,970831691	4,911210702
2,76854928	6,627928123
2,061685634	4,887196592
2,27998176	5,340500606
2,27998176	5,340500606
2,01873385	4,645971938
2,01873385	4,645971938
1,84569952	4,15171171
1,84569952	4,15171171
3,523608175	7,860423421
1,816860465	3,984089648
2,527805865	5,516018479
1,717201452	3,7471302
2,153316107	4,698469725
2,325581395	4,639006775
1,937984496	3,68046246
1,890716582	3,470039912
2,76854928	5,058804027
2,76854928	5,058804027
1,4249886	2,566158088
1,6999864	3,012538338
2,07641196	3,636043216
2,583979328	4,437032068
2,583979328	4,437032068
2,583979328	4,437032068
2,004811548	3,364777581
1,937984496	3,118786362
1,937984496	3,118786362
1,875468867	2,895132655
2,27998176	3,474499418
1,816860465	2,691293478
1,816860465	2,691293478
1,514050388	2,198020285
1,514050388	2,198020285
2,153316107	3,097797377
1,418037436	1,975764768
1,70998632	2,334638367
1,70998632	2,334638367

1,70998632	2,334638367
1,356589147	1,839001571
1,661129568	2,178291625
1,661129568	2,178291625
1,661129568	2,178291625
1,4249886	1,847455232
1,404336591	1,770007728
1,84569952	2,248752181
1,84569952	2,248752181
1,84569952	2,248752181
1,490757305	1,667750218
1,68520391	1,846162327
1,453488372	1,563570827
1,61498708	1,679506874
1,61498708	1,679506874
1,38427464	1,377592472
1,550387597	1,531540293
1,242297754	1,214906659
2,153316107	2,087484578
1,352082207	1,294496013
1,352082207	1,294496013
1,321353066	1,217242729
1,435544071	1,281742365
1,937984496	1,721568072
1,291989664	1,145340919
1,263902932	1,078348343
1,38427464	1,17594164
1,761804087	1,43948402
1,21124031	0,957535043
1,291989664	0,994844838
1,291989664	0,994844838
1,18652112	0,903028463
1,250312578	0,917145122
1,250312578	0,917145122
1,118067979	0,759635486
1,076658053	0,718195651
1,174536058	0,782671939
1,38427464	0,895092168
1,13999088	0,724373623
1,13999088	0,724373623
1,076658053	0,678416004
1,291989664	0,776152959
1,21124031	0,6772029
1,01999184	0,537232486
1,13999088	0,594099447
0,993838203	0,499740374
1,01999184	0,463675624
0,901388138	0,377170632
0,861326443	0,329049728
0,861326443	0,329049728
0,880902044	0,328864994
0,842601955	0,30762773
0,842601955	0,295095132

0,842601955	0,295095132
0,824674254	0,287765937
0,745378652	0,216420683
0,717772036	0,196018818
0,731314904	0,194859889
0,69213732	0,177878081
0,69213732	0,177878081
0,704721635	0,171710442
0,668270516	0,161701175
0,645994832	0,1472368
0,645994832	0,1472368
0,625156289	0,134271427
0,605620155	0,122622744
0,645994832	0,125943661
0,553709856	0,094118131
0,605620155	0,0988162
0,587268029	0,087663361
0,50999592	0,072965042
0,46142488	0,052634076
0,450694069	0,048602946
0,430663221	0,041530262
0,421300977	0,038427482
0,421300977	0,038427482
0,365657452	0,022667239
0,312578145	0,011869611

Genes
NCOA2;DLX1;CREBBP;RUNX3;ESR1;FOXO1;RUNX1;SMAD7;KAT2B;RBL1;CREB1;SIN3A;SP1;MYOD1;SNIF
TGFBR3;PPP2CA;ITCH;OCLN;SMURF2;XIAP;CTNNB1;RNF111;CTGF;SMAD7
RANBP3;FZD5;APC;CUL3;CTNNB1;LRP6
NCOA2;KAT2B;CREBBP;RXRA;NRIP1;CDK1;MAPK1
MAP2K1;RAP1A;APC;SH3KBP1;GAB1;MAPK1;CTNNB1;PTPN11;RAF1;CRK;EIF4E;SH3GL2
SIN3A;TNKS;XRCC5;SP1;E2F1;TERF2IP;MAPK1;HNRNPC;MXD1;ESR1;RAD9A
MAPK10;CSNK1G3;KAT2B;RALA;CREBBP;CSNK1A1;CTNNB1;SOD2;FOXO1
NCOA2;CCND3;XRCC5;CARM1;ZNF318;NRIP1;PTK2B;CTNNB1;APPL1;PIAS1
CREBBP;MAP2K1;RAP1A;MAPK1;PTPN11;CALM1;PIAS1;SMAD7
CREBBP;MAP2K1;SP1;MMP2;NFATC3;CDK1;ESR1;CKS1B
BMPR2;SMURF2;XIAP;SOSTDC1;MAPK1;SMAD9;SMAD7;BMPR1A
FZD5;APC;CSNK1A1;CTNNB1;LRP6
NRP2;SHC2;MAPK1;PTPN11;CALM1;HIF1A
CREBBP;APH1B;APC;CSNK1A1;DTX1;MAPK1;CTNNB1;LRP6
MAP2K1;PDE3B;MAPK1
LYN;SPRED1;MAP2K1;CREBBP;GAB1;PTPN11;RAF1;EPOR
RCAN1;CREBBP;CSNK1A1;CAMK4;RCAN2;NFATC3;CHP1;CALM1
LYN;NCKAP1;MAP2K1;GAB1;PTPN11;ACTN4;EPS8;PPP2CA;MAPK10;RAP1A;S1PR1;MAPK1;RAF1;CRK
ROCK1;CDH2;CTNNB1;PTPN11;CALM1;FGFR1
MAP3K2;MAP2K1;RAP1A;CREB1;MAPK1;RAF1
MAP2K1;ROCK1;GAB1;PTK2B;MAPK1;CTNNB1;PTPN11;RAF1;CALM1
BMPR2;MAPK1;SMAD9;INHBA;SMAD7
RCAN1;CREBBP;MAML1;MYOD1;GATA6;E2F1;HIF1A
KAT2B;CCND3;CREBBP;RRM1;RBL1;CBX5;SP1;E2F1;CDK1
ITCH;NRG3;ERBB4;MAPK1;GRIN2B;EREG
RAP1A;CREB1;GAB1;MAPK1;PTPN11;CRK
MAP2K1;SHC2;RAP1A;GAB1;ELMO1;MAPK1;PTPN11;CRK
MAP2K1;EFNB3;RAP1A;ROCK1;MAPK1;CRK
MAP2K1;MAPK1;ANTXR2;CALM1
RAP1A;PTPRM;CTNNB1;CLDN1;CRK
RCAN1;RCAN2;NFATC3;CHP1;CALM1
LYN;RAP1A;ROCK1;MMP2;JAM2;CTGF
NCOA2;KAT2B;CREBBP;RXRA;CARM1;TRIM24;FOXO1
EPS8;PPP2CA;MAP3K2;NCKAP1;MAP2K1;RALA;CREB1;GAB1;MAPK1;RAF1;CALM1
NCOA2;CREBBP;CREB1;PFKFB3;SP1;ID2;HIF1A;HK2
NCOA2;APC;TBL1XR1;SALL4;MMP2;ID2;CAMK4;IGF2BP1;CTNNB1
CREBBP;MAF;MAT2A;SIN3A;SP1;MYOD1;PAX5;ATP2B1;HIPK2
MAF;CREB1;SP1;NFATC3;CDK1;CTNNB1;HIF1A;ESR1
LYN;RAP1A;GAB1;PTPN11;EPOR
CREB1;CDC37;RUVBL1;CTNNB1;PARVA;TNS1
HRK;CREB1;MMP2;CUL3;MAPK1;SELE;ESR1
CRP;GAB1;PTPN11;IL6R;FOXO1;PIAS1
ITCH;MAF;EGR4;E2F1;NFATC3;CALM1
CDH2;GAB1;PTK2B;MAPK1;PTPN11;IL17RD;FGFR1
MAP2K1;MAPK1;RAF1
CSNK1G3;CREBBP;MAP2K1;SIN3A;CSNK1A1;PIAS1
LYN;MAP2K1;MAPK1;PTPN11;RAF1
NRG3;ERBB4;EREG
KAT2B;CREBBP;MYOD1;FOXO1
ELK4;MEF2A;CREB1;EIF4E;ESR1
LYN;GPC1;SLIT2;FGFR1
ENAH;NCKAP1;RAP1A;CTNNB1;CRK

GNA13;LYN;ADCY9;MMP2;GAB1;PTK2B;CRK
EOMES;MAP2K1;EGR4;NFATC3;MAPK1;RAF1;CALM1
KAT2B;HDAC5;CREBBP;SIN3A;MXD1;GATAD2B;SMAD7
DCX;CALM1;LRP8;PAFAH1B2
GNA13;S1PR1;MAPK1;S1PR3
MAP2K1;PTK2B;MAPK1;PTPN11;RAF1;IKZF3
MAP2K1;CREB1;MAPK1;RAF1
GNA13;LYN;ROCK1;SELE;RAB11A;TGM2
EPS8;ENAH;APC;MAPK1;CTNNB1;RAF1;CDC42BPA
PPP2CA;CSNK1G3;KAT2B;CREBBP;CSNK1A1;HIPK2
MAPK10;MAP2K1;MAPK1;PTPN11;RAF1
CREBBP;NFIA;SP1;NRIP1;ESR1
MAP2K1;RAP1A;ELMO1;MAPK1;RAF1;CRK
LYN;MAP2K1;S1PR1;MAPK1;PTPN11;RAF1
MAPK10;ROCK1;FZD5;CSNK1A1
ITCH;APH1B;MAML1;ADAM12;DTX1;RAB11A
GNA13;S1PR1;S1PR3
CREB1;CDC37;SIK3;SIK2;ESR1
PPP2CA;LYN;ELMO1;RAB11A
LYN;EFNA3;ROCK1;CRK
GNA13;SNX1;CREBBP;CDH2;CTNNB1
MMP2;MAPK1;PTPN11;CRK;FOXO1
MAP2K1;ADCY9;PTK2B;MAPK1;RAF1;CRK
SFRP1;RBL1;CREB1;SP1;ID2;DNMT3A
BMPR2;SMAD9
PPP2CA;CCND3;CREBBP;MYOD1;E2F1;RAF1
PTP4A1;ROCK1;MAPK1
CREBBP;GPAM;ID2;RUVBL1;COMMD3-BMI1;EIF4E;MTDH
CREB1;CAMK4;AGAP2;GAB1
CREB1;MAPK1;CRK
GNA13;MMP2;STRN;ESR1
ENAH;NCKAP1;ROCK1;CTNNB1
GNA13;MAPK1
LYN;PTPN11
CREBBP;RRM2B;APC;SP1;MMP2;CARM1;JMY;TYRP1;E2F1;SCN3B
MAP2K1;GAB1;PTPN11;RAF1;IL6R
PTPN11;RAF1;CRK
GNA13;ROCK1
ADCY9;CREB1
MAPK1;RAB11A
RAP1A;LRP8;GRIN2B
CCND3;SP1;PTPN11
MAP2K1;ELMO1;MAPK1
MMP2;PTK2B;MAPK1
MAPK1;CALM1
PTK2B;CRK;JAM2
LYN;PTK2B;MAPK1
LYN;MAP2K1;MAPK1;RAF1;CALM1
NCOA2;SET;NRIP1;LCOR;ESR1
HIF1A;RBX1
MAPK10;NCOA2;CREBBP;CREB1;MAPK1;SELE
GAB1;MAPK1;PTPN11
HDAC5;CAMK4;ESR1

RAF1;FOXO1;KPNA1
LYN;GNA13;ITCH;PTK2B;PTPN11;CRK;FOXO1
CYTH3;ACAP2;TSHR
E2F1;PTPN11;CALM1
SP1;MMP2;NFATC3
MAPK10;APH1B;SORT1;E2F1;XIAP
MAP2K1;PDCD4;MAPK1;RAF1;EIF4E
CTNNB1;FMN1
S1PR1;MAPK1
CREB1;RAF1
PPP2CA;CLSPN;RAD9A
PPP2CA;RUVBL1
SH3KBP1;RAF1;SH3GL2
GNA13;MAPK1
ABCA1;RXRA
CREBBP;GAB1;PIAS1
ADAM12;TGM2
ITCH;SP1;CDK1;NSG1;GRAMD4
RAB3GAP2
GNA13;SNX1;ROCK1
MAP2K1;MAPK1;RAF1
MAP2K1;MAPK1;RAF1
LNPEP;CALM1
NRP2
ITCH;DLX6;RUNX1
PPP2CA;CDK1;CLSPN
FZD5;LRP6
PIAS1
CYTH3;LYN;RAP1A
MAPK10;TNFAIP3
LYN;RALA
RALA;CTNNB1;TSHR
ADAM12;FGFR1
CALM1;SOS2
LYN;CDH2;CRK
ROCK1;PTK2B;MAPK1;PTPN11
MMP2;MAPK1
CRK
CREBBP;SP1
SMC1A;RAD9A
NCKAP1;CTNNB1;CRK
NCOA2
XRCC5
EPS8;ELMO1
APH1B
CBX5;CUL3
AP2M1
CD47;L1CAM
ROCK1;SH3GL2
CREB1;SP1
CRK
ARHGEF10;ARHGEF5
TNFAIP3

SLC24A2
USP1;RAD9A
PTPN11
CALM1
ITCH;SP1
LYN
MAPK1
DFFA;XIAP
XRCC5
LYN
APC
MAPK10
CREBBP
SP1;IL13RA1
DFFA
RAP1A;PTPN11
JAM2;TGM2
MAPK10
CRK
PTPN11
RXRA
TNFAIP3
MAPK1
RAP1A
EOMES

P1
