# Supplementary material for: Circulating miR-330-3p in Late Pregnancy is Associated with Pregnancy Outcomes Among Lean Women with GDM
Source: Sci Rep. 2020 Jan 22;10:908. doi: 10.1038/s41598-020-57838-6 (PMC6976655; doi:10.1038/s41598-020-57838-6)
Supplement: Supplementary file 1 — Supplementary data. [file 41598_2020_57838_MOESM1_ESM.zip › Supplimentary File_EnrichR_Analysis NURSA_Endogenous_Complexome.pdf]

Term	Overlap	P.value	Adjusted.P.value
BL2746 (C1D)	14/97	0,00044404	0,797495179
BL4923 (TRIM29)	32/354	0,001540601	1
a-CLSPN (CLSPN)	24/259	0,004122345	1
ab_N_YW (RFWD3)	33/394	0,004392393	1
ab_M_YW (RFWD3)	37/460	0,005168788	1
BL2378 (RFWD3)	24/268	0,006282132	1
BL360 (HLTF)	13/115	0,006363472	1
BL2805 (UBR5)	15/142	0,006643321	1
BL5230 (TAF2)	7/44	0,006864818	1
BL963 (ROCK2)	12/104	0,007301214	1
JQ021 (CCAR1)	10/81	0,008662607	1
BL4102 (PRMT6)	29/351	0,008697481	1
BL978 (PLK1)	14/133	0,008816863	1
JQ007 (MSH6)	28/337	0,009133538	1
BL2227 (FIGNL1)	23/263	0,009665891	1
BL7740 (CCNK)	16/162	0,009701237	1
BL5480 (BRD3)	11/96	0,010486532	1
BL1000 (FANCI)	11/96	0,010486532	1
BL2377 (RFWD3)	9/71	0,01053461	0,995797838
BL5901 (MARVELD2)	10/85	0,01201107	1
SC-14016 (PIAS1)	6/38	0,012465912	1
BL446 (UBE3A)	8/61	0,012726362	1
a-Mip1 ()	17/182	0,013240819	1
BL567 ()	13/126	0,013285839	0,994223648
BL6566 (KNTC1)	14/141	0,014297494	1
SC-11421 (HDAC7)	16/170	0,0149079	1
CBC2202 (RXRB)	11/101	0,015004677	0,99808891
ZMYND8-D23 (ZMYND8)	15/156	0,015088287	0,967805813
BL7901 (POLK)	15/156	0,015088287	0,934433199
BL2763 (HNRNPU)	19/214	0,015176509	0,908567003
BL7137 (CCDC28A)	13/129	0,01592693	0,922734391
BL351 (HELLS)	14/143	0,016008971	0,898503471
BL2700 (STK24)	25/306	0,016020407	0,871898536
BL2151 (TPR)	12/116	0,016627521	0,878324371
CBC552 (TACC3)	21/248	0,018072885	0,92739717
BL6564 (BTF3)	8/65	0,018234756	0,909711741
BL1751 (MED14)	11/104	0,018339958	0,890231495
BL3463 (CASC5)	21/249	0,018814567	0,889235849
BL8409 (ASAP1)	11/105	0,019565439	0,901013528
BL1730 (SPEN)	20/235	0,019698321	0,884454624
BL4945 (TRIP10)	8/66	0,019843043	0,8692221
BL1052 (N4BP2)	27/346	0,021721587	0,928856455
BL2851 (MED18)	7/55	0,022346869	0,933371547
BL2754 (LCOR)	25/319	0,025257304	1
BL5423 (ERCC3)	27/351	0,025513549	1
SC-8987 (HNF4A)	19/227	0,026304708	1
BL2429 (NCOA4)	23/289	0,02650039	1
BL5895 (MAP4K4)	18/212	0,026500501	0,991560413
EPI-2602-1 (COPS3)	11/110	0,026615725	0,975547807
BL6227 (PBXIP1)	8/70	0,027287786	0,980177264
BL6647 (EPS15)	18/213	0,027607774	0,972226716
BL2271 (TCF12)	14/155	0,029756494	1

BL5315 (POLI)	10/99	0,03158489	1
BL7252 (MLH3)	11/114	0,033451758	1
BL2148 (TPR)	11/114	0,033451758	1
BL1041 (QKI)	10/100	0,033538624	1
BL7553 (DICER1)	10/100	0,033538624	1
BL4004 (USP15)	10/100	0,033538624	1
BL3602 (TP53BP2)	20/249	0,033748405	1
BL3734 (CBX4)	13/143	0,033789229	1
CBC183 (BICD2)	14/158	0,034247457	1
BL2793 (TRIM24)	22/281	0,034503456	0,999487198
BL1828 (RCN1)	12/129	0,034699522	0,98921178
BL6872 (ALMS1)	20/250	0,034980463	0,981639246
BL3066 (PPM1D)	13/144	0,035463137	0,979873752
BL6198 (NR2F6)	13/144	0,035463137	0,96502718
BL2844 (MED12)	10/101	0,035575323	0,953631041
BL4408 (PAPOLA)	14/159	0,035847808	0,946803857
BL8936 (STK11IP)	8/74	0,036474739	0,949400456
BL406 (RFC3)	23/299	0,037019378	0,949811469
BL5999 (FOXM1)	17/206	0,038362413	0,970406943
BL991 (INCENP)	19/237	0,038403752	0,957960265
BL6443 (CCNT2)	20/253	0,03887654	0,956469383
BL409 (UPF1)	40/581	0,03924371	0,952455447
BL4711 (PDCD4)	10/103	0,039903315	0,955551384
BL2734 (SRA1)	5/37	0,040155428	0,948936163
BL5726 (DFFA)	5/37	0,040155428	0,936612317
BL3841 (KDM5B)	18/223	0,040665659	0,936352858
a-CCND1 (Ab3) (CCND1)	17/208	0,041429904	0,941874789
BL4015 (PPP1R13B)	26/351	0,041458956	0,930753566
BL6690 (KDM5D)	26/351	0,041458956	0,919262781
SC-9959 (HDAC2)	8/76	0,041766483	0,914787848
BL405 (RFC3)	18/224	0,042182131	0,912760333
SC-585 (EP300)	10/104	0,042197291	0,902218269
BL5439 (GEMIN5)	9/90	0,042399895	0,895884839
BL7546 (FOXP3)	26/352	0,042665566	0,891015779
BL2443 (RPL7)	5/38	0,044350814	0,915563926
BL1741 (CDK7)	16/195	0,045484687	0,928301113
BL1870 (TRIP4)	20/258	0,046062815	0,929537257
BL7007 (ZNF574)	7/64	0,046145498	0,920859048
BL1858 (TRIP6)	14/165	0,04659188	0,919549625
CBC191 (ERCC6L)	10/106	0,047051385	0,918524871
BL1882 (DDIT4)	10/106	0,047051385	0,908648259
BL2852 (MED18)	8/78	0,047543369	0,908381812
BL5973 (ZC3H11A)	11/121	0,048243236	0,912051074
BL9055 (CUL2)	5/39	0,048798539	0,912939333
BL8759 (CDKN1C)	10/107	0,049613756	0,918621711
BL3061 (UBR4)	10/107	0,049613756	0,90924802
BL2397 (DDB2)	17/213	0,049863745	0,904598845
SC-286 (CSK)	15/182	0,049957778	0,897241699
BL2385 (ERCC4)	24/325	0,05001418	0,889361057
BL7874 (RAPGEF6)	6/52	0,050312853	0,885900829
SC-30169 (NR0B2)	6/52	0,050312853	0,87729985
BL1582 (E4F1)	26/358	0,050455302	0,871324251
BL956 (NF1)	8/79	0,050617506	0,86580038

BL8865 (PRKCA)	7/66	0,053034329	0,898581652
BL2031 (RFWD2)	28/393	0,05322037	0,893306399
BL1003 (ATF7IP)	23/311	0,053405767	0,888118125
BL7446 (ZNF777)	9/94	0,053411269	0,880060906
BL7759 (ABCC4)	9/94	0,053411269	0,872060352
GTX111188 (BRE)	12/138	0,053450305	0,864835559
BL4548 (HMG20B)	18/231	0,05395512	0,865208888
BL6423 (CCNC)	15/184	0,053987625	0,858068799
BL6040 (WDR43)	6/53	0,054369367	0,856555988
BL3172 (MAVS)	12/139	0,055887278	0,872813482
BL5074 (FANCI)	12/139	0,055887278	0,865289228
SC-543 (ESR1)	26/362	0,056191328	0,862560904
BL8550 (ITPR1)	9/95	0,056429679	0,858878836
BL8118 (HTATSF1)	7/67	0,056707536	0,855854909
BL2275 (MKRN1)	7/67	0,056707536	0,848722785
BL294 (RPRD2)	20/265	0,057664216	0,855908525
BL5039 (CHD9)	10/110	0,057854045	0,851687413
BL5947 (ZNRF2)	10/110	0,057854045	0,844763125
BL7984 (ABCB9)	5/41	0,058454216	0,84664332
BL2149 (TPR)	5/41	0,058454216	0,839870174
BL2760 (MED17)	14/171	0,059404968	0,846756533
BL1476 (PNKP)	16/203	0,061119926	0,864341631
BL9487 (EYA3)	11/126	0,061184247	0,858491471
BL9548 (TNFAIP3)	3/18	0,062669827	0,872519456
BL5361 (SSH1)	9/97	0,062791752	0,867492207
BL2294 (EDC4)	16/204	0,063303478	0,867885853
BL1856 (RBM14)	12/142	0,063641743	0,865913408
BL8568 (MAP3K2)	10/112	0,063816827	0,86176708
BL1707 (PHB2)	22/301	0,064013101	0,857966641
BL7558 (UPF1)	18/237	0,065737428	0,874551262
BL3628 (KDM3A)	18/237	0,065737428	0,868120738
BL2366 (KIAA0528)	18/237	0,065737428	0,86178409
BL640 (TCERG1)	10/113	0,066941036	0,871203624
BL7502 (VPRBP)	26/369	0,067325909	0,869908869
BL2764 (HNRNPU)	17/222	0,067961201	0,871845118
SC-28595 (POU4F1)	7/70	0,068652616	0,874468776
BL994 (MORC2)	7/70	0,068652616	0,868310546
BL9277 (PIK3R4)	16/207	0,070169576	0,881290624
BL2848 (MED28)	17/223	0,070213746	0,875721449
BL7432 (ZNF326)	22/305	0,071553267	0,886273569
BL4048 (USP32)	8/85	0,071729411	0,882370015
BL2839 (UIMC1)	21/289	0,072455875	0,885243204
BL825 (HLTF)	13/161	0,073512533	0,892084516
SC-28236 (BAP1)	17/225	0,074867816	0,902433546
BL9641 (TDRD3)	8/86	0,075698728	0,906366109
BL3114 (PPP4R1)	25/357	0,075718452	0,900598271
CBC2031 (ZMYND8)	25/357	0,075718452	0,894673282
BL2448 (SMARCA4)	9/101	0,076834299	0,901924195
BL2790 (PTEN)	30/442	0,076915815	0,897018204
BL4927 (TRIM37)	14/178	0,077133301	0,89375102
BL1308 (WRN)	16/210	0,07751568	0,892424113
SC-100753 (GMEB1)	6/58	0,07753903	0,887006987
BL2695 (PRMT1)	6/58	0,07753903	0,881393019

SC-6243 (TP53)	22/308	0,07759182	0,876445963
BL1743 (CDK7)	26/375	0,078025332	0,875834357
BL4165 (DTL)	22/309	0,079678748	0,888838708
BL4957 (ZNHIT3)	20/276	0,079785633	0,884537021
BL1018 (INTS5)	8/87	0,079797566	0,879241892
BL7507 (SOS1)	16/211	0,080072339	0,876889759
BL5655 (ANKS3)	10/117	0,080400765	0,875150147
BL5138 (GSK3B)	9/102	0,080621837	0,87227
BL2296 (EDC4)	18/244	0,081563329	0,877172091
BL7535 (NOTCH1)	18/244	0,081563329	0,871950828
BL2400 (DDB2)	23/327	0,082523471	0,876994997
N2 (ESCO2)	23/327	0,082523471	0,871836203
EPI-1615-1 (CHUK)	6/59	0,082748589	0,869102145
BL5503 (HDAC6)	6/59	0,082748589	0,864049225
BL2683 (PHLPP2)	15/196	0,082885937	0,860480595
EPI-2424-1 (SUFU)	21/294	0,083107896	0,857826329
BL285 (RFC5)	25/361	0,083497926	0,856927288
BL4874 (FBXO11)	25/361	0,083497926	0,852058383
BL2194 (USP4)	25/361	0,083497926	0,847244494
BL6510 (FBXO38)	8/88	0,084025832	0,847811201
BL3752 (CBX8)	20/279	0,086682611	0,869731672
SC-10784 (POLD1)	7/74	0,086747796	0,865550226
BL3924 (PPP3CA)	5/46	0,086991823	0,863189585
a-GAS2 (GAS2)	5/46	0,086991823	0,858446785
a-ABIN1 (TNIP1)	29/431	0,087777887	0,861470414
a-HCV5 ()	6/60	0,088147657	0,860397782
BL4907 (SUB1)	4/33	0,088215109	0,856401819
BL6410 (BCOR)	13/166	0,088407189	0,853652214
BL8288 (TOR1AIP1)	13/166	0,088407189	0,849087229
BL2356 (MLF1)	13/166	0,088407189	0,844570807
CBC1842 (DEAF1)	11/135	0,089779507	0,853142827
BL6699 (EIF3H)	17/231	0,090038745	0,851103084
BL9484 (EYA2)	10/120	0,091513274	0,86051225
BL8325 (CCDC86)	8/90	0,092869453	0,868716342
BL938 (SP1)	31/468	0,092955793	0,865018677
BL5834 (KIAA1524)	5/47	0,093435321	0,864999158
BL3250 (DDX31)	5/47	0,093435321	0,860563265
BL6840 (AGFG1)	5/47	0,093435321	0,856172636
BL8736 (PRCC)	16/216	0,093674263	0,854004952
BL173 (MDM2)	6/61	0,093734489	0,85023809
BL1326 (FOXO3)	26/383	0,094013037	0,848479467
CBC193 (ERCC6L)	14/184	0,094802431	0,851325831
BL269 (MCM8)	14/184	0,094802431	0,847090379
BL3106 (APC)	22/316	0,095340028	0,847676687
BL553 (TRIM28)	10/121	0,095411477	0,844133067
BL1035 (KDM1)	23/333	0,095754505	0,843015156
BL2000 (RIF1)	18/250	0,09697021	0,849553646
BL2222 (COL4A3BP)	8/91	0,097483801	0,849907314
BL2273 (TCF12)	22/317	0,097729153	0,847930236
CBC681 (GIT1)	13/169	0,09818625	0,847800505
BL2295 (EDC4)	13/169	0,09818625	0,843744044
BL1689 (MAD1L1)	13/169	0,09818625	0,839726215
BL2310 (MLLT3)	26/385	0,098323636	0,836915877

BL3800 (PALB2)	38/593	0,099390103	0,842002948
BL1873 (NCOA6)	10/122	0,099406414	0,838187414
BL172 (MDM2)	6/62	0,099506996	0,83511479
BL9121 (CDC7)	5/48	0,10011571	0,836315422
BL2605 (PPARGC1B)	5/48	0,10011571	0,832443591
BL323 (MSH2)	9/107	0,101222052	0,83776408
SATB1 mouse (SATB1)	33/507	0,101480584	0,836051046
BL7111 (SATB2)	20/285	0,10161751	0,833356386
BL167 (TP53)	8/92	0,102225566	0,834532349
BL7683 (RNASEN)	17/236	0,104084732	0,845865058
BL4901 (CTDP1)	4/35	0,104307076	0,84385364
BL2986 (RBBP4)	26/388	0,105027314	0,845870205
BL6881 (TSGA14)	13/171	0,105057289	0,842334335
BL9499 (EYA4)	12/155	0,105176893	0,839545332
AB81 (BRCA1)	30/457	0,105451483	0,83801267
BL5687 (ANKRD57)	16/220	0,105544866	0,835059819
SC-126 (TP53)	31/475	0,106895084	0,842033207
CBC621 (ZNF687)	20/287	0,106936334	0,838679722
BL6185 (USP1)	7/78	0,107292669	0,837815795
BL3923 (PPP4C)	10/124	0,107685177	0,837240594
BL2265 (DOCK9)	10/124	0,107685177	0,833631798
a-HAUSP (USP7)	23/338	0,107776654	0,830759103
BL9613 (IMPACT)	13/172	0,108598055	0,833513277
BL5689 (ANKRD57)	11/140	0,108672803	0,830537681
BL6311 (ELL)	15/205	0,110084708	0,837763289
a-PDS1 ()	9/109	0,110231692	0,835342275
a-PARC (CUL9)	9/109	0,110231692	0,831832434
BL9419 (KIAA1804)	9/109	0,110231692	0,828351963
BL4808 (TRRAP)	18/255	0,111128309	0,831610176
BL2850 (MED28)	14/189	0,111295558	0,829405904
BL2371 (SYK)	10/125	0,111968123	0,830970034
BL1024 (INTS8)	8/94	0,112087573	0,828433259
BL9460 (ARNTL)	12/157	0,112724954	0,829729582
BL5036 (CHD8)	12/157	0,112724954	0,826342931
BL308 (SMC1A)	12/157	0,112724954	0,822983813
BL333 (AURKA)	21/306	0,11273785	0,819745664
BL4726 (ATXN1)	7/79	0,112802756	0,816910282
BL1795 (BCL11A)	7/79	0,112802756	0,813629518
BL8710 (SASH1)	4/36	0,112819132	0,810492648
BL2769 (PRMT2)	17/239	0,113128264	0,80947555
BL8691 (ARHGEF5)	5/50	0,114167849	0,813672447
BL8779 (SEPT9)	12/158	0,116613892	0,827820353
BL508 (BRE)	15/207	0,116780938	0,825742383
BL4273 (TIPIN)	11/142	0,116828228	0,822837244
BL1872 (NCOA6)	8/95	0,117205543	0,822270134
a-HCV4 ()	6/65	0,117912758	0,824012892
BL1571 (ITCH)	7/80	0,118458914	0,824620965
SC-48849 (BRE)	17/241	0,119413365	0,828055613
BL5038 (CHD8)	12/159	0,120578963	0,832922376
EPI-2461-1 (RPA2)	10/127	0,120818282	0,831377907
BL1152 (AXL)	10/127	0,120818282	0,828204709
BL9178 (GNB2L1)	5/51	0,12152875	0,829907359
BL8428 (SCFD1)	8/96	0,122446389	0,833006495

BL2590 (KAT2B)	7/81	0,124259039	0,842148052
BL1753 (MED14)	6/66	0,124400603	0,839937907
BL1825 (TAOK3)	9/112	0,124554191	0,837825196
CBC21 (MLST8)	9/112	0,124554191	0,834698982
BL1760 (CTBP1)	11/144	0,125320565	0,83671277
BL7002 (ZMYM4)	14/193	0,125642471	0,835755102
BL2287 (ZC3H13)	13/177	0,127345859	0,843960013
BL2995 (CRTC2)	24/363	0,128316984	0,847269494
BL8524 (CLASP2)	11/145	0,129691632	0,85320942
BL2737 (ZNF335)	7/82	0,130200832	0,853433192
BL7968 (ZAK)	7/82	0,130200832	0,850329798
BL2849 (MED28)	7/82	0,130200832	0,847248893
BL4073 (ILKAP)	7/82	0,130200832	0,844190233
BL6112 (KDM6A)	6/67	0,13105894	0,846697324
BL9240 (GREB1)	13/178	0,131302001	0,84522722
BL322 (MLH1)	22/330	0,132271245	0,848425555
BL2780 (SUV39H1)	14/195	0,133195127	0,851311201
CBC2232 (AKAP8)	8/98	0,133290542	0,848900047
BL2785 (PSMC5)	8/98	0,133290542	0,8459004
SC-8051 (JUNB)	10/130	0,134793004	0,852423365
BL326 (RFX5)	10/130	0,134793004	0,849432406
BL5990 (ZNF592)	28/435	0,135107463	0,848437077
BL443 (EP300)	13/179	0,135326052	0,846848745
BL442 (EP300)	7/83	0,136281804	0,849868474
BL7711 (EPS8)	7/83	0,136281804	0,846927753
BL1026 (INTS7)	7/83	0,136281804	0,844007312
BL2051 (ESR1)	5/53	0,136889868	0,844859804
a-HCV6 ()	5/53	0,136889868	0,841966448
BL5697 (ARAP1)	5/53	0,136889868	0,839092842
BL1213 (AURKC)	12/163	0,137191322	0,838080321
BL1569 (TRIM21)	12/163	0,137191322	0,835239371
a-BRCC36 (BRCC3)	28/436	0,137679188	0,835377775
SC-8396 (CCND1)	33/524	0,138048553	0,834798657
SC-8002 (ESR1)	22/332	0,138157341	0,83265297
BL6998 (ZKSCAN1)	20/298	0,139230876	0,836316569
BL988 (PDS5A)	13/180	0,139417514	0,834646187
EPI-1614-1 (HDAC4)	13/180	0,139417514	0,831873276
BL1705 (PHB2)	22/333	0,141156899	0,839462885
BL3196 (SIRT2)	17/248	0,143007195	0,847659809
BL2745 (C1D)	11/148	0,143295887	0,846577014
CBC182 (BICD2)	11/148	0,143295887	0,843801352
BL2431 (NCOA5)	13/181	0,143575846	0,84268699
BL580 (MDC1)	25/386	0,144050146	0,842716817
EPI-2367-1 (AMPH)	8/100	0,144606297	0,843223733
BL7408 (ZC3H3)	6/69	0,144871287	0,842035054
BL6090 (PHC3)	15/215	0,145900308	0,845280496
BL6379 (BCAR1)	12/165	0,14594073	0,842795982
BL2436 (MED1)	11/149	0,147991569	0,851900186
NB120-3333 (PSME3)	28/440	0,148256104	0,850696367
BL989 (PDS5A)	7/85	0,148850429	0,851386529
BL6238 (TRERF1)	19/284	0,149536979	0,852598142
BL2459 (DDX17)	19/284	0,149536979	0,849900046
BL5902 (MARVELD2)	4/40	0,149700503	0,848145435

BL5312 (HMGN1)	8/101	0,150436148	0,849633086
CBC1841 (DEAF1)	8/101	0,150436148	0,846969659
BL3131 (PPP2CB)	24/371	0,151100154	0,848049617
BL475 (AHCTF1)	21/319	0,151365126	0,846890235
BL1075 (AURKB)	6/70	0,15201679	0,847894888
BL4677 (DBF4)	6/70	0,15201679	0,845269827
BL324 (PMS2)	20/302	0,152233114	0,843860101
BL1914 (RBBP8)	11/150	0,152766182	0,844209423
CBC802 ()	15/217	0,153751128	0,847046091
BL3121 (SMEK1)	21/320	0,154612692	0,84918775
BL4464 (CEP170)	10/134	0,154691609	0,847030881
BL2217 (HPS3)	12/167	0,15497824	0,846021031
BL6370 (KAT2B)	7/86	0,155332227	0,845383876
BL1736 (CARM1)	7/86	0,155332227	0,842829846
CBC2152 (RFWD3)	27/425	0,155380113	0,840550249
SC-163 (CDK2)	11/151	0,157618711	0,850099713
BL320 (BRCA2)	21/321	0,157898806	0,849060647
CS-2947B (CDKN1A)	6/71	0,159315798	0,854122907
MBNL1 (MBNL1)	6/71	0,159315798	0,851580874
BL2079 (RECQL5)	4/41	0,159564969	0,850381854
BL-PDS5B (PDS5B)	12/168	0,159603014	0,848068085
BL4505 (SMARCAD1)	12/168	0,159603014	0,845566409
BL5889 (ENAH)	13/185	0,160865501	0,849748353
BL2178 (RICTOR)	13/185	0,160865501	0,847256422
BL6116 (CENPT)	5/56	0,161427642	0,847731127
BL7163 (DNAJC9)	9/119	0,161585729	0,846087375
BL8561 (CAMSAP1L1)	9/119	0,161585729	0,843627818
EPI-2593-1 (RUNX1)	9/119	0,161585729	0,84118252
BL4607 (SAFB2)	16/236	0,161727209	0,839485742
BL4761 (DLG1)	7/87	0,161941511	0,838175658
BL9498 (EYA4)	8/103	0,162428941	0,838282694
BL4168 (DTL)	19/288	0,163485197	0,841316373
BL9238 (GREB1)	12/169	0,164297289	0,843079805
BL1731 (SPEN)	10/136	0,165162226	0,845103584
BL8149 (SIPA1)	10/136	0,165162226	0,842702721
BL4031 (BCAS2)	14/203	0,16585807	0,843855791
SC-28195 (KLF9)	20/306	0,165887569	0,841621677
BL4938 (TRIM56)	15/220	0,165941451	0,839523508
BL3007 (RMI1)	30/482	0,166540305	0,840186485
CS-2123B (SRC)	6/72	0,166763522	0,838955981
BL2731 (SIN3A)	19/289	0,167078263	0,83819151
CBC421 (C14orf43)	19/289	0,167078263	0,835856715
EPI-2341-1 (CDK4)	9/120	0,167267281	0,834477877
BL117 (ATR)	28/447	0,167866966	0,835149781
BL5763 (IRS2)	23/359	0,168151356	0,83425369
BL328 (RFXANK)	8/104	0,168587188	0,834111818
CBC2182 (NFRKB)	7/88	0,168674966	0,832253403
BL3283 (C7orf27)	17/255	0,169017646	0,831659429
BL2581 (RPTOR)	12/170	0,169060124	0,829595581
BL4152 (USP37)	4/42	0,169660024	0,830270851
BL1461 (MAD2L1)	31/501	0,169902106	0,82919615
BL6912 (CEP152)	5/57	0,169976421	0,82731071
BL5295 (ARID5B)	10/137	0,170523104	0,827728366

BL2418 (UBAP2L)	10/137	0,170523104	0,825497293
BL5506 (UACA)	19/290	0,170713061	0,824195316
EPI-1550-1 (SRC)	11/154	0,172632767	0,831229087
BL6260 (MBD4)	11/154	0,172632767	0,829006549
BL3936 (PPP5C)	16/239	0,173753371	0,832162811
SC-6954 (BRCA1)	12/171	0,173890527	0,830604752
a-TRF2 (4A794) (TERF2)	6/73	0,174354982	0,830614185
BL6392 (PRKAR2A)	13/188	0,174501933	0,829115004
BL7766 (MAPK3)	8/105	0,174850023	0,828576888
BL4773 (MTOR)	10/138	0,175965695	0,831669444
BL5966 (RANBP3)	20/309	0,176545677	0,832220569
BL1460 (MAD2L1)	22/344	0,176584174	0,830222975
CBC2231 (AKAP8)	11/155	0,177785647	0,833689352
CBC32 (POLDIP3)	19/292	0,178106365	0,833018311
BL2800 (CCNA1)	14/206	0,179078125	0,835387824
BL7869 (RAPGEF2)	14/206	0,179078125	0,833223607
BL5231 (TAF2)	4/43	0,179971645	0,835217248
BL8602 (MAST2)	10/139	0,181488415	0,840085549
ab92734 (PJA2)	21/328	0,181958816	0,840097774
EPI-1746-1 (VAV1)	6/74	0,182085029	0,838524903
BL2266 (TDP1)	7/90	0,182500436	0,838288447
BL1340 (DCLRE1C)	7/90	0,182500436	0,836149956
BL2458 (UBE2L3)	24/381	0,182592417	0,834442701
BL2989 (RBBP7)	15/224	0,182944461	0,833929573
BL5166 (CDKN2A)	12/173	0,183749851	0,835480336
BL4553 (TRIM33)	13/190	0,183899341	0,834048525
BL235 (CHEK1)	37/615	0,187072329	0,846302024
BL2445 (SAFB)	15/225	0,187324757	0,845314733
BL2466 (NCOA7)	20/312	0,187551396	0,84421631
BL1789 (PAXIP1)	20/312	0,187551396	0,84210577
BL1327 (FOXO3)	13/191	0,188687249	0,845093014
BL2667 (HDAC2)	17/260	0,189004574	0,844408495
BL5786 (WRAP53)	6/75	0,189948359	0,846519236
CBC411 (AHCTF1)	16/243	0,1904732	0,846757099
BL6413 (BCOR)	15/226	0,19175547	0,850352651
SC-583 (CREBBP)	10/141	0,192767592	0,852735454
CBC651 (AGAP3)	10/141	0,192767592	0,850640281
BL6582 (NUMB)	14/209	0,192798654	0,848692115
BL6598 (FBXW7)	19/296	0,193376435	0,849154222
BL1755 (MED23)	13/192	0,193533326	0,847770376
BL964 (ROCK2)	8/108	0,194239408	0,84879313
CBC184 (BICD2)	16/244	0,194771469	0,849052327
BL8105 (AHDC1)	23/367	0,195187518	0,848805767
SC-12738 (CSNK2A1)	7/92	0,196779476	0,85366169
BL5105 (BRF1)	6/76	0,197939526	0,856625031
BL8292 (NOTCH2)	6/76	0,197939526	0,854565836
SC-8005 (ESR1)	12/176	0,199018277	0,857162649
BL1145 (LMO1)	11/159	0,199103203	0,855476921
BL2195 (USP4)	16/245	0,199115756	0,853489017
BL7222 (GCN1L1)	3/30	0,200028832	0,855361386
BL4952 (HMGN3)	21/333	0,200236536	0,854215722
BL700 (DIDO1)	15/228	0,200765129	0,854441167
BL4336 (TRIP12)	8/109	0,200893485	0,852966191

BL2735 (SRA1)	4/45	0,201187717	0,852200801
BL3721 (KDM4B)	4/45	0,201187717	0,850195623
BL5502 (HDAC6)	19/298	0,201246099	0,848445995
BL2730 (SIN3A)	17/263	0,201529929	0,847652815
BL1757 (MED23)	9/126	0,203229051	0,852802281
BL2041 (XRN2)	9/126	0,203229051	0,850814396
BL8641 (MINK1)	13/194	0,203395882	0,849532569
SC-954 (CDC2)	13/194	0,203395882	0,847561496
BL653 (CUL7)	7/93	0,20407947	0,848441502
BL3415 (RAD52)	7/93	0,20407947	0,846482053
BL2791 (PTEN)	27/441	0,204393207	0,845829951
BL6535 (TRAF3IP1)	11/160	0,204601982	0,844747494
BL5345 (PTPN12)	11/160	0,204601982	0,84281
BL4869 (PCM1)	11/160	0,204601982	0,840881373
BL2463 (TGS1)	15/229	0,205342505	0,841998032
BL2840 (UIMC1)	52/896	0,205798569	0,841945854
a-POT1 (POT1)	5/61	0,205806685	0,840065468
BL2437 (PUS1)	5/61	0,205806685	0,838160558
BL4941 (TRIM3)	14/212	0,206997039	0,841101089
BL2457 (UBE2L3)	14/212	0,206997039	0,839202441
BL1732 (SPEN)	14/212	0,206997039	0,837312346
BL459 (TRIP4)	14/212	0,206997039	0,835430745
BL5054 (POLE3)	9/127	0,209514619	0,84369564
BL4386 (MAGED2)	17/265	0,210092503	0,844130058
BL6780 (NIPBL)	20/318	0,210562902	0,844131634
BL1115 (CENPJ)	7/94	0,211481118	0,845924472
BL1109 (CENPF)	14/213	0,211831767	0,845444117
BL4731 (ATXN2)	4/46	0,212063518	0,844492412
BL8680 (MKL1)	4/46	0,212063518	0,842624066
BL2766 (PRMT2)	4/46	0,212063518	0,840763969
BL5418 (DLG5)	22/354	0,212463891	0,84049592
BL3695 (DLGAP5)	13/196	0,213478458	0,842653429
BL1027 (INTS9)	13/196	0,213478458	0,840805505
BL9197 (BRCC3)	3/31	0,213592618	0,839414316
BL9124 (ELL2)	3/31	0,213592618	0,837581533
BL2606 (PPARGC1B)	3/31	0,213592618	0,835756737
BL4982 (RBM5)	3/31	0,213592618	0,833939874
EPI-1490-1 (CDC25C)	6/78	0,214282986	0,834820483
CBC2132 (ZNF295)	6/78	0,214282986	0,833013513
BL170 (TP53)	6/78	0,214282986	0,831214347
BL990 (INCENP)	17/266	0,214435561	0,830013507
EPI-2418-1 (KIF22)	8/111	0,214470356	0,828362922
BL5700 (MIB2)	8/111	0,214470356	0,826585319
BL552 (H2AFX)	5/62	0,21513122	0,827356897
BL1657 (SMG1)	18/284	0,216089594	0,829266904
BL130 (MSH6)	23/373	0,216807082	0,830246308
BL533 (CCAR1)	18/285	0,220345536	0,842001238
BL5558 (TOM1)	11/163	0,22148041	0,84454101
BL677 (LEO1)	11/163	0,22148041	0,842751728
BL6583 (NUMB)	11/163	0,22148041	0,840970012
BL4905 (C19orf2)	10/146	0,222245342	0,842094166
BL4547 (HMG20B)	10/146	0,222245342	0,840321336
EPI-1571-1 (NEDD8)	9/129	0,222315043	0,838818942

BL1804 (MTA1)	6/79	0,222623827	0,838223048
a-REST (TW) (REST)	13/198	0,223771993	0,840783473
BL1690 (MAD1L1)	23/375	0,224254496	0,840837316
BL4974 (USP20)	5/63	0,224585398	0,840323699
BL5993 (ZNF638)	5/63	0,224585398	0,838576664
BL7534 (NOTCH1)	5/63	0,224585398	0,836836878
PHF8 (PHF8)	12/181	0,225661817	0,83910688
BL2593 (RPL7A)	16/251	0,22610755	0,839027189
BL8224 (ASXL2)	3/32	0,227344696	0,841878502
BL5118 (PAK1)	25/412	0,228231088	0,843421881
BL4651 (KIF22)	8/113	0,228384854	0,842257079
BL5415 (DHX57)	8/113	0,228384854	0,840531142
BL8731 (MLLT1)	9/130	0,228824946	0,840428637
BL7270 (NRBF2)	9/130	0,228824946	0,838713477
BL1823 (TAOK3)	16/252	0,230753373	0,84405918
BL6322 (SFN)	12/182	0,231157913	0,843820347
BL2533 (OBFC1)	21/341	0,231240545	0,842409773
BL5030 (CHD6)	14/217	0,231654002	0,842207669
BL3118 (PPP4R2)	26/431	0,231714895	0,840727175
BL5778 (TNKS1BP1)	17/270	0,232203499	0,84080138
BL439 (NCOA3)	11/165	0,233034774	0,842113591
CBC41 (NAT10)	15/235	0,233769071	0,843070787
BL8882 (SIRT6)	5/64	0,234160515	0,84279015
BL8579 (MAP3K7IP1)	7/97	0,234254533	0,841442282
BL5271 (TOPORS)	7/97	0,234254533	0,839762757
BL5046 (POLA2)	4/48	0,234278972	0,838177359
BL8546 (DYRK1B)	4/48	0,234278972	0,836511003
GTX105364 (BRE)	10/148	0,234507975	0,835667308
BL2270 (TCF12)	10/148	0,234507975	0,834012521
BL3942 (P4HA2)	10/148	0,234507975	0,832364275
BL2846 (MED27)	9/131	0,235404591	0,833898711
BL1214 (AURKC)	9/131	0,235404591	0,832257178
BL1309 (WRN)	9/131	0,235404591	0,830622095
BL3822 (JMJD1C)	30/505	0,23695018	0,83443632
CBC2032 (ZMYND8)	19/307	0,238468817	0,838140892
BL1686 (BUB3)	15/236	0,23865879	0,837170288
BL8231 (TOP3A)	13/201	0,239586875	0,838787578
BL1859 (TRIP6)	6/81	0,239614479	0,837252149
NB106A (BIN1)	6/81	0,239614479	0,835626416
BL8633 (MASTL)	7/98	0,242020994	0,842383149
BL275 (RFC2)	9/132	0,24205138	0,840859338
SC-28748 (TGFB1I1)	9/132	0,24205138	0,839236058
BL4857 (CIC)	9/132	0,24205138	0,837619033
BL892 (BLM)	9/132	0,24205138	0,836008227
BL8103 (SHC1)	8/115	0,242611306	0,836333793
BL2290 (BTBD12)	25/416	0,242885788	0,835676005
BL2451 (TGFB1I1)	20/326	0,243170895	0,835057222
SC-8023 (IFI16)	20/326	0,243170895	0,833463601
BL1921 (KIAA1967)	24/398	0,243178407	0,831901751
BL973 (PNKP)	5/65	0,243847828	0,832605892
BL976 (PLK1)	5/65	0,243847828	0,831025995
SC-50 (RB1)	11/167	0,244814867	0,832741479
BL8106 (AHDC1)	28/471	0,245011346	0,831834363

BL1761 (CTBP1)	17/273	0,245924435	0,83335903
BL6096 (MOV10)	26/435	0,246148979	0,83254909
BL2291 (BTBD12)	26/435	0,246148979	0,830984148
BL3907 (USP28)	18/291	0,246638915	0,831075968
BL4488 (KDM5C)	14/220	0,246998585	0,830729323
SC-102125 (TAF10)	14/220	0,246998585	0,829176558
a-AKT (BS) (AKT1)	10/150	0,247015462	0,827686138
BL1096 (DYRK2)	20/327	0,247389816	0,827396854
BL2229 (FIGNL1)	12/185	0,247955113	0,827746064
BL1936 (SMG5)	15/238	0,248559881	0,828225502
BL1049 (C11orf30)	9/133	0,248762671	0,827366217
BL6524 (BRAF)	8/116	0,249833257	0,829390999
a-TSPYL1 (TSPYL1)	8/116	0,249833257	0,827860757
BL6346 (ATF2)	8/116	0,249833257	0,826336152
BL5259 (TAF6)	7/99	0,249867886	0,824931476
BL4625 (RBMX2)	7/99	0,249867886	0,82341784
BL1100 (HIPK2)	13/203	0,250365165	0,823545486
BL2776 (NF2)	13/203	0,250365165	0,822039919
BL2467 (SEC16A)	11/168	0,250785124	0,821916209
BL436 (NCOA2)	26/437	0,253507907	0,829326415
BL9701 (CAMK1D)	5/66	0,253638584	0,828245268
BL6624 (FOXC2)	5/66	0,253638584	0,826742101
BL3927 (PPP3CA)	3/34	0,255304963	0,830666146
BL2498 (DOT1L)	21/347	0,255791151	0,83074305
BL1868 (TRIP4)	19/311	0,255887155	0,829554747
BL237 (RAD17)	20/329	0,255916588	0,828155301
BL1232 (BRD8)	6/83	0,25697738	0,830092399
BL2045 (XRN1)	4/50	0,257018171	0,828733635
CBC592 (KLHL13)	8/117	0,257123198	0,827586493
BL2416 (RC3H1)	7/100	0,257790801	0,828250945
BL6476 (EEF2K)	7/100	0,257790801	0,826771926
BL1798 (BCL11B)	18/294	0,260241648	0,833144386
BL6339 (ANKRD17)	11/170	0,262876927	0,840083562
BL2596 (PIAS2)	5/67	0,263524039	0,840655727
BL7235 (MAP3K1)	5/67	0,263524039	0,839165203
BL4914 (TRIM16)	21/349	0,264199517	0,839827136
BL4224 (BPTF)	8/118	0,264477711	0,839226093
a-ATM (Ab-3) (ATM)	8/118	0,264477711	0,837745976
BL3030 (ERBB2)	8/118	0,264477711	0,836271072
SC-965 (MDM2)	6/84	0,265783387	0,838922606
CBC572 (ESPL1)	7/101	0,265785296	0,837456827
BL2238 (KIAA1429)	7/101	0,265785296	0,835990178
BL2843 (MED12)	10/153	0,266197926	0,835824258
BL6632 (FOXK1)	10/153	0,266197926	0,834365577
BL1826 (TAOK3)	20/332	0,268919834	0,84142861
BL2226 (MAPK14)	11/171	0,268994742	0,840199229
EPI-1081-1 (AKT1)	3/35	0,269461014	0,840194413
BL2313 (MLLT3)	3/35	0,269461014	0,83873827
BL587 (FEN1)	17/278	0,26948234	0,837353431
BL2555 (UHRF1)	17/278	0,26948234	0,835907224
BL6174 (TDP1)	12/189	0,271015108	0,839212299
BL2293 (EDC4)	12/189	0,271015108	0,837767871
CBC2201 (RXRB)	8/119	0,271893349	0,839038581

BL7338 (LRWD1)	10/154	0,272695506	0,840070548
BL8716 (SEC31A)	5/68	0,27349548	0,841092264
BL1015 (INTS6)	7/102	0,273846901	0,840733392
BL2841 (MED12)	7/102	0,273846901	0,839298694
CBC22 (MLST8)	7/102	0,273846901	0,837868883
SC-7216 ()	7/102	0,273846901	0,836443936
SC-50347 (PIAS4)	18/297	0,274122011	0,835862702
BL5987 (ZNF318)	16/261	0,274255182	0,834851367
EPI-2488-1 (TRIB3)	6/85	0,27466437	0,834682249
EPI-1474-1 (RPS6KA3)	6/85	0,27466437	0,833272313
BL531 (MCM10)	27/461	0,274934956	0,832686647
BL9430 (C7orf20)	11/172	0,275157972	0,831959121
BL7656 (C2orf3)	11/172	0,275157972	0,83056087
BL2759 (MED17)	9/137	0,276198815	0,832303811
SC-232 (POU2F1)	9/137	0,276198815	0,830909668
BL5899 (NIN)	9/137	0,276198815	0,829520187
BL5049 (POLD3)	14/226	0,278784074	0,835886807
BL9456 (CRY2)	10/155	0,279241014	0,835861434
BL325 (NCOA2)	10/155	0,279241014	0,83447065
BL7902 (POLK)	10/155	0,279241014	0,833084486
BL6498 (EVI1)	8/120	0,279366639	0,832077087
BL6388 (CREB1)	4/52	0,280169541	0,833086912
BL540 (SETDB1)	4/52	0,280169541	0,831709909
BL2762 (HNRNPU)	11/173	0,281364696	0,833879527
BL4146 (USP36)	7/103	0,281971127	0,834300072
BL5157 (SGOL2)	7/103	0,281971127	0,832927868
BL1164 (SNIP1)	12/191	0,282800985	0,834007502
BL4783 (AKT1S1)	12/191	0,282800985	0,832640276
BL2196 (USP4)	9/138	0,283191823	0,832426373
BL674 (PAF1)	5/69	0,283544244	0,83210043
ABM-2052 (6H2.1) (PTEN)	5/69	0,283544244	0,830743007
BL755 (CSNK2A2)	5/69	0,283544244	0,829390005
BL7008 (ZNF768)	5/69	0,283544244	0,828041403
BL2761 (MED17)	5/69	0,283544244	0,82669718
BL2257 (PRKRIR)	6/86	0,283614281	0,82556118
TRAF4 (TRAF4)	6/86	0,283614281	0,82422532
BL5491 (RAD23B)	6/86	0,283614281	0,822893777
BL702 (TBK1)	3/36	0,283701185	0,821818271
BL2796 (CKLF)	3/36	0,283701185	0,820494893
BL8110 (RAD54L2)	33/574	0,284213533	0,820655154
BL5670 (KANK2)	21/354	0,28566867	0,823532795
CBC561 (BAT3)	10/156	0,285832144	0,822683542
BL6599 (FBXW7)	8/121	0,286894088	0,824418852
JQ008 (IK)	18/300	0,288259014	0,827017873
CBC194 (ERCC6L)	12/192	0,288752161	0,827111455
BL5913 (NUCKS1)	13/210	0,289401206	0,827650581
BL999 (FANCI)	7/104	0,290153472	0,828482728
BL3057 (TIPRL)	7/104	0,290153472	0,827167676
BL462 (RBM39)	9/139	0,290232827	0,826082659
BL2218 (HPS3)	9/139	0,290232827	0,824775566
AB80 (BRCA1)	35/613	0,290938591	0,825475054
BL458 (TRIP4)	10/157	0,292466575	0,828501528
BL900 (IFFO1)	6/87	0,292627078	0,827650758

BL9016 (SP3)	5/70	0,29366174	0,829271203
SC-582 (MLH1)	5/70	0,29366174	0,827969365
BL2157 (USP34)	17/283	0,293811159	0,827092228
BL2738 (ZNF335)	11/175	0,29390085	0,826049964
BL4670 (PPP2R5D)	11/175	0,29390085	0,824759261
EPI-1625-1 (ITK)	8/122	0,294472189	0,825073403
BL4961 (ABI1)	14/229	0,295159786	0,825711801
BL1242 (MAML2)	18/302	0,297815524	0,831845537
SC-9966 (MCM7)	3/37	0,29800186	0,83107351
EPI-2120-1 ()	3/37	0,29800186	0,829785024
BL7062 (CHUK)	7/105	0,298389432	0,82957805
BL1691 (MAD1L1)	21/357	0,298833201	0,829527712
BL2588 (NCOR2)	12/194	0,30076262	0,833595163
BL1023 (INTS8)	6/88	0,301696743	0,834895765
a-HCV10 ()	6/88	0,301696743	0,83361131
BL6436 (CCNL2)	17/285	0,303733756	0,837950578
BL2298 (DDX19B)	5/71	0,303839462	0,836956555
BL2230 (FIGNL1)	9/141	0,304447506	0,837347198
BL1739 (CARM1)	36/636	0,305743997	0,839627246
BL6415 (BCR)	10/159	0,305855981	0,838652432
BL664 (RFC3)	7/106	0,306674504	0,839614954
BL5668 (KANK2)	7/106	0,306674504	0,838337001
BL5529 (CCDC99)	24/414	0,306945455	0,837802489
BL6657 (GRLF1)	20/341	0,309299984	0,842948059
BL1011 (ZHX1)	8/124	0,309766268	0,842939722
BL958 (ATAD5)	16/268	0,309895215	0,842014835
BL2127 (HDAC3)	22/378	0,310456361	0,842265293
BL1867 (TRIP4)	6/89	0,310817292	0,841972635
BL2133 (SSBP3)	15/250	0,310932831	0,841017114
BL713 (NCOA6)	9/142	0,311615493	0,841596128
BL2749 (SNW1)	3/38	0,312340461	0,84228749
BL1664 ()	3/38	0,312340461	0,84102469
BL6691 (KDM5D)	3/38	0,312340461	0,839765671
BL441 (CREBBP)	10/160	0,312606247	0,839223946
BL7075 (NFKBIB)	12/196	0,312905909	0,838774645
BL2787 (PSMC5)	11/178	0,312982306	0,837729092
BL2223 (COL4A3BP)	11/178	0,312982306	0,836482471
BL3893 (RAD9A)	17/287	0,313754508	0,837300292
BL1013 (INTS3)	5/72	0,314069009	0,836896052
BL3263 (NHEJ1)	5/72	0,314069009	0,835656206
BL8688 (SET)	5/72	0,314069009	0,834420029
BL8931 (SRPK1)	7/107	0,315004196	0,835668443
BL1019 (INTS5)	16/269	0,315093104	0,834671407
BL6439 (CCNL2)	4/55	0,315434093	0,834344081
BL7174 (TNRC6A)	4/55	0,315434093	0,833117104
SC-32856 (RNF14)	4/55	0,315434093	0,831893731
BL8108 (RAD54L2)	31/546	0,316270874	0,832877552
BL2132 (RBM15)	14/233	0,317418149	0,834674956
BL3038 (ERBB4)	8/125	0,3174752	0,833604472
BL4485 (KDM5C)	9/143	0,318820109	0,835913747
SC-197 (MAX)	22/380	0,319220934	0,835744604
BL7814 (ARHGEF11)	10/161	0,319390401	0,834971122
BL2847 (MED27)	11/179	0,319408758	0,833805419

BL5855 (CDCA3)	15/252	0,321740146	0,838672427
BL2794 (TRIM24)	15/252	0,321740146	0,83745696
BL1039 (RCOR1)	18/307	0,322119646	0,837231381
BL5032 (CHD7)	7/108	0,323374032	0,839277111
BL4460 (AATF)	5/73	0,324342097	0,840574901
BL6836 (NUP188)	5/73	0,324342097	0,839363698
BL1050 (C11orf30)	19/326	0,325108803	0,840137282
BL2390 (CUL4A)	8/126	0,3252207	0,839218933
BL8252 (QSER1)	8/126	0,3252207	0,838014889
BL262 (MCM6)	8/126	0,3252207	0,836814295
BL4588 (CHAF1B)	16/271	0,325558335	0,836484649
BL2882 (CHEK2)	16/271	0,325558335	0,835289671
BL4277 (TIMELESS)	9/144	0,326058501	0,835379555
SC-67041 (PRAME)	9/144	0,326058501	0,834189556
BL1095 (DYRK2)	10/162	0,326206072	0,833379952
SC-8405 (NFATC3)	3/39	0,326695464	0,833444677
BL6804 (NUP50)	3/39	0,326695464	0,832262486
BL1545 (TTK)	18/308	0,327044422	0,831971362
BL2784 (PARK7)	15/253	0,327179288	0,8311372
SC-48847 (BRE)	14/235	0,328705561	0,833835012
BL2693 (PRMT1)	14/235	0,328705561	0,83265894
BL6494 (ESCO2)	17/290	0,328953187	0,832112569
BL8921 (RPS6KA1)	6/91	0,329187318	0,831533646
SC-6062 (RORA)	13/217	0,330106326	0,832683936
BL2777 (NF2)	16/272	0,330823562	0,833322745
BL4675 (DBF4)	12/199	0,33133987	0,83345435
BL6501 (PTK2)	11/181	0,332348577	0,83482244
BL7552 (DICER1)	11/181	0,332348577	0,833656487
BL4172 (NCRNA00153)	8/127	0,332999259	0,834123667
BL2729 (SIN3A)	10/163	0,333050886	0,833091074
CBC12 (MAP3K1)	10/163	0,333050886	0,831932394
BL5978 (ZFP106)	2/23	0,334368124	0,834062709
BL7633 (COBRA1)	14/236	0,334384186	0,832945905
SC-596 (CCNA2)	14/236	0,334384186	0,83179224
BL1356 (TAF15)	5/74	0,334650579	0,831303512
BL2728 (SIN3A)	5/74	0,334650579	0,830155303
BL5008 (RBM26)	5/74	0,334650579	0,829010261
BL8514 (CKAP2)	18/310	0,336950984	0,833559183
CBC174 (AGAP3)	12/200	0,337536012	0,833857878
BL1682 (BUB1)	15/255	0,338122615	0,834159638
BL2254 (PRKRIR)	6/92	0,338425072	0,833760535
BL2379 (RFWD3)	6/92	0,338425072	0,832618397
EPI-2603-1 (PTTG1)	6/92	0,338425072	0,831479384
BL7226 (HERC4)	4/57	0,339152763	0,832128911
a-ARP5 (ACTR5)	4/57	0,339152763	0,830993674
BL6755 (PHF8)	17/292	0,339186191	0,829943324
BL8473 (MADD)	10/164	0,339922468	0,830613269
BRD4-C (BRD4)	10/164	0,339922468	0,829484718
BL5026 (CHD3)	10/164	0,339922468	0,82835923
CBC541 (CYLD)	7/110	0,340216351	0,827951988
BL2502 (REST)	24/422	0,340677028	0,827951209
BL9273 (KIAA0226)	3/40	0,341046404	0,82772884
BL993 (MORC2)	20/348	0,341883039	0,828639594

BL2795 (TRIM24)	20/348	0,341883039	0,827522829
BL5883 (CIZ1)	20/348	0,341883039	0,82640907
BL3977 (USP10)	18/311	0,341931014	0,825414113
BL8109 (RAD54L2)	23/404	0,343274573	0,827545145
BL6534 (TRAF3IP1)	12/201	0,343755004	0,827592476
BL6486 (ERF)	12/201	0,343755004	0,826484587
BL4891 (CDC20)	19/330	0,344391186	0,82690718
BL1656 (SMG1)	5/75	0,34498645	0,827230526
CBC181 (BICD2)	11/183	0,345390856	0,82709597
BL1737 (CARM1)	14/238	0,345804582	0,826984061
SC-71539 (MAGEA1)	16/275	0,346736884	0,828110963
BL8954 (SRPK2)	6/93	0,347690285	0,829285194
BL1585 (EGFR)	9/147	0,347947823	0,828798794
BL1777 (C21orf7)	9/147	0,347947823	0,827701048
BL2094 (MSH2)	21/368	0,34860564	0,828168955
BL3173 (MAVS)	8/129	0,348641582	0,827160214
BL6350 (ATF7)	8/129	0,348641582	0,826068973
BL4189 (USP19)	7/111	0,348680023	0,825071569
BL2739 (ZNF335)	4/58	0,351038378	0,829559115
SC-11354 (DBF4)	4/58	0,351038378	0,828469024
BL8979 (PKD2)	4/58	0,351038378	0,827381795
BL894 (PAFAH1B1)	4/58	0,351038378	0,826297415
BL8578 (MAP3K7IP1)	10/166	0,353736456	0,831558476
BL1012 (ZHX1)	13/221	0,353920063	0,830902528
BL4793 (XPC)	19/332	0,354129275	0,830308326
BL2432 (NCOA5)	17/295	0,354667177	0,830485332
BL4554 (ATXN10)	9/148	0,355292848	0,830867129
BL2413 (RC3H1)	5/76	0,355341865	0,829901157
BL8308 (ZMYND8)	5/76	0,355341865	0,828823363
BL1857 (RBM14)	3/41	0,355373883	0,827822949
BL596 (APTX)	8/130	0,356498409	0,829366766
BL7041 (NFKB2)	18/314	0,356967482	0,829383697
BL465 (AHCYL2)	6/94	0,356977271	0,828334857
BL2709 (FANCD2)	7/112	0,357166224	0,827703919
BL5338 (PPP1R14B)	7/112	0,357166224	0,826637291
BL1547 (DYRK2)	7/112	0,357166224	0,825573407
BL3169 (ESPL1)	7/112	0,357166224	0,824512259
BL3434 (CRTC1)	21/370	0,357862349	0,825058767
BL2640 (TERF2)	11/185	0,358519476	0,825514076
EPI-2589-1 (RPA1)	15/259	0,360236009	0,828404445
BL5117 (ILK)	10/167	0,360674136	0,82835134
CBC361 (MALT1)	10/167	0,360674136	0,82729342
BL2398 (DDB2)	21/371	0,362509802	0,830443374
BL1550 (HIPK2)	14/241	0,363072892	0,830673776
BL2842 (MED12)	7/113	0,365670652	0,835552787
BL2583 (NCOR1)	7/113	0,365670652	0,834491094
BL3655 (PSIP1)	5/77	0,365709148	0,833519834
BL1759 (CTBP1)	5/77	0,365709148	0,832463408
BL2126 (HDAC3)	5/77	0,365709148	0,831409657
BL2381 (TAOK1)	15/260	0,365804597	0,830575292
BL7244 (MAST4)	13/223	0,365938532	0,829830307
BL1032 (ZMYM3)	6/95	0,366280435	0,829558211
BL1805 (MTA1)	10/168	0,367629135	0,831564139

SC-185 (BCL3)	10/168	0,367629135	0,830518147
BL5214 (DOCK10)	3/42	0,369659561	0,834055995
BL554 (TRIM28)	3/42	0,369659561	0,833009501
BL1045 (ABL2)	9/150	0,370038864	0,832819298
BL5021 (CHD1)	9/150	0,370038864	0,831776971
BL597 (APTX)	11/187	0,371718363	0,834507724
BL2755 (LCOR)	13/224	0,371970343	0,834030882
BL9429 (C7orf20)	8/132	0,372266236	0,833653567
CBC173 (AGAP3)	8/132	0,372266236	0,832615393
BL6548 (RAI1)	8/132	0,372266236	0,831579802
BL697 (CC2D1A)	26/467	0,37285265	0,831855106
BL2438 (PUS1)	19/336	0,373766689	0,832859768
a-BARD1 (BARD1)	23/411	0,374251504	0,832906693
BL835 (MLL4)	4/60	0,374805161	0,833106522
BL3688 (PRMT5)	4/60	0,374805161	0,832076724
BL8333 (C1orf55)	12/206	0,375131849	0,831773829
BL129 (MSH6)	20/355	0,37522219	0,830948277
BL1966 (RANBP1)	17/299	0,375512906	0,830567956
BL901 (IFFO1)	6/96	0,375594271	0,829726089
BL2586 (NCOR2)	6/96	0,375594271	0,82870677
BL6754 (PHF8)	15/262	0,376981648	0,830747288
BL2136 (ROCK1)	8/133	0,380170463	0,836747734
BL4935 (TRIM47)	8/133	0,380170463	0,835723563
BL2881 (CHEK2)	14/244	0,380474166	0,835368708
BL840 (UBE2A)	23/413	0,383196736	0,840319093
BL8235 (BAT3)	3/43	0,383886151	0,840804302
BL7864 (RAPGEF1)	3/43	0,383886151	0,83978018
BL1167 (MCPH1)	13/226	0,384071542	0,839163611
BL3342 (EVI5)	13/226	0,384071542	0,838143972
BL9173 (MALT1)	9/152	0,384840943	0,838803803
BL6869 (CEP192)	6/97	0,384913372	0,837944747
BL6687 (ASH1L)	6/97	0,384913372	0,836930286
BL3203 (SIRT1)	14/245	0,386297869	0,838924998
BL3461 (NSL1)	5/79	0,386449512	0,838240729
BL2153 (NEK7)	5/79	0,386449512	0,837229582
BL2440 (RBM9)	4/61	0,386664701	0,836686509
BL8507 (BOP1)	4/61	0,386664701	0,835679666
BL5784 (WDR44)	4/61	0,386664701	0,834675243
BL6487 (ERF)	18/320	0,387393879	0,835245386
BL4267 (PPP2R3B)	8/134	0,388083774	0,835729565
BL2155 (USP34)	8/134	0,388083774	0,834728691
CBC682 (GIT1)	15/264	0,388203861	0,8339882
BL8516 (CKAP2L)	15/264	0,388203861	0,832991798
CBC412 (AHCTF1)	13/227	0,390137909	0,836142822
BL6987 (ZCCHC8)	7/116	0,391251074	0,837529116
SC-81280 (NCOA2)	22/396	0,391445721	0,836948232
BL2225 (MAPK14)	22/396	0,391445721	0,835953049
BL977 (PLK1)	11/190	0,391613541	0,835318195
BL7201 (EPS8L2)	9/153	0,39225613	0,835696333
BL9431 (C7orf20)	6/98	0,394232438	0,838911681
BL6024 (WDR4)	6/98	0,394232438	0,837918886
BL2272 (TCF12)	20/359	0,394513595	0,837525315
BL2594 (PIAS2)	8/135	0,396002874	0,839694407

BL4184 (USP16)	8/135	0,396002874	0,838704201
BL1681 (BUB1)	8/135	0,396002874	0,837716328
BL2687 (ZBTB7A)	17/303	0,396534729	0,837854556
BL2703 (RP6-213H19.1)	5/80	0,396808168	0,837447085
BL1341 (DCLRE1C)	5/80	0,396808168	0,836464166
BL1780 (C21orf7)	14/247	0,397972172	0,837934375
BL1794 (BCL11A)	14/247	0,397972172	0,836953187
BL6425 (CCNH)	14/247	0,397972172	0,835974294
BL1536 (TREX1)	11/191	0,398263196	0,835608295
BL3388 (E2F1)	11/191	0,398263196	0,834633256
BL5398 (FAM175B)	27/492	0,398461931	0,83407649
BL2601 (MED24)	4/62	0,398494592	0,833173793
GREB1 mAb (GREB1)	4/62	0,398494592	0,832204986
BL7649 (MAP4K1)	4/62	0,398494592	0,83123843
BL5826 (ORC6L)	15/266	0,399461665	0,832289038
BL2013 (AGAP3)	6/99	0,403546277	0,839825161
BL9241 (GREB1)	8/136	0,403924514	0,839639382
BL4953 (TRIP11)	11/192	0,404918561	0,840732643
BL1034 (ADNP)	23/418	0,405694418	0,841370871
ab70171 (BAT3)	17/305	0,40709209	0,843295725
BL1106 (CENPC1)	9/155	0,407101274	0,84234319
BL2204 (STK3)	5/81	0,407149855	0,841474269
BL1774 (STK10)	5/81	0,407149855	0,840507057
BL6417 (BCR)	7/118	0,408319465	0,841953799
BL5113 (PDPK1)	2/27	0,409482246	0,843383159
BL2500 (DOT1L)	10/174	0,409592915	0,84264476
BL5722 (ZNF830)	4/63	0,410285043	0,843102903
BL2150 (TPR)	4/63	0,410285043	0,842139356
BL5783 (WDR44)	4/63	0,410285043	0,84117801
BL6305 (POLL)	4/63	0,410285043	0,840218856
BL7872 (RAPGEF6)	4/63	0,410285043	0,839261887
BL6046 (WDR62)	1/10	0,411342481	0,840467685
SC-7300 (CREBBP)	1/10	0,411342481	0,839512608
BL1104 (CENPB)	11/193	0,411577707	0,83903923
BL1426 (FOXO1)	8/137	0,411845485	0,83863321
BL2241 (NUP214)	3/45	0,412098092	0,838197251
BL7865 (RAPGEF1)	3/45	0,412098092	0,837249064
BL1756 (MED23)	6/100	0,412849816	0,837828553
BL6497 (EVI1)	6/100	0,412849816	0,836882922
BL5344 (PTPN12)	6/100	0,412849816	0,835939424
BL5431 (ERCC5)	18/325	0,412994453	0,835290583
BL5963 (RAF1)	12/212	0,413176318	0,834718411
BL4545 (HMG20B)	12/212	0,413176318	0,833780525
BL756 (CSNK2A2)	12/212	0,413176318	0,832844744
BL1869 (TRIP4)	19/344	0,41350278	0,832568377
CBC801 ()	20/363	0,41391938	0,832473915
BL1031 (ZMYM3)	13/231	0,414473575	0,832656086
BL2365 (KIAA0528)	13/231	0,414473575	0,831725744
BL2292 (BTBD12)	23/420	0,414734574	0,831320642
BL3693 (DLGAP5)	10/175	0,416604554	0,834137991
BL2128 (HDAC3)	10/175	0,416604554	0,833209107
BL2439 (RBM9)	10/175	0,416604554	0,83228229
CS-2126S ()	7/119	0,416846095	0,83183954

BL4893 (CDC20)	5/82	0,417467868	0,832155707
BL1105 (CENPC1)	12/213	0,419534832	0,835348735
SC-5278 (CHEK2)	8/138	0,419762631	0,834876728
BL2130 (RBM15)	14/251	0,421391296	0,837188903
BL2845 (MED27)	9/157	0,421948438	0,837369497
BL1042 (AGAP1)	15/270	0,422046061	0,836638769
BL6340 (ANKRD17)	6/101	0,422138098	0,835898594
BL3348 (ATN1)	10/176	0,423615287	0,83789984
BL457 (TCERG1)	11/195	0,42489971	0,839515819
BL810 (PRKAA2)	7/120	0,425362518	0,839506685
EPI-2416-1 (HMGB3)	7/120	0,425362518	0,838585163
BL1999 (DDB1)	12/214	0,425893978	0,838712264
BL5752 (FKBP5)	2/28	0,42762859	0,841205857
SC-81983 (PROX1)	2/28	0,42762859	0,840285501
BL7063 (CHUK)	8/139	0,427672846	0,839454023
BL2602 (MED24)	5/83	0,427755717	0,838700074
BL5432 (ERCC5)	26/480	0,427834099	0,837938976
CBC171 (AGAP3)	10/177	0,430622936	0,842482346
BL1028 (INTS9)	10/177	0,430622936	0,841565608
BL1855 (RBM14)	11/196	0,431558799	0,84247783
SRC3_Wu (NCOA3)	12/215	0,43225212	0,842915101
BL373 (TOPBP1)	23/424	0,432858677	0,843182411
BL6076 (CCNE1)	14/253	0,433115192	0,842768022
BL2753 (LCOR)	17/310	0,433558693	0,842717979
BL9569 (MAPK7)	4/65	0,433710529	0,842101741
BL5346 (PTPN12)	4/65	0,433710529	0,841192344
BL2801 (CCNA1)	8/140	0,435573076	0,843893468
BL480 (UBE3A)	9/159	0,436776892	0,845313898
BL2446 (SAFB)	9/159	0,436776892	0,84440398
BL1238 (MAML1)	9/159	0,436776892	0,843496019
BL2156 (USP34)	9/159	0,436776892	0,842590008
BL8472 (MADD)	10/178	0,437625353	0,843320958
BL735 (TERF2IP)	22/406	0,437707794	0,842575774
BL8699 (ARID3B)	5/84	0,438007127	0,84224925
BL4054 (USP33)	5/84	0,438007127	0,841348449
CBC622 (ZNF687)	19/349	0,43849957	0,841394475
BL5941 (WNK1)	30/559	0,438779874	0,841033782
BL7566 (HERC2)	13/235	0,438850861	0,840273077
BL595 (APTX)	17/311	0,438856891	0,83938975
BL4198 (USP24)	3/47	0,439891843	0,840474203
BL9565 (MAPK6)	3/47	0,439891843	0,839581032
EPI-1622-1 (NCK1)	7/122	0,442349738	0,843375934
BL7838 (PDLIM7)	7/122	0,442349738	0,84248158
BL4506 (SMARCAD1)	7/122	0,442349738	0,84158912
BL1165 (TRIM24)	7/122	0,442349738	0,84069855
BL2786 (PSMC5)	10/179	0,444620415	0,844120788
BL1168 (MCPH1)	11/198	0,444863887	0,843691174
BL313 (SMC3)	12/217	0,444958934	0,842981271
BL5424 (ERCC3)	12/217	0,444958934	0,842092988
BL665 (ARID3A)	4/66	0,44532807	0,841904435
EPI-1114-1 (EGFR)	4/66	0,44532807	0,841019152
BL1040 (QKI)	4/66	0,44532807	0,840135728
BL1484 (MLST8)	4/66	0,44532807	0,839254159

BL1880 (NIPBL)	4/66	0,44532807	0,838374437
BL3413 (RAD52)	2/29	0,445476512	0,837775724
BL2243 (NUP214)	5/85	0,448216045	0,842046044
CBC562 (BAT3)	6/104	0,449863757	0,844258419
BL327 (RFXAP)	6/104	0,449863757	0,843377147
BL7836 (PDLIM7)	15/275	0,450309187	0,843331909
BL1304 (SETX)	15/275	0,450309187	0,842453438
BL5590 (MYBL2)	7/123	0,450813237	0,842518807
BL2138 (ROCK1)	7/123	0,450813237	0,841643008
BL5254 (TAF3)	7/123	0,450813237	0,840769028
BL2442 (RNF4)	13/237	0,451026267	0,840293751
JQ006 (MSH2)	13/237	0,451026267	0,83942298
BL2920 (SPHK1)	13/237	0,451026267	0,838554012
BL1038 (RCOR1)	8/142	0,451331657	0,838254039
BL2671 (FANCB)	9/161	0,451566513	0,83782382
BL2750 (SNW1)	10/180	0,451606034	0,837032443
BL5428 (ERCC4)	21/390	0,452257172	0,837375135
BL476 (AHCTF1)	20/371	0,452881924	0,837668317
BL5624 (AKAP2)	3/48	0,453599363	0,838132156
BL2767 (PRMT2)	3/48	0,453599363	0,837270766
BL5495 (RAD18)	23/429	0,45554644	0,840001444
BL2263 (DOCK9)	15/276	0,455954716	0,839891969
BL3964 (PES1)	15/276	0,455954716	0,839031424
SC-372 (RELA)	14/257	0,456540427	0,839249341
SC-15402 (MKI67)	4/67	0,456871178	0,838998606
BL6523 (E2F4)	4/67	0,456871178	0,83814161
BL1022 (FAM184B)	12/219	0,457642533	0,838699989
BL4419 (FAM91A1)	12/219	0,457642533	0,837845045
CBC601 (RAD18)	5/86	0,458376644	0,838334474
BL73 (CLSPN)	7/124	0,459251941	0,839080861
BL6694 (UTY)	7/124	0,459251941	0,838228137
BL2453 (ZFPM2)	15/277	0,461595267	0,841649848
BL4398 (ARFGEF2)	14/258	0,462384743	0,842234278
BL1053 (N4BP2)	14/258	0,462384743	0,84138095
BL2158 (USP34)	25/469	0,462816596	0,841314379
EPI-1006-1 (CASP8)	2/30	0,463008482	0,840812168
BL625 (PUM2)	2/30	0,463008482	0,839962863
BL921 (CCL18)	2/30	0,463008482	0,839115272
BL4827 (CEP350)	10/182	0,465540745	0,84285401
SC-67074 (SENP1)	10/182	0,465540745	0,842005215
BL1285 (ASH2L)	3/49	0,467165176	0,844093215
BL1490 (NIPBL)	3/49	0,467165176	0,84324488
SC-20022 (FANCD2)	3/49	0,467165176	0,842398249
SC-28746 (HEY1)	3/49	0,467165176	0,841553316
BL1244 (MAML3)	15/278	0,467229743	0,840826271
CBC602 (RAD18)	7/125	0,467662423	0,840762474
BL1193 (SETD1A)	6/106	0,468186182	0,840862384
BL4557 (ATXN10)	6/106	0,468186182	0,840022361
BL1744 (CITED1)	6/106	0,468186182	0,839184016
BL1802 (MTA1)	4/68	0,468332155	0,838608724
BL6028 (WDR20)	4/68	0,468332155	0,837773456
BL4994 (MAK16)	4/68	0,468332155	0,836939851
BL1586 (EGFR)	4/68	0,468332155	0,836107903

BL2152 (NEK7)	4/68	0,468332155	0,835277607
BL2147 (BUB1B)	4/68	0,468332155	0,834448959
BL1996 (DDB1)	5/87	0,468483321	0,833891026
BL1861 (NRIP1)	19/355	0,46849197	0,83308077
BL3631 (KDM2A)	12/221	0,470290482	0,835451736
BL1462 (MAD2L1)	20/375	0,472344369	0,838271231
BL1354 (FUS)	20/375	0,472344369	0,837443718
BL5634 (AKAP12)	10/183	0,472485823	0,83686838
BL2752 (LCOR)	15/279	0,472857057	0,836700763
BL4381 (NASP)	9/164	0,473635768	0,83725378
BL2779 (SUV39H1)	8/145	0,474821665	0,838524788
BL860 (CSPG4)	8/145	0,474821665	0,83770109
BL1005 (WAPAL)	7/126	0,476041345	0,83902871
BL3555 (SUPT5H)	12/222	0,476597292	0,839185036
CBC52 (NAT13)	12/222	0,476597292	0,838363111
BL5892 (MAP4K4)	20/376	0,477200223	0,838602348
BL1231 (BRD8)	5/88	0,478530702	0,840118416
BL7746 (OBFC2A)	4/69	0,479703714	0,841355342
RFX5 (RFX5)	4/69	0,479703714	0,840534507
BL1408 (MLL)	4/69	0,479703714	0,839715273
BL1684 (BUB3)	14/261	0,479869416	0,839187412
BL2417 (UBAP2L)	2/31	0,480209569	0,838965355
BL2727 (SCAND1)	3/50	0,480578809	0,8387945
BL1836 (CAMK4)	3/50	0,480578809	0,837980136
a-ATR (YW) (ATR)	17/319	0,481143779	0,838151529
BL3200 (SIRT1)	13/242	0,481336965	0,837675571
BL757 (BANP)	8/146	0,482601167	0,839062629
BL8104 (AHDC1)	23/435	0,482722393	0,838461719
BL1103 (CENPB)	12/223	0,482890679	0,837943632
BL2608 (HDAC1)	19/358	0,483437487	0,838082748
BL5507 (HDAC7)	7/127	0,484385463	0,838916385
BL2587 (NCOR2)	11/204	0,48454569	0,838385414
BL7844 (ARHGEF16)	6/108	0,486339746	0,840679677
EPI-2698-1 (APBB1IP)	5/89	0,488513638	0,843625475
BL342 (MEN1)	5/89	0,488513638	0,842815076
BL7356 (WDR89)	5/89	0,488513638	0,842006233
BL1685 (BUB3)	12/224	0,489169186	0,842327765
BL1258 (MDM4)	15/282	0,489685347	0,842408893
BL2712 (FANCE)	8/147	0,490350996	0,842746784
a-SKI (SKI)	4/70	0,49097898	0,843019357
BL3443 (NDC80)	4/70	0,49097898	0,842214181
BL7536 (EEA1)	4/70	0,49097898	0,841410542
BL3314 (RNF40)	11/205	0,491107188	0,840827941
CBC652 (AGAP4)	11/205	0,491107188	0,840027152
BL3837 (KDM5A)	14/263	0,491473811	0,839854391
BL2384 (ERCC4)	13/244	0,493382044	0,842313831
BL1074 (AURKB)	16/302	0,493411477	0,841564114
BL1016 (INTS6)	18/341	0,495086087	0,843619176
BL2289 (BTBD12)	15/283	0,495273398	0,84313841
BL906 (TP73)	6/109	0,495343248	0,842458782
BL3282 (C7orf27)	9/167	0,495511445	0,841947545
BL5829 (PHF6)	20/380	0,496563689	0,842937982
BL1424 (FOXO1)	17/322	0,496896436	0,842706325

BL463 (RBM39)	11/206	0,497649384	0,84318707
BL5202 (ROBO1)	1/13	0,497890471	0,842800458
BL4422 (ZRANB2)	5/90	0,498427214	0,842914572
BL6649 (GRB10)	16/303	0,498812269	0,842772188
BL3259 (HMGB3)	4/71	0,502151483	0,847616601
BL8995 (CASC3)	4/71	0,502151483	0,846820717
BL6925 (FGFR1OP)	4/71	0,502151483	0,846026326
BL2798 (CCNA1)	8/149	0,5057515	0,851293059
BL7903 (C16orf53)	8/149	0,5057515	0,850495968
SC-9086 (NR2C2)	3/52	0,50691194	0,851649995
SC-5606 (XRCC4)	3/52	0,50691194	0,85085406
BL780 (MCM5)	3/52	0,50691194	0,850059612
BL1014 (INTS3)	12/227	0,507901227	0,850924071
BL1007 (WAPAL)	5/91	0,508266738	0,850742835
SC-12340R (CDC2)	5/91	0,508266738	0,849950709
BL1776 (STK10)	5/91	0,508266738	0,849160057
BL6202 (ESRRA)	14/266	0,50877938	0,849226548
BL1865 (FHL2)	7/130	0,509177998	0,849102771
BL391 (RAD21)	9/169	0,509958551	0,849615545
BL1752 (MED14)	9/169	0,509958551	0,848828135
BL5777 (WTAP)	15/286	0,511959112	0,851369042
SC-6233 (ATF2)	6/111	0,513185032	0,85261824
BL666 (BIRC6)	4/72	0,513215153	0,851880235
HEXIM1 (JW) (HEXIM1)	4/72	0,513215153	0,851093642
BL1114 (CENPH)	4/72	0,513215153	0,850308501
BL1477 (PNKP)	8/150	0,513397279	0,84982628
MTMR3 (MTMR3)	2/33	0,513571053	0,849331134
EPI-1786-1 (INPP5D)	2/33	0,513571053	0,84854978
BL9749 (SIK2)	2/33	0,513571053	0,847769863
BL8125 (EPHA2)	2/33	0,513571053	0,846991379
BL1581 (E4F1)	2/33	0,513571053	0,846214322
BL1572 (ITCH)	2/33	0,513571053	0,845438691
BL444 (NRIP1)	2/33	0,513571053	0,844664479
BL1801 (BCL11B)	9/170	0,517134891	0,849747725
BL1004 (ATF7IP)	9/170	0,517134891	0,848970991
BL2262 (DOCK9)	13/248	0,517280285	0,848434148
BL1733 (SPEN)	7/131	0,51735239	0,847778186
BL555 (TRIM28)	17/326	0,517750167	0,847656608
BL2447 (SMARCA4)	5/92	0,518027749	0,847338649
BL4341 (SFRS8)	3/53	0,5198147	0,849487898
BL1245 (MAML3)	3/53	0,5198147	0,848715637
BL7207 (FOXP4)	3/53	0,5198147	0,847944778
BL4659 (PTPN11)	3/53	0,5198147	0,847175318
SC-100432 (MAK)	3/53	0,5198147	0,846407253
BL7800 (ARHGEF6)	3/53	0,5198147	0,84564058
BL7009 (ZNF768)	3/53	0,5198147	0,844875294
BL131 (MSH6)	14/268	0,520236183	0,844795826
BL3467 (SUPT6H)	10/190	0,52050759	0,844473018
BL2461 (DDX5)	20/385	0,520582508	0,843832297
BL2393 (CDT1)	8/151	0,521003598	0,843753346
BL6517 (BMI1)	8/151	0,521003598	0,842993208
BL1113 (CENPH)	6/112	0,522016281	0,843871504
BL5930 (SPAG5)	17/327	0,52293133	0,84459053

BL1874 (NCOA6)	4/73	0,52416431	0,845821294
BL2311 (MLLT3)	4/73	0,52416431	0,845062029
BL5519 (PCNT)	7/132	0,52547721	0,846418897
BL1551 (HIPK2)	7/132	0,52547721	0,845660457
BL1425 (FOXO1)	14/269	0,525937303	0,845643148
BL2756 (CRABP2)	2/34	0,529712675	0,850951667
EPI-1640-1 (MAP2K6)	2/34	0,529712675	0,85019121
a-HCV15 ()	2/34	0,529712675	0,849432111
BL7352 (WDR70)	2/34	0,529712675	0,848674366
BL1854 (RBM14)	11/211	0,530016495	0,8484043
BL5747 (FKBP4)	11/211	0,530016495	0,847648819
BL8804 (STIL)	6/113	0,530783321	0,848119969
BL3661 (CDCA7L)	6/113	0,530783321	0,847366085
BL1097 (DYRK3)	6/113	0,530783321	0,846613539
CBC31 (POLDIP3)	16/309	0,530937843	0,846108577
BL1575 (CDC2L6)	9/172	0,531383325	0,846067777
BL6228 (PBXIP1)	14/270	0,531618853	0,845693056
BL119 (ATR)	14/270	0,531618853	0,844944655
CBC632 (AKAP13)	14/270	0,531618853	0,844197577
BL974 (PNKP)	3/54	0,532531736	0,844900175
BL1915 (SLK)	3/54	0,532531736	0,844154455
BL758 (BANP)	3/54	0,532531736	0,843410051
BL6636 (FOXK2)	19/368	0,532706306	0,842943195
BL2460 (DDX17)	7/133	0,533549804	0,843534725
BL1009 (FKBP15)	7/133	0,533549804	0,84279283
BL2125 (HDAC3)	7/133	0,533549804	0,842052238
BL2097 (NMI)	4/74	0,53499366	0,843589652
SC-834 ()	4/74	0,53499366	0,842849661
BL7029 (ZAP70)	4/74	0,53499366	0,842110967
BL6688 (ASH1L)	4/74	0,53499366	0,841373567
BL4546 (HMG20B)	18/349	0,53545559	0,84136329
a-USP9X (USP9X)	8/153	0,536088848	0,841622002
BL6507 (FBXO28)	5/94	0,537297506	0,842782813
BL2074 (RECQL)	5/94	0,537297506	0,8420474
BL2603 (MED24)	5/94	0,537297506	0,84131327
EPI-1650-1 (MAP2K4)	6/114	0,539482998	0,843999534
SC-28673 (ATF1)	6/114	0,539482998	0,843264983
BL1017 (INTS5)	7/134	0,541567612	0,845787332
BL482 (MAP3K7IP3)	8/154	0,543563459	0,848166787
BL5589 (MYBL2)	12/233	0,544778157	0,849324279
BL2768 (PRMT2)	12/233	0,544778157	0,848587658
SC-101092 (ITGB3BP)	3/55	0,545056593	0,848285651
BL2054 (ESR1)	3/55	0,545056593	0,847551204
SC-48809 (RFX1)	3/55	0,545056593	0,846818028
SC-198 (CCNE1)	2/35	0,545485494	0,846751898
BL3069 (DDX20)	2/35	0,545485494	0,846020679
BL2789 (PTEN)	2/35	0,545485494	0,845290722
BL5622 (AKAP1)	4/75	0,545698286	0,844891484
BL3256 (EIF4ENIF1)	5/95	0,546798449	0,845865646
BL2224 (COL4A3BP)	5/95	0,546798449	0,845137706
CBC332 (ARAF)	6/115	0,548112302	0,846439977
BL4396 (ARFGEF1)	18/352	0,550363463	0,849186237
BL8209 (UTP3)	8/155	0,550989968	0,849423161

CBC612 (RNF20)	9/175	0,552467548	0,850970597
BL1002 (ATF7IP)	13/254	0,552505364	0,8502996
CBC611 (RNF20)	14/274	0,554129382	0,852068811
BL1819 (IRF3)	4/76	0,556273637	0,854634261
BL453 (TRIP6)	4/76	0,556273637	0,853903805
BL4950 (HMGN3)	12/235	0,556859272	0,854072803
BL1352 (FUS)	12/235	0,556859272	0,853344072
a-PTEN (WG) (PTEN)	12/235	0,556859272	0,852616583
BL7015 (ZNF8)	3/56	0,557383521	0,852692337
EPI-1618-1 (TEC)	3/56	0,557383521	0,851966641
BL4494 (SMARCA5)	7/136	0,55742915	0,851311865
BL2388 (CUL4A)	7/136	0,55742915	0,850588575
BL448 (PPARGC1A)	8/156	0,5583664	0,851295462
BL9239 (GREB1)	9/176	0,559411678	0,852165711
BL1355 (FUS)	9/176	0,559411678	0,851443537
BL2185 (TSC2)	9/176	0,559411678	0,850722586
BL2030 (RFWD2)	16/315	0,56245066	0,854620461
BL2758 (MED17)	6/117	0,565148461	0,857993775
BL2269 (TDP1)	6/117	0,565148461	0,857269118
BL695 (CC2D1A)	6/117	0,565148461	0,856545685
BL5062 (POLH)	6/117	0,565148461	0,855823471
BL2600 (BRIP1)	14/276	0,565241826	0,85524374
BL699 (DIDO1)	7/137	0,565268266	0,85456381
ab81302 (C19orf21)	5/97	0,565514604	0,854217181
BL2268 (TDP1)	4/77	0,56671552	0,855311827
EPI-1236-1 (MAP2K2)	4/77	0,56671552	0,854593681
BL2129 (RBM15)	4/77	0,56671552	0,85387674
BL2788 (PTEN)	10/197	0,567064525	0,85368641
BL5856 (CDCA3)	11/217	0,567909769	0,854242835
BL3961 (PES1)	21/415	0,56896054	0,855107221
BL7116 (MYCBP2)	3/57	0,569507444	0,85521352
BL2164 (AKT2)	3/57	0,569507444	0,854499056
BL4520 (SMARCA1)	1/16	0,571724118	0,857108945
EPI-1629-1 (SHC1)	8/158	0,57296149	0,85824757
BL4284 (HGS)	5/98	0,574723317	0,860169231
BL440 (CREBBP)	5/98	0,574723317	0,85945302
BL5619 (DAXX)	18/357	0,574856561	0,858937092
CBC42 (NAT10)	13/258	0,575471549	0,859141232
a-HCV9 ()	2/37	0,57590642	0,859076354
BL2595 (PIAS2)	4/78	0,577020093	0,860023309
CBC702 (PALLD)	4/78	0,577020093	0,859310189
a-FTO (78) (FTO)	26/516	0,579053004	0,861623193
BL1803 (MTA1)	7/139	0,580752411	0,863436531
BL3199 (SIRT2)	6/119	0,581870567	0,864383406
BL2799 (CCNA1)	11/220	0,586386029	0,870371329
BL1797 (BCL11A)	11/220	0,586386029	0,869652608
BL7129 (CCDC124)	10/200	0,586437567	0,869011444
BL2394 (CDT1)	12/240	0,586512442	0,868405892
BL5576 (CEP110)	9/180	0,58672537	0,868005572
BL1051 (N4BP2)	9/180	0,58672537	0,867291164
BL4516 (ATRX)	14/280	0,587144994	0,867197704
BL461 (RBM14)	4/79	0,587183851	0,866542479
EPI-1931-1 (PLCG2)	4/79	0,587183851	0,865831032

BL2380 (RFWD3)	4/79	0,587183851	0,865120752
BL9162 (CARD11)	4/79	0,587183851	0,864411637
BL1546 (DYRK2)	4/79	0,587183851	0,863703683
SC-97 (CDC25A)	4/79	0,587183851	0,862996888
BL2507 (TLK1)	15/300	0,587598618	0,862900341
BL2465 (NCOA7)	16/320	0,588119328	0,862959406
SC-584 (EP300)	7/140	0,588393424	0,862656807
BL5618 (DAXX)	6/120	0,590107827	0,864464648
BL6323 (ABL1)	6/120	0,590107827	0,863760112
EPI-2263-1 (PLCG2)	2/38	0,590548963	0,863701903
EPI-2610-1 (BAG2)	2/38	0,590548963	0,862999135
BL8719 (CBL)	2/38	0,590548963	0,86229751
BL8107 (AHDC1)	20/400	0,590649368	0,861743513
BL2285 (ZC3H13)	11/221	0,592467221	0,863694099
BL4650 (KIF22)	10/201	0,592808028	0,86349004
BL1111 (CENPF)	15/301	0,592822329	0,862811104
BL698 (DIDO1)	5/100	0,592827344	0,862119765
BL4565 (KIF1B)	5/100	0,592827344	0,861422257
SC-797 (GADD45A)	5/100	0,592827344	0,860725877
BL9584 (ELF4)	5/100	0,592827344	0,860030622
BL6259 (MBD4)	5/100	0,592827344	0,859336489
SC-66857 (DCLK1)	5/100	0,592827344	0,858643476
BL1491 (NIPBL)	3/59	0,593129159	0,858388372
BL2120 (MYST1)	3/59	0,593129159	0,857697238
BL8483 (AAK1)	3/59	0,593129159	0,857007217
BL8964 (DGCR8)	3/59	0,593129159	0,856318304
TIF2 (NCOA2)	16/321	0,593179758	0,85570349
BL6983 (ZBTB40)	9/181	0,593430639	0,855378353
SC-7230 (ATM)	1/17	0,593840785	0,855283119
BL3029 (HIST4H4)	1/17	0,593840785	0,854597796
BL2444 (RPL7)	8/161	0,594433361	0,854765665
BL4249 (PPP2R2B)	7/141	0,595964551	0,856281867
BL6197 (NR2F6)	7/141	0,595964551	0,855597389
BL5845 (CCDC82)	4/80	0,597203617	0,856691451
BL750 (PELP1)	4/80	0,597203617	0,856007739
BL1570 (TRIM21)	4/80	0,597203617	0,855325117
BL8233 (TOP3A)	13/262	0,597950182	0,855711973
BL464 (AHCYL2)	6/121	0,598259624	0,855473156
BL239 (RAD17)	23/462	0,601267352	0,859090027
BL754 (CSNK2A1)	12/243	0,603885965	0,862145623
CBC642 (CC2D1B)	11/223	0,60450683	0,862346519
BL3004 (DDX10)	3/60	0,604619888	0,861823269
BL2591 (RPL7A)	3/60	0,604619888	0,861139825
BL5800 (GSG2)	3/60	0,604619888	0,860457463
BL1492 (NIPBL)	3/60	0,604619888	0,859776183
EPI-1594-1 (BMX)	2/39	0,604811304	0,85936796
BL1704 (PHB2)	6/122	0,606323923	0,860836179
BL2546 (CTCF)	13/264	0,608988901	0,863936861
BL749 (PELP1)	7/143	0,610890194	0,865950109
BL2462 (DDX5)	18/365	0,612954432	0,868190978
BL1576 (ESCO1)	18/365	0,612954432	0,867506825
BL9709 (LIMK1)	1/18	0,614816365	0,869456844
BL404 (RAD9A)	1/18	0,614816365	0,868772771

BL2367 (KIAA0528)	8/164	0,615362139	0,868860379
BL6995 (ZFYVE19)	3/61	0,615893424	0,868927407
SC-25359 (FOXO4)	4/82	0,616800048	0,869523458
BL5499 (STC2)	4/82	0,616800048	0,868841479
BL6680 (SETD7)	10/205	0,617822284	0,86959939
BL2433 (UBA3)	10/205	0,617822284	0,868918419
BL435 (NCOA1)	15/306	0,618506827	0,869200518
BL274 (RFC1)	2/40	0,618693656	0,868783273
BL2420 (DHX36)	2/40	0,618693656	0,868104536
BL2454 (ZFPM2)	14/286	0,619097415	0,86799294
CBC551 (TACC3)	5/103	0,619161958	0,8674063
BL4310 (SAPS2)	5/103	0,619161958	0,866730223
EPI-2305-1 (TNFSF9)	6/124	0,622182559	0,870280278
BL2492 (PARP10)	6/124	0,622182559	0,869603017
BL2666 (HDAC2)	8/165	0,622211052	0,868966601
BL1574 (CDC2L6)	8/165	0,622211052	0,868291413
BL2838 (UIMC1)	17/347	0,622544753	0,86808259
BL2685 (ZBTB7A)	7/145	0,625516383	0,871549592
BL1998 (DDB1)	4/83	0,626371905	0,872065071
BL2748 (SNW1)	4/83	0,626371905	0,871389575
CBC571 (ESPL1)	4/83	0,626371905	0,870715125
BL2373 (INPPL1)	4/83	0,626371905	0,870041718
BL5549 (DEK)	18/368	0,626845413	0,870026555
BL7949 (PIK3R2)	3/62	0,626947593	0,86949643
BL5201 (ROBO1)	3/62	0,626947593	0,868825523
BL1834 (CAMK4)	3/62	0,626947593	0,868155649
BL1827 (RCN1)	5/104	0,627712366	0,868545
BL4934 (TRIM47)	5/104	0,627712366	0,867876374
BL3632 (KDM2A)	11/227	0,628067491	0,867699396
BL6398 (BCAR3)	8/166	0,62899415	0,868311679
BL6318 (PHF12)	8/166	0,62899415	0,867644772
BL1796 (BCL11A)	10/207	0,630033894	0,86841203
CBC752 (USP9X)	12/248	0,632064442	0,870542744
BL863 (ST14)	2/41	0,632197223	0,8700584
BL1866 (FHL2)	7/146	0,632713548	0,870102244
BL2854 (MED18)	7/146	0,632713548	0,86943652
SC-242 (ATF2)	1/19	0,63470968	0,87151268
SC-952 (CCNF)	1/19	0,63470968	0,870846895
BL6193 (CASP8AP2)	1/19	0,63470968	0,870182126
BL8570 (MAP3K2)	8/167	0,635710257	0,870889108
BL2430 (NCOA4)	8/167	0,635710257	0,870225321
BL4319 (ANKRD28)	4/84	0,635790137	0,86967181
a-REGy (PSME3)	5/105	0,636145778	0,869496055
CBC641 (CC2D1B)	12/249	0,637576169	0,870788441
BL7700 (ARHGAP29)	6/126	0,637670087	0,870254921
CBC582 (KDM5C)	3/63	0,637780712	0,869744995
BL511 (PAK4)	3/63	0,637780712	0,869085098
BL1758 (CTBP1)	3/63	0,637780712	0,868426201
BL434 (NCOA1)	11/229	0,63957417	0,870208492
BL2253 (PRKRIR)	7/147	0,63983167	0,869899833
BL7394 (CDK2)	7/147	0,63983167	0,869241815
CBC192 (ERCC6L)	7/147	0,63983167	0,868584792
BL7477 (CHTF18)	12/250	0,643044842	0,872287414

BL1544 (TTK)	15/311	0,643409084	0,872122803
BL8796 (DAB2IP)	5/106	0,644460603	0,872889323
BL1822 (IRF3)	5/106	0,644460603	0,872231532
BL8230 (TOP3A)	14/291	0,644797693	0,872030615
BL7613 (IQWD1)	4/85	0,645053048	0,871719544
SC-7160 (XBP1)	2/42	0,645324095	0,871430131
BL6014 (UTP18)	2/42	0,645324095	0,870775413
BL456 (TCERG1)	7/148	0,646869548	0,872205486
BL1706 (PHB2)	10/210	0,64796037	0,873020874
BL5104 (BRF1)	3/64	0,648391557	0,872946954
EPI-2118-1 (AKT1)	3/64	0,648391557	0,872293062
BL7655 (C2orf3)	3/64	0,648391557	0,871640148
BL1123 (RNF41)	7/149	0,653826066	0,878288418
BL2255 (PRKRIR)	18/374	0,653904557	0,877737358
BL6306 (POLL)	4/86	0,654159206	0,877423402
BL3571 (PPP6C)	4/86	0,654159206	0,876768608
BL992 (INCENP)	8/170	0,655445872	0,877838022
BL2001 (RIF1)	9/191	0,6574264	0,879834437
SC-56 (PCNA)	2/43	0,658077154	0,880049567
BL4928 (TRIM37)	2/43	0,658077154	0,879394768
SC-5299 (SIN3A)	2/43	0,658077154	0,878740943
SC-247 (CCNE1)	2/43	0,658077154	0,878088089
BL2014 (AGAP3)	2/43	0,658077154	0,877436205
BL7142 (CCDC43)	3/65	0,65877934	0,877720842
BL7986 (ABCD3)	3/65	0,65877934	0,877070197
BL2118 (MYST1)	10/212	0,659640964	0,877566794
BL4782 (AKT1S1)	14/294	0,659778438	0,877099981
BL5721 (ZNF830)	5/108	0,660728995	0,877713961
BL8261 (PPIG)	5/108	0,660728995	0,877065244
BL8517 (CKAP2L)	19/396	0,661518005	0,87746406
BL4187 (USP19)	9/192	0,663497356	0,879440038
CS-9665B (CASP3)	18/377	0,667049685	0,883496486
BL9428 (C7orf20)	6/130	0,667487195	0,883424467
BL7513 (ORC1L)	8/172	0,66824946	0,883782055
CBC581 (KDM5C)	5/109	0,668680226	0,88370102
BL671 (HUWE1)	3/66	0,668943677	0,88339915
BL2994 (CRTC2)	3/66	0,668943677	0,882750069
BL1037 (KDM1)	9/193	0,669505094	0,882842253
BL8111 (RAD54L2)	16/337	0,670211836	0,883125795
BL8704 (DOCK4)	2/44	0,670459987	0,882805086
BL2317 (CDK5RAP2)	2/44	0,670459987	0,882158342
a-NEK7 (NEK7)	2/44	0,670459987	0,881512546
BL1881 (NIPBL)	2/44	0,670459987	0,880867694
BL1520 (FTSJD2)	2/44	0,670459987	0,880223784
BL8445 (MAP2K1)	1/21	0,671469795	0,88090559
BL8315 (CCDC50)	4/88	0,671896795	0,880822368
BL3742 (CBX6)	4/88	0,671896795	0,880179901
BL5123 (PAK2)	13/276	0,672036097	0,879720721
BL-ATM (ATM)	11/235	0,672925501	0,880243409
BL1734 (TBL1XR1)	17/358	0,672948617	0,879632981
BL2016 (AGAP3)	7/152	0,674197613	0,880624663
SC-9019 (PTK2B)	8/173	0,674542682	0,880435071
CBC2142 (ESCO1)	6/131	0,67469366	0,879992602

SC-1751 (TIA1)	5/110	0,676508189	0,881718946
BL8901 (QSK)	5/110	0,676508189	0,881079556
BL2252 (HPS5)	5/110	0,676508189	0,880441092
BL6997 (ZKSCAN1)	5/110	0,676508189	0,879803553
BL265 (MCM7)	5/110	0,676508189	0,879166937
BL5844 (ZFC3H1)	11/236	0,678306727	0,88086687
BL1821 (IRF3)	3/67	0,678884566	0,880980261
BL2435 (MED1)	4/89	0,680526583	0,88247346
BL3390 (E2F1)	8/174	0,680762551	0,882142526
SC-867 (KIF23)	6/132	0,681799199	0,882848855
SC-67022 (SPDEF)	10/216	0,682327323	0,882896162
BL6644 (EPS15L1)	11/237	0,683635767	0,883952367
BL4577 (USP47)	5/111	0,684212105	0,884061108
BL8293 (NOTCH2)	1/22	0,688439703	0,888884046
BL9793 (NAGK)	1/22	0,688439703	0,888245479
BL4285 (HGS)	3/68	0,688602361	0,887817545
BL6638 (GNL1)	3/68	0,688602361	0,88718066
BL9062 (CDC6)	3/68	0,688602361	0,886544688
BL2249 (HPS5)	6/133	0,688803101	0,886167887
EPI-2516-1 (VTCN1)	4/90	0,688996313	0,885781946
BL561 (COPS2)	4/90	0,688996313	0,885148339
BL6380 (BCAR1)	9/197	0,692890686	0,889515133
BL437 (NCOA2)	14/301	0,693362475	0,889485003
BL2751 (SNW1)	7/155	0,693804949	0,889417336
BL4071 (ILKAP)	7/155	0,693804949	0,888782945
BL1824 (TAOK3)	2/46	0,694132378	0,888568604
BL4540 (SMARCAL1)	2/46	0,694132378	0,887935721
BL1116 (CENPJ)	2/46	0,694132378	0,887303738
BL1778 (C21orf7)	2/46	0,694132378	0,886672654
EPI-1605-1 (PIK3CG)	2/46	0,694132378	0,886042468
BL6059 (WDR91)	2/46	0,694132378	0,885413176
BL3513 (KDM4A)	17/363	0,694597351	0,88537746
BL2757 (CRABP2)	6/134	0,695704759	0,886160104
SC-28676 (SATB1)	6/134	0,695704759	0,885532067
BL521 (EGLN1)	4/91	0,697305711	0,886941258
BL8551 (ITPR1)	3/69	0,698097744	0,887320275
BL7037 (NFKB1)	9/198	0,698572878	0,887296244
BL4839 (WDHD1)	10/219	0,698732887	0,886872273
BL6011 (PHIP)	8/177	0,698975824	0,886554082
BL562 (COPS2)	5/113	0,69924541	0,886270117
BL1073 (AURKB)	14/303	0,702590251	0,889881588
BL4578 (BTAF1)	2/47	0,705431946	0,892851145
BL1166 (TRIM24)	2/47	0,705431946	0,892222376
BL2485 (WNT9B)	2/47	0,705431946	0,891594493
BL2210 (LATS1)	4/92	0,705454701	0,890996233
BL268 (MCM8)	7/157	0,706443415	0,891617972
BL6077 (METTL3)	5/114	0,706573954	0,891156476
BL1944 (SMG7)	3/70	0,707371709	0,891536554
BL2145 (TIFA)	3/70	0,707371709	0,890911353
BL1832 (NIPBL)	8/179	0,7107409	0,894527439
BL1742 (CDK7)	7/158	0,712631313	0,896278598
BL1573 (CDC2L6)	7/158	0,712631313	0,895651392
BL897 (MTOR)	5/115	0,713776749	0,896463666

EPI-1480-1 (PTK2B)	2/48	0,716381176	0,899105934
BL1154 (TYRO3)	2/48	0,716381176	0,898478067
BL4960 (ABI1)	8/180	0,716509059	0,898011353
BL668 (BIRC6)	5/116	0,720853697	0,902826526
BL961 (DNMT1)	5/116	0,720853697	0,902197379
BL663 (RFC3)	4/94	0,721272096	0,902092399
BL6503 (PTK2)	4/94	0,721272096	0,901464638
BL3609 (MAZ)	6/138	0,722280216	0,902096848
BL8346 (KIAA1688)	6/138	0,722280216	0,901469957
BL2569 (C17orf71)	10/224	0,724878638	0,904084746
BL8672 (MLLT4)	3/72	0,725260757	0,903933601
SC-866 (E2F4)	3/72	0,725260757	0,90330674
BL2154 (NEK7)	3/72	0,725260757	0,902680748
BL7980 (UVRAG)	2/49	0,7269861	0,904201548
BL7647 (MAP3K7)	6/139	0,728664922	0,905662423
BL5711 (AZI1)	4/95	0,728941259	0,905379324
CBC701 (PALLD)	4/95	0,728941259	0,904753629
BL2434 (UBA3)	11/246	0,729178076	0,904422531
BL4214 (THRAP3)	17/372	0,731407591	0,90656179
BL6324 (ABL1)	12/268	0,733483085	0,908507325
BL1297 (IRS1)	3/73	0,733879166	0,908371456
BL2441 (RNF4)	11/247	0,733963431	0,907850084
BL9755 (STK17A)	1/25	0,734273137	0,907608089
BL8669 (MLLT6)	1/25	0,734273137	0,906983875
CBC591 (KLHL13)	5/118	0,734630243	0,906801317
BL5022 (CHD1)	5/118	0,734630243	0,906178514
BL1098 (DYRK3)	6/140	0,734945729	0,905945456
BL2512 (TLK2)	17/373	0,735320598	0,905785867
BL7841 (ARHGEF12)	4/96	0,736451512	0,906557172
SC-8410 (HDAC1)	2/50	0,737253058	0,906922254
BL759 (BANP)	11/248	0,738692916	0,90807151
BL2228 (FIGNL1)	8/184	0,738812674	0,907597511
CBC331 (ARAF)	6/141	0,741122664	0,909812922
BL2193 (USP4)	5/119	0,741330221	0,909446091
BL3347 (ATN1)	5/119	0,741330221	0,908825309
BL5515 (FAM129B)	5/119	0,741330221	0,908205373
BL2383 (TAOK1)	9/206	0,741602024	0,907919043
BL3453 (ZWINT)	3/74	0,742282769	0,908133415
BL1745 (CITED1)	3/74	0,742282769	0,907515217
BL6044 (WDR46)	3/74	0,742282769	0,906897859
BL282 (RFC4)	4/97	0,743803628	0,908138216
BL3622 (DTX3L)	12/271	0,746991302	0,911410583
BL1036 (KDM1)	12/271	0,746991302	0,910791839
BL8814 (MTUS1)	2/51	0,747188653	0,910414397
BL7153 (DDX43)	2/51	0,747188653	0,909797167
BL2450 (SMARCE1)	2/51	0,747188653	0,909180773
BL1738 (CARM1)	10/229	0,749496111	0,911371032
BL4457 (AATF)	14/314	0,750278197	0,911704764
BL9187 (C19orf62)	3/75	0,750473779	0,911325833
BL975 (PNKP)	3/75	0,750473779	0,910710072
BL6604 (CDC42EP3)	11/251	0,752544729	0,912606572
BL1239 (MAML1)	11/251	0,752544729	0,911990778
BL3720 (KDM4B)	10/230	0,754234282	0,913421963

BL1110 (CENPF)	10/230	0,754234282	0,912806449
BL2604 (PPARGC1B)	2/52	0,756799709	0,915294463
SC-187 (ATF2)	2/52	0,756799709	0,914678517
BL1806 (MTA1)	4/99	0,758037257	0,915558113
BL2003 (RIF1)	4/99	0,758037257	0,914942818
BL132 (MSH6)	4/99	0,758037257	0,91432835
BL5621 (AKAP1)	3/76	0,758454597	0,914217755
EPI-2476-1 (DACH1)	3/76	0,758454597	0,913604598
EPI-1526-1 (BAG1)	3/76	0,758454597	0,912992262
BL8369 (NFYA)	3/76	0,758454597	0,912380747
BL8312 (PAWR)	3/76	0,758454597	0,911770051
BL2213 (LATS2)	6/144	0,759031668	0,911853429
EPI-1785-1 (JAK3)	1/27	0,761021692	0,913632994
BL2286 (ZC3H13)	12/275	0,764280428	0,916932297
BL701 (TBK1)	4/100	0,764920995	0,917088189
SC-48788 (FOXG1)	8/189	0,764952424	0,916514045
BL1687 (BUB3)	8/189	0,764952424	0,915903036
EPI-2180-1 (EZR)	2/53	0,766093226	0,91665785
BL8376 (NFYC)	2/53	0,766093226	0,916047559
BL1485 (MLST8)	5/123	0,766884096	0,916383125
BL1601 (TSC1)	12/276	0,768473348	0,917671631
BL2251 (HPS5)	6/146	0,770455534	0,919427335
SC-67285 (PPARGC1B)	6/146	0,770455534	0,918816826
BL3600 (TP53BP2)	4/101	0,771651031	0,919631886
BL821 (NOC3L)	4/101	0,771651031	0,91902205
BL4797 (XPO6)	1/28	0,773369566	0,92045841
EPI-1728-1 (MAP2K3)	1/28	0,773369566	0,919848835
BL3241 (DHX29)	3/78	0,77379609	0,91974704
SC-10771 (SYK)	2/54	0,775076347	0,92065947
BL7623 (EML4)	4/102	0,778228766	0,923793036
BL1108 (CENPE)	4/102	0,778228766	0,923182869
BL3032 (ERBB2)	5/125	0,778921915	0,923395221
BL4912 (PML)	13/300	0,779091305	0,922986796
EPI-1962-1 (PRKD2)	6/148	0,78147079	0,925195478
BL2452 (TGFB1I1)	14/322	0,781612969	0,924754211
BL4543 (ARID1B)	2/55	0,783756324	0,92667963
BL2455 (CALCOCO1)	2/55	0,783756324	0,926069973
BL8868 (PRKCD)	2/55	0,783756324	0,925461117
BL1437 (PIM1)	8/193	0,784475195	0,925701347
BL8654 (BCCIP)	5/126	0,78475876	0,925427927
BL8037 (SKP2)	1/29	0,785080013	0,925199281
BL4507 (SMARCAD1)	1/29	0,785080013	0,924592593
BL2519 (VCP)	14/323	0,785329448	0,924280268
BL57 (HELLS)	6/149	0,786826506	0,92543576
CBC751 (USP9X)	6/149	0,786826506	0,924830108
RET (RET)	11/259	0,787004219	0,924433994
BL7442 (ZNF639)	3/80	0,788329565	0,925385555
SC-17748 (CHEK2)	3/80	0,788329565	0,924781122
BL2256 (PRKRIR)	3/80	0,788329565	0,924177479
BL2389 (CUL4A)	7/172	0,789988066	0,925517656
EPI-1958-1 (KCNJ11)	5/127	0,790475465	0,925484964
BL2670 (FANCB)	4/104	0,790933423	0,925417868
BL2853 (MED18)	4/104	0,790933423	0,924815383

BL9468 (CLOCK)	6/150	0,792081762	0,925555527
BL3987 (USP14)	2/56	0,792140489	0,925022314
BL588 (RCHY1)	3/81	0,795300825	0,928109345
EPI-1616-1 (ABL1)	1/30	0,796185909	0,928538892
BL8187 (OXR1)	4/105	0,797063618	0,928959285
BL6794 (XPA)	4/105	0,797063618	0,928356847
BL1099 (HIPK2)	6/151	0,797237235	0,927957274
BL8515 (CKAP2)	7/174	0,799638602	0,930149566
BL8287 (TOR1AIP1)	7/174	0,799638602	0,929547527
BL4237 (PPP2R1A)	14/327	0,799751113	0,929076972
BL6899 (CEP76)	2/57	0,800236223	0,929039597
BL4144 (USP35)	2/57	0,800236223	0,928439442
BL5324 (DUSP10)	2/57	0,800236223	0,927840062
BL3439 (MIS12)	3/82	0,802079325	0,929377076
BL996 (INTS1)	13/307	0,805177971	0,932365981
BL2212 (LATS1)	9/220	0,80636318	0,933136773
BL4374 (PAPOLA)	1/31	0,806718435	0,932946754
BL4148 (USP36)	1/31	0,806718435	0,932346402
BL4209 (TCEB3)	11/264	0,806718496	0,931746893
BL2449 (SMARCE1)	5/130	0,806915174	0,931375099
BL2928 (BHLHE40)	6/153	0,807251782	0,93116519
BL1124 (RNF41)	2/58	0,808050934	0,931488753
BL2032 (RFWD2)	15/351	0,808388813	0,931280505
BL2063 (CUL5)	3/83	0,808668342	0,931005348
BL1860 (TRIP6)	4/107	0,808888522	0,930662258
BL703 (TBK1)	9/221	0,810480701	0,931897144
CBC691 (GTPBP2)	9/221	0,810480701	0,93130092
BL7067 (IKBKB)	14/331	0,813465981	0,934133569
BL2221 (COL4A3BP)	9/222	0,814531907	0,934759939
EPI-2298-1 (BRAF)	9/222	0,814531907	0,93416303
BL8229 (RNF219)	2/59	0,815592036	0,934781937
BL2099 (NMI)	1/32	0,816707165	0,935463054
EPI-1804-1 (MAG)	1/32	0,816707165	0,934866838
EPI-2269-1 (USO1)	5/132	0,81729444	0,934943194
BL5657 (ANKZF1)	8/201	0,819883674	0,937308134
BL6695 (UTY)	4/109	0,820145299	0,937010787
BL5638 (AKAP13)	10/246	0,821673132	0,938159533
BL8271 (VPS4B)	7/179	0,822283088	0,938259483
BL1799 (BCL11B)	13/312	0,822393573	0,93778975
BL5626 (AKAP2)	2/60	0,822866924	0,937734134
BL969 (TRPM2)	2/60	0,822866924	0,937139502
BL866 (PXN)	2/60	0,822866924	0,936545624
BL3627 (KDM3A)	4/110	0,825565558	0,939022003
BL449 (PPARGC1A)	4/110	0,825565558	0,938427685
BL2747 (C1D)	6/157	0,826118398	0,93846214
BL8639 (MINK1)	1/33	0,826180149	0,937939032
BL8402 (RAB3GAP1)	3/86	0,827332306	0,938653709
UHRF2 (UHRF2)	3/86	0,827332306	0,938061125
BL4774 (MTOR)	3/86	0,827332306	0,937469288
BL5554 (TWISTNB)	15/357	0,827492558	0,937059669
BL8876 (RIOK2)	4/111	0,83084979	0,940268571
BL1871 (NCOA6)	4/111	0,83084979	0,939676463
BL1931 (PPP1R10)	5/135	0,832017748	0,940405208

SC-7898 (CHEK1)	3/87	0,833197469	0,941146324
CBC542 (CYLD)	7/182	0,834879284	0,942453296
BL7472 (ADRM1)	7/182	0,834879284	0,941861303
BL5659 (ANKZF1)	6/159	0,834985077	0,941389327
EPI-2399-1 (CTNNBL1)	1/34	0,835163993	0,941000334
BL7720 (ARHGEF2)	2/62	0,83664746	0,942080776
BL460 (RBM14)	5/136	0,836705463	0,941555771
BL8525 (CLASP2)	5/136	0,836705463	0,940966194
BL5646 (ANKIB1)	5/136	0,836705463	0,940377354
BL844 (PRKD2)	11/273	0,838752241	0,942088196
BL2617 (EIF2AK4)	6/160	0,839280223	0,94209205
EPI-1664-1 (MALT1)	4/113	0,841018605	0,943453726
BL3234 (DDX27)	4/113	0,841018605	0,942864803
BL2219 (HPS3)	4/113	0,841018605	0,942276616
BL2607 (HDAC1)	11/274	0,842044562	0,942837926
BL3564 (CDK5RAP3)	10/252	0,84300431	0,943324449
BL7055 (REL)	2/63	0,843167667	0,942919758
BL5316 (POLI)	3/89	0,844414733	0,943726733
BL8482 (AAK1)	4/114	0,845907492	0,944807125
CBC631 (AKAP13)	4/114	0,845907492	0,944219923
BL2505 (PITX1)	6/162	0,847599405	0,945520827
BL3816 (C12orf5)	2/64	0,849450754	0,946997861
BL1475 (PNKP)	5/139	0,850128587	0,947165597
BL1800 (BCL11B)	5/139	0,850128587	0,94657839
BL330 (CDC25A)	4/115	0,850668951	0,946593207
BL5015 (RBM34)	1/36	0,851763893	0,947224738
BL8090 (PRKRA)	1/36	0,851763893	0,946638584
BL5061 (POLH)	5/140	0,854394611	0,948975091
BL5754 (ZFPM1)	3/91	0,854971748	0,949029209
BL970 (TRPM2)	3/91	0,854971748	0,948443027
SC-25378 (KAT5)	2/65	0,855503801	0,948447424
CBC2181 (NFRKB)	6/164	0,855564427	0,947929494
BL6616 (CDC42EP1)	5/141	0,858559013	0,950660905
BL6591 (NUMBL)	5/141	0,858559013	0,950075162
BL8232 (TOP3A)	6/165	0,859416951	0,950438943
BL2382 (TAOK1)	4/117	0,85981848	0,95029784
BL3918 (PPP1CA)	3/92	0,860011638	0,949926754
BL1754 (MED23)	3/92	0,860011638	0,949342902
BL2837 (UIMC1)	11/280	0,860716895	0,949537803
BL1316 (GSK3B)	2/66	0,861333792	0,949635046
BL3431 (CRTC1)	7/189	0,861514471	0,949251528
BL922 (CUL9)	7/190	0,865016991	0,952526373
BL5018 (RBM34)	1/38	0,866693525	0,953787727
BL2053 (ESR1)	1/38	0,866693525	0,953203656
BL7981 (UVRAG)	1/38	0,866693525	0,9526203
SC-13064 (NR0B1)	1/38	0,866693525	0,952037658
BL1112 (CENPH)	1/38	0,866693525	0,951455728
BL867 (ASH2L)	1/38	0,866693525	0,950874508
SC-12739 (CSNK2B)	2/67	0,8669476	0,950572582
BL2792 (TRIM24)	10/260	0,868288738	0,951462217
EPI-2046-1 (CTNNA2)	9/238	0,870705114	0,953528284
BL5906 (NFRKB)	14/351	0,871815226	0,954162185
BL2726 (SCAND1)	2/68	0,872351988	0,954168191

BL2386 (ERCC4)	1/39	0,873585143	0,954935433
CBC1592 (ERBB2)	1/39	0,873585143	0,954354572
BL1010 (FKBP15)	5/145	0,874231862	0,954480501
BL6012 (PHIP)	4/121	0,876687086	0,956579591
BL2547 (CTCF)	4/121	0,876687086	0,95599879
BL1975 (CSE1L)	2/69	0,877553593	0,956363018
BL7340 (LRWD1)	5/146	0,877911866	0,956173263
BL746 (SPAG9)	1/40	0,880120803	0,957998159
BL4917 (TRIM16)	1/40	0,880120803	0,957417906
EPI-1970-1 (MAP1LC3A)	7/195	0,881454616	0,958288432
BL1033 (ADNP)	5/147	0,881499811	0,95775781
BL2250 (HPS5)	5/147	0,881499811	0,957178755
BL667 (BIRC6)	2/70	0,882558929	0,95774975
BL3967 (USP3)	2/70	0,882558929	0,957171399
BL1296 (IRS1)	3/97	0,882968025	0,957037159
BL3509 (KDM4A)	17/423	0,884831035	0,958478009
BL1683 (BUB1)	12/312	0,886155487	0,95933409
BL4014 (BCAS3)	1/41	0,886318873	0,95893295
EPI-2214-1 (AKT1)	1/41	0,886318873	0,958355627
SC-9118 (PDPK1)	1/41	0,886318873	0,957778999
BL2370 (SYK)	3/98	0,887135472	0,958084972
BL6033 (WDR42A)	2/71	0,887374376	0,957767055
BL4037 (CORO1A)	4/124	0,888158441	0,958037573
BL8262 (PPIG)	3/99	0,89116978	0,960708839
BL1008 (FKBP15)	4/125	0,891769556	0,960778718
BL4036 (CORO1A)	2/72	0,89200618	0,960457494
BL4911 (PML)	15/382	0,89202957	0,959907194
EPI-1482-1 (RPS6KA3)	1/42	0,892196776	0,959512221
BL5433 (ERCC6)	12/315	0,893261928	0,960082838
BL773 (TREX1)	8/223	0,894221189	0,960539028
SC-939 (BAG1)	5/151	0,894963501	0,960761774
BL5995 (ZNF638)	9/247	0,89569808	0,960975957
BL586 (FEN1)	14/362	0,897154773	0,961964162
BL1020 (FAM184B)	10/271	0,897600229	0,961867549
BL7526 (SOS2)	1/43	0,897771034	0,961476909
BL2239 (KIAA1429)	1/43	0,897771034	0,960903919
BL4805 (UBE4B)	13/340	0,898397386	0,961001611
BL1107 (CENPE)	1/44	0,903057318	0,965411276
BL3588 (HIST1H3A)	1/44	0,903057318	0,964836968
BL5675 (ANKRD26)	2/75	0,904860104	0,966188315
UHRF1 (UHRF2)	6/179	0,904964689	0,965725836
BL5772 (YAP1)	4/129	0,905210594	0,965414624
BL4508 (SMARCAD1)	4/130	0,908331074	0,96816772
BL1519 (FTSJD2)	2/76	0,908816996	0,968111106
BL1163 (SNIP1)	7/205	0,909343201	0,968097444
BL1740 (CDK7)	8/229	0,909344521	0,967525331
SC-28749 (NCOA4)	3/104	0,909462536	0,967077984
BL1118 (SMC5)	4/131	0,91136001	0,968522236
BL2220 (HPS3)	4/131	0,91136001	0,967949484
EPI-1597-1 (BLNK)	2/77	0,912619361	0,968714168
SC-14031 (FOXH1)	6/182	0,91287631	0,968414561
CBC692 (GTPBP3)	5/158	0,915355282	0,970471125
BL2679 (PHLPP1)	2/78	0,916272592	0,970870545

BL1025 (INTS7)	2/78	0,916272592	0,970298098
EPI-2282-1 (PDK2)	1/47	0,917333173	0,970848779
BL809 (PRKAA1)	8/233	0,918371274	0,971375034
BL2725 (SCAND1)	2/79	0,919781935	0,9722945
BL5475 (BRD1)	2/79	0,919781935	0,971722562
BL8493 (NARG1)	1/48	0,921608729	0,973080116
BL2242 (NUP214)	1/48	0,921608729	0,972508388
BL616 (CUL9)	1/48	0,921608729	0,971937332
BL3033 (ERBB2)	2/80	0,923152495	0,972994062
BL1820 (IRF3)	4/136	0,925205852	0,97458634
BL3821 (KDM3B)	2/81	0,926389233	0,975260881
BL1021 (FAM184B)	8/238	0,928559692	0,976973174
BL8690 (ARHGEF5)	1/50	0,929508438	0,977398802
BL1353 (FUS)	1/50	0,929508438	0,97682689
BL4155 (USP38)	1/50	0,929508438	0,976255646
BL751 (PELP1)	1/50	0,929508438	0,97568507
BL438 (NCOA3)	4/138	0,93017663	0,975816137
BL2264 (DOCK9)	3/111	0,930397399	0,97547795
BL4287 (PPP2R5A)	10/288	0,932113462	0,976706989
PHF2-C Ab (PHF2)	10/288	0,932113462	0,97613748
BL7583 (WDR21A)	1/51	0,933154815	0,976658536
BL1688 (MAD1L1)	11/313	0,934238635	0,977223406
BL2182 (TSC2)	2/84	0,935344002	0,977810144
SC-251 (E2F1)	1/52	0,93661274	0,978566889
BL7051 (RELA)	3/114	0,937925444	0,979368661
SC-8408 (CHEK1)	1/53	0,939891944	0,98085179
EPI-2064-1 (ERBB2)	3/115	0,940262661	0,980668838
BL2464 (TGS1)	1/54	0,943001654	0,982954713
BL2765 (HNRNPU)	5/172	0,945950379	0,985456427
BL5196 (CEP72)	1/55	0,945950624	0,984885403
SC-28241 (MGMT)	1/56	0,948747154	0,987224733
BL861 (CSPG4)	1/57	0,951399117	0,98941101
BL1862 (NRIP1)	10/303	0,953729901	0,991260938
BL7341 (WDFY3)	6/203	0,953845328	0,990807524
SC-9087 (NR2C1)	1/58	0,95391398	0,990306074
BL1835 (CAMK4)	2/92	0,954441331	0,990281127
BL6127 (OXSR1)	5/178	0,955684606	0,990998587
BL4268 (PPP2R3B)	1/59	0,956298824	0,991063294
BL5212 (DOCK10)	2/93	0,956409321	0,990606194
BL1846 (IGBP1)	1/60	0,958560365	0,992261911
BL6158 (SFRS18)	1/60	0,958560365	0,991690331
EPI-1586-1 (MAPK1)	1/60	0,958560365	0,99111941
BL3041 (ERBB4)	2/95	0,960103841	0,99214413
BL2131 (RBM15)	6/208	0,960578848	0,992064181
BL2267 (TDP1)	2/96	0,961836591	0,992792252
BL1577 (ESCO1)	1/62	0,962738684	0,9931526
BL3194 (CDX2)	1/63	0,964667232	0,994570809
BL8332 (C1orf55)	10/315	0,966388562	0,995773872
BL1121 (SMC6)	2/99	0,966611894	0,99543289
BL3238 (DDX28)	1/65	0,968230286	0,996528134
BL843 (PRKD2)	1/65	0,968230286	0,995957384
BRD4-N (BRD4)	4/161	0,969295471	0,996482351
BL3624 (DTX3L)	1/66	0,969874831	0,996507549

BL469 (AURKA)	4/162	0,970407455	0,996484728
BL2970 (ASZ1)	2/103	0,972091904	0,997644034
BL8216 (PHRF1)	2/103	0,972091904	0,997074277
BL5013 (RBM27)	1/69	0,974315473	0,998784584
BL2368 (KIAA0528)	7/250	0,976007047	0,999947893
BL1493 (NIPBL)	1/80	0,985689806	1
BL169 (TP53)	4/182	0,98609614	1
EPI-1540-1 (CCNA2)	1/82	0,987133925	1
BL7271 (NRBF2)	1/84	0,988432409	1
CBC1591 (ERBB2)	1/91	0,992029152	1
BL1578 (MCM8)	3/167	0,9928168	1
BL3195 (CDX2)	1/95	0,993557336	1
SC-33172 (GADD45B)	1/112	0,997393155	1

Old.P.value	Old.Adjusted	Odds.Ratio	Combined.Score	Genes
0	0	2,797091025	21,59241476	GALNT7;SEPT10;SF3B
0	0	1,751850392	11,34425218	SET;SF3B3;HNRNPU;H
0	0	1,795815749	9,861422578	MTMR3;SF3B3;HNRNF
0	0	1,623184984	8,810454911	RBM28;SET;SF3B3;HN
0	0	1,558813616	8,207336126	SET;SF3B3;NUFIP2;DC
0	0	1,735508504	8,799107648	FYTTD1;MTMR3;SF3B3
0	0	2,190765083	11,07909572	CPSF7;SF3B3;CUL3;DI
0	0	2,047166721	10,26478714	SET;SF3B3;TPM3;HNR
0	0	3,083157153	15,35827172	HNRNPK;TRIM3;PDCD
0	0	2,236135957	11,00115077	ABCA1;DOCK5;HNRNF
0	0	2,392573452	11,36170827	DIAPH2;VPS13C;CARM
0	0	1,601183772	7,597171686	SET;SF3B3;HNRNPU;D
0	0	2,03998368	9,651344662	KLF10;EIF2B2;XRCC5;
0	0	1,610194834	7,561156311	SET;SF3B3;PFKFB3;HN
0	0	1,694815339	7,862505889	FNBP4;SF3B3;XRCC5;
0	0	1,914058762	8,872622945	FNBP4;RBM28;ZNF462
0	0	2,220607235	10,12078067	RBM28;RABGAP1L;SE
0	0	2,220607235	10,12078067	PCMT1;NPAT;CAPZB;R
0	0	2,456600066	11,18511944	PCMT1;HNRNPK;RFWI
0	0	2,27998176	10,08191194	FNBP4;CAPZB;CRBN;T
0	0	3,05997552	13,41725036	MEF2A;RUVBL1;HNRN
0	0	2,541619011	11,09182788	FNBP4;HNRNPK;SIN3A
0	0	1,810205299	7,828143871	RRM1;SET;RANBP3;FA
0	0	1,999507813	8,63998627	KLF10;XRCC5;HNRNPU
0	0	1,924239925	8,173538191	NCKAP1;CRBN;HNRNF
0	0	1,823985408	7,671434602	MAP2K1;SET;FAM114A
0	0	2,110676184	8,863559428	HNRNPK;MAGEE1;RFV
0	0	1,863446631	7,814990603	SF3B3;SH3KBP1;USP9
0	0	1,863446631	7,814990603	OTUD4;KANK2;DOCK5
0	0	1,72064044	7,206053366	CPSF7;XRCC5;HNRNF
0	0	1,953007632	8,084951424	KANK2;TMOD3;HNRNF
0	0	1,897327479	7,844701683	MAML1;XRCC5;HNRNF
0	0	1,583320667	6,54527649	SET;SF3B3;HNRNPU;H
0	0	2,004811548	8,213103519	HNRNPK;CHST7;SF3B
0	0	1,641035259	6,586036623	PSMD11;SH3KBP1;HNI
0	0	2,385211688	9,55140324	PCMT1;SF3B3;XPO4;H
0	0	2,049791294	8,196445265	MED14;HNRNPK;SH3K
0	0	1,634444756	6,493851475	FNBP4;SF3B3;CRBN;H
0	0	2,030269472	7,987061038	SF3B3;CAPZB;XPO4;F
0	0	1,649348507	6,477357517	KCMF1;SEPT10;SF3B3
0	0	2,349072117	9,208131988	HNRNPK;TPM3;RUVBL
0	0	1,51230004	5,791275421	OTUD4;SET;SF3B3;NU
0	0	2,466525722	9,375434615	HNRNPK;SIN3A;HNRN
0	0	1,518796627	5,587105866	RBM28;HNRNPU;RSF1
0	0	1,490757305	5,468911193	SET;DCTN2;HNRNPU;I
0	0	1,622101561	5,901217395	CPSF7;SF3B3;XRCC5;
0	0	1,542340602	5,599615345	RBM28;MAP2K1;SET;X
0	0	1,645458534	5,973987999	FNBP4;DFFA;PFKFB3;
0	0	1,937984496	7,027622215	SHC2;CPSF7;HNRNPK
0	0	2,214839424	7,976336846	FNBP4;HNRNPK;CRBN
0	0	1,637733377	5,878902507	KANK2;CRBN;USP9X;I
0	0	1,750437609	6,152276868	PCYT1B;HNRNPU;DLS

0	0	1,957560097	6,76351976	KCMF1;HNRNPK;SF3B
0	0	1,86998504	6,353556437	HNRNPK;SF3B3;CRBN
0	0	1,86998504	6,353556437	CPSF7;HNRNPK;SF3B
0	0	1,937984496	6,579568918	MED14;HNRNPK;HNRN
0	0	1,937984496	6,579568918	PCMT1;ACAP2;CPSF7;
0	0	1,937984496	6,579568918	USP15;PSMD11;SART3
0	0	1,556614053	5,275088145	SET;TPM3;TMOD3;HNF
0	0	1,761804087	5,968310796	SF3B3;HNRNPU;RC3H
0	0	1,717201452	5,794083186	FNBP4;SF3B3;HNRNPU
0	0	1,517283235	5,108231093	RBM28;MGAM;HNRNP
0	0	1,802776275	6,059183986	FYTTD1;MAP2K1;HNRI
0	0	1,550387597	5,198396233	SF3B3;ATL3;TPM3;TYF
0	0	1,749569337	5,842269562	SEPT10;SF3B3;XRCC5
0	0	1,749569337	5,842269562	TCERG1;SF3B3;NFATC
0	0	1,918796531	6,40130298	CREBBP;DIAPH2;MED
0	0	1,706401443	5,67971091	SF3B3;XRCC5;HNRNP
0	0	2,095118374	6,937220484	PCMT1;UHRF1BP1L;HI
0	0	1,490757305	4,914003838	RBM28;CREBBP;UHRF
0	0	1,599307594	5,214825708	UHRF1BP1L;HNRNPU;
0	0	1,553658457	5,064305275	SF3B3;HNRNPU;ZFP91
0	0	1,532003554	4,974973661	KANK2;SET;HSPA4L;HI
0	0	1,334240617	4,320223224	SET;SF3B3;PSMD11;DC
0	0	1,881538346	6,060991712	FNBP4;PCMT1;HNRNF
0	0	2,618897968	8,419750834	MEF2A;HNRNPK;HNRN
0	0	2,618897968	8,419750834	PCMT1;DFFA;RUVBL1;
0	0	1,564292418	5,009445163	FNBP4;SF3B3;CALCOC
0	0	1,583929636	5,042839671	SET;SF3B3;CRBN;TPM
0	0	1,435544071	4,569410493	SET;SF3B3;HNRNPU;D
0	0	1,435544071	4,569410493	NCKAP1;SET;SF3B3;HI
0	0	2,03998368	6,478296822	DDX19B;SET;HNRNPK
0	0	1,55730897	4,930064231	FNBP4;TCERG1;MAP2
0	0	1,863446631	5,898552576	CREBBP;HSPH1;MAT2
0	0	1,937984496	6,125212005	SF3B3;SP1;RUVBL1;HI
0	0	1,431465821	4,515362957	SF3B3;HNRNPU;DLST;
0	0	2,5499796	7,944778207	PCMT1;XPO4;TRIM24;I
0	0	1,590141125	4,914139627	RBM28;ROCK1;SH3KB
0	0	1,502313563	4,623744469	ZNF462;SET;SF3B3;ST
0	0	2,119670543	6,52001306	PCMT1;SF3B3;HNRNP
0	0	1,644350482	5,042119583	HNRNPU;HNRNPR;ETI
0	0	1,82828726	5,588187379	FNBP4;MED14;HNRNP
0	0	1,82828726	5,588187379	NPAT;HNRNPK;SF3B3;
0	0	1,987676406	6,054686857	ACAP2;SF3B3;HNRNPI
0	0	1,761804087	5,340908466	FNBP4;HNRNPK;CAPZ
0	0	2,484595508	7,503614852	PCMT1;RUVBL1;HNRN
0	0	1,811200464	5,439917306	PPP2CA;CAPZB;DCTN
0	0	1,811200464	5,439917306	HNRNPK;CAPZB;LUZP
0	0	1,546748189	4,637864274	FNBP4;HNRNPU;DEK;I
0	0	1,597239969	4,786252657	SF3B3;HNRNPU;ACTN
0	0	1,431127013	4,286867578	FYTTD1;KANK2;DOCK
0	0	2,236135957	6,684916596	HNRNPK;RUVBL1;RAF
0	0	2,236135957	6,684916596	NXF1;CTNNB1;HNRNP
0	0	1,407474774	4,203659088	RBM28;SF3B3;HNRNP
0	0	1,962515945	5,855083513	RBM28;SEPT10;HNRN

0	0	2,055438102	6,036443209	SEPT10;SF3B3;HNRNF
0	0	1,380752313	4,050180171	ROCK1;HNRNPU;GIGY
0	0	1,433236123	4,199147567	SEPT10;MAP2K1;SF3B
0	0	1,855517071	5,436170576	PCMT1;XPO4;SH3KBP
0	0	1,855517071	5,436170576	MAPK10;CALCOCO2;T
0	0	1,68520391	4,935967211	PCMT1;CPSF7;HNRNF
0	0	1,510117789	4,408943959	HNRNPU;LRRC40;HNF
0	0	1,579878665	4,6116665	MEF2A;SF3B3;HSPA4L
0	0	2,193944713	6,38866695	UTP15;PCMT1;TBL1XF
0	0	1,673080141	4,82586334	FNBP4;PCMT1;DDX19I
0	0	1,673080141	4,82586334	KCMF1;HNRNPK;TBL1
0	0	1,391922566	4,007335094	SET;SF3B3;HNRNPU;H
0	0	1,835985312	5,278017217	PCMT1;SF3B3;CRBN;T
0	0	2,024759921	5,810753559	SF3B3;ARL3;RUVBL1;I
0	0	2,024759921	5,810753559	HNRNPK;RUVBL1;HNF
0	0	1,462629808	4,173056128	FNBP4;SF3B3;CRBN;X
0	0	1,761804087	5,020845507	TCERG1;SF3B3;CAPZI
0	0	1,761804087	5,020845507	FNBP4;PCMT1;ZNRF2;
0	0	2,363395727	6,710889265	CRBN;APC;KIF5C;HNR
0	0	2,363395727	6,710889265	CPSF7;HNRNPK;HNRN
0	0	1,586653973	4,479722992	MAP3K2;HNRNPU;HNF
0	0	1,527475465	4,26916767	SF3B3;XRCC5;HNRNP
0	0	1,691891227	4,72691656	PCMT1;HNRNPK;SF3B
0	0	3,22997416	8,946625228	ARHGEF10;PCMT1;GA
0	0	1,798129945	4,977100603	OTUD4;ABCA2;HNRNF
0	0	1,51998784	4,194885247	SEPT10;SF3B3;HNRNF
0	0	1,637733377	4,511113156	NCKIPSD;ENAH;ARHG
0	0	1,7303433	4,761452059	MAP3K2;HNRNPK;CAF
0	0	1,416467074	3,893397023	PCYT1B;SF3B3;HNRNI
0	0	1,471886959	4,006604118	FNBP4;SF3B3;XRCC5;
0	0	1,471886959	4,006604118	FNBP4;CPSF7;SF3B3;'
0	0	1,471886959	4,006604118	USP13;SEPT11;SF3B3;
0	0	1,715030528	4,637344982	TCERG1;HNRNPK;TBL
0	0	1,365517531	3,684453245	SF3B3;DCTN2;SH3KBI
0	0	1,484042182	3,990319797	TCERG1;RBM28;CPSF
0	0	1,937984496	5,191271411	SP1;RUVBL1;HNRNPU
0	0	1,937984496	5,191271411	TCERG1;TRIM3;HNRN
0	0	1,497959031	3,97983814	FNBP4;CPSF7;SF3B3;)
0	0	1,477387284	3,924252605	AFF4;MED12L;GATAD2
0	0	1,397890456	3,686674832	FNBP4;FYTTD1;SF3B3
0	0	1,823985408	4,805936014	HNRNPK;CRBN;CALC(
0	0	1,408224028	3,696274782	FNBP4;FYTTD1;RBM28
0	0	1,564832202	4,084680521	SH3KBP1;HNRNPU;FC
0	0	1,464254953	3,795394475	FNBP4;SF3B3;XRCC5;
0	0	1,802776275	4,652954598	KANK2;SART3;HNRNP
0	0	1,357132	3,502395885	SET;SF3B3;HNRNPU;H
0	0	1,357132	3,502395885	SET;HNRNPU;HNRNPF
0	0	1,726916878	4,431448533	PURA;PCYT1B;HNRNF
0	0	1,315374092	3,37399212	KANK2;RBM28;SET;SF
0	0	1,524257469	3,90548324	FNBP4;SET;HNRNPU;I
0	0	1,476559616	3,775969048	FNBP4;CPSF7;SF3B3;I
0	0	2,004811548	5,126250728	DIAPH2;CRBN;PDE3B;
0	0	2,004811548	5,126250728	HNRNPK;PABPN1;HNF

0	0	1,38427464	3,538611952	SET;SF3B3;CRBN;XRC
0	0	1,343669251	3,427326355	RBM28;SET;SF3B3;HN
0	0	1,379794787	3,490539138	SF3B3;CRBN;HNRNPU
0	0	1,404336591	3,550741244	FNBP4;SF3B3;CRBN;H
0	0	1,782054709	4,505501697	TCERG1;CAPZB;CUL3
0	0	1,469561703	3,71038586	MAP2K1;CRBN;LRRC4
0	0	1,656397005	4,17533225	HNRNPK;SF3B3;KLHL7
0	0	1,70998632	4,305721154	NCKAP1;PCMT1;CPSF
0	0	1,429660694	3,583266559	SF3B3;HNRNPU;PAPS
0	0	1,429660694	3,583266559	OTUD4;SF3B3;MAML1;
0	0	1,363108361	3,400508978	FNBP4;MAP2K1;CPSF7
0	0	1,363108361	3,400508978	NCKAP1;TSFM;SF3B3;
0	0	1,970831691	4,911210702	HNRNPK;CRBN;CDC37
0	0	1,970831691	4,911210702	PCMT1;HNRNPK;ASXL
0	0	1,4831514	3,693476906	PHLPP2;SF3B3;HNRNI
0	0	1,38427464	3,443543139	CRBN;FAM114A1;EDEI
0	0	1,342094526	3,332331438	SET;SF3B3;HNRNPU;H
0	0	1,342094526	3,332331438	RBM28;SET;SF3B3;PHI
0	0	1,342094526	3,332331438	NCKAP1;USP15;SET;P
0	0	1,761804087	4,363338631	SEPT10;CPSF7;CNOT6
0	0	1,389236198	3,397379874	FNBP4;RBM28;CPSF7;
0	0	1,833228577	4,481786064	FUBP1;CAPRIN1;RUVE
0	0	2,106504887	5,143960963	RCAN1;HNRNPK;NFAT
0	0	2,106504887	5,143960963	PCMT1;TPM3;HNRNPF
0	0	1,303980287	3,172513184	SF3B3;PSMD11;HSPA4
0	0	1,937984496	4,706864245	HNRNPK;RUVBL1;HNF
0	0	2,349072117	5,703493121	PCMT1;HNRNPK;SUB1
0	0	1,517698702	3,681636527	DOCK5;SF3B3;HSPA4L
0	0	1,517698702	3,681636527	SF3B3;HNRNPU;LRRC
0	0	1,517698702	3,681636527	SEPT10;TPM3;XRCC5;
0	0	1,579098478	3,806256665	FNBP4;SET;MAT2A;DIF
0	0	1,426222356	3,433652004	LUZP1;XRCC5;TPM3;T
0	0	1,61498708	3,861872171	CPSF7;HNRNPK;SF3B3
0	0	1,722652885	4,093988808	PCMT1;TBL1XR1;XRC(
0	0	1,283707679	3,049616065	SET;SF3B3;HSPA4L;HN
0	0	2,061685634	4,887196592	SEPT10;SF3B3;HNRNF
0	0	2,061685634	4,887196592	CREBZF;ACAP2;PFKFI
0	0	2,061685634	4,887196592	CRBN;DLST;MTSS1L;A
0	0	1,435544071	3,399270467	CPSF7;CSNK1A1;USP9
0	0	1,906214258	4,512560201	PCMT1;CPSF7;KIF5C;(
0	0	1,315603052	3,110509	SF3B3;PDE3B;HNRNPU
0	0	1,474553421	3,47398921	FNBP4;SF3B3;HNRNPU
0	0	1,474553421	3,47398921	FNBP4;SEPT10;CPSF7
0	0	1,349229712	3,171102058	FYTTD1;SF3B3;CRBN;.
0	0	1,601640079	3,763143706	PPP2CA;SET;HNRNPK
0	0	1,33854785	3,140189884	RBM28;SF3B3;EIF2B2;
0	0	1,395348837	3,255839245	FNBP4;CPSF7;SET;YLI
0	0	1,703722634	3,966383947	HNRNPK;CAPZB;PTPN
0	0	1,344973467	3,12781027	FNBP4;RBM28;MAP2K
0	0	1,490757305	3,459882369	FNBP4;CPSF7;XRCC5;
0	0	1,490757305	3,459882369	XRCC5;HNRNPU;DLST
0	0	1,490757305	3,459882369	SET;SF3B3;TPM3;YLPI
0	0	1,308768751	3,035677122	SET;SF3B3;HNRNPU;D

0	0	1,241878766	2,867128908	TCERG1;RBM28;SET;S
0	0	1,588511882	3,667141063	FNBP4;RBM28;HNRNP
0	0	1,875468867	4,327695658	PCMT1;SF3B3;DDI2;TM
0	0	2,01873385	4,645971938	PCMT1;CDC37;RUVBL
0	0	2,01873385	4,645971938	PCMT1;CPSF7;HNRNF
0	0	1,630080417	3,733599175	OTUD4;HNRNPK;SF3B
0	0	1,261410027	2,885964604	TCERG1;SET;SF3B3;H
0	0	1,35998912	3,109668725	OTUD4;MAP2K1;PSMD
0	0	1,68520391	3,843231336	SART3;RBBP5;XRCC5;
0	0	1,396005781	3,15853286	KANK2;TNKS;HNRNPU
0	0	2,214839424	5,006458647	XPO4;XRCC5;DLST;HN
0	0	1,298649405	2,926551666	KANK2;SF3B3;HNRNPU
0	0	1,473321547	3,319760991	PFKFB3;CRBN;HNRNF
0	0	1,500375094	3,37901223	PCMT1;HNRNPK;SF3B
0	0	1,272199888	2,86181913	RBM28;SET;SF3B3;HN
0	0	1,40944327	3,169301126	MAP2K1;CPSF7;PSMD
0	0	1,264789882	2,827953115	SF3B3;AGAP2;HNRNP
0	0	1,350511844	3,019098437	TPM3;HNRNPU;LRRC4
0	0	1,739216855	3,882271095	HNRNPK;CRBN;USP1;
0	0	1,562890723	3,482969712	PPP2CA;IGBP1;PCMT1
0	0	1,562890723	3,482969712	PCMT1;HNRNPK;SF3B
0	0	1,318746846	2,937764713	FNBP4;USP15;LUZP1;H
0	0	1,464755724	3,251906792	SEPT10;CPSF7;SF3B3
0	0	1,522702104	3,379505932	MAP2K1;DIAPH2;PSMD
0	0	1,418037436	3,128906882	FNBP4;DOCK5;WDR37
0	0	1,600170685	3,528649726	PCMT1;JPH3;ZNF292;N
0	0	1,600170685	3,528649726	MAP2K1;MARCKS;FAM
0	0	1,600170685	3,528649726	PCMT1;HNRNPK;RBBF
0	0	1,367989056	3,005567459	NAPA;SET;SF3B3;HNR
0	0	1,435544071	3,151831652	HNRNPU;PDHB;CDC73
0	0	1,550387597	3,394637314	PCMT1;SEPT10;HNRN
0	0	1,649348507	3,609557658	PCMT1;NECAP2;HNRN
0	0	1,481262035	3,233305376	PCMT1;NPAT;HSPH1;H
0	0	1,481262035	3,233305376	HNRNPK;XPO4;RUVBL
0	0	1,481262035	3,233305376	USP13;C20ORF112;MA
0	0	1,32998936	2,902954563	DOCK5;CPSF7;SET;SF
0	0	1,717201452	3,7471302	PCMT1;C20ORF112;HN
0	0	1,717201452	3,7471302	ENAH;DOCK5;HNRNPK
0	0	2,153316107	4,698469725	HNRNPU;DLST;HNRNF
0	0	1,378482696	3,004035009	RBM28;SEPT10;DFFA;Z
0	0	1,937984496	4,205592161	PCMT1;RUVBL1;GFPT
0	0	1,471886959	3,162918566	PCMT1;SEPT10;SEPT1
0	0	1,404336591	3,015750225	KANK2;CRBN;HNRNPU
0	0	1,501255596	3,223271669	PCMT1;DDX19B;HNRN
0	0	1,631986944	3,498696225	SET;HNRNPK;RBBP5;F
0	0	1,788908766	3,824347528	FNBP4;HNRNPK;XPO4
0	0	1,695736434	3,617326474	SF3B3;RBBP5;HNRNPU
0	0	1,367043006	2,905190785	CREBBP;CPSF7;ZNF29
0	0	1,462629808	3,094120878	HNRNPK;TBL1XR1;RU
0	0	1,525972044	3,225092576	PCMT1;ALDH6A1;HNR
0	0	1,525972044	3,225092576	FNBP4;PCMT1;ITCH;H
0	0	1,8999848	4,004416356	DIAPH2;DLST;GAPVD1
0	0	1,61498708	3,391605274	FNBP4;MEF2A;PCMT1

0	0	1,674801416	3,492608882	KAT2B;SF3B3;HNRNPK
0	0	1,761804087	3,672037086	HNRNPK;XPO4;SP1;HN
0	0	1,55730897	3,243896991	ENAH;PCMT1;XPO4;LU
0	0	1,55730897	3,243896991	FNBP4;CAPZB;CAPZA
0	0	1,480404823	3,074623618	C20ORF112;HNRNPK;F
0	0	1,405791862	2,916055061	HNRNPU;LRRC40;GIG
0	0	1,423378443	2,93336746	SRPK2;SEPT10;HNRNI
0	0	1,281312064	2,630856099	FNBP4;HDAC5;SET;SF
0	0	1,470195135	3,003014269	FNBP4;SF3B3;LUZP1;F
0	0	1,654377009	3,372740619	C20ORF112;VPS13C;H
0	0	1,654377009	3,372740619	LETM1;WDR37;ST13;D
0	0	1,654377009	3,372740619	FNBP4;HNRNPK;RUVE
0	0	1,654377009	3,372740619	DDX19B;HNRNPK;TBL
0	0	1,735508504	3,526740947	TBL1XR1;RBBP5;XRC(
0	0	1,415381935	2,873586613	CPSF7;SEPT11;SF3B3
0	0	1,291989664	2,613566642	FNBP4;CPSF7;SET;SF;
0	0	1,391373484	2,804925613	SF3B3;MSL2;HNRNPU
0	0	1,58202816	3,188141129	EOMES;HNRNPK;SP1;
0	0	1,58202816	3,188141129	PSMD11;HNRNPK;RUV
0	0	1,490757305	2,987499969	HNRNPK;SF3B3;XRCC
0	0	1,490757305	2,987499969	FNBP4;HNRNPK;TBL1X
0	0	1,247438296	2,496978267	SF3B3;HNRNPU;DLST;
0	0	1,407474774	2,815045552	CREBBP;HNRNPU;HNI
0	0	1,634444756	3,257498161	PCMT1;SEPT10;KIAA1
0	0	1,634444756	3,257498161	EPS8;NCKAP1;CPSF7;
0	0	1,634444756	3,257498161	SF3B3;MTMR3;SET;TB
0	0	1,82828726	3,635692848	RUVBL1;CCDC6;HNRN
0	0	1,82828726	3,635692848	MAPK10;RUVBL1;HNR
0	0	1,82828726	3,635692848	CAPZB;XPO4;SH3KBP
0	0	1,426737052	2,834040257	MEF2A;CREBBP;MED1
0	0	1,426737052	2,834040257	SNX1;WDR37;TBL1XR
0	0	1,244577199	2,467783796	DCTN2;HNRNPU;RSF1
0	0	1,220486419	2,416745967	SET;SF3B3;PSMD11;IR
0	0	1,284206594	2,541909846	SEPT11;SF3B3;NUFIP2
0	0	1,300660736	2,564410986	HNRNPU;HNRNPR;SM
0	0	1,399655469	2,757716183	ABCA1;USP13;WDR37
0	0	1,399655469	2,757716183	HDAC5;DCTN2;HNRNF
0	0	1,280350118	2,506776044	FNBP4;MAP2K1;SET;TI
0	0	1,328457114	2,58366355	FNBP4;SRPK2;SYT5;C
0	0	1,440393882	2,798460107	MEF2A;PCMT1;DDX19
0	0	1,440393882	2,798460107	FNBP4;HNRNPK;SF3B
0	0	1,391922566	2,701571153	FNBP4;RBM28;HNRNP
0	0	1,255171306	2,432012142	RBM28;SF3B3;SH3KBF
0	0	1,550387597	2,998047165	MEF2A;HNRNPK;HNRN
0	0	1,68520391	3,25566162	CPSF7;HNRNPK;TRPS
0	0	1,352082207	2,602530706	PHC2;HNRNPU;PPWD
0	0	1,40944327	2,712550671	HNRNPK;XPO4;DCTN2
0	0	1,430726809	2,733546602	MED14;HNRNPK;SF3B
0	0	1,233262861	2,354069499	SF3B3;PSMD11;HSPA4
0	0	1,595987232	3,040057723	ABCA1;MAF;CAPZB;PH
0	0	1,296538923	2,463698256	FNBP4;UTP15;SF3B3;(
0	0	1,296538923	2,463698256	FNBP4;RBM28;SET;SF
0	0	1,937984496	3,68046246	DIRAS2;HNRNPU;DLS

0	0	1,535037225	2,907692919	CCDC112;HNRNPK;XR
0	0	1,535037225	2,907692919	SEPT10;ZZZ3;HNRNPU
0	0	1,253682693	2,369225083	IGBP1;MAP2K1;SET;SF
0	0	1,275789167	2,408766891	SEPT10;SF3B3;CRBN;2
0	0	1,661129568	3,129176589	KANK2;SF3B3;CDC37;(
0	0	1,661129568	3,129176589	RBM28;PCMT1;SART3
0	0	1,283433441	2,41586104	ZNF462;SEPT10;CPSF
0	0	1,42118863	2,67019564	PCMT1;HNRNPK;TNKS
0	0	1,339620619	2,508332489	RBM28;CRBN;DYRK1A
0	0	1,271802326	2,374241346	RBM28;SET;SF3B3;CR
0	0	1,446257087	2,69918108	HNRNPK;SP1;HNRNPU
0	0	1,39256371	2,596394037	FNBP4;CAPZB;TPM3;T
0	0	1,577429241	2,937471473	KAT2B;SF3B3;ZZZ3;AT
0	0	1,577429241	2,937471473	FNBP4;LETM1;HNRNP
0	0	1,23119015	2,292329331	RBM28;SET;SF3B3;HN
0	0	1,411776785	2,608365451	NPAT;PSMD11;RBL1;SU
0	0	1,267840325	2,340180835	NCOA2;CPSF7;SET;SF
0	0	1,637733377	3,008298221	CCND3;PRUNE2;HNRN
0	0	1,637733377	3,008298221	FRMPD4;CAPRIN1;G3E
0	0	1,890716582	3,470039912	PCMT1;HNRNPU;DLST
0	0	1,38427464	2,540234926	PPP2CA;HNRNPK;SF3
0	0	1,38427464	2,540234926	NPAT;RBM28;SF3B3;C/
0	0	1,361826943	2,488312024	NCKAP1;EIF2B2;CRBN
0	0	1,361826943	2,488312024	SF3B3;ESCO1;HNRNP
0	0	1,7303433	3,155624087	SET;HNRNPK;HNRNPU
0	0	1,46570256	2,671564559	PSMD11;HSPH1;HNRN
0	0	1,46570256	2,671564559	PCMT1;CAPZB;CAPZA
0	0	1,46570256	2,671564559	HNRNPK;TBL1XR1;SP
0	0	1,313887794	2,393698936	SEPT10;CPSF7;XRCC5
0	0	1,55929787	2,838733042	TTC9;CPSF7;ITCH;SF3
0	0	1,505230677	2,735778823	PPP2CA;PCMT1;HNRN
0	0	1,278531438	2,315462408	FNBP4;SEPT10;DFFA;S
0	0	1,376083666	2,485314094	SEPT10;SEPT11;HNRN
0	0	1,4249886	2,566158088	RBM28;HNRNPK;KIF5C
0	0	1,4249886	2,566158088	PPP2CA;HNRNPK;SIPA
0	0	1,336541032	2,401260164	FNBP4;CRBN;HNRNPU
0	0	1,266656533	2,275478817	SHC2;SET;SF3B3;DCTI
0	0	1,321353066	2,373309012	HNRNPU;DLST;SMC1A
0	0	1,206214417	2,162160962	OTUD4;SF3B3;HNRNP
0	0	1,61498708	2,892730146	CLCN5;HSDL1;HNRNP
0	0	1,274107454	2,279751465	XRCC5;CSNK1A1;HNR
0	0	1,274107454	2,279751465	FNBP4;SEPT10;SET;SF
0	0	1,453488372	2,599073058	PCMT1;HNRNPK;CRBN
0	0	1,21395002	2,166395153	RBM28;SET;SF3B3;HN
0	0	1,241605666	2,213647288	FNBP4;MAP2K1;SF3B3
0	0	1,490757305	2,653998547	RBM28;HNRNPK;XPO4
0	0	1,541578576	2,743673332	CPSF7;HNRNPK;RUVE
0	0	1,291989664	2,296837412	FNBP4;SET;SF3B3;XR(
0	0	1,367989056	2,43160173	SF3B3;MYO1C;RBBP5;
0	0	1,84569952	3,274194731	USP37;WDR37;CRBN;(
0	0	1,199152083	2,125536463	SET;HNRNPU;GIGYF2;
0	0	1,6999864	3,012538338	PCMT1;USP9X;RUVBL
0	0	1,414587223	2,502241387	OTUD4;PCMT1;PCYT1

0	0	1,414587223	2,502241387	PCMT1;HNRNPK;MYO
0	0	1,26971398	2,244563727	SF3B3;CRBN;HNRNPU
0	0	1,38427464	2,431601156	PCMT1;MAP2K1;DDX1
0	0	1,38427464	2,431601156	OTUD4;HNRNPK;SF3B
0	0	1,297395479	2,270595691	DCTN2;XRCC5;HNRNF
0	0	1,35998912	2,379068859	TCERG1;HNRNPK;XRC
0	0	1,592863969	2,782194858	HNRNPK;RUVBL1;HNF
0	0	1,340095662	2,339565086	SF3B3;HNRNPU;GAPV
0	0	1,476559616	2,574864063	PCMT1;XRCC5;RUVBL
0	0	1,404336591	2,439987384	MEF2A;ENAH;SHC2;HI
0	0	1,254358897	2,175278644	NCKAP1;RBM28;WDR3
0	0	1,239408689	2,149082129	FNBP4;IGBP1;MAP2K1
0	0	1,375343836	2,375461808	DFFA;HSPH1;HNRNPK
0	0	1,261017309	2,175726921	FNBP4;SET;SF3B3;XR
0	0	1,317076842	2,265284077	SET;XRCC5;HNRNPU;I
0	0	1,317076842	2,265284077	OTUD4;RBM28;HSPA4I
0	0	1,802776275	3,091681929	FAM101B;PSD4;RAVEF
0	0	1,39423345	2,379347858	CPSF7;HNRNPK;CRBN
0	0	1,240782757	2,114262675	FNBP4;NCKAP1;RBM2
0	0	1,571338781	2,676432288	OTUD4;TBL1XR1;RBBI
0	0	1,507321275	2,563957584	HNRNPK;XPO4;KLHL7
0	0	1,507321275	2,563957584	XPO4;KIF5C;RUVBL1;I
0	0	1,220777635	2,07593095	MAP2K1;SET;SF3B3;XI
0	0	1,297757475	2,204335373	SET;SF3B3;HNRNPU;H
0	0	1,344266703	2,277429696	ABCA1;CCND3;HNRNF
0	0	1,325989392	2,245386325	WDR37;GATA6;HNRNF
0	0	1,165941892	1,954421696	RBM28;SF3B3;PFKFB3
0	0	1,291989664	2,163968346	SF3B3;USP9X;YLPM1;I
0	0	1,242297754	2,079236679	MAP2K1;SET;PSMD11;
0	0	1,242297754	2,079236679	SET;SF3B3;HNRNPU;H
0	0	1,319047039	2,199727794	TPM3;TFEB;HNRNPU;/
0	0	1,267143709	2,111041225	XRCC5;HNRNPU;ARID
0	0	1,550387597	2,575198513	UTP15;SEPT10;HNRNI
0	0	1,276039174	2,115984017	FNBP4;SEPT10;SF3B3
0	0	1,286272896	2,124323824	MAP2K1;HNRNPU;LRF
0	0	1,374457089	2,262727479	CREBBP;HNRNPK;XPC
0	0	1,374457089	2,262727479	FNBP4;HNRNPK;SF3B
0	0	1,298171433	2,136931519	SF3B3;XRCC5;HNRNP
0	0	1,243976535	2,043998432	SEPT10;MAP2K1;HNRI
0	0	1,312177003	2,154995576	CPSF7;SET;HNRNPU;H
0	0	1,435544071	2,352374132	RBM28;ACAP2;ROCK1
0	0	1,270809506	2,07895331	FNBP4;PCYT1B;CPSF
0	0	1,214540692	1,984309968	FNBP4;FYTTD1;RBM2
0	0	1,474553421	2,397139599	USP2;HNRNPU;PTK2B
0	0	1,52998776	2,478264567	FUBP1;HNRNPU;HNRI
0	0	1,52998776	2,478264567	MAML1;TBL1XR1;CEP
0	0	1,321353066	2,133137707	HNRNPK;MYO1C;CAP
0	0	1,340743991	2,163869603	FNBP4;MED14;HNRNP
0	0	1,265622528	2,04254888	USP15;SET;DCTN2;HN
0	0	1,937984496	3,118786362	CEP170;PGAM5;GATA
0	0	1,222152385	1,96553382	UTP15;SEPT10;MAP2K
0	0	1,2749898	2,047148568	CPSF7;HNRNPU;LRRC
0	0	1,422373942	2,282882352	KANK2;TNKS;RUVBL1;

0	0	1,722652885	2,762303	ATXN1L;HNRNPU;HNR
0	0	1,722652885	2,762303	PCMT1;HNRNPK;XRC(
0	0	1,235627699	1,980991375	MAP2K1;SF3B3;DCTN2
0	0	1,252689598	2,006579969	DCTN2;HNRNPU;MTSS
0	0	1,38427464	2,205733118	MED14;HNRNPK;RBBF
0	0	1,38427464	2,205733118	PCMT1;HNRNPK;CDKI
0	0	1,298649405	2,068230392	HNRNPU;HNRNPR;HN
0	0	1,298649405	2,068230392	SET;CRBN;DCTN2;HNF
0	0	1,458698008	2,318229682	PSMD11;HNRNPK;SP1
0	0	1,458698008	2,318229682	FNBP4;DFFA;HNRNPK
0	0	1,18652112	1,883851041	SET;SF3B3;ATL3;DCTN
0	0	1,332364341	2,114047494	PCMT1;SF3B3;TMOD3
0	0	1,332364341	2,114047494	HNRNPK;CAPZB;XRC(
0	0	1,332364341	2,114047494	SET;HNRNPK;TBL1XR
0	0	1,269422159	2,009591678	CPSF7;MTMR3;HNRNF
0	0	1,124723145	1,778026916	RBM28;HNRNPU;HNRI
0	0	1,588511882	2,511148135	HNRNPK;SP1;HNRNPL
0	0	1,588511882	2,511148135	CAPZB;SH3KBP1;CAP.
0	0	1,279801082	2,015751708	FNBP4;XRCC5;DYRK1.
0	0	1,279801082	2,015751708	FNBP4;RBM28;SET;SF
0	0	1,279801082	2,015751708	SF3B3;ST13;HNRNPU;
0	0	1,279801082	2,015751708	CRBN;HNRNPU;SMC1.
0	0	1,37337484	2,14653236	ZZZ3;IGSF3;KIF5C;XR(
0	0	1,243235337	1,939704915	FNBP4;ABCA1;SET;SF
0	0	1,218858174	1,898945502	FNBP4;RBM28;CBX5;P
0	0	1,443179944	2,242152592	HNRNPK;APC;RUVBL1
0	0	1,273792627	1,976878865	TCERG1;CSNK1A1;HN
0	0	1,68520391	2,613531239	PCMT1;HNRNPK;HNRI
0	0	1,68520391	2,613531239	FNBP4;PCMT1;HNRNF
0	0	1,68520391	2,613531239	FNBP4;SF3B3;DLST;TA
0	0	1,204397144	1,865590981	RBM28;CPSF7;MTMR3
0	0	1,28539788	1,984936281	FNBP4;CPSF7;HNRNP
0	0	1,28539788	1,984936281	HNRNPU;HNRNPR;NU
0	0	1,875468867	2,895132655	PCMT1;CDH2;FAM129.
0	0	1,875468867	2,895132655	HNRNPU;AFF4;AFF1
0	0	1,875468867	2,895132655	PCMT1;HNRNPK;RUVE
0	0	1,875468867	2,895132655	KANK2;SF3B3;DLST
0	0	1,490757305	2,296448678	HNRNPK;CRBN;RUVBI
0	0	1,490757305	2,296448678	OTUD4;NPAT;CAPRIN1
0	0	1,490757305	2,296448678	HNRNPK;TRIM24;HNR
0	0	1,23856152	1,907070148	FYTTD1;RBM28;PCYT
0	0	1,396745583	2,150406805	SF3B3;RUVBL1;HNRNI
0	0	1,396745583	2,150406805	FNBP4;PCMT1;DOCK5
0	0	1,562890723	2,401392711	ABCA2;HNRNPK;HNRI
0	0	1,228300033	1,881832013	FNBP4;SF3B3;PSMD11
0	0	1,195003845	1,826858956	FNBP4;FYTTD1;RBM2
0	0	1,223990208	1,851356608	PHC2;SF3B3;HNRNPU
0	0	1,307842298	1,971469124	RBM28;C20ORF112;DD
0	0	1,307842298	1,971469124	RUVBL1;ZBTB10;HNRI
0	0	1,307842298	1,971469124	MEF2A;HNRNPK;NFIA;
0	0	1,327386641	1,99635415	RUVBL1;HNRNPU;HNF
0	0	1,327386641	1,99635415	HNRNPK;SF3B3;RUVB
0	0	1,352082207	2,03307165	KANK2;MARCKS;WDR

0	0	1,471886959	2,211174279	FNBP4;XPO4;XRCC5;F
0	0	1,272414063	1,904966254	MAP2K1;HNRNPU;HNF
0	0	1,188630491	1,776971359	FNBP4;MAP2K1;CPSF
0	0	1,538082933	2,297125709	OTUD4;CRBN;RUVBL1
0	0	1,538082933	2,297125709	KCMF1;PCMT1;HNRNF
0	0	1,538082933	2,297125709	SET;CRBN;HNRNPU;A
0	0	1,2848516	1,912781429	FNBP4;PCMT1;HNRNF
0	0	1,235368603	1,836677487	FNBP4;SRPK2;SF3B3;
0	0	1,816860465	2,691293478	PCMT1;LUZP1;CEP17C
0	0	1,175961466	1,737361494	SF3B3;PRUNE2;HNRN
0	0	1,372024422	2,026100182	HNRNPK;RBBP5;RUVE
0	0	1,372024422	2,026100182	C1QBP;HNRNPU;DLST
0	0	1,341681574	1,978709295	UTP15;HNRNPK;ZNF3
0	0	1,341681574	1,978709295	HNRNPK;CAPZB;APC;
0	0	1,230466347	1,804362974	SET;SF3B3;ATP8A2;YT
0	0	1,277791975	1,871523381	PCMT1;HNRNPK;CAPF
0	0	1,193480188	1,74760921	RBM28;SF3B3;XRCC5;
0	0	1,250312578	1,828595135	MAP2K1;USP15;HNRN
0	0	1,169085775	1,709492826	SET;SF3B3;HSPA4L;HN
0	0	1,220212461	1,781682413	RBM28;SF3B3;XRCC5;
0	0	1,291989664	1,881870271	FNBP4;CREBBP;DIAPH
0	0	1,237011381	1,797898969	MEF2A;WDR37;SF3B3;
0	0	1,514050388	2,198020285	PCMT1;XRCC5;DIRAS
0	0	1,398545513	2,029774844	CUL3;RUVBL1;GFPT1;
0	0	1,398545513	2,029774844	OTUD4;HNRNPK;CRBN
0	0	1,61498708	2,343738185	TBL1XR1;RUVBL1;DLS
0	0	1,61498708	2,343738185	IGBP1;NOS1AP;HNRNI
0	0	1,309448984	1,899048925	PCMT1;MTMR3;HNRNI
0	0	1,309448984	1,899048925	CREBBP;HNRNPK;TBL
0	0	1,309448984	1,899048925	FNBP4;DDX19B;HNRN
0	0	1,33143973	1,925860438	PCMT1;MED14;SET;HN
0	0	1,33143973	1,925860438	FNBP4;DIAPH2;HNRNF
0	0	1,33143973	1,925860438	SF3B3;SP1;RUVBL1;PI
0	0	1,151277918	1,657731259	SET;SF3B3;PPM1H;HN
0	0	1,199404086	1,719365814	SEPT11;NECAB3;BCL1
0	0	1,231769807	1,764781734	FNBP4;MAP2K1;SET;H
0	0	1,253422808	1,790939634	RBM28;SF3B3;HSPA4L
0	0	1,435544071	2,050996245	PCMT1;NFIA;XRCC5;H
0	0	1,435544071	2,050996245	RBM28;HNRNPK;ESCC
0	0	1,38427464	1,963913075	SF3B3;SP1;TRPS1;RU
0	0	1,321353066	1,874478412	HNRNPK;SF3B3;RUVB
0	0	1,321353066	1,874478412	HNRNPK;XPO4;USP9X
0	0	1,321353066	1,874478412	FNBP4;HNRNPK;SF3B
0	0	1,321353066	1,874478412	HNRNPK;HNRNPU;HN
0	0	1,348163128	1,909396264	SHC2;HNRNPK;CAPZE
0	0	1,164654144	1,648176566	RBM28;SF3B3;HNRNP
0	0	1,188947544	1,681160903	RBM28;SF3B3;CRBN;H
0	0	1,188947544	1,681160903	MCTS1;TCERG1;SF3B
0	0	1,168633867	1,652401448	SET;SF3B3;XRCC5;HN
0	0	1,490757305	2,103772963	ENAH;SEPT10;HNRNP
0	0	1,490757305	2,103772963	KANK2;SF3B3;SIN3A;F
0	0	1,276516734	1,796382003	HNRNPK;TBL1XR1;FUI
0	0	1,152092694	1,620361642	FYTTD1;USP15;SET;SI

0	0	1,206803532	1,692820681	SF3B3;XRCC5;HNRNP
0	0	1,158335561	1,62377601	SET;SF3B3;PSMD11;HI
0	0	1,158335561	1,62377601	FYTTD1;RBM28;SF3B3
0	0	1,198753297	1,678050702	ST13;HNRNPU;DLST;S
0	0	1,233262861	1,724561082	MAP2K1;SF3B3;HNRNI
0	0	1,233262861	1,724561082	FNBP4;SET;SF3B3;ST1
0	0	1,291989664	1,806594761	PCMT1;HNRNPK;SART
0	0	1,185311618	1,655631394	CPSF7;PSMD11;CRBN
0	0	1,257071025	1,75299503	PPP2CA;CPSF7;HNRN
0	0	1,2214188	1,700302294	TCERG1;SEPT10;SF3E
0	0	1,31141808	1,824518227	FNBP4;HNRNPK;XPO4
0	0	1,336541032	1,853731026	HNRNPK;FUBP1;RUVE
0	0	1,336541032	1,853731026	PCMT1;TSFM;DDX19B
0	0	1,336541032	1,853731026	PCMT1;SF3B3;XRCC5;
0	0	1,370292068	1,900352498	PCMT1;MED14;ADCY9
0	0	1,370292068	1,900352498	PCMT1;HNRNPK;SF3B
0	0	1,241073815	1,71868217	FYTTD1;TPM3;TMOD3
0	0	1,241073815	1,71868217	SF3B3;CRBN;XRCC5;H
0	0	1,26891842	1,755115662	FYTTD1;HNRNPK;CAP
0	0	1,153034254	1,582378393	DOCK5;PSMD11;HNRN
0	0	1,468170073	2,014101658	CARM1;RUVBL1;HNRN
0	0	1,468170073	2,014101658	ACAP2;HNRNPK;RUVE
0	0	1,70998632	2,334638367	RCAN1;VPS13C;CALM
0	0	1,172843643	1,599047968	FNBP4;CPSF7;SF3B3;I
0	0	1,183977666	1,613783739	XRCC5;TPM3;HNRNPU
0	0	1,178106077	1,60564515	CPSF7;SET;SF3B3;TNI
0	0	1,400952648	1,903568527	HNRNPK;RUVBL1;HNF
0	0	1,550387597	2,106369756	TPM3;ACTN4;TARDBP
0	0	1,325117604	1,79977465	TIAL1;MEF2A;SF3B3;H
0	0	1,356589147	1,839001571	HNRNPK;ZZZ3;RUVBL
0	0	1,356589147	1,839001571	PCMT1;HNRNPK;EEF2
0	0	1,18652112	1,597229073	MEF2A;SF3B3;KLK13;I
0	0	1,253989968	1,675417515	HSPH1;RXRA;KIF5C;XI
0	0	1,446257087	1,928743903	SEPT10;HNRNPU;GAB
0	0	1,446257087	1,928743903	PCMT1;RUVBL1;HNRN
0	0	1,166122476	1,552168158	FNBP4;SEPT10;SET;XI
0	0	1,313887794	1,747468533	RBM28;SIN3A;HNRNPU
0	0	1,313887794	1,747468533	HNRNPK;SF3B3;RUVB
0	0	1,313887794	1,747468533	PCMT1;TPM3;ST13;SC
0	0	1,38427464	1,834265832	FNBP4;HNRNPK;RUVE
0	0	1,343157572	1,77977304	PCMT1;HNRNPK;TPM3
0	0	1,343157572	1,77977304	MEF2A;CPSF7;XPO4;F
0	0	1,266656533	1,676439128	MED14;HNRNPK;ROCI
0	0	1,266656533	1,676439128	PCMT1;HNRNPK;TBL1
0	0	1,16746054	1,533274911	UTP15;NCOA2;SF3B3;I
0	0	1,246656693	1,636939333	KCMF1;PCMT1;SET;HI
0	0	1,661129568	2,178291625	HNRNPU;RC3H1;GPR7
0	0	1,661129568	2,178291625	RUVBL1;AFF4;AFF1
0	0	1,185098433	1,553963187	FNBP4;SET;SF3B3;PSI
0	0	1,185098433	1,553963187	RBM28;USP15;SF3B3;
0	0	1,230466347	1,606473129	PCMT1;CAPZB;SH3KB
0	0	1,230466347	1,606473129	PCMT1;SEPT10;MAP2I
0	0	1,30284672	1,69675642	HNRNPK;SP1;KCNC2;I

0	0	1,258431491	1,635205209	DIAPH2;HNRNPK;RUV
0	0	1,4249886	1,847455232	HNRNPK;C1QBP;HNRI
0	0	1,32998936	1,722583711	RUVBL1;EIF3J;HNRNP
0	0	1,32998936	1,722583711	FNBP4;HNRNPK;TBL1X
0	0	1,32998936	1,722583711	FNBP4;NCKAP1;PCMT
0	0	1,32998936	1,722583711	SF3B3;CRBN;RUVBL1;
0	0	1,174536058	1,520063397	SEPT10;SEPT11;SET;S
0	0	1,188036473	1,536958372	FNBP4;SF3B3;XRCC5;
0	0	1,367989056	1,767722842	FCHSD2;HNRNPK;RBE
0	0	1,367989056	1,767722842	RUVBL1;HNRNPU;ANK
0	0	1,13504515	1,465593828	SF3B3;SH3KBP1;HNRI
0	0	1,239408689	1,599345246	PCMT1;HNRNPK;SART
0	0	1,239408689	1,599345246	PCMT1;HNRNPK;SP1;U
0	0	1,273128501	1,638050834	DOCK5;NXF1;HNRNPK
0	0	1,273128501	1,638050834	FNBP4;HNRNPK;XRCC
0	0	1,273128501	1,638050834	HNRNPK;ROCK1;TPM3
0	0	1,200521369	1,533447223	MAP2K1;HNRNPU;PAR
0	0	1,250312578	1,594998777	DDX19B;HNRNPK;TBL
0	0	1,250312578	1,594998777	EOMES;KANK2;PCMT1
0	0	1,250312578	1,594998777	PSMD11;HNRNPK;WDI
0	0	1,291989664	1,647584292	MAP3K2;CAPZB;TBL1X
0	0	1,490757305	1,896780496	MED14;CREB1;XRCC5
0	0	1,490757305	1,896780496	HNRNPK;RUVBL1;HNF
0	0	1,232244477	1,562613659	NPAT;RBM28;CPSF7;H
0	0	1,317076842	1,667354219	HNRNPK;CRBN;KIF5C;
0	0	1,317076842	1,667354219	ABCA1;PCMT1;HNRNF
0	0	1,217581882	1,537820361	ENAH;SET;SF3B3;XPO
0	0	1,217581882	1,537820361	HSPH1;HNRNPK;SART
0	0	1,263902932	1,594578858	USP15;HNRNPK;SART
0	0	1,404336591	1,770007728	RUVBL1;HNRNPU;HNF
0	0	1,404336591	1,770007728	PCMT1;HNRNPU;HNRI
0	0	1,404336591	1,770007728	PCMT1;HNRNPK;HNRI
0	0	1,404336591	1,770007728	HNRNPK;HNRNPU;DLS
0	0	1,404336591	1,770007728	FNBP4;WDR37;HNRNF
0	0	1,352082207	1,703813049	HNRNPK;RUVBL1;HNF
0	0	1,352082207	1,703813049	XPO4;ESCO1;RUVBL1
0	0	1,352082207	1,703813049	HNRNPK;XRCC5;RUVE
0	0	1,61498708	2,034615246	E2F1;HNRNPU;APCDD
0	0	1,61498708	2,034615246	HNRNPK;RUVBL1;MAC
0	0	1,114172271	1,401661525	FYTTD1;SET;SF3B3;HN
0	0	1,14965182	1,440424789	KANK2;SEPT10;MAP2K
0	0	1,242297754	1,555792276	PCMT1;ACAP2;HNRNF
0	0	1,281312064	1,599900265	SF3B3;TBL1XR1;RUVB
0	0	1,162790698	1,446390523	TDRKH;FNBP4;SRPK2
0	0	1,21124031	1,5045864	FNBP4;HNRNPK;CAPZ
0	0	1,199704688	1,48756339	RBM28;SF3B3;HNRNP
0	0	1,304412642	1,614008827	FNBP4;SUB1;RUVBL1;
0	0	1,304412642	1,614008827	KAT2B;HNRNPK;ST13;
0	0	1,254810105	1,552290228	HNRNPK;SF3B3;MAT2A
0	0	1,254810105	1,552290228	HNRNPK;HNRNPU;LRF
0	0	1,106516433	1,366152838	RBM28;SET;SF3B3;HN
0	0	1,234385029	1,517558993	CRBN;XRCC5;RBBP5;F
0	0	1,336541032	1,642416805	ABCA1;WDR37;HNRNF

0	0	1,38427464	1,696188701	PCMT1;SP1;ZNF318;HI
0	0	1,38427464	1,696188701	HNRNPK;RUVBL1;HNF
0	0	1,164160298	1,425884528	KANK2;ARHGEF10;CS
0	0	1,218161683	1,491654589	MEF2A;TCERG1;DIAPH
0	0	1,218161683	1,491654589	CCDC112;PPP2CA;SF3
0	0	1,270809506	1,553654487	PCMT1;HNRNPK;RFWI
0	0	1,184794015	1,445731177	NCKAP1;NUFIP2;HNRN
0	0	1,155090097	1,399138725	SF3B3;CRBN;MAML1;C
0	0	1,571338781	1,902350018	RUVBL1;HNRNPR;TAR
0	0	1,571338781	1,902350018	RABGAP1L;SET;RUVBI
0	0	1,291989664	1,562475228	SET;HNRNPK;SF3B3;K
0	0	1,13999088	1,376960461	FNBP4;MAP2K1;DCTN
0	0	1,198753297	1,440222923	DFFA;HNRNPK;RRM2E
0	0	1,321353066	1,583420887	PCMT1;HNRNPK;TPM3
0	0	1,321353066	1,583420887	CPSF7;HNRNPU;HNRN
0	0	1,155990752	1,37748293	SEPT10;XRCC5;HNRN
0	0	1,364777814	1,62579949	DDX19B;HNRNPK;RUV
0	0	1,237011381	1,47112395	CAPZB;SH3KBP1;CAPI
0	0	1,096972356	1,299920072	TCERG1;RBM28;SET;S
0	0	1,218858174	1,443909289	ARHGEF10;SH3PXD2A
0	0	1,279801082	1,512684362	PCMT1;HNRNPK;RUVE
0	0	1,279801082	1,512684362	KANK2;MAP2K1;SF3B3
0	0	1,123469273	1,32691295	RBM28;SF3B3;CRBN;T
0	0	1,136647798	1,333792144	MAP2K1;SHC2;HNRNF
0	0	1,250312578	1,465287873	HNRNPK;ATXN1L;IGF2
0	0	1,157005669	1,355456502	FNBP4;CPSF7;SF3B3;
0	0	1,127927485	1,319350238	PCYT1B;SET;XRCC5;H
0	0	1,306506402	1,526718088	HNRNPK;TPM3;RUVBL
0	0	1,162790698	1,35834694	SRPK2;RBM28;NEK7;H
0	0	1,228300033	1,432179716	DIAPH2;PSMD11;HNRN
0	0	1,52998776	1,780387798	PCMT1;HNRNPK;RUVE
0	0	1,52998776	1,780387798	PCMT1;HNRNPU;DIP2I
0	0	1,52998776	1,780387798	KANK2;DLST;ZNF687
0	0	1,21124031	1,408443407	FNBP4;CREBBP;HNRN
0	0	1,18652112	1,37856282	HNRNPK;ZZZ3;SF3B3;
0	0	1,197630868	1,391178343	PSMD11;HNRNPK;CAF
0	0	1,197630868	1,391178343	FNBP4;PCMT1;HNRNF
0	0	1,147935067	1,330622529	CPSF7;SF3B3;HNRNPU
0	0	1,345822567	1,558654372	KLF10;CPSF7;RUVBL1
0	0	1,345822567	1,558654372	ENAH;PCMT1;SP1;HNI
0	0	1,345822567	1,558654372	SET;HNRNPU;MLEC;P
0	0	1,267840325	1,464570245	DFFA;HNRNPK;G3BP1
0	0	1,152704533	1,331243614	CPSF7;SET;HNRNPU;S
0	0	1,40944327	1,62622342	PCMT1;SUB1;RUVBL1;
0	0	1,40944327	1,62622342	HNRNPK;CSNK1A1;CA
0	0	1,40944327	1,62622342	FUBP1;PDE3B;SORBS
0	0	1,100320868	1,266641227	FYTTD1;RBM28;SET;SI
0	0	1,164454204	1,336252297	ZNF275;SF3B3;HNRNF
0	0	1,240310078	1,423076682	FNBP4;HNRNPK;RRM2
0	0	1,219710522	1,394285561	SHC2;HNRNPK;CAPZE
0	0	1,121991024	1,281169945	SF3B3;XRCC5;HNRNP
0	0	1,203717078	1,373851769	CPSF7;DFFA;HNRNPK
0	0	1,190940193	1,359200541	SET;HNRNPK;ST13;CA

0	0	1,1535622	1,308152292	MAP2K1;SET;WDR37;C
0	0	1,1535622	1,308152292	CRBN;HNRNPU;HNRN
0	0	1,136277555	1,287211837	SEPT10;SET;XRCC5;C
0	0	1,256101062	1,418069809	SF3B3;RUVBL1;HNRNI
0	0	1,327386641	1,49457957	PCMT1;HNRNPK;TARD
0	0	1,327386641	1,49457957	RPS6KA6;HNRNPK;CR
0	0	1,129500166	1,269101161	CRBN;HNRNPU;HNRN
0	0	1,230466347	1,382122861	PCMT1;CRBN;TPM3;HI
0	0	1,230466347	1,382122861	FNBP4;PCMT1;MED14
0	0	1,230466347	1,382122861	FNBP4;HNRNPK;CAPZ
0	0	1,144197488	1,284034001	FNBP4;CPSF7;SF3B3;I
0	0	1,144197488	1,284034001	FNBP4;CPSF7;SF3B3;'
0	0	1,21124031	1,357410928	PCMT1;PCYT1B;SET;E
0	0	1,21124031	1,357410928	KIF5C;SP1;ZNF318;HN
0	0	1,196286726	1,340111462	PCMT1;HNRNPK;RUVE
0	0	1,490757305	1,667750218	HNRNPC;TARDBP;PUM
0	0	1,490757305	1,667750218	KLHL7;DLST;KPNA1
0	0	1,132588342	1,265847861	FNBP4;SF3B3;HNRNPI
0	0	1,149002666	1,283719756	SF3B3;HNRNPU;DLST;
0	0	1,154543955	1,284537383	OTUD4;CREBBP;HNRN
0	0	1,154543955	1,284537383	TPM3;ANGEL2;HSPA4I
0	0	1,136059877	1,263116616	SF3B3;HNRNPU;HNRN
0	0	1,277791975	1,419790872	PCMT1;SF3B3;HNRNP
0	0	1,161004537	1,28678832	SF3B3;STAU1;HNRNPI
0	0	1,13999088	1,261023816	FNBP4;MAP2K1;SET;S
0	0	1,168633867	1,290885397	HNRNPK;XRCC5;SIN3/
0	0	1,177780633	1,297408907	UTP15;NCKAP1;DDX1!
0	0	1,177780633	1,297408907	FNBP4;HNRNPK;SP1;F
0	0	1,220777635	1,342385416	NEURL1B;HNRNPK;SF
0	0	1,188947544	1,307200255	ENAH;HNRNPK;TBL1X
0	0	1,188947544	1,307200255	PCMT1;HNRNPK;CRBI
0	0	1,68520391	1,846162327	DOCK5;HNRNPU
0	0	1,14965182	1,259402975	MAP2K1;SF3B3;XRCC!
0	0	1,14965182	1,259402975	ARHGEF10;NUFIP2;LU
0	0	1,309448984	1,433412345	RUVBL1;NRIP1;HNRNI
0	0	1,309448984	1,433412345	HNRNPK;SIN3A;HNRN
0	0	1,309448984	1,433412345	PABPN1;RUVBL1;HNRI
0	0	1,12528132	1,224101059	RBM28;MAP2K1;SF3B3
0	0	1,162790698	1,262887293	FNBP4;HNRNPK;SP1;F
0	0	1,13999088	1,236145329	WDR37;SF3B3;XRCC5
0	0	1,263902932	1,369378871	PCMT1;SET;CRBN;HNI
0	0	1,263902932	1,369378871	HNRNPK;XPO4;RUVBL
0	0	1,263902932	1,369378871	PCMT1;HNRNPK;HSPA
0	0	1,35998912	1,470562551	UTP15;DYRK3;DPYSL3
0	0	1,35998912	1,470562551	RUVBL1;ZFP91;TARDE
0	0	1,128278645	1,219901737	MAP2K1;SET;HNRNPU
0	0	1,181697863	1,275096572	HNRNPK;SF3B3;RUVB
0	0	1,181697863	1,275096572	ABCA2;HNRNPK;XRCC
0	0	1,181697863	1,275096572	ABCA1;HNRNPK;NFIA;
0	0	1,233262861	1,32967138	PCMT1;CAPRIN1;RUVI
0	0	1,102171277	1,186840495	RBM28;SF3B3;DCTN2;
0	0	1,453488372	1,563570827	UBN2;ATXN1L;HNRNPI
0	0	1,113784193	1,195409639	SET;SF3B3;CRBN;XRC

0	0	1,113784193	1,195409639	UTP15;RBM28;SF3B3;I
0	0	1,113784193	1,195409639	SEPT10;HNRNPU;HNR
0	0	1,121663052	1,203708529	SF3B3;CRBN;ST13;HN
0	0	1,103308005	1,179684113	FYTTD1;TCERG1;RBM
0	0	1,157005669	1,235480821	FNBP4;SEPT10;MAP2K
0	0	1,157005669	1,235480821	HNRNPK;SF3B3;TBL1X
0	0	1,115809255	1,189427112	FNBP4;CPSF7;SF3B3;Y
0	0	1,291989664	1,375000179	HNRNPK;KLHL7;CEP1
0	0	1,164908714	1,238389509	FNBP4;SET;HNRNPK;X
0	0	1,13999088	1,210535174	FNBP4;SF3B3;XRCC5;
0	0	1,127554616	1,194293496	FNBP4;RBM28;SF3B3;
0	0	1,250312578	1,320884199	SRPK2;HNRNPK;CAPR
0	0	1,18652112	1,252613601	XPO4;CRBN;RUVBL1;C
0	0	1,18652112	1,252613601	MAP2K1;C1QBP;RUVB
0	0	1,105915066	1,165428742	MAP2K1;RNF180;HNRI
0	0	1,20185085	1,266403304	PCMT1;HNRNPK;SF3B
0	0	1,20185085	1,266403304	CAPZB;XRCC5;RUVBL
0	0	1,222152385	1,287660508	PCMT1;HNRNPK;SIN3A
0	0	1,336541032	1,399170972	HNRNPK;HNRNPU;HN
0	0	1,336541032	1,399170972	HNRNPK;TBL1XR1;RU
0	0	1,336541032	1,399170972	PCMT1;HNRNPK;ZNF3
0	0	1,336541032	1,399170972	PCMT1;CRBN;RUVBL1
0	0	1,16746054	1,213228632	ABCA2;MAP2K1;HSPH
0	0	1,13999088	1,184090517	FNBP4;TPM3;HNRNPU
0	0	1,109087513	1,15133626	SET;SF3B3;XRCC5;HN
0	0	1,116804625	1,157652267	SEPT10;CPSF7;USP9X
0	0	1,178504085	1,219531236	PCMT1;CPSF7;CAPRIN
0	0	1,2749898	1,319200012	HNRNPK;ATXN1L;RC3I
0	0	1,2749898	1,319200012	SEPT10;CAPZB;HNRN
0	0	1,418037436	1,467080051	HNRNPK;HNRNPU;TAF
0	0	1,192605844	1,23008408	XRCC5;USP9X;CAPZA
0	0	1,110946526	1,14439778	DOCK5;MAP2K1;HNRN
0	0	1,237011381	1,274224598	KANK2;TDG;CAPRIN1;
0	0	1,21124031	1,247037298	HNRNPK;SF3B3;KLHL7
0	0	1,21124031	1,247037298	HNRNPK;ALDH2;SP1;S
0	0	1,21124031	1,247037298	PCMT1;FUBP1;RUVBL
0	0	1,21124031	1,247037298	HNRNPK;TBL1XR1;RU
0	0	1,099937146	1,130302964	MAP2K1;SF3B3;HNRNI
0	0	1,152315106	1,18201291	RBM28;HNRNPK;SF3B
0	0	1,122384843	1,145950304	UTP15;XRCC5;SH3KBI
0	0	1,160469758	1,183424314	FNBP4;PCMT1;HNRNF
0	0	1,160469758	1,183424314	FNBP4;DCTN2;FRMPD
0	0	1,096972356	1,113101979	FNBP4;XRCC5;TPM3;H
0	0	1,125800122	1,140606263	SF3B3;HNRNPU;DEK;S
0	0	1,200521369	1,20775116	MED14;HNRNPK;RUVE
0	0	1,200521369	1,20775116	CREBBP;HNRNPK;TBL
0	0	1,258431491	1,265877555	PCMT1;WDR37;HNRNI
0	0	1,258431491	1,265877555	TNKS;HNRNPU;KCNR(
0	0	1,258431491	1,265877555	HNRNPK;KIF5C;ATXN1
0	0	1,118067979	1,124391745	FNBP4;SRPK2;RBM28;
0	0	1,129766747	1,135743106	SF3B3;SH3KBP1;HNRI
0	0	1,223990208	1,229321937	HNRNPK;KIF5C;SUB1;
0	0	1,1535622	1,154347354	DDX19B;HNRNPK;SAR

0	0	1,1535622	1,154347354	SF3B3;TBL1XR1;SIN3A
0	0	1,38427464	1,377592472	ABCA1;DLST;RAPGEF6
0	0	1,38427464	1,377592472	PCMT1;XPO4;TARDBP
0	0	1,162790698	1,155985164	PCMT1;TPM3;RBBP5;C
0	0	1,162790698	1,155985164	HNRNPK;CAPZB;DCTN
0	0	1,13999088	1,128156408	PCMT1;HNRNPK;RBBP
0	0	1,124723145	1,112285	RBM28;CRBN;HNRNPU
0	0	1,174536058	1,160613098	PCMT1;HNRNPK;SF3B
0	0	1,174536058	1,160613098	EOMES;FNBP4;HNRNP
0	0	1,174536058	1,160613098	SF3B3;LUZP1;TBL1XR
0	0	1,078963531	1,064475183	ALAS2;PFKFB3;ROCK1
0	0	1,09588409	1,07848529	FNBP4;SF3B3;CRBN;Z
0	0	1,084516871	1,06589272	CPSF7;SF3B3;HNRNPU
0	0	1,291989664	1,26789271	HNRNPK;RBBP5;RUVB
0	0	1,291989664	1,26789271	ZNF292;RUVBL1;DLST
0	0	1,128923007	1,106883853	RBM28;HNRNPK;DCTN
0	0	1,091822251	1,070244484	FNBP4;RBM28;SEPT10
0	0	1,101864095	1,079234492	RBM28;SET;SF3B3;PSI
0	0	1,21124031	1,186101968	ABCA1;ANGEL2;KLHL7
0	0	1,21124031	1,186101968	FNBP4;HNRNPK;FUBP
0	0	1,109533108	1,082414757	HNRNPU;HNRNPR;SM
0	0	1,16570496	1,127394697	HNRNPK;ROCK1;RUVB
0	0	1,16570496	1,127394697	FNBP4;RUVBL1;HNRN
0	0	1,111958317	1,074526462	FNBP4;CPSF7;SF3B3;Y
0	0	1,079264974	1,035238249	FNBP4;SEPT10;RBM28
0	0	1,352082207	1,294496013	SEPT10;HNRNPU;DLST
0	0	1,352082207	1,294496013	ITCH;SF3B3;CEP170
0	0	1,114769843	1,066752735	SET;SF3B3;CRBN;ST13
0	0	1,114769843	1,066752735	FAM114A1;C21ORF59;I
0	0	1,14749082	1,095767861	DDX19B;HNRNPK;RUV
0	0	1,198753297	1,144494099	HNRNPK;ALDH2;XRCC
0	0	1,198753297	1,144494099	FNBP4;PCMT1;GABRB
0	0	1,107419712	1,05331841	CPSF7;SF3B3;HNRNPU
0	0	1,226572466	1,166168737	HNRNPK;CRBN;TARDB
0	0	1,226572466	1,166168737	FNBP4;HNRNPK;KLHL7
0	0	1,270809506	1,207519848	RUVBL1;CEP170;TARD
0	0	1,270809506	1,207519848	PCMT1;RUVBL1;HNRN
0	0	1,270809506	1,207519848	CAPZB;DLST;RAB11A;J
0	0	1,090116279	1,033771798	MAP2K1;SF3B3;XIAP;G
0	0	1,157005669	1,095145264	HSPH1;VPS13C;CARM
0	0	1,157005669	1,095145264	SF3B3;TBL1XR1;RUVB
0	0	1,101127555	1,041914047	FNBP4;CPSF7;SET;SF3
0	0	1,101127555	1,041914047	SF3B3;HNRNPU;SPATA
0	0	1,109858963	1,044660286	KANK2;SEPT10;XRCC5
0	0	1,169473403	1,097440616	PCMT1;DCTN2;HNRNP
0	0	1,076658053	1,009806651	FNBP4;NCOA2;SF3B3;
0	0	1,076658053	1,009806651	MAP2K1;SET;CRBN;XR
0	0	1,121991024	1,051843909	PCYT1B;HNRNPK;TBL
0	0	1,13999088	1,066849362	PCMT1;HNRNPK;CRBN
0	0	1,18652112	1,104431181	PCMT1;HNRNPK;HNRN
0	0	1,18652112	1,104431181	TTC9;HNRNPK;CRBN;I
0	0	1,079657101	1,004190882	FNBP4;SEPT10;MAP2K
0	0	1,148435257	1,063834406	HNRNPK;SF3B3;TPM3

0	0	1,148435257	1,063834406	PCMT1;HNRNPK;ESC(
0	0	1,148435257	1,063834406	FNBP4;HNRNPK;SF3B:
0	0	1,087318034	1,005760106	RBM28;SET;HNRNPU;[
0	0	1,21124031	1,119552228	PPP2CA;SIN3A;HNRNI
0	0	1,21124031	1,119552228	HNRNPK;XPO4;RUVBL
0	0	1,098452751	1,01208492	CPSF7;SF3B3;PSMD11
0	0	1,098452751	1,01208492	TCERG1;CPSF7;SET;T
0	0	1,098452751	1,01208492	HNRNPU;ACTN4;GAP\
0	0	1,116116726	1,027544153	FNBP4;SET;HNRNPK;K
0	0	1,116116726	1,027544153	PCMT1;HNRNPK;CRBN
0	0	1,063528077	0,978598252	TNKS;HNRNPU;PHF8;I
0	0	1,250312578	1,150364279	FNBP4;HNRNPK;HNRN
0	0	1,250312578	1,150364279	SF3B3;NFIA;HNRNPU;I
0	0	1,250312578	1,150364279	CPSF7;HNRNPK;USP9
0	0	1,0928484	1,002838647	FNBP4;CPSF7;SF3B3;(
0	0	1,174536058	1,065849316	HNRNPK;RUVBL1;HNF
0	0	1,13999088	1,033432816	SET;HNRNPK;DCTN2;S
0	0	1,110303618	1,003791432	SEPT10;HNRNPK;SF3E
0	0	1,066355106	0,962017664	FNBP4;RBM28;SF3B3;:
0	0	1,08018808	0,970782152	SET;SF3B3;GFPT1;HNI
0	0	1,12528132	1,011282776	SEPT10;HNRNPK;SF3E
0	0	1,196286726	1,074952109	ACAP2;ROCK1;CAPRII
0	0	1,196286726	1,074952109	PCMT1;SF3B3;CAPZB;
0	0	1,14965182	1,029749353	PCMT1;DDX19B;HNRN
0	0	1,435544071	1,281742365	HNRNPU;DLST
0	0	1,113784193	0,994154306	RBM28;HNRNPK;SF3B
0	0	1,230466347	1,096226325	PCMT1;SEPT10;HNRN
0	0	1,230466347	1,096226325	HNRNPK;SNRPD3;HNF
0	0	1,230466347	1,096226325	DLST;PGAM5;HELZ;TA
0	0	1,230466347	1,096226325	HNRNPK;CRBN;RUVBI
0	0	1,230466347	1,096226325	CRBN;HSPA4L;HNRNP
0	0	1,937984496	1,721568072	CEP170
0	0	1,937984496	1,721568072	CREBBP
0	0	1,104550749	0,980573143	HNRNPK;SF3B3;RBBP
0	0	1,131669779	1,003912224	HNRNPK;SF3B3;CAPR
0	0	1,291989664	1,145340919	HNRNPK;HNRNPU;DLS
0	0	1,291989664	1,145340919	PCMT1;CRBN;DLST
0	0	1,162790698	1,028687668	HNRNPK;SIN3A;FUBP1
0	0	1,162790698	1,028687668	ENAH;MAP2K1;EEF2K;
0	0	1,162790698	1,028687668	HNRNPK;XRCC5;RUVE
0	0	1,073345259	0,949181879	XRCC5;HNRNPU;LRR(
0	0	1,096972356	0,969592867	PCMT1;MAP2K1;HNRN
0	0	1,096972356	0,969592867	HNRNPK;RBBP5;HNRN
0	0	1,096972356	0,969592867	PCMT1;HNRNPK;XPO4
0	0	1,070398414	0,94525925	SEPT10;CPSF7;SET;R(
0	0	1,067760053	0,941854122	RBM28;CSNK1A1;HNR
0	0	1,090640626	0,960577431	FNBP4;SF3B3;GAB1;SI
0	0	1,090640626	0,960577431	FNBP4;ST13;VPS13C;I
0	0	1,061277224	0,934047642	FNBP4;RBM28;CPSF7;
0	0	1,107419712	0,969676434	FNBP4;HNRNPK;SF3B:
0	0	1,107419712	0,969676434	HNRNPK;CRBN;TBL1X
0	0	1,107419712	0,969676434	SET;HNRNPK;SIN3A;H
0	0	1,13999088	0,997535571	CREBBP;HNRNPK;SF3

0	0	1,181697863	1,03226945	RUVBL1;HNRNPU;SNF
0	0	1,091822251	0,948366332	FNBP4;HNRNPK;SF3B
0	0	1,123469273	0,975245356	MTMR3;RUVBL1;HNRN
0	0	1,080947528	0,934147754	SF3B3;HNRNPU;SMC1
0	0	1,110946526	0,958604825	FNBP4;HNRNPK;RBBF
0	0	1,076658053	0,928769189	MTMR3;WDR37;ESCO
0	0	1,151277918	0,992888293	CAPZB;CAPZA1;RUVB
0	0	1,101127555	0,945791027	HNRNPK;RBBP5;HNRN
0	0	1,093222023	0,935691041	TCERG1;XRCC5;NKRF
0	0	1,130490956	0,966358918	SF3B3;RUVBL1;HNRNI
0	0	1,130490956	0,966358918	PCMT1;SF3B3;SORT1;
0	0	1,086720278	0,927586222	SET;HNRNPK;SF3B3;K
0	0	1,38427464	1,17594164	PCMT1;CDC37
0	0	1,38427464	1,17594164	DDI2;NRIP1
0	0	1,11538676	0,947405895	CYTH3;HNRNPK;KIF5C
0	0	1,16746054	0,991410994	HNRNPK;SP1;HNRNPU
0	0	1,049741602	0,891251382	SF3B3;HNRNPU;ADD3
0	0	1,094906495	0,922483281	FNBP4;HNRNPK;SF3B
0	0	1,094906495	0,922483281	HNRNPK;CAPZB;RUVE
0	0	1,08764436	0,914003581	FNBP4;HNRNPK;FUBP
0	0	1,081665765	0,907243104	FNBP4;HNRNPK;RXRA
0	0	1,051265175	0,880270572	RBM28;CPSF7;SF3B3;
0	0	1,072402488	0,897334449	HNRNPU;SMC1A;GIGY
0	0	1,062765691	0,888183151	RBM28;SEPT10;XRCC
0	0	1,192605844	0,996276626	PCMT1;DCTN2;HNRNF
0	0	1,192605844	0,996276626	HNRNPK;RUVBL1;HNF
0	0	1,107419712	0,920368436	FNBP4;HNRNPK;FAM1
0	0	1,096972356	0,908658139	PCMT1;HSPH1;HNRNF
0	0	1,096972356	0,908658139	PCMT1;HNRNPK;SP1;U
0	0	1,096972356	0,908658139	HNRNPK;SF3B3;CRBN
0	0	1,096972356	0,908658139	PCMT1;CPSF7;HNRNF
0	0	1,088755335	0,899738801	KIF5C;TBL1XR1;SP1;F
0	0	1,050139382	0,867629073	SEPT10;SET;SF3B3;XF
0	0	1,1535622	0,95228878	MEF2A;DFFA;DIAPH2;I
0	0	1,1535622	0,95228878	PCMT1;HNRNPK;CRBN
0	0	1,055063193	0,869790347	ACBD5;SET;SF3B3;HN
0	0	1,040063236	0,856759805	SET;SF3B3;LUZP1;HNI
0	0	1,07207653	0,882957564	MAP2K1;HNRNPU;GAF
0	0	1,059348438	0,872460209	MEF2A;SF3B3;CRBN;N
0	0	1,237011381	1,015866394	PCMT1;PSMD11;DLST
0	0	1,237011381	1,015866394	PCMT1;RUVBL1;HNRN
0	0	1,111958317	0,906973747	PCMT1;HNRNPK;FCHS
0	0	1,111958317	0,906973747	HNRNPK;XPO4;RUVBL
0	0	1,111958317	0,906973747	HNRNPK;SF3B3;NSD1
0	0	1,111958317	0,906973747	HNRNPK;MAT2A;C21O
0	0	1,082672903	0,877543589	PCMT1;HNRNPK;SIN3A
0	0	1,076658053	0,872078935	HNRNPK;CRBN;DCTN2
0	0	1,071696496	0,867831191	USP13;HNRNPK;XPO4
0	0	1,071696496	0,867831191	HNRNPK;SF3B3;SART
0	0	1,174536058	0,950133936	HNRNPK;SP1;FUBP1;T
0	0	1,174536058	0,950133936	ITCH;EIF3J;HNRNPU;H
0	0	1,174536058	0,950133936	HNRNPK;HNRNPU;CEI
0	0	1,174536058	0,950133936	DFFA;CNOT6;CAPZB;C

0	0	1,174536058	0,950133936	HNRNPK;RUVBL1;HNF
0	0	1,336541032	1,080741454	HNRNPK;RUVBL1
0	0	1,13999088	0,914819788	PCMT1;HNRNPK;HNRI
0	0	1,118067979	0,893124447	ACAP2;HNRNPK;RUVE
0	0	1,118067979	0,893124447	HNRNPK;FUBP1;RUVE
0	0	1,057082452	0,84336242	HDAC5;MAP2K1;CPSF
0	0	1,057082452	0,84336242	FNBP4;CPSF7;SEPT11
0	0	1,102918006	0,878697129	HNRNPK;XPO4;RUVBL
0	0	1,102918006	0,878697129	RAB2B;SEPT10;HNRNI
0	0	1,102918006	0,878697129	PCMT1;ARHGEF10;HN
0	0	1,06302947	0,846415636	FNBP4;RBM28;SET;SF:
0	0	1,06302947	0,846415636	SRPK2;KANK2;OTUD4
0	0	1,06302947	0,846415636	RBM28;ST13;TPM3;HN
0	0	1,091822251	0,868602281	MAP2K1;HNRNPK;KIF5
0	0	1,083345371	0,861294888	HNRNPK;SF3B3;CRBN
0	0	1,076658053	0,855884027	HSPH1;HNRNPK;TBL1.
0	0	1,043530113	0,828045628	SEPT10;MAP2K1;SF3B
0	0	1,044735577	0,827559958	FNBP4;SRPK2;CPSF7;
0	0	1,21124031	0,957535043	KIF5C;DLST;KIF1B
0	0	1,21124031	0,957535043	SF3B3;HNRNPU;DLST
0	0	1,039012667	0,816931619	MAP2K1;CPSF7;SF3B3
0	0	1,053252444	0,827184216	FNBP4;HNRNPU;DLST
0	0	1,053252444	0,827184216	SEPT10;SHC2;SF3B3;U
0	0	1,055711399	0,827760108	MAP2K1;PSMD11;VPS
0	0	1,157005669	0,906344803	RBM28;RUVBL1;HNRN
0	0	1,157005669	0,906344803	HNRNPK;DLST;RSF1;C
0	0	1,061909313	0,830059356	NCKIPSD;PCMT1;HNR
0	0	1,061909313	0,830059356	PCMT1;HNRNPK;CRBI
0	0	1,126735172	0,878925619	HNRNPK;SP1;RUVBL1
0	0	1,094023506	0,851321313	USP47;HNRNPK;XPO4
0	0	1,094023506	0,851321313	USP15;CAPZB;KLHL7;I
0	0	1,049450088	0,811295039	UHRF1BP1L;ROCK1;C
0	0	1,051619494	0,811175065	SF3B3;HNRNPU;LRRC
0	0	1,051619494	0,811175065	SF3B3;TPM3;HNRNPU
0	0	1,033040776	0,795879845	SET;SF3B3;PSMD11;HI
0	0	1,291989664	0,994844838	DPYSL3;HNRNPU
0	0	1,291989664	0,994844838	NRIP1;PUM2
0	0	1,291989664	0,994844838	CRBN;HNRNPU
0	0	1,064826646	0,814119236	PCMT1;C20ORF112;HI
0	0	1,064826646	0,814119236	SART3;SP1;KLHL7;HNI
0	0	1,18652112	0,903028463	RBBP5;RUVBL1;TARDI
0	0	1,18652112	0,903028463	SF3B3;ATXN1L;HNRNF
0	0	1,18652112	0,903028463	RUVBL1;HNRNPU;TAR
0	0	1,18652112	0,903028463	HSPA4L;ATP2B1;CTGF
0	0	1,045675088	0,795689923	SET;SF3B3;CRBN;HSP.
0	0	1,085271318	0,824815493	HNRNPK;TNKS;SP1;C(
0	0	1,096972356	0,832480514	TPM3;RBBP5;HNRNPL
0	0	1,096972356	0,832480514	PCMT1;HNRNPK;CAPZ
0	0	1,096972356	0,832480514	KIF5C;RUVBL1;HOOK3
0	0	1,13999088	0,864771434	FNBP4;HNRNPK;UBN2
0	0	1,13999088	0,864771434	CRBN;RUVBL1;HNRNF
0	0	1,13999088	0,864771434	SESTD1;RUVBL1;HNR
0	0	1,13999088	0,864771434	CARM1;CAPRIN1;G3BI

0	0	1,13999088	0,864771434	NEK7;HNRNPU;HNRNI
0	0	1,13999088	0,864771434	TBL1XR1;PDE3B;RUVE
0	0	1,113784193	0,844532186	HNRNPK;RUVBL1;HNF
0	0	1,037231139	0,786466319	SET;SF3B3;TPM3;HNR
0	0	1,052299274	0,793859548	UTP15;HNRNPK;XRCC
0	0	1,033591731	0,775242341	MAP2K1;HNRNPU;LRF
0	0	1,033591731	0,775242341	SET;SF3B3;ATP8A2;TP
0	0	1,059007921	0,793988581	CAPRIN1;RUVBL1;HSF
0	0	1,041927148	0,780363987	RBM28;YLPM1;HNRNF
0	0	1,063528077	0,794792265	MAP2K1;CREBBP;HNF
0	0	1,069232825	0,796381703	SF3B3;TBL1XR1;RUVE
0	0	1,069232825	0,796381703	ENAH;PCMT1;HNRNPI
0	0	1,076658053	0,799150054	CPSF7;XPO4;SIN3A;RU
0	0	1,047559187	0,77632872	PCMT1;HNRNPK;CAPZ
0	0	1,047559187	0,77632872	SF3B3;XRCC5;SP1;SIN
0	0	1,030842817	0,762637228	SEPT10;MAP2K1;CRBI
0	0	1,101127555	0,811569446	HNRNPK;SP1;RUVBL1
0	0	1,123469273	0,825285506	DCTN2;CRBN;HNRNPU
0	0	1,123469273	0,825285506	FNBP4;SF3B3;HNRNPI
0	0	1,123469273	0,825285506	SF3B3;RBBP5;HNRNPI
0	0	1,039531914	0,763267223	MEF2A;HNRNPU;HNRI
0	0	1,250312578	0,917145122	TERF2IP;SNX6
0	0	1,162790698	0,852051221	RUVBL1;HNRNPU;TAR
0	0	1,162790698	0,852051221	PCMT1;HNRNPU;CDC
0	0	1,032781706	0,755571878	CREBBP;SET;SF3B3;H
0	0	1,041066052	0,761214696	SET;HNRNPU;ACTN4;S
0	0	1,061909313	0,773669649	PCMT1;HNRNPK;TBL1
0	0	1,024681458	0,746289387	FNBP4;RBM28;SET;HS
0	0	1,042861612	0,759166742	FNBP4;MAP2K1;HNRN
0	0	1,028539258	0,747576546	SET;CRBN;XRCC5;HNI
0	0	1,068180431	0,774296519	HNRNPK;CRBN;TBL1X
0	0	1,04499164	0,757141951	HNRNPK;SF3B3;TBL1X
0	0	1,076658053	0,776106625	CDH2;KIF5C;G3BP1;HI
0	0	1,088755335	0,779971136	PCMT1;HNRNPK;HNRI
0	0	1,088755335	0,779971136	XPO4;RBBP5;HNRNPU
0	0	1,088755335	0,779971136	FNBP4;UBN2;CCDC6;H
0	0	1,03820598	0,742365931	TCERG1;HDAC5;HNRN
0	0	1,030842817	0,736013774	CPSF7;SF3B3;XRCC5;
0	0	1,05468544	0,751604519	FNBP4;EDEM3;PCMT1
0	0	1,107419712	0,787767401	HNRNPK;RUVBL1;TAR
0	0	1,107419712	0,787767401	SF3B3;ZZZ3;RUVBL1;H
0	0	1,107419712	0,787767401	TBCEL;CPSF7;HNRNP
0	0	1,03989412	0,739461293	SEPT10;HNRNPK;RUV
0	0	1,03989412	0,739461293	FNBP4;PCYT1B;HNRN
0	0	1,031626728	0,732812563	MAP2K1;HNRNPU;LRF
0	0	1,032532723	0,72945491	FNBP4;RBM28;MAP2K
0	0	1,026746753	0,725306037	HNRNPU;PAPSS2;PHF
0	0	1,022983018	0,719181224	SET;SF3B3;PSMD11;TF
0	0	1,027200263	0,721757488	FNBP4;USP47;SF3B3;H
0	0	1,066780457	0,749417885	DFFA;HNRNPK;KLHL7;
0	0	1,044422782	0,733356943	FNBP4;DDX19B;HNRN
0	0	1,01999184	0,714038686	UTP15;SF3B3;XRCC5;I
0	0	1,023159517	0,715570809	HNRNPU;LRRC40;MAT

0	0	1,03484609	0,722177172	PCMT1;SET;HNRNPK;S
0	0	1,490757305	1,03961712	HNRNPK
0	0	1,076658053	0,749674538	SF3B3;CAPZB;CAPZA1
0	0	1,02335815	0,711771656	ARHGEF10;USP9X;HN
0	0	1,091822251	0,752105519	SF3B3;RUVBL1;HNRNI
0	0	1,091822251	0,752105519	FBXW4;TRIM24;DLST;I
0	0	1,091822251	0,752105519	HNRNPK;RUVBL1;KCN
0	0	1,040528589	0,709338574	HNRNPK;ZZZ3;XRCC5
0	0	1,040528589	0,709338574	HNRNPK;RBBP5;RUVE
0	0	1,118067979	0,759635486	KLHL7;CCDC6;TARDBI
0	0	1,118067979	0,759635486	HNRNPU;HNRNPR;AFI
0	0	1,118067979	0,759635486	RUVBL1;HNRNPU;TAR
0	0	1,024485196	0,694056229	SET;HNRNPK;CRBN;TI
0	0	1,064826646	0,720620255	RBM28;XPO4;RBBP5;F
0	0	1,064826646	0,720620255	FRMPD4;DPYSL5;ACT
0	0	1,064826646	0,720620255	HNRNPK;KIF5C;FUBP1
0	0	1,01999184	0,689250097	SET;XRCC5;HNRNPU;I
0	0	1,043530113	0,704338604	PCMT1;HNRNPK;XPO4
0	0	1,032062749	0,695017713	USP13;HNRNPK;RUVE
0	0	1,032062749	0,695017713	PCMT1;MED14;HNRNF
0	0	1,016425435	0,680507518	CPSF7;CRBN;YTHDC1
0	0	1,047559187	0,698846441	TBL1XR1;XRCC5;RUVI
0	0	1,076658053	0,718195651	PCMT1;HNRNPU;HNRI
0	0	1,076658053	0,718195651	HNRNPK;KIF5C;RUVBI
0	0	1,076658053	0,718195651	FNBP4;PCMT1;ZNF318
0	0	1,033591731	0,689101096	NPAT;HNRNPK;KIF5C;(
0	0	1,174536058	0,782671939	PCMT1;SNRPD3
0	0	1,174536058	0,782671939	HNRNPU;DLST
0	0	1,174536058	0,782671939	PCMT1;SIK2
0	0	1,174536058	0,782671939	FAM49A;ATP2B1
0	0	1,174536058	0,782671939	RUVBL1;TARDBP
0	0	1,174536058	0,782671939	XIAP;TARDBP
0	0	1,174536058	0,782671939	UBA6;JAKMIP2
0	0	1,025991792	0,676591855	PCMT1;HNRNPK;BCL1
0	0	1,025991792	0,676591855	SEPT10;HNRNPK;CRB
0	0	1,01587897	0,669637361	HNRNPU;DLST;HNRNF
0	0	1,035564235	0,682468966	FNBP4;HNRNPK;FUBP
0	0	1,010605412	0,665243601	FNBP4;MAP2K1;SET;S
0	0	1,053252444	0,692752011	RUVBL1;CCDC6;HNRN
0	0	1,096972356	0,71773023	PCMT1;HNRNPU;DLST
0	0	1,096972356	0,71773023	CAPZB;CRBN;GATAD2
0	0	1,096972356	0,71773023	ABCA2;TARDBP;FOXP
0	0	1,096972356	0,71773023	PCMT1;RC3H1;PTPN1
0	0	1,096972356	0,71773023	RPS6KA6;GFPT1;JMY
0	0	1,096972356	0,71773023	PCMT1;RUVBL1;HNRN
0	0	1,096972356	0,71773023	PCMT1;SET;HNRNPU
0	0	1,012379961	0,661562336	FNBP4;RBM28;CPSF7;
0	0	1,01999184	0,666004498	PCMT1;HNRNPK;RRM2
0	0	1,006745193	0,657210194	FNBP4;RBM28;CPSF7;
0	0	1,026746753	0,66943717	PCMT1;SF3B3;HNRNP
0	0	1,026746753	0,66943717	PHC2;DDX19B;HNRNP
0	0	1,03820598	0,674892548	SF3B3;DCTN2;RBBP5;
0	0	1,007514876	0,653177056	RBM28;SET;SF3B3;CR

0	0	1,061909313	0,685940401	HNRNPK;RBBP5;HNRN
0	0	1,061909313	0,685940401	DLST;PGAM5;AFF4;AF
0	0	1,027719051	0,661284238	SF3B3;CAPZB;CRBN;H
0	0	1,027719051	0,661284238	HNRNPK;SF3B3;RUVB
0	0	1,008616466	0,64810998	XRCC5;HNRNPU;LRR(
0	0	1,13999088	0,724373623	RUVBL1;ATP11A
0	0	1,13999088	0,724373623	CRBN;FUBP1
0	0	1,13999088	0,724373623	CPSF7;HNRNPU
0	0	1,13999088	0,724373623	SF3B3;SNRPD3
0	0	1,010323671	0,641401104	HNRNPK;CAPZB;DCTN
0	0	1,010323671	0,641401104	PCMT1;HNRNPK;SF3B
0	0	1,029018317	0,651781641	PCMT1;HNRNPK;PABF
0	0	1,029018317	0,651781641	EDEM3;HNRNPU;MAPI
0	0	1,029018317	0,651781641	MEF2A;XRCC5;RUVBL
0	0	1,003487118	0,635318051	FNBP4;PRDM8;HSPA4I
0	0	1,014061655	0,64116241	FNBP4;HNRNPK;SF3B
0	0	1,00488085	0,634912348	SET;HNRNPU;DLST;PD
0	0	1,00488085	0,634912348	SF3B3;XRCC5;TPM3;H
0	0	1,00488085	0,634912348	FNBP4;OTUD4;CSNK1
0	0	1,076658053	0,678416004	FYTTD1;SEPT10;HNRN
0	0	1,076658053	0,678416004	PCMT1;HNRNPK;TARD
0	0	1,076658053	0,678416004	PTP4A1;HNRNPK;DLS
0	0	1,000589821	0,630156488	FNBP4;KANK2;SF3B3;
0	0	1,01999184	0,640761791	HNRNPK;RBBP5;RUVE
0	0	1,01999184	0,640761791	PCMT1;ACAP2;PCYT1I
0	0	1,01999184	0,640761791	FNBP4;HNRNPK;RBBF
0	0	1,047559187	0,655248672	PHLPP2;HNRNPK;RUV
0	0	1,047559187	0,655248672	CAPZB;CAPZA1;RUVB
0	0	1,047559187	0,655248672	HNRNPK;CRBN;RUVBI
0	0	1,047559187	0,655248672	FNBP4;HNRNPU;TNFA
0	0	0,999533551	0,624345963	FNBP4;SF3B3;CSNK1A
0	0	1,013325227	0,631763055	HNRNPK;USP9X;VPS1
0	0	1,030842817	0,640362983	RBM28;PCMT1;SART3
0	0	1,030842817	0,640362983	HNRNPK;TPM3;RUVBL
0	0	1,030842817	0,640362983	RUVBL1;AGAP2;HNRN
0	0	1,01999184	0,629481853	PCMT1;HNRNPK;SF3B
0	0	1,01999184	0,629481853	HNRNPK;XRCC5;HNRI
0	0	1,012379961	0,620879833	HNRNPK;XRCC5;CUL3
0	0	1,006745193	0,613720749	HNRNPK;TRIM41;XPO
0	0	0,998103603	0,606224792	HNRNPK;ZZZ3;RUVBL
0	0	0,998103603	0,606224792	FNBP4;RBM28;HNRNP
0	0	1,057082452	0,641507029	SF3B3;HNRNPU;PGAM
0	0	1,057082452	0,641507029	HNRNPK;XPO4;ESR1
0	0	1,057082452	0,641507029	HNRNPK;XRCC5;HNRI
0	0	1,107419712	0,671183906	XIAP;SMAD9
0	0	1,107419712	0,671183906	SF3B3;HNRNPK
0	0	1,107419712	0,671183906	CRBN;RUVBL1
0	0	1,033591731	0,626035189	PCMT1;SHC2;KIF5C;LF
0	0	1,01999184	0,615743584	HNRNPK;SP1;HNRNPU
0	0	1,01999184	0,615743584	FNBP4;PCMT1;TRIM3;I
0	0	1,011122346	0,607962672	HNRNPK;CAPRIN1;RU
0	0	0,991014799	0,591810627	SET;SF3B3;ST13;TPM3
0	0	1,000250063	0,596187723	PCMT1;ZZZ3;SIN3A;HN

0	0	0,996677741	0,591389287	OTUD4;HNRNPK;HNRI
0	0	0,991881829	0,58847569	SEPT10;SET;CRBN;HN
0	0	0,990211056	0,584578105	SF3B3;HNRNPU;GIGYI
0	0	1,01999184	0,598220067	PCMT1;DENND5B;CAF
0	0	1,01999184	0,598220067	TIAL1;HNRNPK;HNRNI
0	0	0,989609104	0,579359451	SEPT10;HNRNPK;RXR
0	0	0,989609104	0,579359451	FNBP4;HNRNPK;SF3B
0	0	0,989609104	0,579359451	PCMT1;HNRNPK;MYO
0	0	1,03820598	0,60683319	RABGAP1L;HNRNPK;A
0	0	1,03820598	0,60683319	PCMT1;HNRNPK;RUVE
0	0	0,99749202	0,582954156	XRCC5;RUVBL1;HNRN
0	0	0,99749202	0,582954156	PCMT1;DOCK5;HSPH1
0	0	0,993838203	0,579149176	FNBP4;CPSF7;HNRNP
0	0	0,991014799	0,575650392	SEPT10;HNRNPK;SF3I
0	0	0,991014799	0,575650392	CPSF7;HNRNPK;SF3B
0	0	0,991014799	0,575650392	PCMT1;HNRNPK;CRBI
0	0	0,984373077	0,566459323	SF3B3;CRBN;XRCC5;T
0	0	0,993838203	0,567150486	MED14;HNRNPK;RUVE
0	0	0,993838203	0,567150486	HNRNPK;HNRNPU;SM
0	0	0,993838203	0,567150486	HNRNPK;TPM3;HNRNI
0	0	0,993838203	0,567150486	MED14;CRBN;RUVBL1
0	0	0,983035614	0,560823419	SF3B3;CRBN;XRCC5;H
0	0	0,990211056	0,564870703	HNRNPK;CAPZB;RCAI
0	0	0,99896108	0,569426955	PCMT1;CAPRIN1;G3BI
0	0	1,006745193	0,57172841	PCMT1;HNRNPK;HNRI
0	0	1,006745193	0,57172841	HNRNPK;CRBN;RUVBI
0	0	1,006745193	0,57172841	SH3PXD2A;HNRNPU;H
0	0	0,983748475	0,558062981	PCMT1;SF3B3;SART3;
0	0	0,982388454	0,555828246	MAP2K1;HNRNPK;TPM
0	0	0,980666853	0,553041382	FNBP4;MAP2K1;CPSF
0	0	1,01999184	0,5742385	PCMT1;PABPN1;CAPR
0	0	1,01999184	0,5742385	PCMT1;CPSF7;DIP2B
0	0	1,21124031	0,6772029	DLST
0	0	0,981257973	0,546498647	FNBP4;HNRNPK;XRCC
0	0	0,9887676	0,547645291	SUB1;RUVBL1;HNRNP
0	0	0,9887676	0,547645291	CREBBP;HNRNPK;EIF
0	0	0,97713504	0,540975892	SEPT10;MAP2K1;CRBI
0	0	0,976503816	0,539582308	FNBP4;RBM28;DIRAS2
0	0	1,047559187	0,578053737	HNRNPK;TARDBP
0	0	0,993838203	0,546489952	RUVBL1;HNRNPU;PCD
0	0	0,993838203	0,546489952	ACAP2;RUVBL1;HNRN
0	0	0,976503816	0,533523858	RBM28;SET;SF3B3;HN
0	0	0,975963415	0,530368537	BCLAF1;HNRNPK;SIN3
0	0	0,97713504	0,529125708	HNRNPK;HNRNPU;TAF
0	0	0,968992248	0,51722573	CPSF7;HNRNPK;SP1;F
0	0	0,968992248	0,51722573	PCMT1;PHC2;HNRNPK
0	0	0,968992248	0,517140569	DFFA;CAPRIN1;SERBF
0	0	0,968992248	0,517016857	PCMT1;RIC8B;HNRNPI
0	0	0,968992248	0,516665138	PPP2CA;PCMT1;XPO4
0	0	0,968992248	0,516665138	OTUD4;PCMT1;HDAC5
0	0	0,968992248	0,515972366	FNBP4;CREBBP;CPSF
0	0	0,981257973	0,522438724	HNRNPK;HNRNPU;SM
0	0	0,981257973	0,522438724	HNRNPK;ERBB4;RUVE

0	0	0,981257973	0,522438724	HNRNPK;XPO4;HNRNI
0	0	0,981257973	0,522438724	DOCK5;HNRNPU;TARD
0	0	0,981257973	0,522438724	PCMT1;C1QBP;CAPRII
0	0	0,981257973	0,522438724	C20ORF112;SF3B3;HS
0	0	0,968992248	0,515224019	SF3B3;XRCC5;HNRNP
0	0	0,968992248	0,51436571	SEPT10;MAP2K1;SF3E
0	0	0,968992248	0,513914212	CREBBP;HNRNPK;SP1
0	0	0,968992248	0,511094962	PCMT1;HNRNPK;USP9
0	0	0,968992248	0,511094962	PCMT1;HNRNPK;FUBF
0	0	1,01999184	0,537232486	HNRNPU;PGAM5
0	0	1,01999184	0,537232486	HNRNPK;CEP170
0	0	1,01999184	0,537232486	DLST;FBXO30
0	0	0,968992248	0,510206127	RBM28;SET;ATP8A2;HI
0	0	0,964607668	0,504933269	FNBP4;HNRNPK;SF3B
0	0	0,964171391	0,504150432	RBM28;HNRNPK;KIF5C
0	0	0,965773005	0,504964594	SF3B3;HNRNPU;HNRN
0	0	0,968992248	0,506639611	HNRNPU;DEK;TARDBF
0	0	0,968992248	0,506639611	PCMT1;HNRNPU;SNIP
0	0	0,968992248	0,506639611	CAPRIN1;KLHL7;RUVE
0	0	0,968992248	0,506639611	HNRNPK;RBBP5;RUVE
0	0	0,968992248	0,506639611	MAP2K1;HNRNPK;RBE
0	0	0,968992248	0,506639611	DOCK5;SF3B3;RUVBL
0	0	0,985415845	0,514725165	HNRNPK;ITPK1;TARDE
0	0	0,985415845	0,514725165	PCMT1;BCLAF1;HNRN
0	0	0,985415845	0,514725165	HNRNPU;HOOK3;CALM
0	0	0,985415845	0,514725165	BCLAF1;HNRNPK;RAB
0	0	0,965973581	0,50448723	NCOA2;SF3B3;STAU1;
0	0	0,9636387	0,502860343	SET;HNRNPK;RUVBL1
0	0	1,13999088	0,594099447	RUVBL1
0	0	1,13999088	0,594099447	CPSF7
0	0	0,962973663	0,500887536	PPP2CA;PSMD11;HNR
0	0	0,962119963	0,497968366	HNRNPK;CARM1;RUVI
0	0	0,962119963	0,497968366	HNRNPK;CAPRIN1;RU
0	0	0,968992248	0,499512748	CPSF7;RUVBL1;HNRN
0	0	0,968992248	0,499512748	XPO4;RUVBL1;HNRNP
0	0	0,968992248	0,499512748	RUVBL1;HNRNPU;HNF
0	0	0,961595361	0,494498334	CPSF7;HNRNPU;HNRN
0	0	0,960984048	0,493686782	CAPRIN1;RUVBL1;G3E
0	0	0,964797476	0,490807525	FNBP4;SF3B3;CRBN;H
0	0	0,957029381	0,482696811	HNRNPK;RBBP5;RUVE
0	0	0,955956478	0,481173342	FNBP4;HNRNPK;RXRA
0	0	0,968992248	0,487553588	HNRNPK;DDI2;DLST
0	0	0,968992248	0,487553588	HSPA4L;HNRNPU;LNP
0	0	0,968992248	0,487553588	XPO4;ZNF318;HNRNPU
0	0	0,968992248	0,487553588	DPYSL3;HNRNPU;TAR
0	0	0,993838203	0,499740374	HNRNPU;LRRC40
0	0	0,953107129	0,476878487	FNBP4;HNRNPK;HNRN
0	0	0,954310547	0,473295314	SEPT10;MAP2K1;SET;I
0	0	0,948663739	0,467537589	ENAH;HSPH1;SF3B3;U
0	0	0,955718382	0,467790393	FNBP4;SRPK2;RBM28;
0	0	0,955718382	0,467790393	CPSF7;SET;ESCO1;XR
0	0	1,076658053	0,523720552	CAPRIN1
0	0	1,076658053	0,523720552	PCMT1

0	0	0,945358291	0,459013368	PCMT1;HNRNPK;WDR
0	0	0,953107129	0,461953244	SEPT10;RUVBL1;HNRI
0	0	0,945358291	0,456806939	TTC9;XRCC5;HNRNPU
0	0	0,945358291	0,456806939	HNRNPK;UBN2;DIRAS
0	0	0,945358291	0,455241473	PCMT1;PURA;HNRNPI
0	0	0,945358291	0,455241473	FNBP4;RBM28;SET;HN
0	0	0,9499924	0,456421045	CPSF7;SET;SF3B3;YTI
0	0	0,968992248	0,465256812	TARDBP;GIGYF2
0	0	0,968992248	0,465256812	ABCA2;HNRNPU
0	0	0,948663739	0,454877285	HNRNPU;DLST;PDHB;
0	0	0,940769173	0,450993825	PCMT1;RUVBL1;HNRN
0	0	0,940769173	0,450993825	PCMT1;SIN3A;FUBP1;
0	0	0,937734434	0,444975361	SF3B3;HNRNPU;HNRN
0	0	0,937734434	0,444975361	HDAC5;HNRNPK;RUVE
0	0	0,939628847	0,445831273	HNRNPK;SF3B3;HNRN
0	0	0,939628847	0,445831273	FNBP4;HNRNPK;RUVE
0	0	0,949444854	0,449979668	RBM28;CPSF7;HNRNP
0	0	0,935578722	0,438952727	RBM28;RUVBL1;HNRN
0	0	0,933968432	0,436920693	HNRNPU;RAF1;TARDB
0	0	0,933968432	0,436920693	SF3B3;ATXN1L;HNRNI
0	0	0,933968432	0,436920693	CPSF7;PSMD11;HNRN
0	0	0,933968432	0,436920693	HNRNPK;XPO4;HNRNI
0	0	0,947927199	0,44273444	KANK2;RBM28;SF3B3;
0	0	0,937734434	0,43782101	CAPZB;CAPZA1;DLST
0	0	0,937734434	0,43782101	RBBP5;CAPRIN1;HNRI
0	0	0,937734434	0,43782101	IGBP1;HNRNPU;TARDI
0	0	0,931723315	0,433878609	XPO4;LUZP1;CRBN;HS
0	0	0,931723315	0,433878609	PCMT1;BCLAF1;RBBP
0	0	0,93911143	0,436787908	RRM1;HNRNPK;JPH3;
0	0	0,933968432	0,433018887	HNRNPK;CRBN;KIF5C;
0	0	0,933968432	0,433018887	HNRNPK;CRBN;CAPRI
0	0	0,936224394	0,4325185	SET;HNRNPK;SERBP1
0	0	0,937734434	0,430198729	FNBP4;HNRNPK;XRCC
0	0	0,945358291	0,433497705	RUVBL1;FBXO30
0	0	0,929170649	0,425316241	HNRNPK;CAPRIN1;RU
0	0	0,929170649	0,425316241	MED14;RUVBL1;HNRN
0	0	1,01999184	0,463675624	CMC1
0	0	1,01999184	0,463675624	HNRNPC
0	0	1,01999184	0,463675624	NPAT
0	0	0,928375807	0,420565744	MAP3K2;C20ORF112;X
0	0	0,928375807	0,420565744	FNBP4;TCERG1;HNRN
0	0	0,92284976	0,417946422	HNRNPK;TBL1XR1;RU
0	0	0,92284976	0,417430354	HNRNPR;SNRPD3;TAF
0	0	0,933968432	0,420361939	FNBP4;RBM28;HNRNP
0	0	0,92284976	0,415221701	HNRNPK;XPO4;RUVBL
0	0	0,92284976	0,415061615	HNRNPK;RUVBL1;CDC
0	0	0,92284976	0,415061615	PCMT1;TPM3;MYH10
0	0	0,92284976	0,415061615	XPO4;XRCC5;GATAD2I
0	0	0,930909583	0,416072536	CPSF7;HNRNPK;SP1;C
0	0	0,92284976	0,412098701	HNRNPK;IGSF3;XPO4;
0	0	0,92284976	0,412098701	HNRNPK;CRBN;HNRN
0	0	0,92284976	0,412098701	FNBP4;SET;HNRNPK;F
0	0	0,930232558	0,410735646	FNBP4;CPSF7;HNRNP

0	0	0,93471921	0,412187379	FNBP4;HNRNPU;HNRN
0	0	0,91414363	0,401621313	HNRNPK;HNRNPU;HN
0	0	0,91414363	0,401621313	FNBP4;PCMT1;SET;HN
0	0	0,932363675	0,409138584	FYTTD1;SF3B3;HNRNI
0	0	0,911992704	0,399838323	SF3B3;RUVBL1;USP1;
0	0	0,92284976	0,404210609	HNRNPU;CTNNB1
0	0	0,92284976	0,404210609	KANK2;HNRNPU
0	0	0,916614289	0,399286929	TCERG1;XPO4;RUVBL
0	0	0,92284976	0,400448266	HNRNPK;CAPZB;SERE
0	0	0,908430233	0,393586946	HNRNPK;HNRNPU;PG
0	0	0,908430233	0,393586946	PCMT1;HNRNPU;JAKM
0	0	0,908430233	0,393586946	C20ORF112;HNRNPU;
0	0	0,910462515	0,386868193	HNRNPK;CAPRIN1;HN
0	0	0,932719811	0,396213663	SET;SF3B3;CSNK1A1;
0	0	0,901388138	0,382553202	HNRNPK;RBBP5;RUVE
0	0	0,901388138	0,382553202	IGBP1;RUVBL1;DPYSL
0	0	0,911992704	0,385261791	HNRNPK;SF3B3;FUBP
0	0	0,913186412	0,383010891	ENAH;HNRNPK;SF3B3
0	0	0,901388138	0,377170632	RUVBL1;HNRNPU
0	0	0,901388138	0,377170632	HNRNPU;TARDBP
0	0	0,901388138	0,377170632	SIN3A;HNRNPU
0	0	0,901388138	0,377170632	HNRNPU;GAPVD1
0	0	0,901388138	0,377170632	HNRNPU;TARDBP
0	0	0,894454383	0,373315421	ENAH;RUVBL1;HNRNI
0	0	0,894454383	0,373315421	CUL3;HNRNPU;DLST
0	0	0,91414363	0,38033822	HNRNPK;PHF20;RUVB
0	0	0,92284976	0,383768181	SF3B3;CRBN;TPM3;HN
0	0	0,897215045	0,371816246	HNRNPK;HNRNPU;XIA
0	0	0,897215045	0,371816246	PCMT1;HNRNPU;SMAI
0	0	0,929841046	0,384227129	MAP3K2;OTUD4;RBM2
0	0	0,908430233	0,372665707	PCMT1;CAPRIN1;CAM
0	0	0,925297637	0,37464445	FNBP4;SF3B3;HNRNPI
0	0	0,894454383	0,361569832	PCMT1;HNRNPK;FCHS
0	0	0,901388138	0,363343908	HNRNPK;KIF5C;RUVBI
0	0	0,888983714	0,357770892	PCMT1;HNRNPK;RUVE
0	0	0,880902044	0,354171434	HNRNPK;HNRNPC;TAI
0	0	0,880902044	0,354171434	KAT2B;CREB1;HNRNP
0	0	0,90372334	0,36258872	NPAT;C20ORF112;HNR
0	0	0,920111333	0,368193079	MEF2A;SET;ST13;HNR
0	0	0,880902044	0,352176933	HNRNPU;DLST
0	0	0,880902044	0,352176933	DLST;HK2
0	0	0,880902044	0,352176933	NEK7;HNRNPU
0	0	0,880902044	0,352176933	XPO4;RUVBL1
0	0	0,880902044	0,352176933	HNRNPU;TARDBP
0	0	0,92284976	0,367558366	MAP2K1
0	0	0,880902044	0,350291164	CRBN;RUVBL1;TARDB
0	0	0,880902044	0,350291164	ACAP2;HNRNPK;HNRI
0	0	0,912818784	0,362793641	FNBP4;SEPT10;XRCC
0	0	0,907141679	0,359337553	HNRNPK;KIF5C;TBL1X
0	0	0,920271967	0,36450712	SET;SF3B3;XRCC5;HN
0	0	0,89249286	0,351849261	HNRNPK;USP9X;RUVE
0	0	0,896177802	0,352843416	SRPK2;CRBN;TPM3;AT
0	0	0,887626487	0,349277941	HNRNPK;USP9X;CCD

0	0	0,880902044	0,344265968	FNBP4;HNRNPK;HNRN
0	0	0,880902044	0,344265968	HNRNPK;PAPOLG;RUV
0	0	0,880902044	0,344265968	HNRNPK;RUVBL1;HNF
0	0	0,880902044	0,344265968	HNRNPK;MAGEE1;TAF
0	0	0,880902044	0,344265968	HNRNPK;RBBP5;HNRN
0	0	0,903297858	0,350620207	HNRNPK;SF3B3;TBL1X
0	0	0,867754252	0,336084841	PCMT1;HNRNPK;HNRI
0	0	0,871004268	0,335239434	FNBP4;HNRNPK;HNRN
0	0	0,891027355	0,342637184	ZNF275;HNRNPK;CUL3
0	0	0,880902044	0,337403184	PCYT1B;HNRNPK;CAF
0	0	0,897215045	0,342956673	SUB1;RUVBL1;CCDC6
0	0	0,899486475	0,342101698	KIF5C;CAPRIN1;SERBI
0	0	0,872965989	0,331279519	USP47;SF3B3;HNRNPU
0	0	0,880902044	0,328864994	DLST
0	0	0,880902044	0,328864994	PCMT1
0	0	0,85499316	0,318990509	RUVBL1;HSPA4L;HNRI
0	0	0,85499316	0,318990509	PCYT1B;HNRNPU;GNL
0	0	0,85499316	0,318990509	SP1;HNRNPU;JAKMIP2
0	0	0,87427872	0,325930952	HNRNPK;HNRNPU;DLS
0	0	0,861326443	0,320860774	HNRNPK;KIF5C;RUVBI
0	0	0,861326443	0,320860774	HNRNPK;RUVBL1;HNF
0	0	0,885373628	0,324828562	HNRNPK;RUVBL1;HNF
0	0	0,901388138	0,330090468	NCOA2;CPSF7;SF3B3;
0	0	0,875218805	0,319948847	SF3B3;HNRNPU;TERF2
0	0	0,875218805	0,319948847	ACAP2;HNRNPK;CRBN
0	0	0,842601955	0,30762773	HNRNPU;CEP170
0	0	0,842601955	0,30762773	HNRNPU;ZNF800
0	0	0,842601955	0,30762773	PCYT1B;APC
0	0	0,842601955	0,30762773	PCMT1;NOS1AP
0	0	0,842601955	0,30762773	ITCH;FRAS1
0	0	0,842601955	0,30762773	HNRNPK;DLST
0	0	0,907596045	0,330748831	MAP2K1;CRBN;NEK7;H
0	0	0,867754252	0,314847194	HNRNPK;RUVBL1;ATX
0	0	0,867754252	0,314847194	SF3B3;HNRNPU;HNRN
0	0	0,851861317	0,307122715	TNKS;CCDC6;HNRNPU
0	0	0,842601955	0,3028279	PCMT1;HNRNPK;RAB1
0	0	0,880902044	0,315993456	FNBP4;HNRNPK;SP1;F
0	0	0,884924427	0,317233678	PCMT1;HNRNPK;TBL1
0	0	0,875925196	0,313703082	MEF2A;HNRNPK;SIN3A
0	0	0,857515264	0,306779096	PCMT1;DDX19B;HNRN
0	0	0,895438381	0,316073106	SF3B3;ST13;HNRNPU;
0	0	0,824674254	0,287765937	DLST;FBXO30
0	0	0,824674254	0,287765937	UTP15;HNRNPU
0	0	0,824674254	0,287765937	NCKAP1;HK2
0	0	0,842601955	0,293994538	HNRNPK;TERF2IP;TAF
0	0	0,864069521	0,300274676	PCMT1;HNRNPK;SF3B
0	0	0,8499932	0,295225933	PCMT1;RBM28;HNRNF
0	0	0,830564784	0,287540694	HNRNPU;SMG7;SNX8
0	0	0,830564784	0,287540694	HNRNPK;VAPA;HNRNF
0	0	0,866138322	0,29574062	NXF1;SF3B3;NUFIP2;C
0	0	0,858600726	0,290886271	FNBP4;CPSF7;DDX19E
0	0	0,858600726	0,290886271	FNBP4;HNRNPK;RRM2
0	0	0,842601955	0,284112775	SRPK2;PCMT1;HNRNF

0	0	0,80749354	0,269333725	CRBN;HNRNPU
0	0	0,80749354	0,269333725	PCMT1;CEP170
0	0	0,861326443	0,287135563	ENAH;NCKAP1;RUVBL
0	0	0,835338145	0,273422113	PCMT1;KLHL7;HNRNP
0	0	0,835338145	0,273422113	FUBP1;RUVBL1;SNRPI
0	0	0,824674254	0,269453097	RUVBL1;CCDC6;HNRN
0	0	0,824674254	0,269453097	SF3B3;SUB1;FAM73A;I
0	0	0,842601955	0,274133894	PCMT1;SF3B3;CRBN;X
0	0	0,842601955	0,274133894	HNRNPK;KIAA0355;HN
0	0	0,86517165	0,278369872	RBM28;HNRNPK;SF3B
0	0	0,80749354	0,259386324	HNRNPK;RUVBL1;ZBT
0	0	0,80749354	0,259386324	FUBP1;RUVBL1;HNRN
0	0	0,80749354	0,259386324	HNRNPK;ROCK1;HNRI
0	0	0,79101408	0,252213195	CRBN;SERBP1
0	0	0,83654007	0,264799475	HNRNPK;XRCC5;HNRI
0	0	0,815993472	0,257986232	PCMT1;HNRNPK;HNRI
0	0	0,815993472	0,257986232	SEPT10;ACAP2;HNRNI
0	0	0,866578433	0,273697794	PCMT1;SET;HNRNPK;S
0	0	0,885638076	0,277013769	SRPK2;RBM28;SF3B3;
0	0	0,867754252	0,268961074	FNBP4;UHRF1BP1L;HI
0	0	0,796431985	0,246424727	PCMT1;WDR37;HNRNI
0	0	0,863070019	0,266944168	FNBP4;RBM28;SET;HN
0	0	0,775193798	0,239437363	PCMT1
0	0	0,775193798	0,239437363	DLST
0	0	0,821179871	0,253242	CPSF7;HNRNPU;HNRI
0	0	0,821179871	0,253242	PFKFB3;HNRNPK;DCT
0	0	0,830564784	0,255779585	PCMT1;HNRNPK;FUBI
0	0	0,883263711	0,271558268	RBM28;SF3B3;HNRNP
0	0	0,80749354	0,247021868	CSNK1A1;CAPRIN1;SE
0	0	0,775193798	0,23629774	RUVBL1;TARDBP
0	0	0,859589897	0,260346558	HNRNPK;ZZZ3;FRMPD
0	0	0,842601955	0,255064776	HNRNPK;ROCK1;CRBI
0	0	0,824674254	0,247063441	NRP2;HNRNPU;CEP17
0	0	0,8142792	0,243721183	HNRNPK;SF3B3;RUVB
0	0	0,8142792	0,243721183	DIAPH2;CRBN;CAPRIN
0	0	0,8142792	0,243721183	PCMT1;HNRNPU;DLST
0	0	0,846692256	0,253112329	RBM28;HNRNPK;SF3B
0	0	0,78566939	0,234149134	PCMT1;SP1;HNRNPU
0	0	0,78566939	0,234149134	HOOK3;GAPVD1;SNX8
0	0	0,78566939	0,234149134	PCMT1;NOS1AP;HNRI
0	0	0,799168864	0,236536578	HNRNPK;XRCC5;HNRI
0	0	0,858148116	0,250323297	FNBP4;PCMT1;SEPT1(
0	0	0,858148116	0,250323297	PCMT1;RBM28;HNRNI
0	0	0,75999392	0,221490787	PCMT1;RUVBL1
0	0	0,75999392	0,221490787	PCMT1;SMC1A
0	0	0,75999392	0,221490787	HNRNPU;HNRNPR
0	0	0,846281439	0,244028766	HNRNPK;SF3B3;NFIA;(
0	0	0,864069521	0,248256861	SEPT10;SF3B3;HNRNI
0	0	0,775193798	0,222519819	PCMT1;HNRNPU;PGAI
0	0	0,775193798	0,222519819	FYTTD1;HNRNPK;TARI
0	0	0,849315915	0,241456136	MAP3K2;OTUD4;CPSF
0	0	0,849315915	0,241456136	PCMT1;SET;HNRNPK;S
0	0	0,842601955	0,237657769	HNRNPK;CRBN;FUBP1

0	0	0,842601955	0,237657769	MAP3K2;RBM28;HNRN
0	0	0,745378652	0,207704715	SEPT10;CRBN
0	0	0,745378652	0,207704715	SEPT10;ZNRF2
0	0	0,783024039	0,216915467	SEPT10;RUVBL1;TARD
0	0	0,783024039	0,216915467	HNRNPK;RUVBL1;HNF
0	0	0,783024039	0,216915467	HNRNPK;RUVBL1;HNF
0	0	0,76499388	0,211499649	CRBN;KIF5C;GIGYF2
0	0	0,76499388	0,211499649	HNRNPK;RUVBL1;HNF
0	0	0,76499388	0,211499649	CRBN;HNRNPU;NPTXI
0	0	0,76499388	0,211499649	CRBN;XRCC5;HNRNPU
0	0	0,76499388	0,211499649	SF3B3;HNRNPU;PGAM
0	0	0,80749354	0,222635481	HNRNPK;SF3B3;RFWD
0	0	0,717772036	0,196018818	CPSF7
0	0	0,845665962	0,227332351	RBM28;HNRNPK;RUVE
0	0	0,775193798	0,207738547	PCMT1;HNRNPK;UBN2
0	0	0,820310898	0,219795445	ACAP2;HNRNPK;SF3B
0	0	0,820310898	0,219795445	GABPB2;HNRNPK;RUV
0	0	0,731314904	0,194859889	HNRNPU;PGAM5
0	0	0,731314904	0,194859889	HNRNPK;CRBN
0	0	0,787798576	0,209097185	LUZP1;RUVBL1;HNRNI
0	0	0,842601955	0,221898717	HNRNPK;SF3B3;CRBN
0	0	0,796431985	0,207688226	TFEB;LRRC40;CEP170
0	0	0,796431985	0,207688226	HNRNPK;SP1;CAPZA1
0	0	0,767518612	0,198958372	HNRNPK;CAPZA1;HNF
0	0	0,767518612	0,198958372	RBBP5;HNRNPU;DLST
0	0	0,69213732	0,177878081	DLST
0	0	0,69213732	0,177878081	DLST
0	0	0,745378652	0,191150037	STRN;ASPRV1;CC2D1I
0	0	0,717772036	0,182883823	RUVBL1;HNRNPU
0	0	0,75999392	0,190556889	HNRNPK;CAPRIN1;DLS
0	0	0,75999392	0,190556889	ENAH;HNRNPK;HNRNI
0	0	0,775193798	0,193677888	PCMT1;CAPZB;CAPRIN
0	0	0,839793282	0,209635105	SEPT10;MAP2K1;EIF2I
0	0	0,78566939	0,193728399	KANK2;PCMT1;HNRNF
0	0	0,842601955	0,207613402	FNBP4;SF3B3;XRCC5;
0	0	0,704721635	0,171710442	TARDBP;GATAD2B
0	0	0,704721635	0,171710442	NOS1AP;TARDBP
0	0	0,704721635	0,171710442	CAPRIN1;GATAD2B
0	0	0,803309636	0,194995643	DFFA;HNRNPK;NKRF;
0	0	0,769041467	0,186399441	HNRNPK;SF3B3;SP1;R
0	0	0,668270516	0,161701175	DLST
0	0	0,668270516	0,161701175	DLST
0	0	0,83999328	0,202986031	MAP2K1;HNRNPU;SM(
0	0	0,780396441	0,187098099	RBM28;HNRNPK;SF3B
0	0	0,780396441	0,187098099	FNBP4;HNRNPK;RUVE
0	0	0,823082218	0,197146027	FNBP4;CPSF7;HNRNP
0	0	0,726744186	0,172848145	HNRNPK;RUVBL1;CEF
0	0	0,726744186	0,172848145	HNRNPK;HNRNPU;PH/
0	0	0,726744186	0,172848145	PCMT1;DLST;TARDBP
0	0	0,788714621	0,185929566	FNBP4;RIC8B;NUFIP2;
0	0	0,762986022	0,179393778	PCMT1;HNRNPK;HNRI
0	0	0,745378652	0,174822214	G3BP1;DYRK1A;HNRN
0	0	0,745378652	0,174822214	PCMT1;RUVBL1;HNRN

0	0	0,775193798	0,180690432	PCMT1;HNRNPK;FUBF
0	0	0,69213732	0,161279428	PCMT1;HOOK3
0	0	0,717772036	0,164394803	PCMT1;HNRNPK;DLST
0	0	0,645994832	0,1472368	PFAS
0	0	0,738279808	0,167457203	USP15;HNRNPK;HNRN
0	0	0,738279808	0,167457203	CRBN;HNRNPU;HNRN
0	0	0,770060065	0,174497909	PCMT1;RIC8B;HNRNPI
0	0	0,779648935	0,174325916	RBM28;RUVBL1;HNRN
0	0	0,779648935	0,174325916	KANK2;HNRNPK;FCHS
0	0	0,829718133	0,185404424	MAP2K1;SF3B3;HNRNI
0	0	0,67999456	0,151535643	PFKFB3;HNRNPK
0	0	0,67999456	0,151535643	HNRNPU;NUDT5
0	0	0,67999456	0,151535643	HNRNPK;TARDBP
0	0	0,709018718	0,156372495	PCMT1;HNRNPK;GAP\
0	0	0,820644901	0,177827138	FNBP4;SF3B3;HNRNPI
0	0	0,792811839	0,17062979	HNRNPK;CRBN;CAPZE
0	0	0,625156289	0,134271427	PAPOLG
0	0	0,625156289	0,134271427	DLST
0	0	0,80749354	0,173433866	PCMT1;SF3B3;SART3;:
0	0	0,745378652	0,159911098	HNRNPK;RUVBL1;HNF
0	0	0,75999392	0,162729641	FNBP4;PCMT1;HNRNF
0	0	0,668270516	0,142428619	XRCC5;RAB11A
0	0	0,828198503	0,176167869	FNBP4;CRBN;HNRNPL
0	0	0,700476324	0,14875764	PCMT1;CAPZA1;RUVB
0	0	0,724480185	0,153658023	HNRNPK;RUVBL1;HNF
0	0	0,789224455	0,165837959	PCMT1;HNRNPK;SART
0	0	0,789224455	0,165837959	FNBP4;HNRNPK;RUVE
0	0	0,819691328	0,169226235	MAP2K1;FAM114A1;SH
0	0	0,78566939	0,161173537	CPSF7;RUVBL1;HNRN
0	0	0,78566939	0,161173537	HNRNPK;FUBP1;RUVE
0	0	0,656943897	0,133912104	DLST;KCNRG
0	0	0,605620155	0,122622744	CDC42BPA
0	0	0,605620155	0,122622744	HNRNPU
0	0	0,734085036	0,148105956	HNRNPK;CRBN;SUB1;
0	0	0,771337113	0,153182005	HNRNPK;LUZP1;HNRN
0	0	0,711186971	0,141009715	PCMT1;HNRNPK;MB;H
0	0	0,787798576	0,154733576	HSPH1;NUFIP2;SH3KB
0	0	0,757871032	0,148293045	MEF2A;PCMT1;SIPA1L
0	0	0,80749354	0,157894218	SET;ATP8A2;HNRNPU;
0	0	0,645994832	0,125943661	RUVBL1;HNRNPU
0	0	0,645994832	0,125943661	MARCKS;HNRNPU
0	0	0,645994832	0,125943661	FNBP4;HNRNPU
0	0	0,704721635	0,135085696	SEPT10;RANBP3;HNRI
0	0	0,704721635	0,135085696	HNRNPK;RUVBL1;PDC
0	0	0,740631018	0,141473246	PCMT1;HNRNPK;RUVE
0	0	0,587268029	0,112134385	TRHDE
0	0	0,676041103	0,128142809	RUVBL1;HNRNPU;RAE
0	0	0,676041103	0,128142809	HNRNPK;CEP170;GAT/
0	0	0,676041103	0,128142809	CAPRIN1;HNRNPU;CD
0	0	0,8142792	0,154187972	SEPT10;MAP2K1;SEPT
0	0	0,698372791	0,129412849	HNRNPK;SF3B3;TARDI
0	0	0,698372791	0,129412849	SF3B3;RBBP5;HNRNPI
0	0	0,717772036	0,131999359	HNRNPK;HNRNPU;HN

0	0	0,668270516	0,121949082	ITCH;HNRNPU;GPRAS
0	0	0,745378652	0,134517095	PCMT1;TPM3;RUVBL1
0	0	0,745378652	0,134517095	PCMT1;XRCC5;FUBP1
0	0	0,731314904	0,131886373	HNRNPK;XRCC5;RUVE
0	0	0,56999544	0,102671668	LUZP1
0	0	0,625156289	0,111498182	RBBP5;HNRNPU
0	0	0,7124943	0,12702574	HNRNPK;SF3B3;HNRN
0	0	0,7124943	0,12702574	HSPH1;CAPZB;RUVBL
0	0	0,7124943	0,12702574	HNRNPK;CRBN;XRCC
0	0	0,780872874	0,137308623	PCMT1;RBM28;HNRNF
0	0	0,726744186	0,127333308	FNBP4;ENAH;HSPH1;C
0	0	0,686012211	0,118777181	PCMT1;HNRNPK;HNRI
0	0	0,686012211	0,118777181	HNRNPU;HNRNPR;PG.
0	0	0,686012211	0,118777181	NFIA;HNRNPU;TARDBI
0	0	0,778022973	0,133759532	RBM28;HNRNPK;SF3B
0	0	0,769041467	0,131339369	FNBP4;DDX19B;HNRN
0	0	0,615233173	0,104952287	PCMT1;HNRNPK
0	0	0,653253201	0,110472639	PSMD11;HNRNPK;HNF
0	0	0,67999456	0,113793875	NFIA;UBA6;RUVBL1;HI
0	0	0,67999456	0,113793875	HNRNPK;SF3B3;HNRN
0	0	0,717772036	0,118681564	HNRNPK;XPO4;CRBN;
0	0	0,605620155	0,0988162	SIN3A;RAB11A
0	0	0,697116725	0,113189213	FNBP4;HNRNPK;HNRI
0	0	0,697116725	0,113189213	SF3B3;CAPZB;RUVBL1
0	0	0,674081564	0,10902072	PCMT1;HNRNPU;XIAP
0	0	0,538329027	0,086372691	HNRNPU
0	0	0,538329027	0,086372691	CEP170
0	0	0,69213732	0,108916195	HNRNPK;NFIA;RUVBL1
0	0	0,638895988	0,100106602	EIF3J;HNRNPU;TARDE
0	0	0,638895988	0,100106602	ALDH2;RUVBL1;HNRN
0	0	0,596302922	0,093061862	ABCA2;CDC42BPA
0	0	0,709018718	0,110602581	HNRNPK;SP1;CAPRIN
0	0	0,687228545	0,104802258	SP1;RUVBL1;DLST;TAF
0	0	0,687228545	0,104802258	HNRNPK;SF3B3;HNRN
0	0	0,704721635	0,106766092	HNRNPK;SF3B3;SIN3A
0	0	0,662558802	0,100068894	CRBN;HNRNPU;MTSS
0	0	0,631951466	0,095304194	PCMT1;HNRNPU;TARD
0	0	0,631951466	0,095304194	HNRNPU;HNRNPC;ZNI
0	0	0,761351052	0,114194769	HNRNPK;RUVBL1;HNF
0	0	0,587268029	0,087663361	APC;HNRNPU
0	0	0,717772036	0,106993558	PCMT1;SEPT10;HNRN
0	0	0,713994288	0,103533549	PCMT1;TPM3;CAPRIN
0	0	0,50999592	0,072965042	HNRNPU
0	0	0,50999592	0,072965042	HNRNPU
0	0	0,50999592	0,072965042	CRBN
0	0	0,50999592	0,072965042	TTC9
0	0	0,50999592	0,072965042	HNRNPK
0	0	0,50999592	0,072965042	RBBP5
0	0	0,578502835	0,08259675	HNRNPU;RAB11A
0	0	0,745378652	0,105270551	FBXO40;HNRNPK;TBL
0	0	0,73285128	0,101464666	PCMT1;HNRNPK;RUVE
0	0	0,772985269	0,106036399	RBM28;SF3B3;HNRNP
0	0	0,56999544	0,077839877	RBBP5;HNRNPU

0	0	0,496919102	0,067158458	DLST
0	0	0,496919102	0,067158458	HNRNPU
0	0	0,668270516	0,089822006	HNRNPK;RUVBL1;HNF
0	0	0,640656032	0,084313634	HNRNPK;CSNK1A1;DL
0	0	0,640656032	0,084313634	HNRNPK;TBL1XR1;RU
0	0	0,561734637	0,073372234	RUVBL1;AFF4
0	0	0,663693321	0,086418891	SART3;HNRNPU;SNRF
0	0	0,484496124	0,061868268	HNRNPU
0	0	0,484496124	0,061868268	HNRNPK
0	0	0,695686742	0,08778298	KANK2;PCMT1;HNRNF
0	0	0,6591784	0,083142496	PCMT1;WDR37;RUVBL
0	0	0,6591784	0,083142496	OTUD4;HNRNPK;SH3F
0	0	0,553709856	0,069174816	PCMT1;HNRNPU
0	0	0,553709856	0,069174816	HNRNPK;RUVBL1
0	0	0,599376648	0,074602188	PCMT1;CAPZB;HNRNF
0	0	0,778859017	0,095300078	PSMD11;CRBN;DCUN1
0	0	0,745378652	0,090088588	FNBP4;TCERG1;RBM2
0	0	0,472679145	0,057042206	HNRNPR
0	0	0,472679145	0,057042206	HNRNPU
0	0	0,472679145	0,057042206	DDI2
0	0	0,59326056	0,071047448	HNRNPK;ATXN1L;HNR
0	0	0,545911126	0,065230001	CRBN;FNTB
0	0	0,625156289	0,074146741	HNRNPK;RNF180;RUV
0	0	0,587268029	0,06766521	HNRNPK;HNRNPU;DLS
0	0	0,620155039	0,071037225	HNRNPK;FUBP1;RUVE
0	0	0,538329027	0,061521435	SART3;HNRNPK
0	0	0,760988676	0,08694752	SEPT10;HNRNPU;LRR
0	0	0,46142488	0,052634076	HNRNPU
0	0	0,738279808	0,08333365	FNBP4;HNRNPK;USP9
0	0	0,695241075	0,077729426	FNBP4;FAM49A;CPSF7
0	0	0,641716721	0,071212808	HNRNPK;TRIM41;RUVI
0	0	0,706148197	0,077783557	HNRNPK;RUVBL1;HNF
0	0	0,749496766	0,081340551	SF3B3;HNRNPU;LRRC
0	0	0,71512343	0,077255134	PCMT1;XPO4;XRCC5;S
0	0	0,450694069	0,048602946	SOS2
0	0	0,450694069	0,048602946	DLST
0	0	0,740994072	0,079392169	FNBP4;SET;SF3B3;HNI
0	0	0,440451022	0,044912461	CRBN
0	0	0,440451022	0,044912461	PGAM5
0	0	0,516795866	0,05166663	HNRNPK;TARDBP
0	0	0,649603742	0,064869009	HNRNPK;RUVBL1;LRR
0	0	0,600925425	0,059844758	SART3;TBL1XR1;PTK2
0	0	0,596302922	0,057332348	HNRNPK;RUVBL1;HNF
0	0	0,50999592	0,04876149	SET;HNRNPU
0	0	0,661750804	0,062887964	HNRNPK;RUVBL1;EIF3
0	0	0,677025151	0,064338544	PCMT1;RBM28;PCYT1
0	0	0,559033989	0,053053149	SF3B3;HNRNPU;SMAF
0	0	0,591750991	0,054924717	RBM28;RUVBL1;HNRN
0	0	0,591750991	0,054924717	HNRNPU;NUPL1;GIGY
0	0	0,503372596	0,046026576	HNRNPK;RUVBL1
0	0	0,638895988	0,05823849	HNRNPK;SP1;FUBP1;Y
0	0	0,613286233	0,054240876	HNRNPK;WDR37;GAP\
0	0	0,496919102	0,043451287	APC;TARDBP

0	0	0,496919102	0,043451287	RUVBL1;TERF2IP
0	0	0,412337127	0,035578321	HNRNPU
0	0	0,665402402	0,056661365	KCMF1;PRKAB2;KIF5C
0	0	0,490628986	0,04102574	HNRNPK;RUVBL1
0	0	0,490628986	0,04102574	DFFA;TARDBP
0	0	0,40374677	0,032959673	USP9X
0	0	0,40374677	0,032959673	DKK3
0	0	0,40374677	0,032959673	FNBP4
0	0	0,484496124	0,038740718	PCMT1;HNRNPU
0	0	0,56999544	0,044310889	PSMD11;HNRNPK;HNF
0	0	0,47851469	0,036587613	CBX5;BACH1
0	0	0,65142336	0,048283898	PCMT1;HNRNPK;ANGI
0	0	0,387596899	0,028333098	ARHGEF5
0	0	0,387596899	0,028333098	TTC9
0	0	0,387596899	0,028333098	XRCC5
0	0	0,387596899	0,028333098	HNRNPU
0	0	0,561734637	0,040658795	HNRNPK;TPM3;G3BP1
0	0	0,523779594	0,037787279	HNRNPU;DLST;WDFY4
0	0	0,672911283	0,047306155	RBM28;HNRNPK;TBL1
0	0	0,672911283	0,047306155	MAP2K1;HNRNPK;RXF
0	0	0,37999696	0,02628977	HNRNPU
0	0	0,681080813	0,046329416	HNRNPK;TBL1XR1;FUI
0	0	0,46142488	0,030842055	DYRK1A;HNRNPU
0	0	0,372689326	0,024405702	E2F1
0	0	0,50999592	0,032682995	HNRNPK;HSPA4L;HNR
0	0	0,365657452	0,022667239	TET3
0	0	0,505561173	0,031140554	PCMT1;HNRNPU;PGAI
0	0	0,358886018	0,021062031	HNRNPU
0	0	0,563367586	0,031303613	HNRNPK;HNRNPU;HN
0	0	0,352360817	0,019578896	HNRNPU
0	0	0,34606866	0,018207693	GATAD2B
0	0	0,33999728	0,016939216	HNRNPU
0	0	0,639598844	0,030300849	SEPT10;SET;HNRNPK;
0	0	0,572803299	0,027067104	HNRNPK;RUVBL1;HNF
0	0	0,334135258	0,015765096	HNRNPK
0	0	0,421300977	0,019644887	PCMT1;HNRNPK
0	0	0,544377667	0,024675186	SET;HSPH1;HSPA4L;HI
0	0	0,328471948	0,014677716	PPP2CA
0	0	0,416770859	0,018575185	HNRNPU;GATAD2B
0	0	0,322997416	0,013670136	IGBP1
0	0	0,322997416	0,013670136	HNRNPU
0	0	0,322997416	0,013670136	SF3B3
0	0	0,407996736	0,016611111	HNRNPU;RAB11A
0	0	0,559033989	0,022483905	CPSF7;HNRNPK;HNRN
0	0	0,40374677	0,015710072	HNRNPK;HNRNPU
0	0	0,312578145	0,011869611	SMC1A
0	0	0,307616587	0,011065607	GIGYF2
0	0	0,615233173	0,021034384	RBM28;HNRNPK;CAPZ
0	0	0,391512019	0,013295049	CREBBP;HNRNPU
0	0	0,298151461	0,009625916	XRCC5
0	0	0,298151461	0,009625916	PCMT1
0	0	0,481486831	0,015015547	CAPRIN1;RUVBL1;HNF
0	0	0,293634015	0,008981752	C1QBP

0	0	0,47851469	0,014374217	NPAT;HNRNPK;RUVBL
0	0	0,376307669	0,010651361	CRBN;HNRNPU
0	0	0,376307669	0,010651361	CAPZB;HNRNPU
0	0	0,280867318	0,007308205	HNRNPU
0	0	0,542635659	0,013178163	PCMT1;HNRNPK;RUVE
0	0	0,242248062	0,00349166	JAKMIP2
0	0	0,425930658	0,005963636	HNRNPK;USP9X;HNRN
0	0	0,236339573	0,003060493	CDC73
0	0	0,23071244	0,002684343	C1ORF115
0	0	0,212965329	0,001704316	HNRNPK
0	0	0,348140927	0,002509791	HNRNPK;HNRNPU;TAF
0	0	0,203998368	0,001318545	LRRC40
0	0	0,17303433	0,000451663	SORD

3;HNRNPU;DLST;CTDSPL2;ESR1;PCMT1;DDX19B;HNRNPK;TBL1XR1;SIN3A;RBBP5;RUVBL1
INRNPR;GIGYF2;PHF8;HSPH1;RBBP5;RUVBL1;CEP170;SNRPD3;PTGFRN;RBM7;KPNA1;FNBP4;MAP2
'U;HNRNPR;LNPEP;SMARCA1;SMC1A;ADD3;GTPBP4;CDC73;SMAD7;C20ORF112;BCLAF1;HNRNPK;S
RNPU;HNRNPR;MED17;PHF8;CAPZB;RUVBL1;G3BP1;AP2M1;FNBP4;SEPT10;CPSF7;CRBN;CSNK1A1
TN2;HNRNPU;DLST;HNRNPR;GIGYF2;CDC73;PCMT1;NPAT;CAPZB;G3BP1;ATXN1L;CEP170;SNRPD3;
3;ROCK1;LUZP1;HNRNPU;LRRC40;HNRNPR;ACTN4;HCFC2;SMC1A;GIGYF2;GATAD2B;HK2;PCMT1;DI
EK;GAPVD1;SMARCA1;SMC1A;CDC42BPA;HNRNPK;RUVBL1;SNRPD3;TARDBP;RAPGEF6
NPU;DLST;HNRNPR;PDHB;SMC1A;HNRNPK;SIN3A;RUVBL1;CCDC6;TERF2IP;HNRNPC;TARDBP
4;ASB7;CEP170;GAPVD1;HCFC2
'K;XPO4;ROCK1;GPAM;RUVBL1;HSPA4L;FAM129A;HNRNPU;TARDBP;DYNLL2
1;CAPRIN1;RUVBL1;ZNF318;G3BP1;HNRNPU;HOOK3;C12ORF55
LST;HNRNPR;BCLAF1;CAPZB;PABPN1;KIF5C;RUVBL1;ATXN1L;SNRPD3;MYH10;MAP3K2;XRCC5;TPM
HNRNPU;LRRC40;DEK;SMARCA1;HNRNPK;RBBP5;SUB1;FUBP1;RUVBL1;SNRPD3;TARDBP
IRNPU;HNRNPR;AFF4;AFF1;PPP2CA;BCLAF1;CAPZB;KIF5C;SIN3A;RBBP5;RUVBL1;CEP170;ZNF148;
TPM3;TMOD3;HNRNPU;DLST;ACTN4;PDHB;PTP4A1;HNRNPK;MYO1C;CAPZB;ZNF629;SP1;CAPZA1;C
;HNRNPU;HNRNPR;HK2;GATAD2B;HNRNPK;RBBP5;FUBP1;RUVBL1;SNRPD3;HIVEP2;HNRNPC;TARD
T;HNRNPK;NSD1;RUVBL1;HNRNPU;SOSTDC1;HNRNPR;AFF4;AFF1
RBBP5;SUB1;KLHL7;CAPRIN1;RUVBL1;NOS1AP;IRS4;GIGYF2
D3;RUVBL1;DLST;SMC1A;TARDBP;GATAD2B;PHF8
TPM3;TERF2IP;DLST;ACTN4;GAPVD1;MYH10;JAKMIP2
IPU;CEP170;TARDBP;PIAS1
A;XRCC5;ZNF318;HNRNPR;TARDBP;GATAD2B
AM114A1;TPM3;HSPA4L;GFPT1;HNRNPU;ACTN4;NUDT5;ADD3;SORBS3;HK2;PCMT1;SART3;KIF5C;AT
U;DEK;SMARCA1;CDC42BPA;DYNLL2;SMAD7;XPO4;FUBP1;RUVBL1;SNRPD3;TARDBP
'U;DLST;HNRNPR;PDHB;ZBTB44;GAPVD1;GATAD2B;HNRNPK;CAPZB;CAPZA1;HNRNPC;TARDBP
1;HNRNPU;HNRNPR;GAPVD1;GIGYF2;GATAD2B;HNRNPK;RXRA;IGF2BP1;CCDC6;SNRPD3;HNRNPC
VD3;RUVBL1;GFPT1;HNRNPU;HNRNPR;HELZ;HNRNPC;ZNF148;MAPRE2
X;HNRNPU;ZBTB44;PCMT1;DIAPH2;ADAMTS2;HNRNPK;CAPZB;CAPZA1;RUVBL1;CCDC6;ZNF687;TA
;CRBN;FNDC3B;HNRNPU;MTSS1L;GIGYF2;HNRNPK;XPO4;SIN3A;CAPRIN1;RUVBL1;G3BP1;TARDBP
'U;LRRC40;DLST;DEK;SMC1A;CDC42BPA;GATAD2B;MED17;PHF8;HNRNPK;XPO4;TBL1XR1;SUB1;KLH
'U;LRRC40;AFF4;PCMT1;DDX19B;CAPZB;CAPZA1;RUVBL1;PDCD4;CEP170;UBE2K
'U;HNRNPR;DEK;CDC42BPA;DYNLL2;HSPH1;XPO4;RBBP5;RUVBL1;SNRPD3;TARDBP;RAD9A
INRNPR;GIGYF2;PHF8;PPP2CA;PCMT1;RBBP5;RUVBL1;CEP170;SNX8;FNBP4;XRCC5;TPM3;SMC1A;I
3;CAPRIN1;FUBP1;G3BP1;HNRNPU;HNRNPR;HNRNPC;TARDBP;S100PBP;GATAD2B
RNPU;HOOK3;GAPVD1;DYNLL2;GIGYF2;PCMT1;RNF214;ACAP2;CAPZB;FUBP1;CAPZA1;RUVBL1;G3E
INRNPU;DLST;SNRPD3;TARDBP;JAKMIP2
BP1;RBBP5;KLHL7;RUVBL1;NOS1AP;YLPM1;CCDC6;HNRNPU;MED17
INRNPU;HNRNPR;PTPN11;CDC42BPA;FAM107B;HNRNPK;TBL1XR1;SP1;SIN3A;CARM1;RUVBL1;TERF
RMPD4;SH3KBP1;CAPZA1;RUVBL1;HNRNPU;RAB3GAP2;SNRPD3;JAKMIP2
3;ROCK1;XRCC5;HNRNPU;MED17;PHF8;HNRNPK;XPO4;TBL1XR1;SP1;FUBP1;KLHL7;RUVBL1;TMEM1
1;HSPA4L;HNRNPU;DLST;HNRNPR;TARDBP
FIP2;HSPA4L;HNRNPU;HNRNPR;GIGYF2;PCMT1;NPAT;RNF214;KIF5C;RBBP5;TRPS1;RUVBL1;N4BP2
PU;MAPRE3;RBM12;TARDBP;MED17
;MYPN;CDC73;PHF8;PCMT1;PABPN1;TRPS1;RUVBL1;SNRPD3;CPSF7;SEPT11;CRBN;LRRC40;DEK;G
INRNPR;MED17;PHF8;NXF1;MED14;RUVBL1;CEP170;ZNF148;ZC3H14;KPNA1;SEPT10;CREBBP;LRR
CSNK1A1;HNRNPU;HNRNPR;PROX1;GAPVD1;DYNLL2;BCLAF1;ITCH;CAPZB;CAPZA1;TRA2B;NRIP1;S
RCC5;ST13;TPM3;HNRNPU;HNRNPR;ACTN4;SMC1A;AFF4;PAPSS2;GATAD2B;PHF8;HNRNPK;MAT2A;
CRBN;XRCC5;HNRNPU;DLST;NUDT5;GATAD2B;PCMT1;HNRNPK;RAP1A;RUVBL1;TRIM24;STRN;TRIP
;TPM3;SP1;FUBP1;RUVBL1;HNRNPU;HNRNPR;TARDBP;GIGYF2
;XRCC5;TMOD3;HNRNPU;DLST;PDHB
INRNPU;LRRC40;HNRNPR;GAPVD1;DYNLL2;PUM2;HNRNPK;CAPRIN1;SERBP1;RUVBL1;G3BP1;PGA
T;PDHB;GATAD2B;PHF8;PCMT1;HNRNPK;TBL1XR1;SP1;RUVBL1;CCDC6;TARDBP;APPL1

3;SH3KBP1;HNRNPU;STRN;CEP170;SNRPD3;MYH10;DYNLL2
l;XRCC5;SP1;RUVBL1;HNRNPU;CEP170;HNRNPC;TARDBP;GATAD2B
3;HNRNPU;HNRNPR;SNRPD3;ACTN4;HNRNPC;TARDBP;CDC73;PHF8
NPU;ZFP91;HNRNPR;CEP170;HNRNPC;SMC1A;TARDBP;MED17
;CREBBP;CRBN;RUVBL1;HNRNPU;DLST;PDHB;TARDBP
3;CAPRIN1;G3BP1;HNRNPU;DLST;SNRPD3;PDHB;SMNDC1
RNPU;RASSF8;HNRNPR;MAT2B;PFAS;HK2;PCMT1;HSPH1;XPO4;CAPZB;MAT2A;RBBP5;CAPZA1;SER
l1;DLST;CDC42BPA;MED17;PCMT1;MED14;SP1;MFSD5;RUVBL1;SNRPD3;TARDBP
U;HNRNPR;RSF1;HK2;PCMT1;ALDH6A1;ADAMTS2;HNRNPK;XPO4;TBL1XR1;RUVBL1;PGAM5
U;LRRC40;HNRNPR;DEK;CDC42BPA;GATAD2B;RAB11A;PHF8;HNRNPK;TBL1XR1;FUBP1;SP4;RUVBL
NPK;CAPZB;HNRNPU;CEP170;ZNF148;SMC1A;TARDBP;GIGYF2;GATAD2B;APPL1
RP1;HNRNPU;HNRNPR;SMC1A;GIGYF2;GATAD2B;PIAS1;MED14;HNRNPK;MYO1C;SP1;SIN3A;RUVBL
;HNRNPU;HNRNPR;ADD3;PPP2CA;HNRNPK;APC;RUVBL1;CCDC6;HNRNPC;TARDBP
C3;HNRNPU;HNRNPR;GATAD2B;PPP2CA;HNRNPK;KIF5C;SP1;RUVBL1;TARDBP;KPNA1
14;HNRNPK;G3BP1;HNRNPU;HNRNPR;TARDBP;MED12L;MED17
U;HNRNPR;ESR1;GATAD2B;PHF8;HNRNPK;NFIA;TBL1XR1;PAPOLG;SUB1;ZNF318;HNRNPC
NRNPK;XRCC5;ANO6;GPC5;TARDBP;AFF4
F1BP1L;CPSF7;ST13;TPM3;TMOD3;HNRNPU;HNRNPR;ACTN4;PHF8;ENAH;PCMT1;HNRNPK;NFIA;CA
;PDHB;DYNLL2;GATAD2B;PPP2CA;PCMT1;ACAP2;HNRNPK;SP1;TRPS1;RUVBL1;NOS1AP;HNRNPC;T
1;HNRNPR;SMC1A;CDC42BPA;PTP4A1;HNRNPK;KIF5C;SIN3A;RBBP5;FUBP1;KLHL7;RUVBL1;ANKRD
NRNPU;ACTN4;SMARCA1;SMC1A;CDC42BPA;DYNLL2;AFF4;GATAD2B;AFF1;HNRNPK;NFIA;TBL1XR1;
CTN2;PALM2;HNRNPU;HNRNPR;PFAS;PAPSS2;PCMT1;SNX1;HSPH1;SART3;MAT2A;RUVBL1;UBXN7;
K;RUVBL1;HSPA4L;PDCD4;HNRNPU;KIF1B;TARDBP;JAKMIP2
NPU;HNRNPC;SMC1A
;HNRNPU;DLST
CO2;HNRNPU;ZBTB34;ACTN4;SMARCA1;SMC1A;DYNLL2;HNRNPK;ZZZ3;SP1;RBBP5;FUBP1;RUVBL1
l3;TMOD3;DYRK1A;HNRNPU;HNRNPR;GTF2H1;ACTN4;HNRNPK;MYO1C;CAPZB;KIF5C;CAPZA1;TRIP
LST;HNRNPR;PCMT1;BCLAF1;HSPH1;CAPZB;TRPS1;RUVBL1;CEP170;SNRPD3;MYH10;TPM3;PDAP1
NRNPU;DLST;PHAX;AFF4;CDC73;HK2;TXNDC17;PHF8;RBM3;HSPH1;RUVBL1;SNRPD3;DIP2B;ZNF687
;ST13;RUVBL1;SMC1A;TARDBP;GATAD2B
K1;XRCC5;CALCOCO2;YLPM1;HNRNPU;HNRNPR;DEK;PAPSS2;GATAD2B;PHF8;HNRNPK;TBL1XR1;F
A;SP1;RUVBL1;HSPA4L;HNRNPU;HNRNPR;MAT2B;CGGBP1
NRNPU;HNRNPR;PHAX;ZNF687;TARDBP;HIF1A
;HNRNPR;PHF8;MED14;RIC8B;RBBP5;RUVBL1;CEP170;SNRPD3;ZNF148;KPNA1;FNBP4;MAP2K1;XRC
HNRNPU;ADD3
P1;FUT11;HNRNPU;SMARCA1;GATAD2B;PHF8;PCMT1;HNRNPK;CAPZB;RBBP5;CAPZA1;RUVBL1;TA
13;HNRNPU;LRRC40;HNRNPR;ACTN4;GAPVD1;SMC1A;GATAD2B;FBXO30;HSPH1;HNRNPK;FUBP1;R
U;DLST;HNRNPR;TARDBP;GATAD2B
FA;SMC1A;GIGYF2;PAPSS2;GATAD2B;PHF8;ENAH;HNRNPK;SP1;RUVBL1;SNRPD3;ZNF148
K;CRBN;SIN3A;RUVBL1;HNRNPU;HNRNPR;GTF2H1;SRGAP2
TBL1XR1;FUBP1;RUVBL1;HNRNPU;NBEAL1;HNRNPR;TARDBP
K;HNRNPU;DLST;DIP2B;CDC42BPA;TARDBP
B;XRCC5;CAPZA1;RUVBL1;ATXN1L;HNRNPU;HNRNPR;TARDBP;DYNLL2
PU;DLST;TARDBP
l2;CAPZA1;RUVBL1;ATXN1L;HNRNPU;HNRNPR;STRN;PGAM5
1;XRCC5;TMOD3;HNRNPU;DLST;ACTN4;TARDBP;IL18BP
DYNLL2;GIGYF2;GATAD2B;CDC73;PHF8;HNRNPK;XPO4;RBBP5;DDI2;RUVBL1;JMY;UBXN7;SNRPD3;K
l4;CDC42BPA;GATAD2B;RAB11A;PCMT1;RNF214;HNRNPK;SP1;SALL4;CAPZA1;RUVBL1;HIVEP2;TARD
5;XRCC5;MSL2;HNRNPU;DLST;HNRNPR;DYNLL2;GATAD2B;PHF8;ADAT2;HNRNPK;TBL1XR1;SP1;SIN
GEF6;CTGF;DKK3;JAKMIP2
R;PCDHA10;TNS1;STXBP5L
U;HNRNPR;PCMT1;MED14;SIN3A;RBBP5;RUVBL1;ZNF687;NCOA2;CRBN;TPM3;ACTN4;SMARCA1;SM
PK;XPO4;DIRAS2;RUVBL1;TARDBP;PAPSS2

PK;SP1;DLST;SNRPD3;TARDBP
/F2;PAPSS2;SNX1;RXRA;RBBP5;RUVBL1;ATXN1L;ZC3H14;KPNA1;FNBP4;SEPT10;MAP2K1;XRCC5;LF
33;CRBN;HNRNPU;LRRC40;CBFA2T2;SMC1A;GATAD2B;CDC73;RUNX1;PHF8;HNRNPK;TBL1XR1;SP1;I
'1;SUB1;RUVBL1;TRIM3;LRRC40;AFF4;DYNLL2
TPM3;USP9X;XRCC5;HNRNPU;DLST;SMC1A;MYH10
PK;NUFIP2;CRBN;RUVBL1;DYRK1A;HNRNPU;HNRNPC;TARDBP;MYH10;RAB11A
RNPR;ACTN4;PDHB;SMC1A;GATAD2B;CNOT6;HNRNPK;RXRA;CAPZB;SIN3A;RBBP5;FUBP1;HNRNPC;
L;HNRNPU;DLST;SMC1A;DYNLL2;MED17;MED14;HNRNPK;TBL1XR1;SP1;RUVBL1;SNRPD3;TARDBP
R1;HNRNPU;SMC1A;TARDBP
B;HSPH1;HNRNPK;XRCC5;RUVBL1;CCDC6;HNRNPU;SMC1A;TARDBP;GATAD2B
XR1;XRCC5;KIAA0355;USP9X;RUVBL1;TFEB;CALM1;SMC1A;DYNLL2;RAB11A
HNRNPR;PPP2CA;CAPZB;RUVBL1;SNRPD3;SRGAP2;MYH10;CPSF7;SEPT11;USP9X;TPM3;TMOD3;AC
TPM3;RUVBL1;ATXN1L;HNRNPU;SNRPD3;GAPVD1
HNRNPU;CTNNB1;APCDD1;TNRC6B
RNPU;DLST;TRIP12;HNRNPC;TARDBP
RCC5;TMOD3;HNRNPU;ZFP91;HNRNPR;DEK;GATAD2B;PHF8;PCMT1;BCLAF1;HNRNPK;SP1;RBBP5;F
B;XPO4;C1QBP;RUVBL1;HNRNPU;ACTN4;HNRNPC;DYNLL2
;HNRNPK;CREB1;XRCC5;SESTD1;RUVBL1;HNRNPU;DLST
RNPU;CTNNB1
NPU;HNRNPC;TARDBP
RNPR;SMC1A;GATAD2B;RAB11A;PIAS1;PCMT1;HNRNPK;SP1;CARM1;RUVBL1;ATXN1L;TARDBP
'U;HNRNPR;ACTN4;SMC1A;HNRNPK;SP1;FUBP1;TRPS1;RUVBL1;CCDC6;SNRPD3;HNRNPC;ZNF148;T
33;FUBP1;RUVBL1;HNRNPU;DLST;HNRNPR;HNRNPC;TARDBP;GATAD2B
APVD1
PK;CAPZB;TPM3;CAPZA1;HNRNPU;ACTN4;TARDBP
PU;DLST;HNRNPR;PDHB;SMARCA1;SMC1A;GATAD2B;PCMT1;HNRNPK;SP1;SIN3A;RUVBL1;CEP170;T
EF10;HNRNPK;TBL1XR1;SIN3A;HNRNPR;HNRNPC;ZBTB44;ZNF148;SMC1A;GATAD2B
PZB;CAPZA1;CARM1;RUVBL1;HNRNPU;TARDBP;CDC73;JAKMIP2
PU;LRRC40;HNRNPR;ACTN4;GAPVD1;GIGYF2;GATAD2B;PHF8;PIAS1;NXF1;HNRNPK;MYO1C;CAPZB;
HNRNPU;LRRC40;DLST;HNRNPR;PDHB;SMC1A;GATAD2B;HNRNPK;NKRF;SP1;RBBP5;RUVBL1;CEP1
YLPM1;HNRNPU;GTPBP4;GATAD2B;HNRNPK;NKRF;TBL1XR1;SP1;KLHL7;CAPRIN1;RUVBL1;G3BP1;S
;CRBN;HNRNPU;DLST;HNRNPR;SMC1A;ZBTB4;DYNLL2;GATAD2B;HNRNPK;MAF;CAPZB;UBN2;TRPS1
L1XR1;RUVBL1;HNRNPU;RASSF8;SNRPD3;TARDBP;GIGYF2;RAB11A
P1;HNRNPU;HNRNPR;GIGYF2;PHF8;TIAL1;SART3;CAPZB;RBBP5;RUVBL1;CEP170;SNRPD3;KPNA1;F
7;SF3B3;XRCC5;HNRNPU;HNRNPR;GATAD2B;CDC73;HNRNPK;XPO4;TBL1XR1;RBBP5;KLHL7;TRIP12
I;ZNF687;GAPVD1;TARDBP;GATAD2B
PU;SMARCA1;CDC42BPA;TARDBP;KPNA1
XRCC5;HNRNPU;DLST;ZFP91;SMC1A;GATAD2B;DDX19B;HNRNPK;SART3;RUVBL1;TARDBP;TECPR2;
2B;MED17;PHF8;PIAS1;MED14;CNOT6;DIAPH2;HNRNPK;TBL1XR1;SP1;RBBP5;RUVBL1;HIVEP2;HNRN
;CRBN;LUZP1;HNRNPU;HNRNPR;SMC1A;GIGYF2;GATAD2B;PIAS1;TIAL1;HNRNPK;TBL1XR1;SP1;RU
OCO2;RUVBL1;HNRNPU;SPATA2;SRGAP2;SMAD7
3;CREBBP;DFFA;HSPA4L;HNRNPU;DLST;HNRNPR;SMARCA1;SMC1A;SORBS3;MED12L;GATAD2B;PHI
OXP2;HNRNPK;ZZZ3;CAPZB;CAPRIN1;FUBP1;CAPZA1;RUVBL1;G3BP1;HNRNPC;TARDBP
HNRNPU;HNRNPR;SMC1A;GATAD2B;PHF8;NPAT;MED14;HNRNPK;CAPZB;CAPZA1;ZNF318;CEP170;H
'K;KLHL7;RUVBL1;HNRNPU;SMC1A;TARDBP
HNRNPR;PDHB;PHF8;PPP2CA;PCMT1;UBN2;FNBP4;MEF2A;DFFA;CRBN;ACTN4;SMC1A;DYNLL2;HNRI
R;XPO4;CAPZB;MB;G3BP1;CEP170;ZNF687;MYH10;FNBP4;XRCC5;TPM3;TMOD3;ACTN4;SMARCA1;CI
PK;XRCC5;FUBP1;HNRNPU;DLST;STRN;PELO
3B3;ATL3;HNRNPU;HNRNPR;CAPZB;RBBP5;KIF5C;C1QBP;RUVBL1;CEP170;CLSPN;ZNF687;RBM12;S
HNRNPR;SMC1A;GIGYF2;HNRNPK;SP1;RUVBL1;CEP170;ZNF148;RBM7;LPHN2;TARDBP
USP9X;YLPM1;GIGYF2;GATAD2B;PCMT1;HNRNPK;SP1;SERBP1;RUVBL1;CEP170;TARDBP;ZC3H14;SI
TMTC3;HNRNPU;SNRPD3
RNPU;CRK;GATAD2B;JAKMIP2

;C5;TPM3;TMOD3;YLPM1;HNRNPU;HNRNPR;ACTN4;PDHB;PCMT1;HNRNPK;MYO1C;CAPZB;CAPZA1;
RNPU;HNRNPR;AFF4;MED14;SART3;CAPZB;RUVBL1;G3BP1;ZNF687;FNBP4;SEPT10;CPSF7;CSNK1A
J;LRRC40;DLST;GAPVD1;SMC1A;DYNLL2;GATAD2B;HK2;PHF8;DIAPH2;RIC8B;HNRNPK;SIN3A;RBBP5
INRNPU;DLST;HNRNPR;PTPN11;GAPVD1;GATAD2B;PHF8;NPAT;CAPZB;KIF5C;RUVBL1;PGAM5;CEP17
;HNRNPU;SPATA2;TARDBP;PHF8;KPNA1
-0;GAPVD1;CBFA2T2;SMC1A;CDC42BPA;GATAD2B;HNRNPK;RBBP5;RUVBL1;CEP170;TARDBP;RAF1;U
7;NEK7;HNRNPU;XIAP;HNRNPR;HNRNPC;TARDBP;MTDH
7;HNRNPK;APC;FUBP1;RUVBL1;HNRNPU;RAB11FIP5
S2;GATAD2B;MED17;HNRNPK;ZZZ3;SIN3A;FUBP1;CAPRIN1;RUVBL1;CCDC6;CEP170;SNRPD3;TARDE
;HNRNPU;RASSF8;DLST;SMC1A;DYNLL2;AFF4;GATAD2B;PCMT1;HNRNPK;SART3;SP1;RBBP5;RUVBL
7;SF3B3;YLPM1;HNRNPU;LRRC40;DEK;GAPVD1;BACH1;GATAD2B;TIAL1;MED14;HNRNPK;ZZZ3;FUBF
;HNRNPU;HAUS3;HNRNPR;GAPVD1;SMC1A;GATAD2B;HNRNPK;CAPZB;KIF5C;TBL1XR1;SP1;C1QBP;
7;RUVBL1;HNRNPU;TARDBP
L3;HNRNPU;DLST;CALM1
PU;LRRC40;DLST;GAPVD1;GIGYF2;PUM2;PCMT1;HNRNPK;CDC37;CAPRIN1;RUVBL1;CEP170;TARDB
M1;HNRNPU;LRRC40;GAPVD1;GATAD2B;PCMT1;DDX19B;HNRNPK;APC;TBL1XR1;KLHL7;RUVBL1;PD
INRNPR;RBM3;RBBP5;RUVBL1;G3BP1;DAG1;CEP170;ZNF148;PPARGC1B;SEPT10;CPSF7;XRCC5;SM
F20;HNRNPU;HNRNPR;PHF8;PPP2CA;PCMT1;RBBP5;RUVBL1;SNRPD3;KPNA1;FNBP4;DEK;GAPVD1;
FKFB3;PSMD11;HNRNPU;HNRNPR;NUDT5;PFAS;HK2;PCMT1;HSPH1;MAT2A;TRA2B;RUVBL1;G3BP1;
6;HNRNPK;CAPZB;CAPZA1;HNRNPU;BACH1
;SF3B3;XRCC5;YLPM1;HNRNPU;HNRNPR;NUDT5;GTPBP4;HNRNPK;TBL1XR1;KIF26B;CAPRIN1;DDI2;
3L1;CCDC6;HNRNPU;SMC1A;TARDBP
C3;CALM1;SNX8
R;ACTN4;MYH10
4L;HNRNPU;HNRNPR;PDHB;PFAS;HK2;PPP2CA;PCMT1;HSPH1;CAPZB;ALDH2;MAT2A;C1QBP;RUVBL
RNPU;PGAM5;TARDBP;FAM117B
1;TARDBP
L;PRUNE2;HNRNPU;DLST;HNRNPR;CBFA2T2;GTPBP4;GATAD2B;KIF5C;RBBP5;MYH10
:40;PCMT1;GLUD2;HSPH1;TBL1XR1;CAPZA1;RUVBL1;ATXN1L;STRN;CEP170;SNRPD3
;HNRNPU;DLST;PCMT1;HNRNPK;RUVBL1;ZNF318;HNRNPC;TARDBP;NUPL1;KPNA1
RAS2;HNRNPU;HNRNPR;STRN;SNRPD3;HNRNPC;AFF1;GATAD2B
MOD3;HSPA4L;HNRNPU;HNRNPR;ACTN4;SMC1A;GATAD2B;LETM1;HNRNPK;CAPZB;CAPZA1;RUVBL
3;FUBP1;RUVBL1;MSL2;HNRNPU;DLST;TARDBP;GATAD2B
C5;RUVBL1;HNRNPU;DLST;HNRNPC;TARDBP
NRNPU;HNRNPR;PHF8;PPP2CA;PCMT1;SART3;XPO4;TRA2B;RUVBL1;G3BP1;SNRPD3;ZNF148;KPNA
PK;TRPS1;HNRNPU
B3;HNRNPU;TARDBP
GFG2;FBXO30
9X;SGIP1;HSPA4L;HNRNPU;HNRNPR;MYPN;HK2;NCKIPSD;ALDH6A1;ATXN7;RUVBL1;NOS1AP;CEP17
CARM1;PDHB;HOOK3
U;GIGYF2;MED17;SNX1;MED14;BCLAF1;RIC8B;XPO4;TRIM3;RUVBL1;SNX8;MYH10;KPNA1;TPM3;GAF
U;XIAP;HNRNPR;ADD3;GATAD2B;PHF8;ADD2;HNRNPK;RUVBL1;STRN;HNRNPC;APPL1
7;HNRNPU;SMC1A;CDC73;GATAD2B;TIAL1;HNRNPK;RUVBL1;PDCD4;TARDBP;UBE2K;KPNA1
XRCC5;TPM3;AGAP2;YLPM1;HNRNPU;ZFP91;HNRNPR;ACTN4;PCMT1;HNRNPK;APC;KIF5C;RUVBL1;
;SF3B3;POGK;RUVBL1;HNRNPU;HNRNPR;HNRNPC;TARDBP
XRCC5;CSNK1A1;HNRNPU;HNRNPR;SMC1A;DYNLL2;GATAD2B;PHF8;PPP2CA;NPAT;C20ORF112;HNI
PM1;HNRNPU;HNRNPR;SMARCA1;CBFA2T2;SMC1A;ZBTB4;GATAD2B;BCLAF1;HNRNPK;SP1;SP4;RU
N20A;CAPZA1;HNRNPU;TRIP12;TARDBP;PHF8
1;PFKFB3;HNRNPU;DLST;HNRNPR;PDHB;GAPVD1;SMC1A;GATAD2B;MED17;HNRNPK;CAPZB;RBBP5
;HNRNPU;HNRNPR;ADAMTS5;HNRNPK;CAPZB;XPO4;SP1;CAPZA1;RUVBL1;PTK2B
T;RAB11A;MED17;PHF8;PCMT1;HNRNPK;SUB1;FUBP1;RUVBL1;SNRPD3;TARDBP
M1;HNRNPU;ACTN4;GAPVD1;AFF4;HNRNPK;ATXN7;FUBP1;HNRNPC;TARDBP
DLST;AFF4;GIGYF2;PAPSS2;AFF1;PHF8;PCMT1;RXRA;RBBP5;UBN2;RUVBL1;ZNF800;SNRPD3;ZC3H1

;F3B3;PSMD11;HNRNPU;DLST;HNRNPR;PDHB;MTDH;PPP2CA;PCMT1;NXF1;CAPZB;RBBP5;TRA2B;RU
'K;TBL1XR1;FUBP1;UBN2;RUVBL1;HNRNPU;TARDBP;PHF8
MOD3;DLST;PDHB
.1;HNRNPU;TARDBP
'K;RUVBL1;DLST
33;TPM3;XRCC5;HNRNPU;HNRNPC;LRIG2;GATAD2B
NRNPU;HNRNPR;PPP2CA;PCMT1;CAPZB;TRPS1;RUVBL1;G3BP1;SNRPD3;MYH10;CRBN;TPM3;XRC(
)11;XRCC5;HNRNPU;LRRC40;DLST;GAPVD1;SMC1A;SORBS3;GATAD2B;MED17;HNRNPK;ZZZ3;TBL1X
;ZNF318;HNRNPU;CEP170;ESR1;GATAD2B
J;LRRC40;HNRNPR;GAPVD1;GIGYF2;PCMT1;HNRNPK;CAPZB;CAPRIN1;SERBP1;RUVBL1;G3BP1;CEF
IRNPR
U;DLST;HNRNPR;PDHB;GIGYF2;PPP2CA;NPAT;RUVBL1;CEP170;ZNF687;KPNA1;FNBP4;CPSF7;CRBN
'U;DLST;XIAP;AFF4;PCMT1;HNRNPK;ZZZ3;TBL1XR1;RUVBL1;CEP170;TARDBP
33;NUFIP2;RBBP5;CAPRIN1;FUBP1;RUVBL1;HNRNPU;NUDT5;TARDBP;GATAD2B
RNPU;HNRNPR;FMN1;PDHB;MTDH;PHF8;PPP2CA;BCLAF1;CAPZB;KIF5C;RUVBL1;IGF2BP1;SNRPD3
)11;LRRC40;GAPVD1;GATAD2B;CDC73;DIAPH2;HNRNPK;SP1;RBBP5;RUVBL1;ANKRD40;TARDBP;UBE
U;HNRNPR;PDHB;PFAS;MTDH;SART3;CAPZB;KIF5C;RUVBL1;G3BP1;SNRPD3;MYH10;USP9X;TPM3;X
40;ACTN4;SMC1A;GIGYF2;GATAD2B;PHF8;HNRNPK;MYO1C;CAPZB;MB;CAPZA1;CAPRIN1;RUVBL1;G
HNRNPU;DLST;AFF4;KPNA1
1;SET;HNRNPK;USP9X;SUB1;CCDC6;HNRNPU;TARDBP
33;RUVBL1;HNRNPU;TARDBP;ZC3H14;DYNLL2;GIGYF2;JAKMIP2
HNRNPU;ZFP91;HNRNPR;SMC1A;GIGYF2;GATAD2B;PHF8;DHX40;HNRNPK;SART3;TBL1XR1;SIN3A;C
;HNRNPU;ADD3;GATAD2B;PCMT1;HNRNPK;FUBP1;RUVBL1;IGF2BP1;HNRNPC;TARDBP
)11;HNRNPK;CRBN;SP1;RBBP5;RUVBL1;PDCD4;GAPVD1;GATAD2B
';HNRNPU;HNRNPR;AFF4;AFF1;PHF8;HNRNPK;SP1;SIN3A;RUVBL1;CCDC6;CEP170;TARDBP
NOS1AP;HNRNPU;HNRNPR;LSM14B;RAB11A;BCMO1
/114A1;XRCC5;HECW2;HNRNPU;LRRC40;HNRNPR;GAPVD1
'5;CDC37;FUBP1;RUVBL1;HNRNPU;SMC1A;TARDBP
.NPU;MTSS1L;HCFC2;CDC42BPA;HNRNPK;VAPA;ATXN7;SUB1;SERBP1;RUVBL1;EPC1;CEP170;SNRP
3;GATAD2B;MED17;MED14;DDX19B;HNRNPK;RBBP5;RUVBL1;STRN;HNRNPC;ZNF148;KPNA1
PK;RXRA;CRBN;TBL1XR1;RUVBL1;HNRNPU;SMC1A;JAKMIP2
NPK;XPO4;FUBP1;SMC1A;TARDBP;SMAD7
INRNPK;RBBP5;FUBP1;RUVBL1;HNRNPU;ZNF687;RBM12;GATAD2B;RAB11A
.1;YLPM1;HNRNPU;ZFP91;CBFA2T2;SMC1A;TARDBP;AP2M1;GATAD2B;KPNA1
\F;HNRNPK;TPM3;TRPS1;RUVBL1;HNRNPU;STRN;ACTN4;SMC1A;TARDBP
3B3;NUFIP2;ST13;HNRNPU;HNRNPR;ACTN4;NUDT5;SMC1A;GATAD2B;HNRNPK;MAT2A;FUBP1;RUVE
NRNPK;RUVBL1;HNRNPU;DLST;TARDBP
K;FUBP1;RUVBL1;KIF1B;MTDH
'R;ADD3
XRCC5;HNRNPU;LRRC40;SMAD9;BACH1;GATAD2B;NPAT;HNRNPK;NFIA;PQLC1;NKRF;FUBP1;RUVBL
1;HNRNPU;ARHGEF5
11;MTMR3;HNRNPK;SP1;CAPZA1;RUVBL1;HNRNPU;SIX1;TARDBP;PHF8
J;GAPVD1;SMC1A;GATAD2B;JAKMIP2;HNRNPK;SP1;FUBP1;RUVBL1;FAM84A;TARDBP;UNKL;CGGBP
IPK;FUBP1;CAPZA1;RUVBL1;HNRNPU;LRRC40;STRN;BSN;PAFAH1B2
UBP1;HNRNPU;TERF2IP;TARDBP;PHF8
I;XRCC5;RUVBL1;TARDBP
U;XIAP;HNRNPR;HNRNPC;TARDBP
92;HNRNPU;FNDC3A;SMC1A;GATAD2B;NXF1;RXRA;SP1;C1QBP;CAPRIN1;RUVBL1;TFAM;TEAD1;ZC3
VBL1;HNRNPU;ZFP91;TARDBP;AFF4;AP2M1;CDC73;AFF1;GATAD2B;KPNA1
.NPK;SF3B3;TBL1XR1;XRCC5;RUVBL1;HNRNPU;HNRNPC;TARDBP
NRNPK;DDI2;RUVBL1;HNRNPU;TARDBP;FBXO30;PHF8
I;ELAVL2;HK2
;SF3B3;ITPK1;HNRNPU;PGAM5;ZNF687

<;RUVBL1;JMY;TERF2IP;SMC1A
NRNPU;CDC42BPA;TARDBP
JZP1;TRIM3;IGF2BP1;HNRNPU;LRRC40;GIGYF2
1;FAM129A;HNRNPU;STRN;CEP170;SLIT2;DYNLL2
RUVBL1;FOXJ2;HNRNPU;LCOR;SNRPD3;HNRNPC;TARDBP;GATAD2B;PHF8
YF2;PUM2;PCMT1;HNRNPK;NFIA;CAPRIN1;SERBP1;RUVBL1;G3BP1;TRIP12;CEP170;TARDBP
PU;ARID5B;DLST;MTSS1L;PDHB;MED17;PHF8;MED14;FUBP1;TRA2B;TARDBP
3B3;PSMD11;CRBN;DCTN2;ST13;HNRNPU;DLST;HNRNPR;SMC1A;PHF8;PPP2CA;ALDH6A1;HNRNPK;
RUVBL1;GAB1;HNRNPU;DLST;CEP170;SNRPD3;SMC1A;MAPRE2
INRNPU;DLST;HNRNPR;TARDBP;GATAD2B
NMT3A;CCDC6;HNRNPU;WDFY3
3L1;HNRNPU;DLST;ZNF148;TARDBP
1XR1;ST13;HNRNPU;XIAP;SNRPD3
C5;HNRNPU;DLST;CGGBP1
;CRBN;XRCC5;HNRNPU;HCFC2;PCMT1;HNRNPK;SNRPD3;TARDBP;KPNA1;MARK1
3B3;HNRNPU;HNRNPR;MAT2B;CDC42BPA;DYNLL2;GATAD2B;FAM133B;PPP2CA;HNRNPK;CAPZB;SP
;HNRNPR;SMC1A;GATAD2B;RUNX1;HNRNPK;PABPN1;TBL1XR1;RUVBL1;ZNF318;HNRNPC;TARDBP
CAPRIN1;KLHL7;HNRNPU;GIGYF2;SULF2
/BL1;HNRNPU;DLST;HNRNPR;TARDBP;GIGYF2
;5;RUVBL1;HNRNPU;HNRNPR;SNRPD3;HNRNPC;TARDBP;KPNA1
XR1;XRCC5;RUVBL1;HNRNPU;HNRNPR;WDFY3;TARDBP;PHF8
;HNRNPR;BMI1;PPP2CA;CAPZB;SIN3A;KIF5C;UBN2;RUVBL1;SNRPD3;ZNF687;MYH10;SEPT10;CRBN;
RNPR;SMC1A;HIF1A;HNRNPK;RUVBL1;TRIP12;HIVEP2;HNRNPC;ZNF687;RBM7;TARDBP
549;CREBBP;HNRNPK;RUVBL1;HNRNPU
;USP9X;HNRNPU;CBFA2T2;FBXO30
L1XR1;RUVBL1;SNRPD3;TARDBP
IPU;TARDBP;ESR1
NPU;DLST;HNRNPR
1;CAPZA1;GIGYF2
14;HNRNPK;APC;SP1;RUVBL1;TRIM3;CCDC6;HNRNPU;TARDBP;MED17
1;FUBP1;RUVBL1;HNRNPU;DEK;SMC1A;TARDBP;MED12L;GATAD2B;PHF8
I;PCMT1;CAPZB;XPO4;SIN3A;RBBP5;RUVBL1;SNRPD3;CLSPN;SRGAP2;KPNA1;FNBP4;MAP2K1;LRR
S4;HNRNPU;HNRNPR;ETFA;PDHB;PFAS;MED17;NXF1;MED14;RUVBL1;IGF2BP1;ZNF687;TSFM;RRM
2;XRCC5;USP9X;HSPA4L;IRS4;HNRNPU;HNRNPR;ACTN4;ESR1;GATAD2B;HSPH1;HNRNPK;C1QBP;IG
IC1A;GIGYF2;GATAD2B;PHF8;PIAS1;HNRNPK;CAPZB;SP1;FUBP1;KLHL7;RUVBL1;ZNF318;PDCD4;PG
;HSPA4L;SORD;HNRNPU;DLST;SMC1A;DPYSL5;SP1;RBBP5;CAPRIN1;RUVBL1
PU;LRRC40;GAPVD1;PCMT1;HNRNPK;RUVBL1;PDCD4;SNRPD3;SNX8;KPNA1;APPL1
PM3;HSPA4L;HNRNPU;LRRC40;HNRNPR;GAPVD1;DDX19B;HNRNPK;CAPZB;CAPZA1;CAPRIN1;RUVE
PSF7;SF3B3;YLPM1;HNRNPU;HNRNPR;GTPBP4;GATAD2B;NXF1;HNRNPK;CAPRIN1;SERBP1;G3BP1;
B;HNRNPK;XRCC5;RUVBL1;HNRNPU;CEP170;ACTN4;SMC1A;ESR1
3;CRBN;TBL1XR1;SP1;RUVBL1;HNRNPU;HNRNPR;GTF2H1;PHF8
PU;DLST;HNRNPR;DEK;GATAD2B;PHF8;HNRNPK;FUBP1;SNRPD3;HNRNPC;TARDBP
P1;HNRNPU;HNRNPR;CDC73;PPP2CA;PCMT1;NXF1;SART3;CAPZB;SNRPD3;KPNA1;XRCC5;ACTN4;D
NPR;DEK;RBM7;TARDBP;MYH10;PFAS
i1;RUVBL1;HNRNPU;TARDBP
1;DEK;BMI1;SMC1A;GNL1;GATAD2B;PCMT1;HNRNPK;TBL1XR1;MB;RUVBL1;HNRNPC;PHACTR4
2;RUVBL1;HNRNPU;DLST;HNRNPC;ZNF148;SMC1A;TARDBP;UBE2K;GATAD2B
3;RUVBL1;HNRNPU;HNRNPC;ZNF687;TARDBP;AFF4;GATAD2B;MED17
IL;HNRNPU;DLST;HNRNPR;MAT2B;PCMT1;HSPH1;CAPZB;XPO4;MAT2A;KIF5C;RUVBL1;CRBN;ST13;T
HF20;ANGEL2;HNRNPU;SMC1A
CRBN;ROCK1;XRCC5;HNRNPU;HNRNPR;GTF2H1;ETFA;PHF8;HNRNPK;XPO4;RRM2B;SP1;RBBP5;RU
3B3;HNRNPU;DLST;HNRNPR;SMARCA1;SMC1A;GTPBP4;GIGYF2;GATAD2B;PHF8;HNRNPK;CAPZB;R
T;SNX8

;CC5;RUVBL1;TFAM;DLST;RSF1;SMC1A
J;HNRNPR;SNRPD3;HNRNPC;TARDBP;AFF4
F3B3;XRCC5;HNRNPU;LRRC40;HNRNPR;GTF2H1;ACTN4;SMC1A;PAPSS2;GATAD2B;PPP2CA;PCMT1;
XRCC5;HNRNPU;PTPN11;GAPVD1;SMARCA1;PFAS;GATAD2B;CDC73;PCMT1;HSPH1;HNRNPK;RBBP
CNKSR3;HNRNPU;HNRNPR
;TPM3;HNRNPU;LHX6
7;SEPT11;SET;SF3B3;TPM3;TMOD3;HNRNPU;HNRNPR;ACTN4;SMC1A;SOD2;HNRNPK;MYO1C;CAPZ
;FUBP1;RUVBL1;GFPT1;HNRNPU;HNRNPR;SMC1A;TARDBP;GATAD2B
;HNRNPU;PHF8;PIAS1;HNRNPK;XPO4;CAPZB;KIF5C;TBL1XR1;SIN3A;RBBP5;CAPRIN1;RUVBL1
BN;ST13;HNRNPU;SMC1A;ADD3;PPP2CA;PCMT1;NXF1;HNRNPK;RRM2B;SP1;UBN2;RUVBL1;CCDC6;
J;HNRNPR;CEP170;SNRPD3;HNRNPC;ZNF148;SRGAP2;TARDBP
MOD3;CAPZA1;RUVBL1;HSPA4L;HNRNPU;ACTN4;MYH10;GIGYF2;PUM2
XN7;SNAP23;RUVBL1;HNRNPU
K;VAPA;CARM1;KIF1B;TARDBP
RNPU;HNRNPR;MED17;MED14;SIN3A;KIF5C;C1QBP;RUVBL1;CEP170;SNRPD3;FNBP4;XRCC5;ITPK1;
UB1;HNRNPU;NUDT5;SMTNL2;MYH10;DYNLL2;GATAD2B;CKS1B
3B3;DCTN2;LUZP1;HNRNPU;SMC1A;GIGYF2;GATAD2B;PHF8;DDX19B;HNRNPK;TBL1XR1;SIN3A;FUB
NPU;CEP170;TARDBP;HK2
BP1;HNRNPU;HNRNPR;MBNL3
;CEP170
B3;XRCC5;SUB1;G3BP1;SERBP1;HNRNPU;HNRNPR;SNRPD3;DEK;SMC1A
APRIN1;DDI2;RUVBL1;G3BP1;HNRNPU;HNRNPR;CEP170;ZNF687;TARDBP
;HNRNPU;DLST;GIGYF2;ENAH;HNRNPK;CAPZB;RUVBL1;CEP170;TARDBP;UBE2K
U;HNRNPR;ACTN4;HCFC2;SMC1A;GATAD2B;PCMT1;FUBP1;CEP170;HNRNPC;RAF1
J;DLST;TARDBP
IPK;HSPA4L;HNRNPU;HNRNPR;NPTXR;TARDBP;MTDH
1;RUVBL1;HNRNPU;SNRPD3;HOOK3;CDC73;RAB11A
1;RUVBL1;HNRNPU;ZNF148;TARDBP;GATAD2B;RUNX1
5;HNRNPU;ZFP91;GATAD2B;DDX19B;HNRNPK;NFIA;SP1;RBBP5;FUBP1;RUVBL1;HNRNPC;TARDBP;K
3B3;CCDC6;DLST;GIGYF2
IPK;XRCC5;SUB1;RUVBL1;HNRNPU;TARDBP
SET;XRCC5;HNRNPU;HNRNPR;GAPVD1;SMC1A;DYNLL2;PHF8;ACAP2;HNRNPK;SP1;FUBP1;RUVBL1;
IPK;SF3B3;CRBN;RBBP5;CAPZA1;NOS1AP;HNRNPU;HNRNPR;TARDBP;KPNA1
C;TBL1XR1;RUVBL1;HNRNPU;HNRNPR;HNRNPC;TARDBP;ZBTB4
A1L1;SORT1;UBN2;RUVBL1;HNRNPU;DLST;XIAP;SORBS3
J;DLST;PPWD1;HNRNPR;PDHB;PCMT1;HNRNPK;XPO4;TRA2B;RUVBL1;SNRPD3;HNRNPC
N2;ST13;HSPA4L;YLPM1;SORD;HNRNPU;HNRNPR;ACTN4;SMC1A;GIGYF2;GATAD2B;PPP2CA;HNRN
;DYNLL2;PHF8;SMAD7;HNRNPK;TBL1XR1;SP1;RBBP5;PAPOLG;RUVBL1;SNRPD3;TARDBP;KPNA1
U;HNRNPR;GIGYF2;PHF8;CAPZB;RBBP5;RUVBL1;G3BP1;CEP170;SNRPD3;KPNA1;FNBP4;SEPT10;P
K;PRUNE2;HNRNPU;HK2
RNPU;HNRNPR;GTF2H1;GAPVD1;RAB11A;PHF8;SMAD7;HNRNPK;SP1;SIN3A;CAPRIN1;CEP170;SNRP
F3B3;CSNK1A1;HNRNPU;HNRNPR;YOD1;SMC1A;GIGYF2;PHF8;PCMT1;HNRNPK;SP1;KLHL7;CAPRIN
N;TBL1XR1;CDC37;RUVBL1;TARDBP;GATAD2B;RAB11A
RNPU;HNRNPR;MED17;SART3;RUVBL1;G3BP1;CEP170;SNRPD3;SNX8;ZC3H14;MEF2A;CREBBP;USF
3;XRCC5;NEK7;HNRNPU;LRRC40;HNRNPR;GTF2H1;SMC1A;DYNLL2;GATAD2B;PHF8;PIAS1;NXF1;HN
4;HNRNPU;CEP170;TARDBP;GIGYF2;GATAD2B
3L1;HNRNPC;CDC42BPA;TARDBP;GATAD2B
CC5;HNRNPU;DLST;GTF2H1;SMC1A;GATAD2B;HNRNPK;ZFP62;SP1;RBBP5;RUVBL1;G3BP1;ZNF148;
;FUBP1;UBN2;HNRNPU;HNRNPR;ACTN4;TARDBP;MYH10;PFAS;GATAD2B
CAPRIN1
;PAPSS2;MED17;TIAL1;RIC8B;CAPZB;RBBP5;RUVBL1;ATXN1L;SNRPD3;SNX8;APPL1;KPNA1;MAP2K1
1;HNRNPU;DLST
B;CRBN;RUVBL1;HNRNPU;ARID5B;CDC42BPA;TARDBP;GATAD2B

1C;TPM3;SP1;HNRNPU;ACTN4;ZNF148;TARDBP;RAB11FIP5
J;DLST;HNRNPR;MAT2B;SMC1A;GATAD2B;PCMT1;HNRNPK;MAT2A;SP1;CAPZA1;TRA2B;RUVBL1;TRIF
9B;HNRNPK;DCTN2;RUVBL1;PDCD4;UBXN7;LRRC40;RAB3GAP2;GAPVD1
3;CRBN;RBBP5;RUVBL1;HNRNPU;DLST;CALM1;TARDBP;GATAD2B
PU;LRRC40;DLST;GATAD2B;MED17;ACAP2;HNRNPK;TBL1XR1;SP1;RBBP5;FUBP1;RUVBL1;TARDBP;K
CC5;RBBP5;RUVBL1;ZNF318;DYRK1A;HNRNPU;ZNF148;RBM7;TARDBP;DYNLL2
RNPU;TERF2IP;HNRNPC;TARDBP
D1;SMC1A;GATAD2B;MED17;MED14;HNRNPK;CAPZB;SP1;RBBP5;RUVBL1;CEP170
1;SNAP23;HNRNPU;DLST;MAPK1;CALM1
NRNPK;CAPZB;RUVBL1;IGF2BP1;HNRNPU;LRRC40;SNRPD3
37;RANBP3;CRBN;XRCC5;HNRNPU;DLST;HNRNPR;PDHB;DYNLL2;RAB11A;PCMT1;HNRNPK;SIN3A;RI
;CPSF7;FAM114A1;DCTN2;HNRNPU;LRRC40;GAPVD1;ADD3;PCMT1;DDX19B;HNRNPK;CAPZA1;RUVI
;CAPRIN1;RUVBL1;G3BP1;HNRNPU;CEP170;SNRPD3;ZNF148;DYNLL2
CC5;HNRNPU;HNRNPR;SMC1A;ADD3;PIAS1;ADD2;ACAP2;HNRNPK;SART3;SP1;RUVBL1;ZNF318;CEF
DLST;SMC1A;PHF8;BCLAF1;HNRNPK;SP1;FUBP1;KLHL7;ZNF318;TARDBP;KPNA1
L;HNRNPU;PHF8;PCMT1;CYB5R4;HNRNPK;FUBP1;UBN2;RUVBL1;MAPRE3;TRIP12;TARDBP
R2;DEK
N;TPM3;FUBP1;RUVBL1;HNRNPU;STRN;TARDBP;MYH10
8;CPSF7;SF3B3;PSMD11;CRBN;XRCC5;HSPA4L;HNRNPU;HNRNPR;ACTN4;PFAS;GATAD2B;HNRNPK;
P5;CTNNB1;CEP170;TARDBP
;RUVBL1;HNRNPU;DLST;TARDBP
DEK;NUDT5;SMC1A;SMARCA1
RCC5;ST13;TPM3;HSPA4L;HNRNPU;HNRNPR;NUDT5;PFAS;GATAD2B;HK2;PCMT1;TTC9;HSPH1;HNRI
HNRNPR;ACTN4;GATAD2B;HNRNPK;SIN3A;FUBP1;SERBP1;RUVBL1;SNRPD3;TARDBP;ZNF687;KPNA1
PK;SP1;RUVBL1;HNRNPU;ZFP91;PGAM5;BSN;ZNF148;TARDBP;KPNA1
PU;PCMT1;HNRNPK;CAPZB;XPO4;SP1;CAPZA1;RUVBL1;TRIM24;HNRNPC;TARDBP
3;LUZP1;HNRNPU;HNRNPR;PDHB;MTDH;PHF8;NPAT;SART3;C1QBP;TRA2B;RUVBL1;C10ORF2;CEP17
HNRNPU;HNRNPR;PCMT1;HNRNPK;KIF5C;FUBP1;CAPRIN1;TRA2B;G3BP1;TRIP12;SNRPD3;HNRNPC
MAML1;XRCC5;HNRNPU;HNRNPR;GAPVD1;SMC1A;GATAD2B;MED17;SMAD7;PCMT1;HNRNPK;SH3P
HNRNPR;LNPEP;PARVA;ACTN4;DEK;NUDT5;ADD3;PCMT1;HNRNPK;SP1;CARM1;RUVBL1;SNRPD3;HN
ACTN4;FOXO1;MYO1C;CAPZB;RUVBL1;NOS1AP;HNRNPC;ZNF687;TARDBP;MYH10
5B;HNRNPR;PDHB;GAPVD1;GATAD2B;CDC73;PHF8;HNRNPK;MIER3;SIN3A;TRPS1;RUVBL1;HNRNPC
PK;RUVBL1;HNRNPU;HOOK3
;XRCC5;HNRNPU;SMAD9;ZFP91;HNRNPR;SMC1A;CDC42BPA;HNRNPK;KIF5C;RUVBL1;MAPK1;CEP1
RC40;DLST;HCFC2;SMC1A;GATAD2B;ITCH;RIC8B;HNRNPK;FUBP1;RUVBL1;RAB3GAP2;TARDBP;UBE2
D4;CRBN;TBL1XR1;SP1;RUVBL1;HNRNPU;CEP170;GATAD2B
3;TBL1XR1;SP1;RUVBL1;HNRNPU;HNRNPR;KPNA1;PHF8
U;HOOK3;SMC1A;ADD3;GATAD2B;PCMT1;HNRNPK;TBL1XR1;SP1;RUVBL1;SNRPD3;TARDBP
NPU;GAPVD1;HCFC2;CBFA2T2;SMC1A;GIGYF2;GATAD2B;HNRNPK;TBL1XR1;SP1;RBBP5;RUVBL1;RE
HNRNPR;SMARCA1;SMC1A;CDC42BPA;HNRNPK;FUBP1;RUVBL1;CEP170;TARDBP;ZC3H14
;RBBP5;DPYSL3;HNRNPU;TARDBP;LMOD3
7;SF3B3;XRCC5;HNRNPU;HNRNPR;KCNRG;CTGF;MED14;HNRNPK;TBL1XR1;SP1;RUVBL1;SNRPD3;T
3;CPSF7;SET;WDR37;SF3B3;XRCC5;HNRNPU;LRRC40;DLST;PDHB;HIPK1;SMC1A;GATAD2B;HNRNPK
;ATP2B2;C12ORF55;PHACTR4;JAKMIP2
NPR;HNRNPC;SOX6;TARDBP
170;TARDBP;SNX8;JAKMIP2
ZB;TPM3;CAPZA1;HNRNPU;HNRNPR;SNRPD3;ACTN4;TARDBP;ESR1;KPNA1
K;CAPZB;RBBP5;RUVBL1;HNRNPU;LRRC40;GAPVD1;CALM1;TARDBP
IRNPU;HNRNPR;GTF2H1;ACTN4;PAPSS2;PPP2CA;HNRNPK;KIF5C;SP1;FUBP1;HNRNPC;TARDBP;ZNF
D2B
K1;PSMD11;XRCC5;HNRNPU;LRRC40;DLST;ZFP91;PDHB;MAT2B;DEK;SMC1A;PAPSS2;GATAD2B;HNR
C40;SMC1A;CDC42BPA;GATAD2B;MED17;HNRNPK;XPO4;RBBP5;FUBP1;DDI2;RUVBL1;UBE2K;KPNA1
;HNRNPU;HNRNPR;CEP170;TRIP12;GATAD2B

RNPC;TARDBP
C5;PTPRM
2;TPM3;HNRNPU;PARVA;GAPVD1;DYNLL2;GATAD2B;ACAP2;HNRNPK;CAPZB;FUBP1;CAPZA1;RUVBL
S1L;HNRNPR;GATAD2B;PHF8;HNRNPK;CAPZB;SP1;SIN3A;FUBP1;CAPZA1;RUVBL1;TARDBP;ZNF687;
P5;CSNK1A1;RUVBL1;STRN;AFF4;AFF1;MED17
N2AIPNL;NKRF;CAPRIN1;RUVBL1;HNRNPU;DLST;KPNA1
RNPK;SART3;FCHSD2;TBL1XR1;SP1;RBBP5;RUVBL1;PGAM5;TARDBP;ZNF687;APPL1
RNPU;ATP2B2;DYNLL2;GATAD2B;CNOT6;HNRNPK;SP1;SUB1;RUVBL1;ZNF148
;FUBP1;HNRNPU;TARDBP;UBL7
;RUVBL1;HNRNPU;TARDBP;GATAD2B
N2;HNRNPU;DLST;HNRNPR;PDHB;CTDSPL2;PHF8;CAPZB;XPO4;RUVBL1;G3BP1;RBM12;ZNF462;SEP
;CARM1;RUVBL1;DPYSL3;CCDC6;HNRNPU;DLST;HNRNPR;CEP170
C5;CAPZA1;IGF2BP1;HNRNPU;UBXN7;HNRNPR;HNRNPC;GAPVD1;KPNA1
1;CAPRIN1;FUBP1;RUVBL1;CCDC6;HNRNPU;CEP170;ACTN4;TARDBP
PU;HNRNPR;HCFC2;GATAD2B;HNRNPK;KIF5C;TBL1XR1;RBBP5;RFWD3;RUVBL1;HNRNPC;TARDBP;K
NPR;ELK4;RBM3;PCMT1;CAPZB;XPO4;DDI2;RUVBL1;UBXN7;CEP170;SNRPD3;APPL1;KPNA1;MAP2K
J;TERF2IP;TARDBP
ZA1;HNRNPU;DLST
A;HNRNPU;DLST;PDHB;SMC1A;GATAD2B;PHF8;HNRNPK;TRIM3;RUVBL1;TARDBP;KPNA1
3B3;HNRNPU;DLST;HNRNPR;ACTN4;HNRNPK;KIF5C;FUBP1;RUVBL1;TARDBP;UBL7
HCFC2;GATAD2B;ENAH;HNRNPK;TBL1XR1;RBBP5;RUVBL1;SNRPD3;HNRNPC;TARDBP;CC2D1B
A;GATAD2B;PHF8;JAKMIP2;HNRNPK;FUBP1;KLHL7;RUVBL1;PGAM5;SNRPD3;TARDBP;ZNF687
CC5;RUVBL1;HNRNPU;SMC1A;CDC42BPA;DNAL1
3B3;XRCC5;HNRNPU;HNRNPR;SMARCA1;GATAD2B;PCMT1;HNRNPK;NFIA;KIF5C;SP1;RUVBL1;SNRF
PCDH11Y;XRCC5;HSPA4L;HNRNPU;SMC1A;GATAD2B;HNRNPK;SART3;SP1;RUVBL1;ATXN1L;USP1;CE
I;CEP170;GAPVD1;CDC42BPA;AFF4
IRNPU;LRRC40;HNRNPR;GATAD2B;MED17;HNRNPK;RBBP5;FUBP1;RUVBL1;TARDBP;SRGAP2;UBE2I
NPU;DLST
PU;TARDBP
ARDBP
3;SF3B3;TPM3;TMOD3;MSL2;HNRNPU;DLST;SMC1A;FBXO30;CDC73;NHSL2;HNRNPK;CAPZB;TBL1XR
U;HNRNPR;SMC1A;GIGYF2;PHF8;HNRNPK;RUVBL1;PGAM5;SNRPD3;TARDBP;CTBS
DT5;DYNLL2;GATAD2B;PPP2CA;HNRNPK;CAPZB;SUB1;CAPZA1;RUVBL1;STRN;MARK1
A

BL1

L1;HNRNPU;TARDBP;RAB11A
I;G3BP1;HNRNPU;TARDBP
NPU;BSN;ZC3H14;DKK3
1B;SF3B3;HNRNPU;HNRNPR;KCNRG;SMARCA1;MED17;ENAH;ACAP2;HNRNPK;FUBP1;RUVBL1;CCD
PU;HNRNPR;CEP170;SIK2;CDC73;HK2
;SF3B3;RUVBL1;ATXN1L;HNRNPR;TARDBP
NPU;HNRNPR;TARDBP
I;TPM3;HNRNPU;MTSS1L;ACTN4;SMC1A;PPP2CA;PCMT1;HSPH1;HNRNPK;RXRA;SP1;FUBP1;RUVBL
3;MAP2K1;SF3B3;XRCC5;HNRNPU;GAPVD1;SMC1A;GATAD2B;PHF8;PPP2CA;PCMT1;HNRNPK;TBL1X
;HNRNPR;GTF2H1;ACTN4;PHF8;CTGF;PPP2CA;PCMT1;HNRNPK;TBL1XR1;TRA2B;SERBP1;TRIP12;S
DX19B;HNRNPK;CRBN;TET3;HNRNPU;XIAP;TARDBP;GATAD2B;RAB11A
NPU;TERF2IP;TRIP12;SNRPD3;HNRNPC;TARDBP;AFF4;CDC73;AFF1
;SP1;RUVBL1;CCDC6;HNRNPU;CEP170;TRIP12;TARDBP;SH3GL2
RNPR;CEP170;ACTN4;HNRNPC;NUDT5;SMARCA1;TARDBP;GATAD2B
L1;HNRNPU;DLST;HNRNPR;SNRPD3;ACTN4;PDHB;GATAD2B
37;CRBN;XRCC5;SIN3A;HNRNPU;RBM7;CDC42BPA

RAVER2;CDC73;GATAD2B
RNPR;ACTN4;GAPVD1;PCMT1;HNRNPK;KIF5C;CAPRIN1;RUVBL1;G3BP1;SNRPD3;TARDBP
7;MBNL2;SET;SF3B3;PSMD11;VPS13C;HNRNPU;PARVA;SMC1A;GATAD2B;HNRNPK;TBL1XR1;FUBP1;(
I;DLST;TARDBP
PK;RUVBL1;SMC1A
TP2B2;JAKMIP2
PK;CRBN;FUBP1;VPS13C;RUVBL1;HNRNPU;ZFP91;PGAM5;TARDBP;PHF8
HNRNPU;AFF4;GTPBP4;GATAD2B;PHF8;NPAT;MED14;HNRNPK;SIN3A;CAPZA1;TRIP12;CEP170;TARD
)
PU;HNRNPR;BACH1;PHF8;SART3;NKRF;RBBP5;TRA2B;RUVBL1;CEP170;KPNA1;FNBP4;MAP2K1;XR(
3L1;HNRNPU;HAUS3;ZC3H14;GIGYF2;GATAD2B
T;HNRNPR;PGAM5;TARDBP;SORBS3;CDC73
18;HNRNPU;HNRNPR;PGAM5;TARDBP;AFF4;AFF1
ST13;FUBP1;RUVBL1;ZBTB10;HNRNPU;FGFR1
THDC1;HNRNPU;HNRNPR;ACTN4;SMC1A;DYNLL2;HNRNPK;FUBP1;DDI2;SERBP1;RUVBL1;SNRPD3;T
RIN1;G3BP1;SERBP1;HNRNPU;LRRC40;CEP170;GAPVD1;ZBTB4;GIGYF2;PUM2
;HNRNPU;ZFP91;HNRNPR;SMARCA1;SMC1A;GATAD2B;MED17;MED14;HNRNPK;CAPRIN1;TRA2B;RU
PU;GAPVD1;SMC1A;GIGYF2;GATAD2B;RAB11A;MED17;HNRNPK;RUVBL1;TARDBP;ZC3H14;KPNA1
NRNPU;DLST;HNRNPR;PDHB;MYPN;PHF8;PPP2CA;PCMT1;HSPH1;SIN3A;SNRPD3;FNBP4;CRBN;ST1
;TNKS;HNRNPU;DLST;HNRNPR;PDHB;CNOT6;HNRNPK;CAPZB;TBL1XR1;MB;CAPZA1;RUVBL1;PGAM
H2;HSPH1;TRPS1;NFATC3;HNRNPU;XIAP;HNRNPR;ACTN4;HNRNPC
;CSNK1A1;SNAP23;HNRNPU;HNRNPR;GATAD2B;PCMT1;SP1;MB;RBBP5;TRPS1;CEP170;TARDBP
2;HNRNPU;KCNRG
HNRNPU;TARDBP;GNL1;TNS1
N;HNRNPU;HNRNPR;CEP170;HNRNPC
ST;TARDBP
PU;CEP170
PK;SF3B3;RUVBL1;HNRNPU;HNRNPR;HNRNPC;TARDBP;JAKMIP2
L1XR1;SP1;CARM1;HNRNPU;DLST;PDHB;TARDBP;DCP2
PK;TPM3;FUBP1;RUVBL1;CCDC6;HNRNPU;SMC1A;TARDBP
NRNPK;SF3B3;XRCC5;HNRNPU;TARDBP;MED17
PK;APC;SP1;NOS1AP;HNRNPU;TARDBP;PHF8
RUNE2;HNRNPU;HNRNPR;CEP170;SMC1A;NUPL1
IRNPU;DLST;HNRNPR;PDHB;PFAS;PCMT1;SART3;SIN3A;RUVBL1;SNRPD3;CPSF7;CRBN;DYRK1A;AC
I1B;TPM3;HNRNPU;HNRNPR;ACTN4;PDHB;CBFA2T2;HNRNPK;MYO1C;XPO4;CAPZB;MB;CAPZA1;RU
NRNPU;ACTN4;SOD2;GATAD2B;PHF8;HNRNPK;FUBP1;RUVBL1;CEP170;HNRNPC;TARDBP;ZC3H14
L;HNRNPU;AFF4;GATAD2B;AFF1;MED17;MED14;HNRNPK;CEP170;HNRNPC;TARDBP
INRNPU;ZFX;UBE2K
D1;DLST;BACH1;KPNA1
VBL1;HNRNPU;EPC1;GATAD2B
3L1;HNRNPU;CEP170;ZNF687;TARDBP;GIGYF2;PHF8
K;CCDC6;HNRNPU;HNRNPR;STRN;TARDBP;SYTL4
3;ATXN1L;HNRNPU;DLST;PARVA;HOOK3;PDHB
RNPR;ACTN4;HNRNPC;LPHN2;CDC42BPA;TARDBP;SH3GL2
3;CAPZA1;RUVBL1;HNRNPU;TARDBP;MED17
U;DLST;HNRNPR;PHF8;PCMT1;SART3;RBBP5;RUVBL1;CEP170;FNBP4;XRCC5;YLPM1;LRRC40;ACTN
HNRNPU;HNRNPR;GAPVD1;SMARCA1;SMC1A;GATAD2B;FBXO30;PHF8;PTP4A1;HNRNPK;KIF5C;FUBI
3;XRCC5;HNRNPU;ACTN4;SMC1A;SOD2;RAB11A;PCMT1;DDX19B;HSPH1;HNRNPK;RAP1A;FUBP1;DI
RNPU;ZFP91;HNRNPR;ACTN4;DEK;NUDT5;CTGF;PPP2CA;PCMT1;HNRNPK;SP1;MB;TBL1XR1;FUBP1
K;PCDHA8;PCDHA7
RUVBL1;HNRNPU
BP1;RUVBL1;HSPA4L;E2F1;HNRNPU;HNRNPR;PGAM5;SMC1A;TARDBP
F3B3;HNRNPU;HNRNPR;PHF8;RXRA;RBBP5;RUVBL1;ZNF148;MYH10;ZC3H14;MAP2K1;XRCC5;YLPM

'U;HNRNPR;GATAD2B;PHF8;C20ORF112;HNRNPK;XPO4;APC;SP1;CAPRIN1;RUVBL1;LCOR;SNRPD3;I
NRNPU;HNRNPR;NUDT5;PAPSS2;PCMT1;RUVBL1;IGF2BP1;SNRPD3;SRGAP2;RRM1;CRBN;TPM3;XR
3;HNRNPU;SPATA2;HNRNPR;CAPZB;SIN3A;KIF5C;TRA2B;RUVBL1;ZNF687;SRPK2;MAP3K2;CPSF7;YL
;MC1A;DYNLL2;GATAD2B;PCMT1;HNRNPK;MAT2A;KIF5C;SP1;RBBP5;FUBP1;CAPRIN1;RUVBL1;SNRF
PU;ZFP91;HNRNPK;SP1;TRA2B;RUVBL1;G3BP1;HNRNPC;ZNF148;TARDBP;ZNF687;KPNA1
13;TPM3;HNRNPU;ACTN4;SMC1A;PPP2CA;PCMT1;HNRNPK;FUBP1;RUVBL1;TARDBP
T3;TPM3;XRCC5;SUB1;HNRNPU;HNRNPR;ACTN4;MYH10
;ROCK1;HNRNPU;LRRC40;SMC1A;GATAD2B;PCMT1;HSPH1;HNRNPK;XPO4;CAPZB;RUVBL1;PDCD4;
IPK;SF3B3;FUBP1;RUVBL1;YLPM1;HNRNPU;DLST;RBM12;TARDBP;UBE2K
33;CRBN;TPM3;HNRNPU;HNRNPR;ACTN4;SMG7;PCMT1;HNRNPK;SP1;RUVBL1;SNRPD3;ZNF148
I;FUBP1;RUVBL1;HNRNPU;TARDBP;DYNLL2;RAB11A
3L1;HNRNPU;DLST;RAF1;TARDBP;GATAD2B
;HNRNPK;RUVBL1;HNRNPU;ZNF687;TARDBP
;RUVBL1;HNRNPU;HNRNPR;PGAM5;TARDBP
);RUVBL1;HNRNPU;TARDBP;MED17
33;HNRNPU;TERF2IP;DLST;RAB11A
;PARVA;ACTN4;SMC1A;GIGYF2;PCMT1;MED14;HNRNPK;XPO4;RUVBL1;KPNA1
HNRNPU;HNRNPR;ACTN4;AFF4;PHF8;HNRNPK;XPO4;SP1;RUVBL1;TARDBP
'ZB;TBL1XR1;XRCC5;RUVBL1;HNRNPU;PGAM5;TARDBP;ZC3H14;PHF8
NPU;HNRNPR;GIGYF2;MYPN;PCMT1;CAPZB;KIF5C;RUVBL1;MAPK1;SNRPD3;NCOA2;GFPT1;YLPM1;S
NPU;TARDBP;PCDHA6
3L1;NUDT5;TARDBP
1
HNRNPU;HNRNPR;SMC1A;DYNLL2;GIGYF2;GATAD2B;HK2;PHF8;PIAS1;HNRNPK;RXRA;TBL1XR1;SP1
J;SMC1A;GIGYF2;MED12L;GATAD2B;CDC73;MTDH;MED17;PHF8;NPAT;HNRNPK;UBN2;RUVBL1;G3BP
KS;HNRNPU;HNRNPR;KCNRG;SMARCA1;CBFA2T2;SMC1A;HNRNPK;SART3;APC;KIF5C;SP1;RBBP5;F
RNPU;SMC1A;TARDBP;KPNA1
;SNX6
INRNPU;ZFP91;SNRPD3;HNRNPC;TARDBP
1;CCDC6;HNRNPU;RC3H1;SNRPD3
2K;NSD1;HNRNPU;ATP2B2;MYH10
HNRNPU;RC3H1;HNRNPR;ZBTB44;ADD3;CDC42BPA;PPP2CA;PCMT1;HNRNPK;SP1;SIN3A;RUVBL1;SI
RCC5;FUBP1;RUVBL1;HSPA4L;HNRNPU;ACTN4;CDC42BPA;GATAD2B
33;EPOR;PIAS1
NPU;TARDBP;C9ORF64
RCC5;HNRNPU;DLST;SMC1A;DYNLL2;PAPSS2;GATAD2B;PHF8;PCMT1;HNRNPK;ZZZ3;RXRA;SP1;RBE
U;ZFP91;TARDBP;CDC73;GATAD2B;KPNA1
3L1;HNRNPU;HNRNPC;TARDBP;GATAD2B;KPNA1
)RT1;TMOD3;HNRNPU;HNRNPR;CEP170
3L1;HNRNPU;HOOK3;TARDBP
3;SP1;EIF3J;HNRNPU;TARDBP
FUBP1;TRPS1;TFEB;GAPVD1
K1;RUVBL1;HNRNPU;SMC1A;TARDBP;FOXP2;GATAD2B;MED17
XR1;SP1;SIN3A;KIF26B;RUVBL1;HNRNPU;HNRNPC;GATAD2B
HNRNPU;ZFP91;SMC1A;GIGYF2;MYPN;GATAD2B;SNX1;HNRNPK;RBBP5;FUBP1;ZNF318;G3BP1;CEP
NRNPK;SART3;CAPZB;RUVBL1;HNRNPU;DLST;PDHB;TARDBP
75

MD11;YLPM1;HNRNPU;ACTN4;GAPVD1;GATAD2B;HNRNPK;SERBP1;RUVBL1;G3BP1;EIF3J;HNRNPC;
TPM3;HNRNPU;DLST;HNRNPR;PDHB;PHF8;PCMT1;HSPH1;HNRNPK;DPYSL3;RUVBL1;TRIM24;HNRNI
P1;CAPZA1;RUVBL1;HNRNPU;DLST;SNRPD3;HNRNPC;PDHB;TARDBP;RAB11FIP5
K1;HNRNPK;KIF5C;SP1;RUVBL1;HNRNPU;HNRNPR;TARDBP;DYNLL2;GATAD2B
RUVBL1;G3BP1;CCDC6;HNRNPU;TARDBP

BL1;MTSS1L;CEP170;SMC1A;TARDBP;CDC73;GATAD2B;RAB11FIP5
NPU;HNRNPR;PGAM5
'U;STRN;SNRPD3;DEK;CDC42BPA
XR1;RUVBL1;HNRNPU;SMARCA1;PHF8
'1;CAPZB;HNRNPU;STRN;DYNLL2
;HNRNPU;ACTN4;HNRNPC;TARDBP
SF3B3;TPM3;ADK;HNRNPU;HNRNPR;PDHB;GATAD2B;PPP2CA;HNRNPK;TBL1XR1;SP1;CDC37;CAPZA
HNRNPU;SMC1A;GIGYF2;HNRNPK;RBBP5;CAPRIN1;RUVBL1;ZNF318;G3BP1;SNRPD3;ZNF148;TARDI
3P5;RUVBL1;HNRNPU;ATP2B1
(RD40;TRIP12;DYNLL2;FBXO30
NPU;HNRNPR;PHF8;RXRA;CAPZB;XPO4;SIN3A;RUVBL1;G3BP1;CEP170;SNRPD3;FNBP4;SEPT10;CPS
T3;ROCK1;SORT1;UBN2;RUVBL1;GFPT1;HNRNPU;DLST;TARDBP
UBN2;RUVBL1;HNRNPU;XIAP;SNRPD3;TARDBP;DYNLL2;GATAD2B
(;CAPZB;CAPZA1;HNRNPU;GATAD2B;MED17;PHF8
C5;ST13;SIN3A;RUVBL1;HNRNPU;HNRNPR;TARDBP
3;CSNK1A1;RUVBL1;HNRNPU;DLST;HNRNPR;MYH10
RVA;SMC1A;DYNLL2;AFF1;MED17;HNRNPK;CDC37;RUVBL1;CEP170;TARDBP;ZC3H14;KPNA1
1XR1;FUBP1;RUVBL1;HNRNPU;SMC1A;TARDBP;CGGBP1;GATAD2B
1;NCOA2;SEPT10;RBBP5;RUVBL1;HNRNPU;TARDBP;RAB11A
R37;KIF5C;RUVBL1;ZNF318;HNRNPU;EPC1;KCNRG;DYNLL2
XR1;RBBP5;RUVBL1;HNRNPU;CEP170;GATAD2B
;MED17
RNPU;GATAD2B
NRNPK;PAPOLG;CAPRIN1;RAVER2;HNRNPU;CEP170;TARDBP;KPNA1
;RUVBL1;HNRNPU;LRRC40;TARDBP
PK;FUBP1;DLST;TARDBP;TRAK2
04;UBN2;RUVBL1;HNRNPU;TERF2IP;HNRNPR;SRGAP2;TARDBP;GATAD2B
T3;CAPZB;CRBN;RUVBL1;HSPA4L;HNRNPU;PGAM5;SNRPD3;TARDBP;HK2
'3;RUVBL1;HNRNPU;CEP170;TARDBP;GATAD2B;PHF8
RNPC;TARDBP;CDC73
NPR;HNRNPC;MED17
NPU;HNRNPR;TARDBP
ST;HNRNPR;HNRNPC
PK;HNRNPU;TARDBP
RNPU;DLST;TARDBP;PCDHAC1
;HNRNPU;GAPVD1;RAB11A
BL1;HNRNPU;XIAP;TARDBP
01
CROD2
NRNPU;DLST;HNRNPR;ZBTB4;PHF8;MED14;SIN3A;UBN2;RUVBL1;G3BP1;CEP170;KPNA1;FNBP4;MAF
K1;PSMD11;CRBN;XRCC5;TPM3;LRRC40;GAPVD1;GATAD2B;CDC73;HNRNPK;TBL1XR1;RBBP5;FUBP
PK;XRCC5;FUBP1;RUVBL1;HNRNPU;ZNF148;TARDBP;GATAD2B
BL1;HNRNPU;MTSS1L;SMC1A;PFAS;GATAD2B
;SEPT10;CPSF7;SET;SF3B3;XRCC5;USP9X;HNRNPU;HNRNPR;ACTN4;PPP2CA;HNRNPK;SERBP1;RU
ZB;APC;CAPZA1;RUVBL1;HNRNPU;TERF2IP;HNRNPR;TARDBP;RAB11A;PHF8
U;DLST;HNRNPR;PDHB;PCMT1;HNRNPK;KIF5C;SUB1;TRIP12;TARDBP;KPNA1
CCDC6;HNRNPU;CEP170;GIGYF2
SNPH;TET3;HNRNPU;TARDBP
A;ZNF318;HNRNPU;HNRNPR;HNRNPC;ZNF148;GATAD2B
RC40;GAPVD1;NUPL1;GIGYF2;KPNA1;PUM2;STXBP5L
RNPU;DLST;HNRNPR;PDHB;MTDH;PHF8;PCMT1;NXF1;CAPZB;SIN3A;RUVBL1;SNRPD3;KPNA1;CRBN
RUVBL1;HNRNPU;CEP170;DEK;SMC1A;TARDBP;CDC73
PK;RUVBL1;SNX8;MED17

NRNPU;HNRNPR
RNPU;RBM7;TARDBP
NK1A1;SEMA3A;HNRNPU;AFF4;GATAD2B;HK2;AFF1;HNRNPK;KIF5C;SP1;SIN3A;CEP170;HNRNPC;TA
H2;HNRNPK;KLHL7;RUVBL1;HNRNPU;UBXN7;DEK;GATAD2B;PHF8
3B3;CRBN;DPYSL5;ZNF318;HNRNPU;HNRNPR;ACTN4;DEK;TARDBP
D3;RUVBL1;USP2;HNRNPU;LRRC40;TARDBP
NPU;CBFA2T2;FOXO1;ENAH;CAPZB;TBL1XR1;CAPZA1;G3BP1;CCDC6;CTNNB1;MAPRE3;CGGBP1
CSNK1A1;HNRNPU;HNRNPR;GTPBP4;PCMT1;HSPH1;HNRNPK;TBL1XR1;SP1;TRPS1;RUVBL1;TRIP12
RDBP
L1
KIF5C;XRCC5;RUVBL1;SNRPD3
2;HNRNPU;LRRC40;GAPVD1;ADD3;RAB11A;PCMT1;RIC8B;HNRNPK;CAPZB;CAPZA1;RUVBL1;PDCD4
3;TBL1XR1;RUVBL1;HNRNPU;SNRPD3;HNRNPC;TARDBP;GATAD2B;PHF8;TRAK2
3;HNRNPU;ACTN4;TARDBP
NPR;CEP170;MAP3K9;TARDBP
PU;ACTN4;SMC1A;CDC42BPA;GATAD2B;MED17;PHF8;HNRNPK;SP1;SIN3A;RBBP5;RUVBL1;SNRPD3
VBL1;HNRNPU;SNRPD3
RIN1;CAPZA1;KLHL7;RUVBL1;G3BP1;HNRNPU;SMC1A
SF3B3;HNRNPU;HNRNPR;CDC73;HK2;PHF8;TIAL1;PCMT1;NXF1;SART3;MAT2A;RBBP5;RUVBL1;G3BP
A;RUVBL1;TRIM3;NOS1AP;HNRNPU;TERF2IP;DLST;CEP170;SLC25A24
BL1;HNRNPC;DEK;TARDBP;GATAD2B
3;RUVBL1;HNRNPU;CEP170;RAB11FIP5
TPM3;HNRNPU;DLST;HNRNPR;ZBTB44;PDHB;DEK;SMARCA1;GATAD2B;RAB11A;PHF8;PCMT1;HNRNF
PU;DLST;KCNRG;GAPVD1;SMC1A;GATAD2B;PIAS1;BCLAF1;HNRNPK;NFIA;SP1;FUBP1;RUVBL1;FAM1
2BP1;HNRNPU;HNRNPR;HNRNPC;CDC73;PHF8
YLPM1;HNRNPU;HNRNPR;CDC42BPA;HNRNPK;KLHL7;CAPRIN1;TRA2B;SERBP1;RUVBL1;G3BP1;TAF
HNRNPU;LRRC40;HNRNPR;C1ORF52;SMC1A;MED12L;GATAD2B;PHF8;HNRNPK;ZZZ3;XPO4;TBL1XR1
L1;HNRNPU;TARDBP;SNX6
HNRNPU;LRRC40;HNRNPR;GTPBP4;GATAD2B;HNRNPK;RBBP5;TRPS1;CARM1;RUVBL1;TARDBP;SSB
NPK;RBBP5;RUVBL1;HNRNPU;DEK;SMC1A;MAPRE2
BL1
B

NPK;SF3B3;CAPRIN1;RUVBL1;HNRNPU;SNRPD3;TARDBP;GATAD2B
CAPZB;SP1;RUVBL1;HNRNPU;MAPRE3;GAPVD1;TARDBP;SMC1A;GATAD2B
PZB;SUB1;RUVBL1;HNRNPU;HNRNPR;RSF1;SNRPD3;TARDBP;GATAD2B
PK;LUZP1;TPM3;RUVBL1;HNRNPU;HNRNPR;GAPVD1;MYH10;GATAD2B
U;DLST;HNRNPR;SMC1A;FOXP2;PHF8;MED14;HNRNPK;SP1;SIN3A;KLHL7;CAPRIN1;RUVBL1;TARDBF
;HNRNPU;SMARCA1
RNPU;TARDBP
GAM5;TARDBP
;PGM3;MSL2;HNRNPU;HNRNPR
SMARCA1;SMC1A;AFF4;GATAD2B;PHF8;NXF1;MED14;HNRNPK;RUVBL1;CEP170;SNRPD3;TARDBP;ZN
;HNRNPU
ARM1;HNRNPU
3;CTGF
F3B3;HNRNPU;HNRNPR;PPP2CA;PCMT1;CAPZB;RUVBL1;CEP170;KPNA1;MAP2K1;CPSF7;CSNK1A1;
PU;GAPVD1;CDC42BPA;MED17;ENAH;NPAT;MED14;HNRNPK;APC;SP1;RUVBL1;TARDBP
2B;TPM3;ST13;HNRNPU;ADD3;CDC42BPA
3;TBL1XR1;RUVBL1;ZNF687;SMC1A;TARDBP;KPNA1
U;HNRNPR;GAPVD1;SMC1A;GATAD2B;NXF1;RNF214;HNRNPK;XPO4;CAPZB;KIF5C;FUBP1;UBN2;CAI
;CAPRIN1;G3BP1;SERBP1;HNRNPU;LRRC40;HNRNPR;GIGYF2
APZA1;HNRNPU;HNRNPC;ZNF687;ZNF148;TARDBP;MYH10;GATAD2B

:RBN;LRRC40;PARVA;GATAD2B;HNRNPK;RBBP5;FUBP1;ZNF318;PDCD4;STRN;TARDBP;UBE2K
PR;NUDT5;GATAD2B;HNRNPK;ZZZ3;SP1;SUB1;CAPRIN1;RUVBL1;G3BP1;TRIM24;ZNF148;TARDBP
SNK1A1;HNRNPU;HNRNPR;SMC1A;GATAD2B;PHF8;PPP2CA;NPAT;C20ORF112;HNRNPK;CAPRIN1;RU
PU;HNRNPR;SNRPD3;TARDBP;GATAD2B
)BP;GATAD2B;NFE2L1
RBN;HNRNPU;ADD3
PR;SMC1A;BACH1;GATAD2B;HK2;GLS;PHF8;HNRNPK;SP1;TRPS1;TRA2B;RUVBL1;NOS1AP;CCDC6;Z
NRNPU;ACTN4;UBE2K;CGGBP1;KPNA1
;YLPM1;HNRNPU;HNRNPR;TARDBP;MED17
!B;DCTN2;CAPZA1;RUVBL1;XIAP;GATAD2B
HNRNPU;PPWD1;SMARCA1;SMC1A;GATAD2B;PHF8;NPAT;HNRNPK;APC;SP1;UBN2;RUVBL1;TARDBP
YLPM1;HNRNPU;HNRNPR;GTPBP4;GATAD2B;HNRNPK;NKRF;CAPRIN1;RUVBL1;G3BP1;SNRPD3;HNF
EIF2B2;HNRNPK;XRCC5;SP1;HNRNPU;TARDBP
IRNPU;DEK;ZNF687;TARDBP;SNX8;GATAD2B
BL1;RAVER2;HNRNPU;LRRC40;DEK;SMC1A;TARDBP;GATAD2B
M2

U;HNRNPR;SMARCA1;SMC1A;CDC42BPA;PPP2CA;HNRNPK;KIF5C;SP1;RUVBL1;USP1;TERF2IP;CEP1
;PDHB;SMC1A;GIGYF2;GATAD2B;HNRNPK;SP1;TRPS1;RUVBL1;ZNF148;TARDBP;ZNF687;KPNA1
NPU;GAPVD1;SMC1A;ADD3;EPS8;NXF1;RXRA;CAPRIN1;RUVBL1;TFAM;HNRNPC;ZC3H14
L;HNRNPU;HNRNPR;ACTN4;GAPVD1;PCMT1;HNRNPK;CAPRIN1;SERBP1;CEP170;HNRNPC;MYH10
NPR;MED17;PPP2CA;PCMT1;HNRNPK;TBL1XR1;ATXN7;SP1;CAPZA1;G3BP1;CCDC6;RAB3GAP2;HNRI
K;RUVBL1;KIAA0141;HNRNPU
U;HNRNPR;SMARCA1;DYNLL2;PHF8;RUVBL1;TRIP12;SNRPD3;HNRNPC;TARDBP;MYH10
F3B3;XRCC5;HNRNPU;SMC1A;PHF8;HNRNPK;KIF5C;TBL1XR1;SP1;RUVBL1;G3BP1;TARDBP;KPNA1
A;RBBP5;RUVBL1;HNRNPU;PGAM5;SNRPD3;TARDBP;DYNLL2;ZC3H14;HK2
9B;HNRNPK;SF3B3;TNKS;PHF20;RUVBL1;NOS1AP;HNRNPU;TARDBP
RUVBL1;TRIM24;HNRNPU;DLST;PGAM5;SMC1A;TARDBP;GATAD2B
P1;HNRNPU;HNRNPR;KCNRG;ZNF687;CDC73
(R1;SIN3A;G3BP1;HNRNPU;MAPRE3;SNRPD3;TARDBP;TGM2
N;CARM1;RFWD3;RUVBL1;YLPM1;HNRNPU;ZBTB20;GATAD2B

5;HNRNPU;SMC1A;DYNLL2;GATAD2B;PIAS1;PCMT1;NPAT;HSPH1;HNRNPK;RUVBL1;TARDBP
IZP1;USP9X;TNKS;HNRNPU;HNRNPR;ADD3;HNRNPK;RUVBL1;RLIM;CEP170;SNRPD3;ZNF423
PU;DLST;TARDBP
PR;SNRPD3;PDHB
NPU;HNRNPC;RAB11FIP5
3;PFKFB3;XRCC5;HNRNPU;ZFP91;HNRNPR;DEK;SMC1A;GIGYF2;HNRNPK;RUVBL1;PDCD4;CEP170;T
RUVBL1;HNRNPU;HNRNPR;GTF2H1;ZNF148;TARDBP;SMC1A;MED17;PHF8
;HNRNPU;LRRC40;HNRNPR;GAPVD1;SMARCA1;CDC42BPA;NPAT;HNRNPK;XPO4;RUVBL1;CEP170;T/
RNPU;ACTN4;PFAS
L1;HNRNPU;LRRC40;DYNLL2
A4L;HNRNPU;SMARCA1;TARDBP
3;SMC1A
3P;GATAD2B
I;DLST;HNRNPR;SMC1A;GATAD2B;PHF8;HNRNPK;MYO1C;CAPZB;SP1;SIN3A;RBBP5;RUVBL1;ZNF148
BL1;HNRNPU;STRN;TRIP12;GAPVD1;CBFA2T2;TARDBP;GATAD2B
C5;ST13;RUVBL1;HNRNPU;RSF1;ZNF687;TARDBP;GATAD2B
;FUBP1;HNRNPU;DLST;ZNF148;SMARCA1;GATAD2B;TRAK2
BL1;HNRNPU;SPATA2;PTK2B;ADD3
TPM3;HNRNPU;SPATA2;HNRNPR;GTF2H1;PDHB;ACTN4;CDC73;SMAD7;CDH6;NXF1;HNRNPK;MYO1C
U
C5;HNRNPU;ZFP91;HNRNPR;GTF2H1;ACTN4;PHF8;PCMT1;HNRNPK;CREB1;SP1;SERBP1;RUVBL1;T

HNRNPU;DLST;GATAD2B;PHF8;PCMT1;BCLAF1;HNRNPK;NFIA;SP1;SUB1;RUVBL1;TRIM24;HNRNPC;Z
RNPR;GAPVD1;SMC1A;DYNLL2;GIGYF2;PHF8;PCMT1;HNRNPK;TBL1XR1;SP1;UBN2;RUVBL1;CEP170;
RNPU;LRRC40;SMC1A;DYNLL2;GATAD2B;HNRNPK;SP1;RBBP5;FUBP1;RUVBL1;G3BP1;CEP170;SNR
I28;MAP2K1;SET;SF3B3;ST13;TPM3;XRCC5;HNRNPU;DLST;HNRNPR;DEK;SMARCA1;GATAD2B;PCMT
K1;HNRNPK;RUVBL1;ADK;HNRNPU;DLST;CDC42BPA;SNX8;GATAD2B;KPNA1
XR1;RUVBL1;ZNF318;HNRNPU;SNRPD3;ZNF148;SMC1A;DYNLL2;GIGYF2;RBX1
YLPM1;HNRNPU;HNRNPR;SMC1A;GTPBP4;NXF1;HNRNPK;NKRF;SP1;SERBP1;RUVBL1;G3BP1;TARD
70;GAPVD1;JAKMIP2
XRCC5;SP1;RUVBL1;HNRNPU;HNRNPR;GTF2H1;ZBTB44;PHF8
HNRNPU;ZFP91;HNRNPR;PHF8;CTGF;HNRNPK;SART3;RUVBL1;TRIP12;CEP170;TARDBP
CRBN;HNRNPU;HNRNPR;SMARCA1;SMC1A;GATAD2B;PHF8;TBL1XR1;RUVBL1;SNRPD3;HNRNPC;TA
RIN1;RUVBL1;HNRNPR;TARDBP
G3BP1;SERBP1;SORD;HNRNPU;GIGYF2;PUM2
BL1;USP2;SERBP1;HNRNPU;SH3GL2;GIGYF2;APPL1
NPU;LRRC40;GAPVD1;SMC1A;GATAD2B;CDC73;HNRNPK;SIN3A;RBBP5;SUB1;FUBP1;RUVBL1;ZNF31
33;RUVBL1;ATXN1L;HNRNPU;CDC42BPA;TARDBP
.1;G3BP1;HNRNPU;DLST;SMARCA1;SLC25A24
A;SERTAD2;HNRNPU;PGAM5;GATAD2B
RNPR;GATAD2B
VBL1;SNX8
318;DLST
1;HNRNPU
1;HNRNPK;RUVBL1;IGF2BP1;HNRNPU;LRRC40;TARDBP;KPNA1
J;HNRNPR;ACTN4;SMC1A;FAM117B;GATAD2B;NXF1;TERF2IP;CEP170;HNRNPC;TNRC6B
RNPU;LRRC40;DLST;GTF2H1;NUDT5;SMC1A;GIGYF2;GATAD2B;PHF8;PCMT1;HNRNPK;CAPZB;FUBP
K;YLPM1;HNRNPU;HNRNPR;SMC1A;GATAD2B;HNRNPK;RUVBL1;CEP170;HNRNPC;TARDBP;NUPL1;Z
N1;RUVBL1;G3BP1;HNRNPU;HNRNPR;ADD3;JAKMIP2
H1;TARDBP;FBXO30
PU;DLST;ZNF687
RDBP
.1;RUVBL1;HNRNPU;CEP170;TARDBP;GATAD2B
NPU;HNRNPR;SMC1A;GATAD2B;PHF8;PIAS1;TIAL1;HNRNPK;SP1;CAPZA1;RUVBL1;CEP170;BTRC;ZNI
PDE3B;TRIM3;HNRNPU
7;HNRNPU;DLST;PGAM5;CDC73
SWAP70;PDCD4;HNRNPU;DLST
1;HNRNPU;CEP170;GAPVD1;SMC1A
VBL1;HNRNPU;RSF1;TARDBP;GATAD2B
PU;HNRNPR;GAPVD1;SMC1A;GATAD2B;PHF8;DDX19B;HNRNPK;XPO4;SP1;MB;SIN3A;FUBP1;CARM1
33;XRCC5;RUVBL1;HNRNPU;TERF2IP;STRN;SNRPD3;PDHB;TARDBP
P1;USP9X;ZNF292;HNRNPU;ZFP91;HNRNPR;GATAD2B;AFF1;NXF1;ATXN7;KLHL7;RUVBL1;HNRNPC
PK;RUVBL1;HNRNPU;HNRNPR;SNRPD3;SMC1A;TARDBP;DYNLL2
D2;RUVBL1;HNRNPU;HNRNPR;CEP170;CDC42BPA;TARDBP;DCP2
HNRNPU;LRRC40;ACTN4;SMC1A;DYNLL2;GIGYF2;GATAD2B;PHF8;HNRNPK;XPO4;NFIA;CAPZB;SIN3A
SMC1A;GATAD2B;SMAD7;HNRNPK;XPO4;SNPH;RBBP5;RUVBL1;TARDBP;RAF1;KPNA1
BL1;CCDC6;HNRNPU;SMARCA1;MED17
L1XR1;FUBP1;HNRNPU;ZNF148;TARDBP
PK;RUVBL1;HNRNPU
G;RBM12;GATAD2B
1L;HNRNPU;PHF8
;MAGEE1;HNRNPU;ZFP91;HNRNPR;PDHB;GAPVD1;AFF4;PHF8;HNRNPK;CEP170;TARDBP;KPNA1
NPU;ZFP91;ACTN4;DDX19B;HNRNPK;CAPZB;TBL1XR1;RBBP5;CAPZA1;RUVBL1;TARDBP
HNRNPU;SNRPD3;DYNLL2
RT3;SF3B3;HNRNPU;HNRNPC;SMC1A;CDC73;GATAD2B;KPNA1

\;MMP2;RUVBL1;ATXN1L;HNRNPU;DLST;TARDBP;GATAD2B
6
[illegible]
CDC37;RUVBL1;G3BP1;HNRNPU;CEP170;MED17
N2;TBL1XR1;RUVBL1;ANKRD40;SMC1A;TARDBP;KPNA1
P5;FUBP1;RUVBL1;HNRNPU;PGAM5;GAPVD1;SMC1A;TARDBP;KPNA1
J;DEK;DYNLL2;GIGYF2;GATAD2B;PHF8;ZZZ3;RUVBL1;LCOR;TARDBP;KPNA1
33;FUBP1;RUVBL1;HNRNPU;DLST;TARDBP
PK;SF3B3;RUVBL1;HNRNPU;DLST;PHF8
1;FUBP1;RUVBL1;HNRNPU;HNRNPR;TARDBP
1;PRUNE2;HNRNPU;HNRNPR;ETFA;PHF8;KIF5C;C1QBP;RUVBL1;APBB2;PRKG1;KPNA1;CRBN;USP9X
ZNF292;HNRNPU;HNRNPR;DEK;DYNLL2;GATAD2B;CDC73;HNRNPK;KIF5C;GPC1;CAPRIN1;RUVBL1;ST
U;HNRNPR;GAPVD1;SMC1A;GIGYF2;HK2;MTDH;PHF8;HNRNPK;CREB1;XPO4;DPYSL5;UBN2;PAPOLG
3L1;TARDBP
T;TARDBP
N2;RBBP5;RUVBL1;HNRNPU;TFAM;TARDBP;SMC1A;ADD3;ZC3H14;CDC73
0;CPSF7;SET;SF3B3;CRBN;HNRNPU;SMC1A;GATAD2B;PHF8;HNRNPK;RXRA;FUBP1;RUVBL1;HNRNP
MD11;XRCC5;TPM3;YLPM1;HNRNPU;HNRNPR;MED12L;HSPH1;HNRNPK;MAT2A;SP1;RUVBL1;TARDB
7;RUVBL1;HNRNPU;CEP170
P1;CAPRIN1;RUVBL1;HNRNPU
IC1A;GATAD2B;PHF8;PIAS1;HNRNPK;PABPN1;RUVBL1;SNRPD3;HNRNPC;ZNF148;TARDBP;SNX8;AGI
BL1;CCDC6;HNRNPU;MTMR9;GAPVD1;GATAD2B
PU;DLST;HNRNPR;PDHB;SMC1A;GATAD2B
YLPM1;HNRNPU;SMARCA1;GATAD2B;HK2;HNRNPK;SP1;SERBP1;RUVBL1;SNRPD3;TARDBP
3;SET;SF3B3;XRCC5;HNRNPU;HNRNPR;ACTN4;HOOK3;SMARCA1;PAPSS2;GATAD2B;PHF8;PCMT1;H
T

3;HNRNPU;LRRC40;GAPVD1;HNRNPK;RUVBL1;RAB3GAP2;CEP170;SNRPD3;TARDBP
HNRNPU;LRRC40;DLST;GAPVD1;SMC1A;GATAD2B;RIC8B;HNRNPK;KIF5C;RUVBL1;UBE2K
/BL1;MSL2;HNRNPU;SMC1A;TARDBP;KPNA1;APPL1
C5;HNRNPU;CEP170;OTC
31;HNRNPU;TARDBP;SNX8
U;SMC1A;GIGYF2;GATAD2B;PCMT1;HNRNPK;RBBP5;RUVBL1;SNRPD3;ZNF148;TARDBP;ZC3H14
3P;RAPGEF6;UBE2K
7;RUVBL1;TARDBP
DBP;GATAD2B
NPU;TARDBP
JAKMIP2
GAPVD1;SMC1A;DYNLL2;GIGYF2;GATAD2B;HNRNPK;TBL1XR1;SP1;SNPH;FUBP1;RUVBL1;CEP170;SN
I1;RUVBL1;HNRNPU;DLST;HNRNPR;CDC73
3L1;HNRNPU;HNRNPR;HNRNPC;SMARCA1;TARDBP
3B3;HNRNPU;ZFP91;HNRNPR;HIPK1;HNRNPK;TBL1XR1;SP1;RUVBL1;G3BP1;PCDHA3;CEP170
A2;HNRNPR;DEK;GAPVD1;SMC1A;CDC73;MED14;HNRNPK;CAPRIN1;RUVBL1;STRN;CEP170;TARDBP
5;HNRNPU;HNRNPR;SMC1A;GNL1;HNRNPK;KLHL7;CAPRIN1;RUVBL1;CEP170;TARDBP
PU;HNRNPR;PRICKLE1;RBM7;GATAD2B
STAU1;CSNK1A1;YLPM1;HNRNPU;HNRNPR;SMARCA1;DYNLL2;SORBS3;GIGYF2;RAB11A;PHF8;PCM
RCC5;HNRNPU;LRRC40;GAPVD1;SMC1A;GIGYF2;PAPSS2;GATAD2B;PHF8;HNRNPK;RRM2B;RBBP5;F
1XR1;CAPZA1;RUVBL1;HNRNPU;LRRC40;RAB3GAP2;DEK;TARDBP;HK2
N;FUBP1;HNRNPU;ACTN4;TARDBP;MYH10;DYNLL2
NPU;DLST;SMARCA1;LRP8
RUVBL1;HNRNPU;ACTN4
K1;SEPT11;SET;SF3B3;CRBN;SFMBT2;HNRNPU;HNRNPR;SMARCA1;SMC1A;GATAD2B;HNRNPK;RUVI
;ST13;FUBP1;RUVBL1;HNRNPU;TARDBP

)1;HNRNPU;HNRNPR;ACTN4;TARDBP;ASPRV1
3;RUVBL1;HNRNPU;TERF2IP;HNRNPR;TARDBP
)LST;ZFP91;HNRNPR;SMARCA1;GATAD2B;HNRNPK;CAPZB;FUBP1;RUVBL1;CEP170;HNRNPC;TARDI
PU;STRN;DYNLL2
.1;SMC1A;TARDBP
I;CSNK1A1;ZBTB16;HNRNPU;GIGYF2;ENAH;PCMT1;HNRNPK;CAPZB;SERBP1;RUVBL1;STRN
'PM3;TMOD3;VPS13C;HNRNPU;SMARCA1;SMC1A;GATAD2B;ENAH;HNRNPK;RUVBL1;TARDBP
/D1;SMC1A;DYNLL2;GATAD2B;HNRNPK;TBL1XR1;SP1;RBBP5;RUVBL1;ZNF148;TARDBP;SRGAP2
(IF5C;XRCC5;RUVBL1;HNRNPU;HNRNPR;HOOK3;TARDBP;PHF8
\;XRCC5;RUVBL1;E2F1;HNRNPU;DLST;PGAM5;PDHB;TARDBP
PCMT1;SNX1;XPO4;RUVBL1;KPNA1;SNX6;MAP2K1;WDR37;XRCC5;NEK7;LRRC40;GAPVD1;SMC1A;G
NPU;TARDBP
KLHL23
)X;ATXN1L
GFPT1;YLPM1;IRS4;HNRNPU;DLST;HNRNPR;PDHB;GATAD2B;HNRNPK;CAPRIN1;SERBP1;RUVBL1
RNPU;SMARCA1;SMC1A;TARDBP
SUB1;RUVBL1;YLPM1;HNRNPU;GTF2H1
B3;RBBP5;RUVBL1;HNRNPU;SNRPD3;GAPVD1;SMC1A;CCDC50;GATAD2B
XRCC5;HNRNPU;DLST;HNRNPR;SMC1A;SMARCA1;GATAD2B;PCMT1;HNRNPK;SART3;XPO4;SP1;UBI
RNPU;HNRNPR;ACTN4;SMC1A;MTDH;RNF126;HNRNPK;UBL3;MYO1C;SP1;PGAM5;SNRPD3;HNRNPC
B3;CRBN;RUVBL1;HNRNPU;HNRNPR;HNRNPC;TARDBP
N1;DLST;TARDBP
;CAPZA1;HNRNPU
IPK;RUVBL1;HNRNPU;TARDBP;GATAD2B

3;HNRNPU;HNRNPR;HNRNPC;ZNF687;HCFC2;SMC1A;TARDBP
PK;HNRNPU
RNPC;TARDBP
RDBP
L1;DLST
'U;RAPGEF6

'5;CAPRIN1;G3BP1;HNRNPU;HNRNPR;PGAM5;HNRNPC;TARDBP;GIGYF2
IN1;RUVBL1;HNRNPU;HNRNPC;TARDBP;FOXO1
ST

1;HNRNPU;HNRNPR;MED17
;NKRF;RBBP5;GATAD2B
BL1;HNRNPU;HNRNPC;DEK
C40;DLST;GTF2H1;GAPVD1;SMC1A;GATAD2B;GTF2H5;HNRNPK;FUBP1;RUVBL1;UBXN7;TARDBP;MYI
IPK;CDC37;RUVBL1;HNRNPU;HNRNPR;HNRNPC;SMARCA1;TARDBP;RAF1;SMC1A
NPU;DLST;ZFP91;CEP170;SNRPD3;ACTN4;PDHB;TARDBP;GATAD2B;KPNA1
4;RUVBL1;ZNF318;EIF3J;HNRNPU;DEK;TARDBP;ZNF687;MYH10;GATAD2B
OCK1;CSNK1A1;TPM3;HNRNPU;LRRC40;HNRNPR;HOOK3;GATAD2B;AP3M2;HNRNPK;KIF5C;RUVBL1
NPU;SMAD9;ZFP91;HNRNPR;ADD3;GTPBP4;PHF8;PURA;BCLAF1;HNRNPK;SART3;SH3PXD2A;KIF5C
MC1A;GATAD2B;PHF8;FAM133B;HNRNPK;TERF2IP;TRIP12;CEP170;SNRPD3;ZNF423
HNRNPU;GAPVD1;ADD3;CDC42BPA;GIGYF2;HNRNPK;TBL1XR1;RUVBL1;CEP170;PAFAH1B2
;SET;SF3B3;XRCC5;HNRNPU;HNRNPR;GAPVD1;SMC1A;SMARCA1;GTPBP4;PAPSS2;GATAD2B;PHF8;
3;RUVBL1;ATXN1L;HNRNPU;HNRNPR;GTF2H1;SMC1A;TARDBP
R1;CALCOCO2;RBBP5;RUVBL1;HNRNPU;SMC1A;GATAD2B;PHF8
NRNPU;HNRNPR;HNRNPC;ZNF687;TARDBP;GATAD2B;PHF8
B3;SP1;HNRNPU;HNRNPR;TARDBP

RPD3;SMC1A;TARDBP
3;CAPRIN1;G3BP1;HNRNPU;HNRNPR;HNRNPC;TARDBP;NUPL1;ZC3H14;GATAD2B
NPU;WDFY3;ZNF687;DTX4;C12ORF55;MTDH
A;GATAD2B;CDC73;MED17;PHF8;HNRNPK;SP1;RBBP5;SNRPD3;HNRNPC;ZNF148;TARDBP
P5;HNRNPU;DLST;ZNF148;TARDBP;PIAS1;PHF8
1;HNRNPU;LRRC40;GIGYF2;PCMT1;DIAPH2;HNRNPK;SP1;RUVBL1;G3BP1;RAB3GAP2;TARDBP;UBE2
L1;DPYSL3;HNRNPU;HNRNPC
NPU;LRRC40;ZBTB34;KIF1B;CDC42BPA;TARDBP;KANK4;GIGYF2
F;RUVBL1;HNRNPU;HNRNPR;DEK;TARDBP;SMC1A;PHF8;KPNA1
PU;DLST;ACTN4;HNRNPC;KCNRG
;RUVBL1;HNRNPU;HNRNPR;TARDBP
KIF5C;FUBP1;RUVBL1;HNRNPU;HNRNPR;HNRNPC;TARDBP;SMC1A;RAB11A

C;RUVBL1;HNRNPU;CDC42BPA;TARDBP;SMAD7
J;WDFY3;TARDBP
;MTDH;PHF8;PPP2CA;KIF5C;TRA2B;RUVBL1;MARK1;FNBP4;MAP2K1;CPSF7;YLPM1;GTF2H1;GAPVD
3;CRBN;TBL1XR1;XRCC5;RUVBL1;HNRNPU;HNRNPR;ZNF148
BL1;HNRNPU;SNRPD3;ADD3;TARDBP;IKZF3;HK2;SMAD7
P1;RUVBL1;HNRNPU;LRRC40;DLST;HNRNPC;TARDBP;SMC1A;GATAD2B
A;LUZP1;SIN3A;HNRNPU;ZFP91;TERF2IP;CEP170;TARDBP;ZNF687;SMC1A
CSNK1A1;XRCC5;HNRNPU;HNRNPR;DEK;SMC1A;SMARCA1;MTDH;RBM3;NXF1;BCLAF1;HNRNPK;VA
YF2;GATAD2B;PIAS1;HNRNPK;SIN3A;RBBP5;SUB1;FUBP1;RUVBL1;CEP170;ZNF148;TARDBP
5;HNRNPU;DEK;SMC1A;GATAD2B;HK2;PHF8;HNRNPK;XPO4;TBL1XR1;RBBP5;SUB1;LCOR;TARDBP;N
PU;STRN
RNPU;CDC42BPA
77A1;HNRNPU;TARDBP;GATAD2B;KPNA1;PHF8
PK;XRCC5;RUVBL1;HNRNPU;MAT2B;DEK;TARDBP
USP9X;TRA2B;YLPM1;HNRNPU;HNRNPR;TARDBP
I;MAML1;RBBP5;HNRNPU;RC3H1;CEP170;KPNA1
PK;SF3B3;TBL1XR1;RBBP5;RUVBL1;HNRNPU;TARDBP
UBP1;RUVBL1;HNRNPU;HNRNPR;CEP170;TARDBP;GATAD2B
RCC5;HNRNPU;HNRNPR;ACTN4;SMC1A;GATAD2B;PHF8;PCMT1;HNRNPK;SART3;RRM2B;MAT2A;SP1
RUVBL1;SNRPD3
N;CALCOCO2;TARDBP
RNPU;HNRNPR;ACTN4;PPP2CA;ALAD;HNRNPK;MYO1C;CAPZB;C1QBP;G3BP1;CCDC6;SNRPD3;HNR
RNPU;DLST;HNRNPR;NUDT5;PHF8;PPP2CA;PCMT1;SNX1;HSPH1;CAPZB;RUVBL1;G3BP1;CEP170;ZN
PVD1;CDC42BPA;GTPBP4;GATAD2B;HNRNPK;RUVBL1;CEP170;TARDBP;SRGAP2;UBE2K;KPNA1
NFATC3;HNRNPU;AFF4;GATAD2B;CDC73;PHF8;JAKMIP2;PCMT1;RUVBL1;ATXN1L;ZNF148;TARDBP;TE

NPU
SD2;HNRNPU;SNRPD3;MYH10;ERGIC2
L1;HNRNPU;DLST;SMC1A;GATAD2B
;HNRNPU;HNRNPR;CEP170;ZNF687
ORF59;RUVBL1;GABRA3;HNRNPU;GIGYF2
A;HNRNPU;DLST;CLSPN;DEK;TARDBP;PTPN4;PHF8
2;RUVBL1;HNRNPU;HNRNPR;GAPVD1;TARDBP;SMC1A;GIGYF2;KPNA1
-;CRBN;FUBP1;HSPA4L;RUVBL1;ZNF318;HNRNPU;TARDBP;SMC1A;CDC42BPA
3;XRCC5;RUVBL1;HNRNPU;GTF2H1;CEP170;TARDBP;SMC1A;GTF2H5;KPNA1
TARDBP
HOOK3
P170;HNRNPC
CAPZA1

RNPU;GATAD2B

NPU;HNRNPR;SNRPD3
3L1;HNRNPU;TARDBP;PAFAH1B2
3L1;HNRNPU;DEK;TARDBP
7;SF3B3;HNRNPU;ACTN4;SMC1A;GATAD2B;ENAH;HNRNPK;TBL1XR1;CCDC6;CEP170;UBE2K;KPNA1
;SF3B3;YLPM1;HNRNPU;HNRNPR;GAPVD1;SMC1A;GATAD2B;HNRNPK;RUVBL1;NOS1AP;ZNF148;TA
1;HNRNPU;HCFC2;TARDBP;PAPSS2
PK;ROCK1;FUBP1;RUVBL1;HAUS3
IRNPK;RUVBL1;HNRNPU;SMC1A;TARDBP
3B3;HNRNPU;DLST;HNRNPR;SMARCA1;GTPBP4;HNRNPK;RUVBL1;HNRNPC;TARDBP
;SET;SF3B3;HNRNPU;HNRNPR;HNRNPK;SIN3A;FUBP1;HNRNPC;TARDBP;WDFY4
IRNPU;DLST;ACTN4;PCMT1;HNRNPK;MYO1C;RUVBL1;TRIP12;MYH10;KPNA1
5C;RUVBL1;DEK;ADD3;TARDBP;GATAD2B
I;NFIA;FUBP1;HNRNPU;TARDBP;DYNLL2;GATAD2B
XR1;RUVBL1;HSPA4L;HNRNPU;HNRNPR;HNRNPC;SMC1A;TARDBP
33;HNRNPU;LRRC40;GAPVD1;SMC1A;GIGYF2;GATAD2B;PIAS1;MED14;HNRNPK;FUBP1;CAPZA1;RUV
SF3B3;YLPM1;HNRNPU;LRRC40;SMC1A;DYNLL2;GATAD2B;PIAS1;HNRNPK;TBL1XR1;SERBP1;RUVB

3;CRBN;TPM3;HNRNPU;HNRNPR;GTF2H1;ACTN4;SMC1A;SMARCA1;GIGYF2;GATAD2B;PPP2CA;PCM
;ZFP91;SMC1A;DYNLL2;AFF4;PHF8;PCMT1;HNRNPK;SP1;RUVBL1;NOS1AP;ZNF148;TARDBP
JSP9X;YTHDC1;HNRNPU;GAPVD1;SMC1A;GATAD2B;HNRNPK;RUVBL1;G3BP1;ZC3H14;UBE2K;KPNA
13C;HSPA4L;HNRNPU;LRRC40;GAPVD1;GIGYF2;HSPH1;CAPZB;DDI2;IGF2BP1;STRN;CEP170
IPU;KPNA1
CDC42BPA
NPK;SF3B3;SERBP1;RUVBL1;HNRNPU;TERF2IP;HNRNPR;NUDT5;SMARCA1;GATAD2B
N;CAPZB;SUB1;CAPZA1;RUVBL1;HNRNPU;HNRNPR;DEK;TARDBP;C9ORF64
;HNRNPU;C11ORF57
-;RBBP5;HNRNPU;CLSPN;GATAD2B
HSPA4L;HNRNPU;STRN;KPNA1
SNK1A1;HNRNPU;GAPVD1;AFF4;GATAD2B;AFF1;PHF8;HNRNPK;KIF5C;SIN3A;RUVBL1;ANKRD40;TAF
40;GAPVD1;SMARCA1;SMC1A;GATAD2B;PHF8;HNRNPK;FUBP1;RUVBL1;CEP170;TARDBP;KPNA1
;HNRNPR;NUDT5;CDC42BPA;IKZF3;PCMT1;BCLAF1;HNRNPK;RUVBL1;TRIM24;CEP170;TARDBP
NRNPU;HNRNPR;NUDT5;HSPH1;MAT2A;KIF5C;RUVBL1;SNRPD3;APPL1;KPNA1;FNBP4;ST13;CSNK1A

NRNPK;SF3B3;CRBN;RUVBL1;HNRNPU;DLST;HNRNPR;PDHB
RNPU;HNRNPR;CEP170;SMC1A;ADD3;TARDBP;GATAD2B
BP
PU
RDBP
[illegible]
A4L;HNRNPU;DLST;HNRNPR;PDHB;PPP2CA;HNRNPK;RUVBL1;CCDC6;STRN;SNRPD3;SNX6
CDC6;HNRNPU;HNRNPR;HNRNPC
J;HNRNPR;HCFC2;TARDBP
ZB;RUVBL1;HNRNPU;DLST
3;ADD3;MYH10;GATAD2B
2;RUVBL1
PU;NUDT5
NPR;TARDBP
P1;HNRNPU

PR;SRGAP2
3L1;TARDBP
RNPU;TARDBP;GATAD2B
NPU;HNRNPR;ACTN4;NUDT5;SMC1A;GATAD2B;NPAT;HNRNPK;CAPZB;MAT2A;KIF5C;RBBP5;FUBP1;I
C5;KIF5C;RUVBL1;DLST;STRN;HNRNPC;TARDBP;SMC1A;GIGYF2;KPNA1
RC40;GAPVD1;SMC1A;GIGYF2;PAPSS2;GATAD2B;PCMT1;HNRNPK;XPO4;KIF5C;RBBP5;RUVBL1;SNRI
PM3;TMOD3;YLPM1;HNRNPU;HNRNPR;SMC1A;GATAD2B;HNRNPK;NKRF;CAPZA1;SERBP1;RUVBL1;T
PA4L;NOS1AP;KIAA0141;HNRNPU;HNRNPR;SMC1A;CDC42BPA;TARDBP
PU;LRRC40;DEK;GATAD2B;PHF8;PIAS1;HNRNPK;ZZZ3;CAPZB;RBBP5;RUVBL1;TARDBP;UBE2K
RNPK;RUVBL1;HNRNPU;TERF2IP;HNRNPR;TARDBP;GATAD2B
3L1;HNRNPU;HNRNPR;DEK;TARDBP;GATAD2B
K;FUBP1;RUVBL1;HNRNPU;PGAM5;TARDBP
UVBL1;HNRNPU;TARDBP;APPL1
ZB;SP1;TMOD3;RUVBL1;EIF3J;HNRNPU;HNRNPR;PDHB;TARDBP;RBM7
N3A;GRID1;RUVBL1;HNRNPU;HNRNPR;CEP170;TARDBP;PAX2;PIAS1
N;NUFIP2;HNRNPU;DLST;PTPN11;SMC1A;DYNLL2;GIGYF2;PAPSS2;GATAD2B;HNRNPK;RBBP5;FUBP1
;HNRNPU;TARDBP
J;ZNF687
K;HNRNPU
R;TARDBP
NPR;SMARCA1;SMC1A;ADD3;GATAD2B;PHF8;HNRNPK;SP1;RBBP5;NOS1AP;CEP170;TARDBP

RDBP
73
NRNPU;GIGYF2;GATAD2B;PHF8;ENAH;HNRNPK;XPO4;SIN3A;UBN2;CARM1;HNRNPC;TARDBP;ZNF68
SMC1A;DYNLL2;GATAD2B;HNRNPK;SP1;FUBP1;RUVBL1;SNRPD3;TARDBP;KPNA1
XR1;FUBP1;RUVBL1;ADD3;TARDBP;KPNA1
PA4L;HNRNPU;HNRNPR;GAPVD1;SMC1A;PHF8;PPP2CA;HNRNPK;SIN3A;TBL1XR1;FUBP1;C1QBP;CA
IPK;SF3B3;CAPRIN1;RUVBL1;G3BP1;YLPM1;HNRNPU;ZFP91;TARDBP;KPNA1
RNPU;ARID5B;NUDT5;GATAD2B;CDC73;HNRNPK;SP1;SIN3A;TRPS1;RUVBL1;TRIP12;CEP170;ZNF148
KR1;HNRNPU;DLST;GAPVD1;CDC42BPA
KR1;SIN3A;CAPRIN1;GAB1;HNRNPU;HNRNPR;HNRNPC;TARDBP;SMC1A
NRNPU;FBXO30;TNRC6B
NPU;TARDBP;RAB11A
J;HCFC2;TARDBP
HNRNPU;TARDBP
NPK;SF3B3;FUBP1;RUVBL1;HNRNPU;PGAM5;ACTN4;HNRNPC;TARDBP;GATAD2B
YTHDC1;HNRNPU;HNRNPR;ACTN4;PCMT1;HSPH1;HNRNPK;RFWD3;TRA2B;RUVBL1;HNRNPC;TARD
;HNRNPK;SF3B3;RUVBL1;HNRNPR;LONRF3
RDBP;GATAD2B
HNRNPU
K;HNRNPU
BL1;HNRNPU;CEP170;SNRPD3;HNRNPC;TARDBP;SMC1A;UBE2K;AFF4
PK;EIF2B2;RBBP5;RUVBL1;CEP170;ACTN4;TARDBP;SMC1A;KPNA1
RC40;DEK;GAPVD1;SMC1A;PAPSS2;GATAD2B;DDX19B;HNRNPK;KIF5C;RUVBL1;TARDBP;KPNA1
1;SET;HNRNPU;ACTN4;GAPVD1;GIGYF2;HNRNPK;FUBP1;PGAM5;CEP170;TARDBP
F8;NPAT;HNRNPK;SART3;XPO4;TBL1XR1;SP1;RBBP5;UBN2;RUVBL1;USP1;PDCD4;TARDBP;KPNA1
PM3;HNRNPU;HNRNPR;NUDT5;PPP2CA;HNRNPK;CAPZB;MAT2A;SUB1;FUBP1;TERF2IP;CEP170;SNR
HNRNPU;HNRNPR;GAPVD1;GATAD2B;HNRNPK;CAPZB;SNPH;RBBP5;FUBP1;RUVBL1;HNRNPC;TARD
;RUVBL1;HNRNPU;TARDBP
PK;CAPRIN1;RUVBL1;G3BP1;HNRNPU;BSN;TARDBP
HNRNPU;HNRNPR;GTF2H1;DEK;GAPVD1;GATAD2B;PHF8;HNRNPK;RRM2B;SP1;FUBP1;TRA2B;SERB
2B;DEK;GAPVD1;SMC1A;GATAD2B;PHF8;LRP6;HNRNPK;ZNF629;MAT2A;TBL1XR1;RUVBL1;TARDBP;

SF3B3;MAT2A;FUBP1;SERBP1;RUVBL1;HNRNPU;SNRPD3;ACTN4

1;PGAM5;SLC25A24
RNPU;SMC1A;DYNLL2;GATAD2B;HNRNPK;XPO4;RBBP5;CAPRIN1;RUVBL1;G3BP1;UBXN7;TARDBP;M
PU;SMC1A
HNRNPR
NRG;ARHGEF5
;SP1;RUVBL1;HNRNPU;ZNF148;TARDBP
BL1;DLST;SNRPD3;PDHB;TARDBP;DYNLL2
P
F1
RDBP
BL1XR1;SP1;CAPRIN1;RUVBL1;HNRNPU;TARDBP;ZNF687;SMC1A;GATAD2B
FUBP1;APPL1
N4;CALM1;PIAS1
1;RUVBL1;TGM2
HNRNPR;SMC1A;HNRNPK;UBN2;TCEA3;RUVBL1;NRIP1;SNRPD3;ZNF148;PPARGC1B;KPNA1
4;HNRNPU;HNRNPR;NUDT5;TARDBP
BL1;TRIM3;HNRNPU;HNRNPR;SNRPD3;SMC1A;TARDBP
PK;HNRNPU;SMC1A;TARDBP;GATAD2B;KPNA1;MED17
;HNRNPU;HNRNPR;PDHB;PCMT1;HNRNPK;SP1;TRA2B;RUVBL1;HNRNPC;TARDBP;ZC3H14;UBE2K
BL1;SNRPD3;ACTN4;TARDBP
NPR;CLSPN
L1;HSPA4L
3;PTGFRN
CAPRIN1;CARM1;RUVBL1;HNRNPU;HCFC2

1B;ABHD2;RUVBL1;HNRNPU;SNRPD3;TARDBP;DYNLL2
N;SP1;RUVBL1;TARDBP;SH3GL2;GATAD2B;PHF8
PR;PDHB;PHF8;HNRNPK;SART3;CAPZB;TBL1XR1;SP1;ATXN1L;CEP170;TARDBP
P1;KLHL7;TARDBP;PIAS1;PHF8
F3B3;TMOD3;VPS13C;YLPM1;HNRNPU;HNRNPK;CAPRIN1;SERBP1;RUVBL1;G3BP1;HNRNPC;TARDB
NPU;HNRNPR;GATAD2B
T
B
2
1

NPU

;HNRNPU;SMC1A;PHF8;HNRNPK;RXRA;SP1;RUVBL1;PDCD4;ZNF148;TARDBP;KPNA1
2B;SP1;HNRNPU;ZFP91;HNRNPR;TARDBP;GATAD2B;FAM107B
;SET;SF3B3;HNRNPU;ZFP91;HNRNPR;GTPBP4;GATAD2B;CDC73;HNRNPK;NKRF;FUBP1;RUVBL1;SNI
U;DLST;HNRNPC;TARDBP;DYNLL2;TRAK2
K;NUFIP2;TFEB;HNRNPU;BMI1;FBXO30
PSD3;UBXN7;SNRPD3
BN;HNRNPU;DLST;HNRNPR;PDHB;GATAD2B;MED17;PCMT1;HNRNPK;SP1;SUB1;RUVBL1;CEP170;TA

NPU;TARDBP
F1
HNRNPU;NUDT5;TARDBP;HK2
3L1;HNRNPU;DEK;TARDBP;GATAD2B
C40;DEK;SMC1A;GATAD2B;FOXO1;PHF8;HNRNPK;XPO4;CAPZB;RUVBL1;TARDBP;RNF111

N2;CAPZA1;YLPM1;HNRNPU;HNRNPR;HNRNPC;TARDBP;SMC1A;GATAD2B
33;XPO4;CDC37;HNRNPU;HNRNPR;BSN;TARDBP;GATAD2B;KPNA1
PN1;RBBP5;ZNF318;HNRNPU
K1;HNRNPR;SNRPD3;TARDBP
.1;HNRNPU;DEK;TARDBP
L;HNRNPU;HNRNPR;PCMT1;NXF1;BCLAF1;HNRNPK;SART3;SP1;FUBP1;RUVBL1;ZNF318;CEP170;TA
3;CDC37;TRA2B;HNRNPU;SNRPD3;NUDT5;TARDBP
DHB;DEK;PCMT1;HNRNPK;CREB1;FUBP1;RUVBL1;CEP170;SNRPD3;HNRNPC;TARDBP
INRNPU;HNRNPR;ACTN4;SMARCA1;PCMT1;HNRNPK;MYO1C;CAPZB;CAPZA1;HNRNPC;TARDBP
A1;HNRNPU;GATAD2B;HNRNPK;ZZZ3;SP1;DPYSL3;RUVBL1;G3BP1;CEP170;TARDBP;NFE2L1
NPU
DBP
T
XRCC5;HNRNPU;HNRNPR;GTF2H1;ACTN4;SMC1A;PHF8;PCMT1;HNRNPK;TBL1XR1;SP1;SIN3A;KLHL
3L1;EIF3J;HNRNPU;HNRNPR;TARDBP
B;HNRNPK;SF3B3;RUVBL1;HNRNPU
P5;RUVBL1;DLST;GATAD2B;PHF8
/BL1;GAPVD1
L1;SMARCA1
L1;HNRNPU
AIP3;ZFP91
A1;HNRNPU;HNRNPR;ACTN4;SMC1A;ESR1;AFF4;C20ORF112;HNRNPK;SIN3A;CAPRIN1;RUVBL1;G3B
3C;HNRNPU;HNRNPR;SNRPD3;HNRNPC;GLS
;RUVBL1;HNRNPU
L1;HNRNPU;GATAD2B
IPU;TARDBP;HK2
33;RUVBL1;HNRNPU;TARDBP
NPU;HNRNPR;ACTN4;TARDBP
3;RUVBL1;HSPA4L;HNRNPU;TARDBP
4;CRBN;CAPRIN1;RUVBL1;HNRNPU;CEP170
1;HNRNPU;LRRC40;STRN;CEP170;SMARCA1;SMC1A;CDC42BPA;AFF4;GATAD2B
PK;ZNF292;TET3;RUVBL1;HNRNPU;DEK;TARDBP;GATAD2B;PHF8;KPNA1
M5

NPU

RRC40
J;TARDBP;GATAD2B
HNRNPU;ADD3
VBL1;HNRNPU;GAPVD1;GIGYF2
3;GFPT1;HNRNPU;DLST;HNRNPR;PDHB;PAPSS2;HSPH1;HNRNPK;RUVBL1;CCDC6;HNRNPC;ZNF148;
NRNPU;DLST;HNRNPR;TARDBP;GATAD2B

NPU;LRRC40;STRN;SMC1A;TARDBP;UBE2K;GATAD2B
IRNPU;GATAD2B;PCMT1;HNRNPK;RBBP5;FUBP1;RUVBL1;ATXN1L;TARDBP;KPNA1
F2;GATAD2B;PHF8;HNRNPK;ATXN7;RUVBL1;CCDC6;IGF2BP1;STRN;CEP170;HNRNPC;TARDBP
PRIN1;RUVBL1
PU;JAKMIP2
A;CAPZB;XRCC5;CAPZA1;DPYSL3;RUVBL1;HNRNPU;DEK;GATAD2B;KPNA1
3;SP1;CAPZA1;RUVBL1;HNRNPU;ACTN4;HNRNPC;TARDBP;GIGYF2;PHF8
1C;DCTN2;MAT2A;TPM3;GPC1;TMOD3;RUVBL1;HNRNPU;ACTN4;MYH10
ATXN7
BL1
IPU;ZFP91;RSF1;GTPBP4;KPNA1
I;CAPRIN1;HSPA4L;HNRNPU;XIAP
K;KLHL7;RUVBL1;HNRNPU;CEP170;GATAD2B
B3;CRBN;RUVBL1;HNRNPU;SNRPD3;ACTN4;KPNA1
3;TBL1XR1;HNRNPU;HNRNPR;CEP170;HNRNPC;TARDBP
N;TPM3;SP1;DYRK1A;HNRNPU;ACTN4;TARDBP
TPM3;GAB1;HNRNPU;LRRC40;ACTN4;GAPVD1;PCMT1;HNRNPK;CAPRIN1;RUVBL1;PDCD4;TARDBP;S
BL1;DLST;GATAD2B;MED17
C1A;TARDBP;DTL;GIGYF2
PU;HNRNPC;TARDBP;MTDH
;HNRNPU;DLST;UBE2K
HNRNPU;DLST;HNRNPR;PHF8;RNF214;HNRNPK;C1QBP;RUVBL1;G3BP1;CEP170;TARDBP
N2;HNRNPU;ZFP91;AFF4;AFF1
P1;HNRNPU;TGM2
NPU;DYNLL2
L1;TARDBP
HNRNPC;TARDBP
CRBN;TPM3;HNRNPU;DLST;ACTN4;PDHB;PFAS
M3;FUBP1;RUVBL1;PDCD4;XIAP;TARDBP;SMC1A;GATAD2B;KPNA1
7;SF3B3;NUFIP2;YLPM1;HNRNPU;HNRNPR;GAPVD1;NUDT5;GATAD2B;HNRNPK;RXRA;CAPRIN1;SER
IN1

C5;RUVBL1;TET3;HNRNPU;STRN;CEP170
U;STRN;SMC1A
3J;HNRNPU;HNRNPC
N;XRCC5;HNRNPU;HNRNPR;GAPVD1;SMARCA1;ADD3;ADD2;PCMT1;HNRNPK;KIF5C;RUVBL1;SNRPI
2;HNRNPU;ZFP91;HNRNPR;GTF2H1;GTPBP4;HNRNPK;SART3;TBL1XR1;CAPRIN1;G3BP1

DHAC2;PIAS1
IPU;CEP170
RNPU;HNRNPR;PDHB;HK2;PHF8;PCMT1;RNF214;PABPN1;KIF5C;IGF2BP1;N4BP2;KPNA1;TGM2;ACTI
3A;HNRNPU;CDC73;GATAD2B;TGM2
RDBP;DYNLL2;GIGYF2;GATAD2B
RBBP5;RUVBL1;HNRNPU;STRN;WDFY3;TARDBP;SMC1A;PHF8
K;XPO4;RUVBL1;HNRNPU;TARDBP;DYNLL2;UBE2K;GATAD2B;KPNA1
P1;RUVBL1;G3BP1;HNRNPU;TERF2IP;HNRNPR;CEP170;APPL1
K;SP1;RUVBL1;HNRNPU;CEP170;CALM1;TARDBP;NUPL1;GATAD2B;KPNA1
;CRBN;HNRNPU;HNRNPR;DEK;CBFA2T2;TARDBP
5;HNRNPK;APC;HNRNPU;TARDBP;N4BP2;CDC73
7;SF3B3;YLPM1;DYNLL2;HNRNPK;FUBP1;CAMK4;CAPRIN1;SERBP1;G3BP1;SNRPD3;TARDBP
C1A;TARDBP
BL1;PRUNE2

PU;TARDBP
DBP;GATAD2B
N1;G3BP1
PA4L;HNRNPU
U;SMC1A;DYNLL2;GATAD2B;PHF8;PCMT1;HNRNPK;KIAA0355;RUVBL1;NRIP1;TARDBP;PPARGC1B;K
33;PSMD11;HNRNPU;DLST;HNRNPR;GAPVD1;GIGYF2;ACAP2;HNRNPK;RUVBL1;HNRNPC;TARDBP;ZN
1;RUVBL1;HNRNPU;ZFP91;HNRNPR
9X;HNRNPU;DYNLL2;GATAD2B
P1;HNRNPU;HNRNPR;GATAD2B

NRNPU;DLST;HNRNPR;GAPVD1;SMC1A;SMARCA1;AFF4;GATAD2B;MED17;PHF8;PPP2CA;HNRNPK;S
3;RUVBL1;HNRNPU;DLST;MTSS1L;HNRNPR;TARDBP;ZC3H14;PHF8
C;RUVBL1;HNRNPU;GTF2H1;TARDBP;GTPBP4;KPNA1;SMAD7
NPR;DEK;GATAD2B;ENAH;HNRNPK;NFIA;SP1;TRA2B;RUVBL1;CCDC6;ZNF148;TARDBP;ZNF687
P;GIGYF2;GATAD2B
1;KIF1B;CALM1
BL1;HNRNPU;HNRNPR
BL1;HNRNPU;DLST
BP5;RUVBL1;LRRC40
1;HNRNPU;TARDBP
BP
IPK
M1
11FIP5
HNRNPU;HNRNPR;ACTN4;DEK;SMARCA1;DYNLL2;PCMT1;HNRNPK;SUB1;RUVBL1;SNRPD3;HNRNP
;HNRNPU;DLST;SNRPD3;TARDBP;DYNLL2;GATAD2B

NPK;APC;SP1;HNRNPU;STRN;TARDBP
BL1;DLST;YOD1;PDHB;TARDBP
VBL1;HNRNPU;HNRNPR;PGAM5;TARDBP
PU;DLST
PU;TARDBP
RNPR;TARDBP
NPR;SMARCA1;GATAD2B;NPAT;HNRNPK;FCHSD2;RUVBL1;STRN;HNRNPC;ZNF148;TARDBP
BP1;GATA6;HNRNPU;XIAP
INRNPU;ZFP91;HNRNPR;GTF2H1;DEK;PHF8;PCMT1;HNRNPK;SART3;SIN3A;KIF5C;TBL1XR1;RUVBL1
BL1;EIF3J;DAG1;HNRNPU;STRN;SNRPD3;DEK;SMARCA1;TARDBP;ZNF687
A;RRM2B;SP1;RUVBL1;HNRNPU;TRIP12;HNRNPC;CC2D1B;KPNA1

EP
U
DBP

NPU;HNRNPR;TARDBP;TNRC6B
HNRNPU;SMC1A;GIGYF2;PCMT1;HNRNPK;SP1;RUVBL1;CEP170;SNRPD3;TARDBP
USP9X;CAPRIN1;HNRNPU;DYNLL2
;SF3B3;USP9X;HNRNPU;HNRNPR;SMARCA1;GTPBP4;HNRNPK;RXRA;NKRF;SUB1;RUVBL1;SNIP1;TR
RCC5;TPM3;HNRNPU;ZFP91;HNRNPR;SMARCA1;GATAD2B;PHF8;HNRNPK;FUBP1;TRPS1;RUVBL1;G3

.37;SF3B3;HNRNPU;DLST;DEK;SMC1A
NPU
J;HNRNPR
2;HNRNPU
<;KIF5C;SUB1;FUBP1;RUVBL1;HNRNPU;TARDBP;SMC1A
IRNPK;SF3B3;FUBP1;RUVBL1;HNRNPU;HNRNPR;TARDBP
HDC1;HNRNPU;HNRNPR;ACTN4;GATAD2B;PCMT1;HNRNPK;KIF5C;RBBP5;RUVBL1;TARDBP;SRGAP2

SMC1A;GIGYF2;HNRNPK;KIF5C;SP1;SIN3A;RUVBL1;CEP170;TARDBP;MYH10;KPNA1
IPU;RAB3GAP2;HOOK3
RUVBL1;HNRNPU
IPR;PGAM5;SNRPD3;TNRC6B
3L1;ZNF318;HNRNPU;TARDBP
IPU;DLST;RSF1;SNRPD3;HNRNPC;GATAD2B
3L1;HNRNPU;ZNF148;SMC1A;TARDBP;PHF8
PU;HOOK3;DEK;GAPVD1;SMC1A;GATAD2B;PCMT1;HNRNPK;ZZZ3;SIN3A;RUVBL1;KIF1B;TRIP12;TARD
IPU;HNRNPR;SNRPD3;TARDBP;GATAD2B
3P;SNX8
PU;CDC73
IPK;HNRNPU
PU;TARDBP
CRBN;ROCK1;XRCC5;TPM3;VPS13C;HNRNPU;HNRNPR;DEK;SMARCA1;GTPBP4;PCMT1;RUVBL1;TR

NPU
BP
SPA4L;HNRNPU
5;HNRNPU;TARDBP
RUVBL1;HNRNPU;ACTN4;TARDBP;SMC1A;UBE2K;CDC73;KPNA1
;RUVBL1;HNRNPU;DLST;SNRPD3;TARDBP
IN1;RUVBL1;HNRNPU;ZNF148;HK2;PHF8
;RUVBL1;HNRNPU;GTF2H1;TARDBP;AFF4;GATAD2B;KPNA1
C5;USP9X;KIF5C;ZNF318;HNRNPU;LRRC40;HNRNPR;UBE2K;GATAD2B;KPNA1

VBL1;HNRNPU;LRRC40;TARDBP;GATAD2B
IPR;HNRNPC;KCNRG;TARDBP;MED17

<PO4;RUVBL1;HNRNPU;PDAP1;SMARCA1;CBFA2T2
IPK;HNRNPU;DLST;SMC1A;TARDBP;PHF8
VBL1;HNRNPU
RDBP;GATAD2B;PHF8
PK;EIF2B2;RRM2B;RUVBL1;HNRNPU;HNRNPR;HNRNPC;GTPBP4;CC2D1B;KPNA1
.1;HNRNPU;DLST;TARDBP
C42BPA

B
CAPRIN1;SERBP1;G3BP1;HNRNPU;ACTN4;ZNF148;TARDBP;GIGYF2
;HNRNPU;SNRPD3;SMC1A;GATAD2B
PU;GAPVD1;DYNLL2;AP2M1;CKS1B
RUVBL1;HNRNPU;HNRNPR;PHF8
K;SF3B3;SERBP1;RUVBL1;G3BP1;YLPM1;HNRNPU;SNRPD3;TARDBP;SMNDC1

NPR;GAPVD1;SMC1A;GNL1;HNRNPK;SART3;MYO1C;RUVBL1;UBXN7;TERF2IP;CEP170;TARDBP;KPN
RNPR;SNRPD3;TARDBP
IRNPK;GAPVD1
PU;GAPVD1;GATAD2B;PHF8;HNRNPK;KIF5C;TBL1XR1;SP1;RUVBL1;G3BP1;CEP170;TARDBP
TARDBP

.1;HNRNPU;LRRC40;GIGYF2;KPNA1
BP1;RUVBL1;HNRNPU;DEK;TARDBP;ZNF687;SMC1A;GATAD2B
AM5
MIP2
TARDBP
RNPU;GAPVD1;TARDBP;KPNA1;TRAK2
TPM3;HNRNPU;HNRNPR;ACTN4;SMARCA1;DYNLL2;GATAD2B;HSPH1;HNRNPK;NSD1;TRPS1;RUVBL
BL1;HNRNPC
.3;GATAD2B
1;HNRNPU;HNRNPR;BSN;SMC1A;TARDBP
;SUB1;RUVBL1;HNRNPU;HNRNPC;SMC1A;TARDBP

PC

BL1;MSL2;HNRNPU;HNRNPR;HNRNPC;HCFC2;TARDBP;ASPRV1
IRNPU;LRRC40;ACTN4;PCMT1;HSPH1;HNRNPK;SIPA1L1;SERBP1;RUVBL1;SNRPD3;TARDBP
P;HCFC2;MTDH
D9;HNRNPR;SORBS3
28;SF3B3;DCTN2;HNRNPU;HNRNPR;DEK;AFF4;PHF8;HNRNPK;CAPZB;SIN3A;CDC37;RUVBL1;BSN;TA
K4;G3BP1;KIAA0141;HNRNPU;HNRNPR;PDHB;GIGYF2
U;HNRNPR;ZBTB44;GTPBP4;PHF8;BCLAF1;HNRNPK;SART3;RBBP5;STRN;TRIP12;SNRPD3;HNRNPC
SD2;RUVBL1;HNRNPU;TARDBP
L1;HNRNPU;CALML4;ZNF148;GATAD2B;PHF8
BL1;G3BP1;TARDBP
RDBP
U
RNPK;RUVBL1;HNRNPU;SNRPD3;TARDBP;ZNF687;GIGYF2
NPU;DLST;PARVA;PDHB;PHF8;PCMT1;HNRNPK;SIN3A;FUBP1;PGAM5;CEP170;HNRNPC;TARDBP

P;CCDC50
NPU;CCDC50
5;HNRNPU;SMC1A;PHF8;PIAS1;HNRNPK;ZZZ3;SP1;RUVBL1;ATXN1L;TARDBP
R1;SP1;RUVBL1;HNRNPU;HNRNPR;ZNF148;TARDBP;SMC1A;GATAD2B
RNPU;HNRNPR;SMC1A;GATAD2B;PHF8;ENAH;HNRNPK;TBL1XR1;SP1;RBBP5;RUVBL1;TERF2IP;CEP
BL1;HNRNPU;SMC1A;TARDBP;RAB11A
TXN7;TMOD3;HNRNPU;HOOK3;MYH10
C6;HNRNPU;HNRNPR;WDFY4

NPU;CEP170;TARDBP
VBL1;CEP170;TARDBP
RNPU;TARDBP;GIGYF2
RDBP;GATAD2B;RAB11A
NPU;BMI1;RBM7
XR1;RUVBL1;HNRNPU;DLST;HNRNPR;DEK;TARDBP;SMC1A;GATAD2B
NPU
NPU;PHF8
3;E2F1;HNRNPU;SMC1A;TARDBP;GATAD2B
PZB;HNRNPU;TERF2IP;TARDBP
;HNRNPU;HNRNPR;DEK;TARDBP;CDC42BPA;GATAD2B;PHF8
P1;G3BP1;HNRNPU;LRRC40;HNRNPR;CEP170;DYNLL2;GIGYF2;PUM2
U;HNRNPR;SNRPD3

NPU
L1
2
ST;NUPL1;GIGYF2;PUM2
L1;HNRNPU
RNPU;TARDBP
RNPU;LRRC40;TARDBP;SMC1A;DYNLL2;GATAD2B;PHF8
PFKFB3;HNRNPU;HNRNPR;SMARCA1;DYNLL2;PCMT1;ITCH;NOS1AP;TRIP12;SNRPD3;HNRNPC
2IP;HNRNPC;PDHB;SMC1A;GATAD2B
N;RUVBL1;PCDHA5;HNRNPU;TARDBP

HNRNPU;GAPVD1;SMC1A;GIGYF2;GATAD2B;PIAS1;PCMT1;HNRNPK;RBBP5;FUBP1;RUVBL1;TARDBP
N1L;HNRNPU;HNRNPC;TARDBP
NPR;GTF2H1;SNRPD3;DYNLL2
J;SNX8
11A
RUVBL1;HNRNPU;ZNF148;TARDBP;GATAD2B;KPNA1
XR1;SP1;HNRNPU;TERF2IP;HNRNPR;HNRNPC;TARDBP;KPNA1
A;RUVBL1;HNRNPU;DLST;SNRPD3;GATAD2B
NPK;CAPRIN1;HNRNPU
ACTN4;HNRNPK;SIN3A;CDC37;FUBP1;RUVBL1;CCDC6;CEP170;SNRPD3;HNRNPC;TARDBP

RDBP;GATAD2B
33;RUVBL1;HNRNPU;DEK;SMC1A
PK;HNRNPU;PGAM5

PU
CAPRIN1;FUBP1;G3BP1;LRRC40;GTPBP4
3;HNRNPK;TRA2B;RUVBL1;TARDBP
2B;RUVBL1;HNRNPU;SMC1A;PUM2
PK;RUVBL1;HNRNPU

.1;HNRNPU;HNRNPR;ADD3;DYNLL2;APPL1
'U;HNRNPR;KPNA1
D3;GAPVD1;TARDBP
IPU;TARDBP
HNRNPU
KRCC5;HNRNPU;HNRNPR
IRNPU;ZNF687;CCDC50;RAB11A
3;CRBN;CAPZB;RUVBL1;HNRNPU;EPC1;HNRNPR;TARDBP
B34
PU
NPU

NPU;CEP170;TARDBP;AFF4
NPU;HNRNPR
PK;RUVBL1
SF3B3;PSMD11;CRBN;TPM3;RUVBL1;HNRNPU;LRRC40;TARDBP
HNRNPU;HNRNPR;DEK;SMARCA1;GTPBP4;BCLAF1;HNRNPK;SIN3A;C1QBP;RUVBL1;PGAM5;SNRPD
NRNPK;SF3B3;SART3;NFIA;HNRNPU;HNRNPR;SNRPD3;TARDBP;DYNLL2;PHF8
PR
IRNPK;SF3B3;RUVBL1;HNRNPU;DLST;HNRNPR;TARDBP;CDC42BPA

NPR;HNRNPC;GATAD2B
N2;GATAD2B;KPNA1
P1;RUVBL1;HNRNPU;TARDBP
U;HNRNPR;DEK;GAPVD1;SMC1A;DYNLL2;GATAD2B;PCMT1;RNF214;HNRNPK;UBN2;RUVBL1;SNRPD
ERBP1;HNRNPU

)4;RBBP5;FUBP1;RUVBL1;HNRNPU;HNRNPR;ZBTB44;TARDBP;GATAD2B
N;RUVBL1;HNRNPU;RAB3GAP2;GAPVD1;TARDBP
'0;GAPVD1;TARDBP;AP2M1
L1;HNRNPU;TARDBP
I1;SRGAP2;GIGYF2
T;PDHB;TARDBP
3;RUVBL1;HNRNPU;UBXN7;HNRNPR;TARDBP;CDC73

3
NPU
NPU;SMC1A
);HNRNPK;CRBN;RUVBL1;HNRNPU;SNRPD3;TARDBP;SMC1A;PHF8;KPNA1
PK;EIF2B2;CAPRIN1;RUVBL1;G3BP1;HNRNPU;TARDBP;CDC42BPA;DYNLL2;GATAD2B

CARM1;RUVBL1;HNRNPU;HNRNPR;SNRPD3;TARDBP;KPNA1
PU;LRRC40;SMC1A;GATAD2B;HNRNPK;ZZZ3;SIN3A;RBBP5;RUVBL1;USP1;PDCD4;TARDBP
M5
DBP
7;SIPA1L1;NUFIP2;CSNK1A1;CAPRIN1;RUVBL1;DYRK1A;HNRNPU;GAPVD1
SF3B3;MAML1;SP1;RUVBL1;HNRNPU;TARDBP;SMC1A;CDC73
1;RUVBL1;HNRNPU;DLST;ACTN4;TARDBP;AFF4;GATAD2B

ɪPK;TBL1XR1;SP1;HNRNPU;ZFP91;HNRNPR;ZNF148;TARDBP

DBP;GATAD2B
RNPU;BSN
RNPU;TARDBP

RNPU
R
U
M5
D3;HNRNPU;DLST;TARDBP

BL1;HNRNPU;DLST;HNRNPR;HNRNPC;SMARCA1;TARDBP;ZC3H14;GATAD2B;KPNA1
2;HSPA4L
3;SIN3A;HNRNPU;HNRNPR;HNRNPC;TARDBP
VBL1;HNRNPU;HNRNPR;TARDBP;GTPBP4;JAKMIP2

PU;GAPVD1;DYNLL2
I;XRCC5;TPM3;SP1;DYRK1A;HNRNPU;STRN;SNRPD3;ACTN4;TARDBP
);CDC42BPA;TARDBP;GIGYF2
;HNRNPU;TARDBP;PPARGC1B
RNPU;RASSF8
T;TARDBP

B

ST;TARDBP
PU;GIGYF2
N1;HNRNPU;CEP170
B2;ESCO1;LRRC40;GAPVD1;SMC1A;GATAD2B;HNRNPK;SP1;RUVBL1;CEP170;TARDBP
PK;HNRNPU;PGAM5;MYH10
CSNK1A1;HNRNPU;DLST;HNRNPR;SMARCA1;SMC1A;HNRNPK;SIN3A;CEP170;HNRNPC;TARDBP

CAPRIN1;RUVBL1;HNRNPU;TARDBP;KPNA1
RUVBL1;HNRNPU

C1A;DYNLL2;GATAD2B;PHF8;PIAS1;HNRNPK;RBBP5;FUBP1;RUVBL1;TARDBP;SRGAP2;KPNA1
3;HNRNPU;HNRNPR;PHF8
BL1;HNRNPR;RSF1;GIGYF2
K;SF3B3;SERBP1;YLPM1;HNRNPU;SNRPD3;HNRNPC;TARDBP;SMNDC1
P170
ACTR4

RUVBL1;HNRNPU;CEP170;NUPL1
NPU;CEP170;IKZF3
IPU;STRN
IPU;TARDBP

P1;RUVBL1;HNRNPU;GATAD2B

-

NPU;RAB3GAP2
PC;TARDBP
K;FUBP1;SMC1A;GATAD2B
IPU;HNRNPR;SMC1A;TARDBP;KPNA1
GD2;TPM3;RUVBL1;HNRNPU;ACTN4
PU;ZFP91;GAPVD1;PAPSS2;GATAD2B;HNRNPK;TBL1XR1;CARM1;RUVBL1;TARDBP;WDFY4;UBE2K

VD1
U;GIGYF2;PHF8;PPP2CA;HNRNPK;CAPZB;RUVBL1;TERF2IP;CEP170;TARDBP;ZNF687
B;TPM3;SP1;HNRNPU;TERF2IP;SNRPD3;TARDBP

XRCC5;RUVBL1;HNRNPU;HNRNPR;SNRPD3;HNRNPC;TARDBP;CDC73
RNPU;SMC1A;PHF8
PK;SF3B3;YLPM1;HNRNPU

J;HNRNPR;GIGYF2;GATAD2B;PHF8;HNRNPK;RBBP5;KLHL7;CAPRIN1;RUVBL1;G3BP1;UBE2K;KPNA1
BL1
RNPU;SMC1A
T3;CAPZB;HNRNPU;LRRC40;GAPVD1;RIMKLA;KPNA1
BL1;HNRNPU;HNRNPR;GAPVD1;ZNF148;TARDBP;DYNLL2
H3KBP1;GAPVD1;SMC1A;GATAD2B;HNRNPK;APC;CDC37;RUVBL1;SNRPD3;N4BP2;UBE2K;KPNA1
PU;DLST;CEP170;HNRNPC;PDHB;GAPVD1;TARDBP
BL1;HNRNPU;SNRPD3;TARDBP;ASPRV1;MYH10;RAB11A

HNRNPU;TARDBP
NPU;DLST;SNRPD3;DYNLL2;GTPBP4;GIGYF2
HNRNPU
BP1;KIF26B;MSL2;IGF2BP1;HNRNPU;CEP170;DYNLL2;EIF4E
.1;CAPZA1;RUVBL1;HNRNPU;ACTN4
;ACTN4;SMARCA1;GATAD2B;MED14;HNRNPK;SIN3A;FUBP1;RUVBL1;HNRNPC;TARDBP

NPU;MYH10
CD4;HNRNPU
BL1;HNRNPU;RBM7;TARDBP

B3GAP2
AD2B
C42BPA
T11;HNRNPU;PHF8;PCMT1;HNRNPK;SP1;TRA2B;RUVBL1;TERF2IP;HNRNPC;TARDBP;UBE2K;KPNA1
BP;SLC25A24
U;HNRNPR
RNPR;GAPVD1;CBFA2T2

;P2
;HSPA4L;HNRNPU;CEP170;RAF1
;HNRNPU;LRRC40;SMC1A;PAFAH1B2
3L1;SMC1A;GATAD2B;MED17

IPU;DLST;SNRPD3
.1;HNRNPU;TARDBP
5;CEP170;KPNA1
PK;TPM3;RUVBL1;HNRNPU;TRIP12;ACTN4;MYH10;KPNA1;JAKMIP2
CAPZB;ATXN1L;HNRNPU
NPU;TARDBP
AM5;TARDBP
P;PUM2
3;SIN3A;RUVBL1;HNRNPU;HNRNPR;HNRNPC;PDHB;TARDBP;GATAD2B
PK;RUVBL1;HNRNPU;XIAP;HNRNPR;TARDBP;SMC1A;PHF8

RNPU
NRNPU
IPU;TARDBP
HNRNPU;TARDBP;PAPSS2

NPU;HNRNPC;TARDBP
1;HNRNPU;SNRPD3
;CDC42BPA

1;TERF2IP;GATAD2B
3P
PU

1;RUVBL1;HSPA4L;HNRNPU
RDBP;SNX6
IPU;ZBTB4;KPNA1
;HNRNPU;HNRNPR;DYNLL2
1L;HOOK3
DBP
F687
RNPU;TRIP12;ZNF148;TARDBP;SMC1A;MYH10;PAPSS2;GATAD2B;PHF8

PK;RUVBL1;HNRNPU;ZFP91;TARDBP
1;HNRNPU;KIF1B;ACTN4;TARDBP

1XR1;RUVBL1;TRIM24;HNRNPU;SMC1A;GATAD2B;PHF8;KPNA1
3L1;HNRNPU;LRRC40;RAB3GAP2;PGAM5;KCNRG;APPL1
U;HNRNPR;PHF8;PCMT1;HNRNPK;TBL1XR1;SP1;RUVBL1;TERF2IP;HNRNPC;TARDBP;ZNF687

RNPU;LRRC40;UBE2K
.ST;GAPVD1
VBL1;PDHB

PD3;SRGAP2;CDC42BPA

PK;RUVBL1;HNRNPU;PGAM5;TARDBP
.1;HNRNPU;KPNA1
PXD2A;HNRNPU;CEP170

PU
ID5;HNRNPU;ACTN4;DEK;GAPVD1;SMC1A;DYNLL2;GATAD2B;TIAL1;HNRNPK;RBBP5;TBL1XR1;RUVB
28;HNRNPK;HNRNPU;HNRNPR;HNRNPC;SMARCA1;TARDBP;GTPBP4;KPNA1;JAKMIP2

RNPU

BL1;TARDBP
ST
BL1;HNRNPU

C40;ZFP91;GAPVD1;SMC1A;GATAD2B;HNRNPK;SP1;RUVBL1;ZNF318;CEP170;ZNF148;TARDBP;KPN

X;SP1;RBBP5;TDG;RUVBL1;HNRNPU;SNRPD3;SMTNL2;TARDBP;GATAD2B
7;HNRNPK;SF3B3;SH3KBP1;NKRF;HNRNPU
BL1;HNRNPU;HNRNPR
RNPU;DLST;SNRPD3;HNRNPC;TARDBP;SMC1A;GATAD2B
40;SMC1A;PAPSS2;PHF8;PCMT1;HNRNPK;RUVBL1;G3BP1;CEP170;ZNF148;TARDBP;KPNA1
SNPH;RUVBL1;HNRNPU;CBFA2T2;TARDBP;CDC73;AFF1

RNPU;HNRNPR;ACTN4;SMC1A;HNRNPK;TBL1XR1;FUBP1;SNRPD3;TARDBP;UBL7

RC40;SNX8;GATAD2B;KPNA1
B;TARDBP
RNPU;TARDBP

3J;HNRNPU;SNIP1;HNRNPR;TARDBP
B;HNRNPK;RUVBL1;HNRNPU;TARDBP;GATAD2B
RCA1
IPU;GATAD2B
F2;PUM2

YLPM1;HNRNPU;TARDBP
VD1;SMC1A;KPNA1

;TPM3;RUVBL1;DUSP19;HNRNPU;SRGAP2

RNPU;TARDBP

EL2;CAPRIN1;RUVBL1;HNRNPU;TARDBP;CDC73

;HNRNPU
4
XR1;SP1;RUVBL1;HNRNPU;HNRNPR;ANKRD40;CEP170;TARDBP
RA;RUVBL1;CEP170;GAPVD1;SMC1A;GIGYF2;PHF8;KPNA1

BP1;RUVBL1;HNRNPU;HNRNPR;CEP170;TARDBP;SMC1A;GATAD2B;KPNA1

RNPU

M5

RNPR;HNRNPC;GATAD2B

;XPO4;FUBP1;RUVBL1;HNRNPU;HNRNPC;TARDBP;GATAD2B
RNPU;DLST;PAFAH1B2;MED17

NRNPU;GAPVD1

NPU;HNRNPR;HNRNPC;TARDBP

ZB;SUB1;RUVBL1;PDCD4;HNRNPU;GAPVD1;GATAD2B;KPNA1

RNPU;ZNF687

1;HNRNPU

3L1;HNRNPU;LRRC40;SMC1A;GIGYF2

NPU;SMC1A

RDBP

2K1;XRCC5;SFMBT2;GAPVD1;HCFC2;SMC1A;DYNLL2;GATAD2B;PIAS1;DIAPH2;HNRNPK;NFIA;TBL1X
SNPH;CAPRIN1;G3BP1;IGF2BP1;HNRNPC;CLSPN;ZNF148;TARDBP;LPHN2;RAPGEF6
1;TPM3;XRCC5;VPS13C;TMOD3;YLPM1;MSL2;GAPVD1;GATAD2B;HNRNPK;MYO1C;SP1;RFWD3;CAPF
;MYH10;KPNA1;TSFM;SEPT11;PCYT1B;CRBN;TPM3;XRCC5;TMOD3;ZFP91;ACTN4;GAPVD1;SMC1A;S
IAPH2;HNRNPK;ZZZ3;FUBP1;RFWD3;CAPRIN1;G3BP1;CEP170;TARDBP

M3;ACTN4;SMC1A;DYNLL2;RAB11A;HNRNPK;MYO1C;APC;FUBP1;CAPZA1;CARM1;TERF2IP;HNRNPC

MAP2K1;GTF2H1;SMC1A;GATAD2B;ALDH6A1;HNRNPK;KLHL7;EIF3J;CCDC6;TARDBP;NUPL1;CRK
EP170;HNRNPC;TARDBP;ZNF687;MYH10;CD244;RAB11FIP5
DBP;RAB11FIP5

XN1L

;TARDBP;KPNA1

RDBP

HL7;RUVBL1;PTGFRN;TARDBP

DYNLL2;GATAD2B;PTP4A1;HNRNPK;SP1;FUBP1;STRN;TARDBP;UBE2K

BP1;CCDC6;RAB3GAP2;STRN;CEP170;KPNA1;PHACTR4

F2IP;STRN;SNRPD3;TARDBP;ZNF687;CGGBP1;APPL1

108;CEP170;SLIT2;TARDBP;KPNA1

;CPSF7;CSNK1A1;CBFA2T2;DYNLL2;ACAP2;HNRNPK;CAPRIN1;HNRNPC;TARDBP;UBE2K;LNX2

GAPVD1;SMC1A;GATAD2B;HNRNPK;SUB1;FUBP1;KLHL7;LCOR;TARDBP
C40;ACTN4;SMC1A;CDC42BPA;DYNLL2;GATAD2B;HNRNPK;TBL1XR1;SP1;FUBP1;TARDBP;UBE2K
SNRPD3;HNRNPC;TARDBP;SMNDC1
;KIF5C;FUBP1;UBN2;KIAA0895;RUVBL1;HNRNPC;MYH10
12;HNRNPC;TARDBP

M5;CEP170;CGGBP1;AP2M1

BP1;CEP170;TARDBP;KPNA1

1;TRIM24;UBXN7;TRIP12;SNRPD3;RBM12;TARDBP;UBE2K

1;CEP170;ZNF148;CALM1;TARDBP

PZB;TBL1XR1;RBBP5;RUVBL1;TRIP12;ANKRD40;TARDBP;MYH10
ARDBP;SRGAP2;MYH10
40;CEP170;HNRNPC;TARDBP;KPNA1
;SIN3A;RBBP5;FUBP1;RUVBL1;TARDBP
SNRPD3;KPNA1;SNX6;CRBN;ST13;USP9X;TPM3;XRCC5;TMOD3;SORD;PDAP1;ACTN4;DEK;SMC1A;S

;TARDBP;SRGAP2;ZC3H14
12;MYH10
I;ACTN4;DEK;SMC1A;HNRNPK;SUB1;FUBP1;SERBP1;CCDC6;HNRNPC;TARDBP
7;CPSF7;XRCC5;ACTN4;DIAPH2;HNRNPK;TBL1XR1;SUB1;FUBP1;TARDBP

UBP1;RUVBL1;OCIAD1;TARDBP

C5;LRRC40;SMC1A;DYNLL2;GATAD2B;PIAS1;HNRNPK;SP1;FUBP1;TERF2IP;TARDBP

RDBP;KPNA1
RUVBL1;HNRNPC;TARDBP;MYH10;UBE2K

KPNA1
DBP
3A;FUBP1;TRIM3;RUVBL1;TERF2IP;CEP170;TARDBP;ZNF687;RAPGEF6

IC1A;GATAD2B;HNRNPK;ZZZ3;TBL1XR1;SP1;STRN;MAPRE3;HNRNPC;TARDBP;PAFAH1B2

RRC40;HOOK3;GAPVD1;SMC1A;GATAD2B;FOXP2;HNRNPK;RRM2B;GPAM;TBL1XR1;PDCD4;TARDBP;
RBBP5;FUBP1;C1QBP;RUVBL1;CCDC6;TRIP12;TARDBP;KPNA1

;TARDBP;ZNF687;NUPL1

TN4;SMARCA1;DYNLL2;ESR1;HNRNPK;MYO1C;CAPZA1;SERBP1;STRN;HNRNPC;TARDBP

RUVBL1;SNRPD3;HNRNPC;TARDBP

TARDBP

TARDBP

;SP1;SUB1;RUVBL1;CEP170;SNRPD3;TARDBP;ZNF687
170;TARDBP;KPNA1
SNRPD3;TARDBP;RAB11FIP5
1;RUVBL1;TARDBP

NBP4;XRCC5;GAPVD1;SMC1A;GATAD2B;HNRNPK;SP1;FUBP1;CAPZA1;TERF2IP;TARDBP
2;TARDBP;KPNA1

KPNA1
NPC;ZNF148
VBL1;ZNF318;CEP170;ZNF148;TARDBP;RBM7;SNX8

F8;HNRNPK;TBL1XR1;SUB1;RUVBL1;SNRPD3;TARDBP

HNRNPC;TARDBP

NPK;SP1;FUBP1;CAPZA1;CAPRIN1;SERBP1;CCDC6;ANKRD40;TARDBP
DC42BPA;ESR1;HNRNPK;MYO1C;SP1;CAPZA1;CAPRIN1;HNRNPC;TARDBP

SEPT10;RABGAP1L;TPM3;LRRC40;SMAD9;SMC1A;SMARCA1;GATAD2B;HNRNPK;ZZZ3;FUBP1;CAPZA

MNDC1

;CAPRIN1;SERBP1;G3BP1;TARDBP;SRGAP2;MYH10
\1;GTF2H1;ACTN4;SMARCA1;GATAD2B;GTF2H5;HNRNPK;CDC37;TRIP12;ANKRD40;TARDBP
;FUBP1;RUVBL1;RAB3GAP2;CEP170;DTL;KPNA1
70;SNRPD3;HNRNPC;TARDBP;KPNA1

UBE2K;SOS2

BP;UBE2K;DCP2;TNRC6B
_1;CEP170;TARDBP
P1;RUVBL1;HNRNPC;CLSPN;TARDBP;SNX8;UBE2K;C11ORF57
UBN2;CAPZA1;RUVBL1;CEP170;HNRNPC;ZNF148;TARDBP;KPNA1

3P
CD4;RAB3GAP2;SNRPD3;TARDBP;UBE2K;APPL1;KPNA1
IARCA1;SMC1A;GATAD2B;HNRNPK;TBL1XR1;SP1;CAPRIN1;ZNF318;TERF2IP;TARDBP
;HCFC2;GATAD2B;RAB11A;HNRNPK;TBL1XR1;SP1;SUB1;HNRNPC;TARDBP
SNRPD3;ST13;TPM3;ACTN4;HNRNPK;NFIA;FUBP1;HNRNPC;TARDBP

;SERBP1;RUVBL1;ZNF318;G3BP1;TARDBP

.1;IGF2BP1;KPNA1;ST13;TPM3;XRCC5;GFPT1;ACTN4;HNRNPK;CDC37;CAPZA1;CTNNB1;HNRNPC;TA

_1;TARDBP;MYH10

.1;FNBP4;UTP15;CSNK1A1;XRCC5;ACTN4;SMC1A;GATAD2B;HNRNPK;SP1;FUBP1;CAPRIN1;SERBP1;

70;SNRPD3

PVD1;SMC1A;GATAD2B;HNRNPK;SP1;CAPRIN1;RAB3GAP2;TERF2IP;TARDBP;UBE2K

;G3BP1;CTNNB1;STRN;ZNF148;TARDBP;ZNF687

RNPK;XPO4;CAPRIN1;RUVBL1;G3BP1;TARDBP;ZNF687;PAFAH1B2;APPL1
VBL1;HNRNPC;MYH10

5;RFWD3;RUVBL1;CEP170;TARDBP;ZNF687;ZC3H14;PHACTR4

4;SEPT10;DEK;SMC1A;GATAD2B;HNRNPK;SP1;FUBP1;TARDBP;UBE2K

UVBL1;G3BP1;CEP170;ZNF148;ZNF687;MAP2K1;CSNK1A1;XRCC5;SMC1A;SMARCA1;DYNLL2;GTPBF

C5;VPS13C;TMOD3;YLPM1;PDAP1;ACTN4;DEK;SMARCA1;GATAD2B;HNRNPK;MYO1C;NFIA;SUB1;FUI
KR1;SP1;FUBP1;RUVBL1;TARDBP;KPNA1

P170;TARDBP;NUPL1

I;FNDC3B;DEK;HCFC2;GATAD2B;HNRNPK;SP1;SUB1;HNRNPC;TARDBP;UBE2K

;ZNF148;FNBP4;CRBN;XRCC5;TFEB;ACTN4;DEK;SMC1A;SMARCA1;HNRNPK;FUBP1;PGAM5;HNRNP
E2K;KPNA1
XRCC5;TMOD3;YLPM1;ACTN4;GAPVD1;SMC1A;HNRNPK;MYO1C;CAPZA1;CAPRIN1;PGAM5;TRIP12;H
G3BP1;CEP170;ZNF687;NUPL1;MYH10

CAPRIN1;RUVBL1;CEP170;SNRPD3;HNRNPC;TARDBP;NUPL1;KPNA1

D3;HNRNPC;TARDBP

BL1;CEP170;SNRPD3;BSN;TARDBP;UBE2K

L1;KPNA1

1

H14;ARHGEF5

;MAT2A;SP1;SIN3A;UBN2;RUVBL1;CEP170;SNRPD3;TARDBP

1;RBBP5;FUBP1;CAPZA1;ZNF318;STRN;HNRNPC;TARDBP

;TMOD3;ACTN4;CDC42BPA;HNRNPK;MYO1C;SP1;TBL1XR1;CAPZA1;CAPRIN1;WDFY3;HNRNPC;TARD

C40;GAPVD1;SMC1A;GATAD2B;PIAS1;TTC9;DDX19B;HNRNPK;SP1;RAB3GAP2;ANKRD40;TARDBP;UE
1;XRCC5;SMAD9;ACTN4;GAPVD1;SMC1A;SMARCA1;GATAD2B;LETM1;HNRNPK;MYO1C;FUBP1;CAPF
;F2BP1;TRIP12;CEP170;HNRNPC;TARDBP;MYH10;KPNA1
AM5;CEP170;ZNF148;TARDBP;KPNA1

3L1;G3BP1;PDCD4;UBXN7;RAB3GAP2;SNX8;UBE2K;KPNA1
;TARDBP;SMNDC1

)EK;SMARCA1;HNRNPK;SUB1;CAPZA1;SERBP1;TERF2IP;TRIP12;HNRNPC;TARDBP

'PM3;GFPT1;SMAD9;ACTN4;SMC1A;RAB11A;ACAP2;HNRNPK;FUBP1;CAPZA1;TERF2IP;HNRNPC

IVBL1;TARDBP;KPNA1
BBP5;PGAM5;HNRNPC;TARDBP

;HNRNPK;CDH2;FUBP1;RUVBL1;CCDC6;TMEM108;TARDBP;SOS2;KPNA1
5;FUBP1;RUVBL1;UBXN7;CEP170;TARDBP;KPNA1

B;CAPZA1;RUVBL1;STRN;TARDBP

;CEP170;SNRPD3;TARDBP;KCTD16

;ZFP91;ACTN4;SMC1A;ENAH;MARCKS;HNRNPK;RFWD3;CAPRIN1;SERBP1;HNRNPC;TARDBP

P1;RUVBL1;EPC1;HNRNPC;TARDBP;KPNA1

PNA1

;CEP170;TARDBP;DTL

PK;KIF5C;FUBP1;RUVBL1;TARDBP

CYT1B;CPSF7;CRBN;YLPM1;ZFP91;SMC1A;SMARCA1;DYNLL2;GATAD2B;HNRNPK;SP1;CAPRIN1;SEI

D3;HNRNPC;TARDBP;ZNF687;MXD1
1;RUVBL1;G3BP1;TARDBP

P9X;TPM3;XRCC5;TMOD3;GTF2H1;ACTN4;SMARCA1;GATAD2B;HNRNPK;MYO1C;CAPRIN1;HNRNPC;
RNPK;SP1;SIN3A;RBBP5;RUVBL1;CEP170;SNRPD3;TARDBP

TARDBP

;CPSF7;XRCC5;NEK7;LRRC40;GAPVD1;SMC1A;DYNLL2;GATAD2B;DDX19B;HNRNPK;RRM2B;FUBP1;

P12;SNRPD3;HNRNPC;TARDBP

KPNA1

UVBL1;SNRPD3;HNRNPC;TARDBP;CGGBP1
BL1;G3BP1;PDCD4;UBXN7;RAB3GAP2;SH3GL2;APPL1;KPNA1

P170;APPL1;KPNA1

;SART3;SP1;CAPRIN1;SERBP1;G3BP1;TARDBP

NPK;TBL1XR1;CAPRIN1;SERBP1;RUVBL1;G3BP1;TARDBP;UBE2K
1

70;SNRPD3;RBM7;CPSF7;CSNK1A1;RC3H1;DEK;SMC1A;SMARCA1;DYNLL2;HNRNPK;SP1;SUB1;SERE
C
PXD2A;RUVBL1;CEP170;HNRNPC;TARDBP;MARK1
NRNPC;ZNF148;TARDBP;RBM7

C;ZNF687;KPNA1

70;TARDBP
2K

BM12;TARDBP;UBE2K;CGGBP1;KPNA1

TARDBP
;RXRA;TBL1XR1;SP1;RUVBL1;HNRNPC;TARDBP;KPNA1

F687

NPK;SIN3A;RUVBL1;TARDBP;UBE2K;KPNA1

L1;CEP170;TARDBP;SNX6;KPNA1
;DTL;KPNA1

T10;XRCC5;SMC1A;SMARCA1;GATAD2B;ACAP2;HNRNPK;FCHSD2;CAPZA1;HNRNPC;TARDBP

KPNA1
1;LRRC40;GAPVD1;SMC1A;GNL1;GTPBP4;DDX19B;TBL1XR1;PGAM5;PSMD11;SF3B3;DCTN2;DLST;M

PD3;TARDBP
EP170;HNRNPC;ZNF148;TARDBP;KPNA1

K

R1;RBBP5;RUVBL1;TRIP12;TARDBP;MYH10;UBE2K

C6;HNRNPC;TARDBP

L1;BSN;TARDBP
KR1;SP1;SIN3A;RBBP5;RUVBL1;ZNF148;TARDBP;UBE2K;KPNA1
SNRPD3;HNRNPC;TARDBP

CAPZA1;SERBP1;RUVBL1;TARDBP;NUPL1;UBE2K;PAFAH1B2;KPNA1

)BP

CC5;LRRC40;SMC1A;GATAD2B;PIAS1;HNRNPK;SP1;ZNF318;CALM1;TARDBP

ARDBP

VBL1;G3BP1;ZNF148;TARDBP;ZNF687;TEAD1;KPNA1

3;ACTN4;SMC1A;TTC9;HNRNPK;TBL1XR1;CCDC6;STRN;TARDBP;UBE2K
5;TARDBP

TN4;SMC1A;SMARCA1;GATAD2B;HNRNPK;TBL1XR1;FUBP1;CAPZA1;CARM1;SERBP1;EIF3J;TERF2IF
VBL1;SNRPD3;TARDBP;ZNF687

I4;SMC1A;SMARCA1;GTPBP4;GATAD2B;PUM2;HNRNPK;FUBP1;PGAM5;TARDBP
P1;CAPRIN1;RUVBL1;CEP170;HNRNPC;TARDBP
PYSL3;RUVBL1;G3BP1;TFAM;TARDBP
1;CAPZA1;RUVBL1;ZNF318;CEP170;HNRNPC;TARDBP;RAI2

1;LRRC40;GAPVD1;SMARCA1;GTPBP4;GATAD2B;HNRNPK;FUBP1;PGAM5;TRIP12;HNRNPC;TARDBP;

HNRNPC;TARDBP
.CC5;LRRC40;ACTN4;DEK;SMC1A;GATAD2B;HNRNPK;MYO1C;FUBP1;HNRNPC;TARDBP
.PM1;SMC1A;SMARCA1;GTPBP4;GATAD2B;HNRNPK;VAPA;APC;CAPZA1;HNRNPC;TARDBP
PD3;TARDBP;KPNA1

CEP170;TARDBP;MYH10;UBE2K;KPNA1

SORD;ACTN4;GAPVD1;SMARCA1;DYNLL2;HNRNPK;MYO1C;SUB1;TRIP12;HNRNPC;TARDBP

1;RUVBL1;CEP170;ZNF148;TARDBP;UBE2K
'1;ZNF148;TARDBP;ZNF687
FUBP1;CAPRIN1;RUVBL1;NUPL1

NRPD3;TARDBP;ZNF687

3P5;FUBP1;RUVBL1;TARDBP;NUPL1

170;HNRNPC;EMB;ZNF148;TARDBP

TARDBP;WDFY4
PC;TARDBP

\1;HNRNPC;TARDBP
BP;KPNA1

SF7;LRRC40;ACTN4;SMC1A;DYNLL2;GATAD2B;HNRNPK;FUBP1;CAPZA1;SERBP1;HNRNPC;TARDBP

?2K1;CSNK1A1;LRRC40;GAPVD1;SMC1A;GATAD2B;PUM2;PTP4A1;HNRNPK;SP1;TBL1XR1;TRIP12;HN
1;PDCD4;CEP170;TARDBP;SNX8;APPL1;KPNA1

IVBL1;HNRNPC;TARDBP

I;CSNK1A1;XRCC5;ACTN4;DEK;GAPVD1;SMC1A;DYNLL2;GATAD2B;HNRNPK;TBL1XR1;CAPZA1;CAPI

ARDBP;UBE2K

2;TARDBP;ZNF687;RAF1

4;UBXN7;RAB3GAP2;CEP170;TARDBP;UBE2K;KPNA1

;TARDBP;UBE2K

P1;KPNA1;FNBP4;CREBBP;CPSF7;USP9X;XRCC5;TTC33;ACTN4;DEK;SMC1A;DYNLL2;GATAD2B;HNRN

PK;SP1;CAPRIN1;RUVBL1;TRIP12;HNRNPC;SNRPD3;ZNF148;TARDBP
71B;ZNF148;TARDBP;UBE2K;KPNA1

RDBP;SMNDC1
;SP1;SIN3A;RUVBL1;CEP170;ZNF148;TARDBP;KPNA1

3P2

P;RAD9A

NF687

;TPM3;PDAP1;DEK;GAPVD1;SMC1A;GATAD2B;PTP4A1;HNRNPK;TBL1XR1;FUBP1;CAPZA1;PGAM5;TF

PZA1;RUVBL1;EIF3J;SNRPD3;HNRNPC;TARDBP;KPNA1

JVBL1;CCDC6;TERF2IP;HNRNPC

ZNF148;TARDBP;ZNF687

[illegible]

RNPC;TARDBP

170;HNRNPC;ZNF687;KPNA1

NPC;TARDBP;KPNA1

TARDBP;ZC3H14;KPNA1

ARDBP

3;TARDBP

C;CAPZB;C1QBP;TRA2B;RUVBL1;ZNF318;TRIP12;HNRNPC;ZNF148

TRIP12;SNRPD3;HNRNPC;TARDBP

ZNF148;TARDBP;ZNF687;KPNA1
;SNRPD3;HNRNPC;ZNF148;TARDBP;NUPL1
PD3;ZNF148;TARDBP
'1;HNRNPK;FUBP1;RUVBL1;EIF3J;PGAM5;HNRNPC;TARDBP

BP;UBE2K;SMNDC1;KPNA1

RDBP;ZNF687

18;UBXN7;TERF2IP;TARDBP;RAF1;UBE2K;KPNA1

'1;CAPZA1;RUVBL1;TARDBP
C3H14;UBE2K;KPNA1

F148;TARDBP;KPNA1

l;RUVBL1;SNRPD3;ZNF148;TARDBP;KPNA1

\;SUB1;FUBP1;CEP170;SNRPD3;TARDBP

X;XRCC5;LRRC40;KCNRG;SMC1A;SMARCA1;GATAD2B;HNRNPK;SNPH;TARDBP;CGGBP1
TRN;HNRNPC;ZNF148;TARDBP
G;NOS1AP;TRIM24;CEP170;HNRNPC;ZNF148;TARDBP;KPNA1

C;ZNF148;TARDBP;KPNA1;TNRC6B
3P;GPRASP2

FG2

NRNPK;SP1;KIF5C;RUVBL1;CEP170;ZNF148;TARDBP;KPNA1

NRPD3;HNRNPC;MAPRE2

[illegible]

IT1;HSPH1;HNRNPK;SUB1;RUVBL1;TRIP12;HNRNPC;TARDBP
UBP1;RUVBL1;HNRNPC;EMB;TARDBP;SNX8;UBE2K

BL1;NOS1AP;CEP170;ZNF148;TARDBP;ZC3H14

BP;UBE2K;KPNA1

;ATAD2B;RAB11A;PIAS1;DDX19B;HNRNPK;SP1;CARM1;PDCD4;RAB3GAP2;TARDBP;UBE2K

N2;FUBP1;RUVBL1;G3BP1;SNRPD3;ZNF687;TARDBP;KPNA1
C;TARDBP

H10;UBE2K;APPL1;KPNA1

;HNRNPC;TARDBP;UBE2K;KPNA1
C;TRA2B;RUVBL1;HNRNPC;SLIT2;ZNF148

;HNRNPK;FUBP1;CAPRIN1;RUVBL1;TERF2IP;CEP170;HNRNPC;TARDBP

2K

)1;SMC1A;GATAD2B;GTF2H5;HNRNPK;SP1;TBL1XR1;FUBP1;SERBP1;TARDBP;UBE2K

APB;C1QBP;TRA2B;RUVBL1;G3BP1;PGAM5;HNRNPC;TARDBP

NUPL1

;SIN3A;CAPRIN1;RUVBL1;TERF2IP;TARDBP;MYH10

RNPC;TARDBP;ZNF687;ZC3H14
NF148;KPNA1;CRBN;TPM3;ACTN4;SMC1A;SMARCA1;GATAD2B;RAB11A;HNRNPK;SP1;CAPRIN1;HNRN

ECPR2;KPNA1

|
.RDBP

/BL1;RAB3GAP2;CEP170;TARDBP;MAPRE2;UBE2K;KPNA1
.L1;G3BP1;CEP170;ZNF148;TARDBP;SMNDC1

IT1;HNRNPK;CAPZB;RUVBL1;TERF2IP;HNRNPC;ZNF148;TARDBP;SOS2

\1

RDBP

A1;TPM3;GAPVD1;HNRNPK;ZZZ3;TBL1XR1;SUB1;TARDBP;SSBP2;UBE2K

HNRNPC;TARDBP;UBE2K

PD3;HNRNPC;TARDBP;SNX8;UBE2K;KPNA1
ERF2IP;HNRNPC;TARDBP;ZNF687;UBE2K

1;RUVBL1;STRN;CEP170;SNX8;KPNA1

37;NUPL1

APZA1;RUVBL1;PGAM5;CEP170;HNRNPC;TARDBP;UBE2K;KPNA1

3;TARDBP;ZNF687;KPNA1

BP

RPD3;TARDBP;ZNF687
DBP

3P1;RUVBL1;HNRNPC;TARDBP;PELO
;SOS2;KPNA1

/IYH10;KPNA1

3P;ZNF687;KPNA1

P1;CEP170;TRIP12;HNRNPC;TARDBP

\RDBP

.RDBP

.7;CAPRIN1;ZNF318;TARDBP

;P1;SNRPD3;TARDBP;ZNF687

;TARDBP;UBE2K

;RGAP2

RBP1;RUVBL1;G3BP1;RAB3GAP2;CEP170;TARDBP;KPNA1

D3;HNRNPC;TARDBP;KPNA1

N4;SMC1A;DYNLL2;HNRNPK;MYO1C;SUB1;SERBP1;PDCD4;CTNNB1;HNRNPC

PNA1
NF687;MAPRE2

IN3A;FUBP1;FBXO3;HNRNPC;TARDBP

C;TARDBP

;G3BP1;HNRNPC;SNRPD3;ZNF148;TARDBP;RAD9A;METAP2

IP12;HNRNPC;TARDBP
BP1;TARDBP;ZNF687

2

DBP;KPNA1

RIM24;CEP170;HNRNPC

A1

1;TRIP12;HNRNPC;TARDBP

ARDBP;UBE2K;KPNA1

;TARDBP;RAPGEF6;KPNA1

P170;TARDBP

P;MYH10;UBE2K

)3;HNRNPC;TARDBP

)3;TARDBP;ZNF687

3L1;TARDBP;KPNA1

A1

R1;SP1;FUBP1;ZNF318;TARDBP

RIN1;SERBP1;HNRNPC;TARDBP;WDFY4
MARCA1;GATAD2B;MYO1C;SP1;RFWD3;CAPRIN1;SERBP1;TARDBP

;TARDBP

OD2;HNRNPK;MYO1C;FCHSD2;SUB1;FUBP1;CARM1;SERBP1;HNRNPC;TARDBP

CGGBP1

\1;HNRNPC;TARDBP

\RDBP

TERF2IP;HNRNPC;TARDBP

P4;HNRNPK;SP1;FUBP1;CAPZA1;CAPRIN1;SERBP1;ZNF318;PGAM5;HNRNPC;TARDBP

BP1;CAPZA1;CAPRIN1;CCDC6;TARDBP

C;TARDBP

INRNPC;TARDBP;TNRC6B

)BP

3E2K
RIN1;HNRNPC;TARDBP;MAPRE2;TNRC6B

RBP1;HNRNPC;TARDBP

TARDBP

TARDBP;NUPL1;UBE2K

3P1;ZNF318;STRN;PGAM5;TRIP12;WDFY3;HNRNPC;TARDBP;CGGBP1

TDH;NPAT;SNX1;HSPH1;SNX6;RRM1;XRCC5;ST13;NEK7;HOOK3;DEK;SMARCA1;GATAD2B;DIAPH2;HI

P;HNRNPC;TARDBP

;ARHGEF5

\RNPC;TARDBP;NUPL1;UBE2K;ARHGEF5

RIN1;SERBP1;PGAM5;TRIP12;HNRNPC;TARDBP;SSBP2

NPK;TBL1XR1;FUBP1;CARM1;TRIP12;HNRNPC;TARDBP

RIP12;HNRNPC;TARDBP;UBE2K

ΛPC;TARDBP

NRNPK;FUBP1;CAPZA1;CAPRIN1;PDCD4;RAB3GAP2;TRIP12;HNRNPC;TARDBP;UBE2K
