# Supplementary material for: Circulating miR-330-3p in Late Pregnancy is Associated with Pregnancy Outcomes Among Lean Women with GDM
Source: Sci Rep. 2020 Jan 22;10:908. doi: 10.1038/s41598-020-57838-6 (PMC6976655; doi:10.1038/s41598-020-57838-6)
Supplement: Supplementary file 1 — Supplementary data. [file 41598_2020_57838_MOESM1_ESM.zip › Supplimentary File_EnrichR_Analysis Pfam_Domains_2019.pdf]

Term	Overlap	P.value	Adjusted.P.value
Cadherin_2	17/65	2,04148E-08	1,24122E-05
Cadherin	19/114	5,6382E-06	0,001714014
RRM_1	24/206	0,000167329	0,033911946
zf-RING_2	9/52	0,001241762	0,18874776
Sortilin-Vps10	3/5	0,001266489	0,154005045
Sortilin_C	3/5	0,001266489	0,128337537
WW	7/38	0,002954873	0,256651841
Glyco_hydro_47	3/7	0,004099378	0,311552727
PID	5/26	0,009632009	0,650695713
ZZ	4/17	0,009778786	0,594550177
BTB	13/129	0,01592693	0,880324854
PDEase_I	4/21	0,02088453	1
MH1	3/12	0,021233575	0,993077964
Vps5	2/5	0,02396506	1
DTC	2/5	0,02396506	0,971383749
Motile_Sperm	2/5	0,02396506	0,910672264
Septin	3/13	0,026564546	0,950073181
GATA	3/14	0,03254043	1
LCCL	2/6	0,034729741	1
PH_BEACH	2/6	0,034729741	1
SH3_1	10/101	0,035575323	1
Sec7	3/15	0,039153343	1
RAWUL	2/7	0,04698191	1
Ras	12/136	0,048794168	1
HECT	4/28	0,053862291	1
Pkinase	25/347	0,058478052	1
Yippee-Mis18	2/8	0,060539704	1
ERAP1_C	2/8	0,060539704	1
Ephrin	2/8	0,060539704	1
Cation_ATPase_N	3/18	0,062669827	1
ig	7/70	0,068652616	1
LSM	3/19	0,071669081	1
Activin_recp	2/9	0,07523604	1
GBP_C	2/9	0,07523604	1
NTF2	2/9	0,07523604	1
PAX	2/9	0,07523604	1
TIG	3/20	0,081209097	1
ThiF	2/10	0,090917569	1
Amidohydro_1	2/10	0,090917569	1
IL6Ra-bind	2/10	0,090917569	1
NUDIX	3/22	0,101803296	1
KH_1	4/35	0,104307076	1
WH1	2/11	0,107443713	1
GBP	2/11	0,107443713	1
Fz	3/23	0,112800229	1
PWWP	3/23	0,112800229	1
CRAL_TRIO_2	2/12	0,124685754	1
His_Phos_1	2/12	0,124685754	1
Pentaxin	2/12	0,124685754	1
Reprolysin	4/38	0,130718162	1
SAM_2	4/38	0,130718162	1
UBA	3/25	0,136044545	1

EF-hand_1	2/13	0,142525984	1
Med26	2/13	0,142525984	1
Peptidase_M1	2/13	0,142525984	1
Chromo	3/26	0,148230982	1
UQ_con	4/40	0,149700503	1
Myotub-related	2/14	0,160856918	1
ADH_N	2/15	0,179580553	1
CAP	2/15	0,179580553	1
Clat_adaptor_s	2/15	0,179580553	1
Cyt-b5	2/15	0,179580553	1
OAR	2/15	0,179580553	1
C2	9/123	0,184859436	1
ArfGap	3/29	0,18668155	1
FYVE	3/29	0,18668155	1
F-box-like	4/44	0,190485643	1
SH3_9	6/76	0,197939526	1
Arf	3/31	0,213592618	1
FKBP_C	2/17	0,217857207	1
T-box	2/17	0,217857207	1
FERM_M	4/47	0,2230987	1
cNMP_binding	3/32	0,227344696	1
Homeodomain	14/217	0,231654002	1
Tap-RNA_bind	1/5	0,232733649	1
AA_permease_C	1/5	0,232733649	1
Transketolase_C	1/5	0,232733649	1
AWS	1/5	0,232733649	1
YTH	1/5	0,232733649	1
CIDE-N	1/5	0,232733649	1
zf-RING_9	1/5	0,232733649	1
DAN	1/5	0,232733649	1
DMAP_binding	1/5	0,232733649	1
DOMON	1/5	0,232733649	1
EpoR_lig-bind	1/5	0,232733649	1
FAD_binding_6	1/5	0,232733649	1
Hexokinase_1	1/5	0,232733649	1
Hexokinase_2	1/5	0,232733649	1
ING	1/5	0,232733649	1
KA1	1/5	0,232733649	1
Neuralized	1/5	0,232733649	1
PGM_PMM_I	1/5	0,232733649	1
Pribosyltran	1/5	0,232733649	1
SLED	1/5	0,232733649	1
SRF-TF	1/5	0,232733649	1
Lig_chan-Glu_bd	2/18	0,237255632	1
MAM	2/18	0,237255632	1
Sulfotransfer_1	3/33	0,241257655	1
CH	5/66	0,253638584	1
PAS	2/19	0,256736405	1
PX	4/50	0,257018171	1
PDZ	8/117	0,257123198	1
SH2	7/100	0,257790801	1
EF-hand_7	6/84	0,265783387	1
RA	3/35	0,269461014	1

ArgoL1	1/6	0,272334438	1
BRO1	1/6	0,272334438	1
zf-MIZ	1/6	0,272334438	1
Cullin_Nedd8	1/6	0,272334438	1
DUSP	1/6	0,272334438	1
E2F_CC-MB	1/6	0,272334438	1
EF-hand_2	1/6	0,272334438	1
EF-hand_3	1/6	0,272334438	1
EFhand_Ca_insen	1/6	0,272334438	1
GF_recep_IV	1/6	0,272334438	1
Occludin_ELL	1/6	0,272334438	1
PET	1/6	0,272334438	1
PH_12	1/6	0,272334438	1
RBD	1/6	0,272334438	1
SMC_N	1/6	0,272334438	1
SMC_hinge	1/6	0,272334438	1
TFIIS_C	1/6	0,272334438	1
Disintegrin	2/20	0,276239439	1
RasGEF_N	2/20	0,276239439	1
HLH	7/106	0,306674504	1
ACBP	1/7	0,309893194	1
Abhydrolase_3	1/7	0,309893194	1
zf-Di19	1/7	0,309893194	1
CTP_transf_like	1/7	0,309893194	1
CUT	1/7	0,309893194	1
Cadherin_pro	1/7	0,309893194	1
Furin-like	1/7	0,309893194	1
GAF	1/7	0,309893194	1
Glyco_hydro_18	1/7	0,309893194	1
PKD	1/7	0,309893194	1
Peptidase_C48	1/7	0,309893194	1
Recep_L_domain	1/7	0,309893194	1
SIX1_SD	1/7	0,309893194	1
TFIIS_M	1/7	0,309893194	1
Y_phosphatase	3/38	0,312340461	1
Bromodomain	3/38	0,312340461	1
Abhydrolase_1	2/22	0,315101353	1
Spectrin	2/22	0,315101353	1
F5_F8_type_C	2/23	0,334368124	1
Transket_pyr	1/8	0,345515118	1
Trefoil	1/8	0,345515118	1
BIR	1/8	0,345515118	1
FA_desaturase	1/8	0,345515118	1
Glyco_tran_10_N	1/8	0,345515118	1
LRAT	1/8	0,345515118	1
LisH	1/8	0,345515118	1
Exo_endo_phos	2/24	0,353472123	1
Pro_isomerase	2/24	0,353472123	1
BACK	4/60	0,374805161	1
Transglut_C	1/9	0,379299997	1
Transglut_N	1/9	0,379299997	1
Band_3_cyto	1/9	0,379299997	1
Biotin_lipoyl	1/9	0,379299997	1

Calx-beta	1/9	0,379299997	1
Macro	1/9	0,379299997	1
PAZ	1/9	0,379299997	1
Peptidase_M24	1/9	0,379299997	1
FERM_C	2/26	0,39105785	1
adh_short	3/44	0,398037404	1
SAM_1	4/62	0,398494592	1
cEGF	2/27	0,409482246	1
zf-C3HC4	2/27	0,409482246	1
Myb_DNA-binding	2/27	0,409482246	1
BRICHOS	1/10	0,411342481	1
Filamin	1/10	0,411342481	1
HMG_box_2	1/10	0,411342481	1
Laminin_G_1	1/10	0,411342481	1
MBT	1/10	0,411342481	1
PAS_9	1/10	0,411342481	1
RHD_DNA_bind	1/10	0,411342481	1
RHD_dimer	1/10	0,411342481	1
Kinesin	3/45	0,412098092	1
zf-C4	3/46	0,426053988	1
PH	10/177	0,430622936	1
E2F_TDP	1/11	0,441732347	1
Hairy_orange	1/11	0,441732347	1
Hist_deacetyl	1/11	0,441732347	1
R3H	1/11	0,441732347	1
RINGv	1/11	0,441732347	1
zf-C2H2	36/675	0,443177091	1
FERM_N	2/29	0,445476512	1
Hormone_recep	3/48	0,453599363	1
ABC_tran	3/49	0,467165176	1
Forkhead	3/49	0,467165176	1
zf-B_box	4/68	0,468332155	1
dDENN	1/12	0,470554753	1
CS	1/12	0,470554753	1
IRS	1/12	0,470554753	1
NAD_binding_1	1/12	0,470554753	1
BTB_2	3/50	0,480578809	1
Pkinase_Tyr	7/128	0,49269163	1
Cyclin_N	2/32	0,497067219	1
UBX	1/13	0,497890471	1
fn2	1/13	0,497890471	1
Globin	1/13	0,497890471	1
HTH_Tnp_Tc5	1/13	0,497890471	1
IBR	1/13	0,497890471	1
PAS_3	1/13	0,497890471	1
PHD	3/52	0,50691194	1
TUDOR	1/14	0,523816115	1
BAR	1/14	0,523816115	1
zf-UBP	1/14	0,523816115	1
Myosin_N	1/14	0,523816115	1
PB1	1/14	0,523816115	1
ARID	1/15	0,548404355	1
uDENN	1/15	0,548404355	1

RnaseA	1/15	0,548404355	1
TSP_1	3/56	0,557383521	1
TRAM_LAG1_CLN8	1/16	0,571724118	1
dsrm	1/16	0,571724118	1
Cys_knot	1/16	0,571724118	1
ECH_1	1/16	0,571724118	1
G-alpha	1/16	0,571724118	1
Pou	1/16	0,571724118	1
TGF_beta	2/37	0,57590642	1
zf-RING_UBOX	2/38	0,590548963	1
Aminotran_1_2	1/17	0,593840785	1
Cyclin_C	1/17	0,593840785	1
OB_NTP_bind	1/17	0,593840785	1
PCI	1/17	0,593840785	1
EF-hand_8	2/39	0,604811304	1
Myosin_head	2/39	0,604811304	1
CBS	1/18	0,614816365	1
Carb_anhydrase	1/18	0,614816365	1
GRAM	1/18	0,614816365	1
HA2	1/18	0,614816365	1
NTR	1/18	0,614816365	1
SRCR	1/18	0,614816365	1
zf-MYND	1/19	0,63470968	1
Guanylate_cyc	1/19	0,63470968	1
IGFBP	1/19	0,63470968	1
ubiquitin	2/42	0,645324095	1
DSPc	2/42	0,645324095	1
PG_binding_1	1/20	0,653576518	1
RhoGAP	3/66	0,668943677	1
EF-hand_5	1/21	0,671469795	1
PLAT	1/21	0,671469795	1
EGF	3/67	0,678884566	1
JmjC	1/22	0,688439703	1
LIM	3/69	0,698097744	1
fn3	6/135	0,702503663	1
Tubulin	1/23	0,704533848	1
Peptidase_M10	1/23	0,704533848	1
Ricin_B_lectin	1/23	0,704533848	1
VWA	2/47	0,705431946	1
RhoGEF	3/70	0,707371709	1
CUB	2/48	0,716381176	1
Rhodanese	1/24	0,719797384	1
C1_1	2/49	0,7269861	1
MARVEL	1/25	0,734273137	1
HMG_box	2/50	0,737253058	1
Sushi	2/51	0,747188653	1
EGF_CA	3/75	0,750473779	1
SH3_2	2/52	0,756799709	1
hEGF	1/28	0,773369566	1
C2-set_2	1/28	0,773369566	1
zf-CCHC	1/28	0,773369566	1
Ets	1/28	0,773369566	1
Metallophos	1/28	0,773369566	1

S_100	1/28	0,773369566	1
Thioredoxin	1/29	0,785080013	1
I-set	5/134	0,827220937	1
Laminin_G_2	1/34	0,835163993	1
SOCS_box	1/36	0,851763893	1
DEAD	2/67	0,8669476	1
Lectin_C	2/85	0,938092244	1
Histone	1/66	0,969874831	1
Ig_2	1/75	0,981330738	1
Trypsin	1/121	0,9983853	1
V-set	7/380	0,99975989	1

Old.P.value	Old.Adjusted	Odds.Ratio	Combined.Score
0	0	5,068574836	89,74928654
0	0	3,22997416	39,03729013
0	0	2,257846015	19,6332141
0	0	3,354203936	22,44373082
0	0	11,62790698	77,57566148
0	0	11,62790698	77,57566148
0	0	3,56997144	20,79258306
0	0	8,305647841	45,65548196
0	0	3,726893262	17,30271119
0	0	4,55996352	21,10141337
0	0	1,953007632	8,084951424
0	0	3,69139904	14,28108748
0	0	4,84496124	18,66362223
0	0	7,751937984	28,92370822
0	0	7,751937984	28,92370822
0	0	7,751937984	28,92370822
0	0	4,472271914	16,22619769
0	0	4,15282392	14,22455135
0	0	6,45994832	21,70645267
0	0	6,45994832	21,70645267
0	0	1,918796531	6,40130298
0	0	3,875968992	12,55918396
0	0	5,53709856	16,93240671
0	0	1,70998632	5,164405756
0	0	2,76854928	8,087831292
0	0	1,396242432	3,964077155
0	0	4,84496124	13,58747994
0	0	4,84496124	13,58747994
0	0	4,84496124	13,58747994
0	0	3,22997416	8,946625228
0	0	1,937984496	5,191271411
0	0	3,05997552	8,065164774
0	0	4,306632214	11,14179549
0	0	4,306632214	11,14179549
0	0	4,306632214	11,14179549
0	0	4,306632214	11,14179549
0	0	2,906976744	7,298627913
0	0	3,875968992	9,293806274
0	0	3,875968992	9,293806274
0	0	3,875968992	9,293806274
0	0	2,642706131	6,037824525
0	0	2,214839424	5,006458647
0	0	3,523608175	7,860423421
0	0	3,523608175	7,860423421
0	0	2,527805865	5,516018479
0	0	2,527805865	5,516018479
0	0	3,22997416	6,724672735
0	0	3,22997416	6,724672735
0	0	3,22997416	6,724672735
0	0	2,03998368	4,150778681
0	0	2,03998368	4,150778681
0	0	2,325581395	4,639006775

0	0	2,981514609	5,80867905
0	0	2,981514609	5,80867905
0	0	2,981514609	5,80867905
0	0	2,236135957	4,268746712
0	0	1,937984496	3,68046246
0	0	2,76854928	5,058804027
0	0	2,583979328	4,437032068
0	0	2,583979328	4,437032068
0	0	2,583979328	4,437032068
0	0	2,583979328	4,437032068
0	0	2,583979328	4,437032068
0	0	1,418037436	2,393873437
0	0	2,004811548	3,364777581
0	0	2,004811548	3,364777581
0	0	1,761804087	2,921385574
0	0	1,52998776	2,478264567
0	0	1,875468867	2,895132655
0	0	2,27998176	3,474499418
0	0	2,27998176	3,474499418
0	0	1,649348507	2,474255328
0	0	1,816860465	2,691293478
0	0	1,250312578	1,828595135
0	0	3,875968992	5,650622543
0	0	3,875968992	5,650622543
0	0	3,875968992	5,650622543
0	0	3,875968992	5,650622543
0	0	3,875968992	5,650622543
0	0	3,875968992	5,650622543
0	0	3,875968992	5,650622543
0	0	3,875968992	5,650622543
0	0	3,875968992	5,650622543
0	0	3,875968992	5,650622543
0	0	3,875968992	5,650622543
0	0	3,875968992	5,650622543
0	0	3,875968992	5,650622543
0	0	3,875968992	5,650622543
0	0	3,875968992	5,650622543
0	0	3,875968992	5,650622543
0	0	3,875968992	5,650622543
0	0	3,875968992	5,650622543
0	0	3,875968992	5,650622543
0	0	3,875968992	5,650622543
0	0	3,875968992	5,650622543
0	0	2,153316107	3,097797377
0	0	2,153316107	3,097797377
0	0	1,761804087	2,505091275
0	0	1,468170073	2,014101658
0	0	2,03998368	2,773776792
0	0	1,550387597	2,106369756
0	0	1,325117604	1,79977465
0	0	1,356589147	1,839001571
0	0	1,38427464	1,834265832
0	0	1,661129568	2,178291625

0	0	3,22997416	4,201306255
0	0	3,22997416	4,201306255
0	0	3,22997416	4,201306255
0	0	3,22997416	4,201306255
0	0	3,22997416	4,201306255
0	0	3,22997416	4,201306255
0	0	3,22997416	4,201306255
0	0	3,22997416	4,201306255
0	0	3,22997416	4,201306255
0	0	3,22997416	4,201306255
0	0	3,22997416	4,201306255
0	0	3,22997416	4,201306255
0	0	3,22997416	4,201306255
0	0	3,22997416	4,201306255
0	0	3,22997416	4,201306255
0	0	3,22997416	4,201306255
0	0	3,22997416	4,201306255
0	0	1,937984496	2,493192356
0	0	1,937984496	2,493192356
0	0	1,279801082	1,512684362
0	0	2,76854928	3,243431827
0	0	2,76854928	3,243431827
0	0	2,76854928	3,243431827
0	0	2,76854928	3,243431827
0	0	2,76854928	3,243431827
0	0	2,76854928	3,243431827
0	0	2,76854928	3,243431827
0	0	2,76854928	3,243431827
0	0	2,76854928	3,243431827
0	0	2,76854928	3,243431827
0	0	2,76854928	3,243431827
0	0	2,76854928	3,243431827
0	0	2,76854928	3,243431827
0	0	2,76854928	3,243431827
0	0	1,52998776	1,780387798
0	0	1,52998776	1,780387798
0	0	1,761804087	2,034638719
0	0	1,761804087	2,034638719
0	0	1,68520391	1,846162327
0	0	2,42248062	2,574415889
0	0	2,42248062	2,574415889
0	0	2,42248062	2,574415889
0	0	2,42248062	2,574415889
0	0	2,42248062	2,574415889
0	0	2,42248062	2,574415889
0	0	2,42248062	2,574415889
0	0	1,61498708	1,679506874
0	0	1,61498708	1,679506874
0	0	1,291989664	1,26789271
0	0	2,153316107	2,087484578
0	0	2,153316107	2,087484578
0	0	2,153316107	2,087484578
0	0	2,153316107	2,087484578

0	0	2,153316107	2,087484578
0	0	2,153316107	2,087484578
0	0	2,153316107	2,087484578
0	0	2,153316107	2,087484578
0	0	1,490757305	1,399671698
0	0	1,321353066	1,217242729
0	0	1,250312578	1,150364279
0	0	1,435544071	1,281742365
0	0	1,435544071	1,281742365
0	0	1,435544071	1,281742365
0	0	1,937984496	1,721568072
0	0	1,937984496	1,721568072
0	0	1,937984496	1,721568072
0	0	1,937984496	1,721568072
0	0	1,937984496	1,721568072
0	0	1,937984496	1,721568072
0	0	1,937984496	1,721568072
0	0	1,937984496	1,721568072
0	0	1,291989664	1,145340919
0	0	1,263902932	1,078348343
0	0	1,094906495	0,922483281
0	0	1,761804087	1,43948402
0	0	1,761804087	1,43948402
0	0	1,761804087	1,43948402
0	0	1,761804087	1,43948402
0	0	1,761804087	1,43948402
0	0	1,033591731	0,84112231
0	0	1,336541032	1,080741454
0	0	1,21124031	0,957535043
0	0	1,18652112	0,903028463
0	0	1,18652112	0,903028463
0	0	1,13999088	0,864771434
0	0	1,61498708	1,217446632
0	0	1,61498708	1,217446632
0	0	1,61498708	1,217446632
0	0	1,61498708	1,217446632
0	0	1,162790698	0,852051221
0	0	1,059835271	0,750227499
0	0	1,21124031	0,84669333
0	0	1,490757305	1,03961712
0	0	1,490757305	1,03961712
0	0	1,490757305	1,03961712
0	0	1,490757305	1,03961712
0	0	1,490757305	1,03961712
0	0	1,490757305	1,03961712
0	0	1,118067979	0,759635486
0	0	1,38427464	0,895092168
0	0	1,38427464	0,895092168
0	0	1,38427464	0,895092168
0	0	1,38427464	0,895092168
0	0	1,38427464	0,895092168
0	0	1,291989664	0,776152959
0	0	1,291989664	0,776152959

0	0	1,291989664	0,776152959
0	0	1,03820598	0,60683319
0	0	1,21124031	0,6772029
0	0	1,21124031	0,6772029
0	0	1,21124031	0,6772029
0	0	1,21124031	0,6772029
0	0	1,21124031	0,6772029
0	0	1,21124031	0,6772029
0	0	1,047559187	0,578053737
0	0	1,01999184	0,537232486
0	0	1,13999088	0,594099447
0	0	1,13999088	0,594099447
0	0	1,13999088	0,594099447
0	0	1,13999088	0,594099447
0	0	0,993838203	0,499740374
0	0	0,993838203	0,499740374
0	0	1,076658053	0,523720552
0	0	1,076658053	0,523720552
0	0	1,076658053	0,523720552
0	0	1,076658053	0,523720552
0	0	1,076658053	0,523720552
0	0	1,076658053	0,523720552
0	0	1,01999184	0,463675624
0	0	1,01999184	0,463675624
0	0	1,01999184	0,463675624
0	0	0,92284976	0,404210609
0	0	0,92284976	0,404210609
0	0	0,968992248	0,412108201
0	0	0,880902044	0,354171434
0	0	0,92284976	0,367558366
0	0	0,92284976	0,367558366
0	0	0,867754252	0,336084841
0	0	0,880902044	0,328864994
0	0	0,842601955	0,3028279
0	0	0,861326443	0,304138383
0	0	0,842601955	0,295095132
0	0	0,842601955	0,295095132
0	0	0,842601955	0,295095132
0	0	0,824674254	0,287765937
0	0	0,830564784	0,287540694
0	0	0,80749354	0,269333725
0	0	0,80749354	0,265492182
0	0	0,79101408	0,252213195
0	0	0,775193798	0,239437363
0	0	0,775193798	0,23629774
0	0	0,75999392	0,221490787
0	0	0,775193798	0,222519819
0	0	0,745378652	0,207704715
0	0	0,69213732	0,177878081
0	0	0,69213732	0,177878081
0	0	0,69213732	0,177878081
0	0	0,69213732	0,177878081
0	0	0,69213732	0,177878081

0	0	0,69213732	0,177878081
0	0	0,668270516	0,161701175
0	0	0,723128543	0,137165527
0	0	0,56999544	0,102671668
0	0	0,538329027	0,086372691
0	0	0,578502835	0,08259675
0	0	0,455996352	0,029141356
0	0	0,293634015	0,008981752
0	0	0,258397933	0,004869698
0	0	0,160164008	0,000258826
0	0	0,356997144	8,5729E-05

Genes

PCDH11Y;PCDHB15;PCDHA13;PCDHA12;PCDHA11;PCDHA10;PCDHA1;PCDHA5;PCDHA4;PCDHA3;PCDH
PCDH11Y;PCDHB15;PCDHA13;PCDHA12;PCDHA11;PCDHA10;CDH6;CDH2;PCDHA1;PCDHA5;PCDHA4;PC
RBM28;CPSF7;CELF1;CELF3;SRSF1;HNRNPR;ELAVL2;U2SURP;TIAL1;RBM3;SART3;PABPN1;TRA2B;RAV
RNF126;ZNRF2;RNF148;ZNRF3;RFWD3;LTN1;RLIM;RNF111;RNF165
SORT1;SORCS1;SORL1
SORT1;SORCS1;SORL1
DRP2;FNBP4;TCERG1;ITCH;SMURF2;HECW2;APBB2
EDEM3;MAN1A2;EDEM1
SHC4;SHC2;NOS1AP;APBB2;APPL1
KCMF1;DRP2;CREBBP;ZZZ3
ZBTB14;ZBTB16;KLHL32;RHOBTB3;ZBTB10;ZBTB20;ZBTB34;KLHL23;ZBTB44;BTBD9;BACH1;ZBTB4;KLHL
PDE1B;PDE3B;PDE5A;PDE7B
NFIA;SMAD9;SMAD7
SNX1;SNX6
DTX1;DTX4
VAPA;VAPB
SEPT10;SEPT11;SEPT12
TRPS1;GATA6;GATAD2B
CRISPLD1;CRISPLD2
NBEAL1;WDFY3
EPS8;NCKIPSD;LYN;DOCK5;FCHSD2;SH3PXD2A;SRGAP2;CRK;SORBS3;FRK
CYTH3;PSD4;PSD3
PCGF3;BMI1
RAP2C;RAB3C;RAB2B;RAP2A;RALA;RAP1A;RAP2B;DIRAS2;RHOBTB3;AGAP2;RND3;RAB11A
ITCH;SMURF2;HECW2;TRIP12
DYRK3;BMPR2;ROCK1;RPS6KA6;MAPK1;PIM3;MAP3K9;PRKG1;MARK1;SRPK2;MAP3K2;CSNK1G3;MAP2I
CRBN;YPEL2
LNPEP;TRHDE
EFNA3;EFNB3
ATP2B4;ATP2B2;ATP2B1
CXADR;IGLON5;IL1RAPL1;PTPRM;EMB;IL6R;FGFR1
NAA38;SNRPD3;LSM5
BMPR2;BMPR1A
ATL3;ATL2
NXF1;G3BP1
PAX5;PAX2
EBF1;PLXNA2;PLXNA3
MOCS3;UBA6
DPYSL5;DPYSL3
IL6R;IL13RA1
NUDT5;DCP2;NUDT4
TDRKH;HNRNPK;FUBP1;IGF2BP1
ENAH;SPRED1
ATL3;ATL2
FZD3;SFRP1;FZD5
NSD1;DNMT3A;GLYR1
SESTD1;PRUNE2
PFKFB3;PGAM5
CRP;NPTXR
ADAM19;ADAMTS5;ADAMTS2;ADAM12
TNKS2;TNKS;STIM2;PPP1R9A
USP13;UBE2K;MARK1

S100B;HPCAL4
TCEA3;TCEANC2
LNPEP;TRHDE
CBX6;CBX5;CDYL2
CDC34;UBE2G1;UBE2K;UBE2J1
MTMR3;MTMR9
ADH1B;SORD
CRISPLD1;CRISPLD2
AP3M2;AP2M1
PGRMC1;CYB5R4
PRRX1;SHOX2
SYT5;ITCH;RIMS3;SMURF2;HECW2;SYTL4;SYT7;RAB11FIP5;CC2D1B
ACAP2;AGAP2;AGFG2
MTMR3;WDFY3;WDFY2
FBXW4;FBXL17;FBXO3;BTRC
FCHSD2;EFS;SH3KBP1;MAP3K9;SORBS3;ARHGEF5
ARL5B;ARL3;ARL5A
AIPL1;FKBP6
EOMES;TBX5
FRMPD4;FRMPD2;PTK2B;PTPN4
RAPGEF6;PRKG1;KCNH1
DLX1;ONECUT2;PRRX1;DLX6;SHOX2;SIX1;HOXD12;POU3F1;PHOX2B;GBX2;HOXA3;LHX6;LHX4;HOXC8
NXF1
SLC7A14
PDHB
NSD1
YTHDC1
DFFA
PLEKHM3
DAND5
DIP2B
FRRS1L
EPOR
CYB5R4
HK2
HK2
ING4
MARK1
NEURL1B
PGM3
PRTFDC1
SFMBT2
MEF2A
GRID1;GRIN2B
NRP2;PTPRM
HS3ST3B1;CHST7;CHST3
MAPRE3;PARVA;ACTN4;SMTNL2;MAPRE2
NCOA2;HIF1A
SNX1;SH3PXD2A;SNX8;SNX6
FRMPD4;FRMPD2;CNKSR3;PPP1R9A;SYNJ2BP;PTPN4;RAPGEF6;LNX2
SHC4;LYN;SHC2;PTPN11;CRK;FRK;TNS1
KCNIP2;CHP1;CALML4;CALM1;HPCAL4;SLC25A24
ARHGAP20;RASSF8;RAPGEF6

AGO1
BROX
PIAS1
CUL3
USP15
E2F1
DRP2
DRP2
ACTN4
ERBB4
OCLN
PRICKLE1
ELMO1
RAF1
SMC1A
SMC1A
TCEA3
ADAM19;ADAM12
RAPGEF6;SOS2
HEYL;ID2;MYOD1;ID4;TFEB;MXD1;ATOH1
ACBD5
NCEH1
KCMF1
PCYT1B
ONECUT2
CDH2
ERBB4
PDE5A
CTBS
SORCS1
SENP2
ERBB4
SIX1
TCEA3
PTPRM;PTPN11;PTPN4
KAT2B;CREBBP;TRIM24
ABHD4;ABHD2
DRP2;ACTN4
NRP2;BTBD9
PDHB
MGAM
XIAP
FADS6
FUT11
FAM84A
TBL1XR1
CNOT6;ANGEL2
PPWD1;PPIC
KLHL7;KLHL32;KLHL23;BTBD9
TGM2
TGM2
SLC4A10
DLST

FRAS1
MACROD2
AGO1
METAP2
FRMPD2;PTPN4
SDR16C5;HSDL1;HPGD
PHC2;SFMBT2;CNKSR3;BFAR
EFEMP1;FBLN5
DTX1;DTX4
ZZZ3;MIER3
ITM2C
TRIM3
TFAM
SLIT2
SFMBT2
KCNH1
NFATC3
NFATC3
KIF5C;KIF26B;KIF1B
RXRA;RORB;ESR1
CYTH3;ACAP2;SWAP70;GAB1;KIF1B;GAB3;PLEKHA3;SOS2;PLEKHM3;APPL1
E2F1
HEYL
HDAC5
NKRF
MARCH7
PRDM8;ZNF275;PLAG1;ZNF292;PRDM6;GLIS3;ZBTB20;ZBTB44;ZNF25;IKZF3;MECOM;ZNF629;SALL4;PRD
FRMPD2;PTPN4
RXRA;RORB;ESR1
ABCA1;ABCA2;TAP2
FOXJ2;FOXP2;FOXO1
TRIM41;TRIM3;TRIM24;TRIM44
DENND5B
CYB5R4
IRS4
CYB5R4
KCNC2;KCNRG;KCTD16
LYN;ERBB4;PTK2B;RAF1;FRK;FGFR1;BMPR1A
CCND3;CCNYL1
UBXN7
MMP2
MB
POGK
RNF217
HIF1A
TRIM24;RSF1;PHF8
TDRKH
SH3GL2
USP13
MYH10
MAP3K2
ARID5B
DENND5B

RNASE6
ADAMTS5;ADAMTS2;CTGF
TMEM56
STAU1
CTGF
CDYL2
GNA13
POU3F1
INHBA;GDF6
TRIM3;RC3H1
ALAS2
CCND3
DHX40
PSMD11
KCNIP2;MICU3
MYO1C;MYH10
CLCN5
CA12
GRAMD4
DHX40
SFRP1
LOXL4
CBFA2T2
ADCY9
CTGF
DDI2;UBL7
PTP4A1;DUSP19
MMP2
ARHGAP20;ARHGAP31;SRGAP2
NECAB3
DENND5B
NRXN3;SLIT2;SELE
PHF8
LHX6;PRICKLE1;LHX4
FNDC3B;PTPRM;FNDC3A;L1CAM;SORL1;EPOR
TUBD1
MMP2
GALNT7
COL6A5;ANTXR2
ARHGEF10;SOS2;ARHGEF5
NRP2;LRP12
MOCS3
RAF1;CDC42BPA
OCLN
TFAM;SOX6
SUSD1;SELE
EFEMP1;SUSD1;FBLN5
CRK;SH3GL2
SLIT2
CADM3
CNBP
ELK4
PPP2CA

S100B
TXNL1
EMB;LRIG2;L1CAM;MYPN;FGFR1
NRXN3
ASB7
DHX40;DDX19B
SELE;DGCR2
SOS2
CD244
KLK13
CADM3;CXADR;IGSF3;PTGFRN;SCN3B;MPZL3;JAM2

A2;PCDHA9;PCDHA8;PCDHAC2;PCDHA7;PCDHAC1;PCDHA6
CDHA3;PCDHA2;PCDHA9;PCDHA8;PCDHAC2;PCDHA7;PCDHAC1;PCDHA6
ER2;G3BP1;IGF2BP1;RBMS1;HNRNPC;RBM12;TARDBP;RBM7;SRSF10;PPARGC1B;SRSF9

.7

K1;CSNK1A1;NEK7;DYRK1A;HIPK1;CDC42BPA;HIPK2;MAPK10;CAMK4;SIK3;CDK1;SIK2;TRIB2

)M16;ZNF800;HIVEP2;ZNF148;ZNF687;ZNF423;ZNF367;ZNF366;KLF10;ZBTB14;EGR4;BCL11B;ZBTB16

;ZFP91;ZBTB34;ZFY;ZFX;ZNF70;ZFP62;SP1;SP4;ZNF236;ZNF597
