# Supplementary material for: Circulating miR-330-3p in Late Pregnancy is Associated with Pregnancy Outcomes Among Lean Women with GDM
Source: Sci Rep. 2020 Jan 22;10:908. doi: 10.1038/s41598-020-57838-6 (PMC6976655; doi:10.1038/s41598-020-57838-6)
Supplement: Supplementary file 1 — Supplementary data. [file 41598_2020_57838_MOESM1_ESM.zip › Supplimentary File_EnrichR_Analysis Phosphatase_Substrates_DEPOD.pdf]

Term	Overlap	P.value	Adjusted.P.value	Old.P.value	Old.Adjusted
CDC25A	3/8	0,00630859	0,37220681	0	0
PTPN1	6/38	0,012465912	0,367744398	0	0
PPP2CA	10/88	0,015102333	0,297012551	0	0
PTPN11	4/21	0,02088453	0,308046814	0	0
PTPRJ	3/17	0,054236191	0,639987052	0	0
PPP5C	2/8	0,060539704	0,595307092	0	0
DUPD1	1/3	0,146956321	1	0	0
DUSP4	1/3	0,146956321	1	0	0
PTPN7	1/3	0,146956321	0,963380328	0	0
PTPRZ1	1/3	0,146956321	0,867042295	0	0
PPM1F	1/4	0,190979919	1	0	0
PTPN23	1/4	0,190979919	0,9389846	0	0
PTPRF	1/4	0,190979919	0,866755015	0	0
DUSP1	1/5	0,232733649	0,980806093	0	0
DUSP9	1/5	0,232733649	0,91541902	0	0
PTEN	1/5	0,232733649	0,858205331	0	0
PTPRA	1/5	0,232733649	0,807722665	0	0
DUSP22	1/6	0,272334438	0,892651769	0	0
PTPN12	1/7	0,309893194	0,962299919	0	0
PTPRE	1/7	0,309893194	0,914184923	0	0
PPP3CA	2/25	0,372378865	1	0	0
PTPRC	1/9	0,379299997	1	0	0
DUSP3	1/13	0,497890471	1	0	0
ACP1	1/15	0,548404355	1	0	0
PTPN6	1/19	0,63470968	1	0	0
PPM1A	1/21	0,671469795	1	0	0
PPP1CA	1/46	0,912824654	1	0	0

Odds.Ratio	Combined.Score
7,26744186	36,81572007
3,05997552	13,41725036
2,202255109	9,233848743
3,69139904	14,28108748
3,41997264	9,967191743
4,84496124	13,58747994
6,45994832	12,38772527
6,45994832	12,38772527
6,45994832	12,38772527
6,45994832	12,38772527
4,84496124	8,021254818
4,84496124	8,021254818
4,84496124	8,021254818
3,875968992	5,650622543
3,875968992	5,650622543
3,875968992	5,650622543
3,875968992	5,650622543
3,22997416	4,201306255
2,76854928	3,243431827
2,76854928	3,243431827
1,550387597	1,531540293
2,153316107	2,087484578
1,490757305	1,03961712
1,291989664	0,776152959
1,01999184	0,463675624
0,92284976	0,367558366
0,421300977	0,038427482

Genes
CDK1;MAPK1;RAF1
GAB1;CTNNB1;PTPN11;RAF1;CRK;EPOR
MAP2K1;BMPR2;RBL1;CREB1;CAMK4;PPP1R1B;CDK1;MAPK1;RAF1;PRKG1
GAB1;PTK2B;SPRY1;SELE
LYN;OCLN;MAPK1
CDC37;RAF1
MAPK1
MAPK1
MAPK1
ADD2
CAMK4
CTNNB1
CTNNB1
MAPK1
MAPK1
CREB1
LYN
MAPK1
PTK2B
MAPK1
RCAN1;PPP1R1B
LYN
MAPK1
CTNNB1
LYN
CDC42BPA
RAF1
