# Supplementary material for: Circulating miR-330-3p in Late Pregnancy is Associated with Pregnancy Outcomes Among Lean Women with GDM
Source: Sci Rep. 2020 Jan 22;10:908. doi: 10.1038/s41598-020-57838-6 (PMC6976655; doi:10.1038/s41598-020-57838-6)
Supplement: Supplementary file 1 — Supplementary data. [file 41598_2020_57838_MOESM1_ESM.zip › Supplimentary File_EnrichR_Analysis PPI_Hub_Proteins.pdf]

Term	Overlap	P.value	Adjusted.P.value	Old.P.value	
SMAD3	40/328	4,09214E-07	0,000157547		0
CREBBP	40/334	6,56798E-07	0,000126434		0
GRIN1	23/149	2,38463E-06	0,000306028		0
UBC	54/540	2,6161E-06	0,000251799		0
GSK3B	65/696	2,70501E-06	0,000208285		0
CRK	28/215	6,27928E-06	0,000402921		0
AR	30/243	8,7767E-06	0,000482719		0
SMAD4	28/221	1,06321E-05	0,00051167		0
CDK2	61/675	1,51392E-05	0,000647622		0
EP300	38/357	1,99392E-05	0,000767661		0
MAPK3	35/326	3,49951E-05	0,001224827		0
HDAC4	18/127	9,17704E-05	0,002944299		0
SMAD1	21/162	9,34785E-05	0,002768403		0
SMAD2	37/370	9,5988E-05	0,00263967		0
PARP1	19/140	0,000107312	0,002754336		0
CTNNB1	34/337	0,000153094	0,003683821		0
YWHAB	66/812	0,00017332	0,003925188		0
MAPK10	22/182	0,000181428	0,003880534		0
SP1	23/195	0,000188942	0,003828555		0
SHC1	23/195	0,000188942	0,003637128		0
SUMO1	23/198	0,000236806	0,004341452		0
HDAC3	20/164	0,00031132	0,005448103		0
SRC	45/513	0,000374283	0,006265178		0
GRIN2B	24/219	0,00041927	0,006725797		0
ESR1	50/591	0,00042247	0,006506044		0
CDK5	18/146	0,000534188	0,007910087		0
RPS6KA3	35/375	0,000538239	0,007674886		0
HIST1H3A	16/122	0,000539113	0,007412809		0
MYC	43/498	0,000690843	0,009171539		0
PRKCB	32/338	0,000717991	0,009214223		0
CSNK2A1	47/564	0,000856536	0,010637621		0
RB1	22/209	0,001219229	0,014668846		0
PPP1CA	20/184	0,001350952	0,015761106		0
PRKACA	38/440	0,001371283	0,01552776		0
JUN	22/211	0,001379639	0,01517603		0
PRKCD	19/173	0,001559338	0,016676256		0
HDAC1	31/346	0,002076535	0,021607184		0
CDH1	15/127	0,002309773	0,023401651		0
TP53	41/502	0,002566097	0,02533198		0
MAPK8	31/352	0,002701024	0,025997353		0
NCOR1	16/142	0,002728269	0,025619116		0
CHUK	17/157	0,003114437	0,028549005		0
SMARCA4	16/144	0,003141312	0,028125698		0
UBE2I	21/212	0,003255084	0,028481988		0
FYN	36/433	0,003405235	0,029133679		0
INSR	20/202	0,004042654	0,033835254		0
YWHAZ	40/500	0,004108211	0,033652369		0
PRKCA	43/547	0,004108611	0,032954487		0
CSNK2A2	26/289	0,004312889	0,033886981		0
CSNK1E	19/195	0,005927111	0,045638754		0
NEDD4	15/141	0,006229759	0,047028573		0
MAPK1	33/406	0,006845268	0,050681309		0

DLG4	33/409	0,007611438	0,055290636	0
HDAC2	20/214	0,007613777	0,054283411	0
SFN	17/173	0,008229164	0,057604146	0
BRCA1	20/216	0,008403976	0,057777335	0
YWHAG	34/428	0,008560048	0,05781787	0
ABL1	29/351	0,008697481	0,057733276	0
AKT1	29/355	0,010079962	0,06577602	0
YWHAH	17/177	0,010225166	0,065611483	0
APC	15/150	0,010802087	0,068177106	0
MAPK9	20/222	0,011179906	0,069423609	0
PRKCE	18/193	0,011185666	0,068356849	0
CREB1	13/124	0,011721655	0,070513084	0
RELA	24/283	0,011903138	0,070503201	0
IKBKE	35/454	0,011923686	0,069554835	0
PIK3R1	25/301	0,013282558	0,076325145	0
TBP	15/154	0,013534698	0,076630274	0
ARRB2	26/323	0,016923262	0,094426896	0
FOS	16/173	0,017340959	0,095375272	0
ESR2	28/361	0,021148315	0,114677482	0
PTK2	15/163	0,021628257	0,115651098	0
SGK1	13/135	0,022384778	0,118056706	0
MEPCE	15/165	0,023840485	0,124034956	0
CBL	16/180	0,024199204	0,12422258	0
MAP3K3	19/227	0,026304708	0,133254113	0
PTPN11	15/168	0,027470831	0,137354153	0
IKBKB	15/169	0,028767466	0,141993263	0
MCC	23/292	0,029380679	0,143184324	0
EWSR1	18/215	0,029926031	0,144019025	0
CALM1	27/359	0,032621646	0,155053501	0
HNRNPK	16/188	0,034333999	0,161202311	0
HNRNPA1	12/130	0,036510606	0,169356426	0
ATM	18/222	0,039189085	0,179616641	0
CHD3	12/132	0,040331335	0,182677223	0
STAT3	16/192	0,040429161	0,180991011	0
MAP1LC3A	28/383	0,04076986	0,180418345	0
EGFR	33/467	0,042393726	0,185472553	0
DYNLL1	15/183	0,051945598	0,224708487	0
HSPA8	15/183	0,051945598	0,222211726	0
NFKB2	15/184	0,053987625	0,228409182	0
SNCA	24/328	0,054472313	0,227954789	0
PDGFRB	12/140	0,058397767	0,241754197	0
PIK3CA	11/125	0,058431313	0,239319741	0
PRKCZ	13/156	0,060349407	0,244573911	0
KAT5	11/126	0,061184247	0,245374325	0
MAPK14	37/552	0,063001019	0,250055589	0
JAK2	13/159	0,068041778	0,267306984	0
ERBB2	14/175	0,069159965	0,268955419	0
SMAD9	11/129	0,06994741	0,269297529	0
GRB2	49/767	0,072018052	0,274524257	0
HSPA1A	12/145	0,072073034	0,272040375	0
ACTB	24/339	0,073239317	0,273758611	0
NR3C1	16/209	0,075013163	0,277692961	0
SUMO2	13/162	0,076351771	0,279956493	0

POLR2A	13/165	0,08528818	0,309773106	0
RAF1	13/166	0,088407189	0,318100634	0
YWHAE	16/215	0,090844241	0,323842896	0
CUL1	12/151	0,091006733	0,321445801	0
SIN3A	14/183	0,091696956	0,320939345	0
CDK1	42/659	0,092582273	0,321118696	0
PPP2CA	14/184	0,094802431	0,325883357	0
PRKG1	10/121	0,095411477	0,325074501	0
TNIK	10/121	0,095411477	0,32222297	0
SETDB1	11/138	0,100857952	0,337654883	0
EIF2C2	12/156	0,108912518	0,361476892	0
MAP1LC3B	22/322	0,110248777	0,362784436	0
CAV1	10/125	0,111968123	0,365319722	0
IRS1	12/157	0,112724954	0,364698382	0
EIF1B	12/159	0,120578963	0,386857507	0
CASP3	14/193	0,125642471	0,399771498	0
GABARAPL2	34/539	0,131951023	0,416402818	0
HTT	10/130	0,134793004	0,421913062	0
SMURF1	10/131	0,139634202	0,433541677	0
HSP90AA1	16/231	0,142703651	0,439527246	0
MAP3K1	13/184	0,15644598	0,478029385	0
UBB	10/135	0,159884578	0,484689468	0
CDK4	10/135	0,159884578	0,480902831	0
NFKB1	20/305	0,162413731	0,484723149	0
CCDC85B	10/136	0,165162226	0,489134285	0
SYK	10/136	0,165162226	0,485400435	0
BCL2	9/121	0,173041408	0,504704108	0
PLCG2	9/121	0,173041408	0,50090934	0
YWHAQ	13/188	0,174501933	0,501367494	0
PRKAB1	15/223	0,178615302	0,50938438	0
CALM3	12/172	0,178787463	0,506126274	0
STAT1	10/139	0,181488415	0,510022188	0
XPO1	9/124	0,190899161	0,532580993	0
SMURF2	9/124	0,190899161	0,528749476	0
TNFRSF1B	9/124	0,190899161	0,524972694	0
SLC2A4	38/635	0,192142267	0,524643779	0
NCK1	16/244	0,194771469	0,528077575	0
ARRB1	17/263	0,201529929	0,542580577	0
CAMK2A	11/160	0,204601982	0,547026133	0
IL7R	9/127	0,209514619	0,556297436	0
GABARAP	29/479	0,21111487	0,556707021	0
RFC1	9/128	0,215877432	0,565393275	0
LYN	13/197	0,218599441	0,56865395	0
MDM2	13/197	0,218599441	0,564837481	0
ARF6	11/163	0,22148041	0,568466385	0
TGFBR1	14/216	0,226628011	0,577826384	0
CASP8	9/130	0,228824946	0,579589502	0
SRRM2	11/166	0,238897484	0,601147264	0
EEF1A1	12/185	0,247955113	0,619887782	0
MAP3K7	11/168	0,250785124	0,622917888	0
FLNA	9/135	0,262368016	0,64751081	0
TRAF6	32/550	0,265275232	0,650515696	0
PRKDC	18/296	0,269465654	0,656609346	0

SKP1	12/190	0,27688808	0,67045227	0
PLCG1	15/244	0,279162599	0,671735003	0
PTPN6	11/173	0,281364696	0,67282862	0
PRKACB	11/174	0,287612974	0,68352466	0
RNPS1	10/157	0,292466575	0,690795285	0
C1ORF103	8/122	0,294472189	0,691291419	0
TAF9	8/122	0,294472189	0,687101774	0
PAK1	11/176	0,300226355	0,696308112	0
XRCC6	9/141	0,304447506	0,701869998	0
RAC1	15/249	0,305567009	0,700257728	0
ATN1	8/124	0,309766268	0,705680551	0
GNAI2	8/124	0,309766268	0,701529489	0
LCK	12/196	0,312905909	0,704495759	0
TUBB	8/125	0,3174752	0,710627627	0
IGF1R	13/215	0,318335377	0,708434221	0
231403	17/288	0,318799344	0,705389354	0
PIN1	9/145	0,333327815	0,733321193	0
PPP2R1A	9/146	0,340625203	0,745117631	0
ITGB1	8/129	0,348641582	0,758344685	0
VHL	18/314	0,356967482	0,772092587	0
DIPA	8/134	0,388083774	0,834705323	0
UBQLN4	10/171	0,388574731	0,831118174	0
ACTG1	14/246	0,392130982	0,834090763	0
TNFRSF1A	10/173	0,402582576	0,851616987	0
MTOR	8/138	0,419762631	0,883107175	0
HSP90AB1	8/138	0,419762631	0,878307679	0
MAPK13	8/139	0,427672846	0,890021868	0
HGS	7/122	0,442349738	0,915616393	0
GNAI3	7/125	0,467662423	0,9628344	0
TRAF2	15/279	0,472857057	0,968350889	0
ACTA1	9/165	0,48095215	0,979717342	0
GC20	9/165	0,48095215	0,974560935	0
PCNA	8/146	0,482601167	0,972782457	0
RUVBL1	8/147	0,490350996	0,983255904	0
DNM1	7/129	0,500956793	0,999317955	0
COPS5	7/132	0,52547721	1	0
NFKBIA	7/134	0,541567612	1	0
EIF2C1	9/174	0,545480311	1	0
RPOB	9/174	0,545480311	1	0
JAK1	7/136	0,55742915	1	0
TOP1	7/136	0,55742915	1	0
RHOA	7/139	0,580752411	1	0
GABARAPL1	25/499	0,590272046	1	0
VCL	6/121	0,598259624	1	0
HSPA5	6/121	0,598259624	1	0
SQSTM1	8/165	0,622211052	1	0
COPS6	7/147	0,63983167	1	0
MED19	6/127	0,645270972	1	0
IKBKG	16/332	0,646994664	1	0
PHLDA3	6/129	0,660180622	1	0
H2AFX	8/172	0,66824946	1	0
EPB41	8/172	0,66824946	1	0
RPS27A	8/173	0,674542682	1	0

PTP4A3	6/137	0,715791663	1	0
CCT3	9/202	0,720631197	1	0
RUVBL2	7/161	0,730665961	1	0
ALB	7/164	0,747903691	1	0
UBA1	6/144	0,759031668	1	0
NPM1	6/144	0,759031668	1	0
ARF1	6/145	0,764794911	1	0
APP	10/247	0,825373959	1	0
RIF1	6/157	0,826118398	1	0
CDC42	8/205	0,835823282	1	0
TAF1	5/138	0,845759399	1	0
RPA2	6/162	0,847599405	1	0
MAP3K14	6/166	0,86318436	1	0
RTN1	4/123	0,8844425	1	0
EEF1G	4/124	0,888158441	1	0
BTK	4/128	0,9019964	1	0
CLPB	4/132	0,91429955	1	0
AGP1	6/189	0,929144567	1	0
TCP1	4/139	0,932548414	1	0
VIM	6/199	0,947728384	1	0
TAF6	3/124	0,957910269	1	0
PMP3	4/156	0,963126998	1	0
PMP2	5/190	0,970516182	1	0
MDFI	4/163	0,971481709	1	0
CCT2	5/193	0,973425804	1	0
ATG12	4/165	0,973521628	1	0
RPL3	4/166	0,974489519	1	0
PLK1	4/166	0,974489519	1	0
CLTC	2/120	0,987126262	1	0
ERG11	2/121	0,987705584	1	0
SPF1	3/155	0,988075418	1	0
GSTK1	2/122	0,988259467	1	0
GFA1	2/124	0,989295188	1	0
SDHA	2/127	0,990683579	1	0
RPL7A	4/193	0,990940551	1	0
ATXN1	4/194	0,991290139	1	0
MCM2	2/129	0,991509729	1	0
PWP2	2/131	0,992264103	1	0
CLPP	2/131	0,992264103	1	0
CCT8	5/232	0,993540909	1	0
SAC1	2/136	0,99387461	1	0
TSGA14	2/137	0,994154784	1	0
HTZ1	2/138	0,994422382	1	0
RPL5	4/207	0,994808097	1	0
SUA7	2/146	0,996171814	1	0
APJ1	2/147	0,996348388	1	0
RPL27A	2/147	0,996348388	1	0
CCT4	4/218	0,99667467	1	0
RPS3	3/189	0,997223135	1	0
KAP123	2/153	0,997251458	1	0
LSM1	2/154	0,997378889	1	0
YKE2	2/157	0,997727266	1	0
RPLL	2/159	0,997933727	1	0

TDH3	1/121	0,9983853	1	0
RPLS	1/122	0,998468975	1	0
RPSJ	1/122	0,998468975	1	0
BZZ1	1/124	0,998623528	1	0
GAP1	2/169	0,998718793	1	0
HRR25	1/126	0,998762462	1	0
SET3	1/128	0,998887353	1	0
BFR2	1/129	0,998944982	1	0
RPOC	1/130	0,99899962	1	0
HMT1	2/175	0,999039405	1	0
TIG	1/132	0,999100537	1	0
OST1	1/132	0,999100537	1	0
RPLV	1/133	0,999147102	1	0
DED1	1/133	0,999147102	1	0
SEC63	2/178	0,999168479	1	0
BMS1	1/134	0,99919125	1	0
TAF2	2/180	0,999244815	1	0
CCT6	1/138	0,999346084	1	0
RPL20A	2/184	0,999377232	1	0
AAR2	1/140	0,999411965	1	0
SSC1	3/225	0,999439504	1	0
RSC4	1/144	0,999524408	1	0
KAR2	1/144	0,999524408	1	0
RPLC	1/154	0,999719851	1	0
RPS4A	1/155	0,999734255	1	0
RNR2	1/158	0,999773127	1	0
HSP60	1/159	0,999784761	1	0
RPS0A	1/160	0,99979579	1	0
SSE2	3/249	0,999810454	1	0
CAJ1	2/211	0,99983084	1	0
GCN1	1/165	0,999842907	1	0
DJP1	1/165	0,999842907	1	0
HHT1	1/166	0,999850913	1	0
RPN1	1/166	0,999850913	1	0
RPSG	1/174	0,999901644	1	0
SCJ1	1/180	0,999927727	1	0
CDC14	1/184	0,999941003	1	0
RPSE	1/194	0,999964045	1	0
ADH1	1/202	0,999975373	1	0
TUB1	2/254	0,999976703	1	0
TUB2	2/255	0,999977656	1	0
GIM3	1/205	0,999978513	1	0
RPL4A	1/208	0,999981186	1	0
RPL4B	1/212	0,999984139	1	0
SSA3	3/312	0,999986447	1	0
NAF1	1/216	0,999986519	1	0
RPLD	1/223	0,999989625	1	0
GCN5	1/229	0,999991493	1	0
SSB1	19/3026	0,999992747	1	0
SSA1	14/2314	0,999993157	1	0
SSA2	15/2163	0,999993261	1	0
SSE1	10/1780	0,999993564	1	0
SSB2	6/1156	0,999994231	1	0

HTB1	1/245	0,999994294	1	0
HSP82	3/997	0,999994456	1	0
GROL	2/763	0,999994856	1	0
HSP78	4/746	0,999994889	1	0
HSP104	3/360	0,999994959	1	0
TEF1	1/703	0,999994976	1	0
SSZ1	3/645	0,999995101	1	0
HSC82	5/477	0,999995345	1	0
HSP42	3/373	0,99999536	1	0
SIS1	3/507	0,999995445	1	0
UBC7	4/494	0,99999547	1	0
ECM10	3/492	0,999995486	1	0
VMA2	2/491	0,99999549	1	0
URA2	3/415	0,999995659	1	0
PSA1	2/434	0,99999566	1	0
SWA2	1/284	0,999995968	1	0
ECM4	1/303	0,999996051	1	0

Old.Adjusted	Odds.Ratio	Combined.Score
0	2,363395727	34,76325351
0	2,320939516	33,04063635
0	2,991519692	38,72960525
0	1,937984496	24,91051663
0	1,809899314	23,20364501
0	2,523886786	30,23175826
0	2,392573452	27,85771356
0	2,455364972	28,11793511
0	1,751363767	19,43702545
0	2,06284064	22,32575407
0	2,080658201	21,34818466
0	2,74674968	25,53439268
0	2,512202125	23,30765534
0	1,937984496	17,92885125
0	2,630121816	24,03871339
0	1,955236584	17,17569644
0	1,575209073	13,64189507
0	2,342618622	20,18084884
0	2,285827867	19,59885289
0	2,285827867	19,59885289
0	2,251194112	18,79357032
0	2,363395727	19,08368465
0	1,6999864	13,41373833
0	2,123818626	16,51692549
0	1,639580792	12,73854462
0	2,389295954	18,00277952
0	1,80878553	13,61510552
0	2,541619011	19,12716889
0	1,673360107	12,17804155
0	1,834778221	13,28205675
0	1,61498708	11,40603117
0	2,03998368	13,6873455
0	2,106504887	13,91756365
0	1,673713883	11,03313645
0	2,020647342	13,30784865
0	2,128422279	13,7570441
0	1,736344491	10,72549516
0	2,288958066	13,8953623
0	1,582816023	9,442082164
0	1,70674771	10,09391833
0	2,183644503	12,8924288
0	2,09845455	12,11166466
0	2,153316107	12,40980793
0	1,919701624	10,99516228
0	1,611257318	9,155875097
0	1,918796531	10,57420741
0	1,550387597	8,519019447
0	1,523461304	8,370917388
0	1,743515464	9,495442225
0	1,888292586	9,683576738
0	2,061685634	10,47010066
0	1,575209073	7,85115345

0	1,563654972	7,627670214
0	1,811200464	8,834666171
0	1,904377829	9,141148556
0	1,794430089	8,575671756
0	1,539520394	7,329116908
0	1,601183772	7,597171686
0	1,583142264	7,278030848
0	1,861341041	8,530346049
0	1,937984496	8,775224681
0	1,745931978	7,845584936
0	1,80744668	8,121078656
0	2,031757939	9,033840384
0	1,643520421	7,282362107
0	1,494040911	6,617448497
0	1,609621675	6,955663872
0	1,887647236	8,121599751
0	1,55998752	6,363292298
0	1,792355603	7,267435643
0	1,50314587	5,796423662
0	1,783421315	6,83719969
0	1,866207293	7,090419667
0	1,761804087	6,582752095
0	1,722652885	6,410741672
0	1,622101561	5,901217395
0	1,7303433	6,219944877
0	1,720104582	6,103808609
0	1,526494637	5,384584638
0	1,622498648	5,693390865
0	1,457537086	4,988827675
0	1,649348507	5,560975091
0	1,788908766	5,921560787
0	1,571338781	5,090127288
0	1,761804087	5,656495011
0	1,61498708	5,18120793
0	1,416803287	4,533504443
0	1,369453712	4,328507522
0	1,588511882	4,698116495
0	1,588511882	4,698116495
0	1,579878665	4,6116665
0	1,418037436	4,126577881
0	1,661129568	4,71840137
0	1,705426357	4,843246011
0	1,61498708	4,534244451
0	1,691891227	4,72691656
0	1,299011347	3,591252468
0	1,584515626	4,25859709
0	1,550387597	4,141601743
0	1,652544919	4,39578866
0	1,238086575	3,257205792
0	1,603849238	4,218244278
0	1,372024422	3,586503241
0	1,483624495	3,842723443
0	1,555172744	4,000532673

0	1,526896876	3,758791668
0	1,517698702	3,681636527
0	1,44222102	3,459324143
0	1,540120129	3,691393473
0	1,48261109	3,542352415
0	1,235134277	2,939196653
0	1,474553421	3,47398921
0	1,601640079	3,763143706
0	1,601640079	3,763143706
0	1,544770251	3,543768092
0	1,490757305	3,30532246
0	1,324088786	2,919636773
0	1,550387597	3,394637314
0	1,481262035	3,233305376
0	1,462629808	3,094120878
0	1,405791862	2,916055061
0	1,222476306	2,475911167
0	1,490757305	2,987499969
0	1,479377478	2,912493515
0	1,342326924	2,61349061
0	1,369228177	2,5399792
0	1,435544071	2,631787416
0	1,435544071	2,631787416
0	1,270809506	2,309833914
0	1,4249886	2,566158088
0	1,4249886	2,566158088
0	1,441476071	2,528672436
0	1,441476071	2,528672436
0	1,340095662	2,339565086
0	1,303577015	2,2454387
0	1,352082207	2,327687311
0	1,39423345	2,379347858
0	1,40660165	2,329346317
0	1,40660165	2,329346317
0	1,40660165	2,329346317
0	1,159738754	1,913011349
0	1,270809506	2,07895331
0	1,252689598	2,006579969
0	1,332364341	2,114047494
0	1,37337484	2,14653236
0	1,173310029	1,824911138
0	1,362645349	2,088995923
0	1,278873018	1,944544663
0	1,278873018	1,944544663
0	1,307842298	1,971469124
0	1,256101062	1,864613351
0	1,341681574	1,978709295
0	1,284206594	1,838625234
0	1,257071025	1,75299503
0	1,26891842	1,755115662
0	1,291989664	1,728691367
0	1,127554616	1,496250746
0	1,178504085	1,545389309

0	1,223990208	1,571777108
0	1,191383912	1,520159259
0	1,232244477	1,562613659
0	1,225162612	1,526723578
0	1,234385029	1,517558993
0	1,270809506	1,553654487
0	1,270809506	1,553654487
0	1,21124031	1,457386838
0	1,237011381	1,47112395
0	1,16746054	1,384125086
0	1,250312578	1,465287873
0	1,250312578	1,465287873
0	1,18652112	1,37856282
0	1,240310078	1,423076682
0	1,171804579	1,341305886
0	1,143949182	1,307755142
0	1,202886929	1,321526276
0	1,194647977	1,286603041
0	1,20185085	1,266403304
0	1,110946526	1,14439778
0	1,157005669	1,095145264
0	1,133324267	1,07129717
0	1,102918006	1,032507012
0	1,120222252	1,019239869
0	1,123469273	0,975245356
0	1,123469273	0,975245356
0	1,11538676	0,947405895
0	1,111958317	0,906973747
0	1,085271318	0,824815493
0	1,041927148	0,780363987
0	1,057082452	0,773771136
0	1,057082452	0,773771136
0	1,061909313	0,773669649
0	1,05468544	0,751604519
0	1,051619494	0,726916646
0	1,027719051	0,661284238
0	1,012379961	0,620879833
0	1,002405774	0,607546679
0	1,002405774	0,607546679
0	0,99749202	0,582954156
0	0,99749202	0,582954156
0	0,975963415	0,530368537
0	0,970934116	0,511849041
0	0,960984048	0,493686782
0	0,960984048	0,493686782
0	0,939628847	0,445831273
0	0,92284976	0,412098701
0	0,915583227	0,401103222
0	0,933968432	0,406665949
0	0,901388138	0,374294043
0	0,901388138	0,363343908
0	0,901388138	0,363343908
0	0,896177802	0,352843416

0	0,848752334	0,283794031
0	0,863458439	0,282892979
0	0,842601955	0,264407554
0	0,827188504	0,240282598
0	0,80749354	0,222635481
0	0,80749354	0,222635481
0	0,801924619	0,215034139
0	0,784609108	0,15058117
0	0,740631018	0,141473246
0	0,756286633	0,135630987
0	0,702168296	0,117627485
0	0,717772036	0,118681564
0	0,700476324	0,103058969
0	0,630238861	0,077391931
0	0,625156289	0,074146741
0	0,605620155	0,062466539
0	0,587268029	0,052617469
0	0,615233173	0,045214062
0	0,55769338	0,038946077
0	0,584316934	0,031370418
0	0,468867217	0,020161839
0	0,496919102	0,01866925
0	0,50999592	0,015262751
0	0,475579017	0,013759851
0	0,502068522	0,013522549
0	0,469814423	0,012607582
0	0,466984216	0,01206758
0	0,466984216	0,01206758
0	0,322997416	0,004185182
0	0,320328016	0,003962655
0	0,375093773	0,004499719
0	0,317702376	0,003752064
0	0,312578145	0,003364129
0	0,305194409	0,002856647
0	0,401654818	0,003655354
0	0,399584432	0,00349557
0	0,300462713	0,002561901
0	0,295875496	0,002297762
0	0,295875496	0,002297762
0	0,417669072	0,002706513
0	0,28499772	0,001751091
0	0,282917445	0,001658566
0	0,280867318	0,001570956
0	0,374489758	0,001949379
0	0,265477328	0,001018247
0	0,26367136	0,000964588
0	0,26367136	0,000964588
0	0,355593486	0,001184436
0	0,307616587	0,000855398
0	0,253331307	0,00069725
0	0,251686298	0,000660564
0	0,246877006	0,000561724
0	0,243771635	0,00050422

0	0,160164008	0,000258826
0	0,158851188	0,000243392
0	0,158851188	0,000243392
0	0,156289072	0,000215276
0	0,229347278	0,00029403
0	0,153808293	0,000190461
0	0,151405039	0,000168554
0	0,150231356	0,00015858
0	0,14907573	0,000149207
0	0,221483942	0,000212859
0	0,146817007	0,000132116
0	0,146817007	0,000132116
0	0,14571312	0,000124332
0	0,14571312	0,000124332
0	0,217751067	0,00018114
0	0,144625709	0,000117013
0	0,215331611	0,000162677
0	0,140433659	9,18618E-05
0	0,210650489	0,000131227
0	0,138427464	8,14241E-05
0	0,258397933	0,000144871
0	0,134582257	6,40215E-05
0	0,134582257	6,40215E-05
0	0,125843149	3,52598E-05
0	0,125031258	3,32309E-05
0	0,122657247	2,78308E-05
0	0,121885817	2,62374E-05
0	0,121124031	2,47372E-05
0	0,233492108	4,42617E-05
0	0,183695213	3,10766E-05
0	0,117453606	1,84526E-05
0	0,117453606	1,84526E-05
0	0,116746054	1,74067E-05
0	0,116746054	1,74067E-05
0	0,111378419	1,09552E-05
0	0,107665805	7,78157E-06
0	0,105325244	6,21406E-06
0	0,099896108	3,5918E-06
0	0,095939827	2,36276E-06
0	0,152597204	3,55511E-06
0	0,151998784	3,39623E-06
0	0,094535829	2,03129E-06
0	0,093172332	1,75299E-06
0	0,091414363	1,44993E-06
0	0,186344663	2,52545E-06
0	0,089721504	1,20953E-06
0	0,086905134	9,01639E-07
0	0,084628144	7,19954E-07
0	0,12168442	8,82602E-07
0	0,117250575	8,02386E-07
0	0,134395596	9,05697E-07
0	0,108875533	7,00753E-07
0	0,100587431	5,80288E-07

0	0,079101408	4,5133E-07
0	0,058314478	3,23304E-07
0	0,050799069	2,61322E-07
0	0,103913378	5,31099E-07
0	0,161498708	8,14104E-07
0	0,027567347	1,38494E-07
0	0,090138814	4,41561E-07
0	0,203143029	9,45683E-07
0	0,155870067	7,23198E-07
0	0,114673639	5,22389E-07
0	0,156921822	7,10892E-07
0	0,118169786	5,33416E-07
0	0,078940305	3,56014E-07
0	0,140095265	6,08188E-07
0	0,089308041	3,87608E-07
0	0,068238891	2,7515E-07
0	0,063959884	2,52586E-07

Genes
HDAC5;PSMD11;PLAG1;HIF1A;JPH1;DCAF7;FOXO1;JPH3;MECOM;SIN3A;PRDM16;FBXO3;MAPK1;BTRC;F
SET;MAML1;HIF1A;FOXO1;MTDH;PCMT1;NPAT;MECOM;CDH2;SERTAD2;TDG;RUVBL1;E2F1;SNIP1;MAPK
MAP2K1;RALA;ATP2B4;HNRNPU;PTPN11;L1CAM;GRIN2B;LRP8;RAB11A;PPP2CA;MAPK10;GRM5;RAP2A;
CALCOCO2;SH3KBP1;TNFAIP3;BACH1;SPRED1;EEF2K;SCN5A;CCDC50;SH3GL2;UBL7;GPR37;USP2;DIO
JPH1;AFF4;AFF1;MED14;CCND3;GRM5;SIN3A;PAPOLG;DPYSL3;KIF1B;BSN;APPL1;FNBP4;MEF2A;MAP2K
SH3KBP1;IRS4;DTX1;HNRNPR;EFS;PCDHA1;ERBB4;PCDHA5;PTK2B;PCDHA3;PCDHA2;PCDHA9;PCDHA
HIF1A;MED12L;FOXO1;MED14;SART3;NSD1;TDG;TRIM3;RUVBL1;NRIP1;TRIM24;MAPK1;NCOA2;CREBBP
DLX1;RALA;PSMD11;FOXO1;ARL5B;EFEMP1;MECOM;TDG;SNIP1;MAPK1;APBB2;BTRC;ZNF423;HOXC8;M
BTG1;SH3KBP1;JPH1;AFF4;AFF1;AMOT;CKS1B;SIN3A;DPYSL3;BSN;FNBP4;PCYT1B;RUNX1;ADCY9;RBL1
SET;MAML1;GATA6;HNRNPU;DTX1;HIF1A;ING4;ATXN7;TDG;SNIP1;MAPK1;EMB;BTRC;ZNF148;N4BP2;ME
CRP;ROCK1;GATA6;HIF1A;ELK4;EPS8;PPP2CA;NCKIPSD;RPS6KA6;RXRA;C1QBP;EIF4EBP2;MAPK1;BSN
MEF2A;HDAC5;CBX5;ZBTB16;IKZF3;RUNX3;HIF1A;ESR1;PPP2CA;RXRA;MECOM;SP1;SIN3A;CAMK4;LCO
RAB2B;CREBBP;PSMD11;SMURF2;ZBTB44;GDF6;RUNX3;FBXO30;RUNX1;PIAS1;SMAD7;HIPK2;KAT2B;RA
PSMD11;ROCK1;DCUN1D1;RSF1;ANTXR2;LITAF;DCAF7;RXRA;MECOM;PRDM16;FBXO3;SNIP1;MAPK1;RN
CREBBP;XRCC5;SWAP70;SMARCA1;HIF1A;ESR1;FOXO1;MED17;MED14;HNRNPK;RXRA;SP1;CARM1;E2F
SH3KBP1;PTPRM;HIF1A;FOXO1;CDC73;CDH6;GNA13;RXRA;CDH2;RBBP5;RUVBL1;IGF2BP1;BTRC;SOX6
NCKAP1;DIRAS2;PDE3B;TNFAIP3;KIAA1549;EFR3A;GRM5;CAPZB;CDH2;DPYSL5;C1QBP;TRIM3;BSN;AP2
MEF2A;USP47;MAP2K1;CREBBP;PCYT1B;IRS4;NFATC3;GRIN2B;AFF4;MAPK10;GRM5;HSPH1;RAP1A;HN
KLF10;CREBBP;PHC2;CBX5;BCL11B;ZBTB16;GATA6;HIF1A;ESR1;RUNX1;PURA;RXRA;RBL1;SP1;MAN1A2
LYN;MME;USP9X;CALCOCO2;GAB1;IRS4;PTPN11;HIF1A;ESR1;EPOR;EPS8;MAPK10;MYO1C;CDH2;SP1;E
MEF2A;MCTS1;NCOA2;ZNF462;ZBTB16;ARID5B;PROX1;SENP2;SOD2;IKZF3;HIF1A;ESR1;HIPK2;PIAS1;MA
HDAC5;CREBBP;USP15;BCL11B;ZBTB16;PROX1;CBFA2T2;HIF1A;ESR1;RUNX1;SMAD7;KAT2B;RBL1;RXR
BMPR2;SH3KBP1;YTHDC1;FOXO1;EPS8;PPP2CA;BCLAF1;RXRA;EFS;CDH2;SH3PXD2A;G3BP1;DAG1;PT
MAP2K1;RALA;ATP2B4;PTPN11;ACTN4;L1CAM;GRIN2B;RAB11A;PPP2CA;MAPK10;RAP2A;GRM5;CDH2;C
RBM28;HNRNPU;HNRNPR;MED17;MED14;CAPZB;SIN3A;RUVBL1;TRIM24;AP2M1;NCOA2;GTPBP4;PIAS1;
MEF2A;CSNK1G3;MAP2K1;SET;CSNK1A1;AGAP2;MAPK10;PURA;C20ORF112;SIPA1L1;CDC37;DPYSL3;PF
SLC24A2;GABRB1;PDE1B;YTHDC1;PDE3B;RSF1;ADD3;FOXO1;BCLAF1;JPH3;EEF2K;ATXN7;TRA2B;E2F1
CREBBP;SET;CBX5;ROCK1;DNMT3A;DYRK1A;PAX5;DEK;SMARCA1;GATAD2B;PHF8;KAT2B;ATXN7;MYOD
SET;SF3B3;IRS4;HNRNPU;ETFA;HIF1A;GIGYF2;MED14;HSPH1;C1QBP;RUVBL1;IGF2BP1;EPC1;SNIP1;MA
BMPR2;PDE3B;ADD3;ADD2;PPP2CA;GNA13;BCLAF1;GRM5;EEF2K;ATXN7;CLSPN;BSN;EIF4E;JAM2;ABCA
KANK2;LUZP1;DCTN2;HIF1A;PPP2CA;BCLAF1;PGRMC1;HSPH1;SIN3A;KIF5C;G3BP1;UBXN7;MAPK1;BSN;
CBX5;ZBTB16;DNMT3A;MRFAP1;PAX5;PAX2;KAT2B;PURA;CCND3;RBL1;PABPN1;SP1;SIN3A;RBBP5;ID2;M
STAU1;ROCK1;YLPM1;PPP1R9A;ESR1;PPP2CA;HEYL;CCND3;SPRED1;CDC34;CREB1;RBL1;ID2;PPP1R1
HDAC5;GABRB1;PFKFB3;NUFIP2;LUZP1;PDE3B;PPP1R9A;LRP6;ADD2;GNA13;BCLAF1;RAP1A;RXRA;EEF
NCOA2;CREBBP;USP9X;HOXD12;SENP2;HIF1A;ESR1;RUNX1;PIAS1;MAPK10;ITCH;MAF;CREB1;SP1;MYC
LYN;HDAC5;GABRA1;CREBBP;TRIM41;IRS4;ADRA2A;ADD2;PPP2CA;GNA13;GRM5;HNRNPK;PDP2;TBL1X
BACH1;HIF1A;RAP1A;MECOM;SIN3A;NRIP1;E2F1;SOX6;ZNF148;APPL1;MEF2A;CREBBP;CBX5;BCL11B;Z
GABRA1;CREBBP;SET;XRCC5;CSNK1A1;USP9X;PTPRM;SIX1;ACTN4;GNA13;CDH2;C1QBP;CTNNB1;TGM
PSMD11;ARL3;BACH1;HIF1A;MED17;ING4;PPP2CA;SIN3A;RBBP5;TDG;TRIM3;IGF2BP1;E2F1;PCDHA4;MA
CUL3;FUT11;IRS4;BACH1;AFF4;ADD2;ELK4;BCLAF1;TMEM245;RXRA;RAP1A;EEF2K;ATXN7;MAPK1;APBB
HDAC5;CREBBP;SF3B3;ZBTB16;CBFA2T2;BACH1;ESR1;RUNX1;PIAS1;RXRA;TBL1XR1;SP1;SIN3A;ERBB4
MAP3K2;CREBBP;TPM3;IRS4;TNFAIP3;HAUS3;PTPN11;ESR1;PIAS1;SESTD1;CDC37;CAPZA1;CTNNB1;BT
CREBBP;CBX5;HIF1A;ESR1;MED17;RAP1A;CREB1;RBL1;RXRA;PABPN1;SP1;SIN3A;MYOD1;CARM1;CTN
MAPK1IP1L;TFAP2B;HDAC5;TRIM41;DNMT3A;RORB;BMI1;HIPK1;IKZF3;ESR1;HIPK2;PIAS1;HNRNPK;CRE
SHC4;SHC2;SH3KBP1;YTHDC1;AGAP2;HNRNPR;ADD2;NCKIPSD;MED14;EFS;G3BP1;PLXNA2;DAG1;PTK
LYN;SHC4;SHC2;GAB1;IRS4;DYRK1A;PTPN11;EPOR;PPP2CA;RBM3;SNX1;FUBP1;ADAM12;PTK2B;MAPK
NCKAP1;HDAC5;SET;SF3B3;ATL3;ATL2;HNRNPU;TNFAIP3;DCAF7;GIGYF2;FOXO1;HSPH1;PABPN1;TRA2
GPM6A;GABRB1;PFKFB3;YTHDC1;SNAP23;ADD3;FOXO1;PITPNC1;ADD2;PPP2CA;GRM5;C1QBP;PLXNA2
YTHDC1;PPP1R9A;HSPH1;KIF5C;SIN3A;GSE1;CREBBP;XRCC5;TPM3;L1CAM;ZFY;ZFX;ESR1;GATAD2B;O
CSNK1G3;CSNK1A1;NFATC3;PDAP1;GAPVD1;ESR1;LRP6;OCLN;PGRMC1;CDC34;APC;C1QBP;PPP1R1B;
RALA;SMURF2;SH3KBP1;LAPTM5;HNRNPU;LITAF;SMAD7;ITCH;HNRNPK;ERBB4;RUVBL1;MAPK1;SCN5A
ROCK1;GATA6;HIF1A;FOXO1;ELK4;PPP2CA;RPS6KA6;RXRA;TNKS2;C1QBP;E2F1;EIF4EBP2;MAPK1;MAP

NAPA;GPM6A;NAPB;SF3B3;PDHB;LRP8;NCKIPSD;GNA13;SIPA1L1;ALDH2;ERBB4;PTK2B;MAPK1;KIF1B;S
CREBBP;BCL11B;ZBTB16;DEK;IKZF3;RUNX3;HIF1A;ESR1;GATAD2B;RUNX1;SMAD7;MAF;CREB1;RBL1;SF
MAP3K2;HDAC5;WDR37;DYRK1A;HNRNPU;ESR1;RND3;DCAF7;FAM117B;ITCH;SIPA1L1;APC;CDK1;RAF1;
NCOA2;CREBBP;XRCC5;XIAP;SMC1A;BACH1;ESR1;UBE2J1;MED17;PIAS1;ELK4;CREB1;RBL1;SP1;SUB1;
NCKAP1;HDAC5;USP37;SF3B3;NUFIP2;SRSF1;IRS4;TNFAIP3;DCAF7;FOXO1;NCKIPSD;BCLAF1;KIF5C;C1
SHC4;SHC2;YTHDC1;HNRNPR;NCKIPSD;MED14;EFS;ERBB4;TRIM3;E2F1;HOXC8;SNX8;PCDHA7;LYN;CR
PHLPP2;PFKFB3;YTHDC1;CELF1;PDE3B;AGAP2;XIAP;FOXO1;PPP2CA;EEF2K;TRA2B;S1PR1;SRSF10;PP
MAP3K2;NCOA2;HDAC5;CREBBP;CSNK1A1;RASSF8;TNFAIP3;ESR1;GRIN2B;DCAF7;REEP1;C1QBP;NRIP
CSNK1G3;CREBBP;CSNK1A1;GIGYF2;FBXO30;PPP2CA;APC;TBL1XR1;CTNNB1;MAPRE3;BTRC;MYH10;M
PCYT1B;MAP2K1;CXADR;GFPT1;NFATC3;IRS4;DUSP19;AFF4;ADD2;MAPK10;ADCY9;RXRA;RAP1A;EEF2K
MAP2K1;IRS4;PDHB;HIF1A;GRIN2B;HK2;TIAL1;GNA13;OCLN;GRM5;HNRNPK;CREB1;CAPZB;NRIP1;PTK2
CREBBP;DYRK3;CSNK1A1;DYRK1A;PIAS1;HIPK2;CREB1;MYOD1;CARM1;CAMK4;CCDC6;RNF111;PRKG1
CREBBP;IRS4;HNRNPU;ESR1;MTDH;PIAS1;ING4;PPP2CA;KAT2B;HSPH1;RXRA;NKRF;SP1;SIN3A;NSD1;C
NAPA;SET;DCTN2;CALCOCO2;DLST;TNFAIP3;ETFA;PDHB;NUDT5;PFAS;PCMT1;PGRMC1;CAPZB;MAT2A;
SH3KBP1;AGAP2;IRS4;RXRA;EFS;CDH2;DAG1;PTK2B;SNX8;MME;GABRA3;GAB1;GAB3;GTF2H1;PTPN11;
SET;CBX5;PAX5;ESR1;PIAS1;HNRNPK;RXRA;ATXN7;SP1;SIN3A;SUB1;RUVBL1;E2F1;CTNNB1;TEAD1
SF3B3;PFKFB3;HNRNPU;HNRNPR;BCLAF1;C1QBP;IGF2BP1;MAPK1;BSN;MYH10;AP2M1;RAB11FIP5;SRP
NCOA2;CREBBP;SET;NFATC3;SLC4A10;ZFP91;ESR1;RUNX1;ELK4;MAF;MAN1A2;CARM1;SERBP1;MAPK1
KANK2;RBM28;SF3B3;STAU1;MED17;PURA;MED14;BCLAF1;NKRF;SIN3A;NRIP1;TRIM24;MAPK1;SCN1A;N
SHC4;LYN;SHC2;ROCK1;ATP2B4;PTPN11;SELE;PIAS1;EFS;APC;PTK2B;MAPK1;CD47;CRK;TNS1
PFAS;FOXO1;PPP2CA;LETM1;GRM5;MARCKS;CREB1;CDC37;PPP1R1B;MAPK1;CTNNB1;BSN;MARK1
USP15;SET;LRRC40;HNRNPR;CDC73;PURA;HNRNPK;SART3;CAPZB;RUVBL1;IGF2BP1;TRIP12;SNRPD3;
LYN;SH3KBP1;PTPN11;UBE2G1;GAPVD1;EPOR;ITCH;MYO1C;PTK2B;SPRY1;SCN5A;IL6R;CRK;SH3GL2;P
LYN;MAP3K2;MAP2K1;ST13;TMOD3;ZBTB16;GAB1;ACTN4;RCAN1;RNF126;MYO1C;CAPZB;CDC37;C1QBF
LYN;IRS4;GAB1;HNRNPU;GAB3;PTPN11;ACTN4;SELE;GRIN2B;EPOR;ERBB4;PTK2B;CTNNB1;SPRY1;CRK
MAP3K2;CREBBP;SNAP23;IRS4;HNRNPU;TNFAIP3;PTPN11;SNX1;CDC37;C1QBP;CDK1;CTNNB1;BTRC;C
TSFM;DFFA;RRM1;TPM3;CNBP;HSPA4L;DCUN1D1;ETFA;PDHB;NUDT5;SOD2;PFAS;LETM1;PCMT1;PGRM
MAPK1IP1L;CREBBP;DFFA;CPSF7;CXADR;WDR37;PRUNE2;EPT1;MTMR9;DYNLL2;ESR1;ITCH;PTK2B;MA
NCKAP1;NAPB;ADCYAP1R1;RALA;DCTN2;PDE1B;DIRAS2;MAT2B;ADD2;GRM5;SCN5A;SH3GL2;KCNH1;S
LYN;TCERG1;HNRNPU;ESR1;SORBS3;MAPK10;HNRNPK;ALDH2;C1QBP;CARM1;SERBP1;CDK1;RAF1;RB
PURA;HNRNPK;ROCK1;PABPN1;CDK1;IGF2BP1;CTNNB1;HNRNPR;HNRNPC;TARDBP;ESR1;PIAS1
USP15;XRCC5;TIPARP;SMC1A;HIF1A;CREB1;RRM2B;SP1;C1QBP;RFWD3;RUVBL1;DCX;E2F1;UBXN7;CT
TCERG1;PCMT1;HSPH1;CREB1;BCL11B;SP1;SIN3A;FUBP1;SERBP1;IKZF3;ESR1;GATAD2B
LYN;CREBBP;PTPN11;HIF1A;ESR1;TSHR;PIAS1;SP1;MYOD1;PTK2B;MAPK1;PCBD2;ZNF148;IL6R;KPNA1;
SET;SF3B3;CALCOCO2;HSPA4L;SRSF1;IRS4;HNRNPU;HNRNPR;TIAL1;RBM3;PCMT1;PURA;HSPH1;MAT2
SHC4;SHC2;DCTN2;SH3KBP1;IRS4;EPS8;PPP2CA;SNX1;ERBB4;PTK2B;MAPK1;APBB2;AP2M1;CCDC50;S
KANK2;DNMT3A;BACH1;DYNLL2;DCAF7;FBXO30;AMOT;FAM107B;TXNDC17;PPP2CA;RBL1;TRPS1;TRIM2
TCERG1;MAP2K1;GPR37;MME;ST13;ESR1;RBX1;MAPK10;HSPH1;HNRNPK;SERP1;SP1;RUVBL1;CTNNB1
HDAC5;PSMD11;XRCC5;USP9X;USP2;HNRNPU;SP1;NKRF;SIN3A;RUVBL1;TRIM3;E2F1;HNRNPC;BTRC;C
LYN;DCTN2;CSNK1A1;ARL3;TMOD2;AGAP2;DYRK1A;UQCR10;SOD2;DYNLL2;VAPA;VAPB;TRIM3;TFAM;C
SHC4;LYN;SHC2;SNX1;SH3KBP1;GAB1;S1PR1;PDAP1;PTPN11;RAF1;SOS2;CRK
MAP2K1;GABRB1;SH3KBP1;IRS4;GAB1;CTNNB1;PTPN11;ESR1;GRIN2B;FGFR1;APPL1
MAP2K1;CREBBP;TRIM41;IRS4;LNPEP;ADD2;PPP2CA;GRM5;SP1;C1QBP;MAPK1;RAF1;MYH10
FAM101B;CREBBP;CREB1;RUVBL1;CDK1;E2F1;EPC1;BMI1;TBX5;PTPN4;ESR1
PPP1R9A;JPH1;GIGYF2;FOXO1;ELK4;PPP2CA;EEF2K;SIN3A;PAPOLG;TRA2B;PLXNA2;E2F1;GSE1;MAPK
LYN;SH3KBP1;PTPN11;EPOR;TSHR;PPP2CA;G3BP1;PTK2B;MAPK1;RAF1;IL6R;IL13RA1;FGFR1
SHC4;LYN;SHC2;IRS4;PTPN11;ESR1;ERBB4;CDC37;CDK1;PTK2B;CTNNB1;AGTR2;CALM1;CRK
KAT2B;HEYL;UBA6;MAN1A2;SMAD9;C10ORF2;LNPEP;TRIP12;AFF1;SMAD7;BMPR1A
TCERG1;ALAS2;SH3KBP1;IRS4;HNRNPU;HNRNPR;PCMT1;MED14;CAPZB;RUVBL1;DAG1;AP2M1;TMOD3
MAPK10;TCERG1;MAP2K1;RAP1A;GPR37;MME;ST13;TRIM24;TERF2IP;PGAM5;ESR1;RBX1
LYN;PCYT1B;MME;STAU1;CSNK1A1;TPM3;HNRNPU;SMAD9;PARVA;ACTN4;SYNJ2BP;PPP1R9A;GRIN2B;
NCOA2;CREBBP;SET;ZBTB16;HNRNPU;ZBTB20;MAPK10;MED14;CREB1;TDG;NRIP1;TRIM24;MAPK1;CAL
PSMD11;XRCC5;CUL3;SENP2;GATAD2B;PIAS1;PHF8;HNRNPK;RBL1;ZMAT3;TDG;RUVBL1;SOX6

TCERG1;CREBBP;XRCC5;HNRNPU;GTF2H1;CDC73;MED17;KAT2B;MED14;ITCH;RXRA;CDK1;MAPK1
LYN;MAP2K1;XIAP;GRIN2B;RCAN1;RAP2A;EFEMP1;RAP1A;HNRNPK;CDC37;MAPK1;RAF1;PRKG1
MAP3K2;HDAC5;MAP2K1;ADH1B;DYRK1A;MSL2;TNFAIP3;GRIN2B;CDC37;SIK3;HIVEP2;HNRNPC;CALM1;
FBXW4;CDC34;EBF1;DCUN1D1;E2F1;HNRNPU;FBXO3;UBXN7;CTNNB1;BTRC;RBX1;HIPK2
KLF10;HDAC5;ZBTB16;CBFA2T2;IKZF3;ZBTB4;ESR1;RUNX1;MAF;RXRA;SP1;ID2;RLIM;MXD1
SET;YTHDC1;FUT11;HNRNPU;AFF4;GIGYF2;FOXO1;PHF8;CKS1B;BCLAF1;TMEM245;GRM5;RXRA;EEF2K
MAP3K2;IGBP1;HDAC5;MAP2K1;SET;XRCC5;CSNK1A1;GRIN2B;APC;CAMK4;MAPK1;STRN;SIK2;JAM2
ABCA1;GNA13;GABRB1;CREB1;APC;DCX;PDE5A;RAF1;PRKG1;MARK1
PCMT1;RAP2A;RRM1;SET;CMPK1;SORD;TFAM;MAPK1;CALM1;SOD2
TCERG1;PSMD11;CBX5;APC;SIN3A;DNMT3A;SNIP1;PCDHA4;PGAM5;TARDBP;PIAS1
SART3;SF3B3;TMEM33;C1QBP;RUVBL1;IGF2BP1;HNRNPU;HNRNPC;CRK;GIGYF2;DCP2;TNRC6B
XRCC5;MMP2;HSPA4L;SRSF1;YLPM1;HNRNPU;HNRNPR;PDHB;TIAL1;PCMT1;HSPH1;HNRNPK;CAPZB;C
BMPR2;GJA3;DAG1;S1PR1;MAPK1;CTNNB1;STRN;PTPN11;ESR1;LRP6
LYN;MAPK10;ROCK1;CDK1;HNRNPU;MAPK1;CTNNB1;PTPN11;SIK2;ESR1;GRIN2B;CRK
PCMT1;PGRMC1;SET;TPM3;CDC37;CAPZA1;RUVBL1;CMPK1;ETFA;CALM1;SOD2;MTDH
MEF2A;LYN;DFFA;ROCK1;HNRNPU;XIAP;RBX1;PPP2CA;SP1;EIF3J;CTNNB1;PDE5A;BTRC;TGM2
RBM28;CALCOCO2;CUL3;HSPA4L;SRSF1;HNRNPU;DLST;HNRNPR;TIAL1;PCMT1;BCL2L13;PURA;HSPH1;
PPP2CA;TCERG1;KAT2B;CREBBP;SP1;SIN3A;WDFY3;UBE2K;GPRASP2;TGM2
MAP3K2;OCLN;SMURF2;RLIM;FBXO3;LONRF3;APBB2;RUNX3;FBXO30;SMAD7
ST13;CSNK1A1;MMP2;IRS4;AHSA2;HIF1A;ESR1;CNOT6;SP1;SIN3A;CDC37;MYOD1;MAPK1;CALM1;RAF1;
MAP2K1;USP9X;IRS4;DTX1;ESR1;DCAF7;HSPH1;RAP1A;CDC37;MAPK1;RAF1;CALM1;MYH10
LYN;ITCH;CDC34;SMURF2;SH3KBP1;USP1;UBXN7;XIAP;ESR1;CCDC50
LYN;NCOA2;CCND3;RBL1;SP1;CELF1;CDC37;MYOD1;SMC1A;RUNX3
CREBBP;RSF1;C1ORF52;ESR1;RXRA;NKRF;SP1;SIN3A;C1QBP;CARM1;TRIM3;RUVBL1;E2F1;CTNNB1;BT
EPS8;CCDC112;FCHSD2;ZBTB16;USP2;NRIP1;SIX1;HNRNPC;RBM7;SYTL4
SHC4;LYN;SHC2;SP1;ERBB4;SWAP70;HNRNPU;PTK2B;PTPN11;EPOR
HRK;PPP2CA;MAPK10;BCLAF1;CDK1;MAPK1;BFAR;RAF1;RAD9A
LYN;PHC2;PLA2G12A;SH3KBP1;ERBB4;GAB1;PTPN11;SOS2;EPOR
CSNK1G3;HDAC5;DFFA;CSNK1A1;SRSF1;SMAD9;ESR1;KIF5C;NRIP1;E2F1;CTNNB1;RAF1;SRSF10
HDAC5;PRKAB2;RRM1;SET;ST13;HSPA4L;ETFA;NUDT5;MAT2A;CDC37;CAPZA1;CMPK1;CDK1;MYH10;PAF
GRM5;RALA;MARCKS;EEF2K;VAPA;PDE1B;MYOD1;CAMK4;ATP2B4;ATP2B1;ESR1;ADD2
ING4;CREBBP;SMURF2;E2F1;MAPK1;PTPN11;ACTN4;BTRC;KPNA1;PIAS1
SET;HNRNPK;RANBP3;APC;SERTAD2;NRIP1;PHAX;BACH1;CRK
SMURF2;LAPTM5;UBE2G1;ZBTB44;PRICKLE1;RUNX3;RNF111;SMAD7;DGCR2
SMURF2;CSNK1A1;C1QBP;IRS4;CDK1;HNRNPU;HNRNPC;CALM1;MYH10
NAPA;NCKAP1;DCTN2;MRFAP1;MTMR9;DLST;ETFA;PDHB;HK2;GLS;MTDH;GNA13;SNX1;ALDH2;C1QBP;G
NCKAP1;EIF2B2;CSNK1A1;HNRNPR;NCKIPSD;MED14;EFS;SP1;ERBB4;DAG1;ID4;PTPN4;HOXC8;SNX8;S
PFKFB3;BMPR2;ROCK1;XRCC5;TMOD3;HNRNPU;ADRA2A;MAPK10;BCLAF1;ITCH;HNRNPK;MYO1C;G3BF
HDAC5;GABRB1;GRM5;CREB1;VAPA;CAMK4;GFPT1;ACTN4;RAF1;CALM1;GRIN2B
LYN;NAPA;CAPZB;TPM3;CAPZA1;PTK2B;MAPK1;CALM1;SOD2
HDAC5;RBM28;SF3B3;CUL3;SRSF1;HNRNPU;HNRNPR;PDHB;MTDH;TIAL1;PPP2CA;NXF1;HSPH1;NKRF;C
CREBBP;TNKS2;XRCC5;MMP2;TRIM3;CDK1;CD47;L1CAM;SMC1A
LYN;CREBBP;MME;SH3KBP1;GAB3;PTPN11;EPOR;HNRNPK;EFS;CDK1;PTK2B;RBM12;RAF1
CHRM3;CREBBP;SET;USP2;HIF1A;ESR1;HIPK2;PIAS1;KAT2B;RRM2B;JMY;E2F1;PIM3
CHRM3;DDX19B;RALA;MAT2A;RUVBL1;HSPA4L;SERBP1;RAB3GAP2;SOD2;TARDBP;GLS
BMPR2;SMURF2;CSNK1A1;XIAP;SMAD7;TGFBR3;GNA13;OCLN;RAP2A;ARHGAP31;FBXO3;CTNNB1;ARL5
LYN;CUL3;CDK1;TNFAIP3;XIAP;MAPK1;CTNNB1;BFAR;PIAS1
FNBP4;MAPK10;SF3B3;CDK1;SNRPD3;HNRNPC;PDHB;RBM7;CDC42BPA;ESR1;PRKG1
MRPL42;RAP2A;SET;PSMD11;HNRNPK;MME;ZBTB16;PCDHA4;KIF1B;RAF1;CALM1;PTPN4
HDAC5;PPM1L;CDC37;RUVBL1;NRIP1;XIAP;CALM1;IL17RD;PRKG1;SMAD7;HIPK2
MAP2K1;GRM5;RALA;APC;G3BP1;CDK1;SELE;ESR1;CRK
SET;PSMD11;HSPA4L;XIAP;TNFAIP3;ETFA;NUDT5;PFAS;PCMT1;PGRMC1;RAP1A;CAPZB;MAT2A;RUVBL1
LYN;PRKAB2;PHC2;GABRB1;XRCC5;HNRNPU;SMC1A;ESR1;ENAH;ACAP2;SP1;RUVBL1;DCX;E2F1;PTK2E

FBXW4;DDX19B;CDC34;HNRNPK;CDK1;HNRNPU;FBXO3;UBXN7;CTNNB1;BTRC;RBX1;CKS1B
LYN;PLA2G12A;AGAP2;GAB1;GTF2H1;PTPN11;GRIN2B;SELE;EPOR;EFS;ERBB4;ID4;SOS2;ARHGEF5;FGF
LYN;IRS4;SPATA2;PTK2B;CTNNB1;PTPN11;ACTN4;IL6R;ESR1;EPOR;IL13RA1
ADCY9;RAP1A;HNRNPK;XK;TRIM3;SIK3;MAPK1;CTNNB1;TRAPPC8;RAF1;GRIN2B
SRPK2;NXF1;BCLAF1;SART3;SF3B3;TRA2B;SRSF1;SNIP1;ZFP91;PGAM5
RRM1;PSMD11;ARL3;ID2;PCDHA4;BMI1;ESR1;GPRASP2
NCOA2;KAT2B;SF3B3;ATXN7;SIN3A;RUVBL1;TRIM24;KPNA1
PPP2CA;MAP3K2;MAP2K1;SH3KBP1;CDK1;MAPK1;CD47;RAF1;ESR1;FOXO1;PRKG1
KAT2B;CREBBP;CBX5;XRCC5;TRIM3;GSE1;TERF2IP;ESR1;SMAD7
NCKAP1;SET;STAU1;SMURF2;GRIN2B;NCKIPSD;MAPK10;ARHGAP31;APC;ELMO1;PLXNA2;PTK2B;FRK;P
MAPK10;ITCH;EFEMP1;CDK1;MAPK1;CBFA2T2;FBLN5;GIGYF2
GRM5;PLA2G12A;ADCY9;CDK1;AGTR2;ADRA2B;ADRA2A;TSHR
PPP2CA;ACAP2;EFS;SH3KBP1;G3BP1;PTK2B;MAPK1;PTPN11;RAF1;RUNX3;ESR1;CCDC50
PSMD11;BMPR2;STAU1;RBBP5;CDK1;DLST;ESR1;TGM2
SHC4;SHC2;GAB1;IRS4;PTPN11;ESR1;GNA13;EFS;CDK1;CTNNB1;PTK2B;CRK;FGFR1
MCTS1;MAP2K1;RRM1;SET;TPM3;GFPT1;NUDT5;SOD2;PFAS;PCMT1;PGRMC1;VAPA;CAPZA1;RUVBL1;M
BCLAF1;HNRNPK;CAPRIN1;G3BP1;CDK1;HNRNPU;CTNNB1;HNRNPC;RAF1
PPP2CA;MAP2K1;RAP1A;CDK1;MAPK1;STRN;GRIN2B;JAM2;RAB11A
EPS8;NCKIPSD;PPP2CA;PTK2B;ACTN4;CD47;L1CAM;TGM2
MCTS1;SET;CSNK1A1;SOD2;HIF1A;PFAS;PAPSS2;RAB11A;TXNDC17;RBX1;PCMT1;KAT2B;PGRMC1;RAP
EPS8;FCHSD2;ZBTB16;USP2;NRIP1;SIX1;RBM7;SYTL4
NXF1;RXRA;TRIM24;SMAD9;TRIB2;RAI2;HK2;DKK3;PPIC;TNRC6B
TPM3;PARVA;ACTN4;PPP1R9A;RBX1;PPP2CA;OCLN;RAP2A;PGRMC1;HNRNPK;CAPZB;RUVBL1;MYH10;S
VAPA;SH3KBP1;CSNK1A1;C1QBP;RUVBL1;MAPK1;PTPN11;CALM1;MYH10;RNF111
RAP1A;EEF2K;RUVBL1;DPYSL3;IRS4;RC3H1;EIF4EBP2;HIF1A
HSPH1;TPM3;CDC37;CTNNB1;RAF1;CALM1;HIF1A;ESR1
ELK4;CCND3;MARCKS;HNRNPK;EEF2K;G3BP1;SERBP1;U2SURP
SNX1;ITCH;SF3B3;DCTN2;MAT2A;APC;RHOBTB3
GRM5;PLA2G12A;ADCY9;S1PR1;AGTR2;ADRA2A;TSHR
SMURF2;SH3KBP1;CALCOCO2;ZBTB16;ITPK1;USP2;SPATA2;TNFAIP3;AFF4;ITCH;NECAP2;RUVBL1;NRIP
EPS8;OCLN;PCYT1B;PGRMC1;CDH2;SMURF2;TPM3;CSNK1A1;PARVA
PCMT1;PGRMC1;SET;TPM3;CDC37;CAPZA1;RUVBL1;ETFA;SOD2
CCND3;MME;KIAA0101;XRCC5;SUB1;CDK1;CLSPN;RAD9A
CREBBP;MME;RUVBL1;EPC1;CTNNB1;PELO;ESR1;SMNDC1
CAPZB;DYRK1A;CDK1;GRIN2B;SH3GL2;PRKG1;PIAS1
NCKIPSD;CUL3;HIF1A;MYH10;ESR1;AMOT;RBX1
MAP3K2;HDAC5;CDC34;HNRNPU;BTRC;CALM1;TGM2
SART3;SF3B3;TMEM33;IGF2BP1;HNRNPU;HNRNPC;CRK;DCP2;TNRC6B
ZZZ3;MAT2A;VAPA;USP9X;PSD3;MRFAP1;DAG1;CTNNB1;CTGF
PTK2B;PTPN11;RAF1;IL6R;EPOR;TSHR;IL13RA1
HNRNPK;SRSF1;CDK1;HNRNPU;HNRNPR;HNRNPC;ESR1
DIAPH2;RAP1A;SMURF2;ROCK1;PRKG1;ARHGEF5;TGM2
SF3B3;CALCOCO2;CUL3;SRSF1;HNRNPU;HNRNPR;TIAL1;PPP2CA;NXF1;HSPH1;CAPZB;ALDH2;PABPN1;
RBM3;CTNNB1;ACTN4;SELE;EIF4E;SORBS3
TCERG1;APC;DPYSL5;RAF1;ESR1;TSHR
MAP3K2;SMURF2;CDC37;CDK1;C10ORF2;MAPK1;WDFY3;DIP2B
PSMD11;CRBN;CUL3;BTBD9;DTL;RBX1;CKS1B
MED14;RUVBL1;TARDBP;AFF4;U2SURP;MED17
MAP3K2;CREBBP;SET;NECAB3;TRIM41;IRS4;HNRNPU;TNFAIP3;SENP2;DDX19B;ZZZ3;MYO1C;CDC37;CA
IGBP1;MYO1C;XPO4;TMEM33;SLC25A12;MAPRE2
XRCC5;RUVBL1;HNRNPR;HNRNPC;CALM1;SOD2;ESR1;PRKG1
PCMT1;TMEM33;CDK1;PTPN11;MYH10;PFAS;PAFAH1B2;TXNDC17
ADAM19;SMURF2;DTX1;XIAP;BMI1;TARDBP;TGM2;SMAD7

MYO1C;TMEM33;FNTB;LTN1;RAB3GAP2;PSPH
PPP2CA;IGBP1;EIF2B2;CUL3;STRN;RAF1;PAFAH1B2;DCP2;CDC73
MME;RUVBL1;HNRNPU;EPC1;CTNNB1;ESR1;APPL1
USP37;RANBP3;ST13;PLAG1;ZNF292;SCN5A;SORBS3
SET;HSPH1;CDK1;CTNNB1;BTRC;UBE2J1
TCERG1;SWAP70;CNBP;CDK1;HNRNPU;ESR1
CHRM3;RALA;HNRNPK;PSD3;EPT1;BACH1
MAPK10;SET;NECAB3;RAP1A;GPC1;CDK1;XIAP;APBB2;ATP2B2;RAB11A
RRM1;PSMD11;ARL3;ID2;PCDHA4;BMI1
DIAPH2;STAU1;ROCK1;ST13;ETFA;SRGAP2;CDC42BPA;METAP2
CREB1;RBL1;APC;SIN3A;SUB1
CREBBP;RPA4;TRIM24;CDK1;FAM107B;RAD9A
STAU1;CDC37;C1QBP;RUVBL1;HNRNPU;CALM1
NDC1;EPT1;GRIN2B;FGFR1
HDAC5;PSMD11;SMURF2;PTPN4
KCMF1;LYN;EFS;EPOR
DCTN2;TRPS1;SMAD9;CDC73
NDC1;ALG6;ERI1;EPT1;NSG1;SEC62
PPP2CA;IGBP1;STRN;CDC73
CREB1;ROCK1;FUBP1;SERBP1;CDK1;PRKG1
SH3KBP1;SIN3A;MAPK1
NDC1;ALG6;EPT1;NSG1
NDC1;ALG6;EPT1;NSG1;SEC62
MYOD1;SIX1;CTNNB1;CBFA2T2
PPP2CA;IGBP1;CDC34;ARL3;STRN
OTUD4;CDC37;SERBP1;WDFY3
CNBP;RASSF8;ESR1;SMNDC1
HAUS3;CTNNB1;CLSPN;PRKG1
ESR1;GRIN2B
EPT1;SEC62
ALG6;EPT1;SEC62
XPO4;RAB3GAP2
UTP15;NDC1
DNMT3A;RUNX3
FNBP4;STAU1;RASSF8;ESR1
CPSF7;ATXN1L;WDFY3;CRK
PHC2;GTF2H1
UTP15;SEC62
NAPB;ADK
PPP2CA;IGBP1;CDC34;CUL3;STRN
ALG6;EPT1
TCERG1;SMC1A
RUVBL1;RAF1
UTP15;PDCD4;RASSF8;ESR1
ARL3;SEC62
CDC34;CDC73
CDC37;ESR1
PPP2CA;IGBP1;CUL3;STRN
HNRNPK;MAPK1;ESR1
UTP15;ALG14
LSM5;DCP2
CDC73;DCP2
CRP;PPIC

NDC1
PPIC
CRP
NSG1
ALG6;NSG1
DCP2
SOD2
UTP15
ADK
HNRNPK;LSM5
ADK
EPT1
PPIC
DCP2
SEC62;CDC73
UTP15
CDK1;SOD2
CDC34
UTP15;CDC37
LSM5
CDC34;LSM5;CDC73
RAF1
VPS54
CRP
CDC37
DCP2
CDC34
UTP15
CDC34;EPT1;LSM5
SOD2;CDC73
UTP15
CDC34
RAF1
ESR1
PPIC
CDC34
PGAM5
CRP
NDC1
UTP15;NSG1
UTP15;CDC73
CDC37
SEC62
CDC37
CDC34;SUB1;CDC73
GAB1
PPIC
SOD2
UTP15;COA1;ARL3;CUL3;NPR3;CMC1;GAB1;EPT1;IRS4;ALG14;SOD2;LSM5;CDC73;CDC34;DDI2;CDC37;S
UTP15;ARL3;CUL3;CMC1;EPT1;ALG14;SOD2;CDC73;CDC34;DDI2;CDC37;SUB1;VPS54;DCP2
UTP15;ARL3;CUL3;MRPL27;GAB1;EPT1;IRS4;ALG14;NSG1;CDC73;CDC34;DDI2;SUB1;VPS54;DCP2
CDC34;COA1;ARL3;DDI2;SUB1;CMC1;EPT1;ALG14;VPS54;CDC73
CDC34;DDI2;CDC37;SUB1;VPS54;RSF1

RAF1
CDC37;EPT1;CDC73
PDHB;IL18BP
CDC34;CDC37;LSM5;CDC73
CDC34;COA1;CDC73
NDC1
IRS4;FMN1;CDC73
CDC34;ARL3;CDC37;LSM5;CDC73
CDC37;CUL3;CDC73
CDC34;CDC37;ALG14
CDC34;ARL3;SUB1;CDC73
ARL3;CDC37;CDC73
NDC1;UTP15
UTP15;CDC73;DCP2
UTP15;CDC73
CDC73
EPT1

RNF111;RAB11FIP5;TGM2;CREBBP;SMURF2;USP9X;TPM3;ZBTB16;DYRK1A;SMAD9;RUNX3;ESR1;RU
1;N4BP2;ABCA1;LYN;NCOA2;CREBBP;EBF1;PAX5;PROX1;DEK;ESR1;RUNX1;HIPK2;PIAS1;MAPK10;K
CDH2;ERBB4;PTK2B;MAPK1;CTNNB1;BSN;RAF1;CALM1;MYH10;APPL1
2;RUNX3;RUNX1;KAT2B;MYOD1;PGAM5;RAF1;HDAC5;USP15;INSIG2;UBA6;RNF180;DLST;XIAP;HIF1A
K1;PCYT1B;CXADR;CBFA2T2;ADRA2A;RUNX1;CREB1;RBL1;SNPH;DCX;TERF2IP;DCTN2;TNKS;YTHDC
3;PCDHA7;AP2M1;TNS1;PCDHA6;GAB1;PTPN11;PCDHA12;PCDHA11;PCDHA10;MAPK10;SP1;ID4;ELM
P;XRCC5;ZBTB16;GTF2H1;PAX5;ESR1;RUNX1;PIAS1;KAT2B;SP1;CDC37;ZNF318;CDK1;CTNNB1;CALM
MEF2A;RAB2B;CREBBP;USP9X;SMAD9;ESR1;FBXO30;PIAS1;SMAD7;OCLN;RBL1;SP1;MYOD1;CTNNB
1;SNPH;CDC37;MYOD1;DCX;TFAM;TERF2IP;RAPGEF6;DTL;NUFIP2;YTHDC1;ADD3;GIGYF2;FOXO1;AD
F2A;NCOA2;CREBBP;MAP2K1;ZBTB16;PAX5;PROX1;DEK;RUNX3;ESR1;RUNX1;HIPK2;SMAD7;KAT2B
;LYN;NCOA2;MAP2K1;PLA2G12A;RANBP3;GAB1;PTPN11;GRIN2B;SORBS3;ESR1;GATAD2B;EPOR;RU
R;MAPK1;CTNNB1;ZNF148
AP2A;MECOM;SNIP1;MAPK1;ZNF423;HOXC8;SNX6;BMPR1A
NF111;TGM2;MEF2A;CREBBP;CPSF7;SMURF2;ST13;USP9X;SMAD9;SENP2;RUNX3;ESR1;GATAD2B;R
F1;TERF2IP;MAPK1;CTNNB1;ZNF423;TARDBP
;APPL1;NCOA2;CSNK1G3;CREBBP;CSNK1A1;USP9X;PTPN11;ACTN4;GRIN2B;ESR1;RBX1;SMAD7;KA
2M1;DFFA;MAP2K1;TPM3;SGIP1;TMOD2;DYRK1A;ACSL6;ACTN4;GAPVD1;PGAM5;SLC25A12;RAF1;MA
RNPK;RXRA;NFIA;SNPH;MYOD1;CDK1;CCDC6;KIF1B;CRK
2;SIN3A;SUB1;SP4;MYOD1;E2F1;S1PR1;MAPK1;CRK
ERBB4;DAG1;PTK2B;MAPK1;SOS2;CRK;AP2M1;FGFR1
AF;CREB1;SP1;SIN3A;TDG;TRIM24;HIVEP2;SOX6;ZNF687
A;TBL1XR1;SP1;SIN3A;NRIP1;LCOR;ZNF148
K2B;HOXC8;IL6R;PPARGC1B;TGM2;LYN;MAP3K2;GABRA1;CREBBP;GAB1;ATP2B4;PTPN11;GAB3;L1C
APZB;CTNNB1;PTK2B;MAPK1;BSN;CALM1;RAF1;SLC25A12;MYH10;AP2M1;APPL1
MYOD1;LCOR;PGAM5;GLYR1;HDAC5;STAU1;SRSF1;HIF1A;FOXO1;MTDH;BCLAF1;RXRA;RBBP5;NSD
PP1R1B;PLXNA2;CDK1;DCX;CTNNB1;PLXNA3
;S1PR1;MAPK1;BSN;RBM7;SRSF10;MARK1;FNBP4;ABCA1;CREBBP;MAP2K1;RANBP3;GRIN2B;ESR1;
01;CARM1;MAPK1
APK1;SLIT2;EIF4E;PELO;UTP15;TFAP2B;CREBBP;MAP2K1;USP9X;XRCC5;DNMT3A;SMC1A;ESR1;U2S
A1;LYN;PCYT1B;TRIM41;GFPT1;NFATC3;PTPN11;GRIN2B;OCLN;MARCKS;CREB1;APC;SP4;PPP1R1B;
;MYH10;RNF111;FNBP4;MEF2A;ABCA1;LYN;CREBBP;MAP2K1;CBX5;MME;PDAP1;L1CAM;ESR1;PIAS1
MYOD1;CDK1;E2F1;MAPK1;RAF1;FRK;TGM2
B;CDK1;DCX;CCDC6;MAPK1;CALM1;PHACTR4
F2K;UBXN7;MAPK1;BTRC;TEAD1;TGM2;ABCA1;GABRA1;CREBBP;CSNK1A1;GFPT1;ATP2B1;ESR1;MA
OD1;TDG;NRIP1;CTNNB1;MAPK1;MAPRE3;HOXC8;NFE2L1
XR1;C1QBP;PTK2B;MAPK1;RAF1;JAM2
BTB16;DNMT3A;RUNX3;ESR1;GATAD2B;RUNX1;SMAD7;MAPK10;CREB1;RBL1;SP1;MYOD1;CCDC6;C
I2;FGFR1
APK1;ZNF148;CSNK1G3;NCOA2;CREBBP;CSNK1A1;DYRK1A;GTF2H1;HIPK1;S100B;ESR1;GTPBP4;HIF
32;BSN;MEF2A;MAP3K2;MAP2K1;ESR1;PAX2;ITCH;HNRNPK;NFIA;SP1;FUBP1;PPP1R1B;CDK1;DCX;CT
4;MYOD1;ATXN1L
TRC;CALM1;MYH10;TRIM44
NB1;HNRNPC
B1;TDG;RUVBL1;SNIP1;HNRNPC;UBE2K;RNF111;LNX2
2B;SCN5A;HOXC8;SNX8;PLXNA3;FNBP4;LYN;C21ORF59;PTPN11;GAB3;GRIN2B;RUNX3;ITCH;EFNA3;
1;CALM1;RAF1;CRK;SNX6;FGFR1
3;RUVBL1;CEP170;HIVEP2;SRGAP2;KPNA1;TNS1;MARK1;MAP3K2;TSFM;DFFA;MAP2K1;CSNK1A1;MS
2;E2F1;S1PR1;MAPK1;EIF4E;JAM2;PRKG1;LYN;GABRA1;CREBBP;TRIM41;XRCC5;MMP2;GFPT1;PTPN
CLN;CDC34;XK;SP1;CDC37;PPP1R1B;SERBP1;CDK1;CTNNB1;HNRNPC;CALM1;KCTD16
G3BP1;CTNNB1;KIF1B;HNRNPC;BSN;AP2M1
;N4BP2;RAPGEF6
3K2;NCOA2;CREBBP;MAP2K1;PLA2G12A;GAB1;L1CAM;GRIN2B;SORBS3;ESR1;EPOR;RUNX1;RCAN1

CN5A;SH3GL2;IL13RA1;LYN;SEPT11;ST13;TPM3;TXNL1;TMOD2;ATP2B4;ATP2B2;ACTN4;ATP2B1;GRIN
P1;SIN3A;ZNF318;NRIP1;MXD1;APPL1
;SRSF10;ARHGEF5;MARK1
;CDK1;E2F1;KIF1B;CLSPN;KPNA1
QBP;TRA2B;CEP170;KIF1B;HIVEP2;SRGAP2;SRSF10;MYH10;RAB11FIP5;MARK1;MAP3K2;DFFA;RRM
EBBP;PTPN11;SORBS3;ESR1;ENAH;ACAP2;CREB1;SP1;MYOD1;CDK1;CTNNB1;PTPN4;SOS2;CRK;R
ARGC1B;APPL1;SRPK2;CREBBP;RANBP3;ESR1;PIAS1;SMAD7;CREB1;CDC37;PDCD4;CTNNB1;PGAM
P1;KIF1B;RAF1;SRGAP2;MARK1
MAPRE2;NUPL1;PRKG1
K;PPP1R1B;DCX;CTNNB1;RAPGEF6;CRK;MARK1
B;MAPK1;RAF1;JAM2

CAMK4;CARM1;RUVBL1;SNIP1;MAPK1;BTRC;CALM1;MYH10;TGM2
RUVBL1;G3BP1;IGF2BP1;MLEC;BTRC;MYH10;RRM1;ST13;TPM3;SOD2;ESR1;RAB11A;TMEM33;CDC3
;ESR1;EPOR;TSHR;ADAM12;ID4;CTNNB1;PTPN4;SOS2;CRK;FGFR1

K2;MAP2K1;XRCC5;TMOD3;TGFBR3;MAPK10;ITCH;HNRNPK;MYO1C;CAPZA1;SERBP1;HNRNPC;CAL
I;CTNNB1;FRK
NCOA2;CREBBP;ZFP91;HCFC2;SMC1A;ESR1;GTPBP4;PIAS1;MYO1C;SP1;MYOD1;SERBP1;CTNNB1;C

HNRNPC;KPNA1
RKG1;IL13RA1
P;CAPZA1;RUVBL1;PCBD2;CALM1;MYH10
K
ALM1;MYH10
IC1;CAPZB;CAPZA1;CMPK1;TFAM;MLEC;STRN;MYH10;PAFAH1B2
APK1;CALM1;SSBP2;SMNDC1;PRTFDC1
YT5;SEPT11;WDR37;LAPTM5;HOOK3;ATP2B1;GRIN2B;ESR1;MARCKS;FUBP1;CAMK4;MYOD1;STRN;C
M7;TARDBP;PSPH

NNB1;BSN;ZNF148;RAD9A

FGFR1
A;C1QBP;RUVBL1;IGF2BP1;SNRPD3;SRSF10;TRIM41;YLPM1;HNRNPK;SERBP1;RAB3GAP2;HNRNPC
H3GL2;APPL1;SNX6;LYN;MAP2K1;USP9X;GAB1;PTPN11;ESR1;EREG;ENAH;HNRNPK;VAPA;CDK1;CT
4;STRN;UBE2K
;RAF1
ALM1
TNNB1;PTK2B;MAPK1;WDFY3;CD47;CALM1;SLC25A12;MYH10;AP2M1;MARK1

1;ZNF148;FNBP4;MEF2A;NCOA2;MAP2K1;YLPM1;SLC4A10;CBFA2T2;SORBS3;ESR1;GATAD2B;RUNX

;GAB1;GAB3;ACTN4;ADAM12;SOS2;CRK;SHC4;SHC2;LUZP1;DCTN2;DLST;DTX1;NCKIPSD;BCLAF1;E

ESR1;MARCKS;HNRNPK;CAPZB;CAPZA1;CARM1;RUVBL1;CALM1;MYH10;TGM2;PHACTR4
M1;RAF1;RAD9A

RAF1;MYH10;MARK1

;RBBP5;TRA2B;PLXNA2;E2F1;KIF1B;APPL1;MEF2A;LYN;MAP2K1;CXADR;ZBTB16;TMOD3;NFATC3;GA

1QBP;CARM1;SERBP1;IGF2BP1;WDFY3;SNRPD3;HNRNPC;TECPR2;TNRC6B

RAP1A;CAPZB;PABPN1;C1QBP;G3BP1;IGF2BP1;SNRPD3;SRSF10;RAB11FIP5;ST13;XRCC5;SMC1A;G

FKBP6

TRC;CALM1;MYH10;UBE2K;KPNA1;TNRC6B

FAH1B2

GPC1;TRA2B;DPYSL3;RUVBL1;MLEC;MAPK1;PTGFRN;EIF4E;AP2M1;RAB2B;HSDL1;ST13;SORT1;TXNI
OS2;CRK
P1;MAPK1;CALM1;RAF1;RAB11FIP5

C1QBP;G3BP1;IGF2BP1;SNRPD3;SRSF10;GABRA1;XRCC5;U2SURP;HNRNPK;SERBP1;RAB3GAP2;WD

5A;SNX6

;G3BP1;MLEC;EIF4E;MAP2K1;SEPT11;ST13;IL17RD;RAB11A;RUNX1;RCAN1;RNF126;MARCKS;ITCH;C
3;BSN;RAF1;RAD9A

FR1

LXNA3;METAP2

APK1;TARDBP;CRK

1A;SP1;UBXN7;CALM1;UBE2K

SH3GL2

1;CALM1;APPL1

;C1QBP;G3BP1;IGF2BP1;SNRPD3;YLPM1;U2SURP;HNRNPK;CARM1;SERBP1;HNRNPC;TECPR2;TNR

PZA1;CALM1;MYH10

;UB1;VPS54;DCP2

NX1;HIPK2;RBX1;SMAD7;KAT2B;HEYL;ITCH;RBL1;SP1;MYOD1;SIK3;CTNNB1;SLC25A12;CRK
AT2B;MAF;CREB1;NFIA;APC;SP1;CAMK4;MYOD1;CARM1;CTNNB1;FGFR1

\;FOXO1;LRP6;ERBB4;USP1;E2F1;MAPK1;EIF4E;MARK1;CREBBP;SMURF2;ZBTB16;LAPTM5;GRIN2B;
C1;CUL3;FUT11;GSKIP;ADD3;GIGYF2;FOXO1;LRP6;ADD2;BCLAF1;RXRA;ATXN7;TRA2B;IGF2BP1;E2F
IO1;SOS2;FGFR1
11;RAF1;RAD9A
1
DD2;PPP2CA;NPAT;BCLAF1;RXRA;ATXN7;TRA2B;IGF2BP1;E2F1;GSE1;MAPK1;MARK1;LYN;CREBBP;S
;MAF;CREB1;SP1;SUB1;CAMK4;MYOD1;CARM1;JMY;CTNNB1
INX1;RCAN1;ARHGAP31;HNRNPK;SP1;ID2;CCDC6;RAF1;METAP2

UNX1;HIPK2;SMAD7;KAT2B;ITCH;RBL1;SP1;SIK3;CTNNB1;CALM1;TNRC6B

T2B;APC;TBL1XR1;CARM1;SERBP1;CTNNB1;FGFR1;TNRC6B
APRE2;NAPA;NAPB;HDAC5;GPM6A;RALA;DCTN2;PRUNE2;AGAP2;NRXN3;DLST;PDHB;ADD3;ADD2;M

AM;CDC42BPA;GRIN2B;RUNX3;ESR1;OCLN;HNRNPK;SP1;CDC37;FUBP1;CARM1;ADAM12;CDK1;ID4

1;TDG;G3BP1;NRIP1;MAPK1;PPARGC1B;SRPK2;CREBBP;XRCC5;ZBTB16;GTF2H1;DEK;ESR1;U2SU

PUM2;CREB1;SNPH;CDK1;DCX;TRIP12;RAF1;FGFR1

URP;MAPK10;KAT2B;GLUD2;HNRNPK;CREB1;RBL1;TMEM33;SP1;CARM1;CDK1;CTNNB1;HNRNPC;S
CDK1;DCX;CTNNB1;RAF1
;MARCKS;OCLN;ITCH;HNRNPK;CDC34;XK;CREB1;APC;SP1;CDC37;SUB1;CAPZA1;PPP1R1B;CDK1;C

RCKS;ITCH;ADCY9;CREB1;APC;PPP1R1B;SIK3;DCX;CTNNB1;PDE5A;RAF1;CRK

TNNB1;LCOR;MXD1;RAD9A

PK2;PIAS1;MAPK10;KAT2B;ITCH;CREB1;RRM2B;SP1;HECW2;CARM1;SERBP1;JMY;CDK1;RAF1;UBE2
TNNB1;CRK

;HNRNPK;SP1;CDK1;CTNNB1;PTPN4;RAF1;SOS2;CRK;CD244

L2;ACTN4;GRIN2B;ADRA2B;ADRA2A;HNRNPK;RBL1;APC;SIK3;CMPK1;CTNNB1;HNRNPC;CALM1;RA
11;ATP2B2;ATP2B1;L1CAM;SMC1A;GRIN2B;ESR1;ADRA2A;ENAH;MARCKS;OCLN;XK;RBL1;MYOD1;S

1;MARCKS;RBL1;SP1;ID2;RAF1;NUPL1;METAP2

N2B;DYNLL2;APC;PGAM5;SLC25A12;CALM1

1;DYRK1A;MSL2;RASSF8;GRIN2B;GTPBP4;PGAM5;RAF1;RAPGEF6
AD9A
M5;SIK2;RAF1;MXD1

7;CAPZA1;SERBP1;EIF3J;TFAM;HNRNPC;CALM1;UBE2K

LM1;RAF1

CALM1

CALM1

C;TARDBP;SRSF9;TNRC6B
NNB1;CALM1;SOS2;CRK;FGFR1

1;SMAD7;RCAN1;CREB1;RBL1;KANSL1;SP1;FUBP1;ID2;TERF2IP;KCTD16;FGFR1

FS;ERBB4;PTK2B;MAPK1;HOXC8;SRSF10;SNX8;MYH10;TFAP2B;PTPN11;HELZ;ESR1;EPOR;HNRNPK

\PVD1;CBFA2T2;ESR1;U2SURP;RUNX1;MAPK10;MARCKS;HNRNPK;FUBP1;ID2;MYOD1;PPP1R1B;CDI

GTPBP4;RAP2A;HNRNPK;CARM1;SERBP1;HNRNPC;TARDBP;TECPR2;TNRC6B

L1;LNPEP;PARVA;ACTN4;SOD2;ALDH6A1;HNRNPK;CAPRIN1;CTNNB1;PAFAH1B2

DFY3;HNRNPC;TECPR2;TNRC6B

CDC37;CAPZA1;HNRNPC;CALM1;PAFAH1B2

C6B

;ESR1;EPOR;SMAD7;RCAN1;ITCH;HNRNPK;APC;SP1;SP4;CDK1;PDCD4;CTNNB1;TARDBP;FGFR1
1;GSE1;MAPK1;GFPT1;NFATC3;YLPM1;SLC4A10;HELZ;GRIN2B;SORBS3;ESR1;U2SURP;RCAN1;C20C

ЗORT1;ZBTB16;GFPT1;NFATC3;SORBS3;ESR1;GATAD2B;U2SURP;PUM2;RCAN1;MARCKS;HNRNPK;A

TDH;NCKIPSD;AP3M2;PPP2CA;PURA;PGRMC1;HSPH1;PLXNA2;S1PR1;MYH10;MAP3K2;GABRA1;SEF

;CTNNB1;RAF1;SOS2;CRK;FGFR1

RP;HNRNPK;MYO1C;SP1;CAPRIN1;RLIM;CDK1;CTNNB1;STRN;TRIP12;CALM1

LC25A12;RAF1;MXD1

CTNNB1;HNRNPC;CALM1;TARDBP;RAD9A

K

F1
ERBP1;CALM1;RAF1

;MYO1C;SP1;ID4;SPRY3;SPRY1;HNRNPC;PTPN4;FGFR1

K1;DCX;RAD9A

)RF112;MARCKS;ARHGAP31;NFIA;APC;KANSL1;FUBP1;ID2;PPP1R1B;CCDC6;CTNNB1

\PC;KANSL1;SP1;ID2;PPP1R1B;CCDC6;CTNNB1

'T11;CSNK1A1;ATP2B4;SLC4A10;ATP2B2;ATP2B1;L1CAM;GRIN2B;SYT7;STXBP5L;LETM1;VAPA;VAPB;

;CTNNB1
