# Supplementary material for: Circulating miR-330-3p in Late Pregnancy is Associated with Pregnancy Outcomes Among Lean Women with GDM
Source: Sci Rep. 2020 Jan 22;10:908. doi: 10.1038/s41598-020-57838-6 (PMC6976655; doi:10.1038/s41598-020-57838-6)
Supplement: Supplementary file 1 — Supplementary data. [file 41598_2020_57838_MOESM1_ESM.zip › Supplimentary File_EnrichR_Analysis Reactome_2016.pdf]

Term
YAP1- and WWTR1 (TAZ)-stimulated gene expression_Homo sapiens_R-HSA-2032785
Developmental Biology_Homo sapiens_R-HSA-1266738
Signaling by WNT in cancer_Homo sapiens_R-HSA-4791275
Signaling by NOTCH1_Homo sapiens_R-HSA-1980143
Axon guidance_Homo sapiens_R-HSA-422475
Nuclear signaling by ERBB4_Homo sapiens_R-HSA-1251985
Signaling by Wnt_Homo sapiens_R-HSA-195721
Signaling by NOTCH_Homo sapiens_R-HSA-157118
TCF dependent signaling in response to WNT_Homo sapiens_R-HSA-201681
Diseases of signal transduction_Homo sapiens_R-HSA-5663202
S33 mutants of beta-catenin aren't phosphorylated_Homo sapiens_R-HSA-5358747
S45 mutants of beta-catenin aren't phosphorylated_Homo sapiens_R-HSA-5358751
phosphorylation site mutants of CTNNB1 are not targeted to the proteasome by the destruction complex_Homo
T41 mutants of beta-catenin aren't phosphorylated_Homo sapiens_R-HSA-5358752
S37 mutants of beta-catenin aren't phosphorylated_Homo sapiens_R-HSA-5358749
Misspliced GSK3beta mutants stabilize beta-catenin_Homo sapiens_R-HSA-5339716
Retrograde transport at the Trans-Golgi-Network_Homo sapiens_R-HSA-6811440
Mitochondrial biogenesis_Homo sapiens_R-HSA-1592230
Disassembly of the destruction complex and recruitment of AXIN to the membrane_Homo sapiens_R-HSA-464
Transcriptional activation of mitochondrial biogenesis_Homo sapiens_R-HSA-2151201
CRMPs in Sema3A signaling_Homo sapiens_R-HSA-399956
MAPK3 (ERK1) activation_Homo sapiens_R-HSA-110056
BMAL1:CLOCK,NPAS2 activates circadian gene expression_Homo sapiens_R-HSA-1368108
Beta-catenin phosphorylation cascade_Homo sapiens_R-HSA-196299
Signaling by EGFR_Homo sapiens_R-HSA-177929
Post NMDA receptor activation events_Homo sapiens_R-HSA-438064
NGF signalling via TRKA from the plasma membrane_Homo sapiens_R-HSA-187037
Antigen processing: Ubiquitination & Proteasome degradation_Homo sapiens_R-HSA-983168
CREB phosphorylation through the activation of Ras_Homo sapiens_R-HSA-442742
RORA activates gene expression_Homo sapiens_R-HSA-1368082
Constitutive Signaling by NOTCH1 HD+PEST Domain Mutants_Homo sapiens_R-HSA-2894862
Signaling by NOTCH1 in Cancer_Homo sapiens_R-HSA-2644603
Signaling by NOTCH1 PEST Domain Mutants in Cancer_Homo sapiens_R-HSA-2644602
Constitutive Signaling by NOTCH1 PEST Domain Mutants_Homo sapiens_R-HSA-2644606
Signaling by NOTCH1 HD+PEST Domain Mutants in Cancer_Homo sapiens_R-HSA-2894858
Negative feedback regulation of MAPK pathway_Homo sapiens_R-HSA-5674499
Reduction of cytosolic Ca++ levels_Homo sapiens_R-HSA-418359
Physiological factors_Homo sapiens_R-HSA-5578768
NOTCH1 Intracellular Domain Regulates Transcription_Homo sapiens_R-HSA-2122947
Ca-dependent events_Homo sapiens_R-HSA-111996
MAPK family signaling cascades_Homo sapiens_R-HSA-5683057
Activation of NMDA receptor upon glutamate binding and postsynaptic events_Homo sapiens_R-HSA-442755
Signaling by ERBB4_Homo sapiens_R-HSA-1236394
L1CAM interactions_Homo sapiens_R-HSA-373760
AMER1 mutants destabilize the destruction complex_Homo sapiens_R-HSA-4839748
Truncations of AMER1 destabilize the destruction complex_Homo sapiens_R-HSA-5467348
truncated APC mutants destabilize the destruction complex_Homo sapiens_R-HSA-4839744
AXIN missense mutants destabilize the destruction complex_Homo sapiens_R-HSA-5467340
AXIN mutants destabilize the destruction complex, activating WNT signaling_Homo sapiens_R-HSA-4839735
APC truncation mutants have impaired AXIN binding_Homo sapiens_R-HSA-5467337
Circadian Clock_Homo sapiens_R-HSA-400253
Platelet homeostasis_Homo sapiens_R-HSA-418346

Intra-Golgi and retrograde Golgi-to-ER traffic_Homo sapiens_R-HSA-6811442
Activation of gene expression by SREBF (SREBP)_Homo sapiens_R-HSA-2426168
RAF-independent MAPK1/3 activation_Homo sapiens_R-HSA-112409
Signaling by BMP_Homo sapiens_R-HSA-201451
Chromatin organization_Homo sapiens_R-HSA-4839726
Chromatin modifying enzymes_Homo sapiens_R-HSA-3247509
Regulation of lipid metabolism by Peroxisome proliferator-activated receptor alpha (PPARalpha)_Homo sapiens
Regulation of cholesterol biosynthesis by SREBP (SREBF)_Homo sapiens_R-HSA-1655829
PI3K events in ERBB2 signaling_Homo sapiens_R-HSA-1963642
Class I MHC mediated antigen processing & presentation_Homo sapiens_R-HSA-983169
MAPK1 (ERK2) activation_Homo sapiens_R-HSA-112411
Inactivation of Cdc42 and Rac_Homo sapiens_R-HSA-428543
Recycling pathway of L1_Homo sapiens_R-HSA-437239
CaM pathway_Homo sapiens_R-HSA-111997
Calmodulin induced events_Homo sapiens_R-HSA-111933
PI-3K cascade:FGFR1_Homo sapiens_R-HSA-5654689
PI-3K cascade:FGFR3_Homo sapiens_R-HSA-5654710
PI3K events in ERBB4 signaling_Homo sapiens_R-HSA-1250342
PIP3 activates AKT signaling_Homo sapiens_R-HSA-1257604
PI-3K cascade:FGFR4_Homo sapiens_R-HSA-5654720
PI-3K cascade:FGFR2_Homo sapiens_R-HSA-5654695
Signalling by NGF_Homo sapiens_R-HSA-166520
Downstream signal transduction_Homo sapiens_R-HSA-186763
Downstream signaling of activated FGFR2_Homo sapiens_R-HSA-5654696
Downstream signaling of activated FGFR4_Homo sapiens_R-HSA-5654716
Downstream signaling of activated FGFR3_Homo sapiens_R-HSA-5654708
cGMP effects_Homo sapiens_R-HSA-418457
GAB1 signalosome_Homo sapiens_R-HSA-180292
PI3K/AKT activation_Homo sapiens_R-HSA-198203
Synthesis of pyrophosphates in the cytosol_Homo sapiens_R-HSA-1855167
Signaling by FGFR4_Homo sapiens_R-HSA-5654743
Downstream signaling of activated FGFR1_Homo sapiens_R-HSA-5654687
PPARA activates gene expression_Homo sapiens_R-HSA-1989781
Signaling by PDGF_Homo sapiens_R-HSA-186797
Signaling by FGFR3_Homo sapiens_R-HSA-5654741
IGF1R signaling cascade_Homo sapiens_R-HSA-2428924
Signaling by Type 1 Insulin-like Growth Factor 1 Receptor (IGF1R)_Homo sapiens_R-HSA-2404192
IRS-related events triggered by IGF1R_Homo sapiens_R-HSA-2428928
Apoptotic execution phase_Homo sapiens_R-HSA-75153
Signaling by FGFR1_Homo sapiens_R-HSA-5654736
Uptake and function of anthrax toxins_Homo sapiens_R-HSA-5210891
Activated NOTCH1 Transmits Signal to the Nucleus_Homo sapiens_R-HSA-2122948
MAPK targets/ Nuclear events mediated by MAP kinases_Homo sapiens_R-HSA-450282
Pre-NOTCH Transcription and Translation_Homo sapiens_R-HSA-1912408
Deactivation of the beta-catenin transactivating complex_Homo sapiens_R-HSA-3769402
Transcriptional regulation of white adipocyte differentiation_Homo sapiens_R-HSA-381340
DAP12 signaling_Homo sapiens_R-HSA-2424491
Signal transduction by L1_Homo sapiens_R-HSA-445144
Regulation of FZD by ubiquitination_Homo sapiens_R-HSA-4641263
Signaling by FGFR2_Homo sapiens_R-HSA-5654738
mRNA Splicing - Major Pathway_Homo sapiens_R-HSA-72163
IRS-mediated signalling_Homo sapiens_R-HSA-112399
HATs acetylate histones_Homo sapiens_R-HSA-3214847

Processing of Capped Intron-Containing Pre-mRNA_Homo sapiens_R-HSA-72203
Oncogene Induced Senescence_Homo sapiens_R-HSA-2559585
Signaling by Robo receptor_Homo sapiens_R-HSA-376176
DAG and IP3 signaling_Homo sapiens_R-HSA-1489509
LRR FLII-interacting protein 1 (LRRFIP1) activates type I IFN production_Homo sapiens_R-HSA-3134973
Adaptive Immune System_Homo sapiens_R-HSA-1280218
Transcriptional activity of SMAD2/SMAD3:SMAD4 heterotrimer_Homo sapiens_R-HSA-2173793
PLC beta mediated events_Homo sapiens_R-HSA-112043
Insulin receptor signalling cascade_Homo sapiens_R-HSA-74751
Signaling by FGFR_Homo sapiens_R-HSA-190236
MAPK1/MAPK3 signaling_Homo sapiens_R-HSA-5684996
NICD traffics to nucleus_Homo sapiens_R-HSA-157052
Notch-HLH transcription pathway_Homo sapiens_R-HSA-350054
ERBB2 Activates PTK6 Signaling_Homo sapiens_R-HSA-8847993
Advanced glycosylation endproduct receptor signaling_Homo sapiens_R-HSA-879415
Signal regulatory protein (SIRP) family interactions_Homo sapiens_R-HSA-391160
GABA A receptor activation_Homo sapiens_R-HSA-977441
G-protein mediated events_Homo sapiens_R-HSA-112040
EGFR interacts with phospholipase C-gamma_Homo sapiens_R-HSA-212718
PLC-gamma1 signalling_Homo sapiens_R-HSA-167021
Membrane Trafficking_Homo sapiens_R-HSA-199991
Hemostasis_Homo sapiens_R-HSA-109582
Signaling by SCF-KIT_Homo sapiens_R-HSA-1433557
TRAF6 Mediated Induction of proinflammatory cytokines_Homo sapiens_R-HSA-168180
SEMA3A-Plexin repulsion signaling by inhibiting Integrin adhesion_Homo sapiens_R-HSA-399955
Activation of Rac_Homo sapiens_R-HSA-428540
Regulation of pyruvate dehydrogenase (PDH) complex_Homo sapiens_R-HSA-204174
DAP12 interactions_Homo sapiens_R-HSA-2172127
Nuclear Events (kinase and transcription factor activation)_Homo sapiens_R-HSA-198725
Phospholipase C-mediated cascade: FGFR1_Homo sapiens_R-HSA-5654219
Translocation of GLUT4 to the plasma membrane_Homo sapiens_R-HSA-1445148
MAP kinase activation in TLR cascade_Homo sapiens_R-HSA-450294
RSK activation_Homo sapiens_R-HSA-444257
mRNA Splicing_Homo sapiens_R-HSA-72172
EPH-ephrin mediated repulsion of cells_Homo sapiens_R-HSA-3928665
Fc epsilon receptor (FCERI) signaling_Homo sapiens_R-HSA-2454202
Ca2+ pathway_Homo sapiens_R-HSA-4086398
G0 and Early G1_Homo sapiens_R-HSA-1538133
Nitric oxide stimulates guanylate cyclase_Homo sapiens_R-HSA-392154
Prolactin receptor signaling_Homo sapiens_R-HSA-1170546
ERBB2 Regulates Cell Motility_Homo sapiens_R-HSA-6785631
CREB phosphorylation through the activation of CaMKII_Homo sapiens_R-HSA-442729
Apoptotic cleavage of cellular proteins_Homo sapiens_R-HSA-111465
Role of LAT2/NTAL/LAB on calcium mobilization_Homo sapiens_R-HSA-2730905
Negative regulation of the PI3K/AKT network_Homo sapiens_R-HSA-199418
G1 Phase_Homo sapiens_R-HSA-69236
Cyclin D associated events in G1_Homo sapiens_R-HSA-69231
MAP2K and MAPK activation_Homo sapiens_R-HSA-5674135
Spry regulation of FGF signaling_Homo sapiens_R-HSA-1295596
GRB2 events in ERBB2 signaling_Homo sapiens_R-HSA-1963640
Rap1 signalling_Homo sapiens_R-HSA-392517
Sema3A PAK dependent Axon repulsion_Homo sapiens_R-HSA-399954
Cardiac conduction_Homo sapiens_R-HSA-5576891

Transport of nucleosides and free purine and pyrimidine bases across the plasma membrane_Homo sapiens_R
Activation of NIMA Kinases NEK9, NEK6, NEK7_Homo sapiens_R-HSA-2980767
Post-transcriptional silencing by small RNAs_Homo sapiens_R-HSA-426496
Muscle contraction_Homo sapiens_R-HSA-397014
EGFR downregulation_Homo sapiens_R-HSA-182971
Platelet calcium homeostasis_Homo sapiens_R-HSA-418360
Glycosaminoglycan metabolism_Homo sapiens_R-HSA-1630316
Interleukin-3, 5 and GM-CSF signaling_Homo sapiens_R-HSA-512988
Signaling by Insulin receptor_Homo sapiens_R-HSA-74752
Gap junction trafficking_Homo sapiens_R-HSA-190828
N-glycan trimming in the ER and Calnexin/Calreticulin cycle_Homo sapiens_R-HSA-532668
Platelet sensitization by LDL_Homo sapiens_R-HSA-432142
Signaling by VEGF_Homo sapiens_R-HSA-194138
EPH-Ephrin signaling_Homo sapiens_R-HSA-2682334
Signaling by PTK6_Homo sapiens_R-HSA-8848021
Semaphorin interactions_Homo sapiens_R-HSA-373755
Opioid Signalling_Homo sapiens_R-HSA-111885
Signaling by the B Cell Receptor (BCR)_Homo sapiens_R-HSA-983705
Interaction between L1 and Ankyrins_Homo sapiens_R-HSA-445095
COPI-independent Golgi-to-ER retrograde traffic_Homo sapiens_R-HSA-6811436
Myogenesis_Homo sapiens_R-HSA-525793
CDO in myogenesis_Homo sapiens_R-HSA-375170
Synthesis of UDP-N-acetyl-glucosamine_Homo sapiens_R-HSA-446210
Downregulation of ERBB4 signaling_Homo sapiens_R-HSA-1253288
RNF mutants show enhanced WNT signaling and proliferation_Homo sapiens_R-HSA-5340588
Degradation of beta-catenin by the destruction complex_Homo sapiens_R-HSA-195253
Toll Like Receptor 10 (TLR10) Cascade_Homo sapiens_R-HSA-168142
Toll Like Receptor 5 (TLR5) Cascade_Homo sapiens_R-HSA-168176
MyD88 cascade initiated on plasma membrane_Homo sapiens_R-HSA-975871
TP53 Regulates Transcription of Genes Involved in G2 Cell Cycle Arrest_Homo sapiens_R-HSA-6804114
SHC1 events in ERBB2 signaling_Homo sapiens_R-HSA-1250196
MyD88-independent TLR3/TLR4 cascade_Homo sapiens_R-HSA-166166
Toll Like Receptor 3 (TLR3) Cascade_Homo sapiens_R-HSA-168164
TRIF-mediated TLR3/TLR4 signaling_Homo sapiens_R-HSA-937061
Ion transport by P-type ATPases_Homo sapiens_R-HSA-936837
Disease_Homo sapiens_R-HSA-1643685
Neurotransmitter Receptor Binding And Downstream Transmission In The Postsynaptic Cell_Homo sapiens_R-
FRS-mediated FGFR2 signaling_Homo sapiens_R-HSA-5654700
FRS-mediated FGFR4 signaling_Homo sapiens_R-HSA-5654712
FRS-mediated FGFR3 signaling_Homo sapiens_R-HSA-5654706
FRS-mediated FGFR1 signaling_Homo sapiens_R-HSA-5654693
PI5P, PP2A and IER3 Regulate PI3K/AKT Signaling_Homo sapiens_R-HSA-6811558
TRAF6 mediated induction of NFkB and MAP kinases upon TLR7/8 or 9 activation_Homo sapiens_R-HSA-9751
Signalling to ERKs_Homo sapiens_R-HSA-187687
Gap junction trafficking and regulation_Homo sapiens_R-HSA-157858
VEGFA-VEGFR2 Pathway_Homo sapiens_R-HSA-4420097
Formation of Incision Complex in GG-NER_Homo sapiens_R-HSA-5696395
ARMS-mediated activation_Homo sapiens_R-HSA-170984
Gap junction assembly_Homo sapiens_R-HSA-190861
MyD88 dependent cascade initiated on endosome_Homo sapiens_R-HSA-975155
Toll Like Receptor 7/8 (TLR7/8) Cascade_Homo sapiens_R-HSA-168181
Downstream signaling events of B Cell Receptor (BCR)_Homo sapiens_R-HSA-1168372
Pre-NOTCH Expression and Processing_Homo sapiens_R-HSA-1912422

Adherens junctions interactions_Homo sapiens_R-HSA-418990
Epigenetic regulation of gene expression_Homo sapiens_R-HSA-212165
Intra-Golgi traffic_Homo sapiens_R-HSA-6811438
Phospholipase C-mediated cascade; FGFR3_Homo sapiens_R-HSA-5654227
Integration of provirus_Homo sapiens_R-HSA-162592
AKT phosphorylates targets in the nucleus_Homo sapiens_R-HSA-198693
Role of Abl in Robo-Slit signaling_Homo sapiens_R-HSA-428890
Adrenoceptors_Homo sapiens_R-HSA-390696
PI3K/AKT Signaling in Cancer_Homo sapiens_R-HSA-2219528
Cell-Cell communication_Homo sapiens_R-HSA-1500931
Prolonged ERK activation events_Homo sapiens_R-HSA-169893
SUMOylation_Homo sapiens_R-HSA-2990846
Signaling by Leptin_Homo sapiens_R-HSA-2586552
Signaling by NOTCH2_Homo sapiens_R-HSA-1980145
SMAD2/SMAD3:SMAD4 heterotrimer regulates transcription_Homo sapiens_R-HSA-2173796
Negative regulation of FGFR1 signaling_Homo sapiens_R-HSA-5654726
SUMOylation of RNA binding proteins_Homo sapiens_R-HSA-4570464
Signaling by ERBB2_Homo sapiens_R-HSA-1227986
Signaling by TGF-beta Receptor Complex_Homo sapiens_R-HSA-170834
Toll Like Receptor 9 (TLR9) Cascade_Homo sapiens_R-HSA-168138
Signalling to RAS_Homo sapiens_R-HSA-167044
Phospholipase C-mediated cascade; FGFR4_Homo sapiens_R-HSA-5654228
Cleavage of Growing Transcript in the Termination Region_Homo sapiens_R-HSA-109688
RNA Polymerase II Transcription Termination_Homo sapiens_R-HSA-73856
Post-Elongation Processing of the Transcript_Homo sapiens_R-HSA-76044
Establishment of Sister Chromatid Cohesion_Homo sapiens_R-HSA-2468052
ER Quality Control Compartment (ERQC)_Homo sapiens_R-HSA-901032
POU5F1 (OCT4), SOX2, NANOG repress genes related to differentiation_Homo sapiens_R-HSA-2892245
Regulation of gene expression by Hypoxia-inducible Factor_Homo sapiens_R-HSA-1234158
MASTL Facilitates Mitotic Progression_Homo sapiens_R-HSA-2465910
Activation of the AP-1 family of transcription factors_Homo sapiens_R-HSA-450341
eNOS activation_Homo sapiens_R-HSA-203615
Acetylcholine regulates insulin secretion_Homo sapiens_R-HSA-399997
HS-GAG degradation_Homo sapiens_R-HSA-2024096
ERK/MAPK targets_Homo sapiens_R-HSA-198753
CD209 (DC-SIGN) signaling_Homo sapiens_R-HSA-5621575
Transcriptional Regulation by TP53_Homo sapiens_R-HSA-3700989
Constitutive Signaling by Aberrant PI3K in Cancer_Homo sapiens_R-HSA-2219530
Cell-cell junction organization_Homo sapiens_R-HSA-421270
NCAM signaling for neurite out-growth_Homo sapiens_R-HSA-375165
EPHA-mediated growth cone collapse_Homo sapiens_R-HSA-3928663
Fatty acid, triacylglycerol, and ketone body metabolism_Homo sapiens_R-HSA-535734
Oxidative Stress Induced Senescence_Homo sapiens_R-HSA-2559580
TP53 Regulates Transcription of Cell Cycle Genes_Homo sapiens_R-HSA-6791312
GRB2 events in EGFR signaling_Homo sapiens_R-HSA-179812
SHC1 events in EGFR signaling_Homo sapiens_R-HSA-180336
SOS-mediated signalling_Homo sapiens_R-HSA-112412
SHC1 events in ERBB4 signaling_Homo sapiens_R-HSA-1250347
RAF/MAP kinase cascade_Homo sapiens_R-HSA-5673001
CTLA4 inhibitory signaling_Homo sapiens_R-HSA-389513
MAPK6/MAPK4 signaling_Homo sapiens_R-HSA-5687128
MyD88:Mal cascade initiated on plasma membrane_Homo sapiens_R-HSA-166058
Toll Like Receptor TLR1:TLR2 Cascade_Homo sapiens_R-HSA-168179

Toll Like Receptor TLR6:TLR2 Cascade_Homo sapiens_R-HSA-168188
Toll Like Receptor 2 (TLR2) Cascade_Homo sapiens_R-HSA-181438
Interleukin-2 signaling_Homo sapiens_R-HSA-451927
Vesicle-mediated transport_Homo sapiens_R-HSA-5653656
Phospholipase C-mediated cascade; FGFR2_Homo sapiens_R-HSA-5654221
Biosynthesis of the N-glycan precursor (dolichol lipid-linked oligosaccharide, LLO) and transfer to a nascent pro
Import of palmitoyl-CoA into the mitochondrial matrix_Homo sapiens_R-HSA-200425
Interleukin-6 signaling_Homo sapiens_R-HSA-1059683
RHO GTPases activate IQGAPs_Homo sapiens_R-HSA-5626467
Sodium/Calcium exchangers_Homo sapiens_R-HSA-425561
CLEC7A (Dectin-1) induces NFAT activation_Homo sapiens_R-HSA-5607763
Apoptotic cleavage of cell adhesion proteins_Homo sapiens_R-HSA-351906
Hormone ligand-binding receptors_Homo sapiens_R-HSA-375281
Heme biosynthesis_Homo sapiens_R-HSA-189451
Factors involved in megakaryocyte development and platelet production_Homo sapiens_R-HSA-983231
Downregulation of SMAD2/3:SMAD4 transcriptional activity_Homo sapiens_R-HSA-2173795
PI3K Cascade_Homo sapiens_R-HSA-109704
Signalling to p38 via RIT and RIN_Homo sapiens_R-HSA-187706
Chondroitin sulfate/dermatan sulfate metabolism_Homo sapiens_R-HSA-1793185
Golgi-to-ER retrograde transport_Homo sapiens_R-HSA-8856688
Integration of energy metabolism_Homo sapiens_R-HSA-163685
Frs2-mediated activation_Homo sapiens_R-HSA-170968
Signal Transduction_Homo sapiens_R-HSA-162582
Metabolism of fat-soluble vitamins_Homo sapiens_R-HSA-6806667
mRNA 3'-end processing_Homo sapiens_R-HSA-72187
Post-Elongation Processing of Intron-Containing pre-mRNA_Homo sapiens_R-HSA-112296
Platelet Aggregation (Plug Formation)_Homo sapiens_R-HSA-76009
SUMO E3 ligases SUMOylate target proteins_Homo sapiens_R-HSA-3108232
Post-Elongation Processing of Intronless pre-mRNA_Homo sapiens_R-HSA-112297
Processing of Capped Intronless Pre-mRNA_Homo sapiens_R-HSA-75067
DARPP-32 events_Homo sapiens_R-HSA-180024
Activated TLR4 signalling_Homo sapiens_R-HSA-166054
Ethanol oxidation_Homo sapiens_R-HSA-71384
Defective EXT2 causes exostoses 2_Homo sapiens_R-HSA-3656237
Defective EXT1 causes exostoses 1, TRPS2 and CHDS_Homo sapiens_R-HSA-3656253
Inhibition of replication initiation of damaged DNA by RB1/E2F1_Homo sapiens_R-HSA-113501
NOTCH2 intracellular domain regulates transcription_Homo sapiens_R-HSA-2197563
ERKs are inactivated_Homo sapiens_R-HSA-202670
PECAM1 interactions_Homo sapiens_R-HSA-210990
Beta-catenin independent WNT signaling_Homo sapiens_R-HSA-3858494
Interleukin receptor SHC signaling_Homo sapiens_R-HSA-912526
Amino acid synthesis and interconversion (transamination)_Homo sapiens_R-HSA-70614
RAF activation_Homo sapiens_R-HSA-5673000
Synthesis and interconversion of nucleotide di- and triphosphates_Homo sapiens_R-HSA-499943
Apoptosis_Homo sapiens_R-HSA-109581
TP53 Regulates Metabolic Genes_Homo sapiens_R-HSA-5628897
WNT5A-dependent internalization of FZD2, FZD5 and ROR2_Homo sapiens_R-HSA-5140745
Retrograde neurotrophin signalling_Homo sapiens_R-HSA-177504
IRF3-mediated induction of type I IFN_Homo sapiens_R-HSA-3270619
Activation of DNA fragmentation factor_Homo sapiens_R-HSA-211227
Apoptosis induced DNA fragmentation_Homo sapiens_R-HSA-140342
TNFR1-induced proapoptotic signaling_Homo sapiens_R-HSA-5357786
Synthesis of PE_Homo sapiens_R-HSA-1483213

Metabolism of carbohydrates_Homo sapiens_R-HSA-71387
VEGFR2 mediated cell proliferation_Homo sapiens_R-HSA-5218921
Heparan sulfate/heparin (HS-GAG) metabolism_Homo sapiens_R-HSA-1638091
Diseases associated with glycosaminoglycan metabolism_Homo sapiens_R-HSA-3560782
A tetrasaccharide linker sequence is required for GAG synthesis_Homo sapiens_R-HSA-1971475
Regulation of Hypoxia-inducible Factor (HIF) by oxygen_Homo sapiens_R-HSA-1234174
Cellular response to hypoxia_Homo sapiens_R-HSA-2262749
PTK6 Regulates RHO GTPases, RAS GTPase and MAP kinases_Homo sapiens_R-HSA-8849471
TNFR1-induced NFkappaB signaling pathway_Homo sapiens_R-HSA-5357956
Downregulation of TGF-beta receptor signaling_Homo sapiens_R-HSA-2173788
Ligand-gated ion channel transport_Homo sapiens_R-HSA-975298
Hedgehog 'on' state_Homo sapiens_R-HSA-5632684
PKB-mediated events_Homo sapiens_R-HSA-109703
PKMTs methylate histone lysines_Homo sapiens_R-HSA-3214841
Negative regulation of MAPK pathway_Homo sapiens_R-HSA-5675221
Programmed Cell Death_Homo sapiens_R-HSA-5357801
Cell surface interactions at the vascular wall_Homo sapiens_R-HSA-202733
Cell junction organization_Homo sapiens_R-HSA-446728
Glycerophospholipid biosynthesis_Homo sapiens_R-HSA-1483206
Regulation of TP53 Activity_Homo sapiens_R-HSA-5633007
Uptake and actions of bacterial toxins_Homo sapiens_R-HSA-5339562
Pyruvate metabolism_Homo sapiens_R-HSA-70268
Early Phase of HIV Life Cycle_Homo sapiens_R-HSA-162594
Regulation of IFNG signaling_Homo sapiens_R-HSA-877312
Golgi Cisternae Pericentriolar Stack Reorganization_Homo sapiens_R-HSA-162658
Lysosphingolipid and LPA receptors_Homo sapiens_R-HSA-419408
Methylation_Homo sapiens_R-HSA-156581
Signaling by Hedgehog_Homo sapiens_R-HSA-5358351
FCERI mediated MAPK activation_Homo sapiens_R-HSA-2871796
Retinoid metabolism and transport_Homo sapiens_R-HSA-975634
EPHB-mediated forward signaling_Homo sapiens_R-HSA-3928662
Innate Immune System_Homo sapiens_R-HSA-168249
Recognition of DNA damage by PCNA-containing replication complex_Homo sapiens_R-HSA-110314
Formation of the beta-catenin:TCF transactivating complex_Homo sapiens_R-HSA-201722
Toll Like Receptor 4 (TLR4) Cascade_Homo sapiens_R-HSA-166016
N-Glycan antennae elongation_Homo sapiens_R-HSA-975577
Calnexin/calreticulin cycle_Homo sapiens_R-HSA-901042
mRNA decay by 5' to 3' exoribonuclease_Homo sapiens_R-HSA-430039
Processing of Intronless Pre-mRNAs_Homo sapiens_R-HSA-77595
Signaling by EGFRvIII in Cancer_Homo sapiens_R-HSA-5637812
Constitutive Signaling by EGFRvIII_Homo sapiens_R-HSA-5637810
Negative regulation of TCF-dependent signaling by WNT ligand antagonists_Homo sapiens_R-HSA-3772470
p130Cas linkage to MAPK signaling for integrins_Homo sapiens_R-HSA-372708
Recycling of bile acids and salts_Homo sapiens_R-HSA-159418
Synthesis of bile acids and bile salts via 27-hydroxycholesterol_Homo sapiens_R-HSA-193807
Transport of Mature Transcript to Cytoplasm_Homo sapiens_R-HSA-72202
Negative regulation of FGFR3 signaling_Homo sapiens_R-HSA-5654732
SUMOylation of DNA damage response and repair proteins_Homo sapiens_R-HSA-3108214
Phase 0 - rapid depolarisation_Homo sapiens_R-HSA-5576892
Inositol phosphate metabolism_Homo sapiens_R-HSA-1483249
STING mediated induction of host immune responses_Homo sapiens_R-HSA-1834941
MAP3K8 (TPL2)-dependent MAPK1/3 activation_Homo sapiens_R-HSA-5684264
Metabolism of Angiotensinogen to Angiotensins_Homo sapiens_R-HSA-2022377

Transmission across Chemical Synapses_Homo sapiens_R-HSA-112315
DNA Damage Bypass_Homo sapiens_R-HSA-73893
HS-GAG biosynthesis_Homo sapiens_R-HSA-2022928
Negative regulation of FGFR4 signaling_Homo sapiens_R-HSA-5654733
Regulation of TNFR1 signaling_Homo sapiens_R-HSA-5357905
Hyaluronan metabolism_Homo sapiens_R-HSA-2142845
G1/S-Specific Transcription_Homo sapiens_R-HSA-69205
RHO GTPases Activate ROCKs_Homo sapiens_R-HSA-5627117
Ras activation uopn Ca2+ infux through NMDA receptor_Homo sapiens_R-HSA-442982
PKA-mediated phosphorylation of CREB_Homo sapiens_R-HSA-111931
Metabolism of porphyrins_Homo sapiens_R-HSA-189445
Signaling by Interleukins_Homo sapiens_R-HSA-449147
RNA Polymerase I Transcription Initiation_Homo sapiens_R-HSA-73762
Synthesis of substrates in N-glycan biosythesis_Homo sapiens_R-HSA-446219
Asymmetric localization of PCP proteins_Homo sapiens_R-HSA-4608870
Glycolysis_Homo sapiens_R-HSA-70171
TGF-beta receptor signaling activates SMADs_Homo sapiens_R-HSA-2173789
Regulation of insulin secretion_Homo sapiens_R-HSA-422356
Catabolism of glucuronate to xylulose-5-phosphate_Homo sapiens_R-HSA-5661270
Hyaluronan biosynthesis and export_Homo sapiens_R-HSA-2142850
Electric Transmission Across Gap Junctions_Homo sapiens_R-HSA-112303
Transmission across Electrical Synapses_Homo sapiens_R-HSA-112307
N-glycan trimming and elongation in the cis-Golgi_Homo sapiens_R-HSA-964739
G2 Phase_Homo sapiens_R-HSA-68911
Loss of Function of FBXW7 in Cancer and NOTCH1 Signaling_Homo sapiens_R-HSA-2644607
FBXW7 Mutants and NOTCH1 in Cancer_Homo sapiens_R-HSA-2644605
PTK6 Expression_Homo sapiens_R-HSA-8849473
Cyclin B2 mediated events_Homo sapiens_R-HSA-157881
G2/M DNA replication checkpoint_Homo sapiens_R-HSA-69478
Tachykinin receptors bind tachykinins_Homo sapiens_R-HSA-380095
Muscarinic acetylcholine receptors_Homo sapiens_R-HSA-390648
NGF-independant TRKA activation_Homo sapiens_R-HSA-187024
SMAC binds to IAPs_Homo sapiens_R-HSA-111463
SMAC-mediated dissociation of IAP:caspase complexes_Homo sapiens_R-HSA-111464
SMAC-mediated apoptotic response_Homo sapiens_R-HSA-111469
Trafficking of myristoylated proteins to the cilium_Homo sapiens_R-HSA-5624138
Visual phototransduction_Homo sapiens_R-HSA-2187338
Pyruvate metabolism and Citric Acid (TCA) cycle_Homo sapiens_R-HSA-71406
Miscellaneous transport and binding events_Homo sapiens_R-HSA-5223345
Oxygen-dependent proline hydroxylation of Hypoxia-inducible Factor Alpha_Homo sapiens_R-HSA-1234176
Regulation of signaling by CBL_Homo sapiens_R-HSA-912631
Sphingolipid de novo biosynthesis_Homo sapiens_R-HSA-1660661
Sialic acid metabolism_Homo sapiens_R-HSA-4085001
Transport of the SLBP independent Mature mRNA_Homo sapiens_R-HSA-159227
E2F mediated regulation of DNA replication_Homo sapiens_R-HSA-113510
Inactivation, recovery and regulation of the phototransduction cascade_Homo sapiens_R-HSA-2514859
Smooth Muscle Contraction_Homo sapiens_R-HSA-445355
Global Genome Nucleotide Excision Repair (GG-NER)_Homo sapiens_R-HSA-5696399
Ion channel transport_Homo sapiens_R-HSA-983712
Cytosolic sensors of pathogen-associated DNA_Homo sapiens_R-HSA-1834949
Transport of the SLBP Dependant Mature mRNA_Homo sapiens_R-HSA-159230
Nuclear Pore Complex (NPC) Disassembly_Homo sapiens_R-HSA-3301854
Negative regulation of FGFR2 signaling_Homo sapiens_R-HSA-5654727

Negative regulators of RIG-I/MDA5 signaling_Homo sapiens_R-HSA-936440
The phototransduction cascade_Homo sapiens_R-HSA-2514856
Striated Muscle Contraction_Homo sapiens_R-HSA-390522
Defective B3GALT6 causes EDSP2 and SEMDJL1_Homo sapiens_R-HSA-4420332
Defective B4GALT7 causes EDS, progeroid type_Homo sapiens_R-HSA-3560783
Defective B3GAT3 causes JDSSDHD_Homo sapiens_R-HSA-3560801
Regulation of TP53 Activity through Methylation_Homo sapiens_R-HSA-6804760
Constitutive Signaling by Ligand-Responsive EGFR Cancer Variants_Homo sapiens_R-HSA-1236382
Signaling by Ligand-Responsive EGFR Variants in Cancer_Homo sapiens_R-HSA-5637815
Signaling by EGFR in Cancer_Homo sapiens_R-HSA-1643713
Deadenylation-dependent mRNA decay_Homo sapiens_R-HSA-429914
Asparagine N-linked glycosylation_Homo sapiens_R-HSA-446203
Ion homeostasis_Homo sapiens_R-HSA-5578775
Mitotic G1-G1/S phases_Homo sapiens_R-HSA-453279
Regulation of TP53 Degradation_Homo sapiens_R-HSA-6804757
Fructose metabolism_Homo sapiens_R-HSA-5652084
SUMO is proteolytically processed_Homo sapiens_R-HSA-3065679
IRF3 mediated activation of type 1 IFN_Homo sapiens_R-HSA-1606341
Telomere Extension By Telomerase_Homo sapiens_R-HSA-171319
FGFR1b ligand binding and activation_Homo sapiens_R-HSA-190370
Phosphorylation of Emi1_Homo sapiens_R-HSA-176417
Proton-coupled monocarboxylate transport_Homo sapiens_R-HSA-433692
Scavenging by Class F Receptors_Homo sapiens_R-HSA-3000484
Activation of TRKA receptors_Homo sapiens_R-HSA-187015
Molybdenum cofactor biosynthesis_Homo sapiens_R-HSA-947581
Transport and synthesis of PAPS_Homo sapiens_R-HSA-174362
Synthesis of Lipoxins (LX)_Homo sapiens_R-HSA-2142700
Protein repair_Homo sapiens_R-HSA-5676934
Transport of Mature mRNA derived from an Intron-Containing Transcript_Homo sapiens_R-HSA-159236
Chondroitin sulfate biosynthesis_Homo sapiens_R-HSA-2022870
eNOS activation and regulation_Homo sapiens_R-HSA-203765
Metabolism of nitric oxide_Homo sapiens_R-HSA-202131
Regulation of TP53 Expression and Degradation_Homo sapiens_R-HSA-6806003
RHO GTPases Activate WASPs and WAVEs_Homo sapiens_R-HSA-5663213
XBP1(S) activates chaperone genes_Homo sapiens_R-HSA-381038
Metabolism of nucleotides_Homo sapiens_R-HSA-15869
Post-translational protein modification_Homo sapiens_R-HSA-597592
NOTCH2 Activation and Transmission of Signal to the Nucleus_Homo sapiens_R-HSA-2979096
RHO GTPases activate PAKs_Homo sapiens_R-HSA-5627123
Regulation of Insulin-like Growth Factor (IGF) transport and uptake by Insulin-like Growth Factor Binding Protei
Toll-Like Receptors Cascades_Homo sapiens_R-HSA-168898
Transport of Mature mRNA Derived from an Intronless Transcript_Homo sapiens_R-HSA-159231
Diseases of glycosylation_Homo sapiens_R-HSA-3781865
C-type lectin receptors (CLRs)_Homo sapiens_R-HSA-5621481
Degradation of AXIN_Homo sapiens_R-HSA-4641257
RNA Polymerase II Transcription_Homo sapiens_R-HSA-73857
Defective CHST6 causes MCDC1_Homo sapiens_R-HSA-3656225
Defective ST3GAL3 causes MCT12 and EIEE15_Homo sapiens_R-HSA-3656243
Defective B4GALT1 causes B4GALT1-CDG (CDG-2d)_Homo sapiens_R-HSA-3656244
2-LTR circle formation_Homo sapiens_R-HSA-164843
Nef Mediated CD8 Down-regulation_Homo sapiens_R-HSA-182218
Neurofascin interactions_Homo sapiens_R-HSA-447043
NrCAM interactions_Homo sapiens_R-HSA-447038

PTK6 promotes HIF1A stabilization_Homo sapiens_R-HSA-8857538
PP2A-mediated dephosphorylation of key metabolic factors_Homo sapiens_R-HSA-163767
Nectin/Necl trans heterodimerization_Homo sapiens_R-HSA-420597
Binding of TCF/LEF:CTNNB1 to target gene promoters_Homo sapiens_R-HSA-4411364
CREB phosphorylation_Homo sapiens_R-HSA-199920
CREB phosphorylation through the activation of Adenylate Cyclase_Homo sapiens_R-HSA-442720
Apoptotic factor-mediated response_Homo sapiens_R-HSA-111471
Phosphate bond hydrolysis by NUDT proteins_Homo sapiens_R-HSA-2393930
Histidine catabolism_Homo sapiens_R-HSA-70921
Transport of Mature mRNAs Derived from Intronless Transcripts_Homo sapiens_R-HSA-159234
Signaling by FGFR1 in disease_Homo sapiens_R-HSA-5655302
Mitotic Prophase_Homo sapiens_R-HSA-68875
mTORC1-mediated signalling_Homo sapiens_R-HSA-166208
Cyclin A/B1 associated events during G2/M transition_Homo sapiens_R-HSA-69273
IRE1alpha activates chaperones_Homo sapiens_R-HSA-381070
GABA receptor activation_Homo sapiens_R-HSA-977443
Cellular Senescence_Homo sapiens_R-HSA-2559583
Immune System_Homo sapiens_R-HSA-168256
mTOR signalling_Homo sapiens_R-HSA-165159
NS1 Mediated Effects on Host Pathways_Homo sapiens_R-HSA-168276
Dual Incision in GG-NER_Homo sapiens_R-HSA-5696400
PCP/CE pathway_Homo sapiens_R-HSA-4086400
Generic Transcription Pathway_Homo sapiens_R-HSA-212436
Deadenylation of mRNA_Homo sapiens_R-HSA-429947
Branched-chain amino acid catabolism_Homo sapiens_R-HSA-70895
Cellular responses to stress_Homo sapiens_R-HSA-2262752
Neurotransmitter Clearance In The Synaptic Cleft_Homo sapiens_R-HSA-112311
Organic anion transporters_Homo sapiens_R-HSA-428643
Cleavage of the damaged pyrimidine_Homo sapiens_R-HSA-110329
Recognition and association of DNA glycosylase with site containing an affected pyrimidine_Homo sapiens_R-H
Depyrimidination_Homo sapiens_R-HSA-73928
Insulin-like Growth Factor-2 mRNA Binding Proteins (IGF2BPs/IMPs/VICKZs) bind RNA_Homo sapiens_R-HSA
Release of Hh-Np from the secreting cell_Homo sapiens_R-HSA-5362798
Digestion of dietary carbohydrate_Homo sapiens_R-HSA-189085
HuR (ELAVL1) binds and stabilizes mRNA_Homo sapiens_R-HSA-450520
Activation of PUMA and translocation to mitochondria_Homo sapiens_R-HSA-139915
AMPK inhibits chREBP transcriptional activation activity_Homo sapiens_R-HSA-163680
Nef and signal transduction_Homo sapiens_R-HSA-164944
Recycling of eIF2:GDP_Homo sapiens_R-HSA-72731
Regulation of cytoskeletal remodeling and cell spreading by IPP complex components_Homo sapiens_R-HSA-4
Orexin and neuropeptides FF and QRFP bind to their respective receptors_Homo sapiens_R-HSA-389397
Hydrolysis of LPC_Homo sapiens_R-HSA-1483115
Synthesis of PG_Homo sapiens_R-HSA-1483148
Growth hormone receptor signaling_Homo sapiens_R-HSA-982772
Sema4D induced cell migration and growth-cone collapse_Homo sapiens_R-HSA-416572
Synthesis of bile acids and bile salts via 7alpha-hydroxycholesterol_Homo sapiens_R-HSA-193368
Endogenous sterols_Homo sapiens_R-HSA-211976
Elastic fibre formation_Homo sapiens_R-HSA-1566948
Host Interactions with Influenza Factors_Homo sapiens_R-HSA-168253
Condensation of Prophase Chromosomes_Homo sapiens_R-HSA-2299718
TNF signaling_Homo sapiens_R-HSA-75893
Degradation of GLI1 by the proteasome_Homo sapiens_R-HSA-5610780
GLI3 is processed to GLI3R by the proteasome_Homo sapiens_R-HSA-5610785

Degradation of GLI2 by the proteasome_Homo sapiens_R-HSA-5610783
Signaling by Retinoic Acid_Homo sapiens_R-HSA-5362517
Intrinsic Pathway for Apoptosis_Homo sapiens_R-HSA-109606
Transcriptional regulation of pluripotent stem cells_Homo sapiens_R-HSA-452723
Netrin-1 signaling_Homo sapiens_R-HSA-373752
Constitutive Signaling by AKT1 E17K in Cancer_Homo sapiens_R-HSA-5674400
HDMs demethylate histones_Homo sapiens_R-HSA-3214842
Synthesis of IP3 and IP4 in the cytosol_Homo sapiens_R-HSA-1855204
Glyoxylate metabolism and glycine degradation_Homo sapiens_R-HSA-389661
Cellular response to heat stress_Homo sapiens_R-HSA-3371556
Bicarbonate transporters_Homo sapiens_R-HSA-425381
Organic cation transport_Homo sapiens_R-HSA-549127
Displacement of DNA glycosylase by APEX1_Homo sapiens_R-HSA-110357
Crosslinking of collagen fibrils_Homo sapiens_R-HSA-2243919
Cohesin Loading onto Chromatin_Homo sapiens_R-HSA-2470946
Processing and activation of SUMO_Homo sapiens_R-HSA-3215018
Formation of annular gap junctions_Homo sapiens_R-HSA-196025
Nef Mediated CD4 Down-regulation_Homo sapiens_R-HSA-167590
A third proteolytic cleavage releases NICD_Homo sapiens_R-HSA-157212
Small interfering RNA (siRNA) biogenesis_Homo sapiens_R-HSA-426486
Prostanoid ligand receptors_Homo sapiens_R-HSA-391908
Amine ligand-binding receptors_Homo sapiens_R-HSA-375280
Gastrin-CREB signalling pathway via PKC and MAPK_Homo sapiens_R-HSA-881907
Regulation of actin dynamics for phagocytic cup formation_Homo sapiens_R-HSA-2029482
Neuronal System_Homo sapiens_R-HSA-112316
Glucose metabolism_Homo sapiens_R-HSA-70326
RIG-I/MDA5 mediated induction of IFN-alpha/beta pathways_Homo sapiens_R-HSA-168928
N-glycan antennae elongation in the medial/trans-Golgi_Homo sapiens_R-HSA-975576
Attenuation phase_Homo sapiens_R-HSA-3371568
ZBP1(DAI) mediated induction of type I IFNs_Homo sapiens_R-HSA-1606322
Regulation of HSF1-mediated heat shock response_Homo sapiens_R-HSA-3371453
Kinesins_Homo sapiens_R-HSA-983189
Hedgehog 'off' state_Homo sapiens_R-HSA-5610787
COPI-dependent Golgi-to-ER retrograde traffic_Homo sapiens_R-HSA-6811434
Peptide hormone metabolism_Homo sapiens_R-HSA-2980736
VEGFR2 mediated vascular permeability_Homo sapiens_R-HSA-5218920
RNA Pol II CTD phosphorylation and interaction with CE_Homo sapiens_R-HSA-167160
RNA Pol II CTD phosphorylation and interaction with CE_Homo sapiens_R-HSA-77075
Interleukin-6 family signaling_Homo sapiens_R-HSA-6783589
Integrin alphaIIb beta3 signaling_Homo sapiens_R-HSA-354192
Sema4D in semaphorin signaling_Homo sapiens_R-HSA-400685
Synthesis of bile acids and bile salts_Homo sapiens_R-HSA-192105
Base-Excision Repair, AP Site Formation_Homo sapiens_R-HSA-73929
Gap junction degradation_Homo sapiens_R-HSA-190873
Activation of Ca-permeable Kainate Receptor_Homo sapiens_R-HSA-451308
Ionotropic activity of Kainate Receptors_Homo sapiens_R-HSA-451306
Activation of PPARGC1A (PGC-1alpha) by phosphorylation_Homo sapiens_R-HSA-2151209
SLBP independent Processing of Histone Pre-mRNAs_Homo sapiens_R-HSA-111367
E2F-enabled inhibition of pre-replication complex formation_Homo sapiens_R-HSA-113507
Signal attenuation_Homo sapiens_R-HSA-74749
Regulated proteolysis of p75NTR_Homo sapiens_R-HSA-193692
Signaling by FGFR3 fusions in cancer_Homo sapiens_R-HSA-8853334
Netrin mediated repulsion signals_Homo sapiens_R-HSA-418886

GP1b-IX-V activation signalling_Homo sapiens_R-HSA-430116
Dectin-2 family_Homo sapiens_R-HSA-5621480
Adenylate cyclase activating pathway_Homo sapiens_R-HSA-170660
Tetrahydrobiopterin (BH4) synthesis, recycling, salvage and regulation_Homo sapiens_R-HSA-1474151
Ligand-independent caspase activation via DCC_Homo sapiens_R-HSA-418889
Glycoprotein hormones_Homo sapiens_R-HSA-209822
Thyroxine biosynthesis_Homo sapiens_R-HSA-209968
Urea cycle_Homo sapiens_R-HSA-70635
Organelle biogenesis and maintenance_Homo sapiens_R-HSA-1852241
Fcgamma receptor (FCGR) dependent phagocytosis_Homo sapiens_R-HSA-2029480
G alpha (z) signalling events_Homo sapiens_R-HSA-418597
The role of Nef in HIV-1 replication and disease pathogenesis_Homo sapiens_R-HSA-164952
Synthesis of PA_Homo sapiens_R-HSA-1483166
Antigen activates B Cell Receptor (BCR) leading to generation of second messengers_Homo sapiens_R-HSA-[illegible]
SLBP Dependent Processing of Replication-Dependent Histone Pre-mRNAs_Homo sapiens_R-HSA-77588
CDC6 association with the ORC:origin complex_Homo sapiens_R-HSA-68689
Signaling by NOTCH3_Homo sapiens_R-HSA-1980148
Signaling by NOTCH4_Homo sapiens_R-HSA-1980150
FGFR1c ligand binding and activation_Homo sapiens_R-HSA-190373
Signaling by activated point mutants of FGFR1_Homo sapiens_R-HSA-1839122
Signaling by FGFR4 in disease_Homo sapiens_R-HSA-5655291
Recruitment of NuMA to mitotic centrosomes_Homo sapiens_R-HSA-380320
Condensation of Prometaphase Chromosomes_Homo sapiens_R-HSA-2514853
Androgen biosynthesis_Homo sapiens_R-HSA-193048
Surfactant metabolism_Homo sapiens_R-HSA-5683826
Activation of BH3-only proteins_Homo sapiens_R-HSA-114452
PIWI-interacting RNA (piRNA) biogenesis_Homo sapiens_R-HSA-5601884
mRNA Capping_Homo sapiens_R-HSA-72086
Phospholipid metabolism_Homo sapiens_R-HSA-1483257
Death Receptor Signalling_Homo sapiens_R-HSA-73887
Regulation of RAS by GAPs_Homo sapiens_R-HSA-5658442
Unfolded Protein Response (UPR)_Homo sapiens_R-HSA-381119
Molecules associated with elastic fibres_Homo sapiens_R-HSA-2129379
Transport of Ribonucleoproteins into the Host Nucleus_Homo sapiens_R-HSA-168271
RNA Polymerase I Promoter Escape_Homo sapiens_R-HSA-73772
Tight junction interactions_Homo sapiens_R-HSA-420029
Erythrocytes take up carbon dioxide and release oxygen_Homo sapiens_R-HSA-1237044
O2/CO2 exchange in erythrocytes_Homo sapiens_R-HSA-1480926
Reversible hydration of carbon dioxide_Homo sapiens_R-HSA-1475029
Keratan sulfate degradation_Homo sapiens_R-HSA-2022857
Hyaluronan uptake and degradation_Homo sapiens_R-HSA-2160916
Endosomal/Vacuolar pathway_Homo sapiens_R-HSA-1236977
Chk1/Chk2(Cds1) mediated inactivation of Cyclin B:Cdk1 complex_Homo sapiens_R-HSA-75035
Peptide hormone biosynthesis_Homo sapiens_R-HSA-209952
Purine ribonucleoside monophosphate biosynthesis_Homo sapiens_R-HSA-73817
Lysine catabolism_Homo sapiens_R-HSA-71064
Gene Expression_Homo sapiens_R-HSA-74160
FGFR1 mutant receptor activation_Homo sapiens_R-HSA-1839124
NOD1/2 Signaling Pathway_Homo sapiens_R-HSA-168638
Metabolism of non-coding RNA_Homo sapiens_R-HSA-194441
snRNP Assembly_Homo sapiens_R-HSA-191859
Nucleotide Excision Repair_Homo sapiens_R-HSA-5696398
Activation of anterior HOX genes in hindbrain development during early embryogenesis_Homo sapiens_R-HSA[illegible]

Activation of HOX genes during differentiation_Homo sapiens_R-HSA-5619507
trans-Golgi Network Vesicle Budding_Homo sapiens_R-HSA-199992
Clathrin derived vesicle budding_Homo sapiens_R-HSA-421837
Nuclear Envelope Breakdown_Homo sapiens_R-HSA-2980766
Nuclear Receptor transcription pathway_Homo sapiens_R-HSA-383280
Vpr-mediated nuclear import of PICs_Homo sapiens_R-HSA-180910
RNA Polymerase I Transcription Termination_Homo sapiens_R-HSA-73863
Thrombin signalling through proteinase activated receptors (PARs)_Homo sapiens_R-HSA-456926
The activation of arylsulfatases_Homo sapiens_R-HSA-1663150
Mitotic Telophase/Cytokinesis_Homo sapiens_R-HSA-68884
Initiation of Nuclear Envelope Reformation_Homo sapiens_R-HSA-2995383
Nuclear Envelope Reassembly_Homo sapiens_R-HSA-2995410
TP53 Regulates Transcription of Genes Involved in G1 Cell Cycle Arrest_Homo sapiens_R-HSA-6804116
POU5F1 (OCT4), SOX2, NANOG activate genes related to proliferation_Homo sapiens_R-HSA-2892247
FGFRL1 modulation of FGFR1 signaling_Homo sapiens_R-HSA-5658623
p38MAPK events_Homo sapiens_R-HSA-171007
DEx/H-box helicases activate type I IFN and inflammatory cytokines production_Homo sapiens_R-HSA-313496
Platelet Adhesion to exposed collagen_Homo sapiens_R-HSA-75892
Signaling by Activin_Homo sapiens_R-HSA-1502540
Purine salvage_Homo sapiens_R-HSA-74217
Host Interactions of HIV factors_Homo sapiens_R-HSA-162909
mRNA Splicing - Minor Pathway_Homo sapiens_R-HSA-72165
Formation of the Early Elongation Complex_Homo sapiens_R-HSA-113418
Formation of the HIV-1 Early Elongation Complex_Homo sapiens_R-HSA-167158
Formation of TC-NER Pre-Incision Complex_Homo sapiens_R-HSA-6781823
G1/S Transition_Homo sapiens_R-HSA-69206
LGI-ADAM interactions_Homo sapiens_R-HSA-5682910
Organic cation/anion/zwitterion transport_Homo sapiens_R-HSA-549132
SUMOylation of transcription factors_Homo sapiens_R-HSA-3232118
Regulation of TP53 Activity through Association with Co-factors_Homo sapiens_R-HSA-6804759
TRAF3-dependent IRF activation pathway_Homo sapiens_R-HSA-918233
Regulation of necroptotic cell death_Homo sapiens_R-HSA-5675482
Bile salt and organic anion SLC transporters_Homo sapiens_R-HSA-425471
Repression of WNT target genes_Homo sapiens_R-HSA-4641265
Inhibition of adenylate cyclase pathway_Homo sapiens_R-HSA-997269
Adenylate cyclase inhibitory pathway_Homo sapiens_R-HSA-170670
Synthesis of Prostaglandins (PG) and Thromboxanes (TX)_Homo sapiens_R-HSA-2162123
Positive epigenetic regulation of rRNA expression_Homo sapiens_R-HSA-5250913
HSF1-dependent transactivation_Homo sapiens_R-HSA-3371571
Purine metabolism_Homo sapiens_R-HSA-73847
Golgi Associated Vesicle Biogenesis_Homo sapiens_R-HSA-432722
Sphingolipid metabolism_Homo sapiens_R-HSA-428157
The citric acid (TCA) cycle and respiratory electron transport_Homo sapiens_R-HSA-1428517
Interactions of Vpr with host cellular proteins_Homo sapiens_R-HSA-176033
Bile acid and bile salt metabolism_Homo sapiens_R-HSA-194068
Depolymerisation of the Nuclear Lamina_Homo sapiens_R-HSA-4419969
WNT5A-dependent internalization of FZD4_Homo sapiens_R-HSA-5099900
Regulation of innate immune responses to cytosolic DNA_Homo sapiens_R-HSA-3134975
Association of licensing factors with the pre-replicative complex_Homo sapiens_R-HSA-69298
Constitutive Signaling by NOTCH1 HD Domain Mutants_Homo sapiens_R-HSA-2691232
Signaling by NOTCH1 HD Domain Mutants in Cancer_Homo sapiens_R-HSA-2691230
NRIF signals cell death from the nucleus_Homo sapiens_R-HSA-205043
Glycogen breakdown (glycogenolysis)_Homo sapiens_R-HSA-70221

FGFR1 ligand binding and activation_Homo sapiens_R-HSA-190242
GRB2:SOS provides linkage to MAPK signaling for Integrins_Homo sapiens_R-HSA-354194
Eicosanoid ligand-binding receptors_Homo sapiens_R-HSA-391903
RNA Polymerase I Promoter Clearance_Homo sapiens_R-HSA-73854
RNA Polymerase I, RNA Polymerase III, and Mitochondrial Transcription_Homo sapiens_R-HSA-504046
Degradation of DVL_Homo sapiens_R-HSA-4641258
Meiotic synapsis_Homo sapiens_R-HSA-1221632
JNK (c-Jun kinases) phosphorylation and activation mediated by activated human TAK1_Homo sapiens_R-HS/
Regulated Necrosis_Homo sapiens_R-HSA-5218859
RIPK1-mediated regulated necrosis_Homo sapiens_R-HSA-5213460
Cell-extracellular matrix interactions_Homo sapiens_R-HSA-446353
Regulation of KIT signaling_Homo sapiens_R-HSA-1433559
RHO GTPases activate CIT_Homo sapiens_R-HSA-5625900
PKA activation_Homo sapiens_R-HSA-163615
Acyl chain remodelling of PI_Homo sapiens_R-HSA-1482922
Defective B3GALTL causes Peters-plus syndrome (PpS)_Homo sapiens_R-HSA-5083635
Activation of ATR in response to replication stress_Homo sapiens_R-HSA-176187
COPI-mediated anterograde transport_Homo sapiens_R-HSA-6807878
Metabolism_Homo sapiens_R-HSA-1430728
B-WICH complex positively regulates rRNA expression_Homo sapiens_R-HSA-5250924
Transport of inorganic cations/anions and amino acids/oligopeptides_Homo sapiens_R-HSA-425393
CLEC7A (Dectin-1) signaling_Homo sapiens_R-HSA-5607764
RNA Polymerase I Transcription_Homo sapiens_R-HSA-73864
Transport of vitamins, nucleosides, and related molecules_Homo sapiens_R-HSA-425397
O-glycosylation of TSR domain-containing proteins_Homo sapiens_R-HSA-5173214
DNA Damage Recognition in GG-NER_Homo sapiens_R-HSA-5696394
Stimuli-sensing channels_Homo sapiens_R-HSA-2672351
ABC transporters in lipid homeostasis_Homo sapiens_R-HSA-1369062
Trafficking of GluR2-containing AMPA receptors_Homo sapiens_R-HSA-416993
KSRP (KHSRP) binds and destabilizes mRNA_Homo sapiens_R-HSA-450604
Tristetraprolin (TTP, ZFP36) binds and destabilizes mRNA_Homo sapiens_R-HSA-450513
Butyrate Response Factor 1 (BRF1) binds and destabilizes mRNA_Homo sapiens_R-HSA-450385
Unblocking of NMDA receptor, glutamate binding and activation_Homo sapiens_R-HSA-438066
PKA activation in glucagon signalling_Homo sapiens_R-HSA-164378
Acyl chain remodelling of PG_Homo sapiens_R-HSA-1482925
Acyl chain remodelling of PS_Homo sapiens_R-HSA-1482801
Gene Silencing by RNA_Homo sapiens_R-HSA-211000
G alpha (s) signalling events_Homo sapiens_R-HSA-418555
Collagen degradation_Homo sapiens_R-HSA-1442490
Association of TriC/CCT with target proteins during biosynthesis_Homo sapiens_R-HSA-390471
Activation of SMO_Homo sapiens_R-HSA-5635838
Signaling by cytosolic FGFR1 fusion mutants_Homo sapiens_R-HSA-1839117
Tie2 Signaling_Homo sapiens_R-HSA-210993
Activation of IRF3/IRF7 mediated by TBK1/IKK epsilon_Homo sapiens_R-HSA-936964
CD22 mediated BCR regulation_Homo sapiens_R-HSA-5690714
Amine-derived hormones_Homo sapiens_R-HSA-209776
Cytosolic sulfonation of small molecules_Homo sapiens_R-HSA-156584
MHC class II antigen presentation_Homo sapiens_R-HSA-2132295
Diseases associated with O-glycosylation of proteins_Homo sapiens_R-HSA-3906995
Gap-filling DNA repair synthesis and ligation in TC-NER_Homo sapiens_R-HSA-6782210
FCERI mediated Ca+2 mobilization_Homo sapiens_R-HSA-2871809
Histidine, lysine, phenylalanine, tyrosine, proline and tryptophan catabolism_Homo sapiens_R-HSA-6788656
TP53 Regulates Transcription of Genes Involved in Cytochrome C Release_Homo sapiens_R-HSA-6803204

Phosphorylation of the APC/C_Homo sapiens_R-HSA-176412
Other semaphorin interactions_Homo sapiens_R-HSA-416700
Ephrin signaling_Homo sapiens_R-HSA-3928664
HDL-mediated lipid transport_Homo sapiens_R-HSA-194223
Citric acid cycle (TCA cycle)_Homo sapiens_R-HSA-71403
Collagen biosynthesis and modifying enzymes_Homo sapiens_R-HSA-1650814
Dual incision in TC-NER_Homo sapiens_R-HSA-6782135
Signaling by FGFR in disease_Homo sapiens_R-HSA-1226099
Collagen formation_Homo sapiens_R-HSA-1474290
Glucose transport_Homo sapiens_R-HSA-70153
Glucagon-like Peptide-1 (GLP1) regulates insulin secretion_Homo sapiens_R-HSA-381676
Transmembrane transport of small molecules_Homo sapiens_R-HSA-382551
Regulation of gene expression in beta cells_Homo sapiens_R-HSA-210745
Synthesis, secretion, and inactivation of Glucagon-like Peptide-1 (GLP-1)_Homo sapiens_R-HSA-381771
Signaling by Hippo_Homo sapiens_R-HSA-2028269
Synthesis of PC_Homo sapiens_R-HSA-1483191
HIV Transcription Elongation_Homo sapiens_R-HSA-167169
Tat-mediated elongation of the HIV-1 transcript_Homo sapiens_R-HSA-167246
Formation of HIV-1 elongation complex containing HIV-1 Tat_Homo sapiens_R-HSA-167200
Vasopressin regulates renal water homeostasis via Aquaporins_Homo sapiens_R-HSA-432040
Voltage gated Potassium channels_Homo sapiens_R-HSA-1296072
Platelet activation, signaling and aggregation_Homo sapiens_R-HSA-76002
Regulation of PLK1 Activity at G2/M Transition_Homo sapiens_R-HSA-2565942
Metabolism of lipids and lipoproteins_Homo sapiens_R-HSA-556833
Formation of HIV elongation complex in the absence of HIV Tat_Homo sapiens_R-HSA-167152
RNA Polymerase II Transcription Elongation_Homo sapiens_R-HSA-75955
Formation of RNA Pol II elongation complex_Homo sapiens_R-HSA-112382
RNA Polymerase II HIV Promoter Escape_Homo sapiens_R-HSA-167162
RNA Polymerase II Promoter Escape_Homo sapiens_R-HSA-73776
RNA Polymerase II Transcription Initiation And Promoter Clearance_Homo sapiens_R-HSA-76042
RNA Polymerase II Transcription Initiation_Homo sapiens_R-HSA-75953
RNA Polymerase II Transcription Pre-Initiation And Promoter Opening_Homo sapiens_R-HSA-73779
HIV Transcription Initiation_Homo sapiens_R-HSA-167161
Interleukin-1 signaling_Homo sapiens_R-HSA-446652
Nef-mediates down modulation of cell surface receptors by recruiting them to clathrin adapters_Homo sapiens_
TP53 regulates transcription of additional cell cycle genes whose exact role in the p53 pathway remain uncertai
SHC-mediated cascade:FGFR1_Homo sapiens_R-HSA-5654688
RIP-mediated NFkB activation via ZBP1_Homo sapiens_R-HSA-1810476
VxPx cargo-targeting to cilium_Homo sapiens_R-HSA-5620916
Synthesis of very long-chain fatty acyl-CoAs_Homo sapiens_R-HSA-75876
Respiratory electron transport_Homo sapiens_R-HSA-611105
O-linked glycosylation_Homo sapiens_R-HSA-5173105
Integrin cell surface interactions_Homo sapiens_R-HSA-216083
Macroautophagy_Homo sapiens_R-HSA-1632852
Regulation of TP53 Activity through Phosphorylation_Homo sapiens_R-HSA-6804756
RMTs methylate histone arginines_Homo sapiens_R-HSA-3214858
RA biosynthesis pathway_Homo sapiens_R-HSA-5365859
Intrinsic Pathway of Fibrin Clot Formation_Homo sapiens_R-HSA-140837
Signaling by FGFR3 point mutants in cancer_Homo sapiens_R-HSA-8853338
Signaling by FGFR3 in disease_Homo sapiens_R-HSA-5655332
Nephrin interactions_Homo sapiens_R-HSA-373753
SALM protein interactions at the synapse_Homo sapiens_R-HSA-8849932
Loss of Nlp from mitotic centrosomes_Homo sapiens_R-HSA-380259

Loss of proteins required for interphase microtubule organization?from the centrosome_Homo sapiens_R-HSA-
Regulation of TP53 Activity through Acetylation_Homo sapiens_R-HSA-6804758
RNA Polymerase III Transcription Termination_Homo sapiens_R-HSA-73980
MicroRNA (miRNA) biogenesis_Homo sapiens_R-HSA-203927
SHC-mediated cascade:FGFR4_Homo sapiens_R-HSA-5654719
APC/C:Cdc20 mediated degradation of Cyclin B_Homo sapiens_R-HSA-174048
Incretin synthesis, secretion, and inactivation_Homo sapiens_R-HSA-400508
Thromboxane signalling through TP receptor_Homo sapiens_R-HSA-428930
Acyl chain remodelling of PE_Homo sapiens_R-HSA-1482839
Nucleotide-binding domain, leucine rich repeat containing receptor (NLR) signaling pathways_Homo sapiens_R
Phase 1 - Functionalization of compounds_Homo sapiens_R-HSA-211945
HIV Infection_Homo sapiens_R-HSA-162906
DNA Repair_Homo sapiens_R-HSA-73894
Hexose transport_Homo sapiens_R-HSA-189200
Glutamate Neurotransmitter Release Cycle_Homo sapiens_R-HSA-210500
TRAF6 mediated NF-kB activation_Homo sapiens_R-HSA-933542
Insulin processing_Homo sapiens_R-HSA-264876
Costimulation by the CD28 family_Homo sapiens_R-HSA-388841
ISG15 antiviral mechanism_Homo sapiens_R-HSA-1169408
Antiviral mechanism by IFN-stimulated genes_Homo sapiens_R-HSA-1169410
AURKA Activation by TPX2_Homo sapiens_R-HSA-8854518
Amino acid and oligopeptide SLC transporters_Homo sapiens_R-HSA-425374
NoRC negatively regulates rRNA expression_Homo sapiens_R-HSA-427413
Antigen Presentation: Folding, assembly and peptide loading of class I MHC_Homo sapiens_R-HSA-983170
ATF4 activates genes_Homo sapiens_R-HSA-380994
Regulation of IFNA signaling_Homo sapiens_R-HSA-912694
Basigin interactions_Homo sapiens_R-HSA-210991
Acyl chain remodelling of PC_Homo sapiens_R-HSA-1482788
Cytokine Signaling in Immune system_Homo sapiens_R-HSA-1280215
Transport to the Golgi and subsequent modification_Homo sapiens_R-HSA-948021
Regulation of ornithine decarboxylase (ODC)_Homo sapiens_R-HSA-350562
Anchoring of the basal body to the plasma membrane_Homo sapiens_R-HSA-5620912
Vpu mediated degradation of CD4_Homo sapiens_R-HSA-180534
Cargo trafficking to the periciliary membrane_Homo sapiens_R-HSA-5620920
Aquaporin-mediated transport_Homo sapiens_R-HSA-445717
Termination of O-glycan biosynthesis_Homo sapiens_R-HSA-977068
PD-1 signaling_Homo sapiens_R-HSA-389948
FGFR2 alternative splicing_Homo sapiens_R-HSA-6803529
SHC-mediated cascade:FGFR2_Homo sapiens_R-HSA-5654699
TAK1 activates NFkB by phosphorylation and activation of IKKs complex_Homo sapiens_R-HSA-445989
WNT ligand biogenesis and trafficking_Homo sapiens_R-HSA-3238698
G alpha (12/13) signalling events_Homo sapiens_R-HSA-416482
Deposition of new CENPA-containing nucleosomes at the centromere_Homo sapiens_R-HSA-606279
Nucleosome assembly_Homo sapiens_R-HSA-774815
Negative epigenetic regulation of rRNA expression_Homo sapiens_R-HSA-5250941
Transcription-Coupled Nucleotide Excision Repair (TC-NER)_Homo sapiens_R-HSA-6781827
Keratan sulfate biosynthesis_Homo sapiens_R-HSA-2022854
Caspase activation via extrinsic apoptotic signalig pathway_Homo sapiens_R-HSA-5357769
Phase 1 - inactivation of fast Na+ channels_Homo sapiens_R-HSA-5576894
Vif-mediated degradation of APOBEC3G_Homo sapiens_R-HSA-180585
GPVI-mediated activation cascade_Homo sapiens_R-HSA-114604
RNA polymerase II transcribes snRNA genes_Homo sapiens_R-HSA-6807505
RHO GTPase Effectors_Homo sapiens_R-HSA-195258

PERK regulates gene expression_Homo sapiens_R-HSA-381042
Energy dependent regulation of mTOR by LKB1-AMPK_Homo sapiens_R-HSA-380972
Extension of Telomeres_Homo sapiens_R-HSA-180786
Adrenaline,noradrenaline inhibits insulin secretion_Homo sapiens_R-HSA-400042
Assembly of collagen fibrils and other multimeric structures_Homo sapiens_R-HSA-2022090
SCF-beta-TrCP mediated degradation of Emi1_Homo sapiens_R-HSA-174113
Regulation of APC/C activators between G1/S and early anaphase_Homo sapiens_R-HSA-176408
Centrosome maturation_Homo sapiens_R-HSA-380287
Recruitment of mitotic centrosome proteins and complexes_Homo sapiens_R-HSA-380270
Metabolism of vitamins and cofactors_Homo sapiens_R-HSA-196854
HSF1 activation_Homo sapiens_R-HSA-3371511
NEP/NS2 Interacts with the Cellular Export Machinery_Homo sapiens_R-HSA-168333
Regulation of Glucokinase by Glucokinase Regulatory Protein_Homo sapiens_R-HSA-170822
Termination of translesion DNA synthesis_Homo sapiens_R-HSA-5656169
Activation of the pre-replicative complex_Homo sapiens_R-HSA-68962
Activation of Kainate Receptors upon glutamate binding_Homo sapiens_R-HSA-451326
Glucagon-type ligand receptors_Homo sapiens_R-HSA-420092
Metabolism of steroid hormones_Homo sapiens_R-HSA-196071
Metabolism of proteins_Homo sapiens_R-HSA-392499
tRNA processing in the nucleus_Homo sapiens_R-HSA-6784531
Ribosomal scanning and start codon recognition_Homo sapiens_R-HSA-72702
Translation initiation complex formation_Homo sapiens_R-HSA-72649
RNA Polymerase I Chain Elongation_Homo sapiens_R-HSA-73777
Antigen processing-Cross presentation_Homo sapiens_R-HSA-1236975
DNA Replication Pre-Initiation_Homo sapiens_R-HSA-69002
M/G1 Transition_Homo sapiens_R-HSA-68874
Mitotic G2-G2/M phases_Homo sapiens_R-HSA-453274
Amino acid transport across the plasma membrane_Homo sapiens_R-HSA-352230
Trafficking of AMPA receptors_Homo sapiens_R-HSA-399719
Glutamate Binding, Activation of AMPA Receptors and Synaptic Plasticity_Homo sapiens_R-HSA-399721
Export of Viral Ribonucleoproteins from Nucleus_Homo sapiens_R-HSA-168274
Packaging Of Telomere Ends_Homo sapiens_R-HSA-171306
rRNA modification in the nucleus_Homo sapiens_R-HSA-6790901
Activation of the mRNA upon binding of the cap-binding complex and eIFs, and subsequent binding to 43S_Hor
NIK-->noncanonical NF-kB signaling_Homo sapiens_R-HSA-5676590
Metabolism of polyamines_Homo sapiens_R-HSA-351202
Class B/2 (Secretin family receptors)_Homo sapiens_R-HSA-373080
Mitochondrial translation elongation_Homo sapiens_R-HSA-5389840
Telomere Maintenance_Homo sapiens_R-HSA-157579
The role of GTSE1 in G2/M progression after G2 checkpoint_Homo sapiens_R-HSA-8852276
SCF(Skp2)-mediated degradation of p27/p21_Homo sapiens_R-HSA-187577
Cell Cycle_Homo sapiens_R-HSA-1640170
Keratan sulfate/keratin metabolism_Homo sapiens_R-HSA-1638074
Activation of Matrix Metalloproteinases_Homo sapiens_R-HSA-1592389
Regulation of beta-cell development_Homo sapiens_R-HSA-186712
Nuclear import of Rev protein_Homo sapiens_R-HSA-180746
DNA methylation_Homo sapiens_R-HSA-5334118
Gluconeogenesis_Homo sapiens_R-HSA-70263
TRAF6 mediated IRF7 activation_Homo sapiens_R-HSA-933541
Signal amplification_Homo sapiens_R-HSA-392518
Respiratory electron transport, ATP synthesis by chemiosmotic coupling, and heat production by uncoupling pro
Meiosis_Homo sapiens_R-HSA-1500620
APC/C-mediated degradation of cell cycle proteins_Homo sapiens_R-HSA-174143

Regulation of mitotic cell cycle_Homo sapiens_R-HSA-453276
SLC-mediated transmembrane transport_Homo sapiens_R-HSA-425407
RHO GTPases activate PKNs_Homo sapiens_R-HSA-5625740
HDACs deacetylate histones_Homo sapiens_R-HSA-3214815
CD28 co-stimulation_Homo sapiens_R-HSA-389356
Rev-mediated nuclear export of HIV RNA_Homo sapiens_R-HSA-165054
Glucagon signaling in metabolic regulation_Homo sapiens_R-HSA-163359
Detoxification of Reactive Oxygen Species_Homo sapiens_R-HSA-3299685
Chromosome Maintenance_Homo sapiens_R-HSA-73886
Regulation of mRNA stability by proteins that bind AU-rich elements_Homo sapiens_R-HSA-450531
TP53 Regulates Transcription of DNA Repair Genes_Homo sapiens_R-HSA-6796648
Dectin-1 mediated noncanonical NF-kB signaling_Homo sapiens_R-HSA-5607761
Cell death signalling via NRAGE, NRIF and NADE_Homo sapiens_R-HSA-204998
FCERI mediated NF-kB activation_Homo sapiens_R-HSA-2871837
Activated PKN1 stimulates transcription of AR (androgen receptor) regulated genes KLK2 and KLK3_Homo sap
Lipoprotein metabolism_Homo sapiens_R-HSA-174824
Synthesis of PIPs at the plasma membrane_Homo sapiens_R-HSA-1660499
Cytochrome P450 - arranged by substrate type_Homo sapiens_R-HSA-211897
Lysosome Vesicle Biogenesis_Homo sapiens_R-HSA-432720
Generation of second messenger molecules_Homo sapiens_R-HSA-202433
Interactions of Rev with host cellular proteins_Homo sapiens_R-HSA-177243
Base Excision Repair_Homo sapiens_R-HSA-73884
Resolution of Abasic Sites (AP sites)_Homo sapiens_R-HSA-73933
Fanconi Anemia Pathway_Homo sapiens_R-HSA-6783310
RNA Polymerase II Pre-transcription Events_Homo sapiens_R-HSA-674695
Hedgehog ligand biogenesis_Homo sapiens_R-HSA-5358346
Mitochondrial translation_Homo sapiens_R-HSA-5368287
ER-Phagosome pathway_Homo sapiens_R-HSA-1236974
Class A/1 (Rhodopsin-like receptors)_Homo sapiens_R-HSA-373076
HIV Life Cycle_Homo sapiens_R-HSA-162587
HDR through Single Strand Annealing (SSA)_Homo sapiens_R-HSA-5685938
Translesion synthesis by Y family DNA polymerases bypasses lesions on DNA template_Homo sapiens_R-HSA
NCAM1 interactions_Homo sapiens_R-HSA-419037
Steroid hormones_Homo sapiens_R-HSA-209943
O-linked glycosylation of mucins_Homo sapiens_R-HSA-913709
Activation of NF-kappaB in B cells_Homo sapiens_R-HSA-1169091
Triglyceride Biosynthesis_Homo sapiens_R-HSA-75109
Infectious disease_Homo sapiens_R-HSA-5663205
TCR signaling_Homo sapiens_R-HSA-202403
Interferon gamma signaling_Homo sapiens_R-HSA-877300
Transcription of the HIV genome_Homo sapiens_R-HSA-167172
Assembly of the pre-replicative complex_Homo sapiens_R-HSA-68867
Extracellular matrix organization_Homo sapiens_R-HSA-1474244
COPII (Coat Protein 2) Mediated Vesicle Transport_Homo sapiens_R-HSA-204005
Cyclin E associated events during G1/S transition_Homo sapiens_R-HSA-69202
Formation of Fibrin Clot (Clotting Cascade)_Homo sapiens_R-HSA-140877
Gamma carboxylation, hypusine formation and arylsulfatase activation_Homo sapiens_R-HSA-163841
Presynaptic phase of homologous DNA pairing and strand exchange_Homo sapiens_R-HSA-5693616
GABA B receptor activation_Homo sapiens_R-HSA-977444
Activation of GABAB receptors_Homo sapiens_R-HSA-991365
tRNA modification in the nucleus and cytosol_Homo sapiens_R-HSA-6782315
Cell Cycle, Mitotic_Homo sapiens_R-HSA-69278
Cyclin A:Cdk2-associated events at S phase entry_Homo sapiens_R-HSA-69656

PRC2 methylates histones and DNA_Homo sapiens_R-HSA-212300
Class C/3 (Metabotropic glutamate/pheromone receptors)_Homo sapiens_R-HSA-420499
Rho GTPase cycle_Homo sapiens_R-HSA-194840
Glycosphingolipid metabolism_Homo sapiens_R-HSA-1660662
Intraflagellar transport_Homo sapiens_R-HSA-5620924
RNA Polymerase III Abortive And Retractive Initiation_Homo sapiens_R-HSA-749476
RNA Polymerase III Transcription_Homo sapiens_R-HSA-74158
Metabolism of amino acids and derivatives_Homo sapiens_R-HSA-71291
G2/M Transition_Homo sapiens_R-HSA-69275
Cdc20:Phospho-APC/C mediated degradation of Cyclin A_Homo sapiens_R-HSA-174184
Lipid digestion, mobilization, and transport_Homo sapiens_R-HSA-73923
S Phase_Homo sapiens_R-HSA-69242
Downstream TCR signaling_Homo sapiens_R-HSA-202424
Resolution of Sister Chromatid Cohesion_Homo sapiens_R-HSA-2500257
Transcriptional regulation by small RNAs_Homo sapiens_R-HSA-5578749
APC:Cdc20 mediated degradation of cell cycle proteins prior to satisfation of the cell cycle checkpoint_Homo sa
Non-integrin membrane-ECM interactions_Homo sapiens_R-HSA-3000171
Viral Messenger RNA Synthesis_Homo sapiens_R-HSA-168325
Homologous DNA Pairing and Strand Exchange_Homo sapiens_R-HSA-5693579
Fatty Acyl-CoA Biosynthesis_Homo sapiens_R-HSA-75105
Biological oxidations_Homo sapiens_R-HSA-211859
Phase II conjugation_Homo sapiens_R-HSA-156580
TP53 Regulates Transcription of Cell Death Genes_Homo sapiens_R-HSA-5633008
ERCC6 (CSB) and EHMT2 (G9a) positively regulate rRNA expression_Homo sapiens_R-HSA-427389
Signaling by FGFR2 in disease_Homo sapiens_R-HSA-5655253
Classical antibody-mediated complement activation_Homo sapiens_R-HSA-173623
Iron uptake and transport_Homo sapiens_R-HSA-917937
APC/C:Cdc20 mediated degradation of mitotic proteins_Homo sapiens_R-HSA-176409
SUMOylation of DNA replication proteins_Homo sapiens_R-HSA-4615885
Activation of APC/C and APC/C:Cdc20 mediated degradation of mitotic proteins_Homo sapiens_R-HSA-17681
Regulation of DNA replication_Homo sapiens_R-HSA-69304
tRNA processing_Homo sapiens_R-HSA-72306
Signaling by Rho GTPases_Homo sapiens_R-HSA-194315
ABC-family proteins mediated transport_Homo sapiens_R-HSA-382556
NRAGE signals death through JNK_Homo sapiens_R-HSA-193648
ER to Golgi Anterograde Transport_Homo sapiens_R-HSA-199977
DNA Replication_Homo sapiens_R-HSA-69306
Platelet degranulation_Homo sapiens_R-HSA-114608
Degradation of the extracellular matrix_Homo sapiens_R-HSA-1474228
G2/M DNA damage checkpoint_Homo sapiens_R-HSA-69473
Mitotic Prometaphase_Homo sapiens_R-HSA-68877
Assembly of the primary cilium_Homo sapiens_R-HSA-5617833
Regulation of activated PAK-2p34 by proteasome mediated degradation_Homo sapiens_R-HSA-211733
Cross-presentation of soluble exogenous antigens (endosomes)_Homo sapiens_R-HSA-1236978
FCGR activation_Homo sapiens_R-HSA-2029481
Creation of C4 and C2 activators_Homo sapiens_R-HSA-166786
Complex I biogenesis_Homo sapiens_R-HSA-6799198
Processing of DNA double-strand break ends_Homo sapiens_R-HSA-5693607
GPCR ligand binding_Homo sapiens_R-HSA-500792
Response to elevated platelet cytosolic Ca2+_Homo sapiens_R-HSA-76005
Formation of the ternary complex, and subsequently, the 43S complex_Homo sapiens_R-HSA-72695
CDK-mediated phosphorylation and removal of Cdc6_Homo sapiens_R-HSA-69017
Ubiquitin-dependent degradation of Cyclin D1_Homo sapiens_R-HSA-69229

Ubiquitin-dependent degradation of Cyclin D_Homo sapiens_R-HSA-75815
Regulation of Apoptosis_Homo sapiens_R-HSA-169911
p75 NTR receptor-mediated signalling_Homo sapiens_R-HSA-193704
Neurotransmitter Release Cycle_Homo sapiens_R-HSA-112310
Autodegradation of the E3 ubiquitin ligase COP1_Homo sapiens_R-HSA-349425
G alpha (q) signalling events_Homo sapiens_R-HSA-416476
Mitochondrial translation initiation_Homo sapiens_R-HSA-5368286
Mitochondrial translation termination_Homo sapiens_R-HSA-5419276
Nonhomologous End-Joining (NHEJ)_Homo sapiens_R-HSA-5693571
p53-Independent DNA Damage Response_Homo sapiens_R-HSA-69610
p53-Independent G1/S DNA damage checkpoint_Homo sapiens_R-HSA-69613
Ubiquitin Mediated Degradation of Phosphorylated Cdc25A_Homo sapiens_R-HSA-69601
Cap-dependent Translation Initiation_Homo sapiens_R-HSA-72737
Eukaryotic Translation Initiation_Homo sapiens_R-HSA-72613
RHO GTPases Activate Formins_Homo sapiens_R-HSA-5663220
M Phase_Homo sapiens_R-HSA-68886
Arachidonic acid metabolism_Homo sapiens_R-HSA-2142753
Interferon Signaling_Homo sapiens_R-HSA-913531
Stabilization of p53_Homo sapiens_R-HSA-69541
AUF1 (hnRNP D0) binds and destabilizes mRNA_Homo sapiens_R-HSA-450408
Mitochondrial protein import_Homo sapiens_R-HSA-1268020
ECM proteoglycans_Homo sapiens_R-HSA-3000178
Hh mutants that don't undergo autocatalytic processing are degraded by ERAD_Homo sapiens_R-HSA-536276
Hh mutants abrogate ligand secretion_Homo sapiens_R-HSA-5387390
CDT1 association with the CDC6:ORC:origin complex_Homo sapiens_R-HSA-68827
Initial triggering of complement_Homo sapiens_R-HSA-166663
G2/M Checkpoints_Homo sapiens_R-HSA-69481
DNA Damage/Telomere Stress Induced Senescence_Homo sapiens_R-HSA-2559586
PI Metabolism_Homo sapiens_R-HSA-1483255
TNFR2 non-canonical NF-kB pathway_Homo sapiens_R-HSA-5668541
Chaperonin-mediated protein folding_Homo sapiens_R-HSA-390466
Autodegradation of Cdh1 by Cdh1:APC/C_Homo sapiens_R-HSA-174084
Late Phase of HIV Life Cycle_Homo sapiens_R-HSA-162599
p53-Dependent G1/S DNA damage checkpoint_Homo sapiens_R-HSA-69580
p53-Dependent G1 DNA Damage Response_Homo sapiens_R-HSA-69563
HDR through Homologous Recombination (HRR)_Homo sapiens_R-HSA-5685942
Potassium Channels_Homo sapiens_R-HSA-1296071
G1/S DNA Damage Checkpoints_Homo sapiens_R-HSA-69615
Transport of glucose and other sugars, bile salts and organic acids, metal ions and amine compounds_Homo sa
Protein folding_Homo sapiens_R-HSA-391251
APC/C:Cdc20 mediated degradation of Securin_Homo sapiens_R-HSA-174154
Amyloid fiber formation_Homo sapiens_R-HSA-977225
Interferon alpha/beta signaling_Homo sapiens_R-HSA-909733
Peptide ligand-binding receptors_Homo sapiens_R-HSA-375276
Nonsense-Mediated Decay (NMD)_Homo sapiens_R-HSA-927802
Nonsense Mediated Decay (NMD) enhanced by the Exon Junction Complex (EJC)_Homo sapiens_R-HSA-975
L13a-mediated translational silencing of Ceruloplasmin expression_Homo sapiens_R-HSA-156827
3' -UTR-mediated translational regulation_Homo sapiens_R-HSA-157279
Switching of origins to a post-replicative state_Homo sapiens_R-HSA-69052
Orc1 removal from chromatin_Homo sapiens_R-HSA-68949
GTP hydrolysis and joining of the 60S ribosomal subunit_Homo sapiens_R-HSA-72706
APC/C:Cdh1 mediated degradation of Cdc20 and other APC/C:Cdh1 targeted proteins in late mitosis/early G1_
Removal of licensing factors from origins_Homo sapiens_R-HSA-69300

Binding and Uptake of Ligands by Scavenger Receptors_Homo sapiens_R-HSA-2173782
HDR through Homologous Recombination (HR) or Single Strand Annealing (SSA)_Homo sapiens_R-HSA-5693
DNA Double-Strand Break Repair_Homo sapiens_R-HSA-5693532
Senescence-Associated Secretory Phenotype (SASP)_Homo sapiens_R-HSA-2559582
Influenza Infection_Homo sapiens_R-HSA-168254
Complement cascade_Homo sapiens_R-HSA-166658
Homology Directed Repair_Homo sapiens_R-HSA-5693538
Translation_Homo sapiens_R-HSA-72766
Cell Cycle Checkpoints_Homo sapiens_R-HSA-69620
G alpha (i) signalling events_Homo sapiens_R-HSA-418594
Separation of Sister Chromatids_Homo sapiens_R-HSA-2467813
Major pathway of rRNA processing in the nucleolus_Homo sapiens_R-HSA-6791226
Influenza Life Cycle_Homo sapiens_R-HSA-168255
Formation of a pool of free 40S subunits_Homo sapiens_R-HSA-72689
Synthesis of DNA_Homo sapiens_R-HSA-69239
Mitotic Anaphase_Homo sapiens_R-HSA-68882
Metabolism of water-soluble vitamins and cofactors_Homo sapiens_R-HSA-196849
Mitotic Metaphase and Anaphase_Homo sapiens_R-HSA-2555396
rRNA processing_Homo sapiens_R-HSA-72312
Selenoamino acid metabolism_Homo sapiens_R-HSA-2408522
Signaling by GPCR_Homo sapiens_R-HSA-372790
Influenza Viral RNA Transcription and Replication_Homo sapiens_R-HSA-168273
Immunoregulatory interactions between a Lymphoid and a non-Lymphoid cell_Homo sapiens_R-HSA-198933
GPCR downstream signaling_Homo sapiens_R-HSA-388396

Overlap	P.value	Adjusted.P.value	Old.P.value	Old.Adjusted
11/29	9,59953E-08	0,000146873	0	0
68/786	2,05984E-05	0,015757742	0	0
9/34	4,07576E-05	0,020786399	0	0
13/72	7,07991E-05	0,027080665	0	0
47/515	0,000112274	0,034355824	0	0
9/39	0,000130505	0,033278897	0	0
31/295	0,000142116	0,031062405	0	0
17/122	0,000175777	0,033617444	0	0
23/199	0,000254983	0,043347071	0	0
29/288	0,000468209	0,071636019	0	0
5/15	0,000704559	0,097997719	0	0
5/15	0,000704559	0,089831243	0	0
5/15	0,000704559	0,082921147	0	0
5/15	0,000704559	0,076998208	0	0
5/15	0,000704559	0,071864994	0	0
5/15	0,000704559	0,067373432	0	0
9/49	0,000796304	0,071667371	0	0
9/49	0,000796304	0,06768585	0	0
7/31	0,00084304	0,06788687	0	0
8/40	0,000860022	0,06579167	0	0
5/16	0,000981516	0,071510417	0	0
4/10	0,00115204	0,080119125	0	0
8/42	0,001204829	0,080147329	0	0
5/17	0,001331814	0,084903164	0	0
32/355	0,001612009	0,098654929	0	0
7/35	0,001801029	0,105983604	0	0
33/374	0,001948574	0,110419217	0	0
25/260	0,00211634	0,115642858	0	0
6/27	0,002162854	0,114109215	0	0
6/27	0,002162854	0,110305574	0	0
9/57	0,002412653	0,119076115	0	0
9/57	0,002412653	0,115354986	0	0
9/57	0,002412653	0,111859381	0	0
9/57	0,002412653	0,108569399	0	0
9/57	0,002412653	0,105467416	0	0
3/6	0,002435749	0,103519319	0	0
4/12	0,002499253	0,103347496	0	0
4/12	0,002499253	0,100627825	0	0
8/47	0,002556811	0,100305645	0	0
6/28	0,002634198	0,100758083	0	0
26/284	0,00342238	0,127713194	0	0
7/39	0,003443716	0,125449667	0	0
29/330	0,003747459	0,133339809	0	0
12/96	0,003834924	0,133350763	0	0
4/14	0,004653013	0,158202436	0	0
4/14	0,004653013	0,154763252	0	0
4/14	0,004653013	0,151470417	0	0
4/14	0,004653013	0,148314784	0	0
4/14	0,004653013	0,145287951	0	0
4/14	0,004653013	0,142382192	0	0
9/63	0,004821424	0,144642714	0	0
11/87	0,005053796	0,14869822	0	0

18/179	0,005269752	0,152126804	0	0
7/42	0,005283161	0,14968957	0	0
5/23	0,005598899	0,155751184	0	0
5/23	0,005598899	0,152969913	0	0
21/226	0,006773382	0,181811842	0	0
21/226	0,006773382	0,178677155	0	0
13/116	0,006838018	0,177324882	0	0
8/55	0,006892506	0,175758902	0	0
4/16	0,007791259	0,1954201	0	0
26/305	0,008561833	0,211283955	0	0
3/9	0,009102597	0,221063081	0	0
3/9	0,009102597	0,21760897	0	0
5/26	0,009632009	0,226722671	0	0
5/26	0,009632009	0,223287479	0	0
5/26	0,009632009	0,21995483	0	0
13/122	0,010303907	0,231837911	0	0
13/122	0,010303907	0,228477942	0	0
13/122	0,010303907	0,225213971	0	0
13/122	0,010303907	0,222041943	0	0
13/122	0,010303907	0,218958027	0	0
13/122	0,010303907	0,215958602	0	0
35/450	0,010511029	0,217322633	0	0
28/341	0,010608578	0,216414983	0	0
27/329	0,012017666	0,241934597	0	0
27/329	0,012017666	0,238792589	0	0
27/329	0,012017666	0,235731146	0	0
4/18	0,012068057	0,233723138	0	0
13/125	0,012484866	0,238773056	0	0
13/125	0,012484866	0,23582524	0	0
3/10	0,01250999	0,233418101	0	0
27/332	0,013411182	0,247218165	0	0
27/332	0,013411182	0,244275092	0	0
12/113	0,013730089	0,247141597	0	0
29/364	0,013852569	0,246446864	0	0
27/333	0,013903551	0,244510723	0	0
24/288	0,014492534	0,251972463	0	0
24/288	0,014492534	0,249141311	0	0
24/288	0,014492534	0,246373074	0	0
7/51	0,015207159	0,255680807	0	0
27/336	0,015468259	0,257243871	0	0
3/11	0,016549986	0,272273967	0	0
5/30	0,017613657	0,286690379	0	0
5/30	0,017613657	0,283672585	0	0
6/41	0,01782629	0,284106503	0	0
6/42	0,019916815	0,314151829	0	0
9/79	0,020260427	0,316310753	0	0
27/344	0,02033455	0,314261225	0	0
4/21	0,02088453	0,319533305	0	0
4/21	0,02088453	0,316369609	0	0
28/361	0,021148315	0,317224724	0	0
13/134	0,021191986	0,314793574	0	0
23/284	0,022186118	0,326391926	0	0
11/107	0,022196407	0,323433366	0	0

17/193	0,022308289	0,321997001	0	0
5/32	0,022876958	0,327119119	0	0
5/32	0,022876958	0,324090238	0	0
5/32	0,022876958	0,321116933	0	0
2/5	0,02396506	0,333332192	0	0
52/762	0,024356391	0,335723234	0	0
6/44	0,024584124	0,335836689	0	0
6/44	0,024584124	0,332864683	0	0
23/287	0,024703702	0,331549681	0	0
28/366	0,024771108	0,329563434	0	0
20/241	0,025023875	0,330056277	0	0
3/13	0,026564546	0,347382527	0	0
3/13	0,026564546	0,344438607	0	0
3/13	0,026564546	0,341544165	0	0
3/13	0,026564546	0,338697964	0	0
3/13	0,026564546	0,335898807	0	0
3/13	0,026564546	0,333145538	0	0
6/45	0,02717002	0,337968542	0	0
5/34	0,029062326	0,358591602	0	0
5/34	0,029062326	0,355722869	0	0
31/420	0,029259059	0,35528857	0	0
39/552	0,02948755	0,355243713	0	0
25/325	0,030707511	0,367050722	0	0
8/72	0,031653983	0,37543096	0	0
3/14	0,03254043	0,382975829	0	0
3/14	0,03254043	0,380052349	0	0
3/14	0,03254043	0,377173165	0	0
27/359	0,032621646	0,375271562	0	0
4/24	0,032833178	0,374886283	0	0
6/47	0,032866475	0,372486716	0	0
7/60	0,03415701	0,384266363	0	0
7/60	0,03415701	0,381461499	0	0
2/6	0,034729741	0,385047127	0	0
13/144	0,035463137	0,390349636	0	0
6/48	0,035984006	0,39325378	0	0
29/395	0,036158026	0,392353053	0	0
7/61	0,03693506	0,397962266	0	0
4/25	0,03754088	0,401661169	0	0
4/25	0,03754088	0,398871855	0	0
3/15	0,039153343	0,413135279	0	0
3/15	0,039153343	0,410305585	0	0
3/15	0,039153343	0,40751439	0	0
5/37	0,040155428	0,415120301	0	0
14/162	0,04097051	0,420703892	0	0
9/90	0,042399895	0,432478928	0	0
5/38	0,044350814	0,449382418	0	0
5/38	0,044350814	0,446425954	0	0
5/38	0,044350814	0,443508138	0	0
3/16	0,046390758	0,460895194	0	0
3/16	0,046390758	0,457921676	0	0
3/16	0,046390758	0,454986281	0	0
3/16	0,046390758	0,452088279	0	0
12/135	0,046573515	0,450996696	0	0

2/7	0,04698191	0,452090074	0	0
2/7	0,04698191	0,449264511	0	0
2/7	0,04698191	0,446474049	0	0
16/196	0,047265279	0,4463943	0	0
4/27	0,048056222	0,451079871	0	0
4/27	0,048056222	0,448329384	0	0
11/121	0,048243236	0,447346371	0	0
20/261	0,050808631	0,468296416	0	0
23/311	0,053405767	0,489286368	0	0
4/28	0,053862291	0,490531576	0	0
3/17	0,054236191	0,491014035	0	0
3/17	0,054236191	0,488125718	0	0
24/328	0,054472313	0,487383855	0	0
9/95	0,056429679	0,501961675	0	0
7/67	0,056707536	0,501517512	0	0
7/67	0,056707536	0,498635228	0	0
8/81	0,057143326	0,499595935	0	0
18/233	0,057704343	0,501634347	0	0
4/29	0,060030377	0,518906649	0	0
4/29	0,060030377	0,515991443	0	0
4/29	0,060030377	0,513108809	0	0
4/29	0,060030377	0,510258204	0	0
2/8	0,060539704	0,511744462	0	0
2/8	0,060539704	0,508932679	0	0
2/8	0,060539704	0,506151626	0	0
8/82	0,060596989	0,50387714	0	0
8/82	0,060596989	0,50115348	0	0
8/82	0,060596989	0,498459106	0	0
8/82	0,060596989	0,495793549	0	0
3/18	0,062669827	0,510025722	0	0
3/18	0,062669827	0,507327173	0	0
9/97	0,062791752	0,505638847	0	0
9/97	0,062791752	0,502991523	0	0
9/97	0,062791752	0,500371775	0	0
6/55	0,06305942	0,499901101	0	0
47/725	0,063605857	0,501633821	0	0
12/142	0,063641743	0,499342904	0	0
18/236	0,063661452	0,496949087	0	0
18/236	0,063661452	0,494426502	0	0
18/236	0,063661452	0,491929399	0	0
18/236	0,063661452	0,489457392	0	0
8/83	0,064178868	0,490968339	0	0
8/83	0,064178868	0,48852571	0	0
19/253	0,06552082	0,496271557	0	0
4/30	0,066556032	0,501629209	0	0
23/320	0,068705671	0,51529253	0	0
5/43	0,069121735	0,515884167	0	0
18/239	0,07002669	0,520101147	0	0
3/19	0,071669081	0,529727992	0	0
8/85	0,071729411	0,527624995	0	0
8/85	0,071729411	0,525100473	0	0
15/192	0,072333242	0,526999333	0	0
6/57	0,072520364	0,525858562	0	0

4/31	0,073433514	0,529968287	0	0
10/115	0,073477619	0,527796984	0	0
5/44	0,074831689	0,53501161	0	0
5/44	0,074831689	0,532523184	0	0
2/9	0,07523604	0,532921947	0	0
2/9	0,07523604	0,530466086	0	0
2/9	0,07523604	0,528032755	0	0
2/9	0,07523604	0,525621647	0	0
8/86	0,075698728	0,526450248	0	0
11/131	0,076214522	0,527638997	0	0
18/242	0,076808017	0,529352549	0	0
9/101	0,076834299	0,527159095	0	0
18/243	0,079162089	0,540705337	0	0
4/32	0,080655885	0,548460019	0	0
4/32	0,080655885	0,546033204	0	0
4/32	0,080655885	0,543627772	0	0
5/45	0,080789402	0,542139408	0	0
5/45	0,080789402	0,539771987	0	0
7/73	0,081992234	0,545426599	0	0
8/88	0,084025832	0,55653473	0	0
18/246	0,086508031	0,57050555	0	0
5/46	0,086991823	0,571233862	0	0
6/60	0,088147657	0,576350065	0	0
6/60	0,088147657	0,573897511	0	0
6/60	0,088147657	0,571465742	0	0
2/10	0,090917569	0,586936205	0	0
2/10	0,090917569	0,584470086	0	0
2/10	0,090917569	0,582024605	0	0
2/10	0,090917569	0,579599502	0	0
2/10	0,090917569	0,577194525	0	0
2/10	0,090917569	0,574809423	0	0
2/10	0,090917569	0,572443953	0	0
2/10	0,090917569	0,570097871	0	0
3/21	0,0912632	0,569929372	0	0
3/21	0,0912632	0,567612586	0	0
3/21	0,0912632	0,56531456	0	0
24/348	0,091567171	0,564910371	0	0
6/61	0,093734489	0,575958907	0	0
6/61	0,093734489	0,573655071	0	0
19/266	0,095509446	0,582189054	0	0
4/34	0,096102152	0,583477354	0	0
16/217	0,096559299	0,583935684	0	0
8/91	0,097483801	0,587205573	0	0
5/48	0,10011571	0,600694262	0	0
17/235	0,101172884	0,604666066	0	0
17/235	0,101172884	0,60231328	0	0
17/235	0,101172884	0,599978733	0	0
17/235	0,101172884	0,597662212	0	0
17/235	0,101172884	0,595363512	0	0
3/22	0,101803296	0,596777941	0	0
8/92	0,102225566	0,596966092	0	0
8/92	0,102225566	0,594696259	0	0
8/92	0,102225566	0,592443622	0	0

8/92	0,102225566	0,590207986	0	0
8/92	0,102225566	0,587989159	0	0
18/252	0,102489181	0,587297554	0	0
32/492	0,10617464	0,606146264	0	0
5/49	0,107028285	0,608748236	0	0
7/78	0,107292669	0,607991789	0	0
2/11	0,107443713	0,606601038	0	0
2/11	0,107443713	0,604370887	0	0
2/11	0,107443713	0,602157074	0	0
2/11	0,107443713	0,59995942	0	0
2/11	0,107443713	0,59777775	0	0
2/11	0,107443713	0,595611888	0	0
2/11	0,107443713	0,593461665	0	0
2/11	0,107443713	0,591326911	0	0
11/141	0,112708147	0,618076934	0	0
3/23	0,112800229	0,616372679	0	0
7/79	0,112802756	0,614192942	0	0
17/239	0,113128264	0,613781009	0	0
5/50	0,114167849	0,61723254	0	0
9/110	0,114899009	0,618998182	0	0
9/110	0,114899009	0,616826259	0	0
17/240	0,116245266	0,621871528	0	0
140/2465	0,116574771	0,621461323	0	0
5/51	0,12152875	0,645621486	0	0
5/51	0,12152875	0,643387502	0	0
5/51	0,12152875	0,641168924	0	0
4/37	0,121626858	0,639481421	0	0
8/96	0,122446389	0,641585532	0	0
3/24	0,1242241	0,648678746	0	0
3/24	0,1242241	0,646472356	0	0
3/24	0,1242241	0,644280924	0	0
9/112	0,124554191	0,643810515	0	0
2/12	0,124685754	0,642320548	0	0
2/12	0,124685754	0,64016511	0	0
2/12	0,124685754	0,63802409	0	0
2/12	0,124685754	0,635897343	0	0
2/12	0,124685754	0,633784727	0	0
2/12	0,124685754	0,631686102	0	0
2/12	0,124685754	0,62960133	0	0
11/144	0,125320565	0,630725213	0	0
17/245	0,132593866	0,665143	0	0
3/25	0,136044545	0,680222723	0	0
3/25	0,136044545	0,678007014	0	0
3/25	0,136044545	0,675805692	0	0
12/163	0,137191322	0,679296837	0	0
7/84	0,142499285	0,703302922	0	0
2/13	0,142525984	0,701172846	0	0
2/13	0,142525984	0,698925498	0	0
2/13	0,142525984	0,696692509	0	0
2/13	0,142525984	0,694473743	0	0
2/13	0,142525984	0,692269064	0	0
2/13	0,142525984	0,690078339	0	0
2/13	0,142525984	0,687901436	0	0

19/282	0,142821502	0,687160059	0	0
17/248	0,143007195	0,685896577	0	0
5/54	0,144876791	0,692692157	0	0
3/26	0,148230982	0,706521505	0	0
3/26	0,148230982	0,704327339	0	0
3/26	0,148230982	0,702146759	0	0
3/26	0,148230982	0,699979639	0	0
3/26	0,148230982	0,697825856	0	0
3/26	0,148230982	0,695685286	0	0
3/26	0,148230982	0,693557808	0	0
3/26	0,148230982	0,691443302	0	0
7/85	0,148850429	0,692222359	0	0
4/40	0,149700503	0,694065967	0	0
4/40	0,149700503	0,691969091	0	0
4/40	0,149700503	0,689884847	0	0
12/166	0,150423863	0,69113667	0	0
8/101	0,150436148	0,689123671	0	0
7/86	0,155332227	0,709427781	0	0
8/102	0,156377792	0,712077448	0	0
11/151	0,157618711	0,715598301	0	0
3/27	0,160752832	0,727668146	0	0
3/27	0,160752832	0,725521632	0	0
2/14	0,160856918	0,723856133	0	0
2/14	0,160856918	0,721733388	0	0
2/14	0,160856918	0,719623056	0	0
2/14	0,160856918	0,71752503	0	0
2/14	0,160856918	0,715439201	0	0
10/136	0,165162226	0,732458568	0	0
19/289	0,167078263	0,738814285	0	0
4/42	0,169660024	0,748068692	0	0
4/42	0,169660024	0,74591907	0	0
48/807	0,169943567	0,745024807	0	0
3/28	0,173579701	0,758791265	0	0
5/58	0,178696931	0,778935342	0	0
9/122	0,178906143	0,777631816	0	0
2/15	0,179580553	0,77835197	0	0
2/15	0,179580553	0,776153236	0	0
2/15	0,179580553	0,773966889	0	0
2/15	0,179580553	0,771792825	0	0
2/15	0,179580553	0,76963094	0	0
2/15	0,179580553	0,767481133	0	0
2/15	0,179580553	0,765343302	0	0
2/15	0,179580553	0,763217349	0	0
2/15	0,179580553	0,761103173	0	0
2/15	0,179580553	0,759000678	0	0
6/74	0,182085029	0,767465825	0	0
3/29	0,18668155	0,784677942	0	0
6/75	0,189948359	0,796221887	0	0
4/44	0,190485643	0,796292442	0	0
4/44	0,190485643	0,794122708	0	0
2/16	0,198607672	0,825732984	0	0
2/16	0,198607672	0,823495225	0	0
2/16	0,198607672	0,821269562	0	0

14/211	0,202212645	0,83392277	0	0
4/46	0,212063518	0,872196726	0	0
3/31	0,213592618	0,876130579	0	0
3/31	0,213592618	0,873787983	0	0
3/31	0,213592618	0,871457882	0	0
2/17	0,217857207	0,886493422	0	0
2/17	0,217857207	0,88414198	0	0
2/17	0,217857207	0,88180298	0	0
2/17	0,217857207	0,879476323	0	0
2/17	0,217857207	0,877161912	0	0
2/17	0,217857207	0,87485965	0	0
24/392	0,220853436	0,884570043	0	0
4/47	0,2230987	0,891229793	0	0
5/63	0,224585398	0,894832446	0	0
5/63	0,224585398	0,892508206	0	0
3/32	0,227344696	0,90113312	0	0
3/32	0,227344696	0,898804611	0	0
6/80	0,231069631	0,911176639	0	0
1/5	0,232733649	0,915379134	0	0
1/5	0,232733649	0,913032008	0	0
1/5	0,232733649	0,910696888	0	0
1/5	0,232733649	0,908373682	0	0
1/5	0,232733649	0,906062298	0	0
1/5	0,232733649	0,903762648	0	0
1/5	0,232733649	0,901474641	0	0
1/5	0,232733649	0,89919819	0	0
1/5	0,232733649	0,896933207	0	0
1/5	0,232733649	0,894679606	0	0
1/5	0,232733649	0,892437301	0	0
1/5	0,232733649	0,890206208	0	0
1/5	0,232733649	0,887986242	0	0
1/5	0,232733649	0,885777321	0	0
1/5	0,232733649	0,883579363	0	0
1/5	0,232733649	0,881392285	0	0
1/5	0,232733649	0,879216008	0	0
1/5	0,232733649	0,877050451	0	0
7/97	0,234254533	0,880612863	0	0
4/48	0,234278972	0,878546146	0	0
2/18	0,237255632	0,887533294	0	0
2/18	0,237255632	0,885368579	0	0
2/18	0,237255632	0,883214398	0	0
3/33	0,241257655	0,895932555	0	0
3/33	0,241257655	0,893763227	0	0
3/33	0,241257655	0,891604378	0	0
3/33	0,241257655	0,889455934	0	0
3/33	0,241257655	0,887317819	0	0
3/33	0,241257655	0,885189958	0	0
6/82	0,248252399	0,908675047	0	0
13/203	0,250365165	0,914221245	0	0
5/66	0,253638584	0,923969129	0	0
3/34	0,255304963	0,927830386	0	0
3/34	0,255304963	0,925631736	0	0
3/34	0,255304963	0,923443482	0	0

3/34	0,255304963	0,921265549	0	0
3/34	0,255304963	0,919097865	0	0
3/34	0,255304963	0,916940358	0	0
2/19	0,256736405	0,919922012	0	0
2/19	0,256736405	0,917772661	0	0
2/19	0,256736405	0,915633331	0	0
2/19	0,256736405	0,913503951	0	0
2/19	0,256736405	0,911384453	0	0
2/19	0,256736405	0,909274766	0	0
2/19	0,256736405	0,907174825	0	0
4/50	0,257018171	0,906077883	0	0
16/259	0,26434221	0,929755361	0	0
4/51	0,268549185	0,942385902	0	0
9/136	0,269256615	0,942706228	0	0
3/35	0,269461014	0,941267927	0	0
1/6	0,272334438	0,949138246	0	0
1/6	0,272334438	0,946981114	0	0
1/6	0,272334438	0,944833764	0	0
1/6	0,272334438	0,942696132	0	0
1/6	0,272334438	0,940568149	0	0
1/6	0,272334438	0,938449753	0	0
1/6	0,272334438	0,936340877	0	0
1/6	0,272334438	0,934241458	0	0
1/6	0,272334438	0,932151432	0	0
1/6	0,272334438	0,930070737	0	0
1/6	0,272334438	0,92799931	0	0
1/6	0,272334438	0,925937089	0	0
1/6	0,272334438	0,923884014	0	0
5/68	0,27349548	0,925770098	0	0
2/20	0,276239439	0,932994133	0	0
2/20	0,276239439	0,930939079	0	0
2/20	0,276239439	0,92889306	0	0
3/36	0,283701185	0,951892134	0	0
3/36	0,283701185	0,949809219	0	0
4/53	0,29186584	0,975010338	0	0
6/87	0,292627078	0,975423592	0	0
30/521	0,292687632	0,973504516	0	0
2/21	0,295710622	0,981425708	0	0
2/21	0,295710622	0,97930141	0	0
2/21	0,295710622	0,977186288	0	0
9/140	0,297319001	0,980383776	0	0
3/37	0,29800186	0,980522249	0	0
6/88	0,301696743	0,990549394	0	0
8/123	0,302097423	0,989741022	0	0
4/54	0,303624962	0,992620069	0	0
8/124	0,309766268	1	0	0
1/7	0,309893194	1	0	0
1/7	0,309893194	1	0	0
1/7	0,309893194	1	0	0
1/7	0,309893194	1	0	0
1/7	0,309893194	1	0	0
1/7	0,309893194	0,998182288	0	0
1/7	0,309893194	0,996085267	0	0

1/7	0,309893194	0,993997038	0	0
1/7	0,309893194	0,991917546	0	0
1/7	0,309893194	0,989846737	0	0
1/7	0,309893194	0,987784556	0	0
1/7	0,309893194	0,98573095	0	0
1/7	0,309893194	0,983685865	0	0
1/7	0,309893194	0,981649248	0	0
1/7	0,309893194	0,979621047	0	0
1/7	0,309893194	0,97760121	0	0
3/38	0,312340461	0,983294044	0	0
3/38	0,312340461	0,98127496	0	0
7/107	0,315004196	0,987615615	0	0
2/22	0,315101353	0,985899938	0	0
2/22	0,315101353	0,983887898	0	0
4/55	0,315434093	0,9829209	0	0
4/55	0,315434093	0,980923094	0	0
10/161	0,319390401	0,991211591	0	0
84/1547	0,325697023	1	0	0
3/39	0,326695464	1	0	0
3/39	0,326695464	1	0	0
3/39	0,326695464	1	0	0
6/91	0,329187318	1	0	0
45/812	0,330180729	1	0	0
2/23	0,334368124	1	0	0
2/23	0,334368124	1	0	0
21/367	0,343997772	1	0	0
1/8	0,345515118	1	0	0
1/8	0,345515118	1	0	0
1/8	0,345515118	1	0	0
1/8	0,345515118	1	0	0
1/8	0,345515118	1	0	0
1/8	0,345515118	1	0	0
1/8	0,345515118	1	0	0
1/8	0,345515118	1	0	0
1/8	0,345515118	1	0	0
1/8	0,345515118	1	0	0
1/8	0,345515118	1	0	0
1/8	0,345515118	1	0	0
1/8	0,345515118	1	0	0
1/8	0,345515118	1	0	0
1/8	0,345515118	1	0	0
1/8	0,345515118	1	0	0
1/8	0,345515118	1	0	0
2/24	0,353472123	1	0	0
2/24	0,353472123	1	0	0
2/24	0,353472123	1	0	0
2/24	0,353472123	1	0	0
3/41	0,355373883	1	0	0
3/41	0,355373883	1	0	0
3/41	0,355373883	1	0	0
3/41	0,355373883	1	0	0
4/59	0,362926182	1	0	0
4/59	0,362926182	1	0	0

4/59	0,362926182	1	0	0
3/42	0,369659561	1	0	0
3/42	0,369659561	1	0	0
3/42	0,369659561	1	0	0
3/42	0,369659561	1	0	0
2/25	0,372378865	1	0	0
2/25	0,372378865	1	0	0
2/25	0,372378865	1	0	0
2/25	0,372378865	1	0	0
6/96	0,375594271	1	0	0
1/9	0,379299997	1	0	0
1/9	0,379299997	1	0	0
1/9	0,379299997	1	0	0
1/9	0,379299997	1	0	0
1/9	0,379299997	1	0	0
1/9	0,379299997	1	0	0
1/9	0,379299997	1	0	0
1/9	0,379299997	1	0	0
1/9	0,379299997	1	0	0
1/9	0,379299997	1	0	0
1/9	0,379299997	1	0	0
3/43	0,383886151	1	0	0
24/432	0,384032586	1	0	0
6/97	0,384913372	1	0	0
17/301	0,386005728	1	0	0
5/79	0,386449512	1	0	0
5/79	0,386449512	1	0	0
2/26	0,39105785	1	0	0
2/26	0,39105785	1	0	0
2/26	0,39105785	1	0	0
5/80	0,396808168	1	0	0
3/44	0,398037404	1	0	0
6/99	0,403546277	1	0	0
5/81	0,407149855	1	0	0
5/81	0,407149855	1	0	0
2/27	0,409482246	1	0	0
2/27	0,409482246	1	0	0
2/27	0,409482246	1	0	0
2/27	0,409482246	1	0	0
2/27	0,409482246	1	0	0
2/27	0,409482246	1	0	0
2/27	0,409482246	1	0	0
1/10	0,411342481	1	0	0
1/10	0,411342481	1	0	0
1/10	0,411342481	1	0	0
1/10	0,411342481	1	0	0
1/10	0,411342481	1	0	0
1/10	0,411342481	1	0	0
1/10	0,411342481	1	0	0
1/10	0,411342481	1	0	0
1/10	0,411342481	1	0	0
1/10	0,411342481	1	0	0
1/10	0,411342481	1	0	0

1/10	0,411342481	1	0	0
1/10	0,411342481	1	0	0
1/10	0,411342481	1	0	0
1/10	0,411342481	1	0	0
1/10	0,411342481	1	0	0
1/10	0,411342481	1	0	0
1/10	0,411342481	1	0	0
1/10	0,411342481	1	0	0
18/326	0,418130626	1	0	0
7/120	0,425362518	1	0	0
3/46	0,426053988	1	0	0
2/28	0,42762859	1	0	0
2/28	0,42762859	1	0	0
3/47	0,439891843	1	0	0
1/11	0,441732347	1	0	0
1/11	0,441732347	1	0	0
1/11	0,441732347	1	0	0
1/11	0,441732347	1	0	0
1/11	0,441732347	1	0	0
1/11	0,441732347	1	0	0
1/11	0,441732347	1	0	0
1/11	0,441732347	1	0	0
1/11	0,441732347	1	0	0
1/11	0,441732347	1	0	0
2/29	0,445476512	1	0	0
2/29	0,445476512	1	0	0
2/29	0,445476512	1	0	0
2/29	0,445476512	1	0	0
9/161	0,451566513	1	0	0
3/48	0,453599363	1	0	0
4/67	0,456871178	1	0	0
5/86	0,458376644	1	0	0
2/30	0,463008482	1	0	0
2/30	0,463008482	1	0	0
2/30	0,463008482	1	0	0
2/30	0,463008482	1	0	0
1/12	0,470554753	1	0	0
1/12	0,470554753	1	0	0
1/12	0,470554753	1	0	0
1/12	0,470554753	1	0	0
1/12	0,470554753	1	0	0
1/12	0,470554753	1	0	0
1/12	0,470554753	1	0	0
1/12	0,470554753	1	0	0
1/12	0,470554753	1	0	0
1/12	0,470554753	1	0	0
85/1631	0,478323602	1	0	0
2/31	0,480209569	1	0	0
2/31	0,480209569	1	0	0
3/50	0,480578809	1	0	0
3/50	0,480578809	1	0	0
6/108	0,486339746	1	0	0
5/89	0,488513638	1	0	0

5/89	0,488513638	1	0	0
4/70	0,49097898	1	0	0
4/70	0,49097898	1	0	0
3/51	0,493830654	1	0	0
3/51	0,493830654	1	0	0
2/32	0,497067219	1	0	0
2/32	0,497067219	1	0	0
2/32	0,497067219	1	0	0
1/13	0,497890471	1	0	0
1/13	0,497890471	1	0	0
1/13	0,497890471	1	0	0
1/13	0,497890471	1	0	0
1/13	0,497890471	1	0	0
1/13	0,497890471	1	0	0
1/13	0,497890471	1	0	0
1/13	0,497890471	1	0	0
1/13	0,497890471	1	0	0
1/13	0,497890471	1	0	0
1/13	0,497890471	1	0	0
1/13	0,497890471	1	0	0
7/129	0,500956793	1	0	0
3/52	0,50691194	1	0	0
2/33	0,513571053	1	0	0
2/33	0,513571053	1	0	0
3/53	0,5198147	1	0	0
6/112	0,522016281	1	0	0
1/14	0,523816115	1	0	0
1/14	0,523816115	1	0	0
1/14	0,523816115	1	0	0
1/14	0,523816115	1	0	0
1/14	0,523816115	1	0	0
1/14	0,523816115	1	0	0
1/14	0,523816115	1	0	0
1/14	0,523816115	1	0	0
1/14	0,523816115	1	0	0
1/14	0,523816115	1	0	0
1/14	0,523816115	1	0	0
4/73	0,52416431	1	0	0
2/34	0,529712675	1	0	0
2/34	0,529712675	1	0	0
3/54	0,532531736	1	0	0
4/74	0,53499366	1	0	0
8/153	0,536088848	1	0	0
2/35	0,545485494	1	0	0
2/35	0,545485494	1	0	0
1/15	0,548404355	1	0	0
1/15	0,548404355	1	0	0
1/15	0,548404355	1	0	0
1/15	0,548404355	1	0	0
1/15	0,548404355	1	0	0
1/15	0,548404355	1	0	0
1/15	0,548404355	1	0	0
1/15	0,548404355	1	0	0

1/15	0,548404355	1	0	0
1/15	0,548404355	1	0	0
1/15	0,548404355	1	0	0
4/76	0,556273637	1	0	0
6/116	0,556668364	1	0	0
3/56	0,557383521	1	0	0
3/57	0,569507444	1	0	0
1/16	0,571724118	1	0	0
1/16	0,571724118	1	0	0
1/16	0,571724118	1	0	0
1/16	0,571724118	1	0	0
1/16	0,571724118	1	0	0
1/16	0,571724118	1	0	0
1/16	0,571724118	1	0	0
1/16	0,571724118	1	0	0
2/37	0,57590642	1	0	0
2/37	0,57590642	1	0	0
4/78	0,577020093	1	0	0
97/1908	0,579194291	1	0	0
3/58	0,581423931	1	0	0
5/99	0,583828473	1	0	0
5/99	0,583828473	1	0	0
4/79	0,587183851	1	0	0
2/38	0,590548963	1	0	0
2/38	0,590548963	1	0	0
2/38	0,590548963	1	0	0
5/100	0,592827344	1	0	0
1/17	0,593840785	1	0	0
1/17	0,593840785	1	0	0
1/17	0,593840785	1	0	0
1/17	0,593840785	1	0	0
1/17	0,593840785	1	0	0
1/17	0,593840785	1	0	0
1/17	0,593840785	1	0	0
1/17	0,593840785	1	0	0
1/17	0,593840785	1	0	0
5/101	0,601717403	1	0	0
7/142	0,60346403	1	0	0
2/39	0,604811304	1	0	0
2/39	0,604811304	1	0	0
1/18	0,614816365	1	0	0
1/18	0,614816365	1	0	0
1/18	0,614816365	1	0	0
1/18	0,614816365	1	0	0
1/18	0,614816365	1	0	0
1/18	0,614816365	1	0	0
1/18	0,614816365	1	0	0
5/103	0,619161958	1	0	0
3/62	0,626947593	1	0	0
3/62	0,626947593	1	0	0
3/62	0,626947593	1	0	0
2/41	0,632197223	1	0	0
1/19	0,63470968	1	0	0

1/19	0,63470968	1	0	0
1/19	0,63470968	1	0	0
1/19	0,63470968	1	0	0
1/19	0,63470968	1	0	0
1/19	0,63470968	1	0	0
3/63	0,637780712	1	0	0
3/63	0,637780712	1	0	0
3/63	0,637780712	1	0	0
4/85	0,645053048	1	0	0
2/42	0,645324095	1	0	0
2/42	0,645324095	1	0	0
29/594	0,648918979	1	0	0
1/20	0,653576518	1	0	0
1/20	0,653576518	1	0	0
1/20	0,653576518	1	0	0
1/20	0,653576518	1	0	0
2/43	0,658077154	1	0	0
2/43	0,658077154	1	0	0
2/43	0,658077154	1	0	0
2/43	0,658077154	1	0	0
2/43	0,658077154	1	0	0
12/253	0,659185869	1	0	0
4/87	0,663107434	1	0	0
32/659	0,665731451	1	0	0
2/44	0,670459987	1	0	0
2/44	0,670459987	1	0	0
2/44	0,670459987	1	0	0
2/44	0,670459987	1	0	0
2/44	0,670459987	1	0	0
2/44	0,670459987	1	0	0
2/44	0,670459987	1	0	0
2/44	0,670459987	1	0	0
2/44	0,670459987	1	0	0
2/44	0,670459987	1	0	0
1/21	0,671469795	1	0	0
1/21	0,671469795	1	0	0
1/21	0,671469795	1	0	0
1/21	0,671469795	1	0	0
1/21	0,671469795	1	0	0
1/21	0,671469795	1	0	0
4/88	0,671896795	1	0	0
5/110	0,676508189	1	0	0
3/67	0,678884566	1	0	0
3/67	0,678884566	1	0	0
4/89	0,680526583	1	0	0
2/45	0,682476808	1	0	0
1/22	0,688439703	1	0	0
1/22	0,688439703	1	0	0
1/22	0,688439703	1	0	0
1/22	0,688439703	1	0	0
1/22	0,688439703	1	0	0
1/22	0,688439703	1	0	0
3/69	0,698097744	1	0	0

3/69	0,698097744	1	0	0
1/23	0,704533848	1	0	0
1/23	0,704533848	1	0	0
1/23	0,704533848	1	0	0
1/23	0,704533848	1	0	0
1/23	0,704533848	1	0	0
1/23	0,704533848	1	0	0
1/23	0,704533848	1	0	0
1/23	0,704533848	1	0	0
2/47	0,705431946	1	0	0
4/92	0,705454701	1	0	0
10/222	0,714602028	1	0	0
13/285	0,715320934	1	0	0
2/48	0,716381176	1	0	0
1/24	0,719797384	1	0	0
1/24	0,719797384	1	0	0
1/24	0,719797384	1	0	0
3/72	0,725260757	1	0	0
3/72	0,725260757	1	0	0
3/72	0,725260757	1	0	0
3/72	0,725260757	1	0	0
2/49	0,7269861	1	0	0
3/73	0,733879166	1	0	0
1/25	0,734273137	1	0	0
1/25	0,734273137	1	0	0
1/25	0,734273137	1	0	0
1/25	0,734273137	1	0	0
1/25	0,734273137	1	0	0
29/620	0,735227708	1	0	0
7/162	0,736500524	1	0	0
2/50	0,737253058	1	0	0
4/97	0,743803628	1	0	0
2/51	0,747188653	1	0	0
2/51	0,747188653	1	0	0
2/51	0,747188653	1	0	0
1/26	0,74800173	1	0	0
1/26	0,74800173	1	0	0
1/26	0,74800173	1	0	0
1/26	0,74800173	1	0	0
1/26	0,74800173	1	0	0
1/26	0,74800173	1	0	0
3/75	0,750473779	1	0	0
2/52	0,756799709	1	0	0
2/52	0,756799709	1	0	0
3/76	0,758454597	1	0	0
3/76	0,758454597	1	0	0
1/27	0,761021692	1	0	0
1/27	0,761021692	1	0	0
1/27	0,761021692	1	0	0
2/53	0,766093226	1	0	0
2/53	0,766093226	1	0	0
3/77	0,766227792	1	0	0
11/255	0,77022526	1	0	0

1/28	0,773369566	1	0	0
1/28	0,773369566	1	0	0
1/28	0,773369566	1	0	0
1/28	0,773369566	1	0	0
2/54	0,775076347	1	0	0
2/54	0,775076347	1	0	0
3/79	0,781162352	1	0	0
3/79	0,781162352	1	0	0
3/79	0,781162352	1	0	0
6/148	0,78147079	1	0	0
1/29	0,785080013	1	0	0
1/30	0,796185909	1	0	0
1/30	0,796185909	1	0	0
1/30	0,796185909	1	0	0
1/30	0,796185909	1	0	0
1/30	0,796185909	1	0	0
1/30	0,796185909	1	0	0
1/30	0,796185909	1	0	0
50/1074	0,797790715	1	0	0
2/57	0,800236223	1	0	0
2/57	0,800236223	1	0	0
2/57	0,800236223	1	0	0
2/57	0,800236223	1	0	0
3/82	0,802079325	1	0	0
3/82	0,802079325	1	0	0
3/82	0,802079325	1	0	0
7/175	0,804335687	1	0	0
1/31	0,806718435	1	0	0
1/31	0,806718435	1	0	0
1/31	0,806718435	1	0	0
1/31	0,806718435	1	0	0
1/31	0,806718435	1	0	0
2/58	0,808050934	1	0	0
2/58	0,808050934	1	0	0
2/58	0,808050934	1	0	0
3/84	0,815071228	1	0	0
3/84	0,815071228	1	0	0
3/84	0,815071228	1	0	0
2/59	0,815592036	1	0	0
2/59	0,815592036	1	0	0
2/59	0,815592036	1	0	0
25/566	0,816686652	1	0	0
1/32	0,816707165	1	0	0
1/32	0,816707165	1	0	0
1/32	0,816707165	1	0	0
1/32	0,816707165	1	0	0
1/32	0,816707165	1	0	0
1/32	0,816707165	1	0	0
1/32	0,816707165	1	0	0
1/32	0,816707165	1	0	0
4/109	0,820145299	1	0	0
3/85	0,821291395	1	0	0
3/85	0,821291395	1	0	0

3/85	0,821291395	1	0	0
11/268	0,821495979	1	0	0
2/60	0,822866924	1	0	0
2/60	0,822866924	1	0	0
1/33	0,826180149	1	0	0
1/33	0,826180149	1	0	0
1/33	0,826180149	1	0	0
1/33	0,826180149	1	0	0
3/86	0,827332306	1	0	0
3/86	0,827332306	1	0	0
2/61	0,829882961	1	0	0
2/61	0,829882961	1	0	0
2/61	0,829882961	1	0	0
4/111	0,83084979	1	0	0
1/34	0,835163993	1	0	0
1/34	0,835163993	1	0	0
1/34	0,835163993	1	0	0
2/62	0,83664746	1	0	0
1/35	0,84368393	1	0	0
1/35	0,84368393	1	0	0
1/35	0,84368393	1	0	0
1/35	0,84368393	1	0	0
1/35	0,84368393	1	0	0
1/35	0,84368393	1	0	0
2/64	0,849450754	1	0	0
2/64	0,849450754	1	0	0
3/90	0,849773978	1	0	0
2/65	0,855503801	1	0	0
13/323	0,856236087	1	0	0
5/141	0,858559013	1	0	0
1/37	0,859426581	1	0	0
1/37	0,859426581	1	0	0
1/37	0,859426581	1	0	0
1/37	0,859426581	1	0	0
2/66	0,861333792	1	0	0
2/66	0,861333792	1	0	0
2/66	0,861333792	1	0	0
14/348	0,864108072	1	0	0
4/118	0,86421104	1	0	0
3/93	0,864897235	1	0	0
2/67	0,8669476	1	0	0
2/67	0,8669476	1	0	0
11/283	0,869376222	1	0	0
2/68	0,872351988	1	0	0
2/68	0,872351988	1	0	0
1/39	0,873585143	1	0	0
1/39	0,873585143	1	0	0
1/39	0,873585143	1	0	0
1/39	0,873585143	1	0	0
1/39	0,873585143	1	0	0
1/39	0,873585143	1	0	0
19/462	0,874405322	1	0	0
2/69	0,877553593	1	0	0

1/40	0,880120803	1	0	0
1/40	0,880120803	1	0	0
4/122	0,880619471	1	0	0
1/41	0,886318873	1	0	0
1/41	0,886318873	1	0	0
1/41	0,886318873	1	0	0
1/41	0,886318873	1	0	0
13/335	0,887195649	1	0	0
6/173	0,887273389	1	0	0
2/71	0,887374376	1	0	0
2/71	0,887374376	1	0	0
4/124	0,888158441	1	0	0
3/99	0,89116978	1	0	0
3/99	0,89116978	1	0	0
2/72	0,89200618	1	0	0
2/72	0,89200618	1	0	0
1/42	0,892196776	1	0	0
1/42	0,892196776	1	0	0
1/42	0,892196776	1	0	0
1/42	0,892196776	1	0	0
7/199	0,893372444	1	0	0
3/100	0,895074371	1	0	0
1/43	0,897771034	1	0	0
1/43	0,897771034	1	0	0
1/43	0,897771034	1	0	0
1/43	0,897771034	1	0	0
1/43	0,897771034	1	0	0
2/74	0,900743148	1	0	0
1/44	0,903057318	1	0	0
2/75	0,904860104	1	0	0
2/75	0,904860104	1	0	0
3/103	0,906043438	1	0	0
14/367	0,907242987	1	0	0
1/45	0,908070493	1	0	0
1/45	0,908070493	1	0	0
4/131	0,91136001	1	0	0
3/105	0,912768376	1	0	0
3/105	0,912768376	1	0	0
3/106	0,915964103	1	0	0
2/78	0,916272592	1	0	0
3/107	0,919052811	1	0	0
6/187	0,924793567	1	0	0
1/49	0,925663348	1	0	0
1/49	0,925663348	1	0	0
1/49	0,925663348	1	0	0
1/49	0,925663348	1	0	0
1/49	0,925663348	1	0	0
2/81	0,926389233	1	0	0
17/447	0,927545311	1	0	0
3/110	0,927706934	1	0	0
1/50	0,929508438	1	0	0
1/50	0,929508438	1	0	0
1/50	0,929508438	1	0	0

1/50	0,929508438	1	0	0
1/50	0,929508438	1	0	0
2/83	0,932480377	1	0	0
1/51	0,933154815	1	0	0
1/51	0,933154815	1	0	0
6/191	0,933272072	1	0	0
2/84	0,935344002	1	0	0
2/84	0,935344002	1	0	0
1/52	0,93661274	1	0	0
1/52	0,93661274	1	0	0
1/52	0,93661274	1	0	0
1/52	0,93661274	1	0	0
3/114	0,937925444	1	0	0
3/114	0,937925444	1	0	0
3/114	0,937925444	1	0	0
9/268	0,93871086	1	0	0
1/53	0,939891944	1	0	0
6/196	0,94267301	1	0	0
1/54	0,943001654	1	0	0
1/54	0,943001654	1	0	0
1/54	0,943001654	1	0	0
1/55	0,945950624	1	0	0
1/55	0,945950624	1	0	0
1/57	0,951399117	1	0	0
1/58	0,95391398	1	0	0
1/58	0,95391398	1	0	0
4/150	0,954211894	1	0	0
1/59	0,956298824	1	0	0
1/60	0,958560365	1	0	0
2/95	0,960103841	1	0	0
2/95	0,960103841	1	0	0
1/62	0,962738684	1	0	0
3/128	0,96406885	1	0	0
1/63	0,964667232	1	0	0
1/63	0,964667232	1	0	0
1/64	0,966496048	1	0	0
2/99	0,966611894	1	0	0
1/65	0,968230286	1	0	0
2/101	0,969470187	1	0	0
2/101	0,969470187	1	0	0
1/66	0,969874831	1	0	0
1/68	0,972913143	1	0	0
1/68	0,972913143	1	0	0
5/193	0,973425804	1	0	0
2/106	0,975621261	1	0	0
2/106	0,975621261	1	0	0
2/106	0,975621261	1	0	0
2/106	0,975621261	1	0	0
1/70	0,975645263	1	0	0
1/70	0,975645263	1	0	0
2/107	0,976698744	1	0	0
1/71	0,97690626	1	0	0
1/72	0,97810202	1	0	0

1/74	0,980311144	1	0	0
2/112	0,981430592	1	0	0
3/145	0,981940401	1	0	0
1/77	0,983214376	1	0	0
3/147	0,983369569	1	0	0
1/80	0,985689806	1	0	0
2/118	0,985886753	1	0	0
3/151	0,985909834	1	0	0
4/182	0,98609614	1	0	0
6/240	0,986340236	1	0	0
3/162	0,991118006	1	0	0
3/166	0,992504365	1	0	0
2/136	0,99387461	1	0	0
1/96	0,993891226	1	0	0
1/97	0,994207821	1	0	0
3/173	0,994442873	1	0	0
1/98	0,994508015	1	0	0
3/174	0,994676621	1	0	0
3/180	0,995891116	1	0	0
1/111	0,997250643	1	0	0
45/1293	0,998794891	1	0	0
1/128	0,998887353	1	0	0
1/157	0,999760855	1	0	0
21/983	0,999994192	1	0	0

Odds.Ratio	Combined.Score
7,350975675	118,7841708
1,676627808	18,09131592
5,12995896	51,85294347
3,499138674	33,43659315
1,768646045	16,08507317
4,472271914	40,0004265
2,036526081	18,04131956
2,7004702	23,34905293
2,239881578	18,53348481
1,951442722	14,96092138
6,45994832	46,88590967
6,45994832	46,88590967
6,45994832	46,88590967
6,45994832	46,88590967
6,45994832	46,88590967
6,45994832	46,88590967
3,55956336	25,39936894
3,55956336	25,39936894
4,376094024	30,97616695
3,875968992	27,35873171
6,05620155	41,94775145
7,751937984	52,45132753
3,69139904	24,81143421
5,6999544	37,74061275
1,746915602	11,23314645
3,875968992	24,49378819
1,70998632	10,67143851
1,863446631	11,47522948
4,306632214	26,42690116
4,306632214	26,42690116
3,05997552	18,44255869
3,05997552	18,44255869
3,05997552	18,44255869
3,05997552	18,44255869
3,05997552	18,44255869
9,689922481	58,30911925
6,45994832	38,70648143
6,45994832	38,70648143
3,298697015	19,68990491
4,15282392	24,66435389
1,774211158	10,07294041
3,478433711	19,72690738
1,703077284	9,514543289
2,42248062	13,47772695
5,53709856	29,73555012
5,53709856	29,73555012
5,53709856	29,73555012
5,53709856	29,73555012
5,53709856	29,73555012
5,53709856	29,73555012
2,76854928	14,76934109
2,450325225	12,95637812

1,948811225	10,2230193
3,22997416	16,93549945
4,213009774	21,84523656
4,213009774	21,84523656
1,800782054	8,994464644
1,800782054	8,994464644
2,171879177	10,82737654
2,81888654	14,0305019
4,84496124	23,5210893
1,652052357	7,864497643
6,45994832	30,3565599
6,45994832	30,3565599
3,726893262	17,30271119
3,726893262	17,30271119
3,726893262	17,30271119
2,065065447	9,448153758
2,065065447	9,448153758
2,065065447	9,448153758
2,065065447	9,448153758
2,065065447	9,448153758
2,065065447	9,448153758
1,507321275	6,86634607
1,591306918	7,234228281
1,590443204	7,031949831
1,590443204	7,031949831
1,590443204	7,031949831
4,306632214	19,02322652
2,015503876	8,83443341
2,015503876	8,83443341
5,813953488	25,4722545
1,576071729	6,79549564
1,576071729	6,79549564
2,058036633	8,82520189
1,543998637	6,60720957
1,571338781	6,718433394
1,61498708	6,838051798
1,61498708	6,838051798
1,61498708	6,838051798
2,65997872	11,13464158
1,55730897	6,492366848
5,285412262	21,67743134
3,22997416	13,04612629
3,22997416	13,04612629
2,836074872	11,42110301
2,76854928	10,84216754
2,207830439	8,608520067
1,521092482	5,925315185
3,69139904	14,28108748
3,69139904	14,28108748
1,50314587	5,796423662
1,880134213	7,246285813
1,569494486	5,977087813
1,99232051	7,586407507

1,707032976	6,491499825
3,028100775	11,43902942
3,028100775	11,43902942
3,028100775	11,43902942
7,751937984	28,92370822
1,322509105	4,913069721
2,642706131	9,792955667
2,642706131	9,792955667
1,553088621	5,747673751
1,48261109	5,482810435
1,608285889	5,931237616
4,472271914	16,22619769
4,472271914	16,22619769
4,472271914	16,22619769
4,472271914	16,22619769
4,472271914	16,22619769
4,472271914	16,22619769
2,583979328	9,316902113
2,8499772	10,08411019
2,8499772	10,08411019
1,430417128	5,051612573
1,369228177	4,824868637
1,490757305	5,192677377
2,153316107	7,435166443
4,15282392	14,22455135
4,15282392	14,22455135
4,15282392	14,22455135
1,457537086	4,988827675
3,22997416	11,03461162
2,474022761	8,449535228
2,260981912	7,634855326
2,260981912	7,634855326
6,45994832	21,70645267
1,749569337	5,842269562
2,42248062	8,053974604
1,42282406	4,723571454
2,223916635	7,335798167
3,100775194	10,1777513
3,100775194	10,1777513
3,875968992	12,55918396
3,875968992	12,55918396
3,875968992	12,55918396
2,618897968	8,419750834
1,674801416	5,350827645
1,937984496	6,125212005
2,5499796	7,944778207
2,5499796	7,944778207
2,5499796	7,944778207
3,63372093	11,15790341
3,63372093	11,15790341
3,63372093	11,15790341
3,63372093	11,15790341
1,722652885	5,28289965

5,53709856	16,93240671
5,53709856	16,93240671
5,53709856	16,93240671
1,58202816	4,828317222
2,871088142	8,714854065
2,871088142	8,714854065
1,761804087	5,340908466
1,485045591	4,424974073
1,433236123	4,199147567
2,76854928	8,087831292
3,41997264	9,967191743
3,41997264	9,967191743
1,418037436	4,126577881
1,835985312	5,278017217
2,024759921	5,810753559
2,024759921	5,810753559
1,914058762	5,478404972
1,497155405	4,270520265
2,673082064	7,51912473
2,673082064	7,51912473
2,673082064	7,51912473
2,673082064	7,51912473
4,84496124	13,58747994
4,84496124	13,58747994
4,84496124	13,58747994
1,890716582	5,300642972
1,890716582	5,300642972
1,890716582	5,300642972
1,890716582	5,300642972
3,22997416	8,946625228
3,22997416	8,946625228
1,798129945	4,977100603
1,798129945	4,977100603
1,798129945	4,977100603
2,114164905	5,842870663
1,25634857	3,461302776
1,637733377	4,511113156
1,478123768	4,071013089
1,478123768	4,071013089
1,478123768	4,071013089
1,478123768	4,071013089
1,867936864	5,129506462
1,867936864	5,129506462
1,455403377	3,966537916
2,583979328	7,001837457
1,392926357	3,730150281
2,253470344	6,02101599
1,459569913	3,880819526
3,05997552	8,065164774
1,823985408	4,805936014
1,823985408	4,805936014
1,514050388	3,976610163
2,03998368	5,352688448

2,500625156	6,530069643
1,68520391	4,399687257
2,202255109	5,709376829
2,202255109	5,709376829
4,306632214	11,14179549
4,306632214	11,14179549
4,306632214	11,14179549
4,306632214	11,14179549
1,802776275	4,652954598
1,627315226	4,18904016
1,441476071	3,699470869
1,726916878	4,431448533
1,435544071	3,64090981
2,42248062	6,098748804
2,42248062	6,098748804
2,42248062	6,098748804
2,153316107	5,417548418
2,153316107	5,417548418
1,858341298	4,647954556
1,761804087	4,363338631
1,418037436	3,470672184
2,106504887	5,143960963
1,937984496	4,706864245
1,937984496	4,706864245
1,937984496	4,706864245
3,875968992	9,293806274
3,875968992	9,293806274
3,875968992	9,293806274
3,875968992	9,293806274
3,875968992	9,293806274
3,875968992	9,293806274
3,875968992	9,293806274
3,875968992	9,293806274
2,76854928	6,627928123
2,76854928	6,627928123
2,76854928	6,627928123
1,336541032	3,195245208
1,906214258	4,512560201
1,906214258	4,512560201
1,38427464	3,251010693
2,27998176	5,340500606
1,428928661	3,340260719
1,703722634	3,966383947
2,01873385	4,645971938
1,401946231	3,211752968
1,401946231	3,211752968
1,401946231	3,211752968
1,401946231	3,211752968
1,401946231	3,211752968
2,642706131	6,037824525
1,68520391	3,843231336
1,68520391	3,843231336
1,68520391	3,843231336

1,68520391	3,843231336
1,68520391	3,843231336
1,38427464	3,153374914
1,260477721	2,826835566
1,9775352	4,419123035
1,739216855	3,882271095
3,523608175	7,860423421
3,523608175	7,860423421
3,523608175	7,860423421
3,523608175	7,860423421
3,523608175	7,860423421
3,523608175	7,860423421
3,523608175	7,860423421
3,523608175	7,860423421
1,511902798	3,300413616
2,527805865	5,516018479
1,717201452	3,7471302
1,378482696	3,004035009
1,937984496	4,205592161
1,585623679	3,43081668
1,585623679	3,43081668
1,372739018	2,954207065
1,10068085	2,365607938
1,8999848	4,004416356
1,8999848	4,004416356
1,8999848	4,004416356
2,095118374	4,413990065
1,61498708	3,391605274
2,42248062	5,052490523
2,42248062	5,052490523
2,42248062	5,052490523
1,55730897	3,243896991
3,22997416	6,724672735
3,22997416	6,724672735
3,22997416	6,724672735
3,22997416	6,724672735
3,22997416	6,724672735
3,22997416	6,724672735
3,22997416	6,724672735
1,480404823	3,074623618
1,344723936	2,716966923
2,325581395	4,639006775
2,325581395	4,639006775
2,325581395	4,639006775
1,426737052	2,834040257
1,61498708	3,146670379
2,981514609	5,80867905
2,981514609	5,80867905
2,981514609	5,80867905
2,981514609	5,80867905
2,981514609	5,80867905
2,981514609	5,80867905
2,981514609	5,80867905

1,305734235	2,541167299
1,328457114	2,58366355
1,794430089	3,466608554
2,236135957	4,268746712
2,236135957	4,268746712
2,236135957	4,268746712
2,236135957	4,268746712
2,236135957	4,268746712
2,236135957	4,268746712
2,236135957	4,268746712
2,236135957	4,268746712
1,595987232	3,040057723
1,937984496	3,68046246
1,937984496	3,68046246
1,937984496	3,68046246
1,400952648	2,653822098
1,535037225	2,907692919
1,577429241	2,937471473
1,51998784	2,820307726
1,411776785	2,608365451
2,153316107	3,936019159
2,153316107	3,936019159
2,76854928	5,058804027
2,76854928	5,058804027
2,76854928	5,058804027
2,76854928	5,058804027
2,76854928	5,058804027
1,4249886	2,566158088
1,274107454	2,279751465
1,84569952	3,274194731
1,84569952	3,274194731
1,152704533	2,042925397
2,07641196	3,636043216
1,67067629	2,877011543
1,429660694	2,460294438
2,583979328	4,437032068
2,583979328	4,437032068
2,583979328	4,437032068
2,583979328	4,437032068
2,583979328	4,437032068
2,583979328	4,437032068
2,583979328	4,437032068
2,583979328	4,437032068
2,583979328	4,437032068
2,583979328	4,437032068
1,571338781	2,676432288
2,004811548	3,364777581
1,550387597	2,575198513
1,761804087	2,921385574
1,761804087	2,921385574
2,42248062	3,915755568
2,42248062	3,915755568
2,42248062	3,915755568

1,28586649	2,055374563
1,68520391	2,613531239
1,875468867	2,895132655
1,875468867	2,895132655
1,875468867	2,895132655
2,27998176	3,474499418
2,27998176	3,474499418
2,27998176	3,474499418
2,27998176	3,474499418
2,27998176	3,474499418
2,27998176	3,474499418
1,18652112	1,791950623
1,649348507	2,474255328
1,538082933	2,297125709
1,538082933	2,297125709
1,816860465	2,691293478
1,816860465	2,691293478
1,453488372	2,129413051
3,875968992	5,650622543
3,875968992	5,650622543
3,875968992	5,650622543
3,875968992	5,650622543
3,875968992	5,650622543
3,875968992	5,650622543
3,875968992	5,650622543
3,875968992	5,650622543
3,875968992	5,650622543
3,875968992	5,650622543
3,875968992	5,650622543
3,875968992	5,650622543
3,875968992	5,650622543
3,875968992	5,650622543
3,875968992	5,650622543
3,875968992	5,650622543
3,875968992	5,650622543
3,875968992	5,650622543
1,398545513	2,029774844
1,61498708	2,343738185
2,153316107	3,097797377
2,153316107	3,097797377
2,153316107	3,097797377
1,761804087	2,505091275
1,761804087	2,505091275
1,761804087	2,505091275
1,761804087	2,505091275
1,761804087	2,505091275
1,761804087	2,505091275
1,418037436	1,975764768
1,241073815	1,71868217
1,468170073	2,014101658
1,70998632	2,334638367
1,70998632	2,334638367
1,70998632	2,334638367

1,70998632	2,334638367
1,70998632	2,334638367
1,70998632	2,334638367
2,03998368	2,773776792
2,03998368	2,773776792
2,03998368	2,773776792
2,03998368	2,773776792
2,03998368	2,773776792
2,03998368	2,773776792
2,03998368	2,773776792
1,550387597	2,106369756
1,1972105	1,592901456
1,51998784	1,998360232
1,28248974	1,682742468
1,661129568	2,178291625
3,22997416	4,201306255
3,22997416	4,201306255
3,22997416	4,201306255
3,22997416	4,201306255
3,22997416	4,201306255
3,22997416	4,201306255
3,22997416	4,201306255
3,22997416	4,201306255
3,22997416	4,201306255
3,22997416	4,201306255
3,22997416	4,201306255
3,22997416	4,201306255
3,22997416	4,201306255
1,4249886	1,847455232
1,937984496	2,493192356
1,937984496	2,493192356
1,937984496	2,493192356
1,61498708	2,034615246
1,61498708	2,034615246
1,462629808	1,801171619
1,336541032	1,642416805
1,115921975	1,371076798
1,84569952	2,248752181
1,84569952	2,248752181
1,84569952	2,248752181
1,245847176	1,511149881
1,571338781	1,902350018
1,321353066	1,583420887
1,260477721	1,508799043
1,435544071	1,711114005
1,250312578	1,465287873
2,76854928	3,243431827
2,76854928	3,243431827
2,76854928	3,243431827
2,76854928	3,243431827
2,76854928	3,243431827
2,76854928	3,243431827
2,76854928	3,243431827

2,76854928	3,243431827
2,76854928	3,243431827
2,76854928	3,243431827
2,76854928	3,243431827
2,76854928	3,243431827
2,76854928	3,243431827
2,76854928	3,243431827
2,76854928	3,243431827
2,76854928	3,243431827
1,52998776	1,780387798
1,52998776	1,780387798
1,267840325	1,464570245
1,761804087	2,034638719
1,761804087	2,034638719
1,40944327	1,62622342
1,40944327	1,62622342
1,203717078	1,373851769
1,052299274	1,180456389
1,490757305	1,667750218
1,490757305	1,667750218
1,490757305	1,667750218
1,277791975	1,419790872
1,074006186	1,190122484
1,68520391	1,846162327
1,68520391	1,846162327
1,108928458	1,183359845
2,42248062	2,574415889
2,42248062	2,574415889
2,42248062	2,574415889
2,42248062	2,574415889
2,42248062	2,574415889
2,42248062	2,574415889
2,42248062	2,574415889
2,42248062	2,574415889
2,42248062	2,574415889
2,42248062	2,574415889
2,42248062	2,574415889
2,42248062	2,574415889
2,42248062	2,574415889
2,42248062	2,574415889
2,42248062	2,574415889
2,42248062	2,574415889
2,42248062	2,574415889
1,61498708	1,679506874
1,61498708	1,679506874
1,61498708	1,679506874
1,61498708	1,679506874
1,418037436	1,467080051
1,418037436	1,467080051
1,418037436	1,467080051
1,418037436	1,467080051
1,313887794	1,33169862
1,313887794	1,33169862

1,313887794	1,33169862
1,38427464	1,377592472
1,38427464	1,377592472
1,38427464	1,377592472
1,38427464	1,377592472
1,550387597	1,531540293
1,550387597	1,531540293
1,550387597	1,531540293
1,550387597	1,531540293
1,21124031	1,186101968
2,153316107	2,087484578
2,153316107	2,087484578
2,153316107	2,087484578
2,153316107	2,087484578
2,153316107	2,087484578
2,153316107	2,087484578
2,153316107	2,087484578
2,153316107	2,087484578
2,153316107	2,087484578
2,153316107	2,087484578
2,153316107	2,087484578
1,352082207	1,294496013
1,076658053	1,030391764
1,198753297	1,144494099
1,094542739	1,041898593
1,226572466	1,166168737
1,226572466	1,166168737
1,490757305	1,399671698
1,490757305	1,399671698
1,490757305	1,399671698
1,21124031	1,119552228
1,321353066	1,217242729
1,174536058	1,065849316
1,196286726	1,074952109
1,196286726	1,074952109
1,435544071	1,281742365
1,435544071	1,281742365
1,435544071	1,281742365
1,435544071	1,281742365
1,435544071	1,281742365
1,435544071	1,281742365
1,435544071	1,281742365
1,937984496	1,721568072
1,937984496	1,721568072
1,937984496	1,721568072
1,937984496	1,721568072
1,937984496	1,721568072
1,937984496	1,721568072
1,937984496	1,721568072
1,937984496	1,721568072
1,937984496	1,721568072
1,937984496	1,721568072
1,937984496	1,721568072

1,937984496	1,721568072
1,937984496	1,721568072
1,937984496	1,721568072
1,937984496	1,721568072
1,937984496	1,721568072
1,937984496	1,721568072
1,937984496	1,721568072
1,937984496	1,721568072
1,070052789	0,93304472
1,130490956	0,966358918
1,263902932	1,078348343
1,38427464	1,17594164
1,38427464	1,17594164
1,237011381	1,015866394
1,761804087	1,43948402
1,761804087	1,43948402
1,761804087	1,43948402
1,761804087	1,43948402
1,761804087	1,43948402
1,761804087	1,43948402
1,761804087	1,43948402
1,761804087	1,43948402
1,761804087	1,43948402
1,761804087	1,43948402
1,336541032	1,080741454
1,336541032	1,080741454
1,336541032	1,080741454
1,336541032	1,080741454
1,083345371	0,861294888
1,21124031	0,957535043
1,157005669	0,906344803
1,126735172	0,878925619
1,291989664	0,994844838
1,291989664	0,994844838
1,291989664	0,994844838
1,291989664	0,994844838
1,61498708	1,217446632
1,61498708	1,217446632
1,61498708	1,217446632
1,61498708	1,217446632
1,61498708	1,217446632
1,61498708	1,217446632
1,61498708	1,217446632
1,61498708	1,217446632
1,61498708	1,217446632
1,61498708	1,217446632
1,009985789	0,744831981
1,250312578	0,917145122
1,250312578	0,917145122
1,162790698	0,852051221
1,162790698	0,852051221
1,076658053	0,776106625
1,088755335	0,779971136

1,088755335	0,779971136
1,107419712	0,787767401
1,107419712	0,787767401
1,13999088	0,804334959
1,13999088	0,804334959
1,21124031	0,84669333
1,21124031	0,84669333
1,21124031	0,84669333
1,490757305	1,03961712
1,490757305	1,03961712
1,490757305	1,03961712
1,490757305	1,03961712
1,490757305	1,03961712
1,490757305	1,03961712
1,490757305	1,03961712
1,490757305	1,03961712
1,490757305	1,03961712
1,490757305	1,03961712
1,490757305	1,03961712
1,490757305	1,03961712
1,051619494	0,726916646
1,118067979	0,759635486
1,174536058	0,782671939
1,174536058	0,782671939
1,096972356	0,71773023
1,03820598	0,674892548
1,38427464	0,895092168
1,38427464	0,895092168
1,38427464	0,895092168
1,38427464	0,895092168
1,38427464	0,895092168
1,38427464	0,895092168
1,38427464	0,895092168
1,38427464	0,895092168
1,38427464	0,895092168
1,38427464	0,895092168
1,38427464	0,895092168
1,061909313	0,685940401
1,13999088	0,724373623
1,13999088	0,724373623
1,076658053	0,678416004
1,047559187	0,655248672
1,013325227	0,631763055
1,107419712	0,671183906
1,107419712	0,671183906
1,291989664	0,776152959
1,291989664	0,776152959
1,291989664	0,776152959
1,291989664	0,776152959
1,291989664	0,776152959
1,291989664	0,776152959
1,291989664	0,776152959
1,291989664	0,776152959

1,291989664	0,776152959
1,291989664	0,776152959
1,291989664	0,776152959
1,01999184	0,598220067
1,002405774	0,587194881
1,03820598	0,60683319
1,01999184	0,5742385
1,21124031	0,6772029
1,21124031	0,6772029
1,21124031	0,6772029
1,21124031	0,6772029
1,21124031	0,6772029
1,21124031	0,6772029
1,21124031	0,6772029
1,21124031	0,6772029
1,047559187	0,578053737
1,047559187	0,578053737
0,993838203	0,546489952
0,98524369	0,538058619
1,002405774	0,543579722
0,978780049	0,526728574
0,978780049	0,526728574
0,981257973	0,522438724
1,01999184	0,537232486
1,01999184	0,537232486
1,01999184	0,537232486
0,968992248	0,506639611
1,13999088	0,594099447
1,13999088	0,594099447
1,13999088	0,594099447
1,13999088	0,594099447
1,13999088	0,594099447
1,13999088	0,594099447
1,13999088	0,594099447
1,13999088	0,594099447
1,13999088	0,594099447
0,959398265	0,487343017
0,95534447	0,482514726
0,993838203	0,499740374
0,993838203	0,499740374
1,076658053	0,523720552
1,076658053	0,523720552
1,076658053	0,523720552
1,076658053	0,523720552
1,076658053	0,523720552
1,076658053	0,523720552
1,076658053	0,523720552
0,940769173	0,450993825
0,937734434	0,43782101
0,937734434	0,43782101
0,937734434	0,43782101
0,945358291	0,433497705
1,01999184	0,463675624

1,01999184	0,463675624
1,01999184	0,463675624
1,01999184	0,463675624
1,01999184	0,463675624
1,01999184	0,463675624
0,92284976	0,415061615
0,92284976	0,415061615
0,92284976	0,415061615
0,911992704	0,399838323
0,92284976	0,404210609
0,92284976	0,404210609
0,946154047	0,409161867
0,968992248	0,412108201
0,968992248	0,412108201
0,968992248	0,412108201
0,968992248	0,412108201
0,901388138	0,377170632
0,901388138	0,377170632
0,901388138	0,377170632
0,901388138	0,377170632
0,901388138	0,377170632
0,919202133	0,383077247
0,891027355	0,366050307
0,941054687	0,382885901
0,880902044	0,352176933
0,880902044	0,352176933
0,880902044	0,352176933
0,880902044	0,352176933
0,880902044	0,352176933
0,880902044	0,352176933
0,880902044	0,352176933
0,880902044	0,352176933
0,880902044	0,352176933
0,880902044	0,352176933
0,92284976	0,367558366
0,92284976	0,367558366
0,92284976	0,367558366
0,92284976	0,367558366
0,92284976	0,367558366
0,92284976	0,367558366
0,880902044	0,350291164
0,880902044	0,344265968
0,867754252	0,336084841
0,867754252	0,336084841
0,871004268	0,335239434
0,861326443	0,329049728
0,880902044	0,328864994
0,880902044	0,328864994
0,880902044	0,328864994
0,880902044	0,328864994
0,880902044	0,328864994
0,880902044	0,328864994
0,842601955	0,3028279

0,842601955	0,3028279
0,842601955	0,295095132
0,842601955	0,295095132
0,842601955	0,295095132
0,842601955	0,295095132
0,842601955	0,295095132
0,842601955	0,295095132
0,842601955	0,295095132
0,842601955	0,295095132
0,824674254	0,287765937
0,842601955	0,293994538
0,872965989	0,293342321
0,883992928	0,296158827
0,80749354	0,269333725
0,80749354	0,265492182
0,80749354	0,265492182
0,80749354	0,265492182
0,80749354	0,259386324
0,80749354	0,259386324
0,80749354	0,259386324
0,80749354	0,259386324
0,79101408	0,252213195
0,796431985	0,246424727
0,775193798	0,239437363
0,775193798	0,239437363
0,775193798	0,239437363
0,775193798	0,239437363
0,775193798	0,239437363
0,906476619	0,278809565
0,837400708	0,256115097
0,775193798	0,23629774
0,799168864	0,236536578
0,75999392	0,221490787
0,75999392	0,221490787
0,75999392	0,221490787
0,745378652	0,216420683
0,745378652	0,216420683
0,745378652	0,216420683
0,745378652	0,216420683
0,745378652	0,216420683
0,745378652	0,216420683
0,775193798	0,222519819
0,745378652	0,207704715
0,745378652	0,207704715
0,76499388	0,211499649
0,76499388	0,211499649
0,717772036	0,196018818
0,717772036	0,196018818
0,717772036	0,196018818
0,731314904	0,194859889
0,731314904	0,194859889
0,755058895	0,201053892
0,835993312	0,218254665

0,69213732	0,177878081
0,69213732	0,177878081
0,69213732	0,177878081
0,69213732	0,177878081
0,717772036	0,182883823
0,717772036	0,182883823
0,73594348	0,181757635
0,73594348	0,181757635
0,73594348	0,181757635
0,78566939	0,193728399
0,668270516	0,161701175
0,645994832	0,1472368
0,645994832	0,1472368
0,645994832	0,1472368
0,645994832	0,1472368
0,645994832	0,1472368
0,645994832	0,1472368
0,645994832	0,1472368
0,902227419	0,203821274
0,67999456	0,151535643
0,67999456	0,151535643
0,67999456	0,151535643
0,67999456	0,151535643
0,709018718	0,156372495
0,709018718	0,156372495
0,709018718	0,156372495
0,775193798	0,168789594
0,625156289	0,134271427
0,625156289	0,134271427
0,625156289	0,134271427
0,625156289	0,134271427
0,625156289	0,134271427
0,668270516	0,142428619
0,668270516	0,142428619
0,668270516	0,142428619
0,69213732	0,141528082
0,69213732	0,141528082
0,69213732	0,141528082
0,656943897	0,133912104
0,656943897	0,133912104
0,656943897	0,133912104
0,856000219	0,173339867
0,605620155	0,122622744
0,605620155	0,122622744
0,605620155	0,122622744
0,605620155	0,122622744
0,605620155	0,122622744
0,605620155	0,122622744
0,605620155	0,122622744
0,605620155	0,122622744
0,711186971	0,141009715
0,683994528	0,134663
0,683994528	0,134663

0,683994528	0,134663
0,795441398	0,156406239
0,645994832	0,125943661
0,645994832	0,125943661
0,587268029	0,112134385
0,587268029	0,112134385
0,587268029	0,112134385
0,587268029	0,112134385
0,676041103	0,128142809
0,676041103	0,128142809
0,635404753	0,118484305
0,635404753	0,118484305
0,635404753	0,118484305
0,698372791	0,129412849
0,56999544	0,102671668
0,56999544	0,102671668
0,56999544	0,102671668
0,625156289	0,111498182
0,553709856	0,094118131
0,553709856	0,094118131
0,553709856	0,094118131
0,553709856	0,094118131
0,553709856	0,094118131
0,553709856	0,094118131
0,605620155	0,0988162
0,605620155	0,0988162
0,645994832	0,105158187
0,596302922	0,093061862
0,77999376	0,121062159
0,687228545	0,104802258
0,523779594	0,079347307
0,523779594	0,079347307
0,523779594	0,079347307
0,523779594	0,079347307
0,587268029	0,087663361
0,587268029	0,087663361
0,587268029	0,087663361
0,779648935	0,113873523
0,656943897	0,095873263
0,625156289	0,090738049
0,578502835	0,08259675
0,578502835	0,08259675
0,753280193	0,105443642
0,56999544	0,077839877
0,56999544	0,077839877
0,496919102	0,067158458
0,496919102	0,067158458
0,496919102	0,067158458
0,496919102	0,067158458
0,496919102	0,067158458
0,496919102	0,067158458
0,797006611	0,106967258
0,561734637	0,073372234

0,484496124	0,061868268
0,484496124	0,061868268
0,635404753	0,0807788
0,472679145	0,057042206
0,472679145	0,057042206
0,472679145	0,057042206
0,472679145	0,057042206
0,752053685	0,090013116
0,672133351	0,080388578
0,545911126	0,065230001
0,545911126	0,065230001
0,625156289	0,074146741
0,587268029	0,06766521
0,587268029	0,06766521
0,538329027	0,061521435
0,538329027	0,061521435
0,46142488	0,052634076
0,46142488	0,052634076
0,46142488	0,052634076
0,46142488	0,052634076
0,681703089	0,076863192
0,581395349	0,064446784
0,450694069	0,048602946
0,450694069	0,048602946
0,450694069	0,048602946
0,450694069	0,048602946
0,450694069	0,048602946
0,523779594	0,054753371
0,440451022	0,044912461
0,516795866	0,05166663
0,516795866	0,05166663
0,564461504	0,055694304
0,739285639	0,071965733
0,430663221	0,041530262
0,430663221	0,041530262
0,591750991	0,054924717
0,553709856	0,05053883
0,553709856	0,05053883
0,548486178	0,048145077
0,496919102	0,043451287
0,543360139	0,045865949
0,621813207	0,048616302
0,39550704	0,030550809
0,39550704	0,030550809
0,39550704	0,030550809
0,39550704	0,030550809
0,39550704	0,030550809
0,47851469	0,036587613
0,737041084	0,055435538
0,528541226	0,039661417
0,387596899	0,028333098
0,387596899	0,028333098
0,387596899	0,028333098

0,387596899	0,028333098
0,387596899	0,028333098
0,466984216	0,032645545
0,37999696	0,02628977
0,37999696	0,02628977
0,608790941	0,042042196
0,46142488	0,030842055
0,46142488	0,030842055
0,372689326	0,024405702
0,372689326	0,024405702
0,372689326	0,024405702
0,372689326	0,024405702
0,50999592	0,032682995
0,50999592	0,032682995
0,50999592	0,032682995
0,650815689	0,041162642
0,365657452	0,022667239
0,59326056	0,035023619
0,358886018	0,021062031
0,358886018	0,021062031
0,358886018	0,021062031
0,352360817	0,019578896
0,352360817	0,019578896
0,33999728	0,016939216
0,334135258	0,015765096
0,334135258	0,015765096
0,516795866	0,024221975
0,328471948	0,014677716
0,322997416	0,013670136
0,407996736	0,016611111
0,407996736	0,016611111
0,312578145	0,011869611
0,454215116	0,016620897
0,307616587	0,011065607
0,307616587	0,011065607
0,302810078	0,010319183
0,391512019	0,013295049
0,298151461	0,009625916
0,383759306	0,011898671
0,383759306	0,011898671
0,293634015	0,008981752
0,28499772	0,007826171
0,28499772	0,007826171
0,502068522	0,013522549
0,365657452	0,009024726
0,365657452	0,009024726
0,365657452	0,009024726
0,365657452	0,009024726
0,276854928	0,006826196
0,276854928	0,006826196
0,362240093	0,008540543
0,272955563	0,006377492
0,269164513	0,005959652

0,261889797	0,005207748
0,34606866	0,006486706
0,40096231	0,007307403
0,251686298	0,00426057
0,39550704	0,00663276
0,242248062	0,00349166
0,328471948	0,00466883
0,385030032	0,005463721
0,425930658	0,005963636
0,484496124	0,006663719
0,358886018	0,003201864
0,350238162	0,002635146
0,28499772	0,001751091
0,201873385	0,001236981
0,199792216	0,001160597
0,336066676	0,001872774
0,19775352	0,001089053
0,334135258	0,00178348
0,322997416	0,001329893
0,174593198	0,00048068
0,674472562	0,000813303
0,151405039	0,000168554
0,123438503	2,95233E-05
0,414014999	2,40469E-06

Genes

NCOA2;KAT2B;CREBBP;RXRA;TBL1XR1;CARM1;HIPK1;TBX5;TEAD1;CTGF;HIPK2
NRP2;MED17;MED14;CCND3;RPS6KA6;SPRED1;CDH2;DPYSL5;SALL4;DPYSL3;HOXA3;SCN5A;AP2M1;SH
AMER1;PPP2CA;TNKS2;FZD5;APC;TNKS;CSNK1A1;CTNNB1;LRP6
HDAC5;CREBBP;MAML1;DTX1;DTX4;HIF1A;RBX1;KAT2B;NEURL1B;HEYL;ITCH;APH1B;TBL1XR1
SHC2;NRP2;PSMD11;ROCK1;CUL3;SEMA3A;AGAP2;PPP2CA;RPS6KA6;SPRED1;RAP1A;APH1B;EFNB3;DI
APH1B;NRG3;ERBB4;PTPN11;BTRC;S100B;ESR1;EREG;RBX1
AMER1;PSMD11;TNKS;CUL3;XIAP;PRICKLE1;CDC73;LRP6;PPP2CA;ZNRF3;TNKS2;RBBP5;RUVBL1;RSPO
HDAC5;CREBBP;MAML1;DTX1;DTX4;HIF1A;RBX1;KAT2B;NEURL1B;HEYL;ITCH;APH1B;CREB1;TBL1XR1;A
AMER1;CREBBP;PSMD11;FZD5;SMURF2;CSNK1A1;TNKS;CUL3;XIAP;CDC73;RBX1;LRP6;PPP2CA;SFRP1
AMER1;HDAC5;PSMD11;MAML1;TNKS;FOXO1;LRP6;PPP2CA;NEURL1B;APH1B;TNKS2;ERBB4;TRIM24;CF
AMER1;PPP2CA;APC;CSNK1A1;CTNNB1
AMER1;PPP2CA;APC;CSNK1A1;CTNNB1
AMER1;PPP2CA;APC;CSNK1A1;CTNNB1
AMER1;PPP2CA;APC;CSNK1A1;CTNNB1
AMER1;PPP2CA;APC;CSNK1A1;CTNNB1
AMER1;PPP2CA;APC;CSNK1A1;CTNNB1
NAPA;NAPB;SCOC;NAA30;RHOBTB3;VTI1A;NAA35;VPS54;NAA38
NCOA2;PRKAB2;CREBBP;CREB1;RXRA;TBL1XR1;CARM1;TFAM;PPARGC1B
AMER1;PPP2CA;FZD5;APC;CSNK1A1;CTNNB1;LRP6
NCOA2;CREBBP;RXRA;CREB1;TBL1XR1;CARM1;TFAM;PPARGC1B
DPYSL5;SEMA3A;DPYSL3;PLXNA2;PLXNA3
MAP2K1;CDK1;PTPN11;IL6R
NCOA2;CREBBP;RXRA;CREB1;TBL1XR1;CARM1;NRIP1;HIF1A
AMER1;PPP2CA;APC;CSNK1A1;CTNNB1
PHLPP2;SHC2;PSMD11;PDE1B;SH3KBP1;CUL3;FOXO1;PPP2CA;SPRED1;RAP1A;ERBB4;MAPK1;SH3GL2;
RPS6KA6;CREB1;CAMK4;MAPK1;RAF1;CALM1;GRIN2B
PHLPP2;ADCYAP1R1;SHC2;RALA;PSMD11;PDE1B;CUL3;FOXO1;PPP2CA;SPRED1;RAP1A;ERBB4;MAPK1
PSMD11;UBA6;CUL3;LTN1;UBE2J1;ZNRF2;RNF217;FBXO3;BTRC;UNKL;RNF111;TRIM41;SMURF2;ZBTB16
RPS6KA6;CREB1;MAPK1;RAF1;CALM1;GRIN2B
NCOA2;CREBBP;RXRA;TBL1XR1;CARM1;NRIP1
KAT2B;HDAC5;NEURL1B;HEYL;CREBBP;APH1B;MAML1;TBL1XR1;RBX1
KAT2B;HDAC5;NEURL1B;HEYL;CREBBP;APH1B;MAML1;TBL1XR1;RBX1
KAT2B;HDAC5;NEURL1B;HEYL;CREBBP;APH1B;MAML1;TBL1XR1;RBX1
KAT2B;HDAC5;NEURL1B;HEYL;CREBBP;APH1B;MAML1;TBL1XR1;RBX1
KAT2B;HDAC5;NEURL1B;HEYL;CREBBP;APH1B;MAML1;TBL1XR1;RBX1
MAP2K1;MAPK1;RAF1
ATP2B4;ATP2B2;ATP2B1;CALM1
KAT2B;HIPK1;TBX5;HIPK2
HDAC5;HEYL;KAT2B;CREBBP;MAML1;TBL1XR1;HIF1A;RBX1
CREB1;ADCY9;PDE1B;CAMK4;MAPK1;CALM1
SHC2;PSMD11;CUL3;FOXO1;PPP2CA;CCND3;SPRED1;RAP1A;ERBB4;IGF2BP1;MAPK1;IL6R;MAP2K1;MM
RPS6KA6;CREB1;CAMK4;MAPK1;RAF1;CALM1;GRIN2B
PHLPP2;SHC2;PSMD11;CUL3;FOXO1;PPP2CA;SPRED1;APH1B;RAP1A;ERBB4;MAPK1;BTRC;MAP2K1;GA
NRP2;RPS6KA6;MAP2K1;DCX;MAPK1;SCN5A;SCN3B;L1CAM;SH3GL2;AP2M1;SCN1A;FGFR1
AMER1;PPP2CA;APC;CSNK1A1
AMER1;PPP2CA;APC;CSNK1A1
AMER1;PPP2CA;APC;CSNK1A1
AMER1;PPP2CA;APC;CSNK1A1
AMER1;PPP2CA;APC;CSNK1A1
AMER1;PPP2CA;APC;CSNK1A1
NCOA2;CREBBP;CREB1;RXRA;TBL1XR1;CARM1;NRIP1;BTRC;HIF1A
PPP2CA;PDE1B;PDE3B;ATP2B4;PDE5A;PTPN11;ATP2B2;ATP2B1;CALM1;LRP8;PRKG1

NAPA;NAPB;SCOC;NAA30;DCTN2;RHOBTB3;DYNLL2;CYTH3;KIF5C;MAN1A2;KIF26B;VTI1A;NAA35;VPS54
NCOA2;CREBBP;RXRA;GPAM;TBL1XR1;SP1;CARM1
MAP2K1;CDK1;MAPK1;PTPN11;IL6R
BMPR2;SMURF2;SMAD9;SMAD7;BMPR1A
NCOA2;CREBBP;PHF20;DNMT3A;MSL2;ARID5B;GATAD2B;PHF8;ING4;KAT2B;ZZZ3;MECOM;KANSL1;TBL1
NCOA2;CREBBP;PHF20;DNMT3A;MSL2;ARID5B;GATAD2B;PHF8;ING4;KAT2B;ZZZ3;MECOM;KANSL1;TBL1
ABCA1;NCOA2;CREBBP;CTGF;MED17;MED14;RXRA;TBL1XR1;SP1;SIN3A;CARM1;TEAD1;PPARGC1B
NCOA2;CREBBP;RXRA;GPAM;TBL1XR1;INSIG2;SP1;CARM1
NRG3;ERBB4;GAB1;EREG
PSMD11;UBA6;CUL3;LTN1;UBE2J1;ZNRF2;RNF217;FBXO3;BTRC;UNKL;RNF111;TRIM41;SMURF2;ZBTB16
MAPK1;PTPN11;IL6R
GPC1;SLIT2;SRGAP2
RPS6KA6;MAPK1;L1CAM;SH3GL2;AP2M1
CREB1;ADCY9;PDE1B;CAMK4;CALM1
CREB1;ADCY9;PDE1B;CAMK4;CALM1
PHLPP2;GAB1;PTPN11;FOXO1;EREG;PPP2CA;CREB1;NRG3;ERBB4;AGO1;MAPK1;FGFR1;TNRC6B
PHLPP2;GAB1;PTPN11;FOXO1;EREG;PPP2CA;CREB1;NRG3;ERBB4;AGO1;MAPK1;FGFR1;TNRC6B
PHLPP2;GAB1;PTPN11;FOXO1;EREG;PPP2CA;CREB1;NRG3;ERBB4;AGO1;MAPK1;FGFR1;TNRC6B
PHLPP2;GAB1;PTPN11;FOXO1;EREG;PPP2CA;CREB1;NRG3;ERBB4;AGO1;MAPK1;FGFR1;TNRC6B
PHLPP2;GAB1;PTPN11;FOXO1;EREG;PPP2CA;CREB1;NRG3;ERBB4;AGO1;MAPK1;FGFR1;TNRC6B
PHLPP2;GAB1;PTPN11;FOXO1;EREG;PPP2CA;CREB1;NRG3;ERBB4;AGO1;MAPK1;FGFR1;TNRC6B
PHLPP2;SHC2;ADCYAP1R1;RALA;PSMD11;PDE1B;CUL3;FOXO1;PPP2CA;SPRED1;RAP1A;APH1B;ERBB4
PHLPP2;SHC2;PSMD11;PDE1B;CUL3;FOXO1;PPP2CA;SPRED1;RAP1A;ERBB4;MAPK1;MAP2K1;GAB1;PTI
PHLPP2;SHC2;PSMD11;PDE1B;CUL3;FOXO1;PPP2CA;SPRED1;RAP1A;ERBB4;MAPK1;MAP2K1;GAB1;PTI
PHLPP2;SHC2;PSMD11;PDE1B;CUL3;FOXO1;PPP2CA;SPRED1;RAP1A;ERBB4;MAPK1;MAP2K1;GAB1;PTI
PHLPP2;SHC2;PSMD11;PDE1B;CUL3;FOXO1;PPP2CA;SPRED1;RAP1A;ERBB4;MAPK1;MAP2K1;GAB1;PTI
PDE1B;PDE3B;PDE5A;PRKG1
PHLPP2;GAB1;PTPN11;FOXO1;EREG;PPP2CA;CREB1;NRG3;ERBB4;AGO1;MAPK1;FGFR1;TNRC6B
PHLPP2;GAB1;PTPN11;FOXO1;EREG;PPP2CA;CREB1;NRG3;ERBB4;AGO1;MAPK1;FGFR1;TNRC6B
ITPK1;IP6K1;NUDT4
PHLPP2;SHC2;PSMD11;PDE1B;CUL3;FOXO1;PPP2CA;SPRED1;RAP1A;ERBB4;MAPK1;MAP2K1;GAB1;PTI
PHLPP2;SHC2;PSMD11;PDE1B;CUL3;FOXO1;PPP2CA;SPRED1;RAP1A;ERBB4;MAPK1;MAP2K1;GAB1;PTI
ABCA1;NCOA2;CREBBP;MED14;RXRA;TBL1XR1;SP1;CARM1;TEAD1;PPARGC1B;CTGF;MED17
PHLPP2;SHC2;PSMD11;PDE1B;CUL3;FOXO1;PPP2CA;SPRED1;RAP1A;ERBB4;MAPK1;MAP2K1;GAB1;PTI
PHLPP2;SHC2;PSMD11;PDE1B;CUL3;FOXO1;PPP2CA;SPRED1;RAP1A;ERBB4;MAPK1;MAP2K1;GAB1;PTI
PRKAB2;MAP2K1;SHC2;PSMD11;CUL3;PDE3B;GAB1;IRS4;PTPN11;IL17RD;GRIN2B;EREG;RBX1;PPP2CA;
PRKAB2;MAP2K1;SHC2;PSMD11;CUL3;PDE3B;GAB1;IRS4;PTPN11;IL17RD;GRIN2B;EREG;RBX1;PPP2CA;
PRKAB2;MAP2K1;SHC2;PSMD11;CUL3;PDE3B;GAB1;IRS4;PTPN11;IL17RD;GRIN2B;EREG;RBX1;PPP2CA;
DFFA;OCLN;ROCK1;APC;CTNNB1;CLSPN;KPNA1
PHLPP2;SHC2;PSMD11;PDE1B;CUL3;FOXO1;PPP2CA;SPRED1;RAP1A;ERBB4;MAPK1;MAP2K1;GAB1;PTI
MAP2K1;ANTXR2;CALM1
NEURL1B;ITCH;APH1B;DTX1;DTX4
PPP2CA;MEF2A;MAPK10;CREB1;MAPK1
KAT2B;CREBBP;MAML1;AGO1;E2F1;TNRC6B
APC;RBBP5;XIAP;CTNNB1;BTRC;SOX6
NCOA2;MED14;CCND3;CREBBP;RXRA;TBL1XR1;CARM1;EBF1;MED17
PHLPP2;SHC2;PSMD11;PDE1B;CUL3;FOXO1;PPP2CA;SPRED1;RAP1A;ERBB4;MAPK1;MAP2K1;GAB1;PTI
MAP2K1;MAPK1;L1CAM;FGFR1
ZNRF3;FZD5;RSPO2;LRP6
PHLPP2;SHC2;PSMD11;PDE1B;CUL3;FOXO1;TIAL1;PPP2CA;SPRED1;RAP1A;ERBB4;MAPK1;MAP2K1;GAI
CPSF7;SF3B3;SRSF1;HNRNPU;HNRNPR;LSM5;ELAVL2;HNRNPK;PABPN1;TRA2B;SNRPD3;HNRNPC;SRS
PRKAB2;MAP2K1;SHC2;PSMD11;CUL3;PDE3B;GAB1;PTPN11;IL17RD;GRIN2B;EREG;RBX1;PPP2CA;SPRE
ING4;NCOA2;KAT2B;CREBBP;ZZZ3;KANSL1;ATXN7;PHF20;RUVBL1;MSL2;EPC1

NDC1;FYTTD1;CPSF7;SF3B3;SRSF1;HNRNPU;HNRNPR;LSM5;ELAVL2;NXF1;HNRNPK;PABPN1;TRA2B;S
SP1;AGO1;E2F1;MAPK1;TNRC6B
ENAH;GPC1;SLIT2;SRGAP2;SOS2
CREB1;ADCY9;PDE1B;CAMK4;CALM1
CREBBP;CTNNB1
SH3KBP1;LTN1;KIF5C;FBXO3;BTRC;UNKL;RNF111;AP2M1;PRKG1;SH3GL2;CXADR;TAP2;GAB1;DYNLL2;
RBL1;SMURF2;USP9X;SP1;RNF111;SMAD7
CREB1;ADCY9;PDE1B;CAMK4;MAPK1;CALM1
PRKAB2;MAP2K1;SHC2;PSMD11;CUL3;PDE3B;GAB1;PTPN11;IL17RD;GRIN2B;EREG;RBX1;PPP2CA;SPRE
PHLPP2;SHC2;PSMD11;PDE1B;CUL3;FOXO1;TIAL1;PPP2CA;SPRED1;RAP1A;ERBB4;MAPK1;MAP2K1;GA
MAP2K1;SHC2;PSMD11;CUL3;PTPN11;IL17RD;GRIN2B;EREG;RBX1;PPP2CA;SPRED1;RAP1A;NRG3;ERB
KAT2B;CREBBP;MAML1
KAT2B;CREBBP;MAML1
NRG3;ERBB4;EREG
CAPZA1;MAPK1;S100B
PTK2B;PTPN11;CD47
GABRA1;GABRB1;GABRA3
CREB1;ADCY9;PDE1B;CAMK4;MAPK1;CALM1
CREB1;ADCY9;PDE1B;CAMK4;CALM1
CREB1;ADCY9;PDE1B;CAMK4;CALM1
NAPA;NAPB;SCOC;RALA;DCTN2;SNAP23;RHOBTB3;GJC1;CYTH3;GJA3;KIF5C;MAN1A2;VTI1A;VPS54;GJ
DOCK5;PDE1B;PDE3B;GATA6;LRP8;PPP2CA;GNA13;RAP1A;CAPZB;SIN3A;KIF5C;C1QBP;MAPK1;KIF1B;J
PHLPP2;SHC2;PSMD11;CUL3;FOXO1;PPP2CA;SPRED1;RAP1A;ERBB4;MAPK1;LYN;MAP2K1;GAB1;PTPN
MEF2A;PPP2CA;MAPK10;MAP2K1;CREB1;MAPK1;BTRC;S100B
SEMA3A;PLXNA2;PLXNA3
GPC1;SLIT2;SOS2
PDP2;RXRA;PDHB
PHLPP2;SHC2;PSMD11;PDE1B;CUL3;FOXO1;PPP2CA;SPRED1;RAP1A;ERBB4;MAPK1;MAP2K1;GAB1;PT
MEF2A;PPP2CA;CREB1;MAPK1
CREB1;ADCY9;PDE1B;CAMK4;CALM1;FGFR1
PRKAB2;RALA;MYO1C;SNAP23;LNPEP;CALM1;RAB11A
MEF2A;PPP2CA;MAPK10;MAP2K1;CREB1;MAPK1;BTRC
RPS6KA6;MAPK1
CPSF7;SF3B3;SRSF1;HNRNPU;HNRNPR;LSM5;ELAVL2;HNRNPK;PABPN1;TRA2B;SNRPD3;HNRNPC;SRS
LYN;EFNA3;APH1B;EFNB3;MMP2;AP2M1
PHLPP2;SHC2;PSMD11;CUL3;FOXO1;PPP2CA;SPRED1;RAP1A;ERBB4;MAPK1;BTRC;LYN;MAP2K1;NFAT
FZD3;FZD5;AGO1;CTNNB1;CALM1;PRKG1;TNRC6B
RBL1;E2F1;DYRK1A;CDK1
PDE1B;PDE3B;PDE5A;PRKG1
PTPN11;BTRC;RBX1
NRG3;ERBB4;EREG
CREB1;CALM1;GRIN2B
OCLN;ROCK1;APC;CTNNB1;CLSPN
LYN;PHLPP2;GAB1;PTPN11;FOXO1;EREG;PPP2CA;CREB1;NRG3;ERBB4;AGO1;MAPK1;FGFR1;TNRC6B
PHLPP2;PPP2CA;NRG3;ERBB4;GAB1;MAPK1;PTPN11;EREG;FGFR1
PPP2CA;CCND3;RBL1;E2F1;CKS1B
PPP2CA;CCND3;RBL1;E2F1;CKS1B
MAP2K1;RAP1A;MAPK1;IL17RD;RAF1
PPP2CA;MAPK1;PTPN11
NRG3;ERBB4;EREG
RAP1A;RAF1;PRKG1
SEMA3A;PLXNA2;PLXNA3
KAT2B;KCNIP2;ATP2B4;ATP2B2;SCN5A;ATP2B1;SCN3B;CALM1;HIPK1;TBX5;SCN1A;HIPK2

SLC29A3;SLC28A3
NEK7;CDK1
AGO1;TNRC6B
TPM3;KCNIP2;TMOD3;TMOD2;ATP2B4;ATP2B2;ATP2B1;HIPK1;TBX5;SORBS3;HIPK2;KAT2B;SCN5A;SCN3
SH3KBP1;SPRY1;SH3GL2;AP2M1
ATP2B4;ATP2B2;ATP2B1;CALM1
HS3ST3B1;CHST7;HPSE2;GPC1;B3GAT2;OMD;CHP1;HAS2;GPC5;PAPSS2;CHST3
LYN;MAP2K1;SHC2;PSMD11;CUL3;PTPN11;IL17RD;GRIN2B;EREG;RBX1;PPP2CA;SPRED1;RAP1A;NRG3;E
PRKAB2;MAP2K1;SHC2;PSMD11;CUL3;PDE3B;GAB1;PTPN11;IL17RD;GRIN2B;EREG;RBX1;PPP2CA;SPRE
GJC1;GJA3;GJA9;AP2M1
EDEM3;EDEM1;MLEC
PPP2CA;PTPN11;LRP8
NCKAP1;NRP2;MAP2K1;SHC2;PSMD11;ROCK1;CUL3;IL17RD;GRIN2B;EREG;RBX1;PPP2CA;SPRED1;RAP
LYN;EFNA3;APH1B;EFNB3;ROCK1;MMP2;MYH10;GRIN2B;AP2M1
SPRED1;NRG3;ERBB4;ELMO1;CRK;HIF1A;EREG
ROCK1;DPYSL5;SEMA3A;DPYSL3;PLXNA2;MYH10;PLXNA3
PPP2CA;ADCY9;CREB1;PDE1B;CAMK4;PPP1R1B;MAPK1;CALM1
LYN;PHLPP2;PSMD11;SH3KBP1;GAB1;PTPN11;FOXO1;EREG;PPP2CA;CREB1;NRG3;ERBB4;AGO1;MAPK
SCN5A;SCN3B;L1CAM;SCN1A
DCTN2;RAB3GAP2;DYNLL2;PAFAH1B2
MEF2A;CDH2;MYOD1;CTNNB1
MEF2A;CDH2;MYOD1;CTNNB1
GFPT1;PGM3
ITCH;ERBB4
FZD5;LRP6
AMER1;PPP2CA;PSMD11;APC;CSNK1A1;CTNNB1;BTRC;RBX1
MEF2A;PPP2CA;MAPK10;MAP2K1;CREB1;MAPK1;BTRC;S100B
MEF2A;PPP2CA;MAPK10;MAP2K1;CREB1;MAPK1;BTRC;S100B
MEF2A;PPP2CA;MAPK10;MAP2K1;CREB1;MAPK1;BTRC;S100B
RBL1;CARM1;CDK1
NRG3;ERBB4;EREG
MEF2A;PPP2CA;MAPK10;MAP2K1;CREB1;MAPK1;PTPN11;BTRC;S100B
MEF2A;PPP2CA;MAPK10;MAP2K1;CREB1;MAPK1;PTPN11;BTRC;S100B
MEF2A;PPP2CA;MAPK10;MAP2K1;CREB1;MAPK1;PTPN11;BTRC;S100B
ATP8A2;ATP2B4;ATP2B2;ATP11A;ATP2B1;CALM1
AMER1;HDAC5;PSMD11;MAML1;TNKS;ANTXR2;FOXO1;LRP6;PPP2CA;NEURL1B;ADAMTS5;ADAMTS2;AP
GABRA1;RPS6KA6;GABRB1;ADCY9;CREB1;CAMK4;GABRA3;MAPK1;RAF1;CALM1;GRIN2B;AP2M1
MAP2K1;SHC2;PSMD11;CUL3;PTPN11;IL17RD;GRIN2B;EREG;RBX1;PPP2CA;SPRED1;RAP1A;NRG3;ERBE
MAP2K1;SHC2;PSMD11;CUL3;PTPN11;IL17RD;GRIN2B;EREG;RBX1;PPP2CA;SPRED1;RAP1A;NRG3;ERBE
MAP2K1;SHC2;PSMD11;CUL3;PTPN11;IL17RD;GRIN2B;EREG;RBX1;PPP2CA;SPRED1;RAP1A;NRG3;ERBE
MAP2K1;SHC2;PSMD11;CUL3;PTPN11;IL17RD;GRIN2B;EREG;RBX1;PPP2CA;SPRED1;RAP1A;NRG3;ERBE
PPP2CA;NRG3;ERBB4;GAB1;MAPK1;PTPN11;EREG;FGFR1
MEF2A;PPP2CA;MAPK10;MAP2K1;CREB1;MAPK1;BTRC;S100B
MAP2K1;SHC2;RALA;PSMD11;CUL3;IL17RD;GRIN2B;EREG;RBX1;PPP2CA;SPRED1;RAP1A;NRG3;ERBB4;
GJC1;GJA3;GJA9;AP2M1
NCKAP1;MAP2K1;SHC2;PSMD11;ROCK1;CUL3;IL17RD;GRIN2B;EREG;RBX1;PPP2CA;SPRED1;RAP1A;NR
GTF2H1;RNF111;GTF2H5;PIAS1;RBX1
MAP2K1;SHC2;PSMD11;CUL3;IL17RD;GRIN2B;EREG;RBX1;PPP2CA;SPRED1;RAP1A;NRG3;ERBB4;MAPK
GJC1;GJA3;GJA9
MEF2A;PPP2CA;MAPK10;MAP2K1;CREB1;MAPK1;BTRC;S100B
MEF2A;PPP2CA;MAPK10;MAP2K1;CREB1;MAPK1;BTRC;S100B
PHLPP2;PSMD11;GAB1;PTPN11;FOXO1;EREG;PPP2CA;CREB1;NRG3;ERBB4;AGO1;MAPK1;BTRC;FGFR1
KAT2B;CREBBP;MAML1;AGO1;E2F1;TNRC6B

CDH6;CADM3;CDH2;CTNNB1
KAT2B;MYO1C;SIN3A;TDG;DNMT3A;TET3;GTF2H1;DEK;GATAD2B;GTF2H5
NAPA;CYTH3;NAPB;MAN1A2;VTI1A
CREB1;ADCY9;PDE1B;CAMK4;CALM1
XRCC5;KPNA1
CREB1;FOXO1
GPC1;SLIT2
ADRA2B;ADRA2A
CREB1;NRG3;ERBB4;GAB1;PTPN11;FOXO1;EREG;FGFR1
CDH6;CADM3;CDH2;CLDN18;PTK2B;CTNNB1;PARVA;PTPN11;ACTN4;CD47;CLDN1
MAP2K1;SHC2;PSMD11;CUL3;IL17RD;GRIN2B;EREG;RBX1;PPP2CA;SPRED1;RAP1A;NRG3;ERBB4;MAPK
NDC1;PHC2;HNRNPK;TDG;HNRNPC;BMI1;SMC1A;SENP2;PIAS1
MAP2K1;SHC2;PSMD11;CUL3;PTPN11;IL17RD;GRIN2B;EREG;RBX1;PPP2CA;SPRED1;RAP1A;NRG3;ERBE
NEURL1B;APH1B;CREB1;MAML1
RBL1;SP1;RNF111;SMAD7
PPP2CA;MAPK1;PTPN11;FGFR1
NDC1;PHC2;HNRNPK;HNRNPC;BMI1
NRG3;ERBB4;CDC37;GAB1;EREG
USP15;RBL1;SMURF2;USP9X;SP1;RNF111;SMAD7
MEF2A;PPP2CA;MAPK10;MAP2K1;CREB1;MAPK1;BTRC;S100B
MAP2K1;SHC2;RALA;PSMD11;CUL3;IL17RD;GRIN2B;EREG;RBX1;PPP2CA;SPRED1;RAP1A;NRG3;ERBB4;
CREB1;ADCY9;PDE1B;CAMK4;CALM1
FYTTD1;CPSF7;PABPN1;SRSF1;SNRPD3;SRSF9
FYTTD1;CPSF7;PABPN1;SRSF1;SNRPD3;SRSF9
FYTTD1;CPSF7;PABPN1;SRSF1;SNRPD3;SRSF9
ESCO1;SMC1A
EDEM3;EDEM1
EOMES;GATA6
CREBBP;HIF1A
PPP2CA;CDK1
MAPK10;MAPK1
ZDHHC21;CALM1
CHRM3;MARCKS
HPSE2;GPC1;GPC5
MEF2A;PPP2CA;MAPK1
LYN;CREBBP;RAF1
PRKAB2;CREBBP;COX7B;PHF20;SETD9;USP2;GTF2H1;HIPK1;GATAD2B;GTF2H5;GLS;HIPK2;PPP2CA;CN
NRG3;ERBB4;GAB1;PTPN11;EREG;FGFR1
CDH6;CADM3;CDH2;CLDN18;CTNNB1;CLDN1
MAP2K1;SHC2;PSMD11;CUL3;IL17RD;GRIN2B;EREG;RBX1;PPP2CA;SPRED1;RAP1A;CREB1;NRG3;ERBB
LYN;EFNA3;ROCK1;MYH10
ABCA1;NCOA2;PRKAB2;CREBBP;ACSL6;MED17;CTGF;MED14;RXRA;GPAM;TBL1XR1;SP1;SIN3A;CARM1;
MAPK10;CBX6;PHC2;AGO1;E2F1;MAPK1;BMI1;TNRC6B
CNOT6;RBL1;CARM1;E2F1;CDK1
MAP2K1;SHC2;PSMD11;CUL3;IL17RD;GRIN2B;EREG;RBX1;PPP2CA;SPRED1;RAP1A;NRG3;ERBB4;MAPK
MAP2K1;SHC2;PSMD11;CUL3;IL17RD;GRIN2B;EREG;RBX1;PPP2CA;SPRED1;RAP1A;NRG3;ERBB4;MAPK
MAP2K1;SHC2;PSMD11;CUL3;IL17RD;GRIN2B;EREG;RBX1;PPP2CA;SPRED1;RAP1A;NRG3;ERBB4;MAPK
MAP2K1;SHC2;PSMD11;CUL3;IL17RD;GRIN2B;EREG;RBX1;PPP2CA;SPRED1;RAP1A;NRG3;ERBB4;MAPK
MAP2K1;SHC2;PSMD11;CUL3;IL17RD;GRIN2B;EREG;RBX1;PPP2CA;SPRED1;RAP1A;NRG3;ERBB4;MAPK
LYN;PPP2CA;PTPN11
CCND3;PSMD11;MMP2;AGO1;IGF2BP1;CDK1;FOXO1;TNRC6B
MEF2A;PPP2CA;MAPK10;MAP2K1;CREB1;MAPK1;BTRC;S100B
MEF2A;PPP2CA;MAPK10;MAP2K1;CREB1;MAPK1;BTRC;S100B

MEF2A;PPP2CA;MAPK10;MAP2K1;CREB1;MAPK1;BTRC;S100B
MEF2A;PPP2CA;MAPK10;MAP2K1;CREB1;MAPK1;BTRC;S100B
MAP2K1;SHC2;PSMD11;CUL3;IL17RD;GRIN2B;EREG;RBX1;PPP2CA;SPRED1;RAP1A;NRG3;ERBB4;PTK2[illegible]
NAPA;NAPB;SCOC;RALA;DCTN2;SNAP23;RHOBTB3;GJC1;CYTH3;HSPH1;GJA3;KIF5C;MAN1A2;VTI1A;VP[illegible]
ADCY9;CREB1;PDE1B;CAMK4;CALM1
ST6GAL2;ALG6;GFPT1;ST8SIA3;PGM3;ALG14;ST6GALNAC3
PRKAB2;RXRA
PTPN11;IL6R
CTNNB1;CALM1
SLC24A2;CALM1
NFATC3;CALM1
OCLN;CTNNB1
GNRHR;TSHR
ALAD;ALAS2
DOCK5;CREBBP;CBX5;CAPZB;KIF5C;SIN3A;CAPZA1;KIF26B;ITPK1;GATA6;KIF1B
SMURF2;USP9X;RNF111
PRKAB2;EEF2K;PDE3B;GAB1;PTPN11;EIF4E;FGFR1
MAP2K1;SHC2;PSMD11;CUL3;IL17RD;GRIN2B;EREG;RBX1;PPP2CA;SPRED1;RAP1A;NRG3;ERBB4;MAPK
CHST7;GPC1;B3GAT2;GPC5;CHST3
NAPA;NAPB;DCTN2;KIF5C;KIF26B;RAB3GAP2;KIF1B;DYNLL2;PAFAH1B2
SYT5;PPP2CA;CHRM3;PRKAB2;MARCKS;ADCY9;RAP1A;KCNC2;ADRA2A
MAP2K1;SHC2;PSMD11;CUL3;IL17RD;GRIN2B;EREG;RBX1;PPP2CA;SPRED1;RAP1A;NRG3;ERBB4;MAPK
NCKAP1;CHRM3;NPFFR1;MAML1;IRS4;AMOT;TIAL1;CCND3;RUVBL1;FNTB;SOX6;BTRC;IL6R;AP2M1;SH3[illegible]
VKORC1;GPC1;GPC5;LRP8;LRP12
FYTTD1;CPSF7;PABPN1;SRSF1;SRSF9
FYTTD1;CPSF7;PABPN1;SRSF1;SRSF9
RAP1A;ADRA2B;CRK;ADRA2A
NDC1;PHC2;HNRNPK;TDG;HNRNPC;BMI1;SMC1A;PIAS1
CPSF7;PABPN1;SNRPD3
CPSF7;PABPN1;SNRPD3
PPP2CA;PPP1R1B;CALM1
MEF2A;PPP2CA;MAPK10;MAP2K1;CREB1;MAPK1;PTPN11;BTRC;S100B
ALDH2;ADH1B
GPC1;GPC5
GPC1;GPC5
PPP2CA;E2F1
CREB1;MAML1
PPP2CA;MAPK1
LYN;PTPN11
FZD3;PSMD11;FZD5;SMURF2;AGO1;CTNNB1;PRICKLE1;CALM1;AP2M1;PRKG1;TNRC6B
MAP2K1;SHC2;PSMD11;CUL3;IL17RD;GRIN2B;EREG;RBX1;PPP2CA;SPRED1;RAP1A;NRG3;ERBB4;MAPK
GLUD2;PSPH;GLS
PPP2CA;MAP2K1;RAF1
RRM1;RRM2B;CMPK1
OCLN;DFFA;PSMD11;ROCK1;APC;E2F1;XIAP;CTNNB1;CLSPN;DYNLL2;KPNA1;APPL1
COX7B;PRKAB2;SESN3;RRM2B;AGO1;GLS;TNRC6B
FZD5;AP2M1
SH3GL2;AP2M1
XRCC5;DTX4
DFFA;KPNA1
DFFA;KPNA1
USP2;TNFAIP3
ETNK1;EPT1

NDC1;HS3ST3B1;MGAM;CHST7;PFKFB3;HPSE2;B3GAT2;OMD;SORD;PAPSS2;HK2;PPP2CA;GPC1;HAS2;(
MAP2K1;SHC2;PSMD11;CUL3;IL17RD;GRIN2B;EREG;RBX1;PPP2CA;SPRED1;RAP1A;NRG3;ERBB4;MAPK
HS3ST3B1;HPSE2;GPC1;B3GAT2;GPC5
GPC1;OMD;GPC5
GPC1;B3GAT2;GPC5
CREBBP;HIF1A;RBX1
CREBBP;HIF1A;RBX1
SPRED1;ELMO1;CRK
USP2;XIAP;TNFAIP3
USP15;SMURF2;SMAD7
GABRA1;GABRB1;GABRA3
ITCH;PSMD11;SMURF2;CSNK1A1;CUL3;CDC73;RBX1
PRKAB2;EEF2K;PDE3B;EIF4E
MECOM;RBBP5;NSD1;PRDM16
PPP2CA;MAP2K1;MAPK1;RAF1
OCLN;DFFA;PSMD11;ROCK1;APC;E2F1;XIAP;CTNNB1;CLSPN;DYNLL2;KPNA1;APPL1
LYN;CXADR;PTPN11;CD47;L1CAM;SELE;JAM2;CD244
CDH6;CADM3;CDH2;CLDN18;CTNNB1;PARVA;CLDN1
PCYT1B;PLA2G12A;ABHD4;GPAM;ETNK1;EPT1;GPCPD1;CDS2
PPP2CA;PRKAB2;PHF20;SETD9;USP2;JMY;CDK1;HIPK1;GATAD2B;RAD9A;HIPK2
MAP2K1;ANTXR2;CALM1
RXRA;PDP2;PDHB
XRCC5;KPNA1
PTPN11;PIAS1
CDK1;MAPK1
S1PR1;S1PR3
MAT2A;MAT2B
ITCH;ADCY9;PSMD11;SMURF2;CSNK1A1;CUL3;GPC5;BTRC;CDC73;RBX1
LYN;MAP2K1;SHC2;PSMD11;CUL3;IL17RD;GRIN2B;EREG;RBX1;PPP2CA;MAPK10;SPRED1;RAP1A;NRG3;
GPC1;GPC5;LRP8;LRP12
LYN;EFNB3;ROCK1;GRIN2B
PHLPP2;CRP;NCKAP1;SHC2;PSMD11;PDE1B;CUL3;TNFAIP3;DTX4;FOXO1;NCKIPSD;PPP2CA;SPRED1;R/
USP1;DTL;RBX1
CREBBP;RBBP5;RUVBL1;CTNNB1;CDC73
MEF2A;PPP2CA;MAPK10;MAP2K1;CREB1;MAPK1;PTPN11;BTRC;S100B
MGAT5;ST8SIA3
EDEM3;EDEM1
LSM5;DCP2
CPSF7;PABPN1
CDC37;GAB1
CDC37;GAB1
SFRP1;LRP6
RAP1A;CRK
NCOA2;RXRA
NCOA2;RXRA
NDC1;FYTTD1;NXF1;SRSF1;EIF4E;SRSF9
PPP2CA;MAPK1;PTPN11
NDC1;PHC2;TDG;BMI1;SMC1A;PIAS1
SCN5A;SCN3B;CALM1;SCN1A
ITPK1;IP6K1;CALM1;NUDT4
XRCC5;DTX4
MAP2K1;BTRC
MME;REN

GABRA1;GABRB1;GABRA3;GRIN2B;GLS;RPS6KA6;ADCY9;CREB1;ALDH2;CAMK4;MAPK1;CALM1;RAF1;A
KIAA0101;USP1;DTL;RBX1
HS3ST3B1;GPC1;GPC5
PPP2CA;MAPK1;PTPN11
USP2;XIAP;TNFAIP3
HAS2;CHP1
E2F1;CDK1
ROCK1;MYH10
CALM1;GRIN2B
ADCY9;CREB1
ALAD;ALAS2
LYN;MAP2K1;SHC2;PSMD11;CUL3;PTPN11;IL17RD;GRIN2B;IL18BP;RBX1;EREG;PPP2CA;SPRED1;RAP1A
KAT2B;GTF2H1;GTF2H5;GATAD2B
ST6GAL2;GFPT1;ST8SIA3;PGM3;ST6GALNAC3
FZD3;PSMD11;SMURF2;FZD5;PRICKLE1
PPP2CA;PFKFB3;HK2
USP15;SMURF2;SMAD7
SYT5;CHRM3;MARCKS;RAP1A;KCNC2;ADRA2A
SORD
HAS2
GJC1
GJC1
MAN1A2
E2F1
RBX1
RBX1
HIF1A
CDK1
CDK1
TAC3
CHRM3
ADCYAP1R1
XIAP
XIAP
XIAP
ARL3
GPC1;FNTB;GPC5;CALM1;LRP8;LRP12;METAP2
RXRA;PDP2;DLST;PDHB
ADD3;ADD2
HIF1A;RBX1
LYN;CRK
PPM1L;VAPA;VAPB
ST6GAL2;ST8SIA3;ST6GALNAC3
NDC1;NXF1;EIF4E
PPP2CA;E2F1;CDK1
FNTB;CALM1;METAP2
TPM3;CALM1;SORBS3
RUVBL1;GTF2H1;RNF111;GTF2H5;RBX1;PIAS1
GABRA1;GABRB1;ATP8A2;GABRA3;ATP2B4;ANO6;ATP2B2;ATP11A;ATP2B1;TPCN1;CLCN5;CALM1;RAF1
CREBBP;XRCC5;CTNNB1;DTX4;S100B
NDC1;NXF1;EIF4E
NDC1;NEK7;CDK1
PPP2CA;MAPK1;PTPN11

ITCH;TNFAIP3;UBE2K
FNTB;CALM1;METAP2
TPM3;TMOD3;TMOD2
GPC1;GPC5
GPC1;GPC5
GPC1;GPC5
SETD9;JMY
CDC37;GAB1
CDC37;GAB1
CDC37;GAB1
CNOT6;LSM5;EIF4E;DCP2
EDEM3;NAPA;NAPB;ST6GAL2;ALG6;DCTN2;EDEM1;ST8SIA3;GFPT1;ALG14;DYNLL2;MAN1A2;MGAT5;PGI
ATP2B4;ATP2B2;ATP2B1;CALM1
PPP2CA;CCND3;PSMD11;RBL1;RPA4;DYRK1A;CDK1;E2F1;CKS1B
PPP2CA;USP2;CDK1
SORD
SENP2
DTX4
RUVBL1
FGFR1
CDK1
EMB
HSPH1
ADCYAP1R1
MOCS3
PAPSS2
HPGD
PCMT1
NDC1;FYTTD1;NXF1;SRSF1;SRSF9
CHST7;CHST3
ZDHHC21;CALM1
ZDHHC21;CALM1
PPP2CA;USP2;CDK1
NCKIPSD;NCKAP1;MAPK1
SERP1;EDEM1;GFPT1;SEC62
RRM1;RRM2B;ADK;CMPK1;NUDT5;PFAS
NAPA;NAPB;DCTN2;BMI1;ADAMTS5;ADAMTS2;MAN1A2;MGAT5;TDG;PGM3;DAG1;MLEC;EDEM3;NDC1;G
NEURL1B;APH1B
CALM1;MYH10
MMP2;KLK13
MEF2A;PPP2CA;MAPK10;MAP2K1;CREB1;MAPK1;PTPN11;BTRC;S100B
NDC1;NXF1;EIF4E
ADAMTS5;ADAMTS2;GPC1;OMD;DAG1;GPC5
LYN;CREBBP;CDC34;PSMD11;NFATC3;BTRC;RAF1;CALM1
PSMD11;TNKS2;SMURF2;TNKS
FYTTD1;CPSF7;PABPN1;SRSF1;GTF2H1;SNRPD3;GTF2H5;SRSF9
OMD
OMD
OMD
XRCC5
AP2M1
DCX
NRP2

HIF1A
PPP2CA
CADM3
CTNNB1
CREB1
CREB1
XIAP
NUDT5
CARNS1
NDC1;NXF1;EIF4E
TRIM24;GAB1;FGFR1
NDC1;PPP2CA;SET;NEK7;CDK1;MAPK1;PHF8
EEF2K;EIF4E
PPP2CA;CDK1
SERP1;EDEM1;GFPT1;SEC62
GABRA1;GABRB1;ADCY9;GABRA3
MAPK10;PHC2;CBX6;SP1;AGO1;E2F1;TERF2IP;MAPK1;BMI1;TNRC6B
NCKAP1;SH3KBP1;LTN1;TNFAIP3;IL18BP;SPRED1;KIF5C;TRIM3;FBXO3;BTRC;UNKL;IL6R;AP2M1;SH3GL2
PRKAB2;EEF2K;EIF4E
NDC1;PABPN1;KPNA1
GTF2H1;GTF2H5;RBX1
FZD3;PSMD11;SMURF2;FZD5;PRICKLE1;AP2M1
COX7B;MAML1;PHF20;SETD9;RORB;ZNF25;MED17;CTGF;GLS;PPP2CA;MED14;RXRA;SESN3;ZNF706;E2I
CNOT6;EIF4E
ALDH6A1;PPM1K
NDC1;PRKAB2;CREBBP;PHC2;CBX6;MTMR3;ST13;HSPA4L;BMI1;SOD2;HIF1A;DYNLL2;RBX1;MAPK10;HS
ALDH2
SLC17A6
TDG
TDG
TDG
IGF2BP1
GPC5
MGAM
SET
E2F1
PRKAB2
ELMO1
EIF2B2
PARVA
NPFFR1
GPCPD1
CDS2
LYN;MAPK1
ROCK1;MYH10
NCOA2;RXRA
NCOA2;RXRA
EFEMP1;LOXL4;FBLN5
NDC1;PABPN1;KPNA1
SET;CDK1;PHF8
USP2;XIAP;TNFAIP3
ITCH;PSMD11;BTRC;RBX1
PSMD11;CSNK1A1;BTRC;RBX1

PSMD11;CSNK1A1;BTRC;RBX1
SDR16C5;RXRA;PDHB
E2F1;XIAP;DYNLL2
EOMES;SALL4;GATA6
AGAP2;PTPN11;SLIT2
CREB1;FOXO1
ARID5B;PHF8
ITPK1;CALM1
DLST;PDHB
NDC1;CREBBP;HSPH1;ST13;HSPA4L;MAPK1
SLC4A10
RUNX1
TDG
LOXL4
SMC1A
SENP2
AP2M1
AP2M1
APH1B
AGO1
PTGFR
CHRM3;ADRA2B;ADRA2A
CHRM3;PTGFR;MAP2K1;SHC2;NPFFR1;PSMD11;CUL3;IL17RD;GRIN2B;RBX1;EREG;PPP2CA;SPRED1;GR
NCKIPSD;NCKAP1;MYO1C;ELMO1;MAPK1;CRK
GABRA1;GABRB1;KCNC2;GABRA3;GRIN2B;GLS;GJC1;RPS6KA6;ADCY9;CREB1;ALDH2;CAMK4;MAPK1;C
PPP2CA;PFKFB3;SLC25A12;CALM1;HK2
CREBBP;ITCH;TNFAIP3;S100B;UBE2K
MGAT5;ST8SIA3
CREBBP;HSPH1
DTX4;S100B
NDC1;HSPH1;ST13;HSPA4L;MAPK1
KIF5C;KIF26B;KIF1B
ITCH;ADCY9;PSMD11;CSNK1A1;BTRC;RBX1
NAPA;NAPB;KIF5C;KIF26B;KIF1B
MME;KIF5C;CTNNB1;REN;INHBA
CTNNB1;CALM1
GTF2H1;GTF2H5
GTF2H1;GTF2H5
PTPN11;IL6R
RAP1A;CRK
ROCK1;MYH10
NCOA2;RXRA
TDG
AP2M1
CALM1
CALM1
PRKAB2
SNRPD3
CDK1
MAPK1
APH1B
GAB1
PTPN11

RAF1
LYN
ADCY9
CALM1
APPL1
INHBA
DIO2
OTC
NCOA2;TSFM;PRKAB2;CREBBP;DCTN2;ARL3;MRPL27;HAUS3;DYNLL2;RAB11A;MRPL42;RXRA;CREB1;TE
NCKIPSD;LYN;NCKAP1;MYO1C;ELMO1;MAPK1;CRK
ADCY9;ADRA2B;ADRA2A
ELMO1;AP2M1
PLA2G12A;GPAM
LYN;SH3KBP1;CALM1
SNRPD3
E2F1
APH1B
APH1B
FGFR1
FGFR1
GAB1
CDK1
CDK1
SRD5A1
GATA6;ADRA2A
E2F1;DYNLL2
TDRKH;FKBP6
GTF2H1;GTF2H5
PCYT1B;PLA2G12A;MTMR3;ABHD4;GPAM;ETNK1;EPT1;GPCPD1;CDS2
USP2;XIAP;TNFAIP3
SPRED1;PSMD11;CUL3;RBX1
SERP1;EDEM1;GFPT1;SEC62;DCP2
EFEMP1;FBLN5
NDC1;KPNA1
GTF2H1;GTF2H5
CLDN18;CLDN1
CYB5R4
CYB5R4
CA12
OMD
CHP1
LNPEP
CDK1
INHBA
PFAS
DLST
TDRKH;COX7B;MAML1;PHF20;SETD9;HNRNPU;PHAX;HNRNPR;RORB;SMG7;ELAVL2;MED17;CTGF;GLS;I
TRIM24;FGFR1
ITCH;TNFAIP3
NDC1;PHAX;SNRPD3
NDC1;PHAX;SNRPD3
RUVBL1;GTF2H1;RNF111;GTF2H5;RBX1;PIAS1
CREBBP;CNOT6;RXRA;RBBP5;HOXA3

CREBBP;CNOT6;RXRA;RBBP5;HOXA3
NAPA;SORT1;SNAP23;SH3GL2
NAPA;SORT1;SNAP23;SH3GL2
NDC1;NEK7;CDK1
RXRA;RORB;ESR1
NDC1;KPNA1
GTF2H1;GTF2H5
GNA13;MAPK1
SUMF1
SMC1A
PPP2CA
PPP2CA
E2F1
SALL4
SPRED1
RALA
S100B
LYN
INHBA
ADK
NDC1;PSMD11;ELMO1;BTRC;AP2M1;KPNA1;RBX1
SF3B3;SRSF1;SNRPD3
GTF2H1;GTF2H5
GTF2H1;GTF2H5
GTF2H1;GTF2H5;RBX1
PPP2CA;PSMD11;RPA4;CDK1;E2F1;CKS1B
LGI2
RUNX1
PIAS1
PHF20
CREBBP
XIAP
EMB
CTNNB1
ADCY9
ADCY9
HPGD
KAT2B;MYO1C;DEK;GATAD2B
CREBBP;HSPH1
ADK;PFAS
NAPA;SORT1;SH3GL2
PPM1L;VAPA;VAPB;SUMF1
COX7B;RXRA;TIMMDC1;PDP2;DLST;ETFA;UQCR10;PDHB
NDC1;KPNA1
NCOA2;RXRA
CDK1
AP2M1
DTX4
E2F1
NEURL1B
NEURL1B
APH1B
CALM1

FGFR1
RAP1A
PTGFR
KAT2B;GTF2H1;GATAD2B;GTF2H5
KAT2B;NFIA;TFAM;GTF2H1;GATAD2B;GTF2H5
PSMD11;CUL3;RBX1
TERF2IP;SMC1A;FKBP6
MAPK10
XIAP
XIAP
PARVA
LYN
MYH10
ADCY9
PLA2G12A
ADAMTS5;ADAMTS2
CLSPN;RAD9A
NAPA;NAPB;DCTN2;DYNLL2
CHRM3;HPSE2;NUDT5;CTGF;NUDT4;GLS;CHP1;PSPH;PRKAB2;OMD;DIO2;ACSL6;ADCY9;TBL1XR1;CMPI
KAT2B;MYO1C;DEK
SLC24A2;SLC4A10;SLC17A6;SLC1A4;CALM1
CDC34;PSMD11;NFATC3;BTRC;CALM1
KAT2B;GTF2H1;GATAD2B;GTF2H5
SLC29A3;SLC28A3
ADAMTS5;ADAMTS2
RUVBL1;RBX1
CLCN5;ANO6;RAF1;CALM1;TPCN1
ABCA2
AP2M1
DCP2
DCP2
DCP2
GRIN2B
ADCY9
PLA2G12A
PLA2G12A
TDRKH;NDC1;AGO1;FKBP6;TNRC6B
GPR27;ADCYAP1R1;ADCY9;PDE1B;PDE3B;PDE7B;TSHR
MMP2;COL19A1
FBXW4;DCAF7
CSNK1A1
TRIM24
PTPN11
PTPN11
LYN
DIO2
PAPSS2
DCTN2;KIF5C;DYNLL2;SH3GL2;AP2M1
ADAMTS5;ADAMTS2;DAG1
GTF2H1;GTF2H5;RBX1
LYN;NFATC3;CALM1
CARNS1;DLST
CREBBP

CDK1
PLXNA2
EFNB3
ABCA1
DLST
ADAMTS2;COL6A5;COL19A1
GTF2H1;GTF2H5;RBX1
TRIM24;GAB1;FGFR1
ADAMTS2;LOXL4;COL6A5;COL19A1
NDC1;HK2
RAP1A;KCNC2
SLC24A2;GABRB1;ATP8A2;SLC1A4;ADD3;HK2;ADD2;SLC22A17;SLC17A6;EMB;NDC1;ABCA2;GABRA1;GA
FOXO1
CTNNB1
AMOT
PCYT1B
GTF2H1;GTF2H5
GTF2H1;GTF2H5
GTF2H1;GTF2H5
ADCY9;RAB11A
KCNC2;KCNH1
LYN;GNA13;RAP1A;MAPK1;SCG3;PTPN11;ACTN4;CALM1;RAF1;ADRA2B;CRK;ADRA2A
DCTN2;CDK1;HAUS3;BTRC
MTMR3;ABHD4;PPM1L;HPGD;INSIG2;MED17;CTGF;MED14;RXRA;SIN3A;TEAD1;PPARGC1B;ABCA1;NCO
GTF2H1;GTF2H5
GTF2H1;GTF2H5
GTF2H1;GTF2H5
GTF2H1;GTF2H5
GTF2H1;GTF2H5
GTF2H1;GTF2H5
GTF2H1;GTF2H5
GTF2H1;GTF2H5
GTF2H1;GTF2H5
MAP2K1;BTRC
AP2M1
CNOT6
FGFR1
S100B
RAB11A
ACSL6
COX7B;TIMMDC1;ETFA;UQCR10
GALNT7;ADAMTS5;ADAMTS2;DAG1;ST6GALNAC3
DAG1;CD47;JAM2
PRKAB2;MTMR3;DYNLL2
PRKAB2;HIPK1;HIPK2;RAD9A
CARM1;DNMT3A
SDR16C5
C1QBP
GAB1
GAB1
ACTN4
GRIN2B
DCTN2;CDK1;HAUS3

DCTN2;CDK1;HAUS3
GATAD2B
NFIA
AGO1
PTPN11
CDK1
CTNNB1
GNA13
PLA2G12A
ITCH;TNFAIP3
NCOA2;RXRA;ALDH2;ADH1B
NDC1;PSMD11;XRCC5;ELMO1;GTF2H1;BTRC;GTF2H5;AP2M1;KPNA1;RBX1
XRCC5;GTF2H1;GTF2H5;RBX1;PIAS1;KIAA0101;TDG;RUVBL1;USP1;CLSPN;DTL;RNF111;RAD9A
NDC1;HK2
GLS
S100B
KIF5C
PPP2CA;LYN;PTPN11
NDC1;EIF4E;KPNA1
NDC1;EIF4E;KPNA1
DCTN2;CDK1;HAUS3
SLC17A6;SLC1A4
SIN3A;GTF2H1;GTF2H5
TAP2
DCP2
PTPN11
L1CAM
PLA2G12A
SHC2;PSMD11;CUL3;IL18BP;PPP2CA;SPRED1;RAP1A;ERBB4;TRIM3;PTK2B;MAPK1;BTRC;EIF4E;IL6R;KP
NAPA;NAPB;DCTN2;MAN1A2;MGAT5;ST8SIA3;DYNLL2
PSMD11;AZIN1
DCTN2;CDK1;HAUS3;RAB11A
PSMD11;BTRC
ARL3;RAB11A
ADCY9;RAB11A
ST6GALNAC3
PTPN11
TIAL1
PTPN11
S100B
WLS
GNA13;ROCK1;SOS2
RUVBL1;RSF1
RUVBL1;RSF1
SIN3A;GTF2H1;GTF2H5
GTF2H1;GTF2H5;RBX1
OMD
APPL1
KCNIP2
PSMD11;RBX1
LYN;PTPN11
SP1;NABP1;PHAX
PPP2CA;NCKIPSD;NCKAP1;NCOA2;DIAPH2;ROCK1;CTNNB1;MAPK1;CALM1;SRGAP2;MYH10

DCP2
PRKAB2
RUVBL1
ADRA2A
LOXL4;COL6A5
PSMD11;BTRC
PSMD11;CDK1;BTRC
DCTN2;CDK1;HAUS3
DCTN2;CDK1;HAUS3
VKORC1;MOCS3;GPC1;GPC5;LRP8;LRP12
HSPH1
NDC1
NDC1
KIAA0101
RPA4
CALM1
ADCYAP1R1
SRD5A1
BMI1;DCAF7;PCMT1;ADAMTS5;ADAMTS2;SERP1;KIF5C;MAN1A2;PGM3;DAG1;SEC62;FBXW4;NDC1;ST6G
NDC1;RPP14
EIF3J;EIF4E
EIF3J;EIF4E
GTF2H1;GTF2H5
PSMD11;TAP2;LNPEP
PSMD11;RPA4;E2F1
PSMD11;RPA4;E2F1
PPP2CA;PSMD11;DCTN2;CDK1;E2F1;HAUS3;BTRC
SLC1A4
AP2M1
AP2M1
NDC1
TERF2IP
UTP15;DIEXF
EIF3J;EIF4E
PSMD11;BTRC
PSMD11;AZIN1;OTC
ADCYAP1R1;FZD3;FZD5
MRPL42;TSFM;MRPL27
RUVBL1;TERF2IP
PSMD11;CDK1
PSMD11;CKS1B
SET;PSMD11;DCTN2;RSF1;PHF8;CKS1B;PPP2CA;CCND3;RUVBL1;E2F1;MAPK1;CLSPN;BTRC;NDC1;ESC
OMD
MMP2
FOXO1
NDC1
DNMT3A
SLC25A12
CREBBP
GNA13
COX7B;TIMMDC1;ETFA;UQCR10
TERF2IP;SMC1A;FKBP6
PSMD11;CDK1;BTRC

PSMD11;CDK1;BTRC
NDC1;SLC24A2;SLC4A10;SLC17A6;SLC1A4;EMB;CALM1;SLC29A3;HK2;RUNX1;SLC28A3
NCOA2;MYH10
TBL1XR1;GATAD2B
LYN
NDC1
ADCY9
SOD2
RUVBL1;TERF2IP;RSF1
PSMD11;SET;DCP2
GTF2H1;GTF2H5
PSMD11;BTRC
APH1B;SOS2
LYN;CDC34;PSMD11;BTRC
NCOA2
ABCA1
MTMR3
NCOA2;RXRA
SH3GL2
ENAH
NDC1
TDG
TDG
USP1
GTF2H1;GTF2H5
PSMD11;GPC5
MRPL42;TSFM;MRPL27
PSMD11;TAP2
PTGFR;CHRM3;NPFFR1;GPR37;ADRA2B;ADRA2A;TSHR;XK;GNRHR;S1PR1;TAC3;S1PR3;AGTR2
NDC1;XRCC5;GTF2H1;GTF2H5;KPNA1
RAD9A
KIAA0101
COL6A5
SRD5A1
GALNT7;ST6GALNAC3
PSMD11;BTRC
GPAM;ACSL6
NDC1;MAP2K1;PSMD11;XRCC5;GTF2H1;ANTXR2;GTF2H5;RBX1;PABPN1;ELMO1;BTRC;CALM1;AP2M1;KI
ENAH;CDC34;PSMD11;BTRC
TRIM3;PTPN11;PIAS1
GTF2H1;GTF2H5
PSMD11;E2F1
ADAMTS5;ADAMTS2;EFEMP1;MMP2;DAG1;LOXL4;CD47;COL6A5;COL19A1;JAM2;FBLN5
NAPA;NAPB
PSMD11;CKS1B
C1QBP
SUMF1
RAD9A
ADCY9
ADCY9
ADAT2
NDC1;SET;PSMD11;ESCO1;DCTN2;NEK7;DYRK1A;HAUS3;SMC1A;PHF8;CKS1B;PPP2CA;CCND3;RBL1;RF
PSMD11;CKS1B

DNMT3A
GRM5
ARHGAP20;ARHGAP31;SRGAP2;SOS2
SUMF1
DYNLL2
NFIA
NFIA
PSMD11;DIO2;DLST;PDHB;PPM1K;PAPSS2;AZIN1;GLS;GLUD2;ALDH6A1;CARNS1;PSPH;OTC
PPP2CA;PSMD11;DCTN2;CDK1;HAUS3;BTRC
PSMD11;CDK1
ABCA1;NCEH1
PSMD11;ESCO1;SMC1A;CKS1B
CDC34;PSMD11;BTRC
PPP2CA;CDK1;SMC1A
NDC1;AGO1
PSMD11;CDK1
DAG1
NDC1
RAD9A
ACSL6
NCOA2;RXRA;ALDH2;MAT2A;ADH1B;MAT2B;PAPSS2
MAT2A;MAT2B;PAPSS2
CREBBP
GATAD2B
GAB1
CRP
SLC22A17
PSMD11;CDK1
NDC1
PSMD11;CDK1
PSMD11;E2F1
NDC1;RPP14;ADAT2
NCKAP1;NCOA2;ROCK1;PPP2CA;NCKIPSD;ARHGAP20;ARHGAP31;DIAPH2;CTNNB1;MAPK1;CALM1;SRG
ABCA2
SOS2
NAPA;NAPB;DCTN2;DYNLL2
PSMD11;RPA4;E2F1
SCG3;ACTN4;CALM1
ADAMTS5;MMP2;COL19A1
CDK1;RAD9A
PPP2CA;CDK1;SMC1A
DCTN2;ARL3;CDK1;HAUS3;DYNLL2;RAB11A
PSMD11
PSMD11
LYN
CRP
TIMMDC1
CLSPN;RAD9A
CHRM3;PTGFR;FZD3;ADCYAP1R1;GPR37;NPFFR1;FZD5;ADRA2B;ADRA2A;TSHR;GRM5;XK;GNRHR;S1PI
SCG3;ACTN4;CALM1
EIF3J
PSMD11
PSMD11

PSMD11
PSMD11
APH1B;SOS2
GLS
PSMD11
CHRM3;PTGFR;GRM5;NPFFR1;GNRHR;TAC3
MRPL42;MRPL27
MRPL42;MRPL27
XRCC5
PSMD11
PSMD11
PSMD11
EIF2B2;EIF3J;EIF4E
EIF2B2;EIF3J;EIF4E
PPP2CA;DIAPH2;SRGAP2
NDC1;PPP2CA;SET;PSMD11;NEK7;CDK1;MAPK1;SMC1A;PHF8
HPGD
NDC1;TRIM3;PTPN11;EIF4E;KPNA1;PIAS1
PSMD11
PSMD11
SLC25A12
DAG1
PSMD11
PSMD11
PSMD11
CRP
PSMD11;CDK1;CLSPN;RAD9A
TERF2IP
MTMR3
PSMD11;BTRC
FBXW4;DCAF7
PSMD11
NDC1;GTF2H1;GTF2H5
PSMD11
PSMD11
RAD9A
KCNC2;KCNH1
PSMD11
EMB;RUNX1
FBXW4;DCAF7
PSMD11
USP9X
PTPN11
NPFFR1;GPR37;XK;TAC3;AGTR2
PPP2CA;SMG7
PPP2CA;SMG7
EIF3J;EIF4E
EIF3J;EIF4E
PSMD11
PSMD11
EIF3J;EIF4E
PSMD11
PSMD11

HSPH1
CLSPN;RAD9A
XRCC5;CLSPN;RAD9A
MAPK1
NDC1;PABPN1;KPNA1
CRP
CLSPN;RAD9A
EIF2B2;EIF3J;EIF4E
PSMD11;CDK1;CLSPN;RAD9A
ADCY9;S1PR1;S1PR3;AGTR2;ADRA2B;ADRA2A
PPP2CA;PSMD11;SMC1A
UTP15;RPP14;DIEXF
NDC1;KPNA1
EIF3J
PSMD11
PPP2CA;PSMD11;SMC1A
MOCS3
PPP2CA;PSMD11;SMC1A
UTP15;RPP14;DIEXF
PAPSS2
GPR27;CHRM3;PTGFR;SHC2;ADCYAP1R1;NPFFR1;PSMD11;ROCK1;PDE1B;CUL3;PDE3B;PPP2CA;GNA1[illegible]
NDC1
CXADR
GPR27;CHRM3;PTGFR;ADCYAP1R1;NPFFR1;ROCK1;PDE1B;PDE3B;ADRA2B;ADRA2A;TSHR;GNA13;GR[illegible]

H3GL2;SCN1A;MEF2A;EOMES;NCOA2;MAP2K1;MMP2;EBF1;IL17RD;RBX1;EREG;ENAH;CREB1;NRG3;

PYSL5;ERBB4;GPC1;DPYSL3;PLXNA2;MAPK1;SCN5A;SLIT2;SRGAP2;SCN3B;MYH10;AP2M1;SH3GL2;

)2;BTRC;SOX6;AP2M1;PRKG1;WLS;CREBBP;FZD3;FZD5;SMURF2;CSNK1A1;RBX1;SFRP1;APC;AGO1
AGO1;E2F1;TNRC6B
;ZNRF3;TNKS2;APC;RBBP5;RUVBL1;RSPO2;CTNNB1;BTRC;SOX6
REBBP;FZD5;CSNK1A1;GAB1;PTPN11;EREG;RBX1;KAT2B;HEYL;CREB1;NRG3;APC;TBL1XR1;CDC37;

;AP2M1;MAP2K1;GAB1;PTPN11;IL17RD;GRIN2B;EREG;RBX1;ADCY9;CREB1;NRG3;AGO1;CAMK4;ADA

;SH3GL2;AP2M1;MEF2A;MAP2K1;GAB1;PTPN11;IL17RD;GRIN2B;EREG;RBX1;ADCY9;CREB1;NRG3;A
;LNPEP;UBE2G1;RBX1;RNF126;ITCH;CDC34;HECW2;RLIM;ASB7;TRIP12;UBE2K

P2;PTPN11;IL17RD;GRIN2B;EREG;RBX1;NRG3;AGO1;CDK1;CALM1;RAF1;FGFR1;TNRC6B

B1;PTPN11;IL17RD;S100B;GRIN2B;ESR1;EREG;RBX1;ITCH;CREB1;NRG3;AGO1;CALM1;RAF1;FGFR1

-;RAB3GAP2;KIF1B;NAA38;PAFAH1B2

1XR1;ATXN7;RBBP5;NSD1;CARM1;PRDM16;RUVBL1;EPC1
1XR1;ATXN7;RBBP5;NSD1;CARM1;PRDM16;RUVBL1;EPC1

;TAP2;LNPEP;UBE2G1;RBX1;RNF126;ITCH;CDC34;HECW2;RLIM;ASB7;TRIP12;UBE2K

;MAPK1;AP2M1;SH3GL2;MEF2A;MAP2K1;GAB1;PTPN11;IL17RD;GRIN2B;RBX1;EREG;ADCY9;CREB1;I
PN11;IL17RD;GRIN2B;EREG;RBX1;ADCY9;CREB1;NRG3;AGO1;CAMK4;CALM1;RAF1;CRK;FGFR1;TNF
PN11;IL17RD;GRIN2B;EREG;RBX1;ADCY9;CREB1;NRG3;AGO1;CAMK4;CALM1;RAF1;FGFR1;TNRC6B
PN11;IL17RD;GRIN2B;EREG;RBX1;ADCY9;CREB1;NRG3;AGO1;CAMK4;CALM1;RAF1;FGFR1;TNRC6B
PN11;IL17RD;GRIN2B;EREG;RBX1;ADCY9;CREB1;NRG3;AGO1;CAMK4;CALM1;RAF1;FGFR1;TNRC6B

PN11;IL17RD;GRIN2B;EREG;RBX1;ADCY9;CREB1;NRG3;AGO1;CAMK4;CALM1;RAF1;FGFR1;TNRC6B
PN11;IL17RD;GRIN2B;EREG;RBX1;ADCY9;CREB1;NRG3;AGO1;CAMK4;CALM1;RAF1;FGFR1;TNRC6B

PN11;IL17RD;GRIN2B;EREG;RBX1;ADCY9;CREB1;NRG3;AGO1;CAMK4;COL6A5;CALM1;RAF1;CRK;FG
PN11;IL17RD;GRIN2B;EREG;RBX1;ADCY9;CREB1;NRG3;AGO1;CAMK4;CALM1;RAF1;FGFR1;TNRC6B
SPRED1;RAP1A;EEF2K;NRG3;ERBB4;MAPK1;CALM1;RAF1;EIF4E;FGFR1
SPRED1;RAP1A;EEF2K;NRG3;ERBB4;MAPK1;CALM1;RAF1;EIF4E;FGFR1
SPRED1;RAP1A;EEF2K;NRG3;ERBB4;MAPK1;CALM1;RAF1;EIF4E;FGFR1

PN11;IL17RD;GRIN2B;EREG;RBX1;ADCY9;CREB1;NRG3;AGO1;CAMK4;CALM1;RAF1;FGFR1;TNRC6B

PN11;IL17RD;GRIN2B;EREG;RBX1;ADCY9;CREB1;NRG3;AGO1;CAMK4;CALM1;RAF1;FGFR1;TNRC6B

B1;PTPN11;IL17RD;GRIN2B;EREG;RBX1;ADCY9;CREB1;NRG3;AGO1;CAMK4;CALM1;RAF1;FGFR1;TN
F9
ED1;RAP1A;EEF2K;NRG3;ERBB4;MAPK1;CALM1;RAF1;EIF4E;FGFR1

\RPD3;HNRNPC;EIF4E;SRSF9

RBX1;EREG;ENAH;RNF126;CDC34;CREB1;NRG3;HECW2;RAF1;PHLPP2;PSMD11;DCTN2;UBA6;CUL3;F

ED1;RAP1A;EEF2K;NRG3;ERBB4;MAPK1;CALM1;RAF1;EIF4E;FGFR1
B1;PTPN11;IL17RD;GRIN2B;EREG;RBX1;ADCY9;CREB1;NRG3;AGO1;CAMK4;CALM1;RAF1;FGFR1;TN
34;CDK1;MAPK1;CALM1;RAF1;IL6R;FGFR1

A9;KIF1B;AP2M1;SH3GL2;PRKAB2;NAA30;SORT1;LNPEP;DYNLL2;RAB11A;MYO1C;KIF26B;RAB3GAP2
AM2;PRKG1;LYN;CREBBP;CXADR;CBX5;ITPK1;ATP2B4;PTPN11;ATP2B2;ACTN4;ATP2B1;L1CAM;ADRA
11;IL17RD;GRIN2B;EREG;RBX1;CREB1;NRG3;AGO1;CALM1;RAF1;FGFR1;TNRC6B

PN11;IL17RD;GRIN2B;EREG;RBX1;ADCY9;CREB1;NRG3;AGO1;CAMK4;CALM1;RAF1;FGFR1;TNRC6B

F9

C3;GAB1;PTPN11;IL17RD;GRIN2B;RBX1;EREG;MAPK10;CDC34;CREB1;NRG3;AGO1;CALM1;RAF1;TNF

B;CALM1;SCN1A

ERBB4;MAPK1;CALM1;RAF1;CRK;FGFR1
ED1;RAP1A;EEF2K;NRG3;ERBB4;MAPK1;CALM1;RAF1;EIF4E;FGFR1

'1A;NRG3;ERBB4;ELMO1;CTNNB1;PTK2B;MAPK1;CALM1;RAF1;CRK;FGFR1

1;BTRC;CALM1;FGFR1;TNRC6B

'H1B;PABPN1;TNKS2;ERBB4;GPC1;TRIM24;DAG1;GPC5;BTRC;AP2M1;KPNA1;NDC1;CREBBP;MAP2K

34;MAPK1;CALM1;RAF1;FGFR1
34;MAPK1;CALM1;RAF1;FGFR1
34;MAPK1;CALM1;RAF1;FGFR1
34;MAPK1;CALM1;RAF1;FGFR1

;MAPK1;CALM1;RAF1;CRK;FGFR1

G3;ERBB4;ELMO1;CTNNB1;PTK2B;MAPK1;CALM1;RAF1;CRK;FGFR1

1;CALM1;RAF1;CRK;FGFR1

;TNRC6B

:1;CALM1;RAF1;CRK;FGFR1

34;MAPK1;CALM1;RAF1;FGFR1

;MAPK1;CALM1;RAF1;FGFR1

IOT6;RBL1;SESN3;RRM2B;AGO1;CARM1;JMY;CDK1;E2F1;RAD9A;TNRC6B

4;MAPK1;COL6A5;CALM1;RAF1;FGFR1

;TEAD1;PPARGC1B

:1;CALM1;RAF1;FGFR1
:1;CALM1;RAF1;FGFR1
:1;CALM1;RAF1;FGFR1
:1;CALM1;RAF1;FGFR1
:1;CALM1;RAF1;FGFR1

3;MAPK1;CALM1;RAF1;FGFR1
S54;GJA9;KIF1B;AP2M1;SH3GL2;PRKAB2;NAA30;SORT1;LNPEP;DYNLL2;RAB11A;MYO1C;KIF26B;RAI

1;CALM1;RAF1;FGFR1

1;CALM1;RAF1;FGFR1
GL2;RNF111;WLS;MEF2A;PRKAB2;MAP2K1;USP2;RBX1;EREG;SFRP1;ADCY9;TBL1XR1;CDC37;ADAM

1;CALM1;RAF1;FGFR1

:CHP1;GPC5;CALM1;SLC25A12;CHST3
:1;CALM1;RAF1;FGFR1

;ERBB4;MAPK1;CALM1;RAF1;FGFR1

AP1A;ERBB4;MAPK1;BTRC;MEF2A;LYN;CREBBP;MAP2K1;XRCC5;GAB1;NFATC3;PTPN11;S100B;IL17I

P2M1

;NRG3;ERBB4;PTK2B;MAPK1;BTRC;CALM1;RAF1;CRK;IL6R;FGFR1

M3;MLEC;ST6GALNAC3

ALNT7;PHC2;ST6GAL2;ALG6;EDEM1;GFPT1;ST8SIA3;ALG14;SMC1A;SENP2;DYNLL2;SUMF1;PIAS1;H

2;RNF111;PRKG1;KPNA1;MEF2A;NDC1;MAP2K1;CXADR;GAB1;TAP2;IL17RD;DYNLL2;RBX1;PIAS1;ERE

F1;TEAD1;RNF111;NCOA2;PRKAB2;CREBBP;SMURF2;USP9X;USP2;GTF2H1;TBX5;HIPK1;ESR1;GATA

PH1;SP1;AGO1;E2F1;TERF2IP;MAPK1;TNRC6B

RM5;RAP1A;CREB1;NRG3;ERBB4;GNRHR;MAPK1;TAC3;CALM1;RAF1;FGFR1

ALM1;RAF1;AP2M1;KCNH1

3L1XR1;CARM1;CDK1;TFAM;PPARGC1B

MED14;SESN3;SIN3A;SNRPD3;TEAD1;RNF111;UTP15;NDC1;NCOA2;PRKAB2;USP2;DNMT3A;DIEXF;K

K1;MTMR3;PFKFB3;PSMD11;ABHD4;INSIG2;CYB5R4;RAP1A;IP6K1;PPARGC1B;ABCA1;SYT5;CREBBP

\BRA3;SLC4A10;ATP2B4;ANO6;ATP2B2;ATP11A;ATP2B1;RAB11A;TPCN1;RUNX1;CLCN5;ADCY9;CALM

A2;PRKAB2;CREBBP;PLA2G12A;PCYT1B;SRD5A1;EPT1;ACSL6;GPCPD1;SUMF1;NCEH1;VAPA;GPAM;

'NA1;LYN;NDC1;MAP2K1;PTPN11;IL17RD;GRIN2B;RBX1;PIAS1;EREG;NRG3;CALM1;RAF1;CRK;FGFR1

GAL2;ALG6;MME;MMP2;ALG14;SMC1A;SENP2;DYNLL2;ADRA2A;SUMF1;PIAS1;ST6GALNAC3;SLC25A

O1;NEK7;DYRK1A;HAUS3;SMC1A;RBL1;RPA4;CDK1;TERF2IP;FKBP6;RAD9A

PNA1

PA4;CDK1;E2F1;MAPK1;BTRC

;AP2;MYH10;SOS2

R1;TAC3;S1PR3;AGTR2

3;GRM5;SPRED1;RAP1A;ERBB4;GNRHR;S1PR1;MAPK1;TAC3;S1PR3;FZD3;MAP2K1;GPR37;FZD5;IL1

/I5;ADCY9;GNRHR;S1PR1;TAC3;S1PR3;AGTR2;PDE7B;SOS2

;TBL1XR1;MYOD1;DCX;COL6A5;RAF1;SOS2;SHC2;PSMD11;ROCK1;CUL3;SEMA3A;GATA6;AGAP2;FO

;PLXNA3;SCN1A;LYN;MAP2K1;MMP2;PTPN11;L1CAM;IL17RD;GRIN2B;RBX1;EREG;ENAH;EFNA3;CRE

;CTNNB1;CALM1;TNRC6B

CTNNB1;FGFR1

AM12;SPRY1;CALM1;RAF1;FGFR1;TNRC6B

GO1;CAMK4;CALM1;RAF1;CRK;FGFR1;TNRC6B

;TNRC6B

NRG3;AGO1;CAMK4;CALM1;RAF1;SOS2;CRK;TNRC6B;FGFR1
RC6B

GFR1;TNRC6B

IRC6B

FOXO1;UBE2J1;PPP2CA;ZNRF2;RAP1A;ERBB4;RNF217;MAPK1;LYN;TRIM41;SMURF2;ZBTB16;LNPEP

IRC6B

2;NAA35;NAA38;CALM1;PAFAH1B2
A2B;SELE;ADRA2A;KIF26B;CAPZA1;SCG3;PDE5A;CD47;CALM1;RAF1;CRK;CD244

RC6B;FGFR1

1;FZD5;CSNK1A1;XRCC5;OMD;GAB1;GTF2H1;PTPN11;GTF2H5;RBX1;EREG;KAT2B;HEYL;CREB1;NR(

B3GAP2;NAA35;NAA38;CALM1;PAFAH1B2

I12;ELMO1;AGTR2;RAF1;PTGFR;SHC2;ADCYAP1R1;PSMD11;TNKS;PDE1B;CUL3;DTX1;DTX4;HIF1A;F

RD;GRIN2B;RBX1;EREG;MAPK10;ITCH;MYO1C;CDC34;CREB1;ADCY9;NRG3;AGO1;CAMK4;CAPZA1;E

INRNPK;HNRNPC;ST6GALNAC3

EG;ENAH;RNF126;CDC34;CREB1;ADCY9;NRG3;HECW2;ELMO1;RAF1;CRK;PHLPP2;CRP;SHC2;PSMD

AD2B;GTF2H5;HIPK2;SMAD7;KAT2B;CNOT6;ZNF70;RBL1;RRM2B;SP1;TBL1XR1;AGO1;CARM1;JMY;CD

:AT2B;RBL1;RRM2B;TBL1XR1;TET3;TFAM;ZNF597;FKBP6;DCP2;SRSF9;FYTTD1;SET;PSMD11;SF3B3;S

';MGAM;SRD5A1;EPT1;AZIN1;ALDH6A1;GLUD2;NCEH1;SP1;CALM1;LRP12;CDS2;ALAS2;COX7B;MOC[illegible]

[illegible]1;RAF1;SLC29A3;SLC28A3

;SP1;VAPB;TBL1XR1;ETNK1;CARM1;CDS2

.12;DCP2;NAPA;NAPB;DCTN2;GATA6;MGAT5;TDG;MLEC;EIF4E;EDEM3;GALNT7;PHC2;EIF2B2;USP9X;

7RD;GRIN2B;ADRA2B;ADRA2A;TSHR;RBX1;EREG;XK;CREB1;ADCY9;NRG3;CAMK4;PPP1R1B;AGTR2

XO1;PPP2CA;RAP1A;APH1B;RXRA;EFNB3;RBBP5;ERBB4;GPC1;PLXNA2;MAPK1;SLIT2;SCN3B;SRGA

B1;NRG3;DCX;COL6A5;CALM1;RAF1;SOS2;FGFR1

';PTPN11;UBE2G1;ITCH;AGO1;RLIM;ASB7;TRIP12;CALM1;UBE2K;TNRC6B;FGFR1

G3;APC;TBL1XR1;CDC37;ELMO1;CTNNB1;CALM1;FGFR1

OXO1;NCKIPSD;ARHGAP20;RAP1A;APH1B;TAC3;SRGAP2;MYH10;LYN;CREBBP;FZD3;FZD5;SMURF2

ELMO1;CTNNB1;CALM1;RAF1;UBE2K;CRK;TNRC6B;FGFR1

[illegible]11;DCTN2;UBA6;PDE1B;CUL3;DTX4;FOXO1;UBE2J1;NCKIPSD;PPP2CA;ZNRF2;RAP1A;ERBB4;RNF21

[illegible]K1;ZNF597;RAD9A;TNRC6B

SRSF1;ZNF25;PPP2CA;NXF1;RXRA;ZNF706;PABPN1;TDG;TRA2B;IGF2BP1;E2F1;RPP14;EIF4E;CREBE

S3;KCNC2;ADK;ETFA;PFAS;HK2;MED17;ALAD;MED14;ALDH2;SIN3A;TEAD1;VKORC1;HS3ST3B1;NDC

;EDEM1;GFPT1;ST8SIA3;KLK13;INHBA;HNRNPK;EIF3J;CTNNB1;REN;HNRNPC

:;PDE7B;CALM1;RAF1;SOS2;FGFR1

AP2;MYH10;PLXNA3;LYN;CREBBP;LGI2;PTPN11;L1CAM;GRIN2B;CNOT6;EFNA3;CARM1;CTNNB1;CALM

;SMAD9;INHBA;GRIN2B;ESR1;SMAD7;DIAPH2;ARHGAP31;SP1;AGO1;PPP1R1B;CDK1;CALM1;LRP12;[illegible]

7;PTK2B;MAPK1;EIF4E;LYN;CREBBP;TRIM41;SMURF2;XRCC5;ZBTB16;NFATC3;LNPEP;UBE2G1;PTPI

3P;CPSF7;EIF2B2;SMURF2;USP9X;GTF2H1;DEK;TBX5;HIPK1;LSM5;ESR1;GATAD2B;GTF2H5;HIPK2;AI

1;NCOA2;PCYT1B;PLA2G12A;CHST7;ITPK1;SORD;GPCPD1;ADRA2A;SUMF1;RRM2B;SLC25A12;CHS

M1;FGFR1

BMPR1A;FGFR1;GPR27;NRP2;BMPR2;SH3KBP1;PDE3B;TNFAIP3;CDC73;SDR16C5;GRM5;SPRED1;El

N11;S100B;GRIN2B;MAPK10;ITCH;MYO1C;AGO1;CAPZA1;CAMK4;RLIM;ASB7;CTNNB1;TRIP12;CALM1

DAT2;SMAD7;CNOT6;ZNF70;MYO1C;HNRNPK;NFIA;SP1;AGO1;CARM1;EIF3J;JMY;CDK1;NABP1;HNRN

T3;PPM1L;HPGD;ADH1B;DLST;ZDHHC21;PPM1K;MAT2B;PDHB;UQCR10;LRP8;PAPSS2;PPP2CA;RXRA

EF2K;GNRHR;RSPO2;PRKG1;NCOA2;GPR37;MMP2;GAB1;IL17RD;ADRA2B;ADRA2A;KAT2B;CREB1;RI

l;UBE2K;TNRC6B;FGFR1

NPC;RAD9A;TNRC6B

A;MAT2A;GPC1;CARNS1;HAS2;GPC5;CA12;RRM1;TIMMDC1;B3GAT2;MARCKS;PDP2;GPAM;VAPA;VAP

BL1;NRG3;COL6A5;CRK;SOS2;PHLPP2;AMER1;HDAC5;RALA;USP15;ROCK1;XIAP;PRICKLE1;PDHB;L

'B;ETNK1;CARM1;OTC

RP8;LRP6;PPP2CA;GNA13;NEURL1B;ZNRF3;RXRA;TNKS2;RBBP5;ERBB4;GPC1;IGF2BP1;E2F1;S1PR

[illegible]1;PTK2B;MAPK1;GPC5;S1PR3;EIF4E;USP9X;CSNK1A1;PTPN11;S100B;TSHR;HEYL;ITCH;XK;APC;CA[illegible]

MK4;CTNNB1;SPRY1;PDE7B;METAP2;TNRC6B
