# Supplementary material for: Circulating miR-330-3p in Late Pregnancy is Associated with Pregnancy Outcomes Among Lean Women with GDM
Source: Sci Rep. 2020 Jan 22;10:908. doi: 10.1038/s41598-020-57838-6 (PMC6976655; doi:10.1038/s41598-020-57838-6)
Supplement: Supplementary file 1 — Supplementary data. [file 41598_2020_57838_MOESM1_ESM.zip › Supplimentary File_EnrichR_Analysis WikiPathways_2019_Human.pdf]

Term
Brain-Derived Neurotrophic Factor (BDNF) signaling pathway WP2380
EGF/EGFR Signaling Pathway WP437
TGF-beta Signaling Pathway WP366
VEGFA-VEGFR2 Signaling Pathway WP3888
ID signaling pathway WP53
Rett syndrome causing genes WP4312
Signaling of Hepatocyte Growth Factor Receptor WP313
Angiopoietin Like Protein 8 Regulatory Pathway WP3915
Androgen receptor signaling pathway WP138
ErbB Signaling Pathway WP673
ESC Pluripotency Pathways WP3931
Common Pathways Underlying Drug Addiction WP2636
Regulation of Wnt/B-catenin Signaling by Small Molecule Compounds WP3664
Mesodermal Commitment Pathway WP2857
Insulin Signaling WP481
Heart Development WP1591
Leptin signaling pathway WP2034
Wnt/beta-catenin Signaling Pathway in Leukemia WP3658
Integrated Breast Cancer Pathway WP1984
mRNA Processing WP411
Mitochondrial Gene Expression WP391
Serotonin Receptor 4/6/7 and NR3C Signaling WP734
Breast cancer pathway WP4262
Energy Metabolism WP1541
Adipogenesis WP236
MET in type 1 papillary renal cell carcinoma WP4205
LncRNA involvement in canonical Wnt signaling and colorectal cancer WP4258
BMP2-WNT4-FOXO1 Pathway in Human Primary Endometrial Stromal Cell Differentiation WP3876
Epithelial to mesenchymal transition in colorectal cancer WP4239
Phosphodiesterases in neuronal function WP4222
Estrogen signaling pathway WP712
Pathways Affected in Adenoid Cystic Carcinoma WP3651
IL-6 signaling pathway WP364
TGF-beta Receptor Signaling WP560
Prion disease pathway WP3995
miRNA regulation of prostate cancer signaling pathways WP3981
Association Between Physico-Chemical Features and Toxicity Associated Pathways WP3680
Human Thyroid Stimulating Hormone (TSH) signaling pathway WP2032
Differentiation of white and brown adipocyte WP2895
Aryl Hydrocarbon Receptor WP2586
MicroRNAs in cardiomyocyte hypertrophy WP1544
Follicle Stimulating Hormone (FSH) signaling pathway WP2035
Primary Focal Segmental Glomerulosclerosis FSGS WP2572
4-hydroxytamoxifen, Dexamethasone, and Retinoic Acids Regulation of p27 Expression WP3879
Chromosomal and microsatellite instability in colorectal cancer WP4216
ncRNAs involved in Wnt signaling in hepatocellular carcinoma WP4336
Hair Follicle Development: Cytodifferentiation (Part 3 of 3) WP2840
Endoderm Differentiation WP2853
Cell Differentiation - Index expanded WP2023
MAPK Cascade WP422
IL-5 Signaling Pathway WP127
NAD metabolism, sirtuins and aging WP3630

PTF1A related regulatory pathway WP4147
Serotonin Receptor 2 and ELK-SRF/GATA4 signaling WP732
AGE/RAGE pathway WP2324
Interferon type I signaling pathways WP585
DNA IR-damage and cellular response via ATR WP4016
Cardiac Hypertrophic Response WP2795
Constitutive Androstane Receptor Pathway WP2875
Mammary gland development pathway - Pregnancy and lactation (Stage 3 of 4) WP2817
White fat cell differentiation WP4149
miR-517 relationship with ARCN1 and USP1 WP3596
Transcription factor regulation in adipogenesis WP3599
Interleukin-11 Signaling Pathway WP2332
Cell Differentiation - Index WP2029
Estrogen Receptor Pathway WP2881
Notch Signaling WP268
B Cell Receptor Signaling Pathway WP23
NRF2-ARE regulation WP4357
Ras Signaling WP4223
BDNF-TrkB Signaling WP3676
Regulation of Microtubule Cytoskeleton WP2038
ERK Pathway in Huntington's Disease WP3853
Sudden Infant Death Syndrome (SIDS) Susceptibility Pathways WP706
Retinoblastoma Gene in Cancer WP2446
Wnt Signaling WP428
Wnt Signaling in Kidney Disease WP4150
Mammary gland development pathway - Embryonic development (Stage 1 of 4) WP2813
miRNAs involved in DNA damage response WP1545
MECP2 and Associated Rett Syndrome WP3584
EPO Receptor Signaling WP581
Endometrial cancer WP4155
Regulation of Actin Cytoskeleton WP51
Oncostatin M Signaling Pathway WP2374
Bladder Cancer WP2828
Nanoparticle-mediated activation of receptor signaling WP2643
Canonical and Non-Canonical TGF-B signaling WP3874
Circadian rhythm related genes WP3594
DNA Damage Response WP707
Dual hijack model of Vif in HIV infection WP3300
FTO Obesity Variant Mechanism WP3407
TGF-B Signaling in Thyroid Cells for Epithelial-Mesenchymal Transition WP3859
IL-2 Signaling Pathway WP49
Splicing factor NOVA regulated synaptic proteins WP4148
Extracellular vesicle-mediated signaling in recipient cells WP2870
Small Ligand GPCRs WP247
miRNA Regulation of DNA Damage Response WP1530
Ethanol effects on histone modifications WP3996
GABA receptor Signaling WP4159
Trans-sulfuration and one carbon metabolism WP2525
Hedgehog Signaling Pathway WP4249
Cytosine methylation WP3585
Heme Biosynthesis WP561
Methylation Pathways WP704
Pyrimidine metabolism and related diseases WP4225

Integrin-mediated Cell Adhesion WP185
Neural Crest Differentiation WP2064
Wnt Signaling Pathway and Pluripotency WP399
Development and heterogeneity of the ILC family WP3893
Initiation of transcription and translation elongation at the HIV-1 LTR WP3414
Hematopoietic Stem Cell Gene Regulation by GABP alpha/beta Complex WP3657
Hereditary leiomyomatosis and renal cell carcinoma pathway WP4206
Chemokine signaling pathway WP3929
Kit receptor signaling pathway WP304
Endothelin Pathways WP2197
Pregnane X Receptor pathway WP2876
Focal Adhesion WP306
Ethanol metabolism resulting in production of ROS by CYP2E1 WP4269
Type 2 papillary renal cell carcinoma WP4241
Exercise-induced Circadian Regulation WP410
TNF alpha Signaling Pathway WP231
Thermogenesis WP4321
IL-3 Signaling Pathway WP286
Methionine metabolism leading to Sulphur Amino Acids and related disorders WP4292
Endochondral Ossification WP474
Angiogenesis WP1539
Non-small cell lung cancer WP4255
Bone Morphogenic Protein (BMP) Signalling and Regulation WP1425
MAPK and NFkB Signalling Pathways Inhibited by Yersinia YopJ WP3849
One carbon metabolism and related pathways WP3940
Translation Factors WP107
Wnt Signaling Pathway WP363
Signaling Pathways in Glioblastoma WP2261
Hypothesized Pathways in Pathogenesis of Cardiovascular Disease WP3668
IL-7 Signaling Pathway WP205
Signal Transduction of S1P Receptor WP26
RAC1/PAK1/p38/MMP2 Pathway WP3303
G13 Signaling Pathway WP524
PDGF Pathway WP2526
Kennedy pathway from Sphingolipids WP3933
MFAP5-mediated ovarian cancer cell motility and invasiveness WP3301
Mammary gland development pathway - Puberty (Stage 2 of 4) WP2814
Osteopontin Signaling WP1434
Purine metabolism WP4224
Sterol Regulatory Element-Binding Proteins (SREBP) signalling WP1982
Vitamin D Receptor Pathway WP2877
ATM Signaling Pathway WP2516
H19 action Rb-E2F1 signaling and CDK-Beta-catenin activity WP3969
Metabolic reprogramming in colon cancer WP4290
Fatty Acid Omega Oxidation WP206
T-Cell antigen Receptor (TCR) Signaling Pathway WP69
T-Cell Receptor and Co-stimulatory Signaling WP2583
miRs in Muscle Cell Differentiation WP2012
Prostaglandin Synthesis and Regulation WP98
Corticotropin-releasing hormone signaling pathway WP2355
Notch Signaling Pathway WP61
TYROBP Causal Network WP3945
IL17 signaling pathway WP2112

ACE Inhibitor Pathway WP554
IL-9 Signaling Pathway WP22
NO/cGMP/PKG mediated Neuroprotection WP4008
Thymic Stromal LymphoPoietin (TSLP) Signaling Pathway WP2203
Genotoxicity pathway WP4286
TCA Cycle Nutrient Utilization and Invasiveness of Ovarian Cancer WP2868
Inhibition of exosome biogenesis and secretion by Manumycin A in CRPC cells WP4301
Simplified Interaction Map Between LOXL4 and Oxidative Stress Pathway WP3670
Transcription co-factors SKI and SKIL protein partners WP4533
Alpha 6 Beta 4 signaling pathway WP244
Monoamine GPCRs WP58
Oxidative Stress WP408
Structural Pathway of Interleukin 1 (IL-1) WP2637
Nucleotide Metabolism WP404
Overview of nanoparticle effects WP3287
Synaptic signaling pathways associated with autism spectrum disorder WP4539
LncRNA-mediated mechanisms of therapeutic resistance WP3672
Robo4 and VEGF Signaling Pathways Crosstalk WP3943
Somatroph axis (GH) and its relationship to dietary restriction and aging WP4186
Thyroxine (Thyroid Hormone) Production WP1981
exRNA mechanism of action and biogenesis WP2805
Hippo-Merlin Signaling Dysregulation WP4541
Copper homeostasis WP3286
Ectoderm Differentiation WP2858
Apoptosis-related network due to altered Notch3 in ovarian cancer WP2864
Nuclear Receptors Meta-Pathway WP2882
Neovascularisation processes WP4331
Senescence and Autophagy in Cancer WP615
Focal Adhesion-PI3K-Akt-mTOR-signaling pathway WP3932
Disorders of the Krebs cycle WP4236
EV release from cardiac cells and their functional effects WP3297
Hfe effect on hepcidin production WP3924
MicroRNA for Targeting Cancer Growth and Vascularization in Glioblastoma WP3593
Non-homologous end joining WP438
Pancreatic adenocarcinoma pathway WP4263
Parkinsons Disease Pathway WP2371
Globo Sphingolipid Metabolism WP1424
Methionine De Novo and Salvage Pathway WP3580
NAD+ biosynthetic pathways WP3645
Vitamin D in inflammatory diseases WP4482
IL-1 signaling pathway WP195
Pathogenic Escherichia coli infection WP2272
RANKL/RANK (Receptor activator of NFKB (ligand)) Signaling Pathway WP2018
GPCRs, Other WP117
The human immune response to tuberculosis WP4197
DNA Damage Response (only ATM dependent) WP710
Glycogen Synthesis and Degradation WP500
Caloric restriction and aging WP4191
Effects of Nitric Oxide WP1995
HIF1A and PPARG regulation of glycolysis WP2456
miRNA regulation of p53 pathway in prostate cancer WP3982
Prolactin Signaling Pathway WP2037
Calcium Regulation in the Cardiac Cell WP536

TNF related weak inducer of apoptosis (TWEAK) Signaling Pathway WP2036
Glycosylation and related congenital defects. WP4521
Physiological and Pathological Hypertrophy of the Heart WP1528
ATR Signaling WP3875
Folate-Alcohol and Cancer Pathway Hypotheses WP1589
Metabolism of Spingolipids in ER and Golgi apparatus WP4142
Vitamin A and Carotenoid Metabolism WP716
PPAR Alpha Pathway WP2878
Pathways Regulating Hippo Signaling WP4540
Integrated Cancer Pathway WP1971
T-Cell antigen Receptor (TCR) pathway during Staphylococcus aureus infection WP3863
Lung fibrosis WP3624
Liver X Receptor Pathway WP2874
Mammary gland development pathway - Involution (Stage 4 of 4) WP2815
Trans-sulfuration pathway WP2333
Vitamin D Metabolism WP1531
G1 to S cell cycle control WP45
Alzheimers Disease WP2059
Ciliary landscape WP4352
Mechanoregulation and pathology of YAP/TAZ via Hippo and non-Hippo mechanisms WP4534
SRF and miRs in Smooth Muscle Differentiation and Proliferation WP1991
Cannabinoid receptor signaling WP3869
Gastric Cancer Network 1 WP2361
PDGFR-beta pathway WP3972
One Carbon Metabolism WP241
PI3K-AKT-mTOR signaling pathway and therapeutic opportunities WP3844
Hepatitis C and Hepatocellular Carcinoma WP3646
Bile Acids synthesis and enterohepatic circulation WP4389
RalA downstream regulated genes WP2290
AMP-activated Protein Kinase (AMPK) Signaling WP1403
Factors and pathways affecting insulin-like growth factor (IGF1)-Akt signaling WP3850
Gastric Cancer Network 2 WP2363
Vitamin B12 Metabolism WP1533
Parkin-Ubiquitin Proteasomal System pathway WP2359
Synaptic Vesicle Pathway WP2267
Development of pulmonary dendritic cells and macrophage subsets WP3892
Dopamine metabolism WP2436
EDA Signalling in Hair Follicle Development WP3930
Ganglio Sphingolipid Metabolism WP1423
ncRNAs involved in STAT3 signaling in hepatocellular carcinoma WP4337
Fluoropyrimidine Activity WP1601
Cardiac Progenitor Differentiation WP2406
G Protein Signaling Pathways WP35
p38 MAPK Signaling Pathway WP400
Arrhythmogenic Right Ventricular Cardiomyopathy WP2118
DNA IR-Double Strand Breaks (DSBs) and cellular response via ATM WP3959
Photodynamic therapy-induced NF-kB survival signaling WP3617
GPCRs, Class C Metabotropic glutamate, pheromone WP501
Oxysterols derived from cholesterol WP4545
Phytochemical activity on NRF2 transcriptional activation WP3
Regulation of sister chromatid separation at the metaphase-anaphase transition WP4240
Role of Osx and miRNAs in tooth development WP3971
Zinc homeostasis WP3529

Deregulation of Rab and Rab Effector Genes in Bladder Cancer WP2291
Hedgehog Signaling Pathway WP47
Interactome of polycomb repressive complex 2 (PRC2) WP2916
Metabolic pathway of LDL, HDL and TG, including diseases WP4522
SREBF and miR33 in cholesterol and lipid homeostasis WP2011
TCA Cycle and Deficiency of Pyruvate Dehydrogenase complex (PDHc) WP2453
Spinal Cord Injury WP2431
Melatonin metabolism and effects WP3298
The effect of progerin on the involved genes in Hutchinson-Gilford Progeria Syndrome WP4320
Preimplantation Embryo WP3527
Cell Cycle WP179
Nuclear Receptors WP170
Striated Muscle Contraction Pathway WP383
Leptin Insulin Overlap WP3935
NOTCH1 regulation of human endothelial cell calcification WP3413
Platelet-mediated interactions with vascular and circulating cells WP4462
Serotonin and anxiety WP3947
Sulfation Biotransformation Reaction WP692
miR-509-3p alteration of YAP1/ECM axis WP3967
TCA Cycle (aka Krebs or citric acid cycle) WP78
Oxidative Damage WP3941
Apoptosis Modulation by HSP70 WP384
Farnesoid X Receptor Pathway WP2879
Apoptosis WP254
Viral Acute Myocarditis WP4298
Pathways in clear cell renal cell carcinoma WP4018
Pyrimidine metabolism WP4022
BMP Signaling Pathway in Eyelid Development WP3927
Hypertrophy Model WP516
Urea cycle and metabolism of amino groups WP497
Selenium Micronutrient Network WP15
Folate Metabolism WP176
Glycerophospholipid Biosynthetic Pathway WP2533
Nicotine Activity on Dopaminergic Neurons WP1602
Histone Modifications WP2369
ATM Signaling Network in Development and Disease WP3878
PI3K-Akt Signaling Pathway WP4172
Fatty Acid Biosynthesis WP357
PI3K/AKT/mTOR - VitD3 Signalling WP4141
Regulation of Apoptosis by Parathyroid Hormone-related Protein WP3872
Type II diabetes mellitus WP1584
Nonalcoholic fatty liver disease WP4396
Selenium Metabolism and Selenoproteins WP28
Amino Acid metabolism WP3925
EBV LMP1 signaling WP262
Hippo-Yap signaling pathway WP4537
Differentiation Pathway WP2848
Non-genomic actions of 1,25 dihydroxyvitamin D3 WP4341
GPCRs, Class B Secretin-like WP334
Triacylglyceride Synthesis WP325
MAPK Signaling Pathway WP382
MTHFR deficiency WP4288
Peptide GPCRs WP24

Human Complement System WP2806
Glucuronidation WP698
Canonical and Non-canonical Notch signaling WP3845
Intraflagellar transport proteins binding to dynein WP4532
Photodynamic therapy-induced unfolded protein response WP3613
Sphingolipid pathway WP1422
IL-4 Signaling Pathway WP395
Lipid Metabolism Pathway WP3965
Statin Pathway WP430
Mitochondrial complex I assembly model OXPHOS system WP4324
Dopaminergic Neurogenesis WP2855
Matrix Metalloproteinases WP129
Oligodendrocyte Specification and differentiation(including remyelination), leading to Myelin Components for CN
Ebola Virus Pathway on Host WP4217
Toll-like Receptor Signaling WP3858
Ovarian Infertility Genes WP34
Nuclear Receptors in Lipid Metabolism and Toxicity WP299
Resistin as a regulator of inflammation WP4481
Fatty Acid Beta Oxidation WP143
Target Of Rapamycin (TOR) Signaling WP1471
Apoptosis Modulation and Signaling WP1772
Photodynamic therapy-induced HIF-1 survival signaling WP3614
Type II interferon signaling (IFNG) WP619
Sleep regulation WP3591
PPAR signaling pathway WP3942
Ferroptosis WP4313
Microglia Pathogen Phagocytosis Pathway WP3937
Nucleotide-binding Oligomerization Domain (NOD) pathway WP1433
DNA Replication WP466
Fas Ligand (FasL) pathway and Stress induction of Heat Shock Proteins (HSP) regulation WP314
Eukaryotic Transcription Initiation WP405
Toll-like Receptor Signaling Pathway WP75
Glycolysis and Gluconeogenesis WP534
Aryl Hydrocarbon Receptor Pathway WP2873
Tryptophan metabolism WP465
GPCRs, Class A Rhodopsin-like WP455
Photodynamic therapy-induced AP-1 survival signaling. WP3611
Regulation of toll-like receptor signaling pathway WP1449
Hematopoietic Stem Cell Differentiation WP2849
RIG-I-like Receptor Signaling WP3865
Oxidation by Cytochrome P450 WP43
Prader-Willi and Angelman Syndrome WP3998
Metapathway biotransformation Phase I and II WP702
Proteasome Degradation WP183
Myometrial Relaxation and Contraction Pathways WP289
Electron Transport Chain (OXPHOS system in mitochondria) WP111
Genes related to primary cilium development (based on CRISPR) WP4536
Cytoplasmic Ribosomal Proteins WP477
NRF2 pathway WP2884

Overlap	P.value	Adjusted.P.value	Old.P.value	Old.Adjusted
22/144	4,6682E-06	0,00220339	0	0
23/162	1,00262E-05	0,002366175	0	0
20/132	1,41576E-05	0,002227457	0	0
28/236	3,59086E-05	0,004237214	0	0
6/16	9,5257E-05	0,008992264	0	0
10/48	0,000139497	0,010973734	0	0
8/34	0,000267181	0,018015611	0	0
17/132	0,000454164	0,026795695	0	0
13/90	0,000696619	0,036533815	0	0
13/91	0,000775073	0,036583433	0	0
15/116	0,000920966	0,039517803	0	0
8/42	0,001204829	0,047389946	0	0
5/17	0,001331814	0,048355105	0	0
17/147	0,001541982	0,051986834	0	0
18/160	0,001561127	0,04912346	0	0
8/44	0,001651626	0,048722977	0	0
11/76	0,001725691	0,047913298	0	0
6/26	0,001758	0,046098659	0	0
17/151	0,002062706	0,051241971	0	0
15/126	0,002136321	0,050417183	0	0
5/19	0,002296337	0,05161291	0	0
5/19	0,002296337	0,049266868	0	0
17/154	0,002543579	0,052198665	0	0
8/47	0,002556811	0,050283941	0	0
15/130	0,002901519	0,054780687	0	0
9/59	0,003074343	0,055811145	0	0
12/94	0,003219741	0,056285837	0	0
4/13	0,003463707	0,058388211	0	0
17/159	0,003551056	0,057796504	0	0
8/53	0,00549151	0,086399752	0	0
5/23	0,005598899	0,085247749	0	0
9/65	0,00594332	0,087663973	0	0
7/43	0,006035445	0,086325154	0	0
8/54	0,006161858	0,085541091	0	0
6/33	0,006219281	0,08387145	0	0
6/33	0,006219281	0,081541688	0	0
9/66	0,006574692	0,083871746	0	0
9/66	0,006574692	0,081664595	0	0
5/25	0,008117773	0,098245873	0	0
7/46	0,008772983	0,103521202	0	0
10/84	0,011095096	0,127728911	0	0
5/27	0,011329548	0,127322538	0	0
9/72	0,011504736	0,126284548	0	0
4/18	0,012068057	0,129457344	0	0
9/73	0,012540103	0,131531745	0	0
10/86	0,012982942	0,133216271	0	0
10/87	0,014012702	0,140723309	0	0
14/141	0,014297494	0,14059202	0	0
4/19	0,014673856	0,14134816	0	0
5/29	0,01531179	0,144543298	0	0
6/40	0,015891236	0,147071831	0	0
3/11	0,016549986	0,150222952	0	0

3/11	0,016549986	0,147388557	0	0
4/20	0,01760904	0,153916053	0	0
8/66	0,019843043	0,170289391	0	0
7/54	0,020379948	0,171773844	0	0
9/80	0,021820871	0,180692122	0	0
7/55	0,022346869	0,181857278	0	0
5/32	0,022876958	0,183015664	0	0
5/32	0,022876958	0,179965403	0	0
5/32	0,022876958	0,17701515	0	0
2/5	0,02396506	0,182443679	0	0
4/22	0,024509309	0,183625302	0	0
6/44	0,024584124	0,181307912	0	0
3/13	0,026564546	0,192899474	0	0
3/13	0,026564546	0,189976754	0	0
6/45	0,02717002	0,191406708	0	0
10/97	0,027920253	0,193799401	0	0
4/23	0,028490455	0,194891229	0	0
16/184	0,028938778	0,195130047	0	0
5/34	0,029062326	0,193203068	0	0
6/46	0,029929579	0,196205019	0	0
3/14	0,03254043	0,210398396	0	0
14/158	0,034247457	0,218443241	0	0
9/87	0,035222731	0,221668387	0	0
11/115	0,035338359	0,219469811	0	0
5/36	0,036210659	0,221966635	0	0
3/15	0,039153343	0,236927924	0	0
3/15	0,039153343	0,233928836	0	0
7/62	0,03985778	0,235160905	0	0
4/26	0,042615227	0,248325769	0	0
7/63	0,042927296	0,247093704	0	0
13/150	0,046765208	0,265941906	0	0
7/65	0,049514039	0,278221741	0	0
5/40	0,053499587	0,297080059	0	0
4/28	0,053862291	0,295616293	0	0
3/17	0,054236191	0,29424692	0	0
16/201	0,056908265	0,305235242	0	0
7/68	0,060534578	0,321037315	0	0
2/8	0,060539704	0,317497116	0	0
2/8	0,060539704	0,314008137	0	0
3/18	0,062669827	0,321523462	0	0
5/42	0,063661977	0,323101648	0	0
5/42	0,063661977	0,319664396	0	0
4/30	0,066556032	0,330678392	0	0
3/19	0,071669081	0,352372983	0	0
7/71	0,072944213	0,354945038	0	0
4/31	0,073433514	0,353679783	0	0
4/31	0,073433514	0,35010726	0	0
4/31	0,073433514	0,346606187	0	0
5/44	0,074831689	0,349708488	0	0
2/9	0,07523604	0,348151085	0	0
2/9	0,07523604	0,344770978	0	0
2/9	0,07523604	0,341455872	0	0
2/9	0,07523604	0,338203911	0	0

9/101	0,076834299	0,342130088	0	0
9/101	0,076834299	0,338932611	0	0
9/102	0,080621837	0,352347289	0	0
4/32	0,080655885	0,349262181	0	0
4/32	0,080655885	0,346087071	0	0
3/20	0,081209097	0,345321567	0	0
3/20	0,081209097	0,342238339	0	0
13/164	0,082239379	0,34351316	0	0
6/59	0,082748589	0,342608195	0	0
4/33	0,088215109	0,362065493	0	0
4/33	0,088215109	0,358944238	0	0
15/198	0,088515047	0,357086341	0	0
2/10	0,090917569	0,363670276	0	0
4/34	0,096102152	0,381178285	0	0
5/48	0,10011571	0,39378846	0	0
8/92	0,102225566	0,398764192	0	0
9/108	0,105672448	0,40883111	0	0
5/49	0,107028285	0,410710166	0	0
2/11	0,107443713	0,408979295	0	0
6/64	0,111599028	0,421397928	0	0
3/24	0,1242241	0,465347421	0	0
6/66	0,124400603	0,46233925	0	0
2/12	0,124685754	0,459778716	0	0
2/12	0,124685754	0,45621454	0	0
5/52	0,129104912	0,468750144	0	0
5/52	0,129104912	0,465171898	0	0
5/52	0,129104912	0,461647869	0	0
7/82	0,130200832	0,462066111	0	0
3/25	0,136044545	0,479201679	0	0
3/25	0,136044545	0,475652037	0	0
3/25	0,136044545	0,472154596	0	0
6/68	0,13788388	0,475045194	0	0
4/39	0,140080408	0,479115599	0	0
4/39	0,140080408	0,475668724	0	0
2/13	0,142525984	0,480516174	0	0
2/13	0,142525984	0,477108258	0	0
2/13	0,142525984	0,473748341	0	0
2/13	0,142525984	0,470435415	0	0
2/13	0,142525984	0,467168503	0	0
6/69	0,144871287	0,471581018	0	0
13/182	0,14780046	0,477820666	0	0
4/40	0,149700503	0,480671002	0	0
2/14	0,160856918	0,513003145	0	0
4/42	0,169660024	0,537446518	0	0
2/15	0,179580553	0,565080139	0	0
7/90	0,182500436	0,570464939	0	0
3/29	0,18668155	0,579695338	0	0
3/29	0,18668155	0,57590648	0	0
4/45	0,201187717	0,616627288	0	0
7/93	0,20407947	0,621454904	0	0
5/61	0,205806685	0,622697149	0	0
5/61	0,205806685	0,618730925	0	0
3/31	0,213592618	0,638074151	0	0

2/17	0,217857207	0,646720765	0	0
2/17	0,217857207	0,64267876	0	0
4/47	0,2230987	0,654053331	0	0
4/47	0,2230987	0,650015965	0	0
5/63	0,224585398	0,650333178	0	0
1/5	0,232733649	0,669818795	0	0
2/18	0,237255632	0,6786949	0	0
2/18	0,237255632	0,674606376	0	0
2/18	0,237255632	0,670566817	0	0
3/33	0,241257655	0,677819127	0	0
3/33	0,241257655	0,673808363	0	0
3/33	0,241257655	0,669844784	0	0
4/49	0,245590145	0,677886248	0	0
2/19	0,256736405	0,704532459	0	0
2/19	0,256736405	0,700460017	0	0
4/50	0,257018171	0,697198716	0	0
1/6	0,272334438	0,734524884	0	0
1/6	0,272334438	0,730351447	0	0
1/6	0,272334438	0,726225168	0	0
1/6	0,272334438	0,722145251	0	0
1/6	0,272334438	0,71811092	0	0
8/120	0,279366639	0,732561408	0	0
4/52	0,280169541	0,730607863	0	0
9/138	0,283191823	0,73443154	0	0
4/53	0,29186584	0,752790581	0	0
19/319	0,292119716	0,749350575	0	0
2/21	0,295710622	0,754461695	0	0
7/105	0,298389432	0,75720329	0	0
18/303	0,302630855	0,763859698	0	0
1/7	0,309893194	0,778029721	0	0
1/7	0,309893194	0,773913162	0	0
1/7	0,309893194	0,769839935	0	0
1/7	0,309893194	0,765809359	0	0
1/7	0,309893194	0,761820769	0	0
6/89	0,310817292	0,760133482	0	0
3/38	0,312340461	0,759921122	0	0
2/22	0,315101353	0,762706864	0	0
2/22	0,315101353	0,758815503	0	0
2/22	0,315101353	0,754963647	0	0
2/22	0,315101353	0,7511507	0	0
4/55	0,315434093	0,748165286	0	0
4/55	0,315434093	0,744424459	0	0
4/55	0,315434093	0,740720855	0	0
6/91	0,329187318	0,769190168	0	0
2/23	0,334368124	0,777447067	0	0
7/110	0,340216351	0,787167244	0	0
3/40	0,341046404	0,78523855	0	0
1/8	0,345515118	0,791665708	0	0
1/8	0,345515118	0,787841236	0	0
1/8	0,345515118	0,784053538	0	0
2/24	0,353472123	0,798271972	0	0
5/76	0,355341865	0,798673144	0	0
9/149	0,362657461	0,81125271	0	0

3/42	0,369659561	0,823015627	0	0
2/25	0,372378865	0,825177579	0	0
2/25	0,372378865	0,821321609	0	0
1/9	0,379299997	0,832695808	0	0
1/9	0,379299997	0,828840734	0	0
1/9	0,379299997	0,825021192	0	0
3/43	0,383886151	0,831166346	0	0
2/26	0,39105785	0,842827878	0	0
6/98	0,394232438	0,845807776	0	0
3/44	0,398037404	0,850107035	0	0
4/62	0,398494592	0,847249764	0	0
4/63	0,410285043	0,86840601	0	0
1/10	0,411342481	0,86675737	0	0
1/10	0,411342481	0,862905115	0	0
1/10	0,411342481	0,859086951	0	0
1/10	0,411342481	0,855302427	0	0
4/64	0,422026674	0,873669255	0	0
5/83	0,427755717	0,881662439	0	0
12/216	0,438607639	0,900099156	0	0
3/47	0,439891843	0,898826624	0	0
1/11	0,441732347	0,898696844	0	0
2/29	0,445476512	0,902424523	0	0
2/29	0,445476512	0,898568008	0	0
2/29	0,445476512	0,894744314	0	0
2/30	0,463008482	0,926016964	0	0
2/30	0,463008482	0,92210972	0	0
3/49	0,467165176	0,926478836	0	0
1/12	0,470554753	0,929296416	0	0
1/12	0,470554753	0,925424348	0	0
4/69	0,479703714	0,939502709	0	0
2/31	0,480209569	0,936607093	0	0
2/31	0,480209569	0,932752742	0	0
3/50	0,480578809	0,929644253	0	0
4/70	0,49097898	0,945886035	0	0
3/51	0,493830654	0,947512474	0	0
1/13	0,497890471	0,951434422	0	0
1/13	0,497890471	0,947597993	0	0
1/13	0,497890471	0,943792379	0	0
1/13	0,497890471	0,940017209	0	0
1/13	0,497890471	0,936272121	0	0
2/33	0,513571053	0,961926734	0	0
3/53	0,5198147	0,969772878	0	0
5/93	0,527706009	0,98061904	0	0
2/34	0,529712675	0,980487775	0	0
4/74	0,53499366	0,986394561	0	0
3/55	0,545056593	1	0	0
2/35	0,545485494	0,997942453	0	0
1/15	0,548404355	0,999408708	0	0
1/15	0,548404355	0,995564829	0	0
1/15	0,548404355	0,991750404	0	0
1/15	0,548404355	0,987965097	0	0
1/15	0,548404355	0,984208576	0	0
2/36	0,560884561	1	0	0

1/16	0,571724118	1	0	0
1/16	0,571724118	1	0	0
1/16	0,571724118	1	0	0
1/16	0,571724118	1	0	0
1/16	0,571724118	1	0	0
1/16	0,571724118	0,999458459	0	0
6/118	0,57355001	0,99895057	0	0
2/37	0,57590642	0,999367022	0	0
2/37	0,57590642	0,995706337	0	0
3/58	0,581423931	1	0	0
6/120	0,590107827	1	0	0
2/38	0,590548963	1	0	0
2/38	0,590548963	1	0	0
1/17	0,593840785	1	0	0
1/17	0,593840785	1	0	0
1/17	0,593840785	1	0	0
1/17	0,593840785	0,997483453	0	0
1/17	0,593840785	0,993946278	0	0
1/17	0,593840785	0,9904341	0	0
1/18	0,614816365	1	0	0
2/40	0,618693656	1	0	0
1/19	0,63470968	1	0	0
1/19	0,63470968	1	0	0
4/84	0,635790137	1	0	0
4/84	0,635790137	1	0	0
4/85	0,645053048	1	0	0
4/85	0,645053048	1	0	0
1/20	0,653576518	1	0	0
1/20	0,653576518	1	0	0
1/20	0,653576518	1	0	0
4/86	0,654159206	1	0	0
3/66	0,668943677	1	0	0
1/21	0,671469795	1	0	0
1/21	0,671469795	1	0	0
3/67	0,678884566	1	0	0
2/45	0,682476808	1	0	0
16/340	0,683733971	1	0	0
1/22	0,688439703	1	0	0
1/22	0,688439703	1	0	0
1/22	0,688439703	1	0	0
1/22	0,688439703	1	0	0
7/155	0,693804949	1	0	0
2/46	0,694132378	1	0	0
4/91	0,697305711	1	0	0
1/23	0,704533848	1	0	0
1/23	0,704533848	1	0	0
2/48	0,716381176	1	0	0
3/71	0,716425533	1	0	0
1/24	0,719797384	1	0	0
1/24	0,719797384	1	0	0
11/246	0,729178076	1	0	0
1/25	0,734273137	1	0	0
3/74	0,742282769	1	0	0

4/97	0,743803628	1	0	0
1/26	0,74800173	1	0	0
1/27	0,761021692	1	0	0
1/27	0,761021692	1	0	0
1/27	0,761021692	1	0	0
1/27	0,761021692	1	0	0
2/54	0,775076347	1	0	0
1/29	0,785080013	1	0	0
1/29	0,785080013	1	0	0
2/56	0,792140489	1	0	0
1/30	0,796185909	1	0	0
1/30	0,796185909	1	0	0
1/30	0,796185909	1	0	0
5/129	0,801552567	1	0	0
1/31	0,806718435	1	0	0
1/32	0,816707165	1	0	0
1/33	0,826180149	1	0	0
1/33	0,826180149	1	0	0
1/34	0,835163993	1	0	0
1/36	0,851763893	1	0	0
3/91	0,854971748	1	0	0
1/37	0,859426581	1	0	0
1/37	0,859426581	1	0	0
1/38	0,866693525	1	0	0
2/67	0,8669476	1	0	0
1/40	0,880120803	1	0	0
1/40	0,880120803	1	0	0
1/41	0,886318873	1	0	0
1/42	0,892196776	1	0	0
1/42	0,892196776	1	0	0
1/43	0,897771034	1	0	0
3/103	0,906043438	1	0	0
1/45	0,908070493	1	0	0
1/46	0,912824654	1	0	0
1/46	0,912824654	1	0	0
9/257	0,918611093	1	0	0
1/50	0,929508438	1	0	0
4/139	0,932548414	1	0	0
1/55	0,945950624	1	0	0
1/60	0,958560365	1	0	0
1/61	0,960704971	1	0	0
1/61	0,960704971	1	0	0
5/183	0,96254397	1	0	0
1/62	0,962738684	1	0	0
4/156	0,963126998	1	0	0
2/103	0,972091904	1	0	0
2/103	0,972091904	1	0	0
1/89	0,991134165	1	0	0
2/146	0,996171814	1	0	0

Odds.Ratio	Combined.Score
2,960809647	36,34315998
2,75145947	31,67015745
2,936340146	32,78500561
2,299303639	23,53230116
7,26744186	67,28874821
4,0374677	35,84249911
4,55996352	37,51748944
2,495889124	19,2109871
2,799310939	20,34895096
2,76854928	19,82988283
2,506014435	17,51726068
3,69139904	24,81143421
5,6999544	37,74061275
2,24120656	14,51110974
2,180232558	14,0894201
3,523608175	22,57221572
2,80497756	17,84562579
4,472271914	28,37020862
2,18183685	13,49190383
2,3071244	14,18574646
5,0999592	30,98959607
5,0999592	30,98959607
2,139333535	12,78077033
3,298697015	19,68990491
2,236135957	13,0646707
2,956247536	17,10089914
2,474022761	14,19706693
5,963029219	33,78303964
2,072058895	11,68746922
2,925259617	15,22466601
4,213009774	21,84523656
2,683363148	13,75354385
3,154858482	16,12166023
2,871088142	14,61204962
3,523608175	17,90028517
3,523608175	17,90028517
2,642706131	13,27834976
2,642706131	13,27834976
3,875968992	18,65774953
2,949106842	13,96720111
2,3071244	10,38494846
3,588860178	16,07931778
2,42248062	10,81636742
4,306632214	19,02322652
2,389295954	10,46230538
2,253470344	9,789343256
2,227568386	9,506796418
1,924239925	8,173538191
4,07996736	17,22434882
3,34135258	13,963954
2,906976744	12,04066138
5,285412262	21,67743134

5,285412262	21,67743134
3,875968992	15,65636772
2,349072117	9,208131988
2,512202125	9,780514918
2,180232558	8,339146213
2,466525722	9,375434615
3,028100775	11,43902942
3,028100775	11,43902942
3,028100775	11,43902942
7,751937984	28,92370822
3,523608175	13,0680136
2,642706131	9,792955667
4,472271914	16,22619769
4,472271914	16,22619769
2,583979328	9,316902113
1,997922161	7,149370558
3,370407819	11,99253845
1,68520391	5,969957498
2,8499772	10,08411019
2,527805865	8,869838268
4,15282392	14,22455135
1,717201452	5,794083186
2,004811548	6,708227023
1,853724301	6,196604081
2,691645134	8,931959959
3,875968992	12,55918396
3,875968992	12,55918396
2,188047012	7,05084507
2,981514609	9,408299464
2,153316107	6,779171796
1,679586563	5,143928297
2,087060227	6,27265751
2,42248062	7,093220319
2,76854928	8,087831292
3,41997264	9,967191743
1,542674226	4,42178979
1,99498404	5,595013601
4,84496124	13,58747994
4,84496124	13,58747994
3,22997416	8,946625228
2,3071244	6,354207732
2,3071244	6,354207732
2,583979328	7,001837457
3,05997552	8,065164774
1,91068894	5,00229892
2,500625156	6,530069643
2,500625156	6,530069643
2,500625156	6,530069643
2,202255109	5,709376829
4,306632214	11,14179549
4,306632214	11,14179549
4,306632214	11,14179549
4,306632214	11,14179549

1,726916878	4,431448533
1,726916878	4,431448533
1,70998632	4,305721154
2,42248062	6,098748804
2,42248062	6,098748804
2,906976744	7,298627913
2,906976744	7,298627913
1,536207223	3,83763156
1,970831691	4,911210702
2,349072117	5,703493121
2,349072117	5,703493121
1,468170073	3,559699795
3,875968992	9,293806274
2,27998176	5,340500606
2,01873385	4,645971938
1,68520391	3,843231336
1,61498708	3,629539863
1,9775352	4,419123035
3,523608175	7,860423421
1,816860465	3,984089648
2,42248062	5,052490523
1,761804087	3,672037086
3,22997416	6,724672735
3,22997416	6,724672735
1,863446631	3,814717371
1,863446631	3,814717371
1,863446631	3,814717371
1,654377009	3,372740619
2,325581395	4,639006775
2,325581395	4,639006775
2,325581395	4,639006775
1,70998632	3,3880701
1,987676406	3,906854855
1,987676406	3,906854855
2,981514609	5,80867905
2,981514609	5,80867905
2,981514609	5,80867905
2,981514609	5,80867905
2,981514609	5,80867905
1,68520391	3,25566162
1,38427464	2,646583827
1,937984496	3,68046246
2,76854928	5,058804027
1,84569952	3,274194731
2,583979328	4,437032068
1,507321275	2,563957584
2,004811548	3,364777581
2,004811548	3,364777581
1,722652885	2,762303
1,458698008	2,318229682
1,588511882	2,511148135
1,588511882	2,511148135
1,875468867	2,895132655

2,27998176	3,474499418
2,27998176	3,474499418
1,649348507	2,474255328
1,649348507	2,474255328
1,538082933	2,297125709
3,875968992	5,650622543
2,153316107	3,097797377
2,153316107	3,097797377
2,153316107	3,097797377
1,761804087	2,505091275
1,761804087	2,505091275
1,761804087	2,505091275
1,58202816	2,221311833
2,03998368	2,773776792
2,03998368	2,773776792
1,550387597	2,106369756
3,22997416	4,201306255
3,22997416	4,201306255
3,22997416	4,201306255
3,22997416	4,201306255
3,22997416	4,201306255
1,291989664	1,647584292
1,490757305	1,896780496
1,263902932	1,594578858
1,462629808	1,801171619
1,154285437	1,420453933
1,84569952	2,248752181
1,291989664	1,562475228
1,151277918	1,376055166
2,76854928	3,243431827
2,76854928	3,243431827
2,76854928	3,243431827
2,76854928	3,243431827
2,76854928	3,243431827
1,306506402	1,526718088
1,52998776	1,780387798
1,761804087	2,034638719
1,761804087	2,034638719
1,761804087	2,034638719
1,761804087	2,034638719
1,40944327	1,62622342
1,40944327	1,62622342
1,40944327	1,62622342
1,277791975	1,419790872
1,68520391	1,846162327
1,233262861	1,32967138
1,453488372	1,563570827
2,42248062	2,574415889
2,42248062	2,574415889
2,42248062	2,574415889
1,61498708	1,679506874
1,2749898	1,319200012
1,170594662	1,187330095

1,38427464	1,377592472
1,550387597	1,531540293
1,550387597	1,531540293
2,153316107	2,087484578
2,153316107	2,087484578
2,153316107	2,087484578
1,352082207	1,294496013
1,490757305	1,399671698
1,18652112	1,104431181
1,321353066	1,217242729
1,250312578	1,150364279
1,230466347	1,096226325
1,937984496	1,721568072
1,937984496	1,721568072
1,937984496	1,721568072
1,937984496	1,721568072
1,21124031	1,044920976
1,16746054	0,991410994
1,076658053	0,887327762
1,237011381	1,015866394
1,761804087	1,43948402
1,336541032	1,080741454
1,336541032	1,080741454
1,336541032	1,080741454
1,291989664	0,994844838
1,291989664	0,994844838
1,18652112	0,903028463
1,61498708	1,217446632
1,61498708	1,217446632
1,123469273	0,825285506
1,250312578	0,917145122
1,250312578	0,917145122
1,162790698	0,852051221
1,107419712	0,787767401
1,13999088	0,804334959
1,490757305	1,03961712
1,490757305	1,03961712
1,490757305	1,03961712
1,490757305	1,03961712
1,490757305	1,03961712
1,174536058	0,782671939
1,096972356	0,71773023
1,041927148	0,666016454
1,13999088	0,724373623
1,047559187	0,655248672
1,057082452	0,641507029
1,107419712	0,671183906
1,291989664	0,776152959
1,291989664	0,776152959
1,291989664	0,776152959
1,291989664	0,776152959
1,291989664	0,776152959
1,076658053	0,622566934

1,21124031	0,6772029
1,21124031	0,6772029
1,21124031	0,6772029
1,21124031	0,6772029
1,21124031	0,6772029
1,21124031	0,6772029
0,985415845	0,547802666
1,047559187	0,578053737
1,047559187	0,578053737
1,002405774	0,543579722
0,968992248	0,511094962
1,01999184	0,537232486
1,01999184	0,537232486
1,13999088	0,594099447
1,13999088	0,594099447
1,13999088	0,594099447
1,13999088	0,594099447
1,13999088	0,594099447
1,13999088	0,594099447
1,076658053	0,523720552
0,968992248	0,465256812
1,01999184	0,463675624
1,01999184	0,463675624
0,92284976	0,417946422
0,92284976	0,417946422
0,911992704	0,399838323
0,911992704	0,399838323
0,968992248	0,412108201
0,968992248	0,412108201
0,968992248	0,412108201
0,901388138	0,382553202
0,880902044	0,354171434
0,92284976	0,367558366
0,92284976	0,367558366
0,867754252	0,336084841
0,861326443	0,329049728
0,911992704	0,346727193
0,880902044	0,328864994
0,880902044	0,328864994
0,880902044	0,328864994
0,880902044	0,328864994
0,875218805	0,319948847
0,842601955	0,30762773
0,851861317	0,307122715
0,842601955	0,295095132
0,842601955	0,295095132
0,80749354	0,269333725
0,818866689	0,273076457
0,80749354	0,265492182
0,80749354	0,265492182
0,866578433	0,273697794
0,775193798	0,239437363
0,78566939	0,234149134

0,799168864	0,236536578
0,745378652	0,216420683
0,717772036	0,196018818
0,717772036	0,196018818
0,717772036	0,196018818
0,717772036	0,196018818
0,717772036	0,182883823
0,668270516	0,161701175
0,668270516	0,161701175
0,69213732	0,161279428
0,645994832	0,1472368
0,645994832	0,1472368
0,645994832	0,1472368
0,751156781	0,166159428
0,625156289	0,134271427
0,605620155	0,122622744
0,587268029	0,112134385
0,587268029	0,112134385
0,56999544	0,102671668
0,538329027	0,086372691
0,638895988	0,100106602
0,523779594	0,079347307
0,523779594	0,079347307
0,50999592	0,072965042
0,578502835	0,08259675
0,484496124	0,061868268
0,484496124	0,061868268
0,472679145	0,057042206
0,46142488	0,052634076
0,46142488	0,052634076
0,450694069	0,048602946
0,564461504	0,055694304
0,430663221	0,041530262
0,421300977	0,038427482
0,421300977	0,038427482
0,678671613	0,057614083
0,387596899	0,028333098
0,55769338	0,038946077
0,352360817	0,019578896
0,322997416	0,013670136
0,317702376	0,012736027
0,317702376	0,012736027
0,529503961	0,020214095
0,312578145	0,011869611
0,496919102	0,01866925
0,376307669	0,010651361
0,376307669	0,010651361
0,217751067	0,001939154
0,265477328	0,001018247

Genes
MEF2A;SHC4;MAP3K2;MAP2K1;SHC2;SORT1;PTPN11;GRIN2B;PPP2CA;MAPK10;MARCKS;RAP1A;CREB1
MEF2A;MAP3K2;MAP2K1;RALA;ROCK1;SH3KBP1;GAB1;PTPN11;FOXO1;EPS8;ELK4;ITCH;RAP1A;CREB1;
MEF2A;KLF10;MAP2K1;CREBBP;SMURF2;ROCK1;RBX1;PIAS1;SMAD7;TGFBR3;ITCH;RBL1;SP1;SIN3A;CD
HDAC5;NRP2;SHC2;ROCK1;FOXO1;AMOT;CTGF;RAP1A;GPC1;PTK2B;MAPK1;EIF4E;PRKG1;MAP2K1;MM
ELK4;RBL1;MYOD1;ID2;PAX5;PAX2
HDAC5;EIF2B2;TBL1XR1;GABRA3;HIVEP2;SMARCA1;SMC1A;GRIN2B;CRK;SCN1A
MAP2K1;RAP1A;GAB1;PTK2B;MAPK1;PTPN11;RAF1;CRK
MAP3K2;PRKAB2;MAP2K1;SHC2;IRS4;DIO2;FOXO1;MAPK10;RPS6KA6;RXRA;SESN3;MAPK1;MAP3K9;RA
NCOA2;CREBBP;ROCK1;FOXO1;PIAS1;KAT2B;CREB1;SIN3A;SP1;CARM1;ZNF318;CTNNB1;RAD9A
SHC4;MAP2K1;SHC2;GAB1;FOXO1;EREG;MAPK10;NRG3;ERBB4;MAPK1;RAF1;SOS2;CRK
MAP2K1;FZD3;BMPR2;FZD5;GAB1;SMAD9;PTPN11;SMAD7;LRP6;APC;MAPK1;CTNNB1;RAF1;FGFR1;BMP
GRM5;MAP2K1;RAP1A;CREB1;CAMK4;MAPK1;RAF1;CALM1
APC;CSNK1A1;TNKS;CTNNB1;DKK3
EOMES;ZNF462;BMPR2;FZD5;GATA6;ARID5B;INHBA;PIAS1;ELK4;ADAM19;PPP2CA;C1QBP;CCDC6;WDFY
MAP3K2;MAP2K1;SHC2;SNAP23;IRS4;GAB1;PTPN11;FOXO1;CYTH3;MAPK10;RPS6KA6;MYO1C;MAPK1;M
BMPR2;GATA6;NFATC3;MAPK1;CTNNB1;PTPN11;TBX5;BMPR1A
MAP2K1;CREB1;ROCK1;SP1;PDE3B;MAPK1;PTPN11;RAF1;EIF4E;ESR1;FOXO1
APC;CSNK1A1;SALL4;ZBTB16;CTNNB1;LRP6
RALA;USP15;BMPR2;BACH1;ESR1;FOXO1;PIAS1;SMAD7;HIPK2;RAP1A;CREB1;SP1;DAG1;E2F1;MAPK1;C
SRPK2;SF3B3;CELF1;SRSF1;HNRNPU;HNRNPR;SMC1A;NXF1;HNRNPK;PABPN1;TRA2B;SNRPD3;HNRNP
CREB1;SP1;CAMK4;TFAM;PPARGC1B
ELK4;MAP2K1;CREB1;RAP1A;MAPK1
SHC4;MAP2K1;SHC2;FZD3;FZD5;CSNK1A1;ESR1;LRP6;HEYL;APC;SP1;E2F1;MAPK1;CTNNB1;RAF1;SOS2
MEF2A;PRKAB2;RXRA;CREB1;CAMK4;TFAM;FOXO1;PPARGC1B
MEF2A;NCOA2;CELF1;IRS4;EBF1;HIF1A;FOXO1;ZMPSTE24;CREB1;RBL1;RXRA;SP1;NRIP1;E2F1;CTNNB
MAP2K1;RAP1A;GAB1;MAPK1;PTPN11;STRN;RAF1;SOS2;CRK
CCND3;SFRP1;FZD3;HNRNPK;FZD5;APC;CSNK1A1;RUVBL1;HNRNPU;CTNNB1;SENP2;LRP6
SFRP1;SMAD9;CTNNB1;FOXO1
MAP2K1;FZD3;NRP2;FZD5;MMP2;PROX1;CLDN1;HIF1A;LRP6;OCLN;CDH2;ID2;CLDN18;MAPK1;CTNNB1;F
ADCY9;CREB1;PDE1B;PDE3B;PPP1R1B;PDE5A;PDE7B;GRIN2B
MAP2K1;CREB1;SP1;MAPK1;ESR1
CREBBP;KANSL1;NSD1;ARID5B;SMC1A;IL17RD;RAF1;DTX4;FOXP2
CRP;CREBBP;MAP2K1;GAB1;MAPK1;PTPN11;IL6R
TGFBR3;CREBBP;SMAD9;CTNNB1;INHBA;ZNF423;RUNX3;SMAD7
RXRA;CREB1;EBF1;MAPK1;PAX5;FGFR1
CREBBP;MAP2K1;MAPK1;CTNNB1;RAF1;FOXO1
PPP2CA;MAP2K1;FZD3;FZD5;APC;ERBB4;MAPK1;CTNNB1;RAF1
GNA13;CCND3;MAP2K1;CREB1;RAP1A;E2F1;MAPK1;RAF1;TSHR
PRDM16;SMAD9;ZNF423;HOXC8;PPARGC1B
MAP2K1;CDC37;NRIP1;E2F1;MAPK1;RAF1;ESR1
HDAC5;RCAN1;MAP2K1;ROCK1;MAPK1;CTNNB1;RAF1;CALM1;PRKG1;LRP6
CREB1;MAPK1;RAF1;FOXO1;APPL1
CDH2;MME;DAG1;CTNNB1;PARVA;ACTN4;CLDN1;PAX2;LRP6
MAP2K1;MAPK1;RAF1;EIF4E
MAPK10;MAP2K1;RALA;APC;CSNK1A1;MAPK1;CTNNB1;RAF1;APPL1
CCND3;SFRP1;FZD3;FZD5;APC;CSNK1A1;CTNNB1;SENP2;LRP6;MTDH
SFRP1;BCL11B;KLK13;SOSTDC1;CTNNB1;RUNX3;GTPBP4;CTGF;DKK3;BMPR1A
EOMES;ZNF462;GATA6;FOXO1;CDC73;PIAS1;ELK4;SFRP1;APC;SP4;C1QBP;CTNNB1;WDFY2;BMPR1A
MEF2A;HDAC5;MYOD1;ID2
MAP3K2;MAPK10;MAP2K1;MAPK1;RAF1
LYN;SPRED1;MAP2K1;MAPK1;PTPN11;RAF1
TFAM;HIF1A;FOXO1

KAT2B;CTNNB1;PROX1
ELK4;MAP2K1;MAPK1;RAF1
MAP2K1;ROCK1;SP1;MMP2;MAPK1;RAF1;HIF1A;FOXO1
CREB1;RAP1A;PDCD4;PTPN11;CRK;EIF4E;PIAS1
XRCC5;SP1;RFWD3;E2F1;USP1;CDK1;CLSPN;SMC1A;RAD9A
MEF2A;HDAC5;MAP2K1;MAPK1;RAF1;CALM1;PRKG1
NCOA2;RXRA;SP1;SMC1A;FOXO1
TTC9;ERBB4;TPM3;EIF4E;ESR1
CREB1;MECOM;EBF1;ZNF423;FOXO1
ID2;USP1
RXRA;CREB1;NRIP1;FOXO1
MAP2K1;CREB1;MAPK1;PTPN11;RAF1;PIAS1
MEF2A;HDAC5;MYOD1
GPAM;SP1;ESR1
KAT2B;CREBBP;APH1B;MAML1;DTX1;DTX4
LYN;MAP2K1;CREB1;NFATC3;GAB1;MAPK1;PTPN11;RAF1;CRK;FOXO1
MAF;CUL3;PGAM5;RBX1
SHC4;MAP2K1;SHC2;PLA2G12A;RALA;GAB1;PTPN11;CALML4;GRIN2B;MAPK10;RAP1A;MAPK1;RAF1;CAL
MAP2K1;EEF2K;CREB1;GAB1;MAPK1
SPRED1;ROCK1;APC;CAMK4;CDK1;MARK1
CREB1;MAPK1;RAF1
GABRA1;ADCYAP1R1;CREBBP;RUNX3;HIF1A;PHOX2B;CREB1;SP1;NOS1AP;CTNNB1;SCN5A;SCN3B;IL6F
NPAT;CCND3;RRM1;SIN3A;E2F1;CDK1;CCDC6;SMC1A;RAF1
CCND3;SFRP1;FZD3;FZD5;APC;CSNK1A1;NFATC3;CTNNB1;PRICKLE1;SENP2;LRP6
MAPK10;FZD3;FZD5;CTNNB1;LRP6
SFRP1;NRG3;ERBB4
CCND3;CREB1;E2F1
CREB1;SIN3A;GRID1;SP1;DLX6;TET3;E2F1
MAP2K1;MAPK1;RAF1;EPOR
MAP2K1;APC;MAPK1;CTNNB1;RAF1;SOS2;FGFR1
CHRM3;NCKAP1;MAP2K1;ROCK1;ENAH;GNA13;APC;MAPK1;RAF1;MYH10;SOS2;CRK;FGFR1
MAP2K1;CREB1;PTK2B;MAPK1;PTPN11;RAF1;HIF1A
MAP2K1;MMP2;E2F1;MAPK1;RAF1
MAPK10;MAP2K1;MAPK1;RAF1
BMPR2;LOXL4;BMPR1A
KLF10;NCOA2;USP2;GFPT1;DYRK1A;HNRNPU;PROX1;RORB;BTBD9;MAPK10;CREB1;SIN3A;ID2;NRIP1;ID
CCND3;CREB1;RRM2B;E2F1;CDK1;SMC1A;RAD9A
RUNX1;RBX1
PRDM16;ARID5B
CDH6;CDH2;MAPK1
MAP2K1;PTK2B;MAPK1;PTPN11;RAF1
CADM3;CDH2;TERF2IP;ATP2B1;GRIN2B
TGFBR3;APC;CTNNB1;RAF1
PTGFR;S1PR1;S1PR3
CCND3;CREB1;RRM2B;E2F1;CDK1;SMC1A;RAD9A
HDAC5;KAT2B;ALDH2;ADH1B
GABRA1;GABRB1;GABRA3;AP2M1
MAT2A;DNMT3A;MAT2B;PSPH
CSNK1G3;SMURF2;CSNK1A1;CUL3;FBXL17
TDG;TET3
ALAD;ALAS2
MAT2A;MAT2B
RRM2B;OTC

MAPK10;MAP2K1;RAP1A;ROCK1;MAPK1;RAF1;CRK;MYPN;TNS1
CDH6;TFAP2B;HDAC5;FZD3;CDH2;GBX2;CTNNB1;PHOX2B;FGFR1
PPP2CA;MAPK10;CCND3;CREBBP;FZD3;FZD5;APC;CTNNB1;LRP6
EOMES;BCL11B;ZBTB16;ID2
HDAC5;CREBBP;SP1;NFATC3
CREBBP;DNMT3A;ZFX
CUL3;PDHB;HIF1A
SHC4;LYN;MAP2K1;SHC2;ROCK1;ADCY9;RAP1A;ELMO1;PTK2B;MAPK1;RAF1;SOS2;CRK
LYN;MAP2K1;MAPK1;PTPN11;RAF1;CRK
MAP2K1;MAPK1;RAF1;CALM1
NCOA2;RXRA;NRIP1;FOXO1
SHC4;MAP2K1;SHC2;ROCK1;XIAP;PARVA;ACTN4;MAPK10;CCND3;RAP1A;CHAD;CTNNB1;MAPK1;RAF1;C
MAP2K1;SP1
CREBBP;TFEB;HIF1A;RBX1
NCKAP1;PURA;BTG1;VAPA;AZIN1
PPP2CA;CREBBP;CDC37;TNFAIP3;MAPK1;BTRC;RAF1;SELE
RPS6KA6;PRKAB2;ADCY9;CREB1;PRDM16;ACSL6;SOS2;PRKG1;FGFR1
LYN;MAP2K1;MAPK1;PTPN11;RAF1
ADK;MAT2B
ADAMTS5;SOX6;CALM1;RUNX3;BMPR1A;FGFR1
CREBBP;MAPK1;HIF1A
MAP2K1;RXRA;E2F1;MAPK1;RAF1;SOS2
BMPR2;BMPR1A
MAPK1;RAF1
PCYT1B;MAT2A;ETNK1;DNMT3A;SOD2
EEF2K;EIF2B2;EIF3J;EIF4EBP2;EIF4E
APC;CSNK1A1;MAPK1;CTNNB1;LRP6
MAP2K1;GAB1;E2F1;MAPK1;RAF1;FOXO1;FGFR1
TGFBR3;MAPK1;CTGF
MAP2K1;PTK2B;MAPK1
S1PR1;MAPK1;S1PR3
MMP2;MAPK1;CTNNB1;PTPN11;CRK;FOXO1
GNA13;MAPK10;ROCK1;CALM1
MAP2K1;MAPK1;PTPN11;RAF1
PCYT1B;ETNK1
CREB1;MAPK1
NRIP1;ESR1
MAP2K1;MAPK1
GRM5;RRM2B
PRKAB2;CREB1;GPAM;INSIG2;SP1;PPARGC1B
TNFAIP3;ATP2B1;HIF1A;FOXO1;ADAMTS5;SFRP1;CDC34;RXRA;SALL4;NRIP1;ID4;SOSTDC1;MXD1
CREB1;CDK1;SMC1A;RAD9A
E2F1;CTNNB1
DLST;PDHB;PSPH;GLS
ALDH2;ADH1B
MAP2K1;CREB1;PTK2B;MAPK1;PTPN11;RAF1;CRK
CSNK1A1;DYRK1A;CALM1
MEF2A;MYOD1;ID2
PTGFR;HPGD;PTGFRN;PPARGC1B
NCOA2;MAP2K1;CREB1;SP1;MAPK1;CTNNB1;RAF1
ITCH;APH1B;MAML1;DTX1;HIF1A
MAF;RNASE6;RUNX3;TMEM106A;IL13RA1
SP1;MAPK1;IL17RD

REN;AGTR2
MAP2K1;MAPK1
CREB1;XIAP;CALM1;GRIN2B
LYN;MAP2K1;MAPK1;PTPN11
AMER1;PRKAB2;RRM2B;ID2;TM7SF3
MAPK1
MAPK1;RAF1
CDC37;LOXL4
SIN3A;TEAD1
GAB1;MAPK1;PTPN11
CHRM3;ADRA2B;ADRA2A
MAPK10;SP1;SOD2
MAPK10;MAP2K1;MAPK1;EIF4E
RRM1;RRM2B
CRP;CCND3
PRKAB2;CAMK4;MAPK1;GRIN2B
HIF1A
SLIT2
FOXO1
TSHR
ERI1
CDH6;CDH2;CTNNB1;TEAD1;AMOT;CTGF;RBX1;FGFR1
APC;SP1;XIAP;FOXO1
CDH6;FZD5;ZBTB16;GATA6;PLXNA2;CTNNB1;SORCS1;BMPR1A;PHF8
SERBP1;PTK2B;MAPK1;SMAD7
NCOA2;ABHD2;TNFAIP3;SLC39A13;SMARCA1;SMC1A;ESR1;FOXO1;TGFBR3;KAT2B;ARL5B;RXRA;GPAM;
SMAD9;MAPK1
MAP2K1;E2F1;MAPK1;INHBA;RAF1;BMI1;IL6R
PHLPP2;MAP2K1;PFKFB3;IRS4;HIF1A;FOXO1;EPOR;PPP2CA;EFNA3;CREB1;CDC37;CHAD;MAPK1;RAF1;
DLST
ERBB4
SMAD7
HIF1A
XRCC5
MAPK10;MAP2K1;RALA;E2F1;MAPK1;RAF1
GPR37;UBE2G1;UBE2J1
ST6GAL2;ST6GALNAC3
MAT2A;MAT2B
TNKS2;TNKS
MED14;RXRA
MAP3K2;MAP2K1;MAPK1;PTPN11
OCLN;ROCK1;CTNNB1;CLDN1
LYN;MAP2K1;MAPK1;PAPSS2
CHRM3;PTGFR;ALG6;GNRHR;S1PR1;GPR135
MED14;PIAS1
MAPK10;CCND3;APC;MAPK1;CTNNB1;SOD2;SOS2
PPP2CA;CALM1;HK2
PRKAB2
MB
HIF1A
SESN3;ZMAT3
MAP2K1;AGAP2;MAPK1;PTPN11;RAF1
GJC1;CHRM3;ADCY9;GJA3;CAMK4;GJA9;ATP2B2;ATP2B1;CALM1

MAPK1;CTNNB1;RAF1
ALG6;ALG14
MAPK1;CALM1
RAD9A
CREB1
ST6GALNAC3
SDR16C5;RXRA;MAPK1
RXRA;CDK1
CDH6;PRKAB2;CDH2;CTNNB1;TEAD1;FGFR1
E2F1;CDK1;BACH1
MAP2K1;CHP1;RAF1;CALM1
MMP2;ATP11A;CTGF;SMAD7
RXRA
E2F1
MAT2B
RXRA
CCND3;CREB1;E2F1;CDK1
MME;CHP1;MAPK1;CALM1;GRIN2B
TFAP2B;DOCK5;CREBBP;APC;VAPB;NEK7;CTNNB1;CEP170;CALM1;DYNLL2;MAPRE2;DCAF7
MAPK10;CTNNB1;TEAD1
MEF2A
MAPK10;MAPK1
APC;RUVBL1
MAP2K1;RAF1
DNMT3A;MAT2B
TFEB;FOXO1
PTPN11;IL6R;HIF1A
MAPK1
RALA
PRKAB2;EEF2K;PFKFB3;PPARGC1B
EIF2B2;EIF4E
CTNNB1;MTDH
CRP;ABCA1;SOD2
GPR37;PSMD11;UBE2G1;UBE2J1
NAPA;SLC17A6;AP2M1
ID2
PPP2CA
SOSTDC1
ST8SIA3
IL6R
RRM1;TDG
SCN5A;INHBA;TBX5
GNA13;ADCY9;PDE1B;PDE7B;CALM1
CREB1;MAP3K9
CDH2;DAG1;CTNNB1;ACTN4
E2F1;SMC1A;RAD9A
MMP2;SELE
GRM5
ESR1
MAF
SMC1A
CTNNB1
SLC30A4;SLC39A13

SYTL4
SIN3A
BCLAF1
ABCA1
ABCA1
DLST
PLXNA2;CDK1;E2F1;MAPK1;CD47;SLIT2
CALM1;FOXO1
CBX5;E2F1
TFAP2B;CELF3;MXD1
CCND3;RBL1;CDK1;E2F1;SMC1A;RBX1
RXRA;ESR1
TPM3;ACTN4
IRS4
SOX6
SELE
GABRA1
PAPSS2
TEAD1
DLST
MAPK10;MAP3K9
MAPK10
RXRA
HRK;MAPK10;DFFA;XIAP
DFFA;CREB1;DAG1;MAPK1
CREBBP;HIF1A;HK2;PSPH
ENTPD1;RRM1;RRM2B;CMPK1
SFRP1
EIF4E
OTC
CRP;ABCA1;DIO2;SOD2
CRP;ABCA1;SOD2
CDS2
PPP1R1B
SET;SETD9;NSD1
CDK1;SMC1A
PHLPP2;MAP2K1;EPOR;PPP2CA;CCND3;EFNA3;CREB1;CDC37;CHAD;MAPK1;COL6A5;RAF1;IL6R;SOS2;[illegible]
ACSL6
RXRA
BCL2L13
MAPK1
PRKAB2;COX7B;ITCH;RXRA;UQCR10;IL6R;SMAD7
SP1;DIO2
PPM1L;DLST;GLS;OTC
MAPK1
TEAD1
INHBA;IL6R
RXRA;SP1;MAPK1
ADCYAP1R1
GPAM
ELK4;MAP3K2;MAPK10;MAP2K1;RAP1A;NFATC3;MAPK1;RAF1;SOS2;CRK;FGFR1
DNMT3A
GNRHR;AGTR2;TSHR

CRP;CSNK1A1;C1QBP;SELE
PGM3
MAML1
DYNLL2
EDEM1
PPP2CA
MAPK1;PTPN11
PRKAB2
ABCA1
COA1;TIMMDC1
GBX2
MMP2
SOX6
CREBBP;C1QBP;MAPK1;ACTN4;KPNA1
MAPK1
NRIP1
ABCA1
MAPK1
ACSL6
PRKAB2
HRK;DFFA;XIAP
HIF1A
PTPN11
BTBD9
RXRA;ACSL6
ACSL6
LYN
XIAP
RPA4
DFFA
GTF2H1
MAPK10;MAP2K1;MAPK1
HK2
CDC37
ALDH2
GPR27;PTGFR;CHRM3;NPFFR1;GPR37;GPR75;AGTR2;ADRA2B;ADRA2A
MMP2
MAPK10;MAP2K1;TNFAIP3;MAPK1
RUNX1
MAPK10
CYB5R4
E2F1
HS3ST3B1;NAA30;CHST7;NAT8L;CHST3
PSMD11
ADCY9;CREB1;SP1;CALM1
COX7B;UQCR10
ARL3;TUBD1
RPS6KA6
RXRA;SLC39A13

;CDH2;APC;CAMK4;IGF2BP1;PTK2B;MAPK1;CTNNB1;RAF1;EIF4E
;SP1;E2F1;PTK2B;MAPK1;RAF1;SOS2;CRK;SH3GL2;AP2M1
K1;SNIP1;MAPK1;BTRC;RAF1;RNF111
P2;GAB1;PTPN11;SOD2;SELE;RAB11A;RCAN1;OCLN;ITCH;CREB1;RCAN2;CTNNB1;RAF1;CRK

F1;SOS2;CRK;EIF4E

R1A

2;TEAD1;FGFR1;BMPR1A
AP3K9;RAF1;SOS2;CRK;EIF4E

TNNB1;BMPR1A
C;SRSF10;SRSF9

2;FGFR1

1

AF1;SOS2

_M1;SOS2;FGFR1

₹;PPARGC1B

)4;BTRC

:RK

;SERTAD2;SP1;CDC37;NRIP1;CDK1;SPRY1

PELO;IL6R;EIF4E;FGFR1

EIF4E;FGFR1
